# Differentially Expressed miRNAs in Hepatocellular Carcinoma Target Genes in the Genetic Information Processing and Metabolism Pathways

**DOI:** 10.1038/srep20065

**Published:** 2016-01-28

**Authors:** Thomas Thurnherr, Way-Champ Mah, Zhengdeng Lei, Yu Jin, Steven G. Rozen, Caroline G. Lee

**Affiliations:** 1 NUS Graduate School for Integrative Sciences and Engineering, National University of Singapore, Singapore; 2 Division of Medical Sciences, Humphrey Oei Institute of Cancer Research, National Cancer Centre Singapore, Singapore 169610, Singapore; 3 Program in Neuroscience and Behavioural Disorders, Duke-NUS Graduate Medical School, Singapore; 4 Cancer and Stem Cell Biology Program, Duke-NUS Graduate Medical School Singapore, Singapore 169547, Singapore; 5 Department of Biochemistry, Yong Loo Lin School of Medicine, National University of Singapore, Singapore 119077, Singapore

## Abstract

To date, studies of the roles of microRNAs (miRNAs) in hepatocellular carcinoma (HCC) have either focused on specific individual miRNAs and a small number of suspected targets or simply reported a list of differentially expressed miRNAs based on expression profiling. Here, we seek a more in-depth understanding of the roles of miRNAs and their targets in HCC by integrating the miRNA and messenger RNA (mRNA) expression profiles of tumorous and adjacent non-tumorous liver tissues of 100 HCC patients. We assessed the levels of 829 mature miRNAs, of which 32 were significantly differentially expressed. Statistical analysis indicates that six of these miRNAs regulate a significant proportion of their *in silico* predicted target mRNAs. Three of these miRNAs (*miR-26a, miR-122*, and *miR-130a*) were down-regulated in HCC, and their up-regulated gene targets are primarily associated with aberrant cell proliferation that involves DNA replication, transcription and nucleotide metabolism. The other three miRNAs (*miR-21, miR-93*, and *miR-221*) were up-regulated in HCC, and their down-regulated gene targets are primarily involved in metabolism and immune system processes. We further found evidence for a coordinated miRNA-induced regulation of important cellular processes, a finding to be considered when designing therapeutic applications based on miRNAs.

Hepatocellular carcinoma (HCC) is one of the most common and lethal cancers worldwide^1^. The strongest risk factors for HCC are chronic hepatitis B and C viral (HBV, HCV) infection, as well as alcoholic liver disease^2^. One of the main reasons for the lethality of HCC is the lack of diagnostic markers for early detection of the disease. At late stages, HCC shows a high clinical heterogeneity with poor prognosis. Although recent advancements in functional genomics have increased our knowledge of HCC^3^ tremendously, our understanding of the molecular mechanisms leading to the disease still remains largely fragmentary.

MicroRNAs (miRNAs) are a class of short, non-coding RNAs that play important roles in gene expression regulation. They control gene expression by binding to the 3′UTR of mRNAs, leading to their degradation or translational repression. There is accumulating evidence suggesting that miRNAs play an important role in carcinogenesis, either as tumor suppressors or onco-miRs^4^.

Numerous miRNAs are associated with HCC. Of these, six were consistently reported in several studies to be differentially expressed in tumorous compared to non-tumorous tissues of HCC patients (see review^5^). MiRNAs (*miR-21, miR-221, miR-222*^6^, *and miR-224*^7^) are consistently up-regulated in the tumors of HCC patients and were reported to dys-regulate proliferation^6^,^8^,^9^ and/or apoptosis^7^,^10^ through targeting various molecules including *PTEN*^6^, *SMAD4*^9^, *CDKN1B/p27*, and *CDKN1C/p57*^8^. M*iR-122*^11^ and *miR-199a*^6^ are consistently down-regulated in HCC tumors and act as tumor suppressors by modulating the expression of cyclin G1^12^ and the *PAK4/Raf/MEK/ERK* pathway^13^, respectively.

Traditionally, the role of miRNAs has been investigated by focusing on a single miRNA or a family of miRNAs and one or several targets with similar functions. Selection of targets for this study was based on *in silico* predictions and the correlation between mRNA and miRNA expression^12^,^14^. Two previous studies on breast cancer and a pan-cancer catalogue integrated miRNA and mRNA expression profiles of tumor tissues to study miRNA-regulation on a whole genome level^15^,^16^. Here we considered all miRNAs and mRNAs in tumor and non-tumorous tissues available from whole genome miRNA and mRNA expression profiling, together with *in silico* miRNA target prediction algorithms. Through integration of these data, we identified miRNAs that coordinately dys-regulate mRNAs to perturb specific pathways that may lead to tumorigenesis.

## Results

**Figure 1:**
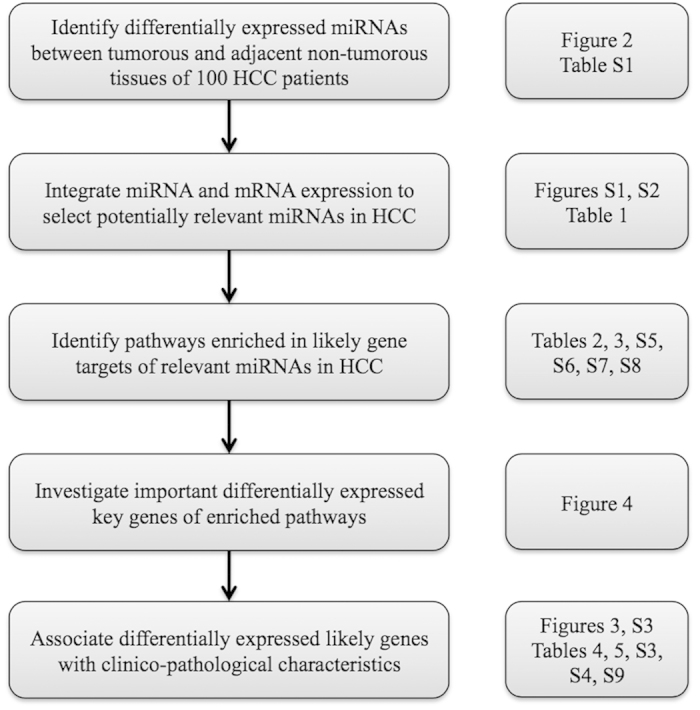
Schematic overview of the workflow implemented for identification of differentially expressed regulatory miRNAs and their targets in HCC.

The workflow in Fig. 1 was employed to elucidate the pathways and clinically relevant gene targets that are modulated by dys-regulated miRNAs in 100 HCCs. The steps of the workflow were as follows: (1) Identify differentially expressed miRNAs between tumorous and adjacent non-tumorous liver from the miRNA expression profiles. (2) Integrate miRNA and mRNA profiles to select potentially relevant miRNAs in HCC. (3) The pathways and clinical significance of these miRNAs and their gene targets were examined.

### miRNAs and gene targets differentially expressed in HCC

**Figure 2:**
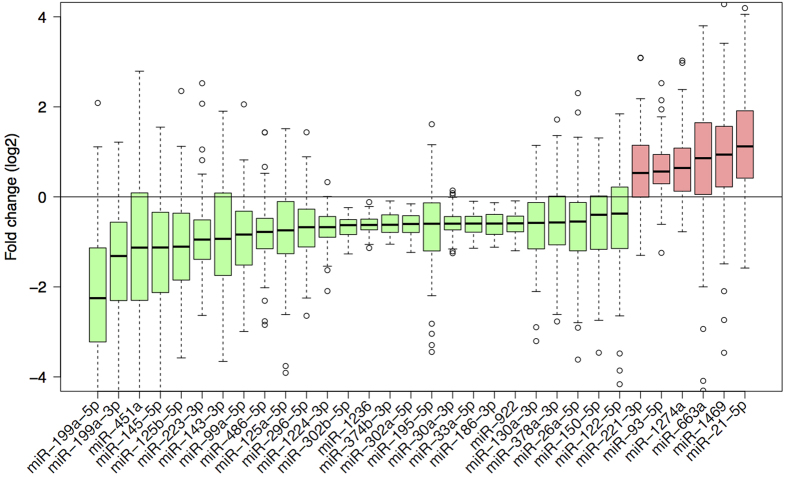
miRNAs with a statistically significant differential expression between tumorous and adjacent non-tumorous tissues of 100 HCC patients (absolute FC >1.5 and FDR < 0.05).

We assessed the levels of 829 human mature miRNAs. We found thirty-two to be significantly differentially expressed (absolute fold change (FC) > 1.5 and FDR < 0.05) between the tumorous and adjacent non-tumorous liver of HCC patients (Fig. 2). Six of these were up-regulated while 26 were down-regulated in the tumors.

To identify the relevant gene targets of these differentially expressed miRNAs in HCC, we employed an integrated approach, which combines miRNA with gene expression profiles as well as *in silico* prediction of likely gene targets of miRNAs. As miRNAs modulate gene expression through mRNA degradation or translational repression, we first examined a negative correlation in expression between miRNA and their gene targets.

We determined the negative correlation between the expression of each of the differentially expressed miRNA and all genes in the human genome and ranked genes according to the strength of the relationship. An example of a strong negative correlation between *miR-93* and *PTPRN2* is shown in Supplementary Fig. S1. We then compared this ranked list of negative gene correlation to the set of *in silico* targets predicted by the MiRanda program^17^ using gene set enrichment analysis (GSEA)^18^. GSEA computes an enrichment score by stepping through the ranked list of genes. If a gene is an *in silico* predicted target, the enrichment score is increased. Otherwise, the enrichment score is decreased. An example of an enrichment plot for *miR-93* is shown in Supplementary Fig. S2. We found eight of the 32 differentially expressed miRNAs are significantly enriched with negatively correlated predicted gene targets (FDR < 0.01; Table 1, MiRanda column). Interestingly, most of these miRNAs were previously reported to be associated with HCC^3^. We also compared the same ranked list of negative gene correlation with the set of *in silico* targets predicted by MirTarget2^19^, which identified seven miRNAs with statistically significant enrichment scores (Table 1, MirTarget2 column). Six of these miRNAs (*miR-21, miR-26a, miR-93, miR-122, miR-130a*, and *miR-221*) were statistically significant against both MiRanda and MirTarget2 (Table 1) and were subjected to further analysis.

### Pathways affected by relevant miRNA targets

We first examined if any of the six abovementioned miRNAs are coordinately dys-regulated in the tumors of HCC patients using Spearman’s correlation. As evident in Supplementary Table S2, only a pair of miRNAs (*miR-93* and *miR-221*) that is up-regulated and a pair of miRNAs (*miR-26a* and *miR-130a*) that is down-regulated in the tumors of HCC patients are correlated (Spearman’s correlation coefficient: 0.6 and 0.56, respectively), suggesting that most of these miRNAs are likely regulated independently in HCC patients.

**Figure 3:**
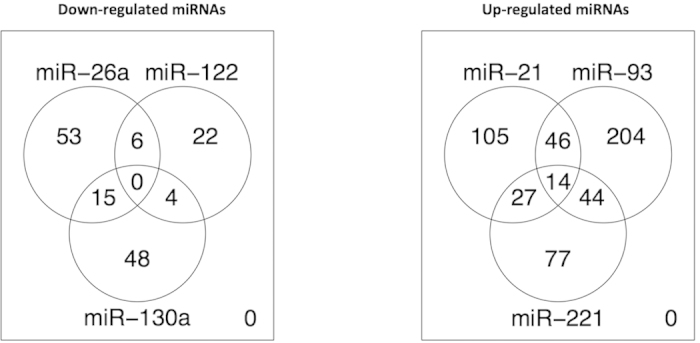
Differentially expressed targets shared by relevant miRNAs. Likely targets were determined by the GSEA pre-ranked function, comparing genes ranked by their negative correlation with the miRNA and the set of predicted targets by MiRanda. Only differentially expressed genes (absolute FC > 1.5 and FDR < 0.05) were considered.

To elucidate the pathways that are likely to be affected by the relevant gene targets of the three up-regulated and three down-regulated miRNAs, we first examined the differentially expressed gene targets of these differentially expressed miRNAs. Fewer differentially expressed gene targets were identified for down-regulated miRNAs than for up-regulated miRNAs (Fig. 3). The list of differentially expressed gene targets of down-regulated and up-regulated miRNAs, their correlation coefficient, and their MiRanda and MirTarget2 predictions are given in Supplementary Tables S3 and S4. For down-regulated miRNAs, out of 148 gene targets, few genes (6, 15, and 4 gene targets respectively) are common between two miRNAs (Fig. 3). On the other hand, for up-regulated miRNAs, out of 517 gene targets, a larger number of genes (46, 27, and 44 gene targets respectively) were common between two and 14 between all three miRNAs. These data suggest that while there is a subset of gene targets that are commonly modulated by two or more significantly differentially expressed miRNAs, the majority of the gene targets of these miRNAs are unique.

Pathways modulated by relevant gene targets of differentially expressed miRNAs were determined using KEGG pathways as defined in MSigDB^20^. Interestingly, although the proportion of differentially expressed gene targets that are common among either the three up-regulated or the three down-regulated miRNAs is relatively low (Fig. 3), all three up-regulated miRNAs (*miR-21, miR-93*, and *miR-221*) coordinately target a set of pathways that is distinct from all three down-regulated miRNAs (*miR-26a, miR-122*, and *miR-130*), which also coordinately target a set of pathways (Table 2). We found only the Cell Growth and Death pathway is targeted by all six miRNAs (Table 2). A closer examination of the Cell Growth and Death pathway revealed that the gene targets of all six differentially expressed miRNAs modulate TP53 signaling. However, the gene targets of all three up-regulated miRNAs are involved in apoptosis, whereas the gene targets of all three down-regulated miRNAs are involved in cell cycle (Table 3). This is consistent with tumorigenesis down-regulating apoptosis and up-regulating cell proliferation.

Gene targets of all three up-regulated miRNAs (*miR-21, miR-93*, and *miR-221*) were found to coordinately target primarily metabolism pathways, particularly the amino acid, carbohydrate, and lipid metabolism pathways, as well as the immune system (Table 2). On the other hand, gene targets of all three down-regulated miRNAs (*miR-26a, miR-122*, and *miR-130*) primarily target Genetic Information Processing, in particular, DNA replication and repair, transcription, cell growth, and nucleotide metabolism (Table 2). This is consistent with the process of tumorigenesis in which pathways not immediately pertinent to the proliferation of the tumor cell, such as amino acid, carbohydrate, or lipid metabolism, are down-regulated so resources can be redirected to ensure the proliferation of the tumor cells.

**Figure 4:**
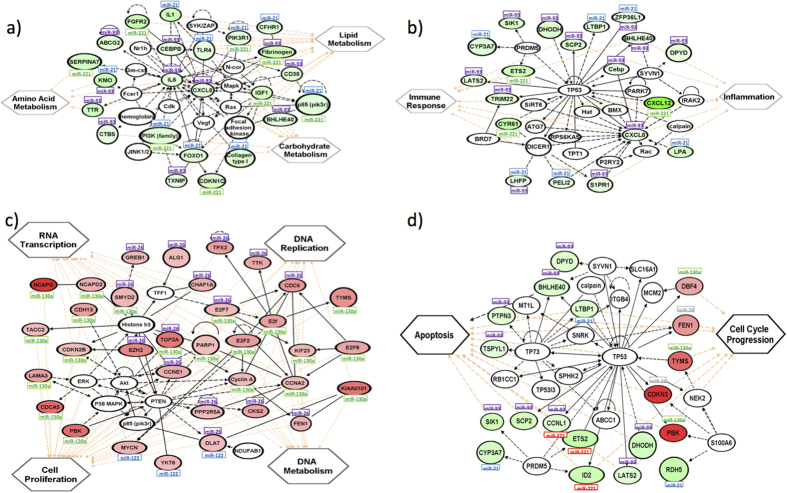
Gene networks inferred from differentially expressed miRNA targets with similar functional annotation based on KEGG gene ontology: (a) Targets of up-regulated miRNAs involved in metabolism; (**b**) Targets of up-regulated miRNAs involved in immune system processes; (**c**) Targets of down-regulated miRNAs associated with DNA replication; and d) Targets of up- and down-regulated miRNAs involved in cell growth and death.

Next, we derived gene networks based on differentially expressed targets using the Ingenuity Pathway Analysis (IPA) program (http://www.ingenuity.com). We found that the network of genes in the metabolism pathways, dys-regulated by the three up-regulated miRNAs, is primarily centered on nodal molecules. IL6 and CXCL8 are nodal molecules of metabolism pathways, both of which are targets of miR-93 (Fig. 4a). We also found that CXCL8 is an important nodal molecule, together with TP53 in the gene network of the inflammation and immune response pathways (Fig. 4b). Moreover, TP53 and TP73 play central roles in the network of genes in the Cell-Cycle Progression and Apoptosis pathways dys-regulated by all six miRNAs (Fig. 4d). The gene network dys-regulated by the three down-regulated miRNAs in the Genetic Information Processing pathways including Cell Proliferation, DNA Replication, RNA transcription, and DNA metabolism centers on *PTEN/AKT*, Histone H3, and Cyclin A/A2 (Fig. 4c).

### Relevant differentially expressed miRNA targets associated with various clinical characteristics

We next evaluated if differentially expressed miRNAs or their relevant differentially expressed gene targets are associated with various clinical characteristics, such as gender, age, hepatitis infection status, liver cirrhosis, stage, grade, size, relapse status, encapsulation, and Alpha Fetoprotein (AFP) levels (Tables 4 and 5). As evident in Table 4, *miR-122* showed significant association with AFP levels (FDR < 0.2). Similarly, *miR-221* was significantly correlated with age (FDR: 0.13) and hepatitis infection (FDR: 0.15). More specifically, HBV-positive patients showed a significantly higher *miR-221* expression compared to HBV-negative patients. Next, we associated the differentially expressed targets of the six miRNAs with various clinical characteristics. While only a single differentially expressed gene target of down-regulated *miR-122* was found to be significantly associated with stage of HCC, 13, 2, and 5 down-regulated gene targets of up-regulated miRNAs were found to be significantly associated with stage of cancer, tumor grade, and encapsulation. No miRNA was significantly associated with relapse status using the Cox proportional hazards model (Supplementary Table S9), although miR-130a showed the lowest p-value. When another cohort of 373 HCC patients from The Cancer Genome Atlas (TCGA)^21^ was assessed, miRNA-130a was found to be significantly associated with patient outcome (time-to-death, 89 events) (HR: 0.52, FDR: 0.02, Supplementary Table S9 and Figure S4).

We employed IPA to obtain a glimpse of the major cancer-related pathways dys-regulated by these genes associated with various clinical characteristics. Interestingly, greater than 75% of the genes dys-regulated by up-regulated miRNAs that are associated with a lower stage of cancer, tumor grade, or encapsulation were found to be in cancer or related pathways (primarily cell cycle, cell death and survival, as well as inflammatory/immune disease/response).

## Discussion

Through profiling, miRNAs have been shown to be differentially expressed in the tumors of HCC patients (see review^5^). Gene targets of many of these dys-regulated miRNAs have been identified. However, thus far, only very few studies have attempted to identify which one/combination of the differentially expressed miRNAs is more likely to play roles in HCC through miRNA-gene interaction networks.

In this study, we integrated miRNA with gene expression profiles, as well as miRNA target prediction programs to identify a subset of miRNAs that are highly correlated with appropriately dys-regulated predicted gene targets. We hypothesized that this subset of miRNAs is most likely to play roles in HCC through their gene targets. We also examined the correlation of these miRNAs or their gene targets with clinical characteristics, as well as pathways that are perturbed by the appropriately dys-regulated predicted gene targets of these miRNAs.

Of the 32 miRNAs that were differentially expressed between the HCC tumorous and non-tumorous livers (Fig. 2), we found that six are highly correlated with appropriately dys-regulated predicted gene targets (Fig. 3). Four (*miR-21, miR-221, miR-26,* and *miR-122*) of these six miRNAs were previously reported to be differentially expressed in HCC in several studies and to dys-regulate important cancer pathways^12^,^22^,^23^,^24^, highlighting the importance of these miRNAs in HCC. We further found these six miRNAs are mainly independently regulated in HCC patients except for two pairs of miRNAs (*miR-93* with *miR-221* and *miR-26a* with *miR-130a*), which are moderately correlated in expression (Supplementary Table S2).

Three (*miR-26a, miR-122*, and *miR-130a*) of these six miRNAs were down-regulated while the other three miRNAs (*miR-21, miR-93*, and *miR-221*) were up-regulated in the tumors of HCC (Fig. 2). Interestingly, the total number of down-regulated predicted gene targets of the three up-regulated miRNAs (517) was ~3.5 times more than the up-regulated gene targets of the three down-regulated miRNAs (148). In addition, the greater percentage of common gene targets was found among the up-regulated miRNAs (34–52%) (Fig. 3 and Supplementary Table S4) than among the down-regulated miRNAs (28–31%) (Fig. 3 and Supplementary Table S3). This perhaps suggests that the up-regulated miRNAs play a more diverse but coordinated role in HCC, down-regulating the expression of a greater number of different gene targets.

Some miRNAs showed significant association with clinical characteristics. Expression of *miR-122* was significantly correlated with AFP levels. Indeed, *miR-122* was previously implicated in the regulation of AFP and high AFP levels are associated with a more aggressive cancer^25^. Similarly, expression of *miR-221* was significantly correlated with age and hepatitis infection. Furthermore, several of the predicted dys-regulated gene targets of these miRNAs were associated with clinical characteristics. The down-regulated gene targets of up-regulated miRNAs were found to be associated with a better prognosis, including a lower stage of cancer and lower grade of tumor, as well as complete encapsulation, and are in cancer or cancer-related pathways, including cell cycle, cell death, and survival as well as inflammatory/immune disease/response. On the other hand, the sole up-regulated gene targets of the down-regulated *miR-122* that showed clinical relevance was found to be associated with a higher stage of cancer.

Notably, although the majority of gene targets of the six miRNAs were unique, pathway enrichment analysis revealed that relevant gene targets of all six up-regulated miRNAs (*miR-21, miR-93*, and *miR-221*) were enriched in metabolism and immune system processes (Table 2) while relevant gene targets of all three down-regulated miRNAs (*miR-26a, miR-122*, and *miR-130a*) were enriched in genetic information processing pathways including cell proliferation, DNA replication, and metabolism, as well as RNA transcription. These observations suggest built-in redundancy of different gene targets regulated by different miRNAs targeting the same set of pathways. This redundancy may pose challenges for any single miRNA to be used as a target for cancer therapy.

The pathways targeted by these six miRNAs represent the various hallmarks of cancer. The predicted gene targets of the three up-regulated miRNAs reside in the metabolism pathway, which has recently generated great interest^26^,^27^,^28^. In fact, these up-regulated miRNAs (*miR-21, miR-93*, and *miR-221*) have previously been implicated in the regulation of lipid metabolism^29^,^30^,^31^,^32^. Closer examination of the pathways leading to the dys-regulation of lipid, amino acid, and carbohydrate metabolism identifies proinflammatory molecules IL6 and CXCL8, both of which are down-regulated by miR-93, as the two nodal molecules that play key roles in metabolism (Fig. 4a). Interestingly CXCL8 was also found to be one of the two key nodal molecules, together with TP53 in the inflammation and immune response pathways (Fig. 4b). As a result, the down-regulation of these pro-inflammatory molecules, IL6 and CXCL8 in cancer cells could perhaps perturb their intrinsic metabolic pathways, as well as help these cells evade the immune response, which potentially leads to a survival advantage of these malignant cells^33^,^34^.

In addition to being a nodal molecule for inflammation and immune response, TP53, together with TP73, were identified as key molecules involved in the cell-cycle progression and apoptosis pathways. Targets of all six miRNAs (Fig. 4d) modulate this biological function. More specifically, the down-regulated miRNAs (*miR-26a, miR-122*, and *miR-130a*) target cell-cycle processes while the up-regulated miRNAs (*miR-21, miR-93*, and *miR-221*) target apoptosis. These observations are consistent with previous literature which reported that *miR-26a*^35^ and *miR-122*^36^ modulated cell-cycle progression, while *miR-221*^37^ and *miR-21*^38^ regulated apoptosis in liver cancer cells. In addition, one of the down-regulated predicted gene targets of miR-93, the tumor suppressor LATS2, was reported to be involved in TP53-mediated apoptosis, suggesting that miR-93 plays a role in the evasion of apoptosis, a hallmark of cancer^39^.

As mentioned earlier, the three down-regulated miRNAs (*miR-26a, miR-122*, and *miR-130a*) target cell proliferation and genetic information processing including DNA replication, transcription, and nucleotide metabolism (Table 2 and Fig. 4). Consistent with our results, *miR-26a*^40^ and *miR-122*^41^ were previously reported to inhibit cell growth in liver cancer cells, probably through dys-regulation of DNA replication^42^ and metabolic processes^43^ respectively. Closer examination reveals that pathways modulated by these down-regulated miRNAs center around key molecules *PTEN/AKT* and Histone H3. Both *miR-26a*^44^ and *miR-122*^45^ have been reported to target the AKT pathway in HCC. Furthermore, *miR-130a* was reported to target *MET*^46^ and to stimulate apoptosis in non-small cell lung cancer^46^. As MET also plays a role in AKT signaling^47^, down-regulation of these three miRNAs (*miR-26a, miR-122*, and *miR-130a*) may perturb the AKT axis, leading to the disruption of cell death.

Indeed, our data confirms that MET expression was found to be ~1.26 fold higher in the tumorous compared to the adjacent non-tumorous tissues and was moderately correlated with *miR-130a* (*rho* = *0.21*, Supplementary Table S5). In addition, the involvement of Histone H3 as a nodal molecule suggests that these pathways may be epigenetically regulated. The gene targets of these miRNAs have been reported to either be direct binding partners of Histone H3 (NCAPD2^48^, CHAF1A^49^, and PARP1^50^) or activated by post-translational modification of Histone H3 (CCNE1^51^, Cyclin A^52^ CDKN2B^53^, TACC2^54^), highlighting the complex gene networks regulated by these miRNAs.

In summary, these data reveal six miRNAs that may play important roles in tumorigenesis through the dys-regulation of gene targets. These six miRNAs generally modulate unique gene targets but coordinately target a few common pathways, with partially overlapping key nodal molecules, highlighting the redundancy of miRNAs and gene targets that focuses on reprogramming critical pathways to facilitate tumor development. Although the redundancy poses a challenge to the identification of a single target for therapy, deeper analyses of the pathways modulated by these miRNAs suggest that key nodal molecules such as *IL6, CXCL8, TP53, TP73, PTEN*, and *AKT* may serve as potential targets for intervention. Many of these molecules have already been explored as targets of therapy in other cancers^33^,^55^,^56^,^57^,^58^ and future focus could be directed towards these molecules in HCC.

In addition to potential targets for intervention, this analysis also highlights several dys-regulated predicted gene targets as potential biomarkers for cancer prognosis, since some of the down-regulated predicted gene targets of the up-regulated miRNAs were found to be associated with better disease prognosis, while the sole up-regulated gene target of the down-regulated miRNA was associated with poorer prognosis.

## Methods

### HCC tumorous and adjacent non-tumorous tissue samples and clinical data

We obtained tumorous and adjacent non-tumorous tissue samples of 100 HCC patients from the National Cancer Centre Singapore (NCCS) Tissue Repository with prior written informed consent. The NCCS Institutional Review Board (NCC IRB) (2008/440/B) and the SingHealth Central Institutional Review Board (CIRB) (2013/455/B) approved this study. All analyses were performed in accordance with relevant guidelines and regulations. The demographics and clinical information of these 100 HCC patients are summarized in Table 5.

### Gene and miRNA expression profiling

We sent samples to Miltenyi Biotec, CA, for gene and miRNA expression microarray profiling. Briefly, total RNA isolation for tumorous and adjacent non-tumorous tissue samples was performed using the standard RNA extraction protocols (Trizol). We assessed total RNA integrity using the Agilent 2100 Bioanalyzer platform (Agilent Technologies), and only samples with RNA integrity number (RIN) >6 were profiled for gene and miRNA expression^59^.

For gene expression profiling, we used 1 μg of each total RNA sample as starting material. We amplified and labeled total RNA samples using the Agilent Low RNA Input Linear Amp Kit (Agilent Technologies) following the manufacturer’s protocol. We measured cRNA yields and dye-incorporation rates with the ND-1000 Spectrophotometer (NanoDrop Technologies). We labeled non-tumorous samples with Cy3 and tumorous samples with Cy5. We performed hybridization according to Agilent’s 60-mer oligo microarray processing protocol using the Agilent Gene Expression Hybridization Kit (Agilent Technologies). Briefly, we combined and hybridized 825 ng of the corresponding Cy3- and Cy5-labeled fragmented cRNA overnight (17 hours, 65 °C) to Agilent Whole Human Genome Oligo Microarrays 4 × 44K (G4112F). We washed microarrays with 6x SSPE buffer containing 0.005% N-lauroylsarcosine for 1 min at room temperature followed by a second wash with pre-heated 0.06x SSPE buffer (37 °C) containing 0.005% N-lauroylsarcosine for 1 min. The second washing step was performed with acetonitrile for 30 seconds. Finally, we detected fluorescence signals using Agilent’s DNA microarray scanner (Agilent Technologies).

For miRNA profiling, we labeled samples according to the undisclosed miRXplore™ user manual. We hybridized fluorescent-labeled samples to miRXplore™ microarrays using the a-Hyb™ hybridization station. We labeled non-tumorous samples with Hy3 and tumorous samples with Hy5. Finally, we detected fluorescence intensity signals using an Agilent laser scanner (Agilent Technologies).

All microarray expression profiles are publicly available on Gene Expression Omnibus (GEO, http://www.ncbi.nlm.nih.gov/geo/) through these GEO IDs: GSE62007 (miRNA profiles), GSE62043 (mRNA profiles), and GSE62044 (combined).

### Pre-processing gene expression profiles

We loaded raw data of gene expression profiles into R using the R/Bioconductor Limma package^60^. We corrected data for background noise (normexp)^61^, followed by within (loess) and between array (quantile) normalization^62^. We detected no batch effects between expression profiles using principal component analysis (PCA).

### Pre-processing miRNA expression profiles

We loaded the raw miRNA expression profiles into R using Limma. Similar to mRNA gene expression profiles, we background corrected and normalized miRNA raw data within and between arrays. Next, we corrected arrays for batch effects using ComBat^63^. We determined differentially expressed miRNAs between tumorous and adjacent non-tumorous tissues of 100 HCC patients using Limma. To account for multiple testing, we employed the Benjamini-Hochberg method to calculate FDR values^64^. MiRNAs with an absolute fold change greater than 1.5 and an FDR smaller than 0.05 were considered significantly differentially expressed.

### Targets of differentially expressed miRNAs

For every differentially expressed miRNA, we calculated the negative Spearman’s correlation coefficient between the miRNA and all genes in expression across tumorous and adjacent non-tumorous tissues. We ranked genes by their negative correlation with the miRNA. Next, we downloaded^65^ and used miRNA target predictions based on the MiRanda target prediction program as a gene set for enrichment analysis. For each miRNA, we detected enrichment of its predicted targets in the ranked list of genes through GSEA’s pre-ranked function (GSEA version 2.0.10)^18^. Similarly, we calculated enrichment scores for target predictions based on the MirTarget2 program^66^. Only miRNAs with a FDR < 10^–3^ in both programs, MiRanda and MirTarget2, were considered for further analysis. Next, we calculated target prediction scores for all genes using the MiRanda target prediction command line tool (version 3.3a)^17^. For that purpose, we downloaded mature miRNA sequences from miRBase^67^ (release 19) and 3’UTR exon sequences from the UCSC genome browser database^68^ (hg19). We normalized prediction scores to zero by subtracting the minimum score achieved by any gene. Next, we calculated the product between the negative correlation with the miRNA and the normalized target prediction score and used it as a ranking metric (Supplementary Tables S5 and S6).

Finally, based on this ranking metric, we identified biological themes for each miRNA using GSEA′s pre-ranked function. The KEGG (excluding human disease) gene sets from MSigDB were employed in our analyses. Then we summarized enrichment results by consolidating pathways to pathway groups and pathway groups to categories based on the KEGG orthology conceptual hierarchy, implemented in the Bioconductor keggorthology package (version 2.1.0). We ranked pathway groups by their total number of underlying pathways, and we considered up- and down-regulated miRNAs separately. For ties, we ranked groups with at least one pathway enriched in all three up-regulated miRNAs, down-regulated respectively, and then more significant FDRs higher. We rearranged categories according to the highest-ranking pathway group in up-regulated miRNAs.

### Gene networks with functional annotation

We collected genes for each pathway group across three up-regulated miRNAs and three down-regulated respectively. We only considered differentially expressed genes of the GSEA core enrichment set. IPA was employed to infer regulatory networks and functional annotation for differentially expressed genes associated with selected pathways in each category in Table 2.

### Association of differentially expressed miRNAs and their targets with clinico-pathological features

MiRNAs and their relevant MiRanda predicted targets with significant differential expression (absolute FC > 1.5, FDR < 0.05) were associated with clinical features, namely gender, age, hepatitis infection status, liver cirrhosis, stage, grade, size, relapse status, encapsulation, and Alpha Fetoprotein (AFP) levels. We evaluated association through the log-rank test and Cox’s proportional hazards model (relapse status) or the Kruskal-Wallis rank sum test (all other clinical parameters). We obtained the genes’ functional annotation through IPA.

## Additional Information

**How to cite this article**: Thurnherr, T. *et al*. Differentially Expressed miRNAs in Hepatocellular Carcinoma Target Genes in the Genetic Information Processing and Metabolism Pathways. *Sci. Rep.* **6**, 20065; doi: 10.1038/srep20065 (2016).

## Supplementary Material

Supplementary Information

## Figures and Tables

**Table 1:** Summary of Differentially Expressed miRNA potentially playing a role in HCC.

**MiRNA**	**HCC vs. non-tumorous tissue**		**GSEA FDR**		**Significant in Both**^1^
	**Fold change**	**FDR**	**MiRanda**	**MirTarget2**	
*miR-21*	2.29	1.33 × 10^–17^	<5.0 × 10^–4^	<6.0 × 10^–4^	Yes
*miR-221*	1.55	2.55 × 10^–10^	<5.0 × 10^–4^	<6.0 × 10^–4^	Yes
*miR-93*	1.53	2.16 × 10^–16^	<5.0 × 10^–4^	<6.0 × 10^–4^	Yes
*miR-663a*	1.64	9.85 × 10^–06^	<5.0 × 10^–4^	2.2 × 10^–1^	No
*miR-122*	−1.67	1.50 × 10^–05^	<5.0 × 10^–4^	<6.0 × 10^–4^	Yes
*miR-26a*	−1.59	7.71 × 10^–10^	<5.0 × 10^–4^	2.8 × 10^–3^	Yes
*miR-130a*	−1.56	1.14 × 10^–12^	<5.0 × 10^–4^	8.0 × 10^–3^	Yes
*miR-195*	−1.61	8.41 × 10^–11^	5.5 × 10^–3^	2.2 × 10	No
*miR-30a*	−1.51	1.13 × 10^–38^	6.9 × 10^–1^	<6.0 × 10^–4^	No

^1^Only miRNAs that showed statistically significant enrichment against both target prediction programs (MiRanda and MirTarget2, GSEA FDR < 10^–3^) were considered for further analysis.

**Table 2:** Pathways enriched in miRNA targets.

**Category**	**Pathway group**	**Up-regulated miRNA**				**Down-regulated miRNA**			
		**miR-21**	**miR-93**	**miR-221**	**Rank**	**miR-26a**	**miR-122**	**miR-130a**	**Rank**
Metabolism (M.)	Amino Acid M.	10 (<0.01)	11 (<0.01–0.11)	11 (<0.01–0.05)	1	*—*	1 (0.18)	*—*	10
	Carbohydrate M.	9 (<0.01–0.07)	8 (<0.01–0.08)	9 (<0.01–0.13)	3	*—*	*—*	*—*	
	Lipid M.	7 (<0.01–0.09)	5 (<0.01–0.09)	5 (<0.01–0.11)	4	1 (0.1)	*—*	*—*	9
	Xenobiotics Biodegradation & M.	3 (<0.01)	3 (<0.01)	3 (<0.01)	6	*—*	*—*	*—*	
	M. of Cofactors & Vitamins	3 (<0.01–0.07)	2 (<0.01–0.03)	2 (<0.01–0.12)	8	*—*	*—*	*—*	
	Energy M.	1 (0.01)	1 (0.05)	1 (0.02)	12	*—*	*—*	*—*	
	Glycan Biosynthesis & M.	*—*	*—*	1 (0.13)	16	1 (0.14)	1 (0.13)	1 (0.12)	7
	Nucleotide M.	*—*	*—*	*—*		2 (0.02–0.11)	1 (0.16)	2 (0.03–0.19)	4
Organismal Systems	Immune System	8 (<0.01–0.12)	12 (<0.01–0.2)	11 (<0.01–0.17)	2	*—*	*—*	*—*	
	Endocrine System	3 (<0.01–0.1)	2 (0.01–0.03)	3 (0.01–0.16)	7	*—*	*—*	*—*	
	Circulatory System	1 (0.07)	1 (0.13)	1 (0.17)	13	*—*	*—*	*—*	
	Development	*—*	2 (0.09–0.12)	1 (0.02)	14	*—*	*—*	*—*	
	Nervous System	1 (0.19)	*—*	1 (0.16)	15	*—*	*—*	*—*	
Environmental Information Processing	Signal Transduction	3 (0.04–0.18)	4 (0.08–0.2)	4 (0.06–0.2)	5	*—*	*—*	*—*	
	Signaling Molecules and Interaction	3 (0.05–0.08)	3 (0.02–0.12)	1 (0.1)	9	*—*	*—*	*—*	
	Membrane Transport	*—*	*—*	1 (0.17)	17	1 (0.01)	1 (0.1)	1 (0.02)	6
Cellular Processes	**Cell Growth and Death**	**2 (**<**0.01**–**0.07)**	**2** (**0.04**–**0.09)**	**2** (**0.01**–**0.12)**	**10**	**2** (**0.02**–**0.09)**	**2** (**0.17)**	**2** (**0.03**–**0.1)**	**2**
	Transport and Catabolism	1 (<0.01)	1 (0.08)	1 (0.01)	13	*—*	*—*	*—*	
	Cell Communication	*—*	2 (0.08–0.12)	2 (0.11–0.2)	11	*—*	*—*	*—*	
Genetic Information Processing	Translation	*—*	*—*	*—*		1 (0.18)	2 (0.08–0.15)	—	8
	Folding, Sorting and Degradation	*—*	*—*	*—*		2 (0.04–0.17)	1 (0.11)	2 (0.12–0.18)	5
	Transcription	*—*	*—*	*—*		2 (0.01–0.06)	1 (0.11)	2 (0.02–0.15)	3
	Replication and Repair	*—*	*—*	*—*		5 (<0.01–0.07)	2 (0.07–0.1)	4 (<0.01–0.15)	1

Number of pathways enriched under each pathway group for every miRNA. Enrichment FDR ranges are given in brackets. Groups were ranked by the number of pathways enriched across up-regulated miRNAs, down-regulated respecively. The category with the highest ranking group was listed first. For ties, pathways in all miRNAs and then lower FDRs were cosidered first.

**Table 3:** Detail of Pathway group “Cell growth and death” enriched in miRNA Targets.

Pathway	**FDR up-regulated miRNA**			**FDR down-regulated miRNA**		
	**miR-21**	**miR-93**	**miR-221**	**miR-26a**	**miR-122**	**miR-130a**
P53 signaling pathway	<0.01	0.04	0.01	0.09	0.17	0.1
Apoptosis	0.07	0.09	0.12	*—*	*—*	*—*
Cell cycle	*—*	*—*	*—*	0.02	0.17	0.03

**Table 4:**
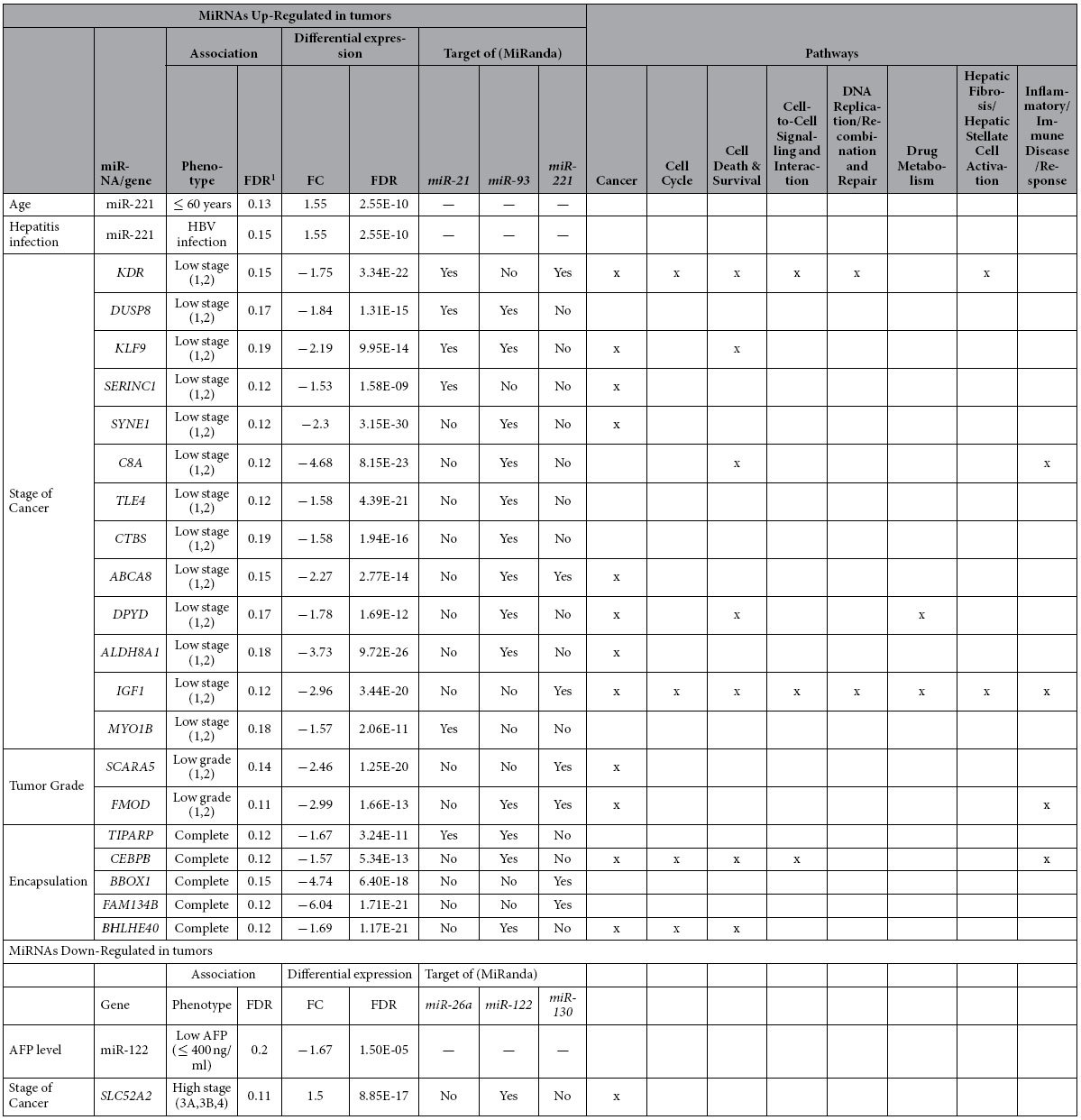
Association of relevant miRNAs and their differentially expressed targets with clinical characteristics.

^1^Association FDR based on differentially expressed miRNAs or targets (absolute FC > 1.5; FDR < 0.05; GSEA core enrichment set). These are 6 relevant miRNAs, 148 down-regulated (targets of upregulated miRNAs), and 517 up-regulated (targets of down-regulated miRNAs) genes.

**Table 5:** Demographic and clinical information of 100 HCC patients.

Age, y (median, range)	63 (34, 84)
>60	56
≤60	44
Gender	
Male	84
Female	16
Viral infection	
HBV	58
HCV	8
None	27
Unknown	7
Edmondson grade	
Low (1, 2)	40
High (3, 4)	47
Unknown	13
Stage	
Low (1, 2)	57
High (3A, 3B, 4)	21
Unknown	22
Cirrhosis	
No	47
Yes	46
Unknown	7
Tumorsize	
>5cm	56
≤5cm	41
Unknown	3
Relapse	
Yes	30
No	49
Unknown	21
Encapsulation	
Complete	19
Incomplete	25
Unknown	56
AFP level (ng/ml)	
≤400	45
>400	33
Unknown	22
