# Supplementary material for: Differentially Expressed miRNAs in Hepatocellular Carcinoma Target Genes in the Genetic Information Processing and Metabolism Pathways
Source: Sci Rep. 2016 Jan 28;6:20065. doi: 10.1038/srep20065 (PMC4730185; doi:10.1038/srep20065)
Supplement: Supplementary Information [file srep20065-s1.pdf]

Supplementary Information

Differentially Expressed miRNAs in Hepatocellular Carcinoma Target Genes in the Genetic Information Processing and Metabolism Pathways

Thomas Thurnherr[1], Way-Champ Mah[2], Zhengdeng Lei[3], Yu Jin[1,2], Steven G. Rozen[1,4] and Caroline G. Lee[1,2,4,5,*]

[1]NUS Graduate School for Integrative Sciences and Engineering, National University of Singapore, Singapore
[2]Division of Medical Sciences, Humphrey Oei Institute of Cancer Research, National Cancer Centre Singapore, Singapore 169610, Singapore
[3]Program in Neuroscience and Behavioural Disorders, Duke-NUS Graduate Medical School, Singapore
[4]Cancer and Stem Cell Biology Program, Duke-NUS Graduate Medical School Singapore, Singapore 169547, Singapore
[5]Department of Biochemistry, Yong Loo Lin School of Medicine, National University of Singapore, Singapore 119077, Singapore

* Corresponding author: Tel: +65-6436-8353; Email: caroline_lee@nuhs.edu.sg

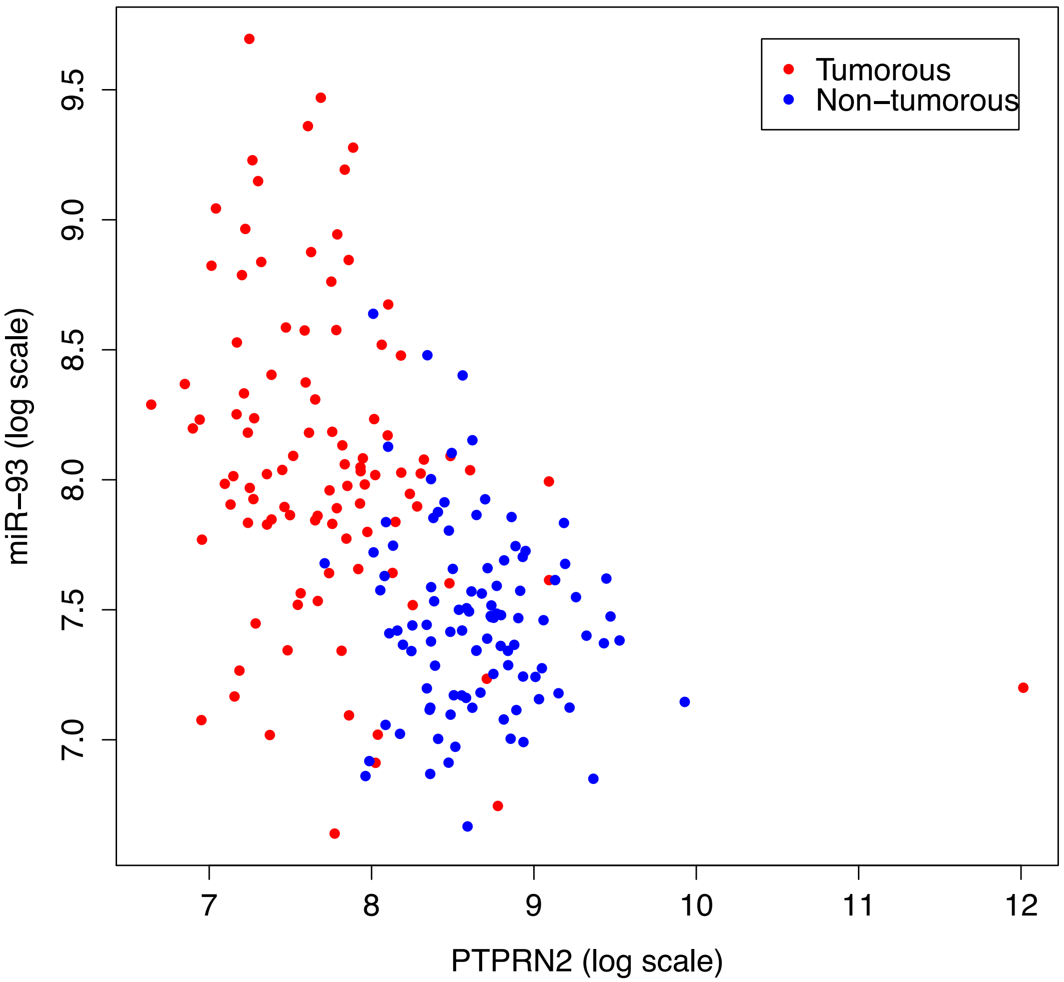

Supplementary Figure S1 - Example of negative correlation: *miR-93* and *PTPRN2*

Expression of *miR-93* and *PTPRN2* across tumorous and adjacent non-tumorous tissues are negatively correlated (Spearman's *rho=-0.53*). The plot also illustrates that *miR-93* is over-expressed (FC: 1.53; FDR: 2.16×10^{-16}) and *PTPRN2* is under-expressed (FC: 0.53; FDR: 1.89×10^{-21}) in tumors.

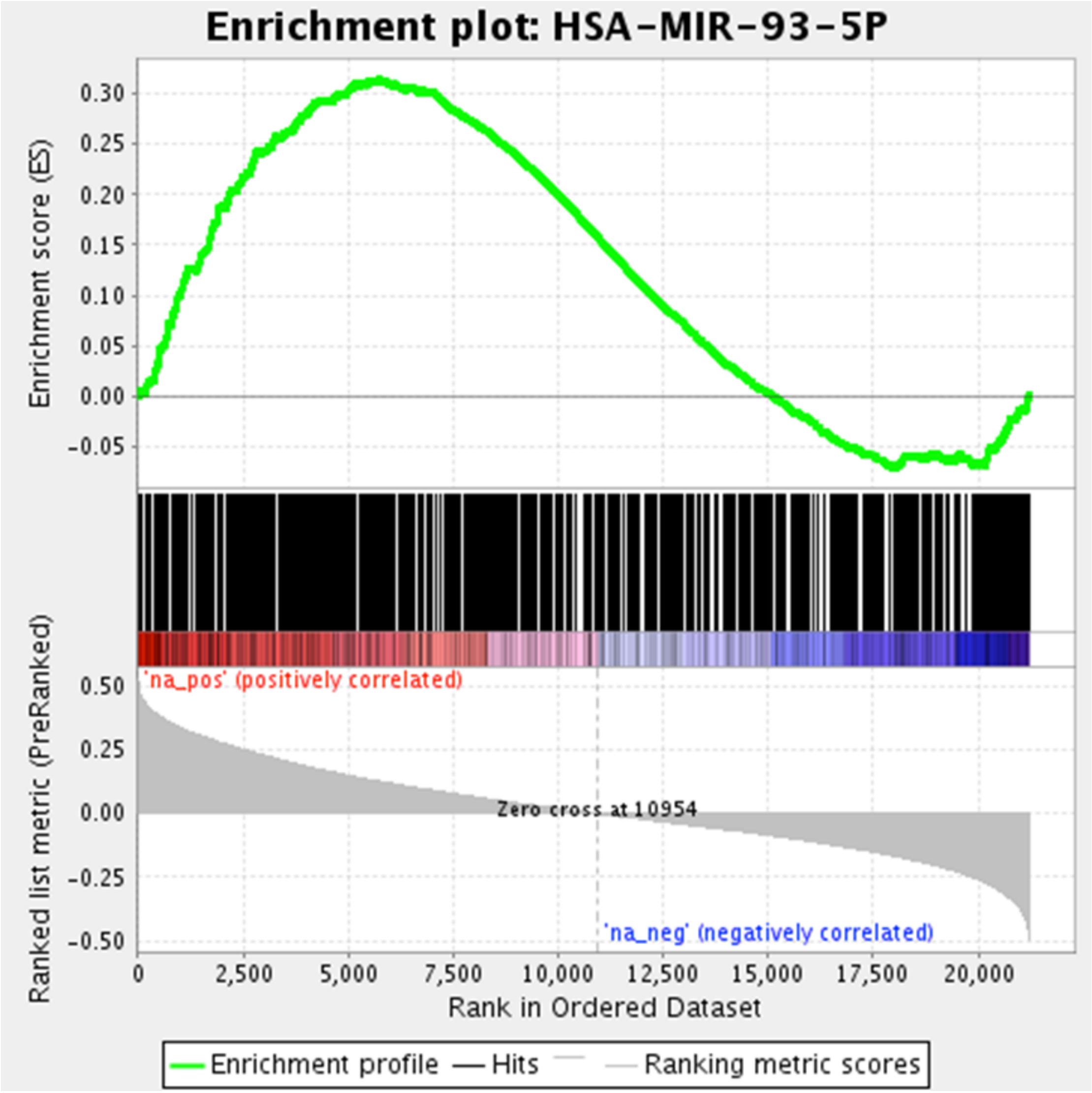

Supplementary Figure S2 - GSEA enrichment plot

Negatively correlated genes with miR-93 are over-represented in MirTarget2 predicted targets (FDR < 6.0×10^{-4})

Down-regulated miRNAs

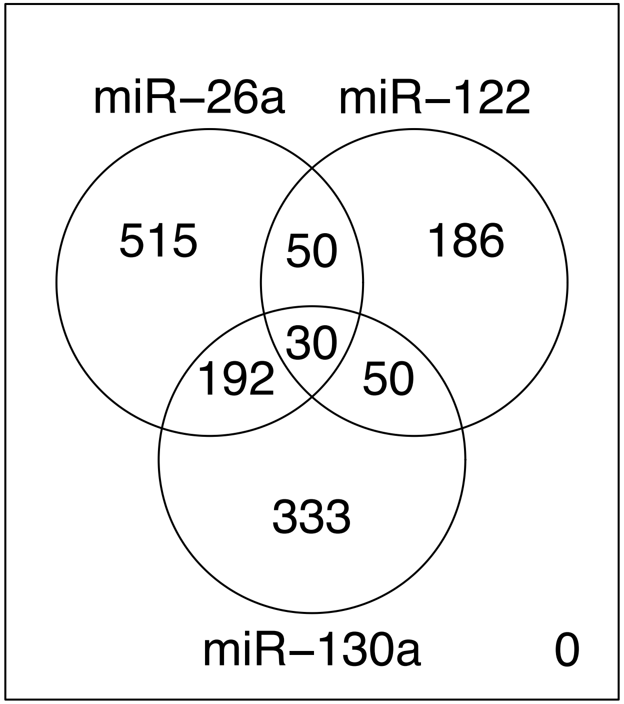

Up-regulated miRNAs

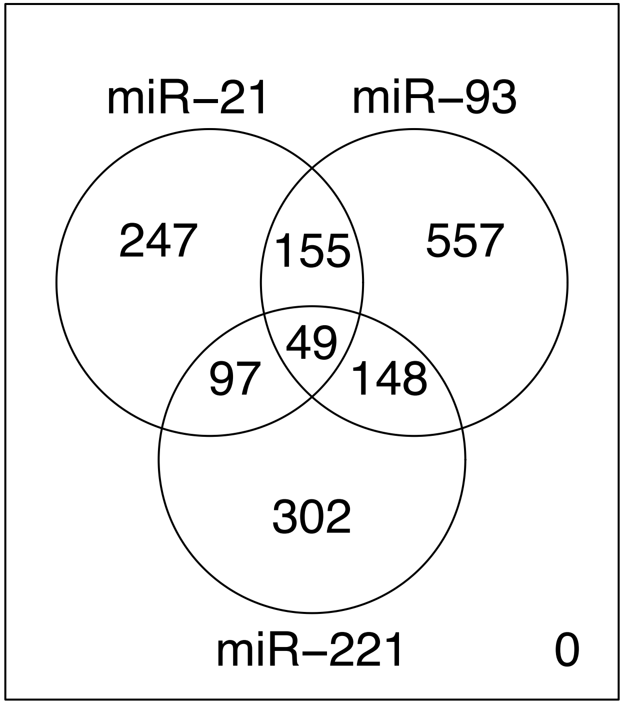

Supplementary Figure S3 - Common targets of differentially expressed miRNAs (GSEA core enrichment set)

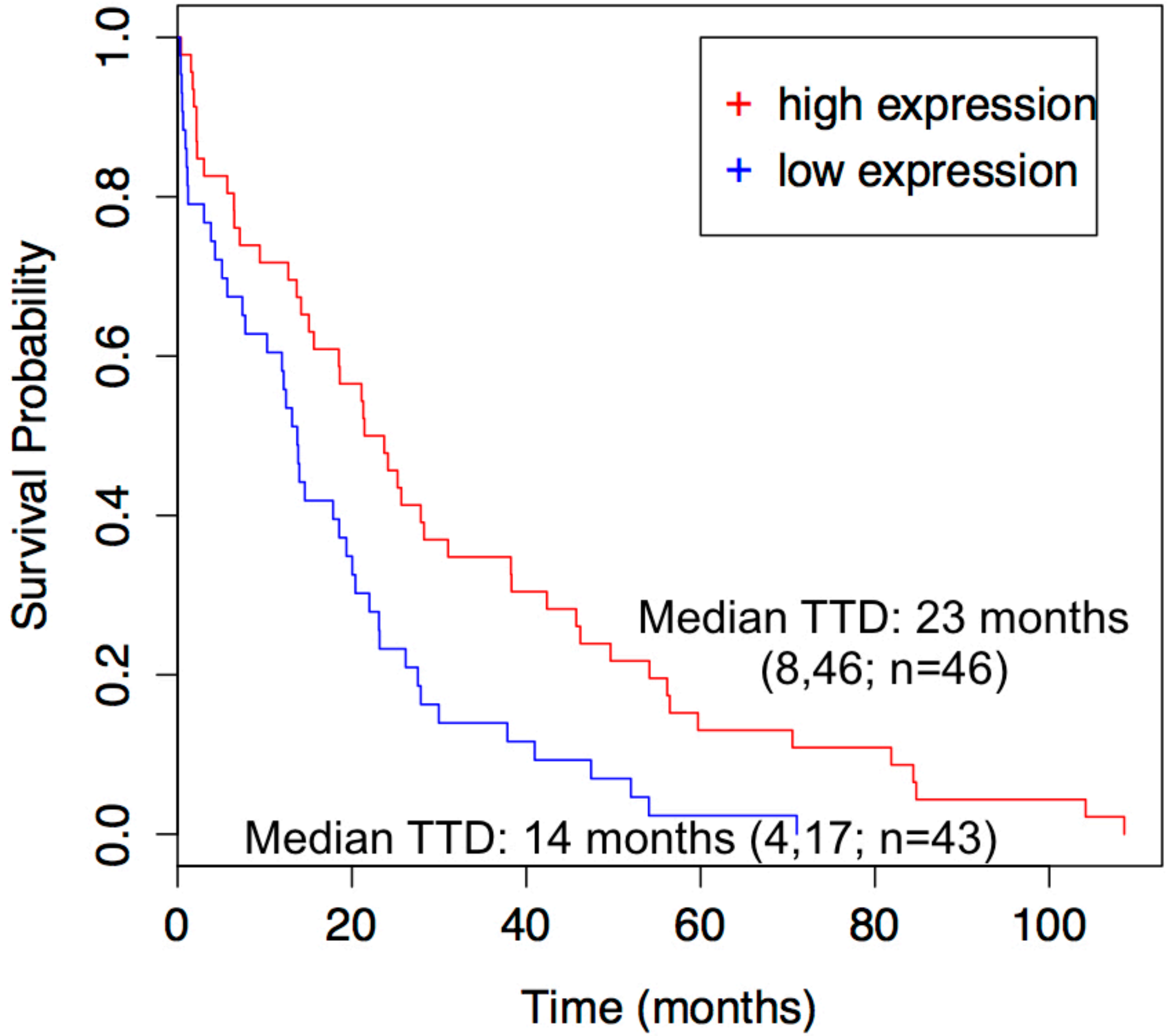

Supplementary Figure S4 – Patients with high miR-130a expression in tumor show significantly better survival

TCGA liver cancer data; log-rank test: p=0.003; cutoff: median miR-130a expression; Median TTD: median time-to-death in months (1st,3rd quartile; number of events).

Table S1. Differentially expressed miRNAs between tumorous (T) and adjacent non-tumorous tissues (NT).

miRNA	T vs. NT Fold change	FDR
miR-199a-5p	-4.51	2.07E-24
miR-199a-3p	-2.78	7.22E-18
miR-145-5p	-2.39	2.32E-14
miR-21-5p	2.29	1.33E-17
miR-451a	-2.19	1.20E-08
miR-125b-5p	-2.13	5.08E-17
miR-223-3p	-1.92	2.76E-18
miR-99a-5p	-1.90	1.90E-16
miR-143-3p	-1.88	1.81E-09
miR-1469	1.80	6.11E-09
miR-486-5p	-1.77	1.27E-21
miR-122-5p	-1.67	1.50E-05
miR-125a-5p	-1.65	1.83E-11
miR-663a	1.64	9.85E-06
miR-1224-3p	-1.63	1.99E-32
miR-296-5p	-1.62	1.68E-17
miR-195-5p	-1.61	8.41E-11
miR-26a-5p	-1.60	7.71E-10
miR-1274a	1.59	9.26E-14
miR-302b-5p	-1.59	4.01E-48
miR-130a-3p	-1.55	1.14E-12
miR-221-3p	1.55	2.55E-10
miR-1236	-1.54	1.29E-51
miR-302a-5p	-1.54	9.68E-45
miR-33a-5p	-1.54	6.15E-45
miR-93-5p	1.53	2.16E-16
miR-186-3p	-1.53	1.13E-40
miR-374b-3p	-1.53	6.15E-45
miR-150-5p	-1.51	2.42E-09
miR-922	-1.51	8.91E-43
miR-30a-3p	-1.51	1.13E-38
miR-378a-3p	-1.51	2.77E-10

Table S2. Correlation between shortlisted miRNAs across tumor and adjacent non-tumorous tissues

Correlation coefficient (upper triangle) / P-value (lower triangle)

	miR-21	miR-93	miR-221	miR-26a	miR-122	miR-130a
**miR-21**		0	0.26	-0.21	-0.29	-0.09
**miR-93**	0.9686		**0.6**	0	-0.16	-0.19
**miR-221**	0.0002	**$<10^{-4}$**		-0.02	-0.22	-0.08
**miR-26a**	0.0024	0.9448	0.734		0.19	**0.56**
**miR-122**	$<10^{-4}$	0.0211	0.0015	0.0088		0.04
**miR-130a**	0.1941	0.0081	0.2326	**$<10^{-4}$**	0.2326	

Table S3. Relevant up-regulated targets of down-regulated miRNAs in HCC tumors

				miR-26a			*miR-122*			*miR-130a*		
	Name	FC	FDR	Corr	MiRanda Pred	MirTarget Pred	Corr	MiRanda Pred	MirTarget Pred	Corr	MiRanda Pred	MirTarget Pred
miR-26 & miR-122	FLVCR1	2.26	6.81E-23	0.3	TRUE	FALSE	0.23	TRUE	FALSE	0.36	FALSE	FALSE
	XPR1	1.52	1.07E-20	0.27	TRUE	FALSE	0.23	TRUE	FALSE	0.42	FALSE	FALSE
	SHCBP1	1.58	4.56E-17	0.27	TRUE	FALSE	0.26	TRUE	TRUE	0.38	FALSE	FALSE
	DLAT	1.52	5.25E-16	0.23	TRUE	FALSE	0.26	TRUE	FALSE	0.29	FALSE	FALSE
	PHF6	1.6	7.63E-14	0.31	TRUE	FALSE	0.29	TRUE	FALSE	0.42	FALSE	FALSE
	FERMT1	1.54	5.19E-06	0.14	TRUE	FALSE	0.16	TRUE	FALSE	0.24	FALSE	FALSE
miR-26 & miR-130a	TOP2A	3.05	5.91E-28	0.28	TRUE	FALSE	0.22	FALSE	FALSE	0.39	TRUE	FALSE
	UBE2O	1.53	3.43E-25	0.27	TRUE	FALSE	0.3	FALSE	FALSE	0.26	TRUE	FALSE
	FANCA	1.6	2.84E-19	0.27	TRUE	FALSE	0.27	FALSE	FALSE	0.28	TRUE	TRUE
	RRAGD	1.94	8.67E-18	0.3	TRUE	FALSE	0.15	FALSE	FALSE	0.33	TRUE	TRUE
	IMPAD1	1.52	2.37E-17	0.37	TRUE	TRUE	0.24	FALSE	FALSE	0.44	TRUE	FALSE
	DLG5	1.88	4.68E-16	0.29	TRUE	TRUE	0.22	FALSE	FALSE	0.37	TRUE	FALSE
	E2F7	1.58	7.60E-16	0.16	TRUE	TRUE	0.08	FALSE	FALSE	0.3	TRUE	TRUE
	PLCB1	1.58	2.61E-15	0.15	TRUE	TRUE	0.23	FALSE	FALSE	0.35	TRUE	TRUE
	PLCB1	1.9	1.56E-14	0.16	TRUE	TRUE	0.26	FALSE	FALSE	0.38	TRUE	TRUE
	MAP2	1.67	1.18E-13	0.12	TRUE	TRUE	0.21	FALSE	FALSE	0.23	TRUE	FALSE
	SMYD2	1.66	5.56E-12	0.26	TRUE	FALSE	0.23	FALSE	FALSE	0.27	TRUE	FALSE
	XK	2.03	2.71E-11	0.14	TRUE	TRUE	0.14	FALSE	FALSE	0.27	TRUE	FALSE
	SPOCK1	1.72	1.30E-08	0.1	TRUE	FALSE	0.14	FALSE	FALSE	0.24	TRUE	TRUE
	SOX2	1.5	5.89E-08	0.13	TRUE	FALSE	-0.02	FALSE	FALSE	0.27	TRUE	FALSE
	SPARCL1	1.96	4.10E-05	0.13	TRUE	FALSE	0.14	FALSE	FALSE	0.26	TRUE	FALSE
miR-26	CDKN3	3.91	9.89E-38	0.33	TRUE	FALSE	0.23	FALSE	FALSE	0.45	FALSE	FALSE
	EZH2	2.75	1.77E-32	0.35	TRUE	TRUE	0.23	FALSE	FALSE	0.45	FALSE	FALSE
	CHAF1A	1.8	9.34E-29	0.23	TRUE	FALSE	0.16	FALSE	FALSE	0.3	FALSE	FALSE
	CSTF2	1.59	4.21E-26	0.26	TRUE	FALSE	0.19	FALSE	FALSE	0.36	FALSE	FALSE
	TPX2	2.18	6.57E-26	0.25	TRUE	FALSE	0.2	FALSE	FALSE	0.37	FALSE	FALSE
	MLF1IP	2.52	1.09E-25	0.31	TRUE	FALSE	0.21	FALSE	FALSE	0.36	FALSE	FALSE
	FEN1	1.75	1.97E-25	0.22	TRUE	FALSE	0.17	FALSE	FALSE	0.34	FALSE	FALSE
	GPSM2	1.96	6.60E-25	0.31	TRUE	TRUE	0.23	FALSE	FALSE	0.44	FALSE	FALSE
	KPNA2	2.03	1.87E-24	0.28	TRUE	TRUE	0.27	FALSE	FALSE	0.35	FALSE	FALSE
	FANCF	1.72	5.63E-24	0.21	TRUE	FALSE	0.26	FALSE	FALSE	0.28	FALSE	FALSE
	CDC6	2.37	5.68E-24	0.31	TRUE	FALSE	0.26	FALSE	FALSE	0.42	FALSE	FALSE
	TTK	1.96	7.68E-24	0.31	TRUE	FALSE	0.27	FALSE	FALSE	0.39	FALSE	FALSE
	MAD2L1	2.41	1.18E-23	0.31	TRUE	FALSE	0.24	FALSE	FALSE	0.4	FALSE	FALSE
	DPH2	1.72	4.07E-23	0.29	TRUE	FALSE	0.25	FALSE	FALSE	0.3	FALSE	FALSE
	TMEM38B	1.68	3.66E-22	0.26	TRUE	TRUE	0.13	FALSE	FALSE	0.42	FALSE	FALSE
	DARS2	1.78	6.44E-22	0.29	TRUE	FALSE	0.21	FALSE	FALSE	0.45	FALSE	FALSE
	CEP41	1.74	1.13E-21	0.36	TRUE	FALSE	0.3	FALSE	FALSE	0.41	FALSE	FALSE
	CKS2	2.09	2.16E-21	0.25	TRUE	TRUE	0.18	FALSE	FALSE	0.38	FALSE	FALSE
	ALG1	1.51	6.25E-21	0.23	TRUE	FALSE	0.14	FALSE	FALSE	0.31	FALSE	FALSE
	WDR12	1.55	9.30E-21	0.3	TRUE	FALSE	0.3	FALSE	FALSE	0.35	FALSE	FALSE
	RAD51AP1	1.87	1.98E-20	0.26	TRUE	FALSE	0.22	FALSE	FALSE	0.36	FALSE	FALSE
	CCNE1	1.85	8.34E-20	0.27	TRUE	FALSE	0.26	FALSE	FALSE	0.32	FALSE	FALSE
	ARHGAP11A	1.54	1.25E-19	0.23	TRUE	FALSE	0.21	FALSE	FALSE	0.37	FALSE	FALSE
	KIF18A	1.58	5.97E-19	0.28	TRUE	TRUE	0.24	FALSE	FALSE	0.41	FALSE	FALSE
	TMCO1	1.54	5.45E-18	0.19	TRUE	FALSE	0.22	FALSE	FALSE	0.35	FALSE	FALSE
	DCAF13	1.88	1.26E-17	0.29	TRUE	FALSE	0.29	FALSE	FALSE	0.39	FALSE	FALSE
	RHEB	1.55	1.76E-17	0.23	TRUE	FALSE	0.23	FALSE	FALSE	0.3	FALSE	FALSE
	ATAD2	1.88	1.82E-17	0.27	TRUE	FALSE	0.21	FALSE	FALSE	0.38	FALSE	FALSE
	SPA17	1.67	4.90E-17	0.28	TRUE	FALSE	0.23	FALSE	FALSE	0.24	FALSE	FALSE
	NMB	2.23	5.06E-17	0.22	TRUE	FALSE	0.18	FALSE	FALSE	0.35	FALSE	FALSE
	MANEAL	1.73	1.05E-16	0.29	TRUE	FALSE	0.07	FALSE	FALSE	0.24	FALSE	FALSE
	GINS4	1.52	1.86E-16	0.23	TRUE	FALSE	0.17	FALSE	FALSE	0.25	FALSE	FALSE
	TTC13	1.57	2.40E-16	0.33	TRUE	TRUE	0.21	FALSE	FALSE	0.39	FALSE	FALSE
	HCN3	1.5	5.24E-16	0.16	TRUE	FALSE	0.09	FALSE	FALSE	0.22	FALSE	FALSE
	SULT1C2	2.41	1.74E-14	0.2	TRUE	FALSE	0.29	FALSE	FALSE	0.23	FALSE	FALSE
	PHYHIPL	1.58	2.15E-14	0.17	TRUE	TRUE	0.1	FALSE	FALSE	0.24	FALSE	FALSE
	GTF2I	1.58	4.98E-14	0.22	TRUE	FALSE	0.23	FALSE	FALSE	0.32	FALSE	FALSE
	TMEM33	1.52	7.26E-14	0.18	TRUE	TRUE	0.21	FALSE	FALSE	0.32	FALSE	FALSE
	LYPD1	2.45	2.09E-13	0.16	TRUE	FALSE	0.11	FALSE	FALSE	0.25	FALSE	FALSE
	PPP2R5A	1.57	8.98E-13	0.25	TRUE	FALSE	0.19	FALSE	FALSE	0.35	FALSE	FALSE
	FAM49B	1.66	7.56E-12	0.29	TRUE	FALSE	0.28	FALSE	FALSE	0.32	FALSE	FALSE
	GOLM1	1.66	1.15E-11	0.11	TRUE	FALSE	0.19	FALSE	FALSE	0.17	FALSE	FALSE
	TMCC1	1.6	1.94E-11	0.24	TRUE	TRUE	0.18	FALSE	FALSE	0.31	FALSE	FALSE
	CHML	1.65	1.73E-10	0.26	TRUE	FALSE	0.19	FALSE	FALSE	0.37	FALSE	FALSE
	FANCE	1.52	3.01E-10	0.19	TRUE	FALSE	0.13	FALSE	FALSE	0.21	FALSE	FALSE
	TMCO3	1.65	9.88E-10	0.21	TRUE	FALSE	0.26	FALSE	FALSE	0.18	FALSE	FALSE
	TPD52	1.52	5.90E-09	0.23	TRUE	FALSE	0.19	FALSE	FALSE	0.27	FALSE	FALSE
	DYNC1I1	1.81	8.72E-09	0.17	TRUE	FALSE	0.18	FALSE	FALSE	0.21	FALSE	FALSE
	GREB1	1.59	1.06E-08	0.13	TRUE	FALSE	0.14	FALSE	FALSE	0.2	FALSE	FALSE
	MNS1	1.73	1.54E-08	0.21	TRUE	FALSE	0.09	FALSE	FALSE	0.31	FALSE	FALSE

	COL4A1	1.61	4.02E-08	0.19	TRUE	FALSE	0.26	FALSE	FALSE	0.2	FALSE	FALSE
	LEF1	1.83	5.00E-06	0.2	TRUE	FALSE	0.27	FALSE	FALSE	0.19	FALSE	FALSE
	SFRP4	1.67	7.74E-06	0.14	TRUE	FALSE	0.15	FALSE	FALSE	0.04	FALSE	FALSE
**miR-122 & miR-130a**	ABCC5	1.54	2.14E-18	0.3	FALSE	FALSE	0.3	TRUE	FALSE	0.28	TRUE	FALSE
	NUP43	1.52	2.40E-18	0.25	FALSE	FALSE	0.3	TRUE	FALSE	0.29	TRUE	FALSE
	LAMC1	1.7	1.22E-13	0.27	FALSE	FALSE	0.36	TRUE	FALSE	0.28	TRUE	FALSE
	RGS5	1.64	8.67E-10	0.17	FALSE	FALSE	0.26	TRUE	FALSE	0.25	TRUE	FALSE
**miR-122**	CENPP	1.93	2.98E-27	0.36	FALSE	FALSE	0.26	TRUE	FALSE	0.42	FALSE	FALSE
	KIF2C	1.96	7.29E-27	0.28	FALSE	FALSE	0.12	TRUE	FALSE	0.33	FALSE	FALSE
	CENPM	1.7	6.31E-25	0.25	FALSE	FALSE	0.17	TRUE	FALSE	0.34	FALSE	FALSE
	ILF2	1.65	3.66E-22	0.34	FALSE	FALSE	0.29	TRUE	FALSE	0.33	FALSE	FALSE
	PSPH	1.88	4.90E-21	0.03	FALSE	FALSE	0.24	TRUE	FALSE	0.24	FALSE	FALSE
	YKT6	1.56	4.94E-21	0.25	FALSE	FALSE	0.22	TRUE	TRUE	0.35	FALSE	FALSE
	KIF11	1.63	2.16E-19	0.31	FALSE	FALSE	0.23	TRUE	TRUE	0.36	FALSE	FALSE
	NCBP2	1.53	4.15E-19	0.36	FALSE	FALSE	0.29	TRUE	FALSE	0.29	FALSE	FALSE
	NME2P1	1.62	1.46E-17	0.27	FALSE	FALSE	0.33	TRUE	FALSE	0.31	FALSE	FALSE
	COL15A1	1.98	1.47E-17	0.26	FALSE	FALSE	0.29	TRUE	FALSE	0.31	FALSE	FALSE
	CACYBP	1.6	1.75E-17	0.28	FALSE	FALSE	0.22	TRUE	FALSE	0.41	FALSE	FALSE
	SLC52A2	1.5	8.85E-17	0.29	FALSE	FALSE	0.2	TRUE	TRUE	0.22	FALSE	FALSE
	CD109	1.74	5.07E-16	0.23	FALSE	FALSE	0.19	TRUE	FALSE	0.37	FALSE	FALSE
	SNRPD1	1.5	6.79E-16	0.31	FALSE	FALSE	0.27	TRUE	FALSE	0.3	FALSE	FALSE
	TUBA1C	1.74	5.56E-15	0.22	FALSE	FALSE	0.26	TRUE	FALSE	0.25	FALSE	FALSE
	SORT1	1.66	7.81E-15	0.35	FALSE	FALSE	0.23	TRUE	FALSE	0.25	FALSE	FALSE
	SRXN1	1.87	1.55E-13	0.17	FALSE	FALSE	0.22	TRUE	FALSE	0.19	FALSE	FALSE
	ISX	2.29	2.37E-11	0.11	FALSE	FALSE	0.14	TRUE	FALSE	0.17	FALSE	FALSE
	HSPE1	1.55	2.52E-10	0.16	FALSE	FALSE	0.22	TRUE	FALSE	0.26	FALSE	FALSE
	IGF2BP1	1.95	4.53E-10	0.18	FALSE	FALSE	0.14	TRUE	FALSE	0.23	FALSE	FALSE
	MYCN	1.72	4.62E-09	0.17	FALSE	FALSE	0.13	TRUE	FALSE	0.28	FALSE	FALSE
	FADS1	1.8	6.45E-06	0.11	FALSE	FALSE	0.14	TRUE	FALSE	0.2	FALSE	FALSE
**miR-130a**	NCAPG	4.74	2.84E-33	0.29	FALSE	FALSE	0.25	FALSE	FALSE	0.42	TRUE	FALSE
	NR2C2AP	1.69	6.26E-32	0.29	FALSE	FALSE	0.33	FALSE	FALSE	0.31	TRUE	FALSE
	CDCA5	3.44	1.08E-31	0.31	FALSE	FALSE	0.24	FALSE	FALSE	0.4	TRUE	FALSE
	PDZD11	1.54	8.39E-31	0.26	FALSE	FALSE	0.15	FALSE	FALSE	0.38	TRUE	TRUE
	FAM83D	3.24	4.00E-29	0.3	FALSE	FALSE	0.2	FALSE	FALSE	0.43	TRUE	FALSE
	PBK	3.38	1.64E-28	0.32	FALSE	FALSE	0.23	FALSE	FALSE	0.43	TRUE	FALSE
	SNRPE	1.95	5.73E-28	0.35	FALSE	FALSE	0.27	FALSE	FALSE	0.44	TRUE	TRUE
	LAGE3	1.95	6.27E-28	0.26	FALSE	FALSE	0.19	FALSE	FALSE	0.42	TRUE	FALSE
	KIAA0101	3.2	1.03E-27	0.25	FALSE	FALSE	0.22	FALSE	FALSE	0.41	TRUE	FALSE
	SNX27	1.58	1.52E-24	0.35	FALSE	FALSE	0.28	FALSE	FALSE	0.33	TRUE	TRUE
	E2F8	2.09	3.06E-24	0.3	FALSE	FALSE	0.23	FALSE	FALSE	0.41	TRUE	FALSE
	E2F2	2.21	4.66E-24	0.28	FALSE	FALSE	0.21	FALSE	FALSE	0.34	TRUE	FALSE
	CDKN2AIPNL	1.62	1.15E-23	0.23	FALSE	FALSE	0.16	FALSE	FALSE	0.38	TRUE	FALSE
	PRIM2	1.64	1.90E-23	0.29	FALSE	FALSE	0.29	FALSE	FALSE	0.4	TRUE	FALSE
	CCNA2	1.59	4.99E-23	0.28	FALSE	FALSE	0.21	FALSE	FALSE	0.38	TRUE	FALSE
	GPR158	2.4	5.99E-23	0.27	FALSE	FALSE	0.27	FALSE	FALSE	0.39	TRUE	FALSE
	CDH13	2.16	1.78E-22	0.29	FALSE	FALSE	0.26	FALSE	FALSE	0.42	TRUE	FALSE
	CENPE	1.7	6.31E-22	0.25	FALSE	FALSE	0.19	FALSE	FALSE	0.34	TRUE	FALSE
	POC1A	1.8	8.64E-22	0.24	FALSE	FALSE	0.2	FALSE	FALSE	0.33	TRUE	FALSE
	MCM3AP-AS1	1.65	3.96E-21	0.35	FALSE	FALSE	0.23	FALSE	FALSE	0.34	TRUE	FALSE
	SQLE	3.76	5.81E-21	0.33	FALSE	FALSE	0.28	FALSE	FALSE	0.35	TRUE	FALSE
	PARPBP	2.03	6.74E-21	0.25	FALSE	FALSE	0.26	FALSE	FALSE	0.4	TRUE	FALSE
	TRIM45	2.07	1.33E-20	0.29	FALSE	FALSE	0.23	FALSE	FALSE	0.29	TRUE	FALSE
	RFX5	1.72	1.41E-20	0.2	FALSE	FALSE	0.2	FALSE	FALSE	0.33	TRUE	FALSE
	NCAPD2	1.77	1.46E-20	0.21	FALSE	FALSE	0.21	FALSE	FALSE	0.25	TRUE	FALSE
	POLQ	1.55	4.64E-20	0.21	FALSE	FALSE	0.16	FALSE	FALSE	0.34	TRUE	FALSE
	LIN9	1.74	2.29E-19	0.26	FALSE	FALSE	0.23	FALSE	FALSE	0.36	TRUE	FALSE
	TYMS	2.52	2.58E-19	0.25	FALSE	FALSE	0.18	FALSE	FALSE	0.35	TRUE	FALSE
	FAM222A	1.71	2.94E-18	0.15	FALSE	FALSE	0.13	FALSE	FALSE	0.25	TRUE	FALSE
	ESCO2	1.71	4.12E-18	0.28	FALSE	FALSE	0.25	FALSE	FALSE	0.35	TRUE	FALSE
	KIF23	1.62	1.34E-17	0.19	FALSE	FALSE	0.15	FALSE	FALSE	0.29	TRUE	FALSE
	UFD1L	1.51	2.91E-17	0.27	FALSE	FALSE	0.17	FALSE	FALSE	0.31	TRUE	TRUE
	DBF4	1.5	2.65E-16	0.32	FALSE	FALSE	0.24	FALSE	FALSE	0.37	TRUE	FALSE
	DSN1	1.57	4.51E-16	0.2	FALSE	FALSE	0.16	FALSE	FALSE	0.3	TRUE	FALSE
	TACC2	1.56	6.90E-16	0.23	FALSE	FALSE	0.09	FALSE	FALSE	0.41	TRUE	FALSE
	PARP1	1.51	1.64E-15	0.35	FALSE	FALSE	0.26	FALSE	FALSE	0.32	TRUE	FALSE
	NOX4	1.53	2.75E-15	0.32	FALSE	FALSE	0.33	FALSE	FALSE	0.35	TRUE	FALSE
	CCT6A	1.64	4.80E-15	0.24	FALSE	FALSE	0.26	FALSE	FALSE	0.33	TRUE	TRUE
	IGSF3	2.18	1.43E-14	0.19	FALSE	FALSE	0.19	FALSE	FALSE	0.27	TRUE	FALSE
	SLC12A7	1.65	3.52E-14	0.23	FALSE	FALSE	0.29	FALSE	FALSE	0.2	TRUE	FALSE
	LAMA3	2.17	1.34E-13	0.17	FALSE	FALSE	0.08	FALSE	FALSE	0.32	TRUE	FALSE
	SUB1	1.51	2.27E-13	0.23	FALSE	FALSE	0.27	FALSE	FALSE	0.26	TRUE	FALSE
	TRIM59	1.59	3.15E-13	0.21	FALSE	FALSE	0.26	FALSE	FALSE	0.25	TRUE	TRUE
	CDKN2B	1.5	1.05E-12	0.17	FALSE	FALSE	0.19	FALSE	FALSE	0.23	TRUE	FALSE
	ATP6V1C1	1.61	7.07E-11	0.31	FALSE	FALSE	0.25	FALSE	FALSE	0.4	TRUE	FALSE
	ANKRD29	1.67	1.24E-10	0.12	FALSE	FALSE	0.13	FALSE	FALSE	0.26	TRUE	FALSE
	LPGAT1	1.62	4.17E-10	0.28	FALSE	FALSE	0.19	FALSE	FALSE	0.35	TRUE	FALSE
	LPPR1	1.61	2.16E-07	0.13	FALSE	FALSE	0.12	FALSE	FALSE	0.18	TRUE	FALSE

Table S4. Relevant down-regulated targets of up-regulated miRNAs in HCC tumors

				miR-21			*miR-93*			*miR-221*		
	Name	FC	FDR	Corr	MiRanda Pred	MirTarget Pred	Corr	MiRanda Pred	MirTarget Pred	Corr	MiRanda Pred	MirTarget Pred
miR-21 & miR-93 & miR-221	RND3	-5.7	7.53E-31	0.36	TRUE	FALSE	0.48	TRUE	TRUE	0.4	TRUE	FALSE
	LRAT	-3.47	1.77E-30	0.38	TRUE	TRUE	0.46	TRUE	FALSE	0.36	TRUE	FALSE
	FAM13A	-3.33	3.25E-30	0.34	TRUE	TRUE	0.38	TRUE	FALSE	0.37	TRUE	FALSE
	MYCT1	-1.55	1.56E-27	0.3	TRUE	FALSE	0.45	TRUE	FALSE	0.31	TRUE	FALSE
	CPEB3	-2.07	1.97E-25	0.37	TRUE	TRUE	0.35	TRUE	TRUE	0.33	TRUE	TRUE
	LRRC55	-2.06	4.80E-22	0.36	TRUE	FALSE	0.39	TRUE	FALSE	0.31	TRUE	FALSE
	PLCXD3	-2.85	5.32E-21	0.24	TRUE	FALSE	0.43	TRUE	TRUE	0.28	TRUE	FALSE
	ZFPM2	-1.58	3.23E-17	0.32	TRUE	TRUE	0.37	TRUE	TRUE	0.29	TRUE	TRUE
	F2R	-1.82	5.38E-16	0.3	TRUE	FALSE	0.33	TRUE	TRUE	0.32	TRUE	FALSE
	PTPN13	-1.91	2.24E-13	0.2	TRUE	FALSE	0.34	TRUE	FALSE	0.26	TRUE	FALSE
	SSFA2	-1.64	5.98E-13	0.19	TRUE	TRUE	0.36	TRUE	TRUE	0.24	TRUE	FALSE
	SCD5	-1.82	1.62E-12	0.19	TRUE	FALSE	0.38	TRUE	FALSE	0.19	TRUE	TRUE
	ELL2	-1.72	4.85E-10	0.25	TRUE	FALSE	0.24	TRUE	TRUE	0.26	TRUE	FALSE
	RECK	-1.7	4.56E-09	0.29	TRUE	TRUE	0.26	TRUE	FALSE	0.21	TRUE	FALSE
miR-21 & miR-93	RNF125	-2.53	9.50E-28	0.28	TRUE	FALSE	0.43	TRUE	TRUE	0.26	FALSE	FALSE
	N4BP2L1	-2.92	2.23E-26	0.38	TRUE	FALSE	0.37	TRUE	FALSE	0.37	FALSE	FALSE
	ANKRD55	-1.9	2.31E-26	0.37	TRUE	FALSE	0.42	TRUE	FALSE	0.34	FALSE	FALSE
	KMO	-3.26	5.47E-26	0.35	TRUE	FALSE	0.38	TRUE	FALSE	0.34	FALSE	FALSE
	BDH2	-2.91	1.38E-23	0.35	TRUE	TRUE	0.34	TRUE	FALSE	0.39	FALSE	FALSE
	KCNJ10	-1.77	3.07E-23	0.29	TRUE	TRUE	0.38	TRUE	FALSE	0.25	FALSE	FALSE
	DCUN1D3	-1.67	6.54E-23	0.43	TRUE	FALSE	0.32	TRUE	TRUE	0.28	FALSE	FALSE
	SORL1	-3.08	2.80E-22	0.36	TRUE	FALSE	0.44	TRUE	TRUE	0.41	FALSE	FALSE
	CILP	-2.72	5.04E-22	0.33	TRUE	FALSE	0.34	TRUE	FALSE	0.24	FALSE	FALSE
	PDLIM5	-1.8	6.50E-22	0.39	TRUE	TRUE	0.34	TRUE	TRUE	0.35	FALSE	FALSE
	ASPN	-4.26	5.25E-20	0.27	TRUE	TRUE	0.4	TRUE	FALSE	0.3	FALSE	FALSE
	FGL2	-2.34	1.17E-19	0.28	TRUE	FALSE	0.39	TRUE	TRUE	0.29	FALSE	FALSE
	GIMAP5	-1.84	2.53E-19	0.29	TRUE	FALSE	0.37	TRUE	FALSE	0.26	FALSE	FALSE
	CNTN3	-2.14	4.58E-19	0.29	TRUE	FALSE	0.29	TRUE	TRUE	0.29	FALSE	FALSE
	PPP1R3B	-1.84	1.67E-18	0.32	TRUE	TRUE	0.29	TRUE	TRUE	0.24	FALSE	FALSE
	TAGAP	-1.88	7.29E-18	0.24	TRUE	FALSE	0.38	TRUE	TRUE	0.22	FALSE	FALSE
	EGR3	-1.9	2.36E-17	0.25	TRUE	FALSE	0.38	TRUE	FALSE	0.26	FALSE	FALSE
	CYTIP	-2.49	4.73E-17	0.23	TRUE	FALSE	0.33	TRUE	FALSE	0.23	FALSE	FALSE
	IL1RAP	-2.41	7.05E-17	0.2	TRUE	FALSE	0.35	TRUE	FALSE	0.32	FALSE	FALSE
	BTG2	-2.15	1.00E-16	0.25	TRUE	FALSE	0.36	TRUE	FALSE	0.15	FALSE	FALSE
	DUSP10	-1.81	6.81E-16	0.24	TRUE	FALSE	0.34	TRUE	FALSE	0.25	FALSE	FALSE
	ZFP36L1	-1.6	6.84E-16	0.37	TRUE	FALSE	0.41	TRUE	FALSE	0.39	FALSE	FALSE
	STRADB	-1.56	1.05E-15	0.29	TRUE	FALSE	0.24	TRUE	FALSE	0.18	FALSE	FALSE
	DUSP8	-1.84	1.31E-15	0.3	TRUE	TRUE	0.28	TRUE	TRUE	0.22	FALSE	FALSE
	LUM	-5.6	1.53E-15	0.23	TRUE	TRUE	0.34	TRUE	FALSE	0.23	FALSE	FALSE
	CD69	-2.81	1.88E-15	0.26	TRUE	TRUE	0.33	TRUE	TRUE	0.23	FALSE	FALSE
	ALDH9A1	-1.59	1.20E-14	0.34	TRUE	FALSE	0.28	TRUE	FALSE	0.27	FALSE	FALSE
	INTS6	-1.54	3.45E-14	0.29	TRUE	FALSE	0.36	TRUE	TRUE	0.33	FALSE	FALSE
	KLF9	-2.19	9.95E-14	0.35	TRUE	FALSE	0.29	TRUE	FALSE	0.34	FALSE	FALSE
	MSR1	-1.83	9.95E-14	0.2	TRUE	FALSE	0.35	TRUE	TRUE	0.29	FALSE	FALSE
	PRG4	-4.19	3.00E-13	0.28	TRUE	FALSE	0.22	TRUE	FALSE	0.29	FALSE	FALSE
	HPS5	-1.65	1.50E-12	0.27	TRUE	FALSE	0.22	TRUE	TRUE	0.22	FALSE	FALSE
	FGD4	-1.75	1.89E-12	0.26	TRUE	TRUE	0.36	TRUE	TRUE	0.35	FALSE	FALSE
	STBD1	-1.67	5.91E-12	0.25	TRUE	FALSE	0.22	TRUE	FALSE	0.16	FALSE	FALSE
	PER2	-1.52	1.06E-11	0.32	TRUE	FALSE	0.24	TRUE	FALSE	0.27	FALSE	FALSE
	TIPARP	-1.67	3.24E-11	0.29	TRUE	FALSE	0.36	TRUE	FALSE	0.34	FALSE	FALSE
	TCTEX1D1	-1.63	4.77E-11	0.26	TRUE	FALSE	0.25	TRUE	FALSE	0.21	FALSE	FALSE
	PTP4A1	-1.62	1.60E-10	0.27	TRUE	FALSE	0.27	TRUE	FALSE	0.28	FALSE	FALSE
	EFHA2	-1.79	1.89E-10	0.18	TRUE	FALSE	0.26	TRUE	FALSE	0.22	FALSE	FALSE
	EXPH5	-1.9	2.14E-10	0.2	TRUE	TRUE	0.26	TRUE	TRUE	0.26	FALSE	FALSE
	TNFRSF17	-1.84	5.34E-09	0.15	TRUE	FALSE	0.38	TRUE	FALSE	0.19	FALSE	FALSE
	OSM	-1.65	2.39E-08	0.17	TRUE	FALSE	0.28	TRUE	FALSE	0.18	FALSE	FALSE
	GDA	-2.04	1.07E-06	0.1	TRUE	FALSE	0.22	TRUE	FALSE	0.23	FALSE	FALSE
	LHFP	-1.69	1.37E-06	0.17	TRUE	FALSE	0.27	TRUE	FALSE	0.19	FALSE	FALSE
	ITIH5	-1.61	2.35E-05	0.24	TRUE	FALSE	0.22	TRUE	FALSE	0.15	FALSE	TRUE
	SMOC2	-1.6	1.28E-03	0.05	TRUE	FALSE	0.16	TRUE	TRUE	0.03	FALSE	FALSE
	LEPREL1	-3.6	1.08E-35	0.36	TRUE	TRUE	0.48	FALSE	FALSE	0.36	TRUE	FALSE
	HSD17B13	-6.7	2.13E-29	0.37	TRUE	FALSE	0.3	FALSE	FALSE	0.27	TRUE	FALSE
	RBMS3	-4.03	3.52E-28	0.36	TRUE	FALSE	0.46	FALSE	FALSE	0.38	TRUE	FALSE
	ANK3	-4.77	3.75E-23	0.29	TRUE	FALSE	0.44	FALSE	FALSE	0.27	TRUE	FALSE
	AKAP2	-2.55	6.13E-23	0.34	TRUE	FALSE	0.36	FALSE	FALSE	0.29	TRUE	FALSE
	KDR	-1.75	3.34E-22	0.34	TRUE	FALSE	0.32	FALSE	FALSE	0.27	TRUE	TRUE
	WDR72	-1.69	1.14E-20	0.3	TRUE	FALSE	0.37	FALSE	FALSE	0.33	TRUE	FALSE
	THBS1	-2.32	3.58E-19	0.32	TRUE	FALSE	0.37	FALSE	FALSE	0.21	TRUE	FALSE

miR-21 & miR-221	HMGCS2	-2.3	2.74E-18	0.32	TRUE	FALSE	0.31	FALSE	FALSE	0.25	TRUE	FALSE
	BCHE	-4.48	2.02E-17	0.29	TRUE	FALSE	0.29	FALSE	FALSE	0.31	TRUE	TRUE
	PRICKLE2	-1.63	6.33E-17	0.28	TRUE	FALSE	0.32	FALSE	FALSE	0.24	TRUE	FALSE
	PGM1	-1.7	2.95E-15	0.33	TRUE	TRUE	0.29	FALSE	FALSE	0.24	TRUE	FALSE
	KLF8	-1.53	3.73E-15	0.23	TRUE	FALSE	0.38	FALSE	FALSE	0.25	TRUE	FALSE
	SLC31A1	-1.6	4.15E-15	0.36	TRUE	TRUE	0.24	FALSE	FALSE	0.27	TRUE	FALSE
	DNAJB14	-1.6	4.49E-15	0.33	TRUE	FALSE	0.37	FALSE	FALSE	0.35	TRUE	FALSE
	CLIC2	-1.62	1.14E-14	0.27	TRUE	TRUE	0.35	FALSE	FALSE	0.29	TRUE	TRUE
	FAM46A	-1.93	1.83E-14	0.36	TRUE	TRUE	0.26	FALSE	FALSE	0.23	TRUE	FALSE
	ABI3BP	-1.8	9.51E-14	0.25	TRUE	FALSE	0.31	FALSE	FALSE	0.25	TRUE	FALSE
	HERPUD1	-1.52	1.58E-13	0.26	TRUE	FALSE	0.35	FALSE	FALSE	0.31	TRUE	FALSE
	TNFRSF11B	-2.68	2.07E-13	0.2	TRUE	TRUE	0.41	FALSE	FALSE	0.24	TRUE	FALSE
	PIK3R1	-1.95	3.12E-12	0.3	TRUE	FALSE	0.25	FALSE	TRUE	0.36	TRUE	TRUE
	GPR180	-1.53	7.66E-12	0.22	TRUE	TRUE	0.22	FALSE	FALSE	0.24	TRUE	FALSE
	UGT2B17	-2.83	1.77E-11	0.22	TRUE	FALSE	0.24	FALSE	FALSE	0.25	TRUE	FALSE
	HPGD	-2.69	9.80E-11	0.17	TRUE	FALSE	0.29	FALSE	FALSE	0.26	TRUE	FALSE
	LYRM5	-1.59	1.38E-10	0.31	TRUE	FALSE	0.22	FALSE	FALSE	0.29	TRUE	FALSE
	ANXA1	-1.93	1.67E-09	0.2	TRUE	FALSE	0.33	FALSE	FALSE	0.28	TRUE	FALSE
	CUX2	-2.05	5.05E-08	0.27	TRUE	FALSE	0.13	FALSE	FALSE	0.18	TRUE	FALSE
miR-21	C7	-6.07	2.77E-37	0.36	TRUE	TRUE	0.46	FALSE	FALSE	0.26	FALSE	FALSE
	AXL	-2.79	4.50E-35	0.39	TRUE	FALSE	0.47	FALSE	FALSE	0.33	FALSE	FALSE
	TMEM27	-7.08	9.29E-32	0.3	TRUE	TRUE	0.4	FALSE	FALSE	0.31	FALSE	FALSE
	RDH5	-2.99	1.99E-30	0.41	TRUE	FALSE	0.36	FALSE	FALSE	0.3	FALSE	FALSE
	ITGA9	-2.07	1.22E-29	0.35	TRUE	FALSE	0.49	FALSE	FALSE	0.28	FALSE	FALSE
	RSPO3	-3.62	1.35E-29	0.38	TRUE	FALSE	0.47	FALSE	FALSE	0.38	FALSE	FALSE
	MS4A6A	-3.06	7.29E-29	0.37	TRUE	FALSE	0.45	FALSE	FALSE	0.34	FALSE	FALSE
	SUCLG2	-1.95	1.17E-27	0.36	TRUE	FALSE	0.38	FALSE	FALSE	0.32	FALSE	FALSE
	CYP2B6	-4.73	6.74E-27	0.39	TRUE	FALSE	0.39	FALSE	FALSE	0.35	FALSE	FALSE
	CLEC10A	-1.53	1.34E-26	0.26	TRUE	FALSE	0.3	FALSE	FALSE	0.08	FALSE	FALSE
	TLR4	-1.61	4.04E-26	0.36	TRUE	TRUE	0.44	FALSE	FALSE	0.46	FALSE	FALSE
	SLC22A10	-3.04	1.25E-25	0.32	TRUE	FALSE	0.36	FALSE	FALSE	0.28	FALSE	FALSE
	IGJ	-6.1	2.11E-25	0.26	TRUE	FALSE	0.4	FALSE	FALSE	0.23	FALSE	FALSE
	CNBP	-1.5	5.91E-25	0.32	TRUE	TRUE	0.37	FALSE	FALSE	0.33	FALSE	FALSE
	FAM105B	-3.7	7.73E-25	0.32	TRUE	FALSE	0.37	FALSE	FALSE	0.3	FALSE	FALSE
	FOXO1	-2.04	1.52E-24	0.33	TRUE	FALSE	0.38	FALSE	FALSE	0.34	FALSE	FALSE
	COTL1	-3.21	3.99E-24	0.25	TRUE	FALSE	0.43	FALSE	FALSE	0.27	FALSE	FALSE
	CFTR	-4.5	4.43E-24	0.26	TRUE	FALSE	0.42	FALSE	FALSE	0.25	FALSE	FALSE
	SLC38A2	-3.28	6.17E-24	0.36	TRUE	FALSE	0.43	FALSE	FALSE	0.39	FALSE	FALSE
	LPA	-2.75	1.85E-23	0.4	TRUE	TRUE	0.37	FALSE	FALSE	0.31	FALSE	FALSE
	SDPR	-2.09	3.48E-23	0.38	TRUE	FALSE	0.39	FALSE	FALSE	0.34	FALSE	FALSE
	ZFP1	-2.14	5.45E-23	0.27	TRUE	FALSE	0.41	FALSE	FALSE	0.37	FALSE	FALSE
	GBA3	-7.15	1.86E-22	0.38	TRUE	FALSE	0.35	FALSE	FALSE	0.35	FALSE	FALSE
	PTN	-3.5	2.12E-22	0.4	TRUE	FALSE	0.4	FALSE	FALSE	0.31	FALSE	FALSE
	KLF6	-2.29	3.62E-22	0.33	TRUE	TRUE	0.44	FALSE	FALSE	0.37	FALSE	FALSE
	IL1B	-3.32	5.40E-22	0.26	TRUE	TRUE	0.46	FALSE	FALSE	0.3	FALSE	FALSE
	DUSP5	-2.47	9.34E-22	0.29	TRUE	FALSE	0.38	FALSE	FALSE	0.21	FALSE	FALSE
	REPS1	-1.77	1.88E-21	0.35	TRUE	FALSE	0.33	FALSE	FALSE	0.3	FALSE	FALSE
	PTPRN2	-1.89	1.89E-21	0.3	TRUE	FALSE	0.53	FALSE	FALSE	0.31	FALSE	FALSE
	FAM150B	-2.23	5.87E-21	0.3	TRUE	FALSE	0.46	FALSE	FALSE	0.33	FALSE	FALSE
	SPRY2	-2.03	1.33E-20	0.37	TRUE	TRUE	0.36	FALSE	FALSE	0.24	FALSE	FALSE
	RETSAT	-1.78	1.98E-20	0.29	TRUE	TRUE	0.31	FALSE	FALSE	0.17	FALSE	FALSE
	IYD	-2.89	3.71E-20	0.35	TRUE	FALSE	0.32	FALSE	FALSE	0.35	FALSE	FALSE
	ACAT1	-2.21	8.14E-20	0.38	TRUE	FALSE	0.35	FALSE	FALSE	0.33	FALSE	FALSE
	ST3GAL6	-2.15	1.79E-19	0.35	TRUE	TRUE	0.32	FALSE	FALSE	0.36	FALSE	FALSE
	PRKAG2	-1.8	2.97E-19	0.35	TRUE	FALSE	0.33	FALSE	FALSE	0.37	FALSE	FALSE
	IFI44L	-2.15	5.43E-19	0.26	TRUE	FALSE	0.39	FALSE	FALSE	0.32	FALSE	FALSE
	CD1C	-1.66	7.73E-19	0.24	TRUE	FALSE	0.36	FALSE	FALSE	0.16	FALSE	FALSE
	FMO2	-1.64	1.06E-18	0.29	TRUE	FALSE	0.42	FALSE	FALSE	0.34	FALSE	FALSE
	NFKBIZ	-2.11	1.13E-18	0.21	TRUE	FALSE	0.38	FALSE	FALSE	0.3	FALSE	FALSE
	BST1	-1.78	1.53E-18	0.27	TRUE	FALSE	0.35	FALSE	FALSE	0.22	FALSE	FALSE
	LONRF1	-1.72	2.47E-18	0.35	TRUE	FALSE	0.4	FALSE	FALSE	0.26	FALSE	FALSE
	RHOB	-1.85	4.26E-18	0.34	TRUE	FALSE	0.26	FALSE	FALSE	0.23	FALSE	FALSE
	PELI2	-1.59	4.48E-18	0.23	TRUE	TRUE	0.41	FALSE	FALSE	0.25	FALSE	FALSE
	SNAI1	-2.28	6.12E-18	0.23	TRUE	FALSE	0.37	FALSE	FALSE	0.21	FALSE	FALSE
	TFR2	-2	7.13E-18	0.4	TRUE	FALSE	0.31	FALSE	FALSE	0.26	FALSE	FALSE
	LRRK2	-1.72	8.08E-18	0.22	TRUE	FALSE	0.32	FALSE	FALSE	0.36	FALSE	FALSE
	TGFBR3	-1.65	1.20E-17	0.31	TRUE	FALSE	0.33	FALSE	FALSE	0.36	FALSE	FALSE
	TAT	-4.91	1.32E-17	0.34	TRUE	TRUE	0.4	FALSE	FALSE	0.37	FALSE	FALSE
	PTPRC	-2.21	5.30E-17	0.22	TRUE	FALSE	0.31	FALSE	FALSE	0.23	FALSE	FALSE
	NCKAP1L	-1.53	8.46E-17	0.2	TRUE	FALSE	0.34	FALSE	FALSE	0.21	FALSE	FALSE
	RAPH1	-1.81	9.70E-17	0.36	TRUE	FALSE	0.33	FALSE	FALSE	0.35	FALSE	FALSE
	SPON1	-3.23	3.56E-16	0.25	TRUE	TRUE	0.33	FALSE	FALSE	0.18	FALSE	FALSE
	SHMT1	-2.3	8.31E-16	0.3	TRUE	FALSE	0.28	FALSE	FALSE	0.2	FALSE	FALSE

	DOCK8	-2.13	9.91E-16	0.2	TRUE	FALSE	0.35	FALSE	FALSE	0.24	FALSE	FALSE
	GNG2	-1.6	1.40E-15	0.28	TRUE	TRUE	0.36	FALSE	FALSE	0.32	FALSE	FALSE
	XDH	-1.71	1.74E-15	0.31	TRUE	FALSE	0.28	FALSE	FALSE	0.27	FALSE	FALSE
	DNAJC12	-2.57	1.94E-15	0.33	TRUE	FALSE	0.3	FALSE	FALSE	0.29	FALSE	FALSE
	FYB	-1.83	6.24E-15	0.24	TRUE	FALSE	0.33	FALSE	FALSE	0.22	FALSE	FALSE
	SLC23A2	-1.74	1.27E-14	0.21	TRUE	FALSE	0.35	FALSE	FALSE	0.22	FALSE	FALSE
	ZNF462	-2.06	4.22E-14	0.25	TRUE	FALSE	0.39	FALSE	FALSE	0.31	FALSE	FALSE
	BTG1	-1.53	4.41E-14	0.15	TRUE	FALSE	0.3	FALSE	FALSE	0.28	FALSE	FALSE
	JHDM1D	-1.57	6.13E-14	0.24	TRUE	FALSE	0.42	FALSE	FALSE	0.38	FALSE	FALSE
	CYP2J2	-2.2	6.29E-14	0.36	TRUE	FALSE	0.32	FALSE	FALSE	0.24	FALSE	FALSE
	PCSK6	-1.89	9.42E-14	0.41	TRUE	TRUE	0.24	FALSE	FALSE	0.23	FALSE	FALSE
	WSB1	-1.74	1.31E-13	0.19	TRUE	FALSE	0.38	FALSE	FALSE	0.3	FALSE	FALSE
	EPHA3	-1.72	1.64E-13	0.23	TRUE	FALSE	0.3	FALSE	FALSE	0.22	FALSE	FALSE
	ANKRD37	-2.24	1.82E-13	0.29	TRUE	FALSE	0.32	FALSE	FALSE	0.23	FALSE	FALSE
	LTBP1	-1.79	2.02E-13	0.29	TRUE	FALSE	0.26	FALSE	FALSE	0.23	FALSE	FALSE
	CCR7	-2.3	3.35E-13	0.18	TRUE	FALSE	0.27	FALSE	FALSE	0.13	FALSE	FALSE
	SLCO1B1	-3.63	3.50E-13	0.28	TRUE	FALSE	0.37	FALSE	FALSE	0.33	FALSE	FALSE
	LURAP1L	-1.79	5.36E-13	0.21	TRUE	FALSE	0.19	FALSE	FALSE	0.22	FALSE	FALSE
	NRG1	-1.97	1.06E-12	0.22	TRUE	FALSE	0.34	FALSE	FALSE	0.2	FALSE	FALSE
	TRPM8	-2.56	1.61E-12	0.29	TRUE	FALSE	0.25	FALSE	FALSE	0.25	FALSE	FALSE
	SELE	-2.12	3.81E-12	0.27	TRUE	FALSE	0.29	FALSE	FALSE	0.24	FALSE	FALSE
	SLCO4C1	-2.12	5.13E-12	0.16	TRUE	FALSE	0.27	FALSE	FALSE	0.17	FALSE	FALSE
	ARVCF	-1.55	5.21E-12	0.26	TRUE	FALSE	0.39	FALSE	FALSE	0.31	FALSE	FALSE
	CLDN1	-1.53	6.55E-12	0.26	TRUE	FALSE	0.21	FALSE	FALSE	0.24	FALSE	FALSE
	UGT2B4	-2.48	2.36E-11	0.3	TRUE	FALSE	0.26	FALSE	FALSE	0.24	FALSE	FALSE
	GRB14	-1.72	2.50E-11	0.18	TRUE	FALSE	0.31	FALSE	FALSE	0.3	FALSE	FALSE
	GJB2	-1.86	3.73E-11	0.24	TRUE	FALSE	0.18	FALSE	FALSE	0.15	FALSE	FALSE
	GBP1	-1.94	4.36E-11	0.29	TRUE	FALSE	0.25	FALSE	FALSE	0.25	FALSE	FALSE
	NFIA	-1.54	1.02E-10	0.29	TRUE	FALSE	0.3	FALSE	FALSE	0.3	FALSE	FALSE
	LRRC32	-1.71	1.79E-10	0.19	TRUE	FALSE	0.32	FALSE	FALSE	0.2	FALSE	FALSE
	RCAN2	-1.98	5.50E-10	0.21	TRUE	FALSE	0.32	FALSE	FALSE	0.17	FALSE	FALSE
	ADRB2	-1.6	6.51E-10	0.25	TRUE	FALSE	0.17	FALSE	FALSE	0.13	FALSE	FALSE
	VCAM1	-2.32	8.23E-10	0.19	TRUE	FALSE	0.28	FALSE	FALSE	0.16	FALSE	FALSE
	MAOA	-1.62	1.52E-09	0.29	TRUE	FALSE	0.26	FALSE	FALSE	0.24	FALSE	FALSE
	SERINC1	-1.53	1.58E-09	0.29	TRUE	FALSE	0.36	FALSE	FALSE	0.28	FALSE	FALSE
	IDH2	-1.59	1.65E-09	0.23	TRUE	FALSE	0.17	FALSE	FALSE	0.19	FALSE	FALSE
	ARMCX2	-1.53	2.66E-09	0.15	TRUE	FALSE	0.29	FALSE	FALSE	0.12	FALSE	FALSE
	CDO1	-2.5	3.22E-09	0.28	TRUE	FALSE	0.27	FALSE	FALSE	0.25	FALSE	FALSE
	COL3A1	-2.33	5.62E-09	0.19	TRUE	FALSE	0.25	FALSE	FALSE	0.17	FALSE	FALSE
	GATM	-2.1	7.08E-09	0.27	TRUE	FALSE	0.25	FALSE	FALSE	0.26	FALSE	FALSE
	CFH	-1.9	7.65E-09	0.28	TRUE	FALSE	0.26	FALSE	FALSE	0.27	FALSE	FALSE
	EPCAM	-3.68	8.36E-09	0.13	TRUE	FALSE	0.3	FALSE	FALSE	0.16	FALSE	FALSE
	RTN4	-1.64	1.04E-08	0.15	TRUE	FALSE	0.2	FALSE	FALSE	0.21	FALSE	FALSE
	SSTR2	-1.62	1.07E-08	0.18	TRUE	FALSE	0.26	FALSE	FALSE	0.07	FALSE	FALSE
	NT5E	-1.54	2.78E-08	0.25	TRUE	TRUE	0.26	FALSE	FALSE	0.18	FALSE	FALSE
	RGS1	-2.39	1.00E-07	0.14	TRUE	FALSE	0.27	FALSE	FALSE	0.19	FALSE	FALSE
	CYP3A7	-2.19	1.70E-07	0.24	TRUE	FALSE	0.21	FALSE	FALSE	0.26	FALSE	FALSE
	MYH11	-1.66	2.92E-07	0.14	TRUE	FALSE	0.22	FALSE	FALSE	0.13	FALSE	FALSE
	CFHR1	-2.07	2.29E-06	0.22	TRUE	FALSE	0.25	FALSE	FALSE	0.22	FALSE	FALSE
	CYB5A	-1.53	4.42E-06	0.24	TRUE	FALSE	0.11	FALSE	FALSE	0.14	FALSE	FALSE
	MAP9	-1.5	7.66E-06	0.13	TRUE	FALSE	0.3	FALSE	FALSE	0.2	FALSE	FALSE
	CXCL14	-17.71	3.08E-53	0.37	FALSE	FALSE	0.46	TRUE	FALSE	0.35	TRUE	FALSE
	CXCL12	-7.98	6.34E-38	0.36	FALSE	FALSE	0.51	TRUE	FALSE	0.3	TRUE	FALSE
	PLSCR4	-2.83	1.88E-26	0.38	FALSE	FALSE	0.49	TRUE	FALSE	0.36	TRUE	FALSE
	MYO10	-3.07	1.17E-24	0.31	FALSE	FALSE	0.48	TRUE	FALSE	0.35	TRUE	FALSE
	NTN4	-2.48	2.68E-23	0.32	FALSE	FALSE	0.42	TRUE	TRUE	0.4	TRUE	FALSE
	PBLD	-3.06	1.83E-21	0.34	FALSE	FALSE	0.35	TRUE	TRUE	0.4	TRUE	FALSE
	TNF	-1.57	2.64E-21	0.31	FALSE	FALSE	0.34	TRUE	FALSE	0.2	TRUE	FALSE
	KRT81	-1.79	3.08E-21	0.33	FALSE	FALSE	0.3	TRUE	FALSE	0.14	TRUE	FALSE
	FXN	-1.56	4.07E-21	0.3	FALSE	FALSE	0.27	TRUE	TRUE	0.18	TRUE	FALSE
	SOCS3	-3.7	6.41E-21	0.23	FALSE	FALSE	0.44	TRUE	FALSE	0.26	TRUE	FALSE
	EGR2	-3.95	6.94E-21	0.25	FALSE	FALSE	0.42	TRUE	FALSE	0.26	TRUE	FALSE
	TSLP	-2.6	8.94E-21	0.23	FALSE	FALSE	0.43	TRUE	FALSE	0.32	TRUE	FALSE
	CALM1	-1.58	1.21E-20	0.32	FALSE	FALSE	0.37	TRUE	FALSE	0.26	TRUE	FALSE
	AKAP13	-1.5	5.54E-20	0.32	FALSE	FALSE	0.34	TRUE	FALSE	0.36	TRUE	FALSE
	AFF1	-1.55	1.94E-19	0.33	FALSE	FALSE	0.33	TRUE	FALSE	0.32	TRUE	FALSE
	NR3C2	-1.68	2.21E-19	0.24	FALSE	FALSE	0.34	TRUE	FALSE	0.29	TRUE	FALSE
	SPG20	-1.85	1.54E-18	0.23	FALSE	FALSE	0.45	TRUE	TRUE	0.34	TRUE	TRUE
	ANTXR2	-2.14	2.22E-18	0.39	FALSE	FALSE	0.4	TRUE	FALSE	0.31	TRUE	TRUE
	ZEB2	-1.99	1.70E-17	0.27	FALSE	FALSE	0.45	TRUE	FALSE	0.32	TRUE	TRUE
	RGL1	-1.76	5.15E-17	0.31	FALSE	FALSE	0.38	TRUE	TRUE	0.33	TRUE	FALSE
miR-221	NR4A2	-2.34	6.45E-17	0.21	FALSE	FALSE	0.37	TRUE	TRUE	0.23	TRUE	FALSE
	KCNJ16	-2.6	8.87E-16	0.28	FALSE	FALSE	0.42	TRUE	FALSE	0.26	TRUE	FALSE

miR-93 &	CCNL1	-1.6	1.49E-15	0.29	FALSE	FALSE	0.41	TRUE	FALSE	0.32	TRUE	FALSE
	MEX3D	-1.61	2.39E-15	0.35	FALSE	FALSE	0.2	TRUE	TRUE	0.18	TRUE	FALSE
	LONP2	-1.67	2.87E-15	0.34	FALSE	FALSE	0.26	TRUE	TRUE	0.33	TRUE	FALSE
	FILIP1	-1.55	1.00E-14	0.24	FALSE	FALSE	0.35	TRUE	FALSE	0.3	TRUE	FALSE
	ABCA8	-2.27	2.77E-14	0.32	FALSE	FALSE	0.34	TRUE	FALSE	0.35	TRUE	FALSE
	PRUNE2	-1.71	3.96E-14	0.3	FALSE	FALSE	0.39	TRUE	FALSE	0.35	TRUE	FALSE
	TGDS	-1.95	4.98E-14	0.25	FALSE	FALSE	0.36	TRUE	FALSE	0.38	TRUE	FALSE
	FMOD	-2.99	1.66E-13	0.21	FALSE	FALSE	0.39	TRUE	FALSE	0.22	TRUE	FALSE
	TMEM25	-2.1	4.40E-13	0.13	FALSE	FALSE	0.28	TRUE	TRUE	0.29	TRUE	FALSE
	SLC22A3	-1.69	3.87E-12	0.23	FALSE	FALSE	0.38	TRUE	FALSE	0.29	TRUE	TRUE
	FGB	-3.18	3.86E-11	0.26	FALSE	FALSE	0.38	TRUE	FALSE	0.31	TRUE	FALSE
	MCTP2	-1.89	5.39E-11	0.14	FALSE	FALSE	0.31	TRUE	FALSE	0.19	TRUE	FALSE
	ZC3H13	-1.56	1.57E-10	0.15	FALSE	FALSE	0.3	TRUE	FALSE	0.34	TRUE	FALSE
	BNIP3L	-1.67	1.70E-10	0.2	FALSE	FALSE	0.39	TRUE	TRUE	0.36	TRUE	FALSE
	TSPAN12	-1.62	2.52E-10	0.1	FALSE	FALSE	0.22	TRUE	FALSE	0.24	TRUE	FALSE
	HOOK1	-1.62	5.46E-10	0.2	FALSE	FALSE	0.25	TRUE	FALSE	0.24	TRUE	TRUE
	PLD1	-1.67	1.61E-09	0.25	FALSE	FALSE	0.19	TRUE	FALSE	0.23	TRUE	FALSE
	FNDC3A	-1.64	6.78E-09	0.23	FALSE	FALSE	0.27	TRUE	TRUE	0.31	TRUE	TRUE
	FBLN1	-2.06	1.46E-08	0.18	FALSE	FALSE	0.28	TRUE	TRUE	0.09	TRUE	FALSE
	GAS2	-1.63	2.63E-07	0.26	FALSE	FALSE	0.1	TRUE	FALSE	0.16	TRUE	FALSE
	AADAC	-1.88	7.17E-07	0.27	FALSE	FALSE	0.21	TRUE	FALSE	0.11	TRUE	TRUE
	FNDC5	-1.87	2.61E-06	0.14	FALSE	FALSE	0.09	TRUE	FALSE	0.14	TRUE	FALSE
	CD5L	-7.45	4.78E-37	0.4	FALSE	FALSE	0.42	TRUE	FALSE	0.32	FALSE	FALSE
	EHD3	-2.53	3.15E-35	0.4	FALSE	FALSE	0.38	TRUE	FALSE	0.3	FALSE	FALSE
	COLEC10	-2.55	7.67E-34	0.39	FALSE	FALSE	0.47	TRUE	FALSE	0.32	FALSE	FALSE
	CYP1A2	-7.25	1.06E-33	0.41	FALSE	FALSE	0.43	TRUE	FALSE	0.36	FALSE	FALSE
	CCL23	-3.03	2.16E-33	0.41	FALSE	FALSE	0.46	TRUE	FALSE	0.35	FALSE	FALSE
	SRPX	-6.03	5.96E-33	0.34	FALSE	FALSE	0.45	TRUE	FALSE	0.29	FALSE	FALSE
	FXYD6	-2.14	3.69E-31	0.38	FALSE	FALSE	0.44	TRUE	FALSE	0.27	FALSE	FALSE
	KCNN2	-4.03	2.78E-30	0.35	FALSE	FALSE	0.32	TRUE	FALSE	0.35	FALSE	FALSE
	SYNE1	-2.3	3.15E-30	0.34	FALSE	FALSE	0.48	TRUE	TRUE	0.38	FALSE	FALSE
	TFPI2	-4.65	3.53E-30	0.38	FALSE	FALSE	0.48	TRUE	FALSE	0.35	FALSE	FALSE
	IDO2	-7.99	7.53E-30	0.37	FALSE	FALSE	0.39	TRUE	FALSE	0.38	FALSE	FALSE
	SFRP1	-4.33	8.11E-30	0.33	FALSE	FALSE	0.43	TRUE	FALSE	0.29	FALSE	FALSE
	CYP39A1	-5.25	1.31E-29	0.4	FALSE	FALSE	0.43	TRUE	FALSE	0.33	FALSE	FALSE
	NAAA	-2.64	1.57E-29	0.44	FALSE	FALSE	0.33	TRUE	FALSE	0.27	FALSE	FALSE
	MAN1C1	-3.93	4.06E-29	0.36	FALSE	FALSE	0.51	TRUE	FALSE	0.38	FALSE	FALSE
	NBLA00301	-2.83	5.33E-29	0.32	FALSE	FALSE	0.43	TRUE	FALSE	0.3	FALSE	FALSE
	CXCL3	-3.1	2.00E-28	0.4	FALSE	FALSE	0.43	TRUE	FALSE	0.37	FALSE	FALSE
	PDGFRA	-5.53	1.56E-27	0.33	FALSE	FALSE	0.39	TRUE	FALSE	0.26	FALSE	FALSE
	MS4A7	-3.06	2.75E-27	0.34	FALSE	FALSE	0.47	TRUE	FALSE	0.33	FALSE	FALSE
	IRF8	-2	2.82E-27	0.33	FALSE	FALSE	0.41	TRUE	FALSE	0.25	FALSE	FALSE
	AADAT	-3.4	9.95E-27	0.38	FALSE	FALSE	0.35	TRUE	FALSE	0.35	FALSE	FALSE
	ALDH8A1	-3.73	9.72E-26	0.36	FALSE	FALSE	0.39	TRUE	FALSE	0.37	FALSE	FALSE
	ITPRIP	-1.84	1.02E-25	0.32	FALSE	FALSE	0.4	TRUE	FALSE	0.29	FALSE	FALSE
	SLC31A2	-2.21	1.18E-25	0.32	FALSE	FALSE	0.4	TRUE	FALSE	0.32	FALSE	FALSE
	ERRFI1	-2.42	1.37E-25	0.41	FALSE	FALSE	0.38	TRUE	FALSE	0.38	FALSE	FALSE
	ANGPTL1	-3.23	7.36E-25	0.3	FALSE	FALSE	0.5	TRUE	FALSE	0.38	FALSE	FALSE
	MEIS3P1	-1.94	9.56E-25	0.33	FALSE	FALSE	0.46	TRUE	FALSE	0.33	FALSE	FALSE
	BMP6	-1.76	1.08E-24	0.38	FALSE	FALSE	0.41	TRUE	FALSE	0.26	FALSE	FALSE
	CDC37L1	-2.19	1.04E-23	0.38	FALSE	FALSE	0.4	TRUE	TRUE	0.41	FALSE	FALSE
	DUSP6	-2.76	2.57E-23	0.33	FALSE	FALSE	0.39	TRUE	FALSE	0.32	FALSE	FALSE
	ITGAD	-2.7	3.09E-23	0.24	FALSE	FALSE	0.4	TRUE	FALSE	0.27	FALSE	FALSE
	SMAD6	-1.55	3.48E-23	0.35	FALSE	FALSE	0.35	TRUE	FALSE	0.28	FALSE	FALSE
	ACADSB	-3.56	3.77E-23	0.35	FALSE	FALSE	0.39	TRUE	TRUE	0.32	FALSE	FALSE
	C8A	-4.68	8.15E-23	0.45	FALSE	FALSE	0.42	TRUE	FALSE	0.36	FALSE	FALSE
	ADRA1B	-3.56	1.30E-22	0.32	FALSE	FALSE	0.45	TRUE	FALSE	0.3	FALSE	FALSE
	ANKS1A	-1.61	1.45E-22	0.32	FALSE	FALSE	0.44	TRUE	FALSE	0.29	FALSE	FALSE
	SIK1	-2.62	3.50E-22	0.27	FALSE	FALSE	0.41	TRUE	FALSE	0.28	FALSE	FALSE
	TMEM71	-1.93	3.59E-22	0.31	FALSE	FALSE	0.41	TRUE	FALSE	0.31	FALSE	FALSE
	CAMK2D	-2.06	6.38E-22	0.36	FALSE	FALSE	0.44	TRUE	FALSE	0.39	FALSE	FALSE
	GLRA3	-2.4	7.93E-22	0.38	FALSE	FALSE	0.32	TRUE	FALSE	0.24	FALSE	FALSE
	CYP4V2	-2.92	8.22E-22	0.34	FALSE	FALSE	0.39	TRUE	FALSE	0.36	FALSE	FALSE
	PLK3	-2.67	9.07E-22	0.28	FALSE	FALSE	0.41	TRUE	FALSE	0.25	FALSE	FALSE
	MT1F	-6.4	9.56E-22	0.36	FALSE	FALSE	0.3	TRUE	FALSE	0.32	FALSE	FALSE
	BHLHE40	-1.69	1.17E-21	0.29	FALSE	FALSE	0.33	TRUE	FALSE	0.35	FALSE	FALSE
	MCL1	-2.11	1.26E-21	0.33	FALSE	FALSE	0.4	TRUE	TRUE	0.35	FALSE	FALSE
	KLF11	-1.7	1.90E-21	0.3	FALSE	FALSE	0.33	TRUE	FALSE	0.23	FALSE	FALSE
	KLC4	-2.39	2.86E-21	0.33	FALSE	FALSE	0.34	TRUE	FALSE	0.27	FALSE	FALSE
	ADRA2B	-1.78	2.86E-21	0.34	FALSE	FALSE	0.45	TRUE	FALSE	0.31	FALSE	FALSE
	TSPYL1	-1.5	4.37E-21	0.33	FALSE	FALSE	0.36	TRUE	FALSE	0.24	FALSE	FALSE
	TLE4	-1.58	4.39E-21	0.29	FALSE	FALSE	0.43	TRUE	TRUE	0.3	FALSE	FALSE
	GIMAP8	-1.9	5.88E-21	0.31	FALSE	FALSE	0.45	TRUE	FALSE	0.37	FALSE	FALSE

DUSP2	-1.89	7.67E-21	0.28	FALSE	FALSE	0.35	TRUE	TRUE	0.19	FALSE	FALSE
NRIP3	-1.54	9.47E-21	0.27	FALSE	FALSE	0.24	TRUE	FALSE	0.14	FALSE	FALSE
LDLR	-2.38	1.23E-20	0.27	FALSE	FALSE	0.37	TRUE	TRUE	0.29	FALSE	FALSE
ADAMTS1	-2.69	1.23E-20	0.32	FALSE	FALSE	0.38	TRUE	FALSE	0.27	FALSE	FALSE
SCARA5	-2.46	1.25E-20	0.32	FALSE	FALSE	0.35	TRUE	TRUE	0.24	FALSE	FALSE
CD97	-1.63	1.28E-20	0.25	FALSE	FALSE	0.42	TRUE	FALSE	0.23	FALSE	FALSE
TRAF4	-1.51	3.39E-20	0.28	FALSE	FALSE	0.31	TRUE	FALSE	0.15	FALSE	FALSE
ACSL1	-3.1	3.70E-20	0.36	FALSE	FALSE	0.34	TRUE	FALSE	0.31	FALSE	FALSE
CYBB	-1.84	7.11E-20	0.3	FALSE	FALSE	0.4	TRUE	FALSE	0.34	FALSE	FALSE
FAM102A	-2.34	9.25E-20	0.35	FALSE	FALSE	0.33	TRUE	TRUE	0.28	FALSE	FALSE
ADAMTSL3	-2.35	1.82E-19	0.36	FALSE	FALSE	0.37	TRUE	FALSE	0.29	FALSE	FALSE
MT1B	-5.38	1.83E-19	0.35	FALSE	FALSE	0.33	TRUE	FALSE	0.35	FALSE	FALSE
ATG2A	-1.61	2.15E-19	0.28	FALSE	FALSE	0.4	TRUE	FALSE	0.25	FALSE	FALSE
LATS2	-1.64	4.76E-19	0.31	FALSE	FALSE	0.36	TRUE	FALSE	0.23	FALSE	FALSE
SAMD10	-1.82	6.07E-19	0.26	FALSE	FALSE	0.28	TRUE	FALSE	0.07	FALSE	FALSE
VSX1	-1.82	8.35E-19	0.34	FALSE	FALSE	0.3	TRUE	TRUE	0.18	FALSE	FALSE
SLC27A2	-3.15	1.11E-18	0.35	FALSE	FALSE	0.33	TRUE	FALSE	0.29	FALSE	FALSE
AOAH	-1.64	1.13E-18	0.24	FALSE	FALSE	0.35	TRUE	FALSE	0.22	FALSE	FALSE
TSPAN9	-1.54	1.54E-18	0.32	FALSE	FALSE	0.27	TRUE	FALSE	0.23	FALSE	FALSE
ALDOB	-3.84	1.73E-18	0.35	FALSE	FALSE	0.33	TRUE	FALSE	0.3	FALSE	FALSE
MAT1A	-3.29	2.22E-18	0.4	FALSE	FALSE	0.43	TRUE	FALSE	0.33	FALSE	FALSE
GPD1	-2.32	3.90E-18	0.33	FALSE	FALSE	0.26	TRUE	FALSE	0.25	FALSE	FALSE
TMEM66	-1.58	4.36E-18	0.33	FALSE	FALSE	0.32	TRUE	FALSE	0.18	FALSE	FALSE
RASSF5	-1.71	7.34E-18	0.25	FALSE	FALSE	0.34	TRUE	FALSE	0.21	FALSE	FALSE
GZMK	-2.43	1.19E-17	0.24	FALSE	FALSE	0.28	TRUE	FALSE	0.14	FALSE	FALSE
BHMT	-5.2	1.91E-17	0.32	FALSE	FALSE	0.31	TRUE	FALSE	0.31	FALSE	FALSE
TRIM22	-2.02	1.93E-17	0.28	FALSE	FALSE	0.33	TRUE	TRUE	0.26	FALSE	FALSE
DHODH	-2.35	3.08E-17	0.32	FALSE	FALSE	0.32	TRUE	FALSE	0.27	FALSE	FALSE
KCNK6	-1.52	3.11E-17	0.2	FALSE	FALSE	0.39	TRUE	FALSE	0.27	FALSE	FALSE
EPAS1	-1.73	5.49E-17	0.36	FALSE	FALSE	0.35	TRUE	FALSE	0.29	FALSE	FALSE
MTHFD1	-2.32	6.15E-17	0.36	FALSE	FALSE	0.34	TRUE	FALSE	0.36	FALSE	FALSE
FOXF1	-1.64	6.42E-17	0.3	FALSE	FALSE	0.38	TRUE	FALSE	0.25	FALSE	FALSE
RNF135	-1.87	6.86E-17	0.24	FALSE	FALSE	0.38	TRUE	FALSE	0.26	FALSE	FALSE
KLF10	-1.98	7.34E-17	0.34	FALSE	FALSE	0.32	TRUE	TRUE	0.28	FALSE	FALSE
PFKFB3	-1.96	8.18E-17	0.2	FALSE	FALSE	0.28	TRUE	FALSE	0.3	FALSE	FALSE
ST6GALNAC6	-1.6	9.64E-17	0.29	FALSE	FALSE	0.34	TRUE	FALSE	0.2	FALSE	FALSE
MPC1	-1.99	1.00E-16	0.26	FALSE	FALSE	0.39	TRUE	FALSE	0.24	FALSE	FALSE
SCP2	-2.43	1.03E-16	0.31	FALSE	FALSE	0.28	TRUE	FALSE	0.31	FALSE	FALSE
PRRG4	-1.61	1.64E-16	0.34	FALSE	TRUE	0.18	TRUE	TRUE	0.17	FALSE	FALSE
REL	-1.55	1.93E-16	0.25	FALSE	FALSE	0.39	TRUE	FALSE	0.31	FALSE	FALSE
CTBS	-1.58	1.94E-16	0.35	FALSE	FALSE	0.31	TRUE	FALSE	0.27	FALSE	FALSE
DHRS12	-1.72	2.20E-16	0.27	FALSE	FALSE	0.27	TRUE	FALSE	0.17	FALSE	FALSE
PXDN	-1.77	2.45E-16	0.26	FALSE	FALSE	0.35	TRUE	TRUE	0.27	FALSE	FALSE
PGM5	-1.61	3.19E-16	0.33	FALSE	FALSE	0.37	TRUE	FALSE	0.27	FALSE	FALSE
ARHGEF26	-1.94	3.31E-16	0.27	FALSE	FALSE	0.32	TRUE	FALSE	0.37	FALSE	FALSE
TMEM220	-1.93	4.16E-16	0.34	FALSE	FALSE	0.2	TRUE	FALSE	0.14	FALSE	FALSE
BHLHE22	-2.06	5.20E-16	0.26	FALSE	FALSE	0.34	TRUE	FALSE	0.21	FALSE	FALSE
PTPN3	-1.57	8.85E-16	0.25	FALSE	FALSE	0.28	TRUE	TRUE	0.24	FALSE	FALSE
CYBRD1	-2.15	8.91E-16	0.25	FALSE	FALSE	0.39	TRUE	TRUE	0.29	FALSE	FALSE
LMO2	-1.65	9.75E-16	0.19	FALSE	FALSE	0.39	TRUE	FALSE	0.2	FALSE	FALSE
CD68	-1.67	1.01E-15	0.21	FALSE	FALSE	0.3	TRUE	FALSE	0.16	FALSE	FALSE
TAPT1	-1.79	1.16E-15	0.3	FALSE	FALSE	0.37	TRUE	FALSE	0.41	FALSE	FALSE
TMEM154	-2.04	1.96E-15	0.22	FALSE	FALSE	0.33	TRUE	TRUE	0.31	FALSE	FALSE
YPEL2	-1.74	2.09E-15	0.19	FALSE	FALSE	0.35	TRUE	TRUE	0.31	FALSE	FALSE
PTGS1	-1.79	2.69E-15	0.24	FALSE	FALSE	0.33	TRUE	FALSE	0.29	FALSE	FALSE
SH3BP5	-1.54	3.16E-15	0.32	FALSE	FALSE	0.3	TRUE	FALSE	0.17	FALSE	FALSE
S1PR1	-1.59	3.38E-15	0.34	FALSE	FALSE	0.27	TRUE	FALSE	0.19	FALSE	FALSE
UGP2	-2.38	3.84E-15	0.29	FALSE	FALSE	0.34	TRUE	FALSE	0.34	FALSE	FALSE
NAV2	-1.92	3.88E-15	0.19	FALSE	FALSE	0.26	TRUE	FALSE	0.22	FALSE	FALSE
ACOT12	-2.67	1.32E-14	0.28	FALSE	FALSE	0.21	TRUE	FALSE	0.22	FALSE	FALSE
NLRP3	-1.71	1.58E-14	0.25	FALSE	FALSE	0.38	TRUE	TRUE	0.19	FALSE	FALSE
PECR	-1.71	1.59E-14	0.27	FALSE	FALSE	0.28	TRUE	FALSE	0.21	FALSE	FALSE
ARL4C	-1.91	2.25E-14	0.19	FALSE	FALSE	0.37	TRUE	TRUE	0.24	FALSE	FALSE
MPDZ	-1.98	2.67E-14	0.26	FALSE	FALSE	0.26	TRUE	FALSE	0.35	FALSE	FALSE
PPP1R1A	-3.03	4.07E-14	0.31	FALSE	FALSE	0.25	TRUE	FALSE	0.23	FALSE	FALSE
APP	-1.7	4.42E-14	0.15	FALSE	FALSE	0.26	TRUE	FALSE	0.23	FALSE	FALSE
EPB41L3	-1.56	4.49E-14	0.26	FALSE	FALSE	0.34	TRUE	FALSE	0.27	FALSE	FALSE
FURIN	-1.53	4.57E-14	0.25	FALSE	FALSE	0.2	TRUE	FALSE	0.17	FALSE	FALSE
CYP3A4	-4	7.87E-14	0.31	FALSE	FALSE	0.33	TRUE	FALSE	0.3	FALSE	FALSE
PKHD1	-1.82	1.05E-13	0.21	FALSE	FALSE	0.4	TRUE	TRUE	0.24	FALSE	FALSE
DDX3X	-1.56	1.52E-13	0.32	FALSE	FALSE	0.29	TRUE	FALSE	0.31	FALSE	FALSE
CCND1	-1.97	2.13E-13	0.31	FALSE	FALSE	0.33	TRUE	FALSE	0.25	FALSE	FALSE
MMAA	-1.6	2.68E-13	0.25	FALSE	FALSE	0.24	TRUE	TRUE	0.19	FALSE	FALSE

AIG1	-1.78	3.00E-13	0.27	FALSE	FALSE	0.32	TRUE	FALSE	0.26	FALSE	FALSE
F2RL2	-1.6	3.68E-13	0.31	FALSE	FALSE	0.2	TRUE	FALSE	0.23	FALSE	FALSE
TTR	-3.38	4.09E-13	0.33	FALSE	FALSE	0.33	TRUE	FALSE	0.3	FALSE	FALSE
ZBTB4	-1.54	4.92E-13	0.21	FALSE	FALSE	0.38	TRUE	TRUE	0.27	FALSE	FALSE
CEBPB	-1.57	5.34E-13	0.26	FALSE	FALSE	0.37	TRUE	FALSE	0.3	FALSE	FALSE
TMEM56	-1.79	5.75E-13	0.33	FALSE	FALSE	0.3	TRUE	TRUE	0.3	FALSE	FALSE
PLIN5	-2.08	7.40E-13	0.27	FALSE	FALSE	0.31	TRUE	FALSE	0.25	FALSE	FALSE
GSPT2	-1.76	9.67E-13	0.19	FALSE	FALSE	0.23	TRUE	FALSE	0.08	FALSE	FALSE
DPYD	-1.78	1.69E-12	0.25	FALSE	FALSE	0.31	TRUE	TRUE	0.27	FALSE	FALSE
MMP23B	-1.61	1.76E-12	0.21	FALSE	FALSE	0.27	TRUE	FALSE	0.15	FALSE	FALSE
SMOC1	-1.89	1.85E-12	0.34	FALSE	FALSE	0.23	TRUE	TRUE	0.27	FALSE	FALSE
RGS2	-2.93	2.20E-12	0.22	FALSE	FALSE	0.31	TRUE	FALSE	0.21	FALSE	FALSE
CDKN1A	-1.57	2.23E-12	0.26	FALSE	FALSE	0.16	TRUE	TRUE	0.08	FALSE	FALSE
ALDH1A3	-1.94	3.83E-12	0.24	FALSE	FALSE	0.27	TRUE	FALSE	0.16	FALSE	FALSE
PRKAG2	-1.71	4.91E-12	0.28	FALSE	FALSE	0.29	TRUE	FALSE	0.29	FALSE	FALSE
HECA	-1.65	5.09E-12	0.26	FALSE	FALSE	0.4	TRUE	FALSE	0.34	FALSE	FALSE
ABCG2	-2.05	5.75E-12	0.29	FALSE	FALSE	0.22	TRUE	TRUE	0.21	FALSE	FALSE
OSBPL5	-1.63	7.14E-12	0.15	FALSE	FALSE	0.33	TRUE	FALSE	0.17	FALSE	FALSE
MYLK	-1.59	1.02E-11	0.3	FALSE	FALSE	0.33	TRUE	FALSE	0.31	FALSE	FALSE
PRKAR2B	-1.69	1.14E-11	0.24	FALSE	FALSE	0.41	TRUE	FALSE	0.31	FALSE	FALSE
02-Mar	-1.71	1.20E-11	0.39	FALSE	FALSE	0.23	TRUE	FALSE	0.24	FALSE	FALSE
HADH	-1.76	1.24E-11	0.3	FALSE	FALSE	0.22	TRUE	FALSE	0.31	FALSE	FALSE
DIP2C	-1.56	1.52E-11	0.28	FALSE	FALSE	0.24	TRUE	FALSE	0.29	FALSE	FALSE
JAM2	-1.58	1.60E-11	0.26	FALSE	FALSE	0.31	TRUE	FALSE	0.16	FALSE	FALSE
SULT1E1	-2.11	1.74E-11	0.22	FALSE	FALSE	0.22	TRUE	FALSE	0.28	FALSE	FALSE
FZD1	-1.72	1.82E-11	0.18	FALSE	FALSE	0.29	TRUE	FALSE	0.21	FALSE	FALSE
AREG	-2.47	1.87E-11	0.21	FALSE	FALSE	0.29	TRUE	FALSE	0.21	FALSE	FALSE
MYO1B	-1.57	2.06E-11	0.27	FALSE	FALSE	0.27	TRUE	FALSE	0.34	FALSE	FALSE
DLL1	-1.65	2.80E-11	0.19	FALSE	FALSE	0.39	TRUE	FALSE	0.32	FALSE	FALSE
FBXO21	-1.5	3.13E-11	0.3	FALSE	FALSE	0.21	TRUE	TRUE	0.23	FALSE	FALSE
TMEM30B	-1.94	3.76E-11	0.29	FALSE	FALSE	0.24	TRUE	FALSE	0.22	FALSE	FALSE
SLC2A2	-2.14	5.28E-11	0.34	FALSE	FALSE	0.24	TRUE	TRUE	0.3	FALSE	FALSE
CTSS	-1.71	5.62E-11	0.23	FALSE	FALSE	0.24	TRUE	TRUE	0.19	FALSE	FALSE
HLA-DPB1	-1.93	7.55E-11	0.2	FALSE	FALSE	0.29	TRUE	FALSE	0.19	FALSE	FALSE
UGCG	-1.52	7.89E-11	0.22	FALSE	FALSE	0.28	TRUE	FALSE	0.21	FALSE	FALSE
NRG1	-1.62	1.53E-10	0.2	FALSE	FALSE	0.35	TRUE	FALSE	0.24	FALSE	FALSE
HEPACAM	-3.28	1.86E-10	0.26	FALSE	FALSE	0.24	TRUE	FALSE	0.18	FALSE	FALSE
SLC25A27	-1.64	1.95E-10	0.14	FALSE	FALSE	0.34	TRUE	FALSE	0.2	FALSE	FALSE
KCNJ8	-2.12	2.16E-10	0.29	FALSE	FALSE	0.23	TRUE	FALSE	0.14	FALSE	FALSE
GATA6	-1.59	2.23E-10	0.29	FALSE	FALSE	0.25	TRUE	FALSE	0.2	FALSE	FALSE
GAMT	-1.74	2.31E-10	0.26	FALSE	FALSE	0.23	TRUE	FALSE	0.12	FALSE	FALSE
RAB31	-1.68	2.38E-10	0.22	FALSE	FALSE	0.29	TRUE	FALSE	0.23	FALSE	FALSE
IQGAP1	-1.54	5.27E-10	0.18	FALSE	FALSE	0.28	TRUE	FALSE	0.26	FALSE	FALSE
HES1	-1.57	5.58E-10	0.17	FALSE	FALSE	0.3	TRUE	FALSE	0.32	FALSE	FALSE
TBC1D10C	-1.86	7.19E-10	0.17	FALSE	FALSE	0.24	TRUE	FALSE	0.12	FALSE	FALSE
PTGER4	-1.78	1.03E-09	0.26	FALSE	FALSE	0.26	TRUE	FALSE	0.19	FALSE	FALSE
TMEM100	-1.61	1.07E-09	0.25	FALSE	FALSE	0.34	TRUE	TRUE	0.2	FALSE	FALSE
PREX1	-1.52	1.30E-09	0.11	FALSE	FALSE	0.3	TRUE	FALSE	0.11	FALSE	FALSE
IQGAP2	-1.68	1.41E-09	0.24	FALSE	FALSE	0.25	TRUE	FALSE	0.31	FALSE	FALSE
AVPR1A	-1.96	1.45E-09	0.27	FALSE	FALSE	0.25	TRUE	FALSE	0.22	FALSE	FALSE
ADAMTS4	-1.81	1.51E-09	0.23	FALSE	FALSE	0.15	TRUE	FALSE	0.09	FALSE	FALSE
ABR	-1.72	1.74E-09	0.16	FALSE	FALSE	0.32	TRUE	FALSE	0.21	FALSE	FALSE
GLIPR2	-1.55	1.82E-09	0.19	FALSE	FALSE	0.21	TRUE	FALSE	0.09	FALSE	FALSE
CD38	-1.74	2.05E-09	0.22	FALSE	FALSE	0.24	TRUE	FALSE	0.12	FALSE	FALSE
MUM1L1	-1.6	3.33E-09	0.11	FALSE	FALSE	0.32	TRUE	TRUE	0.23	FALSE	FALSE
IL6	-1.76	3.73E-09	0.26	FALSE	FALSE	0.2	TRUE	FALSE	0.05	FALSE	FALSE
S100A4	-1.67	4.58E-09	0.1	FALSE	FALSE	0.23	TRUE	FALSE	0.12	FALSE	FALSE
TXNIP	-1.72	7.46E-09	0.2	FALSE	FALSE	0.26	TRUE	TRUE	0.14	FALSE	FALSE
RASD1	-1.71	1.28E-08	0.13	FALSE	FALSE	0.3	TRUE	TRUE	0.16	FALSE	FALSE
TSKU	-1.65	1.57E-08	0.18	FALSE	FALSE	0.18	TRUE	FALSE	0.17	FALSE	FALSE
HLF	-2.18	1.61E-08	0.22	FALSE	FALSE	0.19	TRUE	TRUE	0.17	FALSE	FALSE
THSD4	-1.54	2.10E-08	0.25	FALSE	FALSE	0.2	TRUE	FALSE	0.23	FALSE	FALSE
BHMT2	-1.58	4.06E-08	0.22	FALSE	FALSE	0.18	TRUE	FALSE	0.16	FALSE	FALSE
AMIGO2	-1.54	4.72E-08	0.16	FALSE	FALSE	0.2	TRUE	TRUE	0.11	FALSE	FALSE
TANC1	-1.51	8.26E-08	0.13	FALSE	FALSE	0.41	TRUE	TRUE	0.31	FALSE	FALSE
GPR183	-1.81	9.57E-08	0.18	FALSE	FALSE	0.23	TRUE	FALSE	0.13	FALSE	FALSE
KANK4	-1.69	9.71E-08	0.25	FALSE	FALSE	0.25	TRUE	TRUE	0.25	FALSE	FALSE
SLC24A3	-1.72	1.22E-07	0.21	FALSE	FALSE	0.27	TRUE	FALSE	0.17	FALSE	FALSE
APCDD1	-1.8	1.25E-07	0.22	FALSE	FALSE	0.34	TRUE	TRUE	0.14	FALSE	FALSE
G0S2	-2.24	1.47E-07	0.21	FALSE	FALSE	0.22	TRUE	FALSE	0.22	FALSE	FALSE
CXCL6	-1.77	1.57E-07	0.14	FALSE	FALSE	0.29	TRUE	TRUE	0.22	FALSE	FALSE
TBC1D9	-1.53	1.99E-07	0.2	FALSE	FALSE	0.3	TRUE	TRUE	0.29	FALSE	FALSE
PLAT	-1.77	3.53E-07	0.16	FALSE	FALSE	0.29	TRUE	FALSE	0.19	FALSE	FALSE

	CTBP2	-1.54	4.32E-07	0.12	FALSE	FALSE	0.24	TRUE	FALSE	0.16	FALSE	FALSE
	MBNL2	-1.59	8.92E-07	0.18	FALSE	FALSE	0.34	TRUE	FALSE	0.4	FALSE	FALSE
	IGFBP7	-1.67	9.09E-07	0.16	FALSE	FALSE	0.33	TRUE	FALSE	0.19	FALSE	FALSE
	HGD	-1.53	3.57E-06	0.19	FALSE	FALSE	0.17	TRUE	FALSE	0.18	FALSE	FALSE
	LXN	-1.58	3.90E-05	0.15	FALSE	FALSE	0.26	TRUE	FALSE	0.16	FALSE	FALSE
	IL8	-1.57	1.40E-04	0.09	FALSE	FALSE	0.22	TRUE	TRUE	0.12	FALSE	FALSE
	TNFSF14	-1.51	2.22E-04	0.05	FALSE	FALSE	0.23	TRUE	FALSE	0.09	FALSE	FALSE
miR-221	CRHBP	-15.16	5.25E-56	0.41	FALSE	FALSE	0.46	FALSE	FALSE	0.37	TRUE	FALSE
	CXCL12	-10.44	2.22E-39	0.38	FALSE	FALSE	0.5	FALSE	FALSE	0.3	TRUE	FALSE
	FOS	-7.38	1.33E-34	0.3	FALSE	FALSE	0.48	FALSE	FALSE	0.35	TRUE	TRUE
	ZFP36	-3.62	2.29E-31	0.32	FALSE	FALSE	0.47	FALSE	FALSE	0.34	TRUE	FALSE
	ID2	-2.7	2.75E-30	0.4	FALSE	FALSE	0.46	FALSE	FALSE	0.43	TRUE	FALSE
	ETS2	-3.11	6.87E-30	0.35	FALSE	FALSE	0.44	FALSE	FALSE	0.33	TRUE	FALSE
	NAT2	-5.62	2.58E-29	0.36	FALSE	FALSE	0.41	FALSE	FALSE	0.36	TRUE	FALSE
	CYR61	-3.91	1.68E-28	0.36	FALSE	FALSE	0.44	FALSE	FALSE	0.32	TRUE	TRUE
	CCR1	-3.04	5.56E-28	0.35	FALSE	FALSE	0.45	FALSE	FALSE	0.34	TRUE	FALSE
	GPR128	-3.57	7.40E-28	0.43	FALSE	FALSE	0.36	FALSE	FALSE	0.33	TRUE	FALSE
	MAP2K1	-2.21	1.40E-27	0.38	FALSE	FALSE	0.39	FALSE	FALSE	0.42	TRUE	FALSE
	ATP6V1B2	-2	1.45E-26	0.35	FALSE	FALSE	0.36	FALSE	FALSE	0.31	TRUE	FALSE
	IL1RAP	-5.43	2.01E-26	0.37	FALSE	FALSE	0.4	FALSE	FALSE	0.35	TRUE	TRUE
	ASPA	-3.02	4.63E-26	0.37	FALSE	FALSE	0.37	FALSE	FALSE	0.31	TRUE	FALSE
	DACH1	-2.28	7.39E-26	0.34	FALSE	FALSE	0.43	FALSE	FALSE	0.34	TRUE	FALSE
	ATF3	-2.55	1.23E-25	0.3	FALSE	FALSE	0.41	FALSE	FALSE	0.33	TRUE	FALSE
	TCF21	-1.73	5.84E-25	0.3	FALSE	FALSE	0.43	FALSE	FALSE	0.27	TRUE	FALSE
	AQP3	-2.67	6.87E-25	0.27	FALSE	FALSE	0.38	FALSE	FALSE	0.27	TRUE	TRUE
	SLC9A9	-2.92	1.42E-24	0.36	FALSE	FALSE	0.44	FALSE	FALSE	0.27	TRUE	FALSE
	SLITRK6	-1.97	1.17E-23	0.35	FALSE	FALSE	0.43	FALSE	FALSE	0.34	TRUE	FALSE
	C6	-4.91	4.93E-23	0.43	FALSE	FALSE	0.4	FALSE	FALSE	0.41	TRUE	FALSE
	HEXIM1	-1.56	7.39E-22	0.36	FALSE	FALSE	0.28	FALSE	FALSE	0.27	TRUE	FALSE
	SUN2	-1.68	1.40E-21	0.32	FALSE	FALSE	0.38	FALSE	FALSE	0.26	TRUE	FALSE
	FAM134B	-6.04	1.71E-21	0.29	FALSE	FALSE	0.43	FALSE	FALSE	0.47	TRUE	FALSE
	OR2H1	-1.56	5.47E-21	0.32	FALSE	FALSE	0.36	FALSE	FALSE	0.24	TRUE	FALSE
	DIRAS3	-1.91	8.42E-21	0.36	FALSE	FALSE	0.39	FALSE	FALSE	0.34	TRUE	TRUE
	PPP1R15A	-1.61	1.34E-20	0.25	FALSE	FALSE	0.36	FALSE	FALSE	0.19	TRUE	FALSE
	GIMAP4	-2.27	2.81E-20	0.29	FALSE	FALSE	0.37	FALSE	FALSE	0.28	TRUE	FALSE
	IGF1	-2.96	3.44E-20	0.34	FALSE	FALSE	0.35	FALSE	FALSE	0.37	TRUE	FALSE
	PTGER2	-1.62	4.05E-19	0.27	FALSE	FALSE	0.39	FALSE	FALSE	0.24	TRUE	FALSE
	NAMPT	-2.71	2.35E-18	0.31	FALSE	FALSE	0.36	FALSE	FALSE	0.38	TRUE	FALSE
	GPR126	-1.7	2.92E-18	0.28	FALSE	FALSE	0.37	FALSE	FALSE	0.31	TRUE	FALSE
	SEC62	-1.87	3.22E-18	0.33	FALSE	FALSE	0.4	FALSE	FALSE	0.4	TRUE	FALSE
	KYNU	-2.13	4.73E-18	0.18	FALSE	FALSE	0.35	FALSE	FALSE	0.26	TRUE	FALSE
	BBOX1	-4.74	6.40E-18	0.33	FALSE	FALSE	0.23	FALSE	FALSE	0.3	TRUE	FALSE
	EIF5	-2.11	1.08E-17	0.31	FALSE	FALSE	0.4	FALSE	FALSE	0.36	TRUE	TRUE
	FGFR2	-3.5	1.09E-17	0.27	FALSE	FALSE	0.38	FALSE	FALSE	0.19	TRUE	FALSE
	ELF1	-1.54	1.92E-17	0.32	FALSE	FALSE	0.34	FALSE	FALSE	0.29	TRUE	FALSE
	PNRC1	-1.76	2.91E-17	0.31	FALSE	FALSE	0.32	FALSE	FALSE	0.27	TRUE	FALSE
	ACADL	-2.47	4.74E-17	0.35	FALSE	FALSE	0.3	FALSE	FALSE	0.31	TRUE	FALSE
	GPR126	-2.36	7.39E-17	0.29	FALSE	FALSE	0.4	FALSE	FALSE	0.35	TRUE	FALSE
	SLC6A5	-1.57	1.02E-16	0.32	FALSE	FALSE	0.29	FALSE	FALSE	0.16	TRUE	FALSE
	MAF	-1.75	5.71E-16	0.31	FALSE	FALSE	0.33	FALSE	FALSE	0.24	TRUE	FALSE
	FLJ22763	-2.42	6.12E-16	0.33	FALSE	FALSE	0.24	FALSE	FALSE	0.27	TRUE	FALSE
	SLC45A3	-1.96	9.35E-16	0.15	FALSE	FALSE	0.25	FALSE	FALSE	0.17	TRUE	FALSE
	ID4	-2.44	5.46E-15	0.25	FALSE	FALSE	0.3	FALSE	FALSE	0.17	TRUE	FALSE
	GRK5	-1.65	7.86E-15	0.34	FALSE	FALSE	0.34	FALSE	FALSE	0.22	TRUE	FALSE
	TNN	-1.64	3.93E-14	0.24	FALSE	FALSE	0.33	FALSE	FALSE	0.19	TRUE	FALSE
	CDKN1C	-2.35	4.05E-14	0.24	FALSE	FALSE	0.4	FALSE	FALSE	0.2	TRUE	FALSE
	SLC3A1	-3.49	1.10E-13	0.17	FALSE	FALSE	0.39	FALSE	FALSE	0.22	TRUE	FALSE
	PPARGC1A	-2.41	1.38E-13	0.28	FALSE	FALSE	0.31	FALSE	FALSE	0.34	TRUE	TRUE
	CCDC146	-2.2	2.19E-13	0.3	FALSE	FALSE	0.35	FALSE	FALSE	0.3	TRUE	FALSE
	CYP4X1	-1.63	4.11E-13	0.33	FALSE	FALSE	0.26	FALSE	FALSE	0.24	TRUE	FALSE
	CPS1	-2.82	4.30E-13	0.29	FALSE	FALSE	0.25	FALSE	FALSE	0.27	TRUE	FALSE
	SEC24B	-1.64	5.62E-13	0.3	FALSE	FALSE	0.27	FALSE	FALSE	0.32	TRUE	TRUE
	TTC7B	-1.65	5.64E-13	0.25	FALSE	FALSE	0.31	FALSE	FALSE	0.19	TRUE	FALSE
	CITED2	-1.56	1.94E-12	0.22	FALSE	FALSE	0.23	FALSE	FALSE	0.2	TRUE	FALSE
	SERPINA7	-2.95	2.72E-12	0.33	FALSE	FALSE	0.17	FALSE	FALSE	0.16	TRUE	FALSE
	ANXA3	-2.08	5.46E-12	0.27	FALSE	FALSE	0.36	FALSE	FALSE	0.25	TRUE	FALSE
	ARHGEF17	-1.54	6.03E-12	0.15	FALSE	FALSE	0.31	FALSE	FALSE	0.22	TRUE	FALSE
	ALDH5A1	-1.77	1.68E-11	0.31	FALSE	FALSE	0.21	FALSE	FALSE	0.2	TRUE	FALSE
	COL4A4	-1.7	2.01E-11	0.28	FALSE	FALSE	0.2	FALSE	FALSE	0.12	TRUE	FALSE
	ADD3	-1.57	3.70E-11	0.23	FALSE	FALSE	0.25	FALSE	FALSE	0.17	TRUE	FALSE
	UGT2B15	-3.17	5.47E-11	0.24	FALSE	FALSE	0.24	FALSE	FALSE	0.24	TRUE	FALSE
	METTL7A	-1.8	7.37E-11	0.24	FALSE	FALSE	0.31	FALSE	FALSE	0.24	TRUE	FALSE
	MTTP	-2.4	1.25E-10	0.32	FALSE	FALSE	0.23	FALSE	FALSE	0.22	TRUE	FALSE

	CHSY1	-1.68	8.86E-10	0.23	FALSE	FALSE	0.28	FALSE	FALSE	0.19	TRUE	TRUE
	ARRDC4	-2.03	1.01E-09	0.25	FALSE	FALSE	0.17	FALSE	FALSE	0.29	TRUE	TRUE
	MST1P9	-1.89	2.60E-09	0.27	FALSE	FALSE	0.25	FALSE	FALSE	0.25	TRUE	FALSE
	LAP3	-1.53	6.86E-09	0.25	FALSE	FALSE	0.23	FALSE	FALSE	0.19	TRUE	FALSE
	TUSC3	-1.94	9.04E-09	0.16	FALSE	FALSE	0.36	FALSE	FALSE	0.21	TRUE	FALSE
	NPR3	-1.93	3.14E-08	0.22	FALSE	FALSE	0.31	FALSE	FALSE	0.31	TRUE	FALSE
	DDX3Y	-1.63	1.65E-07	0.17	FALSE	FALSE	0.21	FALSE	FALSE	0.29	TRUE	FALSE
	SH3BGRL	-1.62	2.49E-07	0.16	FALSE	FALSE	0.25	FALSE	FALSE	0.24	TRUE	FALSE
	ZNF385B	-1.51	8.99E-07	0.26	FALSE	FALSE	0.25	FALSE	FALSE	0.24	TRUE	FALSE
	ATP11C	-1.53	2.10E-06	0.17	FALSE	FALSE	0.14	FALSE	FALSE	0.24	TRUE	FALSE
	INSIG1	-1.74	6.14E-06	0.12	FALSE	FALSE	0.2	FALSE	FALSE	0.2	TRUE	TRUE

Table S5. Genes ranked by their product between correlation with miRNA and MiRanda prediction score for down-regulated miRNAs.Genes with multiple transcripts were labeled with "_t1", "_t2" for transcript 1, transcript 2, etc.

Symbol	Differential expression		*miR-26a*				*miR-122*				*miR-130a*			
	FC	FDR	Correlation	Score	Prod (norm)	Rank	Correlation	Score	Prod (norm)	Rank	Correlation	Score	Prod (norm)	Rank
CEP41	1.74	1.13E-21	0.36	156	38.41	1	0.3	142	28	26	0.41	137	35.3	78
CCDC93_t1	1.22	6.25E-14	0.37	152	37.44	2	NA	NA	NA	NA	0.38	151	38.72	28
IMPAD1	1.52	2.37E-17	0.37	148	36.6	3	0.24	134	20.44	347	0.44	133	36.2	63
UBE2H	1.19	8.45E-06	0.32	160	35.43	4	0.12	146	11.17	2411	0.26	139	22.79	865
DSCC1	1.69	9.16E-20	0.35	149	34.87	5	0.29	125	21.54	246	0.49	135	41.92	10
TMEM69	1.38	3.36E-19	0.31	160	33.89	6	0.27	116	17.76	706	0.34	128	26.23	481
EZH2	2.75	1.77E-32	0.35	147	33.75	7	0.23	111	14.11	1504	0.45	115	29.05	295
RRP15	1.59	1.36E-17	0.34	148	33.73	8	0.24	128	19	517	0.39	126	29.73	255
DCTN4	1.28	9.35E-09	0.32	154	33.7	9	0.19	122	13.77	1610	0.36	135	30.94	199
SORT1_t1	1.66	7.81E-15	0.35	147	33.58	10	0.23	157	24.41	100	NA	NA	NA	NA
TMEM206	1.47	2.49E-16	0.38	138	33.32	11	0.32	122	22.96	153	0.37	130	29.43	268
NSL1	1.49	1.98E-20	0.32	155	33.31	12	0.23	143	21.01	293	0.37	136	31.6	182
MGAT4A	1.38	3.76E-09	0.34	147	32.96	13	0.21	147	20.18	381	0.24	141	21.9	981
STMN1	3.2	6.18E-29	0.35	143	32.84	14	0.26	110	15.38	1174	0.4	129	31.24	193
DEPDC1	2.17	5.72E-25	0.34	147	32.56	15	0.27	130	21.55	244	0.43	152	43.75	5
TTC13	1.57	2.40E-16	0.33	149	32.49	16	0.21	111	12.62	1950	0.39	125	29.15	288
DLG5	1.88	4.68E-16	0.29	162	32.47	17	0.22	131	17.92	682	0.37	155	38.61	29
CDC6	2.37	5.68E-24	0.31	154	32.4	18	0.26	128	20.11	392	0.42	144	39.08	23
KLHL12_t1	1.59	1.10E-24	0.39	134	32.35	19	NA	NA	NA	NA	0.44	132	36.41	60
RACGAP1_t1	2.8	9.47E-33	0.36	139	32.32	20	NA	NA	NA	NA	NA	NA	NA	NA
ZNF623	1.38	1.39E-21	0.36	140	32.25	21	0.25	143	22.98	152	0.4	128	31.07	195
SLC30A6	1.19	7.26E-13	0.31	154	32.23	22	0.27	131	21.88	226	0.31	146	29.71	256
PTCD1	1.96	1.16E-29	0.35	141	32.2	23	0.29	136	24.53	98	0.41	132	33.42	111
PTTG3P	1.76	1.63E-26	0.3	156	32.17	24	0.24	133	19.73	427	0.4	127	30.6	215
KTI12	1.25	4.35E-13	0.32	151	32.15	25	0.15	120	10.82	2546	0.19	119	12.9	2922
GRPEL2_t1	1.35	5.71E-20	0.36	139	32.08	26	NA	NA	NA	NA	NA	NA	NA	NA
USP46	1.13	1.73E-05	0.33	148	32.03	27	0.21	151	21.3	258	0.3	162	33.62	104
USP39	1.35	1.39E-20	0.36	138	31.77	28	0.28	115	18.48	587	0.26	120	18.34	1583
FPGT	1.35	1.40E-13	0.3	157	31.64	29	0.21	148	20.71	323	0.35	133	29.33	280
ENAH_t1	2.62	3.81E-20	0.34	144	31.59	30	0.26	130	20.68	325	0.39	147	37.7	43
UBE2A	1.43	2.77E-13	0.29	158	31.57	31	0.21	129	16.86	867	0.37	153	38.18	33
NCAPH	2.22	1.56E-27	0.33	144	31.49	32	0.26	132	21.38	253	0.4	127	30.79	205
PPIA	1.73	7.50E-25	0.33	144	31.45	33	0.33	111	20.15	389	0.37	123	27.05	418
ARF1	1.48	1.83E-17	0.34	141	31.04	34	0.3	127	22.93	156	0.32	126	24.37	683
ANKRD27	1.61	1.27E-20	0.35	139	30.82	35	0.33	145	31.04	5	0.33	108	19.12	1432
KIF11	1.63	2.16E-19	0.31	149	30.7	36	0.23	155	24.45	99	0.36	130	29.02	300
SOAT1	1.21	0.000136616	0.32	144	30.45	37	0.21	131	17.32	773	0.2	131	16.34	1996
RQCD1	1.3	6.19E-17	0.29	154	30.44	38	0.29	121	20.57	332	0.28	138	24.47	668
PHF6_t1	1.6	7.63E-14	0.31	147	30.3	39	0.29	164	33.55	3	0.42	123	30.46	224
TTK	1.96	7.68E-24	0.31	149	30.25	40	0.27	126	20.17	383	0.39	121	27.85	380
RNMT	1.2	1.54E-15	0.29	156	30.24	41	0.16	134	13.84	1581	0.31	137	26.79	434
CNOT6	1.35	1.87E-10	0.32	145	30.05	42	0.26	139	22.95	155	0.32	147	30.77	206
NRAS	1.49	7.26E-16	0.32	143	29.89	43	0.26	127	19.9	410	0.39	139	35.12	81
CPSF6	1.49	5.49E-27	0.32	142	29.64	44	0.26	147	25.4	70	0.37	147	35.87	71
WHSC1_t1	1.39	3.59E-15	0.28	157	29.61	45	0.2	146	19.31	477	0.31	130	24.72	648
MED20	1.43	3.25E-23	0.29	151	29.47	46	0.25	136	21.57	241	0.39	127	29.66	257
H2AFZ_t1	2.08	1.89E-23	0.36	132	29.42	47	NA	NA	NA	NA	0.36	121	25.87	519
CASP2	1.33	3.68E-13	0.33	139	29.31	48	0.24	125	17.72	711	0.33	139	29.19	285
GTF2E1	1.37	1.67E-18	0.3	146	29.21	49	0.22	129	17.46	753	0.27	135	22.78	867
CASK	1.48	9.80E-18	0.3	147	29.2	50	0.14	140	12.53	1975	0.41	137	35.67	73
SKA1_t1	1.08	3.02E-09	0.32	142	29.17	51	NA	NA	NA	NA	NA	NA	NA	NA
NEK2	2.23	7.61E-25	0.33	138	29.15	52	0.24	131	19.08	506	0.46	119	31.53	183
NOX4_t1	1.6	4.54E-15	0.32	140	29.06	53	NA	NA	NA	NA	0.35	152	36.12	66
CPSF3	1.38	7.88E-18	0.31	142	28.9	54	0.2	89	7.72	3873	0.4	104	21.43	1058
CENPA	2.38	4.16E-25	0.32	141	28.74	55	0.28	117	18.48	588	0.4	138	34.85	83
FLVCR1	2.26	6.81E-23	0.3	145	28.73	56	0.23	136	19.93	407	0.36	139	32.07	156
GTSE1_t1	1.48	1.08E-19	0.26	160	28.68	57	NA	NA	NA	NA	NA	NA	NA	NA
HEATR1	1.61	7.94E-20	0.31	142	28.68	58	0.29	132	23.4	135	0.38	141	34.17	97
PHTF2	1.17	4.51E-16	0.27	157	28.67	59	0.24	144	22.39	190	0.31	150	31.28	191
NUDCD1_t1	1.3	1.65E-09	0.3	147	28.67	60	NA	NA	NA	NA	NA	NA	NA	NA
DNAL1	1.29	1.85E-14	0.27	155	28.55	61	0.19	139	16.59	924	0.34	158	36.21	62
INTS7	1.4	2.17E-12	0.28	150	28.45	62	0.14	120	9.57	3031	0.37	121	26.47	459
KIF18A	1.58	5.97E-19	0.28	151	28.41	63	0.24	104	13.2	1773	0.41	111	24.77	641
NOL10	1.22	9.31E-14	0.27	156	28.36	64	0.22	132	18.25	617	0.35	143	32.43	145
SLC33A1	1.29	3.39E-10	0.27	155	28.29	65	0.12	121	8.67	3424	0.24	122	17.56	1723
SEC22C_t1	1.31	3.26E-13	0.3	144	28.29	66	0.27	152	27.81	28	0.29	136	24.94	622
FANCA	1.6	2.84E-19	0.27	153	28.25	67	0.27	120	18.88	539	0.28	142	25.43	570
RBBP5_t1	1.22	1.00E-14	0.29	146	28.17	68	NA	NA	NA	NA	0.36	139	32.34	149
SAE1	1.78	1.06E-25	0.34	133	28.07	69	0.26	129	20.7	324	0.33	126	25.19	599
DLX4	1.44	6.61E-26	0.33	136	28.06	70	0.25	126	18.96	525	0.41	141	37.67	45
CKS2	2.09	2.16E-21	0.25	161	28.05	71	0.18	97	8.49	3505	0.38	140	34.1	99
CBX1_t1	1.73	3.86E-20	0.34	133	28	72	NA	NA	NA	NA	0.41	128	32.15	155
RAB3GAP2	1.36	7.98E-06	0.3	144	27.98	73	0.24	139	21.21	266	0.36	139	31.62	180
RAB11FIP4_t1	1.71	4.10E-11	0.29	147	27.98	74	0.15	145	14.64	1369	NA	NA	NA	NA
RNF2	1.35	2.09E-13	0.31	139	27.96	75	0.27	134	22.27	198	0.32	143	29.42	269
GPSM2_t1	1.29	2.13E-17	0.31	141	27.85	76	NA	NA	NA	NA	NA	NA	NA	NA
UBE2T	3.57	1.08E-35	0.33	135	27.82	77	0.24	83	8.02	3733	0.43	122	30.6	216
CREB1	1.11	6.23E-09	0.27	152	27.77	78	0.18	148	17.69	719	0.25	156	27.02	421
UBTD2	1.24	4.29E-15	0.3	142	27.77	79	0.23	126	17.83	696	0.33	124	24.11	706
PBK	3.38	1.64E-28	0.32	136	27.76	80	0.23	120	16.16	1008	0.43	138	37.68	44
CDC73	1.32	1.14E-08	0.26	158	27.76	81	0.22	129	17.68	723	0.31	144	28.86	313
MPZL1_t1	1.8	3.48E-22	0.32	137	27.65	82	NA	NA	NA	NA	0.36	139	31.71	174
ZNF250	1.25	2.64E-08	0.31	139	27.62	83	0.08	138	7.21	4132	0.26	158	28.02	369
DBF4_t1	1.12	1.84E-12	0.32	136	27.61	84	NA	NA	NA	NA	NA	NA	NA	NA
ATAD2	1.88	1.82E-17	0.27	151	27.58	85	0.21	121	14.67	1360	0.38	120	26.41	465
ZFP64_t1	1.49	1.48E-23	0.26	156	27.57	86	0.19	138	16.99	842	0.33	120	23.41	797
NEIL3	1.39	1.82E-16	0.3	140	27.45	87	0.18	113	11.58	2278	0.39	104	21.01	1118
SLC7A6_t1	1.19	1.02E-15	0.25	158	27.44	88	NA	NA	NA	NA	0.24	138	21.51	1047
RRAGC	1.4	4.94E-10	0.34	130	27.43	89	0.22	124	16.4	960	0.27	149	26.79	433
RPP30	1.23	2.33E-10	0.3	141	27.43	90	0.16	136	14.14	1498	0.23	121	16.5	1955
FANCI	2.1	1.60E-23	0.3	141	27.38	91	0.25	110	14.9	1306	0.42	120	29.61	260
SF3B3_t1	1.14	5.16E-06	0.27	150	27.37	92	NA	NA	NA	NA	0.15	135	12.45	3049
RALA	1.42	6.12E-15	0.32	135	27.35	93	0.28	130	22.58	181	0.34	147	32.72	131
PRKAA2	1.28	5.46E-15	0.29	145	27.29	94	0.24	143	22.09	206	0.36	163	40.86	14
TCHP	1.33	6.95E-19	0.29	144	27.27	95	0.18	135	15.29	1196	0.35	126	26.88	426
ECT2_t1	3.3	3.08E-27	0.32	135	27.21	96	0.3	120	20.83	311	NA	NA	NA	NA
TAF2	1.33	1.98E-08	0.29	142	27.14	97	0.2	124	14.45	1414	0.38	131	30.84	202
SOCS7_t1	1.11	6.77E-14	0.27	152	27.1	98	0.22	130	17.64	728	NA	NA	NA	NA
UCK2	1.9	1.23E-27	0.28	148	27.08	99	0.23	132	19.06	510	0.36	139	32.22	154
H2AFV	2	2.38E-30	0.3	140	27	100	0.31	103	16.46	946	0.38	134	31.8	168
RRM2	2.12	4.03E-30	0.34	130	26.96	101	0.24	124	18.05	653	0.44	133	36.37	61
ZKSCAN5	1.35	1.97E-16	0.34	129	26.9	102	0.26	130	20.87	303	0.4	135	34.39	90

SLC1A4	1.31	6.28E-15	0.26	154	26.9	103	0.16	134	13.83	1589	0.22	126	16.35	1991
RRAGD	1.94	8.67E-18	0.3	140	26.85	104	0.15	122	11.15	2419	0.33	147	32.31	150
DCP2	1.11	1.22E-10	0.28	145	26.84	105	0.13	140	11.75	2216	0.26	140	23.08	836
CENPJ	1.5	4.79E-11	0.26	153	26.84	106	0.28	120	19.34	474	0.27	108	15.89	2101
KCMF1	1.21	2.65E-10	0.31	136	26.83	107	0.22	143	20.83	310	0.25	150	25.49	564
KPNA2	2.03	1.87E-24	0.28	146	26.8	108	0.27	121	19	518	0.35	128	27.08	416
SQLE	3.76	5.81E-21	0.33	131	26.79	109	0.28	93	12.16	2082	0.35	125	26.39	466
LGALS8	1.35	3.34E-07	0.3	140	26.78	110	0.15	160	16.32	977	0.2	152	20.73	1168
DPH2	1.72	4.07E-23	0.29	141	26.77	111	0.25	137	22.13	203	0.3	120	21.16	1092
CHEK1	2.12	2.43E-21	0.29	142	26.69	112	0.26	138	22.87	159	0.37	129	29.49	267
CCNB1	4.1	2.32E-35	0.33	132	26.68	113	0.24	114	15.52	1140	0.43	142	39.32	20
SNX27_t1	1.57	4.08E-24	0.35	127	26.65	114	NA	NA	NA	NA	0.33	144	30.72	209
UBE3C	1.21	3.97E-09	0.29	143	26.63	115	0.26	132	21.55	243	0.29	117	19.3	1387
XPR1	1.52	1.07E-20	0.27	149	26.6	116	0.23	152	23.32	138	0.42	144	39.4	19
RABIF_t1	1.18	2.04E-15	0.34	128	26.58	117	NA	NA	NA	NA	0.39	146	37.85	38
PFN4	1.34	1.07E-14	0.22	170	26.58	118	0.19	100	9.48	3072	0.24	114	15.07	2313
CHML	1.65	1.73E-10	0.26	154	26.57	119	0.19	146	18.11	640	0.37	129	29.37	274
CSE1L	1.44	1.73E-11	0.29	141	26.57	120	0.23	123	16.83	869	0.31	130	25.2	597
DTL	2.11	1.20E-22	0.3	139	26.55	121	0.19	108	11	2475	0.41	121	28.89	311
MAD2L1	2.41	1.18E-23	0.31	136	26.54	122	0.24	129	19.09	504	0.4	129	31.86	167
HTR4	1.46	2.05E-10	0.27	150	26.52	123	0.18	143	16.67	901	0.2	122	14.64	2424
ARHGEF39	1.43	3.16E-22	0.25	155	26.41	124	0.2	138	17.79	700	0.32	126	24.36	685
STX6	1.32	3.92E-16	0.31	135	26.39	125	0.28	167	33.34	4	0.29	167	33.35	112
ORC6	1.75	4.34E-20	0.31	136	26.38	126	0.3	135	25.17	74	0.39	128	30.52	222
SNRPD1	1.5	6.79E-16	0.31	135	26.33	127	0.27	126	20.38	355	0.3	125	22.7	879
BOD1_t1	1.23	1.16E-10	0.29	141	26.3	128	0.23	125	17.32	774	NA	NA	NA	NA
TMEM38B_t1	1.79	3.77E-17	0.26	150	26.29	129	NA	NA	NA	NA	0.42	135	35.56	75
ENAH_t2	1.55	1.78E-20	0.28	144	26.26	130	0.28	130	22.61	177	0.35	147	33.54	108
MECOM_t1	1.3	2.78E-09	0.3	138	26.23	131	NA	NA	NA	NA	NA	NA	NA	NA
TMEM68	1.4	1.58E-15	0.29	141	26.21	132	0.24	124	17.94	676	0.43	124	31.77	170
DIEXF	1.39	1.15E-11	0.29	139	26.16	133	0.17	135	14.78	1336	0.31	137	27.21	411
GMFB_t1	1.29	4.85E-07	0.26	150	26.15	134	NA	NA	NA	NA	0.33	140	29.99	242
ATP2C1_t1	1.38	3.00E-09	0.3	136	26.09	135	0.23	132	18.66	563	0.31	154	32.56	138
LPGAT1	1.62	4.17E-10	0.28	143	26.08	136	0.19	134	15.97	1056	0.35	149	34.76	85
APLN_t1	2.43	9.31E-23	0.3	136	26.05	137	0.16	124	12.17	2079	NA	NA	NA	NA
PNO1	1.28	2.83E-12	0.29	139	26.03	138	0.17	133	13.95	1547	0.32	120	22.74	872
POLR2K_t1	1.69	6.15E-21	0.33	129	25.99	139	0.24	127	18.53	581	NA	NA	NA	NA
SENP5	1.27	2.01E-14	0.26	149	25.96	140	0.17	135	14.33	1446	0.24	134	20.17	1255
CDCA8	3.41	2.41E-33	0.31	134	25.95	141	0.24	126	18.4	600	0.39	136	33.18	116
WDR26_t1	1.17	4.22E-07	0.27	147	25.92	142	NA	NA	NA	NA	0.33	138	28.96	306
SUMO3	1.16	0.00016878	0.28	144	25.91	143	0.08	122	5.94	4820	0.22	118	15.18	2282
ZNF238_t1	1.13	3.46E-07	0.22	167	25.9	144	NA	NA	NA	NA	0.27	146	25.65	544
FAM122B	1.4	1.05E-16	0.31	134	25.87	145	0.31	123	22.62	175	0.3	131	23.99	728
CENPBD1	1.35	4.90E-14	0.25	152	25.86	146	0.07	139	6.18	4674	0.24	150	24.2	699
ATP6V1C1_t1	1.25	8.39E-14	0.31	134	25.85	147	NA	NA	NA	NA	0.4	175	49.5	1
RFC3_t1	1.3	5.12E-20	0.25	152	25.84	148	0.23	139	20.31	366	0.31	127	23.56	781
UXS1	1.34	2.40E-15	0.29	139	25.78	149	0.18	138	16.13	1014	0.33	156	34.49	89
HACE1	1.33	1.64E-11	0.27	146	25.77	150	0.19	138	16.74	889	0.34	143	32.06	159
NSMCE2	1.83	1.13E-20	0.31	133	25.72	151	0.16	102	8.14	3674	0.35	110	20.74	1166
TTL	1.18	2.05E-17	0.31	133	25.69	152	0.28	136	23.76	121	0.3	125	22.74	869
PSMD10	1.58	1.52E-15	0.28	141	25.6	153	0.24	117	15.76	1101	0.38	126	28.72	319
B3GALNT2	1.43	6.33E-20	0.32	131	25.58	154	0.3	123	21.91	223	0.33	138	28.7	322
DSTYK	1.34	8.30E-17	0.27	146	25.58	155	0.26	161	28.5	20	0.28	153	29.07	293
CHCHD7	1.3	2.85E-08	0.28	140	25.56	156	0.21	120	14.98	1286	0.26	150	26.24	479
SIX4	1.36	5.82E-13	0.32	130	25.55	157	0.26	152	26.15	54	0.18	142	16.35	1992
RCOR3_t1	1.27	1.47E-09	0.26	147	25.53	158	NA	NA	NA	NA	NA	NA	NA	NA
REXO4	1.22	1.86E-12	0.25	152	25.5	159	0.19	114	12.07	2112	0.18	118	12.13	3155
SRP19	1.44	1.81E-15	0.25	150	25.48	160	0.23	127	17.49	747	0.38	119	26.05	498
CCNE1	1.85	8.34E-20	0.27	146	25.47	161	0.26	126	19.55	451	0.32	118	21.44	1055
RNF220	1.43	2.46E-26	0.31	132	25.45	162	0.27	122	19.12	495	0.3	144	28.14	364
POGK	1.72	2.99E-21	0.26	148	25.4	163	0.27	127	20.85	307	0.29	133	23.69	766
COPA	1.19	3.41E-16	0.27	144	25.39	164	0.17	131	13.64	1640	0.24	130	19.35	1373
WDYHV1	1.64	1.71E-20	0.35	122	25.34	165	0.26	108	15.23	1216	0.38	120	26.94	423
NCBP2	1.53	4.15E-19	0.36	120	25.3	166	0.29	149	28.78	15	0.29	130	23.14	830
TIMM50	1.34	2.93E-23	0.25	150	25.29	167	0.16	127	12.58	1960	0.24	116	15.68	2160
RAD18	1.37	2.24E-13	0.3	133	25.26	168	0.19	142	17.23	793	0.38	135	31.94	163
METTL13	1.51	2.28E-24	0.27	143	25.26	169	0.22	132	18.12	639	0.31	121	22.26	927
SNRNP27	1.32	1.56E-21	0.32	130	25.21	170	0.24	110	14.37	1432	0.32	145	30.12	235
WDHD1	1.51	1.65E-16	0.26	146	25.21	171	0.19	113	12.13	2092	0.34	126	25.94	513
CDCA5	3.44	1.08E-31	0.31	130	25.17	172	0.24	124	17.98	667	0.4	123	29.1	291
USP1	1.53	9.24E-11	0.3	134	25.17	173	0.2	115	12.91	1860	0.35	119	24.15	701
SNX16	1.25	7.23E-12	0.27	142	25.16	174	0.24	119	16.66	904	0.34	149	34.02	101
WHSC2	1.33	1.59E-15	0.31	131	25.16	175	0.21	111	12.81	1894	0.32	118	21.89	984
SRP9	1.55	1.06E-13	0.28	141	25.15	176	0.2	131	16.06	1029	0.38	116	24.8	634
FAM220A	1.58	2.94E-27	0.31	131	25.12	177	0.28	118	18.84	543	0.4	126	30.47	223
SPATA2	1.32	1.89E-18	0.35	121	25.12	178	0.21	137	18.15	635	0.37	152	37.42	48
HSPA8_t1	1.36	3.47E-08	0.25	151	25.11	179	NA	NA	NA	NA	NA	NA	NA	NA
TIMMDC1	1.35	4.19E-21	0.34	124	25.1	180	0.21	113	13.23	1761	0.25	126	18.83	1495
SASS6	1.26	5.14E-14	0.26	145	25.1	181	0.24	132	19.86	414	0.37	132	30.4	225
JRK	1.22	8.90E-16	0.31	130	25.04	182	0.21	147	20.13	391	0.29	136	24.72	649
GNPAT	1.69	2.02E-18	0.28	138	25.04	183	0.22	145	21.17	271	0.37	118	24.86	627
FAM189B	2.5	4.10E-34	0.32	128	25.03	184	0.24	126	18.5	585	0.39	140	35.36	77
HMMR	3.68	3.63E-32	0.29	135	25.02	185	0.24	115	15.28	1202	0.41	135	35.23	79
CENPP	1.93	2.98E-27	0.36	120	24.96	186	0.26	125	19.4	464	0.42	121	29.86	252
PTBP3_t1	1.17	3.35E-07	0.24	155	24.88	187	NA	NA	NA	NA	0.16	137	14.26	2533
RCN2	1.39	5.16E-10	0.24	154	24.85	188	0.29	139	26.12	56	0.31	128	24.14	702
ABL2	1.27	2.54E-08	0.25	151	24.84	189	0.21	147	20.17	384	0.15	136	12.66	2992
CHTOP	1.37	5.94E-20	0.29	136	24.78	190	0.27	119	18.62	570	0.28	152	28.49	334
DDX20	1.33	2.48E-14	0.29	134	24.78	191	0.24	120	17.03	833	0.35	124	25.59	547
CCDC34_t1	1.12	6.87E-12	0.26	144	24.77	192	0.13	112	8.27	3609	0.32	153	32.7	132
LANCL1	1.3	4.79E-14	0.28	137	24.75	193	0.23	139	20.32	364	0.33	129	26.02	500
CEP97	1.17	1.05E-12	0.31	131	24.73	194	0.2	124	15.16	1234	0.35	124	25.67	540
TMEM167A	1.63	1.27E-25	0.3	132	24.7	195	0.26	132	21.25	263	0.45	135	38.07	35
XPO5	1.52	6.96E-22	0.3	131	24.7	196	0.31	120	21.92	222	0.38	145	36.16	65
HSDL1	1.21	6.85E-10	0.32	127	24.68	197	0.13	129	10.03	2850	0.3	149	30.12	234
APOOL	1.32	6.83E-10	0.26	144	24.66	198	0.09	138	7.56	3960	0.26	153	27.03	420
OAZ1	1.42	5.59E-12	0.32	126	24.66	199	0.32	120	22.74	169	0.29	138	25.28	586
SSR3	1.37	4.25E-11	0.29	135	24.61	200	0.25	135	21.14	274	0.21	129	16.78	1882
KIN_t1	1.19	5.52E-08	0.25	150	24.56	201	0.25	141	22.43	189	NA	NA	NA	NA
SERBP1	1.32	4.96E-09	0.27	142	24.54	202	0.21	141	18.94	526	0.3	154	31.46	184
UBE2D2	1.28	9.06E-15	0.3	131	24.53	203	0.22	154	22.92	157	0.34	150	33.82	103
XPO4	1.2	1.94E-10	0.25	150	24.52	204	0.11	166	12.32	2029	0.33	143	30.35	227
CASC5	1.64	4.10E-19	0.26	146	24.51	205	0.22	105	12.24	2051	0.38	112	23.47	791
PYCR2	1.51	2.49E-20	0.38	115	24.51	206	0.28	115	18.47	589	0.34	122	24.83	630
ZNF92	1.5	1.28E-14	0.24	150	24.5	207	0.3	122	21.85	229	0.29	133	23.99	726
PARP1	1.51	1.64E-15	0.35	119	24.46	208	0.26	125	19.64	439	0.32	167	37.2	53

TRIM37_t1	1.49	1.65E-18	0.33	125	24.45	209	0.22	126	16.8	880	0.43	144	40.1	16
RAB10	1.38	1.88E-14	0.25	147	24.45	210	0.18	128	14.03	1522	0.28	132	22.69	880
DPY19L4	1.35	3.12E-09	0.31	130	24.44	211	0.18	135	15.65	1113	0.38	140	34.06	100
SLC25A40	1.36	1.48E-13	0.28	137	24.43	212	0.25	122	18.17	628	0.31	138	27.32	405
CDC27	1.23	1.45E-05	0.2	171	24.43	213	0.18	124	13.23	1762	0.25	130	20.03	1272
MRPS14	1.44	5.49E-19	0.28	137	24.42	214	0.19	124	14.33	1445	0.26	124	19.12	1427
TWF1	1.28	4.20E-05	0.23	156	24.42	215	0.16	141	14.73	1345	0.39	157	41.3	12
WDR45L	1.44	2.56E-15	0.31	129	24.39	216	0.26	119	17.64	729	0.25	143	23.28	812
RBM24	1.63	2.69E-15	0.23	154	24.39	217	0.17	144	15.98	1053	0.37	129	29.38	272
INCENP	1.86	4.04E-23	0.3	132	24.36	218	0.28	114	17.88	689	0.36	122	26.17	485
RCBTB1	1.46	6.73E-13	0.24	151	24.35	219	0.22	130	17.95	675	0.26	132	21.48	1053
SF3B14	1.22	9.98E-10	0.3	131	24.31	220	0.24	102	12.33	2025	0.22	124	16.32	2004
PSME3_t1	1.47	5.62E-18	0.29	134	24.31	221	NA	NA	NA	NA	0.37	134	30.86	201
UQCRH	1.73	2.51E-19	0.39	112	24.3	222	0.28	95	12.57	1967	0.46	95	20.57	1188
MAPKAPK5	1.38	6.47E-22	0.33	123	24.29	223	0.27	112	16.77	886	0.33	103	17.68	1706
HECTD2_t1	1.32	1.22E-10	0.3	132	24.28	224	0.23	119	16.03	1037	0.3	124	22.34	918
SCAMP3	1.75	2.25E-29	0.24	150	24.28	225	0.19	127	14.4	1424	0.31	88	11.84	3250
CCNA2	1.59	4.99E-23	0.28	138	24.27	226	0.21	118	14.2	1475	0.38	159	41.66	11
WARS2	1.37	5.92E-17	0.28	138	24.26	227	0.26	156	27.45	35	0.29	160	31.89	166
PPIH	1.39	9.10E-18	0.35	120	24.26	228	0.28	91	11.64	2261	0.3	98	14.57	2442
PTMA_t1	1.35	3.57E-09	0.26	142	24.25	229	NA	NA	NA	NA	0.21	118	14.1	2571
ZNF706	1.45	6.40E-10	0.28	138	24.23	230	0.26	131	20.97	297	0.3	135	25.14	606
ZNF572	1.72	5.83E-20	0.28	137	24.22	231	0.24	120	16.64	910	0.35	126	26.61	443
CBLL1	1.25	1.14E-14	0.27	138	24.19	232	0.24	118	16.57	930	0.27	129	21.05	1110
LEMD2	1.26	1.44E-16	0.25	146	24.17	233	0.2	121	14.17	1489	0.2	117	13.17	2838
RMI2	2.01	2.71E-21	0.29	133	24.16	234	0.2	117	13.66	1636	0.29	135	24.7	652
TIA1_t1	1.27	7.14E-17	0.29	134	24.15	235	0.26	124	19.42	460	0.28	138	24.73	643
FAM222B	1.31	1.20E-08	0.24	149	24.14	236	0.25	148	24.59	93	0.21	138	18.51	1550
POP1_t1	1.39	2.23E-18	0.27	138	24.1	237	NA	NA	NA	NA	0.31	130	24.44	677
LSM2	1.39	7.19E-19	0.22	160	24.06	238	0.19	114	12.29	2037	0.33	108	19.07	1437
PCNA	1.73	4.61E-18	0.25	146	24.02	239	0.15	94	6.59	4461	0.37	110	22.36	915
ATF2	1.12	1.86E-06	0.24	151	24.01	240	0.17	126	13.18	1778	0.27	144	25.21	596
SHCBP1	1.58	4.56E-17	0.27	140	24	241	0.26	140	23.69	123	0.38	130	30.57	219
ZFAT	1.48	2.20E-17	0.3	130	24	242	0.22	134	18.58	576	0.27	130	21.62	1031
LSM14B	1.28	7.12E-14	0.29	133	23.98	243	0.18	109	10.87	2526	0.28	129	21.99	969
DGKI	1.09	2.15E-08	0.24	150	23.94	244	0.16	119	11.04	2461	0.28	144	26.14	487
FBXO32	1.65	2.27E-13	0.25	144	23.94	245	0.17	142	15.99	1051	0.27	147	26.58	447
SF3A3	1.24	2.28E-07	0.27	140	23.93	246	0.17	129	13.22	1766	0.24	126	18.17	1612
DDHD2	1.13	4.08E-05	0.24	148	23.92	247	0.14	138	12.49	1981	0.19	140	17.34	1761
DUSP19_t1	1.1	3.37E-11	0.27	140	23.91	248	NA	NA	NA	NA	0.3	146	28.67	324
NSF_t1	1.35	4.33E-11	0.26	141	23.84	249	NA	NA	NA	NA	0.41	128	31.75	173
TPM3_t1	1.19	1.54E-09	0.26	143	23.83	250	NA	NA	NA	NA	NA	NA	NA	NA
IARS	1.57	1.72E-14	0.25	144	23.83	251	0.26	122	18.43	595	0.35	108	20.46	1203
VMA21	1.42	4.03E-15	0.24	148	23.81	252	0.15	140	13.59	1658	0.25	130	20.38	1217
MRPS23	1.61	2.16E-22	0.28	134	23.78	253	0.26	136	22.25	199	0.31	134	25.96	512
NSUN4	1.18	1.38E-06	0.28	136	23.77	254	0.13	144	12.45	1993	0.21	150	21.07	1106
HIST2H2BE	1.87	1.43E-20	0.3	129	23.77	255	0.14	124	10.01	2860	0.28	135	24.13	704
RUFY1	1.22	4.26E-07	0.3	129	23.74	256	0.19	130	15.3	1191	0.27	120	18.81	1502
TAF4	1.42	1.61E-14	0.29	132	23.73	257	0.19	125	14.2	1477	0.33	153	33.5	109
AZIN1	1.43	2.60E-08	0.28	135	23.7	258	0.21	132	16.83	871	0.3	139	26.46	461
ZNRF1_t1	1.17	2.83E-07	0.3	128	23.7	259	NA	NA	NA	NA	0.22	127	16.6	1932
ZNF37A	1.31	9.20E-17	0.24	147	23.68	260	0.31	131	25.45	68	0.26	135	22.07	956
TMED5	1.23	0.003401316	0.23	151	23.67	261	0.12	140	10.42	2711	0.27	138	23.39	803
MRPL13	1.55	9.95E-12	0.3	129	23.66	262	0.17	116	10.96	2488	0.37	110	22.18	938
RHEB	1.55	1.76E-17	0.23	151	23.64	263	0.23	139	20.42	349	0.3	130	24.24	696
E2F6_t1	1.24	1.03E-09	0.24	148	23.64	264	0.15	121	10.49	2683	NA	NA	NA	NA
MLF1IP	2.52	1.09E-25	0.31	127	23.62	265	0.21	142	19.73	428	0.36	120	25.14	607
HSPB11	1.53	6.13E-18	0.32	123	23.6	266	0.25	110	15.11	1247	0.32	111	19.31	1384
CXorf23	1.17	4.23E-05	0.22	155	23.6	267	0.16	143	15.22	1218	0.2	150	20.16	1258
LRRC59	1.55	6.85E-18	0.29	130	23.59	268	0.21	138	18.58	575	0.31	125	23.43	795
TPGS2_t1	1.58	9.72E-14	0.27	138	23.56	269	0.2	147	19.14	492	NA	NA	NA	NA
KPNA4	1.23	0.00078181	0.26	140	23.56	270	0.21	131	16.73	890	0.27	133	22.25	928
NKIRAS2	1.29	4.74E-17	0.22	157	23.54	271	0.19	129	14.79	1333	0.25	121	17.56	1724
TSN	1.34	1.17E-15	0.27	137	23.51	272	0.23	129	18.25	618	0.22	131	18.08	1627
NEK4	1.47	1.71E-14	0.23	152	23.51	273	0.23	121	16.36	969	0.39	117	26.1	491
CEP55	2.32	1.93E-21	0.3	128	23.48	274	0.26	155	27.49	34	0.4	156	42.9	7
AP2B1	1.24	1.42E-08	0.26	139	23.47	275	0.25	126	19.35	470	0.15	135	12.91	2916
COMMD5	1.42	4.25E-14	0.31	126	23.46	276	0.13	123	9.3	3144	0.25	118	16.95	1840
XPOT	1.46	8.15E-14	0.25	143	23.46	277	0.24	124	18.1	643	0.3	124	22.51	897
GALNT10	1.2	2.11E-14	0.23	153	23.45	278	0.2	157	21.34	256	0.27	144	25.64	545
MRE11A	1.14	3.29E-11	0.25	142	23.43	279	0.25	137	22.06	211	0.24	121	16.72	1897
AIDA	1.22	8.27E-08	0.28	135	23.43	280	0.17	119	11.64	2264	0.35	155	36.44	59
BIRC5	5.12	2.55E-36	0.29	130	23.42	281	0.23	128	17.92	680	0.39	122	27.85	381
CCT5	1.81	4.95E-18	0.3	129	23.4	282	0.33	126	24.98	81	0.37	135	31.44	185
INF2_t1	1.72	3.23E-16	0.29	130	23.39	283	0.25	115	16.29	981	0.3	130	24	725
CDKN3	3.91	9.89E-38	0.33	120	23.39	284	0.23	113	14.2	1482	0.45	115	28.93	309
ZNF217	1.19	0.000847825	0.23	153	23.38	285	0.1	121	7.14	4171	0.17	156	18.12	1621
MMP12	1.45	4.08E-08	0.26	139	23.37	286	0.22	106	12.16	2080	0.28	121	19.84	1296
TMEM65	1.33	3.99E-10	0.29	131	23.37	287	0.28	155	29.78	10	0.27	141	25.02	618
DSC1	1.18	2.62E-17	0.29	130	23.37	288	0.15	129	12.08	2109	0.31	149	30.33	228
SNX7	1.27	0.001651602	0.26	140	23.32	289	0.11	99	5.46	5079	0.24	111	14.81	2384
NUP62	1.75	1.07E-23	0.32	123	23.3	290	0.29	139	26.05	59	0.3	120	21.09	1104
APEX2	1.38	1.24E-25	0.27	136	23.3	291	0.11	128	8.53	3487	0.31	156	33.16	117
CLSPN	1.14	3.65E-14	0.24	148	23.25	292	0.28	147	27.02	39	0.29	145	27.54	395
TAF5L_t1	1.1	9.76E-07	0.27	137	23.23	293	0.16	126	12.18	2074	0.3	142	27.84	384
DIP2A_t1	1.22	1.97E-15	0.33	120	23.23	294	0.19	114	11.92	2150	0.27	113	16.9	1856
PTK2	1.38	1.97E-13	0.29	130	23.22	295	0.26	115	17.02	836	0.37	115	24.06	716
BMI1	1.57	4.24E-15	0.27	135	23.22	296	0.19	142	17.53	743	0.46	134	38.99	24
MTR	1.36	1.32E-09	0.27	136	23.15	297	0.15	132	12.22	2059	0.28	135	23.55	783
PRC1	2.69	6.63E-35	0.28	134	23.14	298	0.21	120	14.6	1374	0.39	132	31.93	164
TRAIP	2.09	1.58E-25	0.29	131	23.11	299	0.25	128	19.89	413	0.38	99	18.68	1526
DYNLL1_t1	1.6	9.94E-22	0.34	117	23.1	300	NA	NA	NA	NA	0.35	117	23.6	777
YWHAZ_t1	1.21	0.000505486	0.27	134	23.08	301	NA	NA	NA	NA	NA	NA	NA	NA
DESI2_t1	1.13	1.09E-10	0.27	134	23.08	302	NA	NA	NA	NA	NA	NA	NA	NA
EIF1AD_t1	1.23	5.61E-12	0.25	143	23.07	303	NA	NA	NA	NA	0.28	121	19.95	1279
GMNN	2.26	7.56E-20	0.29	129	23.07	304	0.29	120	20.49	340	0.36	130	28.93	310
TRIM11	1.56	9.08E-21	0.33	120	23.06	305	0.26	151	26.36	47	0.3	122	21.41	1062
TPX2	2.18	6.57E-26	0.25	141	23.04	306	0.2	121	14	1531	0.37	115	23.91	734
RNF38	1.08	7.18E-05	0.23	151	23.03	307	0.14	144	13.34	1732	0.2	152	20.28	1234
SESTD1	1.35	7.18E-11	0.24	147	23.03	308	0.17	145	16.28	984	0.38	169	45.55	4
CENPH	1.7	1.11E-21	0.29	130	23.02	309	0.24	128	19.07	508	0.39	133	32.52	141
FIGNL1	1.66	1.71E-21	0.26	138	23.02	310	0.27	123	19.44	459	0.39	120	27.36	400
EXOC8	1.24	1.29E-08	0.23	151	23.01	311	0.14	152	13.81	1594	0.31	131	24.8	635
MDK	4.1	1.91E-25	0.32	121	22.99	312	0.16	120	11.27	2373	0.36	102	18.66	1530
FAM49B	1.66	7.56E-12	0.29	129	22.97	313	0.28	144	26.03	60	0.32	148	31.04	196
GOLPH3L	1.42	3.52E-18	0.3	126	22.97	314	0.25	131	20.16	387	0.3	126	23.06	839

POLR3A_t1	1.1	5.18E-09	0.23	150	22.95	315	NA	NA	NA	NA	0.22	123	16.04	2064
DAP3	1.6	3.84E-18	0.28	131	22.93	316	0.29	135	25.02	80	0.39	134	32.78	128
ROBO1_t1	1.39	4.30E-16	0.23	148	22.9	317	NA	NA	NA	NA	NA	NA	NA	NA
TRIM45	2.07	1.33E-20	0.29	129	22.9	318	0.23	132	18.45	591	0.29	148	28.23	355
GYG1	1.29	5.99E-08	0.25	142	22.88	319	0.3	120	21.09	286	0.14	116	9.3	4117
SLC9A6	1.36	1.85E-12	0.23	150	22.87	320	0.16	154	16.79	882	0.38	147	37.2	54
KDELR2	1.43	1.38E-16	0.29	129	22.86	321	0.21	132	17.58	739	0.22	137	18.97	1462
ANAPC1	1.36	3.49E-14	0.29	128	22.85	322	0.33	131	26.66	44	0.35	144	32.67	133
GK5_t1	1.05	0.000106186	0.24	144	22.85	323	NA	NA	NA	NA	NA	NA	NA	NA
LRRC37B	1.25	1.95E-11	0.35	116	22.85	324	0.23	99	11.29	2365	0.33	105	18.04	1638
NAPB	1.09	2.69E-11	0.25	140	22.84	325	0.19	140	17.23	794	0.29	141	25.98	508
E2F2	2.21	4.66E-24	0.28	132	22.84	326	0.21	128	16.01	1044	0.34	145	32.41	147
RFX6	1.1	0.000131529	0.26	139	22.82	327	0.09	120	6.63	4432	0.2	130	16.2	2034
MRPL9	1.58	4.32E-25	0.29	129	22.8	328	0.23	140	20.35	361	0.33	120	23.22	817
MCM2	2.17	1.69E-23	0.27	135	22.8	329	0.13	109	7.58	3950	0.32	115	20.89	1136
ATP6AP1	1.53	6.60E-23	0.3	125	22.8	330	0.2	110	12.17	2076	0.32	127	24.73	646
FANCF	1.72	5.63E-24	0.21	157	22.79	331	0.26	117	17.09	821	0.28	130	22.09	953
TCEANC2	1.31	8.12E-18	0.24	145	22.79	332	0.22	142	20.45	346	0.3	132	24.71	650
PSRC1	1.53	5.01E-20	0.3	125	22.78	333	0.17	114	11.18	2408	0.33	110	20.09	1267
E2F8	2.09	3.06E-24	0.3	125	22.74	334	0.23	117	15.21	1220	0.41	142	37.67	46
PIGU	1.21	3.41E-16	0.33	119	22.74	335	0.17	103	8.87	3343	0.26	114	16.85	1862
USP33_t1	1.25	7.83E-10	0.25	141	22.72	336	0.25	131	20.32	363	0.29	147	28.22	356
TCEB1	1.67	4.46E-16	0.26	136	22.72	337	0.26	135	22.04	215	0.37	131	29.94	246
VANGL1	1.25	1.79E-12	0.23	150	22.7	338	0.24	149	24.02	111	0.25	154	25.78	532
KIF2A	1.5	6.34E-11	0.29	128	22.7	339	0.27	133	22.46	187	0.33	128	25.44	569
GORAB	1.41	1.07E-10	0.22	152	22.68	340	0.23	143	21.17	270	0.33	126	25.22	594
ZBTB41_t1	1.07	6.48E-10	0.25	141	22.67	341	NA	NA	NA	NA	NA	NA	NA	NA
VTI1A_t1	1.18	2.03E-09	0.25	140	22.66	342	0.09	157	9.76	2945	NA	NA	NA	NA
EME1	1.7	6.26E-25	0.31	123	22.66	343	0.27	120	19.12	496	0.4	115	26.08	496
MAPK1_t1	1.33	1.43E-16	0.23	149	22.64	344	0.21	135	17.59	736	0.28	151	28.69	323
ATP6V1H	1.26	3.62E-07	0.27	135	22.63	345	0.21	120	15.04	1265	0.29	129	23.13	831
GPR158	2.4	5.99E-23	0.27	134	22.62	346	0.27	140	24.63	92	0.39	150	38.8	27
UBXN2B	1.4	5.50E-13	0.23	147	22.61	347	0.13	131	10.5	2677	0.36	154	37.79	40
HILPDA	1.58	9.57E-07	0.25	140	22.6	348	0.19	120	13.52	1678	0.28	126	20.99	1120
UBAP2	1.22	1.04E-08	0.27	134	22.6	349	0.21	143	19.3	479	0.2	131	16.33	2003
DNAJC21	1.21	2.27E-07	0.22	151	22.59	350	0.12	138	10.24	2778	0.39	136	33.31	113
MCM8	1.66	1.62E-17	0.27	135	22.59	351	0.23	146	22.06	210	0.38	130	30.05	236
PSMB2	1.39	6.05E-14	0.26	136	22.58	352	0.16	131	12.86	1875	0.32	144	29.84	253
FEN1_t1	1.64	3.43E-25	0.22	153	22.57	353	NA	NA	NA	NA	0.34	110	20.15	1259
YEATS2	1.69	4.18E-20	0.3	124	22.55	354	0.33	135	28.1	24	0.34	125	25.5	559
REEP4	1.21	5.19E-09	0.21	159	22.55	355	0.12	121	8.29	3601	0.1	126	7.62	4784
TSEN15	1.47	3.39E-17	0.33	118	22.54	356	0.27	128	21.08	287	0.36	124	26.37	468
RAN	1.85	7.01E-22	0.3	125	22.54	357	0.31	109	18.11	641	0.33	121	23.69	764
ARHGAP11A_t1	1.54	1.25E-19	0.23	146	22.54	358	0.21	116	14.09	1509	0.37	127	28.75	317
CACYBP_t1	1.54	8.78E-12	0.28	131	22.51	359	NA	NA	NA	NA	0.41	137	35.82	72
ESCO2	1.71	4.12E-18	0.28	130	22.49	360	0.25	143	22.79	163	0.35	153	36.11	67
SNRPE	1.95	5.73E-28	0.35	115	22.49	361	0.27	144	25.14	76	0.44	146	42.22	8
RUVBL1_t1	1.34	4.50E-15	0.34	117	22.48	362	NA	NA	NA	NA	0.33	101	16.74	1891
SUV39H2	1.39	1.13E-13	0.26	136	22.48	363	0.19	130	15.55	1133	0.33	125	25.07	612
RFWD3	1.28	5.92E-13	0.24	145	22.46	364	0.12	136	10.24	2779	0.21	142	19.16	1418
CKAP2	2.1	2.35E-25	0.28	129	22.45	365	0.27	119	18.7	559	0.36	127	27.96	373
STAM	1.18	1.64E-10	0.24	142	22.44	366	0.13	129	10.23	2782	0.27	125	19.96	1277
CKS1B_t1	1.92	1.62E-28	0.35	114	22.44	367	NA	NA	NA	NA	0.43	110	25.51	557
COPS7B	1.15	3.44E-13	0.27	132	22.43	368	0.29	124	21.82	231	0.19	120	13.57	2717
DLAT	1.52	5.25E-16	0.23	147	22.43	369	0.26	156	27.43	36	0.29	121	20.3	1228
CHAF1B	1.84	2.62E-14	0.24	145	22.42	370	0.22	129	17.61	731	0.25	124	18.36	1578
TCF19	1.6	5.23E-21	0.3	125	22.41	371	0.2	130	16.02	1041	0.35	112	21.61	1033
MRS2	1.27	6.14E-08	0.28	129	22.39	372	0.2	127	15.08	1254	0.32	141	29.17	286
MMS22L_t1	1.36	9.31E-15	0.25	140	22.38	373	NA	NA	NA	NA	0.35	136	29.98	243
MANEAL	1.73	1.05E-16	0.29	127	22.36	374	0.07	135	5.99	4789	0.24	139	21.74	1011
TTF2	1.25	3.49E-14	0.26	135	22.36	375	0.23	148	22.74	170	0.25	138	21.8	1004
GDPD1	1.15	7.42E-11	0.26	136	22.35	376	0.05	140	4.67	5511	0.39	123	28.44	338
YKT6	1.56	4.94E-21	0.25	138	22.33	377	0.22	150	22.05	213	0.35	120	24.79	637
KHDRBS3	1.31	2.54E-07	0.26	135	22.33	378	0.09	108	5.46	5080	0.36	124	26.72	439
FAM208A	1.26	3.18E-07	0.23	148	22.32	379	0.22	128	17.41	761	0.35	144	32.55	139
RAD21	1.42	2.28E-06	0.24	143	22.32	380	0.24	154	24.58	94	0.32	124	23.55	782
LRRC57	1.31	1.07E-12	0.26	136	22.31	381	0.19	139	17.17	805	0.3	153	30.69	213
TERF1	1.45	2.63E-11	0.3	125	22.3	382	0.21	121	14.92	1300	0.39	130	30.81	204
COG5	1.3	1.48E-11	0.26	135	22.27	383	0.18	140	15.81	1088	0.39	148	38.22	31
CSTB	1.61	5.48E-16	0.32	119	22.27	384	0.24	126	18.59	573	0.25	125	19.12	1429
RNASEH1	1.41	1.11E-17	0.28	130	22.26	385	0.22	104	12.07	2111	0.3	120	20.82	1153
KIAA0196	1.51	1.84E-12	0.34	116	22.25	386	0.23	112	14.48	1408	0.42	124	30.76	207
ASPH_t1	1.56	1.73E-11	0.27	131	22.25	387	0.22	133	18.16	631	0.32	131	25.59	548
PUS7	1.64	4.57E-17	0.25	138	22.25	388	0.3	125	22.65	173	0.34	155	35.96	69
WHSC1_t2	1.62	1.77E-19	0.3	123	22.24	389	0.24	124	18.09	646	0.36	131	28.94	308
SP3	1.29	8.90E-06	0.22	153	22.18	390	0.21	143	19.81	422	0.22	130	17.27	1772
GEMIN5	1.18	3.75E-11	0.25	140	22.18	391	0.21	132	17.56	740	0.21	114	13.71	2673
SLC22A5	1.38	1.11E-12	0.2	161	22.15	392	0.22	129	17.09	822	0.31	129	24.69	653
FAM172A	1.16	4.53E-09	0.2	160	22.13	393	0.16	145	14.94	1295	0.23	139	20.59	1186
DSCR3_t1	1.19	6.09E-09	0.25	138	22.12	394	NA	NA	NA	NA	0.2	131	15.81	2128
UFD1L	1.51	2.91E-17	0.27	132	22.11	395	0.17	116	11.2	2399	0.31	145	29.58	262
MZT1	1.56	1.52E-19	0.29	126	22.09	396	0.38	156	40.39	2	0.33	148	32.66	134
KIF5B	1.15	0.000143694	0.25	138	22.09	397	0.22	140	19.72	429	0.22	125	16.4	1983
VOPP1	1.15	0.002235829	0.26	135	22.08	398	0.18	146	17.24	789	0.06	140	5.01	5915
EXOG_t1	1.23	3.71E-14	0.23	144	22.07	399	NA	NA	NA	NA	0.25	121	18.01	1647
CEP68	1.19	5.69E-16	0.25	140	22.07	400	0.17	136	14.97	1288	0.21	140	18.82	1498
HN1_t1	2.01	1.51E-20	0.25	138	22.05	401	NA	NA	NA	NA	0.29	140	26.34	470
SGTB	1.23	9.03E-12	0.26	136	22.05	402	0.24	132	19.97	403	0.3	150	30.03	237
POLR3G	1.21	3.27E-15	0.22	149	22.04	403	0.15	139	13.49	1687	0.32	131	25.97	509
TCEA1	1.2	2.43E-09	0.23	145	22.04	404	0.14	128	10.89	2521	0.3	143	27.71	389
RASGRF2	1.21	1.07E-07	0.23	146	22.04	405	0.1	130	8.19	3648	0.35	157	37.25	51
CCSAP_t1	1.23	2.65E-13	0.24	143	22.02	406	0.21	139	18.66	565	NA	NA	NA	NA
DENR	1.4	3.99E-13	0.23	146	22.01	407	0.27	130	21.39	251	0.34	125	25.35	580
HAUS3	1.32	2.06E-09	0.24	143	21.98	408	0.21	138	18.17	630	0.3	135	25.3	584
PRRC2C	1.53	2.58E-12	0.27	131	21.98	409	0.29	114	18.69	560	0.37	126	27.88	378
PLEKHA8	1.36	8.92E-17	0.31	120	21.97	410	0.25	105	13.5	1684	0.37	100	18.41	1566
COMMD2	1.24	7.62E-11	0.24	140	21.93	411	0.2	146	19.39	465	0.2	128	15.9	2098
ALG9	1.18	7.32E-06	0.22	150	21.93	412	0.19	142	17.6	732	0.21	139	18.9	1477
FAM199X	1.42	2.86E-11	0.22	150	21.91	413	0.21	149	21.02	291	0.28	128	22.18	939
CDKN2B_t1	1.12	2.04E-09	0.21	154	21.87	414	0.1	136	8.98	3286	0.27	147	25.86	520
SCAI	1.1	2.79E-08	0.25	139	21.87	415	0.18	137	16.09	1022	0.28	153	28.42	342
MTERF	1.23	7.38E-08	0.24	143	21.86	416	0.14	128	11.2	2401	0.28	127	21.78	1007
FAM83D	3.24	4.00E-29	0.3	124	21.86	417	0.2	131	16.33	974	0.43	127	33.16	118
ETV1_t1	1.21	1.08E-07	0.26	135	21.86	418	NA	NA	NA	NA	NA	NA	NA	NA
EFCAB2	1.72	5.15E-22	0.3	122	21.85	419	0.22	132	18.39	602	0.26	110	15.79	2134
HOXD8	1.21	1.48E-13	0.24	142	21.83	420	0.17	119	11.88	2157	0.31	126	23.41	798

KIAA1429_t1	1.56	8.94E-12	0.29	126	21.83	421	0.23	124	17.13	813	0.38	140	34.37	92
PDDC1_t1	1.14	2.58E-10	0.25	136	21.81	422	NA	NA	NA	NA	0.17	152	17.08	1810
DDA1_t1	1.1	3.25E-06	0.24	140	21.8	423	NA	NA	NA	NA	NA	NA	NA	NA
FNBP1L_t1	1.42	1.30E-07	0.24	140	21.78	424	0.22	133	17.88	688	0.34	134	28.95	307
LAMC1	1.7	1.22E-13	0.27	132	21.78	425	0.36	164	41.18	1	0.28	147	27.48	398
RBM8A_t1	1.3	2.70E-12	0.25	136	21.76	426	NA	NA	NA	NA	NA	NA	NA	NA
ASPH_t2	1.84	3.90E-11	0.28	128	21.74	427	0.21	154	21.85	228	0.39	129	30.82	203
MAPK9	1.25	1.13E-09	0.28	128	21.74	428	0.21	147	19.91	408	0.33	143	30.55	221
WDR76	1.3	5.23E-16	0.24	141	21.72	429	0.2	122	14.1	1505	0.4	121	28.41	344
ZNF468_t1	1.06	4.50E-05	0.27	131	21.72	430	NA	NA	NA	NA	0.21	148	20.36	1221
SPRYD7	1.23	1.50E-06	0.21	152	21.69	431	0.1	127	7.48	4002	0.18	132	14.41	2484
FBXW8	1.13	6.16E-15	0.22	147	21.68	432	0.22	151	21.77	233	0.17	133	13.99	2598
FAM203A	1.47	1.27E-17	0.28	128	21.66	433	0.2	145	19.07	509	0.3	124	21.96	971
HNRNPA3_t1	1.44	6.06E-10	0.25	137	21.64	434	NA	NA	NA	NA	NA	NA	NA	NA
MUC15	1.42	9.51E-08	0.27	131	21.63	435	0.02	125	1.21	7676	0.31	143	28.85	314
KIAA0101	3.2	1.03E-27	0.25	136	21.63	436	0.22	128	17.44	758	0.41	137	36.05	68
CAP2	2.72	6.33E-25	0.33	116	21.63	437	0.23	120	16.01	1043	0.42	126	32.23	152
AURKA	2.17	5.53E-26	0.3	123	21.61	438	0.14	130	11.04	2457	0.38	116	25.33	582
STC2	1.48	1.21E-13	0.28	126	21.61	439	0.24	140	21.84	230	0.35	150	34.58	88
UTP11L	1.28	1.04E-09	0.29	125	21.6	440	0.14	106	7.84	3816	0.33	128	25.44	567
PTPRF	1.8	3.76E-17	0.31	119	21.59	441	0.25	130	20.02	399	0.34	127	26.35	469
FAM91A1	1.22	3.55E-08	0.28	127	21.59	442	0.14	142	13.23	1760	0.35	131	28.59	326
B4GALT3	1.41	2.33E-19	0.27	130	21.59	443	0.19	105	10.69	2597	0.25	113	15.86	2109
SEC23B	1.33	3.64E-14	0.25	135	21.57	444	0.09	133	7.5	3988	0.28	161	31.22	194
ILF2	1.65	3.66E-22	0.34	114	21.57	445	0.29	120	20.36	360	0.33	121	23.11	834
SNRPG	1.37	2.31E-22	0.3	123	21.54	446	0.27	114	17.34	769	0.33	106	18.57	1539
SPRTN_t1	1.19	1.34E-12	0.24	139	21.54	447	0.15	156	15.93	1064	0.35	131	28.2	357
PTBP2	1.22	0.000526129	0.26	134	21.53	448	0.21	143	19.58	449	0.23	143	21.63	1028
IL12A	1.04	0.011329225	0.26	134	21.53	449	0.03	101	1.61	7421	0.17	106	9.35	4102
KIAA1328	1.06	0.001244148	0.21	154	21.52	450	0.15	127	11.24	2388	0.08	124	6.05	5425
TAF9_t1	1.58	2.52E-18	0.25	136	21.51	451	NA	NA	NA	NA	0.37	139	32.92	126
ARL4A	1.26	1.80E-05	0.22	146	21.51	452	0.22	120	15.16	1232	0.15	123	11.04	3501
PHF20L1_t1	1.13	7.89E-06	0.27	130	21.5	453	0.17	121	12.08	2108	0.35	136	29.91	247
ARMC8_t1	1.17	2.71E-08	0.28	127	21.49	454	0.16	136	14.06	1517	0.27	146	26.14	486
POP4	1.39	1.66E-21	0.26	134	21.48	455	0.29	140	25.99	62	0.26	117	17.45	1744
ACACA_t1	1.19	2.07E-13	0.28	126	21.47	456	0.17	139	15.56	1131	NA	NA	NA	NA
COG2	1.49	6.10E-24	0.31	120	21.47	457	0.25	122	18	663	0.35	122	25.22	593
NCAPG	4.74	2.84E-33	0.29	123	21.47	458	0.25	123	18.09	647	0.42	138	37.2	55
SOCS5	1.18	2.93E-13	0.22	147	21.47	459	0.27	118	18.61	571	0.37	152	37.95	37
HPS3	1.24	7.24E-08	0.28	127	21.46	460	0.18	116	11.81	2186	0.22	120	15.19	2277
NME1	1.74	1.10E-24	0.3	121	21.46	461	0.29	104	15.85	1078	0.38	138	33.57	106
METTL6	1.52	2.60E-26	0.33	115	21.45	462	0.25	139	22.53	182	0.41	153	41.93	9
AGPAT3_t1	1.08	0.035850219	0.18	166	21.44	463	NA	NA	NA	NA	0.13	159	14.19	2546
GTF2I_t1	1.58	4.98E-14	0.22	148	21.44	464	0.23	123	17.12	817	0.32	119	22.35	916
ZNF318	1.38	3.31E-16	0.25	136	21.44	465	0.22	119	15.06	1260	0.41	136	35.08	82
ARFGEF2	1.3	3.72E-19	0.25	134	21.41	466	0.24	146	23.01	151	0.38	127	29.36	275
CCDC99	1.52	1.36E-22	0.24	141	21.4	467	0.21	98	10.03	2851	0.41	119	28.1	365
GSTCD	1.16	5.18E-10	0.26	131	21.39	468	0.24	138	20.98	295	0.31	156	33	124
DDX46	1.29	5.19E-08	0.26	132	21.38	469	0.17	152	16.98	847	0.43	152	43.41	6
WDR5B	1.25	1.12E-17	0.25	137	21.38	470	0.17	132	14.27	1459	0.24	142	22.49	898
GINS4	1.26	4.96E-15	0.23	142	21.38	471	0.17	135	14.52	1399	0.25	153	25.4	574
TMCC1	1.6	1.94E-11	0.24	140	21.33	472	0.18	130	14.51	1402	0.31	134	25.69	539
ACTB	1.59	7.37E-16	0.28	125	21.31	473	0.41	109	24.21	106	0.27	128	21.18	1089
NCAPG2	1.64	1.43E-21	0.3	122	21.3	474	0.21	108	11.92	2147	0.41	103	21.51	1046
STK32B	1.13	4.44E-13	0.25	136	21.3	475	0.2	136	17.27	786	0.25	127	19.41	1362
SMAD2_t1	1.1	0.000282695	0.21	152	21.27	476	NA	NA	NA	NA	NA	NA	NA	NA
TAF5	1.17	2.16E-11	0.28	127	21.25	477	0.17	121	11.94	2144	0.28	134	23.39	801
CD46_t1	1.3	1.50E-07	0.28	125	21.24	478	0.15	118	9.98	2867	0.22	132	18.42	1564
TMEM237	1.59	4.88E-16	0.23	142	21.22	479	0.3	123	21.76	234	0.26	127	20.21	1245
ZNF2_t1	1.04	0.000111534	0.23	142	21.21	480	NA	NA	NA	NA	NA	NA	NA	NA
LIN9	1.74	2.29E-19	0.26	133	21.2	481	0.23	119	15.53	1134	0.36	129	28.54	329
NDUFAF6	1.42	9.10E-10	0.28	126	21.19	482	0.16	123	11.66	2252	0.34	136	29.56	263
CYB5R1	1.62	1.98E-18	0.29	122	21.18	483	0.23	123	16.98	848	0.36	100	17.9	1661
UBE2Q1	1.62	1.87E-22	0.22	147	21.18	484	0.2	120	13.74	1615	0.34	120	23.81	747
ZKSCAN3	1.32	2.03E-19	0.25	134	21.17	485	0.19	132	15.5	1148	0.27	129	21.15	1093
TOP2A	3.05	5.91E-28	0.28	126	21.16	486	0.22	130	17.42	760	0.39	132	31.61	181
GPATCH2	1.28	4.89E-09	0.29	124	21.16	487	0.18	130	14.32	1451	0.26	120	18.52	1547
LPL	2.24	8.17E-14	0.3	120	21.16	488	0.18	122	12.93	1856	0.35	136	30.19	231
FAM175A_t1	1.2	1.29E-05	0.23	143	21.15	489	NA	NA	NA	NA	0.29	135	24.24	694
PARPBP_t1	2.03	6.74E-21	0.25	135	21.14	490	NA	NA	NA	NA	0.4	123	29.19	284
WDR12	1.55	9.30E-21	0.3	120	21.14	491	0.3	122	21.73	236	0.35	126	26.6	444
EGLN1	1.48	2.00E-13	0.27	129	21.14	492	0.13	121	9.49	3062	0.32	142	29.02	299
BPNT1	1.25	8.71E-10	0.22	147	21.14	493	0.12	133	10.08	2831	0.28	121	19.55	1339
MCM4	1.8	3.32E-19	0.28	126	21.13	494	0.21	131	17.05	829	0.33	131	26.9	425
ZZZ3	1.22	1.25E-06	0.28	125	21.12	495	0.18	127	13.73	1618	0.35	128	27.24	410
CSTF2	1.59	4.21E-26	0.26	130	21.12	496	0.19	135	15.87	1074	0.36	102	18.53	1545
FAM40B_t1	1.17	4.45E-13	0.23	142	21.12	497	NA	NA	NA	NA	NA	NA	NA	NA
SLMO2	1.35	9.55E-14	0.23	142	21.11	498	0.15	136	12.87	1873	0.33	147	32.44	144
SLBP	1.29	6.18E-09	0.22	145	21.1	499	0.17	130	13.32	1740	0.31	134	26.09	494
INTS2	1.27	3.76E-12	0.23	141	21.1	500	0.22	143	20.06	396	0.32	127	24.87	626
RBL1_t1	1.18	2.04E-15	0.23	140	21.09	501	0.22	148	21.47	249	0.27	132	22.19	936
STAM2_t1	1.11	0.002743277	0.21	151	21.08	502	0.18	139	16.26	990	NA	NA	NA	NA
SCFD2	1.21	7.03E-14	0.26	132	21.08	503	0.14	133	11.88	2159	0.25	120	17.44	1747
NOM1	1.12	6.24E-06	0.25	133	21.08	504	0.2	135	17.08	823	0.2	153	20.19	1250
RPS20	1.84	1.62E-19	0.33	114	21.08	505	0.34	135	28.72	17	0.37	120	25.96	510
LRR1_t1	1.27	5.54E-13	0.25	134	21.07	506	0.13	102	6.51	4502	0.29	116	19.44	1358
ARPP19	1.23	1.48E-05	0.22	145	21.07	507	0.19	134	16.19	999	0.27	143	25.07	613
PAFAH1B2	1.12	7.09E-06	0.21	150	21.06	508	0.19	119	13.43	1704	0.15	146	14.68	2412
TRMT6	1.48	2.65E-17	0.28	124	21.06	509	0.25	121	18.04	656	0.37	142	33.62	105
KDM5B	1.34	1.34E-09	0.26	132	21.06	510	0.21	123	15.04	1263	0.2	148	19.26	1396
FNBP1L_t2	1.24	7.82E-13	0.23	140	21.04	511	0.19	133	15.69	1110	0.28	134	23.14	829
ASCC3_t1	1.47	1.25E-07	0.2	154	20.99	512	0.23	122	16.32	976	0.27	123	19.4	1365
CCT6A_t1	1.64	4.80E-15	0.24	139	20.98	513	NA	NA	NA	NA	0.33	167	38.14	34
ACTR8	1.2	8.26E-12	0.27	127	20.97	514	0.18	109	10.47	2694	0.25	120	17.85	1673
SRPRB	1.37	2.15E-15	0.2	154	20.97	515	0.27	150	26.57	45	0.21	148	20.72	1170
FZD6	1.37	7.99E-09	0.26	132	20.96	516	0.17	140	14.99	1282	0.3	146	28.48	335
SPON2	2.45	6.82E-13	0.28	124	20.95	517	0.23	113	14.56	1389	0.29	120	20.45	1205
AP3M2	1.35	1.33E-10	0.23	141	20.95	518	0.28	154	28.94	13	0.29	136	24.87	624
TP53RK_t1	1.24	5.46E-12	0.25	135	20.93	519	NA	NA	NA	NA	0.2	126	15.19	2276
CDCA4	1.13	7.94E-12	0.25	134	20.91	520	0.13	124	9.49	3068	0.22	145	20.72	1169
GM2A_t1	1.24	1.27E-10	0.3	120	20.87	521	0.2	130	15.86	1077	NA	NA	NA	NA
SURF6	1.18	3.96E-08	0.3	120	20.84	522	0.24	115	15.36	1180	0.24	115	15.71	2151
SRGAP2	1.4	1.30E-09	0.3	120	20.82	523	0.21	145	20.39	353	0.26	134	21.63	1026
NFYC	1.21	2.25E-11	0.26	130	20.82	524	0.07	152	7.55	3965	0.26	131	20.88	1138
MED8_t1	1.26	9.04E-09	0.26	131	20.81	525	0.1	126	7.96	3767	0.18	145	16.9	1855
DBR1	1.07	0.000182343	0.21	149	20.79	526	0.09	113	5.77	4914	0.13	134	10.87	3560

MNS1	1.73	1.54E-08	0.21	151	20.77	527	0.09	113	5.72	4937	0.31	141	28.19	358
ITGA6	2.27	5.71E-21	0.29	122	20.76	528	0.29	123	21.11	277	0.36	142	32.87	127
ARMC1	1.42	5.66E-11	0.27	128	20.75	529	0.18	123	13.39	1717	0.42	139	37.37	49
RAD51AP1	1.87	1.98E-20	0.26	130	20.75	530	0.22	129	17.45	756	0.36	111	21.87	991
CTNNA1	1.53	4.39E-19	0.33	113	20.72	531	0.3	121	20.97	296	0.34	112	20.82	1152
CCNE2	1.27	2.09E-13	0.24	135	20.72	532	0.16	138	14.25	1465	0.33	123	24.28	690
DNAJB6_t1	1.34	8.09E-13	0.27	127	20.71	533	0.26	128	20.36	359	0.27	123	19.83	1298
CNIH4	2.01	5.50E-27	0.37	106	20.71	534	0.3	110	17.92	679	0.39	104	21.1	1101
ZSWIM1	1.18	7.38E-12	0.28	123	20.71	535	0.23	154	23.82	119	0.25	147	24.39	680
UBR5	1.21	1.73E-07	0.26	129	20.71	536	0.21	128	16.29	982	0.35	134	29.11	290
NETO2_t1	1.38	7.06E-11	0.23	139	20.71	537	NA	NA	NA	NA	0.28	137	24.67	656
L3MBTL1	1.4	6.70E-12	0.23	140	20.69	538	0.23	120	16.37	966	0.23	123	16.65	1914
PSMD11	1.11	0.000949054	0.23	138	20.67	539	0.15	120	10.78	2561	0.18	135	15.58	2185
H2AFY	1.39	1.85E-16	0.26	131	20.67	540	0.25	120	17.55	741	0.28	128	22.21	934
ZBTB39	1.1	7.69E-09	0.22	142	20.67	541	0.14	136	12.11	2099	0.14	143	13.38	2769
ZNF544	1.54	1.78E-13	0.25	133	20.66	542	0.3	138	26.79	41	0.27	120	19.05	1440
LSM11_t1	1.28	1.14E-12	0.23	140	20.66	543	NA	NA	NA	NA	0.27	124	20.21	1244
ZIC4_t1	1.08	1.27E-08	0.28	124	20.65	544	NA	NA	NA	NA	0.32	137	27.75	388
CHAF1A	1.8	9.34E-29	0.23	141	20.65	545	0.16	100	8.18	3651	0.3	111	18.45	1562
TIGD6	1.34	2.00E-16	0.25	131	20.65	546	0.11	146	10.53	2663	0.34	128	26.57	448
DHX9	1.29	1.21E-06	0.27	127	20.65	547	0.22	109	13.06	1812	0.28	111	16.8	1872
TPD52L2	1.12	5.00E-05	0.23	138	20.63	548	0.13	147	12.42	2000	0.09	126	7.02	5030
BZW2	1.71	1.93E-20	0.28	124	20.62	549	0.26	127	20.37	358	0.33	101	16.64	1921
FANCB	1.13	4.77E-14	0.23	140	20.61	550	0.13	98	6.12	4715	0.25	116	16.67	1908
ABCF2_t1	1.2	2.63E-12	0.25	133	20.61	551	0.19	152	19.11	498	0.29	133	24.03	719
CKLF_t1	1.36	2.85E-16	0.31	116	20.61	552	0.12	103	6.17	4688	0.19	112	11.93	3226
ANKRD45	1.19	1.31E-13	0.27	127	20.6	553	0.23	123	16.83	870	0.31	122	22.36	913
THAP2	1.22	4.48E-10	0.22	145	20.6	554	0.22	128	16.97	851	0.33	150	33.09	119
DCAF13	1.88	1.26E-17	0.29	122	20.59	555	0.29	121	20.62	328	0.39	123	28.57	327
FAM200A	1.32	3.94E-15	0.24	137	20.59	556	0.2	116	13.25	1754	0.36	126	27.36	402
LARS	1.42	2.58E-13	0.29	122	20.59	557	0.22	135	19.12	497	0.39	153	40.43	15
ALG1_t1	1.51	6.25E-21	0.23	140	20.58	558	NA	NA	NA	NA	0.31	136	27.03	419
PPP2R5A_t1	1.44	3.80E-12	0.25	132	20.55	559	NA	NA	NA	NA	NA	NA	NA	NA
GINS2	1.97	1.47E-17	0.27	125	20.54	560	0.12	129	9.75	2959	0.33	101	16.68	1907
RBM12B	1.43	1.30E-17	0.26	129	20.53	561	0.21	123	15.28	1204	0.35	124	25.76	538
DKC1	1.34	4.02E-11	0.24	134	20.53	562	0.25	115	16.18	1002	0.24	142	22.49	899
UBE2O	1.53	3.43E-25	0.27	127	20.51	563	0.3	130	23.93	117	0.26	134	22.14	946
ZNF449	1.24	4.76E-08	0.21	147	20.51	564	0.21	125	16.08	1024	0.32	153	32.48	143
CKAP2L	1.73	3.36E-22	0.29	120	20.51	565	0.22	124	16.11	1017	0.41	133	34.2	96
TRNAU1AP	1.39	1.45E-17	0.21	149	20.51	566	0.19	117	12.82	1891	0.22	121	15.35	2231
RALBP1	1.35	4.98E-14	0.23	138	20.5	567	0.2	133	16.65	907	0.34	155	36.19	64
MCM3	2.22	1.21E-28	0.32	114	20.48	568	0.29	106	16.45	951	0.34	116	22.25	929
NCKAP1	1.16	0.004230771	0.22	142	20.48	569	0.18	146	17.03	831	0.27	148	26.54	452
ZNF642	1.13	5.87E-06	0.27	125	20.48	570	0.19	99	9.13	3213	0.22	114	13.78	2655
PARS2	1.51	1.43E-21	0.27	126	20.47	571	0.17	134	14.65	1365	0.27	127	20.8	1159
SAP30L	1.5	5.04E-23	0.25	133	20.46	572	0.12	130	9.5	3060	0.31	154	32.74	130
SUZ12	1.39	2.74E-16	0.25	131	20.46	573	0.24	128	18.38	605	0.31	146	30.01	240
NOL9	1.22	6.84E-09	0.22	144	20.46	574	0.15	135	13	1833	0.18	142	16.99	1829
CDC23	1.34	1.75E-13	0.28	122	20.45	575	0.15	142	13.54	1675	0.37	131	30.02	238
PTGES3	1.27	1.57E-13	0.23	139	20.44	576	0.15	121	10.35	2739	0.29	147	27.91	376
RASAL2	1.2	5.53E-13	0.28	124	20.43	577	0.33	139	29.14	12	0.29	140	25.77	536
MDM4_t1	1.09	0.012836855	0.2	152	20.42	578	0.19	140	16.86	866	NA	NA	NA	NA
PRIM2_t1	1.64	1.90E-23	0.29	120	20.42	579	NA	NA	NA	NA	0.4	148	39.24	21
ITGB3BP	1.79	1.08E-24	0.32	114	20.41	580	0.25	128	19.82	421	0.42	120	29.51	265
ATP6V1E1	1.26	1.80E-08	0.25	132	20.41	581	0.18	120	12.85	1877	0.21	120	14.53	2453
BTN2A1	1.32	4.10E-12	0.27	125	20.4	582	0.25	126	18.88	536	0.29	120	20.33	1223
ALG6	1.41	1.18E-14	0.26	129	20.4	583	0.17	112	10.56	2649	0.4	120	28.16	360
SLC2A1	1.53	2.02E-06	0.23	138	20.4	584	0.25	123	17.91	683	0.13	143	12.22	3131
PALLD	1.16	0.161534116	0.2	152	20.39	585	0.12	119	8.21	3638	0.09	138	8.27	4506
QKI_t1	1.18	8.85E-09	0.22	141	20.39	586	0.24	136	20.28	370	0.27	147	26.33	471
ABT1	1.28	9.20E-13	0.25	131	20.38	587	0.19	131	15.39	1171	0.27	127	20.65	1179
EIF4E	1.09	0.015707082	0.22	141	20.38	588	0.16	160	18.09	648	0.22	137	18.92	1468
GPD2	1.23	2.47E-09	0.22	141	20.37	589	0.18	134	15.08	1255	0.2	124	15.07	2310
NUP43_t1	1.15	1.59E-14	0.25	131	20.37	590	NA	NA	NA	NA	NA	NA	NA	NA
COPG1	1.23	6.20E-13	0.21	148	20.36	591	0.12	122	8.4	3543	0.08	114	4.99	5930
FAM83H	1.79	6.11E-20	0.25	133	20.35	592	0.14	121	9.89	2900	0.29	130	23.32	810
ASPH_t3	1.23	7.29E-05	0.25	131	20.34	593	0.13	124	9.79	2940	0.25	122	17.85	1671
TTC30A	1.2	6.36E-10	0.19	158	20.33	594	0.17	119	11.78	2198	0.2	142	18.11	1624
HHAT	1.55	2.60E-13	0.23	140	20.31	595	0.14	126	10.57	2644	0.28	127	21.57	1037
ZDHHC20	1.12	5.96E-06	0.21	149	20.31	596	0.15	143	13.58	1663	0.11	130	8.5	4424
SUB1_t1	1.41	2.05E-09	0.23	140	20.31	597	NA	NA	NA	NA	NA	NA	NA	NA
TIGD5	1.81	8.23E-25	0.29	120	20.31	598	0.22	113	13.69	1627	0.31	127	23.49	789
CDH13	2.16	1.78E-22	0.29	120	20.31	599	0.26	128	20.01	400	0.42	143	38.98	25
ZNF473	1.22	7.23E-18	0.29	121	20.3	600	0.18	138	15.84	1081	0.31	143	28.62	325
TK1	3.61	1.84E-30	0.3	118	20.28	601	0.22	153	22.74	168	0.38	119	26.48	457
CENPW	2.91	1.64E-34	0.34	109	20.25	602	0.33	116	21.75	235	0.42	104	22.54	892
HIST1H3J	1.43	7.53E-16	0.27	124	20.24	603	0.23	85	7.96	3766	0.22	93	9.35	4100
NDUFB9	1.6	2.00E-13	0.28	121	20.23	604	0.2	99	9.9	2897	0.38	120	26.56	451
EED	1.37	6.47E-12	0.28	121	20.23	605	0.26	130	20.87	302	0.3	130	24.08	713
CDC42BPA_t1	1.17	0.000489416	0.22	141	20.21	606	NA	NA	NA	NA	0.26	132	21.62	1030
SPC25	3.18	1.22E-29	0.31	115	20.2	607	0.24	118	16.18	1001	0.4	119	27.66	390
SCFD1	1.26	0.000360914	0.25	132	20.2	608	0.14	108	8.14	3671	0.28	104	15.04	2326
TDP1	1.32	8.31E-13	0.24	134	20.2	609	0.27	133	22.09	207	0.24	131	19.32	1381
LAGE3	1.95	6.27E-28	0.26	129	20.2	610	0.19	130	15.02	1273	0.42	160	45.81	3
SLC4A1AP	1.29	5.33E-12	0.25	130	20.2	611	0.16	92	6.8	4348	0.37	104	19.76	1313
TIPRL_t1	1.45	9.25E-20	0.24	133	20.19	612	0.33	125	24.41	101	0.35	125	26.04	499
SH3TC2	1.05	3.60E-05	0.19	157	20.18	613	0.05	144	5.05	5306	0.14	148	13.83	2640
UBE2D1	1.2	3.92E-07	0.2	153	20.17	614	0.23	126	17.23	792	0.26	151	25.9	517
SPOPL	1.13	0.001531832	0.21	145	20.16	615	0.1	128	8.07	3708	0.25	152	25.48	565
DSN1	1.57	4.51E-16	0.2	149	20.16	616	0.16	131	13.02	1822	0.3	144	28.42	343
SIX1	1.77	2.15E-16	0.26	128	20.15	617	0.26	119	18.19	625	0.14	142	12.48	3042
KIF3B	1.22	1.38E-17	0.21	146	20.15	618	0.15	136	12.82	1893	0.29	130	23.46	792
DIP2B	1.16	4.62E-08	0.23	139	20.14	619	0.06	135	5.01	5329	0.13	131	10.21	3792
CNTNAP4_t1	1.12	4.56E-05	0.21	145	20.14	620	-0.02	115	-1.19	9285	0.17	122	12.28	3115
WASF1	1.29	2.92E-14	0.21	145	20.14	621	0.28	123	20.41	351	0.19	117	12.65	2993
PDXK	1.32	1.54E-16	0.28	121	20.13	622	0.25	141	23.04	148	0.28	121	19.85	1294
NSD1	1.16	1.33E-10	0.27	124	20.13	623	0.15	142	13.79	1602	0.3	131	23.91	733
BCOR	1.23	4.02E-13	0.24	133	20.1	624	0.2	113	12.63	1948	0.19	116	12.63	3000
PPP4R1	1.18	3.61E-08	0.28	121	20.06	625	0.12	118	8.16	3665	0.29	110	17.34	1760
DARS2	1.78	6.44E-22	0.29	120	20.06	626	0.21	124	15.29	1195	0.45	112	27.62	393
HJURP	1.58	5.78E-26	0.26	126	20.06	627	0.18	125	13.44	1700	0.34	119	23.64	771
FAM98A	1.23	1.51E-07	0.19	153	20.04	628	0.14	117	9.33	3134	0.26	128	20.12	1262
PLXDC1_t1	1.49	3.82E-08	0.21	144	20.02	629	NA	NA	NA	NA	0.19	135	15.94	2086
FHDC1	1.12	0.000125702	0.2	149	20.01	630	0.18	136	15.51	1144	0.21	122	15.35	2233
TMEM241	1.22	1.65E-12	0.23	136	20.01	631	0.14	135	11.53	2295	0.26	123	19.31	1383
PCDH17	1.15	1.59E-05	0.24	133	20	632	0.2	137	17.45	754	0.19	130	15.19	2279

ZWILCH	1.36	3.29E-13	0.25	130	20	633	0.21	123	15.05	1262	0.37	119	25.36	577
TPD52	1.52	5.90E-09	0.23	138	19.99	634	0.19	131	15.3	1194	0.27	128	21.06	1108
INTU	1.22	6.16E-06	0.2	150	19.98	635	0.23	105	12.9	1866	0.2	114	12.7	2974
NENF	1.24	7.03E-08	0.24	135	19.98	636	0.06	112	3.67	6076	0.11	113	6.81	5101
DHX57	1.36	3.87E-23	0.29	120	19.96	637	0.25	120	17.8	699	0.39	114	24.8	633
TOMM20_t1	1.25	2.50E-10	0.21	147	19.95	638	0.15	122	10.82	2547	NA	NA	NA	NA
TBC1D24	1.24	5.91E-13	0.22	140	19.95	639	0.15	117	10.08	2836	0.26	139	22.8	863
NME6	1.31	4.99E-21	0.23	136	19.94	640	0.24	135	20.79	316	0.26	153	26.33	472
NEDD4L_t1	1.54	2.74E-10	0.22	140	19.91	641	NA	NA	NA	NA	0.29	142	26.48	458
DGCR14	1.19	3.33E-11	0.19	156	19.9	642	0.12	140	10.38	2728	0.15	143	14.4	2488
TXNDC9	1.28	8.03E-09	0.25	131	19.9	643	0.18	119	12.24	2050	0.22	124	16.46	1966
PSME3_t2	1.32	3.37E-12	0.24	134	19.89	644	0.19	122	13.91	1555	0.28	134	23.74	759
MTBP	1.36	3.87E-15	0.29	119	19.89	645	0.23	112	13.97	1541	0.38	102	19.63	1326
BRF2	1.3	3.59E-11	0.28	122	19.88	646	0.12	141	11.36	2340	0.22	110	12.96	2900
EFTUD2	1.31	4.07E-15	0.2	148	19.87	647	0.29	134	24.64	91	0.19	113	11.99	3203
B4GALNT1	1.28	2.95E-12	0.28	120	19.87	648	0.12	128	9.13	3214	0.35	120	24.37	684
EID2B	1.33	6.03E-16	0.24	132	19.87	649	0.21	145	19.64	440	0.3	135	25.54	556
VHL	1.17	8.38E-06	0.21	144	19.86	650	0.16	148	15.89	1071	0.22	152	22.07	958
CHD7	1.39	2.70E-11	0.23	136	19.85	651	0.26	142	23.55	128	0.36	123	25.96	511
TOMM40L	2.19	1.03E-28	0.29	118	19.84	652	0.3	142	27.69	31	0.35	115	22.71	876
RAD51	2.19	3.49E-22	0.3	117	19.83	653	0.26	124	19.59	447	0.39	150	38.9	26
GTF3C2	1.23	5.84E-13	0.22	141	19.82	654	0.24	153	25.08	77	0.18	128	14.21	2544
MOSPD1	1.33	7.81E-08	0.23	137	19.81	655	0.11	127	8.15	3667	0.33	133	27.36	401
S100PBP_t1	1.14	1.51E-05	0.21	143	19.78	656	NA	NA	NA	NA	0.11	131	9.24	4142
XRCC2	1.08	1.55E-07	0.21	144	19.78	657	0.17	139	15.26	1211	0.11	150	10.74	3594
SLC7A11_t1	1.42	1.23E-08	0.19	153	19.78	658	NA	NA	NA	NA	0.28	152	28.47	337
CDC25A	1.56	4.44E-20	0.28	120	19.76	659	0.25	149	24.56	95	0.33	120	23.17	822
AGAP3_t1	1.27	3.95E-11	0.25	130	19.76	660	0.2	119	13.59	1657	0.22	127	16.62	1924.5
BCAT1	1.48	4.36E-06	0.19	155	19.73	661	0.22	133	17.93	678	0.18	142	16.22	2029
RAB3GAP1	1.12	0.001503464	0.26	127	19.72	662	0.2	150	19.98	402	0.19	129	15.36	2230
RFWD2_t1	1.31	1.12E-11	0.22	140	19.7	663	NA	NA	NA	NA	0.31	135	26.43	464
AP3B1	1.36	5.56E-12	0.24	131	19.7	664	0.18	128	14.38	1431	0.39	118	26.52	454
TMC7	1.24	2.14E-10	0.2	147	19.69	665	0.23	147	21.89	224	0.21	152	21.87	993
WDR67	1.8	1.06E-21	0.3	116	19.67	666	0.25	107	14.2	1479	0.43	111	26.49	455
LRRC42_t1	1.55	6.67E-16	0.31	114	19.67	667	NA	NA	NA	NA	0.29	110	17.1	1808
MPPE1	1.18	3.30E-12	0.21	142	19.67	668	0.07	133	5.55	5042	0.27	129	21.25	1077
RTCA	1.38	1.91E-08	0.26	125	19.66	669	0.3	115	19.49	454	0.29	162	32.58	137
PLK4	1.34	5.09E-15	0.28	121	19.66	670	0.21	111	12.85	1876	0.34	121	24.39	679
CACNB4	1.15	1.85E-05	0.19	156	19.66	671	0.15	146	14.08	1514	0.18	147	17.22	1781
DNM1L_t1	1.41	1.53E-11	0.23	136	19.65	672	0.29	135	24.78	89	0.33	153	33.82	102
FNIP1	1.08	7.96E-09	0.18	159	19.63	673	0.12	133	9.81	2932	0.23	139	20.77	1165
CD109_t1	1.74	5.07E-16	0.23	135	19.62	674	0.19	135	16.08	1026	NA	NA	NA	NA
ITGA2_t1	1.34	1.08E-09	0.21	142	19.59	675	NA	NA	NA	NA	NA	NA	NA	NA
CDK4	1.46	2.12E-14	0.24	131	19.58	676	0.21	139	18.64	568	0.25	153	25.25	589
DDB1	1.42	1.63E-30	0.3	115	19.58	677	0.29	132	24.06	109	0.32	115	20.5	1200
MAEA	1.24	6.79E-08	0.22	141	19.58	678	0.16	134	13.47	1695	0.17	136	14.85	2374
PARP12	1.37	4.58E-14	0.24	131	19.57	679	0.09	116	6.01	4775	0.22	122	15.97	2079
CDK16	1.29	3.45E-13	0.31	113	19.56	680	0.21	128	16.46	949	0.26	107	14.65	2420
TRIM32_t1	1.25	1.03E-15	0.24	130	19.56	681	NA	NA	NA	NA	0.28	133	22.85	858
GOLT1B	1.24	2.42E-15	0.24	133	19.55	682	0.25	130	20.28	368	0.32	154	33.03	123
DIAPH3_t1	1.27	1.58E-18	0.26	124	19.54	683	NA	NA	NA	NA	0.34	147	32.51	142
ACER3	1.13	2.35E-06	0.21	145	19.53	684	0.24	140	22.02	218	0.17	138	15.2	2272
CCDC23	1.27	6.13E-07	0.25	129	19.53	685	0.28	110	17.03	834	0.15	95	6.62	5188
ANXA2	2.08	5.66E-14	0.23	134	19.51	686	0.3	125	22.78	164	0.23	129	18.55	1541
FGFBP3	1.17	7.54E-14	0.2	147	19.51	687	0.19	128	14.6	1375	0.16	141	14.33	2514
PHF21A	1.15	2.09E-05	0.2	148	19.51	688	0.2	132	16.16	1007	0.16	132	13.27	2804
FKTN	1.12	6.42E-09	0.19	151	19.51	689	0.17	142	15.91	1066	0.25	152	25.05	616
CANX	1.65	3.42E-15	0.25	128	19.51	690	0.22	141	19.71	431	0.4	142	36.46	58
ATAD5_t1	1.26	3.79E-14	0.24	132	19.5	691	NA	NA	NA	NA	0.34	110	20.66	1176
MIB1	1.11	4.31E-06	0.18	156	19.48	692	0.16	138	14.33	1444	0.3	154	31.42	187
ZFR	1.26	7.23E-08	0.21	142	19.48	693	0.18	119	12.44	1995	0.35	123	25.8	528
YTHDF3	1.24	4.87E-08	0.2	149	19.48	694	0.16	130	12.91	1862	0.34	134	28.71	320
RB1CC1	1.28	0.000262937	0.23	136	19.48	695	0.13	128	10.08	2832	0.27	147	26.49	456
OTUB2_t1	1.14	9.40E-14	0.24	132	19.47	696	NA	NA	NA	NA	NA	NA	NA	NA
ENOPH1	1.41	3.03E-12	0.28	120	19.47	697	0.23	118	15.59	1121	0.46	120	31.91	165
FAF1	1.34	1.09E-18	0.28	120	19.47	698	0.15	154	15.21	1223	0.32	110	19.17	1415
CYSLTR2	1.13	1.21E-16	0.27	123	19.46	699	0.13	151	12.83	1887	0.24	142	21.95	973
UBFD1_t1	1.3	4.19E-12	0.19	151	19.45	700	NA	NA	NA	NA	NA	NA	NA	NA
HEY1	1.79	8.82E-17	0.27	122	19.44	701	0.22	112	13.86	1576	0.36	137	31.31	188
NUSAP1	1.71	2.68E-22	0.24	132	19.44	702	0.21	133	17.34	770	0.34	123	24.83	631
EPG5_t1	1.06	7.82E-05	0.23	133	19.43	703	NA	NA	NA	NA	0.28	152	28.74	318
SOX13	1.35	1.34E-16	0.21	144	19.39	704	0.13	168	15.74	1104	0.29	129	23.25	814
CPNE3_t1	1.15	0.002356692	0.19	154	19.39	705	NA	NA	NA	NA	0.21	142	19.6	1330
GPATCH4	1.85	2.24E-20	0.31	113	19.39	706	0.21	122	14.98	1285	0.39	129	30.61	214
RBBP4_t1	1.06	7.82E-05	0.19	150	19.38	707	NA	NA	NA	NA	NA	NA	NA	NA
SMYD2	1.66	5.56E-12	0.26	125	19.37	708	0.23	120	16.27	986	0.27	140	24.47	669
IRF2BP2_t1	1.25	4.73E-06	0.24	132	19.37	709	NA	NA	NA	NA	0.23	133	19.1	1435
DHRS13	1.81	5.90E-17	0.28	120	19.36	710	0.2	113	12.75	1913	0.28	126	21.24	1079
HMGN4	1.47	2.07E-14	0.25	127	19.35	711	0.28	128	22.12	204	0.31	120	21.52	1045
PGP	1.08	7.49E-09	0.17	165	19.35	712	0.2	135	16.65	908	0.19	142	17.33	1763
RHBDD1	1.17	1.53E-10	0.24	129	19.33	713	0.11	147	10.62	2622	0.17	152	16.97	1837
DNMT3B	1.68	1.20E-19	0.27	122	19.32	714	0.23	136	20.17	386	0.37	120	25.77	534
ANAPC7_t1	1.19	8.10E-13	0.26	123	19.32	715	NA	NA	NA	NA	0.26	124	18.9	1474
MFF	1.28	1.12E-10	0.25	127	19.31	716	0.23	142	21.46	250	0.27	167	31.76	171
RDM1	1.82	1.75E-23	0.26	123	19.31	717	0.18	89	6.85	4325	0.38	79	10.96	3522
CETN2	1.35	1.75E-09	0.24	131	19.31	718	0.14	114	9.14	3212	0.28	130	22.35	917
KIAA1279	1.12	0.004159902	0.19	153	19.31	719	0.04	109	2.63	6737	0.18	116	12	3196
MSH2	1.81	1.52E-20	0.32	110	19.3	720	0.26	95	11.59	2276	0.39	115	25.47	566
PRELID2	1.4	6.28E-11	0.22	139	19.3	721	0.16	124	11.59	2274	0.26	127	20.39	1216
CCDC132_t1	1.22	1.50E-06	0.26	125	19.28	722	0.21	116	13.77	1608	0.32	137	28.09	366
DYNLT3	1.34	1.36E-13	0.26	123	19.28	723	0.15	114	9.62	3012	0.38	125	28.15	361
ERI1	1.11	7.63E-05	0.24	130	19.28	724	0.1	131	7.83	3820	0.24	134	20.32	1226
CCDC88A	1.17	6.43E-10	0.23	135	19.27	725	0.25	127	18.9	532	0.28	153	28.98	301
HNRPLL	1.16	2.25E-06	0.23	134	19.27	726	0.14	140	12.98	1839	0.23	136	19.88	1289
MSX1	1.2	6.81E-13	0.2	144	19.27	727	0.17	132	13.59	1660	0.27	119	18.38	1572
CACUL1	1.1	0.001075503	0.22	136	19.26	728	0.08	140	6.75	4375	0.27	133	22.09	954
SPA17	1.67	4.90E-17	0.28	120	19.26	729	0.23	127	17.84	693	0.24	108	14.06	2582
CD46_t2	1.27	9.95E-08	0.26	125	19.26	730	0.16	120	11.19	2402	0.25	132	20.28	1232
PSMC3IP	1.64	3.75E-18	0.23	134	19.24	731	0.25	112	15.74	1105	0.33	140	29.34	277
LIN28B	1.09	0.000135056	0.19	150	19.23	732	0.04	137	3.44	6224	0.21	139	18.52	1549
RNF8	1.35	2.11E-15	0.22	136	19.21	733	0.13	131	10.38	2725	0.33	135	27.79	386
PDCD5	1.75	3.74E-26	0.35	105	19.21	734	0.29	99	14.43	1417	0.42	85	14.62	2427
GNG4	1.87	2.24E-09	0.22	138	19.2	735	0.11	145	10.52	2672	0.21	124	15.64	2167
TMCO1	1.54	5.45E-18	0.19	149	19.2	736	0.22	140	19.45	458	0.35	130	28.36	348
TMEM136	1.53	8.46E-08	0.23	133	19.2	737	0.24	128	19.05	512	0.16	141	15	2338
PTTG1	3.84	2.70E-41	0.31	112	19.19	738	0.2	99	9.93	2885	0.41	112	25.49	563

YWHAG	1.57	9.31E-17	0.24	129	19.17	739	0.29	140	26.29	50	0.37	128	29.16	287
PFAS	1.32	8.81E-14	0.27	121	19.16	740	0.17	127	12.95	1850	0.22	124	16.64	1917
SAP130	1.41	2.25E-22	0.27	122	19.16	741	0.22	142	20.22	374	0.28	124	20.8	1158
ZNF71	1.32	5.91E-08	0.22	138	19.15	742	0.28	158	30.61	6	0.19	120	13.38	2767
KLHL23	1.46	1.10E-13	0.22	138	19.14	743	0.14	142	12.49	1984	0.3	127	23.07	837
PREB	1.35	7.19E-10	0.21	140	19.14	744	0.08	138	7.38	4046	0.23	110	13.65	2692
DNMT3A_t1	1.66	2.02E-22	0.33	108	19.14	745	0.23	120	16.37	968	0.33	118	22.68	883
MYO19_t1	1.53	5.31E-16	0.26	123	19.13	746	0.23	140	20.38	354	0.36	123	26.43	463
DROSHA	1.55	5.44E-19	0.25	127	19.13	747	0.27	121	18.82	547	0.39	133	32.55	140
WHSC1_t3	1.16	5.86E-10	0.23	132	19.13	748	0.15	146	14.79	1335	NA	NA	NA	NA
ORC1	1.29	2.22E-16	0.29	115	19.12	749	0.2	112	12.57	1966	0.37	114	23.54	784
HSP90AB1	1.46	1.54E-15	0.27	122	19.12	750	0.23	110	13.62	1643	0.3	107	17.36	1755
FAM82B_t1	1.15	0.005786974	0.19	150	19.11	751	NA	NA	NA	NA	0.26	118	17.55	1726
INPP5F_t1	1.1	0.001111845	0.2	147	19.11	752	NA	NA	NA	NA	0.23	141	20.79	1160
CENPL	1.42	1.80E-19	0.29	117	19.11	753	0.19	135	16.03	1036	0.39	146	37.77	41
RPRD1A_t1	1.19	0.000273092	0.21	143	19.1	754	NA	NA	NA	NA	0.24	150	23.9	735
CD34	2.72	8.91E-28	0.31	111	19.09	755	0.25	128	19.46	455	0.38	124	28.19	359
TMEM50A	1.26	1.13E-06	0.23	134	19.08	756	0.23	142	20.72	319	0.15	135	12.56	3018
TMPO_t1	1.33	4.56E-10	0.21	140	19.08	757	0.08	141	7.61	3928	0.31	123	22.96	848
DLGAP4	1.37	6.65E-17	0.27	120	19.08	758	0.23	170	28.08	25	0.24	113	15.43	2209
HLA-C	1.49	1.57E-08	0.26	122	19.08	759	0.2	112	12.32	2026	0.24	122	17.04	1817
HAPLN1	1.16	3.10E-09	0.19	148	19.07	760	0.11	122	7.81	3829	0.28	146	27.3	407
CTTN	1.21	2.04E-06	0.26	123	19.06	761	0.29	147	27.74	30	0.18	122	12.74	2963
PPFIA1_t1	1.29	2.48E-07	0.22	136	19.05	762	0.24	124	17.81	698	0.3	127	23.15	828
CPD	1.39	8.34E-10	0.19	149	19.05	763	0.21	134	17.64	727	0.34	146	33.09	120
GTF2I_t2	1.17	8.56E-12	0.19	148	19.05	764	0.23	123	16.99	843	0.3	119	21.02	1116
TYW5	1.1	0.000181249	0.18	153	19.04	765	0.16	127	12.02	2125	0.16	127	12.68	2981
GATS_t1	1.37	1.21E-18	0.25	126	19.03	766	NA	NA	NA	NA	0.3	107	17.06	1812
MYO19_t2	1.67	7.01E-21	0.29	115	19.01	767	0.15	147	14.59	1381	0.36	91	14.72	2403
PPME1_t1	1.15	1.88E-13	0.25	127	18.99	768	NA	NA	NA	NA	0.25	123	18.32	1590
SGPL1	1.21	3.77E-08	0.19	152	18.99	769	0.01	125	0.95	7832	0.21	132	17.33	1765
SLC2A13	1.09	0.000576021	0.16	172	18.99	770	0.06	137	5.16	5241	0.12	143	11.31	3411
DONSON	1.68	1.73E-13	0.26	124	18.98	771	0.18	114	11.32	2351	0.36	111	22.02	966
WDR41	1.2	2.51E-06	0.23	131	18.98	772	0.08	124	5.77	4911	0.25	123	18.26	1597
B3GALNT1	1.18	2.11E-09	0.25	127	18.98	773	0.22	136	19.02	515	0.19	128	15.21	2267
CXorf36_t1	1.51	2.87E-23	0.26	122	18.97	774	NA	NA	NA	NA	NA	NA	NA	NA
REST	1.07	9.97E-06	0.19	150	18.97	775	0.22	130	17.79	701	0.32	140	28.52	330
TMEM209	1.63	5.15E-23	0.27	120	18.96	776	0.22	121	15.43	1159	0.44	131	35.91	70
PSMD14	1.45	3.02E-13	0.22	136	18.95	777	0.21	124	15.78	1097	0.33	117	21.82	1002
LMBRD2	1.53	5.51E-17	0.27	119	18.95	778	0.26	124	18.89	535	0.4	120	28.06	368
FTH1_t1	1.59	8.15E-14	0.31	111	18.95	779	NA	NA	NA	NA	0.34	125	25.54	554
EEF1E1	1.58	5.79E-13	0.26	123	18.94	780	0.26	116	17.23	795	0.33	114	20.83	1148
LAMP2_t1	1.2	0.001308885	0.2	143	18.94	781	0.13	143	12.45	1991	0.25	152	25.5	561
RUNDC1	1.18	1.13E-07	0.22	135	18.94	782	0.19	139	16.77	885	0.18	153	18.8	1503
COX6C	1.57	3.83E-12	0.27	121	18.93	783	0.15	102	7.9	3790	0.4	124	29.34	278
SLC35E3	1.47	3.16E-15	0.23	131	18.93	784	0.11	139	9.47	3079	0.32	157	34.31	93
URB2	1.31	9.33E-10	0.23	133	18.92	785	0.18	115	11.99	2129	0.28	122	19.81	1301
TREM2	1.39	2.51E-08	0.21	142	18.92	786	0.18	114	11.32	2350	0.06	114	3.98	6422
IPO9	1.44	2.98E-22	0.22	136	18.92	787	0.21	149	20.71	322	0.28	152	29.04	297
SPATS2	1.21	8.30E-19	0.25	126	18.91	788	0.19	128	14.99	1283	0.34	128	26.29	473
DOCK7	1.21	1.04E-05	0.27	120	18.9	789	0.17	114	10.88	2523	0.31	123	22.3	923
HSPA1A	1.59	3.93E-06	0.25	126	18.89	790	0.1	109	6.15	4698	0.33	136	28.26	354
SLC35A2_t1	1.48	9.08E-20	0.26	123	18.89	791	0.23	124	17.16	807	0.22	118	15.28	2253
ESM1	2.78	3.66E-26	0.24	129	18.89	792	0.2	129	15.77	1098	0.44	128	34.25	94
RBM18	1.39	1.10E-16	0.25	127	18.89	793	0.21	122	15.28	1203	0.31	146	30.16	232
KIF2C	1.96	7.29E-27	0.28	118	18.87	794	0.12	150	11.51	2301	0.33	137	29.05	296
NVL	1.42	1.51E-15	0.32	109	18.86	795	0.26	120	17.93	677	0.34	131	27.62	392
PIK3R3	1.1	9.82E-07	0.22	135	18.84	796	0.09	140	7.81	3827	0.21	135	17.45	1743
ADNP	1.18	0.000125292	0.21	139	18.84	797	0.04	119	2.79	6620	0.25	139	22.47	902
GINS3	1.64	2.68E-16	0.2	145	18.84	798	0.17	123	12.58	1959	0.3	130	23.93	731
UBE2Q2_t1	1.11	0.001910301	0.21	138	18.84	799	NA	NA	NA	NA	0.2	133	16.25	2014
TIMELESS	1.79	2.00E-24	0.28	117	18.84	800	0.21	113	13.01	1830	0.37	130	29.96	244
OSGIN2_t1	1.14	0.011419958	0.2	145	18.83	801	0.09	127	6.94	4277	NA	NA	NA	NA
GDAP2	1.13	2.58E-09	0.19	150	18.82	802	0.18	135	15.52	1138	0.24	151	23.85	742
LYSMD4	1.39	2.94E-16	0.22	136	18.82	803	0.23	110	13.64	1641	0.3	151	29.87	250
RPS6KC1	1.3	2.94E-11	0.2	145	18.82	804	0.25	140	22.52	185	0.3	137	26.01	501
SOWAHA	1.86	4.39E-13	0.21	139	18.82	805	0.07	113	4.28	5725	0.29	135	25.05	617
OSTM1	1.27	1.48E-09	0.2	145	18.82	806	0.21	140	19.1	501	0.22	147	20.89	1134
SGOL2	1.58	1.21E-20	0.28	117	18.81	807	0.24	106	13.36	1730	0.38	133	31.76	172
ICK_t1	1.12	1.14E-10	0.21	141	18.81	808	NA	NA	NA	NA	NA	NA	NA	NA
ADAMTS6	1.12	2.34E-05	0.19	150	18.77	809	0.25	144	23.94	115	0.15	126	11.76	3279
COMMD10	1.38	5.86E-10	0.22	134	18.76	810	0.16	134	13.34	1737	0.36	138	32.07	157
CCZ1B	1.31	1.89E-10	0.2	142	18.75	811	0.19	124	13.92	1553	0.27	120	18.81	1501
ZNF772	1.06	0.000404778	0.2	146	18.75	812	0.2	129	15.48	1150	0.13	145	12.59	3009
SUCO	1.64	5.80E-10	0.2	145	18.75	813	0.33	117	22.11	205	0.3	130	23.61	775
MECOM_t2	1.17	8.99E-06	0.21	138	18.75	814	0.14	150	13.97	1540	0.16	131	13.22	2825
TRAF3	1.16	2.99E-09	0.21	138	18.72	815	0.25	155	26.33	49	0.18	132	14.57	2440
TIGD7	1.32	4.56E-13	0.31	111	18.72	816	0.19	90	7.69	3890	0.39	98	18.85	1491
CENPK	1.76	1.30E-21	0.26	122	18.72	817	0.25	105	13.7	1623	0.38	126	29.03	298
DAD1_t1	1.32	5.98E-13	0.24	128	18.71	818	NA	NA	NA	NA	NA	NA	NA	NA
MOB1A	1.06	0.054697347	0.25	125	18.71	819	0.13	118	9.01	3268	0.03	110	1.75	7597
MTPN_t1	1.13	0.000338827	0.2	142	18.7	820	NA	NA	NA	NA	NA	NA	NA	NA
CCAR1	1.26	3.21E-06	0.28	118	18.7	821	0.28	149	27.6	32	0.33	137	28.36	349
GPN1	1.2	8.08E-12	0.29	115	18.69	822	0.19	138	16.32	978	0.23	133	19.03	1446
TSNAX	1.34	3.21E-09	0.21	139	18.68	823	0.18	131	14.92	1299	0.34	132	27.77	387
STX1A_t1	1.46	1.51E-16	0.26	121	18.67	824	0.28	140	25.16	75	NA	NA	NA	NA
PODXL	1.85	7.98E-12	0.24	129	18.67	825	0.27	140	24.08	108	0.23	136	20.08	1268
GEMIN7	1.22	6.16E-12	0.25	124	18.67	826	0.07	134	5.72	4942	0.22	111	13.37	2772
ABCC5	1.54	2.14E-18	0.3	113	18.66	827	0.3	134	25.04	78	0.28	150	28.08	367
SFMBT2	1.02	0.005152465	0.2	142	18.66	828	0.06	129	4.54	5591	0.18	143	17.06	1815
DZIP3_t1	1.21	0.000123464	0.17	157	18.66	829	0.14	110	8.65	3435	NA	NA	NA	NA
SPAST	1.12	3.54E-07	0.23	132	18.64	830	0.2	127	15.56	1130	0.23	136	19.47	1353
CDCA3_t1	1.59	1.47E-20	0.28	116	18.64	831	NA	NA	NA	NA	NA	NA	NA	NA
OPHN1	1.07	2.44E-08	0.18	155	18.64	832	0.17	140	14.86	1315	0.2	163	22.1	952
THAP8	1.23	3.98E-11	0.2	141	18.62	833	0.19	120	13.02	1827	0.18	107	10.14	3819
RBBP7	1.34	8.22E-08	0.22	135	18.62	834	0.16	129	12.71	1923	0.3	123	21.54	1042
ATP2C1_t2	1.18	6.60E-07	0.27	118	18.61	835	0.09	104	5.13	5262	0.37	108	21.52	1044
KLHL18	1.05	5.61E-05	0.2	141	18.61	836	0.14	152	14.15	1492	0.24	142	21.89	985
PUS7L	1.06	2.70E-07	0.22	134	18.6	837	0.18	114	11.65	2257	0.28	125	21.1	1103
CCDC132_t2	1.21	4.31E-12	0.21	138	18.6	838	0.21	108	12.19	2067	0.36	103	19.23	1403
ANLN	2.21	1.39E-25	0.29	115	18.6	839	0.26	112	16.07	1027	0.43	138	38.2	32
HLTF_t1	1.53	9.31E-15	0.26	121	18.6	840	0.16	125	11.92	2149	0.33	127	25.41	573
ZC3H8	1.14	1.61E-12	0.29	115	18.59	841	0.18	130	14.73	1346	0.33	114	20.9	1133
HMGB2	1.67	3.94E-13	0.2	141	18.57	842	0.16	108	9.21	3187	0.24	147	23.57	780
RRP7A	1.07	0.00103893	0.19	147	18.56	843	0.02	151	1.85	7243	0.15	133	12.8	2949
HLTF_t2	1.67	5.49E-14	0.26	121	18.53	844	0.23	140	21.09	285	0.37	127	28.48	336

ZNF493_t1	1.26	1.96E-08	0.23	131	18.53	845	NA	NA	NA	NA	NA	NA	NA	NA
TRMT61A	1.28	7.93E-12	0.18	153	18.52	846	0.13	122	9.22	3181	0.16	127	12.66	2991
TNKS	1.05	4.08E-05	0.17	159	18.52	847	0.09	136	7.59	3934	0.13	160	14.42	2483
FAM72D	1.71	4.65E-20	0.26	120	18.49	848	0.27	148	25.98	63	0.38	135	32.31	151
GMCL1_t1	1.17	6.83E-10	0.22	135	18.49	849	NA	NA	NA	NA	NA	NA	NA	NA
DOCK4_t1	1.03	0.51189669	0.18	154	18.48	850	NA	NA	NA	NA	NA	NA	NA	NA
ZNF707	1.4	5.68E-12	0.26	120	18.48	851	0.18	112	11.34	2346	0.25	145	23.29	811
TBL1XR1	1.14	1.31E-08	0.24	127	18.47	852	0.24	132	19.37	468	0.21	158	22.88	855
POLA1	1.48	2.83E-17	0.25	124	18.47	853	0.2	134	16.8	881	0.38	148	37.35	50
DLGAP5	2.53	3.39E-28	0.32	107	18.46	854	0.23	119	16.09	1023	0.41	107	23.34	809
SMC2	1.53	7.43E-17	0.23	129	18.46	855	0.2	140	18.23	619	0.35	126	26.87	429
PIK3CB	1.14	2.27E-05	0.2	142	18.44	856	0.09	128	7.33	4076	0.2	163	22.16	943
BUB1	2.25	3.41E-25	0.32	108	18.44	857	0.24	110	14.58	1384	0.42	106	23.66	767
CCNYL1_t1	1.23	2.00E-06	0.2	144	18.43	858	NA	NA	NA	NA	0.26	131	21.11	1100
PREPL_t1	1.16	2.37E-09	0.17	159	18.42	859	NA	NA	NA	NA	0.28	144	25.93	515
RPAP3	1.23	8.45E-07	0.22	134	18.41	860	0.18	123	13.5	1683	0.31	124	22.8	864
AMMECR1	1.28	8.25E-11	0.2	144	18.41	861	0.18	127	13.91	1561	0.36	149	35.64	74
BRCA2	1.08	2.87E-08	0.21	136	18.4	862	0.17	111	10.61	2628	0.18	129	13.94	2608
UBE2C	4.02	3.21E-31	0.28	115	18.39	863	0.23	88	8.89	3335	0.37	110	22.27	926
THUMPD2	1.17	5.23E-07	0.23	130	18.39	864	0.17	124	12.89	1871	0.29	115	18.79	1506
GRN	1.23	7.41E-06	0.28	115	18.38	865	0.18	124	12.95	1848	0.15	113	9.22	4154
PARG	1.17	1.71E-13	0.2	140	18.38	866	0.19	129	14.79	1332	0.35	140	31.42	186
SMC1A	1.17	4.98E-08	0.18	153	18.37	867	0.07	131	5.96	4812	0.23	134	19.3	1386
GGCT	1.46	5.20E-11	0.23	129	18.37	868	0.2	120	13.8	1599	0.39	122	28.14	362
ELAVL1_t1	1.21	1.28E-11	0.24	127	18.36	869	NA	NA	NA	NA	NA	NA	NA	NA
HINT3	1.17	1.70E-06	0.21	136	18.36	870	0.21	127	16.46	947	0.11	137	9.71	3978
ABI1	1.28	2.46E-10	0.27	119	18.36	871	0.24	123	17.72	710	0.23	130	18.35	1580
E2F1	1.99	5.33E-24	0.28	115	18.35	872	0.17	123	12.26	2046	0.37	120	26.18	484
EIF2S2	1.34	6.09E-09	0.25	123	18.35	873	0.2	131	15.84	1080	0.35	123	25.67	541
EFCAB7	1.38	2.89E-11	0.24	127	18.35	874	0.22	98	10.52	2668	0.28	104	15.38	2222
MINOS1	1.26	3.86E-07	0.22	132	18.35	875	0.24	131	19.11	500	0.33	129	25.84	522
PTPDC1	1.22	1.02E-09	0.2	142	18.33	876	0.22	145	20.84	308	0.36	138	31.64	179
ARL1_t1	1.34	1.82E-09	0.2	143	18.33	877	NA	NA	NA	NA	0.25	147	23.77	754
SMAP1	1.27	1.05E-13	0.17	156	18.31	878	0.19	142	17.7	714	0.33	122	23.69	765
CDK1	3	1.53E-28	0.33	106	18.31	879	0.22	114	14.06	1518	0.43	107	24.33	686
VAMP4	1.22	3.42E-08	0.18	149	18.31	880	0.1	148	9.8	2937	0.24	142	21.89	986
NACC1	1.46	1.76E-18	0.26	120	18.31	881	0.24	138	20.72	321	0.25	139	22.68	882
PRKRIP1_t1	1.25	5.46E-15	0.23	130	18.28	882	0.14	127	10.93	2504	NA	NA	NA	NA
UTP15	1.2	1.23E-09	0.24	126	18.28	883	0.15	117	10.28	2766	0.37	121	26.13	489
CTDSPL2	1.16	0.000930423	0.21	136	18.27	884	0.11	145	10.52	2669	0.19	139	16.99	1827
FBXL20	1.19	9.20E-13	0.2	141	18.27	885	0.12	139	10.37	2730	0.19	160	20.64	1181
PSMG3	1.54	2.06E-27	0.27	118	18.27	886	0.16	107	8.85	3355	0.32	116	21	1119
MINA	1.19	6.19E-07	0.19	146	18.26	887	0.17	126	12.95	1851	0.18	143	16.66	1912
MKRN3	1.29	3.16E-14	0.18	154	18.25	888	0.14	135	11.75	2211	0.28	125	21.19	1086
SNX12	1.16	7.95E-11	0.26	120	18.25	889	0.14	132	11.53	2294	0.25	132	20.45	1206
PFDN4	1.43	5.90E-14	0.25	123	18.25	890	0.3	114	19.38	467	0.32	123	23.2	819
USP14	1.35	1.88E-10	0.21	138	18.24	891	0.19	139	17.29	781	0.28	133	23.36	804
DCAF17	1.11	4.49E-05	0.22	134	18.24	892	0.1	130	7.77	3849	0.2	160	21.95	972
SCRIB	1.62	1.41E-15	0.34	104	18.23	893	0.24	111	14.54	1394	0.31	112	19.27	1392
IGF2BP3	2.77	1.03E-15	0.21	137	18.23	894	0.19	134	16.33	975	0.32	135	26.85	430
GGA3	1.23	1.70E-12	0.21	138	18.22	895	0.18	132	14.41	1423	0.09	120	5.98	5467
KPNA1	1.04	0.09803983	0.19	147	18.21	896	-0.06	129	-5.07	11698	0.14	139	12.76	2957
LIMS1	1.03	0.560758339	0.17	157	18.21	897	0.23	135	19.41	463	0.07	132	5.84	5527
RPS6KB1	1.14	0.003957672	0.19	146	18.21	898	0.18	134	15.27	1206	0.24	151	24.5	666
ATP8B1	1.24	4.23E-09	0.22	133	18.21	899	0.16	135	13.7	1626	0.27	134	22.33	921
MCM10	1.5	2.17E-16	0.24	126	18.2	900	0.2	127	15.22	1219	0.35	139	31.26	192
UMPS	1.18	1.83E-05	0.19	148	18.2	901	-0.02	146	-1.69	9611	0.22	135	18.66	1528
PIGC	1.7	3.48E-23	0.28	116	18.2	902	0.27	106	15.06	1259	0.35	102	18.39	1571
STIP1_t1	1.38	1.71E-14	0.28	116	18.2	903	NA	NA	NA	NA	0.32	104	17.17	1796
PIP4K2B_t1	1.16	2.49E-07	0.2	143	18.2	904	NA	NA	NA	NA	0.13	131	10.47	3700
SWT1	1.28	1.65E-12	0.22	133	18.19	905	0.19	121	13.22	1769	0.33	140	29.64	258
TMEM199	1.49	9.06E-24	0.29	113	18.19	906	0.22	119	15.23	1215	0.32	126	24.55	663
NCOA6	1.25	7.64E-12	0.22	133	18.19	907	0.2	133	16.52	939	0.2	128	15.8	2131
PCDHB14	1.38	1.25E-07	0.2	140	18.18	908	0.15	97	6.93	4283	0.13	126	9.85	3935
CNTD1	1.11	1.64E-07	0.17	157	18.18	909	0.09	131	7.02	4235	0.22	128	16.8	1871
MAGOHB_t1	1.07	1.46E-06	0.16	163	18.18	910	NA	NA	NA	NA	NA	NA	NA	NA
SPP1	3.56	1.82E-08	0.22	132	18.17	911	0.32	117	21.49	247	0.2	141	17.8	1683
SHARPIN	1.28	5.46E-15	0.24	126	18.17	912	0.13	114	8.46	3527	0.28	90	11.34	3402
MAPRE1_t1	1.11	0.07517819	0.21	138	18.16	913	NA	NA	NA	NA	0.08	136	7.03	5024
UEVLD_t1	1.11	2.90E-09	0.23	130	18.16	914	0.17	133	13.83	1587	0.24	124	18.07	1629
NUDT3	1.26	9.35E-13	0.23	128	18.14	915	0.25	120	17.19	803	0.25	137	21.59	1035
RPL23	1.63	5.07E-16	0.27	117	18.14	916	0.29	80	8.81	3372	0.3	102	15.63	2169
NECAB3	1.59	8.11E-18	0.25	122	18.13	917	0.15	118	9.97	2871	0.3	119	20.82	1155
ZCCHC17_t1	1.25	4.89E-10	0.28	115	18.12	918	NA	NA	NA	NA	0.21	139	18.26	1598
PHF6_t2	1.19	5.68E-13	0.24	127	18.12	919	0.2	82	6.26	4634	0.31	101	15.97	2080
PRLR	1.23	1.70E-08	0.18	152	18.1	920	0.18	151	18.03	658	0.25	140	22.06	959
PYCRL	1.59	1.03E-14	0.21	136	18.09	921	0.17	152	16.93	856	0.27	139	23.81	748
NEU1	1.69	5.38E-23	0.27	116	18.08	922	0.13	117	8.93	3318	0.3	119	20.51	1198
OIP5	3.58	7.82E-30	0.26	120	18.07	923	0.24	107	13.57	1665	0.41	122	29.23	282
MYCBP_t1	1.28	9.70E-08	0.22	133	18.07	924	NA	NA	NA	NA	0.26	142	24.23	698
SIPA1L3_t1	1.43	2.18E-11	0.22	132	18.07	925	NA	NA	NA	NA	NA	NA	NA	NA
BPGM	1.48	5.76E-18	0.27	118	18.07	926	0.23	113	14.64	1370	0.39	123	28.5	332
ZNF174	1.34	1.54E-18	0.23	127	18.05	927	0.21	142	19.7	434	0.29	115	18.98	1457
BAGE4	1.11	1.48E-08	0.2	139	18.04	928	0.2	128	15.96	1059	0.22	124	16.05	2062
ANGEL2_t1	1.22	1.97E-13	0.24	124	18.04	929	NA	NA	NA	NA	0.27	155	28.35	350
ZMYM1	1.37	3.92E-13	0.23	128	18.03	930	0.23	125	17.14	810	0.35	137	30.2	230
PLVAP	2.09	4.16E-28	0.28	115	18.03	931	0.25	141	22.3	194	0.34	115	22.34	919
KIAA1522	1.59	1.41E-09	0.23	127	18.03	932	0.2	129	15.78	1096	0.22	129	17.13	1804
PEX11B	1.5	5.13E-23	0.27	116	18.03	933	0.12	118	8.5	3501	0.3	119	20.91	1131
CXorf56	1.13	4.09E-08	0.21	136	18.03	934	0.11	139	9.43	3099	0.13	133	11.11	3473
PPIAL4A	1.48	4.16E-21	0.31	109	18.03	935	0.32	113	19.91	409	0.34	115	21.87	989
DCAF7	1.26	4.79E-14	0.21	135	18.02	936	0.11	129	8.63	3442	0.16	144	14.92	2356
TMEM194A_t1	1.27	3.00E-08	0.19	147	18.02	937	NA	NA	NA	NA	0.27	135	23.16	825
MEX3C	1.1	3.55E-08	0.21	135	18.02	938	0.19	143	17.5	746	0.14	135	12.13	3154
MAPT	1.76	3.23E-17	0.21	134	18.01	939	0.08	125	6.04	4758	0.32	138	28.02	370
UCHL5_t1	1.37	1.94E-08	0.2	141	18	940	NA	NA	NA	NA	0.33	126	25.27	587
TOB2P1	1.42	2.56E-21	0.21	135	18	941	0.08	149	7.49	3997	0.26	120	17.89	1665
TMEM48	1.43	2.03E-12	0.2	142	18	942	0.2	123	14.51	1404	0.3	141	27.27	408
KIAA0494_t1	-1.02	0.463257745	0.19	147	18	943	NA	NA	NA	NA	NA	NA	NA	NA
PLS1	1.23	0.000587692	0.19	144	18	944	0.04	138	3.1	6437	0.28	135	23.65	770
TFG	1.28	5.83E-14	0.22	130	18	945	0.21	120	14.63	1372	0.24	133	19.96	1278
JRKL	1.24	3.03E-11	0.21	134	17.99	946	0.29	116	19.2	486	0.28	129	22.05	963
VBP1	1.38	1.32E-09	0.24	126	17.99	947	0.15	119	10.05	2844	0.3	127	23.4	800
ZNF257_t1	1.14	2.06E-05	0.23	127	17.98	948	NA	NA	NA	NA	0.17	139	15.55	2191
SETDB1	1.59	2.77E-24	0.25	121	17.98	949	0.27	104	14.79	1331	0.29	111	17.5	1735
G2E3_t1	1.08	1.74E-06	0.16	161	17.98	950	NA	NA	NA	NA	0.23	131	18.99	1456

ACBD3	1.22	6.30E-07	0.22	130	17.97	951	0.19	118	13.04	1817	0.31	165	35.18	80
LYRM2_t1	1.16	1.64E-07	0.22	133	17.96	952	NA	NA	NA	NA	0.17	161	19.16	1417
HMGN1_t1	1.15	0.002642297	0.17	154	17.96	953	NA	NA	NA	NA	NA	NA	NA	NA
OTUD6B	1.27	6.87E-10	0.19	145	17.96	954	0.3	130	24.38	102	0.31	131	24.8	636
PPP3R1	1.07	3.53E-08	0.19	145	17.95	955	0.2	118	13.34	1735	0.19	134	15.74	2144
ADAM9	1.22	0.000104743	0.2	142	17.95	956	0.23	125	17.01	838	0.18	123	12.89	2927
GRWD1	1.25	9.07E-15	0.24	124	17.94	957	0.06	120	4.26	5749	0.16	127	12.37	3079
LOXL2	1.48	4.86E-07	0.2	140	17.93	958	0.23	124	16.99	844	0.21	121	15.05	2318
CDKN2AIPNL	1.62	1.15E-23	0.23	128	17.93	959	0.16	143	15.28	1205	0.38	152	39.15	22
SKP1_t1	1.21	8.88E-06	0.23	129	17.92	960	NA	NA	NA	NA	0.33	146	31.71	175
CASC3	1.41	6.31E-14	0.21	134	17.91	961	0.24	121	17.1	819	0.23	134	19.57	1336
CPSF4	1.55	3.50E-22	0.27	116	17.91	962	0.21	113	12.93	1855	0.35	118	23.81	746
INSM1	1.03	0.15866153	0.2	140	17.89	963	0.03	139	3.09	6446	0.13	147	12.14	3152
AHCYL2	1.1	0.000156958	0.22	130	17.89	964	0.13	148	13	1834	0.19	128	14.68	2411
STIL	1.25	1.87E-13	0.27	117	17.89	965	0.22	130	17.2	801	0.36	130	28.97	304
DPH3	1.47	7.33E-13	0.24	125	17.88	966	0.24	146	22.64	174	0.28	141	25.81	525
MED22	1.44	5.73E-15	0.26	120	17.88	967	0.34	122	24.54	97	0.19	111	11.35	3398
MASTL	1.17	3.65E-14	0.26	119	17.88	968	0.22	141	19.75	426	0.27	155	28.37	347
KCTD6	1.6	1.19E-17	0.22	133	17.86	969	0.14	127	10.56	2651	0.36	134	30.6	217
TIMM21	1.12	3.26E-08	0.23	127	17.86	970	0.14	115	9.42	3100	0.27	140	23.94	729
NAA38_t1	1.27	2.69E-09	0.16	159	17.86	971	NA	NA	NA	NA	0.23	141	20.98	1125
DHFR	1.23	4.09E-06	0.19	144	17.85	972	0.08	129	6.15	4704	0.17	143	15.54	2193
CSNK1A1	1.16	1.31E-05	0.23	129	17.84	973	0.1	120	7.07	4211	0.26	143	24.62	660
WAPAL	1	0.94595441	0.17	157	17.82	974	0.09	126	6.7	4400	0.2	126	15.03	2329
TMEM17	1.19	5.65E-10	0.23	127	17.78	975	0.22	118	14.95	1293	0.23	116	15.05	2323
TMEM109	1.2	1.40E-07	0.27	117	17.77	976	0.12	135	10.46	2696	0.24	131	19.73	1316
UQCRB	1.2	6.56E-05	0.21	136	17.77	977	0.04	136	3.14	6413	0.34	130	27.32	406
IQCB1	1.26	6.80E-08	0.22	129	17.75	978	0.14	114	8.73	3399	0.18	112	11.11	3471
RNF213	1.43	1.31E-13	0.21	136	17.74	979	0.2	135	16.91	860	0.2	138	17.84	1675
CRNKL1_t1	1.27	1.10E-09	0.25	121	17.74	980	NA	NA	NA	NA	0.34	126	25.98	504
EFNA1	1.47	1.74E-05	0.28	113	17.73	981	0.08	128	6.56	4473	0.31	104	16.93	1847
TMEM184B	1.32	4.14E-06	0.18	147	17.73	982	0.25	136	21.54	245	0.18	120	12.67	2989
GFPT1	1.23	4.39E-09	0.18	147	17.73	983	0.2	151	19.83	420	0.19	148	18.24	1603
CC2D1B	1.21	1.04E-12	0.2	139	17.72	984	0.19	131	15.18	1228	0.13	139	11.22	3436
CCT3	2.02	1.15E-32	0.26	119	17.71	985	0.25	109	14.73	1344	0.34	106	19.16	1419
TRIM59	1.59	3.15E-13	0.21	133	17.71	986	0.26	130	20.99	294	0.25	147	23.82	744
PRKDC	1.54	1.06E-18	0.24	124	17.71	987	0.24	129	18.91	530	0.36	142	33.45	110
NUP155	1.4	2.60E-18	0.28	114	17.7	988	0.14	104	7.55	3963	0.46	79	13.4	2758
HELLS	1.43	4.61E-18	0.27	116	17.7	989	0.17	121	11.85	2170	0.31	107	17.91	1660
NDUFAF5	1.26	1.60E-10	0.2	139	17.7	990	0.11	135	9.56	3035	0.28	118	18.87	1484
DUSP18_t1	1.08	2.31E-09	0.25	120	17.7	991	NA	NA	NA	NA	NA	NA	NA	NA
UTP23	1.32	2.05E-09	0.22	129	17.7	992	0.22	133	17.96	673	0.24	138	21.06	1107
ACTL6A	1.33	8.33E-11	0.25	120	17.7	993	0.26	119	18.08	649	0.26	125	19.23	1404
CDKN2B_t2	1.5	1.05E-12	0.17	154	17.69	994	0.19	136	16.57	928	0.23	147	22.1	951
NR2C2AP	1.79	7.71E-32	0.29	110	17.68	995	0.33	109	19.34	473	0.31	146	30	241
ZNF143	1.24	6.08E-09	0.21	134	17.68	996	0.24	126	18.53	580	0.3	126	22.58	887
TMEM39A_t1	1.15	1.21E-06	0.15	168	17.68	997	0.15	141	13.3	1749	NA	NA	NA	NA
ARHGAP21	1.16	0.001395927	0.22	129	17.67	998	0.12	135	10.02	2859	0.32	151	32.63	136
TRIM23_t1	1.14	1.99E-05	0.21	133	17.67	999	0.07	124	4.95	5373	0.24	143	21.97	970
TXNRD1	1.66	1.07E-13	0.23	127	17.66	1000	0.2	112	12.37	2016	0.26	144	24.53	664
GDI1	1.21	1.63E-07	0.19	142	17.65	1001	0.16	130	12.54	1971	0.2	132	15.99	2073
CHMP4C	1.2	1.47E-05	0.22	131	17.63	1002	0.15	144	14.26	1461	0.22	121	15.81	2129
MTFR1	1.3	9.86E-08	0.2	139	17.63	1003	0.17	130	13.57	1669	0.34	151	34.22	95
CTSA	1.57	2.69E-15	0.29	110	17.63	1004	0.13	101	6.84	4333	0.33	137	28.39	346
H3F3A_t1	1.75	3.40E-18	0.31	106	17.62	1005	0.36	112	22.59	179	NA	NA	NA	NA
COX11_t1	1.39	9.56E-16	0.22	129	17.62	1006	0.22	116	14.66	1362	NA	NA	NA	NA
HIST1H4H	1.82	5.95E-21	0.33	104	17.62	1007	0.17	121	11.96	2135	0.31	120	21.9	978
SLC25A43	1.38	2.74E-16	0.25	120	17.62	1008	0.13	127	9.82	2926	0.35	138	30.74	208
CEP152_t1	1.3	9.34E-12	0.2	138	17.61	1009	0.24	115	15.37	1177	NA	NA	NA	NA
SELRC1	1.3	8.01E-11	0.2	136	17.6	1010	0.07	121	5.29	5176	0.24	117	15.98	2077
WDR4	1.6	1.49E-17	0.26	118	17.6	1011	0.14	94	6.27	4632	0.22	102	11.29	3417
DR1_t1	1.29	2.44E-08	0.24	123	17.57	1012	NA	NA	NA	NA	0.23	150	23.2	820
ZDHHC7	-1.02	0.612548473	0.18	147	17.57	1013	0.14	147	13.4	1713	-0.06	146	-6	11440
ZBTB40	1.07	1.29E-08	0.2	137	17.56	1014	0.16	138	14.01	1527	0.15	155	15.44	2206
RFX5	1.72	1.41E-20	0.2	138	17.55	1015	0.2	119	13.47	1693	0.33	151	33.06	122
ZNF200	1.25	1.37E-10	0.19	143	17.55	1016	0.12	119	7.97	3760	0.24	125	17.84	1678
ARHGAP39	1.27	4.53E-14	0.3	109	17.55	1017	0.25	120	17.37	766	0.27	104	14.65	2417
TRIM24	1.78	1.80E-13	0.21	135	17.55	1018	0.26	115	17.19	802	0.3	118	20.65	1180
YIPF1	1.28	1.23E-11	0.24	122	17.54	1019	0.1	112	5.93	4822	0.24	113	15.07	2312
NAA15_t1	1.09	1.93E-07	0.16	162	17.54	1020	NA	NA	NA	NA	0.2	140	18.39	1568
LMBR1	1.16	1.45E-06	0.19	142	17.54	1021	0.2	138	17.63	730	0.19	155	19.53	1342
FAM178A	1.12	0.001015718	0.21	132	17.53	1022	0.13	130	10.55	2653	0.22	149	22.11	950
ZNF215	1.09	2.85E-12	0.23	126	17.53	1023	0.18	134	15.51	1141	0.28	127	21.9	982
SMG7	1.2	2.39E-18	0.2	139	17.53	1024	0.13	131	10.14	2810	0.12	125	8.89	4265
PHB	1.4	5.86E-19	0.25	120	17.52	1025	0.16	109	9.25	3163	0.25	121	17.5	1736
PPP1R2_t1	1.13	5.18E-07	0.22	130	17.52	1026	NA	NA	NA	NA	0.22	138	19	1453
LPAR3	1.04	0.029767073	0.18	148	17.51	1027	-0.03	99	-1.51	9500	0.2	121	13.9	2618
CORO1C	1.1	0.015317273	0.17	152	17.5	1028	0.19	139	16.83	868	0.05	137	4.76	6048
DHX29	1.21	2.43E-05	0.21	134	17.49	1029	0.07	97	3.5	6184	0.38	115	24.47	671
TMEM168	1.29	4.02E-09	0.2	136	17.49	1030	0.26	142	23.73	122	0.32	133	26.59	446
ATP2C1_t3	1.19	4.61E-06	0.26	118	17.48	1031	0.14	104	7.58	3947	0.34	108	19.76	1312
MAFG	1.39	1.20E-13	0.23	125	17.47	1032	0.33	135	28.38	21	0.16	146	15.27	2258
DUS4L	1.36	9.05E-17	0.23	125	17.47	1033	0.2	127	15.47	1151	0.37	153	37.74	42
PDCD10	1.43	2.20E-12	0.25	120	17.45	1034	0.27	136	23.09	144	0.32	120	22.21	935
ZCRB1	1.2	2.18E-06	0.21	133	17.45	1035	0.08	122	6.05	4750	0.32	120	22.72	875
FOXK2	1.36	3.05E-14	0.24	122	17.44	1036	0.14	143	12.94	1853	0.31	122	22.28	924
UBQLN4	1.15	4.17E-14	0.19	140	17.43	1037	0.2	130	16.12	1015	0.23	115	15.21	2269
SLC41A1	1.43	6.99E-09	0.23	127	17.43	1038	0.21	150	20.84	309	0.19	128	15.14	2295
ZIC5	1.3	1.49E-10	0.17	153	17.43	1039	0.08	118	5.45	5084	0.27	146	25.61	546
EIF2B1	1.35	3.07E-21	0.21	132	17.43	1040	0.28	130	22.02	219	0.3	130	23.78	753
ZMYM2	1.04	0.496992942	0.19	140	17.43	1041	0.12	137	10.7	2591	0.15	142	13.84	2638
TRIO	1.26	5.98E-13	0.22	128	17.43	1042	0.21	139	18.32	612	0.23	142	21.11	1097
PRRC1_t1	1.09	0.005057289	0.19	141	17.43	1043	0.07	129	5.6	5009	NA	NA	NA	NA
HTATSF1	1.43	4.11E-10	0.19	140	17.43	1044	0.19	112	11.68	2238	0.31	113	19.27	1390
FUT2	1.25	9.53E-09	0.19	144	17.42	1045	0.1	128	8.17	3662	0.12	143	10.77	3580
EXO1	2.23	9.95E-27	0.32	104	17.42	1046	0.19	108	11.26	2378	0.45	100	22.44	907
MORC2_t1	1.33	9.58E-19	0.25	120	17.42	1047	NA	NA	NA	NA	0.29	108	16.77	1886
PSMD1	1.45	1.18E-11	0.3	109	17.42	1048	0.24	101	12.27	2044	0.34	120	23.63	772
ZNF286A	1.07	5.97E-08	0.17	154	17.4	1049	0.13	134	11.05	2451	0.24	130	19.19	1412
FBXL6	1.2	1.69E-05	0.2	138	17.4	1050	0.03	119	2.05	7114	0.2	98	9.44	4073
RPE	1.27	3.39E-11	0.25	120	17.39	1051	0.24	121	17.28	782	0.18	139	16.34	1997
HSPA13_t1	1.24	1.17E-07	0.18	148	17.39	1052	NA	NA	NA	NA	0.26	133	21.73	1012
SCML2_t1	1.07	1.42E-06	0.22	130	17.39	1053	NA	NA	NA	NA	0.31	147	30.32	229
GAD1_t1	1.17	5.16E-08	0.22	130	17.38	1054	NA	NA	NA	NA	0.15	146	14.45	2476
PIGM	1.48	2.91E-18	0.2	136	17.38	1055	0.19	131	15.42	1160	0.31	143	28.98	302
GNL3L_t1	1.2	1.42E-11	0.21	132	17.37	1056	0.2	156	21.25	262	NA	NA	NA	NA

MATR3	1.17	0.002964128	0.2	137	17.37	1057	0.17	135	14.4	1425	0.25	141	22.68	881
NPAS2	1.23	6.79E-14	0.22	128	17.37	1058	0.21	136	17.77	704	0.24	143	22.08	955
PARD3B_t1	1.1	2.77E-05	0.2	139	17.36	1059	NA	NA	NA	NA	NA	NA	NA	NA
FRMD8	1.3	8.35E-09	0.24	121	17.36	1060	0.23	139	20.17	385	0.12	122	8.47	4436
SYPL1_t1	1.46	9.90E-19	0.26	118	17.35	1061	NA	NA	NA	NA	NA	NA	NA	NA
MCM6	1.85	2.24E-16	0.24	121	17.35	1062	0.23	131	18.92	528	0.35	118	23.6	776
MRPL55	1.47	1.76E-16	0.28	113	17.35	1063	0.13	100	6.72	4389	0.3	96	13.69	2678
EXOC4_t1	1.26	5.14E-11	0.23	126	17.34	1064	0.12	138	10.96	2490	0.27	124	19.81	1303
NAE1	1.38	1.97E-08	0.33	103	17.33	1065	0.22	92	9.05	3252	0.34	114	22.03	965
TOPBP1	1.32	9.49E-12	0.27	115	17.29	1066	0.2	118	13.41	1712	0.31	109	18.22	1605
NAA40_t1	1.2	4.25E-10	0.22	127	17.29	1067	NA	NA	NA	NA	0.19	150	18.75	1513
DIDO1_t1	1.31	2.23E-08	0.16	159	17.29	1068	0.14	147	13.62	1647	0.16	140	14.44	2477
GORASP2	1.21	1.38E-10	0.28	112	17.28	1069	0.21	125	16.04	1035	0.22	143	20.87	1143
DCLRE1B	1.22	6.91E-14	0.21	132	17.28	1070	0.2	131	15.97	1055	0.15	131	12.2	3137
TMTC4	1.21	1.26E-09	0.2	136	17.27	1071	0.08	128	6.33	4603	0.18	126	13.76	2660
APIP	1.46	8.65E-14	0.24	122	17.27	1072	0.13	124	9.3	3142	0.28	115	18.37	1575
SGOL1_t1	1.42	1.05E-19	0.21	132	17.27	1073	0.17	128	13.16	1784	0.38	132	31.04	197
CHCHD2	1.58	1.18E-18	0.25	120	17.27	1074	0.27	104	14.69	1354	0.34	96	15.65	2165
KAT7	1.2	5.43E-16	0.25	120	17.26	1075	0.12	132	9.96	2876	0.17	153	17.5	1739
KIF4A	1.93	1.05E-26	0.29	109	17.24	1076	0.21	104	11.1	2426	0.42	115	27.26	409
SRI_t1	1.46	5.22E-07	0.22	128	17.24	1077	0.19	134	15.58	1122	NA	NA	NA	NA
ZNF26	1.21	8.95E-08	0.19	142	17.24	1078	0.19	139	17.03	830	0.2	131	16.54	1946
PXDNL	1.29	5.44E-19	0.23	126	17.23	1079	0.2	110	11.83	2177	0.36	123	26.25	478
ALKBH4	1.19	3.31E-10	0.18	144	17.22	1080	0.07	135	6.29	4627	0.13	125	9.61	4019
DCAF4	1.36	5.48E-12	0.18	146	17.21	1081	0.19	127	14.83	1319	0.14	123	10.16	3810
MORN4	1.11	6.37E-06	0.2	138	17.21	1082	0.1	127	7.75	3860	0.14	133	11.4	3381
LYRM4	1.26	1.31E-13	0.25	120	17.2	1083	0.15	131	11.86	2165	0.31	133	26.12	490
METTL8	1.09	0.013940471	0.18	144	17.19	1084	0.18	158	19.95	406	0.13	141	12.06	3176
ATP6V0D2_t1	1.22	4.27E-07	0.25	119	17.19	1085	NA	NA	NA		0.31	120	21.39	1066
VPS37C	1.11	0.005823648	0.18	143	17.18	1086	0.21	145	20.05	398	0.05	120	3.49	6675
PBRM1	1.14	1.39E-07	0.2	137	17.17	1087	0.18	115	11.95	2138	0.25	137	21.49	1051
TSEN2	1.47	1.78E-20	0.23	126	17.17	1088	0.21	124	15.52	1137	0.29	120	20.54	1193
NARS	1.21	0.000473746	0.24	122	17.17	1089	0.18	129	14.55	1393	0.26	122	18.69	1525
MKI67	1.25	1.36E-10	0.21	131	17.16	1090	0.08	151	7.59	3938	0.24	134	20.32	1225
AGFG1	1.47	5.85E-13	0.22	128	17.16	1091	0.25	143	23.63	126	0.28	149	27.84	383
ZNF259	1.24	1.43E-07	0.15	165	17.15	1092	0.11	126	8.56	3466	0.14	101	7.21	4941
TMEM33	1.52	7.26E-14	0.18	145	17.15	1093	0.21	148	20.21	376	0.32	129	25.5	558
KIF3A	1.32	3.63E-11	0.2	135	17.15	1094	0.21	152	21.17	269	0.3	142	27.89	377
MYO5A	1.2	0.000469208	0.19	140	17.14	1095	0.14	132	11.15	2417	0.19	145	17.96	1655
POC1A_t1	1.47	5.84E-19	0.24	120	17.14	1096	NA	NA	NA	NA	NA	NA	NA	NA
ATP6V0A2	1.08	0.000945474	0.16	156	17.12	1097	0.17	156	18.19	623	0.08	151	7.61	4791
NUP133	1.28	2.09E-06	0.23	123	17.11	1098	0.2	108	11.65	2253	0.28	149	27.44	399
PLA2G10	1.15	4.68E-05	0.24	120	17.11	1099	0.2	101	10.18	2797	0.23	120	16.19	2037
NMB	2.23	5.06E-17	0.22	127	17.11	1100	0.18	104	9.54	3040	0.35	120	24.37	682
TPTE_t1	1.27	5.99E-09	0.2	135	17.1	1101	0.2	99	9.73	2963	0.27	86	9.85	3936
PJA1	1.3	2.93E-07	0.17	148	17.1	1102	0.16	105	8.98	3283	0.22	135	18.36	1579
TICRR_t1	2.19	3.60E-26	0.29	109	17.09	1103	NA	NA	NA	NA	NA	NA	NA	NA
RRM1	1.38	9.66E-08	0.18	145	17.08	1104	0.04	112	2.37	6908	0.27	109	15.95	2083
TCERG1	1.13	0.000112533	0.22	128	17.07	1105	0.17	125	12.77	1910	0.25	126	19.01	1450
KIAA1468_t1	1.08	0.041098629	0.17	149	17.07	1106	NA	NA	NA		0.21	143	19.14	1425
N4BP3	1.16	2.29E-06	0.23	123	17.07	1107	0.18	125	13.24	1757	0.15	142	13.96	2603
DIMT1_t1	1.15	2.03E-06	0.18	145	17.06	1108	NA	NA	NA	NA	NA	NA	NA	NA
COPS8	1.1	0.0005042	0.2	134	17.05	1109	0.07	121	4.84	5432	0.09	130	7.12	4989
FAT1	1.4	3.94E-06	0.18	146	17.05	1110	0.02	121	1.61	7416	0.14	113	8.64	4367
LAMTOR3	1.09	0.023857575	0.22	129	17.05	1111	0.13	136	10.91	2513	0.21	139	18.77	1510
HDAC4_t1	1.2	6.88E-06	0.18	147	17.05	1112	NA	NA	NA		0.14	143	13.19	2833
DVL3_t1	1.12	1.63E-06	0.18	146	17.04	1113	0.15	139	13.45	1699	NA	NA	NA	NA
SMYD3	2.37	3.52E-23	0.35	98	17.04	1114	0.21	133	17.12	814	0.44	100	22.13	948
FAM53C	1.07	0.073923332	0.18	145	17.03	1115	0.08	127	6.42	4559	0.1	138	9.07	4198
PPP1R12A	1.08	0.009846025	0.18	144	17.03	1116	0.18	134	14.76	1339	0.2	131	16.56	1938
CDK12	1.07	5.00E-05	0.16	154	17.02	1117	0.07	120	4.69	5501	0.22	131	18.16	1614
NRM	1.28	1.16E-12	0.17	149	17.02	1118	0.18	130	14.59	1380	0.15	114	9.34	4104
BCL9	1.33	2.64E-16	0.23	124	17.02	1119	0.23	122	16.81	878	0.3	123	21.56	1040
CHM	1.22	2.72E-05	0.17	151	17.01	1120	0.14	129	11.06	2445	0.23	139	20.3	1229
USP3_t1	1.13	1.49E-12	0.16	154	17.01	1121	NA	NA	NA	NA	NA	NA	NA	NA
STAMBP	1.29	3.33E-21	0.21	130	17	1122	0.14	166	15.99	1050	0.3	151	29.87	251
FAM161A	1.21	3.16E-10	0.21	131	17	1123	0.13	131	10.67	2611	0.24	157	25.33	583
SPIN3	1.19	5.81E-12	0.2	133	17	1124	0.17	137	14.56	1390	0.23	146	21.85	998
STRBP	1.32	9.61E-12	0.18	146	16.98	1125	0.19	150	19.3	480	0.29	137	25.25	591
RPL23AP7	1.23	5.01E-08	0.19	141	16.98	1126	0.16	106	9.12	3218	0.2	118	13.4	2759
RALGAPB	1.2	7.33E-07	0.18	142	16.98	1127	0.16	156	17.3	777	0.23	132	18.56	1540
TATDN2	1.31	1.43E-13	0.23	124	16.97	1128	0.27	127	21.11	279	0.22	127	16.82	1866
HNRNPU_t1	1.23	4.04E-06	0.18	143	16.97	1129	NA	NA	NA		0.16	146	15.28	2254
VLDLR	1.22	0.010184132	0.17	150	16.95	1130	0.12	139	10.58	2640	0.1	120	6.96	5053
FAM111B	1.23	2.19E-15	0.18	144	16.95	1131	0.1	125	7.64	3911	0.28	118	19.27	1394
CYCS	1.44	1.24E-06	0.19	141	16.95	1132	0.15	135	12.96	1844	0.32	128	24.96	621
FKBP1A_t1	1.22	5.82E-11	0.22	128	16.94	1133	0.2	128	15.79	1093	0.14	136	11.94	3223
MRPL47	1.25	1.64E-06	0.21	131	16.94	1134	0.15	102	7.99	3753	0.2	106	11.3	3413
SEMA7A	1.08	6.95E-07	0.24	120	16.94	1135	0.16	133	13.32	1742	0.18	135	15.31	2246
PFDN1	1.08	0.000731086	0.19	140	16.94	1136	0.19	129	15.31	1190	0.04	123	3.09	6878
MRPL48	1.24	7.70E-10	0.19	138	16.93	1137	0.16	129	13.02	1821	0.15	104	7.95	4644
HSPA14	1.34	9.51E-10	0.27	112	16.92	1138	0.17	107	9.41	3105	0.32	108	18.72	1521
ATP6V1A	1.18	0.000252663	0.21	130	16.92	1139	0.1	135	8.56	3470	0.24	132	19.87	1290
TMEM182	1.26	1.20E-08	0.21	130	16.91	1140	0.19	127	14.46	1411	0.23	161	25.79	531
ZNF207	1.21	9.68E-09	0.21	131	16.91	1141	0.26	115	16.66	906	0.17	129	13.16	2842
RPL28	1.27	8.31E-08	0.17	148	16.91	1142	0.18	152	18.07	651	0.13	123	9.75	3965
UBE2V1	1.3	2.87E-17	0.23	122	16.9	1143	0.21	143	19.62	442	0.25	123	18.34	1582
DNAJC18	1.09	0.000170347	0.23	125	16.88	1144	0.21	152	20.94	299	0.12	152	12.43	3055
SERF1B_t1	1.7	4.01E-32	0.28	110	16.88	1145	NA	NA	NA		0.33	127	25.77	535
EXOC2	1.14	1.22E-07	0.21	131	16.87	1146	0.19	150	19.27	482	0.19	135	16.12	2045
SSR2	1.74	4.41E-17	0.29	108	16.87	1147	0.34	117	22.6	178	0.33	110	19.68	1322
TLL2	1.12	1.37E-05	0.19	139	16.87	1148	0.12	125	9.08	3239	0.14	132	11.75	3284
SEMA3A	1.01	0.59031345	0.16	157	16.86	1149	0.06	137	4.98	5348	0.16	150	15.85	2114
DUSP12	1.45	2.67E-16	0.21	132	16.86	1150	0.21	111	12.68	1934	0.29	132	24.13	703
CUL2	1.16	0.000293228	0.24	121	16.85	1151	0.11	135	8.95	3305	0.38	143	35.45	76
CEP170_t1	1.08	8.74E-05	0.23	124	16.84	1152	0.19	131	15.02	1274	NA	NA	NA	NA
LYPLAL1	1.38	7.01E-07	0.2	133	16.84	1153	0.17	129	13.37	1725	0.27	120	19.14	1424
ZNF692	1.45	2.70E-19	0.21	131	16.84	1154	0.17	115	11.04	2458	0.27	88	10.23	3788
CYC1	1.32	2.84E-09	0.21	131	16.83	1155	0.06	92	2.34	6932	0.31	81	9.7	3989
EEF2K	1.18	8.96E-10	0.18	144	16.83	1156	0.25	135	21.09	283	0.17	135	14.3	2522
ROCK2_t1	1.08	0.119844901	0.21	131	16.83	1157	0.15	134	12.43	1999	NA	NA	NA	NA
DNAJC9	1.38	4.77E-11	0.24	121	16.83	1158	0.19	124	13.98	1537	0.26	124	19.54	1341
ACSL4	2.04	8.79E-15	0.17	150	16.83	1159	0.16	125	12.11	2098	0.26	165	29.39	270
CCDC41	1.27	2.24E-07	0.19	140	16.82	1160	0.24	108	13.99	1533	0.31	119	21.11	1099
ZNF234	1.36	3.28E-10	0.18	144	16.82	1161	0.14	141	12.47	1986	0.15	150	14.63	2425
RASGRF1	1.14	6.26E-06	0.18	144	16.82	1162	0.07	127	5.4	5120	0.13	130	10.42	3711

CHRNA5	1.17	4.11E-13	0.17	148	16.8	1163	0.06	143	5.93	4824	0.25	150	24.71	651
SWAP70	1.24	3.82E-05	0.21	131	16.8	1164	0.21	128	16.69	899	0.2	133	16.28	2010
ZNF507	1.19	5.37E-06	0.2	136	16.79	1165	0.14	144	12.79	1904	0.27	152	27.85	382
CHN1	1.38	2.31E-10	0.23	122	16.79	1166	0.16	117	10.53	2664	0.19	126	14.72	2401
ZNF607	1.31	3.41E-17	0.2	133	16.77	1167	0.14	132	11.16	2416	0.21	121	14.64	2422
SEH1L	1.19	5.29E-05	0.21	129	16.77	1168	0.06	131	5.18	5227	0.25	130	19.86	1291
SP2	1.14	6.98E-08	0.21	128	16.77	1169	0.04	129	2.95	6521	0.2	119	14.11	2569
ZNF626_t1	1.29	5.57E-09	0.2	132	16.77	1170	0.28	125	21.1	280	0.14	132	11.88	3241
FSIP1	1.12	0.000123421	0.17	149	16.76	1171	0.09	138	8.15	3668	0.15	138	12.85	2941
CCDC34_t2	1.95	1.43E-24	0.27	113	16.76	1172	0.18	105	9.8	2936	0.37	125	27.58	394
VPS72	1.44	1.12E-26	0.21	129	16.75	1173	0.16	109	9.53	3046	0.28	141	25.79	530
CAPRIN1_t1	1.12	0.000969843	0.19	138	16.75	1174	NA	NA	NA	NA	0.16	150	16	2071
KIAA1143	1.18	0.000107922	0.18	144	16.75	1175	0.29	150	28.87	14	0.29	155	30.72	211
TMEM74	1.47	1.04E-13	0.25	116	16.74	1176	0.1	118	6.83	4337	0.26	117	17.26	1775
DMRTB1	1.15	0.000648201	0.19	139	16.74	1177	0.09	126	6.75	4377	0.16	116	10.58	3664
ATF1	1.23	2.96E-11	0.2	134	16.73	1178	0.13	111	7.96	3768	0.31	151	30.97	198
CENPF	5.41	1.02E-32	0.32	103	16.72	1179	0.25	122	17.96	670	0.42	141	38.55	30
TPM3_t2	1.27	4.34E-12	0.2	134	16.72	1180	0.25	141	22.37	191	0.18	121	12.72	2969
PAIP1	1.47	5.93E-13	0.2	133	16.72	1181	0.12	115	8.1	3697	0.32	118	21.5	1049
RBFA	1.06	0.257443696	0.17	148	16.71	1182	0.14	131	11.16	2413	0.04	132	3.03	6914
RTKN2	1.45	1.18E-13	0.21	128	16.71	1183	0.19	136	16.47	943	0.3	131	24.66	657
CCT4	1.43	2.61E-12	0.27	113	16.71	1184	0.23	121	16.42	955	0.32	119	22.05	962
NIPAL3_t1	1.37	1.54E-11	0.19	138	16.71	1185	0.15	148	14.9	1308	NA	NA	NA	NA
POLQ	1.55	4.64E-20	0.21	131	16.71	1186	0.16	132	13.14	1790	0.34	137	29.51	264
RSRC1	1.27	1.78E-08	0.23	122	16.71	1187	0.2	107	11.47	2314	0.32	131	25.99	503
CXorf57	1.51	5.22E-10	0.25	118	16.69	1188	0.08	126	6.05	4752	0.36	121	25.58	549
SLC25A19	1.21	1.27E-09	0.21	131	16.68	1189	0.11	147	10.8	2553	0.16	102	8.09	4587
SREK1IP1	1.16	4.05E-05	0.16	157	16.68	1190	0.22	132	17.66	725	0.24	140	21.29	1073
PRIM1	1.6	4.58E-18	0.26	113	16.67	1191	0.14	116	9.08	3241	0.33	94	14.65	2419
EXOSC1	1.31	5.53E-16	0.26	115	16.67	1192	0.23	116	15.45	1157	0.2	105	10.92	3537
AJUBA	1.35	0.000435062	0.19	137	16.67	1193	0.05	142	4.49	5615	0.18	120	12.88	2932
RBMX2_t1	1.22	4.36E-10	0.22	125	16.67	1194	NA	NA	NA	NA	NA	NA	NA	NA
ADAM10	1.3	2.11E-08	0.22	124	16.64	1195	0.19	126	14.73	1348	0.29	116	18.91	1473
SEPHS1	1.18	4.30E-08	0.23	123	16.64	1196	0.02	115	1.13	7729	0.24	136	20.78	1164
SMC4	1.59	1.01E-10	0.23	123	16.63	1197	0.22	129	17.05	828	0.32	134	26.87	427
SPCS2	1.25	8.12E-06	0.2	132	16.63	1198	0.23	148	22.53	184	0.18	129	14.17	2551
SDE2	1.17	9.33E-05	0.18	143	16.62	1199	0.09	116	5.74	4931	0.2	135	17.02	1821
SMARCD1	1.23	2.22E-15	0.21	131	16.62	1200	0.12	140	10.38	2727	0.14	134	11.47	3363
CD3EAP	1.36	1.86E-14	0.22	127	16.62	1201	0.21	121	14.92	1303	0.23	132	18.76	1512
PIGF	1.43	3.60E-19	0.29	108	16.62	1202	0.18	105	10.08	2830	0.36	123	26.09	495
FAIM	1.46	8.11E-10	0.25	117	16.62	1203	0.22	135	18.84	544	0.21	104	11.4	3383
GPKOW	1.26	9.35E-17	0.2	132	16.6	1204	0.04	131	3.07	6456	0.25	126	18.73	1517
SPIN4	1.36	6.23E-15	0.2	133	16.58	1205	0.17	119	11.74	2218	0.29	147	27.82	385
TEX19	1.4	3.03E-13	0.26	114	16.58	1206	0.2	118	13.88	1571	0.38	121	26.78	435
GTF2F2	1.21	4.72E-05	0.22	124	16.58	1207	0.24	120	16.46	945	0.18	124	13.35	2779
ARHGEF9	1.18	6.88E-10	0.2	132	16.57	1208	0.15	128	12.04	2120	0.23	131	18.45	1560
OLFML2A	1.55	3.83E-11	0.21	128	16.57	1209	0.24	143	22.08	208	0.23	143	21.7	1018
ATP6V0E1	1.3	2.36E-08	0.26	113	16.57	1210	0.24	115	15.35	1183	0.2	149	19.47	1354
GBAP1	1.89	2.09E-28	0.27	111	16.57	1211	0.11	122	8.11	3688	0.37	127	28.44	339
KIFC1	2.18	3.60E-29	0.29	108	16.57	1212	0.17	92	6.99	4254	0.36	115	23.24	815
XYLT2	1.41	2.01E-13	0.25	117	16.56	1213	0.22	121	15.58	1125	0.29	128	22.85	857
VPS13B_t1	1.24	1.94E-05	0.21	130	16.56	1214	0.15	141	13.78	1603	0.22	125	16.85	1865
SLC41A3	1.4	1.08E-15	0.17	147	16.56	1215	0.23	127	18.05	654	0.26	137	22.36	914
TMX3	1.07	0.007169191	0.2	134	16.55	1216	0.07	158	7.75	3859	0.19	154	20.13	1260
HIST1H4F	1.77	1.53E-19	0.32	102	16.55	1217	0.15	94	6.77	4369	0.31	80	9.35	4099
FAM104A	1.34	9.43E-13	0.18	141	16.54	1218	0.3	151	30.29	8	0.19	151	18.97	1461
MDH1	1.3	1.22E-05	0.17	146	16.53	1219	0.06	112	3.51	6179	0.27	107	15.32	2241
COMMD3	1.34	7.24E-14	0.21	128	16.52	1220	0.15	129	11.75	2210	0.32	118	22.06	960
PPM1F	1.08	3.97E-06	0.21	129	16.51	1221	0.16	139	14.49	1407	0.09	141	7.84	4694
MKRN2	1.23	2.45E-16	0.22	126	16.51	1222	0.19	123	13.79	1601	0.25	120	17.63	1712
MAZ_t1	1.12	8.24E-09	0.18	142	16.5	1223	0.1	113	5.99	4788	0.17	112	10.74	3596
PPP4R4_t1	1.27	9.28E-11	0.21	128	16.5	1224	0.19	130	15.14	1242	0.28	117	18.54	1544
ELK4	1.09	8.61E-11	0.18	140	16.49	1225	0.23	136	19.61	445	0.22	149	21.41	1061
HSPA4_t1	1.36	4.67E-08	0.24	119	16.48	1226	NA	NA	NA	NA	0.39	118	26.23	480
FBXO9_t1	1.29	1.90E-09	0.16	151	16.47	1227	NA	NA	NA	NA	0.23	144	21.79	1005
TRAPPC9	1.41	7.01E-15	0.27	111	16.47	1228	0.07	122	4.82	5437	0.32	100	16.25	2018
DTNA_t1	1.43	9.47E-11	0.23	121	16.46	1229	0.12	118	8.02	3734	0.22	110	13.27	2806
STAU2	1.2	4.63E-07	0.21	129	16.46	1230	0.12	140	10.47	2689	0.27	147	26.56	450
PARP2	1.51	5.43E-18	0.23	121	16.46	1231	0.17	99	8.52	3490	0.31	132	25.78	533
ATG4C	1.09	0.027523048	0.2	134	16.46	1232	0.13	125	9.91	2895	0.27	132	22.31	922
CPA6_t1	1.16	0.000362761	0.19	138	16.45	1233	NA	NA	NA	NA	0.17	95	7.64	4772
SLC38A6	1.62	1.47E-15	0.31	103	16.45	1234	0.22	104	11.77	2202	0.28	101	14.17	2553
DERL1	1.33	3.12E-08	0.21	127	16.45	1235	0.08	128	6.27	4633	0.2	133	16.71	1900
LEP	1.08	0.002227111	0.2	134	16.45	1236	0.17	145	16.05	1030	0.1	140	8.76	4317
RAB23	1.26	3.14E-15	0.22	125	16.44	1237	0.23	131	18.9	531	0.35	138	30.59	218
MAP4K4	1.28	2.23E-08	0.18	143	16.44	1238	0.18	142	16.14	1011	0.29	133	24.29	688
KIF20A	2	5.08E-25	0.27	112	16.43	1239	0.2	127	15.26	1209	0.38	127	29.37	273
MOSPD2	1.07	0.000577623	0.16	153	16.43	1240	0.09	127	6.97	4260	0.16	147	15.22	2265
GLRX2	1.57	4.56E-15	0.25	117	16.42	1241	0.2	91	8.26	3616	0.31	112	19.25	1397
RP9_t1	1.44	4.29E-24	0.33	100	16.42	1242	NA	NA	NA	NA	0.39	112	24.08	712
SLC12A7	1.65	3.52E-14	0.23	122	16.42	1243	0.29	137	24.84	85	0.2	147	19.73	1318
ZNF555	1.23	1.63E-08	0.16	150	16.41	1244	0.16	139	14.11	1503	0.2	148	19.85	1293
PDP2	1.07	0.00251196	0.18	140	16.41	1245	-0.05	143	-5	11655	0.18	160	19.83	1299
PHYHIPL	1.58	2.15E-14	0.17	146	16.4	1246	0.1	129	7.89	3795	0.24	129	19.27	1393
ATP5E	1.51	5.09E-16	0.27	111	16.4	1247	0.26	118	17.51	744	0.39	91	16.11	2047
DYDC2	1.4	1.03E-09	0.18	143	16.39	1248	0.09	113	5.88	4855	0.23	126	17.16	1797
UTP18	1.35	1.55E-12	0.2	131	16.39	1249	0.22	101	11.09	2435	0.32	88	12.06	3175
TEX10	1.16	5.80E-05	0.2	131	16.39	1250	0.22	80	6.6	4451	0.18	94	7.96	4637
LSM4	1.55	5.16E-21	0.2	130	16.38	1251	0.21	125	15.94	1061	0.31	130	24.78	640
ERLEC1	1.24	1.17E-07	0.19	136	16.37	1252	0.17	116	11.51	2303	0.16	132	13.37	2775
WIPF2	1.21	1.77E-11	0.19	137	16.37	1253	0.11	139	9.49	3061	0.1	150	10.17	3803
ZNF326_t1	1.03	0.000643723	0.17	148	16.36	1254	0.09	128	7.07	4213	0.18	132	14.43	2480
SNRPF	1.28	4.49E-09	0.23	120	16.35	1255	0.2	97	9.5	3058	0.24	122	17.08	1811
UFC1	1.47	3.02E-17	0.3	104	16.35	1256	0.23	116	15.2	1226	0.32	109	19.05	1439
BOLA3_t1	1.6	1.14E-24	0.33	100	16.35	1257	NA	NA	NA	NA	NA	NA	NA	NA
NR2C2	1.13	7.07E-12	0.18	143	16.34	1258	0.18	150	18.17	629	0.28	134	23.74	760
TP53BP1	1.21	1.98E-08	0.22	125	16.33	1259	0.15	89	6.02	4772	0.19	106	10.88	3558
LMNB2	1.45	2.37E-12	0.23	121	16.32	1260	0.25	158	26.9	40	0.23	135	19.89	1286
CCDC97	1.42	4.85E-14	0.2	130	16.32	1261	0.22	149	21.55	242	0.12	121	8.73	4327
CDCA2	2.47	2.04E-25	0.3	104	16.31	1262	0.23	110	13.83	1585	0.4	109	23.65	768
TACC2	1.56	6.90E-16	0.23	120	16.31	1263	0.09	133	7.14	4173	0.41	135	34.71	86
ME2	1.07	0.115163191	0.18	142	16.3	1264	0.12	124	8.7	3409	0.09	139	7.61	4790
EPC2	1.06	0.221818951	0.16	155	16.29	1265	0.1	135	8.36	3559	0.11	143	9.94	3898
STXBP4	1.44	1.06E-14	0.19	135	16.29	1266	0.24	160	26.28	51	0.26	159	27.91	375
CENPE	1.7	6.31E-22	0.25	116	16.28	1267	0.19	126	14.26	1463	0.34	120	23.61	774
ATP6V1F	1.19	6.72E-07	0.26	113	16.28	1268	0.16	120	11.32	2354	0.15	103	8.16	4557

TOMM34	1.11	9.67E-09	0.24	119	16.28	1269	0.13	108	7.35	4061	0.19	156	20.44	1208
HCN3	1.5	5.24E-16	0.16	151	16.26	1270	0.09	139	7.63	3912	0.22	122	15.95	2085
PDRG1	1.37	9.30E-20	0.21	126	16.24	1271	0.14	128	10.76	2568	0.18	148	17.68	1705
CKAP5_t1	1.45	1.52E-23	0.28	108	16.24	1272	0.25	124	18.33	611	NA	NA	NA	NA
WDR35	1.14	3.71E-09	0.18	140	16.24	1273	0.21	136	18.12	638	0.17	146	16.43	1976
ZNF785	1.21	2.15E-17	0.22	124	16.24	1274	0.24	125	17.92	681	0.26	133	21.32	1070
NME2	1.75	1.65E-19	0.27	111	16.23	1275	0.37	106	20.51	336	0.33	107	18.95	1464
SCAMP1_t1	1.03	0.119477322	0.17	144	16.22	1276	NA	NA	NA	NA	0.25	147	23.82	745
RNF6	1.03	0.631931936	0.16	154	16.22	1277	0.07	111	4.53	5599	0.13	114	8.22	4528
CBL	1.03	0.020021716	0.18	140	16.22	1278	0.19	154	20.08	395	0.12	137	10.31	3755
CDC45	2.89	3.29E-27	0.28	108	16.22	1279	0.23	113	14.45	1412	0.38	105	20.95	1127
NEK7	1.16	1.03E-16	0.21	129	16.22	1280	0.16	141	15.01	1277	0.26	149	25.8	527
DCK	1.3	1.48E-11	0.23	121	16.21	1281	0.22	131	18.02	661	0.33	125	24.47	670
RIF1_t1	1.09	0.000264794	0.17	144	16.21	1282	0.18	139	15.94	1062	NA	NA	NA	NA
RBMX	1.2	3.87E-11	0.2	132	16.21	1283	0.13	126	10.02	2857	0.13	139	11.81	3261
HLA-G	1.46	1.12E-07	0.29	106	16.2	1284	0.18	109	10.39	2719	0.2	104	10.79	3575
KIAA1586	1.17	1.16E-08	0.2	132	16.2	1285	0.16	120	11.17	2412	0.26	150	25.54	553
PDK1	1.25	6.61E-09	0.21	127	16.19	1286	0.19	142	17.68	722	0.12	141	11.37	3396
UBE2E1	1.05	0.113531051	0.19	134	16.19	1287	0.07	114	4.73	5487	0.14	125	10.74	3597
PRCC	1.35	5.13E-18	0.22	124	16.17	1288	0.18	117	12.23	2054	0.31	112	19.17	1416
TYMS	2.52	2.58E-19	0.25	114	16.16	1289	0.18	110	10.6	2630	0.35	120	24.45	675
PSMB4	1.83	1.02E-26	0.3	104	16.15	1290	0.33	100	16.5	942	0.4	145	37.53	47
VRK1	1.4	4.82E-12	0.19	136	16.15	1291	0.22	139	19.86	415	0.25	134	21.14	1094
PLXNA2	1.09	3.64E-06	0.17	145	16.15	1292	0.11	145	10.89	2519	0.22	132	17.69	1703
ATP9A	1.28	0.002645001	0.19	134	16.14	1293	0.27	132	22.35	192	0.13	141	11.94	3220
NUF2	3.57	6.61E-28	0.32	101	16.14	1294	0.27	136	23.5	129	0.43	111	26.21	482
UHRF1	2.06	6.08E-19	0.22	122	16.14	1295	0.23	120	16.04	1033	0.34	125	25.36	576
METTL21A	1.33	2.38E-11	0.21	128	16.14	1296	0.25	135	21.11	278	0.26	140	23.65	769
CAPZA2	1.1	0.121411791	0.16	152	16.14	1297	0.12	116	7.98	3759	0.21	128	16.49	1957
GINS1	1.23	3.44E-14	0.18	138	16.13	1298	0.13	127	9.68	2991	0.23	150	23.35	806
DNER	1.08	0.000647367	0.18	138	16.13	1299	0.09	124	6.43	4554	0.17	122	12.34	3092
ATAT1	1.14	1.07E-11	0.24	118	16.13	1300	0.2	134	16.75	888	0.24	123	17.54	1728
RDBP	1.74	2.57E-23	0.24	118	16.12	1301	0.23	124	16.82	872	0.29	100	14.37	2496
MIS18A	1.4	1.02E-16	0.2	129	16.12	1302	0.17	117	11.49	2310	0.22	110	13.31	2793
PCOLCE2	1.37	0.027911542	0.24	117	16.11	1303	-0.04	108	-2.1	9896	0.21	121	14.66	2416
ZNF404	1.3	9.99E-06	0.21	126	16.11	1304	0.14	111	8.29	3604	0.2	131	16.45	1968
MRPL30	1.14	1.47E-08	0.23	120	16.1	1305	0.1	138	8.71	3406	0.21	143	19.83	1297
ABCF2_t2	1.42	4.66E-19	0.28	108	16.09	1306	0.18	105	9.89	2898	0.28	88	10.61	3652
HAT1	1.2	0.000355633	0.22	123	16.08	1307	0.18	118	12.47	1987	0.19	135	16.06	2056
CCNB2	3.58	1.84E-33	0.32	100	16.08	1308	0.21	108	12.27	2045	0.42	125	31.64	178
CISD1	1.14	0.001138954	0.19	135	16.08	1309	0.06	124	4.19	5782	0.29	144	27.49	397
LIX1L	1.18	1.46E-08	0.19	133	16.08	1310	0.19	116	12.22	2058	0.15	125	11.48	3360
ADAR	1.32	5.25E-21	0.19	135	16.08	1311	0.19	138	16.97	852	0.3	120	20.82	1154
UBE2Z	1.17	1.56E-06	0.22	122	16.07	1312	0.22	120	15.37	1179	0.09	118	6.22	5349
TTC26	1.18	3.80E-14	0.26	112	16.06	1313	0.21	125	15.4	1165	0.34	115	21.9	979
MYO5C	1.57	6.48E-12	0.23	121	16.06	1314	0.12	138	10.88	2524	0.35	126	26.79	432
CAPRIN1_t2	1.15	5.36E-06	0.23	120	16.05	1315	0.18	117	11.76	2206	0.22	112	13.42	2756
SLC52A2	1.5	8.85E-17	0.29	106	16.04	1316	0.2	151	19.96	404	0.22	119	15.16	2288
MAP4K3	1.15	3.72E-06	0.19	135	16.04	1317	0.16	138	13.93	1552	0.27	131	21.94	974
ACTR5	1.3	7.01E-17	0.21	126	16.04	1318	0.09	131	7.42	4025	0.2	125	15.2	2270
USP6	1.21	3.27E-07	0.17	144	16.03	1319	0.1	120	6.88	4314	0.33	151	33.2	114
QSER1	1.17	4.83E-05	0.19	136	16.03	1320	0.21	135	17.84	692	0.27	135	22.59	884
FOXM1	1.88	4.43E-23	0.23	120	16.03	1321	0.18	133	14.95	1294	0.35	118	23.59	778
PPAT	1.31	9.58E-12	0.19	136	16.02	1322	0.22	122	15.67	1111	0.34	130	26.83	431
VPS39	1.16	4.12E-12	0.2	130	16.02	1323	0.17	147	16.35	973	0.18	139	16.37	1987
PHF16	1.09	1.02E-09	0.2	132	16.02	1324	0.04	132	3.37	6266	0.22	127	17.03	1820
LYPD1	2.45	2.09E-13	0.16	153	16.02	1325	0.11	119	7.31	4086	0.25	130	19.88	1288
STXBP6	2.11	3.46E-19	0.22	124	16.01	1326	0.17	131	13.89	1565	0.25	128	19.59	1333
XPO1	1.38	3.10E-08	0.23	121	16	1327	0.23	112	14.32	1448	0.33	123	24.08	711
FAM120A	1.16	5.33E-07	0.17	143	16	1328	0.15	125	11.01	2471	0.17	143	15.98	2075
RPGRIP1L	1.17	1.23E-10	0.19	134	15.99	1329	0.19	114	12.38	2013	0.24	147	23.44	794
HAUS2	1.17	4.88E-08	0.2	131	15.99	1330	0.15	134	12.42	2004	0.24	135	20.79	1161
FAM21A	1.46	2.18E-14	0.21	125	15.99	1331	0.24	115	15.5	1149	0.29	120	20.57	1189
GDAP1	1.11	1.09E-07	0.15	160	15.98	1332	0.11	128	8.84	3358	0.15	140	13.3	2794
TDG	1.22	1.23E-06	0.15	159	15.98	1333	0.27	134	23.01	150	0.09	124	6.5	5237
SERPINI1	1.53	1.11E-12	0.2	129	15.97	1334	0.17	95	7.72	3871	0.34	119	23.2	818
KIF15	1.87	5.82E-18	0.22	122	15.96	1335	0.25	113	15.58	1123	0.39	118	26.76	436
ORAOV1_t1	1.22	9.63E-07	0.2	128	15.96	1336	NA	NA	NA	NA	0.23	142	21.17	1090
EFCAB11	1.37	8.96E-12	0.21	127	15.95	1337	0.13	100	6.65	4421	0.26	99	12.54	3032
ARID4B	1.21	0.000555974	0.21	127	15.94	1338	0.18	113	11.33	2348	0.25	147	24.61	661
CANT1	1.16	1.43E-10	0.2	129	15.93	1339	0.19	121	13.68	1631	0.15	120	10.16	3806
MARS2	1.13	2.07E-07	0.23	120	15.93	1340	0.19	134	16.11	1018	0.2	126	15.02	2333
SUMO2	1.39	5.59E-15	0.29	105	15.93	1341	0.27	132	22.05	214	0.25	135	21.3	1072
FAM21C	1.48	2.61E-13	0.27	110	15.92	1342	0.27	119	18.43	593	0.32	121	22.52	896
UBE2NL	1.15	1.78E-07	0.2	130	15.92	1343	0.14	120	9.87	2910	0.22	149	22.19	937
TPR_t1	1.36	3.15E-06	0.16	148	15.92	1344	NA	NA	NA	NA	NA	NA	NA	NA
DPY30	1.28	2.10E-11	0.21	126	15.92	1345	0.13	104	6.8	4350	0.16	118	11.16	3452
SPINK1	9.67	2.32E-16	0.33	98	15.9	1346	0.07	79	2	7142	0.27	86	9.69	3991
GZF1	1.06	0.019650304	0.16	151	15.9	1347	0.09	118	5.97	4803	0.18	137	15.7	2154
AP5S1	1.26	8.53E-12	0.2	128	15.9	1348	0.13	128	10.21	2790	0.25	109	14.74	2397
ZBED4	1.14	0.000264144	0.17	145	15.88	1349	0.17	132	13.97	1539	0.09	135	7.92	4653
DEM1	1.24	1.72E-09	0.21	127	15.88	1350	0.13	131	10.27	2767	0.23	121	16.15	2039
UBE2B	1.16	1.09E-05	0.19	134	15.88	1351	0.09	139	7.94	3781	0.24	160	26.44	462
ELL3	1.44	4.67E-10	0.22	121	15.87	1352	0.14	120	10.12	2819	0.36	108	20.97	1126
FAM60A	1.12	0.011672847	0.16	152	15.87	1353	0.17	125	12.58	1962	0.04	124	3.33	6758
UHMK1_t1	1.43	3.74E-12	0.16	149	15.87	1354	NA	NA	NA	NA	0.31	143	28.51	331
TBCE	1.71	5.54E-16	0.32	100	15.87	1355	0.23	113	14.68	1357	0.36	103	18.83	1497
MYNN	1.01	0.659504377	0.17	146	15.87	1356	0.01	148	1.16	7702	0.14	151	14.58	2434
TUBA1B	1.85	9.12E-22	0.29	105	15.86	1357	0.26	137	22.78	165	0.33	108	18.92	1470
ASPM_t1	2.38	3.56E-25	0.33	98	15.86	1358	NA	NA	NA	NA	NA	NA	NA	NA
CERS6	1.01	0.728928034	0.15	156	15.86	1359	0.1	134	8.61	3451	0.19	146	17.89	1664
GAS8_t1	1.14	0.0001036	0.25	114	15.85	1360	NA	NA	NA	NA	NA	NA	NA	NA
ASAP3	1.43	5.45E-13	0.23	120	15.85	1361	0.1	122	7.35	4067	0.27	122	19.49	1350
DYNC1H1	1.33	1.50E-12	0.23	119	15.85	1362	0.25	100	12.51	1978	0.19	86	7	5036
B4GALT4	1.21	2.59E-08	0.15	156	15.85	1363	0.16	121	11.5	2308	0.22	115	13.99	2599
DBNDD1	1.16	1.98E-07	0.23	120	15.85	1364	-0.04	140	-3.6	10876	0.13	104	7.09	4996
TGM3	1.42	3.14E-11	0.17	141	15.84	1365	0.06	150	5.6	5005	0.3	127	23.13	832
SMU1	1.12	4.29E-05	0.15	154	15.83	1366	0.1	159	10.43	2708	0.1	133	8.06	4597
PLCB1_t1	1.9	1.56E-14	0.16	150	15.83	1367	0.26	141	23.43	132	0.38	155	39.62	18
ZNF567	1.24	3.54E-06	0.2	129	15.82	1368	0.17	115	11.07	2439	0.19	159	21.05	1112
RAVER2	1.18	0.000636574	0.19	132	15.81	1369	0.06	130	4.64	5527	0.07	147	6.44	5261
AZI1	2.14	2.10E-30	0.32	99	15.8	1370	0.28	105	15.15	1235	0.37	104	20.19	1251
RPL30_t1	1.48	1.47E-11	0.26	111	15.8	1371	NA	NA	NA	NA	NA	NA	NA	NA
FAM126A_t1	1.16	2.55E-07	0.18	136	15.79	1372	NA	NA	NA	NA	0.2	148	19.97	1274
ZNF273_t1	1.23	1.11E-09	0.18	140	15.79	1373	0.35	135	29.64	11	NA	NA	NA	NA
R3HDM1	1.37	1.85E-15	0.23	120	15.78	1374	0.31	129	24.19	107	0.31	150	30.71	212

ZNF43	1.15	0.003024292	0.2	130	15.77	1375	0.21	143	19.19	489	0.16	157	17.33	1762
ABCB10_t1	1.12	1.03E-06	0.18	136	15.76	1376	NA	NA	NA	NA	0.27	140	23.88	738
RBM22_t1	1.14	1.75E-09	0.2	130	15.76	1377	NA	NA	NA	NA	0.22	108	12.6	3005
WHSC1L1_t1	1.05	0.060963278	0.2	128	15.75	1378	0.05	132	3.72	6048	0.13	157	13.65	2691
FAM54A	2.06	3.95E-23	0.27	108	15.75	1379	0.3	122	21.77	232	0.36	122	25.83	523
SHC3	1.47	8.51E-11	0.18	138	15.75	1380	0.18	141	16.7	898	0.12	148	12.03	3186
KCNE3	1.37	1.91E-08	0.18	136	15.75	1381	0.23	135	19.89	411	0.17	120	11.74	3288
NUAK1	1.21	0.000114489	0.2	130	15.74	1382	0.16	130	12.72	1919	0.11	139	9.7	3984
WSB2	1.32	9.53E-10	0.18	139	15.74	1383	0.2	150	20.18	379	0.19	133	15.63	2168
CDCA7_t1	1.27	1.89E-08	0.18	138	15.73	1384	NA	NA	NA	NA	0.14	132	11.65	3310
EP400_t1	1.23	3.31E-09	0.17	144	15.72	1385	NA	NA	NA	NA	NA	NA	NA	NA
EIF5B	1.15	0.000547928	0.17	144	15.71	1386	0.18	135	15.1	1248	0.23	135	19.77	1308
RBM28	1.32	2.92E-16	0.29	105	15.7	1387	0.2	155	21.05	289	0.34	104	18.47	1556
ANKRD23	1.32	2.10E-12	0.21	125	15.7	1388	0.2	135	17.02	837	0.22	132	18.26	1601
KIF20B	1.19	4.69E-11	0.21	124	15.69	1389	0.19	116	12.73	1917	0.26	122	19.02	1447
SRSF2	1.22	4.40E-08	0.18	137	15.69	1390	0.17	117	11.65	2256	0.16	148	15.45	2203
STK4	1.05	0.004092543	0.17	143	15.69	1391	0.16	136	13.94	1549	0.02	170	2.77	7042
TEX261	1.1	1.33E-05	0.17	144	15.68	1392	0.01	133	1.22	7666	0.11	167	12.29	3111
DAPK2	1.67	6.29E-15	0.18	136	15.68	1393	0.08	135	6.62	4437	0.27	120	19.03	1445
MRRF_t1	1.26	5.07E-10	0.2	127	15.68	1394	0.13	128	10.15	2805	0.22	148	21.25	1078
MED27	1.16	2.13E-08	0.24	115	15.68	1395	0.17	117	11.43	2326	0.06	94	2.54	7162
ZNF563_t1	1.34	1.86E-11	0.18	135	15.67	1396	0.1	131	8.38	3553	NA	NA	NA	NA
TNFRSF11A	1.15	3.55E-06	0.21	126	15.67	1397	0.11	127	8.75	3391	0.18	146	16.94	1842
RINT1	1.38	1.34E-12	0.23	119	15.67	1398	0.21	123	15.24	1214	0.38	109	22.45	905
E2F7	1.58	7.60E-16	0.16	148	15.66	1399	0.08	129	6.61	4440	0.3	157	32.42	146
FAM136A	1.27	5.13E-10	0.15	152	15.66	1400	0.24	120	17	840	0.19	116	12.53	3037
MELK	2.5	9.33E-29	0.31	100	15.66	1401	0.24	104	12.79	1901	0.45	98	21.79	1006
POLR3C	1.34	2.52E-12	0.22	122	15.66	1402	0.13	81	3.94	5932	0.27	108	15.61	2179
CTSL2	2.07	3.55E-12	0.16	149	15.65	1403	0.26	127	20.31	365	0.32	124	23.34	808
KIAA1715	1.16	2.41E-06	0.17	140	15.64	1404	0.13	126	9.71	2972	0.24	159	25.84	521
FZD3	1.11	0.003098431	0.17	140	15.64	1405	0.09	142	8.43	3534	0.11	150	10.91	3544
HSF2_t1	1.14	1.72E-10	0.24	114	15.63	1406	NA	NA	NA	NA	0.25	127	18.99	1455
TSR1	1.13	7.85E-06	0.22	120	15.63	1407	0.07	128	5.69	4955	0.22	129	17.46	1742
TUBG2	1.48	1.89E-22	0.23	118	15.62	1408	0.14	112	8.69	3416	0.36	96	16.73	1895
DNAH5_t1	1.06	8.16E-05	0.19	132	15.62	1409	NA	NA	NA	NA	0.23	152	22.95	849
IPP	1.22	3.40E-12	0.19	132	15.61	1410	0.16	131	12.94	1854	0.25	121	17.74	1691
CCT7	1.25	6.92E-13	0.19	134	15.61	1411	0.2	114	12.64	1946	0.23	120	15.89	2103
BAG2	1.68	2.45E-13	0.22	121	15.61	1412	0.19	115	12.58	1961	0.36	132	29.5	266
KITLG_t1	1.07	0.001161437	0.15	151	15.61	1413	NA	NA	NA	NA	NA	NA	NA	NA
RPLP0	1.48	1.58E-12	0.2	128	15.6	1414	0.22	88	8.45	3528	0.29	118	19.77	1310
AHCTF1_t1	1.2	1.62E-13	0.17	142	15.59	1415	NA	NA	NA	NA	0.26	139	22.96	847
EIF2C2	1.33	1.29E-10	0.21	125	15.59	1416	0.21	133	17.03	835	0.26	126	19.63	1327
KCNA1_t1	1.02	0.018152409	0.15	151	15.59	1417	NA	NA	NA	NA	0.17	152	17.01	1823
GJC1	1.12	5.80E-10	0.18	136	15.59	1418	0.17	143	15.46	1153	0.22	157	23.02	841
ZBTB44_t1	1.04	0.092313105	0.16	150	15.59	1419	NA	NA	NA	NA	0.09	155	8.95	4244
PPP1R16A	1.6	1.30E-10	0.25	112	15.59	1420	0.16	102	8.56	3468	0.33	95	14.97	2344
CHCHD3_t1	1.35	5.66E-14	0.25	113	15.58	1421	NA	NA	NA	NA	0.31	157	33.08	121
UBA52	1.3	1.18E-12	0.25	113	15.57	1422	0.24	132	19.83	419	0.3	147	28.84	315
P4HA2_t1	2	1.29E-15	0.26	111	15.57	1423	0.19	126	14.82	1324	0.33	109	19.69	1321
MPHOSPH6	1.19	1.91E-05	0.2	128	15.57	1424	0.1	125	7.21	4131	0.21	129	16.55	1941
PIGT_t1	1.59	4.13E-24	0.29	104	15.57	1425	0.19	99	9.26	3161	NA	NA	NA	NA
DHFRL1	1.19	2.35E-05	0.17	144	15.56	1426	0.04	134	3.55	6143	0.19	136	16.7	1905
CDC25C	2.3	1.57E-28	0.31	100	15.56	1427	0.2	126	15.44	1158	0.42	126	31.8	169
ADAMTS15	1.18	6.30E-20	0.22	122	15.55	1428	0.09	137	8.07	3703	0.28	140	25.08	611
LPPR4	1.24	5.24E-10	0.16	147	15.55	1429	0.1	142	9.48	3078	0.28	134	23.53	787
UBE2S_t1	1.66	1.19E-28	0.24	115	15.55	1430	NA	NA	NA	NA	NA	NA	NA	NA
EIF2S1	1.17	0.000526391	0.15	151	15.54	1431	0.02	129	1.47	7513	0.16	131	12.88	2931
DHX37	1.37	2.49E-20	0.22	121	15.54	1432	0.27	99	13.12	1796	0.23	122	16.61	1928
RRS1	1.82	1.19E-11	0.19	132	15.53	1433	0.19	103	10.33	2745	0.28	120	19.89	1285
SHC1_t1	1.6	1.36E-16	0.23	118	15.52	1434	0.29	127	22.43	188	0.23	118	15.31	2245
CDK5	1.55	7.00E-22	0.25	113	15.52	1435	0.11	132	9.4	3110	0.28	104	15.31	2244
BCAS4	1.21	2.38E-08	0.24	116	15.52	1436	0.25	131	20.42	348	0.21	124	15.24	2262
COL15A1	1.98	1.47E-17	0.26	109	15.51	1437	0.29	131	23.12	143	0.31	111	18.85	1488
BORA	1.48	1.18E-17	0.23	118	15.51	1438	0.3	121	21.09	282	0.3	117	19.8	1305
HSBP1	1.14	0.000451346	0.22	120	15.51	1439	0.13	123	9.24	3168	0.12	120	8.22	4530
MYB	1.3	8.88E-07	0.2	128	15.51	1440	0.18	131	14.56	1392	0.18	147	17.14	1803
PFKFB2_t1	1.21	4.18E-07	0.22	121	15.49	1441	0.18	132	14.81	1325	0.27	125	20.52	1195
HES6	1.32	8.24E-14	0.22	120	15.48	1442	0.08	117	5.43	5099	0.31	120	21.61	1034
UBA2	1.23	1.87E-05	0.22	120	15.48	1443	0.21	145	20.13	390	0.24	128	18.51	1551
PSMA6	1.44	9.45E-11	0.27	108	15.47	1444	0.23	120	16.36	971	0.32	87	11.79	3266
EFNA4	1.86	1.54E-27	0.26	109	15.47	1445	0.25	134	20.67	327	0.36	123	26.46	460
MIS18BP1	1.11	0.017236362	0.2	126	15.47	1446	0.16	127	12.18	2072	0.14	124	10.61	3648
DENND1B	1.13	2.88E-06	0.2	129	15.47	1447	0.12	116	8.04	3723	0.18	150	18.06	1633
SNRPC_t1	1.46	2.41E-23	0.27	108	15.46	1448	NA	NA	NA	NA	NA	NA	NA	NA
IMPA1	1.28	0.000543896	0.17	141	15.46	1449	0.05	125	3.59	6121	0.29	140	25.93	514
FGF12_t1	1.6	1.38E-08	0.16	145	15.45	1450	NA	NA	NA	NA	NA	NA	NA	NA
CDKL3	1.19	1.43E-11	0.22	120	15.44	1451	0.16	125	12.12	2094	0.34	91	14.11	2567
RFESD	1.22	1.20E-09	0.18	136	15.44	1452	0.02	116	1.45	7518	0.32	105	17.39	1752
ARPC1A	1.46	3.78E-13	0.23	117	15.43	1453	0.19	130	15.14	1240	0.27	120	19.22	1405
ARHGAP11A_t1	1.23	4.44E-16	0.23	117	15.43	1454	0.19	118	13.19	1776	0.29	103	15.16	2285
RPL36A	1.56	9.17E-15	0.17	142	15.43	1455	0.27	120	19.15	491	0.28	132	22.93	851
LRRC14	1.75	1.53E-21	0.23	116	15.42	1457	0.22	147	21.09	284	0.24	121	17.27	1773
ZNF777	1.22	9.29E-09	0.18	137	15.42	1458	0.09	100	4.33	5697	0.19	87	7.2	4950
ZNF208	1.12	7.08E-05	0.19	131	15.4	1459	0.22	130	17.24	790	0.15	134	12.66	2990
BLZF1	1.19	1.46E-07	0.2	128	15.4	1460	0.19	131	15.13	1243	0.24	121	16.8	1876
KLHL7	1.33	1.05E-11	0.21	125	15.4	1461	0.2	119	13.57	1668	0.44	128	34.11	98
ERMP1	1.16	1.11E-06	0.2	127	15.39	1462	0.03	134	2.42	6880	0.26	156	27.91	374
UBE3D	1.29	1.69E-17	0.2	128	15.39	1463	0.23	114	14.43	1418	0.3	140	26.59	445
USP37	1.09	0.056020597	0.16	148	15.39	1464	0.11	138	9.53	3043	0.16	152	16.46	1967
SLC1A3_t1	1.48	1.77E-07	0.19	129	15.39	1465	0.23	124	17.14	811	NA	NA	NA	NA
PRDM10	1.1	0.003899419	0.19	129	15.38	1466	0.17	133	13.84	1584	0.16	120	11.53	3342
INPP4A	1.08	0.000379714	0.17	140	15.38	1467	0.22	152	22.69	172	0.03	146	3.19	6835
TROAP	1.91	4.43E-25	0.21	124	15.38	1468	0.17	120	12.1	2101	0.26	95	11.9	3236
PLCB1_t2	1.58	2.61E-15	0.15	150	15.37	1469	0.23	141	21.26	261	0.35	155	36.64	57
VGLL4	1.19	2.66E-07	0.16	148	15.37	1470	0.17	140	15.11	1245	0.2	149	19.63	1328
PKD1L1	1.07	0.003037482	0.22	121	15.36	1471	0.23	124	16.91	859	0.07	123	4.85	6003
COL4A1	1.61	4.02E-08	0.19	130	15.35	1472	0.26	117	17.27	787	0.2	130	15.82	2126
TMEM52B	1.06	1.32E-08	0.19	132	15.35	1473	0.13	129	10.34	2741	0.05	123	3.34	6752
RAB7L1	1.22	3.28E-09	0.19	131	15.33	1474	0.1	121	7.34	4074	0.18	146	16.93	1844
TOR1B	1.16	0.000271373	0.19	131	15.33	1475	0.05	123	3.8	6005	0.13	127	10.35	3742
CKLF_t2	1.58	5.48E-19	0.28	104	15.33	1476	0.19	103	10.28	2764	0.25	100	12.28	3114
MRAP2	1.98	4.32E-14	0.21	123	15.32	1477	0.21	130	17.15	808	0.28	122	19.95	1281
GPRIN1	1.3	7.05E-19	0.24	115	15.32	1478	0.12	117	8.29	3599	0.28	107	16.24	2022
PSMB3	1.31	9.72E-14	0.26	108	15.31	1479	0.11	88	4.3	5716	0.3	83	9.95	3895
E2F3	1.47	2.89E-11	0.18	134	15.31	1480	0.22	127	16.96	853	0.23	129	17.98	1652
WDR55	1.17	4.21E-09	0.2	126	15.3	1481	0.11	119	7.78	3841	0.22	120	15.27	2257

PCDHA9	1.04	0.000908905	0.15	155	15.3	1482	0.12	137	10.69	2599	0.07	136	5.7	5581
UHRF1BP1	1.06	3.24E-05	0.15	151	15.3	1483	0.22	144	20.58	331	0.19	150	18.98	1458
PIGN	1.09	6.56E-05	0.22	119	15.29	1484	0.03	117	1.93	7189	0.2	114	13	2880
MTMR2	1.15	0.011728203	0.19	131	15.28	1485	0.25	150	25.02	79	0.12	135	10.25	3780
PTPRG	1.28	1.86E-08	0.22	120	15.28	1486	0.19	152	19.55	450	0.29	154	30.55	220
KIF23	1.62	1.34E-17	0.19	132	15.27	1487	0.15	124	11.16	2415	0.29	120	20.42	1210
LYSMD3	1.06	0.000811873	0.17	141	15.27	1488	0.09	138	8.31	3586	0.26	152	26.28	474
FOXP2_t1	1.06	5.42E-05	0.16	143	15.27	1489	0.05	160	5.47	5077	0.09	143	8.61	4378
CAMLG	1.23	9.28E-16	0.22	118	15.26	1490	0.27	129	21.39	252	0.28	133	23.39	802
RTN3_t1	1.28	2.23E-13	0.2	126	15.26	1491	0.16	132	13.14	1789	0.14	137	12.46	3045
LYPLA1_t1	1.15	0.022951162	0.17	142	15.26	1492	NA	NA	NA	NA	0.3	135	25.17	603
ZNF498_t1	1.18	2.47E-17	0.15	149	15.26	1493	NA	NA	NA	NA	0.22	156	23	846
TFRC	1.49	2.35E-06	0.19	132	15.26	1494	0.05	152	4.74	5481	0.29	137	25.06	614
DCUN1D5	1.19	1.38E-05	0.15	154	15.24	1495	0.17	141	15.83	1086	0.1	150	9.58	4031
NXT2	1.38	2.12E-08	0.17	138	15.24	1496	0.11	142	10.39	2721	0.32	141	29.22	283
POPDC3	1.25	9.75E-06	0.16	148	15.23	1497	0.08	116	5.18	5225	0.21	126	15.78	2137
WFS1	1.36	1.90E-15	0.24	114	15.23	1498	0.12	122	8.72	3402	0.25	116	16.8	1874
MOB4_t1	1.12	0.011578362	0.18	137	15.23	1499	0.22	142	19.85	416	NA	NA	NA	NA
CUL4B	1.21	2.93E-06	0.15	150	15.23	1500	0.16	122	11.3	2363	0.33	129	26	502
MYPN	1.02	0.017670822	0.2	125	15.22	1501	0.1	127	8.07	3704	0.23	142	20.81	1156
LLPH	1.13	0.000268445	0.18	134	15.22	1502	0.12	127	9.39	3111	0.23	120	15.86	2112
ANP32E	1.28	1.49E-06	0.19	132	15.22	1503	0.23	130	18.78	548	0.27	124	20.29	1231
JAZF1	1.38	6.44E-07	0.2	126	15.21	1504	0.08	126	6.09	4730	0.26	138	23.06	840
AKR1B1	2.72	2.42E-10	0.2	127	15.21	1505	0.18	100	9.12	3219	0.08	123	6.2	5358
PAQR3_t1	1.04	0.0039349	0.18	136	15.21	1506	0.04	127	2.93	6533	NA	NA	NA	NA
CLCN2	1.21	1.98E-10	0.21	124	15.2	1507	0.07	105	3.73	6044	0.24	100	12.02	3187
ITGAE	1.1	0.061563211	0.21	124	15.19	1508	0.08	117	5.39	5123	0.1	126	7.35	4896
TMEM183B	1.34	4.30E-17	0.27	106	15.19	1509	0.13	114	8.54	3485	0.28	131	22.74	873
GOSR2_t1	1.39	5.24E-16	0.17	139	15.19	1510	0.17	151	17.28	785	0.19	132	15.52	2194
TMCO3_t1	1.65	9.88E-10	0.21	124	15.19	1511	0.26	118	18	664	NA	NA	NA	NA
PLXNC1	1.1	7.99E-07	0.18	136	15.19	1512	0.18	134	15.28	1201	0.2	127	15.16	2290
TPM3_t3	1.24	9.60E-08	0.12	175	15.18	1513	0.21	143	19.13	493	0.17	129	13.17	2836
ENTPD6	1.34	2.85E-17	0.18	135	15.18	1514	0.12	143	11.34	2345	0.16	116	10.54	3674
METTL4	1.14	8.30E-11	0.16	147	15.18	1515	0.07	114	4.66	5514	0.23	150	23.36	805
KIF14	1.27	6.85E-14	0.19	129	15.17	1516	0.2	122	14.08	1512	0.3	140	26.73	437
ATG3	1.1	0.001833792	0.17	137	15.17	1517	0.1	116	6.36	4580	0.12	115	7.55	4819
UBB	1.27	5.96E-10	0.27	107	15.17	1518	0.1	98	4.56	5580	0.34	120	23.73	761
SHOX2_t1	1.13	2.71E-08	0.18	136	15.17	1519	NA	NA	NA	NA	0.18	152	18.07	1631
SATB2	1.41	3.78E-14	0.19	131	15.17	1520	0.12	137	10.55	2652	0.36	163	41.11	13
ZNHIT6	1.07	0.059066496	0.15	149	15.16	1521	0.2	142	18.53	579	0.12	142	10.93	3536
TAF6	1.88	1.51E-29	0.28	105	15.15	1522	0.33	106	18.64	566	0.36	138	32.02	160
GLDN	1.46	4.30E-08	0.14	155	15.15	1523	0.2	138	17.45	757	0.21	126	15.67	2161
PRRT3	1.33	6.30E-12	0.18	136	15.14	1524	0.08	143	7.12	4184	0.23	150	23.12	833
ASRGL1	1.65	2.60E-13	0.14	160	15.14	1525	0.14	131	11.24	2385	0.16	147	15.09	2306
GLE1	1.05	0.000943505	0.16	146	15.13	1526	0.04	116	2.8	6614	0	104	0.04	8595
DDX41	1.36	5.72E-19	0.27	107	15.13	1527	0.09	100	4.66	5516	0.31	94	13.58	2715
SRD5A3	1.35	2.82E-09	0.17	140	15.13	1528	0.03	148	2.9	6556	0.19	134	15.58	2184
ALKBH6	1.3	4.32E-17	0.22	119	15.13	1529	0.12	103	6.62	4436	0.28	87	10.37	3734
NUDT15	-1.04	0.210748958	0.15	148	15.12	1530	0.08	118	5.37	5137	0.02	122	1.2	7914
ZSCAN20	1.38	1.04E-12	0.2	126	15.11	1531	0.18	120	12.28	2040	0.26	123	18.93	1467
PEG10	2.75	8.04E-14	0.15	150	15.11	1532	0.05	163	5.59	5015	0.19	150	19.29	1388
CNTNAP4_t2	1.3	1.84E-10	0.16	145	15.11	1533	0.13	115	8.34	3574	0.19	122	13.38	2768
PAPOLA	1.16	1.87E-07	0.2	126	15.1	1534	0.18	154	18.97	523	0.32	135	27.09	415
EDIL3	1.14	0.000202158	0.19	130	15.09	1535	0.22	126	16.53	937	0.21	127	16.23	2024
PABPC1_t1	1.46	4.64E-08	0.17	138	15.09	1536	0.29	110	17.48	750	NA	NA	NA	NA
BPTF_t1	1.22	1.32E-05	0.17	138	15.09	1537	0.15	123	10.73	2580	0.25	148	24.1	707
LCOR_t1	-1	0.971969127	0.15	148	15.09	1538	NA	NA	NA	NA	0.12	153	12.75	2962
CDC42_t1	1.17	2.42E-05	0.19	131	15.08	1539	0.19	143	17.95	674	0.18	118	12.03	3182
MPP4	1.05	0.000304964	0.19	129	15.08	1540	0.05	111	3.25	6344	0.13	112	8.32	4485
IGF2BP1_t1	1.95	4.53E-10	0.18	132	15.08	1541	NA	NA	NA	NA	0.23	143	21.24	1080
GAB2	1.04	8.59E-07	0.18	134	15.07	1542	0.07	122	4.72	5492	0.11	122	7.74	4737
RNASEH2B	1.24	4.70E-08	0.22	120	15.07	1543	0.24	110	14.46	1410	0.06	134	4.78	6039
SMG8	1.23	4.23E-11	0.15	151	15.06	1544	0.11	96	4.92	5382	0.16	104	8.56	4401
ZNF16	1.26	7.52E-11	0.21	123	15.06	1545	0.08	124	5.94	4821	0.17	105	9.14	4171
BCAP31	1.58	4.89E-17	0.22	119	15.06	1546	0.27	148	26.08	57	0.27	139	24.29	689
TMEM106C	2.02	2.65E-21	0.28	104	15.05	1547	0.16	117	10.7	2593	0.36	119	25.16	604
NRF1	1.08	9.11E-08	0.17	136	15.05	1548	0.19	117	12.59	1956	0.14	148	13.79	2650
NAPG	1.18	9.60E-05	0.17	137	15.05	1549	0.09	143	8.04	3721	0.14	137	11.97	3211
CDT1	3	2.89E-26	0.3	100	15.03	1550	0.19	150	18.83	546	0.38	110	22.74	871
KDM3A_t1	1.13	0.000300231	0.23	115	15.02	1551	NA	NA	NA	NA	NA	NA	NA	NA
ITGB8_t1	1.01	0.729816566	0.15	150	15.02	1552	NA	NA	NA	NA	0.01	152	1.46	7782
RPS10	1.62	4.40E-17	0.31	98	15.02	1553	0.29	86	10.35	2737	0.34	124	25.39	575
ZNF236	1.02	0.328527535	0.16	145	15.02	1554	0.1	124	7.11	4193	0.1	130	8.15	4558
TATDN3	1.32	3.05E-11	0.23	114	15.01	1555	0.1	131	8	3746	0.32	139	28.14	363
POLI	-1	0.962030989	0.18	134	15	1556	0.15	138	12.98	1838	0.12	132	9.7	3983
LAPTM4B	1.93	1.49E-09	0.17	136	14.99	1557	0.24	128	18.35	606	0.24	124	18.09	1625
CREM_t1	1.16	1.22E-05	0.19	129	14.99	1558	0.05	119	3.59	6120	0.22	133	18.6	1536
RRP9	1.37	3.20E-19	0.22	118	14.98	1559	0.19	107	10.94	2494	0.25	112	15.23	2263
TAB3	1.24	6.40E-09	0.15	148	14.98	1560	0.18	154	18.93	527	0.26	140	23.79	751
LACC1	1.07	6.28E-06	0.16	141	14.98	1561	0.12	124	8.83	3367	0.17	128	12.95	2904
MTA3	1.2	2.45E-09	0.26	108	14.97	1562	0.24	97	11.29	2370	0.25	128	19.34	1378
OTX1	1.54	1.58E-14	0.18	133	14.97	1563	0.11	119	7.46	4007	0.24	120	16.82	1867
ELMOD2	1.19	9.78E-08	0.15	148	14.96	1564	0.16	130	12.9	1864	0.25	148	24.47	672
YME1L1	1.06	0.165897668	0.15	149	14.95	1565	0.07	148	7.05	4225	0.21	129	16.98	1834
RGS9BP	1.08	1.58E-06	0.2	123	14.94	1566	0.11	119	7.88	3801	0.24	126	18.13	1617
ARL17B	1.36	1.53E-21	0.2	123	14.93	1567	0.21	105	11.63	2266	0.25	100	12.58	3011
NCOA3	1.06	0.016565054	0.17	140	14.93	1568	0.09	129	7.4	4037	0.12	139	10.36	3738
DDX39A	1.72	1.34E-24	0.3	100	14.92	1569	0.29	89	11.31	2359	0.29	98	14.07	2578
SEMA4F	1.31	6.17E-11	0.16	142	14.92	1570	0.19	135	15.83	1085	0.18	128	14.34	2511
TRIP13	1.29	8.77E-15	0.25	110	14.91	1571	0.19	144	18.2	622	0.37	121	25.98	507
SOX11_t1	1.24	2.34E-06	0.19	129	14.91	1572	NA	NA	NA	NA	0.25	151	25.57	550
MCTS1	1.26	7.71E-07	0.16	144	14.91	1573	0.13	140	11.5	2307	0.25	142	23.23	816
TRMT13	1.29	1.09E-06	0.21	121	14.91	1574	0.26	113	16.17	1005	0.27	116	18.04	1641
IRAK1	1.42	8.06E-15	0.2	124	14.9	1575	0.06	133	5.04	5309	0.22	124	15.94	2088
FAM64A	1.68	9.32E-12	0.18	135	14.9	1576	0.3	124	22	221	0.2	124	14.58	2438
KCTD10_t1	1.05	0.325130464	0.14	158	14.9	1577	NA	NA	NA	NA	-0.03	152	-2.63	10007
IFT81_t1	1.25	2.32E-11	0.15	148	14.9	1578	0.17	108	9.96	2874	0.25	110	15.16	2289
RBM34_t1	1.39	7.29E-12	0.25	109	14.89	1579	NA	NA	NA	NA	0.34	115	21.94	975
FAM20B	1.49	1.01E-10	0.2	126	14.88	1580	0.16	143	14.79	1330	0.26	144	24.73	645
HOXA9	1.18	6.06E-06	0.15	148	14.87	1581	0.04	112	2.62	6739	0.17	128	13.46	2750
ZNF431_t1	1.03	0.006970639	0.17	140	14.86	1582	NA	NA	NA	NA	0.16	132	13.28	2800
UBE2M	1.12	0.000669862	0.21	122	14.85	1583	0.03	104	1.76	7311	0.14	98	6.5	5234
FAM114A2	1.11	7.59E-05	0.16	140	14.85	1584	0.1	140	9.13	3217	0.25	122	18.3	1593
PRDM12	1.17	2.06E-13	0.17	139	14.85	1585	0.14	145	13.17	1781	0.25	125	18.55	1542
MPV17	1.2	7.03E-07	0.25	110	14.85	1586	0.21	130	16.64	911	0.1	127	7.78	4720
CYSTM1	1.43	4.25E-11	0.21	120	14.84	1587	0.13	112	8.25	3624	0.28	133	23.06	838

ATG12_t1	1.34	1.35E-10	0.19	130	14.84	1588	0.18	127	14.12	1502	NA	NA	NA	NA
PRKCI	1.26	2.80E-09	0.19	129	14.84	1589	0.18	126	14.01	1526	0.2	128	15.66	2164
CDKN2C	1.97	8.26E-25	0.23	115	14.83	1590	0.19	114	12.09	2105	0.27	110	16.44	1970
IQCG	1.18	5.00E-13	0.21	120	14.83	1591	0.15	112	9.21	3183	0.25	127	19.48	1352
FAM76A	1.04	0.000162017	0.17	137	14.82	1592	0.07	141	6.08	4734	0.15	140	13.54	2728
TGFBR1	1.08	1.59E-08	0.15	152	14.82	1593	0.17	127	13.2	1772	0.2	161	22.39	910
GTPBP3	1.14	2.87E-05	0.18	132	14.82	1594	0.11	109	6.64	4426	0.07	124	5.37	5750
ZNF512	1.2	1.03E-11	0.18	131	14.82	1595	0.16	135	13.89	1564	0.18	129	13.98	2601
TMEM101	1.46	1.05E-16	0.21	122	14.81	1596	0.17	127	12.98	1840	0.25	120	17.18	1792
GLMN	1.27	7.31E-14	0.19	126	14.81	1597	0.2	105	11.07	2443	0.28	108	16.2	2035
IMMP1L	1.3	1.78E-13	0.19	126	14.81	1598	0.14	104	7.38	4045	0.21	115	13.74	2664
PPP1R35	1.55	1.25E-22	0.22	116	14.8	1599	0.23	84	7.76	3853	0.32	99	15.8	2132
RIT1	1.44	1.17E-12	0.22	117	14.8	1600	0.22	130	17.6	733	0.3	146	28.98	303
ZNF232	1.26	7.91E-11	0.25	109	14.79	1601	0.05	88	1.91	7213	0.26	84	8.84	4283
RAP2A	1.47	3.71E-09	0.2	124	14.79	1602	0.25	118	17.12	816	0.32	139	28.44	340
SLC9A3R1	1.49	7.12E-12	0.19	129	14.79	1603	0.22	127	17.21	800	0.27	112	16.6	1931
DNM1L_t2	1.14	0.004379078	0.17	136	14.79	1604	0.26	135	22.29	195	0.2	153	20.87	1144
DRD5_t1	1.07	0.008592242	0.2	125	14.76	1605	NA	NA	NA	NA	0.16	117	10.5	3686
SF3A1_t1	1.09	0.015352319	0.14	153	14.76	1606	NA	NA	NA	NA	0.14	131	10.99	3515
CCDC117	1.12	7.65E-08	0.2	122	14.76	1607	0.09	124	6.95	4274	0.22	123	16.32	2005
ONECUT2_t1	1.49	1.71E-05	0.15	151	14.76	1608	NA	NA	NA	NA	0.25	153	25.98	505
CEP72	1.72	1.08E-20	0.26	107	14.75	1609	0.23	114	14.43	1419	0.34	128	26.67	442
HBXIP	1.26	9.47E-12	0.23	113	14.75	1610	0.2	108	11.52	2297	0.24	113	15.37	2227
UBQLN2	1.19	5.14E-07	0.18	134	14.74	1611	0.08	127	5.91	4837	0.24	138	21.06	1109
ODF2_t1	1.28	3.03E-12	0.24	112	14.74	1612	0.22	98	10.52	2670	0.24	100	11.97	3210
GNA12	1.43	3.88E-17	0.2	124	14.74	1613	0.31	128	23.93	116	0.24	139	21.19	1088
CCL13_t1	1.27	1.08E-06	0.15	146	14.73	1614	0.15	121	10.46	2699	NA	NA	NA	NA
TTC33	1.25	8.75E-08	0.15	148	14.73	1615	0.14	134	11.68	2241	0.27	136	23.01	843
SPRTN_t2	1.23	1.00E-15	0.22	117	14.72	1616	0.1	156	10.59	2632	0.31	131	25.25	590
GNAZ	2.95	4.89E-14	0.2	124	14.72	1617	0.21	120	14.59	1379	0.26	114	16.49	1960
CCT2	1.35	6.56E-08	0.18	134	14.71	1618	0.22	108	12.59	1957	0.19	104	10.29	3765
ZNF569	1.13	9.52E-10	0.2	123	14.71	1619	0.14	132	11.21	2395	0.22	130	17.8	1684
PIAS3	1.43	5.58E-17	0.2	125	14.71	1620	0.27	136	22.82	161	0.26	113	16.26	2013
SLMAP	1.19	0.000338195	0.17	138	14.71	1621	0.15	132	12.44	1997	0.29	149	29.09	292
HOXA10	1.4	6.65E-10	0.18	131	14.7	1622	0.11	126	8.13	3679	0.25	134	21.4	1063
TMEM81	1.14	3.31E-09	0.2	124	14.7	1623	0.11	140	10.34	2742	0.25	136	21.44	1056
NIF3L1	1.2	1.81E-10	0.21	119	14.7	1624	0.26	127	19.75	425	0.22	104	11.62	3320
TAP1	1.36	4.04E-06	0.14	153	14.69	1625	0.2	134	16.46	948	0.07	104	3.56	6633
ATG5	1.01	0.787061816	0.16	140	14.68	1626	0.09	130	7.44	4014	0.1	137	9.12	4181
MYH8	1.03	0.009142918	0.2	123	14.68	1627	0.06	108	3.3	6316	0.33	109	19.25	1398
BRD7	1.13	0.008883371	0.17	138	14.67	1628	0.2	112	12.64	1947	0.17	117	11.66	3306
UBQLN1_t1	1.06	0.000544388	0.19	126	14.67	1629	NA	NA	NA	NA	NA	NA	NA	NA
KSR1_t1	1.02	0.059494879	0.17	136	14.67	1630	0.12	120	8.08	3699	NA	NA	NA	NA
VPS35_t1	1.1	0.028224988	0.19	128	14.65	1631	NA	NA	NA	NA	0.18	133	15.28	2252
NONO	1.29	5.08E-18	0.22	118	14.65	1632	0.19	152	19.24	483	0.21	130	16.78	1881
CAMSAP2	1.18	0.000130989	0.17	134	14.65	1633	0.21	118	14.35	1437	0.29	151	28.81	316
HIST1H2BE	1.81	3.29E-19	0.29	100	14.64	1634	0.11	100	5.33	5154	0.3	84	10.11	3832
DTX3L	1.04	0.082022176	0.16	141	14.64	1635	0.02	127	1.82	7265	0.1	148	9.44	4072
CKLF_t3	1.43	7.19E-13	0.22	116	14.64	1636	0.15	103	8.19	3649	0.19	112	11.88	3243
TONSL	1.52	4.64E-20	0.3	99	14.63	1637	0.18	135	15.03	1269	0.33	120	23.15	826
RNF214	1.32	7.49E-16	0.29	100	14.63	1638	0.32	112	19.62	443	0.29	117	19.33	1380
HOXC9	1.44	2.47E-06	0.16	142	14.63	1639	0.12	103	6.17	4682	0.25	108	14.32	2518
RNF7	1.12	6.35E-05	0.17	138	14.61	1640	0.22	127	16.81	879	0.06	120	4.23	6329
ANKRD40	1.1	4.06E-07	0.15	148	14.61	1641	0.08	135	6.86	4320	0.14	127	10.81	3569
WDR92	1.13	2.06E-09	0.17	135	14.61	1642	0.16	131	13.3	1748	0.2	128	15.92	2092
PPOX	1.67	5.47E-26	0.28	102	14.61	1643	0.21	88	7.84	3814	0.29	94	12.93	2912
ZNF30	1.4	1.67E-15	0.21	118	14.61	1644	0.15	116	9.84	2919	0.34	120	24.01	723
LARP4B	1.15	8.20E-08	0.17	135	14.61	1645	0.16	136	13.66	1634	0.16	135	13.68	2685
PPP1R8	1.16	2.23E-05	0.19	125	14.6	1646	0.12	126	9.47	3083	0.19	143	17.69	1702
ARHGEF11	1.39	8.47E-20	0.2	123	14.6	1647	0.22	140	19.89	412	0.28	124	20.7	1171
PDS5B	1.09	0.045630634	0.16	144	14.59	1648	0.12	125	9.2	3191	0.14	147	13.94	2606
FUNDC1	1.32	2.97E-12	0.26	107	14.59	1649	0.19	113	11.83	2176	0.3	123	22.16	942
BET1	1.3	5.80E-09	0.19	127	14.59	1650	0.16	144	15.35	1181	0.24	130	18.9	1475
MUC4	1.05	3.67E-05	0.18	133	14.58	1651	-0.02	108	-1.21	9294	0.31	111	19.04	1442
PRKAB1	1.35	9.01E-16	0.15	147	14.58	1652	0.2	146	18.99	522	0.2	122	14.29	2524
TUFT1	1.57	5.53E-11	0.15	147	14.57	1653	0.25	128	19.39	466	0.16	129	12.96	2898
SERAC1	1.11	2.49E-10	0.2	124	14.56	1654	0.21	123	15.3	1193	0.27	113	16.91	1853
ARL8A	1.07	0.001180598	0.13	159	14.56	1655	0.07	143	6.2	4661	0.11	134	9.02	4220
CWC27	1.39	2.68E-12	0.23	112	14.56	1656	0.18	107	10.16	2804	0.36	125	27.34	403
LEF1	1.83	5.00E-06	0.2	123	14.56	1657	0.27	117	18.39	601	0.19	133	15.79	2135
KIF16B_t1	1.16	1.36E-05	0.18	131	14.55	1658	0.17	117	11.25	2382	NA	NA	NA	NA
GMPS	1.7	1.02E-23	0.36	90	14.55	1659	0.22	93	9.36	3120	0.38	93	16.2	2033
PPP4R4_t2	1.32	2.22E-07	0.17	136	14.55	1660	0.14	128	10.75	2575	0.24	148	23.16	824
DYRK2	1.3	3.97E-09	0.2	124	14.55	1661	0.26	141	24.01	112	0.26	165	29.89	248
ADAT2_t1	1.15	2.85E-06	0.13	159	14.55	1662	NA	NA	NA	NA	0.18	137	16.02	2068
RGS5_t1	1.64	8.67E-10	0.17	135	14.54	1663	NA	NA	NA	NA	0.25	139	22.07	957
DIRC2	1.09	0.004316501	0.17	138	14.54	1664	0.09	129	7.42	4024	0.01	131	0.69	8198
COMMD4_t1	1.4	3.10E-20	0.22	115	14.53	1665	NA	NA	NA	NA	NA	NA	NA	NA
STAMBPL1	1.3	6.02E-10	0.25	108	14.52	1666	0.19	114	12.21	2063	0.26	134	21.86	996
MOB1B	-1.11	0.063631487	0.13	164	14.51	1667	0.14	132	11.37	2339	0.08	138	6.82	5097
DTNA_t2	1.18	1.17E-06	0.17	134	14.5	1668	0.12	163	13.57	1667	0.15	143	13.7	2676
GID4	1.14	4.43E-05	0.19	126	14.5	1669	0	123	0.2	8335	0.3	134	25.54	552
RNFT2_t1	1.38	4.49E-13	0.21	119	14.49	1670	0.21	131	17.24	791	NA	NA	NA	NA
DVL2	1.27	8.19E-10	0.21	119	14.48	1671	0.2	113	12.65	1942	0.15	116	9.93	3905
HOXD3	1.38	3.05E-14	0.16	142	14.48	1672	0.15	115	9.68	2988	0.32	116	20.94	1128
MAN1A2	1.04	0.320368737	0.13	162	14.48	1673	0.03	120	2.41	6886	0.22	136	19.05	1438
MID1	1.03	0.681129503	0.16	143	14.48	1674	0.07	154	7.2	4138	0.09	139	8.12	4572
IGF2BP2_t1	1.12	6.16E-07	0.19	125	14.47	1675	NA	NA	NA	NA	0.15	136	13.26	2811
TAF1A	1.37	5.89E-12	0.24	110	14.46	1676	0.21	111	13.07	1808	0.29	123	21.39	1065
IARS2	1.33	2.52E-07	0.25	109	14.46	1677	0.15	108	8.46	3526	0.34	110	20.39	1215
ATP5J2_t1	1.51	1.42E-19	0.3	98	14.45	1678	0.24	131	19.59	448	0.34	100	17.14	1802
EPT1_t1	1.01	0.745024908	0.14	156	14.45	1679	NA	NA	NA	NA	NA	NA	NA	NA
CEP128	1.07	1.71E-10	0.19	126	14.45	1680	0.15	127	11.19	2405	0.21	134	17.52	1732
RAD54L	2.26	2.28E-25	0.29	100	14.45	1681	0.22	88	8.53	3489	0.37	123	26.93	424
PPIL1	1.42	4.34E-11	0.19	128	14.44	1682	0.17	139	15.21	1224	0.26	127	20.26	1238
UPF3B	1.25	7.42E-07	0.2	121	14.44	1683	0.22	120	15.29	1197	0.34	120	23.76	756
SRXN1	1.87	1.55E-13	0.17	133	14.44	1684	0.22	126	16.77	884	0.19	138	16.37	1988
CPSF3L	1.05	0.000261878	0.18	131	14.43	1685	0.04	118	2.88	6562	0.13	120	8.96	4238
TIPIN	1.62	1.25E-16	0.24	111	14.42	1686	0.19	93	8.2	3642	0.4	110	24.1	709
SRPK2	1.3	5.59E-14	0.2	123	14.42	1687	0.26	131	20.81	314	0.36	147	34.6	87
DDX59	1.26	2.36E-09	0.21	120	14.42	1688	0.09	91	3.65	6085	0.29	119	20.2	1248
PIK3C2B	1.43	5.59E-13	0.21	118	14.42	1689	0.25	126	18.84	545	0.2	126	14.89	2364
SKA2_t1	1.25	3.55E-08	0.18	131	14.41	1690	NA	NA	NA	NA	NA	NA	NA	NA
HIST1H2BG	1.9	4.32E-21	0.28	102	14.41	1691	0.12	100	6.23	4645	0.29	88	11.09	3482
GPC3	4.23	8.57E-18	0.21	120	14.41	1692	0.15	122	10.84	2539	0.35	115	22.93	852
ABHD3	1.25	2.33E-05	0.18	131	14.4	1693	0.14	133	11.58	2277	0.18	153	18.26	1599

PPP2CA	1.21	5.51E-08	0.19	125	14.4	1694	0.1	125	7.49	3992	0.25	128	19.24	1399
ZNF764_t1	1.14	1.27E-12	0.2	121	14.4	1695	NA	NA	NA	NA	0.23	120	16.21	2031
H2AFX	1.59	1.58E-17	0.21	120	14.39	1696	0.22	104	11.94	2145	0.21	106	11.65	3309
IDE	1.14	0.000154165	0.15	143	14.38	1697	0.16	126	11.89	2154	0.24	132	19.93	1283
TARBP1	1.7	2.54E-15	0.24	110	14.38	1698	0.24	109	14.04	1521	0.29	130	23.15	827
PIGX_t1	1.19	3.81E-09	0.19	125	14.37	1699	NA	NA	NA	NA	0.19	126	14.81	2383
FSTL5	1.35	1.72E-06	0.15	146	14.37	1700	0.07	139	6.4	4566	0.11	161	12.13	3157
MCM7	1.71	1.05E-21	0.25	108	14.37	1701	0.19	104	10.35	2738	0.31	121	21.88	988
G3BP1	1.16	3.81E-05	0.18	132	14.37	1702	0.17	113	10.51	2674	0.19	140	17.06	1813
RAD51D_t1	1.19	1.09E-09	0.18	129	14.36	1703	0.07	119	5.07	5286	0.17	122	12.37	3084
SSBP1	1.5	2.68E-18	0.32	95	14.36	1704	0.23	117	15.74	1103	0.41	85	14.3	2523
STX16	1.13	4.51E-08	0.17	135	14.35	1705	0.25	139	22.07	209	0.15	158	16.47	1964
HGS	1.69	5.90E-29	0.29	99	14.35	1706	0.3	122	21.71	237	0.34	100	16.86	1860
RALY	1.16	3.73E-08	0.2	122	14.35	1707	0.1	161	11.14	2421	0.11	120	7.37	4887
TTC1	1.27	3.52E-11	0.21	117	14.34	1708	0.17	105	9.28	3156	0.35	107	20.2	1249
SLU7_t1	1.11	0.02299947	0.21	118	14.34	1709	NA	NA	NA	NA	NA	NA	NA	NA
PHTF1	1.18	3.68E-10	0.21	117	14.34	1710	0.18	115	11.79	2194	0.24	142	21.92	977
KPNA3	1.1	0.001062471	0.19	125	14.34	1711	0.13	133	10.92	2509	0.25	153	25.79	529
MSI2_t1	1.34	1.14E-10	0.17	132	14.34	1712	0.1	127	7.86	3803	0.13	99	6.24	5341
MMS19	1.28	3.68E-12	0.21	119	14.34	1713	0.2	115	13.02	1825	0.2	128	15.84	2118
KNTC1	1.61	1.44E-14	0.23	112	14.33	1714	0.26	108	15.34	1185	0.29	131	23.09	835
NUP37	1.57	7.51E-21	0.2	120	14.33	1715	0.29	100	14.63	1371	0.32	120	22.55	890
CNTNAP2_t1	1.04	3.04E-05	0.19	127	14.33	1716	0.17	137	15.02	1271	NA	NA	NA	NA
USP34	1.2	1.05E-08	0.18	130	14.32	1717	0.26	119	18.16	632	0.27	128	21.26	1075
WDFY3	1.05	0.006378545	0.16	137	14.31	1718	0.03	135	2.35	6924	0.19	145	18.34	1584
BUB3_t1	1.41	2.89E-14	0.24	109	14.31	1719	0.16	118	10.76	2570	0.29	103	15.26	2260
ADNP2	1.19	5.42E-08	0.13	160	14.31	1720	0.19	114	12.31	2032	0.16	120	11.28	3421
ZMYND11_t1	1.14	0.001402675	0.19	126	14.3	1721	0.07	138	6.03	4760	0.26	142	24.11	705
LMTK2	1.08	1.85E-07	0.18	131	14.3	1722	0.08	139	6.74	4386	0.21	158	23.19	821
PDZD11	1.54	8.39E-31	0.26	105	14.3	1723	0.15	128	11.68	2242	0.38	151	38.03	36
GABRG2_t1	1.06	0.005304295	0.17	135	14.3	1724	NA	NA	NA	NA	0.16	119	10.88	3557
RAD51B_t1	1.11	5.06E-10	0.21	118	14.3	1725	0.01	113	0.8	7940	0.21	159	22.73	874
PLXNA1_t1	1.02	0.085956548	0.19	125	14.3	1726	NA	NA	NA	NA	0.13	127	9.89	3918
ELOVL1	1.4	9.36E-20	0.26	105	14.29	1727	0.16	127	12.18	2071	0.26	100	13.17	2839
ZNF189	1.25	1.06E-05	0.21	117	14.28	1728	0.24	106	13.31	1744	0.31	152	31.28	190
DNAJC2	1.48	1.75E-09	0.23	112	14.28	1729	0.28	110	16.62	914	0.27	119	18.78	1507
CEP290	1.41	5.60E-08	0.18	129	14.28	1730	0.29	95	12.96	1847	0.22	118	14.97	2342
MND1	2.49	3.72E-28	0.34	92	14.28	1731	0.25	108	14.67	1361	0.44	106	24.5	665
ADCK2_t1	1.2	2.60E-09	0.23	112	14.28	1732	NA	NA	NA	NA	NA	NA	NA	NA
PAK2_t1	1.04	0.055493073	0.15	148	14.27	1733	NA	NA	NA	NA	0.06	130	4.98	5936
SLC48A1	1.14	5.00E-06	0.19	126	14.27	1734	0	138	-0.4	8749	0.13	128	10.46	3705
FAM46D	1.02	0.082097387	0.15	148	14.27	1735	0.02	130	1.99	7152	-0.04	125	-3.19	10260
HPS4	1.28	1.13E-07	0.13	157	14.27	1736	0.21	124	15.87	1072	0.16	131	13.08	2859
NLGN4Y	1.03	0.052774407	0.16	142	14.26	1737	-0.02	139	-1.47	9477	0.02	137	1.36	7826
Sep-02	1.02	0.536035174	0.17	134	14.26	1738	0.14	153	14.51	1403	0	135	-0.17	8716
ACSL3	1.17	0.011594158	0.16	142	14.26	1739	0.19	123	13.54	1672	0.18	151	18.61	1534
DTNA_t3	1.12	1.28E-06	0.16	139	14.25	1740	0.13	122	9.67	2993	0.2	124	14.44	2478
ALMS1	1.39	1.08E-10	0.25	108	14.24	1741	0.24	107	13.96	1542	0.29	98	13.86	2628
FBXO36_t1	1.1	7.82E-09	0.18	131	14.24	1742	0.2	125	14.97	1289	NA	NA	NA	NA
PRPF38B_t1	-1.03	0.376574027	0.13	160	14.23	1743	NA	NA	NA	NA	0.04	138	3.76	6538
WDR82	1.08	3.61E-06	0.16	139	14.23	1744	0.16	145	14.9	1307	0.1	153	9.92	3907
ARID1A	1.25	7.51E-11	0.2	120	14.22	1745	0.18	126	13.87	1574	0.18	142	16.52	1953
KCNJ5	1.26	4.93E-11	0.2	120	14.22	1746	0.23	133	18.99	521	0.22	121	15.75	2141
FANCG	1.82	3.24E-22	0.24	109	14.22	1747	0.19	104	10.14	2813	0.33	128	25.54	555
BCL2L11	1.04	0.101147857	0.15	147	14.22	1748	0.11	138	10.09	2828	0.04	147	3.91	6462
ZNF674	1.07	7.68E-07	0.16	140	14.21	1749	0.13	127	10.14	2812	0.31	130	25.06	615
TXNDC12	1.12	0.000361953	0.17	136	14.21	1750	0.05	110	2.73	6656	0.11	131	9.07	4199
ATP6V1C2_t1	1.13	9.49E-14	0.2	122	14.19	1751	NA	NA	NA	NA	0.28	120	19.36	1371
ZNF445	1.07	1.02E-06	0.15	147	14.19	1752	0.09	145	8.89	3332	0.21	128	16.41	1982
BBS12	1.15	1.77E-05	0.16	140	14.19	1753	0.18	120	12.91	1859	0.17	126	12.95	2908
AGK	1.06	0.006293621	0.16	136	14.19	1754	0.13	122	9.53	3047	0.14	119	9.62	4016
SETD8_t1	1.07	0.006620369	0.17	134	14.18	1755	NA	NA	NA	NA	NA	NA	NA	NA
ZNF229	1.2	2.67E-05	0.18	127	14.18	1756	0.15	126	11.44	2323	0.11	147	10.45	3708
TFAM_t1	1.02	0.645872091	0.15	147	14.18	1757	NA	NA	NA	NA	0.15	147	14.47	2468
NSUN5	1.48	1.82E-25	0.23	112	14.18	1758	0.24	142	22.28	196	0.27	131	22.04	964
GCG	1.1	0.000160373	0.19	126	14.18	1759	0.13	115	8.25	3625	0.13	114	8.13	4569
BOP1	1.97	1.57E-20	0.33	93	14.17	1760	0.2	80	5.99	4792	0.33	95	14.9	2362
ZNF766	1.15	6.50E-06	0.16	136	14.17	1761	0.1	131	8.11	3690	0.14	137	12.16	3148
ALYREF_t1	1.46	1.39E-18	0.26	104	14.17	1762	NA	NA	NA	NA	0.24	107	13.5	2738
ATP5J2_t2	1.55	2.97E-19	0.3	98	14.16	1763	0.22	131	17.69	717	0.37	100	18.52	1548
ACTR3	1.08	0.084662891	0.17	134	14.16	1764	0.26	141	23.58	127	0.15	133	12.2	3139
CELF5	1.11	1.01E-10	0.19	125	14.15	1765	0.11	97	5.35	5147	0.14	118	9.43	4078
ITGB1BP1	1.49	6.18E-15	0.24	110	14.15	1766	0.29	117	19.41	461	0.24	113	15.05	2319
ZSCAN29_t1	1.21	5.88E-12	0.2	120	14.15	1767	NA	NA	NA	NA	NA	NA	NA	NA
MANF	1.95	9.72E-18	0.27	103	14.15	1768	0.25	106	13.89	1566	0.35	128	27.34	404
FTSJD1	1.02	0.15473962	0.18	127	14.15	1769	0	138	0.02	8453	0.11	124	8.39	4474
GEMIN2	1.3	3.29E-10	0.22	113	14.15	1770	0.12	121	8.24	3626	0.26	107	14.92	2357
DEDD	1.33	7.36E-24	0.24	108	14.15	1771	0.18	141	16.72	892	0.32	150	31.66	177
POU2F1_t1	1.12	0.004872878	0.15	145	14.15	1772	NA	NA	NA	NA	0.14	136	11.88	3242
FAM105B	1.15	1.18E-06	0.14	150	14.15	1773	0.05	126	3.45	6221	0.18	138	15.88	2105
FAM84B	1.25	0.000200034	0.16	136	14.14	1774	0.12	139	10.71	2586	0.29	128	22.58	886
ERAL1	1.27	3.88E-17	0.21	116	14.14	1775	0.09	119	5.97	4800	0.21	131	17.18	1790
BROX	1.28	1.12E-17	0.28	100	14.14	1776	0.21	101	10.93	2505	0.32	146	30.91	200
ZNF28	1.22	4.83E-07	0.17	131	14.13	1777	0.21	120	15.03	1268	0.23	124	17.36	1757
DMXL1_t1	1.05	7.12E-05	0.15	145	14.13	1778	0.15	138	13.54	1673	NA	NA	NA	NA
IPO4	1.37	9.93E-17	0.18	128	14.13	1779	0.14	102	7.24	4122	0.17	99	8.52	4416
TXNL4A	1.32	3.69E-10	0.19	126	14.13	1780	0.16	118	11.05	2456	0.2	120	14.09	2575
MIPOL1	1.15	1.71E-08	0.15	145	14.13	1781	0.23	152	23.06	146	0.23	140	20.64	1182
MARCKS	1.16	0.079275489	0.17	132	14.13	1782	0.26	134	22.14	202	0.02	123	1.81	7562
HEATR3	1.06	0.011013914	0.17	133	14.12	1783	0.13	131	10.58	2636	0.17	121	12.07	3174
TTLL7_t1	1.14	0.000767191	0.14	148	14.12	1784	0.16	122	11.53	2293	NA	NA	NA	NA
ATG10_t1	1.25	6.57E-12	0.19	123	14.12	1785	NA	NA	NA	NA	NA	NA	NA	NA
UIMC1	1.16	1.99E-05	0.2	120	14.12	1786	0.24	101	12.45	1990	0.21	115	13.5	2739
TMPO_t2	1.24	8.59E-09	0.17	135	14.11	1787	0.09	130	7.31	4087	0.21	139	19.11	1434
TCP11L1	1.23	9.78E-11	0.21	117	14.11	1788	0.1	132	8.2	3641	0.12	143	11.27	3423
FHIT	1.24	4.69E-16	0.26	104	14.1	1789	0.17	120	12.06	2114	0.28	91	11.55	3339
PNPT1	1.26	8.09E-07	0.17	133	14.09	1790	0.23	139	20.9	301	0.24	152	24.77	642
SFRP4	1.67	7.74E-06	0.14	152	14.08	1791	0.15	140	13.5	1682	0.04	149	3.96	6432
PUSL1	1.38	6.48E-13	0.2	119	14.07	1792	0.14	121	9.75	2954	0.19	107	10.89	3555
EDA2R	1.07	6.77E-09	0.17	135	14.07	1793	0.07	132	5.73	4936	0.08	138	7.33	4902
FERMT1_t1	1.54	5.19E-06	0.14	153	14.07	1794	0.16	129	12.93	1857	NA	NA	NA	NA
LGI2	1.09	0.019498281	0.14	148	14.07	1795	0.12	148	11.31	2358	0.04	149	3.53	6660
ALG8	1.5	1.49E-15	0.23	112	14.07	1796	0.16	98	7.75	3857	0.34	119	23.47	790
NUFIP1	1.08	0.000551856	0.17	132	14.07	1797	0.2	130	15.61	1119	0.17	143	15.48	2199
AGPAT5	1.05	0.222793017	0.15	147	14.07	1798	0.07	135	5.78	4904	-0.02	132	-1.62	9512
CDKN2A_t1	3.04	8.63E-22	0.26	104	14.06	1799	NA	NA	NA	NA	0.3	127	23.4	799

EBF3	1.24	1.38E-09	0.18	126	14.06	1800	0.08	132	6.92	4290	0.24	155	25.11	609
ZMPSTE24	1.26	0.000168757	0.2	120	14.05	1801	0.12	110	7.42	4026	0.27	126	20.82	1151
RABL5	1.33	1.14E-08	0.16	140	14.05	1802	0.19	135	15.9	1068	0.11	148	10.97	3521
HSF2BP	1.15	8.79E-15	0.2	122	14.05	1803	0.18	140	16.65	909	0.27	109	15.94	2089
MIER1_t1	1.06	0.002119905	0.17	132	14.05	1804	0.16	133	13.44	1702	0.23	157	24.49	667
LHFPL1	1.03	0.008510395	0.17	135	14.04	1805	0.04	123	3.06	6467	0.12	127	9.61	4020
ILF3_t1	1.29	3.13E-13	0.19	122	14.04	1806	0.23	137	19.7	433	0.12	123	8.82	4297
PSD2	1.05	0.000276873	0.2	120	14.03	1807	0.04	132	3.47	6201	0.17	123	12.57	3016
DPH5_t1	1.33	1.54E-14	0.18	129	14.02	1808	0.19	105	10.45	2705	0.24	103	12.8	2951
AGAP1	1.35	3.70E-11	0.15	141	14.02	1809	0.26	139	23.26	142	0.22	139	19.48	1351
ATP1B1_t1	1.35	2.12E-05	0.22	115	14.02	1810	NA	NA	NA	NA	NA	NA	NA	NA
GLT25D1	1.16	2.50E-05	0.17	133	14.02	1811	0.19	126	14.35	1436	0.1	122	7.47	4846
AGBL5_t1	1.34	3.78E-12	0.25	106	14.01	1812	0.18	105	9.63	3007	0.19	98	9.23	4143
USP42	1.2	1.80E-11	0.16	137	14	1813	0.13	117	8.76	3388	0.23	126	17.85	1672
ABL1_t1	1.05	0.001724064	0.18	129	13.99	1814	0.15	133	12.67	1935	0.09	133	7.08	5000
CHST1	1.08	1.51E-05	0.15	142	13.99	1815	0.1	132	7.95	3771	0.17	154	17.35	1759
UQCRQ	1.29	0.000645268	0.16	139	13.98	1816	0.09	115	5.99	4787	0.27	144	25.49	562
CENPM	1.7	6.31E-25	0.25	105	13.98	1817	0.17	136	14.5	1405	0.34	103	18.04	1642
HEATR2	1.39	8.15E-23	0.2	120	13.97	1818	0.17	120	11.82	2184	0.27	137	23.81	749
TP53BP2	1.39	2.20E-08	0.19	122	13.96	1819	0.06	120	4.28	5726	0.31	111	18.86	1487
ASAP1_t1	1.13	0.01958934	0.2	120	13.96	1820	0.2	140	18.4	599	NA	NA	NA	NA
GARS	1.6	1.04E-17	0.25	106	13.96	1821	0.23	132	19.21	485	0.38	147	37.21	52
YOD1	1.1	1.47E-11	0.17	130	13.96	1822	0.13	129	10.58	2638	0.24	136	20.53	1194
HABP4	1.28	1.71E-07	0.17	131	13.95	1823	0.15	124	11.29	2369	0.11	151	11.11	3468
METAP1	1.08	0.07493619	0.14	148	13.95	1824	0.18	123	12.82	1889	0.18	146	17.06	1814
ZNF829	1.03	0.003572608	0.14	152	13.95	1825	0.03	140	2.48	6838	-0.01	160	-0.93	9153
ZNF461	1.18	1.19E-08	0.17	134	13.95	1826	0.18	119	12.74	1914	0.2	128	15.74	2145
RPS27A	1.26	1.40E-07	0.22	114	13.94	1827	0.2	104	10.81	2549	0.25	116	16.47	1965
R3HCC1L	1.04	0.06009477	0.14	148	13.93	1828	0.05	141	4.15	5808	0.11	120	7.49	4836
SMS_t1	1.17	2.03E-07	0.19	125	13.93	1829	NA	NA	NA	NA	0.2	120	14.03	2591
MFSD5_t1	1.31	1.62E-15	0.23	111	13.93	1830	NA	NA	NA	NA	0.19	111	11.77	3278
SMG9	1.24	1.00E-15	0.28	100	13.93	1831	0.19	94	8.21	3636	0.22	104	11.75	3281
TBC1D7	1.46	9.82E-19	0.22	113	13.93	1832	0.18	113	11.59	2275	0.32	111	19.35	1374
VPS13B_t2	1.38	9.29E-09	0.17	130	13.93	1833	0.19	141	17.73	708	0.27	125	20.26	1240
DNAJC13	1.15	4.75E-06	0.24	109	13.92	1834	0.18	141	16.25	993	0.32	120	22.37	912
Mar-04	1.07	3.02E-08	0.2	119	13.92	1835	0.26	131	21.36	254	0.22	114	14.21	2543
PHLPP2	1.03	0.419757313	0.14	148	13.92	1836	-0.03	147	-2.52	10164	0.09	132	6.98	5039
TMEM155	1.06	8.29E-08	0.17	132	13.92	1837	0.06	128	4.58	5571	0.18	136	15.91	2096
ENOX2_t1	1.25	1.25E-08	0.16	137	13.92	1838	NA	NA	NA	NA	0.23	134	18.92	1469
LOX	1.36	4.94E-06	0.15	141	13.91	1839	0.18	144	17.3	778	0.1	131	7.93	4647
SULT1C2	2.41	1.74E-14	0.2	120	13.91	1840	0.29	123	21.1	281	0.23	126	17.66	1708
MLEC_t1	1.33	2.47E-10	0.17	133	13.91	1841	NA	NA	NA	NA	0.2	157	21.49	1052
MDH1B	1.1	1.01E-07	0.14	150	13.91	1842	0.06	122	4.03	5891	0.19	138	16.43	1974
DDX10_t1	1.23	8.91E-06	0.2	118	13.91	1843	NA	NA	NA	NA	NA	NA	NA	NA
TRIM14_t1	1.01	0.465675066	0.19	123	13.9	1844	0.05	112	3.19	6392	0.02	100	1.2	7913
KCTD2	1.1	1.14E-08	0.18	129	13.9	1845	0.13	140	11.67	2243	0.15	122	11.04	3502
AKR1C3	2.3	1.85E-15	0.24	108	13.89	1846	0.19	86	6.78	4361	0.26	96	11.95	3217
SPIN2B_t1	1.25	1.62E-09	0.19	122	13.89	1847	0.13	99	6.16	4692	NA	NA	NA	NA
BYSL	1.45	1.21E-13	0.2	120	13.89	1848	0.15	134	12.64	1944	0.27	96	12.35	3088
RAD52	1.08	9.02E-05	0.14	150	13.89	1849	0.03	134	2.48	6843	0.15	125	11.01	3511
PSPC1	1.08	0.005481305	0.18	128	13.88	1850	0.08	107	4.74	5476	0.07	108	4.34	6270
DSP	1.09	0.083892281	0.15	140	13.88	1851	0.07	114	4.78	5459	0.11	115	7.45	4853
DCC	1.01	0.184227306	0.15	141	13.88	1852	-0.07	130	-5.68	12021	0.09	142	7.88	4672
GLA	1.36	1.15E-09	0.28	99	13.87	1853	0.22	57	1.51	7489	0.18	93	7.73	4741
INTS8	1.52	3.29E-16	0.32	93	13.87	1854	0.27	99	13.44	1701	0.4	105	21.86	994
NDRG3	1.27	2.49E-12	0.2	120	13.86	1855	0.24	150	24.01	113	0.2	129	15.87	2106
HSF1	1.23	3.13E-05	0.26	103	13.86	1856	0.11	115	7.24	4124	0.32	119	22.4	909
CDC42_t2	1.15	0.000228376	0.18	129	13.86	1857	0.17	133	14.29	1456	0.12	132	9.44	4070
RPL13A	1.3	5.68E-08	0.16	139	13.86	1858	0.11	123	7.99	3750	0.17	122	12.33	3099
NOSIP_t1	1.12	5.92E-05	0.19	124	13.86	1859	NA	NA	NA	NA	0.08	100	3.8	6526
USP48_t1	-1.03	0.532638113	0.15	140	13.85	1860	NA	NA	NA	NA	NA	NA	NA	NA
MMP1	1.76	8.62E-11	0.26	104	13.84	1861	0.16	125	11.89	2155	0.35	134	29.34	279
MCFD2	1.35	4.60E-08	0.15	143	13.84	1862	0.11	128	8.77	3385	0.21	131	17.37	1754
UBE2E3_t1	1.21	6.58E-07	0.2	120	13.84	1863	NA	NA	NA	NA	NA	NA	NA	NA
DPCD	1.35	7.96E-13	0.22	114	13.84	1864	0.13	129	10.34	2744	0.24	100	12.16	3147
RPS21	1.51	4.16E-13	0.19	121	13.83	1865	0.23	83	7.66	3900	0.23	75	5.8	5540
SMARCE1	1.23	2.43E-10	0.18	129	13.83	1866	0.19	113	11.97	2133	0.24	106	13.33	2789
PRMT2_t1	1.06	0.147862113	0.19	123	13.83	1867	NA	NA	NA	NA	-0.01	133	-0.51	8919
LSM14A	1.27	3.66E-08	0.17	132	13.83	1868	0.24	124	17.64	726	0.19	123	13.98	2600
ARHGEF37	1.61	3.36E-11	0.18	125	13.82	1869	0.07	125	5.21	5215	0.18	126	13.75	2662
RRP12	1.3	2.40E-08	0.2	119	13.82	1870	0.1	113	6.61	4447	0.22	96	10.05	3859
NUP107	1.57	1.44E-12	0.19	121	13.81	1871	0.28	140	24.78	88	0.28	148	27.86	379
PRAME_t1	1.73	7.84E-08	0.15	145	13.81	1872	0.1	110	6.05	4747	NA	NA	NA	NA
ZNF107_t1	1.15	1.95E-12	0.18	125	13.8	1873	NA	NA	NA	NA	NA	NA	NA	NA
ZNF551	1.28	1.23E-07	0.18	126	13.8	1874	0.16	138	14.15	1493	0.13	133	10.49	3694
CNOT4_t1	1.25	3.58E-16	0.19	123	13.79	1875	0.21	118	14.09	1508	0.32	126	24.24	697
NIPAL2	1.1	5.93E-06	0.2	118	13.79	1876	0.08	124	5.96	4807	0.21	150	20.99	1123
OSBPL3_t1	1.37	9.76E-06	0.15	140	13.79	1877	0.27	131	22.27	197	NA	NA	NA	NA
HOMER1	1.03	0.740291692	0.17	130	13.79	1878	0.1	138	9.19	3195	0.14	145	13.04	2871
CEP250	1.4	7.99E-20	0.22	113	13.78	1879	0.23	117	15.14	1241	0.25	139	22.33	920
MSTO1	1.43	9.06E-24	0.21	115	13.78	1880	0.16	116	10.72	2584	0.29	124	21.67	1020
HTRA4	1.17	4.95E-08	0.22	113	13.77	1881	0.18	111	11.03	2462	0	108	-0.06	8658
PRDM9	1.12	1.15E-07	0.25	106	13.76	1882	0.09	133	7.69	3889	0.26	126	19.82	1300
EIF3C	1.28	1.13E-10	0.17	130	13.76	1883	0.22	121	15.51	1142	0.17	124	12.55	3023
IP6K1	1.13	9.82E-06	0.18	128	13.76	1884	0.16	124	11.93	2146	0.2	120	14	2596
RHAG	1.03	0.003112973	0.18	126	13.76	1885	0.11	132	9.02	3262	0.06	120	4.5	6181
FANCD2_t1	2.03	1.35E-21	0.25	104	13.74	1886	0.28	136	23.82	120	0.36	123	26.27	476
YBX1_t1	1.02	0.658361475	0.2	120	13.74	1887	NA	NA	NA	NA	0.03	117	1.7	7623
MID2	1.36	9.13E-09	0.15	140	13.74	1888	0.06	128	4.32	5708	0.24	144	22.48	900
ZNF248_t1	1.11	1.71E-07	0.17	132	13.74	1889	NA	NA	NA	NA	0.21	134	17.7	1701
ASH1L	1.1	1.73E-12	0.18	128	13.73	1890	0.19	124	14.33	1447	0.19	121	13.35	2781
G6PD	1.3	4.61E-10	0.2	120	13.73	1891	0.21	142	19.1	502	0.19	124	13.84	2635
ZNF813	1.11	0.000117615	0.15	141	13.72	1892	0.23	127	17.91	686	0.19	141	16.98	1830
RIBC1_t1	1.07	2.71E-12	0.17	132	13.72	1893	0.1	137	8.5	3497	0.22	154	23.01	845
PRL	1.05	0.000653513	0.19	123	13.72	1894	0.05	102	2.84	6594	0.19	112	11.91	3231
ASAP2	1.46	6.20E-09	0.17	132	13.72	1895	0.17	134	14.14	1495	0.28	159	30.37	226
YY1AP1	1.3	4.16E-09	0.21	115	13.72	1896	0.19	106	10.61	2624	0.23	109	13.84	2637
CSNK2B	1.32	1.12E-14	0.17	132	13.72	1897	0.08	94	3.54	6150	0.24	99	11.91	3230
COPS6	1.33	2.88E-16	0.15	144	13.71	1898	0.1	125	7.83	3819	0.23	111	14.14	2562
TMEM147	1.5	7.17E-19	0.23	110	13.71	1899	0.26	120	17.91	685	0.22	87	8.21	4534
ZSCAN4	1.05	2.16E-05	0.23	110	13.7	1900	0.13	118	9.09	3236	0.14	98	6.72	5143
BTN3A1	1.2	1.55E-08	0.17	129	13.7	1901	0.1	124	7.6	3932	0.11	126	8.69	4349
KIAA0586	1.14	2.65E-06	0.18	127	13.7	1902	0.12	112	7.59	3935	0.23	121	16.35	1993
SH3PXD2B	1.15	1.51E-06	0.17	129	13.69	1903	0.19	137	16.62	915	0.18	136	15.15	2293
CAND1	1.06	0.152351728	0.18	126	13.68	1904	0.16	135	13.6	1653	0.23	135	19.55	1340
PTPN14_t1	1.26	1.09E-07	0.15	144	13.67	1905	NA	NA	NA	NA	0.09	142	8.41	4462

HARS2	1.36	1.46E-15	0.17	132	13.66	1906	0.16	104	8.67	3423	0.24	135	20.2	1247
GUCY1A2	1.03	0.056083147	0.15	142	13.66	1907	0.09	148	8.96	3295	0.14	160	15.11	2303
SP5	2.53	6.74E-12	0.22	113	13.66	1908	0.16	102	8.12	3683	0.21	120	15.01	2337
ZNF219	1.85	1.60E-20	0.27	100	13.66	1909	0.15	106	8.25	3622	0.31	119	21.63	1027
ZNF282	1.16	5.70E-09	0.2	120	13.66	1910	0.09	135	7.63	3915	0.16	152	16.62	1923
PYGO2	1.39	1.24E-25	0.17	131	13.65	1911	0.17	136	14.94	1296	0.21	128	16.06	2055
CLASP2	1.12	0.000281627	0.14	150	13.65	1912	0.16	118	10.99	2481	0.29	137	25.43	571
SBF2	1.04	0.256680654	0.15	139	13.65	1913	0.05	132	3.75	6035	0.15	153	15.83	2121
TTC37	1.12	5.77E-05	0.18	126	13.64	1914	0.09	126	7.1	4196	0.23	113	14.36	2499
CLCN3_t1	1.19	4.36E-08	0.17	130	13.64	1915	0.05	142	4.39	5670	0.29	168	34.37	91
ESF1	1.28	1.76E-13	0.2	118	13.63	1916	0.21	126	16.02	1042	0.33	138	29.39	271
FIP1L1	1.11	0.015873842	0.19	120	13.63	1917	0.25	148	24.8	87	0.15	133	12.18	3143
ANXA2P1	1.59	9.25E-08	0.17	132	13.62	1918	0.24	147	23.68	124	0.17	129	13.04	2872
GBP2	1.44	5.81E-06	0.17	132	13.62	1919	0.11	120	7.72	3874	0.16	111	9.84	3938
TFCP2_t1	1.15	7.48E-07	0.18	124	13.62	1920	NA	NA	NA	NA	0.15	127	11.87	3244
TP63_t1	-1	0.899598694	0.17	132	13.62	1921	NA	NA	NA	NA	0.11	148	10.62	3644
CHL1	1.05	0.014056994	0.17	131	13.61	1922	0.08	136	6.81	4344	0.17	152	17.23	1779
PGAP1_t1	1.12	8.22E-09	0.13	155	13.61	1923	NA	NA	NA	NA	NA	NA	NA	NA
PTGES	1.05	0.027631172	0.16	136	13.61	1924	0.16	124	12.1	2102	0.03	118	1.97	7467
WDR5	1.36	3.72E-12	0.22	112	13.6	1925	0.19	127	14.27	1458	0.23	130	18.72	1519
SPTLC2	1.02	0.527768557	0.14	149	13.6	1926	0.05	139	4.59	5565	0.02	145	1.85	7536
BTBD3_t1	1.15	1.52E-08	0.15	143	13.6	1927	0.2	132	16.7	895	NA	NA	NA	NA
CEP104	1.16	1.74E-10	0.15	143	13.6	1928	0.1	142	9.63	3010	0.2	155	21.07	1105
PAPOLG	1.05	0.102463912	0.16	136	13.59	1929	0.14	131	11.21	2394	0.11	143	10.61	3650
PLEKHS1	1.15	0.010428672	0.17	128	13.59	1930	-0.03	127	-2.05	9857	0.12	115	7.6	4794
MED17	1.04	0.205655826	0.19	120	13.59	1931	0.16	120	11.26	2376	0.13	143	12.2	3140
GNAI3	1.06	0.182818357	0.16	135	13.59	1932	0.08	133	6.26	4635	0.17	138	15.28	2256
PARD3	1.19	2.54E-08	0.18	127	13.59	1933	0.04	124	3.2	6382	0.34	126	25.82	524
ITPA	1.26	3.98E-10	0.18	124	13.58	1934	0.21	115	13.41	1711	0.14	115	8.81	4298
UBLCP1	1.1	0.00297172	0.15	138	13.58	1935	0.2	134	16.39	962	0.24	116	16.1	2050
UEVLD_t2	1.09	0.002903067	0.17	130	13.57	1936	0.16	133	13.37	1726	0.23	124	17.33	1764
NCDN	1.08	0.000219511	0.15	142	13.56	1937	0.12	161	13.63	1642	0.07	136	6.02	5446
MTHFD1L_t1	1.24	6.66E-09	0.21	116	13.56	1938	NA	NA	NA	NA	0.24	123	17.86	1669
GPI	1.36	2.88E-08	0.16	135	13.55	1939	0.11	134	9.21	3186	0.19	118	13.06	2864
LEPRE1	1.21	1.50E-07	0.17	132	13.55	1940	0.16	119	10.94	2496	0.17	120	11.75	3282
KIAA1598	1.22	0.000825648	0.15	143	13.55	1941	0.05	136	4.73	5489	0.27	132	22.54	891
PLK1	2.14	1.00E-26	0.22	111	13.55	1942	0.2	120	13.9	1562	0.31	120	21.51	1048
CKAP4	1.48	2.01E-10	0.18	125	13.53	1943	0.2	104	10.98	2484	0.13	120	8.87	4271
GAPVD1_t1	1.03	0.340045615	0.15	140	13.53	1944	NA	NA	NA	NA	NA	NA	NA	NA
ING5_t1	1.05	0.006608435	0.16	135	13.53	1945	0.11	138	9.46	3086	NA	NA	NA	NA
RAD50	1.34	5.28E-07	0.18	126	13.53	1946	0.17	153	17.49	748	0.33	118	22.24	930
TMEM144	1.06	0.038331793	0.16	137	13.53	1947	0.2	123	14.59	1377	0.12	117	7.85	4685
CSMD2	1.03	0.003386513	0.21	115	13.52	1948	0.11	146	10.85	2534	0.14	127	10.79	3574
ARL6IP6	1.12	0.004115406	0.18	126	13.52	1949	0.26	118	17.47	751	0.1	139	8.91	4258
GPR155	1.01	0.74109709	0.14	148	13.51	1950	-0.06	131	-4.66	11471	-0.01	153	-0.95	9166
ZNF614_t1	1.06	0.026717247	0.17	131	13.51	1951	NA	NA	NA	NA	0.18	153	18.13	1618
ZNF138	1.43	1.42E-17	0.19	120	13.51	1952	0.29	135	24.28	104	0.36	136	31.28	189
NOL8_t1	1.1	0.000391026	0.16	137	13.51	1953	NA	NA	NA	NA	NA	NA	NA	NA
SNRPB2	1.21	2.80E-08	0.18	124	13.5	1954	0.17	140	15.58	1126	0.22	143	20.49	1202
RNASEH2A	1.62	5.57E-26	0.22	111	13.49	1955	0.12	100	6.23	4648	0.27	104	14.65	2418
UBD	4.1	1.32E-11	0.24	107	13.49	1956	0.24	95	10.92	2506	0.22	105	12.33	3098
HNRNPUL2	1.05	0.009486913	0.17	129	13.48	1957	0.19	136	15.99	1052	0.06	141	5.57	5642
PI4K2A_t1	1.05	0.004022828	0.17	128	13.48	1958	NA	NA	NA	NA	0.05	123	3.49	6672
SNN	1.1	0.006671968	0.13	151	13.47	1959	0.19	152	19.45	457	0.1	131	8.26	4509
CDC14A_t1	1.09	6.99E-07	0.19	121	13.47	1960	0.17	135	14.74	1343	0.28	125	21.13	1095
MN1	1.18	1.93E-18	0.17	128	13.47	1961	0.23	124	17.27	788	0.27	143	25.19	598
LPIN1	1.39	6.55E-05	0.16	132	13.47	1962	0.12	140	11.13	2424	0.16	142	14.48	2466
CDK8	1.08	0.039808407	0.13	157	13.46	1963	0.11	99	5.44	5091	0.19	120	13.29	2798
SNAPIN	1.41	3.26E-19	0.19	122	13.46	1964	0.22	117	14.74	1342	0.22	161	24.92	623
CDC123	1.28	5.01E-10	0.19	122	13.46	1965	0.21	117	13.94	1548	0.24	112	15.02	2330
RPP21	1.25	2.53E-14	0.17	128	13.45	1966	0.13	138	11.57	2282	0.14	103	7.4	4866
UBQLN1_t2	1.08	0.034285082	0.18	126	13.45	1967	0.1	135	8.86	3349	0.19	143	17.88	1667
RAD54B	1.5	7.71E-13	0.21	115	13.45	1968	0.24	143	22.19	201	0.36	125	27.21	413
PAQR4	1.3	2.60E-12	0.23	109	13.44	1969	0.18	141	16.13	1012	0.23	123	16.99	1828
RCCD1	1.48	2.95E-15	0.15	139	13.44	1970	0.16	133	13.58	1662	0.34	120	23.75	758
GOPC_t1	1.16	2.85E-10	0.14	145	13.44	1971	NA	NA	NA	NA	0.14	144	12.76	2958
MAGEA5	1.18	3.50E-09	0.18	124	13.43	1972	0.11	116	7.43	4020	0.26	115	17.19	1787
GNG5	1.16	6.10E-05	0.25	104	13.43	1973	0.19	120	13.5	1681	0.14	102	7.51	4825
ZRANB2_t1	1.16	4.41E-10	0.15	139	13.43	1974	0.17	125	13.1	1804	0.18	135	15.31	2243
ZNF485	1.24	3.18E-13	0.15	139	13.42	1975	0.26	131	21.29	259	0.31	120	21.58	1036
OTUD4	1.03	0.021129381	0.21	115	13.42	1976	0.11	117	7.68	3895	0.1	100	4.96	5950
IGSF3	2.18	1.43E-14	0.19	120	13.42	1977	0.19	141	17.33	772	0.27	153	28.32	351
CUTA	1.4	7.67E-14	0.2	116	13.39	1978	0.09	116	5.62	4997	0.27	92	11.22	3438
DNAJC10	1.1	0.146609809	0.13	154	13.39	1979	0.23	145	21.68	239	0.05	130	4.24	6325
TUBA1C_t1	1.5	1.46E-14	0.22	110	13.38	1980	NA	NA	NA	NA	NA	NA	NA	NA
ATP2A2_t1	1.21	2.18E-10	0.18	126	13.38	1981	0.13	120	8.98	3287	NA	NA	NA	NA
CRKL	1.11	2.74E-12	0.17	131	13.38	1982	0.19	132	15.69	1109	0.17	144	16.13	2042
ATXN2L_t1	1.13	1.67E-10	0.19	121	13.38	1983	0.24	120	16.61	919	0.17	115	10.83	3566
SRSF10_t1	1.08	0.09568117	0.16	136	13.37	1984	NA	NA	NA	NA	0.15	127	11.37	3392
UBOX5_t1	1.21	1.74E-08	0.16	133	13.36	1985	NA	NA	NA	NA	NA	NA	NA	NA
AP4B1	1.07	0.00428153	0.2	118	13.35	1986	0.05	124	3.87	5966	0.11	124	7.93	4651
PTBP1	1.22	7.94E-11	0.19	119	13.35	1987	0.12	136	10.3	2756	0.2	123	14.64	2423
PURB_t1	1.26	8.53E-08	0.14	145	13.35	1988	NA	NA	NA	NA	0.23	151	23.01	844
RAB28_t1	1.12	2.93E-05	0.16	136	13.35	1989	0.08	120	5.63	4992	NA	NA	NA	NA
PEA15_t1	1.47	4.69E-11	0.19	121	13.35	1990	0.25	132	20.72	320	NA	NA	NA	NA
FXC1	1.15	0.000113814	0.2	117	13.35	1991	0.09	139	7.81	3828	0.09	138	7.98	4628
SYNCRIP	1.12	0.022683352	0.15	137	13.34	1992	0.19	139	16.82	876	0.18	129	14.27	2530
ANGPT2_t1	1.49	2.17E-08	0.18	124	13.34	1993	NA	NA	NA	NA	0.32	132	26.37	467
SAPCD2	1.35	9.77E-11	0.17	127	13.32	1994	0.23	139	20.61	329	0.12	120	8.64	4370
ZDHHC3	1.19	2.74E-09	0.12	162	13.31	1995	0.11	155	11.84	2173	0.2	162	21.89	987
BBX	1.07	0.3000216	0.13	150	13.3	1996	0.19	141	17.06	826	0.08	148	7.56	4811
DEGS1	1.17	0.003660083	0.15	136	13.3	1997	0.12	129	9.46	3084	0.14	134	11.77	3276
POLR1B_t1	1.15	1.59E-05	0.16	132	13.29	1998	NA	NA	NA	NA	0.24	118	16.47	1962
SLC6A10P	1.45	9.65E-10	0.18	123	13.28	1999	0.15	156	15.4	1168	0.19	120	13.2	2829
ZNF193	1.36	3.24E-19	0.2	117	13.28	2000	0.15	103	7.7	3880	0.3	119	20.6	1185
KIDINS220	1.07	0.242705523	0.18	122	13.28	2001	0.16	135	13.82	1593	0.22	122	15.78	2136
FAM156A	1.17	0.00015679	0.16	133	13.28	2002	0.17	116	10.91	2514	0.12	130	9.82	3947
ASCL2	1.03	0.011077035	0.19	120	13.27	2003	0.02	113	1.28	7620	0.06	109	3.26	6801
ZNF7	1.31	3.19E-10	0.27	100	13.27	2004	0.11	84	3.65	6088	0.24	80	7.1	4994
ZBTB49	1.12	4.93E-07	0.2	115	13.26	2005	0.11	99	5.17	5234	0.18	129	13.96	2604
LYSMD1	1.42	7.00E-23	0.2	115	13.26	2006	0.18	136	15.21	1222	0.28	111	16.96	1839
COL4A5_t1	1.12	7.68E-11	0.22	110	13.26	2007	0.14	116	9.15	3207	0.18	115	11.94	3221
TSPAN17	1.19	5.32E-11	0.21	113	13.25	2008	0.14	155	14.6	1376	0.18	118	12.53	3035
DIS3L2	1.14	2.95E-09	0.2	115	13.25	2009	0.17	113	10.52	2667	0.19	122	13.66	2690
EYA3	1.05	0.001330543	0.16	131	13.25	2010	0.07	135	5.84	4875	0.16	144	15.09	2307
ACYP1	1.36	8.88E-13	0.19	119	13.25	2011	0.2	106	11.1	2427	0.23	116	15.36	2228

AP4E1_t1	1.05	1.80E-07	0.19	121	13.24	2012	NA	NA	NA	NA	0.29	141	25.98	506
ZNF280C	1.21	1.46E-09	0.13	151	13.23	2013	0.11	134	8.95	3300	0.2	126	15.56	2187
SH3KBP1	1.01	0.900108228	0.12	158	13.23	2014	0.14	123	10.58	2633	0.16	132	12.89	2925
NUP205	1.34	6.09E-10	0.25	102	13.23	2015	0.2	110	12.04	2121	0.33	103	17.28	1770
FANCM	1.16	1.04E-06	0.17	127	13.22	2016	0.14	122	10.3	2757	0.24	142	21.77	1009
SPZ1	1	0.951571035	0.18	123	13.21	2017	-0.03	113	-1.74	9645	0.08	112	5.24	5814
NLK	1.25	1.84E-09	0.16	133	13.21	2018	0.14	130	10.93	2503	0.27	128	21.2	1083
CHRAC1	1.09	0.003921019	0.18	124	13.21	2019	0.04	136	3.24	6349	0.19	135	16.25	2017
ILF3_t2	1.41	4.50E-18	0.19	120	13.2	2020	0.24	126	18.07	652	0.21	139	18.99	1454
TRPC6	1.08	1.27E-07	0.15	137	13.19	2021	0.24	117	16.11	1020	0.25	130	19.95	1280
FRMD3	1.09	7.25E-05	0.16	132	13.19	2022	0.06	135	5.42	5109	0.15	130	11.72	3293
TRPM6	1.02	0.008839736	0.16	135	13.19	2023	0.06	137	5.44	5092	0.14	128	10.75	3593
NEDD8	1.49	4.70E-20	0.23	108	13.18	2024	0.23	111	13.95	1544	0.31	118	21.34	1069
GTPBP2	1.2	1.61E-12	0.22	109	13.18	2025	0.22	124	16.13	1013	0.29	120	20.03	1271
ARFGEF1	1.14	0.006466959	0.19	120	13.18	2026	0.15	150	15.41	1163	0.25	133	20.91	1132
SSR1	1.17	2.08E-05	0.13	151	13.17	2027	0.14	141	12.53	1973	0.19	145	17.72	1696
SAC3D1	1.97	1.72E-34	0.26	100	13.17	2028	0.27	88	10.31	2753	0.35	100	17.59	1718
SSRP1	1.29	1.10E-17	0.21	114	13.17	2029	0.11	128	8.5	3499	0.21	130	16.93	1849
PRMT3	1.23	1.14E-13	0.2	117	13.17	2030	0.19	122	13.98	1535	0.33	126	25.13	608
PKMYT1_t1	1.37	7.01E-17	0.17	128	13.16	2031	0.11	100	5.72	4944	0.24	120	17.15	1801
YWHAQ_t1	1.27	5.85E-07	0.16	134	13.16	2032	NA	NA	NA	NA	NA	NA	NA	NA
BUB3_t2	1.24	3.24E-08	0.17	126	13.16	2033	0.08	129	6.51	4507	0.29	126	21.87	990
PHF15_t1	1.1	0.003553519	0.21	113	13.16	2034	NA	NA	NA	NA	0.1	142	8.82	4293
TMEM87B	1.1	0.000138449	0.15	139	13.15	2035	0.23	140	20.87	304	0.14	150	13.83	2639
SLC16A7	1.01	0.876202421	0.15	140	13.14	2036	0.04	137	3.9	5948	0.04	162	4.73	6065
AKAP8	1.21	1.05E-11	0.15	135	13.14	2037	0.18	136	15.08	1258	0.12	126	9.3	4118
NDUFA4L2	1.25	4.78E-11	0.19	121	13.14	2038	0.21	129	16.5	941	0.22	115	14.22	2542
CPSF2	1.15	0.001245238	0.14	145	13.13	2039	0.14	129	11.01	2470	0.25	132	20.41	1211
SKP1_t2	1.11	0.024612787	0.16	130	13.13	2040	0.14	129	10.99	2480	0.23	146	22.27	925
OCLN	1.16	0.018798892	0.15	139	13.13	2041	0	142	-0.27	8653	0.18	132	14.61	2430
VDAC2	1.23	3.93E-09	0.21	113	13.13	2042	0.11	118	7.48	3998	0.23	108	13.45	2752
B4GALT6	1.13	1.13E-11	0.16	131	13.12	2043	0.15	120	10.22	2789	0.23	139	20.87	1142
FANCE	1.52	3.01E-10	0.19	120	13.12	2044	0.13	153	13.38	1721	0.21	116	14.05	2585
TMEM70	1.39	5.00E-07	0.19	120	13.12	2045	0.15	136	13.05	1814	0.25	125	18.84	1492
ZMIZ1	1.14	0.01472352	0.15	137	13.12	2046	0.17	143	15.61	1118	0.09	124	6.99	5038
ZC3HAV1_t1	1.08	1.71E-07	0.17	127	13.12	2047	0.13	136	11.22	2393	0.12	134	10.42	3712
DYNC1I1	1.81	8.72E-09	0.17	129	13.11	2048	0.18	127	14.19	1485	0.21	126	15.65	2166
PASD1	1.04	0.025021455	0.21	113	13.11	2049	0.02	128	1.56	7459	0.15	115	9.91	3912
STK36	1.28	1.98E-12	0.15	140	13.11	2050	0.17	134	13.9	1563	0.16	114	10.5	3688
RBM45	1.23	2.99E-08	0.19	119	13.11	2051	0.27	123	19.64	438	0.25	95	11.33	3405
CBFA2T2_t1	1.37	5.56E-12	0.18	124	13.11	2052	0.17	138	15.38	1172	0.18	132	14.46	2473
CLOCK	1.1	0.004423421	0.13	153	13.1	2053	0.11	133	9.41	3106	0.24	151	23.93	730
ATIC	1.44	4.12E-17	0.22	110	13.1	2054	0.29	105	15.9	1067	0.27	100	13.74	2665
TAX1BP1	1.35	3.78E-08	0.18	123	13.1	2055	0.24	120	16.68	900	0.29	122	20.89	1137
LRR1_t2	1.17	2.14E-09	0.16	134	13.1	2056	0.14	102	7.06	4221	0.22	116	14.23	2540
ATP6V1E2	1.44	1.16E-14	0.23	107	13.09	2057	0.23	102	11.85	2171	0.28	101	14.36	2503
ZC2HC1A	1.24	0.010671451	0.15	136	13.09	2058	0.21	127	16.3	979	0.14	132	11.68	3301
RMI1	1.56	7.96E-15	0.17	127	13.08	2059	0.18	127	13.98	1534	0.28	140	25.5	560
TMEM97	1.41	3.63E-07	0.18	121	13.08	2060	0.06	123	4.47	5627	0.21	120	14.8	2385
ATG7	1.15	3.07E-08	0.15	136	13.08	2061	0.19	122	13.62	1645	0.03	120	2.29	7303
CSRP2	1.74	2.20E-07	0.17	127	13.07	2062	0.07	116	4.86	5417	0.24	104	13.23	2821
HNRNPH2	1.24	0.000229261	0.19	119	13.06	2063	0.14	125	10.57	2643	0.28	142	25.76	537
DCBLD2	1.03	0.225578454	0.12	162	13.06	2064	0.03	148	3.21	6379	0.05	147	4.45	6214
HCFC2	1.14	0.003303171	0.15	138	13.06	2065	0.06	134	5.07	5288	0.24	148	24.01	724
ANKRD26_t1	1.15	0.000112445	0.16	132	13.05	2066	NA	NA	NA	NA	0.2	126	14.92	2355
RPL26L1	1.28	4.42E-10	0.2	114	13.05	2067	0.15	117	9.99	2866	0.29	134	24.32	687
SFT2D3	1.31	2.73E-10	0.13	147	13.04	2068	0.11	117	7.2	4140	0.21	136	17.78	1686
NDUFA6	1.22	3.77E-06	0.18	123	13.04	2069	0.13	130	10.46	2698	0.31	115	20.04	1269
RPP40	1.46	2.84E-15	0.2	115	13.04	2070	0.19	72	4.16	5807	0.28	81	8.76	4318
PSMA7	1.28	3.17E-14	0.22	109	13.03	2071	0.24	122	17.09	820	0.2	113	12.62	3004
ZNF529	1.12	0.011843341	0.13	153	13.03	2072	0.17	131	13.41	1708	0.08	132	6.25	5334
RDH14	1.08	0.01256754	0.13	150	13.03	2073	0.02	92	0.73	7981	0.14	108	8.28	4502
IRX5	1.3	2.72E-08	0.18	122	13.03	2074	0.08	119	5.67	4965	0.28	120	19.39	1366
NCBP1	1.14	0.000634631	0.15	135	13.01	2075	0.1	141	9.02	3261	0.23	133	18.94	1465
PM20D2	1.41	1.43E-08	0.14	140	13.01	2076	0.17	124	12.67	1939	0.22	140	19.49	1349
SOX2	1.5	5.89E-08	0.13	148	13	2077	-0.02	138	-1.41	9437	0.27	128	21.43	1057
MDM1_t1	1.09	1.65E-05	0.16	131	13	2078	0.15	111	9.43	3097	0.17	135	14.62	2428
PUF60	1.47	5.35E-15	0.25	102	13	2079	0.14	102	7.53	3971	0.27	142	24.68	654
MDFIC	1.01	0.809040321	0.15	136	13	2080	0.12	138	10.16	2803	-0.03	147	-2.97	10167
RPS28	1.44	1.47E-14	0.24	104	12.99	2081	0.21	123	15.1	1250	0.29	102	15.03	2328
SLC36A2	1.02	0.166794344	0.15	134	12.99	2082	-0.03	127	-2.15	9933	0.01	135	0.74	8171
SNX11	1.14	7.13E-08	0.19	119	12.99	2083	0.06	130	5.01	5333	0.14	126	10.94	3526
AVL9	1.38	6.72E-18	0.22	109	12.99	2084	0.28	89	10.91	2516	0.29	118	19.65	1324
HUWE1_t1	1.1	2.77E-05	0.15	135	12.98	2085	NA	NA	NA	NA	NA	NA	NA	NA
APOBEC3D	1.33	3.49E-11	0.15	136	12.97	2086	0.02	128	1.3	7609	0.14	123	10.15	3818
FAM118B	1.23	6.38E-12	0.15	134	12.97	2087	0.27	147	26.14	55	0.12	143	11.5	3350
TUBB2B	1.22	3.91E-05	0.14	140	12.97	2088	0.12	124	8.72	3401	0.11	142	9.8	3951
SLC5A6_t1	1.31	0.000137617	0.2	115	12.97	2089	NA	NA	NA	NA	NA	NA	NA	NA
IQCD	1.5	3.77E-12	0.17	126	12.96	2090	0.17	104	9.38	3113	0.29	100	14.34	2512
MPP3	1.39	7.41E-06	0.18	124	12.95	2091	0.29	134	24.69	90	0.15	122	10.93	3534
CCNO_t1	1.06	2.34E-06	0.19	119	12.95	2092	0.06	91	2.38	6900	NA	NA	NA	NA
AGBL5_t2	1.55	1.65E-22	0.24	104	12.94	2093	0.18	87	6.49	4519	0.31	92	13.01	2877
SUPT16H	1.23	2.79E-10	0.16	129	12.94	2094	0.1	133	8.6	3457	0.19	133	15.82	2125
FLOT1	1.12	0.000586106	0.2	115	12.94	2095	0.12	126	9.09	3231	0.13	109	7.61	4789
TMEFF1	1.14	8.06E-09	0.17	125	12.94	2096	0.23	118	15.5	1147	0.22	123	16.34	1994
TXLNG	1.09	1.12E-09	0.12	154	12.93	2097	0.07	139	6.28	4629	0.2	140	18.32	1591
KCNG3	1.06	4.16E-06	0.14	145	12.93	2098	0	134	-0.35	8715	0.13	127	10.23	3787
BRIX1	1.42	4.37E-09	0.18	123	12.92	2099	0.16	101	8.17	3658	0.29	120	20.17	1254
KDM3B	1.07	0.010538988	0.14	143	12.92	2100	0.15	139	13.5	1685	0.07	130	5.54	5667
REPIN1_t1	1.34	2.29E-18	0.16	133	12.92	2101	NA	NA	NA	NA	NA	NA	NA	NA
FGF1_t1	1.06	0.000296562	0.14	142	12.92	2102	NA	NA	NA	NA	0.18	141	16.15	2040
DYM_t1	1.09	1.88E-06	0.15	135	12.92	2103	NA	NA	NA	NA	NA	NA	NA	NA
BMS1_t1	1.26	1.22E-07	0.14	142	12.92	2104	NA	NA	NA	NA	NA	NA	NA	NA
ASB11	-1	0.867883516	0.15	138	12.92	2105	-0.08	136	-6.59	12462	0.03	120	2.02	7438
FAM133A_t1	1.74	2.85E-09	0.17	124	12.91	2106	NA	NA	NA	NA	0.22	133	17.93	1658
CBLN1	1.12	3.73E-08	0.15	137	12.91	2107	0.16	132	13.12	1798	0.16	141	14.47	2470
VPS8	1.1	1.02E-06	0.17	125	12.91	2108	0.16	137	13.91	1560	0.18	118	12.05	3177
PIGG	1.29	4.48E-13	0.2	116	12.91	2109	0.18	100	9.18	3198	0.18	90	7.39	4876
TXN	1.6	4.17E-14	0.19	119	12.91	2110	0.31	135	26.01	61	0.23	116	15.05	2316
PSMB5	1.32	9.56E-13	0.18	121	12.9	2111	0.19	120	12.95	1849	0.2	140	17.99	1651
FATE1	1.08	2.97E-09	0.23	106	12.9	2112	0.08	111	5.04	5308	0.25	120	17.5	1734
RAB11A_t1	1.03	0.494385401	0.13	151	12.9	2113	NA	NA	NA	NA	NA	NA	NA	NA
ZNF775	1.54	5.17E-23	0.25	102	12.9	2114	0.14	116	8.97	3293	0.28	107	15.84	2117
LUC7L3_t1	1.18	0.000910744	0.18	122	12.9	2115	0.22	116	14.38	1428	0.21	129	16.55	1943
CIZ1	1.33	1.02E-21	0.23	107	12.9	2116	0.15	90	5.91	4835	0.24	97	11.38	3391
PUM1_t1	1.07	0.048177641	0.15	136	12.89	2117	NA	NA	NA	NA	NA	NA	NA	NA

SETD4_t1	1.19	8.25E-07	0.29	94	12.89	2118	0.17	104	9.07	3243	0.11	99	5.55	5656
TMEM167B	1.05	0.305200819	0.17	124	12.88	2119	0.1	120	6.71	4398	0.15	147	14.94	2352
PGAM5_t1	1.14	1.40E-09	0.24	104	12.88	2120	NA	NA	NA	NA	NA	NA	NA	NA
RGS3_t1	1.1	1.52E-06	0.2	116	12.87	2121	0.24	124	18.1	645	0.14	104	7.69	4756
KCNE1L_t1	1.45	2.10E-13	0.18	123	12.87	2122	NA	NA	NA	NA	0.28	123	20.25	1241
ANO10	1.28	6.79E-13	0.21	112	12.87	2123	0.21	124	15.47	1152	0.27	111	16.76	1888
THOC1	1.2	3.28E-08	0.24	104	12.86	2124	0.14	92	5.68	4958	0.28	95	12.59	3008
TMEM165	1.11	0.031842075	0.17	126	12.84	2125	0.25	109	14.92	1302	0.03	114	1.98	7462
CHTF18_t1	1.55	4.19E-15	0.23	107	12.84	2126	NA	NA	NA	NA	NA	NA	NA	NA
CLCN7	1.13	6.29E-07	0.2	114	12.84	2127	0.11	136	9.07	3245	0.11	120	7.9	4661
GSDMC	1.14	1.57E-05	0.2	113	12.83	2128	0.04	130	2.92	6536	0.27	122	19.24	1401
XG	1.05	0.00248409	0.15	135	12.83	2129	0.17	132	13.87	1575	0.18	144	16.73	1896
VGLL2_t1	1.03	0.027458357	0.17	127	12.83	2130	-0.02	131	-1.46	9466	0.09	150	8.99	4230
GREB1_t1	1.59	1.06E-08	0.13	151	12.83	2131	0.14	124	10.7	2592	0.2	123	14.48	2465
CGREF1	2.44	7.24E-14	0.24	104	12.82	2132	0.17	116	11.23	2391	0.31	121	21.87	992
NDUFA7	1.23	3.48E-08	0.18	121	12.8	2133	0.03	107	1.44	7528	0.19	117	12.68	2982
NOL12	1.28	4.22E-15	0.16	131	12.8	2134	0.19	155	20.26	372	0.23	150	22.71	877
RNF4	1.17	0.000187129	0.17	124	12.79	2135	0.18	130	14.14	1494	0.15	124	11.05	3496
PACSIN2	1.21	0.00010901	0.14	143	12.79	2136	0.07	120	4.84	5431	0.11	119	7.73	4743
KDELR3	1.61	5.32E-09	0.2	113	12.79	2137	0.16	103	8.41	3537	0.19	95	8.37	4477
TRAF7	1.15	4.34E-09	0.2	113	12.79	2138	0.15	126	11.24	2386	0.15	100	7.51	4826
SHFM1	1.47	2.36E-11	0.16	129	12.78	2139	0.26	116	16.98	846	0.31	98	15.1	2305
PHF20L1_t2	1.24	5.43E-07	0.15	138	12.78	2140	0.13	135	11.07	2440	0.26	140	23.76	757
XK	2.03	2.71E-11	0.14	144	12.78	2141	0.14	127	10.85	2532	0.27	153	28	371
HAUS4	1.2	0.000440478	0.14	139	12.77	2142	-0.1	125	-7.49	12871	0.11	119	7.93	4650
PACRGL_t1	1.13	1.21E-09	0.17	124	12.76	2143	0.19	124	14.36	1435	NA	NA	NA	NA
ASF1A	1.35	5.41E-12	0.17	126	12.76	2144	0.14	128	11.04	2460	0.16	135	13.73	2668
GSTM1	1.07	0.314264256	0.17	127	12.76	2145	0.13	127	10.22	2787	0.06	120	3.87	6487
PDCD2	1.16	0.000179942	0.15	137	12.75	2146	0.17	128	13.18	1780	0.1	137	8.93	4250
ZNF518A	1.1	0.001165893	0.14	141	12.74	2147	0.1	129	7.72	3876	0.18	138	16.19	2036
RNF32	1.08	1.34E-05	0.2	114	12.74	2148	0.01	146	1.43	7536	0.2	101	10.32	3749
DNAJC30	1.49	1.95E-20	0.16	132	12.74	2149	0.14	134	12.03	2123	0.27	133	22.56	889
TAF12	1.11	0.003820855	0.12	155	12.73	2150	0.07	95	3.37	6271	0.21	116	13.61	2710
AP3M1	1.12	3.09E-05	0.15	136	12.73	2151	0.19	134	15.57	1128	0.26	160	28.31	352
HS3ST2	1.14	0.001907684	0.15	133	12.72	2152	0.09	128	7.21	4134	0.13	116	8.52	4418
PDCD6IP_t1	1.05	0.146793672	0.12	159	12.72	2153	NA	NA	NA	NA	NA	NA	NA	NA
RHOQ_t1	1.11	0.039240933	0.12	153	12.72	2154	NA	NA	NA	NA	0.09	126	6.57	5202
MLLT1	1.09	3.89E-11	0.18	120	12.71	2155	0.09	141	8.25	3621	0.19	124	13.7	2677
TLK1	1.12	9.75E-08	0.15	133	12.7	2156	0.09	138	7.54	3967	0.25	139	22.22	933
ZUFSP	1.12	0.000988906	0.15	132	12.7	2157	0.19	128	15.05	1261	0.15	132	12.7	2975
RNGTT	1.13	0.00049589	0.12	154	12.7	2158	0.08	129	6.34	4594	0.19	142	17.79	1685
PRKD3_t1	1.05	0.200788231	0.15	132	12.69	2159	NA	NA	NA	NA	0.18	149	17.52	1731
ZNF320	1.2	8.36E-06	0.13	144	12.69	2160	0.22	136	18.67	561	0.18	137	15.98	2078
VPS54	1.14	0.00749459	0.15	132	12.69	2161	0.16	104	8.49	3502	0.26	127	20.18	1252
CEP350	1.13	0.039948446	0.12	157	12.68	2162	0.15	158	15.91	1065	0.21	143	19.56	1337
CCZ1	1.28	1.57E-10	0.17	124	12.68	2163	0.18	124	13.59	1659	0.26	112	15.91	2094
DCTPP1	1.36	4.49E-13	0.18	119	12.68	2164	0.14	131	11.36	2341	0.18	130	14.58	2437
NCAPD2	1.77	1.46E-20	0.21	110	12.67	2165	0.21	147	20.72	318	0.25	152	25.16	605
RNF34_t1	1.17	3.22E-10	0.18	120	12.67	2166	NA	NA	NA	NA	0.13	104	7.03	5025
TRMT12	1.29	1.05E-09	0.21	111	12.66	2167	0.11	126	8.04	3720	0.24	136	20.3	1230
EXTL2	1.13	2.10E-06	0.15	136	12.65	2168	0.13	121	8.93	3317	0.23	135	19.2	1410
FAM135A	1.12	8.70E-05	0.12	152	12.65	2169	0.2	119	13.93	1550	0.27	140	24.56	662
EXOSC3	1.28	7.61E-10	0.16	130	12.65	2170	0.18	134	15.29	1199	0.17	112	10.6	3656
ERCC1	1.2	1.28E-09	0.18	121	12.65	2171	0.02	151	1.85	7242	0.08	116	5.25	5799
OPA1_t1	1.09	0.096359428	0.13	144	12.64	2172	NA	NA	NA	NA	0.19	146	17.77	1687
MRPL15	1.21	0.000816152	0.17	124	12.63	2173	0.11	120	7.98	3755	0.21	130	16.85	1863
ZNF212	1.25	1.31E-15	0.13	151	12.63	2174	0.05	115	3.28	6334	0.17	121	12.14	3153
NPC1	1.39	4.24E-13	0.18	122	12.63	2175	0.21	130	16.45	950	0.25	126	19.32	1382
ACBD6	1.71	1.31E-27	0.24	102	12.63	2176	0.21	119	14.56	1391	0.32	96	14.82	2380
ZNF691	1.21	2.24E-12	0.14	138	12.63	2177	0.06	150	5.98	4799	0.12	124	9.07	4196
GNPTAB_t1	1.12	0.001484284	0.17	123	12.63	2178	NA	NA	NA	NA	NA	NA	NA	NA
ACTR1A	1.14	6.56E-06	0.17	126	12.62	2179	0.05	147	4.51	5608	0.07	122	5.12	5866
CLDN18	1.11	0.00077769	0.15	134	12.62	2180	0.11	139	9.87	2908	0.04	129	2.86	7012
HIAT1	1.09	0.060733987	0.18	120	12.61	2181	0.11	130	8.44	3532	0.17	134	13.88	2623
ZIC2	1.17	1.50E-11	0.16	130	12.61	2182	0.06	131	5.09	5278	0.27	118	18.33	1586
MYCN	1.72	4.62E-09	0.17	125	12.61	2183	0.13	137	11.73	2222	0.28	128	21.64	1025
NUP50	1.11	0.000963365	0.13	148	12.61	2184	0.12	132	10.04	2847	0.18	127	13.62	2705
PHIP	1.11	0.129518448	0.13	145	12.61	2185	0.19	143	17.78	702	0.07	133	6.07	5414
ZNF79	1.17	1.24E-12	0.23	104	12.6	2186	0.19	108	10.85	2530	0.12	96	5.69	5585
ZBTB9	1.28	4.31E-16	0.18	122	12.6	2187.5	0.19	130	15.27	1208	0.26	128	19.94	1282
EIF1	1.19	3.46E-05	0.18	122	12.6	2187.5	0.16	115	10.1	2824	0.23	141	20.73	1167
ELOVL7	1.1	7.89E-06	0.12	152	12.6	2189	0.11	135	9.43	3095	0.18	130	14.14	2561
NLRP5	1.12	6.54E-12	0.16	130	12.6	2190	0	105	0	8469	0.16	100	8.1	4585
PPP2R3C	1.11	0.074530546	0.12	158	12.59	2191	0.16	104	8.39	3544	0.09	91	3.89	6476
IRX3	2.11	6.26E-12	0.23	104	12.59	2192	0.04	110	2.57	6781	0.26	122	18.53	1546
FIBP_t1	1.36	2.40E-19	0.25	101	12.59	2193	0.17	124	12.49	1982	0.21	121	15.04	2327
MSL3_t1	1.08	1.66E-05	0.16	131	12.59	2194	0.17	131	13.7	1622	0.14	139	12.34	3090
ZNF761_t1	1.18	0.000876948	0.15	132	12.58	2195	NA	NA	NA	NA	0.17	120	12.08	3170
RHOC	1.24	0.00015859	0.25	100	12.57	2196	0.11	140	9.53	3048	0.19	115	12.53	3034
HOXD10	1.34	3.86E-11	0.2	113	12.57	2197	0.04	105	2.17	7030	0.23	117	15.34	2234
ZNHIT3	1.21	1.14E-10	0.16	130	12.57	2198	0.13	144	12.14	2086	0.18	142	16.41	1981
TUBG1	1.46	4.00E-21	0.2	112	12.57	2199	0.17	99	8.54	3481	0.31	107	17.84	1676
HIST1H3A	1.33	1.14E-09	0.29	93	12.57	2200	0.21	97	9.68	2987	0.21	70	4.2	6340
USP32_t1	1.13	0.005387919	0.14	142	12.56	2201	NA	NA	NA	NA	0.2	151	20.37	1220
FABP5	1.66	1.24E-08	0.23	104	12.56	2202	0.3	99	14.86	1314	0.21	108	11.92	3229
SQSTM1	1.73	2.78E-14	0.19	117	12.55	2203	0.18	136	15.18	1229	0.21	120	14.94	2353
AIM1L	1.3	1.68E-16	0.31	91	12.55	2204	0.21	118	14.21	1472	0.25	93	10.78	3578
FAM206A	1.22	2.91E-05	0.17	122	12.55	2205	0.27	118	18.19	624	0.2	117	13.65	2695
SYNJ2	1.04	0.000380415	0.15	134	12.55	2206	0.04	134	3.78	6021	0.15	138	13	2882
ORMDL2	1.37	6.30E-20	0.21	110	12.54	2207	0.17	130	13.39	1720	0.28	113	17.63	1714
DBN1	1.19	0.038114423	0.17	126	12.54	2208	0.14	122	9.87	2906.5	0.03	128	2.02	7435
CHST6	1.13	3.44E-08	0.11	164	12.54	2209	0.27	143	25.22	73	0.09	150	9.29	4120
CD58	1.5	1.66E-14	0.18	120	12.53	2210	0.18	95	8.26	3619	0.26	103	13.67	2688
CHRM2_t1	1.23	3.13E-08	0.18	121	12.53	2211	-0.01	130	-0.51	8822	0.29	155	29.95	245
ARPC5	1.28	3.56E-08	0.19	116	12.52	2212	0.23	117	15.15	1236	0.16	137	14.29	2526
RNF215	1.2	2.51E-12	0.15	136	12.52	2213	0.09	127	7.06	4214	0.23	108	13.6	2711
UBAP2L	1.23	8.92E-10	0.16	126	12.52	2214	0.26	119	18.07	650	0.23	139	20.62	1184
ZNF639	1.17	6.73E-06	0.17	124	12.52	2215	0.17	135	14.35	1438	0.2	115	13.26	2809
YIF1B	1.19	5.54E-11	0.19	116	12.52	2216	0.03	154	3.3	6322	0.22	123	16.14	2041
GON4L_t1	1.34	6.52E-10	0.17	124	12.52	2217	0.19	130	14.99	1280	0.2	125	15.06	2314
NHP2	1.48	1.07E-19	0.28	94	12.52	2218	0.18	105	9.71	2970	0.33	106	18.55	1543
HDAC2	1.11	0.028801153	0.14	142	12.52	2219	0.26	140	23.47	131	0.09	142	7.85	4687
ZFAND3	1.17	2.66E-08	0.16	128	12.51	2220	0.15	127	11.75	2212	0.13	131	10.31	3758
MYEF2	1.18	0.000229366	0.13	148	12.51	2221	0.06	142	5.85	4872	0.1	128	7.98	4629
CAP1	1.1	0.021401612	0.18	120	12.51	2222	0.17	120	12.16	2084	0.07	144	6.77	5120
CEP76	1.14	2.06E-05	0.11	163	12.51	2223	0.04	124	2.86	6574	0.13	138	11.49	3353

PLAGL2	1.2	3.98E-05	0.17	124	12.5	2224	0.06	138	5.44	5094	0.09	130	7.16	4967
AUNIP	1.36	5.99E-17	0.21	110	12.5	2225	0.22	114	14.21	1473	0.3	112	18.8	1505
SUPT3H	1.09	0.000120113	0.14	140	12.5	2226	0.17	143	15.69	1108	0.11	134	9.1	4186
NSMAF	1.18	0.003766463	0.17	122	12.48	2227	0.21	126	16.22	997	0.1	126	7.96	4634
MYPOP	1.11	1.97E-05	0.16	128	12.48	2228	0.03	120	2.13	7057	0.18	131	14.88	2366
PWP1	1.21	1.13E-06	0.19	117	12.48	2229	0.23	129	17.99	666	0.18	126	13.32	2792
FUBP1	1.19	2.40E-05	0.12	155	12.48	2230	0.23	139	20.3	367	0.08	136	6.76	5125
FAM58A	1.38	2.82E-13	0.25	99	12.47	2231	0.21	96	9.62	3015	0.28	109	16.52	1951
SSX2IP	1.32	6.47E-07	0.12	152	12.47	2232	0.08	147	7.92	3787	0.25	130	19.86	1292
RIMS2	1.21	0.000139098	0.13	144	12.47	2233	0.15	131	11.88	2162	0.09	139	8.27	4508
PI15	1.09	0.000174627	0.13	148	12.46	2234	-0.09	131	-7.4	12832	0.1	135	8.13	4565
AACS	1.49	3.35E-15	0.18	120	12.45	2235	0.27	147	26.08	58	0.15	133	12.55	3028
OLA1_t1	1.24	5.14E-06	0.15	135	12.45	2236	0.19	141	17.14	809	NA	NA	NA	NA
ZCCHC10	1.17	2.75E-05	0.16	129	12.45	2237	0.14	115	9.09	3232	0.26	136	22.52	895
CAMK2G_t1	1.1	2.71E-05	0.14	140	12.45	2238	NA	NA	NA	NA	0.2	123	14.49	2461
SRPK1	1.29	1.50E-05	0.12	152	12.45	2239	0.21	124	15.5	1145	0.25	168	28.96	305
CHMP4B_t1	1.13	0.008918108	0.14	136	12.44	2240	NA	NA	NA	NA	0.02	146	2.13	7380
HARBI1	1.22	7.84E-16	0.16	127	12.44	2241	0.12	120	8.09	3698	0.25	111	15.05	2315
CSNK1G1	1.07	6.87E-06	0.14	140	12.44	2242	0.14	135	11.76	2205	0.21	148	20.36	1222
TMED9	1.19	3.05E-06	0.19	116	12.43	2243	0.15	101	7.63	3920	0.08	108	4.59	6124
BLM	1.75	4.51E-15	0.23	105	12.43	2244	0.26	100	13.16	1785	0.34	115	21.82	1001
PMS2	1.36	1.64E-20	0.19	114	12.42	2245	0.19	103	10.2	2794	0.33	88	12.44	3051
PDCL3	1.17	5.11E-10	0.24	102	12.42	2246	0.22	114	13.84	1583	0.25	113	15.44	2207
ARL8B	1.2	1.38E-08	0.17	122	12.42	2247	0.15	135	13.12	1795	0.2	143	18.4	1567
TCF3_t1	1.33	2.02E-07	0.16	129	12.41	2248	0.29	129	23.29	140	NA	NA	NA	NA
FAM216A	1.47	1.67E-13	0.17	121	12.41	2249	0.15	116	9.85	2915	0.25	132	20.56	1190
P4HA2_t2	1.59	4.10E-13	0.2	111	12.4	2250	0.14	126	10.8	2551	0.3	109	17.57	1721
CTNNB1	1.33	1.25E-09	0.19	116	12.4	2251	0.12	117	8.03	3727	0.26	134	21.72	1014
ZDHHC16	1.1	0.002706658	0.16	128	12.4	2252	-0.03	114	-1.66	9593	0.11	116	7.05	5005
BUD31	1.42	9.38E-16	0.3	92	12.4	2253	0.24	120	16.81	877	0.34	89	13.21	2828
GOSR2_t2	1.25	1.17E-10	0.17	123	12.39	2254	0.11	121	7.63	3922	0.18	132	15.05	2321
MAGED2	1.24	5.71E-08	0.2	111	12.39	2255	0.12	129	9.1	3224	0.16	118	10.97	3520
MRPL11	1.15	1.34E-07	0.18	120	12.38	2256	0.04	125	2.85	6588	0.17	118	11.32	3408
DTYMK	2.09	2.93E-28	0.29	93	12.38	2257	0.25	114	15.83	1084	0.32	123	23.54	785
RPS11	1.35	2.40E-10	0.23	104	12.38	2258	0.17	124	12.28	2038	0.21	85	7.21	4940
TESK1	1.1	1.08E-10	0.23	104	12.38	2259	0.09	118	5.97	4801	0.23	100	11.34	3401
AGPS	1.1	0.022790305	0.14	141	12.38	2260	0.05	143	4.51	5607	0.15	131	11.95	3214
EPDR1	1.63	2.13E-07	0.2	113	12.38	2261	0.21	135	18.26	616	0.22	136	18.89	1478
COBRA1	1.17	3.96E-09	0.17	121	12.36	2262	0.09	117	6	4782	0.08	126	6.35	5299
TMEM135	1.07	0.230265433	0.13	143	12.36	2263	0.06	127	4.6	5554	0.22	134	18.45	1561
ZFYVE26_t1	1.2	4.53E-07	0.16	126	12.36	2264	NA	NA	NA	NA	NA	NA	NA	NA
LAMA3_t1	2.17	1.34E-13	0.17	122	12.35	2265	0.08	115	4.97	5359	0.32	122	22.78	866
NEFL	1.04	0.007575537	0.11	166	12.35	2266	0.02	135	1.84	7249	0.09	134	7.3	4914
SLK	1.04	0.360125094	0.15	131	12.35	2267	0.09	120	6.03	4766	0.22	127	16.59	1933
ARHGAP33	1.15	2.07E-13	0.15	135	12.35	2268	0.02	113	1.38	7559	0.14	115	9.22	4151
MIIP	1.26	2.17E-14	0.23	103	12.35	2269	0.06	108	3.41	6240	0.21	91	8.79	4306
VPS13B_t3	1.09	1.56E-06	0.18	120	12.35	2270	0.1	126	7.5	3987	0.2	134	16.53	1948
EXOSC9	1.21	4.28E-06	0.22	107	12.35	2271	0.09	89	3.36	6275	0.19	104	10.29	3768
MICB_t1	1.39	5.14E-16	0.17	122	12.34	2272	0.13	134	10.96	2489	NA	NA	NA	NA
UBA5	1.12	0.007431456	0.13	145	12.34	2273	0.09	130	7.19	4149	0.24	126	18.26	1600
MRPS12	1.32	5.79E-12	0.27	95	12.34	2274	0.13	112	8.21	3637	0.24	108	13.8	2648
SNX3	1.19	6.10E-06	0.16	126	12.34	2275	0.23	134	19.33	475	0.19	123	13.67	2687
SLC26A2	1.14	4.38E-12	0.12	152	12.33	2276	0.18	127	13.87	1573	0.2	132	16.04	2063
ARL2BP	1.27	2.95E-09	0.17	122	12.32	2277	0.17	141	15.78	1094	0.2	137	16.98	1832
RNF115	1.43	3.92E-15	0.21	109	12.32	2278	0.19	114	12.36	2020	0.3	135	25.88	518
HDHD1	1.17	1.04E-05	0.16	126	12.32	2279	0.14	129	10.76	2569	0.2	127	15.4	2217
CWF19L1	1.04	0.086407948	0.14	136	12.31	2280	-0.01	135	-0.6	8872	0.03	150	3.44	6695
TRIM6_t1	1.29	4.40E-07	0.15	130	12.31	2281	NA	NA	NA	NA	0.2	135	16.8	1875
SET	1.15	0.00111306	0.14	135	12.31	2282	0.22	124	16.63	912	0.07	124	5.24	5809
CDC20	1.72	2.40E-24	0.29	92	12.31	2283	0.16	90	6.27	4631	0.33	82	10.71	3610
FBXO16	1.04	0.442976777	0.14	137	12.31	2284	0.07	108	4.16	5803	0.06	123	4.17	6346
LCLAT1	1.32	1.76E-05	0.14	137	12.3	2285	0.09	148	8.78	3381	0.25	143	23.54	786
PROSER1	1.1	0.024116538	0.18	120	12.3	2286	0.21	145	19.53	452	0.05	130	4.1	6370
EDC3	1.32	6.04E-17	0.17	124	12.29	2287	0.16	120	10.99	2479	0.26	121	18.74	1515
GNS	1.18	4.04E-08	0.17	123	12.29	2288	0.16	140	14.03	1523	0.15	136	13.01	2879
ARMC9	1.63	5.63E-15	0.29	93	12.29	2289	0.25	88	9.34	3129	0.19	98	9.13	4179
VCPIP1	1.12	4.38E-05	0.13	146	12.29	2290	0.13	141	11.66	2251	0.19	158	20.45	1207
TTC27	1.2	3.01E-07	0.24	101	12.28	2291	0.24	81	7.32	4081	0.27	88	10.08	3845
BRD8	1.33	7.51E-13	0.25	99	12.27	2292	0.08	116	5.32	5160	0.28	120	19.52	1344
ABCC4_t1	1.29	1.08E-09	0.12	151	12.27	2293	NA	NA	NA	NA	0.13	139	11.56	3335
RECQL4	2.55	9.04E-29	0.34	86	12.26	2294	0.2	93	8.49	3507	0.41	120	28.71	321
PARD3B_t2	1.04	0.000620799	0.14	139	12.26	2295	0.02	145	1.68	7367	0	128	0.02	8607
POLR2L	1.33	1.61E-10	0.16	128	12.25	2296	0.12	116	7.99	3749	0.24	109	13.87	2625
TIPRL_t2	1.46	9.42E-16	0.28	94	12.25	2297	0.24	135	20.81	315	0.33	120	22.81	862
RRP7B	1.19	1.38E-07	0.2	111	12.25	2298	0.14	152	13.96	1543	0.1	120	6.72	5146
NLGN1	1.03	0.017561639	0.12	149	12.25	2299	0.09	119	6.14	4706	0.11	130	8.96	4242
ARL6	1.14	2.19E-10	0.21	108	12.25	2300	0.15	108	8.59	3459	0.21	132	17.35	1758
COA1	1.23	1.93E-09	0.16	127	12.24	2301	0.18	150	17.96	671	0.17	96	7.83	4701
GPNMB	1.08	0.513179431	0.15	133	12.24	2302	0.2	134	16.51	940	0.03	133	2.82	7025
GPR85	-1	0.88972116	0.14	137	12.24	2303	0.02	128	1.27	7626	0.08	139	6.87	5075
OPTN	1.39	1.52E-06	0.14	136	12.24	2304	0.09	140	8.53	3486	0.24	120	16.67	1911
INTS4_t1	1.2	4.51E-09	0.23	104	12.24	2305	NA	NA	NA	NA	NA	NA	NA	NA
MFSD9	1.11	0.02337226	0.17	120	12.23	2306	-0.01	109	-0.42	8761	0.11	140	9.56	4036
ATP6V0B	1.26	6.31E-10	0.19	114	12.23	2307	0.11	132	8.63	3443	0.16	120	11.21	3443
HLA-B	1.26	0.00212304	0.18	118	12.23	2308	0.14	102	7.11	4192	0.06	120	4.17	6345
PRTG	1.06	0.000250931	0.13	146	12.23	2309	0.03	140	2.26	6984	0.17	142	15.45	2204
DHDDS_t1	1.09	9.28E-08	0.17	124	12.22	2310	0.04	136	3.4	6250	0.18	123	12.96	2902
COL4A2	1.38	0.00069754	0.13	147	12.22	2311	0.21	139	18.9	534	0.09	138	8.02	4611
SLC35E4	1.24	2.05E-06	0.16	128	12.22	2312	0.19	127	14.71	1350	0.15	120	10.32	3750
CENPN	1.77	3.19E-22	0.32	88	12.21	2313	0.21	102	10.77	2566	0.44	70	8.85	4278
MPC2	1.56	5.76E-11	0.15	129	12.21	2314	0.17	125	12.69	1931	0.29	136	25.34	581
JKAMP	1.12	0.006672592	0.15	133	12.21	2315	0.09	127	6.86	4318	0.2	139	18.07	1630
SLC39A1	1.37	1.77E-12	0.17	120	12.2	2316	0.14	111	8.83	3363	0.18	125	13.82	2644
SEZ6	1.18	4.47E-09	0.16	127	12.2	2317	0.06	131	4.94	5376	0.28	124	21.05	1113
HIATL1_t1	1.21	9.99E-11	0.18	117	12.2	2318	NA	NA	NA	NA	0.2	128	15.83	2122
UBE2N_t1	1.09	0.010239455	0.16	125	12.2	2319	NA	NA	NA	NA	0.22	137	19.01	1452
POLR2H	1.22	6.49E-12	0.22	105	12.19	2320	0.21	100	10.37	2733	0.19	103	10.12	3831
SMARCA4	1.5	9.15E-17	0.21	108	12.19	2321	0.31	124	22.59	180	0.22	113	14.08	2576
GABRA2	1.1	0.00090661	0.14	135	12.19	2322	-0.01	124	-0.64	8897	0.15	113	9.25	4135
CMC2	1.02	0.503916768	0.14	137	12.18	2323	0.01	101	0.57	8091	0.1	113	6.37	5288
NDRG1_t1	1.27	0.002429816	0.16	126	12.18	2324	0.09	131	7.59	3936	NA	NA	NA	NA
RAB4A	1.25	3.77E-05	0.15	130	12.18	2325	0.1	131	8.04	3722	0.23	139	20.51	1199
EOGT	1.02	0.120465559	0.15	132	12.17	2326	0.1	148	10.13	2816	0.19	153	19.17	1414
PCDHB10	1.12	2.81E-06	0.14	135	12.17	2327	0.21	134	17.5	745	0.19	127	14.51	2456
POLR2D	1.25	6.38E-13	0.15	131	12.17	2328	0.14	127	10.54	2660	0.24	127	18.33	1587
TOR1AIP2_t1	1.12	2.30E-05	0.15	130	12.16	2329	0.12	135	10.61	2626	0.22	135	18.82	1500

PRUNE_t1	1.23	6.14E-11	0.16	128	12.16	2330	NA	NA	NA	NA	0.2	137	17.22	1782
BTBD7_t1	1.13	0.006332077	0.12	149	12.16	2331	0.13	134	10.85	2531	0.17	140	15.31	2242
CARD18	1.2	1.91E-07	0.18	117	12.16	2332	-0.03	113	-1.93	9759	0.2	118	13.56	2720
CCDC91	1.18	0.001553597	0.17	120	12.15	2333	0.12	131	9.72	2969	0.31	113	19.5	1347
RPIA	1.19	1.84E-06	0.14	137	12.15	2334	0.33	136	28.75	16	0.12	125	9.36	4096
DPY19L2	1.08	7.04E-06	0.17	123	12.14	2335	0.09	120	6.63	4435	0.2	129	15.7	2153
IL17RD	1.04	7.50E-06	0.13	141	12.14	2336	0.09	136	7.91	3789	0.19	150	18.85	1490
E2F5	1.44	8.37E-12	0.15	130	12.14	2337	0.23	133	19	520	0.24	141	21.61	1032
ESYT2	1.09	0.023445654	0.14	138	12.14	2338	0.13	141	11.66	2248	0.14	136	11.84	3254
GID8	1.33	3.98E-09	0.15	130	12.13	2339	0.09	124	6.61	4443	0.15	139	13.21	2827
CRTC2	1.39	1.37E-15	0.24	100	12.13	2340	0.23	106	13.02	1826	0.28	124	20.88	1141
FIBP_t2	1.27	6.75E-14	0.24	101	12.13	2341	0.11	124	7.85	3807	0.19	121	13.47	2749
CCDC164	1.12	6.30E-08	0.25	99	12.12	2342	0.19	86	6.95	4272	0.27	104	14.72	2400
NDUFAF2	1.48	1.01E-19	0.24	100	12.12	2343	0.22	85	7.61	3929	0.35	104	18.72	1522
TMA16	1.1	0.049427065	0.14	135	12.12	2344	0.14	123	9.88	2901	0.16	118	10.58	3663
SEMA5B	1.47	2.94E-13	0.18	117	12.11	2345	0.22	134	18.53	582	0.24	120	16.54	1945
CSNK2A1	1.22	2.30E-11	0.17	121	12.11	2346	0.19	128	14.79	1334	0.22	114	13.85	2630
FAM127A	1.09	0.340540172	0.18	116	12.11	2347	0.19	122	13.68	1632	0.18	116	12.1	3164
MTAP	1.08	0.003683266	0.15	132	12.11	2348	0.13	142	11.7	2231	0.23	133	19.12	1430
JAG2	1.27	1.83E-09	0.14	135	12.1	2349	0.2	115	13.26	1753	0.17	123	12.45	3048
CPSF1	1.09	0.013855642	0.15	129	12.1	2350	-0.03	98	-1.58	9544	0.06	110	3.52	6663
UBE2W_t1	1.08	0.023925544	0.12	147	12.09	2351	NA	NA	NA	NA	NA	NA	NA	NA
MRPL10	1.28	2.06E-12	0.15	131	12.09	2352	0.15	131	11.99	2130	0.13	119	9.15	4170
ATP11B	1.07	0.046973949	0.13	140	12.09	2353	0.1	124	7.65	3908	0.14	157	15.16	2286
TRIM9_t1	1.04	0.000518443	0.14	135	12.09	2354	0.05	132	4.17	5801	0.22	132	17.89	1666
DKK2	1.08	9.08E-05	0.14	134	12.08	2355	0.12	125	9.36	3119	0.1	121	7.14	4976
TMEM67	1.1	8.91E-08	0.13	140	12.08	2356	0.2	140	17.58	737	0.25	134	21.01	1117
SLC26A6	1.7	2.16E-15	0.24	100	12.07	2357	0.21	123	15.53	1135	0.31	100	15.37	2226
CGRRF1_t1	1.17	1.89E-07	0.14	137	12.07	2358	NA	NA	NA	NA	0.24	111	14.47	2471
DENND5A	1.19	8.49E-07	0.15	133	12.07	2359	0.21	132	17	841	0.07	114	4.66	6093
OTUD7B	1.15	3.56E-13	0.16	126	12.07	2360	0.17	166	19.64	437	0.26	143	23.73	762
MED1	1.16	0.00054184	0.17	122	12.07	2361	0.17	137	14.89	1309	0.14	127	10.59	3659
ZBTB33_t1	1.24	0.00324564	0.11	157	12.07	2362	NA	NA	NA	NA	NA	NA	NA	NA
PALB2	1.14	2.29E-08	0.17	122	12.07	2363	0.18	118	11.95	2141	0.26	112	16.27	2012
PSMD8	1.18	1.46E-07	0.17	119	12.06	2364	0.06	127	4.62	5538	0.09	126	6.7	5155
RAB6B_t1	1.15	3.89E-05	0.15	130	12.05	2365	NA	NA	NA	NA	0.18	139	15.81	2127
LAMA1_t1	1.05	6.77E-07	0.1	169	12.05	2366	NA	NA	NA	NA	0.16	132	13.39	2765
ZNF571	1.04	0.150310259	0.19	114	12.05	2367	0.1	99	4.85	5426	0.09	113	5.55	5658
ISG20L2	1.28	3.21E-11	0.17	120	12.05	2368	0.09	133	7.29	4096	0.14	121	10	3878
RPL23A_t1	1.16	0.000239429	0.18	116	12.05	2369	NA	NA	NA	NA	NA	NA	NA	NA
LGALSL	1.4	6.04E-09	0.15	130	12.05	2370	0.14	124	10.39	2724	0.27	147	26.25	477
CA5B	1.22	0.000292874	0.14	139	12.05	2371	0.2	142	18.32	613	0.11	147	10.5	3690
EBNA1BP2	1.41	9.52E-09	0.21	108	12.05	2372	0.15	122	10.97	2485	0.31	106	17.45	1746
YWHAB	1.19	0.000108768	0.15	129	12.04	2373	0.25	126	18.73	554	0.19	137	16.56	1939
TLK2	1.09	0.000359716	0.15	132	12.04	2374	0.06	140	5.72	4943	0.13	127	9.95	3893
NR0B1	1.14	9.36E-05	0.26	96	12.04	2375	0.13	95	6	4783	0.22	113	13.71	2674
PDE6D	1.18	6.66E-09	0.21	108	12.04	2376	0.1	132	8.22	3634	0.17	107	9.91	3910
BEND7	1.07	0.062464135	0.17	120	12.04	2377	0	119	0.04	8437	0.17	127	13.06	2865
ABI2	1.21	9.32E-06	0.14	135	12.03	2378	0.16	136	13.36	1727	0.08	134	7.07	5001
USP10	1	0.883715771	0.15	131	12.03	2379	0.08	146	7.99	3752	-0.02	151	-2.44	9917
STRADA	1.15	7.35E-14	0.18	118	12.03	2380	0.14	133	11.46	2317	0.12	139	10.65	3635
DCLRE1C	1.22	5.31E-05	0.14	135	12.03	2381	0.2	132	16.73	891	0.15	160	16.44	1972
SIRT7	1.55	2.36E-19	0.17	120	12.03	2382	0.21	106	11.78	2195	0.23	142	21.29	1074
NFU1	1.17	3.03E-05	0.15	130	12.02	2383	0.18	96	8.3	3592	0.21	120	14.72	2405
ALDH18A1	1.44	3.47E-13	0.16	127	12.01	2384	0.25	126	19.19	487	0.23	115	14.76	2393
LBR	1.24	0.0005928	0.14	138	12.01	2385	0.13	127	10.26	2776	0.14	154	15.04	2325
BAZ1A	1.07	0.149608413	0.13	139	12.01	2386	0.09	124	6.58	4463	0.12	124	8.95	4247
BID	1.17	0.005425359	0.12	149	12.01	2387	0.14	120	9.63	3009	0.16	120	11	3513
FTSJ1_t1	1.16	2.20E-16	0.17	120	12.01	2388	0.07	104	3.71	6055	0.17	104	9.24	4141
YIPF3	1.29	1.93E-09	0.19	114	12.01	2389	0.1	124	7.06	4217	0.24	104	12.69	2976
FAM45A_t1	1.23	6.42E-05	0.12	150	11.99	2390	NA	NA	NA	NA	0.25	142	22.84	859
GSG2	1.11	8.47E-09	0.18	118	11.99	2391	0.12	107	6.61	4449	0.29	100	14.39	2491
HAS3_t1	1.13	1.18E-06	0.11	155	11.98	2392	0.11	150	10.51	2675	0.09	143	8	4621
MAP2_t1	1.67	1.18E-13	0.12	150	11.98	2393	0.21	140	18.47	590	NA	NA	NA	NA
INADL	1.09	0.023839279	0.14	134	11.97	2394	0.06	120	3.89	5953	0.22	125	16.52	1952
MNF1	1.51	5.41E-17	0.21	108	11.97	2395	0.2	126	14.82	1323	0.26	114	16.71	1899
PDCD6	1.4	7.24E-14	0.18	117	11.97	2396	0.25	116	16.71	894	0.28	114	17.61	1715
SNRPA	1.28	7.75E-18	0.25	98	11.97	2397	0.19	105	10.47	2691	0.21	136	18.34	1585
ARF3_t1	1.07	0.015432428	0.17	119	11.97	2398	NA	NA	NA	NA	0.07	140	6.58	5196
LRRC20	1.83	1.90E-12	0.15	129	11.97	2399	0.03	126	2.51	6822	0.28	127	21.65	1023
ZNF714_t1	1.12	0.072454873	0.12	149	11.97	2400	NA	NA	NA	NA	NA	NA	NA	NA
XXYLT1	1.06	0.000947935	0.13	141	11.96	2401	0.14	139	12.16	2083	-0.02	120	-1.33	9356
AGGF1	1.31	3.37E-09	0.17	121	11.96	2402	0.18	110	10.53	2662	0.38	134	31.99	162
STK39_t1	1.46	1.94E-07	0.12	148	11.96	2403	NA	NA	NA	NA	0.05	140	4.77	6040
JTB	1.36	9.77E-12	0.17	119	11.96	2404	0.1	125	7.34	4073	0.27	143	25.21	595
SMG5	1.72	6.93E-25	0.2	111	11.95	2405	0.24	127	18.72	555	0.26	119	18.27	1596
FBXO28	-1.06	0.089541847	0.14	133	11.95	2406	-0.07	137	-6.44	12389	0.12	162	13.5	2740
CXCL17	1.06	0.000618861	0.15	132	11.95	2407	0	131	0.23	8310	0.14	122	10.21	3796
GGT6	1.15	0.000980872	0.15	131	11.94	2408	0.16	139	14.57	1385	0.18	120	12.64	2998
ZNF643	1.21	2.00E-11	0.28	93	11.93	2409	0.2	85	7.15	4164	0.33	111	20.31	1227
GPR107	1.25	8.32E-09	0.14	134	11.93	2410	0.28	153	28.69	18	0.13	135	11.2	3446
TTLL4	1.35	3.22E-10	0.19	112	11.93	2411	0.24	118	16.66	905	0.11	134	9.48	4060
RBM33	1.03	0.003613902	0.13	139	11.92	2412	0.08	142	7.36	4059	0.21	150	21.04	1114
TNNI3K	1.02	0.002706404	0.15	130	11.92	2413	0.08	113	5.03	5313	0.12	119	8.12	4573
NDUFA4	1.3	6.37E-08	0.11	158	11.92	2414	0.05	127	4.01	5902	0.3	134	25.17	600
UTP14A	1.22	6.62E-11	0.17	121	11.92	2415	0.06	104	3.27	6341	0.23	121	16.09	2052
CIRH1A_t1	1.12	0.00098804	0.17	121	11.91	2416	NA	NA	NA	NA	0.05	115	3	6927
ITPKA	2.21	5.31E-16	0.22	104	11.91	2417	0.06	117	4.12	5835	0.27	88	10.41	3719
ADIPOR1	1.2	1.16E-07	0.17	121	11.91	2418	0.06	129	4.6	5558	0.17	114	11.11	3470
SUPT4H1_t1	1.25	2.17E-09	0.18	115	11.91	2419	NA	NA	NA	NA	0.24	127	18.3	1592
TDRKH	1.48	2.40E-23	0.22	104	11.9	2420	0.18	110	10.52	2671	0.34	95	15.26	2259
CDCP1_t1	1.01	0.359683447	0.14	138	11.9	2421	0.08	149	8.14	3672	0.03	138	2.62	7124
GEMIN8P4	1.19	2.25E-13	0.17	121	11.89	2422	0.12	121	8.8	3374	0.2	132	16.19	2038
ZNF48	1.29	1.84E-09	0.16	123	11.88	2423	0.1	115	6.2	4663	0.22	126	16.44	1971
DAND5	1.16	7.35E-10	0.13	141	11.88	2424	0.06	141	5.54	5044	0.15	135	12.99	2885
NACC2	1.15	0.000290119	0.12	149	11.87	2425	0.23	120	15.85	1079	0.06	146	6.19	5362
ASIC1	1.45	3.74E-07	0.16	124	11.87	2426	0.07	145	6.48	4526	0.2	158	21.31	1071
CCDC142	1.4	1.12E-18	0.15	129	11.87	2427	0.11	135	9.68	2983	0.23	160	25.44	568
SPTSSA	1.28	0.004096699	0.12	145	11.87	2428	-0.03	121	-2.17	9942	0.17	150	16.98	1831
ZNF398	1.27	1.36E-08	0.15	131	11.86	2429	0.1	141	9.15	3206	0.17	125	12.44	3053
RPL19	1.34	3.08E-11	0.24	99	11.86	2430	0.26	101	13.4	1715	0.24	88	9.14	4175
TMEM50B	1.25	6.04E-09	0.13	139	11.86	2431	0.19	139	16.54	934	0.2	155	20.85	1147
JMJD4	1.17	4.07E-08	0.18	116	11.85	2432	0.03	122	2.18	7019	0.16	120	11.41	3378
PRELID1	1.24	2.08E-10	0.16	122	11.84	2433	0.15	132	12.27	2042	0.16	144	15.21	2268
NUP160_t1	1.22	2.80E-10	0.14	132	11.83	2434	NA	NA	NA	NA	0.22	124	16.5	1954
RPL37A_t1	1.36	9.93E-11	0.2	108	11.83	2435	0.3	124	21.87	227	NA	NA	NA	NA

N6AMT1	1.22	7.84E-11	0.16	125	11.83	2436	0.1	128	7.47	4006	0.15	135	12.96	2897
PDSS1	1.51	9.58E-15	0.2	110	11.83	2437	0.17	118	11.63	2265	0.31	122	22.14	945
CALN1_t1	1.07	0.01085644	0.14	136	11.82	2438	0.02	155	2.13	7059	NA	NA	NA	NA
ASUN	1.14	0.001950243	0.14	132	11.82	2439	0.24	108	13.86	1578	0.22	133	18.11	1623
EIF3B	1.38	8.14E-20	0.2	108	11.82	2440	0.19	113	12.23	2053	0.22	114	13.84	2634
FKBP9_t1	1.23	6.10E-11	0.16	122	11.81	2441	NA	NA	NA	NA	0.24	134	19.75	1314
SLC12A6	1.03	0.179296933	0.13	139	11.81	2442	-0.02	159	-1.95	9772	0.08	128	6.44	5262
NAA20	1.45	5.66E-17	0.16	123	11.81	2443	0.24	108	13.91	1556	0.24	130	18.8	1504
CCDC11	1.21	1.64E-08	0.17	121	11.8	2444	0.14	110	8.38	3548	0.23	107	12.94	2909
COX6B1	1.28	1.76E-11	0.19	111	11.8	2445	0.16	93	6.99	4250	0.2	84	6.65	5174
TADA2A_t1	1.13	6.71E-10	0.26	95	11.8	2446	0.07	95	3.23	6352	0.21	70	4.23	6328
ZNF696	1.28	1.93E-12	0.14	135	11.79	2447	0.08	151	8.47	3520	0.18	150	18.08	1628
BRMS1L	1.07	0.074434966	0.15	129	11.79	2448	0.05	119	3.29	6328	0.2	138	17.32	1767
VAC14_t1	1.1	1.92E-05	0.16	124	11.79	2449	NA	NA	NA	NA	0.11	104	6.17	5372
DCX	1.11	5.87E-10	0.14	134	11.78	2450	-0.01	128	-1.13	9243	0.07	143	6.19	5364
SNAP47	1.26	1.42E-08	0.22	104	11.78	2451	0.07	115	4.61	5548	0.23	113	14.78	2387
MAGEC2	1.9	2.82E-09	0.15	128	11.78	2452	0.13	102	6.66	4416	0.27	126	20.65	1177
TCF15	1.65	1.41E-16	0.2	108	11.77	2453	0.11	127	8.69	3415	0.25	101	12.55	3026
GATC	1.27	9.62E-10	0.17	120	11.77	2454	0.12	135	10.02	2855	0.17	133	13.91	2615
LETM2	1.08	1.51E-11	0.12	152	11.76	2455	0.03	118	1.78	7292	0.12	111	7.56	4809
METTL5	1.32	3.78E-12	0.24	98	11.76	2456	0.21	71	4.5	5612	0.31	72	6.74	5130
IPO11	1.04	0.001359965	0.13	144	11.76	2457	0	124	-0.07	8529	0.2	110	12.02	3190
ARL5A	1.03	0.484696439	0.12	149	11.76	2458	0.1	143	9.64	3004	0.11	137	9.29	4123
NABP2	1.27	1.65E-15	0.21	107	11.76	2459	0.2	151	20.18	380	0.29	156	30.72	210
ZIC1_t1	1.4	0.017980353	0.14	137	11.76	2460	-0.04	128	-2.78	10328	NA	NA	NA	NA
GSC	1.06	0.001477021	0.16	123	11.75	2461	0.04	105	2.44	6863	0.18	123	13.22	2824
MTL5_t1	1.07	2.10E-06	0.16	125	11.74	2462	0.15	142	13.49	1688	0.18	129	14.38	2493
IPMK	1.05	0.000523844	0.13	142	11.74	2463	0.06	140	5.6	5007	0.22	146	20.91	1130
MBD2_t1	1.11	4.62E-05	0.14	132	11.74	2464	0.15	96	6.93	4282	0.15	139	13.71	2671
PHF19_t1	1.52	1.85E-14	0.24	98	11.73	2465	0.13	100	6.53	4493	0.22	80	6.56	5206
POLR2G	1.3	1.34E-13	0.21	107	11.73	2466	0.29	140	26.47	46	0.23	95	10.21	3793
LRRC1	1.8	2.54E-09	0.17	121	11.73	2467	0.3	123	21.89	225	0.15	134	12.77	2956
ADAT1_t1	1.06	0.007508032	0.13	140	11.72	2468	NA	NA	NA	NA	0.09	153	9.19	4162
NTPCR	1.39	2.53E-13	0.19	111	11.72	2469	0.02	109	1.06	7764	0.23	104	12.33	3094
BAK1	1.2	1.32E-05	0.11	153	11.71	2470	0.15	135	12.94	1852	0.03	126	2.01	7444
BCAS2	1.18	0.000103816	0.17	118	11.71	2471	0.07	131	5.42	5108	0.26	114	16.81	1869
GNAS_t1	1.05	6.23E-06	0.16	123	11.69	2472	0.16	124	12.09	2104	0.17	113	10.99	3514
HS2ST1	1.12	4.24E-07	0.14	135	11.69	2473	0.16	126	12.21	2061	0.24	152	24.79	639
HOMEZ	1.23	7.45E-12	0.17	120	11.68	2474	0.14	129	11.4	2335	0.16	117	10.8	3572
PLOD3	1.48	1.42E-19	0.22	102	11.68	2475	0.14	112	8.79	3380	0.33	104	17.81	1681
DYNC1LI2	1.01	0.9209502	0.17	120	11.67	2476	0.05	124	3.59	6117	0.1	163	11.04	3500
CCDC66	1.11	0.001505604	0.16	125	11.67	2477	0.15	109	9.09	3234	0.26	111	15.57	2186
GALNT1	1.06	0.326259357	0.14	136	11.67	2478	0.08	131	6.47	4532	0.11	142	10.08	3846
SEC23IP	1.01	0.576233367	0.11	155	11.66	2479	0.13	140	11.62	2270	0.09	142	8.69	4348
DAGLA	1.25	3.02E-08	0.17	120	11.66	2480	0.22	127	17.06	824	0.15	131	12.37	3081
DDX4	1.05	1.50E-05	0.12	145	11.66	2481	0.14	127	10.72	2583	0.24	137	20.59	1187
HSPH1	1.44	0.000126643	0.17	118	11.66	2482	0.2	125	15.23	1217	0.22	127	16.62	1924.5
HTR2C	1.07	1.36E-05	0.12	148	11.66	2483	-0.02	125	-1.7	9619	0.16	155	16.42	1979
PRR5L_t1	1.02	0.126249516	0.13	137	11.65	2484	NA	NA	NA	NA	-0.02	152	-1.8	9609
TXLNA	1.16	9.63E-09	0.15	126	11.65	2485	0.14	138	12.71	1924	0.18	142	16.92	1850
MSI1	1.05	0.000177486	0.13	137	11.65	2486	-0.01	122	-1.03	9161	0.02	115	1.2	7912
ZBTB26	1.07	2.85E-06	0.18	116	11.65	2487	0.19	139	16.98	849	0.11	130	8.8	4304
WDR53	1.25	1.11E-18	0.22	104	11.64	2488	0.06	91	2.35	6922	0.25	79	7.25	4925
ATP5F1	1.08	0.137140419	0.14	136	11.64	2489	0.05	135	4.55	5584	0.21	122	14.79	2386
FAM171B_t1	1.01	0.311145865	0.15	127	11.63	2490	NA	NA	NA	NA	NA	NA	NA	NA
TPRX1	1.11	1.32E-14	0.12	147	11.63	2491	0.05	115	3.48	6192	0.22	108	12.79	2953
ICT1	1.45	1.54E-16	0.19	110	11.63	2492	0.16	102	8.56	3471	0.29	114	18.65	1531
IFT52	1.15	1.08E-09	0.22	104	11.62	2493	0.18	144	17.06	825	0.19	111	11.41	3379
COL21A1	1.14	0.000112449	0.15	130	11.62	2494	0.11	130	8.74	3397	0.21	135	17.95	1657
RPN2	1.37	2.12E-12	0.18	116	11.62	2495	0.06	115	3.65	6084	0.26	100	12.98	2889
ZNF419	1.16	1.06E-09	0.21	105	11.62	2496	0.2	127	15.6	1120	0.17	112	10.57	3666
NECAB1	1.22	0.000102806	0.12	145	11.62	2497	0.12	142	11.15	2418	0.15	137	13.36	2778
AGTPBP1	1.07	0.077275514	0.2	109	11.62	2498	0.18	124	13.62	1644	0.21	123	15.28	2255
HTATIP2	1.35	1.75E-05	0.15	128	11.62	2499	0.12	123	8.82	3369	0.18	110	10.64	3640
BRD9	1.21	1.37E-10	0.14	131	11.62	2500	0.14	109	8.5	3496	0.16	135	13.5	2736
PRPF31	1.26	1.51E-13	0.2	107	11.61	2501	0.11	90	4.57	5577	0.22	91	9.08	4194
GMDS	1.16	0.003181549	0.12	145	11.61	2502	-0.02	122	-1.35	9399	0.06	115	3.85	6499
PPHLN1_t1	1.06	1.79E-08	0.17	118	11.6	2503	0.09	107	5.4	5118	0.18	132	14.55	2447
SUPT7L	1.2	1.06E-07	0.15	129	11.6	2504	0.17	126	13.19	1777	0.16	135	13.68	2684
FOXE1	1.06	0.000180281	0.13	136	11.6	2505	-0.03	135	-2.39	10073	0.12	133	10.05	3855
ZNF552	1.22	1.33E-09	0.14	135	11.59	2506	0.15	122	10.47	2690	0.19	122	13.68	2680
AAK1	1.07	1.27E-07	0.12	148	11.58	2507	0.21	148	20.41	350	0.15	160	15.96	2082
GABPB1_t1	1.21	9.68E-07	0.24	99	11.58	2508	0.21	88	7.98	3758	0.25	107	14.25	2535
DMRT3_t1	1.04	0.003553038	0.13	141	11.58	2509	NA	NA	NA	NA	NA	NA	NA	NA
TUBB2A_t1	1.36	2.42E-07	0.17	120	11.58	2510	0.08	110	5	5337	NA	NA	NA	NA
NUCKS1	1.23	2.51E-07	0.1	161	11.57	2511	0.11	136	9.45	3091	0.2	168	24.08	710
NOV	1.43	4.82E-05	0.15	126	11.57	2512	0.13	123	9.29	3149	0.19	172	23.42	796
BICD1	1.12	2.23E-08	0.15	127	11.57	2513	0.17	163	18.71	557	0.14	136	12.41	3067
SF3B1_t1	1.06	0.001026474	0.2	109	11.57	2514	0.13	110	7.53	3973	0.14	106	7.84	4690
ISY1	1.08	0.000621413	0.14	135	11.56	2515	0.02	135	1.75	7315	0.02	162	2.06	7421
SCIN	1.1	2.63E-07	0.14	134	11.56	2516	0.08	147	7.44	4017	0.06	117	4.27	6303
COCH	1.91	4.64E-10	0.17	119	11.56	2517	0.1	125	7.44	4012	0.2	121	13.86	2627
CDC7	1.48	1.84E-12	0.18	115	11.55	2518	0.21	128	16.26	991	0.28	131	22.39	911
ATP2B4_t1	1	0.955646193	0.16	123	11.55	2519	0.15	152	14.85	1318	0.08	134	6.37	5285
FOXD2	1.56	8.25E-11	0.18	114	11.55	2520	0.01	133	0.8	7941	0.25	117	16.5	1956
ERC2_t1	1.28	2.38E-06	0.11	157	11.55	2521	NA	NA	NA	NA	0.22	154	23.34	807
HAUS1	1.31	1.15E-09	0.2	108	11.55	2522	0.14	101	7.01	4241	0.24	130	19.12	1431
SOCS4	1.05	1.11E-06	0.15	125	11.54	2523	0.12	131	9.44	3092	0.18	146	16.99	1825
BRE	1.18	1.29E-10	0.16	120	11.54	2524	0.09	116	6.1	4728	0.24	120	16.65	1916
LRP2	1.06	0.014419806	0.16	120	11.54	2525	0.02	122	1.26	7635	0.17	151	16.81	1870
ZNF615	1.03	0.15145426	0.13	141	11.54	2526	0.13	133	10.48	2688	0.13	130	10.25	3778
NIPA1_t1	1.06	0.000978987	0.12	150	11.54	2527	NA	NA	NA	NA	NA	NA	NA	NA
BLOC1S4	1.23	2.34E-07	0.15	125	11.54	2528	0.13	123	9.34	3126	0.23	122	16.57	1937
WRB	1.25	4.46E-08	0.11	153	11.53	2529	0.06	118	4.08	5858	0.22	125	16.33	2001
GABPA	-1	0.895244993	0.14	130	11.53	2530	0.01	134	0.81	7938	0.14	131	11.09	3481
PAXIP1	1.22	4.72E-13	0.16	122	11.53	2531	0.16	121	11.09	2434	0.24	128	18.78	1508
APC_t1	1.06	1.86E-09	0.12	149	11.53	2532	NA	NA	NA	NA	NA	NA	NA	NA
PAPSS1	1.07	0.028470722	0.2	108	11.53	2533	0.23	134	19.66	436	0.11	120	7.76	4727
ICA1L_t1	1.05	0.000177248	0.21	104	11.52	2534	NA	NA	NA	NA	0.19	106	10.91	3547
PSMD4	1.75	8.22E-25	0.19	112	11.52	2535	0.2	74	4.88	5407	0.3	91	12.25	3124
EEA1_t1	1.09	0.000239415	0.14	132	11.52	2536	NA	NA	NA	NA	NA	NA	NA	NA
BTBD10	1.06	0.050664105	0.15	127	11.52	2537	0.12	130	9.61	3017	0.2	147	19.21	1406
PLCL1	1.04	0.003353161	0.12	150	11.51	2538	0.09	143	8.11	3687	0.1	136	8.36	4481
ERMAP	1.03	0.134920577	0.1	163	11.51	2539	0.02	131	1.61	7423	0	136	-0.01	8630
CPA4	1.1	2.95E-11	0.16	121	11.5	2540	0.07	139	6.53	4492	0.11	123	8.02	4610
HCFC1	1.08	0.00030934	0.17	119	11.5	2541	0.06	127	4.97	5356	0.13	132	10.66	3631

UBE2MP1	1.13	0.000113552	0.16	124	11.5	2542	0.03	112	1.67	7373	0.15	98	6.97	5046
REEP2	1.23	9.78E-07	0.19	110	11.5	2543	0.09	115	6.08	4737	0.13	127	10.17	3802
AURKB	1.28	5.47E-17	0.26	95	11.49	2544	0.14	101	7.27	4107	0.28	124	20.86	1146
LIMK2_t1	1.23	0.001294767	0.14	135	11.49	2545	0.11	121	7.69	3886	0	132	0.15	8517
HOXD9	1.45	2.30E-16	0.15	129	11.49	2546	0.12	124	8.64	3439	0.23	110	13.56	2721
DHX33	1.05	0.000133961	0.12	149	11.48	2547	0.09	135	7.73	3868	0.08	133	7.02	5029
FAM50A	1.47	6.10E-18	0.21	105	11.48	2548	0.17	108	9.86	2911	0.27	104	14.58	2436
PPIL6	1.14	1.56E-05	0.12	142	11.48	2549	0.12	136	10.33	2748	0.09	123	6.81	5099
FAM192A	1.17	1.05E-07	0.16	122	11.47	2550	0.07	138	5.78	4908	0.25	120	17.15	1799
SLC30A5_t1	1.07	6.74E-07	0.19	109	11.47	2551	0.14	111	8.35	3564	0.16	141	14.98	2341
TBC1D5_t1	1.1	7.76E-05	0.16	124	11.47	2552	NA	NA	NA	NA	0.2	127	15.63	2170
NUDCD3	1.11	5.47E-10	0.12	144	11.47	2553	0.06	136	5.14	5256	0.18	138	15.83	2120
ZNF721	1.02	0.808049704	0.12	143	11.47	2554	0.06	135	5.16	5245	0.1	153	10.64	3638
LRPPRC	1.24	3.24E-05	0.14	132	11.46	2555	0.23	139	20.27	371	0.26	158	28.54	328
LCORL	1.12	9.60E-12	0.15	127	11.46	2556	0.17	124	12.92	1858	0.3	142	27.21	412
ATOX1	1.39	1.69E-08	0.21	104	11.46	2557	0.03	107	1.92	7202	0.2	93	8.66	4361
STRA13	1.72	1.31E-14	0.15	124	11.46	2558	0.16	131	13.36	1729	0.21	120	14.87	2368
WWP2	1.09	0.000174627	0.14	130	11.46	2559	-0.08	128	-6.02	12195	0.09	116	6.06	5423
WWTR1	1.11	0.011386748	0.15	124	11.46	2560	0.12	135	10.34	2743	0.04	130	3.49	6671
RAB3B_t1	1.2	1.34E-07	0.12	144	11.46	2561	NA	NA	NA	NA	NA	NA	NA	NA
CAPZA1	1.4	2.84E-08	0.14	129	11.45	2562	0.3	146	28.52	19	0.2	137	16.99	1826
POLE2	1.6	1.26E-16	0.22	102	11.45	2563	0.12	98	5.76	4916	0.38	100	19.08	1436
NRD1	1.27	5.16E-08	0.27	92	11.45	2564	0.21	91	8.54	3484	0.28	89	10.75	3592
JARID2	1.13	5.73E-06	0.11	152	11.45	2565	0.22	141	19.63	441	0.1	151	9.93	3906
TWISTNB_t1	1.27	3.08E-09	0.14	131	11.45	2566	NA	NA	NA	NA	0.33	146	31.99	161
SAP30	1.35	4.02E-08	0.2	107	11.44	2567	0.17	115	10.91	2512	0.24	94	10.48	3697
SPAG5	1.97	7.92E-18	0.21	104	11.44	2568	0.18	99	8.63	3444	0.3	105	16.75	1889
VDAC1_t1	1.15	2.15E-05	0.15	124	11.43	2569	NA	NA	NA	NA	NA	NA	NA	NA
TTC4	1.13	7.68E-09	0.18	115	11.43	2570	0.04	116	2.92	6539	0.16	124	12.18	3145
TSPAN5	1.32	0.000495323	0.11	150	11.43	2571	0.18	120	12.4	2008	0.07	127	5.62	5611
HDDC2_t1	1.11	4.95E-08	0.19	110	11.43	2572	NA	NA	NA	NA	0.1	130	7.91	4657
AICDA	1.03	0.415815317	0.11	153	11.43	2573	-0.05	139	-4.01	11103	0.03	149	2.85	7015
CD63	1.28	1.04E-05	0.21	105	11.43	2574	0.26	102	13.41	1709	0.12	83	3.87	6486
BRMS1	1.34	3.78E-22	0.22	102	11.42	2575	0.2	120	13.78	1605	0.25	108	14.23	2541
STXBP3	1.04	0.572505488	0.16	122	11.42	2576	0.11	120	7.5	3985	0.1	122	6.98	5042
PA2G4_t1	1.25	1.10E-13	0.18	115	11.42	2577	NA	NA	NA	NA	NA	NA	NA	NA
MAS1	1.01	0.234605159	0.18	112	11.41	2578	0.02	92	0.69	8009	0.11	132	9.42	4081
RASL12	1.2	5.57E-08	0.16	120	11.41	2579	0.25	132	20.49	339	0.16	147	15.2	2271
CHMP3	1.11	0.031312872	0.14	129	11.4	2580	0.18	145	17.53	742	0.08	150	8.31	4489
FAM71F1	1.18	5.30E-05	0.16	120	11.4	2581	0.05	120	3.55	6147	0.15	107	8.67	4355
SLC25A35	1.21	8.50E-11	0.13	141	11.4	2582	0.05	137	4.39	5668	0.23	122	16.21	2030
BRCC3	1.08	0.216024644	0.13	136	11.39	2583	0.07	155	7.84	3809	0.14	135	11.65	3308
COPG2	1.22	5.82E-08	0.15	127	11.38	2584	0.21	146	20.53	334	0.16	115	10.23	3790
ZNF678	1.11	0.007099174	0.12	142	11.38	2585	0.21	143	19.61	446	0.15	135	12.43	3054
CCDC75	1.14	6.06E-06	0.14	131	11.38	2586	0.14	100	7.09	4202	0.23	136	19.5	1346
FSD1L	1.07	3.45E-06	0.15	128	11.37	2587	0.11	93	4.93	5379	0.14	68	2.48	7189
TOP3A	1.12	4.47E-06	0.13	136	11.37	2588	0.02	119	1.7	7349	0.19	117	12.84	2942
GREM1	1.09	0.078536401	0.14	131	11.37	2589	0.08	141	7.14	4177	0.08	134	6.74	5132
TMEM64	1.41	4.75E-08	0.11	150	11.37	2590	0	143	0.22	8317	0.28	163	32.07	158
PDCL	1.18	2.97E-08	0.14	129	11.36	2591	0.15	124	10.75	2576	0.14	121	9.69	3993
HSPA5	1.22	3.68E-05	0.13	138	11.36	2592	0.02	134	1.66	7378	0.18	124	13.66	2689
PABPC5	1.04	0.093098425	0.12	144	11.36	2593	0.04	130	2.82	6600	0.09	136	7.99	4625
GNRHR2_t1	1.11	6.55E-05	0.12	145	11.36	2594	NA	NA	NA	NA	0.15	141	14.05	2584
RAP2B	1.18	1.17E-12	0.12	143	11.36	2595	0.29	134	24.04	110	0.2	151	20.67	1174
CNGA3	1.02	0.007845759	0.13	138	11.36	2596	0.1	115	6.38	4572	0.13	130	10.53	3678
EIF2C1	1.1	0.003899328	0.14	130	11.36	2597	0.09	148	9.2	3189	0.04	157	4.79	6034
TOP1MT	1.39	4.59E-11	0.2	106	11.35	2598	0.15	105	8.17	3657	0.29	112	17.7	1699
RAB15	1.21	0.001736135	0.16	122	11.35	2599	0.03	131	2.74	6651	0.13	126	10.08	3844
RBP7	1.91	1.53E-13	0.23	100	11.35	2600	0.12	91	4.86	5416	0.32	118	21.99	968
LTN1	1.04	0.274447176	0.1	164	11.34	2601	0.04	124	2.73	6654	0.15	140	13.29	2796
RNF14	1.15	1.92E-05	0.11	152	11.34	2602	0.07	141	6.05	4751	0.27	134	22.88	856
PDGFB	1.1	6.21E-05	0.18	113	11.34	2603	0.15	146	14.38	1430	0.12	126	8.91	4257
UBE2V2	1.23	3.44E-06	0.13	137	11.34	2604	0.14	118	9.3	3145	0.2	128	15.85	2113
BARD1	1.36	7.26E-11	0.2	107	11.34	2605	0.15	120	10.37	2731	0.22	136	19.34	1376
HIST1H2BI	1.56	3.92E-14	0.23	100	11.33	2606	0.06	80	1.77	7299	0.2	84	6.95	5055
HPS1_t1	1.36	1.62E-11	0.2	106	11.33	2607	0.27	122	19.46	456	0.2	104	10.92	3538
POP5	1.37	1.88E-15	0.15	127	11.33	2608	0.12	126	9.09	3229	0.24	114	15.61	2177
MED31	1.07	0.030118812	0.14	133	11.32	2609	0.04	128	3.28	6332	0.07	108	3.81	6525
BDP1_t1	1.04	0.008562721	0.11	151	11.32	2610	0.13	130	10.53	2666	NA	NA	NA	NA
MAP3K7	1.13	0.002755429	0.11	151	11.31	2611	0.26	120	18.33	610	0.12	119	8.46	4442
IKBKAP	1.22	0.000786883	0.14	129	11.31	2612	0.17	143	15.77	1099	0.16	115	10.71	3611
EDEM3	1.2	0.014647999	0.11	154	11.31	2613	0.12	127	8.92	3319	0.12	141	10.74	3598
MFN1	1.15	6.72E-07	0.16	122	11.3	2614	0.16	130	12.91	1861	0.19	136	16.24	2020
ZMYM5_t1	1.08	0.004868633	0.17	118	11.3	2615	0.11	94	4.95	5374	NA	NA	NA	NA
SF3B4	1.11	2.72E-09	0.18	113	11.3	2616	0.11	120	7.77	3846	0.18	112	11.18	3449
HIST1H3F	1.41	7.99E-17	0.3	88	11.29	2617	0.21	79	6.19	4670	0.24	125	18	1650
MLL_t1	1.03	0.006699525	0.15	125	11.29	2618	NA	NA	NA	NA	NA	NA	NA	NA
AATF	1.42	7.81E-23	0.3	88	11.28	2619	0.15	100	7.62	3923	0.35	88	13.19	2832
TTBK2	1.09	2.21E-06	0.15	123	11.28	2620	0.12	129	9.56	3036	0.17	125	12.56	3017
SLC10A4	1.07	1.06E-07	0.1	166	11.28	2621	0.18	118	12.04	2122	0.18	129	14.5	2459
ZNF682_t1	1.14	1.80E-05	0.11	152	11.27	2622	NA	NA	NA	NA	0.12	143	10.82	3567
GLI4	1.15	1.72E-08	0.21	103	11.27	2623	0.09	113	5.84	4878	0.23	100	11.74	3287
IGSF1	1.27	7.83E-07	0.17	115	11.26	2624	0.06	100	2.93	6529	0.19	123	13.92	2612
ATE1	1.1	0.000703266	0.16	120	11.26	2625	0.03	111	2.05	7109	0.22	124	16.49	1959
FARSB_t1	1.35	2.85E-11	0.18	111	11.26	2626	0.25	104	13.48	1689	NA	NA	NA	NA
STK3	1.08	0.014622147	0.16	120	11.26	2627	0.12	132	9.98	2868	0.27	143	24.81	632
DEF8_t1	1.31	2.17E-10	0.23	99	11.25	2628	NA	NA	NA	NA	0.2	138	17.64	1711
RANBP3_t1	1.06	0.01158354	0.14	132	11.25	2629	0.07	118	4.77	5462	0.07	122	5.39	5739
RPA1	1.12	0.003345268	0.16	120	11.24	2630	0.1	138	9.01	3264	0.11	121	7.62	4782
CSNK1A1L	1.05	0.103092281	0.16	119	11.24	2631	0.11	111	6.75	4380	0.1	139	8.57	4391
NCS1	1.15	0.027664721	0.14	131	11.24	2632	0.2	141	17.97	669	0.09	122	6.77	5122
SLC25A21	1.07	0.003057927	0.15	126	11.24	2633	-0.04	119	-3.05	10505	0.18	159	19.45	1357
ACAD9	1.21	3.90E-12	0.18	114	11.24	2634	0.16	119	11.29	2364	0.17	104	9.25	4137
DUSP9	1.27	1.34E-06	0.16	122	11.23	2635	0.02	125	1.54	7471	0.07	146	6.34	5301
ELP3	1.03	0.544191666	0.14	130	11.23	2636	0	130	0.23	8309	0.02	122	1.53	7730
KDM1B	1.03	0.087199482	0.14	128	11.22	2637	0.07	125	5.37	5142	0.16	137	13.76	2661
MCM9	1.1	1.29E-05	0.15	123	11.21	2638	0.2	116	13.24	1756	0.12	129	9.47	4062
RAB4B	1.1	1.69E-06	0.16	120	11.21	2639	0.06	106	3.14	6415	0.11	95	4.92	5968
DDX55	1.15	7.09E-07	0.13	134	11.2	2640	0.19	140	16.88	865	0.17	110	10.02	3869
ZNF432	1.15	3.01E-05	0.17	115	11.2	2641	0.22	107	12.61	1951	0.15	113	9.4	4084
ZNF557_t1	1.32	1.13E-14	0.13	135	11.2	2642	NA	NA	NA	NA	0.16	144	15.38	2225
FAM3C	1.18	0.006547967	0.15	125	11.2	2643	0.15	126	11.6	2272	0.13	124	9.39	4090
EIF3H	1.37	9.05E-07	0.15	123	11.2	2644	0.26	99	12.52	1976	0.23	106	13.07	2862
HM13_t1	1.37	3.52E-16	0.21	104	11.2	2645	0.2	104	10.75	2574	0.2	128	15.92	2091
SPARCL1	1.96	4.10E-05	0.13	134	11.19	2646	0.14	132	11.26	2379	0.26	123	19.14	1421
PIF1	1.83	8.10E-18	0.17	115	11.19	2647	0.2	98	9.83	2924	0.28	124	20.43	1209

UBL5	1.24	5.39E-09	0.17	114	11.19	2648	0.13	107	7.36	4053	0.23	107	12.86	2937
HS3ST5	1.01	0.37577436	0.14	129	11.19	2649	0.04	132	3.08	6452	0.03	154	3.35	6744
MARK4	1.19	3.39E-09	0.16	122	11.19	2650	0.09	150	8.91	3323	0.19	123	13.62	2707
TUBA3D	1.55	4.45E-17	0.23	99	11.19	2651	0.19	88	7.27	4110	0.24	109	14.04	2587
CLVS1_t1	1.01	0.276848275	0.14	129	11.18	2652	NA	NA	NA	NA	NA	NA	NA	NA
ABCE1	1.06	0.054158518	0.12	141	11.17	2653	0.14	128	10.62	2623	0.18	152	18.05	1636
TRAF2	1.34	1.50E-12	0.16	121	11.17	2654	0.17	120	11.65	2254	0.12	100	5.98	5465
HIST1H2BK	1.68	2.80E-12	0.2	105	11.17	2655	0.05	114	3.51	6175	0.24	107	13.49	2743
HRAS	1.38	1.27E-16	0.21	102	11.16	2656	0.14	108	7.98	3756	0.2	100	10.02	3866
PUM2	1.13	0.005194975	0.15	125	11.16	2657	0.15	156	15.58	1124	0.16	137	13.94	2607
AP1G1	-1.06	0.124848161	0.12	142	11.16	2658	-0.06	138	-5.63	11992	0.07	150	6.86	5084
DDX31	1.07	4.00E-08	0.16	120	11.16	2659	0.2	141	18.51	583	0.15	106	8.24	4517
PHF20L1_t3	1.16	8.30E-07	0.17	115	11.16	2660	NA	NA	NA	NA	0.31	100	15.7	2152
MAGEA1	3.77	3.23E-15	0.16	121	11.16	2661	0.08	116	4.98	5350	0.29	111	17.4	1751
THAP5	1.09	1.10E-06	0.11	152	11.15	2662	0.12	136	10.46	2701	0.24	132	19.47	1355
NEDD1_t1	1.14	6.74E-07	0.14	129	11.15	2663	0.14	108	8.05	3716	NA	NA	NA	NA
THRB	1.07	0.079087582	0.11	147	11.15	2664	0	138	-0.4	8750	0.14	147	13.63	2700
TMEM132B_t1	1.03	0.004568325	0.12	143	11.15	2665	NA	NA	NA	NA	NA	NA	NA	NA
TMEM79	1.65	7.62E-25	0.21	103	11.15	2666	0.18	120	12.9	1863	0.31	104	16.71	1902
KIAA2018	1.02	0.05805506	0.11	149	11.15	2667	0.07	139	5.79	4897	0.2	143	18.37	1574
HIST1H2AM	1.64	3.20E-23	0.29	88	11.14	2668	0.21	80	6.18	4679	0.29	88	11.18	3450
SACS	1.1	0.000141179	0.1	162	11.14	2669	0.17	118	11.44	2320	0.18	143	16.97	1835
ANKRD28	1.04	0.000423412	0.11	152	11.13	2670	0.06	138	5.02	5319	0.2	153	20.1	1264
ZNF329	1.13	0.000707462	0.13	136	11.13	2671	0.15	135	12.4	2009	0.13	126	10.06	3853
NOC3L	1.11	0.013422036	0.14	130	11.13	2672	0.1	137	8.44	3533	0.19	116	12.29	3110
KIAA1919_t1	1.1	0.002327346	0.13	139	11.13	2673	NA	NA	NA	NA	NA	NA	NA	NA
PRDX1	1.19	0.000519987	0.16	121	11.13	2674	0.16	92	6.64	4425	0.12	125	9.05	4206
TRAPPC4	1.22	4.41E-06	0.16	120	11.13	2675	0.24	167	27.84	27	0.07	145	6.31	5315
MALT1	-1	0.843001014	0.1	163	11.13	2676	0.07	134	6.24	4641	0.11	151	10.94	3531
TNK2_t1	1.03	0.00423165	0.16	120	11.13	2677	0.04	121	2.61	6754	0.1	101	4.89	5979
ACTG1	1.48	1.14E-08	0.13	133	11.12	2678	0.27	119	18.97	524	0.17	136	14.37	2495
DMP1	1.04	1.22E-05	0.14	131	11.12	2679	0.11	110	6.71	4396	0.15	126	11.47	3365
GTF2A2	1.22	1.21E-08	0.1	160	11.12	2680	0.12	118	8.23	3633	0.34	120	23.87	739
KLHL13	1.14	0.000633895	0.15	124	11.12	2681	-0.03	117	-2.32	10039	0.19	124	13.78	2652
DYNC1LI1_t1	1.25	1.47E-06	0.2	107	11.12	2682	NA	NA	NA	NA	0.28	125	20.68	1172
TMEM14C	1.34	1.25E-12	0.21	104	11.12	2683	0.19	107	10.57	2642	0.25	112	15.52	2195
YEATS4	1.22	3.75E-08	0.12	139	11.12	2684	0.14	111	8.83	3366	0.21	132	17.4	1750
NCAPD3	1.05	0.06791313	0.13	134	11.12	2685	0.02	108	1.39	7558	0.01	123	0.79	8140
IQCE	1.16	4.92E-10	0.15	124	11.11	2686	0.09	130	6.97	4262	0.19	128	14.82	2377
QRSL1_t1	1.17	3.84E-09	0.13	134	11.11	2687	NA	NA	NA	NA	0.11	136	9.39	4089
EXOSC2	1.17	9.69E-07	0.14	128	11.11	2688	0.22	147	20.86	306	0.14	122	10.23	3786
SLC39A10	1.29	2.64E-09	0.13	135	11.11	2689	0.21	129	16.67	902	0.22	143	20.78	1163
LSM6	1.08	0.015165053	0.17	114	11.11	2690	0.07	107	4.09	5851	0.09	117	5.93	5477
TTC30B	1.25	4.54E-10	0.17	114	11.1	2691	0.19	135	15.96	1057	0.24	126	18.37	1576
CHKA_t1	1.23	4.85E-07	0.12	139	11.1	2692	NA	NA	NA	NA	0.2	133	16.67	1909
YWHAH	1.27	5.84E-07	0.14	127	11.1	2693	0.26	129	20.49	342	0.15	123	11.31	3412
RNF139	1.1	0.0585516	0.15	124	11.1	2694	0.04	124	3.23	6358	0.23	122	16.34	2000
ZNF121	1.06	8.71E-08	0.12	144	11.1	2695	0.03	119	2.1	7078	0.15	107	8.61	4381
ZNF673_t1	1.09	0.008916096	0.17	114	11.1	2696	NA	NA	NA	NA	NA	NA	NA	NA
PRKCA	1.77	5.42E-13	0.13	134	11.1	2697	0.15	147	14.85	1317	0.27	133	22.59	885
ETAA1	1.12	0.003311096	0.13	137	11.09	2698	0.09	115	5.59	5016	0.22	113	13.64	2698
CCDC127	1.24	4.08E-10	0.16	119	11.09	2699	0.13	125	9.86	2912	0.11	121	8.04	4600
MTF2	1.08	0.071455011	0.12	144	11.09	2700	0.16	127	12.27	2043	0.1	128	7.47	4847
ZNF664	1.1	4.35E-11	0.13	134	11.09	2701	0.14	127	11.1	2429	0.3	119	20.38	1218
HEATR6	1.21	1.02E-15	0.2	105	11.09	2702	0.24	108	13.86	1577	0.1	118	7	5032
CLN3_t1	1.48	1.63E-18	0.21	104	11.09	2703	NA	NA	NA	NA	0.32	124	23.99	727
PSEN2_t1	1.13	5.62E-07	0.13	133	11.09	2704	-0.03	137	-2.81	10348	0.15	119	10.32	3751
HSPB1	1.99	4.01E-22	0.33	84	11.09	2705	0.21	104	11.58	2279	0.39	80	11.84	3252
PDE4B_t1	1.05	8.99E-06	0.11	155	11.09	2706	0.03	136	2.56	6792	0.13	151	13.03	2873
RFC4	2.26	2.22E-26	0.31	86	11.08	2707	0.24	71	5.03	5312	0.4	101	20.21	1246
INTS9	1.09	0.047811653	0.13	136	11.08	2708	0.12	108	6.77	4364	-0.02	104	-0.84	9104
BRPF3	1.22	9.68E-09	0.14	131	11.08	2709	0.09	142	8.48	3516	0.23	126	17.7	1700
IRF6	1.25	6.31E-05	0.13	134	11.08	2710	0.05	120	3.41	6242	0.14	135	12.1	3163
RPL7A	1.32	1.28E-08	0.18	110	11.08	2711	0.25	106	14.25	1464	0.19	88	7.05	5011
POLR3K	1.2	2.48E-07	0.14	127	11.07	2712	0.12	130	9.56	3033	0.07	141	5.92	5484
TBX19	1.36	2.56E-18	0.17	115	11.06	2713	0.17	145	16.37	965	0.26	164	30.15	233
RHBDD3	1.32	1.68E-13	0.18	112	11.06	2714	0.19	90	7.56	3956	0.2	88	7.55	4814
POLD3	1.17	1.38E-08	0.14	129	11.06	2715	0.13	135	11.07	2441	0.15	136	12.98	2890
PXMP4	1.37	4.81E-08	0.13	133	11.05	2716	0.04	150	3.76	6030	0.26	153	27.07	417
MPHOSPH9	1.19	2.03E-09	0.16	118	11.04	2717	0.2	135	17.3	779	0.21	154	21.7	1017
ULBP2_t1	1.13	2.14E-06	0.14	127	11.04	2718	0.09	110	5.36	5144	NA	NA	NA	NA
NHP2L1	1.2	2.23E-07	0.18	110	11.04	2719	0.22	112	13.59	1661	0.09	117	6.23	5344
RHO_t1	1.09	0.000601498	0.15	124	11.04	2720	NA	NA	NA	NA	0.08	120	5.82	5531
PMFBP1	1.23	2.39E-15	0.22	100	11.04	2721	0.13	106	7.14	4179	0.21	103	11.06	3494
MAP7D2	1.17	0.000286612	0.15	126	11.03	2722	0.09	141	8.4	3542	0.16	148	15.44	2208
Sep-14	1.39	1.21E-08	0.15	126	11.03	2723	0.06	131	4.78	5456	0.23	140	20.88	1140
CAD	1.48	1.10E-21	0.22	100	11.02	2724	0.16	133	13.14	1788	0.2	100	9.85	3931
BRWD3	1.07	2.24E-05	0.11	147	11.01	2725	0.18	138	15.41	1161	0.2	150	19.98	1273
TNFSF4	1.38	3.86E-09	0.16	120	11.01	2726	0.15	128	11.79	2193	0.23	131	18.84	1493
MMP11	2.07	8.87E-14	0.22	100	11.01	2727	0.22	115	14.54	1395	0.18	114	11.61	3322
GAD1_t2	1.33	2.01E-10	0.21	102	11	2728	-0.05	108	-2.72	10290	0.21	100	10.35	3743
HN1L	1.25	2.25E-06	0.14	128	11	2729	0.19	133	16.05	1031	0.14	121	9.98	3886
ZNF446	1.29	5.32E-11	0.17	115	11	2730	0.1	143	8.9	3327	0.19	127	14.87	2367
NDUFA1_t1	1.33	5.70E-08	0.18	110	11	2731	0.08	81	2.38	6898	NA	NA	NA	NA
MRPS10_t1	1.2	6.16E-08	0.15	121	10.99	2732	0.19	131	15.52	1139	NA	NA	NA	NA
KLHL5_t1	1.12	0.018571084	0.12	141	10.99	2733	NA	NA	NA	NA	0.26	130	21.05	1111
ZDHHC9	1.44	4.39E-15	0.12	139	10.99	2734	0.14	143	13.04	1818	0.26	131	21.35	1068
B3GAT2	1.1	1.62E-13	0.15	121	10.99	2735	0.27	104	14.36	1434	0.19	104	10.09	3840
ARPC4	1.08	0.000841266	0.17	113	10.98	2736	0.17	120	12.05	2117	0.09	116	5.93	5480
SLC7A6OS	1.24	5.42E-08	0.2	104	10.98	2737	0.17	104	9.17	3201	0.08	108	4.83	6011
SLC6A14_t1	1.03	0.008259871	0.14	130	10.98	2738	NA	NA	NA	NA	0.16	129	12.33	3102
SEMA3F	1.11	4.01E-08	0.18	112	10.97	2739	0.12	122	8.55	3480	0.22	100	11.15	3454
MARVELD2	1.16	9.22E-06	0.14	131	10.97	2740	-0.08	123	-6.03	12204	0.24	119	16.61	1929
CCDC135	1.15	4.22E-09	0.24	95	10.97	2741	0.07	130	5.62	4993	0.14	101	7.05	5010
POT1	1.17	1.60E-06	0.15	125	10.96	2742	0.09	126	6.61	4445	0.29	123	21.36	1067
ZNF514	1.16	5.10E-05	0.14	127	10.95	2743	0.16	135	13.29	1751	0.05	135	4.67	6087
SAMD8	-1.01	0.51346874	0.1	157	10.95	2744	0.01	143	1.17	7696	-0.03	166	-3.86	10572
GGNBP2_t1	1.2	5.51E-06	0.21	101	10.95	2745	NA	NA	NA	NA	0.28	141	25.66	543
ARCN1	1.05	0.000203789	0.12	142	10.95	2746	0.12	143	10.91	2511	0.11	137	9.76	3960
GATAD1	1.33	1.96E-10	0.12	138	10.94	2747	0.17	134	14.3	1454	0.19	132	15.2	2273
GTPBP4	1.32	5.45E-07	0.17	116	10.94	2748	0.19	112	11.74	2217	0.25	104	13.54	2725
HNRNPUL1_t1	1.21	1.96E-09	0.14	128	10.93	2749	0.2	126	15.29	1200	NA	NA	NA	NA
MDM1_t2	1.07	3.62E-12	0.15	122	10.93	2750	NA	NA	NA	NA	0.27	153	27.64	391
SMYD1	1.04	0.001937312	0.12	142	10.93	2751	0.15	123	11.09	2433	0.2	155	21.26	1076
IQCC	1.21	7.13E-15	0.2	104	10.93	2752	0.1	132	8.57	3463	0.3	121	21.17	1091
FAM86C1_t1	1.2	1.38E-08	0.16	120	10.93	2753	NA	NA	NA	NA	0.16	121	11.64	3313

PRPF4B	1.27	0.001614881	0.13	131	10.92	2754	0.25	146	23.64	125	0.19	142	17.12	1806
FLOT2_t1	1.1	0.032391964	0.18	111	10.92	2755	0.05	114	3.27	6340	NA	NA	NA	NA
PPIP5K2	1.11	0.080797703	0.16	120	10.92	2756	0.06	132	5.12	5267	0.23	139	20.83	1150
TTC7A_t1	1.09	0.002006296	0.15	124	10.92	2757	NA	NA	NA	NA	0.07	133	6	5450
APH1B	1.16	0.004358795	0.12	140	10.92	2758	0.16	149	15.45	1155	0.01	147	0.59	8266
RECQL	1.18	0.000648051	0.14	126	10.92	2759	0.23	128	18.1	644	0.16	124	12.13	3158
CNOT10	1.33	8.14E-13	0.17	115	10.91	2760	0.12	95	5.39	5130	0.24	113	14.95	2349
NPM1_t1	1.44	7.40E-07	0.17	116	10.91	2761	0.33	100	16.58	925	0.17	112	10.51	3683
TTI1	1.13	8.80E-13	0.22	100	10.9	2762	0.1	124	7.29	4092	0.28	107	16.1	2048
FDX1L	1.33	9.30E-15	0.19	108	10.9	2763	0.16	115	10.48	2686	0.2	108	11.44	3370
TRIP12_t1	1.03	0.328757913	0.13	135	10.9	2764	NA	NA	NA	NA	0.11	103	5.68	5591
CALM3	1.04	0.362843377	0.16	120	10.9	2765	0.07	150	6.91	4292	-0.06	134	-4.89	11012
KRT37	1.04	1.98E-05	0.16	118	10.89	2766	0.05	112	3.29	6331	0.06	141	5.61	5616
SLX4	1.2	1.49E-11	0.19	107	10.89	2767	0.22	125	16.71	893	0.19	120	13.47	2747
EBF1	1.12	0.000164471	0.13	135	10.89	2768	0.23	141	21.19	268	0.15	147	14.93	2354
TSEN34	1.12	0.002037412	0.18	112	10.89	2769	0.17	129	13.75	1612	0.03	151	3.29	6781
EDDM3A	1.08	0.003518269	0.14	129	10.89	2770	-0.06	120	-4.12	11160	0.17	100	8.67	4358
UBXN2A	1.13	2.18E-05	0.14	128	10.88	2771	0.12	126	8.74	3392	0.25	124	18.37	1573
RFC1_t1	1.17	0.000374035	0.15	122	10.87	2772	NA	NA	NA	NA	0.27	125	20.4	1213
ITGAV_t1	1.08	0.002740655	0.14	127	10.87	2773	NA	NA	NA	NA	0.12	136	10.49	3695
SUV420H2	1.11	3.42E-07	0.15	122	10.87	2774	0.08	131	6.6	4450	0.14	119	9.33	4108
DCLK1_t1	1.03	0.000322469	0.12	140	10.87	2775	NA	NA	NA	NA	0.18	131	14.46	2474
STRN	1.06	4.22E-08	0.12	141	10.87	2776	0.14	158	14.99	1281	0.22	150	21.5	1050
ZNF670	1.11	9.84E-05	0.13	136	10.87	2777	0.09	137	7.62	3924	0.16	125	12.25	3123
ZNF76	1.07	0.001039886	0.12	138	10.87	2778	0.01	120	0.36	8234	0.08	118	5.47	5704
SMR3B	1.07	0.000908463	0.23	98	10.86	2779	0.09	120	6.36	4584	0.2	109	11.52	3345
KLB	1.14	0.055084645	0.13	132	10.86	2780	0.07	135	5.73	4934	0.21	153	21.77	1008
RIPK2	1.15	0.019156246	0.12	138	10.86	2781	0.16	140	14.03	1524	0.16	118	10.71	3609
SMURF1_t1	1.11	4.96E-05	0.16	116	10.85	2782	NA	NA	NA	NA	NA	NA	NA	NA
SYT6_t1	1.09	2.11E-13	0.13	133	10.85	2783	NA	NA	NA	NA	NA	NA	NA	NA
GUF1	1.14	2.79E-05	0.12	141	10.85	2784	0.09	125	6.59	4458	0.19	134	16.05	2061
ZFYVE16	1.01	0.162376778	0.12	137	10.85	2785	0.08	120	5.9	4847	0.24	131	19.3	1385
FANCD2_t2	1.08	5.93E-08	0.12	144	10.85	2786	0.14	120	9.81	2929	0.11	128	8.46	4439
ZNF718	1.09	1.87E-07	0.12	144	10.85	2787	0.08	115	5.46	5081	0.14	136	11.7	3297
CRIPT	1.25	1.21E-08	0.16	118	10.84	2788	0.14	127	10.66	2614	0.26	121	18.47	1557
PSMA1	1.17	0.000290303	0.2	105	10.84	2789	0.19	106	10.41	2714	0.26	108	14.85	2375
GPN2	1.21	9.71E-09	0.25	94	10.84	2790	0.11	108	6.63	4430	0.18	100	9.23	4150
ZMYM4	1.05	0.271391606	0.13	136	10.84	2791	0.17	124	12.21	2062	0.16	131	13.19	2831
NMT1	1.18	4.00E-08	0.12	144	10.84	2792	0.05	120	3.82	5994	0.1	139	8.52	4419
DMXL2	1.04	0.030710216	0.15	120	10.83	2793	0.13	130	10.56	2646	0.17	131	13.43	2755
RUFY2_t1	1.16	1.69E-07	0.15	120	10.83	2794	0.12	136	10.44	2707	0.19	154	19.73	1317
DHX16	1.35	7.79E-21	0.18	109	10.83	2795	0.11	96	5.09	5277	0.27	86	9.7	3986
NPM1_t2	1.21	0.003522288	0.13	136	10.83	2796	0.26	117	17.28	784	0.1	123	7	5031
CNOT2	1	0.941144483	0.13	132	10.82	2797	0.09	120	6.17	4687	0.16	132	12.98	2888
HMGCS1_t1	1.48	0.000119599	0.11	148	10.82	2798	NA	NA	NA	NA	0.19	134	15.62	2172
PRDM13	1.09	0.00090643	0.14	125	10.81	2799	0.08	110	5.08	5281	0.11	150	11.12	3464
PYGB	1.47	3.82E-11	0.14	126	10.81	2800	0.18	131	14.66	1363	0.11	139	9.92	3909
ZMYND19	1.31	5.77E-14	0.21	101	10.81	2801	0.18	123	13.45	1698	0.16	100	8.12	4571
SLC4A11_t1	1.2	0.000237841	0.17	113	10.8	2802	0.03	109	1.86	7235	NA	NA	NA	NA
ANO8	1.2	1.25E-08	0.18	109	10.8	2803	0.07	107	3.74	6037	0.14	104	7.3	4917
CHCHD6	1.19	5.11E-09	0.15	123	10.8	2804	0.1	104	5.43	5104	0.15	104	8	4623
SNHG1	1.34	3.17E-08	0.15	121	10.8	2805	0.17	125	13.03	1819	0.13	127	9.91	3911
HNRNPR	1.28	5.45E-08	0.19	106	10.8	2806	0.24	131	19.11	499	0.3	131	24.06	715
FDFT1	1.27	0.000346085	0.15	121	10.8	2807	0.06	104	3.06	6463	0.14	120	9.96	3892
GRPR	1.32	4.97E-08	0.11	148	10.8	2808	0.07	159	7.42	4023	0.17	122	11.94	3219
STARD4	1.1	0.082773538	0.14	126	10.8	2809	0.06	135	4.73	5484	0.16	125	11.89	3240
AIFM2_t1	1.43	8.21E-10	0.14	127	10.8	2810	NA	NA	NA	NA	NA	NA	NA	NA
DUSP13	1.14	8.20E-07	0.16	117	10.79	2811	0.12	130	9.6	3021	0.11	123	7.95	4638
PDZD2	1.1	0.000235073	0.11	149	10.79	2812	0.09	133	7.1	4200	0.15	131	11.91	3232
TMEM106B	1.28	8.15E-07	0.11	150	10.79	2813	0.13	139	11.2	2398	0.24	133	20.28	1233
KIAA0100	1.3	7.36E-10	0.17	115	10.78	2814	0.17	120	12.14	2088	0.14	96	6.57	5203
LIN54	1.04	0.000592481	0.11	145	10.77	2815	0.09	139	8.25	3623	0.21	131	17.25	1778
CIT	1.39	9.07E-06	0.14	128	10.77	2816	0.13	134	10.55	2657	0.14	144	13.35	2782
GPATCH1	1.22	4.22E-14	0.22	100	10.77	2817	0.08	123	5.96	4809	0.19	127	14.53	2454
VAMP1	1.06	3.59E-07	0.13	135	10.76	2818	0.04	117	2.57	6778	0.15	135	13.09	2854
FAM70A	1.05	0.014754343	0.15	123	10.76	2819	0.05	135	3.9	5950	0.02	144	1.65	7650
SNRPD2	1.6	1.68E-24	0.36	80	10.75	2820	0.34	120	23.95	114	0.3	80	8.96	4239
CNST	-1.02	0.416901219	0.13	131	10.75	2821	0.05	135	4.37	5678	0.09	129	7.22	4935
SLC2A4	1.17	0.000812797	0.09	171	10.75	2822	0.08	124	5.63	4987	0.05	125	4.1	6371
OXGR1_t1	1.01	0.497916238	0.13	131	10.74	2823	0.04	126	3.19	6391	NA	NA	NA	NA
ZNF285	1.23	8.16E-07	0.16	117	10.74	2824	0.16	117	10.65	2617	0.12	123	8.7	4344
PIEZO2	1.14	0.010038834	0.13	133	10.73	2825	-0.03	130	-2.17	9939	0.17	120	12	3198
S100A10	1.81	3.46E-11	0.17	112	10.73	2826	0.19	106	10.4	2718	0.22	128	17.17	1794
Sep-11	1.07	0.157746379	0.12	138	10.73	2827	0.11	126	8.16	3663	0.12	132	9.85	3937
FTSJ2	1.24	5.65E-12	0.11	144	10.73	2828	0.11	128	8.5	3498	0.15	99	7.37	4885
ARHGAP19	1.09	3.94E-05	0.13	131	10.73	2829	-0.01	128	-0.56	8857	0.13	137	10.9	3549
MUC13	1.8	3.30E-09	0.14	127	10.72	2830	0.04	125	2.86	6580	0.19	128	14.74	2395
ARL15	1.08	7.83E-07	0.14	127	10.72	2831	0.11	131	9.2	3188	0.31	137	26.87	428
ZNF192	1.05	9.14E-07	0.11	151	10.71	2832	0.08	137	7.27	4108	0.17	135	14.27	2531
CAPG	1.32	0.000214078	0.18	109	10.71	2833	0.2	113	12.37	2018	0	107	0.05	8582
SEC22A	1.28	8.54E-12	0.12	136	10.71	2834	0.09	127	6.97	4258	0.22	129	17.11	1807
POLR1D_t1	1.19	0.00011207	0.2	103	10.7	2835	0.11	113	6.92	4286	0.09	116	6	5451
SMAD5	1.07	0.107694842	0.1	157	10.7	2836	0.1	135	8.23	3631	0.15	160	16.44	1973
ATL3	1.22	1.44E-05	0.19	107	10.7	2837	0.04	138	3.83	5992	0.21	120	14.47	2469
AGTR2	1.01	0.329739233	0.14	127	10.7	2838	0.08	138	6.82	4340	0.12	133	9.84	3939
FOXF2	1.19	3.86E-06	0.16	115	10.69	2839	0.15	123	10.84	2540	0.08	142	7.42	4861
IPO13	1.26	5.97E-14	0.17	114	10.69	2840	0.14	92	5.96	4811	0.24	109	14.36	2501
AAGAB	1.27	1.56E-09	0.13	133	10.69	2841	0.19	123	14.21	1474	0.25	138	22.13	947
EHMT2_t1	1.39	1.57E-18	0.24	94	10.69	2842	0.24	143	22.32	193	0.3	95	13.4	2760
SH3PXD2A	1.11	8.79E-08	0.12	142	10.69	2843	0	155	-0.46	8792	0.18	150	18.47	1558
SEC61G	1.62	2.91E-17	0.31	84	10.68	2844	0.33	93	14.39	1427	0.36	90	14.28	2527
ZNF184	1.12	0.000829043	0.17	113	10.68	2845	0.05	113	3.45	6219	0.29	138	25.17	602
SNTB1	1.34	6.31E-05	0.13	131	10.68	2846	0.09	132	7	4245	0.29	155	30.01	239
KIAA0907	1.47	2.79E-13	0.14	124	10.68	2847	0.23	112	14.2	1483	0.25	129	19.36	1372
RAB35_t1	-1	0.948702243	0.15	120	10.67	2848	NA	NA	NA	NA	-0.07	124	-5.46	11224
KRT10	1.31	1.95E-06	0.17	112	10.66	2849	0.11	126	8.61	3450	0.24	116	15.87	2107
TULP4	1.37	1.06E-11	0.14	126	10.66	2850	0.25	156	26.78	42	0.21	133	17.71	1697
HOXA13	2.75	3.78E-16	0.12	137	10.66	2851	0.08	117	5.29	5175	0.16	125	12.36	3085
GON4L_t2	1.29	4.00E-14	0.19	105	10.66	2852	0.23	106	12.9	1867	0.24	107	13.44	2753
AAED1	1.07	0.050326451	0.16	117	10.65	2853	0.17	120	11.96	2136	0.09	128	7.02	5027
ZBED5	1.27	7.31E-05	0.15	123	10.65	2854	0.2	103	10.7	2596	0.18	104	9.73	3972
ZRANB3	1.12	2.79E-11	0.15	120	10.65	2855	0.23	105	12.84	1881	0.27	116	17.77	1688
TMEM132A	1.41	2.69E-05	0.16	116	10.65	2856	0.15	100	7.27	4109	0.12	104	6.3	5322
GOLGA3	1.21	3.25E-06	0.11	147	10.65	2857	0.24	153	24.87	84	0.05	119	3.65	6590
RAB5C	1.12	0.000567288	0.16	116	10.65	2858	-0.06	105	-3.46	10768	0.08	126	6.05	5427
ZNF443	1.05	0.040381793	0.12	138	10.64	2859	0.03	129	2.62	6740	0.1	120	6.72	5144

AKR1B10	4.03	4.20E-10	0.17	112	10.64	2860	0.17	107	9.49	3064	0.12	108	7.04	5014
ZNF480	1.26	1.11E-05	0.14	128	10.64	2861	0.2	136	17.35	767	0.18	127	13.58	2712
ZNF627	1.15	2.42E-06	0.14	126	10.64	2862	0.03	124	2.06	7107	0.25	113	15.44	2205
PDIA6	1.17	0.000453946	0.11	144	10.63	2863	0.01	139	0.49	8143	0.16	111	9.65	4002
ABHD16A	1.13	1.30E-05	0.11	145	10.63	2864	-0.01	106	-0.71	8945	0.06	131	4.99	5933
PIP4K2C	1.21	1.47E-18	0.14	124	10.63	2865	0.02	135	1.58	7441	0.16	127	12.56	3021
RPAP1	1.36	6.26E-18	0.15	121	10.63	2866	0.18	100	8.84	3359	0.25	108	14.57	2443
PTPN12	1.07	0.279907586	0.12	138	10.63	2867	0.27	114	17	839	0.23	135	19.2	1408
PCNP	1.04	0.451561496	0.12	137	10.63	2868	0.16	125	11.95	2140	0.07	138	6.24	5342
MPZ	1.06	4.90E-07	0.18	108	10.62	2869	-0.03	139	-2.49	10148	0.14	120	9.6	4026
APH1A_t1	1.18	0.003331654	0.13	129	10.62	2870	0.05	131	3.99	5910	0.1	134	8.18	4544
TCOF1	1.13	9.01E-09	0.15	120	10.62	2871	0.13	128	9.95	2879	0.2	140	17.73	1693
TUSC2	1.29	8.58E-11	0.15	120	10.61	2872	0.12	115	7.77	3847	0.25	129	19.68	1323
PDPK1_t1	1.17	8.28E-06	0.11	144	10.61	2873	NA	NA	NA	NA	NA	NA	NA	NA
KCNS1	1.03	0.005084251	0.14	127	10.61	2874	-0.01	142	-1.16	9264	0.01	145	0.9	8081
ZFP14	1.28	6.35E-13	0.1	153	10.61	2875	0.19	136	16.6	920	0.24	168	28.5	333
WDR85	1.3	1.45E-12	0.14	125	10.61	2876	0.27	108	15.4	1164	0.12	120	8.57	4398
APOBEC2	1.05	0.000163418	0.15	123	10.6	2877	0.14	143	13.45	1697	0.08	109	4.5	6184
BAIAP2L1	1.23	1.35E-05	0.15	120	10.6	2878	0.04	142	4.05	5879	0.25	120	17.27	1774
FASTK	1.29	1.21E-10	0.23	97	10.6	2879	0.11	98	5.23	5203	0.24	143	22.23	932
ZNF8	1.13	1.28E-10	0.2	102	10.6	2880	0.15	147	14.92	1304	0.22	104	12.01	3193
CCP110	1.03	0.028729594	0.12	140	10.6	2881	0.14	135	11.81	2188	0.14	155	14.56	2445
ANKRD30A	1.04	0.179449049	0.13	134	10.6	2882	0.06	102	3	6494	0.04	121	2.58	7140
PVRL1_t1	1.09	2.57E-06	0.17	113	10.6	2883	0.13	101	6.86	4321	0.09	95	4.22	6333
CYB5R4	1.11	0.009435843	0.13	131	10.58	2884	0.13	104	7.25	4118	0.09	159	10.02	3867
CLEC2D_t1	1.24	8.85E-05	0.13	132	10.57	2885	0.27	133	22.62	176	NA	NA	NA	NA
LIMK1	1.18	1.20E-05	0.19	105	10.56	2886	0.19	147	18.9	533	0.1	127	8.05	4598
CELSR3	1.72	2.89E-17	0.14	126	10.54	2887	0.23	131	18.49	586	0.23	129	18.01	1646
ADPRHL2	1.04	0.12180803	0.12	139	10.54	2888	0.01	150	1	7808	-0.03	107	-1.69	9543
DPF2	1.11	0.000229749	0.2	104	10.54	2889	0.03	123	1.92	7203	0.1	142	9.14	4172
EGLN3	1.22	0.017980353	0.12	136	10.54	2890	0.08	142	7.53	3975	0.06	144	5.56	5652
EAF1_t1	1.13	2.32E-08	0.11	144	10.54	2891	0.14	124	10.14	2811	NA	NA	NA	NA
DYNLRB1	1.44	2.45E-28	0.26	90	10.54	2892	0.21	112	13.05	1816	0.26	102	13.27	2808
OR2C3	1.09	6.24E-13	0.13	134	10.54	2893	0.12	124	8.85	3354	0.25	126	19.26	1395
HNRNPAB	1.08	0.018845161	0.14	125	10.54	2894	0.02	125	1.71	7339	0.14	114	8.81	4299
TRMT1L_t1	1.16	0.001718771	0.14	125	10.54	2895	NA	NA	NA	NA	0.21	134	18.01	1645
CNTNAP3	1.12	0.005619513	0.1	158	10.53	2896	-0.02	114	-1.38	9420	0.03	152	2.97	6939
PPHLN1_t2	1.14	0.001405447	0.15	118	10.53	2897	0.24	120	16.91	858	0.19	118	12.96	2899
OSBP2_t1	1.1	7.87E-08	0.16	114	10.52	2898	0.1	146	9.92	2890	NA	NA	NA	NA
WDR20	1.12	4.70E-05	0.16	114	10.52	2899	0.08	123	5.55	5040	0.17	144	15.98	2074
UBE2J1	1	0.951419422	0.09	166	10.52	2900	0.15	128	11.82	2183	-0.07	144	-6.33	11588
SUV39H1	1.23	2.96E-11	0.16	114	10.52	2901	0.07	122	5.34	5150	0.26	150	26.09	492
RGAG1	1.16	3.66E-08	0.15	120	10.52	2902	-0.02	130	-1.22	9308	0.14	127	11.11	3467
POP7	1.23	2.65E-08	0.14	123	10.52	2903	0.19	140	16.89	863	0.24	142	22.12	949
NRL	1.1	0.004534563	0.13	131	10.52	2904	-0.05	118	-3.71	10939	0.08	114	5.01	5918
DUSP23	1.59	8.18E-13	0.21	99	10.52	2905	0.08	122	5.96	4810	0.37	124	27.51	396
ATG14	1.1	8.10E-05	0.13	128	10.52	2906	0.07	136	6.38	4574	0.12	160	12.67	2987
HIST2H2AB_t1	1.76	9.72E-16	0.19	104	10.51	2907	NA	NA	NA	NA	0.17	89	6.48	5248
ZNF253_t1	1.23	1.72E-06	0.18	108	10.51	2908	0.26	123	19.09	503	NA	NA	NA	NA
SLC44A3	1.19	0.010913153	0.14	126	10.51	2909	0.07	109	4.19	5783	0.09	114	5.66	5596
PGK1	1.31	3.30E-08	0.14	123	10.5	2910	0.12	125	9.34	3128	0.2	145	19.37	1368
HCCS_t1	1.03	0.015012646	0.14	125	10.5	2911	NA	NA	NA	NA	NA	NA	NA	NA
BMP8B_t1	1.1	7.40E-08	0.12	140	10.5	2912	NA	NA	NA	NA	NA	NA	NA	NA
DHX35	1.21	1.79E-17	0.14	124	10.5	2913	0.11	112	7.09	4201	0.21	139	18.84	1494
FBXW2	1.06	0.003785542	0.11	147	10.5	2914	0.02	135	1.7	7357	0.22	151	21.9	980
POLA2	1.31	6.28E-16	0.18	108	10.49	2915	0.14	114	8.78	3384	0.25	96	11.42	3375
MYO9B	1.13	2.45E-05	0.14	123	10.49	2916	0.24	126	18.38	604	0.02	123	1.69	7632
PNMA1	1.28	0.000803109	0.15	119	10.48	2917	0.22	130	17.78	703	0.07	127	5.1	5881
ZBTB24	-1.01	0.677576876	0.09	162	10.48	2918	0.09	134	7.63	3921	-0.02	150	-2.43	9910
ZNF382	1.12	9.16E-05	0.14	126	10.48	2919	0.29	113	18.29	615	0.11	119	7.86	4683
STK24	1.16	0.00049563	0.12	135	10.47	2920	0.2	146	18.76	551	0	134	-0.11	8679
TCP11L2	-1.01	0.522218162	0.13	128	10.46	2921	0.04	118	2.83	6598	0.08	114	5.1	5878
DTD1	1.33	5.90E-05	0.15	119	10.46	2922	0.21	122	15.32	1187	0.15	108	8.67	4357
ATP2B4_t2	1.05	0.001545056	0.14	123	10.46	2923	0.16	152	16.21	998	0.06	134	4.86	5995
ENY2	1.3	8.76E-07	0.14	125	10.46	2924	0.17	125	12.85	1879	0.26	127	19.69	1320
IFT80	1.23	2.09E-08	0.14	126	10.46	2925	0.23	119	15.76	1100	0.17	138	14.57	2441
AQR	1.09	0.010985991	0.17	113	10.46	2926	0.16	117	10.95	2493	0.17	117	11.39	3387
KRIT1_t1	1.08	1.10E-07	0.11	142	10.46	2927	0.17	118	11.43	2325	0.23	135	19.4	1364
TAGLN3	1.09	6.42E-11	0.16	114	10.46	2928	0.05	120	3.48	6195	0.29	107	16.34	1998
CCDC86	1.39	9.03E-15	0.23	95	10.46	2929	0.22	126	16.82	873	0.21	115	13.51	2734
CUX1_t1	1.11	0.001128356	0.11	141	10.46	2930	0.15	155	15.81	1089	0.09	136	7.38	4877
PKN2	-1.02	0.544831181	0.12	134	10.45	2931	0.07	144	6.42	4558	0.14	148	14.11	2568
FBXO4_t1	1.15	5.65E-08	0.17	112	10.45	2932	0.06	107	3.43	6229	NA	NA	NA	NA
FAF2_t1	1.06	0.008688384	0.12	138	10.45	2933	NA	NA	NA	NA	0.13	130	10.45	3706
GTF3C3	1.18	1.11E-06	0.15	118	10.45	2934	0.18	148	17.22	796	0.26	119	18.15	1615
LUC7L2	1.03	0.439303604	0.12	134	10.44	2935	0.01	126	0.89	7877	0.17	130	13.53	2730
CTNNA2_t1	1.37	8.27E-08	0.14	125	10.43	2936	NA	NA	NA	NA	0.21	127	16.12	2046
SNX13_t1	1.16	0.004677443	0.12	139	10.42	2937	NA	NA	NA	NA	NA	NA	NA	NA
L1CAM_t1	1.04	0.000595799	0.16	117	10.42	2938	0.03	124	1.95	7177	0.1	127	7.73	4740
PLCB3	1.03	0.100848108	0.16	115	10.41	2939	0.15	153	15.21	1221	0.07	113	4.15	6351
MDM2	1.06	0.000102104	0.12	134	10.41	2940	0.05	120	3.5	6180	0.16	138	13.85	2629
GCN1L1	1.3	2.06E-11	0.12	140	10.41	2941	0.2	117	13.34	1736	0.09	110	5.68	5588
TRAF3IP1	1.11	5.22E-08	0.14	125	10.4	2942	0.18	120	12.32	2030	0.25	127	19.33	1379
MED28	1.01	0.578256837	0.13	128	10.4	2943	0.06	129	4.35	5688	0.06	130	4.66	6096
VPS29	1.23	5.33E-06	0.15	118	10.39	2944	0.21	111	12.96	1846	0.2	130	15.94	2087
CRMP1	1.07	1.27E-09	0.13	130	10.39	2945	0.08	137	6.55	4481	0.09	148	9.18	4164
PSMD2	1.27	8.23E-10	0.2	103	10.38	2946	0.1	114	6.37	4578	0.16	89	6.43	5263
TRIM39	1.16	9.83E-07	0.16	116	10.38	2947	0.06	122	4.6	5555	0.16	131	13.09	2856
KIAA1804_t1	1.03	0.26444016	0.12	134	10.38	2948	NA	NA	NA	NA	0.06	151	6.46	5256
BTN2A2_t1	1.32	1.76E-09	0.15	119	10.37	2949	0.15	130	11.81	2187	NA	NA	NA	NA
FMN2_t1	1.08	0.011910473	0.15	121	10.37	2950	NA	NA	NA	NA	NA	NA	NA	NA
POC5	1.29	3.35E-12	0.19	105	10.37	2951	0.18	96	8.36	3562	0.3	99	14.72	2404
HUS1B	1.06	0.000420232	0.15	117	10.37	2952	0.2	88	7.55	3961	0.1	120	7.25	4930
RPL38	1.59	1.79E-17	0.23	95	10.37	2953	0.29	114	18.42	596	0.31	90	12.55	3022
SBNO1	1.05	0.361601486	0.09	160	10.36	2954	0.19	132	15.27	1207	0.11	148	11.09	3479
MRTO4	1.22	2.08E-07	0.15	120	10.36	2955	0.14	133	11.51	2304	0.1	122	7.38	4881
GALNTL5	-1	0.689269451	0.12	136	10.36	2956	0.02	94	1.02	7796	0.11	95	5.11	5871
SLC4A7	1.02	0.45810567	0.1	158	10.36	2957	0.24	125	17.74	707	-0.01	147	-0.96	9177
TFAP2A	1.04	0.016840205	0.13	129	10.35	2958	-0.02	128	-1.37	9411	0.13	135	11.1	3476
PSMC4	1.36	1.32E-14	0.17	111	10.35	2959	0.07	105	3.85	5975	0.16	105	8.7	4339
TRMT112	1.41	1.32E-17	0.21	100	10.35	2960	0.28	91	11.67	2246	0.28	96	12.68	2986
TRPM4	1.3	1.70E-07	0.16	115	10.35	2961	0.04	118	2.73	6657	0.27	111	16.25	2019
CDS1	1.17	0.000462268	0.11	145	10.35	2962	0	134	0.41	8198	0.1	170	12.42	3060
CCDC167	1.38	8.15E-11	0.19	104	10.35	2963	0.15	112	9.25	3162	0.3	111	18.05	1635
ZNF646	1.28	4.83E-12	0.17	111	10.34	2964	0.15	100	7.58	3946	0.2	108	11.59	3324
MBD1_t1	1.04	0.000411891	0.16	115	10.34	2965	0.11	113	7.16	4162	0.09	111	5.66	5599

UFM1	1.02	0.555855868	0.11	142	10.34	2966	0.11	128	8.39	3546	0.02	133	1.59	7690
B4GALT7	1.19	5.96E-10	0.17	110	10.33	2967	0.09	97	4.23	5758	0.17	111	10.57	3665
MYBPC1	1.19	7.92E-06	0.17	111	10.33	2968	0.14	126	10.55	2654	0.2	120	14.33	2516
TRDN	1.1	5.01E-14	0.13	129	10.33	2969	0.11	139	9.48	3070	0.18	136	15.05	2320
ADAMTS14	1.17	2.35E-08	0.15	121	10.33	2970	0.16	132	12.8	1898	0.08	139	7.26	4922
TMEM216	1.08	0.015775165	0.15	121	10.33	2971	0.11	154	11.6	2273	-0.04	106	-2.03	9721
NUDT1	1.75	4.13E-23	0.22	97	10.33	2972	0.19	80	5.73	4932	0.29	84	9.7	3987
FASTKD2	1.06	0.070878956	0.11	146	10.32	2973	0.05	141	4.77	5464	0.13	140	11.9	3234
RHOU	1.03	0.692230043	0.11	142	10.32	2974	-0.05	145	-4.66	11469	0.16	136	13.33	2785
EFNA3	1.04	0.002436239	0.15	118	10.31	2975	0.02	122	1.73	7324	0.09	118	6	5448
PCDHB6	1.04	0.016601182	0.1	155	10.31	2976	0.05	117	3.61	6106	0.1	130	7.85	4686
CBX8	1.38	2.62E-21	0.21	98	10.31	2977	0.22	102	11.67	2245	0.31	87	11.48	3358
LAMA3_t2	1.17	1.35E-09	0.14	122	10.31	2978	0.09	115	6.04	4759	0.31	122	22.14	944
ANAPC11	1.53	1.28E-14	0.19	104	10.31	2979	0.28	132	22.72	171	0.3	116	19.76	1311
FGF14	1.2	8.83E-06	0.09	160	10.31	2980	0.09	139	7.97	3762	0.11	145	10.18	3801
HIST3H3	1.55	1.16E-17	0.27	88	10.31	2981	0.35	88	13.34	1733	0.3	115	19.56	1338
EPPK1_t1	1.36	4.15E-07	0.14	124	10.3	2982	NA	NA	NA	NA	0.07	101	3.34	6749
SLC25A30	1.06	0.018799002	0.11	142	10.3	2983	-0.03	124	-2.3	10022	0.04	125	2.91	6986
RPA2	1.09	0.009367737	0.12	136	10.3	2984	0.14	122	9.82	2925	0.07	165	7.53	4821
PSMC2	1.21	0.000195686	0.12	137	10.3	2985	0.13	127	9.91	2892	0.2	143	18.98	1459
CETN3	1.16	2.29E-05	0.18	106	10.29	2986	0.09	112	5.28	5177	0.23	131	18.7	1523
INIP	1.08	0.01501187	0.14	123	10.29	2987	0.14	132	11.25	2383	0.14	127	11.12	3465
USP21	1.22	3.66E-13	0.21	98	10.29	2988	0.11	110	6.49	4521	0.18	101	8.99	4229
ATXN3	1.07	0.124168637	0.11	146	10.29	2989	0.05	127	3.93	5937	0.17	153	17.25	1777
ZNF57	1.36	1.53E-08	0.21	99	10.29	2990	0.12	121	8.84	3362	0.22	95	9.93	3900
ZNF91	1.09	0.160680891	0.13	131	10.29	2991	0.12	139	10.5	2679	0.15	122	10.62	3645
TRIM65_t1	1.04	0.00034588	0.1	150	10.29	2992	NA	NA	NA	NA	NA	NA	NA	NA
IMMP2L	1.25	1.89E-06	0.14	125	10.29	2993	-0.02	105	-0.98	9130	0.23	136	20.16	1256
MFAP3	1.12	8.38E-12	0.11	140	10.28	2994	0.16	135	13.43	1703	0.27	139	23.79	752
STAT5B	1.1	2.40E-06	0.15	120	10.28	2995	0.08	122	5.71	4946	0.1	123	7.2	4949
SPOCK1	1.72	1.30E-08	0.1	149	10.27	2996	0.14	142	13.22	1767	0.24	155	25.23	592
GART_t1	1.1	0.000449192	0.13	129	10.27	2997	0.09	115	5.55	5035	0.13	124	9.74	3969
SLC25A5	1.2	0.000226711	0.13	131	10.27	2998	0.07	140	6.23	4646	0.22	118	14.7	2407
VPS28	1.31	6.08E-09	0.17	111	10.26	2999	0.12	107	7	4248	0.21	124	15.67	2163
COPS3	1.06	0.143304448	0.11	144	10.26	3000	0.08	110	5.06	5303	0.12	109	7.15	4972
LOH12CR1	1.06	0.317294098	0.13	131	10.26	3001	0.04	115	2.6	6761	0.04	110	2.28	7304
SRRD_t1	1.27	3.51E-10	0.18	108	10.25	3002	0.14	100	6.93	4285	NA	NA	NA	NA
GPX7_t1	1.19	0.001281131	0.1	152	10.25	3003	NA	NA	NA	NA	0.01	110	0.63	8237
HTN3	1.04	0.004878775	0.13	129	10.24	3004	0.1	125	7.84	3812	-0.03	118	-1.88	9657
RFK	1.22	6.55E-06	0.11	144	10.24	3005	0.1	128	7.62	3925	0.18	135	14.96	2346
DNTTIP1	1.16	1.21E-09	0.17	109	10.24	3006	0.1	120	7.03	4231	0.16	106	8.73	4330
SPSB2	1.58	3.36E-21	0.19	104	10.24	3007	0.25	99	12.18	2073	0.26	100	12.9	2923
ANKRD29	1.67	1.24E-10	0.12	132	10.23	3008	0.13	151	13.61	1650	0.26	132	21.53	1043
ZNF543	1.16	5.24E-14	0.14	123	10.23	3009	0.21	120	14.75	1341	0.12	124	9.04	4213
FBL	1.54	4.97E-18	0.26	89	10.23	3010	0.21	100	10.42	2710	0.24	132	19.61	1329
MRPL22	1.17	4.42E-07	0.12	135	10.23	3011	0.11	127	8.35	3571	0.1	144	9.86	3930
ZNF74	1.28	7.59E-14	0.13	128	10.23	3012	0.21	150	21.04	290	0.16	140	14.53	2450
ABCB8	1.07	0.001328199	0.12	136	10.23	3013	0.04	125	2.9	6554	0.1	124	7.2	4944
YARS2	1.15	1.90E-07	0.14	121	10.22	3014	0.2	135	17.14	812	0.15	131	11.96	3213
WNK4_t1	1.31	6.44E-09	0.16	114	10.22	3015	0.09	120	6.29	4625	NA	NA	NA	NA
ZNF765	1.19	0.000156425	0.13	131	10.21	3016	0.24	136	20.51	337	0.2	132	16.22	2027
GMPPA	1.23	1.93E-11	0.17	110	10.21	3017	0.13	120	9.29	3152	0.14	91	5.91	5491
GABARAPL2	1.02	0.540824877	0.1	155	10.21	3018	0.05	107	2.95	6519	0.02	122	1.54	7724
SLC12A9_t1	1.24	1.11E-07	0.14	124	10.2	3019	0.19	112	11.76	2207	NA	NA	NA	NA
PCDH19	-1.02	0.136635553	0.12	138	10.2	3020	-0.09	140	-8.46	13307	-0.03	143	-2.78	10080
TIMM10	1.29	8.83E-12	0.15	119	10.2	3021	0.08	127	6.16	4697	0.25	93	10.77	3582
CXCL5	1.15	0.001794527	0.15	120	10.2	3022	0.07	120	4.89	5394	0.13	151	12.86	2936
MANBAL	1.24	6.88E-15	0.14	125	10.2	3023	0.13	127	10.38	2729	0.14	127	11.14	3457
SMARCC2	1.16	1.70E-05	0.14	124	10.19	3024	0.14	150	13.7	1625	0.11	120	7.97	4632
LECT1	1.1	1.14E-09	0.14	123	10.19	3025	0.02	143	1.99	7151	0.11	139	10.13	3827
ADAMDEC1	1.33	1.31E-05	0.15	120	10.19	3026	0.19	149	18.7	558	0.08	124	6.12	5389
PCDHB2_t1	1.38	2.42E-07	0.18	106	10.19	3027	NA	NA	NA	NA	0.15	117	10.38	3729
LRP11	1.25	9.16E-12	0.11	142	10.19	3028	0.11	143	10.68	2604	0.22	134	18.21	1608
CCDC138	1.2	6.02E-10	0.22	97	10.18	3029	0.18	103	9.52	3049	0.29	108	16.73	1892
GRIA2_t1	1.06	8.68E-11	0.11	140	10.18	3030.5	NA	NA	NA	NA	NA	NA	NA	NA
FOXD4_t1	1.23	2.14E-11	0.11	140	10.18	3030.5	0.14	125	10.15	2806	NA	NA	NA	NA
ZKSCAN4	1.16	1.33E-15	0.17	109	10.18	3032	0.11	116	7.39	4041	0.17	125	12.79	2954
METTL2B	1.44	4.22E-15	0.15	120	10.18	3033	0.19	115	12.42	2001	0.29	121	20.26	1239
ACTR2	1.04	0.475952633	0.13	127	10.18	3034	0.24	123	17.18	804	0.11	140	9.58	4029
RPUSD1	1.25	1.56E-16	0.22	97	10.17	3035	0.12	113	7.71	3878	0.2	115	13.22	2822
SPIN1	1.05	0.001353572	0.1	150	10.17	3036	0.11	126	8.3	3595	0.16	136	13.78	2656
ZNF223	1.07	0.005479522	0.11	144	10.17	3037	0.13	128	10.42	2709	0.04	123	2.75	7050
EEF1D	1.57	1.62E-13	0.24	93	10.17	3038	0.24	88	9.26	3158	0.28	84	9.4	4085
LYG2	1.18	1.98E-11	0.16	113	10.17	3039	0.12	104	6.62	4438	0.3	98	14.25	2536
MAGT1	1.1	0.019080105	0.1	149	10.17	3040	-0.01	139	-0.84	9040	0.15	138	13.57	2716
ZNF746	1.21	7.53E-08	0.13	127	10.16	3041	0.12	137	10.68	2601	0.05	136	4.43	6230
HSP90AA1_t1	1.23	0.005839517	0.15	120	10.16	3042	NA	NA	NA	NA	0.22	123	16.41	1980
KCNA7	1.04	3.46E-05	0.13	129	10.16	3043	0	136	-0.33	8700	0.22	126	17.05	1816
STC1	1.08	1.01E-08	0.12	136	10.15	3044	0.1	136	8.22	3635	0.16	154	16.87	1859
MEA1	1.53	3.58E-26	0.27	88	10.15	3045	0.17	135	14.19	1486	0.26	103	13.71	2672
TNFSF9	1.06	1.37E-06	0.11	139	10.15	3046	0.02	121	1.34	7580	0.03	150	3.27	6796
SART1	1.14	1.26E-06	0.13	128	10.14	3047	0.06	115	3.75	6034	0.08	104	4.42	6238
AKAP4	1.02	0.036254311	0.14	120	10.14	3048	0.04	118	2.58	6770	0.14	100	6.93	5058
ASB1	1.08	0.083978631	0.13	126	10.14	3049	0.06	135	5.18	5226	0.03	125	1.92	7500
MEP1A_t1	1.49	1.60E-07	0.15	118	10.14	3050	NA	NA	NA	NA	0.1	125	7.14	4975
TRMT10C	1.15	0.006713386	0.17	111	10.13	3051	0.18	111	11.18	2406	0.13	122	9.34	4107
FOXRED2	1.28	1.57E-09	0.12	136	10.13	3052	0.06	151	6.07	4740	0.18	152	18.24	1604
LANCL3	1.01	0.108654456	0.12	132	10.12	3053	0.07	121	4.86	5415	0.08	144	7.68	4760
ZNF317	1.14	1.38E-07	0.13	129	10.12	3054	0.08	136	7.28	4104	0.05	145	4.99	5926
TM2D1	1.1	0.016163981	0.15	119	10.12	3055	0.08	122	5.92	4827	0.18	108	10.28	3771
TRRAP	1.23	6.30E-10	0.16	113	10.11	3056	0.24	130	19.23	484	0.15	123	11.11	3474
ABHD10_t1	1.06	0.045498898	0.13	125	10.11	3057	0.11	111	6.75	4381	NA	NA	NA	NA
HNRNPC	1.08	0.036643343	0.12	132	10.11	3058	0.12	137	10.39	2720	0.13	132	10.28	3772
SGSM1	1.04	0.000109713	0.12	131	10.11	3059	0.11	135	9.66	2998	0.13	120	8.8	4305
FGF13	1.69	5.95E-05	0.13	125	10.1	3060	0.18	119	12.4	2010	0.1	132	8.51	4423
WIPI2	1.21	5.23E-09	0.13	127	10.1	3061	0.13	148	13.12	1794	0.18	137	15.9	2100
MED23_t1	1.14	2.36E-06	0.14	124	10.1	3062	0.09	140	8.48	3513	0.17	119	11.73	3289
NUP153	1.11	0.010708882	0.14	124	10.1	3063	0.11	122	8.1	3696	0.21	140	18.88	1482
EFCAB13	1	0.809810027	0.11	145	10.1	3064	0.01	135	0.63	8045	0.04	116	2.96	6954
TMEM203	1.19	6.41E-10	0.12	134	10.09	3065	0.08	123	5.92	4831	0.17	129	13.39	2764
AAR2	1.15	3.72E-13	0.14	120	10.09	3066	0.02	135	1.47	7514	0.09	132	7.14	4979
KIAA1704	1.12	0.000404561	0.15	117	10.09	3067	0.22	116	14.45	1413	0.09	130	7.22	4938
CD99L2_t1	1.28	2.96E-05	0.14	123	10.09	3068	NA	NA	NA	NA	0.23	155	24.62	659
CCKBR	1.03	0.00365757	0.15	117	10.09	3069	0.02	114	1.3	7605	0.12	120	8.34	4483
KDELC1	1.41	1.17E-10	0.15	118	10.08	3070	0.12	118	8.28	3608	0.21	110	12.89	2929
MNAT1	1.15	5.71E-05	0.15	118	10.08	3071	0.07	113	4.41	5655	0.13	110	7.62	4787

POLG2	1.48	1.77E-17	0.18	105	10.08	3072	0.25	92	10.29	2761	0.35	107	19.93	1284
IPO8	1.06	3.91E-05	0.13	129	10.08	3073	0.12	129	9.25	3164	0.2	128	15.61	2178
LRIF1	1.28	3.78E-08	0.14	123	10.07	3074	0.12	132	9.51	3057	0.31	115	19.88	1287
HIST1H4I	1.69	5.62E-20	0.3	84	10.07	3075	0.16	97	7.29	4099	0.28	97	13.34	2783
GRAMD2	1.03	0.000771192	0.14	124	10.06	3076	0.15	127	11.32	2352	0.09	138	7.5	4830
ARSK	1.13	0.000115598	0.12	136	10.06	3077	0.04	126	3.15	6411	0.23	126	17.72	1695
TRAF5	1.25	0.004364745	0.13	125	10.06	3078	0.22	127	17.16	806	0.13	130	10.49	3692
POLK_t1	1.02	0.804002844	0.11	144	10.06	3079	NA	NA	NA	NA	0.21	124	15.73	2146
FAM134A	1.22	4.87E-12	0.13	127	10.06	3080	0.04	146	3.55	6146	0.08	135	7.19	4954
ABCD3	1.13	0.000453328	0.13	125	10.05	3081	0.11	123	8.14	3677	0.18	129	14.33	2515
MCU	1.03	0.232923576	0.15	118	10.05	3082	0.1	143	9.73	2964	0.11	129	9.05	4210
HOOK3_t1	1.1	0.000103153	0.09	161	10.04	3083	NA	NA	NA	NA	0.19	147	18.16	1613
HUS1	1.21	1.56E-07	0.12	136	10.04	3084	0.08	127	5.89	4849	0.23	136	19.52	1345
OSR2_t1	1.81	1.42E-10	0.17	108	10.04	3085	0.16	118	10.85	2533	NA	NA	NA	NA
ATP10A_t1	1	0.912740741	0.14	122	10.03	3086	NA	NA	NA	NA	0.11	134	9.39	4088
METTL15_t1	1.04	0.011253658	0.1	154	10.03	3087	NA	NA	NA	NA	NA	NA	NA	NA
NIPA2	1.2	7.10E-09	0.13	126	10.03	3088	0.15	126	11.24	2389	0.2	139	17.81	1680
TYW3_t1	1.14	0.002802011	0.11	142	10.02	3089	0.08	132	6.96	4271	NA	NA	NA	NA
SND1	1.25	3.83E-13	0.15	118	10.02	3090	0.17	115	11.06	2446	0.14	100	7.03	5022
MTFP1	1.29	2.30E-07	0.13	128	10.02	3091	0.06	118	4.03	5886	0.11	96	5.07	5892
TBCD_t1	1.1	2.90E-06	0.14	120	10.02	3092	NA	NA	NA	NA	NA	NA	NA	NA
PRICKLE4	1.47	8.32E-21	0.2	99	10.02	3093	0.3	122	21.57	240	0.23	103	12.42	3062
RHBDL3	1.3	8.57E-06	0.12	135	10.01	3094	-0.08	144	-7.75	13009	0.14	124	10.02	3871
GRK6	1.17	7.97E-09	0.16	113	10.01	3095	0.14	126	10.58	2634	0.09	139	8.2	4539
EMG1	1.26	3.42E-11	0.17	110	10.01	3096	0.17	103	9.23	3174	0.18	120	12.39	3071
MFSD6	1.11	5.18E-06	0.14	120	10.01	3097	0.19	124	13.88	1570	0.15	143	13.63	2704
VPS45_t1	1.39	1.18E-18	0.18	106	10.01	3098	NA	NA	NA	NA	0.33	122	24.02	720
RPL7	1.42	6.27E-08	0.17	110	10	3099	0.2	86	7.35	4064	0.27	91	10.95	3523
OGT	1.18	0.010127627	0.12	132	10	3100	0.14	118	9.66	3002	-0.02	152	-2.3	9841
RAB11FIP2_t1	1.12	0.002066309	0.11	137	10	3101	NA	NA	NA	NA	0.19	137	16.65	1915
APC2	1.07	3.98E-05	0.12	132	10	3102	0.06	132	4.8	5446	0.15	125	11.14	3456
BRI3BP	1.45	7.49E-15	0.19	103	10	3103	0.08	136	6.7	4404	0.25	117	16.73	1894
PDGFA	1.43	7.81E-05	0.12	136	9.99	3104	0.11	142	9.8	2935	0.13	120	8.84	4285
AHI1	1.04	0.213206409	0.12	136	9.99	3105	0.11	143	10.22	2785	0.1	150	9.84	3940
SAP18_t1	1.07	0.038172759	0.15	116	9.99	3106	0.06	120	4.1	5842	NA	NA	NA	NA
ZNF420	1.07	0.002415942	0.11	144	9.99	3107	0.09	119	6.36	4581	0.12	118	8.36	4479
NPAT	1	0.933392726	0.12	132	9.99	3108	0.11	124	8.11	3694	0.07	155	7.12	4987
KRTCAP2	1.69	2.82E-31	0.25	90	9.98	3109	0.23	73	5.22	5213	0.29	89	11.49	3354
SYCP3	1.05	7.90E-07	0.14	120	9.98	3110	0.03	104	1.81	7267	0.11	104	5.75	5561
FN3KRP	1.34	1.24E-15	0.16	111	9.98	3111	0.09	116	6.11	4721	0.09	124	6.41	5272
GSK3A	1.36	1.56E-16	0.26	88	9.98	3112	0.28	108	16.02	1040	0.23	112	14.48	2464
TUBB3	1.26	3.34E-07	0.13	126	9.98	3113	0.1	120	6.93	4280	0.12	120	8.11	4580
NKAP	1.33	1.12E-11	0.21	98	9.98	3114	0.04	100	2.25	6987	0.38	94	16.77	1883
HAUS6_t1	1.12	0.003619014	0.12	135	9.97	3115	NA	NA	NA	NA	NA	NA	NA	NA
TWSG1_t1	1.1	6.67E-05	0.12	133	9.97	3116	0.12	130	9.47	3082	NA	NA	NA	NA
PARP4	1.02	0.637031287	0.14	120	9.97	3117	-0.04	111	-2.65	10248	0.06	116	4.2	6339
SPATA5	1.13	1.91E-09	0.12	130	9.96	3118	0.09	131	7.41	4032	0.14	137	11.97	3208
RALGPS1_t1	1.24	5.77E-06	0.1	147	9.95	3119	NA	NA	NA	NA	0.06	158	6.54	5216
ZNF792	1.18	3.23E-13	0.16	114	9.95	3120	0.19	137	16.17	1006	0.17	129	13.14	2847
KIAA1467	1.01	0.517136653	0.1	152	9.95	3121	-0.04	124	-2.81	10347	0.07	144	6.54	5217
VRK2	1.11	0.019598327	0.17	108	9.94	3122	0.19	138	16.98	850	0.14	129	11.28	3419
PRSS12	1.04	0.019544528	0.14	121	9.94	3123	0.03	137	2.75	6645	0.08	134	6.5	5235
ALG3	1.33	1.56E-12	0.21	98	9.94	3124	0.11	103	6.02	4774	0.23	109	13.62	2708
ACAP2_t1	1	0.967491003	0.1	153	9.93	3125	NA	NA	NA	NA	0.11	136	9.11	4182
MKX_t1	1.08	4.10E-05	0.11	142	9.93	3126	NA	NA	NA	NA	NA	NA	NA	NA
DRAM1	1.06	0.034172488	0.11	144	9.93	3127	0.14	131	11	2477	0.02	121	1.76	7590
CASP8AP2	1.15	0.000728797	0.15	116	9.93	3128	0.2	113	12.78	1905	0.17	126	13.09	2853
PLCE1	1.47	6.72E-08	0.17	108	9.93	3129	0.21	111	12.78	1906	0.28	114	18.17	1611
PPP6R1	1.14	5.00E-05	0.2	100	9.92	3130	0.19	122	13.77	1609	0.12	141	10.6	3653
TCTE3	1.06	3.97E-09	0.17	109	9.92	3131	0.17	92	6.93	4281	0.15	84	5.18	5837
ZNF442	1.06	0.048956022	0.12	136	9.92	3132	-0.01	110	-0.34	8706	0.14	113	8.92	4255
LY6K_t1	1.27	0.004138916	0.15	116	9.91	3133	-0.06	114	-4.16	11185	NA	NA	NA	NA
TALDO1_t1	1.27	3.31E-08	0.18	104	9.91	3134	NA	NA	NA	NA	0.13	119	8.86	4276
HTR3D	1.01	0.490056419	0.15	118	9.9	3135	-0.02	110	-1.1	9221	0.07	102	3.9	6471
PKN3	1.14	2.37E-08	0.14	120	9.9	3136	0.03	96	1.57	7453	0.05	120	3.24	6810
TRAM1	1.27	0.001395385	0.11	140	9.9	3137	0.16	134	13.22	1768	0.19	130	15.4	2216
CCDC14	1.34	9.31E-07	0.11	141	9.9	3138	0.15	113	9.63	3008	0.18	115	11.76	3280
SYS1	1.19	1.47E-08	0.14	122	9.9	3139	0.1	124	7.1	4197	0.16	129	12.55	3024
REXO2	1.16	0.000992932	0.18	105	9.9	3140	0.22	104	11.81	2189	0.17	124	12.72	2966
DAZ2_t1	1.01	0.130501754	0.13	127	9.9	3141	0.02	133	2.03	7126	0.04	139	3.84	6506
CNNM4	1.15	0.000238973	0.15	115	9.9	3142	0.15	128	11.62	2271	0.12	128	9.4	4087
PRKAB2_t1	1.24	4.69E-05	0.12	136	9.89	3143	NA	NA	NA	NA	0.31	128	24.44	676
SMPD4_t1	1.25	1.67E-13	0.14	119	9.89	3144	NA	NA	NA	NA	0.12	120	8.36	4480
MAPK14_t1	1.11	0.005066362	0.11	142	9.89	3145	0.05	137	4.09	5853	0.15	131	12.43	3056
CELSR2_t1	1.06	1.25E-07	0.13	129	9.89	3146	NA	NA	NA	NA	0.14	138	12.72	2967
ULBP1	1.02	0.003756042	0.15	117	9.89	3147	0.14	124	10.06	2838	0.08	123	5.66	5600
TAL2	1.21	3.16E-15	0.17	108	9.89	3148	0.09	113	5.53	5050	0.25	109	14.95	2350
SLC30A8	1.02	0.02689981	0.11	136	9.88	3149	0.14	128	10.68	2600	0.13	128	10.15	3816
PDHA2	1.05	9.63E-05	0.24	91	9.88	3150	0.08	91	3.36	6277	0.19	92	8.01	4619
PAK1IP1	1.32	6.94E-09	0.16	110	9.88	3151	0.22	88	8.25	3620	0.32	128	25.35	579
SNRNP40_t1	1.05	0.02134086	0.16	110	9.88	3152	0.13	124	9.3	3143	NA	NA	NA	NA
TRMT61B	1.12	6.23E-06	0.11	140	9.88	3153	0.1	135	8.64	3440	0.09	122	6.75	5128
MTERFD1	1.44	7.18E-10	0.23	93	9.87	3154	0.21	86	7.74	3862	0.33	96	15.19	2278
ATP8A2	1.12	1.11E-05	0.12	131	9.87	3155	0.26	132	21.31	257	0.11	127	8.59	4385
SLC4A9	1.08	4.38E-08	0.14	122	9.87	3156	0.01	113	0.57	8092	0.21	131	16.93	1846
CDK5R1	1.03	0.015624244	0.1	147	9.86	3157	0.02	127	1.43	7537	0.07	146	6.28	5323
SBF1	1.13	0.001419779	0.12	135	9.86	3158	0.1	144	9.72	2968	0.07	121	5.14	5856
ZNF606	1.21	1.90E-06	0.11	142	9.86	3159	0.2	119	14	1530	0.17	108	9.89	3917
MSMB	1.19	2.22E-05	0.14	120	9.85	3160	0.09	110	5.14	5257	0.14	88	5.5	5684
CNOT1_t1	1.14	1.21E-10	0.17	109	9.85	3161	0.15	117	10.17	2799	0.28	117	19.02	1449
XIAP	1.13	0.000328021	0.11	136	9.84	3162	0.1	135	8.56	3465	0.23	154	24.38	681
MXD3	1.2	1.78E-12	0.2	100	9.84	3163	0.08	96	3.83	5989	0.16	97	7.72	4748
GTF3C4	1.15	1.33E-05	0.13	128	9.83	3164	0.13	121	8.98	3280	0.05	128	4.11	6368
PRR7_t1	1.22	5.39E-10	0.22	95	9.83	3165	NA	NA	NA	NA	0.25	103	13.28	2801
FTL	1.48	1.07E-06	0.23	92	9.83	3166	0.21	93	8.86	3350	0.18	102	9.2	4159
ZNF354B	-1.01	0.873618595	0.13	124	9.82	3167	0.06	95	2.64	6725	0.14	83	4.61	6121
BAG4	-1.03	0.167326648	0.1	149	9.82	3168	0.03	129	2.71	6671	0.04	132	3.49	6673
HAVCR1	1.15	0.000204098	0.19	102	9.82	3169	0.06	124	4.7	5498	0.15	107	8.29	4498
RAB40B	1.33	6.29E-09	0.13	128	9.81	3170	0.14	114	8.65	3436	0.16	157	17	1824
AMPD3_t1	1.03	0.653818416	0.11	140	9.81	3171	NA	NA	NA	NA	-0.03	149	-2.75	10061
ACSL6	1.64	3.30E-07	0.1	144	9.81	3172	0.03	148	3.13	6421	0.15	144	13.94	2605
ZNF354C	-1	0.660649467	0.15	116	9.8	3173	0	118	-0.27	8651	0.1	109	6.1	5401
PCDHB12_t1	1.06	0.002397432	0.13	123	9.8	3174	NA	NA	NA	NA	NA	NA	NA	NA
ROBO2	1.05	0.047876418	0.1	144	9.8	3175	0.02	142	2.1	7082	0.17	160	18.47	1555
LSM3	1.14	1.33E-06	0.18	103	9.8	3176	0.09	126	6.8	4351	0.28	112	17.39	1753
CYP11B1	1.07	3.30E-09	0.12	132	9.8	3177	0.05	124	3.68	6069	0.17	119	11.49	3356

YAE1D1	1.41	3.62E-21	0.21	96	9.8	3178	0.21	111	12.7	1928	0.33	128	25.67	542
TMEM14A	1.47	7.53E-08	0.12	131	9.8	3179	0.01	123	0.51	8129	0.25	132	20.88	1139
POLD1	1.65	5.20E-26	0.29	84	9.79	3180	0.19	78	5.37	5138	0.32	102	16.75	1890
ABLIM1	1.22	7.80E-06	0.12	134	9.79	3181	0.07	143	6.57	4469	0.14	132	11.24	3430
SFT2D2	1.28	9.50E-09	0.15	117	9.79	3182	0.22	116	14.35	1439	0.18	128	13.84	2631
WDR62	1.22	2.38E-15	0.21	97	9.78	3183	0.2	88	7.78	3843	0.2	69	3.81	6520
ZNF641	1.11	3.05E-09	0.11	141	9.78	3184	0.18	135	15.01	1276	0.23	127	18.01	1648
RAMP1	1.2	0.185078327	0.14	119	9.78	3185	-0.03	119	-2.11	9906	0.07	109	4.18	6344
TRIM72_t1	1.11	6.12E-06	0.12	134	9.78	3186	NA	NA	NA	NA	NA	NA	NA	NA
HIST1H2BJ	1.64	3.41E-19	0.28	85	9.77	3187	0.13	104	7.28	4105	0.25	101	13	2881
MTCH1	1.18	7.75E-05	0.17	107	9.77	3188	0.17	118	11.72	2224	0.14	109	8.42	4459
FGD1	1.13	5.01E-11	0.11	143	9.77	3189	0.01	132	0.95	7837	0.1	127	7.96	4636
FAM48A	1.01	0.754440293	0.12	129	9.76	3190	0.21	120	14.72	1349	-0.05	141	-4.4	10799
ACRC	1.13	0.004268947	0.08	167	9.76	3191	0.1	112	6.44	4544	0.13	91	5.53	5669
SPATA12	1.05	6.29E-05	0.16	111	9.76	3192	-0.01	152	-1.26	9337	0.19	123	13.89	2622
RNF216	1.09	1.34E-06	0.13	125	9.76	3193	0.17	129	13.51	1680	0.15	150	15.28	2250
HINT1	1.4	1.19E-07	0.25	89	9.76	3194	0.19	110	11.41	2332	0.29	108	16.57	1936
C1QL2	1.06	6.53E-07	0.12	134	9.75	3195	0.06	133	4.96	5365	0.17	108	9.9	3914
TMEM45B	1.46	0.013504188	0.17	107	9.75	3196	0.02	115	1.43	7540	0.17	127	12.9	2920
COX7B	1.16	0.001453693	0.17	108	9.75	3197	-0.01	95	-0.65	8907	0.24	118	16.66	1913
NCSTN	1.46	8.73E-24	0.17	106	9.75	3198	0.21	108	11.97	2132	0.28	125	20.66	1175
HINFP_t1	1.08	0.000171801	0.12	131	9.75	3199	NA	NA	NA	NA	NA	NA	NA	NA
BCORL1	1.25	5.60E-09	0.15	116	9.74	3200	0.1	115	6.22	4653	0.17	125	12.68	2984
TIGD1	1.44	1.75E-20	0.26	87	9.74	3201	0.26	79	7.58	3944	0.38	84	12.91	2917
NANP	1.09	0.001193056	0.13	127	9.74	3202	0.1	142	9.4	3109	0.21	123	15.35	2232
RPS16	1.39	3.19E-10	0.19	100	9.74	3203	0.18	96	8.29	3602	0.19	96	8.78	4309
ZFY	1.11	0.005678147	0.12	134	9.74	3204	0.13	116	8.26	3615	0.16	143	15.02	2331
CYP51A1	1.28	0.004593425	0.11	140	9.74	3205	0.02	139	1.75	7316	0.19	120	13.2	2830
CISD2	1.15	0.000636892	0.09	157	9.74	3206	0.03	130	2.42	6878	0.08	147	8.11	4578
TINF2	1.13	3.64E-07	0.12	134	9.74	3207	0.03	118	1.96	7165	0.01	125	0.57	8278
METTL3	1.26	2.75E-13	0.16	109	9.73	3208	0.09	108	5.48	5068	0.2	121	14.12	2565
APOA1BP	1.38	8.89E-15	0.16	112	9.72	3209	0.05	88	1.8	7272	0.21	90	8.58	4389
CCDC112	1.31	4.50E-05	0.12	133	9.72	3210	0.22	113	13.85	1580	0.19	132	15.48	2201
IL5RA_t1	1.03	0.015206848	0.11	142	9.72	3211	-0.05	150	-4.86	11571	0.05	143	4.76	6046
ARFIP1	1.01	0.917024517	0.12	128	9.72	3212	0.09	133	7.49	3993	0.13	144	12.2	3136
STYX	1.02	0.183408029	0.1	151	9.72	3213	-0.01	127	-0.88	9067	0.17	160	18.91	1472
PDPR	1.04	0.000458576	0.11	136	9.72	3214	-0.02	141	-1.59	9548	0.14	151	14.39	2492
DMRT2	1	0.797625043	0.13	123	9.72	3215	-0.06	150	-5.67	12010	0.06	121	4.58	6135
WDR33	1.03	0.0018081	0.11	142	9.71	3216	0.09	131	7.55	3966	0.13	136	11.11	3472
VPS53	1.02	0.015293956	0.1	143	9.71	3217	0.17	159	18.1	642	0.06	120	4.24	6320
DMBX1	1.09	4.81E-09	0.12	131	9.71	3218	0.01	131	1.14	7719	0.16	124	11.81	3260
ZNF300	1.21	1.73E-07	0.11	138	9.71	3219	0.22	126	16.7	897	0.23	125	17.23	1780
AGRN	1.13	2.16E-07	0.11	135	9.71	3220	0.13	127	9.85	2914	0.12	143	11.57	3334
PCDHB8	1.23	1.26E-07	0.22	95	9.7	3221	0.15	87	5.72	4938	0.14	82	4.39	6251
RRP1B	1.2	1.49E-08	0.12	128	9.7	3222	0.14	126	10.77	2563	0.16	133	12.97	2894
MLXIP	1.06	2.85E-07	0.12	128	9.7	3223	0.07	134	6.1	4725	0.17	126	12.95	2905
HIST1H4G	1.09	3.60E-08	0.21	96	9.7	3224	0.09	83	2.87	6567	0.14	120	9.82	3946
HERC2P4	1.23	4.61E-09	0.12	128	9.69	3225	0.16	127	12.41	2006	0.28	138	24.26	692
BCAT2	1.64	6.37E-18	0.22	95	9.69	3226	0.05	155	5.75	4926	0.22	100	10.8	3573
ZNRF2	1.17	2.02E-07	0.11	137	9.69	3227	0.1	121	7.41	4028	0.19	143	17.89	1663
CNP	1.07	0.009326064	0.13	124	9.69	3228	0.1	145	9.72	2967	0.05	144	4.5	6182
TERF2	1.04	0.053209077	0.12	129	9.69	3229	-0.06	127	-4.57	11424	0.09	147	8.83	4290
LPPR1	1.61	2.16E-07	0.13	123	9.68	3230	0.12	136	9.96	2875	0.18	152	17.91	1659
METTL9_t1	1.09	0.003396075	0.12	129	9.68	3231	NA	NA	NA	NA	0.16	124	11.99	3206
RICTOR	1.01	0.84773951	0.1	143	9.68	3232	0.08	142	7.28	4103	0.12	148	11.33	3406
COPB2	1.13	0.020399246	0.15	116	9.67	3233	0.22	105	12.18	2070	0.08	111	4.9	5973
HMGN2	1.19	0.000104875	0.12	128	9.67	3234	0.08	128	6.59	4457	0.16	137	14.11	2570
NOL6	1.24	3.59E-10	0.14	120	9.67	3235	0.12	128	9.18	3197	0.15	120	10.51	3684
MPP7	1.13	8.54E-06	0.1	148	9.66	3236	0.08	150	8.39	3545	0.13	146	12.46	3047
ZNF786	1.22	1.09E-12	0.18	104	9.66	3237	0.1	128	7.41	4027	0.2	121	14.16	2554
VPS11_t1	1.14	1.76E-05	0.14	118	9.66	3238	NA	NA	NA	NA	NA	NA	NA	NA
ZNF576	1.18	3.47E-09	0.13	127	9.66	3239	0.02	111	1.37	7566	0.14	118	9.27	4127
SCRN3	1.09	8.04E-06	0.12	128	9.66	3240	0.12	126	8.9	3328	0.19	126	14.4	2487
TBX3	1.26	0.000639179	0.11	140	9.65	3241	-0.04	139	-3.4	10733	0.11	134	9	4225
IK	1.15	0.003333703	0.1	147	9.65	3242	0.05	116	3.37	6263	0.15	108	8.51	4422
COX20	1.34	7.41E-08	0.12	130	9.65	3243	0.15	123	11.29	2366	0.24	150	24.04	718
ATF6	1.23	9.35E-07	0.11	140	9.64	3244	-0.01	141	-1.09	9212	0.16	147	15.6	2181
CSN1S1	1.1	1.13E-15	0.16	111	9.64	3245	-0.01	114	-0.34	8704	0.16	130	12.91	2919
TBC1D3B	1.03	0.266965288	0.14	121	9.64	3246	0.09	114	5.84	4877	0.07	129	5.72	5572
HSPA1L	1.17	2.16E-05	0.11	135	9.64	3247	0.03	166	3.54	6153	0.24	107	13.48	2745
CBWD5	1.05	0.134156242	0.14	120	9.63	3248	0.1	121	6.98	4255	0.15	110	8.82	4292
CPLX3	1.1	1.05E-07	0.12	127	9.62	3249	0.15	130	12.24	2052	0.12	133	10.31	3757
GCNT3	1.3	1.11E-06	0.13	122	9.62	3250	0.06	107	3.31	6314	0.15	122	10.5	3691
TAF9_t2	1.28	1.10E-08	0.15	114	9.62	3251	0.15	121	10.96	2487	0.3	109	17.68	1707
ARV1_t1	1.29	3.12E-09	0.19	101	9.62	3252	NA	NA	NA	NA	NA	NA	NA	NA
CTHRC1	2	1.86E-07	0.16	110	9.62	3253	0.24	138	21.24	264	0.12	123	8.92	4252
ORMDL1	1.21	4.65E-06	0.14	119	9.62	3254	0.23	128	18.21	620	0.12	120	8.72	4336
ZNF669	1.14	2.39E-09	0.18	103	9.61	3255	0.2	119	13.76	1611	0.19	120	13.16	2843
HOXC8	1.07	0.001626715	0.12	132	9.61	3256	0.01	145	1.02	7792	0.09	151	9.2	4158
ATP5SL	1.13	7.41E-06	0.17	108	9.6	3257	0.05	135	4.13	5825	0.08	136	6.5	5233
KATNB1	1.19	9.82E-14	0.16	109	9.6	3258	0.02	120	1.26	7634	0.18	107	10.05	3854
IL23R	1.08	1.74E-09	0.12	131	9.6	3259	0.12	127	9.03	3255	0.17	144	15.69	2158
L3MBTL4	1.02	0.043189165	0.12	128	9.59	3260	0.03	140	2.68	6690	0.18	119	12.11	3161
EHMT2_t2	1.22	1.15E-13	0.22	94	9.59	3261	0.11	143	10.03	2849	0.18	95	8.23	4521
HIST1H2AG	1.44	2.40E-12	0.2	97	9.59	3262	0.15	90	6.17	4686	0.19	104	10.13	3824
CCT8L2	1.05	1.61E-07	0.1	147	9.58	3263	0.08	96	3.49	6189	0.14	99	6.67	5168
MAP1B	1.07	0.281424504	0.1	142	9.58	3264	0.17	158	18.77	549	0.1	142	9.19	4160
TATDN1_t1	1.38	1.88E-10	0.3	82	9.58	3265	0.19	120	12.99	1836	NA	NA	NA	NA
BFSP1	1.38	7.70E-11	0.15	113	9.58	3266	0.02	89	0.6	8077	0.26	114	16.91	1852
ST3GAL2	1.09	6.95E-06	0.12	131	9.58	3267	-0.03	127	-1.95	9778	0.13	128	10.25	3781
OAS3	1.08	0.059231074	0.12	132	9.57	3268	0.03	147	2.96	6513	0	136	0.1	8554
NPEPPS_t1	1.16	6.08E-07	0.14	116	9.57	3269	NA	NA	NA	NA	NA	NA	NA	NA
MAGEB6	1.09	0.002685818	0.14	116	9.56	3270	-0.07	132	-6	12187	0.16	112	9.63	4014
NQO1	1.53	7.41E-07	0.13	126	9.56	3271	0.12	127	9.52	3052	0.13	124	9.94	3897
POLR2J	1.15	1.78E-05	0.13	126	9.55	3272	-0.02	124	-1.81	9698	0.17	92	7.3	4913
MATN3	1.18	1.42E-06	0.11	136	9.54	3273	0.15	123	10.81	2550	0.09	176	10.82	3568
TERT	2.24	1.98E-11	0.17	107	9.53	3274	0.12	120	8.47	3518	0.16	111	9.64	4011
DDX50	1.08	0.118038891	0.13	124	9.52	3275	0.15	97	6.91	4297	0.16	106	8.94	4248
ZNF680_t1	1.07	0.375457539	0.1	149	9.51	3276	0.18	133	14.69	1352	NA	NA	NA	NA
ASB7	1.1	0.000163904	0.14	120	9.51	3277	0.11	104	5.68	4959	0.21	132	16.93	1848
SLC37A3_t1	1.05	0.04187723	0.14	120	9.51	3278	0.15	137	13	1832	0.1	136	8.76	4320
EMC8	1.18	1.51E-06	0.11	133	9.51	3279	0.16	139	14.18	1488	0.18	125	13.63	2701
NETO1_t1	1.01	0.225682485	0.13	124	9.51	3280	0.03	138	2.47	6845	0.15	123	10.75	3591
SNX8	1.26	1.02E-09	0.13	122	9.51	3281	0.09	120	5.97	4802	0.15	123	10.77	3587
HDAC8_t1	1.12	7.41E-06	0.16	110	9.51	3282	NA	NA	NA	NA	0.21	98	10.09	3841
PSMD9	1.08	5.20E-08	0.13	124	9.5	3283	0.08	141	7.16	4159	0.19	145	18.22	1607

FDPSL2A	1.61	1.14E-12	0.1	144	9.49	3284	0.14	143	13	1831	0.15	150	14.68	2413
KRIT1_t2	1.09	1.26E-06	0.1	142	9.49	3285	0.16	118	10.62	2621	0.2	135	16.6	1930
ANXA11_t1	1.25	2.94E-07	0.15	115	9.49	3286	NA	NA	NA	NA	0.09	108	5.14	5858
MEPE	1.01	0.548903095	0.13	121	9.49	3287	0.08	106	4.43	5647	0.02	118	1.27	7869
ZNF416	1.2	4.79E-08	0.14	119	9.49	3288	0.1	125	7.84	3818	0.1	125	7.52	4822
RRM2B	1.01	0.81192563	0.12	129	9.48	3289	-0.01	133	-0.48	8803	0.21	143	19.8	1307
FLYWCH2	1.23	2.25E-06	0.16	108	9.48	3290	0.15	108	8.55	3479	0.23	117	15.59	2182
GGCX	1.14	0.000198339	0.1	146	9.48	3291	0.01	134	0.42	8188	0.02	145	2.23	7334
MAP1LC3B_t1	-1.02	0.707937275	0.13	122	9.48	3292	NA	NA	NA	NA	0.05	131	3.93	6450
MCOLN3	1.17	1.47E-05	0.1	147	9.48	3293	0.11	119	7.93	3782	0.08	140	7.02	5026
NCOA2	1.1	0.000298675	0.15	115	9.48	3294	0.02	135	2	7139	0.25	107	14.34	2507
SPAG1_t1	1.06	0.000438024	0.1	141	9.47	3295	NA	NA	NA	NA	0.17	136	14.26	2534
CDAN1	1.14	6.46E-06	0.13	124	9.47	3296	0.06	135	4.89	5397	0.15	147	14.34	2509
ZNF100_t1	1.07	0.237726601	0.11	140	9.47	3297	NA	NA	NA	NA	0.09	136	7.32	4907
TSSC1	1.27	3.74E-13	0.18	102	9.47	3298	0.22	110	12.96	1845	0.17	95	7.76	4729
ZNF343_t1	1.11	1.50E-06	0.13	124	9.47	3299	NA	NA	NA	NA	0.19	129	14.75	2394
PCDHB9_t1	1.05	0.021748152	0.11	133	9.47	3300	NA	NA	NA	NA	0.05	140	4.74	6061
TGS1	1.13	1.84E-05	0.15	114	9.46	3301	0.13	135	11.06	2448	0.25	118	16.79	1878
COPS5	1.4	9.61E-09	0.14	116	9.46	3302	0.16	124	12.07	2113	0.28	88	10.73	3603
KIAA1009_t1	1.22	8.25E-09	0.15	112	9.46	3303	0.21	143	19.19	488	NA	NA	NA	NA
MTDH	1.09	0.095796199	0.09	161	9.46	3304	0.11	143	10.5	2678	0.16	135	13.84	2633
DEPDC4	1.13	2.21E-14	0.13	120	9.45	3305	0.16	103	8.67	3427	0.29	116	19.41	1361
PURG_t1	1.02	0.034820472	0.1	145	9.45	3306	0.07	132	5.39	5124	0.02	133	1.43	7791
RAB9B	1.05	0.00255294	0.13	125	9.45	3307	0.2	128	15.41	1162	0.1	167	11.33	3403
TRIM27	1.19	5.37E-09	0.11	136	9.45	3308	0.15	135	12.8	1896	0.1	110	6.12	5387
THOC7	1.15	5.90E-06	0.16	108	9.45	3309	0.15	120	10.78	2562	0.31	117	20.99	1122
SERPINB1	1.11	0.130713917	0.12	131	9.45	3310	0.15	111	8.99	3275	0.07	122	4.68	6080
CNOT7_t1	1.05	0.308236101	0.11	135	9.44	3311	0.06	136	5.45	5087	0.1	146	9.42	4080
PGK2	1.03	0.003510501	0.15	113	9.44	3312	-0.01	104	-0.34	8710	0.13	127	9.65	4004
RANBP9	1.11	0.023130927	0.12	130	9.44	3313	0.05	120	3.82	5993	0.28	130	22.43	908
EGF	1.11	1.31E-05	0.11	135	9.44	3314	0.03	119	2.21	7006	0.12	138	10.69	3620
BCCIP_t1	1.15	4.42E-07	0.14	118	9.43	3315	0.13	129	10.49	2681	0.26	123	18.88	1480
FOPNL	1.06	0.000871736	0.12	129	9.43	3316	0.13	110	7.97	3764	0.19	132	15.43	2212
HS6ST1	1.1	3.06E-08	0.13	123	9.43	3317	0.01	131	0.81	7936	0.07	130	5.23	5818
LRRTM4_t1	1.03	0.002013089	0.12	127	9.43	3318	NA	NA	NA	NA	0.11	127	8.66	4363
KCNC1	1.09	0.000704557	0.1	148	9.43	3319	0.01	129	1	7807	0.22	128	16.79	1879
RABL3_t1	1.07	0.063790606	0.11	138	9.42	3320	0.07	139	5.79	4895	NA	NA	NA	NA
WDR91	1.22	3.19E-06	0.11	134	9.42	3321	0.26	131	21.15	273	0.04	128	3.1	6872
USF1	1.17	2.97E-17	0.11	134	9.41	3322	0.19	118	12.86	1874	0.17	124	12.28	3118
AQP12A	1.05	0.000671907	0.17	104	9.41	3323	0.07	88	2.51	6823	0.17	117	11.4	3384
PPP1CB_t1	1.06	0.022656999	0.13	124	9.41	3324	NA	NA	NA	NA	0.17	129	13.57	2718
SLC35B4	1.07	0.035546992	0.1	144	9.4	3325	-0.02	141	-1.77	9667	0.21	125	15.6	2180
NMNAT1	1.1	0.000106146	0.12	131	9.4	3326	0.12	143	11.16	2414	0.12	156	12.54	3031
HOXD1	1.27	1.02E-06	0.12	129	9.39	3327	0.11	136	9.61	3018	0.12	155	12.98	2891
NRIP2_t1	1.03	0.029513955	0.11	135	9.38	3328	NA	NA	NA	NA	NA	NA	NA	NA
KPNB1	1.26	8.16E-07	0.14	116	9.38	3329	0.25	108	14.33	1443	0.17	139	15.09	2308
KIAA1199_t1	1.29	1.43E-05	0.12	125	9.37	3330	0.14	124	10.01	2861	NA	NA	NA	NA
BCLAF1	-1.03	0.67792366	0.11	139	9.37	3331	0.15	125	11.05	2455	0.04	144	3.37	6734
EXOSC5	1.31	3.15E-11	0.12	127	9.37	3332	0.11	113	6.91	4296	0.12	94	5.31	5772
GTF2IRD1	1.82	8.39E-18	0.27	85	9.37	3333	0.25	121	17.59	735	0.36	115	23.16	823
GRIA4	1.1	2.94E-06	0.09	149	9.37	3334	0.04	132	3.32	6305	0.12	139	10.72	3606
PEX19	1.24	2.72E-05	0.09	157	9.36	3335	0.09	132	7.29	4093	0.17	134	14.42	2482
CDYL2	1.05	0.076783618	0.13	124	9.36	3336	0.04	144	3.99	5907	0.08	118	5.11	5873
ACAP3_t1	1.11	5.07E-07	0.13	123	9.36	3337	0.13	119	8.79	3378	NA	NA	NA	NA
TPM3_t4	1.3	1.84E-11	0.11	134	9.36	3338	0.23	141	21.16	272	0.16	121	11.49	3355
ERO1L	1.1	0.023673065	0.12	130	9.35	3339	0.03	133	2.08	7095	0.23	126	17.18	1788
RFXAP	1.11	0.000281304	0.12	127	9.35	3340	0.21	149	20.9	300	0.09	154	9.36	4094
GPD1L_t1	1.07	3.80E-05	0.12	131	9.35	3341	NA	NA	NA	NA	NA	NA	NA	NA
ZFX	1.08	0.000813813	0.1	147	9.34	3342	0.14	136	11.75	2209	0.11	123	8.21	4535
PAICS	1.08	0.121814914	0.11	136	9.34	3343	0.05	138	3.99	5905	0.2	144	19.19	1411
MLH1	1.28	6.03E-09	0.13	120	9.34	3344	0.1	105	5.56	5032	0.23	120	16.07	2054
LIMK2_t2	1.06	0.033613469	0.17	106	9.34	3345	0.04	102	2.3	6954	0.01	128	1.02	8006
LMAN2_t1	1.2	8.89E-07	0.18	101	9.34	3346	0.04	113	2.63	6732	NA	NA	NA	NA
ARHGAP12	1.1	0.21455061	0.1	142	9.34	3347	0.1	129	7.86	3804	0.11	164	12.48	3041
LHFPL2_t1	-1.05	0.506036496	0.12	125	9.33	3348	0.15	143	14.25	1466	NA	NA	NA	NA
EMC2	1.29	7.01E-05	0.18	102	9.33	3349	0.18	116	11.62	2268	0.2	117	13.27	2805
TUBB6	1.31	5.38E-09	0.12	128	9.33	3350	0.1	108	5.99	4790	0.15	129	11.83	3255
CLTA	1.21	3.74E-12	0.16	108	9.33	3351	0.14	123	10.33	2746	0.15	119	10.69	3616
ODC1	1.25	0.000581041	0.12	131	9.33	3352	0.07	119	4.56	5579	0.11	126	8.58	4388
NAT9	1.38	7.16E-17	0.12	127	9.33	3353	0.19	125	14.09	1511	0.16	120	11.39	3385
SLC26A11	1.35	1.92E-15	0.16	108	9.33	3354	0.22	108	13.01	1829	0.22	110	13.39	2762
PSG3	1.02	0.147759181	0.13	124	9.33	3355	0	125	-0.2	8612	0.06	121	4.12	6365
BMPR1A	1.11	0.00096258	0.14	118	9.32	3356	0.16	124	11.94	2143	0.27	124	20.27	1237
ZNF681	1.08	0.283705423	0.12	129	9.32	3357	0.16	130	12.9	1865	0.09	146	8.74	4323
RPS27	1.7	3.10E-16	0.27	84	9.32	3358	0.28	108	16.25	992	0.3	100	15.01	2335
IFT81_t2	1.1	1.42E-09	0.15	112	9.32	3359	0.2	123	14.67	1359	0.24	125	17.63	1713
CATSPER1	1.05	0.001532135	0.18	102	9.32	3360	0.25	116	16.57	929	0.02	120	1.39	7807
PHF21B	1.02	0.228082466	0.13	122	9.32	3361	0.08	134	7.14	4178	0.08	145	7.13	4983
ZSCAN16	1.42	1.15E-14	0.16	108	9.32	3362	0.16	130	12.53	1974	0.26	94	11.31	3409
NGFRAP1	1.3	0.025940673	0.12	126	9.31	3363	0.1	116	6.71	4397	0.17	150	16.64	1920
PIK3C2A	-1.02	0.405729607	0.11	131	9.31	3364	0.04	135	3.56	6136	0.13	150	12.94	2910
UHRF2	1.04	0.361724551	0.13	120	9.31	3365	0.08	116	5.53	5051	0.1	147	10	3877
SLC44A1	1.25	0.001428682	0.11	133	9.3	3366	0.15	147	14.57	1387	0.11	162	11.8	3264
CLCC1	1.11	0.014756488	0.12	128	9.3	3367	-0.01	127	-0.54	8844	0.21	132	17.32	1768
ITGA10	1.03	0.001989044	0.1	142	9.3	3368	0.16	125	12.32	2028	0.06	120	4.38	6256
FAM86A	1.19	5.35E-08	0.13	120	9.3	3369	0.24	142	22.02	217	0.13	121	9.29	4124
TRAPPC6B	1.01	0.826824064	0.09	158	9.3	3370	-0.03	129	-2.07	9868	0.07	127	5.11	5870
PNCK	1.06	0.000253119	0.15	111	9.3	3371	0.06	98	2.65	6722	0.14	103	7.44	4858
LALBA	1.04	0.054795935	0.13	121	9.3	3372	0.05	150	5.24	5198	-0.02	122	-1.1	9250
MCPH1	1	0.910858737	0.13	123	9.3	3373	-0.01	111	-0.82	9033	0.14	104	7.49	4834
EPB41_t1	1.02	0.336435328	0.11	131	9.3	3374	0.11	127	8.42	3536	0.02	145	2.34	7268
DNAJB11	1.46	2.39E-15	0.19	100	9.29	3375	0.07	101	3.42	6231	0.24	131	19.04	1441
MS4A6E	1.02	0.072117566	0.13	120	9.29	3376	0	85	0.13	8375	-0.05	96	-2.48	9941
IER3IP1_t1	1.17	0.000235608	0.14	116	9.29	3377	NA	NA	NA	NA	0.2	175	24.86	628
KIAA0556	1.07	2.59E-08	0.15	114	9.29	3378	0.16	123	11.95	2139	0.2	123	14.34	2513
SLC35G2_t1	1.26	4.28E-05	0.16	108	9.28	3379	NA	NA	NA	NA	0.14	97	6.57	5204
EPHA5	1.02	0.055918893	0.09	155	9.28	3380	0.03	127	2.63	6729	0.05	136	3.9	6467
CT45A1	1.19	0.008574292	0.12	127	9.28	3381	0.01	130	0.58	8089	0.12	92	4.86	5997
MESP1	1.62	1.19E-14	0.15	111	9.28	3382	0.1	112	6.12	4717	0.27	104	14.82	2378
PAX6	1.08	0.00108584	0.09	148	9.28	3383	-0.03	135	-2.32	10037	0.16	127	12.42	3063
MFAP5	1.07	0.043519693	0.11	135	9.28	3384	0.07	140	6.15	4705	0.05	150	5.01	5917
POLR3F	1.2	1.94E-10	0.14	117	9.27	3385	0.13	127	9.99	2863	0.35	122	24.99	619
RAB3IP_t1	1.12	0.000181082	0.08	160	9.27	3386	NA	NA	NA	NA	0.09	154	8.92	4254
GPR26	1.01	0.238680356	0.1	140	9.27	3387	0.11	154	11.39	2338	0.03	145	3.08	6885
MKS1	1.12	1.66E-10	0.13	124	9.26	3388	0.09	99	4.31	5711	0.19	123	13.58	2714
FASTKD3	1.36	3.18E-13	0.14	116	9.26	3389	0.19	115	12.41	2007	0.33	130	26.54	453

LENEP	1.04	6.89E-05	0.11	131	9.26	3390	-0.05	130	-3.65	10908	0.07	116	4.95	5952
HIST1H3H	1.33	3.71E-17	0.26	85	9.26	3391	0.18	110	11.02	2468	0.26	80	7.73	4744
ZNF711	1.04	0.012140246	0.1	141	9.26	3392	0.13	123	9.76	2951	0.04	146	4.01	6413
CASD1	1.12	0.003381885	0.14	117	9.26	3393	0.11	129	8.47	3517	0.2	157	21.75	1010
RNF44	1.1	0.002871632	0.11	138	9.25	3394	0.28	126	21.12	275	0.05	148	4.62	6120
PKMYT1_t2	1.15	8.56E-14	0.12	128	9.25	3395	0.07	100	3.32	6307	0.19	120	13.14	2846
THEM6	1.18	1.95E-05	0.14	118	9.25	3396	-0.07	129	-5.61	11978	0.13	131	10.27	3776
PKDREJ	1.16	2.01E-08	0.12	127	9.25	3397	0.02	113	1.07	7755	0.29	114	18.36	1577
FCF1	1.11	1.46E-06	0.12	130	9.25	3398	0.12	117	8.18	3654	0.18	124	13.34	2784
PTPRR	1.08	2.28E-05	0.12	125	9.24	3399	0.15	115	9.48	3069	0.17	118	11.27	3422
KIAA1244_t1	1.06	0.000148782	0.11	136	9.24	3400	NA	NA	NA	NA	0.11	156	11.25	3428
SLC39A6_t1	1.16	5.33E-06	0.13	120	9.24	3401	0.08	111	4.77	5463	NA	NA	NA	NA
LAIR1_t1	1.08	0.0546342	0.14	115	9.22	3402	0.21	128	16.54	936	-0.05	121	-3.85	10564
LSM1	1.02	0.692768489	0.11	135	9.22	3403	0.03	119	1.84	7251	0.1	118	6.55	5214
DCUN1D1	1.09	0.000606347	0.12	130	9.22	3404	-0.03	122	-1.93	9760	0.2	139	17.86	1670
VAT1	1.27	7.67E-05	0.16	109	9.22	3405	0.14	134	11.68	2239	0.1	131	8.21	4537
RCAN3	1.09	0.009076668	0.13	119	9.22	3406	0.09	119	6.53	4497	0.12	119	8.6	4382
LIG1	1.47	1.87E-14	0.18	102	9.22	3407	0.1	104	5.26	5186	0.22	110	12.96	2901
CSMD1	1.24	4.11E-10	0.09	149	9.22	3408	-0.02	154	-1.7	9616	0.14	151	14.49	2463
CAPNS1	1.06	0.013264531	0.11	131	9.22	3409	0.07	124	4.98	5349	0.01	116	0.48	8327
RASSF4	1.51	9.70E-11	0.13	119	9.22	3410	0.2	116	13.07	1809	0.14	131	11.39	3386
IGBP1	1.14	0.001391731	0.15	111	9.21	3411	0.14	120	9.78	2942	0.12	131	10	3876
SEPT3_t1	1.03	0.002522665	0.11	134	9.21	3412	NA	NA	NA	NA	0.19	126	14.34	2506
CRTAP	1.07	0.244316707	0.11	132	9.21	3413	0.06	127	4.47	5623	0.06	135	4.8	6031
LRRC46	1.12	2.99E-07	0.15	112	9.21	3414	0.01	110	0.6	8071	0.15	103	7.88	4675
PSKH1	1.39	1.88E-10	0.13	119	9.21	3415	0.11	133	9.43	3098	0.16	117	10.66	3630
MSRB3	1.22	0.000126031	0.1	146	9.2	3416	0.16	163	18.5	584	0.05	138	4.69	6077
ATP8B2	1.07	7.50E-06	0.1	143	9.2	3417	0.17	136	14.93	1297	0.12	96	5.4	5733
MPPED2	1.08	8.49E-05	0.12	128	9.2	3418	0.07	131	5.65	4983	0.23	150	23.27	813
PPM1G	1.25	6.14E-10	0.16	106	9.19	3419	0.11	128	8.41	3541	0.18	125	13.18	2835
EIF1AX	1.18	0.002844456	0.11	134	9.19	3420	0.04	148	4.06	5876	0.22	135	18.88	1481
FUNDC2	1.2	0.000169652	0.11	135	9.18	3421	0.12	149	11.44	2321	0.07	133	5.56	5651
ZCCHC3	1.03	0.004337101	0.1	138	9.17	3422	0.01	154	1.33	7588	0.11	133	9.31	4114
DLL3_t1	1.06	0.000498493	0.15	111	9.16	3423	0.11	94	4.68	5506	0.01	119	0.51	8314
SEC22C_t2	1.07	0.014304553	0.12	127	9.16	3424	0.05	124	3.64	6091	0.13	120	9.44	4076
ANKRD16	1.41	4.79E-20	0.29	82	9.16	3425	0.09	106	5.1	5274	0.3	89	11.54	3341
ZFP41	1.09	1.15E-08	0.12	125	9.16	3426	0.02	144	2.18	7018	0.14	131	11.07	3491
DTNB_t1	1.14	4.30E-08	0.16	106	9.15	3427	0.07	142	6.79	4357	0.06	104	3.09	6876
SUN3	1.1	0.004840962	0.12	126	9.15	3428	0.07	114	4.57	5575	0.1	104	5.28	5787
OLFML2B	1.24	3.25E-12	0.18	100	9.15	3429	0.23	111	14.2	1481	0.18	123	13.11	2851
FBXO40	1.08	2.36E-09	0.11	135	9.15	3430	-0.06	132	-4.72	11498	0.11	143	10.15	3817
DPAGT1	1.22	6.20E-10	0.14	116	9.15	3431	0.15	139	13.12	1797	0.16	124	11.72	3291
CEP112_t1	1.09	1.16E-07	0.15	112	9.15	3432	NA	NA	NA	NA	0.16	131	12.59	3010
NF2_t1	1.05	0.000672374	0.18	100	9.15	3433	0.07	126	5.26	5181	0.12	102	6.48	5251
HAUS7	1.05	0.000936077	0.17	104	9.15	3434	0	98	0.13	8386	0.09	119	6.37	5286
RGS20	1.09	5.33E-09	0.1	140	9.14	3435	0.07	117	4.63	5534	0.12	125	9.28	4126
ANKRD34A	1.05	9.37E-05	0.1	145	9.14	3436	0.13	147	12.32	2027	0.17	135	14.15	2557
SOX12	1.15	1.77E-15	0.13	120	9.14	3437	0.08	129	6	4780	0.23	130	18.14	1616
RC3H2	1.08	0.004397956	0.13	118	9.14	3438	0.12	91	4.82	5441	0.18	109	10.72	3604
PIP5K1A	1.08	6.60E-08	0.11	136	9.14	3439	0.13	124	9.63	3006	0.05	123	3.95	6438
EFHC2_t1	1.1	0.000330576	0.14	115	9.14	3440	0.12	130	9.24	3167	NA	NA	NA	NA
ZNF146_t1	1.06	3.01E-05	0.14	117	9.14	3441	0.17	125	12.39	2012	NA	NA	NA	NA
PCDHA1	1.2	9.27E-05	0.17	104	9.13	3442	0.07	89	2.86	6578	0.1	106	5.86	5515
DLG1	1.02	0.525284367	0.09	153	9.13	3443	-0.02	127	-1.84	9721	0.15	135	12.38	3074
UBE2F	1.05	0.131060675	0.13	118	9.12	3444	0.13	128	10.36	2736	0.02	151	1.8	7569
HSPA8_t2	1.05	0.248069788	0.09	151	9.12	3445	-0.03	108	-1.63	9573	0.15	165	16.81	1868
RPS8	1.43	1.58E-08	0.24	88	9.11	3446	0.27	88	10.17	2800	0.26	84	9	4227
UBE3B	1.17	3.43E-11	0.13	120	9.11	3447	0.1	125	7.32	4083	0.2	157	21.69	1019
THBS4	3.12	4.38E-21	0.19	97	9.11	3448	0.18	110	11.1	2428	0.34	92	14.35	2504
VPS13B_t4	1.1	5.81E-08	0.21	94	9.11	3449	0.1	101	4.95	5375	0.29	97	13.41	2757
ARHGAP1	1.13	0.001112974	0.12	128	9.11	3450	0.15	147	14.86	1313	0.08	151	7.88	4674
SNRPB	1.22	1.81E-05	0.19	97	9.1	3451	0.07	105	4.05	5880	0.12	95	5.36	5753
NAF1	1.04	9.44E-05	0.15	109	9.09	3452	0.14	100	7.08	4209	0.2	139	18.04	1637
RBM19	1.17	2.63E-09	0.13	122	9.09	3453	0.12	115	7.52	3977	0.07	120	4.57	6140
TACC3	1.55	1.41E-17	0.15	110	9.09	3454	0.17	108	9.88	2904	0.25	105	13.53	2731
DGCR2	1.25	3.80E-06	0.1	139	9.09	3455	0.13	134	10.89	2520	0.04	151	3.87	6491
ZACN	1.09	2.14E-11	0.2	95	9.09	3456	0.08	100	4.1	5845	0.16	100	7.91	4658
DNM3	1.26	1.16E-07	0.09	151	9.08	3457	0.26	137	22.9	158	0.19	146	18.06	1634
FCRL4_t1	1.03	0.005389257	0.11	133	9.08	3458	NA	NA	NA	NA	0.07	118	4.52	6166
TMEM54	1.16	0.076600325	0.16	108	9.08	3459	0.01	95	0.64	8040	0.06	120	4.46	6205
NKAIN4	1.08	7.18E-11	0.12	123	9.08	3460	-0.02	116	-1.5	9498	0.18	100	9.05	4209
MTHFSD	1.07	0.011789584	0.14	115	9.08	3461	0.06	158	6.32	4608	0.06	119	4.42	6235
SETD4_t2	1.15	2.19E-07	0.11	130	9.08	3462	0.19	120	13.15	1786	0.08	120	5.69	5582
HIST1H2BB	1.67	6.36E-17	0.27	84	9.08	3463	0.08	114	5.17	5237	0.24	100	12.11	3160
RALGAPA1_t1	1.22	0.002413193	0.1	141	9.08	3464	0.15	129	11.74	2220	0.16	138	13.83	2641
ZNF676	1.13	0.000187994	0.13	120	9.08	3465	0.18	127	14.12	1501	0.18	100	8.86	4275
DHCR7	1.56	7.90E-07	0.17	104	9.07	3466	0.08	133	6.87	4315	0.21	116	13.54	2726
GRAMD1A	1.08	0.02616628	0.14	117	9.07	3467	0.08	99	4.14	5818	0.03	120	2.23	7331
ANKRD39	1.26	2.23E-13	0.14	113	9.07	3468	0.07	110	4.14	5820	0.18	111	11.05	3497
RELL2	1.27	1.64E-12	0.17	103	9.07	3469	0.11	104	5.9	4840	0.27	127	20.51	1196
LRP8_t1	1.18	0.004941997	0.11	134	9.06	3470	0.15	138	13.2	1774	NA	NA	NA	NA
UBE2L3	1.15	0.000247639	0.11	129	9.06	3471	0.21	151	21.69	238	0.07	126	5.21	5829
FAM213B	1.33	4.18E-12	0.14	115	9.06	3472	0.21	134	17.87	690	0.09	120	6.47	5252
SRA1	1.29	3.39E-10	0.16	105	9.06	3473	0.06	120	4.36	5681	0.23	140	20.78	1162
CCDC51	1.32	1.20E-16	0.15	112	9.06	3474	0.15	109	8.98	3282	0.25	120	17.73	1694
NUFIP2	-1.12	0.005316005	0.08	158	9.06	3475	-0.03	141	-3.06	10510	0.05	142	4.54	6156
SMARCAL1	1.19	7.05E-09	0.24	88	9.06	3476	0.23	82	7.41	4030	0.09	92	3.75	6546
EP300	1.02	0.598636293	0.13	121	9.06	3477	0.08	122	5.96	4804	0.13	130	10.29	3767
CDK14	1.28	5.85E-08	0.11	131	9.05	3478	0.15	147	14.18	1487	0.29	137	25.26	588
RTN3_t2	1.16	1.96E-05	0.11	132	9.05	3479	0.17	132	13.81	1596	0.15	137	13.26	2810
PSEN2_t2	1.17	0.000968143	0.11	133	9.05	3480	0.04	137	3.23	6357	0.14	119	9.86	3928
OAZ3	1.42	1.82E-14	0.18	100	9.04	3481	0.18	111	10.92	2507	0.35	93	15.17	2284
JPH1	1.08	1.04E-09	0.11	135	9.04	3482	0.02	130	1.33	7584	0.21	128	16.39	1984
SENP2	1.03	0.404705735	0.1	138	9.04	3483	-0.08	124	-5.82	12099	0.01	119	0.6	8258
MRRF_t2	1.08	0.000771755	0.12	127	9.04	3484	0.09	128	7.35	4062	0.04	148	4.13	6359
ADAM15	1.24	6.27E-12	0.18	100	9.03	3485	0.11	128	8.6	3453	0.24	117	15.86	2110
FAM75A2	1.03	0.002930689	0.17	103	9.03	3486	0.12	104	6.54	4485	0.07	91	2.97	6944
TUBA1A	1.3	2.85E-10	0.17	104	9.03	3487	0.1	109	5.87	4856	0.13	102	6.81	5100
TRAM1L1	1.49	2.24E-11	0.13	118	9.03	3488	0.15	114	9.83	2921	0.19	133	16.01	2070
ARMC8_t2	1.01	0.852097657	0.12	123	9.03	3489	0.13	116	8.35	3570	0.02	136	1.83	7550
CEP44	1.04	0.232744648	0.11	130	9.03	3490	0.1	134	8.38	3552	0.19	132	15.18	2280
ZCCHC9	1.35	1.43E-10	0.14	116	9.02	3491	0.1	118	6.74	4384	0.27	120	19.14	1423
SUSD4_t1	1.27	3.22E-05	0.12	124	9.02	3492	-0.06	130	-4.48	11374	0.1	118	6.65	5175
SERPINB5	1.02	0.125071487	0.11	132	9.02	3493	0.08	148	7.69	3885	0.03	131	2.39	7238
EIF4EBP2	1.03	0.404542582	0.09	145	9.02	3494	-0.1	145	-9.08	13552	0.05	147	4.63	6117
FAM131A	1.08	0.120783873	0.11	134	9.02	3495	0.1	128	7.56	3959	0.01	114	0.8	8133

ZNF235	1.03	0.354292072	0.09	146	9.02	3496	0.11	114	7.01	4237	0.02	130	1.42	7801
TIMM8A	1.27	4.37E-08	0.12	127	9.02	3497	0.03	125	1.96	7168	0.23	114	14.77	2389
KIAA0513_t1	1.01	0.915068357	0.1	140	9.02	3498	0.12	142	10.94	2495	NA	NA	NA	NA
FKBP1A_t2	1.14	6.38E-06	0.19	97	9.02	3499	NA	NA	NA	NA	0.17	111	10.42	3717
DENND1A_t1	1.14	2.49E-05	0.12	123	9.01	3500	0.15	111	8.88	3341	0.01	105	0.32	8428
CHUK	1.06	0.031711261	0.1	137	9	3501	-0.03	133	-2.56	10186	0.24	154	24.73	644
UCHL1	1.37	0.008886481	0.11	133	9	3502	0.13	110	7.75	3858	0.09	102	4.59	6130
SYNGAP1	1.04	9.52E-05	0.14	115	9	3503	-0.04	123	-3.02	10480	0.13	124	9.78	3954
SNRNP200	1.24	9.85E-13	0.13	119	9	3504	0.19	114	12.37	2019	0.12	119	8.57	4397
KIFAP3	1.32	8.99E-06	0.15	112	9	3505	0.18	122	13.18	1779	0.23	112	14.39	2490
QPCTL	1.12	2.39E-08	0.13	119	8.99	3506	0.08	139	6.86	4317	0.07	120	5.14	5860
STK17A	1.09	0.02230121	0.1	140	8.99	3507	0.2	138	17.84	694	0.09	143	8.78	4308
ZDHHC17	1.03	0.045602611	0.09	145	8.99	3508	0.15	143	14.1	1507	0.08	136	6.97	5049
KCNK2	1.02	0.190362637	0.11	135	8.99	3509	0.05	132	4.07	5861	0.02	135	1.97	7470
HSPA9	1.18	0.00199401	0.13	120	8.99	3510	0.08	122	5.67	4967	0.3	120	21.11	1098
PAK1	1.08	0.059071625	0.1	144	8.99	3511	0.06	132	5.16	5246	0.03	134	2.49	7185
LAMA4	1.3	0.000256136	0.12	127	8.98	3512	0.2	133	16.27	988	0.15	134	12.71	2970
NAA30_t1	1.12	8.62E-05	0.11	133	8.98	3513	0.04	128	2.98	6510	NA	NA	NA	NA
TTTY5	-1	0.924121959	0.1	142	8.97	3514	-0.05	136	-4.43	11342	0.05	131	3.85	6498
PDE4D	1.04	0.070746463	0.09	151	8.97	3515	0.08	136	6.54	4490	0.02	142	2.03	7434
GGH	1.83	2.39E-07	0.16	105	8.97	3516	0.07	96	3.29	6326	0.3	115	19.59	1334
ZNF34	1.16	5.68E-07	0.24	88	8.97	3517	0.08	110	5.03	5315	0.27	100	13.47	2748
RFC5	1.33	3.62E-11	0.12	123	8.97	3518	0.06	113	3.75	6033	0.18	139	16.39	1985
CYTH3	1.11	1.65E-05	0.1	138	8.97	3519	0.28	137	24.31	103	0.11	136	9.47	4061
SNF8	1.35	7.44E-17	0.16	107	8.97	3520	0.06	94	2.54	6805	0.24	142	21.66	1021
MRPL50	1.19	0.000522701	0.13	121	8.97	3521	0.23	113	14.2	1476	0.14	137	12.48	3043
DDX23	1.16	2.70E-09	0.13	120	8.96	3522	0.19	108	10.79	2557	0.11	159	11.78	3272
LHX9	1.06	6.81E-05	0.11	129	8.96	3523	0.01	142	0.66	8030	0.1	135	8.76	4319
ZFP90	1.08	0.066830206	0.1	139	8.96	3524	0.02	126	1.31	7598	0.14	139	12.04	3180
MITD1	1.15	6.43E-05	0.14	116	8.96	3525	0.18	107	10.27	2769	0.11	115	7.1	4991
CCDC59	1.05	0.093768197	0.11	131	8.96	3526	0.14	124	10.51	2676	0.14	121	9.72	3976
DEFB129	1.08	7.40E-09	0.15	110	8.95	3527	0.04	110	2.46	6851	0.11	97	5.1	5877
LRP12_t1	1.28	4.62E-05	0.12	122	8.95	3528	NA	NA	NA	NA	NA	NA	NA	NA
PMF1	1.29	1.65E-12	0.13	121	8.95	3529	0.03	115	1.74	7321	0.13	106	7.34	4898
MAGOH	1.13	0.003100988	0.14	114	8.95	3530	0.12	90	4.99	5340	0.14	92	5.98	5464
ARHGEF5	1.13	0.057225994	0.12	123	8.95	3531	-0.02	124	-1.35	9400	0.1	148	10.27	3775
BCAN_t1	1.03	0.004897619	0.14	112	8.94	3532	-0.01	123	-0.71	8944	0.2	119	13.67	2686
WDR83	1.28	1.58E-13	0.2	95	8.94	3533	0.02	92	0.8	7939	0.2	80	5.9	5493
ANAPC5	1.2	2.52E-09	0.12	123	8.94	3534	0.2	112	12.12	2093	0.08	114	4.87	5991
STAG1	1.04	0.073442778	0.14	116	8.94	3535	0.12	124	8.68	3419	0.14	126	10.56	3672
GABPB1_t2	1.03	0.287307859	0.11	129	8.94	3536	0.1	115	6.65	4419	0.14	126	10.81	3570
DEK	1.09	0.234069727	0.13	119	8.93	3537	0.09	126	6.81	4346	0.21	158	22.92	853
NUP93	1.25	1.00E-08	0.12	122	8.93	3538	0.11	116	7.38	4042	0.12	121	8.72	4335
KDM1A	1.06	0.005325907	0.12	126	8.93	3539	0.1	96	4.53	5600	0.12	111	7.56	4812
MOBP_t1	1.04	8.28E-05	0.1	138	8.93	3540	NA	NA	NA	NA	0.07	134	5.5	5683
FAM69B	1.27	5.23E-05	0.15	110	8.93	3541	0.14	123	10.26	2774	0.18	106	10.16	3805
SH3GL3	1.25	0.001001882	0.11	130	8.93	3542	-0.04	132	-3.37	10715	0.06	132	5.1	5883
L1CAM_t2	1.05	5.89E-09	0.13	117	8.93	3543	0.17	124	12.66	1940	0.08	127	6.25	5339
AKR1C1_t1	1.32	0.008175573	0.15	110	8.93	3544	NA	NA	NA	NA	0.08	116	5.44	5720
PORCN	1.17	1.23E-06	0.12	124	8.91	3545	0.02	110	1.42	7543	0.07	99	3.58	6623
SPNS1	1.18	1.92E-11	0.15	109	8.91	3546	0.07	108	4.19	5779	0.13	100	6.58	5195
PRPS2	1.02	0.594414998	0.11	128	8.91	3547	0.04	123	2.65	6718	0.02	124	1.74	7609
TBCB	1.3	3.93E-11	0.16	104	8.91	3548	0.18	100	9.1	3228	0.1	107	5.58	5629
MYCL1	1.23	5.19E-05	0.13	120	8.91	3549	0.08	150	7.71	3877	0.16	113	9.93	3902
SLC6A8	1.28	3.48E-07	0.13	120	8.9	3550	0.1	142	8.82	3368	0.1	131	8.29	4499
TEX35_t1	1.04	4.22E-06	0.16	104	8.9	3551	NA	NA	NA	NA	0.14	99	7.04	5019
STXBP5	1.1	0.00016001	0.08	157	8.9	3552	0.17	131	14.05	1519	0.11	162	12.62	3002
ARF5_t1	1.12	0.000370843	0.15	111	8.89	3553	NA	NA	NA	NA	0.13	127	10.28	3774
SPTLC1_t1	1.03	0.279144679	0.12	126	8.89	3554	0.08	131	6.44	4547	0.03	121	2.35	7261
TJP1	1.15	0.033861109	0.13	117	8.88	3555	0.07	123	4.77	5460	0.23	131	18.32	1589
CYB5R3	1.1	0.106157949	0.14	112	8.88	3556	0.17	131	13.82	1590	0.12	119	8.13	4568
NECAP1	-1.02	0.221946023	0.1	141	8.87	3557	-0.01	132	-1.04	9174	0.03	122	2.07	7414
SLC12A2_t1	1.06	0.531616072	0.08	155	8.87	3558	NA	NA	NA	NA	0.03	129	2.31	7288
FAM173B_t1	1.21	5.39E-07	0.12	124	8.87	3559	0.1	131	8.35	3572	NA	NA	NA	NA
HES5	1.28	2.67E-10	0.14	113	8.87	3560	0.09	114	5.92	4829	0.22	132	18.03	1644
FTMT	1.16	2.92E-07	0.15	108	8.87	3561	0.07	116	4.47	5621	0.1	113	6.34	5300
HMGA1	1.03	0.107374538	0.08	165	8.86	3562	0.07	120	4.97	5355	0.02	124	1.8	7570
GPR137B	-1.12	0.099001	0.13	120	8.86	3563	-0.01	132	-1.2	9291	-0.08	130	-6.09	11474
SCML1	1.09	0.145561049	0.11	129	8.85	3564	0.01	119	0.45	8172	0.17	127	13.25	2814
PCSK9	1.34	0.00739979	0.12	123	8.85	3565	0.06	117	3.96	5924	0.08	120	5.59	5628
TMEM8B_t1	1.43	2.20E-12	0.22	90	8.85	3566	0.19	80	5.76	4921	0.22	104	11.79	3269
MZT2B	1.23	7.71E-08	0.18	100	8.85	3567	0.19	80	5.63	4989	0.21	106	11.78	3271
THEM4	1.37	2.82E-17	0.1	136	8.84	3568	0.21	124	15.9	1069	0.22	135	18.76	1511
ACPL2	1.36	6.43E-08	0.09	147	8.84	3569	0.08	127	6.2	4664	0.22	156	22.92	854
SMYD5	1.09	1.97E-06	0.12	126	8.84	3570	0.09	141	7.82	3823	0.1	120	7	5033
PAIP2	1.15	0.007340954	0.13	118	8.83	3571	0.07	132	5.54	5043	0.15	127	11.64	3315
NUDT21_t1	1.02	0.621777514	0.1	141	8.83	3572	NA	NA	NA	NA	-0.01	136	-1.22	9307
SHKBP1	1.1	2.99E-06	0.18	99	8.83	3573	0.06	136	5.07	5294	0.04	94	1.84	7540
UBE2K	1.2	0.000129305	0.09	146	8.82	3574	0.14	132	11.39	2337	0.24	154	24.83	629
CMIP	-1.05	0.028147338	0.11	128	8.82	3575	0.04	140	3.25	6343	0.05	127	3.66	6585
GPAA1	1.39	4.34E-16	0.22	90	8.82	3576	0.03	108	1.72	7333	0.26	70	5.23	5817
HAX1	1.49	5.55E-20	0.21	93	8.82	3577	0.15	107	8.44	3530	0.24	118	16.34	1995
KRT75	1.03	0.001723819	0.13	117	8.82	3578	0.04	115	2.71	6669	0.21	113	13.01	2878
GART_t2	1.19	3.81E-06	0.13	116	8.82	3579	0.15	91	6.16	4695	0.14	110	8.57	4399
DNMT1	1.24	2.48E-09	0.13	117	8.82	3580	0.16	113	10.11	2822	0.11	120	7.95	4641
PRR13	1.12	1.44E-06	0.1	141	8.82	3581	0.12	125	8.67	3425	0.09	163	9.63	4013
ACSBG2	1.07	1.94E-05	0.13	118	8.82	3582	-0.04	131	-3.43	10751	0.07	118	4.64	6105
MRPL45	1.2	8.97E-11	0.13	120	8.81	3583	0.02	136	1.86	7234	0.21	133	17.17	1795
GLP1R	1.02	0.038862863	0.1	139	8.81	3584	-0.06	140	-5.41	11876	0.14	120	9.99	3883
ZFHX3	-1	0.94260035	0.08	155	8.81	3585	0.01	141	0.85	7900	0.05	132	4.26	6310
BTN1A1	1.06	5.53E-10	0.1	136	8.81	3586	-0.01	127	-0.44	8778	0.05	126	3.53	6657
MARS	1.49	6.13E-15	0.23	88	8.81	3587	0.23	105	12.85	1878	0.24	84	8.31	4488
CYTL1	1.1	6.98E-05	0.14	115	8.8	3588	0.17	114	10.74	2579	0.16	138	13.84	2632
METTL1_t1	1.22	3.88E-08	0.14	114	8.8	3589	NA	NA	NA	NA	NA	NA	NA	NA
ZNF254	1.05	0.436146374	0.1	140	8.8	3590	0.17	139	15.35	1182	0.12	147	11.43	3372
ADPRH	1.01	0.556108865	0.1	136	8.8	3591	0.03	144	2.7	6683	0.03	150	3.01	6922
DHX40	1.07	0.017075065	0.11	128	8.8	3592	0.06	139	5.44	5098	0.15	125	11.43	3371
BUB1B	1.46	9.34E-15	0.13	118	8.8	3593	0.08	111	5.07	5284	0.29	130	23.01	842
CCDC43	1.1	0.000132981	0.11	130	8.8	3594	0.16	149	15.83	1083	0.16	119	11.38	3389
PTP4A3	1.23	4.02E-08	0.15	109	8.79	3595	0.13	120	8.84	3356	0.13	120	9.32	4113
CENPQ	1.44	5.50E-12	0.13	120	8.79	3596	0.25	116	16.42	956	0.26	120	17.96	1654
ZAR1	1.06	0.001058967	0.13	118	8.79	3597	0.01	124	1.04	7784	0.1	130	8.19	4541
CD2AP	1.08	0.025244198	0.11	133	8.79	3598	0.14	131	11.48	2313	0.2	145	18.72	1518
KAT6B_t1	1.01	0.544272499	0.11	131	8.78	3599	NA	NA	NA	NA	0.06	127	4.88	5988
HIST1H2AL	1.18	2.27E-12	0.26	84	8.78	3600	0.18	113	11.42	2329	0.25	85	8.68	4352
SUCLA2_t1	-1.02	0.710700987	0.1	141	8.78	3601	NA	NA	NA	NA	0.07	119	4.56	6144

TP53TG3	1.33	7.78E-06	0.11	129	8.78	3602	0.05	136	4.03	5889	0.19	133	15.95	2084
RSU1	1.06	0.000781597	0.12	125	8.78	3603	0.11	135	9.03	3256	0.17	153	17.5	1738
TCTEX1D2	1.38	1.24E-07	0.13	118	8.78	3604	0.17	81	5.37	5141	0.09	106	4.8	6026
EXOC3	1.08	0.00219653	0.1	134	8.78	3605	0.02	106	1.29	7614	0.11	118	7.38	4878
DRG2	1.16	5.03E-06	0.16	106	8.78	3606	0.04	124	2.85	6583	0.14	120	10.01	3873
SDCBP	1.01	0.867631602	0.1	136	8.76	3607	0.04	127	2.76	6632	0.15	120	10.35	3740
ST7L_t1	1.08	5.58E-05	0.15	108	8.76	3608	0.14	88	5.35	5145	0.13	96	6.04	5433
NSUN3	1.05	0.067453146	0.11	127	8.76	3609	0.1	110	6.19	4668	0.08	112	5.08	5891
NDC80	3.5	5.37E-31	0.29	80	8.75	3610	0.22	86	8	3744	0.4	81	12.33	3101
ZNF227	1.09	0.004576554	0.15	110	8.75	3611	0.08	120	5.86	4869	0.1	112	5.93	5482
CTCF	1.02	0.026959391	0.1	135	8.75	3612	0.04	120	2.74	6646	0.05	127	4.06	6390
GNB1	1.17	1.56E-05	0.11	132	8.75	3613	0.09	132	7.15	4170	0.03	126	2.56	7156
ASB4_t1	1.15	7.81E-15	0.11	131	8.75	3614	NA	NA	NA	NA	0.04	136	3.83	6509
MSI2_t2	1.18	4.74E-07	0.11	132	8.74	3615	0.16	134	13.6	1654	0.11	130	8.62	4377
CXADR	1.22	0.001046679	0.09	143	8.74	3616	0.09	147	8.47	3519	0.18	129	14.01	2595
ATL2	1.07	0.001803182	0.12	120	8.74	3617	0.07	119	4.49	5616	0.25	135	21.55	1041
KANSL1L	1.1	2.37E-06	0.12	124	8.72	3618	0.08	135	6.99	4253	0.11	129	8.95	4246
DDX6	1	0.840152446	0.09	150	8.72	3619	0.14	162	15.58	1127	-0.01	149	-1.34	9360
DEF8_t2	1.09	0.001806188	0.11	127	8.71	3620	0.03	131	2.34	6933	0.02	150	2.34	7269
ANO4	1.09	0.000543845	0.11	126	8.71	3621	0.17	137	15.08	1256	0.08	133	6.49	5243
ZNF19	1.03	0.063975395	0.11	127	8.7	3622	-0.06	143	-5.63	11993	0.08	134	6.33	5308
RNF20	1.11	0.001226294	0.12	124	8.7	3623	0.03	127	2.53	6806	0.17	132	13.91	2616
APPBP2	1.16	0.003100988	0.1	135	8.7	3624	0.06	137	5.65	4977	0.2	147	19.42	1360
ZNF304	1.1	0.002381533	0.1	133	8.7	3625	0.15	120	10.21	2791	0.13	143	12.39	3073
SEC61A2	1.27	5.36E-07	0.12	120	8.7	3626	0.01	138	1.21	7674	0.23	131	18.45	1559
SEC61B_t1	1.27	4.77E-07	0.14	113	8.69	3627	0.24	108	14.04	1520	NA	NA	NA	NA
TTC3_t1	-1.01	0.774504306	0.11	132	8.69	3628	NA	NA	NA	NA	NA	NA	NA	NA
SLC10A7	1	0.957758877	0.1	133	8.69	3629	0.09	122	6.32	4609	0.08	135	6.92	5063
SLC30A7	1.1	4.55E-06	0.1	141	8.69	3630	0.18	125	13.66	1637	0.17	153	17.04	1818
FSTL4	1.11	2.23E-09	0.13	119	8.68	3631	-0.01	129	-1.05	9185	0.23	116	14.97	2345
ZNF41	1.04	0.013714339	0.12	124	8.68	3632	0.03	123	2.39	6897	0.19	133	15.55	2190
TACC1	1.06	0.13799154	0.1	134	8.68	3633	0.06	147	5.78	4910	0.01	154	1.09	7974
LRRC3B	1.09	1.01E-05	0.13	117	8.68	3634	0.1	112	5.94	4816	0.21	127	16.22	2028
TRAPPC2	1.14	8.19E-06	0.08	153	8.68	3635	0.1	127	7.65	3905	0.14	129	10.86	3562
PCGF3	1.15	9.45E-07	0.1	138	8.68	3636	0.16	130	12.72	1920	0.11	134	9.04	4215
NF1	1.05	0.073950192	0.11	130	8.68	3637	0.09	120	6.15	4700	0.19	137	16.76	1887
SPTAN1	1.22	6.14E-10	0.14	112	8.67	3638	0.09	120	6.04	4756	0.17	119	11.74	3285
DDR1	-1.13	0.305835425	0.1	135	8.67	3639	0.1	138	8.39	3547	-0.04	131	-3.04	10196
WDR48	1.09	0.01678605	0.12	121	8.67	3640	0.07	129	5.33	5152	0.18	135	14.99	2339
GIGYF1	1.19	1.28E-08	0.1	135	8.66	3641	0.05	129	3.67	6072	0.12	135	9.97	3889
GIT1	1.04	0.013297294	0.16	104	8.66	3642	0.07	154	7.68	3891	0.02	108	1.23	7892
SREBF2	1.18	2.55E-05	0.12	120	8.66	3643	0.07	100	3.36	6279	0.11	115	6.98	5041
DNAJB7	1.03	0.000766704	0.09	144	8.66	3644	0.11	135	9.28	3154	0.15	160	16.27	2011
PEX2	1.23	1.55E-05	0.1	136	8.66	3645	0.08	138	7.17	4155	0.18	155	18.58	1538
ZNF134_t1	1.01	0.834693616	0.09	144	8.65	3646	NA	NA	NA	NA	NA	NA	NA	NA
DAP	1.14	0.000990709	0.1	137	8.65	3647	0.06	129	5.01	5330	0.06	120	4.04	6396
ZNF425	1.22	8.53E-08	0.13	116	8.65	3648	0.02	139	1.63	7403	0.1	124	7.48	4840
GSTM3_t1	-1.01	0.936745897	0.1	136	8.65	3649	NA	NA	NA	NA	NA	NA	NA	NA
NDUFS5	1.11	0.003723426	0.15	107	8.64	3650	0.11	91	4.58	5570	0.16	96	7.33	4903
ZCCHC12	1.03	0.001071834	0.1	140	8.64	3651	-0.01	108	-0.78	9005	0.11	102	5.55	5662
PRR15	1.4	2.54E-05	0.15	109	8.64	3652	0.1	125	7.35	4068	0.05	152	5.51	5678
KRT83	1.02	0.112902564	0.16	104	8.64	3653	0.02	131	1.71	7340	0.09	113	5.41	5727
HIST1H2AJ_t1	1.62	4.46E-15	0.21	92	8.63	3654	NA	NA	NA	NA	NA	NA	NA	NA
TIFA_t1	1.1	0.073415373	0.1	135	8.63	3655	NA	NA	NA	NA	0.04	125	2.67	7098
SLC26A7_t1	1.07	0.00109392	0.09	145	8.63	3656	0.15	138	12.9	1869	0.03	138	2.83	7023
MYL6	1.14	0.000501017	0.13	119	8.63	3657	0.04	94	1.61	7417	0.08	118	5.29	5784
DNASE1	1.1	5.38E-10	0.23	88	8.63	3658	0.13	128	10.26	2775	0.26	111	15.77	2140
CDH18	1.21	4.72E-05	0.11	126	8.63	3659	-0.05	113	-3	10471	0.19	100	9.29	4119
NEK8	1.09	9.03E-08	0.14	111	8.63	3660	0.07	116	4.79	5451	0.1	111	6.08	5411
WIF1	1.08	0.005815169	0.14	113	8.62	3661	0.05	123	3.37	6272	0.17	134	14.03	2589
GUCY2F	1.08	5.53E-08	0.16	104	8.62	3662	0.22	104	11.7	2232	0.17	100	8.7	4345
BTG3	1.31	0.000347337	0.12	120	8.62	3663	0.16	101	7.95	3773	0.1	145	9.55	4042
RNF17_t1	1.01	0.358383886	0.12	122	8.62	3664	NA	NA	NA	NA	NA	NA	NA	NA
ZNF160_t1	-1.01	0.619144574	0.09	145	8.61	3665	NA	NA	NA	NA	NA	NA	NA	NA
CEP19	1.15	3.42E-07	0.12	120	8.61	3666	0.13	123	9.66	2999	0.19	134	15.59	2183
SLC6A9	1.02	0.230951889	0.09	143	8.61	3667	-0.04	112	-2.55	10183	-0.05	135	-3.9	10589
TXNL4B_t1	1.11	0.00100057	0.12	120	8.6	3668	NA	NA	NA	NA	0.1	132	7.89	4669
RALGAPA1_t2	1.06	0.001137841	0.09	141	8.6	3669	0.03	129	2.73	6658	0.1	138	8.54	4407
MR1	1.11	0.007858771	0.09	142	8.6	3670	0	142	-0.43	8765	0	165	0.43	8353
IKBKE	1.07	0.006286217	0.11	127	8.6	3671	0.14	122	10.21	2792	-0.03	120	-1.9	9668
CLPSL2	1.08	1.06E-06	0.17	100	8.6	3672	0.06	107	3.48	6197	0.1	94	4.31	6280
SRP14	1.37	1.57E-07	0.13	118	8.59	3673	0.22	137	18.88	537	0.23	135	19.47	1356
KIAA0232_t1	1.19	3.64E-07	0.09	145	8.59	3674	NA	NA	NA	NA	0.27	139	23.85	741
MSTN	1.02	0.064594707	0.1	139	8.59	3675	0.08	128	6.41	4563	0.01	130	0.9	8078
CERS3	1.02	0.09164305	0.1	134	8.59	3676	0.02	141	1.54	7468	0.05	151	4.91	5971
GOLM1_t1	1.49	1.42E-06	0.11	129	8.59	3677	NA	NA	NA	NA	0.17	126	13.07	2863
ELAVL4	1.06	4.36E-05	0.12	123	8.59	3678	0.06	104	3.42	6234	0.13	116	8.48	4432
ORC5_t1	1.11	0.001133926	0.1	137	8.59	3679	0.1	117	6.45	4543	0.24	120	16.62	1922
FNTA	1.16	0.002402976	0.11	131	8.58	3680	0.2	131	16.44	952	0.12	100	6.12	5392
ANKRD6	1.1	1.33E-05	0.08	151	8.58	3681	0.01	126	1	7812	0.1	122	7.17	4962
PABPN1	1.11	3.48E-05	0.13	116	8.58	3682	0.21	126	16	1049	0.05	123	3.48	6679
CMTM4_t1	1.14	0.010478583	0.09	145	8.58	3683	0.01	143	1.18	7687	0.1	136	8.53	4412
MAGEA9	1.16	0.00151124	0.1	140	8.58	3684	0.01	107	0.68	8017	0.16	130	12.43	3057
CLNS1A	1.09	0.029339049	0.11	126	8.58	3685	0.2	104	10.71	2585	0.02	109	1.2	7915
SDCCAG8_t1	1.07	0.019746054	0.16	105	8.58	3686	NA	NA	NA	NA	NA	NA	NA	NA
DGKH	1.04	0.000420611	0.13	116	8.58	3687	0.17	125	12.49	1980	0.15	131	12.09	3165
CSNK1D	1.17	2.98E-07	0.1	132	8.57	3688	0.12	131	9.49	3065	0.04	160	4.27	6309
PCYT1A	1.19	4.30E-09	0.19	95	8.57	3689	0.12	102	6.04	4754	0.21	110	12.54	3030
SMARCA5	-1.01	0.756533751	0.1	135	8.57	3690	0.16	131	13.24	1759	0.1	139	9.22	4153
NT5DC2	1.37	4.75E-07	0.18	98	8.57	3691	0.14	105	7.48	3999	0.13	90	5	5921
AP1AR	1.09	0.0002375	0.11	131	8.57	3692	0.04	140	3.59	6118	0.19	147	18.03	1643
GDE1	-1.07	0.075634065	0.08	160	8.56	3693	-0.03	125	-2.38	10068	0.01	137	0.65	8224
ZNF626_t2	1.1	0.034940338	0.1	137	8.56	3694	0.16	143	15.33	1186	0.11	130	8.61	4379
EIF3E_t1	1.33	7.69E-05	0.21	90	8.56	3695	NA	NA	NA	NA	NA	NA	NA	NA
UBE2I	1.04	0.237397006	0.11	127	8.56	3696	0.2	117	13.11	1801	-0.05	138	-4.4	10797
AARS2	1.24	9.75E-11	0.11	129	8.56	3697	0.07	135	6.07	4743	0.19	129	15.12	2300
PRDM7	1.12	2.47E-11	0.13	114	8.56	3698	0	142	-0.46	8790	0.19	126	14.69	2410
CENPO	1.01	0.862770833	0.11	127	8.56	3699	-0.03	146	-2.88	10398	0.04	156	4.46	6206
OR4N4_t1	1.12	2.50E-07	0.16	105	8.56	3700	0.08	100	3.88	5960	NA	NA	NA	NA
RRP36	1.22	6.82E-12	0.16	104	8.56	3701	0.14	112	8.87	3345	0.2	108	11.66	3305
MAGEB10	1.06	0.003591006	0.13	118	8.55	3702	-0.03	122	-2.11	9908	0.16	126	12.35	3087
NME7	1.16	0.000320695	0.14	109	8.55	3703	0.15	116	9.65	3003	0.19	146	17.84	1677
PLD6	1.09	0.056142456	0.1	132	8.55	3704	-0.05	127	-3.79	10975	0.1	127	7.49	4833
CNTD2	1.17	2.13E-09	0.12	120	8.54	3705	0.05	121	3.65	6090	0.13	120	9.23	4145
FGFR4	1.08	0.040533954	0.15	107	8.54	3706	0.08	119	5.49	5063	0.13	113	8.09	4589
WLS	-1.07	0.374576334	0.09	141	8.54	3707	-0.1	126	-7.47	12858	-0.02	111	-0.95	9171

ZNF580	1.32	1.16E-09	0.15	106	8.54	3708	0.17	141	15.11	1246	0.19	120	13.06	2866
HNRNPA1L2	1.19	0.00076464	0.14	110	8.54	3709	0.17	109	10.3	2758	0.09	133	7.81	4707
LMAN1	1.15	0.018608097	0.08	154	8.54	3710	0.09	137	7.95	3774	0.04	131	3.56	6635
LRRC15	1.02	0.023453458	0.11	131	8.53	3711	0.07	130	5.94	4819	0.07	131	5.76	5554
RAB42	1	0.943060666	0.1	136	8.53	3712	0.17	131	13.46	1696	-0.1	120	-7.09	11877.5
SYT11_t1	1.01	0.234516872	0.1	135	8.53	3713	NA	NA	NA	NA	NA	NA	NA	NA
ZBTB12	1.04	0.000659888	0.15	107	8.52	3714	0.08	111	4.91	5390	0.14	117	9.09	4189
HIGD1B	2	1.31E-22	0.25	84	8.52	3715	0.26	139	23.49	130	0.33	77	8.97	4237
DDX18	1.1	0.013912305	0.11	131	8.52	3716	0.07	124	4.88	5408	0.16	147	15.13	2298
OTUB1_t1	1.1	5.06E-05	0.14	110	8.52	3717	NA	NA	NA	NA	0.03	123	1.83	7547
PRND_t1	1.27	6.05E-06	0.11	126	8.51	3718	0.16	136	13.85	1579	NA	NA	NA	NA
SDAD1_t1	1.09	0.000225092	0.11	125	8.51	3719	NA	NA	NA	NA	0.14	123	10.06	3852
GTDC1	-1.08	0.168350169	0.11	126	8.5	3720	0.12	124	9.24	3170	0	142	0.15	8519
NUPL2	1.31	1.63E-12	0.16	104	8.5	3721	0.12	99	5.85	4870	0.32	124	23.71	763
TADA2A_t2	1.09	2.31E-06	0.2	92	8.5	3722	0.13	103	6.75	4378	0.19	87	6.89	5070
MTMR6	1.01	0.460647241	0.09	143	8.5	3723	0.01	118	0.84	7908	0.09	149	8.52	4417
FCHO2_t1	-1.01	0.430015439	0.08	151	8.5	3724	0.08	125	5.85	4874	NA	NA	NA	NA
MBLAC1	1.36	2.95E-16	0.2	92	8.5	3725	0.07	113	4.55	5585	0.25	120	17.42	1749
CHRM3	1.34	2.52E-08	0.19	95	8.5	3726	0.01	97	0.69	8005	0.27	97	12.85	2939
FAM122A	-1.01	0.730065958	0.1	139	8.49	3727	-0.06	125	-4.62	11454	0.04	136	3.75	6547
SS18	1.1	3.73E-05	0.1	139	8.49	3728	0.11	135	9.35	3124	0.11	134	9.56	4038
CSNK1E_t1	1.15	2.85E-05	0.13	116	8.49	3729	NA	NA	NA	NA	0.08	132	6.78	5116
BTF3	1.07	0.093795733	0.15	106	8.49	3730	0.03	122	2.18	7022	0.15	112	9.29	4121
INO80D	1.03	0.000726041	0.09	148	8.49	3731	0.11	130	8.72	3405	0.11	146	10.45	3707
DDX54_t1	1.14	3.17E-05	0.11	128	8.49	3732	NA	NA	NA	NA	0.13	120	9.04	4216
SRGAP1	1.07	0.073310989	0.08	154	8.48	3733	0.1	130	7.78	3840	0.06	143	5.24	5811
AFF3	1.04	0.047218123	0.09	148	8.48	3734	0.05	139	4.68	5508	0.12	138	10.24	3782
MMGT1	1.17	0.000425451	0.1	135	8.48	3735	0.12	140	11.05	2452	0.24	153	24.67	655
SNX31	1.07	0.000588915	0.1	136	8.48	3736	0.1	131	8.31	3590	0.16	160	17.45	1745
TM9SF1	1.13	0.000115194	0.11	128	8.48	3737	-0.17	121	-12.06	14527	0.11	120	7.62	4785
ZNF33B	-1.02	0.556154344	0.1	137	8.47	3738	0	149	0.08	8413	0.05	158	5.86	5516
LYST	1.07	0.267931234	0.1	132	8.47	3739	0.1	138	8.48	3510	0.15	123	10.89	3554
TSG101	1.29	2.44E-13	0.19	95	8.47	3740	0.1	104	5.51	5058	0.26	141	23.88	736
AHRR	1.2	4.13E-05	0.12	118	8.47	3741	0.05	139	4.88	5405	0.18	140	16.29	2009
THOC3	1.25	1.16E-12	0.14	111	8.47	3742	0.12	98	5.98	4796	0.19	107	10.59	3658
MBNL3	1.21	0.015432428	0.09	145	8.47	3743	0.06	140	5.24	5197	0.24	149	24.24	695
ZMYND8	1.14	0.000186079	0.12	120	8.47	3744	0.05	133	3.92	5938	0.11	142	10.21	3795
ATRAID	1.09	0.002124043	0.18	98	8.46	3745	0.07	130	5.89	4851	0.03	152	3.3	6773
CALCRL	1.08	0.02840222	0.08	150	8.46	3746	0.1	135	8.89	3334	0.1	131	7.88	4671
FBXW11	1.05	0.093187027	0.11	127	8.46	3747	0.03	137	2.44	6868	0.09	148	8.61	4380
ANGPT1	1.28	9.13E-06	0.09	144	8.46	3748	0.18	128	13.71	1621	0.13	136	11.1	3477
PCDHB11	1.24	0.002003706	0.14	111	8.46	3749	0.06	136	5.02	5322	0.07	124	5.51	5679
SAPCD1	1.62	1.21E-14	0.15	107	8.46	3750	0.23	129	18.42	597	0.22	88	8.17	4548
ZMYND11_t2	1.12	6.59E-05	0.12	118	8.46	3751	0.19	122	14.02	1525	0.21	120	14.63	2426
GPN3	1.2	1.72E-05	0.14	109	8.46	3752	0.05	115	3.38	6261	0.23	119	16.03	2066
COMMD9	1.16	4.77E-07	0.09	145	8.45	3753	0.01	127	1.14	7710	0.05	132	4.36	6264
ZNF221	1.04	0.002861843	0.15	108	8.45	3754	0.18	111	10.77	2565	0.12	112	7.21	4943
TRMT5	1.13	0.000750237	0.11	127	8.45	3755	0.03	132	2.83	6599	0.12	137	10.69	3618
MAP4_t1	1.08	0.034312837	0.12	122	8.45	3756	0.2	134	16.61	918	NA	NA	NA	NA
SOHLH1	1.21	0.001145678	0.13	117	8.45	3757	0.13	107	7.41	4033	0.16	113	10.05	3858
KDM5C	1.13	8.93E-08	0.13	113	8.44	3758	0.14	117	9.25	3165	0.16	128	12.54	3033
GNB5	1.25	0.000211806	0.1	132	8.44	3759	-0.04	121	-2.49	10144	0.16	142	14.61	2429
TRIM28	1.23	1.14E-10	0.15	105	8.44	3760	-0.01	124	-0.63	8891	0.1	93	4.15	6352
RFC2	1.23	7.55E-07	0.13	114	8.44	3761	0.04	147	3.7	6060	0.21	152	21.81	1003
CHRNB3	1.1	7.26E-08	0.11	125	8.43	3762	0.03	110	1.62	7410	0.1	120	7.33	4906
MRPS21	1.55	2.99E-23	0.26	82	8.43	3763	0.29	122	20.61	330	0.33	124	24.25	693
ZNF434	1.18	1.18E-15	0.12	123	8.43	3764	0.19	111	11.51	2305	0.19	147	18.04	1640
DSTN	1.1	0.052188259	0.13	116	8.43	3765	0.09	124	6.32	4610	0.24	114	15.38	2224
SLC26A1_t1	1.35	6.54E-19	0.21	91	8.42	3766	0.14	119	9.35	3122	0.31	117	20.51	1197
CKMT1A	1.18	2.46E-05	0.12	122	8.42	3767	0.05	121	3.24	6348	0.15	130	12.03	3184
SMARCC1_t1	1.1	9.97E-05	0.12	120	8.41	3768	NA	NA	NA	NA	NA	NA	NA	NA
SLC7A1_t1	1.07	0.308755808	0.08	151	8.41	3769	0.09	163	9.77	2943	NA	NA	NA	NA
NAIF1	1.21	3.88E-08	0.09	140	8.4	3770	0.21	142	18.87	540	0.14	147	14.06	2579
NHLRC3_t1	1.02	0.601548477	0.09	140	8.4	3771	0.08	125	5.78	4907	-0.09	135	-7.55	12050
RPS14_t1	1.58	2.75E-17	0.2	91	8.4	3772	0.27	92	11.4	2333	0.26	114	16.64	1919
DOT1L	1.18	9.60E-06	0.12	122	8.4	3773	0.05	146	4.42	5652	0.12	128	9.35	4101
FAM73A	-1	0.929524988	0.09	145	8.4	3774	-0.01	135	-1.12	9233	0.1	156	10.1	3837
TMPRSS15	1.09	2.04E-05	0.12	121	8.4	3775	0.18	123	13.24	1758	0.14	121	10.24	3785
TXNDC16	1.08	0.04902368	0.09	147	8.4	3776	0.04	114	2.24	6990	0.13	121	9.33	4111
NOP10	1.18	3.18E-07	0.14	108	8.4	3777	0.13	119	8.89	3333	0.18	91	7.29	4918
NTM	1.13	0.000456125	0.12	123	8.4	3778	0.17	120	11.78	2200	0.07	127	5.25	5803
ESCO1	1.04	0.454973527	0.09	145	8.4	3779	0.06	116	4.13	5821	0.09	127	7.25	4926
PDIK1L	1.11	0.031376249	0.08	161	8.4	3780	0.04	132	3.13	6420	0.2	147	19.59	1332
PRPF40B	1.3	2.05E-17	0.18	97	8.39	3781	0.17	118	11.9	2153	0.23	131	18.78	1509
WBSCR22	1.28	1.37E-12	0.14	112	8.39	3782	0.16	112	9.62	3013	0.17	120	12.08	3168
UNG	1.09	0.000700712	0.13	113	8.39	3783	0.08	106	4.52	5603	0.09	119	5.92	5483
ARFGAP1	1.18	1.35E-07	0.12	121	8.39	3784	0.12	148	11.75	2213	0.07	141	6.27	5328
BECN1_t1	1.09	0.006010627	0.11	126	8.39	3785	0.09	116	5.91	4839	NA	NA	NA	NA
RBM26	-1.01	0.82634909	0.11	125	8.38	3786	0.18	123	13.49	1686	0.01	134	1.09	7973
CSN2	1.08	1.19E-05	0.14	112	8.38	3787	-0.03	112	-1.62	9567	0.15	125	11.56	3336
KIAA0895	1.07	0.007503704	0.1	131	8.38	3788	0.1	135	8.19	3643	0.17	127	12.89	2930
CYP2R1	1.28	5.24E-08	0.14	109	8.38	3789	0.08	94	3.38	6260	0.2	95	9.09	4190
FAM222A	1.71	2.94E-18	0.15	107	8.37	3790	0.13	120	8.88	3337	0.25	130	20.37	1219
ZDHHC21	1.01	0.616894231	0.09	145	8.37	3791	0.14	142	12.85	1880	0.09	141	8.17	4547
DGUOK	1.15	3.94E-09	0.13	115	8.37	3792	0.23	122	16.66	903	0.1	115	6.19	5367
PPAPDC1B	1.04	0.31856655	0.12	122	8.37	3793	0.05	135	4.37	5677	-0.08	98	-4.05	10657
RDH10	1.1	0.015893422	0.1	138	8.37	3794	-0.01	122	-0.91	9086	0.07	127	5.36	5754
HK2_t1	1.11	0.009019156	0.09	140	8.37	3795	NA	NA	NA	NA	0.02	126	1.73	7616
TAOK3	1.11	0.000955662	0.12	119	8.37	3796	0.15	130	11.94	2142	0.23	142	20.86	1145
DACH2	1.09	0.001041379	0.11	129	8.36	3797	0.03	112	1.95	7178	0.26	135	22.24	931
SPDYE2_t1	1.12	1.08E-09	0.11	124	8.36	3798	NA	NA	NA	NA	0.13	105	7.13	4982
DPYSL3	1.01	0.814174751	0.1	135	8.36	3799	0.11	134	9.21	3184	0	126	0.32	8429
PPP1CC_t1	1.23	1.13E-05	0.11	129	8.36	3800	NA	NA	NA	NA	0.28	133	22.83	861
CCDC155	1.13	1.03E-06	0.11	123	8.36	3801	-0.05	121	-3.66	10915	0.11	108	6.36	5295
BRPF1	1.12	3.67E-07	0.14	110	8.36	3802	0.12	140	11	2478	0.11	120	7.8	4711
ARMCX5	1.19	4.28E-06	0.15	107	8.36	3803	0.04	111	2.59	6766	0.31	130	24.79	638
ZNF85	1.11	0.060227805	0.13	113	8.36	3804	0.18	139	16.11	1021	0.11	105	6.27	5326
LRRC41	1.03	0.236532853	0.14	109	8.35	3805	0.04	117	2.48	6835	-0.02	159	-2.65	10019
HCRTR2	1.03	0.004360491	0.1	130	8.35	3806	-0.01	88	-0.29	8662	0.11	134	9.36	4093
SLC25A46	1.03	0.412315226	0.1	134	8.35	3807	-0.01	128	-0.58	8864	0.22	150	22.01	967
NCOA5	1.06	5.28E-06	0.09	141	8.35	3808	0.09	119	6.07	4739	0.13	115	8.55	4406
XRCC6	1.2	3.54E-07	0.12	118	8.34	3809	0.26	104	13.84	1582	0.12	136	10.7	3613
CD177_t1	1.04	0.014991998	0.09	140	8.34	3810	NA	NA	NA	NA	0.05	124	3.58	6627
TBCC_t1	1.2	1.11E-08	0.08	150	8.34	3811	0.1	133	8.35	3569	NA	NA	NA	NA
ZNRD1	1.3	4.96E-13	0.12	119	8.34	3812	0.21	123	15.51	1143	0.18	113	11.47	3362
HARS	1.17	8.86E-07	0.1	137	8.34	3813	0.07	109	4.18	5792	0.15	147	14.82	2379

TSPAN33_t1	1.16	0.000351822	0.12	118	8.34	3814	-0.05	135	-4.24	11238	NA	NA	NA	NA
TTC5	1.12	1.31E-05	0.17	100	8.33	3815	0.07	138	5.75	4923	0.1	129	7.93	4649
MEIS1	1.09	0.003597334	0.11	126	8.33	3816	0.13	133	10.41	2713	0.15	132	12.43	3058
ZNF708	1.07	0.000572672	0.08	152	8.33	3817	0.29	139	25.94	64	0.05	147	4.48	6195
ACTG2	2.13	1.58E-10	0.19	93	8.31	3818	0.16	100	7.89	3794	0.22	64	3.05	6902
UTP20	1.21	3.27E-10	0.15	106	8.31	3819	0.25	111	15.21	1225	0.3	125	22.47	904
HMGA2_t1	1.06	0.000174346	0.08	151	8.31	3820	0.11	132	9.12	3220	NA	NA	NA	NA
ASCC3_t2	-1.03	0.531213211	0.09	141	8.3	3821	NA	NA	NA	NA	0.08	136	7.16	4965
CCNT2	1.03	0.110079295	0.09	147	8.3	3822	0.11	135	9.54	3041	0.13	141	11.46	3367
FBXW9	1.16	2.31E-07	0.12	119	8.3	3823	0.06	117	4.27	5730	0.12	87	4.39	6252
NAA35_t1	1.09	0.004765405	0.12	118	8.29	3824	NA	NA	NA	NA	0.15	109	8.82	4294
SLC4A1_t1	1	0.798203288	0.12	122	8.29	3825	0.02	135	2.08	7092	NA	NA	NA	NA
CA8	1.14	0.000117642	0.09	142	8.29	3826	0.08	125	6.2	4666	0.12	129	9.22	4156
GAS2L1_t1	1.08	0.062090339	0.13	114	8.29	3827	0.11	146	10.65	2616	0.16	148	15.88	2104
RABGGTB_t1	1.15	0.003434778	0.09	147	8.29	3828	NA	NA	NA	NA	0.17	119	11.78	3270
FTSJ3	1.42	1.45E-19	0.16	102	8.29	3829	0.19	133	15.78	1095	0.23	107	13.39	2763
ACTA1	1.17	9.18E-07	0.11	125	8.29	3830	0.15	145	14.09	1510	0.16	102	8.32	4486
ARHGAP5	1.04	0.263321571	0.08	154	8.29	3831	0	143	-0.33	8701	0.14	139	12.29	3112
LRRC8D	1.28	3.81E-08	0.13	114	8.29	3832	0.08	122	5.6	5003	0.25	113	15.47	2202
MAP3K13	1.09	1.18E-06	0.08	148	8.28	3833	-0.03	135	-2.66	10254	0.19	155	20.18	1253
FAXC	1.1	0.000110368	0.08	155	8.28	3834	0.09	127	6.59	4454	0.07	149	6.7	5156
POLB	1.12	0.014174265	0.13	114	8.28	3835	0.02	98	0.84	7909	0.12	121	8.31	4492
POLE3	1.17	1.44E-07	0.11	122	8.28	3836	0.15	132	12.24	2049	0.11	136	9.57	4033
PDYN	1.04	0.017460728	0.09	138	8.27	3837	0.16	124	12.18	2075	0.14	119	9.76	3959
HYOU1	1.18	5.86E-09	0.1	134	8.27	3838	0.01	120	1.03	7788	0.12	133	9.7	3985
AP3D1	1.25	4.50E-09	0.11	127	8.27	3839	0.21	131	16.92	857	0.11	115	7.42	4859
TMEM127	1.07	0.001841402	0.09	143	8.27	3840	0.03	147	2.8	6612	0.05	128	4.28	6299
SEMA3D	1	0.696826181	0.09	139	8.27	3841	0.07	132	5.98	4794	0.14	139	12.55	3025
DCTN2	1.24	1.06E-13	0.15	104	8.26	3842	0.15	121	10.79	2558	0.14	135	12.24	3125
SPAG6_t1	1.04	0.007777366	0.1	132	8.26	3843	0.04	138	3.52	6174	0.08	130	6.1	5403
FAM110D	1.1	3.84E-07	0.11	128	8.26	3844	0.04	108	2.06	7106	0.03	99	1.42	7793
RFXANK	1.32	1.26E-19	0.22	88	8.26	3845	0.26	120	18.34	609	0.14	80	4.24	6323
ZCCHC11	1.1	0.062197042	0.1	136	8.26	3846	0.09	125	6.6	4452	0.11	130	9.14	4174
CTU1	1.04	0.024116538	0.12	120	8.26	3847	0.02	128	1.87	7228	0.03	120	1.78	7581
BIRC7	1.04	0.001122713	0.13	113	8.25	3848	0.09	102	4.74	5478	0.04	100	1.94	7485
USP49	1.12	4.72E-11	0.09	144	8.25	3849	0.09	130	7.29	4094	0.21	128	16.21	2032
GAR1	1.11	0.001809411	0.11	124	8.25	3850	0.16	114	10.13	2818	0.12	127	9.34	4105
LPCAT1_t1	1.01	0.745549815	0.11	124	8.24	3851	NA	NA	NA	NA	0.05	120	3.17	6851
KRTAP9-3	1.03	0.006248	0.09	143	8.24	3852	-0.09	115	-6.13	12258	0.08	116	5.54	5664
SCPEP1	1.24	0.002666573	0.13	115	8.24	3853	0.16	113	9.83	2920	-0.01	109	-0.52	8934
ALX3	1.1	1.15E-05	0.1	131	8.24	3854	-0.01	116	-0.78	9003	0.06	111	3.79	6533
STOX1	1.27	5.22E-09	0.11	122	8.23	3855	0.12	114	7.77	3844	0.12	115	8.04	4602
RAD51C	1.53	5.84E-19	0.21	90	8.23	3856	0.19	90	7.56	3958	0.34	128	26.2	483
WFDC11	1.06	1.11E-07	0.12	121	8.23	3857	0.04	107	2.01	7136	0.11	92	4.5	6177
SLCO6A1	1.26	2.94E-06	0.14	109	8.23	3858	0.11	92	4.57	5576	0.21	110	12.41	3065
IRAK1BP1	1.13	4.74E-05	0.11	127	8.22	3859	0.16	127	12.02	2124	0.05	137	4.25	6317
BPIFA3	1.05	1.49E-08	0.19	93	8.22	3860	0.07	97	3.12	6425	0.17	103	9.07	4201
RPL27	1.38	1.74E-11	0.24	84	8.22	3861	0.24	76	6.14	4707	0.29	79	8.47	4437
DCT	1.03	0.000913947	0.1	130	8.22	3862	-0.03	106	-1.94	9770	0.07	97	3.33	6757
NUPL1	1.08	0.060997409	0.1	133	8.22	3863	0.08	139	7.4	4038	0.14	138	12.42	3061
PHPT1_t1	1.37	1.40E-14	0.22	88	8.22	3864	NA	NA	NA	NA	0.17	120	11.71	3296
CLIC1	1.14	0.007027995	0.11	125	8.22	3865	0.19	163	21.02	292	-0.07	108	-4.09	10677
FEV	1.01	0.639241715	0.11	125	8.21	3866	-0.09	148	-9.16	13575	0.02	121	1.42	7795
ADCK1	1.11	1.07E-05	0.14	108	8.21	3867	0.04	136	3.51	6178	0.09	123	6.84	5091
NUTF2	1.1	0.000233802	0.14	109	8.21	3868	0.08	137	6.54	4487	0.04	121	2.86	7009
IVL	1.04	0.0083782	0.09	140	8.21	3869	0.06	103	3.21	6367	0.11	96	5.17	5843
PKNOX1_t1	1.08	1.10E-11	0.11	126	8.21	3870	NA	NA	NA	NA	0.16	139	13.97	2602
ORC3	1.17	0.000735853	0.11	125	8.2	3871	0.11	115	7.09	4203	0.1	110	6.12	5390
FSCB	1.01	0.610465558	0.1	130	8.2	3872	0	97	0.23	8301	0.01	110	0.59	8260
FADS1	1.8	6.45E-06	0.11	126	8.2	3873	0.14	139	12.77	1909	0.2	134	16.89	1857
DNAJC24	1.12	0.000124982	0.1	129	8.2	3874	0.04	140	3.31	6308	0.23	150	22.53	893
SLC6A17	1.03	0.00012281	0.1	135	8.2	3875	-0.03	159	-3.43	10753	0.01	122	0.71	8186
LSG1_t1	1.1	1.50E-05	0.12	120	8.19	3876	NA	NA	NA	NA	NA	NA	NA	NA
GSK3B	1.14	2.27E-05	0.08	156	8.19	3877	-0.03	133	-2.31	10033	0.13	142	11.9	3235
NKX2-1_t1	1.04	0.011914162	0.11	122	8.19	3878	0.01	146	0.69	8010	NA	NA	NA	NA
OLFM3	1.03	0.027460392	0.11	122	8.18	3879	0.17	120	12.08	2107	0.03	130	2.09	7404
PRPSAP1	1.25	1.03E-05	0.14	109	8.18	3880	0.08	114	4.87	5413	0.21	111	12.53	3038
PPP1R15B	1.09	0.000585033	0.1	134	8.18	3881	0.1	138	8.72	3400	0.15	152	15.17	2283
WDR1	1.1	0.010072213	0.1	129	8.18	3882	0.19	124	14.23	1468	0.02	142	2.26	7314
DOC2A	1.05	0.000192696	0.18	96	8.17	3883	0.03	128	2.58	6774	0.12	123	8.74	4324
BBS4	1.24	9.84E-07	0.12	118	8.17	3884	0.08	116	5.27	5179	0.25	128	19.6	1331
DYNC2LI1_t1	1.18	9.33E-07	0.15	104	8.17	3885	NA	NA	NA	NA	0.24	111	14.43	2481
HPCA	1.02	0.387476525	0.12	120	8.17	3886	0.01	120	0.49	8145	-0.02	127	-1.48	9437
RPL41	1.16	6.73E-05	0.1	129	8.17	3887	0.05	122	3.81	6000	0.18	124	13.37	2774
ERCC3	1.12	1.70E-06	0.13	114	8.16	3888	0.13	110	7.78	3842	0.08	116	5.16	5847
IMMT_t1	1.08	0.004851154	0.15	103	8.16	3889	NA	NA	NA	NA	0.23	135	19.57	1335
TMOD2	1.04	0.003076273	0.08	147	8.16	3890	0.07	140	6.03	4764	0.12	165	13.71	2675
OR2A20P	1.06	7.44E-05	0.13	114	8.16	3891	-0.01	123	-0.52	8832	0.08	143	7.13	4985
SNAPC2	1.27	7.62E-13	0.13	111	8.16	3892	0.14	110	8.29	3597	0.12	115	7.95	4640
PRMT5_t1	1.16	2.08E-07	0.14	107	8.16	3893	NA	NA	NA	NA	NA	NA	NA	NA
ACSS2	1.27	0.000130971	0.14	110	8.16	3894	0.05	126	4.09	5847	0.09	120	6.63	5184
COMMD7	1.14	2.01E-07	0.13	114	8.15	3895	0.09	134	7.76	3854	0.08	125	6.19	5365
NDUFA8	1.2	2.75E-05	0.13	111	8.15	3896	0.1	131	8.18	3653	0.17	114	10.64	3641
WBSCR27	1.31	1.82E-11	0.18	95	8.15	3897	-0.01	100	-0.4	8746	0.2	94	8.85	4280
CABLES2	1.2	7.32E-08	0.11	121	8.15	3898	0.1	136	9	3271	0.15	130	12.26	3122
COL8A1	1.41	1.17E-05	0.1	132	8.15	3899	0.1	138	9.06	3247	0.06	105	3.34	6748
NARF	1.53	2.44E-21	0.21	88	8.14	3900	0.22	109	12.76	1912	0.27	88	10.22	3791
HBS1L	1.12	6.19E-06	0.1	129	8.14	3901	0.1	132	8.49	3509	0.16	144	15.39	2220
MYO18B	1.27	7.14E-07	0.13	114	8.14	3902	0.06	118	4.23	5759	0.13	100	6.55	5213
KBTBD4_t1	1.08	8.08E-05	0.09	141	8.14	3903	NA	NA	NA	NA	0.11	129	8.7	4343
PHC2	-1	0.612651047	0.09	140	8.14	3904	0.05	140	4.86	5418	-0.03	113	-1.97	9701
MFI2_t1	1.12	0.000308528	0.14	107	8.14	3905	0.13	104	6.96	4263	0.03	121	1.82	7557
CLTC	1.25	0.001269767	0.09	144	8.13	3906	0.19	154	20.15	388	0.19	164	21.42	1060
CHRNA1	1.06	1.61E-07	0.11	122	8.13	3907	-0.01	124	-0.73	8953	0.07	135	5.65	5604
GNAI1	1.37	6.44E-09	0.1	135	8.12	3908	0.13	132	10.44	2706	0.28	140	25.57	551
AKAP14	1.1	3.67E-07	0.16	101	8.12	3909	0.13	99	6.59	4455	0.24	96	10.94	3529
ZNF484	1.02	0.353924364	0.07	171	8.12	3910	0.04	124	3.12	6427	0.05	123	3.81	6523
ZNF75A	1.08	0.001013845	0.11	122	8.12	3911	0.12	116	7.69	3884	0.17	127	12.82	2947
ALG10B	1.08	4.80E-05	0.09	142	8.12	3912	0.06	128	4.41	5656	0.04	148	4.41	6244
SLC7A10	1.26	8.97E-06	0.15	103	8.12	3913	0.11	100	5.59	5017	0.15	116	10.04	3862
OGG1_t1	1.48	3.52E-12	0.16	100	8.11	3914	0.12	134	10.27	2770	0.16	121	11.08	3486
RASA1	1.07	0.010584647	0.1	134	8.11	3915	0.14	147	13.19	1775	0.2	143	19.01	1451
CT45A5	1.23	0.004023247	0.1	130	8.11	3916	0.02	131	1.93	7190	0.19	120	13.23	2819
TPRKB	1.37	1.45E-16	0.18	94	8.11	3917	0.21	93	8.9	3329	0.32	105	17.81	1682
METAP1D	1.17	1.93E-05	0.16	102	8.11	3918	0.17	107	9.75	2953	0.2	136	16.97	1836
NFE2L3_t1	1.17	0.052201528	0.09	142	8.11	3919	0.14	124	10.27	2772	NA	NA	NA	NA

DHDDS_t2	1.07	0.002844548	0.11	124	8.11	3920	-0.01	136	-0.63	8894	0.07	123	4.82	6019
EIF5A2_t1	1.37	3.75E-05	0.1	134	8.1	3921	NA	NA	NA	NA	0.17	159	18.63	1532
CAPN10	1.28	3.55E-12	0.19	92	8.1	3922	0.27	100	13.65	1638	0.13	148	12.81	2948
DUSP3	1.04	0.16939222	0.09	137	8.1	3923	0.02	144	1.72	7337	0.03	132	2.57	7150
CCL26	1.19	9.92E-05	0.11	126	8.1	3924	0.17	88	6.5	4514	0.06	91	2.41	7228
TBC1D19	1.13	0.000194107	0.16	100	8.1	3925	0.13	109	7.38	4048	0.24	120	16.53	1947
DOLK	1.32	7.02E-14	0.11	124	8.09	3926	0.11	108	6.33	4599	0.18	96	8.16	4553
MTMR3	1.08	0.035397894	0.08	149	8.09	3927	0.13	138	11.7	2230	0.02	127	1.38	7815
TMEM245	1.16	0.061704706	0.08	149	8.09	3928	0.01	136	0.45	8168	0.16	134	13.78	2658
SIRPA	1.06	2.16E-05	0.11	121	8.09	3929	0.03	120	2.35	6918	0.06	126	4.74	6058
CLRN1_t1	1.01	0.277872297	0.09	142	8.09	3930	0.06	118	4.12	5832	0.08	122	6.11	5394
RPS6KA3	1.21	0.000717685	0.1	129	8.09	3931	0.2	147	19.34	472	0.16	152	15.93	2090
EXOC6_t1	1.04	0.527947208	0.1	133	8.09	3932	NA	NA	NA	NA	NA	NA	NA	NA
EIF2AK1	1.3	6.93E-21	0.09	141	8.08	3933	0.11	139	10.08	2835	0.31	142	28.43	341
THSD1	1.4	2.88E-05	0.12	116	8.08	3934	0.17	95	7.5	3984	0.06	117	3.97	6428
RALGPS2	1.1	0.004126228	0.1	130	8.08	3935	0.11	131	8.97	3289	0.1	165	11.15	3453
NAA25	1.05	4.32E-05	0.1	135	8.08	3936	0.17	112	10.67	2610	0.15	143	13.92	2610
MSANTD3_t1	1.14	0.002390864	0.14	106	8.08	3937	NA	NA	NA	NA	NA	NA	NA	NA
CCDC111	1.08	0.014570164	0.12	119	8.07	3938	0.01	118	0.74	7978	0.12	111	7.5	4828
CALU	1.19	2.85E-05	0.12	120	8.07	3939	0.16	130	12.53	1972	0.19	128	14.87	2369
CALML3	1.01	0.728997357	0.14	107	8.07	3940	0	135	-0.39	8744	-0.04	127	-3.39	10352
OGFOD1	1.11	0.000362739	0.12	117	8.06	3941	0.11	127	8.26	3617	0.13	130	10.44	3710
TMC5	1.6	5.36E-06	0.09	136	8.05	3942	0.14	141	13.15	1787	0.07	129	5.74	5563
SLC2A5	2.2	1.12E-14	0.11	123	8.05	3943	0.18	108	10.19	2795	0.1	95	4.51	6173
ZNF77_t1	1.15	1.97E-10	0.17	97	8.05	3944	NA	NA	NA	NA	0.25	121	17.54	1727
ACTR6	1.27	1.35E-06	0.14	107	8.04	3945	0.15	120	10.83	2543	0.21	116	14.01	2594
UBA6	1.02	0.185666901	0.09	136	8.04	3946	0.1	152	10.4	2715	0.06	125	4.49	6191
FAM13B	-1	0.951042906	0.09	141	8.04	3947	0.04	127	3.35	6283	0.1	131	8.43	4454
ZNF35	1.13	7.83E-07	0.09	144	8.03	3948	0.1	118	6.99	4252	0.17	115	10.77	3581
WDR47	1.05	0.025562082	0.11	126	8.03	3949	0.13	132	11.01	2472	0.13	164	15.2	2274
IDI1	1.22	0.017489519	0.11	120	8.03	3950	0.08	136	6.53	4495	0.18	123	13.13	2848
HIST1H3B	1.65	2.72E-19	0.27	80	8.03	3951	0.18	88	6.92	4288	0.3	80	8.96	4241
RBBP8	1.04	0.492720609	0.13	114	8.03	3952	-0.01	126	-0.51	8820	0.17	158	18.01	1649
TES	1.2	0.000232593	0.11	125	8.03	3953	0.21	134	17.72	712	0.2	152	20.89	1135
ZNF608	1.06	0.146678704	0.07	160	8.02	3954	0.07	119	4.58	5573	0.08	126	5.87	5512
TBX22	1	0.623589907	0.1	134	8.01	3955	0.05	146	4.88	5402	0.05	110	2.85	7016
MLLT6	1.06	1.16E-07	0.09	136	8.01	3956	0.11	136	9.32	3136	0.11	160	11.69	3299
SLC46A1_t1	1.23	2.20E-07	0.09	138	8.01	3957	0.04	132	3.37	6267	NA	NA	NA	NA
MED19	1.19	1.88E-09	0.14	107	8.01	3958	0.14	117	9.51	3053	0.23	113	14.46	2472
MSH6	1.22	0.00034388	0.17	96	8.01	3959	0.05	91	2.15	7042	0.22	85	7.68	4762
RAB43_t1	1.22	7.48E-07	0.11	123	8.01	3960	NA	NA	NA	NA	0.1	127	7.5	4829
ARHGAP18	1.14	0.011109058	0.12	117	8.01	3961	0.21	130	16.55	933	0.11	127	8.28	4500
BEST3	1.04	0.00010336	0.11	123	8	3962	0.02	128	1.86	7236	0.06	133	5.37	5751
COL5A2_t1	1.13	0.127318345	0.1	134	8	3963	0.05	128	3.97	5922	NA	NA	NA	NA
SIPA1L2	1.57	3.54E-06	0.12	116	8	3964	0.1	119	6.97	4261	0.16	129	12.71	2971
BRAT1	1.37	5.58E-17	0.15	102	8	3965	0.25	114	15.84	1082	0.26	103	13.56	2719
NRBP2_t1	1.21	0.010577708	0.12	119	8	3966	NA	NA	NA	NA	NA	NA	NA	NA
MPZL2_t1	1.16	0.010220228	0.1	128	7.99	3967	0.06	106	3.56	6135	0.11	94	4.96	5949
ROCK1	1.04	0.503052993	0.11	120	7.99	3968	0.12	118	8.31	3589	0.2	134	17.17	1793
SCCPDH	1.27	0.00808372	0.09	138	7.99	3969	-0.04	116	-2.56	10185	0.27	126	20.16	1257
IPO7_t1	1.04	0.538414819	0.1	131	7.99	3970	NA	NA	NA	NA	0.11	129	8.64	4369
FAM81A	1.06	0.023962453	0.09	139	7.98	3971	-0.12	131	-9.51	13705	0.08	146	8.04	4601
CDRT1	1.16	1.76E-08	0.16	99	7.98	3972	0.1	123	7.3	4090	0.25	100	12.58	3012
AMPH	1.04	0.030502068	0.08	144	7.97	3973	-0.02	128	-1.45	9461	0.02	127	1.41	7803
MUC2	1.01	0.280630743	0.15	104	7.97	3974	-0.03	100	-1.49	9490	0.1	106	5.55	5657
TRAPPC3	1.07	0.028403097	0.12	114	7.97	3975	0.03	153	3.14	6417	0.06	116	3.78	6535
MED7	1.22	4.71E-08	0.16	100	7.97	3976	0.14	130	10.84	2538	0.23	132	18.66	1529
MRPL52	1.24	5.61E-08	0.12	115	7.96	3977	0.12	147	11.64	2262	0.19	152	19.13	1426
PLAC4	1.04	0.000176676	0.09	134	7.95	3978	0.07	136	6.11	4722	0.1	155	10.39	3726
PGBD1	1.08	1.41E-06	0.13	109	7.95	3979	0.08	133	6.28	4628	0.15	120	10.51	3685
COX7A2L	1.13	0.000137048	0.09	134	7.95	3980	0.05	112	3.29	6327	0.13	171	16.08	2053
STAC_t1	1.05	0.008559901	0.11	124	7.94	3981	NA	NA	NA	NA	0.07	130	5.34	5760
TMEM160	1.19	5.54E-08	0.21	88	7.94	3982	0.03	90	1.13	7727	0.19	84	6.59	5194
CEP70	1.09	0.020064785	0.11	124	7.94	3983	0.01	131	0.85	7902	0.12	134	10.32	3748
TMEM185A_t1	-1	0.93690727	0.09	136	7.94	3984	NA	NA	NA	NA	0.04	127	3.38	6726
ZNF672	1.24	1.41E-11	0.09	138	7.94	3985	-0.02	114	-1.13	9241	0.15	111	9.13	4177
DHCR24_t1	1.08	0.225586932	0.1	131	7.94	3986	0.05	125	3.78	6017	NA	NA	NA	NA
HKDC1	1.89	7.63E-07	0.1	130	7.94	3987	0.13	131	10.61	2627	0.15	143	13.8	2647
TIGD3	1.21	2.23E-11	0.09	134	7.94	3988	0.02	93	1	7811	0.15	115	9.78	3956
CLGN	2.31	1.67E-12	0.11	124	7.93	3989	0.09	116	6.02	4773	0.27	118	18.59	1537
BRWD1_t1	-1.01	0.725719231	0.07	158	7.93	3990	-0.01	145	-1.41	9436	-0.03	158	-3.02	10189
MKL1	1.1	7.89E-05	0.13	111	7.92	3991	0.14	136	12.05	2118	0.09	106	4.94	5956
TROVE2	1.24	8.05E-05	0.21	88	7.92	3992	0.22	106	12.42	2005	0.25	100	12.68	2983
PKM_t1	1.2	0.003294972	0.09	135	7.92	3993	0.13	140	11.82	2179	NA	NA	NA	NA
LINGO1_t1	1.16	4.46E-05	0.11	120	7.92	3994	NA	NA	NA	NA	0.15	100	7.4	4867
PSCA	1.06	0.000619979	0.11	124	7.92	3995	0.07	127	5.6	5006	0.16	100	7.79	4717
LMNA	1.46	4.71E-13	0.19	91	7.91	3996	0.25	88	9.67	2995	0.21	120	14.43	2479
ZNF195	1.15	0.000264595	0.1	133	7.91	3997	0.2	120	14.31	1453	0.16	128	12.56	3020
SSH1_t1	1.05	0.082066892	0.09	140	7.9	3998	NA	NA	NA	NA	0.02	144	1.9	7508
VCX2_t1	2.71	2.14E-11	0.13	112	7.9	3999	NA	NA	NA	NA	0.21	103	11.35	3397
TYRP1	1.18	4.51E-05	0.1	126	7.9	4000	0.09	130	7.04	4227	0.14	139	12.03	3185
XPO7	-1.03	0.089350672	0.11	121	7.9	4001	0.01	127	0.9	7872	-0.11	141	-10.08	12954
PDE12	1.1	3.46E-06	0.1	128	7.89	4002	0.11	129	8.47	3521	0.2	122	14.3	2521
COMMD8	1.16	0.011580815	0.09	141	7.89	4003	0.18	133	14.95	1292	0.14	135	11.95	3215
TRIL_t1	1.21	5.51E-08	0.09	136	7.89	4004	NA	NA	NA	NA	0.21	142	19.35	1375
COIL	1.11	3.84E-06	0.1	130	7.89	4005	0.04	126	3.37	6268	0.21	125	16.06	2057
SLC18B1	1.12	0.02139844	0.09	134	7.88	4006	0.18	132	14.52	1400	0.06	108	3.25	6807
CRYBG3	1.04	0.086083673	0.09	134	7.88	4007	0.14	163	16	1047	0.12	146	11.99	3200
RIBC1_t2	1.13	7.74E-08	0.18	95	7.88	4008	0.04	138	3.53	6164	0.09	120	6.64	5179
KDM4D	1.12	1.20E-10	0.12	116	7.88	4009	0.13	114	8.34	3575	0.13	120	9.29	4122
CBX4	1.25	1.74E-11	0.11	125	7.88	4010	0.11	117	7.19	4148	0.16	110	9.51	4051
ZSWIM2	1.02	0.009843844	0.08	143	7.87	4011	0.06	122	4.54	5595	-0.01	151	-0.93	9156
KIF22	1.64	1.06E-23	0.25	81	7.87	4012	0.19	122	13.56	1670	0.29	111	17.74	1692
RAG1	1.01	0.130002775	0.08	148	7.87	4013	0.15	142	13.7	1624	0.03	162	3.36	6737
NAA16_t1	1.02	0.00931825	0.1	131	7.87	4014	0	125	-0.27	8655	0.06	111	3.67	6581
USB1	1.04	0.269220596	0.11	122	7.87	4015	0.05	125	3.62	6102	0.03	138	2.33	7273
RHOF	1.08	0.000702102	0.11	124	7.87	4016	0.16	127	12.59	1958	0.01	135	0.5	8317
PAPD4	-1.06	0.088647129	0.08	152	7.87	4017	0.04	127	2.89	6557	0.08	144	7.67	4766
PAQR9	1.35	0.002422598	0.1	129	7.86	4018	0	118	-0.04	8518	0.17	129	13.08	2858
SLC37A3_t2	1.11	0.036631392	0.11	120	7.86	4019	0.14	137	12.35	2021	0.13	136	11.58	3331
KRR1	1.05	0.067424658	0.09	135	7.86	4020	0	122	0	8482	0.17	128	13.12	2849
SENP3	1.08	0.000479369	0.1	128	7.86	4021	-0.02	115	-1.04	9171	0.07	101	3.5	6669
HIST1H3D_t1	1.58	9.01E-14	0.25	82	7.86	4022	NA	NA	NA	NA	0.3	94	13.16	2844
CSPG4	1.34	1.25E-09	0.14	105	7.85	4023	0.27	120	18.75	552	0.18	132	15.08	2309
PRMT7_t1	1.07	0.033744677	0.16	100	7.85	4024	NA	NA	NA	NA	0.05	80	1.6	7682
DNAJC8	1.07	0.014314566	0.12	118	7.85	4025	0.11	122	7.66	3902	0.08	141	7.67	4765

TOMM40_t1	1.19	6.05E-11	0.18	93	7.85	4026	0.2	100	9.91	2893	NA	NA	NA	NA
MTX1	1.52	7.46E-25	0.21	87	7.85	4027	0.14	136	11.64	2263	0.28	104	15.13	2299
QTRTD1	1.13	0.000337782	0.1	129	7.85	4028	0.2	158	21.23	265	0.12	122	8.96	4243
RBX1	1.12	0.000214319	0.13	110	7.85	4029	0.2	99	9.98	2869	0.06	119	3.81	6518
GEMIN6	1.37	2.65E-18	0.19	92	7.85	4030	0.18	89	7.16	4161	0.27	71	5.61	5617
FICD	1.32	1.72E-10	0.2	90	7.84	4031	0.17	108	10.03	2854	0.2	87	7.5	4831
IKBIP_t1	1.08	0.015267852	0.09	137	7.84	4032	0.06	132	4.97	5358	0.14	136	11.99	3201
SNX6	1.02	0.722067708	0.08	147	7.83	4033	0.03	147	2.49	6832	0.03	126	1.95	7479.5
CRYZL1	1.07	0.024420215	0.09	133	7.83	4034	0.13	125	9.41	3103	0.1	131	7.81	4709
RB1_t1	-1.01	0.634159439	0.08	149	7.83	4035	NA	NA	NA	NA	-0.04	123	-2.62	10005
CAV1	1.08	0.230427789	0.09	135	7.83	4036	0.12	113	7.7	3879	0.06	136	5.02	5913
VHLL	1.06	3.28E-06	0.16	98	7.83	4037	0.13	108	7.76	3852	0.12	80	3.72	6557
EDEM2_t1	1.09	0.00092089	0.13	112	7.82	4038	NA	NA	NA	NA	0.09	105	4.7	6069
VAPA	1.05	0.194634046	0.09	136	7.82	4039	-0.06	141	-5.08	11702	0.07	139	5.99	5454
VPS33A	1.31	3.68E-12	0.17	95	7.82	4040	0.17	127	13.39	1719	0.2	118	13.65	2693
NDUFB5	1.05	0.192558679	0.12	116	7.82	4041	-0.02	111	-1.44	9453	0.13	115	8.41	4463
TDRD7	1.05	0.266271345	0.09	140	7.82	4042	-0.05	93	-2.05	9862	0.04	136	3.15	6855
RNF24	1.09	0.000224204	0.08	142	7.82	4043	0.1	152	10.23	2784	0.04	125	3.17	6849
TMEM98_t1	1.29	0.002070692	0.11	118	7.82	4044	NA	NA	NA	NA	NA	NA	NA	NA
NUPR1	1.49	4.04E-08	0.18	93	7.81	4045	0.14	126	10.79	2556	0.24	91	10.01	3875
IMPA2	1.07	0.256005637	0.1	127	7.81	4046	-0.08	134	-6.52	12420	-0.04	104	-2.23	9813
KIRREL2_t1	1.14	0.002367399	0.11	118	7.81	4047	0.02	129	1.56	7456	0.13	106	7.06	5002
LSS_t1	1.36	8.09E-05	0.13	110	7.81	4048	0.08	143	7.56	3957	0.12	130	9.32	4112
MET	1.26	0.020390707	0.1	126	7.81	4049	0.06	150	6.47	4529.5	0.21	163	23.87	740
WWP1	-1.07	0.342609655	0.1	129	7.79	4050	0	119	-0.18	8602	0.16	132	12.98	2892
RNF138	1	0.841361125	0.09	135	7.79	4051	0.04	137	3.36	6274	0.06	127	4.5	6178
TRIM52	1.24	1.80E-11	0.13	111	7.78	4052	0.08	125	6.25	4639	0.24	142	21.89	983
ITPK1	1.1	2.57E-08	0.11	120	7.78	4053	0.02	144	1.44	7529	0.12	140	10.47	3703
CHST10	1.08	8.28E-06	0.12	116	7.78	4054	0.17	124	12.72	1921	0.08	113	5	5922
HNRNPA1_t1	1.08	0.206504787	0.1	125	7.78	4055	0.15	111	9.27	3157	0.06	133	4.59	6128
APOE	1.42	9.47E-05	0.21	87	7.78	4056	-0.02	90	-0.78	8994	0.24	94	10.72	3607
IFI27L1	1.4	1.79E-11	0.15	102	7.78	4057	0.05	116	3.02	6484	0.15	80	4.38	6257
FBXO11	1.05	0.324587727	0.08	149	7.78	4058	0.2	119	14.14	1497	0.1	120	7.31	4911
PCMTD1	1.03	0.32557732	0.08	153	7.78	4059	0.05	124	3.86	5970	0.14	129	11.43	3373
RPL10A	1.23	8.10E-05	0.14	104	7.77	4060	0.2	95	9.2	3192	0.14	94	6.25	5336
ZDHHC24	1.22	4.51E-08	0.16	100	7.77	4061	0.09	141	8.03	3728	0.07	118	4.82	6017
HTR2A	1.04	0.001269504	0.08	149	7.77	4062	0.17	135	14.59	1378	0.1	138	9.17	4168
ERBB3	1.2	0.000537648	0.09	139	7.77	4063	-0.07	125	-5.07	11696	0.12	150	11.84	3251
HIST1H2AE	1.66	1.51E-12	0.13	111	7.76	4064	0.04	88	1.42	7541	0.16	71	3.32	6760
PHF5A	1.04	0.315308937	0.1	128	7.76	4065	0.02	114	1.56	7462	0.04	104	2.32	7278
RMND5B	1.18	1.14E-10	0.13	111	7.76	4066	0.03	99	1.35	7572	0.13	119	9.1	4185
TMSB15A	1.1	0.005012892	0.11	120	7.75	4067	0.1	110	5.82	4886	0.16	132	12.84	2944
CAMTA2	1.01	0.572196151	0.11	120	7.75	4068	-0.11	123	-8.36	13269	-0.01	113	-0.54	8944
BRIP1	1.13	3.81E-09	0.1	125	7.75	4069	0.17	131	13.68	1630	0.17	128	12.88	2933
RPL18	1.19	1.19E-05	0.15	102	7.75	4070	0.11	95	4.74	5477	0.07	89	2.57	7149
FAM204A_t1	-1	0.973355169	0.11	122	7.75	4071	NA	NA	NA	NA	0.14	123	10.5	3687
CXorf59	1.03	0.002378151	0.14	104	7.74	4072	0.09	101	4.75	5471	0.04	126	2.8	7033
NIN_t1	1.03	0.044291924	0.12	115	7.74	4073	0.11	125	8.49	3508	0.06	124	4.4	6249
FBLIM1_t1	1.13	0.101778598	0.09	136	7.74	4074	0.15	136	12.77	1908	0.05	140	4.78	6036
ELF3	-1.15	0.109991608	0.1	127	7.73	4075	-0.02	124	-1.34	9391	-0.01	129	-0.66	9016
IDH3G	1.13	7.96E-05	0.13	109	7.73	4076	0.06	122	4.35	5689	0.03	105	1.55	7715
PYROXD1	1.13	5.54E-05	0.1	128	7.73	4077	0.09	118	5.83	4883	0.21	142	19.14	1422
DHX8	1.15	1.21E-10	0.13	109	7.73	4078	-0.02	114	-1.5	9493	0.12	119	8.12	4574
OSGEPL1	1.26	4.97E-08	0.12	115	7.73	4079	0.13	114	8.51	3494	0.25	121	17.58	1720
PTPRZ1	1.05	1.51E-05	0.12	115	7.73	4080	0.12	124	9.1	3226	0.17	138	14.66	2414
FAM89B	1.05	0.216663815	0.11	118	7.73	4081	0.12	122	9	3273	0.03	126	2.07	7415
SHQ1	1.16	3.05E-11	0.07	153	7.72	4082	0.16	108	9.34	3130	0.24	131	19.24	1400
HIF1AN	1.03	0.025841348	0.1	130	7.72	4083	0.02	149	1.78	7290	0.04	137	3.05	6906
HIST1H3E	1.3	2.23E-08	0.23	84	7.72	4084	0.16	81	4.83	5435	0.17	81	5.24	5812
ARG2	1.38	0.000249864	0.11	122	7.72	4085	-0.01	132	-0.8	9016	0.17	120	11.93	3224
EXOSC8	1.05	0.270842601	0.14	104	7.72	4086	0.19	122	13.82	1591	0.01	128	0.83	8124
TMEM60	1.09	0.02397064	0.13	109	7.71	4087	0.02	110	1.42	7545	0.04	103	2.23	7329
PABPC3	1.24	0.000113023	0.09	138	7.71	4088	0.19	110	11.46	2318	0.14	112	8.99	4232
PNMA2	1.03	0.025220516	0.09	132	7.71	4089	0.03	118	1.9	7215	0.1	135	8.53	4413
POMZP3	1.06	0.00011111	0.14	107	7.71	4090	0.04	113	2.53	6809	0.14	112	8.43	4456
CHD6	1.05	6.80E-05	0.1	126	7.71	4091	0.13	136	11.17	2410	0.13	142	12.31	3107
GNAT1	1.02	0.099270273	0.12	114	7.7	4092	-0.08	141	-7.69	12979	0.16	142	14.74	2396
AGPAT4_t1	1.19	7.09E-05	0.08	141	7.7	4093	0.19	138	16.59	923	NA	NA	NA	NA
LSMD1	1.06	5.10E-06	0.19	90	7.7	4094	0.1	110	5.79	4896	0.18	98	8.67	4359
MIF4GD	1.32	1.99E-13	0.15	100	7.7	4095	0.11	107	6.3	4615	0.22	120	15.33	2240
WDSUB1	1.15	2.41E-05	0.13	109	7.69	4096	0.15	108	8.44	3531	0.24	133	19.96	1276
SMPDL3B	1.1	0.021190699	0.12	113	7.69	4097	0.02	113	1.39	7556	-0.04	95	-1.95	9693
DDX49	1.28	1.66E-12	0.12	112	7.69	4098	0.12	106	6.52	4500	0.13	93	5.58	5631
ZNF519	1.08	0.000237893	0.09	131	7.69	4099	0.13	150	12.67	1936	0.12	135	10.53	3681
ATR	1.3	8.72E-06	0.17	96	7.69	4100	0.25	114	16.01	1045	0.23	106	12.76	2959
MYL12B_t1	1.04	0.547335599	0.11	120	7.69	4101	NA	NA	NA	NA	NA	NA	NA	NA
EXD2	-1	0.859630771	0.08	146	7.69	4102	0	145	-0.19	8609	0	144	0.03	8606
SPDYE1_t1	1.07	1.45E-09	0.1	129	7.69	4103	NA	NA	NA	NA	0.14	128	11.03	3505
ABHD12_t1	1.18	8.37E-05	0.22	85	7.68	4104	NA	NA	NA	NA	0.15	120	10.73	3601
ZNF575	1.13	9.35E-05	0.14	104	7.68	4105	0.01	110	0.54	8111	0.14	118	9.76	3963
SLC17A5	1.2	1.98E-05	0.09	131	7.68	4106	0.1	135	8.78	3383	0.17	152	17.25	1776
HNRNPH1	1.06	0.245127241	0.11	120	7.68	4107	0.13	109	7.94	3780	0.14	154	14.51	2458
SLC39A3_t1	1.13	3.35E-10	0.09	135	7.68	4108	0.23	131	18.59	574	0.18	125	13.38	2771
DCAF4L2	1.76	5.00E-09	0.08	152	7.68	4109	0.05	129	4.12	5829	0.16	131	12.87	2935
ZNF23_t1	1.04	0.234857342	0.11	123	7.67	4110	NA	NA	NA	NA	0.14	110	8.43	4455
ELFN2	1.36	2.13E-12	0.12	115	7.67	4111	-0.07	163	-7.64	12953	0.25	127	18.9	1476
DPP8_t1	1.07	0.234724041	0.09	140	7.67	4112	0.15	122	10.71	2587	0.16	121	11.61	3321
SLC6A3	1.02	0.02324579	0.11	117	7.67	4113	0.05	129	3.81	5999	0.11	139	9.86	3927
TEAD2	1.11	0.000105513	0.11	121	7.67	4114	0.14	120	9.69	2981	0.08	111	4.89	5976
TMEM9	1.13	0.000414553	0.1	127	7.67	4115	-0.03	126	-2.38	10069	0.13	140	11.33	3407
SMEK3P	1.1	6.99E-07	0.08	150	7.67	4116	0.04	128	3.1	6436	0.15	127	11.66	3303
ATP6V0C	1.02	0.582046269	0.12	114	7.66	4117	0.19	110	11.26	2380	0.06	114	3.92	6455
RPA3	1.32	4.77E-08	0.15	100	7.66	4118	0.06	86	2.16	7033	0.2	99	9.86	3925
MRPS35	1.11	0.04065263	0.1	125	7.66	4119	0.09	118	5.86	4866	0.19	129	15.05	2317
ERCC4	1.07	1.53E-06	0.08	146	7.66	4120	0.06	135	5.01	5332	0.24	159	25.8	526
REG1P	1.03	0.075208697	0.1	123	7.66	4121	0.09	127	6.71	4395	0	127	0.07	8575
CDC25B	1.32	1.57E-12	0.1	127	7.65	4122	0.12	134	9.7	2975	0.12	115	8.07	4594
CLN6	1.16	3.72E-08	0.13	111	7.65	4123	0.01	153	0.6	8078	0.12	145	11.77	3277
RBM15	1.13	4.24E-15	0.11	118	7.65	4124	0.03	95	1.19	7684	0.11	129	8.81	4301
MRPL24	1.52	2.15E-15	0.15	100	7.65	4125	0.13	112	8.03	3730	0.21	100	10.42	3715
SAYSD1	1.23	1.09E-07	0.09	132	7.65	4126	0.1	135	8.43	3535	0.2	121	14.35	2505
ZBTB6	1.01	0.608846564	0.07	161	7.64	4127	0.13	126	9.56	3034	-0.01	133	-0.48	8898
PFKFB4	1.26	9.22E-08	0.11	122	7.64	4128	0.13	140	11.27	2371	0.09	110	5.31	5773
TSTA3	1.4	4.75E-10	0.22	84	7.63	4129	0.08	120	5.82	4887	0.23	104	12.21	3133
MTMR1	1.06	1.06E-07	0.11	120	7.63	4130	0.12	110	7.22	4130	0.14	143	12.95	2906
SLC25A12_t1	1.17	0.023736578	0.1	123	7.63	4131	NA	NA	NA	NA	NA	NA	NA	NA

DLL3_t2	1.02	0.018605099	0.12	116	7.63	4132.5	-0.09	107	-4.87	11574	0.08	141	7.63	4780
FAM127B_t1	1.07	0.28979379	0.12	116	7.63	4132.5	0.18	124	13.16	1782	NA	NA	NA	NA
DDX27	1.32	2.74E-13	0.17	95	7.62	4134	0.25	111	15.4	1167	0.25	85	8.86	4272
RBM14	1.11	1.29E-06	0.08	146	7.61	4135	0.02	120	1.64	7399	0.05	135	4.26	6313
OXR1_t1	1.03	0.448117855	0.11	122	7.61	4136	NA	NA	NA	NA	0.13	143	12.23	3126
SLITRK1_t1	1.03	0.029030673	0.11	121	7.61	4137	NA	NA	NA	NA	0.01	125	0.41	8365
LAMB4	1.01	0.314685425	0.1	125	7.6	4138	0.05	107	2.85	6586	0	108	-0.17	8715
ZFP37	1.05	0.001858375	0.08	149	7.6	4139	0.06	112	3.87	5967	0.15	139	13.49	2742
HFE	1.12	0.010817085	0.11	119	7.6	4140	0.03	122	1.92	7197	0.12	125	9.02	4222
TAGLN2	1.18	0.000209175	0.13	107	7.6	4141	0.12	131	9.58	3028	0.17	120	11.85	3249
SMCR7L	1.26	1.72E-11	0.09	133	7.6	4142	0.11	134	9.61	3016	0.22	131	17.54	1729
ZNF33A_t1	1.09	0.003302717	0.09	138	7.59	4143	0	142	0.24	8298	0.13	158	13.9	2617
WDR19	1.12	4.60E-13	0.14	104	7.59	4144	0.2	139	18.16	633	0.2	134	16.98	1833
CPLX1_t1	-1.01	0.516570009	0.12	115	7.59	4145	NA	NA	NA	NA	NA	NA	NA	NA
GSDMD	1.18	1.30E-05	0.17	95	7.59	4146	-0.03	99	-1.43	9449	0.15	88	5.77	5550
SRFBP1	1.07	0.041866576	0.09	136	7.59	4147	0.08	135	6.89	4308	0.26	139	22.7	878
ZNF202	1.1	2.56E-06	0.11	117	7.59	4148	0.22	143	20.53	335	0.04	131	3.37	6730
FAM83A_t1	1.06	4.41E-07	0.09	136	7.59	4149	0.04	132	2.91	6545	0.2	124	14.55	2446
GALNT5	1.09	7.72E-05	0.13	108	7.58	4150	0.02	100	1.25	7643	0.15	80	4.4	6250
ZBTB8A	1.12	0.005791116	0.09	136	7.58	4151	0.07	147	7.16	4163	0.17	152	16.94	1843
TRIM4	1.17	1.99E-12	0.08	143	7.58	4152	0.1	130	8.31	3591	0.22	151	22.05	961
SH3BP5L	1.34	5.66E-17	0.1	123	7.58	4153	0.1	118	6.77	4366	0.17	113	10.61	3647
MSH4	1.07	2.45E-05	0.11	118	7.58	4154	0.21	107	11.74	2221	0.17	111	10.18	3800
NMU	1.21	1.49E-07	0.16	97	7.58	4155	0.1	102	5.18	5229	0.14	105	7.72	4749
ZNF385D	1.32	9.68E-06	0.14	106	7.58	4156	0.05	114	3.2	6381	0.18	129	14.04	2588
CAPNS2	1.12	2.12E-06	0.15	99	7.57	4157	0.1	107	5.93	4826	0.11	95	5.05	5903
ISX	2.29	2.37E-11	0.11	118	7.57	4158	0.14	150	13.83	1586	0.17	128	13.28	2802
MSN	1.05	0.019107002	0.09	136	7.57	4159	0.14	151	14.51	1401	0.1	119	6.56	5205
CHCHD1	1.18	1.34E-06	0.11	117	7.57	4160	0.1	116	6.89	4304	0.22	121	15.39	2219
TOB2	1.15	1.36E-07	0.1	125	7.57	4161	-0.07	150	-6.57	12447	0.1	135	8.73	4332
GRB2_t1	1.08	0.006957305	0.1	127	7.56	4162	NA	NA	NA	NA	NA	NA	NA	NA
UBA1_t1	1.09	0.000784742	0.11	120	7.56	4163	-0.05	125	-3.43	10756	NA	NA	NA	NA
CLCN3_t2	1.04	0.125859917	0.09	130	7.56	4164	0.03	142	2.67	6703	0.11	168	13.24	2816
ACD	1.16	1.57E-08	0.2	87	7.56	4165	0.06	142	5.33	5158	0.07	87	2.62	7125
ZNF367	1.05	8.24E-06	0.09	134	7.56	4166	0.07	127	5.02	5320	0.1	143	9	4228
RCC2	1.12	0.030710216	0.1	124	7.56	4167	0.19	141	17.21	797	-0.01	131	-0.72	9043
TLE1	1.09	0.074209945	0.11	116	7.56	4168	0.02	102	1	7806	0.09	122	6.16	5376
CAPN13	1.21	1.31E-05	0.13	108	7.54	4169	-0.04	115	-2.73	10297	0.12	119	8.39	4472
RPF1	-1	0.916988965	0.12	115	7.54	4170	0.01	126	0.59	8079	0.12	126	9.06	4205
FNTB	1.16	2.23E-08	0.11	116	7.54	4171	0.07	127	5.33	5156	0.06	136	4.75	6054
HTN1	1	0.693785984	0.1	129	7.54	4172	0.04	125	3.01	6487	0.05	118	3.69	6572
CDKL1_t1	1.25	1.62E-11	0.17	94	7.54	4173	NA	NA	NA	NA	0.2	80	5.98	5466
EXOC7_t1	1.36	2.02E-14	0.11	120	7.53	4174	0.17	131	13.78	1604	NA	NA	NA	NA
HSD17B7_t1	1.36	1.39E-06	0.1	128	7.53	4175	0.03	128	2.69	6688	NA	NA	NA	NA
SOD2	1.26	0.013859032	0.11	120	7.53	4176	0.05	100	2.7	6680	0.05	98	2.6	7129
RPL14	1.42	1.90E-12	0.16	96	7.52	4177	0.25	91	10.06	2839	0.27	107	15.3	2247
MKKS_t1	1.24	8.46E-06	0.09	130	7.52	4178	0.23	128	18.15	634	NA	NA	NA	NA
SLC26A7_t2	1.03	0.010621102	0.08	141	7.52	4179	0.11	116	7.47	4005	0.08	111	5.14	5857
FLCN_t1	1.24	3.15E-07	0.12	114	7.52	4180	0.12	88	4.58	5572	0.18	97	8.54	4409
URI1	1.17	0.000115913	0.08	140	7.51	4181	0.14	140	12.38	2015	0.18	131	14.4	2489
TTTY4C	-1.01	0.423314131	0.11	119	7.51	4182	-0.02	136	-1.37	9410	0.01	117	0.55	8290
MAGEA6	1.85	6.47E-10	0.09	130	7.51	4183	-0.01	111	-0.38	8730	0.22	118	14.73	2399
ECE2_t1	1.25	2.87E-06	0.14	105	7.51	4184	0.06	107	3.56	6138	0.19	89	7.33	4904
STAT1_t1	-1.07	0.301217096	0.09	138	7.51	4185	0	118	-0.24	8636	-0.07	132	-5.38	11191
KCNIP1	-1.01	0.267167285	0.1	124	7.51	4186	-0.14	147	-13.96	14987	0.01	147	1.1	7963
CRIP3	1.67	1.27E-18	0.15	100	7.51	4187	0.14	115	9.31	3140	0.29	107	16.8	1873
UQCR10	1.11	0.004733813	0.11	120	7.51	4188	0.11	120	7.53	3974	0.19	117	12.69	2978
ZNF345	1.13	0.000244605	0.08	140	7.51	4189	0.14	121	10.03	2852	0.08	128	5.86	5521
FADD	1.21	7.98E-05	0.13	109	7.51	4190	0.11	115	6.96	4269	0.12	127	9.02	4223
NFS1	1.17	1.96E-05	0.09	138	7.51	4191	0.03	128	2.52	6812	0.21	124	15.41	2215
BLOC1S5	1.13	0.011815151	0.07	156	7.5	4192	0.06	136	4.97	5357	0.13	123	9.48	4059
EIF3I	1.08	0.017729639	0.11	120	7.5	4193	0.04	139	3.32	6300	0.12	150	11.57	3333
AGPAT1	1.35	1.42E-16	0.12	113	7.49	4194	0.13	137	11.55	2288	0.21	120	15.02	2334
ATP9B	1.08	9.61E-10	0.09	129	7.49	4195	0.12	109	7.33	4075	0.21	141	19.11	1433
PGAM1	-1.06	0.125400166	0.08	146	7.49	4196	-0.11	123	-8.18	13183	0.02	115	1.47	7765
MED10_t1	1.12	0.00406946	0.1	122	7.49	4197	NA	NA	NA	NA	0.11	122	7.85	4688
EYA4	1.04	0.16098662	0.1	124	7.49	4198	-0.06	131	-5.1	11720	0.07	148	6.74	5135
PTGDR2	1.11	0.000473526	0.11	116	7.49	4199	0.02	123	1.73	7323	0.16	139	13.9	2620
BBC3	1.21	0.001084242	0.12	113	7.49	4200	0.07	120	4.9	5392	0.04	123	2.57	7147
MEX3B	1.07	0.298587219	0.08	146	7.49	4201	0.16	124	11.52	2298	0.15	132	12.44	3052
KRT38	1.05	6.30E-06	0.11	120	7.49	4202	0.06	122	4.52	5604	0.15	167	17.19	1785
DLX2	1.03	0.056856846	0.11	120	7.48	4203	0.06	120	3.97	5923	0.07	106	3.98	6423
RPLP1_t1	1.84	1.26E-18	0.25	80	7.48	4204	0.31	88	11.83	2174	NA	NA	NA	NA
XPNPEP1	1.07	0.016708845	0.1	122	7.48	4205	0.02	130	1.96	7167	0.1	115	6.62	5185
SCD_t1	1.26	5.50E-06	0.08	141	7.48	4206	NA	NA	NA	NA	0.12	131	10.04	3864
LRP2BP_t1	-1.01	0.649645314	0.09	135	7.48	4207	NA	NA	NA	NA	0.14	147	13.55	2723
PPP1R1B	1.06	0.002002031	0.1	126	7.48	4208	0.07	110	4.32	5709	0.06	121	4.46	6210
IL2RA	1.04	0.380130269	0.08	145	7.47	4209	0.1	139	8.59	3460	-0.06	131	-5.17	11108
PM20D1	-1	0.635725072	0.1	126	7.47	4210	0.15	116	9.73	2966	-0.01	119	-0.79	9080
ENTPD3	1.01	0.22050217	0.08	140	7.47	4211	0.05	136	4.27	5740	0.15	134	12.23	3130
SEC61A1	1.16	1.61E-05	0.1	123	7.47	4212	0.12	140	10.94	2497	0.07	143	6.57	5200
MFAP1	1.02	0.77678614	0.09	133	7.47	4213	-0.03	110	-1.78	9678	0.11	124	7.87	4680
ZFYVE20_t1	1.06	0.02652551	0.08	138	7.47	4214	NA	NA	NA	NA	0.04	137	3.61	6608
TFAP4	1.04	6.82E-06	0.12	110	7.47	4215	0.06	123	4.1	5844	0.18	130	14.34	2510
FLYWCH1_t1	1.12	8.58E-09	0.14	104	7.46	4216	0.07	72	1.62	7409	0.18	108	10.31	3756
TIMM17A	1.25	0.001273339	0.08	140	7.46	4217	0.1	118	6.47	4534	0.17	137	14.91	2359
CYP11B2	1.08	0.083691704	0.11	115	7.46	4218	0.07	120	4.85	5430	0.15	112	9.33	4109
PACS1	1.04	0.124602896	0.11	115	7.45	4219	0.16	133	13.69	1628	0	131	0.02	8610
TUBB_t1	1.23	2.92E-07	0.1	122	7.45	4220	NA	NA	NA	NA	0.1	131	8.45	4445
HSPE1	1.55	2.52E-10	0.16	98	7.45	4221	0.22	130	17.88	687	0.26	132	21.2	1084
PEX1	1.16	0.000422492	0.14	105	7.45	4222	0.05	110	3.04	6473	0.25	103	13.33	2787
CDK20	1.14	0.004350632	0.1	122	7.45	4223	0.02	125	1.35	7578	0.06	108	3.46	6686
MORF4L2	1.27	6.46E-06	0.09	134	7.45	4224	0.14	120	9.96	2877	0.25	127	19.23	1402
ERI3	1.1	5.00E-05	0.14	104	7.45	4225	-0.01	108	-0.51	8823	0.1	117	6.79	5109
RAB24	1.32	7.20E-12	0.11	118	7.45	4226	0.21	119	14.65	1367	0.19	120	13.08	2857
PCGF1	1.2	5.90E-14	0.13	106	7.44	4227	0.11	123	8.24	3629	0.21	88	8.1	4586
ARPC1B	1.1	0.179248425	0.11	119	7.44	4228	0.23	88	8.76	3387	-0.04	110	-2.27	9830
CCDC81	-1	0.726018761	0.09	130	7.44	4229	-0.03	122	-1.9	9747	0.03	103	1.85	7538
BCAS1	1.18	1.76E-05	0.11	120	7.44	4230	0.11	143	10.49	2684	0.13	156	13.75	2663
SFXN3	1.03	0.04076576	0.08	140	7.44	4231	0.06	119	3.87	5965	-0.04	134	-3.29	10308
ZNF354A	1.12	0.004895536	0.12	110	7.44	4232	0.22	111	13.23	1763	0.25	118	16.71	1901
NPFFR2_t1	1.23	3.79E-07	0.13	108	7.44	4233	NA	NA	NA	NA	0.25	167	28.89	312
FNDC3B	1.06	0.39695007	0.08	139	7.43	4234	0.17	138	14.96	1291	0.05	136	4.7	6070
NEURL2	1.25	3.69E-08	0.15	100	7.43	4235	0.01	101	0.53	8124	0.24	103	12.78	2955
AMBRA1	1.25	6.94E-16	0.1	124	7.43	4236	0.15	108	8.6	3454	0.2	156	21.71	1015
LMO3	1.05	0.241479766	0.08	142	7.43	4237	0.14	120	9.99	2862	0.03	147	3.23	6811

RHOBTB2	1.19	0.000528691	0.11	120	7.42	4238	0.15	135	12.98	1841	0.01	139	1.21	7907
DLX6	1.05	2.57E-05	0.1	125	7.42	4239	0.07	129	5.29	5173	0.24	118	16.02	2067
MYO6	-1.01	0.842493577	0.09	137	7.42	4240	0.14	140	12.6	1952	0.08	150	7.73	4742
CCDC87	1.24	3.73E-09	0.11	115	7.42	4241	0.06	104	3.24	6346	0.18	128	14.25	2537
HHIPL2	1.23	5.94E-06	0.11	120	7.42	4242	0.04	111	2.37	6904	0.15	105	8.19	4540
DEAF1	1.19	3.70E-08	0.15	100	7.41	4243	0.11	134	9.28	3155	0.07	110	4.44	6222
MUC17_t1	1.02	0.005465478	0.11	115	7.41	4244	0.14	135	12.18	2068	NA	NA	NA	NA
DNAJB2_t1	1.15	2.57E-05	0.11	115	7.41	4245	0.19	120	13.47	1694	0.03	131	2.23	7333
CREB3L4	1.37	1.31E-08	0.11	119	7.41	4246	0.04	112	2.51	6821	0.11	107	6.07	5416
FIGF	1.55	1.57E-06	0.11	115	7.41	4247	0.09	106	4.87	5414	0.16	104	8.66	4362
VAPB	1.13	6.99E-06	0.07	150	7.4	4248	-0.02	152	-2.47	10133	0.15	150	15.15	2294
CABYR	1.72	5.16E-10	0.14	103	7.4	4249	0.17	121	11.8	2190	0.16	119	11.31	3410
TM4SF5	1.22	0.037294174	0.18	91	7.4	4250	-0.01	120	-0.95	9112	0.16	127	12.23	3128
TP53I3	1.99	1.73E-18	0.18	90	7.4	4251	0.12	88	4.47	5622	0.18	90	7.38	4883
FAM196A	1.05	0.0005412	0.11	120	7.4	4252	0.22	121	15.86	1076	0.2	145	18.66	1527
MUCL1	1.09	0.025014219	0.13	106	7.4	4253	-0.09	111	-5.48	11902	0.22	93	9.38	4091
UPF2	1.05	0.138346664	0.1	125	7.4	4254	0.03	127	2.32	6944	0.13	133	10.53	3680
NOL3	1.11	0.087932448	0.13	109	7.39	4255	0.09	112	5.67	4964	0.1	100	4.8	6028
NEK3_t1	1.13	0.002866524	0.1	124	7.39	4256	NA	NA	NA	NA	0.08	118	5.11	5875
ATRN_t1	1.09	0.129888609	0.08	145	7.39	4257	-0.12	137	-10.23	13971	0.14	153	14.15	2558
CYHR1_t1	1.16	0.000601825	0.15	100	7.39	4258	0.02	108	1.17	7695	NA	NA	NA	NA
SSX7	1.34	1.59E-07	0.13	107	7.39	4259	0.05	120	3.55	6145	0.13	124	9.59	4028
PCBP4	1.23	5.23E-06	0.12	113	7.39	4260	0.14	140	12.7	1930	0.15	115	9.88	3921
BMP7	1.02	0.003080358	0.09	136	7.39	4261	0.12	127	8.87	3342	0.09	131	7.4	4871
NBAS_t1	1.27	2.15E-14	0.19	88	7.38	4262	NA	NA	NA	NA	NA	NA	NA	NA
FLAD1_t1	1.55	8.60E-23	0.24	81	7.38	4263	0.19	88	7.4	4035	NA	NA	NA	NA
LUC7L3_t2	1.17	0.00745316	0.1	125	7.38	4264	0.15	148	14.73	1347	0.13	142	11.77	3275
SH2D4B	1.1	7.27E-11	0.09	129	7.38	4265	0.09	124	6.49	4517	0.19	152	19.64	1325
CLHC1	1.09	0.000637442	0.09	136	7.38	4266	0.12	124	9.01	3263	0.03	139	2.63	7117
KIAA1377_t1	1.02	0.144230989	0.07	160	7.38	4267	NA	NA	NA	NA	0.03	134	2.59	7135
PTPN4	1.02	0.205069681	0.08	148	7.37	4268	0.02	135	1.85	7246	0.16	160	17.13	1805
CCDC77	1.2	1.06E-06	0.09	131	7.37	4269	0.1	119	7.16	4160	0.2	131	15.99	2072
CIAPIN1_t1	1.12	8.27E-08	0.09	128	7.37	4270	NA	NA	NA	NA	0.1	127	7.62	4783
TCAIM_t1	1.04	0.21212844	0.07	160	7.37	4271	-0.05	143	-4.96	11637	0.16	140	14.19	2547
EIF4G2	1.08	0.155596437	0.07	152	7.37	4272	0.18	119	12.17	2077	0.14	128	10.94	3527
STOML3	1.03	0.00186507	0.11	120	7.37	4273	-0.05	124	-3.4	10731	0.14	134	11.8	3262
LMNB1	1.39	1.98E-08	0.11	116	7.36	4274	0.03	129	2.71	6672	0.22	134	18.89	1479
C1GALT1C1	1.05	0.314893465	0.1	123	7.36	4275	0.08	108	4.47	5628	0.06	122	3.99	6420
PAG1_t1	-1.18	0.130869832	0.07	151	7.36	4276	NA	NA	NA	NA	NA	NA	NA	NA
CASZ1_t1	1.05	3.18E-07	0.07	163	7.35	4277	0.12	123	8.63	3441	0.16	138	14.14	2563
BBS10	1.16	3.17E-06	0.09	130	7.35	4278	0.17	130	13.62	1646	0.19	124	14.34	2508
CRELD2	1.34	1.63E-10	0.11	114	7.35	4279	0.11	109	6.39	4567	0.11	123	8.26	4511
HNF4G_t1	1.03	0.004332598	0.1	123	7.35	4280	0.02	128	1.49	7496	NA	NA	NA	NA
MYOZ2_t1	1.04	9.17E-05	0.1	120	7.35	4281	NA	NA	NA	NA	0.07	143	6.55	5209
TKT	1.83	4.81E-14	0.2	87	7.34	4282	0.31	125	23.32	137	0.13	104	7.27	4921
MRPL33_t1	1.17	1.29E-05	0.12	109	7.34	4283	NA	NA	NA	NA	0.25	121	17.82	1679
TTC23	1.23	2.78E-08	0.1	122	7.34	4284	0.09	130	7.1	4199	0.18	107	10.5	3689
CAPSL	1.08	1.23E-09	0.09	132	7.34	4285	0	137	0.17	8353	0.08	140	6.77	5124
ZNF467	1.04	0.000283296	0.12	112	7.33	4286	-0.02	104	-1.13	9238	0.1	110	6.06	5421
ABHD4	1.07	0.018812425	0.1	123	7.33	4287	-0.05	125	-3.47	10781	0.04	135	2.98	6935
NRCAM	1.62	8.79E-08	0.09	135	7.33	4288	0.04	124	2.99	6506	0.12	144	10.88	3559
RBL1_t2	1.07	2.40E-07	0.16	95	7.33	4289	0.02	96	0.69	8006	0.18	102	9.23	4146
WDR37	1.1	0.000469411	0.1	126	7.33	4290	0.1	131	7.98	3754	0.03	128	2.5	7179
TMEM248	1.11	0.000489672	0.08	143	7.33	4291	0.17	127	13.38	1723	0.13	141	11.41	3377
CCL18	1.15	0.124713915	0.1	121	7.33	4292	0.09	117	6.01	4777	-0.03	109	-2.05	9736
TCTA	1.18	2.08E-07	0.09	131	7.33	4293	0.04	127	2.87	6572	0.12	132	9.44	4074
CCDC6	1.1	0.086720081	0.07	155	7.33	4294	0.13	150	13.25	1755	0.13	141	11.69	3300
B2M	-1.03	0.737987924	0.1	123	7.33	4295	0.03	127	2.43	6871	-0.03	150	-3.4	10355
DUT	1.35	3.05E-08	0.1	127	7.32	4296	0.07	125	5.09	5275	0.2	151	20.49	1201
IDUA	1.39	2.79E-14	0.13	108	7.32	4297	0.06	111	3.65	6086	0.19	130	15.29	2248
KRT73	1.01	0.091629037	0.09	136	7.32	4298	0.14	99	6.9	4298	0.07	115	4.3	6291
TLN2	1.27	3.82E-05	0.09	132	7.32	4299	0.08	144	7.65	3907	0.12	128	9.65	4006
CALR3	1.05	0.000552497	0.12	112	7.32	4300	-0.02	109	-1.12	9235	0.04	114	2.5	7180
RABEPK	1.36	0.000139291	0.11	114	7.32	4301	0.15	106	8.48	3515	0.09	120	6.6	5190
ZFYVE9_t1	1.06	0.019314914	0.11	118	7.32	4302	-0.1	139	-8.6	13362	0.16	145	14.77	2390
ZCCHC7	1.11	5.30E-05	0.1	125	7.31	4303	0.09	106	4.79	5447	0.22	131	17.96	1656
PTEN	1.01	0.852696817	0.07	157	7.31	4304	0.02	130	1.43	7534	0.2	143	19.04	1443
ZNF808	1.1	0.056418184	0.12	113	7.31	4305	0.2	123	14.44	1416	0.12	126	8.98	4235
PTPN11	1.14	4.02E-10	0.09	132	7.31	4306	0.11	122	7.76	3856	0.2	151	20.22	1243
AIMP1	1.07	0.163666864	0.09	130	7.3	4307	0.09	134	7.73	3870	0.07	131	5.95	5473
PPP6C_t1	1.02	0.422666822	0.08	137	7.3	4308	NA	NA	NA	NA	NA	NA	NA	NA
ALS2CR12	1.01	0.271814534	0.1	124	7.3	4309	0.05	108	2.79	6621	0.04	116	2.51	7175
HSPBAP1	1.06	0.098281808	0.12	113	7.3	4310	0.16	132	13.05	1813	-0.02	121	-1.12	9260
MEF2D	1.09	3.76E-09	0.08	142	7.3	4311	0.02	145	1.6	7428	0.09	133	7.47	4843
KEL	1.09	0.000436728	0.14	104	7.29	4312	0.05	111	2.86	6577	0.05	112	2.99	6931
UPRT	1.03	0.414054171	0.07	160	7.29	4313	0.08	153	8.38	3554	0.08	133	6.51	5229
TSC1	1.07	0.102463912	0.08	143	7.29	4314	0.09	136	8.05	3715	0.02	149	1.51	7741
LRP1B	1.12	0.001817158	0.09	130	7.29	4315	-0.03	119	-2.18	9947	0.06	141	5.17	5844
SHROOM4	1.01	0.327245881	0.07	161	7.29	4316	0.14	159	15.02	1272	0.02	139	1.49	7755
OXTR	1.2	0.000134509	0.11	119	7.28	4317	0.14	135	11.82	2182	0.16	123	11.95	3216
SEPT5_t1	1.08	2.77E-06	0.13	104	7.28	4318	NA	NA	NA	NA	NA	NA	NA	NA
ADA	1.17	0.009261587	0.13	106	7.28	4319	0.2	124	15.02	1270	0.03	137	2.86	7008
TMEM14B	1.27	2.79E-08	0.14	101	7.27	4320	0.03	107	1.58	7437	0.22	117	14.52	2455
ZBTB25	1.08	0.010559066	0.15	100	7.27	4321	0.08	88	3.22	6363	0.05	106	2.64	7109
SUV420H1_t1	1.06	0.000578691	0.1	124	7.27	4322	0	127	-0.08	8543	0.25	120	17.7	1698
SLC12A8	1.13	6.23E-05	0.11	119	7.27	4323	0.12	132	9.43	3093	0.07	119	4.66	6095
NAGPA	1.19	0.000102104	0.1	124	7.27	4324	0.11	110	6.44	4546	0.12	115	7.67	4763
OXLD1	1.49	3.43E-25	0.15	100	7.27	4325	0.19	123	14.01	1528	0.25	99	12.01	3194
VGLL2_t2	1.04	0.005508358	0.09	127	7.27	4326	-0.08	131	-6.42	12383	0.01	150	1.15	7946
NPL	-1.04	0.657312286	0.09	131	7.26	4327	0.05	129	3.57	6131	-0.05	121	-3.41	10358
DPH5_t2	1.13	0.000147683	0.09	129	7.26	4328	0.08	105	4.22	5769	0.16	103	8.5	4427
SSFA2	1.01	0.449851487	0.1	120	7.26	4329	0.07	118	4.45	5636	0.04	143	3.31	6772
SUFU_t1	1	0.814346132	0.08	143	7.26	4330	NA	NA	NA	NA	NA	NA	NA	NA
ALG14	1.26	1.36E-09	0.14	103	7.25	4331	0.1	114	6.58	4464	0.2	127	15.75	2142
PAX3	1.01	0.472319268	0.1	121	7.25	4332	-0.03	93	-1.44	9454	0	110	-0.14	8689
TRIM33_t1	1.04	0.391911527	0.08	145	7.25	4333	NA	NA	NA	NA	0.14	140	12.33	3097
ZNF319	1.08	0.003374992	0.11	114	7.25	4334	0.02	118	1.31	7599	0.07	123	5.42	5725
SRSF12	1.23	3.22E-06	0.08	144	7.24	4335	0.23	130	18.3	614	0.1	135	8.46	4440
ANO6_t1	1.07	0.000115263	0.09	130	7.24	4336	0.02	138	2	7145	NA	NA	NA	NA
ARPC2	1.15	0.001956165	0.08	138	7.24	4337	0.31	111	18.67	562	-0.01	141	-1.11	9256
FMNL3_t1	1.03	0.001465356	0.09	131	7.24	4338	NA	NA	NA	NA	NA	NA	NA	NA
PFDN2	1.31	2.58E-11	0.14	101	7.23	4339	0.28	106	15.62	1117	0.23	126	17.57	1722
SLC39A13	1.2	2.36E-07	0.11	118	7.23	4340	0.18	127	13.98	1536	0.08	112	4.82	6018
RAE1	1.19	1.29E-10	0.13	105	7.23	4341	0.05	104	2.65	6719	0.08	128	6.47	5254
MIEN1	1.37	3.87E-14	0.14	103	7.23	4342	0.13	130	10.29	2760	0.25	115	16.23	2026
TMEM74B	1.14	0.003775269	0.12	112	7.23	4343	0.04	89	1.6	7426	0.05	104	2.74	7063

MAL2	1.08	0.508432485	0.08	139	7.23	4344	0.09	130	7.58	3941	0.1	143	9.25	4134
ZDHHC2_t1	-1.02	0.728928034	0.09	129	7.23	4345	0.08	122	5.86	4864	NA	NA	NA	NA
IFNK	1.02	0.033873222	0.1	121	7.23	4346	-0.05	99	-2.54	10172	0.13	111	8.08	4591
RPL10_t1	1.07	0.112354898	0.1	119	7.22	4347	0.08	122	5.48	5066	NA	NA	NA	NA
SAAL1	1.22	5.01E-09	0.09	127	7.22	4348	0.06	95	2.72	6664	0.16	87	6.09	5406
HEXA	1.28	1.54E-07	0.11	117	7.22	4349	0.03	122	2.09	7085	0.11	122	8.08	4592
ZMAT2_t1	1.25	2.59E-08	0.1	119	7.22	4350	NA	NA	NA	NA	0.18	113	11.46	3366
MRPL18	1.24	1.16E-10	0.11	114	7.22	4351	0.15	109	9.03	3258	0.16	125	11.99	3204
MPZL2_t2	1.06	0.038740272	0.09	128	7.22	4352	0.09	125	6.45	4541	0.1	122	7.48	4841
ZNF628	1.17	6.37E-08	0.21	84	7.22	4353	0.06	91	2.63	6731	0.13	81	3.99	6417
PPP1R11	1.15	1.04E-06	0.1	120	7.21	4354	0	136	-0.11	8558	0.17	128	13.46	2751
NF2_t2	1.35	1.85E-07	0.11	118	7.21	4355	0.17	136	14.8	1328	0.15	142	14.17	2552
PAQR5_t1	1.24	4.82E-05	0.08	144	7.21	4356	0.13	145	12.57	1964	NA	NA	NA	NA
DUSP22_t1	1.06	0.081401526	0.12	108	7.21	4357	NA	NA	NA	NA	0.06	120	4.49	6190
TMEM41B	1.07	0.092725144	0.08	138	7.21	4358	-0.01	122	-0.42	8763	0.18	142	16.64	1918
AEBP2	-1.04	0.425021887	0.08	142	7.21	4359	0.13	128	9.8	2934	-0.01	138	-1.18	9281
SUGT1	1.02	0.693346376	0.1	119	7.2	4360	0.1	120	6.72	4388	0	148	0.22	8484
B3GNT5	1.4	0.000912386	0.06	164	7.19	4361	0.21	131	17.31	775	0.04	131	3.24	6808
CMTM1	1.16	1.51E-13	0.19	88	7.19	4362	0.1	105	5.74	4929	0.08	103	4.24	6324
NPLOC4	1.32	1.18E-15	0.09	126	7.19	4363	0.17	125	12.54	1970	0.15	130	12.33	3095
SH3BGRL2	1.01	0.792590183	0.08	143	7.18	4364	-0.03	130	-2.69	10268	0.02	131	1.3	7850
AMIGO1	1.07	1.17E-09	0.16	94	7.18	4365	0.08	114	5.25	5188	0.24	143	22.52	894
DAZ2_t2	1.02	0.024905441	0.09	127	7.18	4366	-0.04	133	-3.51	10805	0.07	139	6.21	5353
ALOX15B	1.13	0.021243794	0.13	104	7.18	4367	-0.07	128	-5.77	12078	-0.05	120	-3.37	10338
GUK1	1.13	0.001403807	0.21	84	7.18	4368	0.05	117	3.65	6089	0.17	104	9.04	4212
MLX	1.09	0.003934576	0.09	134	7.18	4369	0.01	120	0.63	8048	0.1	131	8.31	4490
COX8A	1.2	3.94E-08	0.12	108	7.17	4370	0.07	110	4.44	5644	0.15	104	8.22	4529
ANKRD50	1	0.998042892	0.09	130	7.17	4371	0.08	152	7.94	3776	0.06	144	5.91	5492
ZNF277	1.11	0.003846714	0.07	147	7.16	4372	0.07	120	5.12	5269	0.21	120	14.72	2402
CAMKV	1.03	0.080880062	0.1	122	7.16	4373	0.08	144	7.11	4189	0.15	115	9.93	3904
NKAIN1	1.05	0.000368944	0.09	133	7.16	4374	0.05	143	4.79	5452	0.06	120	4.05	6391
PTGFRN	1.08	0.005755094	0.09	132	7.16	4375	-0.05	127	-4.23	11224	0	149	-0.1	8674
YTHDF1	1.06	0.033236821	0.09	128	7.16	4376	0.09	131	7.24	4125	0.11	130	8.44	4448
IBSP	1.06	5.76E-05	0.13	105	7.16	4377	0.07	120	4.64	5528	0.06	127	4.65	6099
HSD17B1	1.41	2.10E-13	0.22	83	7.15	4378	0.07	100	3.3	6323	0.31	84	10.39	3727
GABRD	1.25	7.97E-19	0.17	91	7.15	4379	0.24	103	12.68	1932	0.28	116	18.72	1520
PNMA3	1.19	0.140045108	0.1	120	7.14	4380	-0.04	126	-3.13	10563	0.15	126	11.24	3432
SLC12A3_t1	1.05	0.023445654	0.1	123	7.14	4381	NA	NA	NA	NA	0.08	151	7.83	4699
SPTLC1_t2	1.08	0.096925874	0.08	136	7.14	4382	0.11	127	8.68	3418	0.14	141	12.83	2946
CADPS2	1.22	6.91E-07	0.1	124	7.13	4383	0.21	131	17.21	798	0.2	136	17.27	1771
ANKRD13B	1.06	2.25E-06	0.13	104	7.13	4384	0.11	121	7.89	3797	0.14	120	10.11	3836
PAX8_t1	1.33	9.23E-17	0.1	122	7.13	4385	0.12	161	13.3	1747	0.11	127	8.74	4326
TAF9B	1.15	1.68E-05	0.07	150	7.13	4386	0.02	131	1.78	7294	0.15	140	13.78	2651
CSF1_t1	1.11	0.000220562	0.08	140	7.13	4387	0.06	144	5.22	5210	0.11	146	10.32	3752
PAM_t1	-1.15	0.035482135	0.08	140	7.12	4388	NA	NA	NA	NA	-0.05	123	-3.32	10312
VPS41	1.09	0.017935979	0.1	120	7.12	4389	0.13	142	11.89	2156	0.16	150	16.36	1990
CENPV	1.15	0.014343738	0.11	112	7.12	4390	-0.04	106	-2.39	10079	0.21	120	14.49	2462
ZNF532	1.1	3.66E-05	0.09	127	7.11	4391	0.12	130	9.88	2902	0.23	137	19.8	1306
NDUFC2	1.12	0.022708941	0.1	124	7.11	4392	0.02	150	1.92	7201	0.15	124	10.74	3595
GBAS	1.09	0.043569048	0.11	113	7.11	4393	0.14	124	10.24	2781	0.07	120	4.76	6047
NDUFA2	1.24	2.25E-06	0.11	115	7.11	4394	-0.03	99	-1.66	9591	0.23	122	16.39	1986
DCTN3	1.19	4.11E-08	0.1	119	7.11	4395	-0.04	118	-2.95	10450	0.17	116	10.97	3519
STX3	-1	0.949352243	0.07	153	7.11	4396	0.08	143	7.85	3806	0.01	151	1.05	7989
PIK3R2	1.2	3.41E-11	0.09	131	7.11	4397	0.21	127	15.97	1054	0.11	132	9.26	4129
NGLY1	1.07	0.116575449	0.12	110	7.11	4398	0.07	112	4.61	5544	0.13	108	7.36	4888
PRKCG	1.12	8.31E-06	0.1	120	7.1	4399	-0.04	126	-3.18	10593	0.12	106	6.49	5238
SLC7A13	1.03	0.001389165	0.1	120	7.1	4400	0.04	123	2.81	6607	0.06	103	3.42	6701
DUSP11	1	0.842304631	0.09	126	7.1	4401	0.01	104	0.54	8112	-0.05	136	-4.48	10833
NT5C3	1.25	5.54E-08	0.11	113	7.09	4402	0.2	149	19.77	423	0.22	118	14.78	2388
EMC10	-1.02	0.652117551	0.1	120	7.08	4403	-0.03	125	-2.58	10206	0	124	-0.32	8803
TIMM17B	1.15	4.31E-07	0.12	108	7.08	4404	0.05	135	4.12	5828	0.11	110	6.36	5296
ZNF12	1.15	0.00943081	0.06	167	7.08	4405	0.14	154	14.06	1516	0.16	152	16.56	1940
MDGA1	1.15	0.001791863	0.09	132	7.08	4406	0.01	130	0.73	7982	0.06	131	5.06	5896
EIF2B5	1.08	0.001244676	0.11	113	7.08	4407	-0.01	123	-0.96	9118	-0.01	116	-0.42	8864
SPICE1_t1	1.05	0.034521665	0.08	141	7.08	4408	NA	NA	NA	NA	0.09	141	7.9	4663
PSORS1C1	1.05	3.55E-05	0.13	104	7.07	4409	0.05	100	2.61	6751	0.11	103	5.86	5520
CNOT6L_t1	-1.02	0.047876418	0.07	145	7.07	4410	NA	NA	NA	NA	0.04	139	3.94	6442
RPS2	1.62	5.08E-12	0.27	76	7.07	4411	0.34	78	9.48	3076	0.28	72	6.1	5404
SLC4A2	1.2	4.80E-09	0.14	102	7.07	4412	0.07	88	2.7	6676	0.21	88	8.05	4599
EHD4	1.24	3.63E-06	0.11	112	7.07	4413	0.07	115	4.33	5700	0.17	120	12	3197
NEK11_t1	1.03	0.100876344	0.11	116	7.06	4414	0.04	108	2.19	7015	0	118	0.01	8614
AXDND1_t1	1.16	1.20E-05	0.11	114	7.06	4415	NA	NA	NA	NA	NA	NA	NA	NA
MAPKAPK3	1.2	6.83E-06	0.08	142	7.06	4416	0.07	121	5.07	5285	0.08	126	6.09	5407
AGTRAP	1.06	0.000755852	0.11	116	7.06	4417	0.06	164	7.39	4040	0.04	115	2.36	7260
NAA16_t2	1	0.91536157	0.08	138	7.06	4418	NA	NA	NA	NA	0.03	132	2.75	7053
CHFR	1.03	0.48867576	0.07	153	7.06	4419	0.21	114	13.4	1714	-0.06	119	-4.06	10663
CDK5RAP2	1.26	1.79E-09	0.11	115	7.06	4420	0.08	106	4.27	5731	0.24	130	19.44	1359
GSTM4	-1.08	0.211133509	0.09	129	7.05	4421	0	145	0.23	8306	0.06	120	4.3	6286
CCR8	1.04	5.32E-05	0.11	113	7.05	4422	0.12	122	8.84	3361	0.12	103	6.31	5317
BMP2K_t1	-1.01	0.668229764	0.13	104	7.05	4423	0.12	108	6.92	4291	0.04	107	2.2	7352
ZNF182	1.07	0.117022924	0.11	114	7.05	4424	0.07	117	4.82	5436	0.16	115	10.63	3642
ALG1L_t1	1.71	8.61E-20	0.25	78	7.05	4425	NA	NA	NA	NA	0.34	75	8.49	4430
SNAP29	1.12	0.000144507	0.1	124	7.05	4426	0.04	144	3.98	5918	0.12	150	11.5	3348
TUBA4A	1.48	3.99E-15	0.16	95	7.05	4427	0.23	94	9.93	2884	0.21	124	15.33	2238
Sep-07	1.09	0.003872332	0.09	125	7.05	4428	0.21	143	19.3	478	0.21	142	19.72	1319
TBX5	1.02	0.017274926	0.1	121	7.04	4429	0.09	137	7.54	3969	0.14	130	11.03	3506
RNF219	1.08	0.07410436	0.1	124	7.04	4430	0.2	121	13.87	1572	0.05	150	4.51	6174
GIN1	1.04	0.270225062	0.08	136	7.04	4431	0	134	0.12	8387	0.12	118	7.95	4643
USP33_t2	1	0.913767275	0.12	109	7.04	4432	0.09	78	2.59	6762	0.08	105	4.5	6180
BCL7A	1.22	3.79E-07	0.11	114	7.03	4433	0.25	130	20.2	377	0.13	128	10.16	3808
MAF1	1.04	0.300719848	0.14	102	7.03	4434	0.02	146	1.85	7245	0.04	131	3.11	6867
SYNJ1	-1.02	0.372182204	0.09	128	7.03	4435	0.06	131	4.51	5610	0.12	134	9.96	3890
RPRD2	1.18	3.66E-07	0.07	147	7.03	4436	0.13	121	9.33	3133	0.17	151	16.93	1845
E4F1	1.21	2.63E-08	0.12	107	7.03	4437	0.12	91	5.12	5268	0.06	103	3.06	6899
HOXC11	1.04	0.000371462	0.1	117	7.02	4438	0.12	139	10.68	2603	0.14	123	10.15	3814
ME1_t1	1.08	0.416900226	0.07	146	7.02	4439	0.17	118	11.54	2289	NA	NA	NA	NA
NUP85	1.29	1.70E-13	0.15	96	7.02	4440	0.14	72	3.12	6428	0.14	103	7.64	4777
EIF4ENIF1	1.18	1.78E-06	0.12	107	7.02	4441	0.17	120	12.15	2085	0.07	126	5.46	5708
POGLUT1	1.04	0.234119352	0.08	143	7.02	4442	0.04	146	4.19	5784	0	136	0.17	8505
TRIM62	1.16	3.23E-09	0.09	126	7.01	4443	0.12	129	9.43	3094	0.08	130	6.38	5282
ETV7	1.12	0.017306997	0.1	118	7.01	4444	-0.02	119	-1.34	9392	-0.04	100	-2.17	9783
FBXO5	1.27	2.79E-08	0.12	109	7	4445	0.15	110	9.23	3178	0.15	120	10.53	3677
VASH2	1.14	1.41E-07	0.08	140	7	4446	-0.03	139	-2.37	10059	0.11	126	8.26	4510
ELOVL5	1.12	4.47E-08	0.09	129	7	4447	0.12	123	8.98	3285	0.18	144	16.9	1854
VN1R1	1.09	0.000116534	0.11	114	7	4448	0.14	127	10.49	2680	0.14	97	6.52	5227
CDV3	1.09	0.004458771	0.09	132	6.99	4449	0.08	123	5.56	5034	0.03	146	2.96	6957

HOXB8	1.03	0.118830518	0.09	125	6.98	4450	0.01	96	0.51	8131	0.23	111	14.18	2550
UBC	1.3	9.75E-07	0.23	80	6.98	4451	0.24	140	21.21	267	0.21	152	21.48	1054
ZNF687	1.22	2.61E-09	0.09	129	6.98	4452	0.07	126	5.62	4998	0.09	117	5.7	5578
NIP7	1.02	0.642621386	0.07	154	6.98	4453	-0.02	140	-1.54	9511	0.01	153	0.93	8060
ZBED2	1.22	1.98E-06	0.13	104	6.97	4454	0.15	114	9.88	2905	0.07	128	5.5	5681
ADRM1	1.08	0.000565152	0.14	101	6.97	4455	-0.04	109	-2.09	9891	0.06	135	4.9	5974
PKN1	1.09	0.0052042	0.14	100	6.97	4456	0.05	88	2.09	7087	0.07	104	3.64	6595
NCL	1.1	0.06370597	0.1	121	6.97	4457	0.23	126	17.69	718	0.07	123	5.4	5730
ANO2	1.3	1.24E-05	0.11	113	6.96	4458	0.14	126	10.8	2555	0.2	124	15.13	2297
FAM110B	1.14	0.024789444	0.08	141	6.96	4459	0.02	135	1.95	7174	0.17	135	14.18	2549
TSPAN14	1.02	0.264988685	0.08	132	6.96	4460	0.07	139	6.34	4597	-0.07	130	-5.73	11329
ADSS_t1	1.1	0.01730555	0.1	123	6.96	4461	NA	NA	NA	NA	0.1	124	7.33	4905
TXNL1	-1.02	0.615303469	0.07	147	6.96	4462	0.1	109	5.77	4913	0.01	119	0.98	8030
PON2	1.56	4.38E-07	0.12	106	6.96	4463	0.22	107	12.7	1927	0.27	139	23.62	773
PIP5K1C	1.05	0.034247801	0.08	139	6.95	4464	0.04	145	3.55	6142	0	122	0.22	8480
BUD13	1.05	0.04668425	0.11	112	6.95	4465	0.07	93	3.21	6377	0.01	112	0.73	8174
CDON_t1	1.05	0.119658711	0.1	122	6.94	4466	0.1	136	8.76	3386	NA	NA	NA	NA
PAQR8	-1.05	0.356668256	0.06	164	6.94	4467	0.18	125	13.35	1731	-0.18	136	-15.73	14527
ZC3H15	1.03	0.599039779	0.11	112	6.94	4468	0.17	106	9.64	3005	0.12	112	7.74	4739
MRPS16	1.21	1.32E-06	0.08	141	6.94	4469	0.03	120	2.16	7035	0.12	134	10.14	3820
ZFP112	1.12	0.013568495	0.09	127	6.94	4470	0.14	115	8.8	3376	0.05	130	4.29	6295
HIST1H4E	1.3	5.24E-13	0.23	80	6.94	4471	0.13	94	5.86	4867	0.2	70	4	6414
GTPBP6	1.11	0.017979088	0.13	104	6.94	4472	0.06	120	4.07	5863	0.1	102	5.35	5756
FAM120AOS	1.12	0.000102104	0.09	126	6.94	4473	0.12	132	9.75	2956	0.08	121	6.02	5445
TMEM138	1.22	5.66E-09	0.08	142	6.94	4474	0.17	128	13.21	1770	0.14	120	9.71	3979
MRPL17	1.12	0.00056873	0.09	130	6.94	4475	0.07	129	5.67	4961	-0.04	127	-3.46	10379
PSTK	1.1	0.029822548	0.1	120	6.93	4476	-0.05	116	-3.26	10641	0.16	125	11.82	3256
MRI1	1	0.922915104	0.09	129	6.93	4477	-0.03	136	-2.79	10333	0.01	148	1.46	7778
SERPINB4	1.11	0.002375841	0.12	106	6.93	4478	0	125	0.35	8241	0.19	112	11.54	3340
OSR1	1.07	1.04E-05	0.11	115	6.93	4479	0.09	114	5.81	4889	0.09	120	6.23	5343
WDR96	1.11	4.86E-11	0.1	120	6.93	4480	0.16	88	6.23	4647	0.18	117	12.38	3078
KLHL11	1.05	2.10E-05	0.1	116	6.93	4481	0.08	108	4.58	5569	0.15	103	8.17	4552
CYB5B	1.02	0.631606278	0.08	139	6.92	4482	-0.1	135	-8.57	13347	-0.03	148	-3.15	10247
CRAMP1L	1.03	0.06881169	0.09	124	6.91	4483	0.12	146	11.91	2151	0.01	127	0.61	8253
TMED7	1.09	0.00530979	0.09	125	6.91	4484	-0.01	124	-0.45	8785	0.18	138	15.49	2197
TAPBP_t1	1.15	0.000312327	0.09	124	6.91	4485	-0.03	134	-2.76	10320	0.05	136	3.94	6448
LCT	1.03	0.00179026	0.1	119	6.91	4486	-0.05	111	-3.17	10586	0.07	119	5.1	5879
FUK	1.18	2.32E-06	0.12	106	6.91	4487	0.08	127	6.1	4726	0.09	120	6.5	5236
DFNA5	1.38	0.000124307	0.09	130	6.9	4488	0.18	122	13.02	1820	0.17	143	15.73	2147
DNTTIP2	1.04	0.50667133	0.11	115	6.9	4489	0.11	117	7.27	4112	0.1	117	6.73	5138
SLC6A15_t1	1.02	0.063432342	0.08	140	6.9	4490	NA	NA	NA	NA	0.07	117	4.45	6217
RAB8B	1.04	0.33834009	0.08	141	6.9	4491	0.09	129	6.77	4365	0.03	151	2.96	6956
THAP9	1.01	0.661873609	0.07	152	6.89	4492	0.05	127	3.86	5974	0.16	128	12.75	2961
ERCC2	1.15	7.06E-06	0.15	95	6.88	4493	0.07	118	4.85	5429	0.03	120	1.78	7579
CDK7	1.22	1.82E-06	0.1	119	6.88	4494	0.11	109	6.55	4480	0.22	112	13.33	2786
QRFPR	1.02	0.221350658	0.09	127	6.88	4495	0.04	131	3.47	6204	0.03	121	2.19	7354
ORC5_t2	1.14	6.55E-05	0.12	107	6.88	4496	0.14	87	5.24	5194	0.27	115	17.5	1737
MPHOSPH8	-1.05	0.25878822	0.07	144	6.88	4497	0.01	138	1.12	7737	-0.02	127	-1.49	9441
CEP89_t1	1.27	3.29E-17	0.23	80	6.88	4498	NA	NA	NA	NA	NA	NA	NA	NA
LAPTM4A	1.08	0.053955895	0.08	134	6.87	4499	0.07	118	4.66	5515	0.06	137	5.31	5770
RAB2A	1.22	2.33E-05	0.08	140	6.87	4500	0.19	133	15.56	1129	0.22	129	17.1	1809
RPL27A	1.19	0.000211777	0.09	126	6.87	4501	0.14	135	12.31	2031	0.13	153	13.26	2812
RPL35A	1.21	2.57E-05	0.16	92	6.87	4502	0.21	86	7.63	3914	0.15	117	10.01	3874
NEMF	-1.03	0.659816915	0.08	141	6.87	4503	0.04	111	2.27	6976	0.11	114	7.15	4969
CPXCR1	1.05	4.55E-05	0.1	117	6.87	4504	0.06	114	3.89	5956	0.19	116	12.21	3132
SMURF2	1.08	0.00088483	0.08	133	6.87	4505	0.14	142	12.49	1983	0.13	133	11.08	3490
CHD5	1.03	0.017658742	0.09	126	6.87	4506	-0.06	159	-6.05	12211	-0.02	143	-1.88	9656
ERBB4	1.02	0.418439464	0.07	145	6.86	4507	-0.01	137	-0.99	9137	-0.08	147	-7.75	12140
RNF181	1.19	3.05E-08	0.12	108	6.86	4508	0.13	99	6.51	4504	0.08	107	4.8	6030
FAM175B	1	0.958143683	0.08	131	6.85	4509	-0.07	120	-4.97	11643	0.17	145	15.81	2130
TCFL5	1.31	5.00E-09	0.09	128	6.85	4510	0.13	124	9.82	2927	0.19	129	14.84	2376
DUOX1	1.17	1.35E-06	0.14	98	6.85	4511	0.08	122	5.86	4865	0.09	103	4.89	5978
AKD1	1.09	6.12E-08	0.09	127	6.85	4512	0.17	131	13.51	1679	0.25	133	20.67	1173
ZNF20	1.05	0.006464698	0.11	115	6.85	4513	0.04	131	3.63	6097	0.02	123	1.64	7655
ZSWIM3	1.13	1.62E-06	0.09	126	6.84	4514	-0.02	134	-1.94	9771	0.14	109	8.01	4614
TH1L	1.3	2.99E-12	0.14	100	6.84	4515	0.06	124	4.08	5854	0.2	107	11.62	3319
TTTY7	1.02	0.008581125	0.11	113	6.84	4516	0.06	143	5.65	4980	0.11	127	8.69	4346
PPEF1	1.11	3.49E-09	0.13	104	6.83	4517	0.11	118	7.3	4089	0.23	96	10.41	3721
KLF17	1.04	5.44E-05	0.08	140	6.83	4518	0.09	142	8.62	3449	0.16	156	16.52	1950
ABCF1	1.23	1.68E-13	0.1	121	6.83	4519	0.1	110	6.3	4622	0.19	109	11.25	3429
SOHLH2	1.25	2.78E-05	0.08	131	6.82	4520	0.09	138	7.86	3805	0.07	136	6.17	5371
KCNQ3	1.04	0.010737148	0.07	144	6.82	4521	-0.06	140	-5.51	11925	0.07	143	6.78	5114
DGKB_t1	1.08	0.000154058	0.07	146	6.82	4522	0.18	139	15.62	1115	0.24	127	18.35	1581
ACOT9	1.07	0.096371198	0.13	104	6.81	4523	0.17	110	9.99	2864	0.1	118	6.87	5080
NOA1	1.1	0.001349076	0.12	106	6.81	4524	0.16	126	12.16	2081	0.13	119	9.03	4219
GPR3	1.03	0.00039455	0.09	127	6.81	4525	0.01	125	0.46	8159	0.02	118	1.63	7659
DYRK1A_t1	1.01	0.129335073	0.09	128	6.81	4526	0.03	142	3.06	6464	0.04	128	3.15	6854
ZFYVE9_t2	1.04	4.05E-05	0.15	95	6.8	4527	0.06	104	2.99	6504	0.24	114	15.29	2249
TARDBP	1.2	4.96E-06	0.08	131	6.8	4528	0.22	138	19.69	435	0.15	169	17.75	1690
TTTY11	1.03	0.002138376	0.09	123	6.78	4530	-0.02	111	-1.18	9274	0.09	120	5.95	5471
SAMD13	1.14	3.14E-05	0.07	145	6.78	4531	0.21	158	23.05	147	0.1	119	6.93	5060
RIC8B	1	0.91048759	0.09	122	6.78	4532	-0.03	130	-2.74	10306	0.09	147	8.66	4364
CTLA4	1.05	0.000603601	0.08	132	6.78	4533	0.11	130	8.92	3320	0	122	-0.24	8758
TARBP2	1.19	1.98E-08	0.12	108	6.78	4534	0.06	107	3.52	6169	0.07	100	3.41	6711
XRN1_t1	-1.02	0.556154344	0.07	148	6.78	4535	0.04	135	3.24	6351	0.07	148	6.62	5186
XPNPEP3	1.11	2.49E-09	0.07	147	6.78	4536	0.1	131	7.77	3848	0.11	146	10.83	3565
MFSD11	1.23	1.47E-07	0.11	112	6.77	4537	0.15	139	13.21	1771	0.12	153	12.07	3172
PCDHB15	-1.03	0.510419358	0.1	120	6.77	4538	0.04	104	2.17	7026	-0.04	124	-3.25	10293
DTX2	1.07	2.54E-06	0.13	103	6.77	4539	0.05	102	2.73	6659	0.1	136	8.56	4403
SCN1A	1.03	0.055670922	0.09	124	6.77	4540	-0.04	126	-3.15	10570	0.14	124	10.04	3863
ERVMER34-1	1.06	0.040802672	0.14	99	6.77	4541	0.03	119	2.11	7071	0.22	121	15.75	2143
ATPBD4	1.24	2.48E-07	0.09	124	6.76	4542	0.15	119	10.56	2647	0.26	127	20.04	1270
SMUG1	1.15	0.00037204	0.1	121	6.76	4543	-0.03	128	-1.98	9801	0.15	143	13.68	2681
COX6A1	1.2	0.000150092	0.08	135	6.76	4544	0.04	99	1.9	7214	0.2	109	11.51	3346
PGBD2	1.08	0.008068476	0.08	135	6.76	4545	0	123	0.34	8247	0.09	131	7.18	4957
SMTNL2	1.03	0.010420293	0.11	113	6.76	4546	0.05	133	4.39	5666	0.11	110	6.71	5149
UNC119	1.15	0.000532236	0.08	135	6.76	4547	0.17	108	9.74	2960	0.01	113	0.63	8240
VMAC	1.11	1.54E-08	0.11	113	6.75	4548	0.15	127	11.79	2191	0.07	115	4.65	6100
DUS2L	1.08	0.005824225	0.14	100	6.75	4549	-0.08	111	-4.79	11534	0.09	109	5.6	5619
CDK6	1.04	0.736985454	0.07	147	6.75	4550	0.08	133	6.89	4309	0.04	146	4.28	6298
PSG8	1.12	8.40E-15	0.2	83	6.74	4551	0.11	92	4.52	5605	0.18	87	6.48	5249
PCBP1	1.07	0.001261678	0.08	131	6.74	4552	0.09	120	6.33	4600	-0.01	101	-0.37	8828
HIP1_t1	1.2	8.79E-08	0.07	151	6.74	4553	NA	NA	NA	NA	0.14	135	11.92	3228
TGIF2LY	1.1	6.06E-07	0.12	104	6.74	4554	0.02	113	1.54	7470	0.2	104	11.06	3492
CHAC1	1.08	0.031962046	0.07	148	6.73	4555	-0.11	120	-7.69	12980	0.14	137	11.79	3267
APPL2	1.03	0.318637182	0.09	126	6.73	4556	0.01	123	0.54	8110	0.04	143	3.72	6558

AFAP1L1	1.07	0.130254641	0.1	120	6.73	4557	0.1	139	8.67	3431	0.12	145	11.21	3441
AKTIP	-1	0.959950689	0.09	125	6.73	4558	-0.02	115	-1.15	9254	0.09	131	7.17	4963
NPC2	1.1	0.046733971	0.11	110	6.73	4559	0.12	119	8.51	3493	-0.04	115	-2.9	10133
NOL11	1.2	6.06E-05	0.11	114	6.73	4560	0.11	118	7.17	4154	0.23	101	11.52	3344
SLC35B1	1.24	4.15E-08	0.13	100	6.73	4561	0	95	0.22	8319	0.18	99	8.92	4256
DDIT3	1.44	4.23E-08	0.12	104	6.73	4562	0.07	122	4.69	5502	0.14	116	9.36	4095
KIAA1429_t2	1.13	8.09E-08	0.22	80	6.72	4563	0.16	108	9.16	3205	0.31	79	8.92	4253
SLC5A8	1	0.920747978	0.07	142	6.72	4564	0.02	126	1.53	7477	0.05	127	4.22	6330
SPINK6	1.06	4.84E-05	0.11	112	6.72	4565	-0.03	116	-1.86	9736	0.17	103	8.84	4284
PLEC_t1	1.03	0.014845927	0.16	93	6.72	4566	-0.04	125	-3.24	10623	0.16	131	12.64	2995
RPL37_t1	1.22	1.60E-06	0.07	141	6.72	4567	0.13	109	7.54	3970	NA	NA	NA	NA
FGGY_t1	-1.09	0.4881749	0.12	104	6.71	4568	NA	NA	NA	NA	0.06	148	5.68	5587
CST1	1.12	0.005399545	0.12	104	6.71	4569	-0.01	126	-0.97	9122	-0.06	102	-3.23	10281
DLL4	1.12	5.73E-09	0.12	106	6.71	4570	0.14	114	9.06	3250	0.19	136	16.59	1934
MCM5	1.45	1.60E-14	0.16	93	6.71	4571	0.05	124	3.97	5920	0.18	99	8.67	4356
UBAC1	1.18	2.08E-07	0.08	132	6.71	4572	0.06	116	4.12	5836	0.11	110	6.39	5279
HIST3H2A	1.65	2.41E-15	0.2	84	6.7	4573	0.09	76	2.21	7007	0.17	72	3.69	6574
TMEM86A	-1.01	0.610388386	0.05	174	6.7	4574	0.08	153	8.31	3588	-0.11	128	-8.51	12432
CBR3	1.32	2.60E-06	0.15	94	6.7	4575	0.12	72	2.59	6768	0.09	97	4.26	6311
EPRS	1.44	1.07E-06	0.22	80	6.69	4576	0.28	114	18.04	657	0.3	105	16.69	1906
NUP210	1.1	0.032497146	0.06	153	6.69	4577	0.09	120	6.63	4433	0.02	132	1.68	7634
MAPKAP1	1.11	0.000169439	0.08	133	6.69	4578	0.09	131	7.55	3962	0.08	125	5.99	5455
NT5C3L	1.21	1.77E-07	0.12	104	6.69	4579	0.03	108	1.93	7191	0.22	107	12.46	3046
DCHS2	1.02	0.014895321	0.17	90	6.69	4580	0.11	101	5.66	4970	0.1	102	5.42	5724
KANSL1	1.06	0.040134387	0.09	128	6.68	4581	0.08	137	7.01	4238	0.09	130	6.85	5085
TIRAP_t1	1.2	5.17E-15	0.18	88	6.68	4582	0.24	78	6.61	4442	0.2	86	7.34	4899
SPRR2D	1.05	0.000451292	0.1	116	6.68	4583	-0.05	122	-3.37	10720	0.1	124	7.4	4872
RC3H1	1.09	0.000726604	0.07	145	6.68	4584	0.1	146	9.29	3148	0.04	140	3.57	6629
F13A1	1.44	0.000258761	0.1	118	6.68	4585	0.1	132	8.6	3456	0.06	150	6.04	5435
VWF	1.73	6.74E-07	0.17	89	6.68	4586	0.14	92	5.76	4915	0.22	115	14.27	2532
DEFB119	1.07	2.63E-08	0.12	104	6.67	4587	0.01	115	0.52	8127	0.09	107	5.19	5832
ANKRD17_t1	1	0.910513455	0.08	136	6.67	4588	NA	NA	NA	NA	0.13	127	10.05	3860
CABIN1_t1	1.17	9.23E-07	0.1	116	6.67	4589	NA	NA	NA	NA	0.12	115	7.86	4682
GNA13_t1	-1.09	0.190380795	0.07	151	6.67	4590	NA	NA	NA	NA	NA	NA	NA	NA
CHMP2B	1.02	0.612238854	0.09	127	6.66	4591	0.07	127	5.65	4975	0.2	121	14.45	2475
SLC10A2	1.04	0.000106431	0.08	131	6.66	4592	0.04	132	3.1	6438	0.14	135	11.56	3337
LRTOMT_t1	1.06	0.115494376	0.11	112	6.65	4593	NA	NA	NA	NA	NA	NA	NA	NA
TM9SF3	1.03	0.530433603	0.08	136	6.65	4594	-0.01	139	-1.11	9222	0.16	135	13.22	2823
ZNF45	1.13	1.88E-05	0.07	141	6.65	4595	0.04	110	2.11	7068	0.12	148	11.39	3388
HNF1A	1.18	0.007698002	0.08	132	6.64	4596	0.02	134	1.82	7263	0.11	134	9.49	4056
HS1BP3_t1	1.09	0.008726363	0.1	115	6.64	4597	NA	NA	NA	NA	0.05	119	3.16	6853
TMF1	1.03	0.437373042	0.07	141	6.64	4598	0.14	133	11.53	2296	0.14	140	12.2	3138
CENPI	1.39	2.41E-18	0.22	80	6.64	4599	0.22	76	5.65	4976	0.35	82	11.29	3416
APOO	1.22	2.00E-06	0.15	95	6.64	4600	0.03	123	2.48	6836	0.17	107	9.55	4039
KRTAP4-1	1.09	0.000725625	0.08	130	6.63	4601	0.05	100	2.43	6872	-0.05	121	-3.49	10391
RASSF6	1.02	0.339922796	0.08	134	6.63	4602	0.02	129	1.36	7570	0.07	140	6.36	5293
SAG	1.06	7.71E-07	0.15	95	6.62	4603	0.14	90	5.7	4950	0.15	102	7.9	4662
IQCH	1.18	3.16E-06	0.1	114	6.62	4604	0.05	109	3.17	6403	0.18	112	10.89	3556
SMO	1.37	4.77E-05	0.09	120	6.62	4605	-0.02	131	-1.85	9726	0.17	134	14.1	2572
TMEM115	1.12	1.85E-05	0.08	128	6.62	4606	0.03	136	2.34	6934	0.12	107	6.86	5081
RAD17	1.23	1.42E-06	0.09	124	6.62	4607	0.07	123	5.39	5128	0.25	119	17.48	1740
HSD11B2	1.15	0.006814187	0.11	108	6.61	4608	-0.07	122	-4.91	11602	0.18	104	9.88	3920
SPC24	1.84	1.06E-25	0.22	80	6.61	4609	0.2	72	4.32	5710	0.34	120	23.57	779
NSFL1C_t1	1.08	0.01329318	0.08	138	6.61	4610	NA	NA	NA	NA	NA	NA	NA	NA
NSDHL	1.2	2.19E-05	0.08	136	6.6	4611	0.02	120	1.42	7548	0.11	126	8.51	4421
GDF11	1.1	0.003861103	0.09	127	6.6	4612	0.1	137	8.88	3340	-0.03	116	-2.16	9778
CCNF	1.09	2.56E-09	0.09	120	6.6	4613	0.15	139	13.31	1746	0.2	123	14.48	2467
BLVRA	1.29	0.001187697	0.17	88	6.59	4614	0.25	120	17.66	724	0.04	121	3.03	6911
SLC25A14	1.3	1.24E-11	0.12	104	6.59	4615	0.15	107	8.68	3422	0.25	127	19.12	1428
VPS25	1.16	7.46E-07	0.08	134	6.59	4616	-0.04	125	-2.86	10387	0.15	100	7.49	4835
HYAL4	1.07	0.009100401	0.1	118	6.59	4617	0.03	146	3.35	6284	0.23	141	21.03	1115
NFYB	1.09	0.037628439	0.08	137	6.59	4618	-0.01	131	-1.05	9183	0.15	131	12.2	3135
ANKS1B_t1	1.03	0.157464219	0.1	119	6.59	4619	-0.02	119	-1.64	9583	0.03	112	1.63	7660
DMC1	1.05	0.000643326	0.08	136	6.59	4620	-0.02	136	-2.09	9893	0.13	120	9.44	4069
TBPL1	1.28	7.49E-09	0.09	123	6.58	4621	0.14	110	8.55	3475	0.2	129	15.67	2162
NFXL1	1.18	1.68E-06	0.09	124	6.58	4622	0.16	118	10.58	2635	0.16	137	13.64	2699
NIN_t2	1.23	4.60E-06	0.21	82	6.58	4623	0.2	86	7.31	4085	0.19	83	6.39	5277
ARMC7	1.33	2.59E-12	0.13	101	6.58	4624	0.13	115	8.5	3500	0.13	120	9.34	4103
AFTPH_t1	1.09	0.045467229	0.07	139	6.58	4625	0.04	110	2.35	6923	0.19	154	19.5	1348
HLA-E	1.14	0.069691184	0.09	120	6.58	4626	0.05	129	4.33	5698	-0.01	120	-0.89	9135
SLC39A7	1.11	0.041386172	0.1	117	6.57	4627	-0.02	103	-0.82	9028	0.11	134	9.22	4155
FIG4	1.03	0.191195643	0.1	113	6.57	4628	0.1	107	5.47	5074	0.06	121	4.3	6289
TBXA2R_t1	1.15	2.85E-09	0.08	128	6.57	4629	-0.04	92	-1.68	9602	0.03	104	1.64	7654
CHIT1	1.1	5.48E-05	0.13	100	6.57	4630	0.1	116	6.47	4528	0.04	130	3.02	6918
MED29_t1	1.13	6.19E-07	0.09	122	6.57	4631	NA	NA	NA	NA	0.05	148	5.23	5816
SPEF2_t1	1.29	5.84E-07	0.07	139	6.57	4632	0.15	135	12.68	1933	0.26	133	21.56	1038
Mar-08	1.06	0.000289485	0.13	102	6.57	4633	0.01	133	0.56	8098	0.18	117	12.18	3144
LAMTOR2_t1	1.34	4.55E-10	0.17	88	6.57	4634	NA	NA	NA	NA	0.22	85	7.7	4755
NDUFAF4	-1.04	0.471648367	0.08	131	6.57	4635	0.02	139	1.79	7280	0.05	112	3.21	6827
DNAH9	1.06	5.13E-06	0.12	104	6.57	4636	0.08	116	5.44	5088	0.19	100	9.44	4071
ATF7	1.04	7.14E-05	0.06	155	6.57	4637	0.11	133	8.76	3389	0.11	166	12.37	3082
HIST1H2BH	1.64	2.15E-14	0.19	84	6.57	4638	0.09	84	3.03	6480	0.19	80	5.82	5535
GTF2H2D	1.19	1.89E-08	0.09	125	6.56	4639	0.11	106	5.91	4836	0.21	116	13.65	2694
NAT8L	1.16	7.26E-05	0.08	128	6.56	4640	0.2	141	17.77	705	0.06	125	4.45	6216
PTPN23	1.07	2.01E-07	0.15	95	6.56	4641	0.18	119	12.7	1926	0.22	115	14.1	2574
KDM4A	1.17	9.80E-06	0.08	130	6.56	4642	0.01	130	0.92	7862	0.09	134	7.66	4767
DDX11	1.21	1.43E-11	0.09	120	6.56	4643	0.15	127	11.24	2387	0.08	105	4.44	6224
CNNM2_t1	1.15	2.48E-08	0.08	133	6.55	4644	0.03	110	2.02	7130	0.19	100	9.66	3997
GTF2H3	1.1	0.000104743	0.07	144	6.55	4645	0.17	139	14.83	1321	0.06	152	6.09	5408
TUBB4B	1.44	1.09E-13	0.12	106	6.55	4646	0.21	121	14.7	1351	0.17	120	11.67	3302
EIF2B3	1.01	0.698852128	0.09	126	6.55	4647	-0.06	123	-4.25	11244	0.04	111	2.16	7364
MAGEA10	1.14	0.000389322	0.09	126	6.54	4648	0.08	127	6.17	4683	0.19	131	15.69	2156
SCYL3	1.12	0.005208642	0.11	110	6.54	4649	0.08	123	5.64	4985	0.11	122	8.27	4505
PPHLN1_t3	1.07	1.52E-06	0.08	136	6.54	4650	0.14	147	13.33	1739	0.16	130	13.15	2845
RPS26_t1	1.03	0.384114537	0.11	108	6.54	4651	NA	NA	NA	NA	NA	NA	NA	NA
CCNB3	1.21	2.82E-09	0.08	133	6.54	4652	0.12	112	7.17	4156	0.22	99	10.91	3545
SMAGP	1.14	0.001257992	0.1	118	6.54	4653	0.09	117	6.05	4749	0.09	102	4.51	6175
SPHAR	1.22	0.000344251	0.13	100	6.53	4654	0.02	112	1.14	7723	0.18	104	9.98	3887
SLAMF8	1.09	0.198546712	0.08	127	6.53	4655	0.16	139	14.68	1356	-0.05	136	-4.69	10937
CFHR5	-1.45	0.005921692	0.08	131	6.52	4656	-0.12	120	-8.48	13318	0.01	133	1.02	8009
SFPQ	-1.02	0.598753227	0.06	164	6.52	4657	0.04	119	2.45	6854	-0.07	145	-6.7	11732
ARIH2_t1	1.12	9.36E-06	0.08	130	6.52	4658	NA	NA	NA	NA	NA	NA	NA	NA
TM4SF20	1.16	0.000229347	0.09	121	6.52	4659	0	126	0.19	8342	0.01	139	1.1	7966
CCDC24_t1	1.14	1.38E-08	0.16	91	6.52	4660	NA	NA	NA	NA	0.2	102	10.64	3637
AFF4_t1	1.01	0.854450003	0.07	144	6.51	4661	NA	NA	NA	NA	NA	NA	NA	NA
WRNIP1	1.12	0.000124075	0.11	110	6.51	4662	0.07	113	4.3	5717	0.1	146	9.64	4008

ZDHHC23	1.18	0.001261807	0.07	142	6.51	4663	-0.05	116	-3.11	10548	0.16	143	14.89	2363
TLX2	1.1	7.37E-09	0.11	107	6.51	4664	0.01	120	0.79	7945	0.12	124	9.2	4157
MTL5_t2	1.41	4.21E-07	0.06	151	6.51	4665	0.14	113	8.55	3478	0.15	120	10.61	3651
RIMS4	1.03	0.018586918	0.09	124	6.51	4666	-0.08	132	-6.93	12616	0.11	133	8.88	4267
LPCAT4	1.06	0.00053341	0.09	119	6.5	4667	0.06	91	2.65	6723	0.08	95	3.45	6692
ZFP30_t1	1.02	0.084654917	0.09	124	6.5	4668	NA	NA	NA	NA	0.08	150	7.97	4631
MGARP	1.02	0.490775047	0.08	128	6.5	4669	0.13	124	9.29	3151	0.09	131	7.55	4817
C2CD2_t1	-1.08	0.021708342	0.06	155	6.5	4670	NA	NA	NA	NA	-0.07	129	-5.56	11260
CNR1	1.25	6.29E-05	0.06	150	6.5	4671	0.01	141	0.64	8041	0.15	144	13.65	2696
TRIM16L	1.74	2.62E-13	0.11	110	6.5	4672	0.1	123	6.96	4268	0.21	124	15.91	2097
RBM48	1.13	1.60E-07	0.09	122	6.49	4673	0.1	129	7.61	3926	0.16	126	12.38	3077
HPRT1	-1.01	0.918378414	0.08	135	6.49	4674	-0.05	135	-3.83	10996	0.11	151	10.93	3535
NSRP1	1.07	0.196687164	0.09	123	6.49	4675	0.07	131	5.38	5133	0.19	121	13.73	2669
EPHA3_t1	1.03	0.00133842	0.09	120	6.49	4676	-0.07	138	-6.11	12249	0.13	133	10.57	3667
ARFRP1	1.13	1.01E-06	0.09	119	6.49	4677	0.04	131	3.36	6276	0.13	112	8.01	4613
TBC1D13	1.26	1.17E-06	0.07	143	6.48	4678	0.19	128	14.78	1337	0.08	130	6.25	5332
PDZK1	1.53	0.00024531	0.08	135	6.48	4679	0.15	114	9.34	3127	0.29	142	26.73	438
NFRKB	1.1	3.41E-08	0.11	111	6.48	4680	0.17	120	11.66	2249	0.09	127	6.84	5089
ATP5L	1.22	3.77E-07	0.07	137	6.48	4681	0.26	128	20.47	344	0.16	116	10.73	3600
COX7A2	1.15	0.002913895	0.13	100	6.48	4682	0.08	95	3.4	6248	0.18	108	10.69	3617
ZNF595	1.03	0.373435701	0.07	143	6.47	4683	0.06	128	4.96	5363	0.06	131	4.57	6142
PDHA1	1.17	0.001104163	0.09	125	6.47	4684	-0.02	146	-1.84	9724	0.15	119	10.42	3714
POMT1	1.15	6.50E-06	0.12	104	6.47	4685	0.03	118	2.26	6981	0.16	104	8.68	4354
DOPEY2	1.05	0.301109035	0.08	136	6.47	4686	0.1	130	8.34	3581	-0.02	124	-1.82	9622
HEXB	1.27	2.38E-06	0.1	112	6.47	4687	0.07	100	3.39	6256	0.2	104	11.05	3498
ATRIP	1.22	3.43E-17	0.22	80	6.47	4688	0.16	121	11.09	2431	0.29	89	11.43	3374
TLCD1	1.42	8.40E-18	0.12	103	6.47	4689	0.07	131	5.55	5036	0.25	94	10.94	3528
ZAK_t1	1.11	0.205548291	0.07	144	6.47	4690	0.11	151	11.5	2306	0.15	151	15.11	2302
GABRR2	1.02	0.027587205	0.09	120	6.47	4691	-0.03	139	-2.25	9998	0.05	134	4.42	6234
KCNF1	1.06	7.91E-05	0.13	99	6.47	4692	0.06	118	3.79	6010	0.04	93	1.62	7670
TTLL2	1.05	9.34E-05	0.06	155	6.47	4693	0.01	119	0.6	8072	0.11	128	8.42	4458
RNF121_t1	1.11	3.83E-05	0.07	139	6.46	4694	NA	NA	NA	NA	0.04	120	2.77	7043
MTMR11	1.86	3.79E-09	0.11	107	6.46	4695	0.15	102	8.01	3739	0.14	107	8.1	4582
MECP2	1.1	0.000547266	0.06	155	6.46	4696	0.09	140	7.8	3832	0.05	153	4.7	6068
STK25	1.14	6.87E-06	0.07	138	6.46	4697	0.17	113	10.56	2650	-0.01	111	-0.6	8985
VWA1	1.17	0.004767222	0.09	123	6.46	4698	0.13	129	10.16	2802	0.08	120	5.4	5732
MYO1A	1.52	1.68E-12	0.13	100	6.46	4699	0.07	114	4.24	5754	0.22	100	10.98	3518
PSMC6_t1	1.06	0.353924364	0.1	112	6.45	4700	0.03	92	1.35	7576	NA	NA	NA	NA
NUP35	1.12	0.010723049	0.09	125	6.45	4701	0.12	128	9.35	3123	0.07	124	4.89	5980
RAB6A	1.03	0.15832093	0.08	130	6.45	4702	0.08	132	6.72	4390	0.06	138	5.38	5740
PPP1R14B	1.29	1.57E-08	0.13	99	6.45	4703	0.28	96	13.07	1807	0.2	128	15.33	2239
PSME1	1.22	2.75E-06	0.11	109	6.44	4704	0.04	100	1.94	7180	0.15	108	8.93	4249
GJA3	1.02	0.010697088	0.07	140	6.44	4705	0.05	147	4.58	5568	0.07	127	5.11	5874
ZNF148	-1.03	0.568208566	0.07	145	6.44	4706	-0.02	143	-2.19	9960	0.05	152	4.99	5928
PSME2	1.44	1.20E-09	0.17	88	6.44	4707	0.09	62	1.11	7740	0.22	82	6.93	5059
SDF2L1	1.58	5.39E-13	0.19	84	6.44	4708	0.22	104	11.92	2148	0.23	95	10.35	3741
CTTNBP2NL	-1.06	0.050081265	0.06	152	6.43	4709	0.13	135	11	2476	0.01	139	0.77	8153
NGDN	1.09	0.009950951	0.09	125	6.43	4710	-0.01	117	-0.9	9073	0.09	131	7.46	4848
ZHX2	1.13	0.000306432	0.07	139	6.43	4711	0	133	0.11	8392	0	120	0.35	8402
PCDHB13	1.11	0.000204034	0.09	120	6.42	4712	-0.01	134	-0.99	9139	0.04	128	3.05	6904
CDK5RAP1	1.16	6.14E-08	0.12	104	6.42	4713	-0.01	108	-0.39	8741	0.12	96	5.66	5595
RANBP2	-1.06	0.159436362	0.07	141	6.41	4714	0.16	131	12.63	1949	0.07	134	6.22	5346
SPARC	1.18	0.04564284	0.08	127	6.41	4715	0.16	126	12.44	1998	0.07	130	5.5	5682
ARRDC3_t1	1.01	0.751857318	0.07	143	6.41	4716	0.12	135	9.92	2888	NA	NA	NA	NA
CUL7	1.13	1.02E-06	0.12	104	6.41	4717	0.01	73	0.15	8364	0.08	74	1.85	7534
ISCA1_t1	1.13	0.000586537	0.09	118	6.41	4718	NA	NA	NA	NA	0.2	120	14	2597
SYNRG	-1.02	0.433379518	0.05	167	6.41	4719	-0.08	131	-6.46	12395	0.1	132	7.84	4689
ZBTB8OS	1.09	0.022708388	0.13	98	6.41	4720	0.11	111	7	4249	0.18	95	7.98	4626
PLCG1	1.11	0.001334328	0.11	108	6.4	4721	0.14	130	11.42	2327	0.08	114	5.21	5826
HIST1H1T	1.02	0.048944557	0.16	90	6.4	4722	0.03	76	0.84	7907	0.1	70	2.05	7423
LANCL2_t1	1.06	0.008999847	0.08	131	6.39	4723	0.02	132	1.54	7469	NA	NA	NA	NA
ZNF701	1.01	0.666584119	0.08	132	6.39	4724	0.19	130	14.96	1290	-0.02	148	-2.31	9844
SCOC	1.08	0.010916758	0.08	126	6.39	4725	0.11	124	7.87	3802	0.21	122	14.91	2361
ICA1	1.12	0.085337107	0.09	125	6.39	4726	0.03	125	2.35	6921	0.09	115	5.64	5607
FAP	1.35	0.020797989	0.1	111	6.39	4727	0.16	112	10.04	2846	0.04	114	2.29	7295
BOLA2B	1.85	2.65E-28	0.29	72	6.39	4728	0.28	68	4.98	5346	0.38	100	19.04	1444
SDHAF1	1.14	3.26E-05	0.07	140	6.39	4729	0.03	115	1.68	7370	0.12	107	6.85	5086
PHF14	1.03	0.201558646	0.07	147	6.38	4730	0.06	160	6.58	4462	0.14	152	14.28	2528
PIR	1.57	1.68E-08	0.13	100	6.38	4731	0.16	124	11.69	2235	0.15	115	10.02	3870
ARPC5L	1.04	0.433442968	0.1	113	6.38	4732	0.11	124	8.29	3605	-0.04	120	-2.83	10103
VCX3A	2.97	1.74E-11	0.1	112	6.38	4733	-0.04	104	-2.02	9837	0.2	103	10.6	3654
P2RY2	1.33	0.000127894	0.1	116	6.38	4734	0.09	124	6.3	4617	0.22	125	16.79	1877
ZSCAN21	1.27	3.84E-12	0.11	109	6.38	4735	0.01	125	0.64	8042	0.21	120	15.05	2322
CHD9	1	0.939499476	0.08	131	6.37	4736	0.04	129	3.13	6418	0.07	170	8.77	4313
TP53AIP1	1.03	0.000967308	0.13	100	6.37	4737	-0.01	135	-1.11	9230	0.08	120	5.89	5500
RAX2	1.1	4.62E-12	0.07	144	6.37	4738	0.12	142	11.07	2444	0.14	120	10	3880
RWDD3	-1.01	0.804931447	0.09	119	6.37	4739	0.03	107	1.8	7276	0.11	135	8.93	4251
XKRX	1.06	3.15E-05	0.06	151	6.37	4740	0.04	151	4.46	5631	0.18	134	15.34	2237
CSPG5	1.04	0.004610023	0.1	113	6.36	4741	-0.06	124	-4.4	11324	0.15	134	12.39	3070
PDCD2L	1.49	2.61E-19	0.24	77	6.36	4742	0.19	95	8.35	3567	0.26	84	8.81	4300
GPBP1L1_t1	-1.04	0.199509958	0.08	131	6.36	4743	NA	NA	NA	NA	0.06	122	4.22	6331
SLC25A6	1.06	0.131415739	0.1	111	6.35	4744	0.07	150	7.32	4078	0.05	141	4.18	6342
RPS19	1.21	0.000375573	0.19	83	6.35	4745	0.11	98	5.19	5223	0.06	95	2.87	7007
GJA1_t1	1.01	0.482573191	0.07	139	6.35	4746	NA	NA	NA	NA	0.08	161	8.52	4415
HIST1H2AD	1.79	1.39E-13	0.2	82	6.35	4747	0.11	84	3.63	6094	0.17	80	5.06	5897
IRF2BP1	1.14	0.000293391	0.1	115	6.35	4748	-0.01	141	-1.33	9382	0.06	106	3.53	6654
TYR	1.01	0.140427137	0.1	115	6.35	4749	0.06	116	3.66	6079	0.2	123	14.69	2408
PPP6R3_t1	1	0.726595108	0.07	144	6.35	4750	0.08	130	6.5	4510	NA	NA	NA	NA
PPP1R21_t1	1.04	0.125940485	0.09	120	6.34	4751	NA	NA	NA	NA	0.01	140	0.61	8251
PRKACB	-1.06	0.14795582	0.07	137	6.34	4752	0.12	139	10.66	2615	-0.01	142	-0.62	8994
ATMIN	-1.08	0.023617204	0.07	139	6.34	4753	-0.06	124	-4.67	11474	0.02	155	2.34	7267
DCAF15	1.05	0.005720906	0.12	104	6.33	4754	0.13	104	7.06	4216	0.03	129	2.28	7307
MAPK6_t1	1.1	0.177310507	0.06	149	6.33	4755	NA	NA	NA	NA	0.22	129	17.04	1819
ZNF585B	1.02	0.568777921	0.08	132	6.33	4756	0.14	158	15	1278	0.04	130	3.31	6769
FARSA	1.18	8.83E-10	0.1	114	6.33	4757	0.02	122	1.61	7419	0.07	121	4.92	5969
ZNF770	1.04	0.018362015	0.07	135	6.33	4758	0.07	122	5.07	5292	0.13	147	12.31	3109
SLC30A10	1.09	0.354676397	0.1	114	6.33	4759	-0.02	119	-1.39	9426	0.23	142	20.83	1149
CBX3	1.31	1.41E-05	0.08	133	6.33	4760	0.22	135	18.59	572	0.23	127	17.9	1662
TNFRSF4	1.21	9.34E-12	0.18	85	6.33	4761	0.24	131	19.27	481	0.15	100	7.64	4778
CNPY2	1.29	2.06E-09	0.12	104	6.32	4762	0.04	105	2.45	6857	0.21	102	11.04	3499
TBC1D9B_t1	1.1	0.008577522	0.1	113	6.32	4763	NA	NA	NA	NA	0.12	127	9.45	4067
SNRPA1	1.42	7.76E-09	0.12	104	6.32	4764	0.15	109	8.93	3313	0.22	111	13.61	2709
NCAPH2_t1	1.28	6.91E-09	0.09	120	6.32	4765	0.07	126	5.65	4979	0.08	120	5.45	5711
PRDX2_t1	1.04	0.222818131	0.09	118	6.32	4766	-0.1	108	-5.94	12153	0.07	111	4.29	6294
DAZAP1	1.12	2.12E-05	0.09	120	6.31	4767	0.21	105	11.48	2312	0.02	113	0.99	8026
FAM129A_t1	-1.01	0.888832421	0.07	137	6.31	4768	NA	NA	NA	NA	-0.01	140	-0.76	9057

ITGA7	1.49	1.21E-07	0.08	132	6.31	4769	0.13	104	7.02	4234	0.12	88	4.37	6260
MAP7D1	1.09	0.057414952	0.09	123	6.31	4770	0.17	131	13.72	1619	-0.01	120	-0.47	8891
CRLF3_t1	1.04	0.034894624	0.08	134	6.31	4771	NA	NA	NA	NA	0.07	128	5.45	5712
TDGF1	1.93	1.34E-06	0.06	161	6.31	4772	0.03	120	2.15	7041	0.17	123	12.6	3006
GPR111	1.03	0.004396311	0.08	132	6.3	4773	-0.01	122	-0.49	8811	0.18	119	12.63	3001
RGS12_t1	1.11	3.50E-11	0.21	80	6.3	4774	0.15	110	8.88	3336	0.26	100	12.84	2943
SLC25A3	1.27	7.06E-08	0.08	132	6.3	4775	0.12	122	8.54	3483	0.19	106	10.63	3643
NANOS1	1.34	8.73E-05	0.07	136	6.3	4776	0.16	135	13.91	1558	0.05	147	4.75	6051
TMX2	1.18	4.44E-06	0.09	118	6.29	4777	0.1	146	9.48	3075	0.13	117	8.7	4340
TMPRSS11D	1.01	0.29906608	0.08	124	6.29	4778	0.03	128	2.69	6686	0.02	131	2.02	7440
KY	1.03	0.023505329	0.09	123	6.29	4779	0.01	146	0.93	7854	0.12	133	9.69	3992
CNOT3	1.12	4.52E-08	0.1	116	6.28	4780	0.09	97	4.22	5770	0.12	92	4.86	5998
ATP11A_t1	1.06	2.16E-06	0.07	135	6.28	4781	0.06	153	6.03	4762	0.1	150	10.15	3811
ZNF326_t2	-1.07	0.204149698	0.08	132	6.28	4782	0.05	127	3.81	6002	0.08	115	5.52	5677
FBXO42	1.14	0.00025212	0.11	106	6.28	4783	0.08	120	5.92	4830	0.14	119	9.66	4000
TRIM10_t1	1.05	8.44E-05	0.08	127	6.28	4784	0.05	133	4.25	5753	0.16	135	13.52	2732
CPE	1.33	0.026792394	0.09	120	6.28	4785	0.04	117	2.93	6531	0.22	132	18.42	1565
PDGFRL	1.47	3.06E-10	0.11	108	6.28	4786	0.16	113	10.04	2845	0.11	99	5.16	5846
GNL3_t1	1.22	0.000281627	0.11	105	6.28	4787	NA	NA	NA	NA	0.17	95	7.67	4764
SMPD3_t1	-1	0.70101496	0.09	120	6.28	4788	NA	NA	NA	NA	0.04	129	3.18	6844
FIZ1	1.07	0.000165745	0.13	100	6.28	4789	0.07	140	6.35	4588	0.02	116	1.63	7663
CCDC102B	1.06	0.106593393	0.1	111	6.27	4790	0.19	112	11.88	2158	0.07	118	4.57	6138
BTBD9	1.01	0.337324393	0.06	149	6.27	4791	0.12	146	11.08	2438	0.04	132	3.58	6628
CRB1	1.03	0.005257325	0.08	125	6.27	4792	0.01	123	0.84	7904	0.08	117	5.4	5735
PRSS36	1.16	0.000173544	0.1	113	6.27	4793	0.02	109	1.05	7772	0.07	108	3.91	6463
YIPF6	1.05	0.248559145	0.06	147	6.27	4794	0.01	136	1.28	7618	0.13	145	12.31	3108
GIT2_t1	1.14	0.000346402	0.07	134	6.27	4795	0.19	132	15.7	1107	0.09	147	8.89	4266
LNX1	1.06	0.128123005	0.09	123	6.26	4796	0.07	113	4.61	5547	0.08	120	5.45	5710
XRCC1	1.38	2.63E-18	0.24	76	6.26	4797	0.14	103	7.39	4039	0.19	104	10.2	3797
MFHAS1	1.04	0.020567507	0.07	140	6.26	4798	0.05	127	4.14	5815	0.08	120	5.47	5705
TMPRSS12_t1	1.01	0.270433856	0.08	124	6.26	4799	NA	NA	NA	NA	0.15	136	12.97	2896
DUSP21	1.01	0.417873528	0.09	120	6.26	4800	0.06	105	3.45	6211	0.02	126	1.43	7790
DNAH11	1.15	3.83E-09	0.09	120	6.25	4801	-0.02	135	-1.39	9424	0.16	124	12.14	3151
HS6ST3_t1	1.07	0.00439767	0.07	139	6.25	4802	0.06	145	6.17	4685	NA	NA	NA	NA
SPAG17	1.07	4.74E-05	0.1	112	6.25	4803	0.06	132	5.06	5297	0.11	91	4.57	6137
NPM3	1.14	0.021740279	0.1	113	6.25	4804	0.04	112	2.56	6786	-0.02	110	-0.95	9168
ERCC6	1	0.918017822	0.06	148	6.25	4805	-0.06	124	-4.54	11407	0.15	129	11.97	3209
TSGA13	1.03	0.001460165	0.13	100	6.25	4806	0.09	129	7.11	4191	0.28	103	14.97	2343
SNTB2	-1.11	0.011205759	0.07	145	6.25	4807	0.03	141	3.01	6490	-0.14	153	-14.78	14320
TMEM196	1.02	0.018341795	0.07	140	6.25	4808.5	-0.04	132	-3.25	10636	0.06	143	5.89	5496
CERK	1.1	0.00300405	0.07	140	6.25	4808.5	0.01	131	0.9	7871	0.08	134	6.73	5137
FAM110A	1.21	0.000438813	0.1	113	6.24	4810	0.21	115	13.38	1722	0.01	115	0.4	8374
DPM3_t1	1.42	3.84E-11	0.15	91	6.24	4811	NA	NA	NA	NA	0.24	83	7.98	4627
RNF144A_t1	1.02	0.073941927	0.08	133	6.24	4812	0.12	131	9.54	3038	NA	NA	NA	NA
PRMT8	1.06	5.89E-06	0.09	120	6.24	4813	0.05	124	3.92	5939	0.06	124	4.25	6316
NOP58	1.22	0.00100556	0.09	116	6.24	4814	0.21	106	11.78	2196	0.1	117	6.51	5230
ZFPL1	1.17	2.56E-11	0.1	112	6.24	4815	0.1	100	5.15	5250	0.15	106	8.57	4392
ZFC3H1	1.04	0.43161683	0.07	135	6.23	4816	0.19	133	16.11	1019	0.12	143	10.78	3579
PNMA5	1.12	0.182793424	0.09	120	6.23	4817	-0.08	128	-5.91	12140	0.13	119	9.03	4218
TMEM169_t1	1.04	0.000203795	0.09	119	6.23	4818	NA	NA	NA	NA	NA	NA	NA	NA
SARS2	1.06	0.012232935	0.11	106	6.23	4819	-0.01	92	-0.51	8826	0.03	99	1.33	7838
MRPS7	1.2	1.34E-06	0.1	113	6.23	4820	0.11	108	6.3	4618	0.12	114	7.72	4747
COX7C	1.22	1.41E-05	0.1	111	6.22	4821	0.06	109	3.32	6302	0.23	93	9.99	3881
NOX1_t1	1.08	0.01669839	0.06	151	6.22	4822	-0.02	104	-1.26	9334	0	147	-0.17	8709
PDE4DIP_t1	1.06	0.299578349	0.07	141	6.22	4823	-0.08	152	-7.76	13013	NA	NA	NA	NA
TKTL2	1.04	0.035430069	0.09	122	6.22	4824	-0.08	119	-5.34	11837	0.07	134	6.06	5420
LYZL4	1.04	0.000148769	0.11	106	6.22	4825	0.01	113	0.36	8232	0.22	109	13.24	2817
ZNF671	-1.16	0.014207313	0.09	122	6.22	4826	-0.01	131	-0.88	9064	-0.08	144	-7.14	11894
CKM	1.04	0.000196637	0.11	108	6.22	4827	0.16	125	12.22	2057	0.02	113	1.23	7895
MLKL	-1.04	0.332941166	0.09	120	6.22	4828	0	123	0.14	8372	-0.06	111	-3.41	10360
NOLC1_t1	-1.05	0.259368148	0.07	133	6.21	4829	NA	NA	NA	NA	-0.02	130	-1.21	9305
FKBP3	1.12	0.015530553	0.07	134	6.21	4830	0.02	100	1.18	7688	0.07	110	4.21	6335
ARHGAP22	1.15	0.004160339	0.14	94	6.21	4831	0.21	147	20.18	378	0.02	98	1.08	7978
FAM24B	1.32	4.04E-11	0.11	109	6.21	4832	0.18	115	11.4	2336	0.25	97	11.63	3317
TRIM36_t1	1.03	0.043897608	0.08	126	6.21	4833	NA	NA	NA	NA	0.1	126	7.32	4909
STOML1_t1	1.32	2.37E-11	0.11	107	6.21	4834	0.16	105	9	3274	NA	NA	NA	NA
OGG1_t2	1.3	4.63E-10	0.11	108	6.21	4835	0.21	119	14.22	1470	0.17	118	11.5	3347
NFATC2IP	1.09	1.01E-06	0.07	144	6.2	4836	0.13	144	12.35	2023	0.02	148	1.59	7698
CYB561_t1	1.23	0.018464034	0.08	127	6.2	4837	0.06	132	4.64	5529	NA	NA	NA	NA
SLC38A9	1.25	7.88E-06	0.08	128	6.2	4838	0.05	104	2.66	6712	0.28	102	14.53	2451
FBXO3_t1	1	0.916969558	0.08	131	6.2	4839	-0.03	115	-1.87	9741	0.12	109	6.88	5074
IL3RA_t1	1.18	3.25E-11	0.11	104	6.2	4840	NA	NA	NA	NA	0.13	100	6.28	5324
LILRB4	1.02	0.171254512	0.11	108	6.19	4841	-0.11	107	-6.33	12338	-0.02	111	-0.98	9194
XKR3	1.05	2.60E-05	0.11	104	6.19	4842	0.04	109	2.44	6858	0.15	95	6.77	5123
ALDOA_t1	1.27	1.43E-05	0.12	100	6.19	4843	0.21	140	18.87	541	0.02	91	0.79	8141
LARP4_t1	1.03	0.28889096	0.06	149	6.19	4844	0.03	158	3.49	6188	0.17	147	16.77	1885
XRCC4	1.14	0.000742663	0.09	119	6.18	4845	0.14	120	10.11	2823	0.14	122	9.82	3943
FAM63A_t1	1.1	1.41E-05	0.09	118	6.18	4846	NA	NA	NA	NA	0.14	99	6.89	5071
SLC25A39	1.33	3.25E-18	0.14	94	6.18	4847	0.12	108	7.05	4224	0.24	100	11.86	3247
APOLD1	1.26	0.012946727	0.08	123	6.17	4848	0.1	134	8.69	3414	0.08	138	6.87	5076
EXOC5_t1	1	0.922400293	0.06	146	6.17	4849	NA	NA	NA	NA	NA	NA	NA	NA
CLCN4	1.03	0.009862108	0.06	148	6.17	4850	0	148	0.04	8436	0.06	133	4.92	5965
TRAPPC2L	1.12	0.000601216	0.15	91	6.17	4851	0.07	90	2.78	6622	0.09	92	3.74	6551
TUBD1	1.15	2.80E-05	0.09	117	6.17	4852	0.14	131	11.69	2236	0.19	120	12.99	2884
PLP2	1.3	3.71E-06	0.13	96	6.16	4853	0.17	130	13.34	1734	0.01	100	0.67	8215
PTDSS1	1.2	6.83E-05	0.07	134	6.16	4854	0.1	114	6.22	4649	0.18	128	13.83	2642
RAB3A	1.33	2.49E-08	0.13	98	6.16	4855	0	125	-0.35	8713	0.19	118	12.62	3003
TBCCD1	1.03	0.168184704	0.08	124	6.16	4856	0.02	111	1.01	7802	0.03	120	2.12	7388
SLC22A4	1.41	1.33E-05	0.12	100	6.16	4857	0.14	122	10.26	2773	0.28	120	19.81	1302
PSIP1_t1	1.24	4.83E-09	0.1	110	6.16	4858	0.11	132	9.14	3211	0.13	118	8.59	4386
HIST1H2BL	1.66	1.73E-14	0.21	80	6.15	4859	0.1	100	4.81	5442	0.22	80	6.63	5180
BAMBI	-1.29	0.107576032	0.08	130	6.15	4860	0.21	125	15.65	1112	-0.06	120	-3.91	10593
ERO1LB_t1	1.05	0.38453168	0.07	137	6.15	4861	NA	NA	NA	NA	0.08	127	6.39	5276
EIF2D	1.41	2.84E-16	0.19	83	6.15	4862	0.17	97	8.11	3693	0.29	119	19.8	1304
SRM	1.31	6.36E-13	0.15	92	6.15	4863	0.09	102	4.64	5526	0.17	112	10.42	3716
ALX4	-1	0.983728597	0.07	142	6.15	4864	-0.05	158	-5.05	11687	-0.04	121	-2.49	9945
NOS2	1.4	6.03E-06	0.06	147	6.15	4865	0	104	-0.07	8535	0.14	133	11.24	3434
IFT172	1.16	7.41E-06	0.1	110	6.15	4866	0.16	88	6.21	4654	0.05	82	1.73	7617
ZNF302_t1	1.24	1.88E-08	0.1	113	6.14	4867	NA	NA	NA	NA	0.26	117	17.32	1766
MBD1_t2	1.01	0.888200477	0.09	115	6.14	4868	0.07	113	4.32	5705	0.01	111	0.36	8397
CHRND	1.07	2.52E-06	0.07	139	6.14	4869	0.01	125	0.81	7927	0.07	129	5.47	5700
VAMP5	1.04	0.407220669	0.11	108	6.14	4870	-0.03	107	-1.99	9811	-0.05	120	-3.66	10461
RGS7	1.02	0.141025032	0.12	101	6.13	4871	0.13	133	10.71	2589	0.04	114	2.39	7239
APPL1	1.07	0.000331933	0.08	131	6.13	4872	0.11	139	9.94	2882	0.13	148	13.22	2826
PSMA5	1.12	0.006974144	0.08	126	6.13	4873	0.15	127	11.77	2203	0.11	143	10.66	3628
ANKRA2_t1	1.14	0.000269211	0.09	118	6.13	4874	NA	NA	NA	NA	NA	NA	NA	NA

B3GNTL1	1.32	5.86E-16	0.14	94	6.13	4875	0.25	103	13.33	1738	0.18	108	10.33	3747
NCOR2	1.25	6.01E-06	0.1	112	6.13	4876	0.19	130	15	1279	0.13	104	6.86	5083
PDZD4	1.09	1.34E-09	0.07	132	6.12	4877	-0.06	118	-4.4	11323	0.1	129	7.86	4681
CYP4Z2P	1.04	0.040651334	0.07	138	6.12	4878	0.05	125	3.55	6144	0.05	115	2.97	6940
COL17A1_t1	1.02	0.413356332	0.08	128	6.12	4879	0.03	139	2.39	6896	NA	NA	NA	NA
SFSWAP	1.05	0.056253144	0.08	124	6.12	4880	0.12	105	6.37	4579	0.03	97	1.55	7719
PANK3	1.04	0.366855346	0.08	123	6.12	4881	0.03	133	2.72	6666	0.16	158	17.6	1717
S100A3	1.17	3.39E-11	0.12	101	6.11	4882	0.11	116	7.36	4057	0.14	105	7.89	4666
ARHGEF15	1.01	0.710568143	0.09	120	6.11	4883	-0.03	125	-2.6	10218	0.03	119	1.87	7521
POU1F1	-1.01	0.50115483	0.07	134	6.11	4884	-0.11	104	-6.16	12267	0.01	123	0.47	8333
MAP4K5_t1	-1.02	0.636889143	0.08	126	6.1	4885	NA	NA	NA	NA	NA	NA	NA	NA
YIPF7	1.05	3.56E-06	0.09	115	6.1	4886	0.09	113	5.39	5122	0.16	115	10.48	3698
PRKAR1A	1.18	0.000187753	0.07	132	6.1	4887	-0.02	122	-1.5	9497	0.06	133	4.59	6126
CEBPG	1.1	0.094595785	0.07	141	6.1	4888	0.08	133	7	4246	0.01	143	1.39	7813
RPL9	1.18	0.000453978	0.09	120	6.09	4889	0.07	114	4.48	5619	0.13	110	7.55	4816
CUL9	1.25	6.83E-09	0.12	99	6.09	4890	0.15	108	8.45	3529	0.12	82	3.72	6553
HNF4A_t1	1.13	8.03E-07	0.1	113	6.09	4891	0.04	102	2.22	7001	0.19	112	11.92	3227
GRHL3	1.04	0.015243096	0.06	151	6.09	4892	-0.06	137	-4.79	11529	-0.03	121	-2.31	9847
HRCT1	1.7	4.49E-12	0.12	100	6.09	4893	0.05	100	2.52	6813	0.22	107	12.32	3104
WDR6	1.15	0.000128116	0.12	100	6.09	4894	0.17	139	14.93	1298	0.13	121	8.98	4234
TMEM180	1.2	6.14E-10	0.08	131	6.09	4895	-0.01	122	-0.95	9110	0.17	115	11.33	3404
BABAM1	1.08	0.001764966	0.09	120	6.09	4896	-0.03	123	-2.22	9977	0.03	109	1.81	7565
MVD	1.11	0.010196313	0.11	104	6.09	4897	0.02	101	0.83	7916	0.05	100	2.54	7159
PTPLB_t1	1.22	0.000112502	0.06	144	6.09	4898	NA	NA	NA	NA	0.21	127	16.55	1942
TPTE2	1.02	0.025367086	0.08	125	6.08	4899	0.07	93	2.96	6514	0.04	101	2.22	7341
RAB5A	1.04	0.345123947	0.08	130	6.08	4900	0.06	132	4.59	5560	0.07	151	7.34	4897
RNF157_t1	1.47	1.26E-11	0.07	134	6.08	4901	0.16	138	13.88	1568	NA	NA	NA	NA
FBXO30	1.1	0.033429813	0.07	135	6.08	4902	0.14	128	10.75	2573	0.11	115	7.36	4890
PBX2	1.06	9.88E-05	0.08	126	6.07	4903	0.19	129	15.04	1264	0.04	123	2.59	7133
WASH1	1.12	5.31E-08	0.09	121	6.07	4904	0.13	114	8.54	3482	0.09	121	6.11	5399
TMEM126B	1.05	0.367492374	0.15	91	6.07	4905	0.08	104	4.17	5800	0.08	103	4.49	6189
PAGE4	2.02	7.72E-06	0.11	104	6.07	4906	0.04	109	2.3	6956	0.19	92	8.13	4564
DDN	1.02	0.291450495	0.08	124	6.06	4907	-0.01	132	-0.43	8766	0	128	-0.37	8835
NABP1	-1.07	0.372607109	0.06	149	6.06	4908	0.15	126	11.14	2422	-0.07	158	-7.84	12178
ZNF335	1.19	4.33E-11	0.09	114	6.06	4909	0.19	118	12.6	1953	0.14	88	5.47	5699
LRP10	1.09	0.078704151	0.1	110	6.06	4910	0.07	150	7.29	4095	0	107	-0.05	8653
STXBP1_t1	1.09	0.008494218	0.07	138	6.05	4911	0.01	119	0.49	8142	0.08	143	7.05	5009
ATP1A1	1.24	8.66E-05	0.08	124	6.05	4912	0.11	135	9.47	3080	0.12	116	8.17	4549
CAV3_t1	1.11	0.002520828	0.1	109	6.05	4913	0.1	120	6.81	4343	0.13	137	11.58	3329
ARL3	1.04	0.220115744	0.09	118	6.05	4914	0.04	130	3.48	6193	0.08	137	7.36	4892
PPP4R2_t1	1.13	0.000823977	0.1	110	6.04	4915	NA	NA	NA	NA	0.22	96	9.94	3899
PPA1	1.1	0.111903001	0.09	114	6.04	4916	0.01	106	0.79	7946	0.13	103	6.74	5136
ZNF652	1.08	0.169335239	0.06	150	6.04	4917	0.13	135	10.73	2581	0.11	147	10.34	3746
CSRNP2	-1.02	0.626330059	0.08	129	6.03	4918	0.06	114	3.84	5984	-0.02	122	-1.7	9555
THOC5	1.2	8.95E-08	0.12	100	6.03	4919	0.08	113	5.13	5263	0.2	117	13.18	2834
FASTKD5	1.25	7.40E-08	0.09	119	6.03	4920	0.13	98	6.36	4587	0.22	112	13.68	2682
MTPAP	1.07	0.062004014	0.06	143	6.03	4921	0.12	128	9.26	3160	0.15	138	13.38	2770
FAM84A	1.15	1.42E-08	0.06	155	6.03	4922	0.1	149	9.61	3020	0.12	136	10.11	3833
GJB5	1.03	0.000452092	0.15	91	6.03	4923	-0.07	116	-4.6	11439	0.08	100	3.94	6447
BFSP2	1.13	8.09E-05	0.07	135	6.03	4924	0.14	97	6.36	4586	0.01	104	0.4	8375
CAMKK1_t1	1.01	0.434263386	0.09	116	6.03	4925	0.02	130	1.94	7182	-0.04	105	-2.31	9843
ZBTB7C	1.02	0.05696699	0.08	128	6.03	4926	0.07	151	6.96	4265	0	132	0.2	8492
PLAU	1.2	0.024891389	0.12	102	6.02	4927	0.15	120	10.16	2801	0.02	128	1.38	7817
SRSF10_t2	-1.01	0.876022906	0.07	136	6.02	4928	0.05	118	3.32	6303	0.06	151	5.91	5488
TTC14	1.02	0.158568153	0.06	143	6.02	4929	0.14	133	11.56	2287	0.06	154	6.72	5145
CPLX2	1.2	7.51E-06	0.07	132	6.02	4930	-0.02	143	-1.75	9649	0.17	125	13.07	2860
ZNF323	1.45	1.94E-07	0.08	124	6.02	4931	0.03	138	2.67	6697	0.28	143	26.06	497
BOLA1	1.41	4.38E-11	0.12	100	6.02	4932	0.06	128	4.39	5667	0.23	128	17.65	1709
NDP	1.06	0.000139009	0.09	119	6.01	4933	0.11	110	6.61	4448	0.02	143	1.83	7542
TULP3	1.09	6.08E-07	0.1	111	6.01	4934	0.1	126	7.51	3981	0.12	124	8.58	4390
SLC25A33	1.27	0.00070659	0.12	99	6	4935	0.1	125	7.63	3913	0.11	120	7.6	4797
EIF2S3	1.16	1.27E-07	0.07	135	6	4936	0.14	128	10.57	2645	0.18	151	17.84	1674
RASD2	1.11	5.16E-11	0.08	128	5.99	4937	-0.01	126	-0.64	8904	0	136	0.04	8591
COPB1	1.08	0.142235413	0.08	121	5.99	4938	0.1	116	6.36	4585	0.14	124	10.72	3605
MTRF1	1.06	0.008807238	0.09	117	5.99	4939	0.13	129	10.39	2723	0.13	104	6.79	5107
CPO	1.14	9.62E-09	0.18	84	5.99	4940	0.05	93	2.15	7040	0.22	104	12.04	3181
CDS2_t1	-1.03	0.455552479	0.07	137	5.99	4941	NA	NA	NA	NA	NA	NA	NA	NA
COPZ1_t1	1.08	0.005321306	0.11	104	5.99	4942	NA	NA	NA	NA	NA	NA	NA	NA
BLMH_t1	-1.18	0.043498641	0.08	124	5.99	4943	0.14	120	9.94	2881	NA	NA	NA	NA
TUBB8	1.37	1.80E-09	0.14	93	5.99	4944	0.09	115	5.72	4939	0.19	92	8.09	4588
HTRA2	1.11	1.74E-05	0.08	124	5.99	4945	0.03	120	2.23	6996	0.11	106	6.06	5422
SLC26A8	1	0.842544541	0.1	111	5.98	4946	-0.08	139	-7.09	12692	-0.05	125	-3.57	10424
RUFY3	-1.04	0.059083543	0.06	146	5.98	4947	0.04	143	4.07	5870	0.07	128	5.59	5627
KDM6A	-1.03	0.527268431	0.07	136	5.98	4948	0.02	136	2.14	7054	0.09	130	7.15	4973
SPAG16_t1	1.03	0.035670502	0.11	104	5.98	4949	0.06	108	3.44	6222	-0.03	97	-1.34	9361
FAM135B	1.1	1.25E-05	0.07	131	5.98	4950	0.01	139	1.18	7690	0.15	127	11.49	3352
LCMT1	1.29	3.32E-10	0.12	100	5.98	4951	0.26	130	20.67	326	0.1	109	6.05	5426
CYP27B1	1.08	0.001680694	0.08	122	5.98	4952	-0.06	127	-4.83	11552	0.08	120	5.44	5719
PDE6A	1.1	9.07E-05	0.09	118	5.97	4953	0.08	135	6.49	4518	0.13	121	9.49	4057
NAA10	1.33	1.05E-16	0.16	87	5.97	4954	0.11	106	5.9	4846	0.24	114	15.38	2223
VSIG1	1.07	0.001279973	0.07	131	5.97	4955	0.11	131	8.74	3393	0.02	128	1.76	7589
WASF2	1.03	0.450381079	0.08	123	5.97	4956	0.14	134	11.46	2316	0.03	132	2.15	7371
WHSC1L1_t2	1	0.769060887	0.09	119	5.96	4957	0.07	107	4.16	5804	0.1	126	7.79	4716
VAMP7	1.12	0.098541176	0.07	132	5.96	4958	-0.01	130	-0.51	8821	0.19	135	16.06	2058
PTP4A2	1.04	0.426474106	0.08	127	5.96	4959	0.09	132	7.58	3943	0.08	143	7.13	4986
SCNN1B_t1	1.05	0.000229366	0.11	103	5.96	4960	NA	NA	NA	NA	0.09	101	4.37	6262
AZI2_t1	1.1	0.050682989	0.07	134	5.96	4961	0.04	133	2.92	6543	NA	NA	NA	NA
WTAP_t1	1.21	8.71E-05	0.09	114	5.95	4962	0.2	115	12.97	1842	0.09	126	6.79	5110
BHLHB9	1.12	0.000151087	0.08	121	5.95	4963	0.06	127	4.32	5706	0.11	133	9.18	4167
NIN_t3	1.04	0.429273073	0.06	147	5.95	4964	0.15	130	11.96	2137	0.06	150	6.11	5395
RIMS1	1.03	0.000532729	0.07	141	5.95	4965	0.02	159	2.15	7043	0.1	143	9.43	4079
SLC25A31	1.03	0.003048187	0.11	105	5.95	4966	0.14	111	8.67	3430	0.14	121	10.07	3849
FAM19A4	1.13	0.007786083	0.07	133	5.95	4967	0.05	106	2.7	6679	0.11	139	9.72	3975
RPL32	1.18	4.02E-05	0.08	125	5.95	4968	0.08	120	5.44	5090	0.09	119	6.04	5432
PHF8	-1.03	0.630472875	0.08	126	5.95	4969	0.02	141	2.01	7131	0	147	-0.34	8815
GEN1	1.03	0.015210961	0.06	151	5.94	4970	0.11	135	9.15	3209	0.1	140	8.96	4240
DOCK3	1.18	2.53E-05	0.07	130	5.94	4971	0.15	134	12.42	2003	0.13	151	13.54	2729
ZNF547	1.05	0.006972542	0.06	153	5.94	4972	0.16	153	16.62	913	0.03	137	2.76	7049
ASAP1-IT1	-1.02	0.49857003	0.08	120	5.94	4973	0.07	111	4.05	5881	0	121	-0.24	8759
FAM71C	1.03	0.002402976	0.08	123	5.94	4974	-0.09	120	-6.02	12191	0.1	126	7.31	4910
SCAF8	1.07	0.046744769	0.07	132	5.94	4975	0.11	117	7.68	3892	0.07	126	5.38	5741
DQX1	1.34	2.38E-05	0.13	97	5.93	4976	0.11	142	10.27	2771	0.1	125	7.83	4702
ATP5G1	1.32	3.91E-10	0.14	93	5.93	4977	0.06	100	2.99	6503	0.2	106	11.35	3399
HCG4	1.18	5.33E-07	0.1	111	5.93	4978	0.08	120	5.33	5157	0.17	131	14.13	2564
MMD2	1.03	0.002374807	0.07	138	5.93	4979	0.08	134	6.79	4354	0.09	122	6.42	5267
SLC26A4	1.03	0.136483489	0.07	133	5.93	4980	-0.01	147	-0.75	8976	0.05	133	4.06	6389

MEOX2	1.16	2.46E-05	0.09	114	5.93	4981	0.05	141	4.86	5420	0.14	153	14.24	2538
BANF2	1.18	1.57E-09	0.19	81	5.92	4982	0.16	97	7.56	3955	0.29	90	11.44	3369
GPR64	1.64	2.07E-06	0.06	145	5.92	4983	0	138	-0.01	8489	0.2	125	14.81	2382
DDX51	1.2	5.59E-12	0.07	131	5.92	4984	0.08	143	7.74	3865	0.07	123	5.29	5780
CNOT4_t2	1.03	0.002219582	0.05	160	5.92	4985	0.12	120	8.47	3523	0.12	161	13.56	2722
MEN1	1.05	1.41E-07	0.12	100	5.92	4986	0.18	126	13.42	1705	0.14	115	8.99	4233
EXD1	1.06	6.00E-07	0.08	120	5.91	4987	0.05	135	4.25	5750	0.09	123	6.35	5298
LMX1B	1.01	0.148841044	0.07	132	5.91	4988	-0.04	131	-3.33	10694	0.04	160	4.38	6254
VPS37A	-1.02	0.688956769	0.07	132	5.9	4989	0	128	-0.29	8664	0.07	144	6.49	5241
ALS2CR11	1.03	0.025178141	0.12	98	5.9	4990	0.18	104	9.56	3032	0.07	87	2.65	7103
TBRG4	1.34	1.13E-17	0.12	100	5.9	4991	0.16	111	9.52	3050	0.21	120	15.01	2336
MRPL53	1.23	7.21E-13	0.13	94	5.9	4992	0.04	104	1.93	7194	0.19	117	13.01	2876
C1QL3	1.03	0.00167835	0.07	140	5.89	4993	0.01	131	0.42	8192	0.14	137	11.99	3205
EPYC	1.01	0.449144953	0.08	125	5.89	4994	0.04	108	2.22	6999	-0.04	124	-2.65	10017
MRPS30	1.16	3.11E-06	0.08	124	5.89	4995	0.05	100	2.33	6935	0.19	131	15.43	2210
TPPP	1.02	0.336401238	0.07	136	5.89	4996	-0.07	146	-6.72	12523	0.01	131	0.59	8265
CCNG2_t1	1.06	0.259368148	0.06	142	5.88	4997	0.05	145	4.89	5395	NA	NA	NA	NA
TYSND1	1.15	2.51E-05	0.08	123	5.87	4998	-0.01	130	-0.75	8971	0.07	122	5.28	5788
CPPED1	1.23	0.000930997	0.05	158	5.87	4999	0.04	136	3.47	6208	0.16	165	18.85	1489
CD2BP2	1.01	0.816668103	0.08	128	5.87	5000	0.01	128	1.05	7778	-0.03	151	-3.38	10343
HESX1	1.05	0.048356342	0.09	116	5.87	5001	0.01	105	0.59	8083	0.12	115	8.11	4577
CBX2	1.03	0.002048998	0.14	92	5.86	5002	0.05	104	2.58	6775	0.12	100	6.14	5381
SLC9B1_t1	-1	0.972764927	0.09	116	5.86	5003	NA	NA	NA	NA	NA	NA	NA	NA
ZNF222_t1	1.08	0.018825518	0.11	104	5.86	5004	NA	NA	NA	NA	0	100	0.22	8483
HDAC1	1.06	0.079827063	0.1	108	5.86	5005	0.12	128	9.53	3045	0.01	118	0.38	8393
HMGCR	1.15	0.071007616	0.07	133	5.86	5006	0.09	124	6.75	4376	0.15	125	11.26	3425
TGM4_t1	1.02	0.049457025	0.08	119	5.86	5007	0.05	124	4.07	5867	NA	NA	NA	NA
MAU2_t1	1.05	0.052722041	0.08	128	5.86	5008	NA	NA	NA	NA	NA	NA	NA	NA
HNRNPK_t1	1.06	0.205487442	0.07	138	5.86	5009	0.12	124	8.83	3365	0.07	123	5.44	5722
IQUB_t1	1.02	0.087153121	0.07	133	5.85	5010	0.2	122	14.08	1515	NA	NA	NA	NA
FMR1	1.11	0.083758171	0.07	130	5.85	5011	-0.01	126	-1.06	9188	0.15	156	16.09	2051
TFCP2L1	1.12	0.029679552	0.08	126	5.85	5012	0.16	153	16.78	883	-0.04	150	-3.87	10579
WWC3	1.13	0.027894121	0.06	152	5.85	5013	0.15	141	13.82	1592	-0.05	135	-3.98	10632
LRP4	1.37	2.56E-05	0.08	127	5.85	5014	0.07	127	5.57	5027	0.24	146	22.77	868
SETD6	1.03	0.372324439	0.08	120	5.85	5015	0.05	120	3.77	6025	0.17	131	13.54	2727
CDK2AP1	1.12	0.003806668	0.06	153	5.85	5016	0.15	110	9.09	3235	0.03	134	2.37	7249
IDH3A	1.05	0.25193308	0.08	125	5.84	5017	0.02	134	1.98	7158	0.19	142	17.3	1769
PPAP2A	1.17	0.034011276	0.08	121	5.84	5018	0.1	117	6.53	4494	0.11	101	5.44	5716
GAGE7	2.01	8.53E-08	0.1	106	5.84	5019	-0.08	104	-4.45	11355	0.13	87	4.74	6057
CCDC170	1.41	2.34E-09	0.07	134	5.84	5020	0.07	133	6.2	4662	0.24	128	18.62	1533
PIGW_t1	1.07	0.000112908	0.1	110	5.84	5021	0.1	127	7.34	4071	NA	NA	NA	NA
HNRNPA2B1	1.05	0.222528736	0.06	140	5.84	5022	0.07	130	5.41	5113	0.14	110	8.55	4405
HNRNPL_t1	1.1	2.95E-05	0.08	124	5.84	5023	NA	NA	NA	NA	NA	NA	NA	NA
CDH10	1.07	0.000465416	0.08	126	5.84	5024	0.04	111	2.18	7020	0.17	121	12.28	3117
ENPP4	1.12	0.03066069	0.05	157	5.83	5025	0.08	136	6.71	4394	0.19	151	18.91	1471
PHKA1_t1	1.01	0.635884793	0.07	131	5.83	5026	NA	NA	NA	NA	0.13	145	12.75	2960
ZIC3	1.03	0.025245653	0.07	137	5.83	5027	-0.12	143	-10.89	14205	0.1	140	9.05	4208
ZNF625_t1	1.03	0.063416429	0.11	104	5.83	5028	NA	NA	NA	NA	0.14	100	6.9	5067
CNFN	1.29	2.78E-12	0.15	88	5.83	5029	0.18	120	12.55	1969	0.11	88	4.21	6336
CCHCR1	1.41	2.51E-23	0.18	83	5.83	5030	0.13	78	3.77	6024	0.21	119	14.2	2545
EXOSC7	1.16	8.87E-07	0.1	109	5.83	5031	0.19	92	7.84	3810	0.12	120	8.42	4457
SPOCD1	1.27	1.02E-09	0.08	122	5.83	5032	0.24	90	9.45	3089	0.06	110	3.75	6543
SENP7	-1.01	0.859065266	0.07	131	5.83	5033	0.11	133	8.82	3370	0.03	131	2.2	7349
HRH1	1.02	0.565051958	0.07	136	5.83	5034	0.11	135	9.59	3024	0.12	137	10.23	3789
RFX3	1.01	0.447708372	0.09	114	5.82	5035	0.17	117	11.54	2290	0.09	131	7.17	4961
LRCH1	1.03	0.046985226	0.07	138	5.82	5036	0.13	129	10.14	2814	0.03	131	2.23	7335
MCAT_t1	1.08	0.041544145	0.08	120	5.82	5037	0	134	-0.37	8729	0.06	104	3.12	6861
VWA5A_t1	1.07	0.000194648	0.09	116	5.82	5038	0.23	115	15.03	1267	0	120	0.05	8588
BTC_t1	1.07	5.93E-05	0.09	115	5.82	5039	NA	NA	NA	NA	0.13	125	9.62	4017
SPINK5	1.66	9.13E-14	0.08	121	5.82	5040	0.02	104	1.24	7647	0.26	109	15.34	2235
EMC4	1.16	1.42E-05	0.08	119	5.82	5041	-0.01	114	-0.64	8899	0.18	107	10.1	3839
ZC3H3	1.08	3.65E-05	0.13	94	5.82	5042	-0.01	117	-0.47	8794	0.12	100	5.89	5499
EID2	1.09	0.004481552	0.07	129	5.81	5043	-0.07	119	-5.1	11719	0.13	118	8.73	4331
ADD2_t1	-1	0.655478952	0.07	138	5.81	5044	-0.03	120	-1.79	9688	-0.03	150	-2.8	10090
TBC1D22B	1.01	0.574882443	0.09	118	5.81	5045	0.02	127	1.37	7567	0	131	0.39	8380
ALS2CR8	1	0.926596696	0.06	145	5.8	5046	0.02	139	1.45	7525	0	163	0.34	8410
B3GNT6_t1	1.09	0.01219326	0.08	120	5.8	5047	NA	NA	NA	NA	0.11	121	7.77	4721
ZNF346_t1	1.04	0.000337657	0.08	122	5.8	5048	0.11	154	11.03	2463	NA	NA	NA	NA
SPERT	1.08	0.000126633	0.15	88	5.8	5049	0.01	108	0.49	8146	0.25	126	18.83	1496
ORC4_t1	1.02	0.272768316	0.06	146	5.8	5050	NA	NA	NA	NA	0.11	127	8.18	4546
RAPGEF6	1	0.999261464	0.06	146	5.8	5051	0.06	137	5.22	5211	0.13	136	11.57	3332
ADAMTS9_t1	1.06	0.0052982	0.09	116	5.8	5052	NA	NA	NA	NA	0.08	111	4.93	5959
LETM1	1.1	0.002457248	0.05	160	5.8	5053	-0.03	142	-2.75	10315	0.12	134	10.13	3823
GCC1_t1	1.07	0.026166916	0.07	133	5.8	5054	NA	NA	NA	NA	0.14	122	9.96	3891
SGOL1_t2	1.18	2.31E-12	0.09	111	5.79	5055	0.1	108	5.79	4900	0.18	143	16.78	1880
CHEK2	1.37	5.13E-08	0.11	104	5.79	5056	0.11	113	6.84	4334	0.21	131	16.87	1858
AP5B1_t1	1.05	0.005519708	0.06	152	5.79	5057	NA	NA	NA	NA	NA	NA	NA	NA
COPS4_t1	-1.08	0.074007014	0.11	104	5.79	5058	NA	NA	NA	NA	0.05	111	2.95	6966
ZNF500	1.19	3.42E-08	0.06	149	5.78	5059	0.01	136	1.26	7636	0.08	134	6.87	5077
ADD1_t1	1.09	1.81E-06	0.08	124	5.78	5060	0.06	124	4.08	5859	0.1	135	8.18	4542
SIKE1	1.07	4.83E-05	0.06	139	5.78	5061	0.1	142	8.79	3379	0.12	121	8.25	4513
TTN_t1	1.01	0.1266537	0.08	127	5.78	5062	0	143	-0.41	8753	0	128	0.21	8488
GLYR1	1.01	0.190037805	0.08	120	5.78	5063	-0.05	131	-4.39	11312	-0.07	135	-5.83	11377
CCDC148	1.05	0.002499619	0.08	122	5.78	5064	0.11	123	7.77	3845	0.03	120	2.07	7416
BVES	1.03	0.0053943	0.07	137	5.78	5065	0.01	152	0.61	8064	0.07	126	5.18	5835
RCE1	1.06	0.000587735	0.1	109	5.78	5066	0.07	145	6.67	4412	0.09	130	7.18	4959
MRPL49	1.13	7.58E-06	0.07	129	5.77	5067	0.07	127	5.22	5212	0.09	124	6.3	5319
ARMC2	1.06	8.75E-05	0.11	104	5.77	5068	0.14	108	8.04	3724	0.15	109	8.8	4302
NUP62CL	1.08	0.02306788	0.07	128	5.76	5069	0.03	134	2.44	6867	0.09	151	8.7	4342
OPN3	1.11	0.039667862	0.08	124	5.76	5070	-0.03	115	-1.85	9728	0.17	129	13.69	2679
PLOD2	-1.1	0.267010866	0.06	148	5.76	5071	0.06	124	4.54	5589	0.12	120	8.36	4478
PIN4	1.22	3.22E-10	0.09	115	5.76	5072	0.12	122	8.41	3538	0.24	125	18.33	1588
GAS2L3	1.26	7.44E-11	0.19	80	5.76	5073	0.23	109	13.4	1716	0.27	64	3.84	6504
CADPS	1.1	0.011087294	0.09	114	5.76	5074	0.11	119	7.77	3850	-0.01	141	-0.5	8914
PLEKHG5	-1.05	0.224820131	0.07	132	5.76	5075	0.02	135	2	7141	-0.08	124	-5.6	11273
RHPN1	1.18	6.30E-07	0.09	114	5.75	5076	0.06	132	5.17	5239	0.12	109	7.13	4981
ZSCAN2_t1	1.2	4.13E-06	0.09	116	5.75	5077	0.05	96	2.13	7055	0.12	115	7.93	4652
TMUB2_t1	1.11	8.19E-07	0.1	110	5.75	5078	NA	NA	NA	NA	0.03	135	2.71	7076
ELMO2	1.05	0.141064091	0.08	120	5.74	5079	0.09	142	8.47	3522	0.03	118	1.8	7566
KIAA0930	1.11	0.031343874	0.06	143	5.74	5080	0.13	141	11.51	2302	0	134	0.13	8537
VPS13A_t1	1.23	0.000235588	0.06	145	5.74	5081	0.14	128	10.94	2500	0.21	141	18.74	1514
STK19	1.18	4.33E-06	0.11	100	5.74	5082	-0.02	104	-1.27	9346	0.02	112	1.08	7979
MANSC1	1.03	0.452031132	0.09	113	5.74	5083	0.08	127	6.39	4571	0.02	112	1.06	7988
LRRCC1	1.21	0.000177733	0.09	117	5.74	5084	0.09	115	5.93	4823	0.22	125	16.59	1935
UGGT2_t1	1.1	0.006327953	0.1	106	5.74	5085	NA	NA	NA	NA	0.14	111	8.4	4466
DIO2_t1	1.09	1.07E-10	0.08	126	5.74	5086	NA	NA	NA	NA	0.24	127	18.19	1610

RFPL3-AS1	1.09	0.000165971	0.08	122	5.74	5087	0.09	133	7.36	4058	0.12	116	8.23	4524
ZNF124	1.01	0.880265967	0.06	140	5.73	5088	0.07	137	6.32	4611	0	115	-0.05	8649
RHOXF2_t1	1.53	4.99E-05	0.11	104	5.73	5089	NA	NA	NA	NA	0.17	115	11.22	3435
PUS1	1.4	1.62E-14	0.14	92	5.72	5090	0.31	95	13.98	1538	0.2	137	17.18	1791
ITGB1_t1	1.07	0.407577317	0.08	125	5.72	5091	0.18	136	15.5	1146	0.1	125	7.4	4870
CRISP2	1.06	0.04306635	0.07	133	5.72	5092	0.01	125	0.76	7964	-0.02	122	-1.56	9476
PRR3	1.08	1.78E-08	0.08	126	5.72	5093	0.05	136	4.29	5721	0.09	122	6.56	5207
SPCS3	1.03	0.605094933	0.06	152	5.71	5094	0.02	140	1.7	7354	-0.05	155	-4.76	10968
TRAPPC11_t1	1.01	0.699640233	0.09	112	5.71	5095	-0.03	118	-1.78	9679	0.07	122	5.29	5783
KIAA0195	1.11	0.000338562	0.08	118	5.71	5096	0.12	135	9.84	2917	0.06	119	4.35	6269
SAMD4B	1.05	2.85E-06	0.09	112	5.71	5097	0.02	133	1.91	7210	0.15	119	10.66	3626
MC1R_t1	1.07	0.000851273	0.1	106	5.71	5098	NA	NA	NA	NA	0.09	108	5.48	5698
GABPB2	1.14	0.000650132	0.09	113	5.71	5099	0.13	99	6.49	4523	0.07	142	6.79	5108
GNG3	1.02	0.091595644	0.09	115	5.71	5100	0.09	128	6.88	4312	0.11	119	7.38	4879
HECW1	1	0.689476601	0.08	121	5.7	5101	0.1	136	8.87	3347	0.02	128	1.39	7810
SLC2A6	1.1	0.00384515	0.06	140	5.7	5102	0.11	139	9.97	2872	-0.01	153	-0.81	9089
ZMYM3_t1	1.1	2.75E-06	0.07	128	5.69	5103	0.09	134	7.98	3757	NA	NA	NA	NA
CDC42SE2_t1	1.06	0.070540251	0.05	155	5.69	5104	NA	NA	NA	NA	0.01	135	1.13	7953
PTCD2	1.05	0.010810078	0.07	126	5.69	5105	0.12	127	9.23	3175	0.19	132	15.89	2102
FZD2	-1.01	0.821871858	0.08	121	5.69	5106	0.11	120	7.63	3918	-0.13	122	-9.32	12690
KSR2	-1.01	0.224829427	0.07	132	5.69	5107	-0.06	152	-6.57	12449	0	155	-0.16	8707
EMC7	1.25	1.26E-05	0.07	132	5.68	5108	0.02	106	1.28	7621	0.22	137	18.97	1463
UNC45A	1.18	6.25E-09	0.1	108	5.68	5109	0.06	110	3.84	5985	0.13	112	8.18	4543
GLO1	1.11	0.05342684	0.07	131	5.68	5110	0.02	141	1.52	7485	0.18	132	15.16	2287
ATXN2L_t2	1.02	0.613659925	0.08	121	5.68	5111	NA	NA	NA	NA	0.05	115	3.4	6719
OPRM1_t1	1.03	0.000286009	0.09	113	5.68	5112	0.07	112	4.21	5773	0.13	127	9.98	3885
PCDH15	1.01	0.507051208	0.06	143	5.68	5113	0.02	112	1.3	7604	0.01	130	0.78	8147
GPATCH8	1.12	1.84E-06	0.08	121	5.67	5114	0.08	119	5.31	5164	0.15	152	15.16	2291
SLMO1	1.09	0.07918096	0.08	124	5.67	5115	0.14	106	7.83	3821	0.05	94	2.2	7347
IST1	-1.02	0.606031651	0.07	136	5.67	5116	0.09	141	8.17	3661	-0.04	134	-3.39	10353
PLAG1	1.33	0.000312605	0.06	138	5.67	5117	0.21	140	19.03	514	0.12	150	12.4	3068
SYTL5	1.08	2.02E-10	0.05	159	5.67	5118	0.02	113	1.47	7507	0.13	134	11.09	3480
RNF183	1.09	0.000196283	0.09	110	5.66	5119	0.14	122	10.4	2716	0.16	143	15.24	2261
NPAS4	1.06	1.23E-05	0.08	119	5.66	5120	0.06	122	4.67	5512	0.09	121	6.36	5292
RTP1_t1	1.05	2.67E-05	0.08	120	5.65	5121	NA	NA	NA	NA	0.11	122	7.79	4715
NCK1	-1.02	0.633517189	0.07	133	5.65	5122	0.02	105	0.93	7849	0.07	116	4.51	6172
EIF2B4	1.31	2.29E-17	0.18	81	5.65	5123	0.13	141	11.72	2225	0.21	65	3.18	6842
ZBTB1_t1	-1.09	0.003810949	0.05	155	5.65	5124	NA	NA	NA	NA	-0.01	131	-0.48	8895
HPDL	1.13	4.29E-06	0.09	111	5.64	5125	0.03	135	2.18	7017	0.1	126	7.92	4654
KREMEN2	1.08	0.008048952	0.09	112	5.64	5126	-0.08	88	-3.1	10543	0.1	119	6.71	5153
HOXC4	1.01	0.1347087	0.05	155	5.64	5127	-0.1	125	-7.21	12740	0.05	130	4.23	6327
TLR7	1.03	0.648675984	0.07	134	5.64	5128	0.11	115	7.38	4043	0	140	-0.41	8858
RNPC3	1.06	0.099910104	0.1	105	5.64	5129	0.05	84	1.55	7464	0.14	99	6.74	5131
ANK1	1.01	0.307470468	0.08	120	5.63	5130	-0.02	148	-2.23	9986	0.03	128	2.07	7412
HRH4	1	0.791847216	0.07	136	5.63	5131	-0.04	131	-2.94	10434	0.08	154	8.57	4393
MAST2	1.28	2.97E-11	0.11	103	5.63	5132	0.12	125	9.04	3254	0.22	146	20.8	1157
LDLRAD1	1.45	2.57E-06	0.15	88	5.63	5133	0.05	111	2.91	6546	0.14	117	9.67	3996
ADARB1_t1	1.01	0.17336833	0.08	122	5.63	5134	0.09	141	7.84	3811	-0.01	130	-1.14	9265
KRI1	1.19	8.89E-10	0.07	128	5.62	5135	0.14	135	12.11	2100	0.09	129	7.23	4933
ATF6B	1.18	3.05E-11	0.07	129	5.62	5136	0.12	119	8.17	3660	0.05	104	2.9	6988
NEB	1.99	6.96E-11	0.11	102	5.62	5137	0.15	114	9.32	3138	0.2	111	11.9	3233
RANBP17	1.08	0.255486595	0.07	127	5.61	5138	0.12	122	8.48	3512	0.05	128	3.64	6593
COL24A1	1.43	1.97E-09	0.07	130	5.61	5139	0.12	125	9.19	3194	0.15	120	10.64	3636
HSPD1	1.37	0.000295851	0.09	114	5.61	5140	0.16	148	15.96	1058	0.2	118	13.91	2614
RPL17	1.31	1.81E-06	0.2	78	5.61	5141	0.2	69	3.87	5962	0.19	84	6.6	5191
PPM1E	1.04	0.000414004	0.07	134	5.6	5142	0.12	146	11.82	2185	0.05	138	4.58	6136
ST14	-1.06	0.317588851	0.1	108	5.6	5143	0.05	120	3.71	6056	-0.15	100	-7.71	12118
CINP	1.06	0.035052822	0.1	106	5.6	5144	-0.04	103	-2.29	10016	0.07	90	2.92	6978
TRMT44	1.01	0.573129216	0.1	105	5.6	5145	-0.03	122	-2.43	10103	0.03	123	2.44	7213
CUBN	1.02	0.042758097	0.06	144	5.6	5146	-0.03	155	-3.48	10789	-0.02	121	-1.45	9422
PHF20_t1	1.05	0.067911897	0.07	135	5.6	5147	0.08	136	7.21	4136	NA	NA	NA	NA
MAPRE2	-1	0.861706689	0.06	141	5.6	5148	0.04	131	3.12	6426	0.03	147	2.72	7074
BAG5	-1.01	0.43693703	0.07	131	5.59	5149	0	144	-0.35	8714	0.06	154	6.33	5306
MARCH6_t1	1.1	0.000216937	0.06	138	5.59	5150	NA	NA	NA	NA	NA	NA	NA	NA
RBMY1B	1.73	1.46E-07	0.07	134	5.59	5151	0	110	0.26	8281	0.18	112	11.24	3431
MBD3L2	1.02	0.024707869	0.07	126	5.59	5152	0.14	138	12.13	2090	0.17	98	8.17	4550
HIST1H2BD	1.69	6.73E-16	0.28	70	5.59	5153	0.09	97	4.17	5796	0.25	95	11.47	3361
GCFC1	1.05	0.025624582	0.12	96	5.59	5154	0.12	107	7.03	4229	0.15	98	7.39	4875
ADCK5	1.3	6.58E-09	0.16	84	5.59	5155	0.07	146	7.18	4151	0.14	136	12.28	3113
SLC25A52	1.11	4.22E-09	0.25	72	5.58	5156	0.14	81	4.42	5651	0.31	95	13.92	2611
CASC4_t1	1.01	0.886247116	0.07	130	5.58	5157	NA	NA	NA	NA	0.18	142	16.13	2043
POLR3E	1.23	1.69E-06	0.07	129	5.58	5158	0.22	138	19.52	453	0.12	142	11.1	3475
RNPEP	1.13	0.001576601	0.09	110	5.58	5159	0.02	104	1.17	7697	0.15	101	7.4	4869
MRPL44	1.04	0.212126169	0.08	120	5.58	5160	0.05	121	3.34	6291	0.08	122	5.88	5503
ABL1_t2	1.03	0.000342666	0.07	129	5.58	5161	NA	NA	NA	NA	0	133	0.27	8460
IL12B	1.03	0.001211741	0.07	125	5.58	5162	-0.01	133	-0.66	8910	0.08	139	6.8	5106
SMAD3	1.24	1.23E-05	0.08	121	5.58	5163	0.12	134	9.69	2979	0.19	138	16.49	1958
RTN2	1.19	0.000418149	0.08	122	5.58	5164	0.08	122	5.7	4953	0.06	120	4.04	6395
KCNK16	1.11	2.63E-06	0.11	100	5.57	5165	0.01	120	0.44	8180	0.08	89	3.09	6875
HHLA3_t1	1.05	0.040320361	0.11	100	5.57	5166	-0.03	134	-2.22	9974	0.06	113	3.47	6684
SPCS1	1.37	9.23E-12	0.09	114	5.57	5167	0.17	114	11.14	2420	0.22	130	17.47	1741
SERF2	1.11	0.004946463	0.07	130	5.57	5168	0.12	138	10.49	2682	0.1	120	6.97	5048
QSOX2	1.16	2.61E-05	0.08	121	5.57	5169	0.11	124	7.84	3808	0.08	127	6.34	5304
EAF2	-1.02	0.723805305	0.1	104	5.57	5170	0.11	94	4.72	5495	-0.08	118	-5.65	11295
KCNIP4_t1	1	0.708333388	0.09	115	5.57	5171	NA	NA	NA	NA	0.08	121	5.63	5608
PLEKHM1	1.02	0.117008866	0.07	127	5.56	5172	0.07	144	6.8	4353	-0.01	120	-0.94	9161
JMJD6	1.2	5.14E-06	0.1	108	5.56	5173	0.15	111	9.26	3159	0.11	102	5.53	5671
DRG1	1.24	1.39E-08	0.1	104	5.56	5174	0.15	97	6.85	4328	0.17	102	9	4226
MCF2L2_t1	-1.01	0.580324431	0.07	131	5.56	5175	0.12	131	9.97	2870	NA	NA	NA	NA
ACAD10	1.05	0.000410892	0.1	107	5.56	5176	0.09	108	5.17	5235	0.12	108	7.04	5015
ANKRD5	1.16	9.36E-07	0.09	113	5.56	5177	0.01	114	0.51	8135	0.25	114	15.72	2148
ARPC3	1.16	0.000873131	0.06	145	5.55	5178	0.27	136	23.41	134	0.01	105	0.64	8231
ANKRD54	1.23	3.92E-12	0.08	124	5.55	5179	0.12	124	9.08	3242	0.18	120	12.89	2926
TYK2_t1	1.1	7.04E-05	0.08	120	5.55	5180	NA	NA	NA	NA	0.04	100	2	7449
CATSPER2	1.21	1.45E-05	0.11	101	5.55	5181	0.08	104	4.41	5658	0.13	127	10.34	3745
SMEK2	-1.01	0.910802919	0.07	130	5.55	5182	-0.04	132	-2.96	10458	0.12	124	8.87	4270
ATP2B3	1.01	0.281220815	0.07	134	5.55	5183	-0.02	133	-1.61	9563	-0.02	151	-2.5	9950
ZNF568	-1.01	0.871434225	0.06	139	5.55	5184	0.15	119	10.29	2762	0.08	120	5.85	5522
HOXC10_t1	1.04	0.0260001	0.07	127	5.55	5185	NA	NA	NA	NA	0.08	126	6.4	5275
POTEG	1.15	0.000268753	0.09	112	5.55	5186	-0.01	108	-0.59	8870	0.11	111	6.53	5222
EXOC1	1.03	0.51538064	0.07	126	5.54	5187	0.13	120	8.94	3309	0.1	128	7.75	4730
NOX3	1.05	4.48E-06	0.11	102	5.54	5188	0.06	129	4.45	5638	0.19	104	10.3	3761
ZMYM6_t1	-1.02	0.532315959	0.09	109	5.54	5189	NA	NA	NA	NA	0.11	122	8.27	4507
SDC2	1.14	0.087314361	0.08	116	5.53	5190	-0.07	130	-5.62	11983	0.19	132	15.92	2093
CTDP1_t1	1.06	0.091064211	0.09	109	5.53	5191	0.02	141	1.59	7436	-0.02	115	-1.42	9405
KHNYN	1.14	0.001157051	0.05	154	5.53	5192	0.09	139	8.37	3556	0.03	132	2.37	7254

KIF21A_t1	1.1	0.018574777	0.07	135	5.53	5193	NA	NA	NA	NA	0.23	117	15.28	2251
LYRM7_t1	1.02	0.286586392	0.05	152	5.53	5194	0.03	143	2.62	6744	NA	NA	NA	NA
SLC36A4	1.04	0.328889866	0.08	121	5.53	5195	0.1	144	9.25	3166	0.01	126	0.73	8179
KCNQ2_t1	1.04	0.000153717	0.12	98	5.53	5196	-0.05	104	-2.87	10393	0.01	99	0.34	8409
SKIDA1	1.27	0.000923175	0.08	122	5.53	5197	-0.02	135	-2.08	9885	0.23	148	22.94	850
RBM43	1.01	0.37748088	0.06	143	5.53	5198	0	147	0.34	8243	0.1	131	8.47	4438
SERPINB3	1.06	0.108922075	0.09	110	5.53	5199	-0.01	125	-1.07	9198	0.05	112	2.91	6984
ARL13B	1.04	0.140111994	0.07	127	5.52	5200	0.08	125	6.25	4640	0.15	153	15.54	2192
IDI2	1.07	2.02E-10	0.08	120	5.52	5201	0.07	116	4.73	5488	0.06	123	4.5	6183
ODAM	1.48	8.64E-07	0.07	127	5.52	5202	-0.14	114	-8.68	13388	0.16	134	13.78	2653
ZNF365	1.06	0.002100088	0.08	123	5.52	5203	0.11	131	8.96	3299	0.02	136	2.12	7384
KCNV1	1.03	0.005957762	0.06	135	5.52	5204	0	123	-0.22	8620	0.11	138	10.07	3850
TTLL5	1.06	0.000236696	0.08	120	5.51	5205	-0.04	132	-3.04	10495	0.15	147	14.96	2347
KLK3	1.02	0.007597175	0.07	124	5.51	5206	0.02	125	1.16	7699	0.05	107	2.94	6967
AIPL1	1.01	0.35821309	0.07	131	5.51	5207	-0.01	139	-1.22	9306	0.07	150	6.84	5093
LASP1	1.16	3.94E-05	0.07	130	5.5	5208	0.09	130	6.86	4323	-0.01	126	-0.58	8970
UTP6	1.21	2.13E-07	0.11	101	5.5	5209	0.15	115	9.93	2886	0.17	119	11.96	3212
KLF15	-1.01	0.916339302	0.07	133	5.5	5210	-0.13	109	-7.53	12889	0.09	122	6.47	5255
DCAF16	1.12	8.26E-10	0.06	136	5.5	5211	0.18	137	15.79	1092	0.19	122	13.55	2724
UCKL1	1.14	3.08E-07	0.12	94	5.5	5212	-0.02	113	-1.36	9406	0.21	104	11.4	3380
MTMR7	1.07	0.001721544	0.06	140	5.49	5213	0.09	133	7.18	4153	0.05	130	3.64	6598
TAX1BP3	1.01	0.817656819	0.08	121	5.49	5214	0.09	156	9.23	3173	-0.09	126	-7.16	11901
RSBN1	-1.02	0.103681766	0.06	147	5.49	5215	0.02	142	1.89	7219	-0.01	139	-0.56	8960
NKRF	1.04	0.266640707	0.06	144	5.49	5216	0.03	115	2.16	7036	0.14	119	9.9	3913
ANKHD1_t1	1.04	0.253556502	0.09	111	5.49	5217	-0.05	86	-1.93	9761	0.03	108	1.49	7753
INO80	1.06	0.001072512	0.09	108	5.49	5218	0.03	121	1.78	7295	0.14	164	15.62	2175
CRYGD	1.03	0.002333609	0.11	99	5.49	5219	0	93	0.03	8448	0.16	84	5.59	5625
OMG	-1	0.823357726	0.06	139	5.49	5220	0.08	135	6.91	4295	-0.02	130	-1.57	9486
AKT3_t1	1.02	0.150000222	0.07	129	5.49	5221	0.06	143	5.17	5236	0.02	140	1.89	7515
CHD1L	1.44	1.40E-10	0.09	109	5.49	5222	0.18	92	7.37	4049	0.28	111	16.91	1851
PCDHGA7	1.01	0.46365052	0.09	111	5.49	5223	-0.1	121	-6.91	12609	0.02	117	1.6	7688
PTPRJ	-1	0.871167993	0.06	148	5.48	5224	0.1	131	8.02	3738	0	150	-0.41	8859
MRPL3	1.11	0.000797748	0.08	118	5.48	5225	0.06	127	4.65	5519	0.1	99	4.67	6086
FRRS1	1.04	0.253047109	0.09	110	5.48	5226	-0.13	104	-6.82	12571	0.19	136	16.23	2023
EIF3K	1.26	4.34E-12	0.18	81	5.48	5227	0.11	80	3.31	6313	0.22	75	5.6	5622
EBAG9	1.08	0.11958191	0.07	130	5.48	5228	-0.02	130	-1.91	9751	0.15	125	11.08	3489
SPAG6_t2	1.05	0.009344225	0.07	132	5.48	5229	-0.14	138	-12.28	14576	0.03	130	2.47	7199
KHDRBS1	1.01	0.692729312	0.08	118	5.47	5230	0.12	132	10.12	2821	0	131	-0.03	8638
PIGK	-1	0.990764539	0.06	140	5.47	5231	0.03	129	2.72	6665	0.15	153	15.83	2124
PLEKHN1	1.15	0.000355681	0.09	109	5.47	5232	0.14	120	9.52	3051	0.08	96	3.55	6643
DCAF12L1	1.02	0.274550082	0.06	135	5.47	5233	0	125	0.07	8419	0.1	141	9.1	4184
ADCY5	1.03	0.022495802	0.1	106	5.47	5234	0.07	127	5.13	5265	-0.01	132	-0.42	8866
LRFN3	1.14	4.47E-09	0.12	96	5.47	5235	0.09	105	4.98	5347	0.24	84	8.15	4560
MUS81_t1	1.14	4.40E-09	0.1	107	5.47	5236	NA	NA	NA	NA	0.15	120	10.58	3662
CACNA2D1	1.03	0.012603636	0.09	108	5.46	5237	0.1	132	8.36	3561	-0.07	139	-5.89	11406
INGX	1.13	7.90E-05	0.1	104	5.46	5238	0.01	131	0.53	8120	0.12	120	8.23	4523
GNPDA1	1.04	0.32750089	0.08	117	5.46	5239	0.02	164	2.85	6587	-0.02	121	-1.36	9368
PAFAH1B3	1.96	2.90E-18	0.3	68	5.45	5240	0.17	125	12.79	1903	0.29	96	13.52	2733
RBM17	1.06	0.09862601	0.06	144	5.45	5241	0.1	115	6.46	4536	0.03	140	2.38	7242
NPY2R	1.05	0.02790389	0.07	125	5.45	5242	-0.04	130	-3.36	10712	-0.01	143	-1.26	9319
APOA2	1.61	0.001836358	0.16	85	5.45	5243	0.09	104	4.59	5559	0.21	88	7.91	4659
FAM47B	1.01	0.432446887	0.09	111	5.44	5244	-0.02	93	-0.72	8947	0.03	108	1.61	7677
SSX4B	1.61	1.28E-09	0.1	107	5.44	5245	0.06	122	4.15	5811	0.23	118	15.49	2198
RTEL1	1.22	5.46E-12	0.1	104	5.44	5246	0.08	104	4.44	5641	0.15	88	5.69	5583
PRR18	1.01	0.367698513	0.06	147	5.43	5247	0	120	-0.06	8525	0.01	143	0.59	8263
SAP30BP	1.11	0.000945474	0.08	122	5.43	5248	0.06	134	4.88	5403	-0.04	121	-2.54	9969
ALKBH1	1.08	0.0006822	0.06	134	5.43	5249	0.01	129	0.75	7968	0.11	113	6.97	5045
CNIH3	1.12	0.002849476	0.08	114	5.43	5250	0.1	123	7.12	4182	0.11	127	8.21	4532
SMC3	1.02	0.683676002	0.08	118	5.43	5251	0.09	108	5.13	5264	0.1	126	7.47	4845
KIAA0528_t1	1.01	0.88735873	0.08	120	5.42	5252	NA	NA	NA	NA	0.1	161	10.67	3624
RHOBTB3	1.04	0.506611001	0.06	140	5.42	5253	-0.05	135	-4.46	11363	0.22	125	16.13	2044
HSPBP1_t1	1.11	5.55E-08	0.1	104	5.42	5254	NA	NA	NA	NA	NA	NA	NA	NA
HES2	1.11	7.34E-05	0.07	132	5.41	5255	-0.04	131	-3.42	10743	0.03	136	2.97	6945
SPATA17	1.14	2.91E-05	0.07	127	5.41	5256	0.02	94	0.72	7985	0.11	104	6.11	5396
SSX5	1.05	0.001736913	0.09	108	5.41	5257	0	122	-0.3	8676	0.12	123	8.95	4245
B3GNT1	1.08	0.120596698	0.08	119	5.41	5258	-0.04	118	-3.04	10497	0.04	100	1.86	7527
LRRC23	1.06	0.023960584	0.11	100	5.41	5259	0.1	124	7.74	3863	0.03	94	1.38	7821
MCM3AP	1.17	4.10E-06	0.06	143	5.4	5260	0.13	99	6.48	4527	0.06	120	4.22	6334
WDR60	1.13	9.28E-06	0.08	118	5.4	5261	0.15	125	10.91	2515	0.17	112	10.66	3633
RPL39L	1.53	1.44E-05	0.1	103	5.4	5262	0.16	98	7.7	3882	0.08	129	6.18	5369
PCDH11Y_t1	-1.03	0.000489891	0.06	141	5.4	5263	-0.04	131	-3.27	10652	NA	NA	NA	NA
FAM160B1	-1.04	0.192060334	0.05	149	5.4	5264	-0.05	143	-4.58	11429	0.09	144	8.22	4526
SLC38A1	1.51	0.000148017	0.07	129	5.4	5265	0.26	132	21.11	276	0.12	123	8.8	4303
LGALS3	1.1	0.437938299	0.09	111	5.4	5266	0.19	122	13.36	1728	0.01	96	0.28	8450
WTAP_t2	1.03	0.456761788	0.06	138	5.39	5267	0.18	134	15.37	1176	NA	NA	NA	NA
MCRS1	1.18	2.96E-11	0.1	104	5.39	5268	0.1	121	7.15	4165	0.1	100	4.98	5937
TTC21B	-1	0.940997662	0.06	138	5.39	5269	0	123	0.13	8381	0.1	137	8.82	4295
PCF11	1.05	0.001329001	0.07	131	5.39	5270	0.11	111	6.69	4407	0.11	149	10.71	3608
WASL	1.04	0.002327889	0.07	131	5.39	5271	0.11	124	7.92	3784	0.15	162	16.3	2008
PIGH	1.15	3.18E-05	0.07	128	5.39	5272	0.02	127	1.77	7302	0.14	116	9.04	4211
MAEL	1.35	3.81E-07	0.07	124	5.39	5273	0.07	105	3.94	5934	0.15	151	15.11	2304
S100A1	1.14	1.23E-05	0.15	86	5.39	5274	0.04	118	2.43	6870	0.03	97	1.28	7865
CXorf61	1.14	0.000598194	0.16	84	5.38	5275	0.05	119	3.2	6383	0.05	80	1.46	7777
RPS18	1.44	6.64E-08	0.16	84	5.38	5276	0.2	75	5.09	5276	0.21	78	5.84	5526
GPR113	1.02	0.221447768	0.05	149	5.38	5277	-0.17	136	-14.92	15188	0	107	-0.09	8669
OR51E2_t1	1.06	8.41E-08	0.07	124	5.38	5278	0.11	147	10.88	2522	NA	NA	NA	NA
ARL5B	-1.12	0.00845484	0.05	150	5.38	5279	-0.04	132	-3.08	10521	0.02	130	1.62	7668
ATP1A4	1.22	1.55E-06	0.07	126	5.38	5280	0.05	122	3.7	6061	0.07	100	3.73	6552
MAPKBP1	1.2	3.87E-07	0.06	134	5.37	5281	0.16	144	14.59	1382	0.14	150	14.4	2486
DRAM2_t1	1.03	0.581028823	0.08	119	5.37	5282	0.14	119	9.54	3039	NA	NA	NA	NA
STARD13	1	0.888965401	0.06	134	5.37	5283	0	125	0.01	8466	0	160	0.35	8401
LHFPL4	1.16	0.001464753	0.06	143	5.36	5284	0.03	132	2.41	6885	0.1	119	6.6	5189
CPQ	1.24	0.000230757	0.08	119	5.36	5285	-0.01	127	-0.99	9143	0.15	113	9.46	4065
CDCA7L	1.22	5.73E-05	0.08	120	5.36	5286	0.1	136	8.55	3476	0.01	125	0.62	8244
OTUD7A	1.04	0.007497711	0.11	100	5.36	5287	-0.01	117	-0.73	8949	0.01	106	0.42	8359
PCDHB16	1.28	2.96E-06	0.08	120	5.36	5288	0.13	128	10.14	2809	0.04	121	3.17	6852
ATG9B_t1	1.05	6.44E-06	0.08	116	5.35	5289	0.05	140	4.16	5806	NA	NA	NA	NA
LENG8_t1	1.09	2.59E-11	0.09	111	5.35	5290	NA	NA	NA	NA	0.04	163	4.09	6376
SEPT9_t1	1.14	2.74E-10	0.07	122	5.34	5291	NA	NA	NA	NA	0.09	120	6.25	5333
OR51E1	1.08	1.75E-07	0.07	132	5.34	5292	0.16	119	11.3	2362	0.05	143	4.97	5942
PLCH1_t1	1.07	0.000426443	0.08	117	5.34	5293	0.06	122	4.14	5816	NA	NA	NA	NA
ATP6V0A1	1.1	0.020502361	0.07	123	5.34	5294	-0.01	134	-1.17	9270	0.1	126	7.75	4732
NPPC	1.03	0.167944899	0.07	126	5.34	5295	0.12	124	8.71	3407	0.05	114	3.22	6818
MSL3_t2	1	0.897417631	0.06	136	5.34	5296	0.11	104	6.2	4667	0.01	137	1.26	7872
KIF1B_t1	1.09	1.40E-07	0.06	134	5.34	5297	0.04	145	3.61	6108	0.19	152	18.87	1483
PPM1D	1.01	0.782944774	0.06	142	5.34	5298	-0.04	128	-3.08	10520	0.09	135	7.92	4655

ASTN1	1.04	0.00064349	0.07	127	5.32	5299	-0.08	127	-6.53	12428	0.08	120	5.88	5505
RAPGEF1	1.03	0.006324462	0.07	123	5.31	5300	0.08	123	5.65	4982	-0.03	123	-2.21	9798
RASA4_t1	-1	0.859426332	0.07	131	5.31	5301	NA	NA	NA	NA	NA	NA	NA	NA
MARK1	1.06	0.005803141	0.06	145	5.31	5302	0.17	161	18.71	556	0.09	126	7.05	5006
MED23_t2	1.08	0.000495581	0.07	124	5.31	5303	0.18	127	13.91	1557	0.12	128	9.55	4040
MUTYH_t1	1.09	0.001126591	0.13	91	5.31	5304	0.05	104	2.59	6764	0.04	102	2.18	7358
ACSF2	-1.02	0.677620229	0.07	123	5.31	5305	-0.12	131	-9.59	13733	0.03	104	1.62	7676
RFT1	1.13	5.40E-05	0.06	143	5.31	5306	0.01	132	1.02	7800	0.09	146	8.31	4491
PRPF39	1.04	0.431153432	0.06	133	5.3	5307	0.16	115	10.32	2751	0.09	141	7.87	4677
FAM98C	1.16	1.99E-08	0.07	129	5.3	5308	-0.06	100	-3.11	10556	0.11	103	5.86	5514
CCDC107	1.21	1.56E-06	0.08	117	5.3	5309	0	120	-0.27	8648	0.19	109	10.92	3539
NCAM1_t1	1	0.789641163	0.07	127	5.3	5310	NA	NA	NA	NA	0.08	135	7.18	4956
HRH3	1.01	0.581702235	0.08	120	5.3	5311	-0.05	133	-3.85	11007	0.09	117	6.16	5374
TGFBRAP1	1.07	0.000180801	0.09	110	5.3	5312	0.13	100	6.43	4553	-0.02	88	-0.94	9159
CDK2	1.06	1.87E-05	0.07	131	5.3	5313	0.1	121	7.15	4169	0.13	122	9.5	4055
HORMAD1	1.02	0.223616671	0.09	111	5.3	5314	0.02	116	1.57	7450	0.03	152	3.32	6764
EML4	1.09	0.273915759	0.06	132	5.3	5315	0.11	147	11.14	2423	0.05	131	3.67	6582
ELMO1	1.06	0.108890043	0.06	132	5.29	5316	-0.05	138	-4.07	11135	0.07	152	7.2	4947
PHAX	1.12	0.00918971	0.06	140	5.29	5317	0.02	135	1.72	7334	0.26	147	25.41	572
LRRC37A3	1.25	1.39E-08	0.14	88	5.29	5318	0.25	111	15.3	1192	0.25	101	12.56	3019
LIG3_t1	1.14	8.40E-13	0.16	83	5.28	5319	NA	NA	NA	NA	0.13	98	6.13	5385
GPBP1	1.03	0.628619116	0.06	142	5.28	5320	0.04	162	4.28	5722	0.2	143	18.22	1606
PDE6H	1.02	0.019010186	0.08	120	5.28	5321	-0.01	131	-1.18	9276	0.09	114	5.49	5686
SCRT1	1.05	0.00031587	0.06	138	5.27	5322	-0.04	141	-3.95	11070	0.1	153	10.44	3709
UGT2A3	1.01	0.904833071	0.07	129	5.27	5323	0	127	-0.2	8610	0.12	123	8.91	4259
ATP6V0D1	1.03	0.367794573	0.11	96	5.26	5324	-0.07	119	-4.88	11581	-0.04	146	-4.12	10685
TAF7	1.08	0.224978498	0.1	102	5.26	5325	0.03	100	1.7	7356	0.17	132	13.74	2666
MED13_t1	1.09	9.24E-05	0.06	141	5.26	5326	NA	NA	NA	NA	0.17	137	14.54	2449
DPP10_t1	1.01	0.415741352	0.05	147	5.26	5327	NA	NA	NA	NA	0.07	138	6.5	5232
SOSTDC1	1.1	0.005268622	0.07	126	5.26	5328	-0.02	130	-1.86	9735	0.05	140	4.56	6146
EIF2A_t1	1.04	0.359206833	0.06	131	5.26	5329	0	139	-0.04	8516	NA	NA	NA	NA
PDZD9	1.01	0.251935732	0.09	109	5.25	5330	-0.01	120	-0.49	8810	-0.03	115	-1.99	9704
TMEM174	1.11	1.59E-05	0.07	123	5.25	5331	-0.05	113	-3.21	10603	0.1	113	6.16	5373
PLEKHG2	1.02	0.76778018	0.06	135	5.25	5332	0.11	136	9.16	3203	0.02	124	1.58	7703
MMD	-1.01	0.87996829	0.05	149	5.24	5333	0.15	139	13.16	1783	-0.04	142	-4.07	10667
ZNF667	-1.02	0.59312395	0.06	132	5.24	5334	0.08	135	7.18	4152	0.04	128	2.87	7004
ADRBK2_t1	1.08	0.032846773	0.06	145	5.23	5335	0.15	170	18.35	608	NA	NA	NA	NA
AKNAD1	1.02	0.023066364	0.07	120	5.23	5336	-0.04	87	-1.43	9446	0.01	99	0.6	8256
SLC36A1	1.02	0.094839985	0.06	140	5.23	5337	-0.09	131	-7.58	12921	0.03	134	2.23	7338
LRRC8A	1.06	0.229120352	0.08	117	5.23	5338	-0.02	132	-1.32	9378	0.08	120	5.94	5475
TP73	1.07	2.24E-13	0.07	127	5.23	5339	0.03	129	2.56	6793	0.18	147	17.36	1756
ADSL	1.25	3.08E-06	0.1	103	5.23	5340	0.12	67	2.01	7134	0.14	93	5.97	5469
ANKRD13A_t1	1.06	0.081182589	0.07	122	5.22	5341	NA	NA	NA	NA	NA	NA	NA	NA
BSG	1.29	6.56E-10	0.09	109	5.22	5342	0.11	134	8.93	3311	0.08	110	4.99	5929
TEX15	-1.01	0.570398255	0.06	140	5.22	5343	0.04	121	2.49	6831	-0.03	117	-2.05	9731
GOLGA6A_t1	1.25	1.33E-07	0.08	113	5.22	5344	0.22	119	14.91	1305	NA	NA	NA	NA
AQP10	1.2	2.89E-09	0.08	113	5.22	5345	-0.04	110	-2.59	10211	0.19	101	9.66	4001
IFI27	1.12	0.460178775	0.08	114	5.21	5346	-0.1	109	-6.18	12274	-0.01	109	-0.58	8971
MAPK7	1.03	0.2567909	0.1	103	5.21	5347	0.05	127	3.65	6083	-0.02	128	-1.34	9359
TMX1	1.02	0.6822785	0.05	149	5.21	5348	-0.02	127	-1.28	9349	0.07	138	6.57	5201
PKIB	1.13	0.211388166	0.06	139	5.21	5349	0.06	150	6.47	4529.5	-0.02	127	-1.46	9427
HTT_t1	1.15	1.27E-05	0.08	114	5.21	5350	NA	NA	NA	NA	0.1	135	8.9	4260
ADAT3	-1.03	0.119344453	0.1	100	5.21	5351	-0.05	108	-2.91	10418	-0.01	91	-0.26	8769
FAM193A_t1	1.06	0.021464451	0.07	120	5.21	5352	NA	NA	NA	NA	0.06	138	4.93	5964
DDX53	1.02	0.100569437	0.08	119	5.2	5353	-0.04	107	-2.16	9936	0.24	112	14.76	2392
TMEM198	1.21	9.48E-09	0.09	105	5.2	5354	0.06	120	4.09	5848	0.05	100	2.71	7079
MPHOSPH10	-1.02	0.75151586	0.07	120	5.2	5355	0.1	103	5.56	5033	0.07	112	4.47	6197
WDR46	1.15	1.57E-06	0.1	104	5.2	5356	0.05	115	3.5	6185	0.11	84	3.67	6580
FOXG1	1.07	0.031483644	0.07	126	5.2	5357	0.07	119	4.65	5521	0.1	140	8.83	4286
RUVBL2	1.33	4.83E-14	0.15	84	5.19	5358	0.02	117	1.65	7386	0.16	94	7.13	4984
MAPKAPK2	1.1	0.000837383	0.07	122	5.19	5359	0.03	130	2.75	6640	0.13	126	9.64	4009
ABCA4	1.19	1.87E-06	0.09	109	5.18	5360	0.1	108	5.65	4981	0.14	120	10.02	3868
PSG11	1.02	0.048620276	0.08	116	5.18	5361	-0.02	108	-1.25	9329	0.07	128	5.22	5823
ACTR3B_t1	1.22	1.22E-07	0.07	122	5.18	5362	0.04	112	2.38	6901	0.19	126	14.15	2559
ACRV1	1.02	0.008994907	0.07	120	5.18	5363	0.05	122	3.45	6217	0.01	106	0.72	8180
CLSTN1	1.04	0.449475763	0.07	125	5.18	5364	0.14	160	15.16	1231	0	123	-0.21	8737
SEC11C	1.36	2.38E-06	0.07	125	5.17	5365	0.05	112	3.4	6249	0.25	107	14.04	2586
ZNF791	1.05	0.1732524	0.07	127	5.17	5366	-0.03	121	-2.4	10089	0.08	120	5.66	5597
RBM42	1.2	1.15E-11	0.16	83	5.17	5367	0.12	104	6.46	4538	0.07	87	2.7	7087
SLC37A1	1.13	0.002073498	0.09	110	5.17	5368	0.11	122	8.1	3695	-0.02	139	-1.41	9395
ROMO1	1.36	4.34E-16	0.12	94	5.17	5369	0.15	88	5.76	4919	0.24	104	13.07	2861
ALK	1.04	0.000599754	0.08	116	5.17	5370	0.08	129	6.51	4506	0.1	116	6.58	5198
INTS3	1.3	1.26E-11	0.08	118	5.17	5371	0.12	118	8.16	3664	0.1	109	6.11	5400
METTL12	1.14	6.15E-05	0.08	112	5.16	5372	0.13	116	8.89	3330	0.08	105	4.47	6202
RPL8	1.7	4.01E-15	0.29	68	5.16	5373	0.25	60	2.46	6849	0.28	103	14.85	2372
KIF13A	1.08	0.027777854	0.09	106	5.16	5374	0.13	116	8.31	3585	0.18	116	11.89	3238
C1GALT1	1.14	0.000929399	0.05	153	5.16	5375	0.13	129	10.22	2786	0.18	131	14.24	2539
RBMS2	1	0.845570999	0.06	135	5.16	5376	-0.03	149	-3.31	10683	-0.03	146	-2.4	9894
FAM90A1_t1	1.09	0.014175138	0.09	110	5.16	5377	NA	NA	NA	NA	0.02	111	0.96	8039
SCARA3_t1	1.08	2.53E-05	0.07	125	5.15	5378	0.08	117	5.69	4956	-0.04	86	-1.44	9412
EPHB2	1.15	1.67E-05	0.06	131	5.15	5379	0.01	137	0.68	8016	0.04	121	2.57	7146
GOLGA6L9	1.05	0.236122085	0.05	144	5.15	5380	0.12	118	8.12	3684	0.18	134	15.05	2324
TUBGCP4	1.04	0.14097046	0.06	136	5.15	5381	-0.07	125	-5.44	11896	0.09	144	8.9	4262
SPSB1_t1	-1.06	0.115152867	0.07	124	5.15	5382	NA	NA	NA	NA	0.01	134	1.1	7965
KIF7	1.08	0.00656121	0.07	120	5.15	5383	0.19	109	10.98	2483	0	104	-0.22	8745
EIF3D	1.28	1.02E-06	0.1	100	5.15	5384	0.26	95	11.87	2164	0.16	122	11.19	3448
RFPL1-AS1	1.03	0.227956143	0.06	139	5.14	5385	0.07	152	6.68	4410	0	150	0.41	8364
CYP20A1_t1	-1.01	0.586911717	0.05	155	5.14	5386	NA	NA	NA	NA	NA	NA	NA	NA
GRB10	1.11	0.039553302	0.05	154	5.13	5387	0.12	146	11.72	2226	0.08	146	7.71	4753
RPS6KL1_t1	1.14	2.56E-15	0.07	120	5.13	5388	NA	NA	NA	NA	0.15	120	10.77	3584
CADM4	1.02	0.029040497	0.08	113	5.13	5389	0.07	120	5.15	5251	0.06	131	4.65	6103
SLC25A29_t1	1.27	6.03E-07	0.09	104	5.13	5390	NA	NA	NA	NA	0.14	131	11.59	3327
ADAM20	1.04	8.52E-05	0.07	119	5.12	5391	0.01	125	0.63	8046	0.16	115	10.71	3612
TEF_t1	1.02	0.168222283	0.06	134	5.12	5392	NA	NA	NA	NA	0.06	133	5.23	5821
LRPAP1	1.12	0.002415942	0.06	143	5.12	5393	-0.11	142	-9.68	13767	0.16	153	16.36	1989
CXCL11	1.33	0.00012533	0.07	126	5.12	5394	0.06	133	4.72	5493	0.1	134	8.02	4609
ENTPD1	-1.05	0.198546712	0.07	127	5.12	5395	0.09	135	7.32	4080	-0.12	142	-11.34	13357
ZNF197_t1	1.06	0.000440032	0.07	121	5.11	5396	NA	NA	NA	NA	0.16	150	16.1	2049
PGC	1.48	2.24E-07	0.1	100	5.11	5397	0.08	117	5.08	5280	0.15	119	10.07	3848
BAG3	1	0.997081093	0.06	129	5.11	5398	0.04	112	2.32	6946	0.06	126	4.85	6000
KRTAP5-8	1.02	0.590296702	0.09	109	5.1	5399	-0.04	121	-2.78	10326	0	97	0.07	8574
PAM_t2	1.01	0.678114609	0.06	140	5.1	5400	0.07	140	6.07	4746	-0.02	123	-1.62	9511
SART3	1.09	8.52E-05	0.07	123	5.1	5401	0.11	135	9.75	2952	0	124	-0.01	8631
RRAGB	1.07	0.002488422	0.09	106	5.1	5402	0.03	118	1.9	7217	0.14	135	11.71	3295
IGDCC4	1.17	0.000263654	0.07	125	5.09	5403	0.13	134	10.7	2595	0.07	132	5.4	5731
KLK4	1.08	1.49E-07	0.11	97	5.09	5404	-0.07	120	-5.15	11742	0.14	106	7.63	4781

RHBDF2	1.34	1.70E-05	0.08	111	5.09	5405	0.29	112	17.87	691	0.04	121	3.14	6857
ZDHHC12	1.13	0.000183445	0.1	103	5.09	5406	-0.01	128	-0.81	9026	0.06	105	3.42	6706
NDUFB2	1.21	8.95E-05	0.13	88	5.09	5407	0.1	104	5.2	5219	0.22	138	19.18	1413
RHOA	1.2	3.45E-09	0.07	118	5.09	5408	0.28	142	25.37	71	0.14	128	10.91	3546
BARX1	1.09	0.002621564	0.09	108	5.09	5409	0.14	119	9.41	3107	0.09	101	4.43	6233
SGTA	1.16	8.39E-08	0.07	126	5.09	5410	-0.02	120	-1.44	9456	0.03	118	1.85	7533
SETDB2	-1.13	0.002755304	0.04	165	5.08	5411	-0.03	129	-2.45	10117	-0.1	142	-9.04	12601
ZNF665	1.04	0.393925667	0.07	120	5.08	5412	0.2	139	17.4	765	0.09	153	9.6	4023
SALL3	1.01	0.131639585	0.07	120	5.08	5413	-0.01	122	-0.84	9041	-0.01	161	-0.93	9152
RHBDL2_t1	1.1	0.006926442	0.06	136	5.07	5414	NA	NA	NA	NA	0.12	120	8.31	4487
LIPT1	1.06	0.02560097	0.07	120	5.07	5415	0.04	111	2.48	6840	0.05	126	3.66	6584
COG3	1.03	0.379035789	0.06	136	5.07	5416	0.03	129	2.39	6895	-0.03	120	-2.11	9761
MED30	-1	0.971088259	0.09	108	5.07	5417	0.04	104	2.36	6913	0.1	113	6.04	5434
HDAC11	1.08	8.90E-05	0.08	115	5.07	5418	-0.01	117	-0.76	8981	0.07	135	6.05	5431
TUBAL3	1.06	0.034578622	0.08	114	5.07	5419	-0.05	118	-3.1	10546	0.06	112	4.02	6407
LRRC45	1.46	5.05E-15	0.12	92	5.06	5420	0.09	107	5.13	5260	0.17	140	15.68	2159
NFYA	1.11	0.00235404	0.06	129	5.06	5421	0.13	141	11.76	2204	0.1	160	11.38	3390
CD93_t1	-1.1	0.085607717	0.05	148	5.06	5422	NA	NA	NA	NA	NA	NA	NA	NA
NUDT5	1.25	4.38E-06	0.14	87	5.06	5423	0.04	111	2.2	7011	0.22	117	14.72	2406
MKRN1_t1	1.08	0.006192025	0.07	120	5.06	5424	NA	NA	NA	NA	0.11	141	10.39	3728
KLHL21	1.29	1.91E-05	0.06	129	5.06	5425	0.11	127	8.53	3488	0.04	155	3.74	6550
CD59_t1	-1.09	0.166636402	0.06	137	5.06	5426	NA	NA	NA	NA	NA	NA	NA	NA
HIST1H2BF	1.64	3.10E-14	0.24	71	5.05	5427	0.07	95	3	6493	0.22	68	3.98	6421
TCP1_t1	1.23	4.76E-06	0.08	115	5.05	5428	NA	NA	NA	NA	0.16	119	10.77	3585
SEC13	1.14	0.000714563	0.06	128	5.05	5429	0.09	104	4.73	5482	0.11	100	5.6	5624
TPH2	1.04	0.001545056	0.05	143	5.04	5430	0.08	138	7.19	4147	0.15	115	9.72	3977
SLC13A1	1.04	0.000594818	0.06	128	5.04	5431	0.01	129	0.7	8000	0.05	147	5.25	5806
TRPS1	1.15	0.015869331	0.06	140	5.04	5432	0.16	147	15.8	1090	0.17	146	16.55	1944
MAGIX_t1	1.08	0.099440357	0.06	128	5.04	5433	NA	NA	NA	NA	0.11	127	8.4	4469
PMVK	1.25	9.98E-07	0.05	149	5.04	5434	0.05	139	4.67	5513	0.1	119	7.04	5016
AXIN2_t1	1.06	0.01412396	0.06	141	5.04	5435	NA	NA	NA	NA	0.02	149	2.2	7351
PPP1R14C	1.06	0.00658	0.07	123	5.04	5436	0.12	122	8.95	3303	0.12	136	10.36	3736
RNF31	1.29	7.08E-14	0.19	76	5.04	5437	0.08	106	4.69	5503	0.16	80	4.87	5990
RNF145_t1	-1.01	0.577725956	0.07	122	5.03	5438	NA	NA	NA	NA	0.06	147	6.07	5415
KHSRP	1.07	3.12E-06	0.08	112	5.03	5439	0.14	128	10.53	2665	0.1	114	6.18	5368
WRAP73	1.19	3.78E-11	0.07	122	5.02	5440	0.06	93	2.56	6788	0.08	120	5.55	5659
PNPLA8	1.02	0.263439379	0.07	123	5.02	5441	0.08	139	6.9	4301	0.06	138	5.48	5696
PAGE2B	1.78	7.73E-07	0.07	122	5.02	5442	-0.06	103	-2.94	10441	0.08	88	2.95	6961
MBTD1	1.05	0.00663213	0.05	143	5.02	5443	0.04	144	4	5904	0.07	129	5.27	5791
DKK1	2.01	6.16E-06	0.08	112	5.02	5444	0.07	112	4.37	5673	0.18	123	12.86	2938
TSPYL6	1.03	0.000761414	0.06	131	5.02	5445	0.14	123	10.47	2695	0.07	116	4.82	6021
AK1_t1	1.08	1.04E-05	0.07	126	5.01	5446	NA	NA	NA	NA	0.08	123	5.48	5694
MLST8	1.06	0.006038994	0.09	107	5.01	5447	0.02	150	1.74	7320	0.11	120	7.89	4667
COG6	-1.06	0.158050301	0.06	136	5.01	5448	0.03	125	1.95	7170	-0.02	114	-1.52	9454
B9D1	1.18	0.000299419	0.2	75	5.01	5449	0.11	99	5.6	5010	0.15	100	7.58	4802
TMEM234	1.11	0.000203425	0.09	108	5.01	5450	0.18	110	10.92	2508	0.11	118	7.46	4849
WDR89	1.01	0.687079644	0.06	128	5.01	5451	0	117	-0.09	8549	0.12	115	7.55	4813
AMDHD2	1.07	0.010514537	0.12	92	5	5452	0.04	80	1.09	7746	0.07	100	3.56	6634
SH3GLB2	1.05	0.09916422	0.08	111	5	5453	0.13	132	10.36	2734	-0.03	120	-2.28	9835
RAB27B	1.05	0.076334792	0.06	134	5	5454	0.08	147	7.92	3783	-0.08	149	-7.49	12030
FBLN7	1.09	0.002659818	0.07	126	5	5455	-0.05	109	-2.76	10319	0.03	132	2.54	7160
MRPL1	-1.08	0.058452695	0.05	159	4.99	5456	0	120	-0.05	8519	0.02	103	1	8018
ZDHHC6	-1	0.999909152	0.05	158	4.99	5457	-0.06	137	-5.35	11845	0.09	123	6.37	5287
IL17D	1.31	0.000201952	0.07	117	4.99	5458	0.02	132	1.71	7341	0.05	116	3.59	6619
PCM1_t1	-1.16	0.00301831	0.06	139	4.99	5459	NA	NA	NA	NA	NA	NA	NA	NA
SNX21_t1	1.06	0.06925912	0.05	149	4.99	5460	0.09	120	6.15	4702	0.08	120	5.37	5752
LDLRAP1	1.09	0.000487151	0.07	126	4.98	5461	0.07	116	4.75	5474	0.06	116	4.03	6400
SLC38A10_t1	1.24	4.24E-13	0.08	110	4.98	5462	0.11	137	9.94	2883	0.1	123	7.6	4795
GATA2_t1	1.02	0.137560109	0.07	123	4.98	5463	NA	NA	NA	NA	NA	NA	NA	NA
ZNF430_t1	-1.02	0.281065489	0.06	140	4.97	5464	0.18	155	18.88	538	NA	NA	NA	NA
CACNG1	1.09	0.002503175	0.09	104	4.97	5465	-0.09	108	-4.98	11649	0.12	138	10.21	3794
KIAA0825	1.01	0.123039238	0.06	133	4.97	5466	-0.02	116	-1.07	9196	0.1	139	8.59	4384
DYRK3	1.12	0.000740213	0.11	97	4.97	5467	0.01	92	0.22	8312	0.12	111	7.42	4860
POLE4_t1	1.04	0.264164715	0.11	97	4.97	5468	NA	NA	NA	NA	0.14	142	12.59	3007
RFPL4B	1.08	5.88E-05	0.08	115	4.97	5469	-0.01	106	-0.52	8830	0.14	126	10.29	3764
PAPLN_t1	1.08	3.19E-05	0.06	134	4.97	5470	NA	NA	NA	NA	0.01	128	1.03	8003
ZNF704_t1	1.08	0.000810001	0.05	152	4.96	5471	NA	NA	NA	NA	0.06	147	6.2	5360
COA3_t1	1.19	9.48E-06	0.07	119	4.96	5472	NA	NA	NA	NA	NA	NA	NA	NA
ASNS	1.3	0.043207794	0.08	112	4.96	5473	0.21	92	8.94	3307	0.01	98	0.51	8312
RBM46	1.04	0.066215151	0.05	146	4.96	5474	0	112	0.2	8331	0.08	135	6.49	5246
CYBRD1	1.13	0.051133561	0.06	136	4.96	5475	-0.01	157	-0.6	8871	-0.14	148	-14.13	14144
NRG2_t1	1.27	0.000561633	0.07	121	4.96	5476	0.06	130	4.65	5524	0.07	126	5.2	5830
CTAG1A	2.1	1.65E-07	0.08	109	4.95	5477	-0.01	88	-0.25	8641	0.12	94	5.37	5748
MYOCD	1.04	5.14E-05	0.07	123	4.95	5478	0.17	164	19.62	444	0.03	134	2.72	7069
SUPT5H	1.21	1.37E-11	0.1	102	4.95	5479	0.11	99	5.56	5031	0.05	113	3.35	6747
PRSS35	1.03	0.16846509	0.07	120	4.95	5480	0.07	125	5.4	5117	0.02	127	1.27	7866
CCDC110	1.1	5.64E-05	0.09	105	4.95	5481	0.15	116	9.59	3026	0.15	138	12.85	2940
FIGN	-1.01	0.690203866	0.06	128	4.95	5482	0.1	134	8.17	3659	0.06	128	4.3	6290
HOXD13	1.07	1.21E-07	0.06	128	4.95	5483	-0.05	117	-3.14	10566	0.07	129	5.44	5723
VCX	2.79	1.10E-11	0.08	112	4.94	5484	-0.03	108	-1.82	9701	0.18	103	9.7	3988
HIST1H2BC	1.78	1.84E-17	0.27	68	4.94	5485	0.1	124	7.58	3940	0.29	83	9.47	4064
SP8	1.15	0.000269363	0.06	132	4.94	5486	0.02	125	1.81	7266	0.07	146	6.94	5057
MICALL1	1.03	0.125136053	0.07	122	4.94	5487	0.05	114	3.35	6285	0.02	120	1.18	7925
HMGB3	1.23	0.000669955	0.05	148	4.94	5488	-0.04	130	-3.01	10476	0.2	124	14.86	2370
FAM111A_t1	1.21	7.41E-07	0.06	126	4.94	5489	NA	NA	NA	NA	0.18	135	15.43	2211
LRGUK	1.05	0.066098971	0.07	120	4.94	5490	0.12	107	6.85	4327	0.17	109	9.74	3971
DSC2	1.02	0.370073365	0.05	152	4.94	5491	0.1	140	8.8	3373	0.09	120	6.1	5402
CYLC1	1	0.922043661	0.09	104	4.94	5492	-0.13	101	-6.64	12482	0.08	100	3.79	6530
GPRC5A	1.08	0.013015291	0.07	121	4.94	5493	0.05	122	3.71	6051	0.05	151	5.13	5861
SRP72	1.06	0.319158909	0.06	132	4.93	5494	0.16	119	10.97	2486	0.14	126	10.65	3634
STYXL1	1.23	2.62E-06	0.1	99	4.93	5495	0.07	88	2.56	6795	0.15	84	5.04	5907
ABCB5	1.1	8.63E-05	0.06	134	4.93	5496	0.08	134	7.06	4215	0.13	126	9.81	3948
PSMG2	1.16	2.90E-07	0.08	113	4.92	5497	0.11	131	9.2	3190	0.1	116	6.71	5154
ANKS1B_t2	1.09	0.000732962	0.05	159	4.92	5498	0.06	125	4.75	5475	0.07	114	4.53	6160
FCGR2A	-1.1	0.096603625	0.06	127	4.92	5499	0.08	137	6.64	4423	-0.06	118	-4.28	10745
FGD6	1.02	0.680625056	0.06	139	4.92	5500	0.13	146	12.51	1977	0.11	131	8.63	4375
BSND	1.1	1.36E-06	0.1	99	4.92	5501	-0.04	102	-2.31	10030	0.12	108	7.16	4966
STT3A	1.12	0.011221455	0.07	121	4.92	5502	0.1	112	6.13	4712	0.03	130	2.64	7113
CD163L1	1.13	0.012770815	0.07	118	4.92	5503	0.09	110	5.59	5014	0.14	113	8.86	4274
GAB1	1	0.888397943	0.05	142	4.92	5504	0.07	142	6.61	4444	0.09	139	7.58	4799
RELB	1.27	0.000694332	0.07	120	4.91	5505	0.07	108	4.33	5704	0.07	126	5.18	5840
DBNL_t1	1.05	0.116337563	0.07	123	4.91	5506	0.1	120	7.25	4119	-0.01	114	-0.82	9095
FKBP6	1.14	1.87E-07	0.1	100	4.91	5507	0.13	127	9.79	2939	0.14	114	9.06	4204
EPS15_t1	-1.06	0.04537149	0.05	146	4.91	5508	NA	NA	NA	NA	-0.03	143	-2.34	9867
PITX1	1.19	1.02E-06	0.1	101	4.91	5509	0.17	126	13.28	1752	-0.01	131	-0.63	8998
PTPRS_t1	1.11	0.000142766	0.06	131	4.91	5510	-0.07	149	-6.62	12473	0.1	120	6.92	5065

SH3D21	1.15	1.61E-06	0.13	88	4.91	5511	0.16	113	10.05	2843	0.12	122	8.72	4337
ZNF512B	1.15	0.000436873	0.06	131	4.91	5512	0.05	140	4.12	5830	0.12	120	8.68	4353
ZNF417	1.06	0.000246623	0.06	129	4.9	5513	0.06	135	5.43	5103	0.04	153	4.58	6133
OSBPL8	1.01	0.618831046	0.06	136	4.9	5514	0.07	145	6.42	4556	0.09	154	9.74	3968
PSEN1	1.08	0.01030763	0.06	131	4.9	5515	-0.06	127	-4.31	11271	0.08	151	8.13	4563
PLAC1L	1.02	0.039500369	0.06	135	4.9	5516	0.1	128	7.81	3826	0.11	122	7.99	4624
CLTB_t1	1.07	0.00117178	0.09	103	4.9	5517	NA	NA	NA	NA	0.08	114	4.83	6009
SPTY2D1	-1.02	0.585190426	0.06	136	4.89	5518	0.04	140	3.79	6016	0.1	162	10.9	3550
TECPR2	1.07	0.094015969	0.04	160	4.89	5519	0.02	139	1.65	7390	0.04	125	2.87	7005
NRSN1_t1	1	0.775678586	0.06	126	4.89	5520	0	114	0.18	8348	NA	NA	NA	NA
PACS2	1.08	0.003482144	0.06	126	4.88	5521	0.06	138	5.66	4973	0.03	123	2.41	7229
ZC4H2	-1.02	0.812567064	0.06	129	4.88	5522	-0.07	120	-5.05	11686	0.07	129	5.25	5807
KLK12	1.01	0.118298836	0.08	110	4.88	5523	0.02	104	1.22	7667	0.02	93	1.04	8001
CSTL1	1.11	3.04E-07	0.11	93	4.88	5524	0.07	96	3.32	6301	0.08	120	5.26	5797
IFT27	1.27	5.96E-10	0.15	82	4.88	5525	0.03	77	0.68	8018	0.22	100	11.03	3504
CDH7	1.1	0.005619513	0.12	90	4.88	5526	0.14	69	2.7	6677	0.02	109	1.22	7897
PARD6B	1.18	0.00110789	0.06	133	4.88	5527	0.14	127	10.4	2717	0.21	135	17.52	1733
TNFRSF19_t1	1.08	0.000366607	0.1	98	4.88	5528	-0.03	90	-1.08	9210	0.02	116	1.56	7710
DGAT1	1.03	0.635914746	0.06	135	4.88	5529	-0.14	157	-15.25	15262	0.11	128	8.74	4321
THAP10	1.27	2.76E-06	0.07	124	4.87	5530	0.09	137	7.47	4003	0.22	127	16.73	1893
RTN1	1.06	0.165431953	0.05	141	4.87	5531	0.06	106	3.16	6406	-0.02	152	-1.92	9681
TFPI_t1	1.13	0.235484385	0.07	116	4.86	5532	0.01	110	0.41	8199	0.12	108	7.16	4964
NEUROD6	1.04	0.207987749	0.07	124	4.86	5533	0.03	110	1.67	7375	0.11	118	7.3	4916
CCND2	1.15	0.021922774	0.05	152	4.86	5534	0.19	133	16.12	1016	0	147	-0.34	8814
ZP1	1.05	6.61E-06	0.16	80	4.86	5535	0.11	90	4.54	5593	0.13	83	4.26	6312
SERPINB13_t1	1.02	0.019841283	0.06	132	4.86	5536	NA	NA	NA	NA	NA	NA	NA	NA
NCLN	1.22	3.43E-10	0.07	120	4.85	5537	0.11	135	9.06	3249	0.15	130	12.08	3167
YARS	1.11	0.004751176	0.07	121	4.85	5538	0.08	144	7.43	4021	0.11	116	7.2	4948
SYNE3	1.05	0.007996863	0.07	119	4.85	5539	0.09	109	5.6	5011	-0.01	106	-0.58	8965
RPRML	1.16	0.000270085	0.09	107	4.85	5540	0	106	0.28	8278	0.09	117	6.03	5437
DKK4	1.32	1.24E-05	0.12	92	4.85	5541	-0.03	98	-1.43	9448	0.07	115	4.47	6198
ZFP28	1.01	0.784791111	0.06	133	4.85	5542	0.19	120	13.32	1743	0.06	131	4.68	6079
HAS3_t2	1.02	0.004482295	0.1	100	4.85	5543	-0.08	111	-4.59	11432	0.14	88	5.44	5714
GNGT1_t1	1.27	1.66E-08	0.07	118	4.85	5544	0.05	93	2.25	6985	NA	NA	NA	NA
CHMP2A	1.09	0.006609003	0.09	104	4.84	5545	-0.06	120	-4.06	11128	0.18	104	9.48	4058
HIST1H2AB	1.41	1.76E-14	0.16	81	4.84	5546	0.18	110	10.86	2529	0.21	120	14.37	2494
LEMD3	1.03	0.355208743	0.06	135	4.84	5547	0.05	121	3.35	6281	0.15	131	11.86	3248
BCL2L12	1.36	1.91E-13	0.11	96	4.84	5548	0.18	100	9.03	3257	0.15	120	10.61	3646
RAD9A_t1	1.2	1.83E-08	0.09	106	4.84	5549	0.05	134	3.79	6015	NA	NA	NA	NA
ALS2	1.03	0.01020363	0.05	142	4.84	5550	0.07	143	6.77	4363	0.14	112	8.49	4431
DPP3	1.14	5.79E-05	0.09	104	4.84	5551	0.01	117	0.99	7816	0.04	125	2.69	7093
YTHDF2_t1	-1	0.992291506	0.07	120	4.83	5552	NA	NA	NA	NA	NA	NA	NA	NA
C1D	1	0.912619342	0.07	123	4.83	5553	-0.06	109	-3.31	10679	0.08	149	7.77	4722
FBXO33_t1	-1.06	0.056762101	0.05	154	4.83	5554	-0.09	124	-7.01	12655	NA	NA	NA	NA
PSMD3	1.15	0.000325145	0.08	111	4.83	5555	0.12	119	8.34	3576	0.14	104	7.77	4725
YDJC	1.13	0.000139366	0.12	92	4.83	5556	0.12	108	6.8	4347	0.01	122	0.93	8057
ICMT	1.11	0.015302481	0.07	124	4.83	5557	0.08	143	7.48	4001	0.08	142	7.61	4793
CD300LF_t1	-1.01	0.545696454	0.06	130	4.83	5558	NA	NA	NA	NA	-0.05	120	-3.62	10445
B4GALT2_t1	1.04	0.341908498	0.09	104	4.83	5559	0.06	120	4.48	5618	NA	NA	NA	NA
TDRD3	1.02	0.5010014	0.06	134	4.82	5560	0.04	109	2.12	7064	0.09	125	6.77	5121
KCTD17	1.11	0.002767918	0.07	116	4.82	5561	0.12	120	8.49	3504	0.02	125	1.37	7825
HIST1H4A	1.12	2.44E-09	0.14	84	4.82	5562	0	105	0.04	8441	0.17	131	14.02	2592
HSFY2_t1	1.03	0.009898692	0.08	113	4.82	5563	0.07	104	3.76	6031	0.17	107	9.93	3903
SAR1A	1.12	0.011299504	0.06	129	4.82	5564	0.07	128	5.55	5038	0.01	131	0.42	8360
ZNF624	1.1	0.000800784	0.06	127	4.82	5565	0.17	119	11.66	2247	0.16	124	11.94	3222
TAF1B	1.13	0.000540926	0.09	104	4.82	5566	0.17	136	14.69	1355	0.19	104	10.42	3713
CUZD1	1.14	1.14E-06	0.11	94	4.82	5567	0.15	99	7.52	3978	0.3	84	10.12	3829
FAM26E_t1	-1.03	0.034043434	0.07	118	4.82	5568	NA	NA	NA	NA	NA	NA	NA	NA
BBS9	1.06	0.070949821	0.06	128	4.81	5569	0.07	118	4.56	5581	0.06	119	4.07	6386
WBP5	1.05	0.652923683	0.07	117	4.81	5570	0.09	122	6.7	4403	0.01	123	0.57	8277
COL7A1	1.44	1.62E-06	0.08	110	4.81	5571	0.03	98	1.63	7407	0.09	103	4.54	6153
ZNF408	1.08	3.72E-06	0.14	84	4.81	5572	0.07	116	4.43	5648	0.13	100	6.32	5311
SSU72	1.19	7.08E-08	0.07	116	4.81	5573	0.06	126	4.54	5590	0.13	120	9.18	4165
COG4	1.06	0.025958541	0.06	124	4.81	5574	-0.06	122	-4.34	11283	0.02	112	1.5	7746
PPM1B_t1	1.1	0.016571665	0.05	141	4.8	5575	-0.01	134	-0.46	8791	0.22	126	16.67	1910
ATXN7L1	1.18	1.15E-11	0.07	120	4.8	5576	0.11	136	9.76	2950	0.22	123	15.7	2155
HIRIP3	1.03	0.196320364	0.07	120	4.8	5577	-0.07	102	-3.61	10885	0.08	110	4.67	6088
PSG9	1.06	3.12E-05	0.07	116	4.8	5578	0.09	113	5.75	4924	0.08	120	5.47	5703
EIF1B	1.06	0.086429381	0.06	124	4.8	5579	0	114	-0.11	8561	0.16	131	13.1	2852
RIMKLA	1.18	0.000108876	0.06	136	4.8	5580	0.02	156	2.61	6749	0	145	-0.39	8847
TRUB1	-1	0.941539874	0.06	129	4.79	5581	-0.02	125	-1.41	9439	0.11	134	9.23	4144
ERBB2IP	1.03	0.626216837	0.06	126	4.79	5582	0.11	132	9.33	3132	0.23	156	24.01	722
GRIK5	1.06	4.91E-08	0.07	120	4.79	5583	-0.02	113	-1.08	9209	0.05	99	2.45	7205
CLEC18B	1.09	0.009876103	0.09	102	4.78	5584	0.01	97	0.31	8260	0.03	128	2.43	7215
XRCC6BP1	1.28	5.95E-08	0.07	114	4.78	5585	0.07	122	4.7	5497	0.16	114	10.05	3857
RBBP6_t1	1.03	0.320420563	0.08	110	4.78	5586	0.15	129	11.49	2311	0.06	124	4.81	6025
PRKAR1B	1.09	0.001081357	0.05	149	4.78	5587	0.19	120	12.98	1837	0.05	118	3.55	6645
DLX5	1.04	0.009878819	0.08	108	4.78	5588	0.25	122	18.05	655	0.07	136	5.61	5618
PPID_t1	1.01	0.744350847	0.08	107	4.78	5589	NA	NA	NA	NA	0.16	123	11.66	3304
MX1	-1.15	0.044803154	0.07	121	4.77	5590	-0.09	129	-6.97	12635	-0.08	111	-4.92	11021
SLN	1.17	8.12E-05	0.08	107	4.77	5591	0.17	119	11.66	2250	0.15	126	11.14	3460
SLC35F2	1.02	0.547188503	0.06	126	4.77	5592	0.11	130	8.98	3284	-0.07	123	-5.28	11149
FAM116A	1.04	0.278524994	0.06	131	4.77	5593	0.05	127	3.96	5928	0.04	151	3.81	6524
MYL12A	-1.05	0.475677037	0.09	104	4.77	5594	0.07	116	4.78	5455	0.01	138	1.03	8002
YPEL1	1.17	0.010255867	0.04	179	4.77	5595	0.03	144	3.18	6397	-0.15	144	-14.49	14236
POFUT1_t1	1.08	0.001511599	0.06	133	4.77	5596	0.04	141	3.21	6369	0.05	134	4.14	6354
RIOK1_t1	1.06	0.03424392	0.08	113	4.76	5597	0.13	135	11.09	2436	0.16	123	11.93	3225
ALG10	1.09	0.000309438	0.07	123	4.76	5598	0.03	126	2.42	6877	0.08	120	5.67	5594
DPM2	1.1	0.000328815	0.1	100	4.76	5599	0.04	140	3.85	5977	0.05	126	3.66	6587
CAPN2	1.11	0.143256703	0.07	121	4.76	5600	0.1	120	7.28	4101	0.07	114	4.53	6162
LPCAT2	1.07	0.021790069	0.06	134	4.76	5601	0.11	118	7.65	3906	0.01	142	0.69	8202
DNAH7_t1	1.09	0.000157367	0.07	121	4.76	5602	0.14	107	8.03	3731	NA	NA	NA	NA
PKD2L1	-1.06	0.43096729	0.09	105	4.75	5603	0.1	128	8.01	3741	-0.07	100	-3.26	10294
MAPK1IP1L	1.05	0.121312755	0.05	154	4.75	5604	-0.01	154	-0.74	8966	-0.01	137	-0.86	9115
IL36RN	1.01	0.110523559	0.05	140	4.74	5605	0.06	122	4.52	5606	0.01	119	0.38	8387
ASXL2	1.1	0.063036339	0.06	131	4.74	5606	0.16	145	14.78	1338	0.06	152	5.88	5504
TNPO3	1.05	0.08665809	0.07	120	4.74	5607	-0.01	150	-0.96	9119	0.13	120	9.36	4097
VEGFB	-1.18	0.086413073	0.08	110	4.74	5608	0.11	110	6.3	4616	-0.04	115	-2.76	10065
NLE1	1.27	4.90E-09	0.06	126	4.74	5609	0.06	130	5.04	5310	0.01	142	1.21	7909
ATG13	1.22	6.51E-13	0.05	146	4.74	5610	0.04	144	3.77	6026	0.13	123	9.25	4136
ANKS6	1.31	0.009825748	0.07	122	4.74	5611	0.16	132	13.02	1823	0.13	131	10.36	3735
ZNF776_t1	1.01	0.156247649	0.04	165	4.74	5612	NA	NA	NA	NA	NA	NA	NA	NA
NDUFB3	1.19	3.14E-05	0.06	131	4.74	5613	0.17	113	10.54	2661	0.2	78	5.47	5706
THTPA	1.16	7.98E-06	0.07	115	4.73	5614	-0.04	120	-2.5	10156	0.1	123	7.55	4820
PCMTD2	1.19	0.000135896	0.06	133	4.73	5615	-0.02	127	-1.5	9495	0.22	125	16.61	1927
CCDC140	1.01	0.255390883	0.07	121	4.73	5616	0.02	108	1.08	7748	-0.03	131	-2.53	9967

SLC15A2	-1.01	0.380548746	0.05	136	4.72	5617	-0.06	143	-5.59	11968	0.02	142	2.15	7368
WDR75	1.01	0.774203602	0.09	105	4.72	5618	0.17	103	9.24	3169	0.02	112	1.46	7779
NUDT18	1.06	0.144105433	0.09	100	4.72	5619	-0.01	111	-0.44	8776	-0.02	133	-1.55	9473
PIWIL2	1.1	0.000423174	0.09	104	4.72	5620	-0.04	135	-3.72	10943	0.16	120	10.92	3540
ANKRD1	1.08	0.010633411	0.08	106	4.71	5621	0.04	114	2.85	6589	0.06	119	3.94	6446
RPRM	1.07	1.67E-05	0.05	144	4.71	5622	0.01	118	0.74	7977	0.02	115	1.01	8017
VDAC3	1.05	0.43076372	0.06	130	4.71	5623	0.03	115	2.22	7003	0.07	119	4.75	6053
BCR_t1	1.2	1.11E-06	0.05	147	4.71	5624	0.12	145	11.85	2172	NA	NA	NA	NA
MKI67IP	1.13	0.011612326	0.08	111	4.71	5625	0.14	104	7.37	4052	0.11	122	8.03	4606
PPP1R13L	1.21	2.08E-05	0.1	98	4.71	5626	0.07	133	5.79	4901	0.06	123	4.4	6245
MRPS17	1.46	1.26E-13	0.11	92	4.71	5627	0.25	92	10.32	2752	0.27	95	12.23	3129
FAM19A3	1.02	0.040770346	0.05	148	4.71	5628	0.01	126	0.45	8169	0	124	0.29	8444
CCL20	1.79	8.79E-06	0.08	108	4.71	5629	0.11	134	9.08	3240	0.13	137	11.03	3507
SNAPC3	1.04	0.05451583	0.06	123	4.71	5630	0.09	130	7.11	4187	0.09	119	6.38	5281
METTL16	1.02	0.137276338	0.06	132	4.71	5631	-0.11	149	-10.64	14114	-0.05	124	-3.48	10384
SRP68_t1	1.19	5.85E-07	0.08	109	4.7	5632	NA	NA	NA	NA	0.13	115	8.28	4501
EIF4H_t1	1.14	1.86E-07	0.07	116	4.7	5633	0.12	125	8.91	3325	0.18	150	18.48	1553
KBTBD6	1.03	0.292051533	0.05	140	4.7	5634	0.09	139	7.83	3822	0.08	159	9.23	4149
DDX1	1.18	0.003733904	0.08	107	4.7	5635	0.22	103	11.65	2258	0.19	137	16.49	1961
TJAP1	1.22	9.45E-10	0.08	108	4.7	5636	0.13	120	9.06	3246	0.12	114	7.84	4692
SPACA7	1.03	0.002533338	0.09	100	4.69	5637	0.03	131	2.3	6961	0.17	115	11.37	3394
VEGFA_t1	1.07	0.123704268	0.05	148	4.69	5638	-0.02	129	-1.34	9386	0.08	123	5.73	5566
NIPBL_t1	1.07	0.073877263	0.05	142	4.69	5639	0.13	119	8.72	3403	0.2	125	15.36	2229
TMEM128	1.05	0.173979018	0.08	112	4.69	5640	0.07	113	4.59	5562	-0.01	121	-0.74	9053
INPP5A	1.02	0.300156483	0.06	123	4.69	5641	0	135	0.21	8320	0.07	111	4.27	6308
ZNF350	-1.11	0.097252667	0.06	130	4.68	5642	0.12	103	6.11	4724	-0.06	124	-4.46	10825
TRDMT1_t1	-1.02	0.38394606	0.05	143	4.68	5643	0.13	151	13.11	1800	NA	NA	NA	NA
PELI3	1.24	6.06E-05	0.09	100	4.68	5644	0.07	150	6.9	4299	0.11	122	7.64	4771
PCDH10_t1	1.05	0.012877268	0.06	126	4.68	5645	0.12	121	8.26	3618	0.04	123	2.57	7145
Dec-01	1.02	0.086847588	0.06	130	4.68	5646	-0.02	115	-1.32	9375	0.05	121	3.49	6676
ANKHD1_t2	1.02	0.213783046	0.06	128	4.68	5647	-0.03	106	-1.71	9623	0.12	118	8.39	4473
CHAC2	1.06	0.019312783	0.07	115	4.68	5648	-0.14	121	-10.06	13905	0.16	110	9.82	3945
POLR2I	1.19	8.63E-09	0.11	92	4.67	5649	0.03	97	1.41	7549	0.16	79	4.59	6125
FAM160B2	-1.03	0.430982075	0.08	111	4.67	5650	0.04	160	3.92	5940	-0.11	141	-9.86	12877
SGK196	1.06	5.74E-07	0.08	107	4.66	5651	-0.04	95	-2.02	9838	0.1	112	6.06	5419
COG1	1.21	2.03E-15	0.1	95	4.66	5652	0.03	103	1.51	7493	0.01	78	0.28	8452
OPALIN_t1	1	0.715077388	0.06	129	4.66	5653	NA	NA	NA	NA	NA	NA	NA	NA
WDR11	-1.02	0.566806429	0.06	125	4.66	5654	0	133	-0.29	8666	0.08	130	6.26	5330
TMPRSS11A	-1.01	0.257239546	0.06	125	4.66	5655	0	118	-0.23	8623	0.08	128	5.93	5476
ARID1B	1.13	2.97E-05	0.06	130	4.65	5656	0.09	132	7.66	3901	0.09	147	8.26	4512
GSTTP1	1.02	0.012569781	0.08	111	4.65	5657	0	119	0.17	8354	0.02	132	1.42	7798
BPI	1.03	0.014640234	0.1	97	4.65	5658	0.01	112	0.88	7881	0.13	114	8.07	4595
KCND1	-1.01	0.47094275	0.07	116	4.64	5659	-0.02	125	-1.49	9487	0.13	115	8.64	4371
LENG9	-1.02	0.301448876	0.07	112	4.64	5660	-0.02	123	-1.78	9677	0	102	-0.19	8728
NRG2_t2	1.02	0.073128968	0.07	121	4.64	5661	0.1	130	7.72	3875	0.1	126	7.95	4639
ACOT8	1.14	4.06E-06	0.12	88	4.64	5662	-0.01	124	-0.5	8819	0.16	83	5.26	5796
ATG4B_t1	1.01	0.71563672	0.07	120	4.64	5663	NA	NA	NA	NA	NA	NA	NA	NA
ARID3A	1.24	3.61E-05	0.07	121	4.63	5664	0.18	129	14.17	1490	0.05	120	3.29	6779
GPR32	1.08	6.55E-09	0.06	130	4.63	5665	0.01	96	0.48	8154	0.09	105	4.73	6063
OTUD5	-1.01	0.779004894	0.06	133	4.63	5666	-0.05	128	-3.68	10926	0.01	122	0.89	8085
SIRPB1	1.05	0.005928068	0.06	131	4.63	5667	-0.05	140	-4.56	11413	0.06	111	3.51	6666
KCNH6	1.09	0.001101693	0.07	120	4.63	5668	0.03	124	1.92	7196	0.1	121	7.03	5021
CDK2AP2	1.16	0.000132615	0.09	104	4.63	5669	-0.01	121	-0.47	8797	0.11	147	11.11	3469
GTF3C6	1.19	0.000510071	0.09	102	4.63	5670	0.25	120	17.28	783	0.16	98	7.83	4695
KPTN	1.11	3.87E-05	0.06	124	4.62	5671	0	120	0.18	8345	-0.02	100	-0.93	9150
LSM7	1.18	3.21E-10	0.09	104	4.62	5672	0.15	88	5.82	4885	0.08	123	5.72	5573
RIC8A	1.22	9.23E-08	0.08	107	4.61	5673	0.11	122	8.14	3669	0.1	120	6.71	5148
IL27	1.08	0.04365697	0.08	108	4.61	5674	-0.02	116	-1.15	9257	0.06	102	3	6926
NKX3-2	1.13	0.000143072	0.08	111	4.61	5675	0	126	0.2	8336	0.1	146	9.53	4048
CBFB_t1	-1.11	0.028049985	0.05	143	4.61	5676	0.03	137	2.3	6959	-0.04	145	-3.76	10518
MED12	1.1	5.71E-06	0.08	110	4.6	5677	0.04	104	2.17	7023	0.07	123	5.34	5761
HIPK1_t1	-1.06	0.013753585	0.05	138	4.59	5678	NA	NA	NA	NA	NA	NA	NA	NA
VEZF1	1.08	0.040521037	0.06	130	4.59	5679	0.06	134	5.33	5151	0.06	141	5.71	5577
Sep-10	1.03	0.252512412	0.05	144	4.59	5680	0.03	124	2.56	6794	0.09	133	7.34	4900
CHMP5	1.06	0.033220003	0.06	123	4.59	5681	-0.01	131	-1.06	9187	0.14	127	10.78	3577
GHRHR	1.21	9.32E-08	0.07	120	4.59	5682	-0.06	121	-4.15	11183	0.1	98	4.75	6050
GALNT11_t1	1.08	0.005011836	0.06	121	4.59	5683	NA	NA	NA	NA	NA	NA	NA	NA
TMEM61	1.12	1.13E-08	0.11	90	4.59	5684	0.07	117	4.59	5564	0.08	106	4.42	6239
GAS2L1_t2	1.04	0.000124978	0.09	100	4.58	5685	0.09	146	8.91	3324	0.06	106	3.13	6859
LAIR1_t2	1.06	0.222115645	0.07	115	4.58	5686	0.12	128	9.41	3104	-0.03	121	-2.04	9723
PLA2G7	-1.29	0.002960384	0.08	109	4.58	5687	0.07	99	3.41	6241	-0.06	119	-4.28	10746
SLC52A3	1.11	3.95E-05	0.06	123	4.58	5688	0.21	138	18.35	607	0.1	108	5.58	5635
KCTD1_t1	1.27	1.17E-05	0.08	105	4.58	5689	NA	NA	NA	NA	0.19	119	13.32	2790
FBXL7_t1	1.01	0.166441973	0.06	129	4.58	5690	NA	NA	NA	NA	0.01	138	1.16	7941
SSX3	1.21	3.09E-05	0.07	113	4.58	5691	-0.05	117	-3.24	10628	0.09	132	7.57	4806
MYL6B	1.3	4.21E-07	0.12	88	4.58	5692	0.1	113	6.04	4753	0.15	80	4.5	6179
GPR160	1.14	0.043328236	0.08	110	4.58	5693	0.06	105	3.05	6469	0.03	123	2.45	7202
GPS2	1.02	0.348286178	0.12	89	4.57	5694	-0.11	116	-7.13	12712	0.02	84	0.68	8205
ADAMTS16	1.19	2.98E-08	0.06	122	4.57	5695	0.02	129	1.59	7434	0.15	115	9.6	4024
APLF_t1	1.08	0.017373569	0.06	124	4.57	5696	NA	NA	NA	NA	0.15	132	12.33	3100
TAOK1	1.09	0.00514238	0.05	142	4.57	5697	0.09	151	9.32	3137	0.16	160	17.16	1798
RBMXL1	-1.11	0.004628793	0.05	151	4.57	5698	0.02	151	2.29	6963	-0.03	135	-2.4	9889
BAX_t1	-1.01	0.853941303	0.08	107	4.57	5699	0	95	0.16	8362	-0.04	113	-2.66	10022
RTP4	1.17	0.046499016	0.09	99	4.56	5700	-0.06	100	-2.78	10330	-0.01	109	-0.44	8876
ARNTL2	-1.01	0.846106914	0.05	147	4.56	5701	0.01	135	1.06	7761	0.01	130	1.09	7975
CACNB1_t1	1.11	0.000251808	0.06	127	4.56	5702	0.14	136	11.77	2201	0.04	120	2.7	7084
CECR6	1.01	0.593964001	0.06	127	4.56	5703	0.03	150	2.68	6692	-0.01	154	-1.14	9269
ETV4	1.15	9.16E-07	0.07	119	4.55	5704	0.1	111	6.2	4659	0.11	125	7.94	4645
PPP2R3B	1.22	3.36E-07	0.12	89	4.55	5705	0.1	104	5.18	5231	0.14	108	8.15	4559
UNKL_t1	1.18	8.87E-06	0.06	132	4.55	5706	0.17	131	13.68	1629	0.15	126	11.19	3447
DTNB_t2	1.09	3.00E-06	0.08	106	4.54	5707	0.06	142	5.62	4999	0.08	104	4.46	6209
DAOA	1.03	0.001959518	0.07	111	4.54	5708	0.02	108	1.14	7721	0.09	117	6.15	5379
ICAM5	1.05	8.49E-06	0.07	111	4.54	5709	0.09	104	4.81	5443	0.07	105	3.7	6567
KIAA0319_t1	1.02	0.144117074	0.06	131	4.54	5710	0.09	144	8.5	3495	NA	NA	NA	NA
SPG11	1.11	0.02699716	0.08	108	4.54	5711	0.09	113	5.81	4890	0.12	133	10.17	3804
NUCB2	1.12	0.112229681	0.08	104	4.54	5712	0.02	84	0.84	7905	0.12	88	4.68	6081
NPTN	1.08	0.010135135	0.06	128	4.54	5713	0	125	0.1	8403	0.2	140	18.2	1609
CCDC113	1.16	1.03E-06	0.06	121	4.53	5714	0.15	143	14.32	1449	0.15	131	12.02	3188
STPG1	1.18	9.01E-07	0.08	104	4.53	5715	0.13	152	12.79	1900	0.09	143	8.14	4562
NUDT4	1.18	0.000282688	0.05	143	4.53	5716	0.11	164	12.19	2066	0.11	145	10.77	3586
IL25	1.05	0.000622006	0.06	122	4.53	5717	0.12	120	8.18	3650	0.03	120	1.81	7564
PPP2R1A	1	0.995359747	0.07	111	4.53	5718	-0.02	125	-1.4	9432	-0.08	101	-3.98	10627
DCLRE1A	1.01	0.842544541	0.07	116	4.53	5719	-0.08	110	-4.56	11421	0.19	107	11.08	3484
MAPK3	1.02	0.231107101	0.08	104	4.53	5720	0.03	126	2.66	6714	0.01	100	0.66	8222
BTNL8	1.32	0.007032367	0.09	100	4.53	5721	-0.06	124	-4.22	11220	-0.06	125	-4.31	10755
FAM63B_t1	-1.02	0.70129738	0.05	136	4.53	5722	0.12	141	10.84	2541	0.04	133	3.7	6566

USP11	1.09	0.005028659	0.1	97	4.53	5723	0.05	119	3.28	6338	-0.04	115	-2.9	10132
PSMC1	1.09	0.013812032	0.11	93	4.52	5724	0.02	96	1.05	7775	0.1	98	4.94	5955
SFR1	1.12	0.003229102	0.08	107	4.51	5725	0.14	111	8.75	3390	0.11	139	10.06	3851
ZNF638	-1.01	0.504082339	0.08	107	4.51	5726	0.01	94	0.43	8184	0.11	119	7.83	4696
AIMP2	1.3	2.07E-07	0.09	100	4.51	5727	0.01	83	0.22	8313	0.22	110	12.94	2911
SLC51B	1.98	6.42E-11	0.11	90	4.51	5728	0.05	116	3.15	6412	0.25	93	10.91	3543
SS18L1	1.07	0.076989482	0.05	142	4.5	5729	0.04	126	2.96	6517	0.09	132	7.64	4776
CCDC96	1.03	0.216663815	0.06	127	4.5	5730	0	124	-0.07	8532	-0.04	111	-2.74	10055
RNF43	1.66	3.95E-08	0.06	124	4.5	5731	0.15	126	11.57	2283	0.14	100	7.19	4953
KIAA0391	1.11	0.003818682	0.07	112	4.5	5732	0.04	113	2.82	6603	0.08	119	5.78	5545
TM4SF19	1.16	1.26E-05	0.08	105	4.5	5733	0.08	118	5.68	4960	0.03	108	1.55	7717
RPL39	1.17	0.00018097	0.1	93	4.49	5734	0.14	98	6.75	4382	0.18	125	13.84	2636
GRIN2A	1.06	1.63E-07	0.05	144	4.49	5735	0.06	155	5.88	4852	0.13	161	14.91	2360
PARK2_t1	1.03	0.133481567	0.06	124	4.49	5736	NA	NA	NA	NA	NA	NA	NA	NA
MRPL43_t1	1.14	0.000935084	0.05	144	4.49	5737	0.06	125	4.63	5536	0.09	100	4.47	6200
GFAP_t1	1.01	0.162086472	0.08	106	4.48	5738	0	127	-0.21	8614	NA	NA	NA	NA
GABRR1	1.06	1.84E-07	0.05	141	4.48	5739	0.16	130	13.05	1815	0.02	157	2.3	7291
PDIA2	1.3	4.16E-05	0.1	96	4.48	5740	0.19	90	7.58	3948	0.17	104	9.31	4116
TGFB2	1.08	0.007127539	0.05	135	4.48	5741	0.05	124	4.03	5888	0.07	157	7.69	4759
TLE3	1	0.955741544	0.07	116	4.48	5742	-0.08	124	-5.56	11947	0.01	142	0.87	8096
CCDC67_t1	1.02	0.016877125	0.05	134	4.48	5743	NA	NA	NA	NA	NA	NA	NA	NA
GJA5	1.09	1.57E-05	0.06	121	4.48	5744	0.15	123	10.85	2535	0.12	130	9.58	4030
SNRNP25	1.12	0.005814331	0.05	136	4.48	5745	-0.02	120	-1.74	9646	0.09	113	5.89	5498
CD200	1.06	0.06606461	0.04	159	4.47	5746	0.03	114	1.65	7391	0.05	109	3.02	6919
SPOCK3	1.05	0.006252408	0.05	141	4.47	5747	0.05	128	4.24	5755	0.11	136	9.86	3926
COX7B2	3.11	8.68E-14	0.09	100	4.47	5748	0.02	118	1.43	7539	0.23	111	13.78	2657
DYDC1	1.04	0.002251906	0.09	98	4.47	5749	0.1	90	4.03	5892	0.09	92	3.6	6611
IGDCC3	1.1	0.004379078	0.05	142	4.46	5750	-0.09	149	-8.73	13411	0.03	120	2.13	7381
SERPINB11	1.03	0.004526762	0.07	117	4.46	5751	-0.01	122	-0.43	8771	0	113	0.18	8501
PCNT_t1	1.01	0.850747424	0.05	133	4.46	5752	NA	NA	NA	NA	NA	NA	NA	NA
TAF13	1.09	9.70E-07	0.11	90	4.46	5753	0.13	95	5.66	4972	0.25	105	13.86	2626
CASP3	1.03	0.566578109	0.06	122	4.46	5754	0.11	135	9.22	3180	-0.01	116	-0.97	9184
S100P	1.92	3.36E-06	0.1	95	4.46	5755	0.13	99	6.54	4486	0.14	88	5.24	5810
DCSTAMP	1.05	0.004397142	0.09	100	4.46	5756	0.09	119	6.44	4548	0	106	0.14	8529
ITLN2	1.17	0.007099174	0.06	122	4.46	5757	-0.07	81	-2.27	10008	0.1	104	5.34	5759
COX5B	1.03	0.453466971	0.08	104	4.45	5758	-0.04	85	-1.38	9419	0.07	136	5.92	5487
ZBTB10	1	0.850763765	0.05	137	4.45	5759	0.11	135	9.18	3199	0.06	142	5.59	5626
CCER1	1.02	0.015828632	0.07	117	4.45	5760	0.01	120	0.92	7858	0.06	128	4.63	6108
CAPN9	1.07	1.40E-05	0.09	100	4.45	5761	0.04	131	3.13	6422	0.08	96	3.71	6561
RABGGTA	1.08	0.000486642	0.11	91	4.45	5762	-0.03	80	-0.78	9008	0.06	122	4.33	6275
SCG2	1.06	0.001479625	0.06	130	4.44	5763	0.16	112	10.06	2837	0.05	108	2.84	7019
SMPX	2.39	1.69E-10	0.06	129	4.44	5764	0.08	101	3.9	5949	0.2	109	11.64	3314
ZNF821	1.09	0.000494995	0.1	93	4.44	5765	0	113	0.05	8429	0.11	108	6.53	5218
SPTBN5	1.14	0.001409393	0.07	118	4.44	5766	0	109	0.27	8280	0.11	104	5.71	5575
LOXHD1	1.01	0.57844663	0.09	100	4.43	5767	-0.01	135	-1.27	9341	-0.02	129	-1.74	9580
DIRAS2_t1	1.04	0.003998025	0.05	134	4.43	5768	0.09	134	7.63	3919	NA	NA	NA	NA
CEP63_t1	-1.01	0.801708214	0.1	93	4.43	5769	NA	NA	NA	NA	0.16	114	10.54	3675
ITPKC	1.05	0.273406245	0.07	115	4.43	5770	0.1	121	7.4	4036	-0.01	120	-0.44	8874
MAP2K5	1.07	5.84E-07	0.11	89	4.43	5771	0.04	104	2	7148	0.18	100	9.09	4191
TFE3	1.07	0.002088524	0.06	121	4.42	5772	0.12	144	11.07	2442	0.03	120	2.42	7219
BIRC8	1.03	0.002246137	0.11	92	4.42	5773	0.1	88	3.78	6019	0.18	96	8.4	4465
CA12_t1	1.54	7.34E-09	0.06	127	4.42	5774	NA	NA	NA	NA	0.09	121	6.42	5266
NAV3	1.17	0.001321742	0.06	128	4.41	5775	0.03	124	2.26	6980	0.01	144	1.02	8010
CHD8_t1	1.06	0.0351099	0.06	119	4.41	5776	0.11	104	6.16	4694	NA	NA	NA	NA
DHX36	-1.01	0.898413513	0.05	136	4.41	5777	0.04	114	2.38	6899	0.04	123	2.93	6974
MBD6	1.04	0.054869828	0.05	132	4.41	5778	0.02	127	1.58	7443	0.04	130	2.82	7026
KLHDC8A	1.13	2.53E-05	0.05	132	4.4	5779	0.1	143	8.89	3331	0.02	147	2.22	7343
TSEN54	1.33	4.39E-19	0.1	93	4.4	5780	0.19	111	11.45	2319	0.09	121	6.16	5377
BRDT	1.09	0.004398363	0.08	107	4.4	5781	-0.01	107	-0.72	8946	0.29	127	22.45	906
CITED1	1.03	0.023959668	0.08	106	4.4	5782	-0.06	75	-1.57	9531	0.15	88	5.76	5555
CCDC28B	1.43	8.73E-09	0.13	84	4.39	5783	0.11	117	7.1	4195	0.15	87	5.62	5614
GPR89B	1.19	7.83E-07	0.07	112	4.39	5784	0.12	142	10.98	2482	0.18	112	11.44	3368
MLL2_t1	1.04	0.017895975	0.06	120	4.39	5785	NA	NA	NA	NA	NA	NA	NA	NA
POLR3D	-1.05	0.025632186	0.07	113	4.39	5786	-0.03	120	-2.25	9995	-0.11	127	-8.83	12536
STEAP2	1.48	3.71E-08	0.05	133	4.39	5787	0.13	132	10.63	2620	0.19	146	18.12	1620
FAM108B1	1.04	0.053078931	0.07	109	4.39	5788	0.05	103	2.76	6636	0.14	108	8.16	4556
ENTPD4	-1.02	0.436877361	0.05	135	4.38	5789	-0.04	139	-3.29	10663	-0.05	131	-3.75	10517
IMPG2	1.07	1.99E-09	0.05	142	4.38	5790	0.06	126	4.63	5535	0.02	138	1.37	7824
SLC29A3_t1	1.04	0.003726428	0.06	122	4.38	5791	-0.03	143	-3.23	10622	NA	NA	NA	NA
SLC6A20	1.02	0.010759685	0.05	133	4.38	5792	0.01	148	1.3	7601	0.05	140	4.93	5960
ZNF70	1.09	0.001089535	0.06	125	4.37	5793	0.15	132	12.44	1996	0.04	124	2.75	7052
POLR2A	1.01	0.503865328	0.07	113	4.37	5794	-0.03	125	-2.33	10044	0.02	118	1.49	7754
ODF3	1.04	0.000121424	0.07	111	4.37	5795	-0.01	99	-0.26	8643	0.12	133	10.11	3834
PTPN1	1.01	0.77180781	0.06	125	4.37	5796	0.07	140	6.5	4511	-0.03	125	-2.13	9767
NANS	1.23	2.17E-08	0.18	74	4.37	5797	0.11	95	5.01	5328	0.07	73	1.54	7722
EIF4EBP1	1.1	0.0501861	0.06	121	4.37	5798	0.03	110	1.8	7270	0.07	100	3.52	6665
ZNF709	-1.08	0.059628974	0.04	152	4.37	5799	-0.02	148	-2.19	9958	0.05	132	4.08	6381
CALR	1.08	0.086505393	0.06	118	4.37	5800	-0.03	117	-2.14	9924	0.02	113	1.55	7720
ESPL1	1.35	4.98E-07	0.08	104	4.36	5801	0.07	115	4.61	5549	0.11	93	4.78	6035
LSM5	1.15	0.001552536	0.06	129	4.36	5802	0.04	139	3.29	6325	0.16	131	12.8	2950
ZNF600_t1	1.14	0.0010555	0.14	81	4.36	5803	NA	NA	NA	NA	0.17	115	11.03	3508
AFF2	1.05	3.06E-05	0.05	139	4.36	5804	-0.01	155	-0.75	8975	0.1	148	9.37	4092
SFI1	1.21	0.002381195	0.09	100	4.36	5805	0.12	84	4.22	5768	0.01	111	0.58	8270
PARVB	1.06	0.215937045	0.07	110	4.36	5806	0.1	130	8.19	3645	0.06	118	3.95	6437
ZNF22_t1	-1.04	0.204520718	0.05	134	4.36	5807	NA	NA	NA	NA	NA	NA	NA	NA
PTPN2_t1	1.01	0.824294374	0.06	122	4.36	5808	0.1	120	6.79	4355	0.01	138	1.23	7888
SERINC2_t1	1.12	0.000663937	0.08	102	4.36	5809	NA	NA	NA	NA	0.08	100	3.86	6494
ZNF675	1.07	0.31436737	0.06	118	4.35	5810	0.16	147	15.64	1114	0.1	126	7.44	4854
FRK	1.13	0.003196453	0.05	133	4.35	5811	0.03	118	1.93	7193	0.17	135	14.18	2548
ZNF510	1.02	0.104856016	0.05	145	4.35	5812	0.09	129	6.88	4310	0.06	127	4.57	6139
CNNM1	1.08	0.000356881	0.05	133	4.35	5813	0.03	124	2.17	7032	0.09	129	7.2	4946
MITF_t1	1.02	0.225797407	0.04	149	4.35	5814	-0.03	126	-2.59	10208	0.04	134	3.4	6717
GFER	1.09	0.002625077	0.07	112	4.35	5815	0.09	123	6.92	4287	0.13	131	10.2	3798
CPM	-1.26	0.000910943	0.05	141	4.35	5816	0.03	142	2.76	6634	-0.05	137	-4.38	10790
ENOX1	1.26	1.27E-05	0.08	105	4.35	5817	0.15	108	8.98	3279	0.09	95	3.92	6453
ETNK1	-1.03	0.465537211	0.04	157	4.35	5818	0.12	129	9.74	2961	0.05	148	4.97	5946
TTLL10	1	0.72069447	0.06	125	4.35	5819	0.08	122	6.04	4755	-0.01	113	-0.62	8993
RIMS3	1.15	0.008781675	0.05	131	4.35	5820	0.12	125	8.97	3292	0.11	146	10.38	3730
FGF17	1.02	0.315882946	0.07	108	4.35	5821	-0.09	139	-8.01	13119	0.08	111	4.8	6033
MED26	-1	0.933392726	0.06	119	4.35	5822	-0.02	152	-1.7	9620	-0.1	129	-7.69	12113
CYTH2	1.01	0.751667514	0.04	160	4.34	5823	-0.05	140	-4.27	11251	-0.09	118	-6.21	11526
ZNF573	1.12	0.046210612	0.07	115	4.34	5824	0.23	105	12.77	1911	0.09	88	3.27	6791
OGFRL1	-1.02	0.697822788	0.08	102	4.34	5825	0.14	88	5.22	5208	0	94	0.15	8524
MTNR1B	1	0.916258902	0.08	104	4.34	5826	-0.12	125	-8.64	13369	-0.01	100	-0.31	8800
CTRL	1.03	0.01618917	0.06	124	4.34	5827	0.09	121	6.56	4471	-0.04	109	-2.17	9781
NXT1	1.03	0.540981077	0.04	147	4.34	5828	0.09	121	6.63	4434	-0.02	112	-1.24	9313

TCEB3	-1.08	0.082983741	0.04	150	4.33	5829	-0.04	128	-3.11	10555	-0.05	128	-3.93	10601
APBA3	1.1	0.000278433	0.08	101	4.33	5830	-0.03	112	-1.99	9819	0.09	104	4.8	6032
NME9_t1	1.02	0.024039289	0.06	119	4.33	5831	NA	NA	NA	NA	NA	NA	NA	NA
FADS3	1.22	5.56E-09	0.09	97	4.33	5832	0.08	109	4.55	5587	0.21	106	11.65	3307
CASKIN1	1.16	3.16E-14	0.08	102	4.33	5833	0.16	121	11.42	2330	0.08	115	5.32	5769
ANKZF1	1.15	5.44E-07	0.06	120	4.33	5834	0.06	120	4.34	5693	0.07	126	5.26	5798
TRIP4	1.13	0.014611286	0.07	111	4.33	5835	0.14	103	7.34	4069	0.18	106	10.34	3744
TMEM117	1.22	1.23E-07	0.05	140	4.33	5836	0.23	118	15.87	1073	0.22	148	21.86	995
CNOT1_t2	1.05	0.116166562	0.07	114	4.32	5837	-0.06	137	-5.36	11852	0.15	120	10.64	3639
TMSB10	-1.04	0.63051058	0.06	117	4.32	5838	0.2	124	14.8	1329	-0.11	120	-7.79	12155
BICD2_t1	-1.12	0.000500555	0.05	130	4.32	5839	0.03	130	2.74	6647	NA	NA	NA	NA
ARHGAP35	1.06	0.000246107	0.05	136	4.31	5840	0.03	136	2.63	6735	0.07	159	8.03	4608
TPI1	1.2	1.62E-05	0.07	110	4.31	5841	0.06	102	3.29	6330	0.14	122	9.78	3957
CAMK2D_t1	-1.13	0.000276672	0.05	130	4.31	5842	-0.06	148	-6.06	12218	-0.08	152	-8.33	12373
GFRAL	1.01	0.163411558	0.09	100	4.31	5843	0.08	120	5.31	5166	0.03	115	1.64	7651
MAML1	1.11	0.004286588	0.05	140	4.31	5844	0.21	126	16.24	994	0.04	147	3.53	6656
PPIL2_t1	1.15	2.81E-05	0.06	124	4.31	5845	0.16	122	11.4	2334	0.06	117	4.04	6397
RAB22A_t1	1.09	0.00543546	0.05	141	4.3	5846	0.09	142	8.29	3596	NA	NA	NA	NA
UGDH	1.2	0.003533772	0.05	131	4.3	5847	0.07	123	5.2	5217	0.1	139	9.07	4200
USP54	1.06	2.19E-06	0.05	138	4.3	5848	0.1	135	8.49	3503	0.06	123	4.5	6185
SCAND3	1.11	0.02324579	0.07	115	4.29	5849	0.1	99	5.02	5318	0.21	127	16.04	2065
KIAA1407	1.11	0.005200322	0.06	120	4.29	5850	0.15	129	11.64	2260	0.06	114	4.1	6372
CLASP1	1.01	0.696598092	0.05	132	4.29	5851	0.1	143	9.39	3112	0.07	143	6.72	5142
RAB21	1.04	0.108142058	0.04	146	4.29	5852	0.05	124	3.54	6156	0.05	130	3.92	6456
LGALS14	1.05	0.002703782	0.06	120	4.28	5853	0.09	122	6.3	4614	0.05	88	1.91	7502
SYNE4	1.24	0.000237736	0.09	100	4.28	5854	0.05	86	1.67	7374	0.08	92	3.26	6799
BAIAP2L2	1.86	8.10E-18	0.1	92	4.28	5855	0.13	108	7.29	4097	0.14	106	8.1	4583
SENP6	1	0.931389092	0.05	145	4.28	5856	0.08	123	5.9	4841	0.08	140	7.42	4862
KCNMB3	1.28	1.41E-10	0.17	75	4.28	5857	0.15	82	4.75	5473	0.16	84	5.49	5690
ABRA	1.05	0.104227435	0.05	137	4.28	5858	0.1	130	8.37	3557	0.18	132	14.53	2452
DZIP1L_t1	1.02	0.100274643	0.08	105	4.28	5859	NA	NA	NA	NA	-0.05	107	-3.08	10217
FBN2	1.01	0.637910685	0.06	124	4.27	5860	0.01	128	0.9	7873	-0.1	126	-7.42	12000
ELN	1.01	0.612173318	0.05	142	4.27	5861	-0.02	154	-1.83	9707	0.03	115	1.74	7604
HDGFRP2	1.08	0.002719441	0.06	122	4.27	5862	-0.01	91	-0.29	8669	0.02	108	1.39	7812
RXRG	1.06	3.64E-05	0.07	112	4.27	5863	0.1	107	5.94	4814	0.11	128	8.4	4470
HBBP1	1.01	0.279197648	0.07	114	4.27	5864	0.05	132	4.47	5625	-0.09	122	-6.66	11717
MLH3	1.15	3.12E-05	0.05	137	4.26	5865	0.01	137	1.14	7722	0.14	136	11.72	3292
BACH1	-1.05	0.390988924	0.05	136	4.26	5866	0.08	127	6.51	4505	0	135	-0.22	8746
GLB1_t1	1.09	0.024799132	0.06	120	4.26	5867	NA	NA	NA	NA	0.07	132	5.33	5764
DOK5	1.03	0.279237997	0.05	130	4.25	5868	-0.01	124	-1.07	9201	0	153	0.09	8556
GJB7	1.08	0.00219355	0.06	121	4.25	5869	0.03	157	3.73	6041	0.16	119	11.22	3437
KRT74	1.02	0.048997988	0.06	127	4.25	5870	-0.04	138	-3.42	10746	-0.01	112	-0.87	9121
THRAP3_t1	-1.02	0.225926531	0.04	154	4.25	5871	0.05	124	4.01	5899	NA	NA	NA	NA
SPTBN4_t1	1.05	3.37E-05	0.07	107	4.24	5872	0.04	116	2.51	6820	0.15	100	7.63	4779
FGF11	1.02	0.007334658	0.06	116	4.24	5873	-0.04	137	-3.33	10696	0.08	140	6.89	5072
COPE_t1	1.26	2.28E-12	0.11	88	4.24	5874	0.12	102	6.24	4643	0.1	92	4.28	6302
GABRG3	1.03	0.023769838	0.06	120	4.24	5875	0.04	126	2.82	6604	0.14	118	9.26	4128
MAGEB18	1.01	0.093228946	0.08	104	4.24	5876	0.13	120	8.95	3302	0.04	104	2.13	7379
RNF113B	1.2	4.33E-11	0.06	120	4.24	5877	0	130	0.3	8269	0.15	120	10.68	3623
SSB_t1	1.04	0.540163854	0.07	114	4.23	5878	0.3	110	18.18	627	NA	NA	NA	NA
CYP21A2	1.05	0.368182696	0.09	98	4.23	5879	-0.02	120	-1.68	9604	0.05	100	2.52	7170
EPB41L5_t1	1.03	0.023445794	0.05	143	4.22	5880	NA	NA	NA	NA	0.21	168	25.17	601
EYS	1.06	0.001397089	0.11	88	4.22	5881	0.07	91	2.86	6579	0.13	76	3.27	6797
MVP	1.16	0.01080943	0.14	80	4.21	5882	0.15	119	10.31	2754	0	89	-0.02	8634
GSTM5	-1.23	0.00040173	0.07	107	4.21	5883	-0.01	138	-1.22	9312	-0.02	120	-1.74	9578
BBS7_t1	1.04	0.14194911	0.05	138	4.21	5884	0.14	119	9.84	2918	0.15	138	13.62	2706
TAPBP_t2	1.03	0.00090643	0.08	106	4.21	5885	-0.04	114	-2.36	10054	0.04	103	1.9	7512
DEFA6	1.04	0.000106325	0.06	121	4.21	5886	-0.01	101	-0.56	8859	0.06	105	3.36	6741
NAV1	1.06	0.013315907	0.04	151	4.21	5887	0.13	155	13.61	1651	0.06	150	6.41	5271
CXorf38	-1.08	0.004346025	0.05	138	4.21	5888	0.03	131	2.45	6856	-0.04	124	-2.9	10136
PRPF18	-1	0.943228618	0.06	122	4.21	5889	-0.05	110	-3.09	10529	0.12	120	8.37	4476
CRYBB2	1.18	7.80E-08	0.08	100	4.2	5890	0.24	109	13.93	1551	0.16	113	10.3	3760
PRX_t1	1.11	0.009084565	0.05	141	4.2	5891	-0.03	150	-2.84	10366	-0.02	122	-1.23	9312
DMRT1	1	0.956933088	0.06	122	4.2	5892	-0.11	128	-8.28	13231	0.07	143	6.67	5166
BCL9L	1.06	0.000287956	0.06	120	4.2	5893	0.1	133	8.24	3627	-0.04	145	-3.91	10594
KRT72	1	0.876202421	0.07	106	4.19	5894	-0.13	114	-8.48	13321	-0.02	103	-0.86	9112
FER	1.17	1.77E-09	0.11	88	4.19	5895	0.11	102	5.6	5004	0.21	100	10.56	3670
ROR2	1.04	2.51E-06	0.07	114	4.19	5896	0.06	135	5.17	5233	0.07	117	4.58	6131
IGIP	1.11	0.08285508	0.05	129	4.19	5897	0.03	96	1.58	7440	0.19	112	11.79	3268
TNFSF15	1.1	0.002837577	0.05	136	4.19	5898	0.16	144	14.88	1311	0.01	150	1.48	7761
SPECC1_t1	1.05	0.044226195	0.07	109	4.19	5899	0.17	135	14.12	1499	0.05	127	3.88	6479
POGZ	1.21	2.48E-05	0.06	121	4.19	5900	0.15	133	12.24	2048	0.09	140	8.03	4607
CCDC102A	-1.04	0.490479559	0.07	111	4.19	5901	0.03	114	1.62	7408	-0.06	100	-3.13	10241
KDELR1	1.09	0.004751176	0.07	110	4.18	5902	0.08	112	4.86	5419	0	112	0.16	8515
CTAG2	1.38	3.34E-06	0.07	109	4.18	5903	0.06	107	3.61	6109	0.08	94	3.58	6625
EFCAB4B	1.06	1.05E-06	0.04	157	4.18	5904	0.22	120	15.11	1244	0.05	97	2.17	7361
THPO	-1.04	0.254029425	0.08	102	4.18	5905	-0.05	119	-3.28	10658	0.06	113	3.71	6559
FXYD3	1.24	0.000116623	0.06	122	4.17	5906	0.09	137	7.9	3793	0.01	114	0.93	8062
FTSJ1_t2	1.06	5.21E-05	0.06	120	4.17	5907	-0.07	104	-3.57	10852	0.06	104	3.08	6881
CDHR1	1.04	0.002779948	0.05	134	4.17	5909	-0.03	123	-2.22	9981	0.08	128	6.03	5441
INO80C	1.06	0.089832216	0.07	110	4.16	5910	-0.01	114	-0.39	8739	-0.02	110	-1.19	9285
TRIM3	1.04	0.0006199	0.08	99	4.16	5911	-0.01	134	-1.08	9204	0.11	142	10.57	3668
GTPBP10	1.1	0.053168158	0.04	144	4.16	5912	0.03	139	2.57	6784	0.21	153	21.7	1016
BTRC	1.02	0.399889408	0.05	137	4.16	5913	0.02	130	1.72	7332	0.03	163	3.75	6544
DCAF10_t1	1.08	0.011727548	0.04	155	4.16	5914	NA	NA	NA	NA	NA	NA	NA	NA
VARS	1.21	4.26E-11	0.12	86	4.15	5915	0	88	0.04	8439	0.2	75	4.92	5970
Mar-03	1.1	0.064886908	0.05	133	4.15	5916	0.11	159	12.1	2103	-0.03	128	-2.11	9762
MCAM_t1	1.21	3.34E-05	0.08	104	4.15	5917	NA	NA	NA	NA	0.11	121	7.69	4758
KBTBD3_t1	1.02	0.034298015	0.05	136	4.15	5918	NA	NA	NA	NA	0.01	139	0.64	8233
PROM2	1.01	0.61950391	0.05	129	4.15	5919	0	110	-0.26	8644	-0.05	128	-3.67	10471
RASSF7	1.14	0.008306408	0.07	109	4.15	5920	0.07	112	4.19	5780	-0.02	94	-0.9	9138
HIST1H1C	1.59	3.60E-05	0.08	100	4.15	5921	-0.05	88	-1.83	9709	0.15	70	2.93	6969
RBM10	1.06	0.009387151	0.07	111	4.15	5922	-0.04	120	-2.95	10451	-0.01	112	-0.59	8979
SCN2A	-1	0.918141512	0.04	152	4.15	5923	-0.05	133	-4.02	11110	0.06	138	5.38	5743
TRIM68	1.18	7.12E-05	0.06	116	4.15	5924	-0.05	144	-4.87	11576	0.05	126	3.99	6419
TMEM92_t1	-1.04	0.685924268	0.06	118	4.15	5925	-0.04	120	-2.94	10439	NA	NA	NA	NA
SPEN_t1	-1.01	0.742364658	0.06	124	4.14	5926	-0.01	139	-1.07	9195	NA	NA	NA	NA
CCL28	1.1	0.043465806	0.05	135	4.14	5927	0.12	121	8.29	3598	-0.01	110	-0.54	8945
THADA	1.19	3.11E-08	0.14	80	4.14	5928	0.14	96	6.35	4589	0.16	95	7.16	4968
PLCD3	1.04	0.002631241	0.07	108	4.13	5929	-0.06	120	-3.91	11048	0.04	120	2.48	7193
GFM2_t1	1.11	0.061232151	0.05	130	4.13	5930	-0.02	117	-1.22	9310	0.24	126	18.06	1632
DLX3_t1	1.04	0.000212516	0.06	121	4.13	5931	NA	NA	NA	NA	0.14	128	11.09	3483
MRPS15	1.1	0.027574339	0.13	83	4.13	5932	0.05	88	2	7138	0.14	82	4.49	6187
DDX60	-1.07	0.263648376	0.06	121	4.13	5933	-0.05	110	-2.96	10457	0.02	117	1.38	7814
LCE1B	-1.01	0.347323019	0.1	92	4.12	5934	-0.08	114	-5.24	11797	0.04	116	2.67	7099
OR2S2	1.03	0.000707887	0.14	79	4.12	5935	0	78	0.09	8404	0.14	98	6.59	5193

LAIR2	1.11	0.001704406	0.09	94	4.12	5936	0.18	90	7.05	4223	0.09	101	4.69	6076
SGCE	1.34	0.008731156	0.06	118	4.12	5937	0.04	121	2.51	6824	0.17	127	13.17	2840
SDF2	1.14	1.70E-06	0.06	119	4.12	5938	-0.05	126	-3.85	11004	0.1	123	7.52	4824
ZNF280D	1.06	0.277368127	0.05	131	4.12	5939	0.07	141	6.43	4550	0.13	122	9.63	4012
WDR36	-1.01	0.731473642	0.04	144	4.12	5940	0.06	144	5.78	4903	0.14	139	12.39	3072
ASIC5	1.05	9.47E-06	0.06	116	4.12	5941	0.1	101	4.98	5344	0.15	80	4.54	6154
TMTC2	1.02	0.693109814	0.05	129	4.11	5942	0.18	158	19.36	469	-0.03	135	-2.4	9887
EFTUD1	1.17	0.001293523	0.09	98	4.11	5943	0.07	101	3.62	6105	0.22	92	9.41	4083
ADAM29	1.12	4.89E-17	0.11	88	4.11	5944	0.11	127	8.46	3525	0.17	76	4.54	6159
FOXP2_t2	-1.41	2.70E-07	0.04	143	4.11	5945	-0.09	160	-10.23	13974	-0.1	143	-9.38	12711
KLHDC3	1.06	0.064392077	0.05	129	4.11	5946	-0.06	107	-3.51	10806	0.06	105	3.11	6868
CALM2	1.05	0.4035179	0.05	134	4.11	5947	0.15	104	7.88	3800	0.05	155	5.18	5839
TRIM23_t2	1.02	0.314962978	0.05	133	4.11	5948	-0.01	124	-0.62	8888	0.14	143	13.24	2815
ATP8A1	1.02	0.138731934	0.05	128	4.11	5949	0.04	134	3.22	6360	-0.11	135	-9.1	12624
AHCYL1	1.09	0.014734001	0.05	139	4.1	5950	0.02	129	1.54	7472	0.14	123	10.47	3702
CNOT8	-1.02	0.568963938	0.06	124	4.1	5951	0.08	129	6.63	4431	0.01	129	1.02	8004
RAD51D_t2	1.05	0.000936796	0.05	129	4.1	5952	-0.02	119	-1.46	9465	0.02	122	1.22	7904
WDFY1	-1.04	0.32230463	0.05	132	4.1	5953	0.11	133	9.22	3179	0.03	135	2.24	7322
GBP3	-1.19	0.049620037	0.04	145	4.1	5954	-0.03	119	-1.78	9674	0	124	0.14	8525
CRYGS	1.09	0.001115136	0.07	112	4.1	5955	0.06	104	3.2	6385	0.14	94	6.19	5363
APOPT1	1.03	0.021961113	0.05	133	4.1	5956	0.02	135	1.44	7532	0.1	120	7.23	4932
HIST2H4B	1.79	3.53E-20	0.29	64	4.09	5957	0.15	112	9.23	3177	0.28	136	24.27	691
RARRES1	1.21	0.034773942	0.08	100	4.09	5958	0.15	110	8.93	3316	-0.03	104	-1.59	9492
PPFIA1_t2	1.11	0.004116989	0.06	124	4.09	5959	0.1	115	6.33	4602	0.1	115	6.62	5187
SFRP2_t1	1.06	0.042844846	0.06	122	4.08	5960	NA	NA	NA	NA	-0.01	131	-1.18	9282
FGF19_t1	1.12	2.98E-05	0.05	138	4.08	5961	0.06	120	4.38	5671	NA	NA	NA	NA
PPIC	1.19	0.001280889	0.05	135	4.08	5962	0.12	95	5.32	5162	0.11	120	7.74	4735
ZNF407	1.03	0.114397805	0.06	115	4.08	5963	0.04	130	3.15	6409	0.08	118	5.25	5802
KIAA1609	-1.05	0.427635992	0.05	139	4.08	5964	0.18	127	13.62	1649	-0.14	153	-14.86	14340
HTR3E	1.02	0.085513526	0.07	111	4.08	5965	-0.09	119	-6.52	12419	0.03	104	1.62	7673
SCARA3_t2	1.18	0.00073602	0.07	106	4.08	5966	0.03	139	2.6	6758	0.02	140	2.04	7425
HIST1H2BM	1.64	2.84E-16	0.23	68	4.08	5967	0.07	117	4.89	5398	0.21	88	8.1	4581
GPX1	1.13	0.120644369	0.05	130	4.07	5968	0.03	110	1.69	7360	0.08	125	6.33	5309
UBL4A	1.07	0.000552213	0.06	115	4.06	5969	-0.01	139	-1.09	9211	0.04	118	2.71	7081
IQSEC1_t1	1.09	2.26E-07	0.05	132	4.06	5970	-0.01	128	-1.12	9236	NA	NA	NA	NA
EPS15L1	1.06	0.001962983	0.07	106	4.06	5971	0.04	134	3.58	6123	0.01	113	0.68	8213
ZNF695	1.19	3.32E-05	0.04	144	4.06	5972	0.08	130	6.29	4624	0.11	145	10.28	3773
TAF11	1.12	0.000263999	0.05	133	4.06	5973	0.02	126	1.32	7594	0.16	116	10.28	3770
BIRC3	1.02	0.895180897	0.04	152	4.06	5974	0.04	144	3.6	6111	0.03	156	3.63	6602
CCDC126	1.14	0.00013686	0.05	130	4.06	5975	0.04	116	2.67	6701	0.27	150	26.72	440
SYNGR2	1.11	0.004438897	0.06	117	4.06	5976	0.1	124	7.35	4066	-0.07	118	-4.47	10831
MAGEC1	1.66	3.77E-06	0.05	138	4.06	5977	0.05	114	3.11	6430	0.17	140	15.34	2236
TPM1_t1	1.27	0.003691117	0.06	115	4.05	5978	0.11	120	7.58	3952	0.17	134	14.08	2577
ZHX1	1.08	0.266481653	0.05	133	4.05	5979	0	128	0.14	8369	0.17	128	13.27	2807
LURAP1	-1.03	0.381359858	0.06	119	4.05	5980	-0.04	127	-3.39	10724	0.03	120	2.27	7313
HERC2_t1	1.12	0.000650043	0.04	154	4.05	5981	NA	NA	NA	NA	NA	NA	NA	NA
FEM1B	1.11	0.00260281	0.11	88	4.05	5982	0.01	79	0.38	8221	0.17	106	9.4	4086
KCNB2	-1	0.993221961	0.08	100	4.05	5983	-0.09	97	-4.2	11201	-0.07	116	-4.31	10758
CASQ2	1.04	0.003024292	0.05	139	4.04	5984	0.06	131	4.77	5461	0.07	125	5.18	5836
KBTBD8	1.05	0.004559771	0.04	157	4.04	5985	0.13	138	11.56	2284	0.09	150	8.86	4277
SRY	-1	0.899716752	0.09	96	4.04	5986	0	111	-0.24	8634	-0.13	104	-7.07	11871
MDC1	1.15	0.000977414	0.06	120	4.03	5987	0.11	109	6.21	4655	0.08	126	5.86	5518
CAMTA1_t1	1.05	0.030976102	0.05	130	4.03	5988	NA	NA	NA		0.07	155	7.45	4852
CAMK2A	1.03	0.000610197	0.05	126	4.03	5989	-0.07	142	-6.81	12566	0.07	124	5.31	5774
DACT3	1.02	0.017066905	0.06	119	4.03	5990	-0.03	146	-2.95	10444	0.02	109	1.01	8012
POC1B	1.07	0.082255246	0.06	113	4.02	5991	0.12	128	9.34	3131	0.15	130	11.81	3257
HTR1D	1.06	0.000217761	0.05	125	4.02	5992	0	139	-0.36	8722	0.1	123	7.65	4770
HOXB9	1.07	0.000712118	0.05	124	4.02	5993	-0.02	147	-2.08	9880	0.12	136	10.15	3813
AP1S1	1.06	0.100795744	0.07	111	4.02	5994	-0.03	135	-2.79	10335	0.12	131	9.75	3967
APH1A_t2	1.13	4.26E-06	0.05	129	4.01	5995	0.08	120	5.26	5184	0	134	-0.05	8652
NKX2-2	1.05	0.059038818	0.06	120	4.01	5996	-0.05	121	-3.81	10989	-0.03	125	-1.96	9698
CWF19L2	-1.1	5.64E-05	0.07	111	4.01	5997	-0.03	139	-2.71	10279	0	128	0.3	8440
ATP13A2_t1	-1.01	0.420017129	0.09	93	4.01	5998	-0.01	156	-0.62	8889	NA	NA	NA	NA
CTU2	1.04	0.083193405	0.07	107	4	5999	-0.05	107	-2.85	10378	0.02	93	0.98	8032
DDX17	1.04	0.022613009	0.05	137	4	6000	0.06	135	4.89	5393	0.06	140	4.98	5934
SNIP1	-1.01	0.435883041	0.05	124	4	6001	0.02	127	1.34	7583	0.03	154	2.86	7011
SSX1	1.88	1.47E-10	0.05	127	4	6002	0.1	122	7.01	4236	0.14	124	10.66	3632
ECT2L	1.02	0.100970529	0.05	124	4	6003	0.02	134	2.1	7079	0.07	150	7.35	4894
ZBTB2_t1	-1.04	0.206748149	0.05	134	4	6004	NA	NA	NA		-0.02	114	-1.07	9231
PTPN2_t2	-1.1	0.019232761	0.05	136	4	6005	-0.03	135	-2.39	10082	0.02	142	2.24	7326
FAM188B	1.06	0.009415188	0.06	121	3.99	6006	0.09	111	5.72	4941	0.08	96	3.72	6554
ZNF180	1.06	0.120256534	0.06	120	3.99	6007	0.01	124	0.72	7984	0.03	121	2.2	7348
CCR4	-1	0.786619321	0.05	131	3.99	6008	-0.05	116	-3.5	10799	-0.02	136	-1.45	9416
LEMD1	1.02	0.064763578	0.05	125	3.99	6009	0.07	116	4.4	5665	0.07	142	6.2	5357
CDKN2AIP	-1.14	1.42E-05	0.05	137	3.99	6010	-0.09	115	-6.02	12196	-0.06	136	-5.01	11054
ZNF83	1.02	0.77533545	0.05	124	3.98	6011	0.14	120	9.91	2894	0.09	128	6.9	5069
PVRL4	1.07	0.00259647	0.04	139	3.98	6012	-0.02	128	-1.3	9364	0.19	131	15.69	2157
TYW1	1.08	0.001727665	0.05	127	3.98	6013	0.11	136	9.23	3171	0.12	152	12.68	2985
RAB1B	1.01	0.243076207	0.05	132	3.98	6014	0.01	154	0.93	7853	0.03	125	2.12	7387
KAAG1	1.05	1.46E-07	0.07	104	3.98	6015	0.1	106	5.51	5057	0.08	126	5.82	5533
CIITA	-1.01	0.903372868	0.05	138	3.97	6016	0.13	123	9.76	2948	-0.1	120	-7.03	11857
EIF2B2	1.22	2.92E-09	0.06	120	3.97	6017	0.06	119	4.26	5745	0.08	112	4.74	6062
ARHGDIA	1.1	6.06E-06	0.07	106	3.97	6018	0.18	115	11.74	2219	0.06	103	2.93	6976
LRFN5	1.04	0.007387144	0.06	113	3.97	6019	-0.06	108	-3.26	10647	0.07	116	4.51	6171
REV1_t1	-1.02	0.427984843	0.05	135	3.97	6020	0.01	120	0.91	7864	-0.02	122	-1.69	9546
NRSN2	1.07	0.003646222	0.06	122	3.97	6021	0.09	120	6.45	4540	0	112	0.05	8585
ODF2_t2	1.01	0.731371671	0.05	132	3.97	6022	0	124	0.23	8305	-0.1	119	-6.61	11700
NOL7	1.26	2.92E-08	0.08	101	3.96	6023	0.16	84	5.38	5136	0.17	104	9.26	4130
TMEM200B	1.13	0.001247893	0.04	142	3.96	6024	0.03	118	2.3	6962	0.13	118	8.72	4333
IRAK4	1	0.948561294	0.05	127	3.96	6025	0.11	127	8.38	3549	0.06	143	5.56	5645
IKZF4	1	0.822844493	0.04	141	3.96	6026	-0.15	137	-13.19	14795	0	158	0.07	8570
FABP2	1.03	0.001485488	0.05	129	3.95	6027	0.11	148	10.73	2582	0.14	130	11.49	3351
FAM57A	1.09	0.066016514	0.04	159	3.95	6028	0.03	136	2.35	6920	-0.02	133	-1.51	9450
RAB5B	-1.01	0.805158549	0.04	143	3.95	6029	-0.09	135	-7.43	12841	-0.01	149	-1.13	9264
DTNBP1	1.07	0.14316998	0.05	131	3.95	6030	0.11	126	8.56	3472	0.05	147	5.12	5869
PLA2G4C_t1	1.13	0.00069767	0.07	109	3.94	6031	NA	NA	NA		0.04	140	3.96	6430
MRPL2	1.1	2.00E-06	0.07	105	3.94	6032	0.05	100	2.33	6937	0.09	104	4.86	5993
UQCR11	1.07	0.020238052	0.05	132	3.94	6033	0.05	119	3.21	6375	0.08	139	6.68	5160
UPF3A	1.16	3.43E-06	0.06	119	3.94	6034	0.16	117	10.55	2655	0.12	139	10.98	3517
BBS1	1.07	0.005523636	0.05	129	3.94	6035	0.06	121	4.43	5646	0.01	126	0.78	8148
VPS52	1.17	5.35E-09	0.06	116	3.94	6036	-0.03	132	-2.7	10272	0.11	113	6.63	5182
PCSK1	1.17	0.046666021	0.04	138	3.94	6037	0.14	126	10.93	2502	0.21	125	15.5	2196
DHX15	-1.01	0.926164515	0.05	135	3.94	6038	0.13	141	11.78	2197	0.07	133	6.16	5375
TMEM80_t1	1.06	0.02431287	0.06	120	3.94	6039	NA	NA	NA		0.04	136	3.2	6833
PRPF3	1.2	1.83E-06	0.07	104	3.94	6040	0.15	124	11.3	2360	0.18	117	12.09	3166
EPX	1.02	0.129871685	0.1	89	3.93	6041	-0.11	126	-8.21	13192	0.11	134	8.88	4268

RPL31	1.33	1.49E-08	0.22	68	3.93	6042	0.22	80	6.51	4508	0.24	116	15.91	2095
BNIP1	1.03	0.285628771	0.05	135	3.93	6043	0.08	120	5.37	5143	0.01	122	0.71	8191
NDUFS6	1.37	4.13E-12	0.1	88	3.93	6044	0.09	105	5.13	5258	0.22	94	9.52	4049
CNTNAP1	1.02	0.363559965	0.06	120	3.92	6045	-0.09	135	-7.63	12942	-0.02	125	-1.33	9355
CASP9	1	0.984737653	0.06	118	3.92	6046	-0.08	105	-4.38	11301	-0.02	140	-2.23	9811
MYO1C	1.03	0.557329124	0.05	130	3.92	6047	0.07	141	6.03	4761	0.06	120	4.07	6387
NCAPH2_t2	1.07	0.001668524	0.07	104	3.92	6048	-0.02	117	-1.29	9353	0.05	132	4.23	6326
NAP1L1	1.08	0.353883746	0.05	136	3.92	6049	0.19	136	16.03	1038	0.07	133	5.52	5675
PRKACG	1.04	0.000490617	0.06	115	3.92	6050	-0.1	113	-6.41	12377	0.05	100	2.61	7126
GPR61	1.01	0.197752132	0.05	124	3.92	6051	-0.03	127	-2.21	9972	0.03	120	2.22	7344
PABPC1L2B	1.05	8.01E-06	0.05	123	3.92	6052	-0.08	133	-6.86	12589	0.08	149	8.1	4584
IRGC	1.09	5.13E-11	0.16	75	3.92	6053	0.05	87	1.99	7153	0.05	80	1.58	7699
TRPA1	1.01	0.330735125	0.04	140	3.91	6054	0.07	122	4.86	5425	0.06	150	6.08	5413
OCRL	1.28	4.34E-06	0.05	134	3.91	6055	0.11	155	11.87	2163	0.28	157	29.88	249
GEMIN4	-1.02	0.54502474	0.05	124	3.91	6056	-0.12	107	-6.7	12514	-0.05	107	-3.09	10221
INSL6	1.04	0.004531555	0.08	97	3.91	6057	0.01	98	0.3	8265	0.13	78	3.7	6569
CYP19A1	1.12	0.006413372	0.05	132	3.91	6058	0.03	137	2.38	6902	0.16	131	12.64	2999
ECD	1.14	0.000114408	0.13	81	3.9	6059	0.09	93	3.74	6039	0.19	90	7.61	4792
TTTY1	1	0.592559365	0.05	124	3.9	6060	0	145	0.16	8361	0.07	111	4.48	6196
VPS26B_t1	-1	0.915361953	0.05	128	3.9	6061	NA	NA	NA	NA	-0.06	129	-4.7	10941
ARHGAP20	-1.01	0.173174622	0.04	159	3.9	6062	-0.01	142	-1.29	9354	-0.02	137	-1.66	9526
BCL2L1_t1	1.05	0.000688959	0.04	139	3.9	6063	0.11	141	9.62	3011	0.1	115	6.48	5250
LAMP3	1.11	0.171214598	0.05	123	3.9	6064	0.05	136	3.94	5931	-0.06	120	-3.91	10591
SYT3	1.16	0.000484752	0.07	109	3.9	6065	0.06	139	5.49	5062	0.01	96	0.47	8332
PCDHAC1	1.03	0.002169188	0.06	113	3.9	6066	0.04	143	3.73	6046	0.06	126	4.69	6073
RRP1_t1	1.17	3.23E-06	0.1	88	3.9	6067	0.07	123	5.45	5083	NA	NA	NA	NA
FOXN1	1.04	0.004298056	0.06	110	3.9	6068	-0.03	135	-2.13	9920	0.03	100	1.51	7742
TMEM107	1.09	0.06838417	0.05	125	3.9	6069	0.1	117	6.82	4339	-0.02	140	-1.86	9644
PLEKHF1	-1.08	0.361243482	0.08	101	3.9	6070	0.01	146	0.83	7915	0.07	115	4.47	6203
PPARG	1.22	0.003117215	0.07	109	3.89	6071	0.24	124	17.96	672	0.11	162	12.45	3050
TFF1	1.06	0.527268431	0.07	104	3.89	6072	-0.03	132	-2.07	9872	-0.05	92	-1.9	9667
RAD9B	1.07	0.000143789	0.07	108	3.89	6073	0.13	111	8.14	3676	0.12	131	10.05	3856
ASB17	1.02	0.035110209	0.07	102	3.89	6074	0.06	104	3.4	6252	0.06	93	2.6	7132
AADACL2	1.01	0.253729417	0.06	113	3.89	6075	0.12	102	6.03	4763	0.09	116	5.73	5567
PLEKHA5	1.07	0.216139446	0.06	120	3.89	6076	0.12	163	13.1	1803	0.11	117	7.19	4951
PDGFRB	1.01	0.942400898	0.06	120	3.88	6077	0.09	135	7.58	3942	0	121	0.08	8568
TRMT2A	1.07	0.001634044	0.06	120	3.88	6078	0.06	104	3.4	6251	0.01	103	0.63	8236
ZNF84	1.04	0.393261733	0.05	136	3.88	6079	0.14	131	11.56	2286	0.05	137	4.47	6199
PCDHB5	1.15	0.01963849	0.06	119	3.88	6080	0.03	111	1.68	7369	-0.02	143	-1.85	9637
PIAS2_t1	1.13	0.001119221	0.04	139	3.87	6081	0.04	140	3.79	6014	0.12	130	9.67	3994
FAM3A_t1	1.07	0.008955338	0.05	133	3.87	6082	NA	NA	NA	NA	0.07	132	5.66	5598
SECISBP2L	-1	0.958175563	0.05	128	3.87	6083	-0.01	133	-0.95	9113	0.17	154	17.6	1716
ICOSLG	1.01	0.441537482	0.05	122	3.87	6084	-0.08	143	-7.03	12666	0.05	120	3.27	6795
AP1S3	1.05	0.033284936	0.04	148	3.87	6085	0.05	138	4.4	5662	0.03	133	2.36	7259
PCDHB7	1.09	0.043878444	0.06	113	3.87	6086	0.03	143	2.95	6522	0.02	126	1.38	7819
PRKG2	-1.01	0.319823297	0.05	122	3.87	6087	-0.08	126	-6.21	12290	-0.16	120	-11.4	13374
MFGE8	1.07	0.034929467	0.08	97	3.86	6088	0.09	134	7.97	3763	0.03	106	1.64	7652
SYP	1.02	0.037553488	0.06	112	3.86	6089	0.01	133	0.92	7856	-0.04	104	-2.33	9859
DBF4B_t1	1.1	4.10E-07	0.04	147	3.86	6090	NA	NA	NA	NA	NA	NA	NA	NA
LRRTM3	1.04	6.44E-05	0.04	142	3.85	6091	0.04	122	2.93	6532	0.05	155	5.26	5794
NPM2	1.19	0.011522976	0.1	88	3.85	6092	0.05	108	2.92	6542	-0.02	93	-0.97	9185
FAM195B	1.18	7.18E-05	0.06	118	3.85	6093	0.16	118	10.68	2607	0.06	107	3.41	6713
FRAT2	1.11	0.051323418	0.04	143	3.85	6094	-0.01	139	-0.95	9111	0.09	110	5.26	5795
RPL5	1.21	0.000993044	0.12	83	3.85	6095	0.18	80	5.31	5167	0.12	79	3.41	6710
PSMD12_t1	1.15	0.001979732	0.05	120	3.85	6096	NA	NA	NA	NA	NA	NA	NA	NA
CTF1	1.05	0.005716203	0.07	107	3.85	6097	-0.16	126	-12.5	14641	0.12	118	7.87	4679
RPS25	1.14	0.003012115	0.1	88	3.84	6098	0.26	102	13.77	1606	0.05	84	1.58	7705
FAM227B	1.06	0.000625694	0.08	99	3.84	6099	0.06	103	3.08	6449	0.11	96	5.25	5801
SLC39A3_t2	1.11	0.001076522	0.06	110	3.84	6100	-0.06	110	-3.55	10837	0.08	100	3.87	6490
BPTF_t2	1.11	0.000146496	0.04	138	3.84	6101	0.09	123	6.7	4399	0.12	148	11.62	3318
MAGEB3	1.01	0.163502341	0.04	137	3.84	6102	0.09	109	5.03	5311	0.04	132	2.9	6987
EMC3_t1	1.22	5.85E-07	0.07	106	3.84	6103	NA	NA	NA	NA	0.18	150	17.69	1704
PPP2R3A_t1	1.01	0.679733063	0.04	139	3.83	6104	NA	NA	NA	NA	0.04	137	3.48	6680
GALK2	1.03	0.398690907	0.05	133	3.83	6105	0.05	131	3.79	6012	0.12	121	8.84	4281
SETD7	1.02	0.189223307	0.04	138	3.83	6106	-0.03	139	-2.42	10099	0.12	153	12.58	3013
HIST1H2BN	1.89	1.51E-20	0.27	64	3.83	6107	0.11	113	6.84	4331	0.29	80	8.66	4365
ALG13_t1	-1.02	0.77835415	0.05	130	3.83	6108	0.1	134	8.29	3603	0.06	134	5.41	5728
DEFB103B	1.09	0.001898384	0.1	88	3.82	6109	0.13	106	7.44	4015	0.12	101	6.32	5313
PCGF6	-1	0.971969127	0.06	118	3.82	6110	-0.07	116	-4.62	11449	0.14	124	10.15	3815
LPP	1.07	0.044501476	0.04	145	3.81	6111	0.08	134	6.61	4441	0.04	149	3.64	6592
FGF20	1.03	0.017969909	0.08	97	3.81	6112	0.01	108	0.68	8020	0.18	122	12.73	2964
PTH	1	0.579880525	0.06	113	3.81	6113	0.08	107	4.73	5486	0.1	157	10.48	3696
ARL14EP	1.03	0.078582452	0.04	151	3.81	6114	0.03	125	2.32	6942	0.09	111	5.6	5623
OSCAR_t1	-1.01	0.414250848	0.07	103	3.81	6115	-0.08	115	-4.89	11592	-0.06	122	-3.99	10636
SLC35B3	1.09	0.049562756	0.06	114	3.81	6116	0.03	106	1.83	7256	0.1	128	8.01	4615
FAU_t1	1.16	8.77E-07	0.12	81	3.81	6117	NA	NA	NA	NA	0.14	92	5.75	5557
SEC31A	-1.05	0.243012681	0.06	114	3.8	6118	0.06	117	4.17	5795	0.04	122	2.8	7031
LCN2	2.46	3.90E-10	0.13	80	3.8	6119	0.03	108	1.52	7482	0.1	100	5.19	5833
TIMM9	1.16	9.37E-05	0.05	127	3.8	6120	0.07	98	3.57	6134	0.07	100	3.69	6573
CCDC171	1.04	0.001000069	0.11	84	3.8	6121	0.01	78	0.4	8203	0.25	100	12.54	3029
WBP1	1.14	1.84E-05	0.04	141	3.8	6122	-0.09	107	-5.15	11746	0.05	114	3.35	6743
CXorf58	1.03	0.048217918	0.06	114	3.8	6123	0.09	110	5.24	5193	0.18	84	6.14	5382
CLTCL1	1.13	0.007970404	0.06	118	3.79	6124	-0.08	120	-5.85	12111	0.05	145	4.3	6292
SYNPR	-1	0.912770383	0.04	137	3.79	6125	0.09	112	5.87	4857	0.04	136	3.6	6612
TEP1	1.04	0.395427256	0.05	127	3.79	6126	0.12	140	10.38	2726	-0.06	123	-4.47	10829
SI	1.03	0.20129455	0.05	134	3.78	6127	-0.03	111	-1.64	9580	-0.07	129	-5.24	11134
POTED_t1	1.21	3.13E-05	0.19	70	3.78	6128	0.05	73	1.07	7756	NA	NA	NA	NA
FAM117B	1.03	0.020870579	0.04	150	3.78	6129	0.07	156	7.35	4060	0.01	160	1.43	7789
FBXL4	1.03	0.228670979	0.07	105	3.78	6130	0.05	110	3.07	6459	0.07	105	3.72	6556
MTF1_t1	1.02	0.616186635	0.04	140	3.78	6131	0.05	135	4.09	5850	NA	NA	NA	NA
KLHDC7A	1.05	0.000554215	0.05	119	3.78	6132	-0.05	128	-3.55	10839	0.06	158	5.98	5463
GABBR2	1.09	0.004253533	0.05	132	3.78	6133	-0.12	141	-11.36	14336	0.13	120	9.16	4169
CNGB1	1.03	0.094619992	0.05	128	3.77	6134	-0.01	139	-1.24	9323	0	123	0.21	8487
POMGNT1	1.04	0.239239796	0.06	109	3.77	6135	-0.07	111	-4.55	11412	0.07	99	3.22	6821
GOSR1	1.08	0.000520111	0.05	130	3.77	6136	-0.05	134	-4.32	11276	0.04	134	3.66	6588
MTOR	1.16	8.61E-05	0.05	130	3.77	6137	0.01	118	0.4	8205	0.07	130	5.28	5785
MRPL38_t1	1.1	0.000885571	0.07	106	3.76	6138	NA	NA	NA	NA	0.06	84	1.92	7498
SIRT4	1.2	4.96E-07	0.05	120	3.76	6139	0.02	112	1.08	7752	0.19	120	12.99	2886
TRNP1	1.3	0.025462617	0.06	112	3.76	6140	0.09	117	6.26	4638	0.03	121	2.36	7255
HOXA3	1.37	1.08E-06	0.05	131	3.76	6141	0.01	129	0.94	7842	0.14	161	15.9	2099
MICAL2	1.13	0.029784763	0.04	136	3.76	6142	0.08	129	6.3	4620	0.08	132	6.28	5325
PIK3CA	-1.07	0.081171856	0.07	104	3.76	6143	0.01	124	0.49	8140	0.09	127	6.55	5212
ZNF414	1.08	2.05E-07	0.08	95	3.76	6144	0.04	104	2.28	6970	0.18	92	7.68	4761
PPP3R2	1.07	1.47E-09	0.05	130	3.76	6145	0.03	141	2.87	6568	0.02	122	1.79	7572
HP1BP3	1.01	0.497535395	0.05	128	3.75	6146	0.05	123	3.53	6163	0.03	137	2.48	7192
LDHAL6A	1	0.804755386	0.05	127	3.75	6147	-0.06	118	-4.11	11156	0.04	113	2.57	7148

SUSD4_t2	1.22	5.90E-05	0.1	88	3.75	6148	-0.07	106	-4.09	11145	0.01	99	0.62	8242
DNAJC6	1.05	4.20E-05	0.04	135	3.75	6149	0	123	0.23	8307	0.13	126	10.01	3872
NDUFA5	1.06	0.362587268	0.04	137	3.75	6150	-0.05	104	-2.89	10405	0.18	139	16.31	2006
MID1IP1	1.27	2.75E-06	0.05	122	3.75	6151	0.1	126	7.89	3796	0.09	147	8.3	4495
MTIF2	1.28	6.93E-06	0.1	86	3.75	6152	0.12	90	4.61	5550	0.3	97	13.93	2609
RASGEF1A	1.12	0.001420173	0.04	146	3.74	6153	0.15	145	13.88	1567	0.05	140	4.27	6305
MCMBP	-1.12	0.012044256	0.05	126	3.74	6154	-0.08	124	-5.75	12062	0.03	124	2.2	7350
FAM168B	1.1	0.010932521	0.04	140	3.74	6155	0.1	129	7.53	3972	0.04	138	3.75	6549
MAGEA8	1.74	1.16E-11	0.04	134	3.73	6156	0.03	127	1.99	7155	0.14	111	8.4	4471
BAG6	1.18	3.41E-08	0.07	101	3.73	6157	0.06	85	1.94	7183	0.02	110	1.49	7759
MRGPRX2	1.01	0.359915666	0.05	120	3.73	6158	-0.11	121	-7.59	12924	0.01	143	1.35	7834
NAPEPLD	1.03	0.349060463	0.04	134	3.73	6159	0.04	131	3.54	6154	0.15	132	12.67	2988
TMEM231_t1	-1.02	0.638399987	0.05	128	3.73	6160	NA	NA	NA	NA	NA	NA	NA	NA
ZRANB2_t2	1.07	0.338097773	0.04	139	3.73	6161	0.23	125	17.45	755	0.04	135	3.35	6746
CCL23	-1.1	0.073944788	0.05	122	3.73	6162	-0.15	99	-7.48	12866	-0.03	109	-2.03	9718
MDH2	1.28	2.28E-08	0.06	108	3.72	6163	0.11	98	5.04	5307	0.22	122	15.62	2173
WDR70	1.15	4.71E-06	0.07	104	3.72	6164	0.06	101	2.9	6553	0.17	110	10.4	3722
GRM5	1.08	1.61E-09	0.04	137	3.72	6165	-0.05	131	-3.96	11076	0.03	143	3.2	6830
KLHL25	1.2	0.003130496	0.06	116	3.72	6166	0.01	140	1.24	7649	0.15	136	13.3	2795
ACTR10	1.07	0.082161494	0.04	145	3.72	6167	0	123	0.17	8356	0.06	134	4.63	6106
RAB12	-1.03	0.132812805	0.05	125	3.72	6168	0	124	-0.15	8581	0	133	0.39	8383
GCOM1_t1	-1	0.909002077	0.04	141	3.72	6169	NA	NA	NA	NA	0.09	130	7.14	4978
ZNF211	-1	0.94580621	0.05	120	3.72	6170	0.07	126	5.02	5326	-0.14	118	-9.65	12790
ANKRD20A2_t1	1.03	0.025860191	0.05	124	3.71	6171	NA	NA	NA	NA	0.16	118	10.68	3622
AGR2	1.48	0.000952469	0.05	122	3.71	6172	-0.02	126	-1.57	9529	0.06	104	3.3	6776
HYDIN	1.02	0.039366962	0.06	115	3.71	6173	-0.04	127	-2.81	10349	0.03	104	1.52	7734
BANF1	1.17	4.73E-10	0.09	92	3.71	6174	0.18	128	14.31	1452	0.09	112	5.44	5717
NOMO1	1.13	0.000115349	0.06	109	3.71	6175	-0.01	113	-0.45	8786	0.18	115	11.5	3349
SPAG8	1.1	0.000849088	0.06	109	3.7	6176	0.17	115	11.02	2466	0.12	107	6.64	5178
KRT79	1.03	0.029502831	0.05	121	3.7	6177	0.01	110	0.69	8004	0.06	104	3.18	6845
COMP_t1	2.12	3.27E-16	0.17	72	3.7	6178	NA	NA	NA	NA	0.28	100	14.02	2593
BCL6B	1.01	0.25161301	0.05	123	3.7	6179	0.01	130	0.82	7918	0.06	140	5.78	5544
EPS8L3	1.08	1.22E-06	0.08	97	3.7	6180	0.09	114	5.87	4860	0.02	101	0.87	8101
IZUMO1	1.05	0.004973846	0.09	90	3.7	6181	0.06	56	0.35	8240	0.17	77	4.51	6169
DHX30	1.17	6.58E-08	0.08	99	3.7	6182	0.03	72	0.73	7980	0.15	89	5.87	5509
P2RX4	1.07	0.085163765	0.06	110	3.69	6183	0.12	122	8.67	3429	-0.07	110	-3.9	10588
PLK1S1	1.28	4.60E-06	0.1	88	3.69	6184	0.12	96	5.39	5126	0.2	83	6.58	5197
TSFM	1	0.897554415	0.04	138	3.69	6185	-0.02	123	-1.24	9325	-0.03	120	-2.17	9782
SLC6A15_t2	1.03	0.00053891	0.04	141	3.68	6186	0.16	135	13.6	1655	0.04	128	3.3	6774
REXO1L1	1.02	0.040356629	0.05	128	3.68	6187	0.22	137	19.08	507	0.11	143	9.79	3952
GUCA2A	1.06	2.73E-05	0.07	102	3.67	6188	-0.04	135	-3.78	10971	0.08	95	3.43	6699
RBM47	1.03	0.11932866	0.05	127	3.67	6189	-0.11	137	-9.96	13867	0.1	131	8.23	4520
BNIPL	1.06	0.000305861	0.05	120	3.67	6190	0.05	112	3.28	6336	0.11	117	7.41	4865
TRIM55	1.15	0.282851521	0.05	120	3.67	6191	-0.06	117	-3.87	11017	0.09	115	5.65	5602
PGS1_t1	1.1	1.12E-06	0.07	100	3.67	6192	0.18	150	18.44	592	NA	NA	NA	NA
ARL10	1.02	0.097673238	0.05	123	3.67	6193	-0.05	148	-5.12	11731	0.09	126	7.03	5023
MRPS18A	1.18	3.01E-07	0.06	110	3.67	6194	0.08	129	6.45	4539	0.13	105	7.15	4974
GPANK1	1.22	9.88E-07	0.05	119	3.67	6195	-0.07	127	-5.43	11881	0.09	132	7.58	4804
TOR1AIP2_t2	1.04	0.275137641	0.04	137	3.66	6196	-0.05	141	-4.22	11216	0.12	140	10.77	3588
HIST3H2BB	1.58	3.23E-14	0.2	68	3.66	6197	0.07	92	3.09	6442	0.21	70	4.14	6355
HIST2H2AC	1.56	4.48E-16	0.24	65	3.66	6198	0.16	79	4.75	5469	0.28	80	8.34	4482
HSPA12B	1.18	5.93E-05	0.06	107	3.65	6199	0.1	123	7.09	4204	0.08	121	5.87	5511
ZFP64_t2	1.1	4.19E-06	0.05	117	3.65	6200	-0.08	152	-8.29	13238	0.11	102	5.93	5481
TPM2_t1	1.23	0.010317312	0.08	97	3.65	6201	NA	NA	NA	NA	0	92	-0.11	8680
SLC44A4	1.07	0.049205664	0.07	104	3.65	6202	-0.05	131	-4.07	11136	0.07	120	4.84	6007
ZNF620	1.05	0.000606525	0.05	120	3.65	6203	-0.02	136	-1.54	9515	0.11	134	9.14	4173
NAT14	1.09	0.00891122	0.05	117	3.64	6204	0.01	109	0.36	8231	0.03	120	1.77	7586
TMEM246	1.37	0.004379171	0.05	119	3.64	6205	0.21	120	14.82	1322	0.01	122	0.62	8247
MAPK13	1.08	0.095732178	0.04	144	3.64	6206	0.14	139	12.08	2110	-0.11	133	-8.74	12506
MYOG	-1.01	0.786653266	0.05	120	3.63	6207	-0.11	110	-6.42	12384	-0.04	112	-2.36	9873
TEFM	1.12	0.001207968	0.06	107	3.63	6208	0.11	100	5.48	5071	0.15	99	7.39	4873
CIB4	1.06	6.64E-08	0.07	103	3.63	6209	0.02	127	1.8	7275	0.15	139	13.73	2667
SH3RF2	1.09	0.000330226	0.05	128	3.63	6210	-0.13	120	-9.26	13606	0.12	131	9.43	4077
GLS	1.18	0.08454244	0.05	127	3.63	6211	0.23	136	19.72	430	0.01	120	0.82	8126
CDC42SE1_t1	1.1	0.014794495	0.05	123	3.62	6212	NA	NA	NA	NA	NA	NA	NA	NA
MAPK8IP1_t1	1.02	0.074245841	0.04	133	3.62	6213	NA	NA	NA	NA	-0.05	116	-3.05	10205
HCG4B_t1	-1.02	0.019464467	0.05	116	3.62	6214	-0.03	143	-3.2	10600	NA	NA	NA	NA
ARHGEF17	1	0.9932113	0.05	122	3.62	6215	0.03	126	2.42	6875	-0.11	120	-7.48	12024
DNASE2B	1.04	0.051948013	0.06	108	3.62	6216	0.13	103	6.81	4341	-0.05	112	-3.3	10310
OR7A5	1.04	2.25E-05	0.05	130	3.61	6217	0.13	150	12.5	1979	0.11	117	7.04	5020
RECQL5_t1	1.22	1.33E-10	0.04	136	3.61	6218	0.06	120	4.4	5659	0.01	113	0.33	8415
KRT40	-1.01	0.195181828	0.04	131	3.61	6219	-0.02	106	-1.1	9220	-0.01	117	-0.75	9056
RPRD1B	1.02	0.007767264	0.04	132	3.61	6220	0.06	139	5.27	5180	0.04	126	3.22	6819
PDZK1IP1	1.13	0.4242902	0.07	100	3.61	6221	-0.05	126	-3.6	10872	0.01	108	0.55	8291
FKBPL	1.18	1.28E-10	0.2	68	3.61	6222	0.1	59	0.93	7850	0.21	71	4.46	6211
TTLL12	1.21	7.29E-07	0.06	113	3.61	6223	-0.06	152	-6.52	12421	0.03	120	2.41	7227
EIF6	1.2	8.31E-10	0.07	99	3.61	6224	0.07	141	6.56	4475	0.02	112	1.04	7997
B4GALNT2	1.03	0.013017986	0.05	118	3.61	6225	0	104	0.01	8462	0.07	130	5.49	5692
WNT8B	1.03	0.016410217	0.05	124	3.61	6226	0.08	119	5.45	5085	0.03	116	1.65	7647
KRT33B	1.03	0.003665848	0.03	159	3.61	6227	0.04	102	1.93	7185	0.07	122	4.84	6006
SCUBE1	1.09	0.00041907	0.05	129	3.6	6228	0.01	118	0.8	7942	0.13	126	9.71	3981
IFFO1	1.13	0.008089682	0.06	108	3.6	6229	0.1	117	6.58	4466	0.06	122	4.54	6155
ATP5S_t1	-1.02	0.686697872	0.05	118	3.6	6230	-0.04	131	-3.55	10841	-0.05	123	-3.37	10342
ANKS3	1.04	0.238749147	0.09	92	3.59	6231	0.05	93	2.01	7132	-0.01	100	-0.54	8948
RNASEH2C_t1	-1.12	0.004379078	0.04	147	3.59	6232	NA	NA	NA	NA	-0.12	130	-9.98	12919
EXOSC10	1.16	0.000324046	0.07	100	3.59	6233	0.12	99	6.09	4731	0.08	105	4.63	6114
TRIM37_t2	1.24	2.02E-06	0.07	104	3.59	6234	0.07	110	4.01	5901	0.18	109	10.7	3614
SCARB1	1.26	0.005543268	0.06	115	3.59	6235	-0.02	125	-1.55	9521	0.16	132	12.79	2952
PPM1L	1.04	6.31E-05	0.05	126	3.59	6236	0.07	127	5.26	5182	0.13	124	9.9	3915
SMN2	1.02	0.329125188	0.05	126	3.59	6237	0.03	102	1.78	7291	0.09	138	8.16	4554
GCM1	1.12	7.01E-08	0.04	132	3.59	6238	-0.04	127	-2.75	10316	-0.03	126	-2.46	9927
UBE4B	1.12	0.000183192	0.04	150	3.58	6239	0.1	122	7.26	4117	0.1	148	9.34	4106
FNDC8	1.03	0.001853528	0.07	103	3.58	6240	-0.06	126	-4.21	11213	0.05	86	1.96	7474
MTMR12	1.01	0.690247732	0.04	150	3.58	6241	0.03	133	2.79	6617	0.09	153	9.08	4195
VPS26A	1.05	0.229621216	0.05	116	3.58	6242	0.1	136	8.91	3326	0.12	129	9.86	3929
PDLIM2_t1	1.03	0.001028868	0.05	117	3.58	6243	0.04	128	2.75	6637	0.08	125	5.9	5495
KCNQ4	1.04	2.38E-06	0.04	148	3.58	6244	0.02	122	1.4	7554	0.06	122	4.66	6091
PKIA	1.12	0.003578342	0.04	141	3.57	6245	0.05	129	3.66	6078	-0.03	133	-2.54	9971
ELOVL2	1.27	2.77E-05	0.04	130	3.57	6246	0.08	131	6.8	4349	0.21	142	19.28	1389
GKAP1	1.13	0.016930023	0.04	131	3.57	6247	0.01	119	0.44	8178	0.17	123	12.32	3103
TRMU	1.09	0.002428214	0.06	108	3.57	6248	0.1	95	4.37	5676	0.01	91	0.61	8255
TIAM2	1.11	0.295627285	0.04	147	3.57	6249	0.14	116	9.1	3227	-0.13	128	-10.15	12975
WRAP53	1.06	7.21E-05	0.16	72	3.56	6250	-0.01	60	-0.12	8563	0.1	56	0.63	8238
STOX2	1.09	0.147919291	0.05	123	3.56	6251	0.03	136	2.26	6982	0.09	110	5.15	5854
COL4A5_t2	1.09	6.32E-07	0.06	110	3.56	6252	NA	NA	NA	NA	0.15	115	9.8	3950
ZNF177	1.15	0.019620237	0.05	122	3.56	6253	0.13	123	9.68	2989	0.08	147	8.01	4617

ZNF33A_t2	1.07	0.010896831	0.04	138	3.56	6254	0	142	-0.14	8570	0.09	158	9.64	4010
BHLHE22	1.05	0.003538753	0.05	124	3.56	6255	-0.06	132	-5.18	11766	-0.07	133	-5.52	11247
SSNA1_t1	1.11	0.000161992	0.08	93	3.56	6256	0.12	145	11.62	2267	NA	NA	NA	NA
SPTBN4_t2	1.13	1.94E-07	0.06	112	3.56	6257	0.06	125	4.2	5777	0.13	120	9.19	4161
PAEP	1.28	8.71E-05	0.1	84	3.55	6258	0.1	124	7.43	4019	0.09	101	4.44	6225
MBP_t1	-1.01	0.615527954	0.06	113	3.55	6259	-0.12	127	-9.36	13640	-0.04	128	-3.29	10306
ZNF586_t1	-1.01	0.607887576	0.04	137	3.55	6260	0.05	130	4.14	5814	NA	NA	NA	NA
ZNF429	1.08	0.209766825	0.07	100	3.54	6261	0.2	139	18	665	0.04	126	3.01	6921
ENTHD2	1.13	0.002886777	0.05	120	3.54	6262	0.09	108	5.19	5221	-0.01	115	-0.82	9099
CASZ1_t2	1.01	0.442354597	0.06	114	3.54	6263	0.01	108	0.53	8118	-0.06	121	-4.12	10684
SNHG10	1.17	4.63E-05	0.04	131	3.54	6264	0.03	129	2.04	7115	0.16	131	12.69	2977
FAM83A_t2	1.04	0.000738838	0.07	102	3.54	6265	0.04	126	3.17	6398	0.09	107	5.01	5920
SLC38A7_t1	1.2	3.26E-09	0.07	100	3.54	6266	0.07	112	4.33	5701	NA	NA	NA	NA
RPS6KA4	1.05	0.09609732	0.07	98	3.54	6267	0.15	120	10.67	2608	-0.05	110	-2.92	10146
ZBTB17	1.08	0.000388034	0.06	114	3.54	6268	-0.01	84	-0.39	8738	0.01	103	0.62	8249
CSAG2	1.81	2.53E-09	0.08	96	3.53	6269	0.04	128	2.94	6523	0.12	109	7.26	4923
DISP2	1.33	1.22E-06	0.06	107	3.53	6270	-0.07	130	-5.35	11843	0.15	100	7.69	4757
FOXK1	1.28	5.84E-07	0.05	123	3.53	6271	0.16	147	15.32	1188	0.08	130	6.63	5181
HIST2H2AA4_t	1.71	8.08E-13	0.2	68	3.53	6272	NA	NA	NA	NA	0.16	88	6.15	5378
CLDN23	1.01	0.892489364	0.05	124	3.53	6273	-0.11	112	-7.08	12685	0.03	126	1.95	7481
SGMS1	-1.02	0.687852517	0.04	135	3.53	6274	-0.09	130	-7.54	12892	0.2	140	18.39	1570
ADPRHL1_t1	1.2	1.08E-07	0.06	107	3.52	6275	0.08	129	6.5	4516	0.08	127	6.21	5352
MYO1D	1.04	0.000683661	0.05	118	3.52	6276	0.16	147	15.15	1237	0.05	152	5.08	5888
WSCD1	1.07	0.005921707	0.04	137	3.52	6277	0.08	132	6.54	4491	0	121	-0.13	8688
NOD1	1.05	0.157055897	0.06	113	3.51	6278	0.17	126	12.7	1929	0.04	111	2.4	7233
TPM1_t2	1.1	0.016990648	0.05	125	3.51	6279	0.02	122	1.57	7451	0.16	121	11.12	3463
CACNA1D	1.1	0.000464017	0.04	135	3.51	6280	0.12	120	8.69	3417	0.14	129	11.15	3455
ZNF165	1.05	0.495162626	0.09	88	3.51	6281	-0.04	82	-1.31	9372	0.13	98	6.14	5383
U2AF2	1.03	0.088647129	0.05	120	3.51	6282	0.1	120	6.74	4383	0.03	123	1.98	7463
HTR1E_t1	1.01	0.244813676	0.04	136	3.51	6283	-0.04	100	-1.79	9689	NA	NA	NA	NA
ST13	-1.07	0.018959055	0.04	147	3.51	6284	-0.04	124	-2.85	10377	0.16	142	14.58	2433
ATF7IP	1.05	0.001442271	0.05	120	3.51	6285	0.11	117	7.06	4218	0.08	133	6.65	5173
THRA_t1	-1.07	0.25673199	0.05	125	3.5	6286	0.01	129	0.42	8193	-0.08	105	-4.44	10821
CCDC109B	-1.09	0.240169664	0.06	107	3.5	6287	0.09	96	3.93	5936	-0.13	120	-9.05	12609
NT5M	1.23	1.51E-09	0.06	106	3.5	6288	0.02	120	1.25	7644	0.12	111	7.56	4807
SSBP4_t1	-1.04	0.247234253	0.08	96	3.49	6289	0.09	109	5.42	5107	NA	NA	NA	NA
CEACAM3	-1.1	0.00296847	0.05	120	3.49	6290	-0.17	100	-8.41	13294	-0.03	100	-1.65	9523
PLD2	-1.03	0.02672578	0.06	113	3.48	6291	-0.05	124	-3.8	10979	-0.14	105	-7.76	12144
FDPS	1.93	1.00E-16	0.19	68	3.48	6292	0.2	92	8.35	3573	0.25	110	14.73	2398
RPF2	1.18	0.000229749	0.12	80	3.48	6293	0.12	64	1.64	7401	0.16	76	4.13	6360
TCF12_t1	1.05	0.086926354	0.04	134	3.48	6294	0.17	141	15.25	1212	NA	NA	NA	NA
NOP16	1.13	0.019522166	0.06	106	3.48	6295	0.13	157	14.42	1422	0.08	103	4.33	6276
BCAS3	1.16	5.77E-08	0.07	103	3.48	6296	-0.01	120	-0.92	9090	0.09	123	6.78	5119
OR10J1	1.05	0.000266533	0.06	104	3.47	6297	-0.09	111	-5.44	11891	0.09	80	2.64	7108
MAB21L2	1.07	0.081164414	0.04	136	3.47	6298	-0.03	121	-2.27	10005	0.04	120	2.48	7195
PARP14_t1	-1.06	0.014060704	0.04	143	3.46	6299	NA	NA	NA	NA	NA	NA	NA	NA
KCTD18	1.09	0.000480874	0.05	126	3.46	6300	0.03	115	2.25	6986	0.17	146	16.52	1949
NHEJ1	1.1	0.005882378	0.04	130	3.46	6301	0.06	135	4.91	5386	0.1	115	6.73	5139
ADC	1.07	1.09E-05	0.08	91	3.46	6302	0.12	148	11.9	2152	0.07	112	4.52	6165
SYNGR1_t1	1.11	0.115329326	0.05	113	3.46	6303	-0.02	104	-1.02	9159	0.05	102	2.65	7106
KRTAP12-2	1.02	0.11330825	0.09	88	3.46	6304	-0.04	158	-4.24	11232	0.21	119	14.51	2457
FAM13C	1.05	0.003315287	0.04	140	3.46	6305	0.05	120	3.47	6202	0.17	124	12.46	3044
IP6K2_t1	1.1	0.000320142	0.03	150	3.46	6306	0.11	135	8.97	3291	0.08	115	5.15	5853
PLAC1	1.08	4.14E-05	0.07	100	3.45	6307	0.14	116	9.36	3118	0.04	100	2.12	7383
PCDHA11	-1.06	0.456722705	0.04	129	3.45	6308	0.01	128	0.71	7995	-0.02	127	-1.26	9321
MYL1	1.02	0.093453193	0.06	105	3.45	6309	-0.02	126	-1.4	9430	-0.01	104	-0.4	8854
PSMD6	1.13	0.000146427	0.05	123	3.45	6310	0.14	95	6.13	4710	0.16	110	9.61	4021
RPS7_t1	1.33	1.17E-09	0.2	67	3.44	6311	NA	NA	NA	NA	NA	NA	NA	NA
RBPJ	-1.09	0.211582638	0.04	130	3.44	6312	0.14	121	9.77	2944	-0.02	131	-1.44	9411
RAB1A_t1	1.06	0.303793279	0.05	121	3.44	6313	NA	NA	NA	NA	0.1	144	9.36	4098
KCNE2	1.01	0.55004829	0.05	117	3.44	6314	0.03	116	1.95	7172	-0.02	111	-0.94	9163
ATP6V1D	1.1	0.029848194	0.04	130	3.43	6315	0.05	152	5.57	5026	0.07	132	6.06	5418
GPR39	1.06	2.41E-09	0.05	124	3.42	6316	-0.06	120	-4	11099	0.09	147	9.07	4197
FTHL17	1.18	0.00012381	0.1	84	3.42	6317	0.04	95	1.91	7208	-0.01	115	-0.51	8925
RARS	1.23	3.21E-05	0.07	97	3.42	6318	0.2	105	10.76	2571	0.15	114	9.5	4054
FKBP8	1.05	0.017226049	0.09	89	3.42	6319	-0.02	115	-1.02	9158	0.01	104	0.39	8385
NPRL3	-1.03	0.537134767	0.06	104	3.42	6320	0.06	115	4.13	5822	0.01	116	0.69	8197
MXD4_t1	1.07	0.039708513	0.05	126	3.42	6321	NA	NA	NA	NA	0.03	128	2.25	7318
LYZ	1.43	0.008542659	0.05	113	3.42	6322	0.12	131	9.92	2891	0	119	-0.05	8647
MAP3K15	1.05	0.005199316	0.04	144	3.42	6323	0.1	139	9.05	3251	0.04	120	3.08	6892
LDLRAD3	1.03	0.001984575	0.04	126	3.42	6324	0.08	142	7.46	4009	0.13	147	12.55	3027
ABCC12	-1.01	0.674017447	0.06	106	3.42	6325	-0.18	130	-14.7	15145	0.02	129	1.5	7745
STEAP1	1.45	0.000161059	0.06	107	3.42	6326	0.13	110	7.79	3836	0.17	126	13.05	2869
PCYOX1L	1.06	0.342732458	0.05	120	3.42	6327	0.14	105	7.68	3893	0	114	-0.3	8790
PRDM4	1.05	0.017530234	0.04	129	3.42	6328	0.06	126	4.36	5684	-0.02	117	-1.22	9308
DDX24_t1	-1.01	0.672265386	0.05	123	3.42	6329	NA	NA	NA	NA	-0.09	131	-7.06	11869
GADD45GIP1	1.09	0.001263962	0.07	98	3.41	6330	0	84	-0.17	8594	0.06	88	2.46	7200
ZNF530	1.05	0.004897619	0.04	126	3.41	6331	0.1	115	6.22	4651	0.05	131	4.04	6394
RUSC1	1.12	3.71E-08	0.06	106	3.41	6332	0.04	120	3.05	6470	0.1	123	7.05	5004
MED4	-1.13	0.023119836	0.03	153	3.4	6333	0.02	126	1.44	7527	-0.12	133	-10.36	13038
SHE	-1.23	0.000130294	0.04	132	3.4	6334	-0.12	143	-11.13	14278	-0.04	152	-3.71	10490
GAS7_t1	1.01	0.623537004	0.04	129	3.4	6335	0.22	143	20.5	338	NA	NA	NA	NA
TRIM7_t1	1.53	8.67E-09	0.08	92	3.4	6336	0.12	106	6.55	4478	0.2	119	13.49	2741
SPATA19	1.03	0.017025433	0.04	137	3.4	6337	-0.04	109	-2.38	10070	0.06	100	2.9	6995
NUDT14	1.02	0.72069447	0.08	90	3.4	6338	0	114	0.13	8377	-0.06	114	-3.75	10516
FOCAD	1.25	1.07E-06	0.05	115	3.4	6339	0.23	118	15.4	1166	0.12	112	7.3	4915
MAGED1	1.05	0.464001327	0.06	109	3.39	6340	-0.04	102	-2.3	10027	0.04	118	2.49	7187
ISCA2	1.09	0.01261285	0.05	120	3.39	6341	0	122	0	8485	0.02	112	1.32	7843
RPP38	1.12	5.90E-05	0.06	107	3.39	6342	0	107	-0.13	8569	0.14	104	7.5	4832
KCNRG	1.05	0.000291085	0.04	129	3.39	6343	0.13	125	9.66	2997	0.01	115	0.83	8121
MRPL37	-1.03	0.393224583	0.11	80	3.39	6344	-0.01	103	-0.45	8784	0.02	160	1.68	7636
COQ3	1.06	0.187938908	0.05	112	3.39	6345	-0.06	93	-2.52	10163	0.03	84	1.16	7937
ANGPTL5	-1	0.946374202	0.05	120	3.39	6346	0.08	113	5.22	5207	0.02	135	2.01	7445
OSMR	-1.01	0.659816915	0.05	124	3.38	6347	0.02	143	2.11	7070	-0.01	149	-1.48	9435
PTPLAD1	1.09	0.232964843	0.04	142	3.38	6348	0.01	141	0.6	8073	0.15	134	12.37	3083
MEAF6_t1	1.04	0.432452889	0.04	131	3.38	6349	NA	NA	NA	NA	0.07	133	5.48	5697
ILDR1	1.11	0.006243299	0.05	122	3.38	6350	0.16	144	15.34	1184	0.11	124	7.82	4705
NUDCD2	1.18	0.000148889	0.07	101	3.37	6351	0.09	97	4.3	5715	0.22	141	20.27	1236
SUPV3L1	1.06	0.072270973	0.06	104	3.37	6352	0.07	72	1.57	7454	0.1	123	7.56	4808
CDC20B	1	0.670590438	0.04	130	3.37	6353	0.01	126	0.61	8059	0.12	119	8.03	4605
IFIT5	-1.05	0.401069504	0.04	133	3.37	6354	-0.06	129	-4.73	11505	-0.01	127	-0.53	8938
PLEKHG4	1.11	0.113409929	0.05	120	3.37	6355	0.1	107	5.83	4880	-0.02	120	-1.13	9262
DBI	1.11	0.134812463	0.05	120	3.37	6356	0.12	111	7.28	4102	0.04	98	2.06	7420
TICAM2	1.04	0.158464322	0.04	131	3.37	6357	-0.06	138	-5.08	11709	0.08	122	5.88	5506
UBE2J2	1.08	0.002385738	0.05	117	3.37	6358	0.02	129	1.65	7387	0.1	128	7.96	4635
GTDC2	1.19	6.88E-05	0.04	129	3.37	6359	0.03	127	2.16	7039	0.17	121	12.07	3173

ACTN2	2	2.94E-08	0.03	150	3.37	6360	0.07	126	5.02	5324	0.05	133	4.48	6192
USH2A_t1	1.03	0.028710513	0.04	131	3.37	6361	-0.03	126	-2.19	9956	0.09	136	7.77	4724
SYCP2L	1.01	0.464008773	0.05	123	3.36	6362	0.01	108	0.56	8100	0.07	127	5.44	5718
GGA1_t1	1.09	0.013650688	0.06	108	3.36	6363	0.19	119	13.23	1764	0.11	140	9.53	4047
RNASE2	-1	0.912560358	0.05	118	3.36	6364	0.05	134	4.26	5747	-0.07	108	-4.3	10754
EMILIN2	-1.03	0.766470394	0.05	114	3.36	6365	0.18	141	16.36	972	-0.09	118	-6.32	11578
DMAP1_t1	1.11	0.00036984	0.15	72	3.36	6366	NA	NA	NA	NA	0.13	95	6.07	5417
PPIL2_t2	1.06	0.003585036	0.05	124	3.36	6367	0.04	122	2.66	6710	0.02	117	1.21	7908
DERL2	-1	0.930881816	0.03	149	3.35	6368	-0.07	118	-4.51	11392	-0.07	115	-4.57	10874
CDK15	1.06	9.43E-08	0.04	129	3.35	6369	0.02	126	1.2	7678	0.09	127	6.72	5141
NUBPL	1.09	0.088543204	0.04	130	3.35	6370	-0.03	131	-2.51	10158	0.11	134	9.24	4140
TEX33	1.02	0.111618572	0.1	84	3.35	6371	-0.09	89	-3.63	10895	0.05	92	1.91	7505
DNAJB1	-1.09	0.120781681	0.05	120	3.35	6372	-0.1	151	-9.96	13870	-0.02	151	-1.52	9455
STX4_t1	1.09	0.006328001	0.08	92	3.35	6373	NA	NA	NA	NA	0.09	120	6.01	5447
RNF26	1.2	5.88E-07	0.05	122	3.35	6374	0.05	121	3.87	5963	0.09	129	7.35	4895
TM9SF4_t1	1.08	0.004985295	0.05	112	3.35	6375	NA	NA	NA	NA	0.05	132	4.35	6267
PDZD7_t1	-1.01	0.220266224	0.05	123	3.35	6376	NA	NA	NA	NA	NA	NA	NA	NA
FAM89A	1.11	0.103795159	0.05	119	3.34	6377	0.12	116	7.76	3855	0.03	129	2.26	7315
DLX1	1.1	0.009637636	0.04	127	3.34	6378	0.07	128	5.25	5190	0.06	143	5.92	5485
EDDM3B	1.03	0.000124185	0.04	127	3.34	6379	-0.09	134	-7.7	12984	0.12	124	8.53	4414
CORIN	1.04	0.011493778	0.04	126	3.34	6380	-0.04	138	-3.11	10553	0.16	134	13.23	2818
SNTA1_t1	1.1	0.003706157	0.07	98	3.34	6381	NA	NA	NA	NA	NA	NA	NA	NA
SULF1	1.26	0.064160632	0.03	153	3.34	6382	0.07	134	5.71	4947	0.01	149	0.63	8239
WNT5A	1.37	2.95E-05	0.03	150	3.34	6383	0.07	134	6.2	4660	0.15	142	14.05	2583
INTS12	1.03	0.493690014	0.07	98	3.34	6384	0.01	114	0.87	7892	0.1	88	3.63	6599
RAG2	1.01	0.643997226	0.05	122	3.33	6385	-0.03	107	-1.86	9733	0.02	100	0.79	8144
LRRC73	1.21	0.000297163	0.05	120	3.33	6386	-0.02	103	-1	9147	0.07	122	5.17	5842
CLEC1A	-1.01	0.212199778	0.05	123	3.33	6387	-0.06	122	-4.07	11133	-0.01	119	-0.87	9116
GLB1L	1.19	1.88E-05	0.04	136	3.33	6388	0.12	151	12.04	2119	0.06	132	4.76	6043
FOXL2	1.06	0.036843749	0.05	118	3.33	6389	0.04	131	3.36	6278	0.03	163	3.7	6568
RNASE7	1.07	2.94E-07	0.05	120	3.33	6390	0.05	127	4.21	5772	0.11	135	9.14	4176
CTDSPL	1.01	0.83650338	0.04	135	3.33	6391	0.11	153	11.26	2377	0.02	132	1.49	7752
OGDH_t1	1.12	8.02E-09	0.06	104	3.33	6392	0.11	143	10.05	2842	0.07	152	7.44	4856
OR2H2	1.08	6.99E-09	0.07	99	3.32	6393	-0.06	76	-1.58	9546	0.13	88	5.09	5884
ZNF585A_t1	1.03	0.482568508	0.05	120	3.32	6394	NA	NA	NA	NA	NA	NA	NA	NA
WWOX_t1	-1.01	0.756007258	0.07	100	3.32	6395	-0.04	88	-1.4	9431	-0.04	76	-0.91	9145
PTCH1	1.05	3.30E-05	0.03	148	3.32	6396	0.04	147	4.02	5894	0.05	128	4.1	6369
FAM218A	1.04	0.013325238	0.04	141	3.32	6397	0.15	123	11.03	2464	0.08	124	5.86	5519
CEACAM1_t1	-1.18	0.000964269	0.04	130	3.32	6398	-0.21	128	-16.1	15441	-0.03	126	-2.41	9896
TNPO1	-1.07	0.108225719	0.04	143	3.31	6399	0.09	150	9.15	3208	0.11	150	10.95	3524
TMEM161B	1.12	8.53E-07	0.04	126	3.31	6400	-0.02	119	-1.59	9553	0.2	128	15.42	2214
DCAKD	1.11	0.001324856	0.04	128	3.31	6401	0.04	118	2.7	6684	0.04	103	1.9	7510
MCTP1	1.1	0.014310747	0.05	120	3.31	6402	0.12	124	8.78	3382	0.02	120	1.6	7687
PAGE2	2.01	4.27E-06	0.05	122	3.31	6403	-0.07	103	-3.52	10814	0.02	88	0.85	8109
DNAJB6_t2	-1.01	0.850846435	0.06	105	3.31	6404	NA	NA	NA	NA	NA	NA	NA	NA
IFNA8	1.01	0.39567275	0.06	110	3.31	6405	-0.08	143	-7.12	12705	0.14	135	11.8	3265
GPX5	1.05	0.00091436	0.05	113	3.31	6406	-0.08	133	-6.91	12607	-0.02	124	-1.14	9267
GPIHBP1	1.14	0.05217286	0.06	106	3.3	6407	-0.03	159	-3.8	10981	0.14	127	10.47	3701
SCN8A	1.07	0.008155873	0.04	139	3.3	6408	-0.11	137	-9.78	13807	0.13	146	12.08	3169
GABRA4_t1	1.01	0.154227135	0.03	151	3.3	6409	NA	NA	NA	NA	0.06	163	7.11	4990
GKN2	1.01	0.579943638	0.08	92	3.3	6410	-0.02	146	-1.7	9621	0.05	112	3.12	6863
ACTRT1	1.01	0.099076829	0.06	109	3.3	6411	0.01	91	0.39	8214	0.11	103	5.74	5565
TEAD4	1.04	0.419430677	0.07	95	3.3	6412	0.02	99	1.16	7698	-0.02	95	-1.08	9236
TNFAIP8L3_t1	-1.01	0.755642196	0.05	120	3.3	6413	NA	NA	NA	NA	NA	NA	NA	NA
DNAJC14_t1	1.04	2.60E-05	0.04	129	3.3	6414	NA	NA	NA	NA	NA	NA	NA	NA
UBR2	-1.04	0.303381047	0.05	122	3.29	6415	0.05	135	4.3	5713	0.06	143	5.95	5472
ABCC10	1.16	1.82E-12	0.04	134	3.29	6416	0.13	132	10.69	2598	0.08	125	6.25	5337
FBLN1_t1	1.05	1.84E-06	0.05	113	3.29	6417	0.02	121	1.71	7345	0.02	131	1.45	7784
TMEM108	1.05	0.009693333	0.04	128	3.29	6418	0.05	144	4.45	5634	0.13	131	10.87	3561
HNRNPA1_t2	1.03	0.540138573	0.04	125	3.29	6419	0.05	111	3.18	6396	-0.03	133	-2.71	10043
RGS14	1.01	0.806411725	0.05	117	3.29	6420	-0.02	120	-1.46	9463	0.06	112	3.86	6495
TFEC	-1.05	0.111373884	0.04	134	3.28	6421	0.12	147	12.12	2095	-0.05	148	-5.31	11161
ARSB	-1.01	0.753889368	0.04	125	3.28	6422	0.02	104	0.82	7922	-0.03	109	-1.76	9594
SRCAP	1.04	0.021531459	0.03	148	3.27	6423	0.02	123	1.73	7327	0.07	126	5.6	5620
ACAN	1.14	6.51E-06	0.05	119	3.27	6424	0.07	111	4.21	5775	0.03	123	2.29	7294
AP5Z1	1.17	3.48E-09	0.04	127	3.27	6425	0.12	151	11.83	2175	0.04	120	3.01	6924
GAL	1.17	6.12E-06	0.05	111	3.27	6426	0.07	104	3.62	6101	0.1	111	6.31	5316
EXOC4_t2	1.1	1.12E-05	0.14	73	3.26	6427	0.14	103	7.51	3982	0.19	94	8.48	4434
VWA3B	1.02	0.040798055	0.05	115	3.26	6428	-0.01	118	-0.94	9106	0.13	128	9.84	3941
TMCO2	1.04	0.001530888	0.11	80	3.26	6429	-0.13	101	-6.68	12506	0.11	100	5.38	5746
NIPBL_t2	-1.03	0.51372559	0.04	142	3.26	6430	0.08	117	5.2	5218	0.06	119	4.05	6393
DDHD1	1.07	0.000841676	0.03	155	3.25	6431	0.18	140	16.27	987	0	150	0.39	8379
C2	1.04	0.462722015	0.03	144	3.25	6432	-0.17	105	-9.39	13659	0.07	110	3.97	6424
HS6ST2_t1	1.34	0.001555198	0.04	126	3.25	6433	NA	NA	NA	NA	NA	NA	NA	NA
KCNJ6	1.11	0.000460247	0.05	116	3.25	6434	-0.03	108	-1.81	9697	0.22	110	13.12	2850
MAN2A2	1.05	0.327277155	0.04	133	3.25	6435	0.07	135	5.62	4996	0.12	124	8.57	4394
SPTA1	1.06	0.048855351	0.04	125	3.24	6436	0.11	104	6.08	4735	-0.08	123	-5.94	11418
HOXB13	1.12	0.001005522	0.05	120	3.24	6437	-0.03	130	-2.8	10337	0.03	125	2.23	7339
TRIM41	1.13	1.59E-05	0.05	121	3.24	6438	0.06	126	4.36	5682	0.03	126	2.21	7345
FOXJ2	-1.01	0.848792703	0.04	134	3.24	6439	0.01	118	0.58	8088	-0.01	135	-0.49	8904
SPANXA1	1.11	0.000882604	0.09	88	3.24	6440	0.13	91	5.25	5189	0.09	94	4.12	6363
NOP56	1.41	6.97E-12	0.23	64	3.24	6441	0.23	126	17.4	764	0.23	80	6.84	5088
FAM165B_t1	1.1	0.012166699	0.04	136	3.23	6442	NA	NA	NA	NA	0.08	118	5.32	5765
SYT16	1.01	0.12472193	0.03	147	3.23	6443	0.02	117	1.03	7787	0.07	156	7.24	4931
ACBD5	-1.08	0.119600582	0.03	149	3.23	6444	-0.11	132	-9.28	13619	0.08	150	7.62	4788
SNX24	1.06	0.090021301	0.04	124	3.23	6445	0.03	126	2.3	6951	0.18	123	12.91	2918
PTF1A	1.05	0.097525966	0.06	105	3.23	6446	-0.1	101	-5.33	11835	0	127	-0.17	8710
IL22RA1	1.26	4.50E-05	0.06	105	3.23	6447	0.04	134	3.53	6166	0.04	137	3.91	6464
ETV6	-1.05	0.0139811	0.04	129	3.22	6448	0.1	129	7.69	3888	-0.11	135	-9.05	12608
TLX1	1.07	0.000183889	0.05	120	3.22	6449	-0.02	139	-1.49	9486	0.18	124	13.02	2875
GPR161_t1	1.04	0.318637182	0.04	140	3.22	6450	NA	NA	NA	NA	NA	NA	NA	NA
ZDHHC22_t1	-1.07	4.16E-05	0.04	142	3.22	6451	-0.02	161	-1.97	9794	NA	NA	NA	NA
IFNB1	1.03	0.000914789	0.05	111	3.22	6452	-0.03	103	-1.62	9569	-0.05	104	-2.85	10109
SON_t1	1.02	0.408733392	0.05	117	3.21	6453	0.02	119	1.71	7342	0.03	127	2.48	7190
MRPS11	1.14	3.16E-05	0.04	126	3.21	6454	0.01	158	1.5	7494	0.14	131	11.24	3433
MYT1L_t1	1.02	0.017914189	0.04	125	3.21	6455	NA	NA	NA	NA	0.13	147	12.87	2934
ZNF589_t1	1.03	0.043943332	0.04	130	3.2	6456	NA	NA	NA	NA	-0.02	115	-1.54	9464
MAD2L2	1.29	1.73E-10	0.05	115	3.19	6457	0.04	106	2.22	6998	0.15	95	6.78	5118
ZC3HC1	1.2	1.06E-07	0.05	109	3.19	6458	0.04	124	2.78	6623	0.15	103	7.75	4731
FAM179B	1.03	0.163084632	0.05	116	3.19	6459	0.06	125	4.18	5790	0.09	154	9.85	3934
COX4I2	1.17	3.45E-14	0.11	80	3.19	6460	0.07	113	4.19	5781	0.16	104	8.57	4395
AKAP11	-1.17	0.014552714	0.03	151	3.19	6461	0.04	147	3.47	6200	-0.06	139	-5.31	11167
SPPL3_t1	-1.01	0.564008423	0.04	131	3.19	6462	NA	NA	NA	NA	-0.12	122	-8.29	12360
AKT1S1	1.04	0.115271949	0.05	108	3.19	6463	-0.09	127	-6.72	12521	0.08	114	5.09	5886
IL9	1	0.659072359	0.04	128	3.18	6464	-0.09	95	-4.1	11154	0.05	123	3.42	6708
GRM3	1.08	6.19E-06	0.06	104	3.18	6465	0.02	109	1.25	7645	0.02	122	1.38	7820

ADPRHL1_t2	1.38	7.84E-05	0.06	107	3.18	6466	0.07	129	5.17	5232	0.04	127	3.08	6883
SLC44A5	1.13	1.52E-09	0.04	126	3.18	6467	0.05	100	2.28	6971	0.09	104	4.85	6002
KARS	-1.07	0.063055437	0.06	104	3.18	6468	-0.09	103	-4.83	11551	-0.06	126	-4.26	10739
FAM217A	1.04	0.001285658	0.04	130	3.18	6469	0.12	121	8.74	3396	0.11	117	7.4	4868
LSS_t2	1.12	0.063574942	0.05	115	3.17	6470	0.04	114	2.61	6748	0.05	108	2.87	7003
ME3	1.14	3.13E-07	0.07	95	3.17	6471	0.12	108	6.74	4385	0.14	99	6.64	5177
WDR83OS_t1	1.01	0.842467421	0.05	114	3.16	6472	NA	NA	NA	NA	0.05	113	3.26	6798
ZNF436	1.3	5.10E-10	0.04	129	3.16	6473	0.17	133	14.2	1478	0.12	131	9.76	3961
CEACAM1_t2	-1.29	0.001700026	0.04	130	3.16	6474	-0.17	128	-13.5	14872	-0.02	126	-1.36	9371
DAZL	1.02	0.554752841	0.04	131	3.15	6475	-0.04	130	-2.82	10353	-0.01	139	-1.31	9348
GLRX3	1.18	6.23E-05	0.06	107	3.15	6476	0.14	110	8.23	3630	0.13	107	7.48	4839
TBX1_t1	1.01	0.679064581	0.05	118	3.15	6477	0.02	98	1.19	7686	-0.06	92	-2.66	10023
HIST1H1E	1.55	4.50E-07	0.08	92	3.15	6478	-0.04	88	-1.34	9387	0.13	71	2.83	7024
GOLGA1	1.06	0.047052059	0.04	134	3.15	6479	0.06	129	4.44	5643	0.09	135	7.78	4719
ENPP2	1.05	0.543265555	0.04	139	3.15	6480	0.04	119	2.77	6626	0.17	143	15.56	2188
ANKK1	1.11	0.010526259	0.06	104	3.14	6481	0.14	93	5.87	4862	0.1	100	4.89	5977
HTR3B	1.03	0.002664036	0.03	144	3.14	6482	0	103	-0.26	8647	0.08	125	5.99	5460
SULT4A1_t1	1.15	0.000597155	0.04	134	3.14	6483	NA	NA	NA	NA	NA	NA	NA	NA
WFDC6	1.02	0.038300458	0.05	109	3.14	6484	-0.04	117	-2.44	10111	0.14	104	7.62	4786
SAMM50	1.24	1.60E-06	0.05	114	3.14	6485	0.05	160	5.35	5146	0.12	80	3.59	6620
CCDC15	1.14	7.81E-11	0.04	129	3.13	6486	0.04	121	3.11	6429	0.18	128	13.82	2645
PRICKLE3	1.05	0.000569544	0.09	86	3.13	6487	-0.05	98	-2.18	9945	0.08	72	1.72	7619
TRA2B	-1	0.974842119	0.04	131	3.13	6488	0.13	152	13.32	1741	-0.05	148	-4.42	10812
SOX9	1.21	0.009241354	0.05	119	3.13	6489	0.16	129	12.84	1884	0.04	151	3.82	6516
ARHGEF35	1.05	0.255179319	0.05	113	3.13	6490	0.01	149	1.23	7660	-0.01	114	-0.37	8834
TSPY3	1.48	7.10E-06	0.09	86	3.13	6491	0.05	108	2.97	6511	0.07	100	3.49	6677
LMLN	1.03	0.000784278	0.03	140	3.13	6492	0.17	153	17.44	759	0.14	146	13.48	2744
ZNF14	-1.04	0.560227031	0.04	120	3.13	6493	0.14	119	9.6	3022	0.08	112	4.66	6098
SHCBP1L	1.03	0.000116635	0.05	112	3.12	6494	-0.04	127	-3.24	10625	0.14	111	8.58	4387
SV2A	1.02	0.02985876	0.06	103	3.12	6495	0.09	144	8.02	3736	0.09	145	8.49	4429
VRTN	1.02	0.281229112	0.04	138	3.12	6496	-0.12	98	-5.86	12116	0.07	128	5.24	5808
KRT82	1.02	0.013127444	0.05	115	3.12	6497	0.02	160	1.99	7150	0.08	119	5.56	5647
FBLIM1_t2	1.05	0.00133219	0.07	95	3.12	6498	0.13	135	10.95	2492	0.03	120	2.32	7279
POF1B_t1	1.03	0.027539396	0.03	142	3.12	6499	NA	NA	NA	NA	-0.02	124	-1.57	9478
KIR3DL2	-1.02	0.101664029	0.03	150	3.12	6500	-0.14	145	-13.6	14897	-0.1	123	-7.61	12078
CEP192	1.08	0.093353243	0.05	113	3.11	6501	0.06	126	4.27	5734	0.13	145	11.89	3237
TOR3A	1.21	0.00347352	0.05	119	3.11	6502	0.16	156	17.4	763	0.06	113	3.54	6649
ZSCAN5A	1.12	8.98E-05	0.04	121	3.11	6503	0.01	103	0.68	8015	0.12	111	7.58	4801
CACNA1C	1.04	0.178723576	0.03	140	3.11	6504	0.18	146	17.7	716	0.02	147	1.78	7580
ADCK3	1.06	0.296440551	0.06	100	3.11	6505	-0.08	112	-5.2	11777	0.04	119	2.93	6975
ANAPC2	1.05	0.011339604	0.1	82	3.11	6506	-0.09	94	-3.8	10976	0.11	123	7.71	4754
SNORD22	1.1	2.66E-05	0.07	93	3.11	6507	0.09	107	4.92	5384	0.1	94	4.44	6228
DTWD1_t1	1	0.998057667	0.04	122	3.11	6508	NA	NA	NA	NA	NA	NA	NA	NA
ALCAM	1.19	9.31E-07	0.04	136	3.1	6509	0.17	155	17.59	734	0.19	140	17.02	1822
RPS5_t1	1.27	4.31E-07	0.16	69	3.1	6510	NA	NA	NA	NA	NA	NA	NA	NA
PRKCD	1.05	0.16863102	0.03	168	3.1	6511	0.12	110	7.14	4174	-0.09	105	-5.02	11057
PTPN18	-1	0.886469537	0.03	140	3.1	6512	0.12	127	9.08	3237	-0.15	120	-10.3	13025
MOV10L1	1	0.671198894	0.1	80	3.1	6513	-0.02	111	-1.52	9505	0.07	96	3.28	6783
BEST2	1.06	5.43E-06	0.05	107	3.1	6514	-0.03	109	-1.58	9537	0.03	100	1.28	7861
GP9	1.01	0.86060112	0.11	78	3.1	6515	0.01	113	0.82	7920	-0.09	83	-3	10177
THAP11	1.01	0.688259075	0.04	124	3.09	6516	-0.09	131	-7.45	12848	-0.05	123	-3.46	10378
ZNF226_t1	1.25	0.000288214	0.11	77	3.09	6517	NA	NA	NA	NA	0.11	70	2.11	7389
LRRC37A2	1.1	2.48E-05	0.09	86	3.09	6518	0.11	81	3.51	6177	0.11	80	3.28	6784
SCG3	1.02	0.329721722	0.05	114	3.09	6519	-0.07	137	-5.87	12122	0	119	-0.04	8643
SLC35F5	1.01	0.728048798	0.04	126	3.09	6520	0.07	118	5.06	5304	0.08	122	5.57	5641
MAGEF1	1.03	0.330029098	0.04	132	3.09	6521	0.01	125	0.82	7921	-0.05	120	-3.58	10426
TCF7	1.06	0.435703252	0.04	124	3.08	6522	0.02	142	1.58	7439	0	146	-0.19	8725
SH3BGR	1.02	0.693147314	0.05	110	3.08	6523	0.04	118	2.55	6796	0.06	115	3.59	6615
WBSCR16	1.08	0.00843359	0.05	118	3.08	6524	0.03	138	2.5	6828	0.07	114	4.67	6089
DNAJC7	1.02	0.614207076	0.06	100	3.08	6525	0.15	107	8.33	3583	-0.05	117	-3.18	10259
RNF111	1.01	0.752212543	0.04	130	3.08	6526	0.03	137	2.24	6991	0.15	137	13.04	2870
ZNF613	1.1	0.040198908	0.14	72	3.07	6527	0.14	69	2.65	6717	0.1	88	3.88	6481
TMTC1_t1	1.11	0.121294289	0.03	149	3.07	6528	NA	NA	NA	NA	NA	NA	NA	NA
PNLIPRP1	1.02	0.186139127	0.17	68	3.07	6529	-0.05	111	-2.76	10318	0.09	93	3.84	6507
KLHL32	1.05	6.56E-05	0.04	128	3.07	6530	-0.12	129	-9.86	13830	0.06	132	5.13	5865
IDS_t1	1	0.975182488	0.03	147	3.06	6531	0.09	135	7.94	3779	0.03	139	3.09	6874
GPR115_t1	1.02	0.085361202	0.04	127	3.06	6532	0.05	120	3.39	6254	NA	NA	NA	NA
KRTAP8-1	1.08	2.34E-05	0.04	124	3.06	6533	-0.03	123	-1.95	9779	0.09	99	4.43	6231
LRRC40	-1.08	0.180314084	0.04	128	3.06	6534	-0.04	125	-3.07	10513	0.12	131	10.04	3861
ZNF396	1.07	7.16E-06	0.04	124	3.06	6535	0.02	138	1.79	7284	0.18	154	18.49	1552
SYCP1_t1	1.01	0.333802437	0.04	122	3.06	6536	NA	NA	NA	NA	0.04	121	2.9	6993
ITIH5_t1	1	0.871434225	0.05	113	3.06	6537	0.01	143	0.69	8008	0.03	156	2.75	7054
CDADC1	1.01	0.772221468	0.04	128	3.06	6538	0.02	124	1.83	7259	0.03	162	2.84	7020
AKAP5	1	0.928839418	0.03	148	3.06	6539	-0.05	150	-5.08	11708	0	139	0.33	8419
LLGL2_t1	1.24	1.48E-07	0.05	112	3.05	6540	0.04	84	1.47	7508	0.05	88	1.9	7513
SEC14L1	-1.16	3.15E-05	0.04	135	3.05	6541	0.06	139	5.63	4991	-0.17	125	-12.79	13787
TFB2M	1.1	0.163803782	0.04	123	3.05	6542	0.09	106	4.96	5370	0.15	139	13.39	2766
ITSN2_t1	1.02	0.027911542	0.03	139	3.05	6543	0.01	103	0.7	8001	0.03	127	1.96	7471
COL6A3_t1	1	0.911427448	0.04	120	3.05	6544	0.12	132	9.71	2971	-0.02	142	-2.11	9764
PTGER3	1.03	0.054388606	0.03	149	3.04	6545	-0.06	143	-5.39	11868	0.1	157	10.98	3516
USP28	1.04	0.021985075	0.04	122	3.04	6546	0.12	115	7.74	3864	0.01	157	1.54	7725
PLEC_t2	1.13	0.00273095	0.07	93	3.04	6547	0.05	125	3.64	6092	0.06	131	4.68	6084
DDO	1.05	0.246004529	0.03	147	3.04	6548	0.03	112	2.03	7121	0.03	125	2.62	7122
MYL3	1.1	0.002236695	0.05	106	3.03	6549	0.04	112	2.6	6757	0.06	100	3.01	6923
MANEA	1.01	0.784791111	0.04	129	3.03	6550	0.05	119	3.51	6176	0.05	132	4.31	6283
PTDSS2	1.1	0.000898027	0.07	91	3.03	6551	0.14	144	12.71	1925	0.02	120	1.7	7628
REG4	1.01	0.139094603	0.05	107	3.03	6552	0.1	125	7.35	4063	0.08	108	4.44	6220
PGBD4	1.17	1.51E-13	0.06	102	3.03	6553	0.14	105	7.58	3949	0.21	94	9.18	4163
UVSSA	1.08	0.008816538	0.04	133	3.02	6554	0.02	128	1.4	7553	0.03	129	2.44	7212
MAT2A	1.01	0.918141512	0.03	146	3.02	6555	0.01	130	0.71	7991	-0.03	130	-2.49	9949
RIPPLY2	1.12	0.000568481	0.05	116	3.02	6556	0.07	87	2.63	6736	0.03	92	1.15	7945
WDR7	-1.12	0.001425363	0.03	143	3.02	6557	-0.07	156	-7.01	12657	-0.01	145	-0.63	8999
FBXO38	1.05	0.072849156	0.04	124	3.02	6558	0.02	112	1.15	7704	0.08	116	5.32	5766
CLDN9_t1	1.06	8.57E-05	0.07	92	3.02	6559	NA	NA	NA	NA	0.05	116	3.07	6897
H2AFJ	1.21	0.000168975	0.04	135	3.01	6560	0.12	147	11.75	2215	0.11	142	9.85	3932
TRA2A	1.1	0.000611574	0.04	129	3.01	6561	0.13	124	9.48	3071	0.16	118	10.6	3655
TBC1D20_t1	1.09	0.000778642	0.03	142	3.01	6562	NA	NA	NA	NA	0.05	119	3.21	6826
RPS4X	1.04	0.431822998	0.08	87	3.01	6563	0.05	84	1.68	7364	0.05	108	2.93	6977
CCKAR	1.04	3.52E-05	0.06	98	3.01	6564	-0.07	129	-5.4	11874	0.1	100	4.99	5931
PTPN2_t3	-1.07	0.101744011	0.04	122	3	6565	0.05	120	3.7	6058	-0.04	138	-3.57	10419
MRPS9	1.14	0.000598291	0.05	106	3	6566	0.12	96	5.57	5028	0.22	96	10.11	3835
RIOK2	-1	0.969250068	0.03	148	3	6567	0.02	136	2.14	7048	0.03	135	2.96	6951
RNF113A	1.17	6.30E-07	0.08	86	3	6568	0.05	85	1.83	7258	0.12	97	5.76	5553
PAX9	1.11	1.90E-10	0.04	117	3	6569	-0.04	113	-2.74	10303	0.13	115	8.64	4368
WIPI1	1.19	0.003326742	0.03	145	3	6570	0.16	119	10.94	2498	-0.04	129	-3.22	10278
RPS13	1.16	0.000307076	0.07	91	2.99	6571	0.11	96	4.99	5341	0.02	107	1.42	7797

MRAS_t1	1.08	0.158464322	0.03	143	2.99	6572	0.09	133	7.1	4198	NA	NA	NA	NA
APOBEC3B	1.1	0.016237537	0.05	113	2.99	6573	0.01	127	1.02	7794	-0.03	95	-1.21	9304
GPR87	1.01	0.436530433	0.04	134	2.99	6574	0.02	79	0.47	8157	0.05	98	2.43	7217
B3GAT3	1.12	4.69E-06	0.06	102	2.99	6575	0.05	108	2.66	6711	0.13	120	8.99	4231
PYCR1_t1	1.14	0.000647893	0.06	100	2.99	6576	0.08	101	4.18	5789	0.05	94	2	7453
STRN3_t1	-1.05	0.334164392	0.04	123	2.99	6577	NA	NA	NA	NA	0.02	137	1.84	7541
PAPPA2	1.08	0.000524479	0.06	100	2.99	6578	0.11	110	6.73	4387	0.12	113	7.47	4844
UTRN	-1.02	0.784647319	0.03	138	2.98	6579	0.19	147	18.19	626	-0.04	147	-3.62	10444
ZC3H13_t1	1.02	0.185733936	0.04	128	2.98	6580	NA	NA	NA	NA	0.06	127	4.8	6027
CMC1	1.12	0.010338673	0.06	101	2.98	6581	0.03	114	1.66	7383	0.14	106	7.98	4630
RGMA	1.14	1.60E-05	0.05	115	2.98	6582	0.14	138	12.33	2024	0.1	123	7.2	4945
ANKDD1A	1	0.87674173	0.03	154	2.97	6583	0.09	113	5.7	4951	0	126	-0.1	8675
TUBB1	-1.01	0.726702449	0.04	128	2.97	6584	0.04	128	3.28	6337	-0.06	120	-4.47	10827
CSRP2BP	1.2	1.29E-05	0.04	122	2.97	6585	0.04	122	3.02	6485	0.14	125	10.31	3759
SLC6A6_t1	1.03	0.000282636	0.03	143	2.97	6586	NA	NA	NA	NA	0.08	151	8.53	4411
HLA-F	1.14	0.098717377	0.06	100	2.97	6587	0.05	99	2.58	6772	0	148	-0.22	8743
FUBP3_t1	1.04	0.009170725	0.03	140	2.97	6588	NA	NA	NA	NA	0.15	142	13.35	2780
NEUROD1	1.01	0.555699495	0.04	130	2.97	6589	-0.13	131	-10.17	13942	-0.04	145	-3.99	10638
ADCY6	1.13	0.000150518	0.03	150	2.97	6590	0.1	137	8.38	3551	0.07	123	4.85	6001
ZNF418	1.04	0.095501506	0.04	121	2.96	6591	0.02	114	1.11	7742	-0.03	126	-2.44	9916
PES1	1.06	7.43E-05	0.06	100	2.96	6592	0.15	104	8.07	3705	0.07	118	4.88	5987
RABEP1_t1	-1.19	9.15E-05	0.04	126	2.96	6593	NA	NA	NA	NA	NA	NA	NA	NA
PIGS	-1	0.871380932	0.04	117	2.96	6594	-0.02	153	-1.74	9643	-0.12	148	-11.4	13372
PSMD5_t1	-1.05	0.09568576	0.04	123	2.96	6595	NA	NA	NA	NA	0.11	127	8.41	4460
ZBTB11	-1.01	0.678088471	0.03	143	2.95	6596	0.06	123	4.3	5714	0.03	141	2.99	6932
CEP135	1.08	0.018836108	0.03	136	2.95	6597	0.14	139	12.55	1968	0.04	153	4.6	6122
RFX4	1.02	0.03349184	0.04	120	2.95	6598	0.04	139	3.78	6020	0.07	147	7.06	5003
DOCK9	1.11	0.01007457	0.04	121	2.95	6599	0.19	134	16.04	1034	0.12	132	9.78	3955
SAMHD1	1	0.933078031	0.04	116	2.95	6600	0.13	121	9.35	3121	0.03	131	2.34	7264
PYCR1_t2	1.49	0.003355355	0.06	100	2.94	6601	0.16	120	11.1	2430	-0.06	113	-3.73	10503
AP5M1_t1	-1.02	0.504611293	0.05	113	2.94	6602	-0.04	134	-3.67	10921	NA	NA	NA	NA
CRH	1.07	1.78E-05	0.04	117	2.94	6603	0.06	103	3.32	6299	0.16	116	10.38	3731
TAMM41	1.03	0.403044896	0.05	110	2.94	6604	-0.01	76	-0.28	8659	0.04	101	2.15	7369
SDHAP3	1.08	2.31E-05	0.04	116	2.94	6605	0	137	-0.17	8596	0.05	126	3.71	6565
GALNT13_t1	-1	0.717444654	0.04	126	2.93	6606	0.01	156	1.06	7767	NA	NA	NA	NA
SPACA4	1.02	0.012199768	0.06	102	2.93	6607	0	120	-0.03	8511	0.1	117	6.46	5257
PRKD1	1.12	0.02847139	0.04	116	2.93	6608	0.07	119	4.61	5543	0.05	134	4.29	6297
CRB3	1.01	0.82461492	0.05	108	2.93	6609	-0.07	96	-3.26	10646	-0.05	94	-2.24	9817
PAGE1	1.32	0.000490091	0.04	122	2.93	6610	-0.04	113	-2.72	10287	0.06	82	2.06	7419
IWS1	1.03	0.301335895	0.05	105	2.93	6611	0.05	137	4.4	5664	0.12	122	8.9	4261
SYN2	1.02	0.052329168	0.04	128	2.92	6612	0.01	118	0.45	8166	0.05	130	3.62	6603
TSGA10	1.02	0.618213706	0.04	123	2.92	6613	0.06	124	4.65	5518	-0.01	125	-0.65	9012
LNPEP	1.08	0.000595074	0.04	131	2.92	6614	0.05	127	4.13	5826	0.13	141	11.65	3312
GALNT4	-1.02	0.649811759	0.04	126	2.92	6615	0.04	134	3.53	6165	0.01	160	1	8023
SCN3A	1.01	0.263002941	0.03	146	2.91	6616	0.06	140	5.55	5037	0.01	159	1.53	7728
PLXND1	-1.11	0.066759075	0.03	162	2.91	6617	0.11	120	7.82	3824	-0.14	120	-9.52	12762
CLIP1	1.12	0.09690954	0.04	133	2.91	6618	0.05	118	3.22	6362	0.17	164	19.52	1343
KCTD3	1.01	0.841097575	0.03	139	2.91	6619	-0.02	128	-1.91	9753	0.11	125	8.55	4404
IL1A	1.05	2.89E-05	0.03	134	2.91	6620	0.03	138	2.56	6785	0.07	118	4.44	6227
HTR6	1.03	0.009931186	0.05	108	2.91	6621	0.01	88	0.29	8272	0.06	84	2.04	7426
HEPACAM2	1.01	0.720956235	0.04	133	2.91	6622	-0.13	121	-9.02	13510	-0.02	127	-1.72	9561
PRSS33_t1	-1.03	0.012044256	0.04	127	2.91	6623	NA	NA	NA	NA	NA	NA	NA	NA
PLEKHF2	1.07	0.271293003	0.04	130	2.91	6624	0	139	-0.06	8524	0.13	141	12.01	3191
IL1RAPL1	1.15	2.79E-05	0.08	85	2.9	6625	0.12	98	5.91	4838	0.1	88	3.69	6575
DSG4	1.02	0.08212877	0.03	134	2.9	6626	-0.07	105	-3.66	10911	0.06	128	4.38	6259
UNC13B	1.27	4.28E-08	0.03	133	2.9	6627	0	129	-0.31	8688	0.13	112	8.22	4531
RIOK1_t2	1.09	0.033082448	0.05	113	2.9	6628	0.13	135	10.82	2545	0.17	123	12.17	3146
SIM1	1.1	4.36E-11	0.03	138	2.9	6629	0.05	120	3.23	6353	0.05	148	4.72	6066
YPEL4_t1	1.02	0.172484307	0.04	128	2.9	6630	NA	NA	NA	NA	-0.03	128	-2.36	9876
CCL1	-1.02	0.131368723	0.04	119	2.89	6631	0	120	0.13	8383	-0.05	147	-5.17	11103
ABCD1	1.2	1.32E-10	0.05	108	2.89	6632	0.11	120	7.55	3964	0.07	110	4.49	6188
ATP1B3	-1.11	0.303383314	0.04	118	2.89	6633	0.25	111	15.4	1170	-0.11	116	-7.22	11925
MRPL4	1.14	2.64E-05	0.05	111	2.89	6634	-0.04	104	-1.95	9781	0.07	123	4.94	5957
TM4SF4	-1.03	0.839421527	0.03	144	2.89	6635	-0.11	114	-6.87	12590	0.06	104	3.27	6794
NACA2	1.21	0.000290209	0.06	95	2.88	6636	0.21	85	7.2	4139	0.14	140	12.89	2928
GPR137	1.13	1.88E-07	0.07	92	2.88	6637	-0.03	100	-1.49	9483	0.14	91	5.73	5570
ZNF44	-1.14	0.009657525	0.03	145	2.88	6638	-0.01	132	-0.56	8858	0.01	148	0.62	8248
SKP2	1.1	0.000452014	0.06	99	2.88	6639	0.03	97	1.48	7502	0.14	68	2.44	7208
XKR8	1.15	0.000592481	0.04	127	2.88	6640	0.02	108	1.3	7606	0.13	135	10.91	3542
GALR1	1.02	0.146856453	0.03	144	2.88	6641	-0.01	110	-0.43	8772	0.07	144	6.26	5331
TMOD3	1.05	0.192960758	0.03	151	2.88	6642	0.09	122	6.55	4477	0.12	162	12.95	2903
OGG1_t3	1.11	0.000533048	0.06	100	2.88	6643	0.03	112	1.56	7457	0.04	121	3.03	6912
CAPN1	-1.01	0.836556791	0.06	101	2.87	6644	-0.04	118	-2.52	10165	-0.05	104	-2.59	9994
FAM124A	-1.01	0.741757052	0.03	137	2.87	6645	-0.02	130	-1.39	9423	-0.08	126	-6.27	11559
ADAM22_t1	1.07	0.000182571	0.11	77	2.87	6646	-0.08	124	-6.04	12209	0.05	100	2.37	7247
GABRA1	1.04	1.86E-05	0.04	130	2.87	6647	-0.05	139	-4.16	11186	0.04	146	3.93	6451
ZNF428	1.03	0.268025015	0.04	116	2.87	6648	0.03	110	1.65	7392	0.01	108	0.52	8304
LNX2	-1.08	0.019239131	0.03	153	2.87	6649	-0.08	132	-6.16	12265	-0.03	135	-2.55	9975
CLDN6	1.06	9.82E-07	0.04	115	2.87	6650	-0.03	134	-2.93	10429	0.03	116	2.21	7346
MBD2_t2	-1.12	0.006310089	0.04	127	2.87	6651	0.08	127	6.53	4498	-0.11	149	-10.44	13070
SKIL	-1.1	0.008581125	0.03	142	2.87	6652	0.06	120	4.36	5686	0.02	147	1.83	7549
PIAS2_t2	-1.04	0.264309678	0.03	158	2.87	6653	0	147	0.2	8332	0.06	120	4.3	6287
ATP5H	1.21	0.000134798	0.08	88	2.87	6654	0.06	120	4.53	5598	0.16	117	10.49	3693
GPR37	1.16	9.21E-05	0.04	130	2.87	6655	0.05	122	3.45	6214	0.11	127	8.48	4435
HIST1H2BO	1.57	2.03E-13	0.2	64	2.87	6656	0.07	101	3.5	6183	0.2	80	5.99	5457
RNF41	1.16	1.42E-07	0.03	134	2.87	6657	0.09	135	7.26	4114	0.11	143	10.08	3842
ATG4D	1.02	0.433438394	0.04	126	2.87	6658	-0.01	110	-0.42	8762	0.01	145	0.9	8077
RETNLB	1.09	1.06E-10	0.05	108	2.86	6659	-0.01	122	-1.05	9177	0.1	116	6.55	5210
UNKL_t2	1.02	0.108922075	0.05	104	2.86	6660	0.02	120	1.69	7359	0.01	130	0.55	8292
SLC25A51	1.17	1.20E-08	0.19	65	2.86	6661	0.08	81	2.5	6827	0.25	95	11.08	3488
HOXB7	1.03	0.108907462	0.05	107	2.86	6662	0.08	125	6.12	4716	0.02	131	1.58	7704
PPFIA3	1.09	2.23E-05	0.05	108	2.86	6663	-0.03	104	-1.58	9535	0.22	110	13.32	2791
FZD1	1.02	0.011298153	0.04	125	2.85	6664	-0.07	143	-6.32	12336	-0.02	135	-1.64	9518
CD40	-1.07	0.086556829	0.05	111	2.85	6665	-0.13	104	-6.78	12554	-0.02	116	-1.36	9370
NGB	1.03	0.036758321	0.05	108	2.85	6666	-0.12	141	-10.67	14120	0.06	135	4.97	5943
SMPD2	1.15	3.56E-08	0.07	88	2.85	6667	0.1	62	1.22	7669	0.05	84	1.79	7575
CLP1	1.12	6.51E-05	0.05	103	2.85	6668	0.04	131	3.32	6306	0.11	140	10.24	3784
TAS2R1	1.02	0.035613795	0.08	87	2.85	6669	0.04	140	3.17	6399	0.03	80	0.83	8123
RNF175	-1.01	0.687621446	0.05	107	2.85	6670	-0.05	103	-2.75	10309	-0.08	84	-2.58	9991
IRX1	1.03	0.039839083	0.05	113	2.84	6671	-0.03	92	-1.37	9409	0.01	111	0.9	8082
HIST1H1D	1.53	4.26E-07	0.08	84	2.84	6672	-0.03	113	-1.95	9775	0.14	91	5.85	5524
STX10	1.15	2.83E-06	0.06	96	2.84	6673	0.12	125	9.1	3223	0.14	125	10.4	3724
RABAC1	-1.02	0.659161848	0.08	84	2.84	6674	-0.02	95	-0.94	9103	-0.05	100	-2.4	9895
RAB18	1	0.978419096	0.03	157	2.84	6675	0.08	137	7.27	4111	0.16	134	13.09	2855
ZNF610	1.03	0.316193044	0.04	119	2.84	6676	0.11	121	7.58	3951	0.03	137	2.6	7131
ARHGAP26	1.01	0.851307019	0.03	151	2.83	6677	0.1	127	8.05	3712	0.07	136	6.03	5440

RNASE1	1.19	0.014365462	0.06	99	2.82	6678	-0.01	106	-0.78	9000	0.05	102	2.51	7177
PCYT1B	1.02	0.019905477	0.04	126	2.82	6679	0.06	135	5.16	5242	0.14	150	13.91	2613
DSG2	-1.04	0.330792769	0.03	131	2.81	6680	-0.01	145	-1.2	9292	-0.02	152	-2.08	9747
E2F4_t1	-1.02	0.501398033	0.04	114	2.81	6681	0.02	124	1.14	7712	NA	NA	NA	NA
KCNH5_t1	1.03	0.004115406	0.04	114	2.81	6682	0.02	120	1.72	7329	0.02	124	1.65	7648
ZNF225	1.06	0.029947841	0.03	145	2.81	6683	0.1	117	6.38	4573	0.01	126	0.78	8149
NPEPL1_t1	-1.01	0.868593052	0.04	126	2.81	6684	NA	NA	NA	NA	NA	NA	NA	NA
UBE2U	1.03	0.006208681	0.05	106	2.81	6685	0.05	104	2.8	6613	0.12	125	8.63	4373
MED21	1.06	0.125402411	0.03	137	2.81	6686	0.05	132	4.03	5887	0.14	123	10.04	3865
ST7-AS1	1.21	4.93E-06	0.04	123	2.81	6687	0.02	141	2.23	6993	0.05	144	4.52	6168
CDHR4	1.09	6.26E-07	0.06	101	2.81	6688	0.04	108	2.1	7084	0.06	90	2.54	7158
NPTX1	1.16	0.001362723	0.04	122	2.8	6689	0.04	125	3.33	6298	0.15	140	13.63	2703
WDR77	1.12	0.000192789	0.04	115	2.8	6690	0.1	123	7.23	4127	0.1	127	7.89	4668
CDC42EP4	1.12	0.000335404	0.04	115	2.8	6691	0.11	120	7.38	4047	0.08	123	5.53	5672
SPAG11B_t1	1.04	5.81E-05	0.06	100	2.79	6692	0.04	101	1.79	7288	0.06	116	3.97	6426
OSBPL7	1.02	0.039894696	0.03	147	2.79	6693	0.07	135	5.88	4853	0.08	117	5.65	5603
LEPREL4	1.19	4.63E-05	0.03	133	2.79	6694	0.09	131	7.21	4135	0.06	121	4.41	6241
PIH1D2	1.09	5.35E-05	0.13	72	2.79	6695	0.15	74	3.5	6187	0.05	92	2.13	7378
CALHM1	1.07	1.37E-08	0.03	132	2.79	6696	0.03	145	2.39	6892	0.06	110	3.75	6542
SEMA6D_t1	1.03	0.002805797	0.05	105	2.79	6697	0.06	111	3.56	6139	0.09	121	6.14	5384
UPF1	1.07	0.034245732	0.04	119	2.78	6698	0.2	125	15.26	1210	-0.02	126	-1.41	9397
ATXN2	1.09	0.004318571	0.03	131	2.78	6699	0.26	107	14.86	1316	0.1	116	6.53	5221
SALL2	1.08	0.401069504	0.04	124	2.78	6700	0.04	136	3.81	5997	0	129	-0.29	8784
BOD1L1_t1	1.06	0.008341372	0.03	134	2.78	6701	NA	NA	NA	NA	0.13	116	8.43	4453
OSGEP	1.01	0.821867692	0.05	106	2.78	6702	-0.01	114	-0.48	8806	-0.09	112	-5.46	11223
ZNF581	1.1	0.00016751	0.06	100	2.78	6703	0.1	120	6.85	4326	0	108	0	8616
DSCR4	1.13	0.000430898	0.04	115	2.78	6704	0.04	120	2.66	6706	0.13	98	6.32	5310
MAP3K10	1.17	5.22E-07	0.05	101	2.77	6705	0.07	104	3.92	5942	0.05	123	3.99	6418
GTPBP5_t1	1.06	0.05202486	0.05	104	2.77	6706	0.04	140	3.42	6238	NA	NA	NA	NA
SLC9A7	1	0.904811267	0.03	144	2.77	6707	-0.11	136	-9.26	13608	-0.05	141	-4.73	10950
SOX21	1.03	0.001277963	0.03	146	2.77	6708	-0.03	116	-1.93	9763	0.15	151	14.66	2415
COX10	1.02	0.508031766	0.04	125	2.77	6709	-0.05	163	-6.04	12206	0.11	142	10.26	3777
GHDC	1.13	0.000137205	0.04	115	2.77	6710	-0.03	120	-2.1	9904	0.1	122	7.45	4851
TPM4_t1	-1.11	0.185147612	0.04	127	2.77	6711	NA	NA	NA	NA	NA	NA	NA	NA
PINX1	1.03	0.096512343	0.15	68	2.77	6712	0.07	76	1.91	7209	0.06	56	0.38	8390
ANAPC4	1.14	0.013276101	0.06	95	2.76	6713	0.19	83	6.26	4637	0.11	97	5.06	5895
BBS5_t1	-1.01	0.831257392	0.04	120	2.76	6714	NA	NA	NA	NA	0	136	0.4	8371
EIF4A3	1.1	0.003183817	0.04	120	2.76	6715	0.15	123	11.06	2447	0.03	107	1.59	7693
VNN2	-1.14	0.263034949	0.03	140	2.76	6716	0.01	119	0.58	8086	-0.03	122	-2.11	9760
MAGEA2B	2.79	2.86E-11	0.04	114	2.76	6717	0.06	111	3.66	6082	0.17	120	12.1	3162
NEDD4_t1	1.16	0.050242891	0.03	133	2.75	6718	NA	NA	NA	NA	0.17	134	14.65	2421
SETMAR	1.03	0.000465238	0.07	90	2.75	6719	-0.02	53	-0.07	8536	0.15	93	6.41	5269
AARS	1.03	0.429303216	0.05	104	2.75	6720	-0.14	126	-10.87	14199	-0.02	104	-1.28	9334
NDUFA12	1.14	0.001421859	0.05	103	2.75	6721	0.04	97	2.08	7097	0.19	102	9.95	3896
FBN3	1.05	0.01032462	0.05	103	2.75	6722	-0.07	113	-4.17	11188	-0.01	125	-1.12	9259
SH3GLB1	-1.08	0.142769304	0.03	134	2.75	6723	0.1	137	8.56	3464	-0.04	150	-3.53	10405
EXTL3	1.15	0.004314992	0.04	124	2.75	6724	0.11	128	8.88	3338	0.03	119	2.25	7319
ADAM30	-1	0.910545625	0.04	124	2.75	6725	0.06	109	3.36	6273	0	104	-0.01	8629
DPP4	1.16	0.051478577	0.04	120	2.75	6726	0.02	125	1.23	7658	0.16	124	11.49	3357
FAM9A	1.09	0.003401777	0.04	120	2.74	6727	0.03	126	2.19	7014	-0.06	125	-4.31	10759
LY6H	1.1	1.42E-05	0.07	88	2.74	6728	0.18	123	13.39	1718	0.11	105	5.89	5497
U2AF1	-1	0.925927315	0.05	109	2.74	6729	0.05	87	1.95	7171	-0.09	100	-4.67	10922
USP40	-1	0.94670005	0.04	127	2.74	6730	0.09	134	7.78	3839	0	114	-0.06	8657
ZNF32	1.11	0.002740907	0.08	84	2.74	6731	0.02	97	1.11	7739	0.09	80	2.64	7110
ARL2	1.1	0.003330728	0.07	90	2.74	6732	0.15	149	14.87	1312	0.01	90	0.5	8316
SLAMF6	-1	0.887236516	0.03	147	2.74	6734	0.05	148	5.19	5222	-0.07	139	-6.24	11542
ZNF583	1.04	0.171406422	0.04	128	2.74	6735	0.09	120	6.32	4607	0.07	147	6.82	5096
MLL3_t1	1.02	0.493983798	0.03	146	2.73	6736	NA	NA	NA	NA	NA	NA	NA	NA
DMTF1	1.12	0.033525681	0.04	121	2.73	6737	0.19	122	13.53	1676	0.11	124	8.08	4590
UXT	1.07	0.014947123	0.07	87	2.73	6738	-0.04	97	-1.77	9672	0.04	72	0.98	8033
ALOX15	1.01	0.307206191	0.04	112	2.73	6739	-0.14	123	-10.38	14030	0.01	110	0.86	8102
FBXL22	1.05	0.000226064	0.04	116	2.73	6740	-0.04	157	-3.85	11003	0	108	0.2	8493
EMC9	1.2	3.19E-06	0.05	104	2.73	6741	-0.08	72	-1.82	9706	0.11	94	4.87	5992
CNNM2_t2	1.06	0.009695305	0.04	119	2.73	6742	-0.08	165	-8.73	13412	0.09	125	6.92	5066
HEATR5B	-1.01	0.795997028	0.04	125	2.72	6743	0.1	117	6.64	4428	0.07	116	4.88	5984
ATP6V1B1	1.08	1.49E-05	0.04	113	2.72	6744	0.17	129	13.23	1765	0.06	102	2.95	6962
CYP2W1	1.09	0.000905923	0.05	104	2.72	6745	0.05	134	3.8	6003	0.1	120	7.3	4912
ADTRP	1.01	0.884545603	0.03	128	2.72	6746	0.02	108	1.18	7693	-0.09	120	-6.18	11511
TNIP1	1.25	1.91E-09	0.04	126	2.72	6747	0.06	115	3.89	5954	0.1	133	8.02	4612
MAPK14_t2	-1.03	0.325125988	0.04	118	2.72	6748	0	80	-0.11	8559	0.01	76	0.29	8448
NR2C1_t1	1.14	3.01E-06	0.04	116	2.72	6749	0.17	136	14.26	1462	0.09	95	4.17	6347
SLC47A2	1.09	0.009972114	0.04	120	2.72	6750	-0.12	134	-9.93	13856	0.1	121	6.83	5095
PITX2	1.1	0.002620875	0.04	117	2.71	6751	0.03	122	2.2	7012	0.01	127	0.8	8131
HYLS1	1.12	0.005206529	0.03	139	2.71	6752	-0.02	114	-1.18	9272	0.08	122	5.83	5529
GLIPR1L2_t1	1.03	0.047134412	0.04	113	2.71	6753	NA	NA	NA	NA	-0.01	121	-0.93	9148
CCDC121_t1	1.07	0.012033539	0.03	133	2.71	6754	NA	NA	NA	NA	0.16	125	11.94	3218
OBP2B	1.02	0.072277072	0.07	89	2.71	6755	-0.05	117	-3.2	10598	0.06	100	3.08	6891
EZH1	1.08	0.004048804	0.03	129	2.71	6756	0.06	126	4.65	5522	0.06	131	5.1	5880
SPAG11B_t2	1.01	0.333323918	0.05	106	2.71	6757	NA	NA	NA	NA	0.03	120	1.92	7499
MRPL43_t2	1.05	0.153845815	0.04	111	2.71	6758	0.07	146	7	4244	0.11	120	7.59	4798
CHST5	1.09	0.002520715	0.05	107	2.7	6759	-0.01	153	-0.52	8831	0.03	131	2.58	7143
BGLAP	1.2	3.86E-09	0.08	82	2.7	6760	-0.01	109	-0.78	8997	0.08	127	5.99	5458
EFHC1	-1	0.942983128	0.03	130	2.7	6761	0.14	147	13.83	1588	-0.06	162	-6.25	11553
POPDC2_t1	1.06	0.128533406	0.07	91	2.69	6762	NA	NA	NA	NA	NA	NA	NA	NA
TRADD	1.05	0.137370231	0.04	114	2.69	6763	0	132	-0.02	8505	-0.12	127	-9.29	12685
SPDYA	1.08	7.22E-05	0.04	125	2.69	6764	0.11	137	9.51	3054	0.17	100	8.74	4325
XYLT1	-1.03	0.13235004	0.03	134	2.69	6765	0.08	167	8.99	3278	-0.08	139	-6.68	11730
MS4A8B	1.12	0.002054558	0.06	97	2.69	6766	0.1	111	6.14	4708	0.05	126	3.42	6700
DAB2	-1.1	0.17611296	0.04	124	2.69	6767	0.12	123	8.48	3511	-0.07	120	-4.87	11004
MORN5	1.02	0.120783873	0.06	98	2.68	6768	0.1	99	5.06	5301	0.03	100	1.41	7804
ANKMY1_t1	1.1	6.08E-06	0.06	96	2.68	6769	0.04	102	1.88	7225	-0.02	103	-1.28	9335
MPP6	1.33	5.87E-06	0.06	93	2.68	6770	0.19	138	16.28	985	0.23	115	14.69	2409
CCDC58	1.16	1.62E-05	0.06	93	2.68	6771	0.09	109	5.23	5204	0.14	95	6.45	5260
AFTPH_t2	1.08	0.049192977	0.03	139	2.68	6772	0.06	110	3.8	6007	0.11	154	11.11	3466
RANGAP1	1.11	0.011602814	0.05	108	2.68	6773	0.03	102	1.47	7511	0.03	121	2.27	7311
ZNF25_t1	-1.03	0.489457478	0.03	137	2.68	6774	NA	NA	NA	NA	0.07	147	7.18	4960
KRTAP4-5	-1	0.921803156	0.04	117	2.68	6775	-0.15	124	-11.08	14267	-0.01	132	-1.21	9302
LACTB2	1.05	0.531005958	0.03	128	2.67	6776	-0.02	111	-0.94	9102	0.17	121	12.02	3189
KLHL35	1.01	0.659461319	0.06	93	2.67	6777	0.08	138	6.65	4417	-0.1	106	-5.88	11393
GTF2A1	1.09	1.75E-08	0.02	163	2.67	6778	-0.02	130	-1.62	9570.5	0.15	153	14.99	2340
LRIG2	-1.01	0.616541673	0.04	123	2.67	6779	-0.02	108	-1	9148	-0.02	142	-1.74	9575
HLA-A	1.13	0.083215581	0.03	132	2.67	6780	0.06	110	3.4	6246	-0.06	122	-4.01	10645
DYNLT1	1.12	0.008981434	0.04	118	2.67	6781	0.13	117	8.92	3321	0.02	126	1.38	7816
IFLTD1	1	0.570185015	0.03	135	2.66	6782	0.02	120	1.08	7750	0.02	112	0.96	8042
SEPN1	-1.02	0.539659199	0.03	129	2.66	6783	0.03	138	2.91	6544	-0.08	128	-6.55	11670
AGAP7	1.07	0.109289651	0.03	144	2.66	6784	0.03	107	1.82	7261	0.14	111	8.4	4468

ASB8_t1	1.02	0.386716731	0.04	117	2.66	6785	NA	NA	NA	NA	NA	NA	NA	NA
SUGP1	1.19	9.42E-11	0.04	112	2.66	6786	0.03	99	1.48	7504	0.05	104	2.61	7127
ZNF524	1.06	0.080892726	0.08	84	2.66	6787	-0.01	113	-0.5	8814	0.08	89	3.08	6886
GSG1L	1.01	0.168952185	0.03	150	2.65	6788	0.05	138	4.27	5739	0.04	122	2.78	7040
FAM47A	1.03	0.008292583	0.05	108	2.65	6789	-0.02	102	-1.19	9279	0	108	-0.01	8623
SLC25A24	-1.14	0.202127743	0.03	134	2.65	6790	0.11	111	6.57	4468	-0.05	135	-3.85	10565
KIAA0753	-1.09	0.024263923	0.03	126	2.65	6791	0.03	127	2.62	6741	-0.1	140	-9.08	12618
HMX1	1.02	0.143261241	0.04	109	2.65	6792	0	104	0.03	8445	-0.02	92	-0.8	9085
AGPAT6	1.08	0.132319919	0.04	125	2.65	6793	0	142	-0.09	8548	0.07	129	5.92	5486
FOXA1	1.1	0.198468615	0.03	139	2.65	6794	0	121	0.34	8245	-0.05	132	-4.03	10649
SLFNL1	1.04	0.0021248	0.03	130	2.65	6795	0.11	122	7.8	3831	0.04	123	3.07	6898
SNX15_t1	1.1	1.00E-07	0.04	112	2.65	6796	-0.04	136	-3.3	10670	0.09	127	7.19	4952
MLPH_t1	1.07	0.03591645	0.03	136	2.64	6797	-0.06	128	-4.39	11319	-0.01	131	-1	9202
INHA	1.06	0.014816371	0.04	120	2.64	6798	-0.04	125	-3.1	10541	-0.01	111	-0.65	9011
SLC10A3	-1.03	0.639484921	0.04	113	2.64	6799	0.17	147	16.23	995	-0.1	123	-7.6	12075
RNF19B	1.07	0.001277963	0.05	106	2.64	6800	0.12	109	7.18	4150	0.06	96	2.78	7037
RPS23	1.21	0.000498103	0.03	139	2.64	6801	0.13	139	11.86	2167	0.12	132	9.64	4007
LRRC31	1.18	3.83E-05	0.04	116	2.63	6802	-0.03	112	-1.8	9692	0.1	104	5.16	5851
PTPRA	1.09	0.011238572	0.05	102	2.63	6803	0.1	118	6.5	4513	0.03	123	2.14	7374
SUGP2_t1	1.09	0.0032078	0.04	120	2.62	6804	0.12	104	6.68	4409	0.04	129	3.19	6837
RAI1_t1	1.1	0.00501412	0.04	111	2.62	6805	NA	NA	NA	NA	0.06	134	5.42	5726
KRT17	1.11	0.01111714	0.04	112	2.62	6806	0.05	105	2.94	6526	-0.02	87	-0.62	8991
PDE5A_t1	1.07	3.67E-10	0.03	134	2.62	6807	0.06	124	4.32	5707	0.1	145	9.31	4115
SOX30	1.05	7.52E-05	0.04	124	2.62	6808	-0.02	124	-1.52	9509	0.05	129	3.61	6607
AMMECR1L	1.01	0.689094138	0.04	121	2.62	6809	0.16	135	13.41	1710	0	135	0.14	8532
KIAA2022	1.02	0.050995291	0.03	140	2.61	6810	0.09	148	8.34	3577	0	140	-0.33	8806
SS18L2	1.09	0.000797892	0.03	130	2.61	6811	0.01	105	0.49	8139	0.08	124	5.91	5489
ACTN4	1.02	0.469695873	0.04	114	2.61	6812	-0.02	124	-1.64	9586	-0.01	118	-0.96	9179
AKAP10	-1.1	0.000663803	0.03	142	2.61	6813	0.03	145	2.88	6561	0	135	0.04	8592
MGAT3	1.01	0.153412562	0.04	122	2.61	6814	-0.03	129	-2.73	10296	0.01	132	0.9	8079
PHF19_t2	1.08	0.001504136	0.04	122	2.61	6815	0.02	145	2.15	7045	-0.02	127	-1.66	9527
PPA2	-1.1	0.033299819	0.03	128	2.61	6816	-0.07	127	-5.72	12039	-0.03	128	-2.05	9737
ADAD1	1.12	0.002261478	0.04	117	2.61	6817	0.07	118	4.48	5620	0.11	86	4.03	6405
PTGES3L-AARS	1.03	0.017365324	0.09	80	2.61	6818	0.1	105	5.38	5134	0.02	100	0.84	8116
HRK	1.06	0.068996665	0.03	128	2.61	6819	0.1	123	7.5	3990	-0.03	113	-1.88	9659
SLC22A8	1.08	5.08E-10	0.06	97	2.61	6820	0.11	113	6.89	4305	0.02	99	0.9	8080
MORC4	1.15	0.073292572	0.04	108	2.61	6821	-0.01	123	-0.59	8868	0.16	113	9.77	3958
ABCC6_t1	1.63	2.76E-08	0.06	96	2.6	6822	0.08	146	7.84	3815	0.22	113	13.58	2713
ZNF333	1.02	0.187773144	0.03	131	2.6	6823	0.01	140	1.07	7757	-0.02	120	-1.19	9292
RPS12	1.3	5.95E-06	0.09	78	2.6	6824	0.22	82	7.19	4146	0.1	70	2.06	7418
PGGT1B	-1	0.985995262	0.03	136	2.6	6825	0.05	132	3.83	5990	0.1	125	7.55	4815
SELT	1.08	0.033787685	0.03	133	2.6	6826	0.06	131	4.61	5541	0.01	136	0.73	8172
SYNJ2BP	1.04	0.179827096	0.03	152	2.6	6827	-0.04	135	-3.26	10644	0.1	126	7.36	4891
ESPN	1.02	0.015857199	0.04	119	2.6	6828	-0.11	135	-9.43	13669	-0.07	113	-4.64	10911
ST8SIA3	1.04	0.024130804	0.03	153	2.59	6829	0.06	163	7.11	4194	0.12	157	12.34	3093
HSPB6	1.01	0.395831937	0.04	115	2.59	6830	-0.07	131	-5.6	11973	-0.07	115	-4.31	10756
ACTRT3	-1.07	0.002166366	0.03	130	2.59	6831	-0.14	108	-7.84	13050	-0.04	119	-2.96	10165
MPL	1.11	6.37E-08	0.03	130	2.59	6832	0.03	132	2.47	6847	0.11	150	10.94	3530
USP12	-1.03	0.157708517	0.03	136	2.59	6833	-0.03	123	-2.05	9861	0.05	140	4.28	6301
B3GALT1	1.06	1.14E-05	0.04	108	2.58	6834	0.01	114	0.37	8224	0.04	131	3.38	6728
MRPL35_t1	1.02	0.597419035	0.04	116	2.58	6835	-0.02	136	-1.64	9584	0.06	128	4.56	6147
IFI6	-1.08	0.279141635	0.05	104	2.58	6836	-0.1	131	-8	13113	-0.06	104	-3.07	10211
PEX5L	1.05	1.90E-07	0.03	132	2.58	6837	0.07	139	6.09	4732	0.1	140	9.09	4188
PAK6	1.06	0.037357736	0.04	117	2.58	6838	0.08	150	8.14	3675	-0.01	142	-0.96	9175
RPL6	1.26	3.67E-06	0.08	81	2.57	6839	0.2	68	3.57	6130	0.2	77	5.27	5790
CHRDL2	-1.14	0.301792528	0.06	96	2.57	6840	-0.05	98	-2.19	9957	0	128	0.13	8535
SERTAD4	1.17	0.002512625	0.03	124	2.57	6841	0.07	126	5.12	5266	-0.01	124	-0.68	9024
CIAO1	1.18	1.53E-07	0.03	128	2.56	6842	0.06	143	5.73	4933	0.11	157	11.35	3400
LATS1	1.02	0.026221263	0.04	123	2.56	6843	0.01	145	0.83	7910	0.07	135	6.27	5327
SEC22B	1	0.996732182	0.04	116	2.56	6844	0.06	138	5.63	4988	-0.04	132	-3.5	10394
SLC35D3	1.02	0.112981561	0.04	116	2.56	6845	-0.12	127	-9.52	13706	0.01	138	1.21	7906
RHD	1.01	0.331604781	0.03	149	2.56	6846	0.07	139	6.16	4696	0.05	134	3.9	6472
CALCR	1.09	0.00071468	0.04	119	2.56	6847	0.1	127	7.77	3851	0.03	143	3.19	6836
PPT1_t1	-1.07	0.083601092	0.03	144	2.55	6848	NA	NA	NA	NA	NA	NA	NA	NA
GDF10	1.04	0.09562822	0.03	125	2.55	6849	0	120	0.34	8244	-0.03	113	-1.75	9587
PHOSPHO2	1.34	1.56E-14	0.25	60	2.55	6850	0.1	55	0.52	8126	0.2	60	2.04	7427
SLC50A1	1.43	4.49E-15	0.03	139	2.55	6851	0.17	110	10.28	2765	0.16	114	10.3	3763
PPP1R9B	1.03	0.016314643	0.04	122	2.55	6852	0.05	154	5.39	5127	0.1	130	8.04	4603
ADAMTS10	-1.04	0.033839071	0.04	107	2.54	6853	0.04	145	3.8	6008	-0.06	106	-3.4	10357
TAP2_t1	1.11	7.85E-12	0.03	124	2.54	6854	0	139	0.25	8294	0.03	143	2.39	7235
MAP1LC3C	1.05	2.80E-05	0.05	105	2.54	6855	0.04	120	3.13	6419	0.12	121	8.77	4312
FOXJ3	-1.08	0.038240527	0.03	140	2.54	6856	-0.05	159	-5.29	11816	0	144	-0.38	8836
AHCY_t1	1.06	0.235981094	0.03	129	2.54	6857	NA	NA	NA	NA	0.06	102	3.27	6793
ITFG3	-1.03	0.524949916	0.04	108	2.54	6858	-0.03	127	-2.02	9839	-0.06	145	-5.63	11292
CCDC50_t1	-1.05	0.149746099	0.03	145	2.53	6859	-0.02	152	-2.4	10085	-0.03	151	-3.51	10401
RBPJL	1.05	2.33E-05	0.03	130	2.53	6860	-0.03	131	-2.41	10094	0.12	104	6.75	5129
USP25	-1.1	0.000665687	0.02	153	2.53	6861	0.02	126	1.46	7517	-0.07	120	-4.74	10959
HIST1H2AA	1.25	4.95E-15	0.08	80	2.53	6862	0.1	97	4.88	5406	0.25	106	13.9	2619
CHPT1	1.16	0.039669624	0.05	101	2.53	6863	-0.09	104	-4.97	11642	0.18	108	10.29	3769
SPTSSB	1.12	0.007392152	0.04	118	2.53	6864	0.07	118	4.55	5583	-0.06	136	-5.51	11244
PAGE5	1.98	6.54E-06	0.03	126	2.53	6865	-0.07	119	-4.72	11497	0.03	108	1.74	7606
ZNF597	1.01	0.545324498	0.04	119	2.53	6866	0.08	144	7.14	4175	0.2	108	11.69	3298
CCT8	1.05	0.469911425	0.04	107	2.53	6867	0.1	106	5.74	4928	0.09	124	6.4	5274
DNM1	1.09	0.004216955	0.03	132	2.52	6868	0.02	125	1.44	7530	0.01	115	0.44	8346
B4GALNT3	1.01	0.162576084	0.04	107	2.52	6869	-0.04	124	-2.94	10437	-0.02	100	-0.79	9081
POU3F1	-1	0.892107662	0.04	121	2.52	6870	-0.02	126	-1.79	9682	0.04	115	2.52	7172
CTR9	1.18	0.022713547	0.03	134	2.52	6871	0.04	118	2.77	6629	0.14	131	11.26	3426
CHPF2	1.04	0.285628771	0.05	98	2.52	6872	0.14	92	5.67	4963	-0.07	88	-2.49	9948
KIAA1524	1.03	0.000850653	0.03	132	2.51	6873	0.17	120	11.85	2169	-0.07	143	-6.66	11719
CLIC5_t1	1.24	0.000531757	0.03	147	2.51	6874	0.04	145	4.2	5776	NA	NA	NA	NA
BSN	1.26	5.46E-06	0.03	148	2.51	6875	0	139	0.1	8402	0.08	136	6.87	5079
PANX2	1.11	0.000109525	0.05	104	2.51	6876	-0.06	124	-4.42	11339	0.06	120	4.29	6296
EIF4G3	1.12	0.008988939	0.04	108	2.51	6877	0.2	120	13.91	1559	0.15	132	12.16	3149
MAP2K7	1.03	0.001843	0.03	134	2.5	6878	-0.01	146	-0.77	8992	0.03	132	2.27	7310
ERGIC2	-1.04	0.370748164	0.05	101	2.5	6879	0.1	103	5.37	5139	0.03	104	1.62	7672
ST6GALNAC4	1.1	7.66E-06	0.03	122	2.49	6880	0.1	151	10.45	2704	0.03	102	1.72	7620
NAA50	-1.09	0.027017706	0.03	149	2.49	6881	-0.01	131	-1.08	9205	0.05	140	4.43	6232
TAS2R5	1	0.998077181	0.05	99	2.49	6882	-0.04	136	-3.76	10954	0.03	105	1.66	7642
TEX28	1	0.839488257	0.04	109	2.49	6883	0.03	93	1.19	7685	0.03	92	1.2	7916
HIST1H1B	1.18	3.08E-15	0.25	60	2.49	6884	0.2	76	5.16	5248	0.27	70	5.39	5738
DIAPH3_t2	1.1	2.06E-10	0.25	60	2.49	6885	0.24	52	0.48	8149	0.29	105	15.77	2138
PML_t1	1.07	0.026471829	0.04	111	2.49	6886	0.12	124	9.01	3265	-0.08	100	-4.15	10694
RAB3C	1.52	1.80E-05	0.03	136	2.49	6887	0.01	103	0.72	7988	0.1	100	5.06	5898
DRAP1_t1	1.13	1.04E-05	0.05	104	2.49	6888	0.2	88	7.78	3838	NA	NA	NA	NA
CDK13_t1	1.15	1.28E-06	0.03	137	2.49	6889	0.1	143	8.96	3296	0.21	130	16.44	1969
TPP1	1.04	0.3369183	0.03	138	2.48	6890	-0.06	133	-4.89	11586	0.03	160	3.83	6510

NUDT12	-1.01	0.852097657	0.03	136	2.48	6891	-0.09	126	-6.85	12587	0.15	144	14.06	2581
FRMD5	1.03	0.044450355	0.04	107	2.48	6892	-0.09	104	-5.05	11689	0.06	114	3.56	6637
OSBPL10	-1.02	0.306532861	0.04	113	2.48	6893	0	142	-0.35	8716	-0.03	138	-2.24	9819
EPSTI1	-1.3	5.86E-06	0.03	125	2.48	6894	-0.07	125	-5.39	11865	-0.23	124	-17.27	14843
KRT26	-1	0.893258506	0.04	114	2.48	6895	-0.16	120	-10.88	14201	0.01	135	0.73	8175
CLDN15	1.25	6.05E-07	0.06	91	2.48	6896	-0.07	127	-5.39	11861	0.1	145	9.66	3999
ACP6	1.38	4.54E-15	0.08	80	2.47	6897	0.08	94	3.34	6288	0.21	82	6.68	5162
DDIT4L	1.1	0.046196282	0.04	120	2.47	6898	0.05	125	3.56	6141	0.09	124	6.86	5082
GPR6	1.09	1.38E-10	0.04	110	2.47	6899	-0.06	126	-4.28	11256	0.09	125	6.49	5240
NUP188	1.03	0.077745565	0.04	115	2.47	6900	-0.09	145	-8.3	13242	0.02	115	1.06	7987
APOA4	-1.03	0.424769157	0.03	127	2.47	6901	-0.02	102	-1.29	9359	-0.1	120	-6.92	11814
LAMP1	1.02	0.82211238	0.04	110	2.47	6902	-0.01	115	-0.81	9020	0.07	120	5.19	5834
RAP1GAP_t1	-1.01	0.842211351	0.03	129	2.47	6903	0.01	125	0.99	7813	NA	NA	NA	NA
HSF4	1.14	9.53E-06	0.06	92	2.46	6904	-0.06	109	-3.48	10783	0.11	103	5.73	5568
PCSK5	1.06	0.452250978	0.05	103	2.46	6905	0.09	92	3.9	5952	0.08	88	3.14	6856
ZNF703	1.05	0.002240102	0.03	132	2.46	6906	-0.04	118	-2.6	10220	-0.02	104	-0.93	9154
ZNF256	1.07	0.245519674	0.07	84	2.46	6907	0.15	95	6.72	4392	0.05	80	1.57	7707
LYPD3	1.04	0.002593065	0.03	123	2.45	6908	0.05	131	4.19	5785	-0.01	104	-0.53	8941
PWP2	-1.01	0.768415951	0.05	100	2.45	6909	0	130	-0.16	8588	-0.07	111	-4.42	10813
ACTL8	1.17	0.005277878	0.04	106	2.45	6910	-0.04	137	-3.57	10854	0.11	123	7.74	4738
DDOST	1.12	5.93E-05	0.05	101	2.45	6911	0.07	143	6.15	4699	-0.01	150	-0.96	9176
BRAP	1.04	0.092368899	0.03	138	2.45	6912	0.04	130	3.58	6124	0.04	134	3.26	6802
SMC1B	1.16	0.000147094	0.04	113	2.44	6913	-0.02	120	-1.72	9634	0.01	132	1.12	7956
ZFHX4	-1.11	0.034249355	0.02	151	2.44	6914	-0.1	125	-7.85	13056	-0.01	134	-0.63	8995
CCBL1	1.19	5.64E-06	0.04	109	2.44	6915	-0.09	110	-5.19	11771	0.17	108	9.69	3990
ZNF230	1.05	0.005047044	0.03	132	2.44	6916	0.02	133	1.47	7510	-0.04	147	-3.85	10556
TNFRSF10D	-1.01	0.558013541	0.02	149	2.43	6917	-0.04	131	-3.42	10741	-0.01	129	-0.86	9114
PLA2G1B	1.35	4.05E-09	0.07	85	2.43	6918	0.03	122	2.37	6906	0.1	119	7.04	5018
KRT20	1.19	0.000280145	0.04	109	2.42	6919	0.09	129	7.14	4172	0.02	122	1.76	7591
FAN1	1.19	0.000508347	0.04	115	2.42	6920	0.03	117	2.23	6995	0.18	119	12.51	3040
PRPH	1.09	0.000192585	0.03	128	2.42	6921	-0.04	120	-2.5	10150	0.04	106	2.02	7439
PPP1R7	-1.04	0.186166234	0.06	92	2.42	6922	-0.1	126	-7.92	13081	-0.1	118	-6.83	11786
PNLDC1	1.02	0.163268819	0.04	108	2.42	6923	0.07	135	5.85	4873	-0.05	100	-2.52	9959
OGDH_t2	1.04	0.173276031	0.03	135	2.42	6924	-0.05	121	-3.24	10630	0.01	142	1.17	7936
LAS1L	1.14	1.18E-05	0.06	88	2.41	6925	-0.01	121	-0.96	9116	0.08	98	3.81	6522
GTPBP1	-1.04	0.29276311	0.03	126	2.41	6926	0.05	142	4.23	5761	-0.08	123	-5.67	11304
RPL12	1.03	0.511226504	0.04	116	2.41	6927	0.18	110	10.9	2518	-0.02	96	-0.95	9167
GTF3C5	1.1	0.000692972	0.04	108	2.41	6928	-0.02	99	-1.05	9182	-0.01	120	-0.59	8976
RBP2	1.1	5.17E-05	0.05	100	2.4	6929	0.14	127	10.67	2612	0.16	119	11.21	3439
TMEM225	1.01	0.236452459	0.04	118	2.4	6930	-0.05	95	-2.34	10050	0.08	111	4.89	5982
SP1	1.02	0.362921846	0.03	134	2.4	6931	0	142	-0.3	8682	-0.03	140	-3.02	10190
AK5	1.04	5.30E-05	0.03	120	2.4	6932	0.08	149	8.02	3737	0.13	130	10.14	3821
IKBIP_t2	1.03	0.481472477	0.05	95	2.4	6933	0.11	98	5.47	5073	0.04	107	2	7450
KLHL1	1.02	0.009361185	0.03	135	2.4	6934	0.12	111	7.36	4055	0.08	139	6.71	5152
BRI3	1.09	0.150437502	0.03	129	2.4	6935	0.06	128	5	5335	0.09	124	6.34	5302
G6PC2	-1.02	0.143964789	0.03	137	2.39	6936	-0.14	130	-10.81	14180	-0.03	125	-1.94	9692
STPG2	-1.02	0.082416137	0.05	101	2.39	6937	-0.09	97	-4.12	11159	-0.04	115	-2.33	9860
ODF4	1.05	1.65E-05	0.04	113	2.39	6938	0.01	136	0.78	7954	-0.07	144	-6.14	11494
LRRFIP2_t1	-1.01	0.882071232	0.03	131	2.39	6939	-0.06	127	-4.75	11513	0.05	115	3.54	6647
FOXD1	1.07	0.009297978	0.03	143	2.39	6940	0.08	127	5.85	4871	-0.1	142	-8.98	12585
NMNAT2_t1	1.02	0.181033582	0.03	128	2.39	6941	NA	NA	NA	NA	0.06	130	5.14	5859
SHC4	1.08	0.00388081	0.03	132	2.39	6942	0.07	127	5.43	5106	0.15	141	14.03	2590
TEAD1	1.05	0.054839972	0.03	136	2.38	6943	0.01	153	0.61	8065	0.09	160	10.4	3723
HDAC10	1.13	5.43E-07	0.06	87	2.38	6944	0.1	120	6.76	4373	0.04	106	2.15	7372
CWC25	-1.04	0.091731495	0.02	147	2.38	6945	-0.04	129	-2.9	10416	-0.05	121	-3.32	10313
DNAJC5B	-1.07	0.017240853	0.04	106	2.38	6946	0.11	131	9.29	3153	-0.15	113	-9.26	12671
PRAMEF1	1.03	0.035761153	0.04	114	2.37	6947	-0.11	107	-6.09	12238	0.02	116	1.35	7835
FLG_t1	-1	0.80055913	0.04	117	2.37	6948	NA	NA	NA	NA	0.06	111	3.42	6702
IGSF8	1.19	5.50E-05	0.03	137	2.37	6949	-0.07	120	-4.98	11647	0.15	100	7.27	4920
VARS2_t1	-1.01	0.860682455	0.04	117	2.37	6950	NA	NA	NA	NA	-0.01	88	-0.22	8747
CEBPZ	-1.03	0.511038743	0.05	101	2.37	6951	-0.07	127	-5.17	11761	0.08	114	4.84	6005
TRHDE	1.02	0.130673764	0.03	129	2.37	6952	0.03	135	2.13	7062	0.05	133	4.03	6402
ZNF385A	-1.02	0.285322201	0.03	130	2.37	6953	-0.11	124	-7.98	13107	-0.11	110	-6.43	11617
NXPH4	1.05	0.000281726	0.04	105	2.36	6954	0.03	135	2.57	6783	0.13	118	9.07	4202
STK38_t1	1.01	0.800869394	0.03	120	2.36	6955	0.07	136	6.36	4583	NA	NA	NA	NA
CORO2A	1.23	0.000900697	0.03	144	2.36	6956	0.02	148	1.61	7420	0.07	130	5.62	5612
ZBTB37	1.09	5.40E-10	0.24	60	2.36	6957	0.2	52	0.41	8201	0.22	52	0.44	8347
GJA9	1.02	0.039357501	0.04	110	2.36	6958	0.06	124	4.6	5553	-0.01	100	-0.37	8829
ARNT_t1	1.04	0.125194603	0.03	134	2.36	6959	NA	NA	NA	NA	0.08	148	8.21	4536
GOLGA6L5	1.03	0.604870215	0.03	144	2.35	6960	0.06	126	4.82	5440	0.16	134	13.78	2654
KLK5	1.02	0.022654855	0.04	109	2.35	6961	-0.08	149	-7.71	12994	0.04	107	2.4	7232
CHMP1A_t1	1.05	0.000541803	0.03	123	2.35	6962	NA	NA	NA	NA	0.03	122	2.29	7297
CXXC1	1.02	0.606919925	0.06	89	2.35	6963	-0.04	114	-2.49	10145	-0.13	115	-8.65	12475
BTBD8	1.05	1.31E-05	0.05	101	2.35	6964	0.05	108	3.14	6414	0.19	105	10.55	3673
PPIB	1.08	0.062372406	0.05	97	2.35	6965	0.07	138	5.75	4927	0.01	120	0.79	8138
LZTS1	1.17	0.029180503	0.03	129	2.35	6966	0.17	139	15.16	1233	-0.01	127	-0.49	8908
FAM210A	-1.01	0.838553034	0.02	151	2.35	6967	-0.04	125	-2.84	10368	0.07	133	5.44	5715
MPV17L	1.13	3.00E-06	0.03	126	2.34	6968	0.07	129	5.24	5195	0.13	145	12.41	3066
FHL3	1.02	0.207079413	0.03	122	2.34	6969	0.03	125	2.61	6747	-0.01	98	-0.33	8807
P2RY4	1.02	0.241325813	0.05	100	2.34	6970	-0.1	124	-7.47	12859	0	108	-0.07	8664
IL17B	1.06	2.80E-05	0.06	88	2.34	6971	0.14	100	6.85	4324	0.11	122	8.08	4593
SPATA4	-1	0.764993438	0.03	122	2.34	6972	0.03	124	2.4	6890	-0.05	115	-3.06	10208
ST3GAL5_t1	-1.1	0.001251414	0.03	138	2.34	6973	NA	NA	NA	NA	NA	NA	NA	NA
CERS5	1.04	0.083892281	0.04	113	2.33	6974	0.03	135	2.55	6803	0.02	118	1.67	7641
PRSS38	1.05	3.08E-05	0.04	104	2.33	6975	0.07	108	3.88	5957	0.15	100	7.56	4810
SPATS2L	1.1	0.069673463	0.02	148	2.33	6976	0.07	150	6.9	4300	0.12	154	12.95	2907
AK8	1.1	0.001588642	0.06	92	2.33	6977	0.12	122	8.69	3412	0.05	84	1.62	7669
GCNT4	1.05	3.12E-06	0.02	148	2.33	6978	0.14	151	14.57	1388	0.14	118	9.23	4148
VIL1_t1	1.33	0.08273612	0.05	94	2.33	6979	NA	NA	NA	NA	0.15	108	8.73	4329
SLC35E1	1.04	0.016898967	0.03	137	2.33	6980	0.02	152	2.48	6839	0.01	153	1.21	7911
CDCP1_t2	1.03	0.101206167	0.05	97	2.32	6981	0.09	109	5.39	5129	0	132	-0.02	8632
EPHA4	1.08	0.006049361	0.03	129	2.32	6982	0.07	145	7.12	4183	0.16	147	15.13	2296
MAGEA4	1.6	7.23E-09	0.03	123	2.32	6983	0.07	110	4.07	5865	0.21	110	12.39	3069
FDCSP	1.18	0.009054206	0.03	121	2.32	6984	0	106	-0.02	8501	0.01	106	0.52	8308
GTSF1	1.65	1.66E-06	0.04	107	2.32	6985	0.04	121	3.18	6393	0.08	100	4.13	6358
DPYSL4	1.07	0.006461697	0.04	115	2.32	6986	0.04	141	3.77	6027	0	114	-0.1	8673
F8A1	1.24	1.75E-06	0.04	104	2.31	6987	0.02	132	1.69	7362	0.1	135	8.84	4282
CLIP4	-1.34	0.003591798	0.03	136	2.31	6988	0.09	128	6.94	4275	-0.12	139	-10.76	13172
LACE1	1.03	0.410358803	0.03	129	2.31	6989	-0.02	108	-1.19	9284	0.03	127	2.63	7120
PRR16_t1	1	0.784758821	0.04	116	2.31	6990	NA	NA	NA	NA	NA	NA	NA	NA
DIRC1	1.05	0.187278756	0.03	141	2.31	6991	-0.04	121	-3.09	10527	0.02	142	2.02	7436
FNBP4	-1.03	0.573232929	0.03	141	2.31	6992	0.08	138	7.26	4115	-0.01	124	-0.49	8906
OTX2	1.03	0.058780321	0.03	126	2.31	6993	-0.1	127	-7.87	13062	0.02	120	1.46	7780
ALPPL2	1.05	4.03E-07	0.04	104	2.31	6994	0.08	127	5.91	4833	0.14	131	11.53	3343
CHRNA9	1.01	0.359617403	0.04	106	2.3	6995	0.03	114	2.17	7025	0.12	129	9.24	4138
SCAF1	1.08	0.002128573	0.05	100	2.3	6996	-0.02	110	-1.13	9240	0.06	107	3.61	6609

NOP9	1.05	0.01112555	0.08	80	2.3	6997	-0.13	88	-5.11	11727	-0.02	97	-0.95	9170
JOSD2	1.05	0.016896787	0.06	88	2.3	6998	-0.09	119	-6.43	12387	0.02	100	0.81	8128
POU2F3	1.07	2.25E-06	0.03	119	2.29	6999	0.03	130	2.08	7093	0.03	134	2.31	7284
SUN1_t1	1.15	0.001163132	0.03	120	2.29	7000	NA	NA	NA	NA	0.14	155	15.21	2266
MTERFD2	1.1	0.00174042	0.02	149	2.29	7001	0.09	96	4.35	5691	-0.01	101	-0.55	8951
FCHSD2	-1.01	0.475919704	0.02	150	2.28	7002	0.03	137	2.64	6728	-0.05	142	-4.84	10996
TMEM150A	1.12	0.000372232	0.04	111	2.28	7003	-0.04	106	-2.34	10047	0.09	120	6.39	5278
NMI	1.06	0.31036413	0.03	116	2.28	7004	0.06	99	3.14	6416	0.06	120	4.09	6378
RTF1_t1	1.03	0.006846633	0.02	148	2.27	7005	NA	NA	NA	NA	0.09	127	7.22	4937
HPSE2_t1	1.02	0.01998868	0.03	125	2.27	7006	0.03	160	2.89	6560	NA	NA	NA	NA
GNG10	1.07	0.124129009	0.02	148	2.27	7007	0.09	117	5.98	4798	0.05	120	3.2	6831
UTS2_t1	-1.02	0.707400712	0.03	126	2.27	7008	0.13	93	5.48	5070	NA	NA	NA	NA
RPL35_t1	1.29	7.61E-10	0.23	60	2.27	7009	NA	NA	NA	NA	NA	NA	NA	NA
WNT2B	1.02	0.170374103	0.02	142	2.27	7010	0.11	162	12.64	1945	-0.08	143	-7.71	12119.5
PPFIA4	1.19	4.34E-05	0.03	116	2.27	7011	0.1	126	7.43	4018	0.11	120	7.81	4708
ZNF233	1.07	0.004872199	0.03	134	2.27	7012	0.11	105	6.01	4778	-0.01	141	-0.97	9183
ACVR1	-1.08	0.062483832	0.03	137	2.27	7013	0.07	145	6.41	4562	0.02	157	2.07	7411
PIWIL1	1.01	0.448612819	0.02	143	2.27	7014	0	116	-0.06	8523	0.07	117	4.45	6213
MFSD1	-1.06	0.197729114	0.04	114	2.27	7015	0	114	-0.07	8530	-0.1	131	-7.99	12236
HOXA11	1.01	0.336992165	0.03	133	2.27	7016	-0.11	131	-8.92	13475	-0.02	128	-1.86	9645
TAZ	1.08	0.005427301	0.03	122	2.27	7017	-0.01	110	-0.38	8733	0.1	107	5.55	5655
M6PR	-1.03	0.293498504	0.02	146	2.26	7018	0.03	117	2.15	7046	-0.1	145	-9.56	12776
RHEBL1	1.07	1.02E-07	0.03	117	2.26	7019	-0.09	124	-6.49	12410	0.05	123	3.89	6473
PIK3R6	1.03	0.000794171	0.03	134	2.26	7020	0.04	125	2.77	6624	-0.06	90	-2.55	9981
HIC2	1.06	0.308834064	0.03	132	2.26	7021	0.01	152	1.53	7479	-0.07	120	-4.78	10978
SLCO1C1	1.01	0.611643995	0.04	112	2.26	7022	0.11	122	8.13	3678	-0.02	126	-1.84	9635
PXK	1.03	0.420314612	0.04	108	2.26	7023	0.1	120	7.13	4180	0.12	108	6.84	5090
ECEL1	1.05	0.138469138	0.05	99	2.26	7024	0.06	104	3.03	6478	0.04	124	2.95	6960
GPR19	1.1	5.28E-05	0.04	111	2.25	7025	-0.09	110	-5.69	12024	0.03	112	1.93	7492
LRRC37A4P	1	0.978250568	0.02	150	2.25	7026	-0.04	155	-3.95	11073	0.04	142	4.14	6357
PRDX2_t2	1.03	0.486728173	0.06	88	2.25	7027	-0.12	113	-7.31	12794	0.08	93	3.43	6698
COQ10A	1.22	0.012454557	0.03	123	2.25	7028	-0.08	111	-4.82	11549	0.14	134	11.78	3273
CCR10	-1	0.917024517	0.06	88	2.25	7029	-0.1	105	-5.62	11984	-0.03	98	-1.2	9296
PRRG3	1.03	0.003761293	0.04	108	2.25	7030	-0.14	121	-9.59	13732	0	98	0.15	8521
TNPO2_t1	1.04	0.0112373	0.03	115	2.25	7031	NA	NA	NA	NA	-0.04	123	-2.77	10073
ANKRD32	1.06	0.083329712	0.04	105	2.25	7032	0.09	108	4.98	5351	0.14	138	12.23	3127
PLCXD1	1.02	0.851123913	0.03	128	2.25	7033	-0.1	133	-8.11	13160	-0.02	136	-1.6	9499
GATA1	-1.01	0.603591667	0.04	104	2.25	7034	0.05	92	1.92	7200	0.02	104	1.25	7878
OR2H1	1	0.645077426	0.02	143	2.24	7035	0.01	143	0.87	7889	0	124	0.06	8579
CENPT	1.01	0.59039986	0.06	88	2.24	7036	0.01	109	0.45	8167	0.07	94	2.9	6992
MYLK3	-1.01	0.697822788	0.02	152	2.24	7037	0.05	139	4.02	5896	-0.12	148	-11.66	13444
LRRC16A	1.03	0.42892172	0.02	153	2.24	7038	-0.02	127	-1.62	9566	0.01	127	0.55	8289
RNPEPL1	1.04	0.159048321	0.04	111	2.24	7039	0.01	125	0.48	8153	-0.05	141	-4.53	10858
IL31RA	1.07	2.88E-11	0.03	115	2.24	7040	0.14	101	7.19	4142	0.13	132	10.75	3589
NFKBIE	1.03	0.641120471	0.04	107	2.23	7041	0.14	127	11.04	2459	-0.05	110	-3.17	10251
SEMA3G	1.09	0.011778978	0.03	120	2.23	7042	0.07	121	4.96	5366	0.11	132	9.08	4193
BIK	1.14	0.03085264	0.04	111	2.23	7043	0.1	125	7.8	3833	-0.05	104	-2.78	10078
PSPH	1.88	4.90E-21	0.03	116	2.22	7044	0.24	132	19.32	476	0.24	116	15.85	2116
MALL_t1	1.08	0.01273105	0.02	140	2.22	7045	NA	NA	NA	NA	-0.03	121	-2.23	9805
UTP14C	-1.06	0.115726175	0.03	136	2.22	7046	-0.06	127	-4.82	11546	-0.07	152	-7.62	12085
ZNF101	1.15	4.47E-06	0.11	71	2.22	7047	0.2	75	5	5338	0.09	60	0.93	8063
FABP6	1.22	0.000561791	0.13	67	2.22	7048	0.01	84	0.23	8308	0.14	64	1.94	7488
PRDM15	1.04	0.001419045	0.04	101	2.21	7049	-0.13	95	-5.7	12032	0.13	88	4.84	6008
EFCAB4A	1.12	0.000650058	0.06	90	2.21	7050	0.04	113	2.25	6989	0	120	-0.26	8773
FKBP11	1.35	9.28E-06	0.07	81	2.21	7051	0.08	77	2.14	7047	0.06	63	0.79	8137
DNAJB8	1.02	0.104962601	0.08	76	2.21	7052	-0.02	80	-0.69	8931	0.16	84	5.58	5634
PI4KA	1.08	0.075626509	0.03	122	2.2	7053	0.14	120	10.1	2825	-0.01	143	-0.66	9015
TAS2R3	1.03	0.001220287	0.04	100	2.2	7054	-0.05	103	-2.79	10332	0.04	91	1.58	7701
CALML5	1.02	0.351483713	0.05	96	2.2	7055	0.07	114	4.44	5642	0	128	-0.17	8714
STAG2	1.04	0.527768557	0.03	123	2.2	7056	0.05	131	3.81	6001	0.17	134	14.36	2500
CCNA1	-1.02	0.02722614	0.04	106	2.2	7057	-0.04	114	-2.84	10363	0	106	0.14	8528
NOB1_t1	-1.04	0.423736526	0.02	147	2.2	7058	NA	NA	NA	NA	-0.06	109	-3.8	10535
MSANTD4	1.05	0.298621916	0.03	136	2.2	7059	0.12	134	9.7	2974	0.06	147	5.72	5571
RAPGEF3	1.01	0.465812622	0.03	125	2.2	7060	0.03	128	2.29	6966	0.01	126	0.41	8367
MSLN	1.04	0.000827302	0.07	80	2.2	7061	-0.02	120	-1.46	9467	0	98	0.17	8504
PLA2G2F	1.02	0.036507718	0.04	107	2.2	7062	0.13	128	9.76	2946	0.02	110	1.46	7776
MT3	1.21	0.000120948	0.06	84	2.2	7063	0.08	92	3.53	6159	0.13	95	5.79	5542
PROSAPIP1	1.14	3.31E-05	0.03	113	2.2	7064	0.03	123	1.91	7207	0.1	120	6.73	5140
GMPPB	1.09	0.033591898	0.03	123	2.2	7065	0.06	120	3.99	5906	0.01	100	0.37	8395
EPM2AIP1	1.02	0.767806942	0.02	159	2.2	7066	-0.02	135	-1.83	9714	0.05	152	5.22	5824
IER5	-1.06	0.366479098	0.03	121	2.19	7067	0.04	120	2.99	6501	-0.1	115	-6.37	11600
TMCC3	1.04	0.303793279	0.03	120	2.19	7068	0.06	146	6.12	4719	0.01	144	0.62	8245
LIN7C	1.02	0.349051379	0.02	142	2.19	7069	0.03	146	2.84	6591	0.11	141	9.87	3924
HLA-DQA2_t1	-1.71	1.95E-08	0.04	104	2.19	7070	NA	NA	NA	NA	NA	NA	NA	NA
RAPGEFL1	1.12	4.23E-05	0.03	116	2.19	7071	0.08	150	8.38	3555	0.07	127	5.75	5556
MGAT5	-1	0.631333662	0.03	136	2.19	7072	0.02	148	1.79	7283	0.05	133	4.01	6412
ZNF17	1.03	0.312080099	0.03	114	2.19	7073	-0.01	130	-0.73	8952	0.03	119	2.38	7246
SRSF7_t1	-1.06	0.054138661	0.02	141	2.19	7074	NA	NA	NA	NA	-0.04	163	-4.12	10686
NMBR	1.01	0.480540328	0.05	94	2.19	7075	-0.02	92	-0.8	9017	0.03	99	1.64	7656
RXFP3	1.02	0.207600673	0.03	122	2.19	7076	-0.1	142	-9.06	13535	0	100	0.2	8495
POM121	1.15	0.001098106	0.02	145	2.19	7077	0.13	127	10.29	2763	0.14	125	10.41	3718
CNTN5	1.01	0.261190623	0.02	140	2.18	7078	0.03	144	3.16	6405	-0.07	123	-4.98	11043
COL5A3	1.19	0.001953623	0.04	112	2.18	7079	-0.14	120	-9.58	13728	0.2	114	12.92	2915
FAM186B	1.03	0.011311042	0.05	97	2.18	7080	-0.02	104	-1.35	9395	0.05	88	1.94	7484
GPX2	1.16	0.249702875	0.04	104	2.17	7081	0.04	119	2.73	6660	0.03	105	1.44	7787
NKAIN2_t1	1.05	3.21E-05	0.02	138	2.17	7082	NA	NA	NA	NA	0.2	159	21.84	999
SNX19	1.03	0.029558338	0.02	140	2.17	7083	0.06	137	5.5	5061	-0.04	136	-3.61	10439
CYBASC3	1.17	1.47E-06	0.03	122	2.17	7084	-0.04	127	-3.17	10585	0.02	123	1.58	7702
SPR	1.15	0.000483373	0.05	96	2.17	7085	0	115	-0.27	8649	0.15	116	9.99	3882
TELO2	1.02	0.469987441	0.04	110	2.17	7086	0.12	106	6.69	4405	-0.05	100	-2.37	9881
MRPS33	1.19	6.02E-05	0.03	125	2.16	7087	0.14	116	8.97	3288	0.18	107	10.08	3843
PLS3	-1.11	0.17113756	0.03	120	2.16	7088	-0.03	124	-2.3	10023	0.16	131	12.69	2979
BAI2	1.04	0.021277793	0.05	94	2.16	7089	0.03	170	3.15	6410	0.08	111	4.59	6123
KATNAL2	1.07	0.126082125	0.03	134	2.16	7090	-0.01	126	-0.95	9114	0.14	122	10.31	3754
FOXH1	1.01	0.709480018	0.05	93	2.16	7091	-0.06	126	-4.41	11333	-0.11	102	-5.48	11233
OFD1	1.07	0.214584249	0.05	94	2.16	7092	0.05	104	2.85	6585	0.1	102	5.22	5822
RGPD2	-1.02	0.293569742	0.03	120	2.16	7093	0.12	108	7	4247	0.06	99	2.93	6971
SKAP2	-1.03	0.590296702	0.02	142	2.15	7094	-0.01	131	-0.98	9136	0.16	139	13.82	2643
HIST1H4J	1.27	0.000610781	0.22	60	2.15	7095	0.05	80	1.6	7431	0.09	70	1.75	7598
PGLYRP3	1.06	7.63E-07	0.06	84	2.15	7096	-0.05	120	-3.54	10831	0.03	107	1.87	7522
GPR112	1.02	0.091811545	0.03	123	2.15	7097	-0.09	105	-4.75	11514	-0.01	114	-0.78	9073
CLEC16A	1.06	0.10237231	0.03	119	2.15	7098	0.1	129	8.07	3707	0.07	139	6.03	5439
GNAS_t2	1.02	0.239119665	0.03	123	2.15	7099	0.13	124	9.38	3114	-0.11	113	-6.67	11721
FAM40A	1.07	0.01198063	0.03	116	2.15	7100	0.05	113	2.94	6527	-0.02	118	-1.4	9393
CDH6_t1	1	0.890498422	0.02	146	2.14	7101	NA	NA	NA	NA	-0.1	131	-7.97	12227
PLA2G4F	1.03	0.069690503	0.04	111	2.14	7102	-0.12	135	-9.97	13871	0.14	132	11.14	3459

PTCD3	1.12	0.016873231	0.02	144	2.14	7103	0.15	135	12.67	1937	0.09	138	8.27	4504
ATP4A	1.04	0.016680254	0.04	104	2.14	7104	-0.06	120	-4.32	11272	0.03	98	1.6	7685
DSC3_t1	-1	0.955400399	0.03	129	2.14	7105	0.05	126	4	5903	NA	NA	NA	NA
RALB_t1	-1.06	0.000431421	0.03	114	2.14	7106	0.03	129	2.07	7102	NA	NA	NA	NA
KRT14	1	0.925688003	0.04	106	2.14	7107	-0.01	108	-0.42	8760	0.01	111	0.42	8355
UGT8	1.08	8.07E-05	0.03	118	2.13	7108	-0.02	127	-1.52	9507	0.07	120	4.66	6094
ADO	-1.06	0.072501103	0.03	126	2.13	7109	0.08	150	7.6	3930	-0.01	128	-0.47	8894
DNPEP	1.07	0.039111039	0.02	166	2.13	7110	-0.05	147	-4.54	11405	-0.04	122	-2.74	10056
RGS12_t2	1.2	2.91E-05	0.04	104	2.13	7111	-0.08	114	-4.89	11593	0.11	103	5.58	5630
ZNF830	1.07	0.025506741	0.03	121	2.13	7112	-0.02	125	-1.14	9245	0.02	125	1.18	7930
RAB11FIP5	-1.05	0.359793804	0.03	131	2.13	7113	0.11	120	8.01	3740	-0.03	120	-1.98	9702
CIDECP	1.14	0.00023414	0.04	106	2.12	7114	0.12	126	9.19	3196	0.11	130	8.83	4288
SURF4_t1	1.05	0.198351779	0.03	129	2.12	7115	0.1	123	7.48	4000	NA	NA	NA	NA
TEX34	1.07	1.65E-05	0.06	85	2.12	7116	0.08	86	2.79	6619	0.14	68	2.58	7139
RSPH6A	1.02	0.252783923	0.04	106	2.12	7117	-0.01	111	-0.68	8921	-0.04	114	-2.43	9905
RPS19BP1	1.12	0.003862986	0.04	110	2.12	7118	0.11	104	5.87	4859	0.07	103	3.62	6604
MICA	1.24	1.03E-08	0.05	96	2.12	7119	-0.08	100	-4.06	11130	0.11	107	6.12	5388
ZNF133	1.17	1.22E-06	0.04	108	2.12	7120	0.05	118	3.29	6329	0.13	116	8.77	4315
PABPC4	-1.07	0.065635105	0.03	133	2.12	7121	-0.03	111	-1.79	9684	-0.03	113	-2.06	9739
SLIRP	1.04	0.315810278	0.08	76	2.12	7122	-0.02	71	-0.39	8743	0.14	80	4.16	6348
BRD3	1.1	0.015645843	0.03	125	2.12	7123	0.09	124	6.92	4289	-0.09	120	-6.02	11446
CHERP	1.15	0.002797922	0.02	139	2.11	7124	0.1	133	8.15	3666	0.03	158	3.55	6642
PIGP	1.14	0.001211184	0.04	104	2.11	7125	-0.04	130	-3.34	10704	0.12	140	10.91	3541
PLGRKT	1.15	0.000400134	0.03	120	2.11	7126	0.05	119	3.2	6386	0.1	104	5.56	5648
ZNF556	1.12	0.001391802	0.05	94	2.11	7127	0.07	97	3.21	6370	0.11	100	5.68	5590
ATXN7L2	1.1	9.25E-06	0.03	111	2.11	7128	-0.04	91	-1.82	9705	0.09	111	5.56	5644
SEZ6L2_t1	1.63	1.58E-07	0.04	103	2.11	7129	0.07	103	3.54	6155	NA	NA	NA	NA
CKB	1.26	0.016691431	0.04	101	2.1	7130	0.08	103	4.06	5874	-0.02	105	-0.88	9131
CSNK1G3_t1	1.03	0.491813777	0.02	135	2.1	7131	0.07	135	5.94	4818	NA	NA	NA	NA
ATCAY_t1	1	0.930293947	0.03	133	2.1	7132	-0.02	140	-1.97	9795	NA	NA	NA	NA
AHSA1	1.09	0.027402968	0.04	104	2.1	7133	0.02	128	1.82	7264	0.08	113	4.81	6024
LCN1	1	0.983881588	0.06	84	2.1	7134	-0.04	111	-2.34	10048	0.02	100	1.13	7951
ELF4_t1	-1.02	0.356714662	0.02	144	2.1	7135	NA	NA	NA		0	117	0.33	8420
RASA2	-1.05	0.00562859	0.05	94	2.1	7136	0.09	68	1.61	7422	-0.05	82	-1.64	9519
YWHAE	-1.04	0.124785513	0.03	131	2.09	7137	-0.12	126	-8.99	13502	-0.06	131	-5	11050
CASP8_t1	1.04	0.006788446	0.04	104	2.09	7138	0.22	115	13.99	1532	0	108	0.24	8472
CD28	1.09	0.117338342	0.02	139	2.09	7139	0.07	147	7.17	4157	0.1	148	9.53	4045
UBE2E2	-1.13	0.002713707	0.02	144	2.09	7140	0.03	119	1.94	7181	0.05	129	3.92	6460
SLC22A16	1.07	0.00297667	0.04	100	2.09	7141	0.09	89	3.69	6067	0.07	127	5.5	5680
CER1	1.01	0.150730838	0.03	120	2.09	7142	0.05	115	2.99	6502	0.04	110	2.36	7256
ZC3H7A_t1	-1.01	0.712667344	0.03	121	2.08	7143	NA	NA	NA		0.01	137	1.04	7998
SLC43A2	1.03	0.014012959	0.03	112	2.08	7144	-0.01	142	-0.87	9061	0	140	-0.14	8692
PTHLH	1.11	0.000298396	0.04	108	2.08	7145	-0.01	120	-0.85	9047	0.04	130	3.25	6806
FGF4	1.04	0.000538068	0.03	111	2.08	7146	-0.07	116	-4.48	11372	0.02	120	1.74	7603
PNKP	1.06	0.004869279	0.03	113	2.08	7147	-0.04	88	-1.58	9542	-0.01	74	-0.24	8762
ZNF155	1.01	0.710084543	0.03	126	2.07	7148	0.05	111	3.3	6319	-0.09	123	-6.39	11608
CTSZ	-1.1	0.20408848	0.03	123	2.07	7149	-0.05	114	-3.51	10809	-0.06	144	-6.08	11469
SPEM1	1.01	0.139999044	0.12	68	2.07	7150	NA	NA	NA	NA	NA	NA	NA	NA
ESRRG	-1	0.966047302	0.03	129	2.07	7151	-0.03	123	-2.13	9921	0.02	143	2.12	7386
LCN6	1.02	0.155670469	0.06	83	2.07	7152	0.01	100	0.6	8067	0.06	95	2.92	6979
IQCA1	1.11	0.000571101	0.03	128	2.07	7153	0.16	138	14.47	1409	0.17	126	13.06	2868
GGT7	1.01	0.780942608	0.03	122	2.07	7154	-0.06	120	-4.22	11222	0.02	111	1.22	7898
HIST1H2BA	1.01	0.229120352	0.06	84	2.07	7155	0.1	88	3.72	6047	0.1	80	3.09	6873
HIST1H4K	1.2	0.004950724	0.21	60	2.06	7156	0.03	64	0.45	8165	0.07	68	1.3	7854
SLC5A11	1.35	2.99E-05	0.07	80	2.06	7157	0.1	107	5.88	4854	0.15	79	4.27	6304
CDH11	1.1	0.444772057	0.03	120	2.06	7158	0.11	128	8.67	3432	0.01	151	1.46	7774
SLC18A3	1.02	0.036636743	0.04	104	2.06	7159	-0.09	120	-5.97	12170	0.02	111	1.09	7972
ACN9	1.11	0.081688009	0.03	118	2.06	7160	0.03	122	2.12	7063	0.24	124	17.65	1710
MIDN	-1	0.775678586	0.02	138	2.06	7161	0	120	-0.06	8527	-0.08	137	-7.25	11944
PSMA8	1.01	0.432458503	0.02	133	2.06	7162	0.02	120	1.45	7520	0.04	138	3.32	6765
LIN7A	-1.11	0.042496159	0.02	140	2.06	7163	-0.02	111	-1	9146	-0.01	110	-0.69	9026
MED14	1.02	0.241156046	0.02	141	2.05	7164	0.03	140	2.87	6573	0.11	133	9.54	4044
MAP6D1	1.11	0.007283315	0.03	121	2.04	7165	0.05	123	3.5	6182	0.01	125	0.59	8262
MYC	1.02	0.032038254	0.03	112	2.04	7166	0.07	96	3.21	6376	0.02	126	1.36	7828
ST8SIA5	1.12	3.35E-08	0.03	125	2.04	7167	0.04	128	2.92	6541	0.11	141	10.25	3779
MTMR9	-1.07	0.000144896	0.02	138	2.04	7168	-0.03	156	-2.71	10281	-0.06	157	-6.08	11467
TANK_t1	-1.01	0.886121627	0.04	101	2.03	7169	0.03	92	1.45	7526	0.02	108	1.22	7902
KAT6A	1.03	0.177448326	0.02	139	2.03	7170	0.02	125	1.23	7663	0.06	133	4.81	6023
RPTOR	1.2	4.81E-09	0.03	112	2.03	7171	0.11	128	8.34	3578	0.13	112	7.83	4700
RIBC2	1.47	2.10E-10	0.07	80	2.03	7172	0.13	108	7.63	3916	0.11	76	2.96	6949
ALDH16A1	-1.05	0.307408303	0.04	104	2.03	7173	-0.05	114	-3.1	10544	-0.03	113	-1.58	9490
ENTHD1	1.03	0.003473924	0.03	115	2.03	7174	0.13	127	10.03	2848	0.08	114	4.89	5983
PTRHD1	-1.02	0.531220534	0.03	124	2.03	7175	-0.07	104	-3.89	11035	-0.05	111	-3.33	10323
RILPL1	1.09	0.00235404	0.03	125	2.03	7176	0.19	131	15.38	1173	-0.01	124	-0.81	9091
SOD1	-1.04	0.643744827	0.03	116	2.02	7177	-0.04	124	-3.26	10639	0.01	115	0.4	8372
PPWD1	1.01	0.886247116	0.03	129	2.02	7178	0.12	105	6.65	4420	0.08	115	5.04	5905
ESR2	1.02	0.006773103	0.05	88	2.02	7179	-0.03	100	-1.45	9460	-0.04	88	-1.51	9451
SPATS1	1.02	0.032890523	0.03	118	2.02	7180	0.07	122	5.14	5255	0.07	120	4.86	5996
SLC35A4	1.11	1.77E-05	0.02	131	2.02	7181	-0.02	140	-2	9820	0.02	114	1.18	7928
MLLT10_t1	-1.05	0.311808732	0.02	142	2.02	7182	NA	NA	NA	NA	NA	NA	NA	NA
GH2	1.03	0.002520529	0.08	75	2.02	7183	0.05	71	1.03	7789	0.08	116	5.12	5868
SERPINB2	-1	0.851350409	0.03	112	2.02	7184	-0.08	106	-4.75	11515	-0.03	113	-1.59	9494
PODNL1	1.05	3.43E-06	0.03	112	2.02	7185	0.1	151	10.58	2639	0.09	108	5.49	5691
NT5C1B_t1	-1.02	0.165875831	0.03	124	2.02	7186	-0.07	135	-5.58	11961	NA	NA	NA	NA
LCE1E	1.02	0.052722041	0.03	127	2.01	7187	0.09	130	7.11	4185	0.02	128	1.43	7792
BPIFB3	1.04	4.36E-05	0.09	72	2.01	7188	0.05	68	0.94	7839	0.11	68	2.03	7431
DOM3Z	1.19	1.24E-07	0.05	94	2.01	7189	0.04	100	2.11	7069	0.13	80	3.93	6449
FTSJD2	1.15	3.77E-08	0.04	104	2.01	7190	0	120	0.34	8246	0.04	136	3.17	6848
CCDC71	1.01	0.744440237	0.04	100	2.01	7191	-0.02	105	-1.02	9157	-0.02	120	-1.57	9484.5
CRLF1	1.05	0.643698717	0.03	108	2.01	7192	0.07	115	4.43	5650	-0.06	100	-2.76	10064
S1PR3	1.06	0.000158975	0.03	124	2.01	7193	-0.03	132	-2.69	10266	0.01	145	1.28	7862
OR2C1	1	0.721758142	0.04	104	2.01	7194	-0.08	140	-6.87	12592	0.03	105	1.45	7783
MAD2L1BP	1.09	0.005951187	0.04	103	2.01	7195	-0.05	133	-4.23	11228	0	107	0	8615
PRKAG3	1.01	0.291008677	0.03	112	2.01	7196	0.1	121	7.25	4120	0.06	130	5.17	5841
OR3A2	1.02	0.118341981	0.04	103	2	7197	-0.06	88	-2.24	9992	0.02	88	0.81	8127
MAPK1_t2	-1.03	0.324673173	0.04	99	2	7198	0.1	106	5.52	5052	-0.11	121	-7.9	12195
LGALS4	1.03	0.854660298	0.05	89	2	7199	-0.03	103	-1.68	9607	0.02	84	0.56	8286
CCDC27	1.03	0.003669384	0.08	75	2	7200	0.01	111	0.7	7999	0.17	108	9.71	3980
B4GALT5_t1	-1.13	0.031957718	0.02	139	2	7201	NA	NA	NA		-0.09	140	-7.7	12115
BMP2	1.04	0.659816915	0.02	131	2	7202	0.03	125	2.12	7067	-0.02	120	-1.57	9482
TRIM40	1.02	0.043238633	0.02	144	1.99	7203	0.06	115	3.7	6057	0.16	130	12.93	2914
INSL4	1.03	0.243883487	0.05	92	1.99	7204	0.01	92	0.4	8207	0.02	103	0.91	8072
MTIF3	-1.05	0.305692614	0.05	88	1.99	7205	-0.05	97	-2.55	10180	-0.01	88	-0.38	8843
KCNH5_t2	1.02	0.008343425	0.03	110	1.99	7206	-0.08	111	-5.05	11684	0.08	121	5.69	5586
ZNF347	-1.07	0.009063734	0.02	134	1.99	7207	0.09	129	6.87	4316	0	121	0.33	8423
DPM1	1.11	0.032543643	0.03	107	1.99	7208	0.1	117	6.55	4482	0.14	112	8.65	4366

DNAJC28	1.05	0.103097287	0.04	103	1.99	7209	0.02	121	1.12	7735	0.08	144	7.64	4774
CACNA1G	1.06	0.004053511	0.03	123	1.99	7210	0.01	136	0.87	7890	0.06	115	3.71	6564
COPE_t2	1.24	5.98E-10	0.05	88	1.98	7211	0.1	102	5.37	5140	0.06	92	2.72	7070
PRRC2A_t1	1.06	0.034033854	0.03	114	1.98	7212	NA	NA	NA	NA	0.07	90	2.74	7059
ADCK4	-1.01	0.744272553	0.03	115	1.98	7213	-0.11	106	-5.95	12154	-0.1	101	-5.21	11125
TAS2R7	1.03	0.007988098	0.04	101	1.98	7214	0.04	95	1.95	7175	-0.04	129	-3.34	10327.5
PNLIP	1.01	0.451531146	0.02	133	1.98	7215	-0.04	84	-1.47	9476	0.09	88	3.3	6777
MYOM1_t1	-1.11	0.185735677	0.03	120	1.97	7216	NA	NA	NA	NA	0.02	108	1.31	7849
LIX1_t1	1.03	0.011743359	0.02	141	1.97	7217	NA	NA	NA	NA	0.06	152	5.87	5508
CREBBP_t1	-1.02	0.712783096	0.02	133	1.97	7218	-0.08	135	-7.19	12732	0.09	147	8.41	4464
KIT	-1.03	0.548289975	0.02	143	1.97	7219	0.13	124	9.75	2958	-0.04	147	-3.59	10432
SLC35B2	1.14	7.30E-06	0.03	118	1.97	7220	0.05	123	3.86	5968	0.09	127	6.8	5105
VPS33B	1.19	4.51E-12	0.03	123	1.97	7221	0.1	134	8.37	3558	0.17	108	9.63	4015
KRT12	1.17	0.002536299	0.02	137	1.96	7222	0.18	105	10.1	2826	0.09	111	5.32	5767
SLC30A2_t1	1.05	0.005222898	0.03	111	1.96	7223	NA	NA	NA	NA	-0.02	120	-1.48	9438
MPLKIP	1.17	0.011347982	0.03	108	1.96	7224	-0.02	135	-1.66	9590	0.06	111	3.85	6496
EXOSC4	1.47	5.56E-15	0.25	58	1.96	7225	0.17	108	9.74	2962	0.27	68	4.94	5958
THY1	1.12	0.008076296	0.03	115	1.96	7226	0.09	127	7.19	4145	0.04	124	2.62	7123
HOXA6	1.01	0.594414998	0.07	80	1.96	7227	-0.07	92	-2.78	10327	0	116	0.08	8567
TIA1_t2	1.08	0.087593695	0.02	134	1.96	7228	0.18	124	13.01	1828	0.1	138	8.57	4400
TMUB1	1.03	0.411349015	0.04	103	1.96	7229	-0.02	109	-1.06	9190	0.01	107	0.69	8200
GK2	-1.01	0.109179859	0.03	108	1.96	7230	-0.08	122	-5.5	11911	0.01	100	0.48	8329
LPHN1	1.05	0.503555629	0.03	124	1.96	7231	0.07	130	5.47	5072	0.07	132	6.05	5428
SPATA5L1	1.22	1.84E-06	0.03	119	1.96	7232	0.14	120	9.85	2916	0.18	118	12	3195
CT47A11	1.55	9.65E-06	0.03	122	1.95	7233	0.07	104	3.54	6157	0.06	102	3.29	6778
RPL13_t1	1.01	0.866589816	0.02	131	1.95	7234	0.11	131	9	3272	NA	NA	NA	NA
CATSPER3	1.07	0.010553688	0.07	80	1.95	7235	-0.05	88	-1.98	9808	0.18	110	10.73	3602
QARS	1.09	0.015152983	0.05	87	1.95	7236	-0.06	105	-3.18	10592	0.06	100	2.97	6942
KPNA5	1.03	0.031287734	0.03	113	1.95	7237	0.23	111	14.33	1442	-0.02	122	-1.25	9315
RHPN2	-1.05	0.137224095	0.03	124	1.95	7238	-0.09	126	-6.5	12413	-0.05	115	-3.23	10282
NMT2	1.16	0.002490171	0.03	123	1.94	7239	-0.03	142	-2.8	10341	0.16	136	13.36	2776
ZFYVE1	1.01	0.879356908	0.03	114	1.94	7240	-0.13	110	-7.93	13085	-0.01	130	-0.47	8892
INPP5E	1.08	0.063563642	0.04	104	1.94	7241	0.13	116	8.63	3445	0.05	113	3.08	6893
FOXP4	1.04	0.000367291	0.03	115	1.94	7242	0.04	153	4.54	5596	0.07	122	4.92	5966
RBMXL2	-1.01	0.547589019	0.03	119	1.94	7243	0.01	123	0.45	8175	-0.05	112	-3.13	10238
MMP9	1.39	0.002934692	0.04	104	1.94	7244	0.1	86	3.53	6158	-0.02	107	-0.88	9129
MAN2A1_t1	-1.07	0.225540232	0.02	162	1.94	7245	NA	NA	NA	NA	0.16	111	9.66	3998
DEFB127	1.02	0.020707073	0.02	128	1.94	7246	0.08	106	4.49	5614	0.05	113	3.21	6824
CERCAM	1.03	0.047114469	0.04	104	1.93	7247	0.04	120	3.04	6476	-0.06	145	-6.09	11477
STAT1_t2	1.02	0.76866018	0.03	119	1.93	7248	0	98	-0.01	8488	-0.07	123	-4.97	11040
TSPEAR	1.05	0.000304723	0.03	116	1.93	7249	0.08	124	6.01	4776	0.11	123	7.71	4752
PPP1R2P9	-1.01	0.602072795	0.03	112	1.93	7250	0.02	120	1.32	7593	-0.01	123	-0.63	8996
CUL3	-1.07	0.002077298	0.02	140	1.92	7251	-0.03	135	-2.57	10197	0	155	-0.01	8624
SFTPA1_t1	1.01	0.341735305	0.03	121	1.92	7252	0.07	153	7.41	4031	NA	NA	NA	NA
JPH2_t1	1.05	3.75E-06	0.02	137	1.92	7253	-0.07	134	-6.18	12276	0.01	133	0.88	8089
BRAF	-1.01	0.675349242	0.03	125	1.91	7254	-0.04	111	-2.23	9985	0	122	0.14	8533
MTMR14_t1	1.03	0.343107506	0.03	115	1.91	7255	0.01	107	0.83	7917	-0.07	120	-4.82	10992
TPM1_t3	1.22	0.001735305	0.04	98	1.91	7256	0.06	113	3.94	5935	0.14	117	9.51	4052
GTSF1L	1.02	0.016841447	0.07	78	1.91	7257	0.06	113	3.52	6170	0.03	88	1.22	7899
CEL	-1	0.886121627	0.04	100	1.91	7258	-0.18	102	-9.12	13566	0.01	103	0.39	8378
DCUN1D2_t1	1.03	0.023154492	0.02	135	1.9	7259	NA	NA	NA	NA	0.08	131	6.11	5398
KNDC1_t1	1.04	0.186515905	0.04	102	1.9	7260	NA	NA	NA	NA	0.07	112	4.37	6261
DMBT1	1.19	0.00157809	0.03	116	1.9	7261	0.06	128	4.34	5695	0.09	127	6.78	5115
GUCY2C	1.32	1.73E-06	0.03	120	1.9	7262	0.26	104	13.81	1595	0.12	120	8.44	4452
ZNF501_t1	1.1	2.33E-07	0.02	126	1.89	7263	NA	NA	NA	NA	0.18	127	13.68	2683
PARM1_t1	1.03	0.605033563	0.02	138	1.89	7264	0.09	145	8.51	3492	NA	NA	NA	NA
NUP98_t1	1.04	0.171667175	0.03	119	1.89	7265	-0.05	132	-3.88	11027	0.07	132	6	5449
SERPINI2	1.01	0.477136269	0.03	120	1.89	7266	-0.03	117	-2.22	9979	0.02	123	1.56	7713
TMED4	1.03	0.381300177	0.03	122	1.89	7267	-0.01	113	-0.5	8817	-0.04	112	-2.66	10021
MYT1	1.07	1.01E-06	0.02	126	1.88	7268	-0.04	138	-3.74	10952	0.03	148	2.79	7036
GYPE	1.06	3.52E-08	0.03	118	1.88	7269	-0.03	100	-1.59	9549	0.09	100	4.25	6314
DRD4	1.01	0.304776175	0.05	91	1.88	7270	-0.12	101	-6.05	12214	-0.03	104	-1.45	9419
ADAM22_t2	1.14	2.58E-05	0.02	126	1.88	7271	0.07	135	6.02	4767	0.01	142	1.05	7991
ANKHD1-EIF4E	1.06	0.185405518	0.03	104	1.88	7272	-0.06	120	-3.86	11010	0.07	102	3.44	6696
SNX25	-1.03	0.31640656	0.03	118	1.87	7273	0.05	113	3	6499	0.02	129	1.5	7747
ELAVL3_t1	1	0.814797101	0.03	124	1.87	7274	-0.06	148	-5.55	11939	0.08	137	6.55	5211
FAM117A	-1.06	0.401314424	0.03	122	1.87	7275	-0.07	114	-4.39	11311	-0.06	115	-3.65	10458
RHBDF1	1.01	0.83933296	0.02	129	1.87	7276	0.06	107	3.41	6239	0.01	103	0.33	8421
ZNF496	1.01	0.427568564	0.04	98	1.87	7277	-0.08	128	-6.37	12362	-0.06	127	-4.68	10928
ZMIZ2	1.38	2.50E-06	0.03	123	1.87	7278	0.23	120	16.41	958	0.08	118	5.41	5729
TRPM3_t1	1.06	5.72E-06	0.03	115	1.87	7279	-0.08	130	-6.08	12236	0.14	142	12.73	2965
LRRC2	1.06	0.10847314	0.02	151	1.87	7280	-0.13	142	-11.99	14497	0.17	152	17.58	1719
KRTAP3-1	1.05	1.95E-05	0.02	126	1.87	7281	-0.06	116	-4.14	11171	0.01	137	0.57	8282
USP48_t2	-1.02	0.485071649	0.02	140	1.87	7282	0.11	131	8.65	3437	0.03	139	2.31	7280
TBL2	1.21	4.07E-08	0.02	128	1.86	7283	0.18	159	19.84	418	0.12	129	9.26	4132
SCN5A_t1	1.02	0.07553165	0.02	151	1.86	7284	-0.07	118	-4.67	11473	0.05	112	3.08	6880
TBC1D21	1.01	0.664329796	0.05	91	1.86	7285	-0.15	115	-9.9	13843	-0.05	100	-2.56	9986
IL1F10	1.02	0.21968782	0.03	118	1.86	7286	-0.1	125	-7.87	13061	-0.01	118	-0.39	8848
WASF3	-1.49	7.15E-07	0.02	128	1.86	7287	0.05	132	3.73	6045	-0.09	145	-8.82	12533
ABHD16B	-1.02	0.480006543	0.03	120	1.86	7288	-0.14	100	-7.15	12717	0.02	110	0.93	8058
COL22A1	1.11	0.00085123	0.03	121	1.86	7289	0.1	133	8.55	3473	0.13	120	8.86	4273
KCNH2_t1	1.1	0.075236927	0.04	97	1.86	7290	0.01	118	0.58	8084	-0.1	88	-3.77	10523
GBF1	1.09	0.0003603	0.03	121	1.85	7291	0.01	130	0.49	8141	0.06	120	4.02	6406
RERE_t1	1	0.899562247	0.02	127	1.85	7292	0.05	166	6.08	4733	NA	NA	NA	NA
CPNE9	1.09	0.000779907	0.03	104	1.85	7293	0.01	113	0.92	7857	0.1	112	6.41	5273
CEND1	1.04	0.002052433	0.04	92	1.85	7294	0	129	-0.17	8592	0.11	138	9.45	4068
METTL18	1.43	2.15E-12	0.18	60	1.85	7295	0.21	106	11.73	2223	0.28	92	11.74	3286
HDAC3	1.01	0.712598046	0.04	102	1.85	7296	-0.06	98	-3.08	10523	-0.05	104	-2.73	10051
TPH1	-1.02	0.11775568	0.02	132	1.84	7297	-0.12	116	-8.1	13149	0.05	125	4.04	6398
AGT	1.18	0.087419558	0.03	118	1.84	7298	0.04	107	2.48	6837	0.11	124	7.88	4673
CSRP3	1.05	6.30E-05	0.03	119	1.84	7299	0.02	111	1.48	7503	0.12	120	8.44	4446
SNCAIP	1.07	0.000244095	0.03	115	1.84	7300	0.12	126	8.93	3312	-0.07	119	-4.54	10861
CHGB_t1	1.07	0.013590549	0.02	126	1.84	7301	0.1	109	6.15	4701	NA	NA	NA	NA
NEIL2	1.04	0.025626772	0.02	124	1.84	7302	0.03	120	1.79	7287	0.11	145	10.52	3682
RNF25	1.05	0.027403897	0.03	109	1.83	7303	0.04	76	0.98	7819	0.01	92	0.39	8381
MS4A14	-1.06	0.132746442	0.03	115	1.83	7304	0.11	126	8.03	3729	-0.04	113	-2.79	10085
LRTM2	1.01	0.25619333	0.03	112	1.83	7305	-0.01	134	-0.9	9080	0.09	120	6.55	5215
TLE6	1.14	6.50E-05	0.07	76	1.83	7306	-0.01	126	-0.53	8836	0.08	118	5.55	5654
CLDN7	-1.12	0.17052926	0.03	113	1.83	7307	-0.04	135	-3.09	10534	-0.11	120	-7.82	12173
TPTE_t2	1.1	0.000743811	0.02	135	1.83	7308	0.09	99	4.27	5738	0.08	86	2.73	7067
RPS15_t1	1.05	0.197649786	0.05	88	1.82	7309	NA	NA	NA	NA	NA	NA	NA	NA
UBR1	1.02	0.522510971	0.02	146	1.82	7310	0.11	122	7.69	3883	0.03	143	2.75	7055
MOB3B_t1	1.15	2.35E-06	0.02	130	1.82	7311	0.07	147	7.24	4123	NA	NA	NA	NA
TIMM22	1.05	0.037773206	0.03	122	1.82	7312	0	141	-0.09	8550	-0.05	113	-2.88	10125
SSR4	-1.06	0.289809715	0.03	108	1.81	7313	-0.03	92	-1.46	9468	-0.12	100	-5.96	11426
LENG1	-1.01	0.643698717	0.04	100	1.81	7314	-0.1	84	-3.46	10774	-0.02	73	-0.38	8838

CRCT1	1.17	1.09E-06	0.03	104	1.81	7315	0.02	111	0.94	7845	0.05	154	5.34	5762
KRTAP2-4_t1	-1.03	0.385704584	0.05	88	1.81	7316	NA	NA	NA	NA	0.03	110	1.66	7645
MOGS	1.06	0.018574165	0.04	100	1.81	7317	0.09	120	6.55	4479	0.03	115	1.67	7638
TYRO3	1.03	0.662939983	0.03	110	1.81	7318	0.07	136	5.71	4949	-0.08	104	-4.34	10771
ZSCAN1	1.05	0.000297807	0.03	107	1.81	7319	-0.14	105	-7.64	12950	-0.01	125	-0.96	9181
BDNF	1.01	0.241101284	0.02	136	1.8	7320	0.03	160	3.62	6104	0.11	146	10.48	3699
CLDN3	-1.09	0.002419708	0.02	146	1.8	7321	-0.08	102	-4.42	11336	-0.09	113	-5.59	11272
BBS7_t2	1.02	0.194395645	0.02	150	1.8	7322	0	126	0.26	8289	0.11	126	8.13	4570
ARHGDIG	1.05	4.04E-06	0.06	78	1.8	7323	-0.03	109	-1.62	9565	0.01	127	0.88	8092
GRSF1	-1	0.968133986	0.01	170	1.8	7324	0.07	149	6.47	4533	0.09	152	9.61	4022
LONP1	1.13	0.002709041	0.04	93	1.8	7325	0.06	88	2.34	6927	0.09	113	5.93	5478
IMP4	1.13	6.75E-05	0.03	104	1.8	7326	0.01	125	0.72	7987	0.09	88	3.31	6771
ZNF827	-1.01	0.565805477	0.03	104	1.79	7327	0.12	142	10.85	2536	-0.11	126	-8.33	12371
CST11	1.05	1.58E-06	0.04	90	1.79	7328	-0.08	102	-4.35	11290	0.05	88	1.81	7560
AMH	1.05	0.147260703	0.05	89	1.78	7329	-0.03	100	-1.38	9416	0	84	0	8619
BPIFC	1	0.985820996	0.03	112	1.78	7330	-0.12	110	-7.31	12793	-0.05	114	-3.47	10381
KBTBD12	1.08	0.000648082	0.02	131	1.78	7331	0.15	126	11.2	2396	0.02	147	1.62	7675
ABCA3	1.11	0.065642028	0.03	101	1.78	7332	0.06	126	4.49	5617	-0.09	113	-5.68	11310
TBL3	-1.02	0.448215106	0.07	76	1.77	7333	-0.12	106	-6.53	12424	0.03	68	0.52	8303
PPAN	1.07	0.054155364	0.05	86	1.77	7334	0.04	120	2.66	6709	-0.01	110	-0.48	8900
MYOM3	1.03	0.000515887	0.02	121	1.76	7335	0.04	138	3.83	5988	0.1	120	6.85	5087
TOMM70A	-1.04	0.345473849	0.02	127	1.76	7336	-0.06	136	-5.35	11847	0	131	-0.17	8718
GGN	1.09	2.64E-08	0.18	60	1.76	7337	0.08	113	4.74	5480	0.17	84	5.64	5605
PNISR	-1.07	0.153309033	0.02	149	1.76	7338	0.1	101	5.06	5302	0	127	-0.09	8670
GRINA	1.11	0.019246972	0.03	109	1.76	7339	-0.08	120	-5.59	11969	0.1	116	6.36	5291
OLAH	1.01	0.751587729	0.02	130	1.75	7340	0.02	127	1.86	7241	0.05	120	3.31	6767
CGA	1.12	0.045691331	0.04	100	1.75	7341	-0.08	138	-6.96	12629	0.05	124	3.95	6435
ISL2	1.04	0.00185699	0.03	106	1.75	7342	0.04	122	2.77	6625	0.02	110	0.93	8056
NR3C1	1.01	0.790345549	0.02	126	1.75	7343	-0.08	163	-9.07	13543	0.13	143	11.81	3259
MYCBPAP	1.09	0.00168599	0.05	85	1.75	7344	0.18	78	5.11	5271	-0.01	105	-0.59	8982
TAF8	1.05	0.001460946	0.02	133	1.74	7345	-0.01	127	-1.11	9228	0.03	126	2.51	7174
RAD51B_t2	1.01	0.156615985	0.02	142	1.74	7346	0.04	127	3.29	6324	-0.02	110	-1.37	9376
CACNA1S	1.03	0.000617713	0.05	86	1.74	7347	-0.05	120	-3.52	10815	0.03	85	1.19	7920
SPINK7	-1.06	0.00238938	0.03	100	1.74	7348	-0.08	103	-4	11098	-0.05	106	-2.92	10145
TAS2R16	1.05	2.36E-08	0.03	106	1.74	7349	-0.08	109	-4.59	11436	0.1	86	3.59	6618
PFN2_t1	1.05	0.011046579	0.02	129	1.74	7350	0.13	129	9.95	2878	NA	NA	NA	NA
CSTF2T	-1	0.943424198	0.02	137	1.74	7351	-0.1	131	-8.22	13201	0.15	137	13.25	2813
FAM125A	1.06	0.009054206	0.05	85	1.74	7352	-0.01	108	-0.8	9014	-0.03	92	-1.21	9301
TWF2	1.06	0.224043609	0.03	109	1.74	7353	0.07	114	4.26	5742	-0.04	102	-2.02	9713
ANKLE1	1.03	0.014411932	0.03	116	1.73	7354	-0.08	166	-9.21	13592	0.06	164	6.37	5284
KCNJ12	1.02	0.089107246	0.02	140	1.73	7355	-0.03	134	-2.64	10246	0.05	128	3.69	6570
PIANP	1.03	9.09E-05	0.02	125	1.73	7356	0.03	154	3.08	6450	0.04	158	4.41	6243
FEM1A	1.03	0.38710852	0.03	112	1.72	7357	-0.08	121	-5.37	11857	-0.01	132	-1.22	9310
RNASEK	-1.03	0.378809596	0.04	96	1.72	7358	-0.04	122	-2.53	10169	-0.03	100	-1.49	9443
ACOT11_t1	1.05	0.000113783	0.06	77	1.72	7359	0.02	52	0.05	8430	0.06	52	0.12	8543
MEPCE_t1	-1.03	0.423053557	0.02	120	1.72	7360	NA	NA	NA	NA	-0.01	119	-0.91	9143
NNAT	1.02	0.020833146	0.02	125	1.72	7361	-0.02	117	-1.26	9332	0.07	130	5.23	5820
SIGMAR1	1.08	0.126752054	0.02	137	1.72	7362	-0.08	112	-4.79	11530	0.12	120	8.18	4545
ZBTB3	1.15	7.29E-05	0.03	108	1.71	7363	-0.01	111	-0.63	8892	0.02	127	1.24	7881
CHRM1	-1.04	0.03422279	0.03	109	1.71	7364	-0.12	125	-8.78	13427	-0.13	112	-7.94	12212
CUEDC2	1.01	0.714887476	0.05	87	1.71	7365	-0.12	104	-6.36	12356	0.01	100	0.49	8324
MNX1_t1	1.03	0.016739924	0.02	130	1.71	7366	NA	NA	NA	NA	0.06	134	5.28	5786
ACTA2	1.01	0.897417631	0.04	96	1.71	7367	0.07	108	4.22	5766	-0.04	95	-1.67	9539
BET1L	1.05	0.097348958	0.03	115	1.7	7368	-0.03	124	-2.37	10063	-0.05	125	-3.73	10502
NOG	1.03	0.003789	0.02	122	1.7	7369	-0.1	120	-6.93	12617	0.06	120	3.94	6445
S1PR5	-1.03	0.019392784	0.03	108	1.7	7370	-0.05	108	-3.17	10584	-0.1	120	-7.34	11970
FOXP1_t1	1.06	0.346864834	0.02	135	1.7	7371	NA	NA	NA	NA	0.08	148	8.12	4576
NR2E1	1.05	0.001415415	0.03	111	1.7	7372	0.08	129	5.94	4815	0.06	136	5.3	5778
GNRHR_t1	-1	0.805300413	0.02	129	1.7	7373	-0.08	133	-6.59	12458	0.05	124	3.57	6631
PSMA3	1	0.932960116	0.04	95	1.69	7374	0.03	104	1.38	7562	0.01	116	0.59	8267
P2RX6	-1	0.772658342	0.03	106	1.69	7375	0.06	114	4.05	5878	-0.04	111	-2.55	9980
CCDC105	1.01	0.128792477	0.05	84	1.69	7376	0	113	0.18	8344	0.17	86	6.3	5321
CGB1	1.03	0.002267363	0.12	64	1.69	7377	0.04	108	2.16	7038	0.03	60	0.28	8453
ATP5G2	1.2	6.71E-11	0.03	114	1.68	7378	0.12	96	5.47	5075	0.14	94	6.05	5430
NDUFS4	1.07	0.115107432	0.02	126	1.68	7379	-0.07	104	-3.57	10853	0.24	109	14.27	2529
NETO1_t2	1.05	0.000251424	0.02	146	1.68	7380	-0.1	141	-8.9	13469	0.08	158	8.45	4443
FBXO31	-1	0.984614146	0.02	132	1.68	7381	-0.08	120	-5.55	11940	-0.05	120	-3.34	10332
TMC1	1.05	1.64E-06	0.02	118	1.68	7382	0.05	114	3.24	6350	0.15	112	9.47	4063
C2CD3	1.04	0.000228798	0.03	108	1.68	7383	0.11	100	5.42	5111	0.12	118	8.16	4555
DIDO1_t2	1.13	0.000785579	0.02	153	1.68	7384	0.1	132	7.95	3769	0.04	142	3.83	6511
TECPR1	1.18	1.66E-06	0.02	129	1.68	7385	0.14	145	13.75	1613	0.09	138	7.6	4796
AURKAIP1	-1	0.917845203	0.04	92	1.67	7386	-0.12	87	-4.45	11361	0	103	-0.08	8668
CDKAL1	1.07	1.72E-07	0.02	139	1.67	7387	0.02	120	1.33	7590	0.14	132	11.73	3290
SGCZ	1.02	0.079746795	0.02	121	1.67	7388	0.11	125	8.27	3610	0.14	114	8.77	4316
LRRC39	1.14	0.001119742	0.02	124	1.67	7389	0.13	113	7.89	3799	0.18	114	11.71	3294
NSG1	1.01	0.220834413	0.02	121	1.67	7390	0.06	119	4.03	5890	-0.03	141	-2.61	10002
MYOF_t1	-1.04	0.675222518	0.02	123	1.67	7391	0.16	116	10.25	2777	-0.07	111	-4.22	10721
KRTAP3-3	1.05	2.09E-10	0.03	108	1.67	7392	0.07	104	4.01	5900	0.04	107	2.24	7323
ANKRD49	1.08	0.032185078	0.02	126	1.67	7393	0.13	136	11.29	2368	0.07	112	4.1	6373
FRYL_t1	-1.05	0.331275967	0.02	125	1.66	7394	NA	NA	NA	NA	0.04	141	3.92	6457
KHDC1_t1	1.11	0.027133539	0.03	100	1.66	7395	0.16	113	9.87	2906.5	NA	NA	NA	NA
ORAI1	-1.05	0.112425059	0.03	100	1.66	7396	0	125	-0.17	8600	-0.05	120	-3.29	10305
ADORA1	1	0.951009764	0.03	112	1.66	7397	-0.13	108	-7.28	12776	-0.14	122	-10.43	13068
CD274	1.01	0.643328482	0.02	145	1.66	7398	-0.03	123	-2.5	10152	-0.04	121	-2.78	10079
DPYSL5	1.04	2.58E-05	0.02	128	1.66	7399	0.06	133	4.96	5364	0.15	141	14.1	2573
ZNF135	1.08	0.139282433	0.02	123	1.66	7400	0.14	139	12.82	1890	0	157	-0.23	8754
MAP4K2	1.12	2.19E-06	0.03	98	1.65	7401	0.17	125	12.73	1918	0.1	104	5.47	5707
DBP	1.19	0.000378725	0.03	102	1.65	7402	-0.09	114	-5.7	12031	0.07	91	2.97	6946
DHRS9	-1.2	0.000223007	0.02	123	1.65	7403	0.04	112	2.6	6756	-0.11	122	-7.81	12169
ELAC2_t1	-1.03	0.500704122	0.03	110	1.65	7404	NA	NA	NA	NA	0.04	118	2.52	7169
NFATC4	1.05	0.012422031	0.03	112	1.65	7405	-0.05	134	-4.3	11264	0.06	143	5.56	5643
POLN	1.03	0.003519019	0.04	92	1.65	7406	0.12	106	6.54	4483	0.06	84	1.9	7509
ABHD2	1.02	0.042826386	0.01	172	1.65	7407	0.07	156	7.9	3791	0	141	0.16	8513
CDH3	1.07	5.77E-06	0.02	122	1.64	7408	0.01	103	0.78	7955	0.06	119	4.35	6266
PALM2-AKAP2	1	0.980655383	0.02	136	1.64	7409	0	123	-0.29	8667	0.02	147	2.18	7355
F2RL3	1.03	0.12074254	0.02	145	1.64	7410	0.03	131	2.04	7120	-0.04	132	-3.38	10344
PRRG1_t1	-1	0.936743666	0.02	139	1.64	7411	NA	NA	NA	NA	NA	NA	NA	NA
HS3ST4_t1	1.04	0.043418141	0.02	130	1.64	7412	0.12	132	9.91	2896	NA	NA	NA	NA
ABHD5	1.02	0.625741186	0.02	145	1.63	7413	-0.15	135	-12.58	14663	0.14	148	13.43	2754
RPN1	1.07	0.062673914	0.02	131	1.63	7414	0.02	116	1.15	7708	0.02	120	1.11	7960
CHMP6	1.04	0.059941295	0.03	115	1.63	7415	-0.06	111	-3.81	10987	-0.03	111	-1.68	9542
MYH6_t1	1.02	0.101341838	0.05	80	1.63	7416	-0.01	104	-0.65	8909	NA	NA	NA	NA
PRRX2	1.07	0.011535649	0.03	103	1.63	7417	0.12	100	5.83	4882	0.1	99	4.86	5999
CORO1B	1	0.987518039	0.05	84	1.63	7418	-0.03	125	-2.21	9969	-0.13	99	-6.29	11565
ENHO	1.08	0.020454771	0.03	110	1.63	7419	-0.13	118	-8.81	13437	0.02	93	1.04	7995
B3GNT3	1.16	0.170270666	0.02	132	1.62	7420	0.12	116	7.7	3881	-0.11	109	-6.74	11753

RANBP1	1.12	0.003818052	0.03	98	1.62	7421	0.18	89	7.05	4226	0.05	113	3.18	6839
ZDHHC13_t1	-1.01	0.892107662	0.02	120	1.62	7422	NA	NA	NA	NA	-0.07	124	-4.87	11003
TTC23L	1.05	0.000569504	0.04	88	1.62	7423	0.1	104	5.59	5013	0.02	87	0.69	8201
POU5F2	1.07	3.25E-11	0.03	112	1.62	7424	0	100	-0.18	8605	0.09	114	5.62	5610
CNTROB_t1	1.03	0.131416979	0.02	115	1.62	7425	-0.05	104	-2.94	10433	-0.07	103	-3.62	10441
ERAP1	-1.02	0.490505492	0.02	121	1.62	7426	0.05	117	3.17	6402	0.06	156	6	5453
ARHGEF18	-1.01	0.853903171	0.02	132	1.62	7427	0.02	137	2.08	7091	-0.09	139	-7.8	12163
TLR5	-1.03	0.494840154	0.03	110	1.62	7428	0.01	125	0.76	7965	-0.04	133	-2.95	10160
SPECC1L	1.04	0.419740212	0.02	117	1.61	7429	-0.02	125	-1.52	9508	-0.02	134	-1.7	9553
QRICH2	1.06	7.32E-09	0.07	72	1.61	7430	0.06	104	3.19	6389	0.12	80	3.54	6648
NFKBIL1	1.09	0.000206141	0.03	108	1.61	7431	0.01	101	0.26	8282	0.07	92	3.12	6862
FADS2	1.18	0.233519538	0.02	121	1.61	7432	0.04	118	2.84	6593	0.11	120	8.03	4604
ZNFX1	-1	0.878285473	0.02	144	1.61	7433	-0.06	146	-6.1	12243	-0.07	153	-7.33	11968
KLHDC9	1.06	0.564406974	0.03	107	1.61	7434	-0.04	116	-2.58	10205	0.02	127	1.42	7796
LYZL1	1.11	5.79E-08	0.03	104	1.61	7435	-0.05	108	-2.66	10257	0.05	116	3.52	6661
PTH2R	1.34	9.11E-06	0.02	132	1.61	7436	0	130	-0.16	8586	0	128	0.3	8437
LACRT	1.02	0.038651514	0.04	88	1.61	7437	-0.02	84	-0.66	8916	-0.02	120	-1.69	9544
NLRC5_t1	-1.03	0.576249521	0.02	126	1.61	7438	0.06	148	5.87	4861	NA	NA	NA	NA
KIF6_t1	1.03	0.083596356	0.02	125	1.61	7439	NA	NA	NA	NA	0.08	115	5	5924
COMMD6	-1.01	0.59219034	0.02	138	1.6	7440	0.07	116	4.92	5381	-0.07	139	-6.13	11489
KRTAP15-1	1.06	1.14E-05	0.04	93	1.6	7441	0.01	66	0.08	8412	0.07	95	3.22	6820
MYBBP1A	-1	0.905958858	0.03	111	1.6	7442	-0.04	120	-3.15	10572	0.02	94	0.69	8196
DDAH2	1.03	0.682733263	0.03	107	1.6	7443	-0.02	95	-0.79	9010	0	89	-0.07	8663
SNRNP48	1.02	0.675859635	0.02	154	1.6	7444	-0.02	123	-1.18	9273	0.13	138	11.59	3328
CREBZF	1.03	0.344184141	0.01	164	1.6	7445	0.12	132	9.66	3001	0.08	128	6.36	5290
VMP1	1.03	0.636320557	0.02	114	1.6	7446	0.13	124	9.87	2909	-0.02	148	-2.16	9779
PPP6R2	-1.02	0.661996369	0.03	112	1.59	7447	-0.08	109	-4.54	11408	-0.07	144	-6.57	11676
EPHA8_t1	1.1	2.92E-11	0.03	104	1.59	7448	0.09	132	7.13	4181	0.06	151	5.77	5548
ABCD2	-1	0.816668103	0.02	130	1.59	7449	0.04	129	3.28	6333	-0.05	130	-3.66	10464
FOXQ1	1.02	0.854391652	0.02	114	1.59	7450	-0.1	115	-6.61	12468	0	148	-0.27	8775
ZNF587	1.11	0.004265236	0.02	141	1.59	7451	0.11	142	9.92	2887	0	153	0.18	8502
TAP2_t2	1.07	5.80E-08	0.03	104	1.59	7452	-0.07	113	-4.42	11338	0.05	103	2.58	7142
NDUFB4	1.09	0.024788646	0.02	131	1.59	7453	0.05	109	2.67	6702	0.03	91	1.33	7839
TCF20	1.19	5.49E-06	0.03	108	1.58	7454	0.17	130	13.42	1706	0.15	123	11.14	3458
KLHL8	-1.09	0.00568422	0.02	136	1.58	7455	-0.08	134	-6.89	12600	0.1	127	7.83	4698
PI4KB	1.17	1.12E-08	0.02	120	1.58	7456	0.1	129	8.12	3686	0.06	120	4.3	6285
CLIC3	1.02	0.521565583	0.05	84	1.58	7457	-0.01	88	-0.55	8849	-0.13	72	-2.77	10069
ACOT7	1.2	3.39E-05	0.03	108	1.58	7458	-0.06	109	-3.34	10705	0.08	110	5.03	5911
GIP	1.06	0.002819064	0.04	88	1.58	7459	-0.14	120	-9.46	13681	0.09	109	5.57	5637
ADD2_t2	-1.01	0.473137595	0.02	120	1.58	7460	0	126	0.02	8452	0.04	136	3.42	6705
NDUFA3	1.02	0.654942589	0.04	88	1.57	7461	-0.09	97	-4.3	11267	0.06	88	2.14	7375
CTCFL	1.02	0.156464664	0.02	126	1.57	7462	0.03	148	3.2	6384	-0.02	152	-1.73	9569
ZNF570	-1.02	0.227746277	0.03	102	1.57	7463	-0.06	105	-3.53	10828	0.05	111	3.18	6840
ALG13_t2	1.19	0.000144219	0.02	130	1.57	7464	0	134	-0.29	8663	0.17	134	14.12	2566
LGR5	1.14	1.61E-05	0.04	94	1.57	7465	-0.07	100	-3.53	10830	-0.03	75	-0.78	9071
OBP2A	-1.02	0.096409567	0.04	88	1.57	7466	-0.19	117	-12.77	14708	0.01	100	0.33	8414
MTSS1	-1.01	0.922200782	0.02	128	1.57	7467	-0.13	126	-9.71	13780	0.18	130	14.06	2580
MBTPS1_t1	-1.08	0.019014263	0.02	119	1.56	7468	-0.04	107	-2.39	10075	NA	NA	NA	NA
FAM73B_t1	1.04	0.003446623	0.02	115	1.56	7469	NA	NA	NA	NA	0.06	149	5.48	5693
RPS24	1.12	0.059645143	0.02	129	1.56	7470	0.12	94	5.49	5064	0.05	91	1.86	7530
FGF5	-1.01	0.585297338	0.02	144	1.56	7471	0.02	152	2.1	7080	-0.02	130	-1.75	9590
FCER2	1.02	0.020388349	0.04	92	1.56	7472	0.01	120	1.02	7798	-0.01	84	-0.33	8809
BCL10_t1	-1.01	0.709397244	0.02	139	1.55	7473	NA	NA	NA	NA	0.06	132	5.05	5901
FBXL8	-1.1	0.017648818	0.05	84	1.55	7474	-0.06	121	-4.2	11202	-0.05	102	-2.62	10006
PLD3	-1.15	1.09E-05	0.03	100	1.55	7475	-0.13	107	-7.29	12783	-0.13	88	-4.99	11045
GOLGA4	-1.04	0.623589907	0.02	120	1.55	7476	0.02	117	1.14	7718	0.12	138	10.7	3615
EIF3M	1.17	0.001216898	0.03	107	1.55	7477	0.17	98	8.19	3644	0.1	108	5.57	5639
OPCML_t1	-1.01	0.284411308	0.01	161	1.55	7478	NA	NA	NA	NA	0.05	152	4.99	5932
GLRA2	1	0.759806069	0.02	124	1.54	7479	-0.03	113	-1.89	9743	0.07	156	7.57	4805
APOD	1.05	0.147340765	0.02	114	1.54	7480	0.07	120	4.83	5434	0.07	105	3.82	6514
CSHL1_t1	1.03	0.037099145	0.05	83	1.54	7481	-0.03	120	-2.28	10014	0.03	120	1.99	7455
PITPNM2	1.3	0.000447825	0.02	119	1.54	7482	0.02	136	1.73	7328	0.16	147	15.97	2081
HELB	-1.05	0.018274675	0.03	108	1.54	7483	0	111	-0.23	8624	-0.04	116	-2.4	9886
DUSP4	-1.02	0.636792545	0.02	146	1.54	7484	-0.05	140	-4.27	11254	-0.14	153	-14.79	14324
MYL2	1.02	0.202615793	0.03	109	1.54	7485	0.08	111	4.63	5532	0	108	-0.19	8724
MIPEP	-1.07	0.063206155	0.03	111	1.54	7486	-0.07	124	-5.3	11825	-0.08	112	-4.78	10977
WDR31_t1	1.06	0.013481843	0.02	122	1.54	7487	NA	NA	NA	NA	NA	NA	NA	NA
MAGED4B	1.22	0.022059351	0.03	97	1.54	7488	0.08	116	5.55	5039	-0.06	126	-4.42	10809
GNRH1	1.01	0.81455297	0.04	93	1.53	7489	0.11	133	9.17	3202	0.03	134	2.92	6981
CGGBP1	1.07	0.027893767	0.02	140	1.53	7490	0.04	127	2.7	6682	0.15	154	15.98	2076
COL11A1	1.29	9.37E-05	0.02	134	1.53	7491	0.04	139	3.85	5982	0.05	138	4.45	6218
LIMD1	1.1	1.82E-05	0.02	127	1.53	7492	0.09	136	8.08	3702	0.1	153	10.56	3671
SERTM1	1.05	0.000100117	0.02	121	1.53	7493	0.08	152	8.67	3428	0.1	120	7.09	4995
ADAM7	1.08	1.24E-14	0.02	120	1.53	7494	-0.05	124	-3.93	11058	0.07	124	5.08	5890
ELK3	1.03	0.094885656	0.02	117	1.53	7495	0.15	124	11.3	2361	0.02	150	2.29	7299
PPM1B_t2	-1.03	0.521936305	0.02	144	1.53	7496	0.07	137	5.75	4925	0.02	131	1.26	7874
ERMN_t1	-1.02	0.125736837	0.01	153	1.52	7497	NA	NA	NA	NA	NA	NA	NA	NA
ZNF574	-1.01	0.773175134	0.02	130	1.52	7498	-0.14	104	-7.79	13029	-0.07	113	-4.51	10843
ADCY2_t1	-1	0.713668212	0.02	128	1.52	7499	NA	NA	NA	NA	NA	NA	NA	NA
ZNF181_t1	1.04	0.197889255	0.02	113	1.52	7500	NA	NA	NA	NA	0.11	115	6.9	5068
LHCGR	1.02	0.034420216	0.02	137	1.52	7501	0.02	129	1.77	7308	-0.02	150	-2.34	9865
CCDC122	1.06	0.02030125	0.02	123	1.52	7502	0.1	120	7.32	4079	0.02	150	1.8	7568
CLUL1	1.03	0.050216791	0.02	121	1.52	7503	0.17	126	12.74	1915	0.04	151	4.24	6319
GGA2_t1	1.08	0.014502731	0.01	153	1.52	7504	NA	NA	NA	NA	-0.05	142	-4.79	10983
CTNS	1	0.917838096	0.01	152	1.52	7505	-0.05	149	-5.1	11723	0	150	0.48	8330
C1QTNF9	-1.01	0.788603653	0.02	126	1.51	7506	-0.21	144	-20.09	16000	0.05	112	3.32	6761
STAU1	-1	0.911013905	0.02	117	1.51	7507	-0.03	127	-2.11	9907	-0.01	125	-0.82	9097
ANKRD2_t1	1.12	0.000240099	0.03	99	1.51	7508	0.12	100	5.79	4902	NA	NA	NA	NA
IL34	-1.05	0.006248	0.04	93	1.51	7509	0.01	114	0.88	7884	-0.12	101	-6.24	11541
SPOP_t1	1.01	0.735391672	0.02	135	1.51	7510	NA	NA	NA	NA	NA	NA	NA	NA
MIF	1.24	0.008540355	0.11	64	1.51	7511	0.23	96	10.78	2560	0.09	104	4.63	6109
RANGRF	-1.12	0.027759167	0.02	111	1.5	7512	0.12	110	7.43	4022	-0.24	101	-12.06	13563
HTR3C	1.03	0.009208218	0.03	108	1.5	7513	-0.03	120	-1.98	9809	0.1	101	5.12	5867
FGF2	1.03	0.698450285	0.02	132	1.5	7514	-0.09	124	-6.88	12595	-0.12	141	-11.27	13336
GABRA3	1.02	0.067424658	0.02	130	1.5	7515	0.08	144	7.3	4088	0.03	152	2.87	7006
GOLGA8A	1.24	5.51E-07	0.02	131	1.5	7516	0.14	124	10.64	2619	0.11	147	10.9	3548
NTRK1	1.01	0.36916605	0.02	113	1.5	7517	0.14	105	7.66	3903	0.04	94	1.94	7483
POMP	-1.01	0.915889232	0.02	121	1.5	7518	0.1	129	7.79	3837	-0.08	130	-6.42	11612
NEDD9	-1.11	0.011271711	0.02	140	1.5	7519	0.04	126	2.84	6595	-0.04	127	-2.85	10117
RPL24	1.13	0.00229574	0.06	74	1.5	7520	0.09	86	3.21	6371	0.07	106	3.81	6521
PIWIL3	1.04	0.000237448	0.02	124	1.5	7521	-0.02	106	-0.9	9076	0.03	121	2.31	7289
STRN4	1.03	0.169437765	0.03	108	1.5	7522	0	149	-0.24	8638	-0.09	127	-6.66	11718
HIST1H4D	1.32	1.03E-07	0.06	75	1.5	7523	0.08	118	5.44	5097	0.03	95	1.55	7716
ASF1B	1.17	4.41E-08	0.03	109	1.49	7524	-0.07	120	-5.09	11712	0.06	106	3.21	6825
PRTFDC1	1.11	0.068513952	0.02	129	1.49	7525	0.08	154	8.11	3691	0.02	116	1.58	7706
VSTM2A	1	0.781582701	0.04	88	1.49	7526	-0.02	119	-1.32	9374	-0.07	107	-3.98	10629

SPRR1A	1.01	0.550405603	0.03	109	1.49	7527	-0.07	104	-3.93	11062	0.05	122	3.55	6644
MRPL21	1.15	7.22E-06	0.03	102	1.49	7528	0.02	109	1.3	7603	0.14	86	4.91	5972
PHLDA2	1.25	0.062820209	0.03	104	1.49	7529	0.06	115	3.94	5933	0.09	127	6.78	5117
IDS_t2	1.03	0.033324978	0.03	100	1.49	7530	0.08	89	3	6495	-0.02	97	-1.02	9216
CHCHD4	1.16	1.18E-05	0.02	120	1.48	7531	-0.02	124	-1.69	9609	0.16	124	12.15	3150
UBL4B	1.04	0.009438587	0.02	117	1.48	7532	-0.1	153	-10.62	14111	0.03	115	1.78	7583
GOLGB1_t1	-1.09	0.230995712	0.02	120	1.48	7533	NA	NA	NA	NA	-0.02	132	-1.54	9469
PLEKHA3	1.04	0.392443837	0.02	131	1.47	7534	0	112	0.22	8316	0.1	135	8.24	4519
UBE2D4	1.09	0.008694563	0.02	139	1.47	7535	0.02	144	1.68	7368	0.07	130	5.38	5745
LRIG3	1.01	0.901328397	0.02	115	1.47	7536	-0.06	106	-3.28	10657	0	131	-0.25	8765
ACVR1B_t1	1.02	0.020560284	0.02	134	1.47	7537	0	127	0.21	8327	0.01	154	1.24	7882
PIAS1	1.06	0.025918184	0.02	114	1.47	7538	0.09	104	4.73	5483	0.14	120	10	3879
BCCIP_t2	-1.08	0.09128921	0.03	108	1.47	7539	-0.09	103	-4.65	11467	0.06	90	2.43	7214
CREB5	-1.01	0.872309698	0.02	140	1.47	7540	0.12	143	11.24	2384	0.02	164	1.87	7525
EGFL6	1.06	5.40E-07	0.02	139	1.46	7541	0.07	125	5.07	5293	0.1	144	9.73	3973
TSPO	-1.05	0.28110433	0.03	103	1.46	7542	0.04	107	2.55	6802	-0.06	100	-2.85	10114
GLUL	1.7	0.001548939	0.02	126	1.46	7543	0.07	120	5.18	5224	-0.01	124	-0.6	8986
CBFA2T2_t2	1	0.818043336	0.02	124	1.46	7544	0.01	138	0.66	8031	0.02	132	1.85	7532
NKX6-2	1.01	0.453284009	0.03	104	1.46	7545	0	93	0.21	8322	0.05	108	2.72	7073
RELT	-1.02	0.419118887	0.02	120	1.46	7546	0.03	123	2.13	7061	-0.11	122	-7.94	12209
TLE2	-1.21	0.003312885	0.04	90	1.46	7547	-0.18	120	-12.39	14613	-0.03	89	-0.98	9192
PSG6	1.06	0.001276123	0.04	88	1.46	7548	-0.02	80	-0.54	8847	0.01	68	0.23	8474
REG3A	2.14	1.66E-05	0.03	97	1.45	7549	-0.01	95	-0.23	8625	0.12	95	5.55	5663
GNL2	1.07	0.234502475	0.06	76	1.45	7550	0.08	72	1.84	7247	0.12	97	5.69	5584
SLC24A2	1.01	0.177025405	0.02	137	1.45	7551	-0.07	144	-6.66	12497	-0.07	178	-9.06	12611
RNLS	1.1	0.029679552	0.02	123	1.45	7552	-0.04	119	-3.03	10489	0.15	127	11.63	3316
EFHB	1.07	0.00021381	0.04	88	1.45	7553	0.12	110	7.01	4242	0.03	113	1.89	7517
PAM16	1.05	0.064294659	0.03	96	1.45	7554	0.05	88	1.97	7163	0.12	81	3.61	6610
RASGEF1C	1.06	0.000665975	0.02	113	1.45	7555	-0.09	118	-5.86	12115	0.07	106	3.92	6454
TMEM62	1.03	0.164671065	0.02	115	1.45	7556	-0.13	102	-6.63	12479	0.01	100	0.71	8190
INTS5	1.06	0.000466834	0.02	120	1.44	7557	-0.05	98	-2.27	10009	0.05	121	3.54	6651
UROD	-1.02	0.549667704	0.03	106	1.44	7558	-0.09	100	-4.35	11287	0	120	-0.17	8711
NRP1_t1	1.03	0.636603687	0.07	72	1.44	7559	0.04	83	1.25	7639	0.07	50	0	8620.5
SF3B1_t2	-1.08	0.21442663	0.02	115	1.44	7560	0.12	114	7.45	4010	-0.07	138	-6.57	11673
NOP2	1.18	2.17E-07	0.03	92	1.44	7561	0.1	98	4.7	5499	0.12	98	5.67	5592
WWC1	-1.09	0.247379018	0.02	131	1.44	7562	-0.07	159	-7.12	12704	0	133	0.08	8569
TOM1L1	1.07	0.113033774	0.02	110	1.43	7563	0	135	-0.04	8515	0.13	144	12.37	3080
OPLAH	1.06	0.462170453	0.07	71	1.43	7564	-0.06	80	-1.68	9605	0.12	73	2.74	7057
ZC3H11A_t1	1.11	0.000784742	0.01	152	1.43	7565	NA	NA	NA	NA	0.13	131	10.38	3733
ZNF263	1.04	0.121355121	0.02	116	1.43	7566	-0.02	117	-1.14	9250	0.01	114	0.68	8211
GBX2	-1	0.717499568	0.02	111	1.43	7567	-0.01	116	-0.34	8707	0.02	111	0.97	8035
TIRAP_t2	1.07	0.001957812	0.03	104	1.43	7568	0.03	133	2.59	6765	0	126	0.11	8551
CXCR5_t1	1.02	0.126436424	0.03	106	1.42	7569	NA	NA	NA	NA	-0.05	111	-2.82	10097
TRIOBP_t1	1.1	0.001122317	0.02	126	1.42	7570	0.05	144	4.29	5720	0.02	130	1.7	7624
TAF6L	1.01	0.678056223	0.03	103	1.42	7571	-0.07	88	-2.71	10277	0	99	-0.12	8685
PHF3	-1.14	0.019076882	0.02	130	1.42	7572	-0.09	111	-5.27	11809	0.08	166	9.11	4183
TRAPPC1	-1.02	0.411250574	0.04	85	1.42	7573	-0.09	122	-6.24	12300	-0.07	105	-3.75	10513
DKKL1	1.05	1.63E-07	0.05	81	1.42	7574	0.12	84	3.91	5946	0.15	80	4.55	6152
LEPROT_t1	-1.08	0.088232236	0.02	133	1.42	7575	NA	NA	NA	NA	-0.03	153	-2.87	10121
STARD3	-1.04	0.251872074	0.02	112	1.42	7576	-0.02	131	-1.65	9588	-0.1	122	-7.14	11895
MRPS24	1.12	0.003694589	0.04	84	1.42	7577	-0.04	108	-2.08	9881	0.15	91	6.02	5444
FZR1	1.06	8.42E-05	0.02	137	1.42	7578	0.05	124	3.37	6265	-0.03	112	-1.86	9648
ST5	1.07	0.054083981	0.02	110	1.42	7579	-0.07	123	-4.92	11606	0.03	122	1.83	7545
SSBP3	1.02	0.312119267	0.02	124	1.41	7580	-0.07	110	-4.29	11261	0.01	112	0.68	8212
DEPDC5	1.1	0.045053823	0.03	104	1.41	7581	-0.04	122	-2.54	10175	0.05	100	2.73	7065
PPP2R5D	1.08	0.000541163	0.02	120	1.41	7582	-0.01	126	-0.61	8879	0.06	117	4.24	6321
PPDPF	1.04	0.641120471	0.03	91	1.41	7583	-0.07	121	-4.67	11477	0.04	103	2.09	7400
SCAND1_t1	1.06	0.011035332	0.06	72	1.41	7584	-0.03	96	-1.6	9554	NA	NA	NA	NA
NHLH1	1.02	0.07678501	0.02	129	1.41	7585	0.13	137	11.33	2347	-0.08	120	-5.89	11400
KCNMA1_t1	-1.02	0.377083926	0.02	134	1.41	7586	NA	NA	NA	NA	NA	NA	NA	NA
SETD2	1.02	0.13535909	0.02	115	1.41	7587	0.06	109	3.57	6133	0.17	116	11.03	3503
KIAA0317	1.14	0.000176766	0.01	148	1.41	7588	0.1	142	8.95	3301	0.02	128	1.21	7910
CACNG5	1.03	0.011416215	0.07	69	1.41	7589	0.02	72	0.41	8197	0.1	88	3.9	6466
KLHL26	1.01	0.746331735	0.03	102	1.4	7590	-0.06	138	-5.69	12022	0.02	128	1.93	7494
POSTN	1.17	0.038636977	0.02	120	1.4	7591	0.03	126	2.32	6939	-0.03	137	-2.37	9879
DIDO1_t3	1.18	1.97E-06	0.02	111	1.4	7592	0.1	147	9.67	2994	0.07	122	5.16	5849
TSSK4	1.03	0.063355464	0.04	88	1.4	7593	-0.04	68	-0.63	8893	-0.01	92	-0.49	8901
SNAPC1	-1.14	0.002047461	0.02	140	1.4	7594	0.05	133	4.38	5672	0	125	0.13	8538
MYADM_t1	-1	0.827355341	0.02	132	1.4	7595	NA	NA	NA	NA	NA	NA	NA	NA
HSD3B2	1.01	0.27964648	0.02	120	1.4	7596	-0.14	124	-10.59	14105	0.02	100	0.87	8095
BMP8A	1.02	0.286661924	0.02	137	1.4	7597	-0.03	128	-2.04	9855	-0.01	128	-0.82	9096
PDZD8	-1.1	0.023272301	0.02	138	1.4	7598	-0.1	122	-7.11	12700	0.06	120	3.86	6493
SSSCA1	1.11	0.00139796	0.08	68	1.39	7599	0.01	92	0.23	8304	NA	NA	NA	NA
PIKFYVE	-1.05	0.324021277	0.01	152	1.39	7600	0.03	131	2.51	6819	0.09	158	9.26	4131
AMZ2	1.08	0.054518933	0.02	125	1.39	7601	0.07	112	4.52	5602	0	121	-0.3	8795
WNT9A	1.02	0.043599141	0.06	74	1.39	7602	-0.07	101	-3.61	10884	0.04	132	3.01	6920
AVPR2_t1	-1.05	0.003598781	0.04	85	1.39	7603	-0.13	104	-7.05	12673	NA	NA	NA	NA
KLHDC5_t1	1.01	0.485249576	0.01	154	1.39	7604	NA	NA	NA	NA	NA	NA	NA	NA
TIMM23	1.14	2.38E-05	0.02	112	1.38	7605	0.15	125	10.96	2491	0.1	159	10.73	3599
IGSF21	-1.04	0.505367975	0.03	104	1.38	7606	0.02	120	1.43	7535	0.01	102	0.65	8229
ICA1L_t2	1.02	0.182275613	0.02	136	1.38	7607	NA	NA	NA	NA	NA	NA	NA	NA
EREG	1.05	0.016765273	0.01	151	1.38	7608	0.03	129	2.25	6988	-0.11	167	-13.41	13957
GFI1	1.01	0.852909333	0.02	126	1.38	7609	0.03	120	1.87	7230	-0.15	132	-12.58	13720
HBZ	1.02	0.132891471	0.05	80	1.38	7610	-0.05	88	-1.96	9787	0.06	84	2.19	7353
FAM173A	1.01	0.766963979	0.03	92	1.38	7611	-0.06	101	-3.23	10618	0.06	68	1.05	7992
PHC3	-1.05	0.022638394	0.02	136	1.38	7612	0.07	140	6.35	4592	-0.09	163	-10.06	12947
YAP1	-1.01	0.75594031	0.02	125	1.37	7613	0.02	124	1.24	7652	-0.03	126	-2.46	9931
TNIP2	1.02	0.590046466	0.02	113	1.37	7615	0	115	0.03	8446	0.03	120	1.99	7457
SRCRB4D	1.42	1.45E-07	0.02	125	1.36	7616	0.05	105	2.68	6694	0.11	117	7.1	4992
ATP13A4_t1	1.01	0.344989302	0.02	123	1.36	7617	0.07	123	5.31	5165	NA	NA	NA	NA
WBSCR28	1.07	1.50E-06	0.1	63	1.36	7618	0.01	64	0.14	8367	0.12	68	2.22	7340
MRGPRX3	1.03	0.003163359	0.02	125	1.36	7619	-0.04	120	-3.06	10506	0.1	165	11.08	3485
ADCY9_t1	1.01	0.627967177	0.02	139	1.36	7620	NA	NA	NA	NA	0.08	134	6.76	5126
CATSPERB	1.06	0.091064211	0.03	97	1.36	7621	-0.02	84	-0.84	9039	0.02	98	1.1	7969
TTTY6	1.04	0.001205069	0.02	107	1.35	7622	0	110	0.16	8358	0.05	100	2.63	7118
TBCA	1.14	0.014008688	0.02	115	1.35	7623	0.14	130	11.41	2331	0.11	124	7.88	4676
DENND1A_t2	-1.05	0.195322888	0.02	111	1.35	7624	-0.2	124	-14.64	15135	NA	NA	NA	NA
RPL26_t1	-1.02	0.786653266	0.02	120	1.35	7625	NA	NA	NA	NA	NA	NA	NA	NA
FOSL2_t1	-1.2	4.89E-05	0.02	122	1.35	7626	NA	NA	NA	NA	NA	NA	NA	NA
MED8_t2	1.14	8.63E-07	0.02	131	1.35	7627	0.02	126	1.64	7395	0.11	145	10.41	3720
MFSD3	1.21	0.000152692	0.05	78	1.34	7628	-0.03	78	-0.89	9070	0.2	68	3.63	6601
ERGIC3_t1	1.05	0.163713801	0.03	102	1.34	7629	-0.12	125	-9.18	13582	0.03	93	1.38	7823
WFDC8_t1	1.02	0.069778788	0.02	114	1.34	7630	NA	NA	NA	NA	0.02	119	1.29	7858
SLC35D2	1.08	0.06889476	0.02	110	1.34	7631	-0.02	109	-1.4	9428	0.08	104	4.15	6353
GALNT9	1.06	0.000273135	0.03	100	1.34	7632	-0.06	120	-4.24	11234	0.13	101	6.66	5172
BSPRY_t1	-1.2	0.054428268	0.02	110	1.34	7633	-0.04	105	-2.32	10035	NA	NA	NA	NA

PRKAR2A	1.03	0.154990441	0.02	120	1.34	7634	0.01	134	0.69	8003	0.11	146	10.36	3739
PLXDC2	-1.16	0.031822223	0.02	115	1.33	7635	0.03	125	2.13	7056	-0.18	116	-12.09	13572
RECQL5_t2	1.12	1.76E-08	0.02	135	1.33	7636	0.02	120	1.27	7625	0.03	131	2.04	7428
COX15	-1.02	0.589936162	0.01	139	1.33	7637	-0.07	151	-7.3	12789	0.1	140	8.69	4350
IL22	1.02	0.014270103	0.01	145	1.33	7638	-0.01	126	-0.96	9117	0.15	126	11.61	3323
GDF3	-1.01	0.554893251	0.04	87	1.33	7639	-0.08	84	-2.88	10400	0	80	0.03	8598
ABCB9_t1	1.03	0.009646239	0.03	96	1.33	7640	NA	NA	NA	NA	NA	NA	NA	NA
EXT2	-1.08	0.003691188	0.02	126	1.32	7641	-0.07	131	-5.27	11808	-0.06	114	-3.55	10416
VCY	1.66	6.77E-08	0.03	91	1.32	7642	-0.02	86	-0.55	8851	0.11	103	5.85	5523
TRAF3IP2	1.02	0.717637899	0.02	135	1.32	7643	0.04	131	2.85	6582	-0.04	123	-3.25	10290
GPRIN3	-1	0.845791323	0.02	129	1.32	7644	0.19	133	16.07	1028	-0.01	121	-0.59	8981
TUBA8	1.17	0.001045015	0.03	92	1.32	7645	0.05	124	3.99	5909	0.1	104	5.54	5666
MTCP1	1.01	0.381045259	0.02	127	1.32	7646	-0.03	113	-1.96	9789	0.06	111	3.47	6682
CXCL10	1.17	0.142324483	0.02	115	1.31	7647	-0.01	118	-0.45	8782	0.02	126	1.74	7607
KCNQ2_t2	1.07	2.26E-06	0.02	108	1.31	7648	0.12	88	4.45	5639	0.15	97	6.99	5037
TAS2R38	1.01	0.168185044	0.03	88	1.31	7649	0.04	99	2.11	7077	0.08	72	1.74	7602
FAM207A	-1.05	0.189272845	0.04	80	1.31	7650	-0.09	117	-5.72	12038	-0.07	120	-4.81	10989
ANAPC10	-1	0.924912152	0.02	132	1.31	7651	-0.05	135	-4.47	11370	0.08	112	5.11	5872
TRMT1	1.22	3.86E-11	0.13	60	1.31	7652	0.12	98	5.86	4868	0.09	64	1.29	7859
CLN5	-1.1	0.004023961	0.02	132	1.31	7653	-0.05	138	-4.45	11359	0.05	144	4.78	6038
MAGEA12	2.4	1.97E-13	0.02	125	1.31	7654	-0.03	111	-1.71	9626	0.2	116	12.93	2913
PNLIPRP2	1.11	0.008391108	0.05	76	1.31	7655	0.03	124	2.47	6844	0.15	120	10.16	3807
ATP5G3	1.07	0.26809779	0.01	148	1.31	7656	0.01	131	1.21	7675	0.04	134	3.41	6715
PSG4	1.03	0.012772859	0.02	114	1.3	7657	0.08	114	5.06	5299	0.03	124	2.14	7373
ATP5I_t1	1.06	0.145561049	0.02	105	1.3	7658	NA	NA	NA	NA	0.12	90	4.75	6049
MAML3	1.01	0.153126626	0.02	116	1.3	7659	0.01	135	0.95	7831	0.02	153	2.24	7327
PRPF38A	1.09	0.009524177	0.02	129	1.29	7660	0.14	108	8.35	3566	0.12	119	8.17	4551
RAET1E	1.05	3.26E-06	0.13	60	1.29	7661	0.12	80	3.52	6168	0.02	68	0.43	8350
ELSPBP1	1.01	0.284265181	0.03	100	1.29	7662	-0.02	104	-0.85	9045	0.04	133	3.25	6803
ZNF141	-1	0.953039077	0.07	68	1.28	7663	0.1	74	2.3	6960	0.06	80	1.76	7593
WBP2NL	-1.01	0.241405575	0.02	119	1.28	7664	-0.08	138	-7.1	12695	0.09	137	7.91	4660
LHX3	1.05	0.000442402	0.03	101	1.28	7665	0.12	120	8.59	3461	0.05	114	2.9	6990
NT5C	1.16	5.83E-06	0.04	84	1.28	7666	0.15	86	5.43	5101	0.01	102	0.26	8462
CCDC7	1.04	0.001299022	0.02	120	1.28	7667	0.1	105	5.74	4930	0.09	108	5.03	5909
WWOX_t2	1.03	0.092915717	0.04	84	1.28	7668	-0.11	129	-8.32	13247	0.13	104	6.96	5051
FCRLA	1.04	0.226486392	0.02	135	1.28	7669	0.05	103	2.44	6862	-0.07	130	-5.34	11179
FBXO22_t1	-1.01	0.828452239	0.02	134	1.28	7670	0.02	134	1.87	7231	0.11	128	8.64	4372
FLRT1	1.01	0.554562043	0.02	123	1.28	7671	0.01	135	0.88	7885	-0.09	121	-6.07	11463
USP29	1.03	0.006956949	0.02	121	1.27	7672	0.02	112	1.16	7701	0.07	111	4.43	6229
MRPS27	1.09	0.012481174	0.02	118	1.27	7673	0.01	133	0.65	8034	0.06	146	5.62	5613
HIP1R	1.22	0.000121626	0.02	104	1.27	7674	0.18	127	14.21	1471	0.02	122	1.31	7847
NSA2_t1	1.04	0.433397984	0.02	117	1.26	7675	NA	NA	NA	NA	0.08	106	4.68	6083
ATP1A3	1.16	0.001736135	0.03	91	1.26	7676	0.05	105	2.72	6663	0.05	120	3.54	6653
PAX7	1.02	0.102766795	0.03	100	1.26	7677	-0.03	109	-1.91	9752	0.01	84	0.34	8405
PATE1	1.03	0.006439397	0.02	120	1.25	7678	-0.07	128	-5.6	11971	0	123	0.35	8398
GPS1	1.24	2.41E-08	0.03	89	1.25	7679	0.01	103	0.4	8209	0.11	104	6.02	5443
IFNGR2	-1.09	0.066726539	0.02	114	1.25	7680	0.17	132	13.91	1554	-0.22	102	-11.29	13346
INSM2	1.04	1.24E-06	0.02	121	1.25	7681	0.06	119	4.22	5767	0.12	143	11.18	3451
CRYBA4	1.03	0.006581615	0.04	85	1.25	7682	0.01	102	0.75	7972	0.06	100	3.1	6870
ZFHX2	1.11	0.027614091	0.02	104	1.25	7683	0.11	119	7.4	4034	0.01	127	0.87	8098
CCNJL_t1	1.01	0.757860577	0.01	149	1.24	7684	NA	NA	NA	NA	0.02	111	1.13	7949
PRX_t2	1.01	0.324979771	0.03	94	1.24	7685	-0.02	96	-0.78	8995	0.01	119	0.77	8154
CA11	-1.01	0.50057408	0.02	107	1.24	7686	-0.13	108	-7.29	12780	-0.07	147	-6.94	11819
LHX5	1.01	0.134464107	0.02	102	1.24	7687	0.1	144	8.94	3308	0.03	100	1.7	7625
PML_t2	-1.02	0.71984425	0.02	104	1.24	7688	0.1	136	9	3269	-0.1	155	-10.17	12981
TMOD4	1.03	0.012160846	0.03	97	1.23	7689	0.21	111	13.08	1806	0.04	114	2.65	7105
ARHGAP32_t1	1.02	0.077619011	0.01	139	1.23	7690	NA	NA	NA	NA	0.03	145	2.89	6997
TRIM38	-1.02	0.570708899	0.01	152	1.23	7691	-0.07	135	-6.18	12279	-0.08	144	-7.37	11987
STH	1.1	5.50E-10	0.14	59	1.23	7692	0.1	52	0.21	8328	0.23	60	2.32	7275
TINAG	1.16	1.42E-06	0.02	101	1.23	7693	0.12	122	8.95	3306	0.14	90	5.78	5546
HLA-DMA_t1	-1.2	0.021057022	0.03	88	1.23	7694	NA	NA	NA	NA	NA	NA	NA	NA
IMPDH2	1.21	2.63E-05	0.04	80	1.23	7695	-0.01	109	-0.76	8986	0.08	83	2.77	7044
DEFB126_t1	1.03	0.004579814	0.07	68	1.23	7696	NA	NA	NA	NA	0.17	88	6.65	5176
ARHGAP36	1.01	0.350736015	0.02	124	1.23	7697	0.01	117	0.68	8022	0.13	119	8.89	4264
MED6_t1	1.01	0.686898061	0.01	133	1.22	7698	-0.05	151	-5.43	11886	NA	NA	NA	NA
VTA1_t1	1	0.993221961	0.02	121	1.22	7699	NA	NA	NA	NA	0.03	134	2.23	7330
CTDP1_t2	1.01	0.407041544	0.02	109	1.22	7700	-0.07	141	-6.18	12273	-0.07	115	-4.41	10802
ACVRL1	-1.06	0.252692903	0.02	113	1.22	7701	0.05	138	4.29	5719	-0.03	120	-2.32	9852
BMP3	1.02	0.084970146	0.01	133	1.22	7702	-0.05	142	-4.52	11399	0.07	155	7	5034
PDC	1.02	0.03584336	0.01	133	1.22	7703	-0.12	116	-7.73	13002	0.06	126	4.89	5975
KIAA0020	1	0.965890538	0.02	108	1.22	7704	0.13	106	7.29	4098	0.05	96	2.31	7285
UNC5D	1.03	0.014142584	0.01	146	1.21	7705	-0.03	133	-2.34	10052	0.12	134	9.8	3949
PPP1CA	1.01	0.672212185	0.02	118	1.21	7706	-0.02	97	-0.73	8950	-0.07	109	-3.91	10592
PPP1R3A	1.01	0.197660478	0.01	149	1.21	7707	-0.06	111	-3.5	10795	0.12	129	9.28	4125
SCMH1	1.02	0.37982881	0.02	116	1.21	7708	0.08	147	7.37	4051	-0.03	110	-1.57	9481
UBE4A	1.05	0.316673783	0.02	129	1.2	7709	0.15	136	12.48	1985	0.08	131	6.59	5192
MOCS3	1.07	0.000116448	0.01	132	1.2	7710	0.06	119	4.26	5741	0.06	136	4.92	5967
STK35_t1	1.02	0.05288862	0.01	151	1.2	7711	0.05	135	3.83	5991	NA	NA	NA	NA
PPFIA2	1.04	8.18E-05	0.01	136	1.2	7712	0.11	114	6.88	4311	0.11	134	9.65	4005
PARN_t1	1.06	0.035110209	0.02	112	1.2	7713	NA	NA	NA	NA	NA	NA	NA	NA
TSSK1B	1.07	2.07E-10	0.02	120	1.2	7714	0.04	118	2.81	6609	0.02	122	1.29	7857
GH1	1.02	0.304504588	0.04	80	1.2	7715	-0.15	120	-10.77	14166	0.04	117	2.7	7085
B3GALT2	1.05	0.001046679	0.02	120	1.2	7716	0.07	126	5.57	5024	-0.03	126	-2.57	9987
DDX56	1.09	0.000742455	0.01	136	1.19	7717	-0.02	119	-1.31	9371	0.07	127	5.24	5813
INTS6	-1.19	1.99E-05	0.01	137	1.19	7718	-0.11	131	-8.67	13384	-0.14	143	-13.37	13945
POTEB	1.23	8.69E-05	0.06	70	1.19	7719	0.05	73	1.26	7632	0.06	71	1.33	7836
CCDC22	1.1	0.000442496	0.02	110	1.19	7720	-0.07	99	-3.27	10655	0.03	94	1.25	7879
ALDH3B1	-1.01	0.82756796	0.01	137	1.19	7721	0.06	129	5.13	5261	-0.14	111	-8.81	12529
SDK1_t1	1.01	0.22608265	0.02	100	1.18	7722	0.01	108	0.84	7903	0.01	114	0.75	8165
FLYWCH1_t2	-1.02	0.312450835	0.02	127	1.18	7723	-0.04	145	-4.02	11107	0	137	0.27	8455
TMBIM1_t1	1.01	0.848230971	0.01	131	1.18	7724	NA	NA	NA	NA	-0.07	154	-7.24	11933
SPDYE3	1.12	1.04E-11	0.01	129	1.18	7725	0.19	132	15.38	1175	0.14	128	11.03	3509
TRIM54	1.02	0.090368574	0.02	107	1.18	7726	-0.01	106	-0.32	8694	0.02	107	1.3	7852
CCRL2_t1	-1.09	0.000760889	0.02	122	1.18	7727	0.05	118	3.4	6247	NA	NA	NA	NA
DGKD	1.07	0.134325256	0.02	125	1.17	7728	0.17	122	12.21	2060	-0.02	121	-1.5	9444
MTRF1L	-1.02	0.340384759	0.01	136	1.17	7729	0.01	150	0.81	7925	0.05	125	4.08	6382
DCTN5	1.03	0.250986054	0.01	151	1.17	7730	0.11	150	11.32	2353	0.06	128	4.67	6090
SNCG	1.1	0.000181829	0.02	120	1.17	7731	0.14	134	11.75	2208	0.03	102	1.38	7818
S100G	-1.01	0.211737034	0.02	102	1.17	7732	-0.03	88	-1.23	9314	0.02	125	1.52	7738
MYRIP	-1.62	0.001249845	0.02	119	1.17	7733	-0.16	128	-12.12	14539	-0.02	132	-1.32	9353
LSM10	1.04	0.140736087	0.03	95	1.17	7734	-0.02	118	-1.57	9527	0.07	103	3.55	6641
POU4F3	1.03	0.004866415	0.06	70	1.17	7735	0	93	0.01	8465	0.14	84	4.93	5962
FABP4	1.23	0.000326146	0.02	105	1.17	7736	0.02	108	1.29	7616	0.04	128	2.89	6998
DUSP8_t1	1.12	0.031477383	0.02	125	1.16	7737	NA	NA	NA	NA	0.05	122	3.4	6716
METTL21C_t1	1	0.659554288	0.01	135	1.16	7738	NA	NA	NA	NA	0.05	107	3.09	6879
TRPM3_t2	1.05	7.94E-06	0.03	95	1.16	7739	0.06	99	3.04	6475	0.1	85	3.58	6624

PRDM16_t1	1	0.610382715	0.02	122	1.16	7740	0.05	130	4.37	5675	-0.01	143	-1.1	9252
RNF148	1.1	9.68E-07	0.02	119	1.15	7741	0.21	110	12.72	1922	0.12	136	10.07	3847
SAGE1	1.07	0.003235158	0.03	83	1.15	7742	0.11	114	7.06	4222	0.08	92	3.34	6751
DHX34	1.05	0.000252663	0.02	106	1.15	7743	-0.04	130	-3.26	10645	0.05	111	3.02	6917
RBM41	1.23	1.30E-07	0.02	124	1.15	7744	0.09	134	7.61	3927	0.2	110	12.08	3171
NDUFS8	1.14	6.77E-07	0.08	64	1.15	7745	0.05	120	3.57	6132	0.12	80	3.67	6579
NDUFB11	1.02	0.637540013	0.04	78	1.15	7746	-0.12	95	-5.54	11937	0.04	84	1.5	7744
FBXO44	1.16	6.89E-06	0.02	122	1.14	7747	-0.05	108	-2.64	10243	0.1	118	6.67	5165
KIF27	-1	0.924597353	0.01	156	1.14	7748	0.04	118	2.55	6798	0.04	94	1.66	7643
FOXP3	1.04	0.018761328	0.02	123	1.14	7749	0.01	138	0.65	8035	0	110	0.12	8541
FGD3	-1.03	0.042216189	0.02	105	1.14	7750	0.02	110	1.35	7575	-0.17	103	-9.05	12607
PSORS1C2	1.05	0.000277792	0.02	118	1.13	7751	-0.01	114	-0.64	8903	0	98	0.04	8593
S100A7	1.16	3.23E-05	0.11	60	1.13	7752	-0.01	80	-0.29	8673	0.25	60	2.52	7173
RWDD4	-1.08	0.009004447	0.01	138	1.13	7753	-0.01	156	-1.47	9472	-0.03	119	-2.34	9866
GJA8	1.05	6.68E-05	0.11	60	1.12	7754	-0.01	71	-0.28	8660	NA	NA	NA	NA
SLC46A2	-1.01	0.476174549	0.01	150	1.12	7755	0.05	120	3.46	6210	-0.1	124	-7.31	11960
OR4K1	1.04	0.000125801	0.04	77	1.12	7756	0.1	108	5.66	4969	-0.05	84	-1.72	9565
FAM81B	1.03	0.008781675	0.02	102	1.12	7757	0.06	85	1.94	7179	0.06	89	2.41	7225
TMEM5	1.07	0.040806716	0.03	93	1.12	7758	0.03	52	0.07	8420	0.09	96	4.31	6282
STAT2	-1.06	0.014680232	0.02	124	1.12	7759	-0.12	120	-8.24	13212	-0.06	132	-5.16	11102
UBL7	1.05	0.005012787	0.02	121	1.11	7760	-0.08	111	-4.98	11648	-0.02	105	-1.27	9327
SIM2_t1	1.04	0.007185674	0.01	148	1.11	7761	-0.01	125	-0.45	8788	0	136	0.41	8361
COX6B2	1.04	0.000332218	0.02	119	1.11	7762	-0.09	127	-7.12	12708	0.03	127	2.29	7296
CDC14A_t2	1.03	0.009787572	0.02	124	1.11	7763	0.12	116	7.67	3896	0.1	118	6.49	5247
PROCA1	1.19	7.19E-13	0.09	63	1.11	7764	0.07	61	0.75	7969	0.23	80	6.79	5112
HIST1H2AK	1.31	1.50E-07	0.03	90	1.11	7765	0.1	98	5.03	5316	0.13	76	3.39	6725
THSD4	1.03	0.002475441	0.01	126	1.11	7766	-0.07	138	-5.95	12155	0.04	134	3.06	6901
ITGB1BP2	1.1	0.003535386	0.03	88	1.11	7767	0.02	84	0.72	7986	0.08	87	2.96	6952
WFDC10B	1.03	0.00294375	0.03	90	1.11	7768	0.07	136	5.65	4978	0.04	88	1.59	7697
IFITM3	-1.03	0.659436799	0.03	88	1.11	7769	-0.08	113	-5.2	11778	-0.05	120	-3.64	10455
RPLP2	1.2	0.000164363	0.07	67	1.11	7770	0.18	114	11.57	2280	0.1	88	3.82	6513
SAMD9_t1	-1.04	0.174684205	0.01	132	1.11	7771	NA	NA	NA	NA	NA	NA	NA	NA
FKBP14	1.1	0.007469707	0.01	149	1.11	7772	0.2	135	17.1	818	0.1	159	11.13	3461
HIGD2A	1.05	0.092252964	0.01	124	1.11	7773	-0.07	120	-4.74	11512	0.01	100	0.65	8226
CSMD3	1.07	0.013724333	0.01	127	1.1	7774	-0.05	140	-4.1	11153	0.03	124	2.29	7302
BRD4	-1.02	0.37748088	0.02	116	1.1	7775	-0.01	114	-0.7	8942	-0.04	114	-2.84	10108
CRYGC	1.02	0.129111633	0.02	104	1.1	7776	-0.04	111	-2.68	10262	0.1	105	5.49	5685
ANKRD13D	1.02	0.361497294	0.02	99	1.1	7777	0.13	120	9.02	3259	-0.1	95	-4.58	10879
ASB5	1.04	0.000588651	0.01	141	1.1	7778	0.08	130	6.57	4467	0.11	133	9.45	4066
BMP4	1.19	0.003053828	0.02	121	1.1	7779	0.05	131	4.35	5692	-0.01	92	-0.42	8865
FRG1	1.02	0.75252549	0.03	92	1.09	7780	-0.03	88	-1.3	9367	0.01	77	0.15	8518
SERPINA9	1.06	1.84E-05	0.02	114	1.09	7781	-0.06	120	-4.25	11245	0.07	119	4.7	6071
KIAA0240	-1.01	0.850846435	0.01	139	1.09	7782	0.06	136	5.21	5214	-0.06	133	-4.92	11024
PIH1D1	1.13	0.00086306	0.02	95	1.09	7783	-0.07	82	-2.09	9888	-0.01	113	-0.49	8910
PDLIM3	-1.53	0.00024614	0.01	125	1.09	7784	0.1	144	9.57	3030	-0.11	137	-9.73	12824
ZNF131	1.02	0.766963979	0.01	133	1.07	7785	0.05	109	2.84	6596	0.13	122	9.53	4046
DUS1L	1.26	3.87E-09	0.02	119	1.07	7786	0.04	110	2.44	6860	0.13	96	5.88	5507
TAC4	1.03	0.010215438	0.02	95	1.07	7787	-0.11	130	-9.04	13523	0.01	122	1.01	8011
KCNT1	1.01	0.405016647	0.02	113	1.07	7788	-0.07	136	-6.17	12271	-0.02	120	-1.39	9388
COQ7	1.03	0.360923323	0.01	136	1.07	7789	0.03	114	1.77	7301	0	144	0.36	8396
PTPRH	-1.18	0.025873504	0.01	155	1.06	7790	-0.1	113	-6.09	12239	-0.09	100	-4.3	10753
TFPT	1.1	0.000669321	0.11	60	1.06	7791	0.01	127	1.02	7799	0.01	80	0.16	8514
NRAP	1.01	0.492356212	0.02	99	1.06	7792	0.05	102	2.4	6889	0.03	76	0.68	8210
ADIG	1.02	0.02292568	0.02	100	1.06	7793	-0.01	91	-0.52	8829	0.04	82	1.33	7837
TTTY10	1	0.746526036	0.02	117	1.06	7794	0.01	131	0.44	8179	0	156	0.52	8305
PRKD2	1.07	0.020864776	0.02	100	1.06	7795	0.01	103	0.54	8116	-0.05	92	-2.24	9816
ZNF311_t1	1.04	0.008126502	0.01	130	1.06	7796	NA	NA	NA	NA	0.09	111	5.23	5815
DCST1	1.02	0.080356285	0.04	74	1.05	7797	-0.08	104	-4.22	11221	0.05	98	2.3	7292
ZNF618	1.09	0.04499131	0.01	143	1.05	7798	0.02	159	1.84	7252	0.01	132	0.78	8151
GUCY2D	1.09	8.52E-07	0.02	115	1.05	7799	0	125	0.21	8324	0	147	0.04	8594
TOR2A_t1	1.04	0.080771582	0.02	117	1.05	7800	NA	NA	NA	NA	-0.01	113	-0.58	8972
WDR24	1.11	0.001505612	0.03	84	1.05	7801	-0.1	56	-0.6	8874	0.04	95	1.63	7661
XKR5	1.03	0.00162253	0.01	140	1.05	7802	0.08	134	6.96	4267	0.05	125	4.1	6374
LZTS2_t1	1.01	0.804755386	0.01	124	1.04	7803	0.12	138	10.83	2544	NA	NA	NA	NA
HOMER3	-1.06	0.433059972	0.02	96	1.04	7804	0.11	116	7.49	3991	-0.07	97	-3.22	10277
SCGB1D1	1.32	0.000624419	0.02	103	1.04	7805	0	95	0.17	8355	0.11	126	8.31	4493
TRIM56	1.17	7.08E-07	0.01	144	1.04	7806	0.14	119	9.51	3056	0.03	136	2.7	7083
TSGA10IP	1.04	0.00014024	0.05	72	1.04	7807	0.02	66	0.4	8208	0.09	57	0.61	8252
WDR54	1.05	0.43625819	0.03	83	1.04	7808	-0.02	104	-0.87	9054	-0.07	90	-2.83	10102
TNFRSF19_t2	1.43	0.00024022	0.01	142	1.04	7809	0.1	163	11.05	2453	-0.04	148	-3.72	10496
IFI30	-1.03	0.714045836	0.02	104	1.04	7810	0.15	98	7.26	4113	-0.14	100	-7.09	11880
POLR1A	1.08	9.86E-07	0.02	108	1.04	7811	0.05	128	4.11	5839	0.05	103	2.41	7226
DPP8_t2	-1	0.973519747	0.01	140	1.03	7812	0.04	122	3.08	6447	0.14	121	10.29	3766
HOXC13	1.02	0.409682996	0.02	111	1.03	7813	-0.14	144	-13.63	14904	0	126	0.14	8531
XAGE5	1.35	1.14E-06	0.01	146	1.03	7814	0.12	80	3.62	6103	0.08	88	2.99	6929
ZNF804A	1.01	0.381928237	0.01	127	1.03	7815	0.03	112	2.04	7119	-0.06	133	-5.2	11116
TSHB	1.07	9.83E-07	0.02	105	1.03	7816	-0.04	115	-2.56	10188	0.04	117	2.4	7231
CRIM1	1	0.824438204	0.01	133	1.03	7817	0.07	123	4.86	5424	0.08	138	6.66	5171
MRPS28	1.08	0.091016435	0.02	103	1.03	7818	0.01	100	0.5	8136	0.08	94	3.45	6693
HIST1H4C	1.35	3.43E-05	0.02	96	1.02	7819	0.08	84	2.61	6745	-0.01	70	-0.11	8681
TRIP10	1	0.91198006	0.02	100	1.02	7820	0.13	128	9.81	2930	-0.05	120	-3.28	10299
PADI4	-1.01	0.334520934	0.02	103	1.02	7821	-0.12	136	-10.71	14137	0.06	126	4.88	5986
CLUAP1	1.01	0.82898773	0.01	141	1.02	7822	0.08	132	6.84	4332	0.01	139	0.76	8159
PDCL2	1.01	0.272537019	0.05	69	1.02	7823	-0.08	88	-3.14	10567	0.15	71	3.08	6890
BHLHA15	1.04	0.02918737	0.03	85	1.02	7824	-0.05	88	-1.99	9816	0.02	68	0.34	8412
GRM4	1.03	0.009882044	0.02	117	1.02	7825	0.05	118	3.43	6230	0.06	125	4.44	6226
PDPN_t1	1.04	0.359434043	0.01	148	1.02	7826	0.07	128	5.22	5206	-0.08	123	-5.93	11416
PTPRE_t1	-1.22	4.39E-05	0.01	137	1.02	7827	NA	NA	NA	NA	NA	NA	NA	NA
NARS2	-1.04	0.404873369	0.01	133	1.01	7828	-0.01	125	-0.66	8913	0.03	131	2.23	7336
TEKT3	1.02	0.092041928	0.02	104	1.01	7829	0.06	104	3.09	6441	-0.02	102	-0.8	9088
ODF1	1.03	0.005096476	0.01	118	1.01	7830	0.05	104	2.45	6855	0.08	89	2.93	6970
EPHX4	1.09	0.002817206	0.02	108	1.01	7831	0	106	-0.11	8560	0.1	126	7.8	4712
TICAM1	-1.07	0.228748488	0.02	101	1.01	7832	-0.11	120	-7.97	13103	-0.09	102	-4.58	10884
FAM154B	1.02	0.153304287	0.01	129	1.01	7833	0.03	136	2.23	6997	0.09	138	8.13	4567
EN1	1.03	0.041886152	0.02	110	1.01	7834	-0.08	112	-4.84	11562	-0.06	108	-3.73	10504
PPP1R36	1.03	0.089513096	0.02	107	1.01	7835	0.15	132	12.65	1943	-0.02	92	-0.8	9087
SERINC3_t1	-1.01	0.764692824	0.01	143	1.01	7836	-0.15	125	-11.55	14387	0.07	152	6.8	5102
KIF9	1.06	0.000932373	0.02	97	1.01	7837	0.12	135	10.29	2759	0.09	124	6.8	5104
ZNF280A	1.04	0.000137297	0.02	94	1	7838	0.03	116	1.68	7363	0.06	104	3.41	6712
ARMCX6	-1.17	0.004691613	0.02	112	1	7839	0.1	134	8.18	3652	-0.14	131	-11.14	13293
ARF4	1.09	0.055732698	0.01	122	1	7840	0.19	122	13.79	1600	0.05	140	4.82	6015
ARHGEF7_t1	1.01	0.643942914	0.01	140	1	7841	0.12	128	9.6	3023	0.02	145	1.61	7680
REG1B	1.24	0.000483373	0.01	131	1	7842	-0.2	87	-7.27	12770	0.07	123	5.18	5838
ZSCAN2_t2	1.06	9.44E-05	0.01	140	1	7843	0.01	126	0.49	8144	0.13	120	9.18	4166
PTPN20B	1.13	1.66E-05	0.01	128	1	7844	0.12	119	7.99	3748	0.13	143	12.43	3059
ARHGAP17	-1.03	0.330394754	0.01	140	1	7845	0.16	111	9.57	3029	-0.13	114	-8.42	12405

SNAI2	1.01	0.89716005	0.01	132	1	7846	0.07	134	6.21	4658	-0.03	130	-2.72	10046
TEX12	1	0.874811495	0.01	141	1	7847	0.14	108	8.23	3632	0.02	126	1.54	7723
STRA6	1.06	0.000219883	0.02	104	1	7848	0.06	129	5.01	5331	-0.03	120	-2.14	9772
DHDH	1.24	6.09E-06	0.04	73	0.99	7849	0.03	102	1.71	7343	0.12	75	2.98	6938
CA4	1.25	5.73E-06	0.02	92	0.99	7850	0	104	0.01	8461	0.1	100	4.99	5925
PKP4	1.04	0.292522788	0.01	121	0.99	7851	0.02	131	1.3	7608	0.04	118	2.96	6950
ENPP6_t1	1.11	0.001337886	0.01	135	0.99	7852	NA	NA	NA	NA	-0.01	144	-0.7	9032
TNFRSF25	1.16	0.072366009	0.01	120	0.99	7853	0.18	108	10.17	2798	0.01	115	0.78	8152
LAYN	-1.04	0.504051061	0.01	136	0.99	7854	0.07	112	4.12	5831	0.01	127	0.42	8358
TFPI_t2	-1.34	0.003108118	0.01	135	0.99	7855	-0.09	130	-6.86	12588	0.06	136	4.93	5963
DNAAF2	1.15	0.000414741	0.01	124	0.99	7856	0.01	120	0.45	8173	0.24	130	19.37	1369
FCHSD1	-1.01	0.623052152	0.01	132	0.98	7857	-0.08	135	-6.6	12465	-0.05	120	-3.29	10307
DNAJB2_t2	1.06	0.020368778	0.02	114	0.98	7858	0.05	117	3.07	6457	-0.05	124	-4.05	10659
KCTD13	1.05	1.22E-06	0.01	121	0.97	7859	0.1	154	10.83	2542	0.04	151	4.28	6300
FAM174A	1.05	0.220433115	0.02	113	0.97	7860	-0.06	102	-3.3	10667	0.1	123	7.55	4818
GPCPD1	-1.07	0.285185591	0.01	145	0.97	7861	0.07	132	5.94	4817	0.05	142	4.63	6107
AXIN1	-1.07	0.094282285	0.01	132	0.97	7862	0.04	120	2.73	6661	-0.07	122	-5.33	11174
TSPAN8	1.18	0.252635627	0.02	104	0.96	7863	0.16	109	9.62	3014	0.06	122	4.06	6388
HCFC1R1	1.02	0.688259075	0.03	80	0.96	7864	0.02	108	1.42	7546	0.02	94	0.73	8176
TLL1	-1.03	0.0025114	0.01	131	0.96	7865	0.03	130	2.65	6721	-0.05	138	-4.79	10979
BDKRB1	1.24	0.000669185	0.03	80	0.96	7866	0.09	70	1.76	7312	0.11	104	5.7	5580
SNX20	-1.03	0.020656111	0.01	141	0.96	7867	0.05	123	3.47	6205	-0.16	118	-11.16	13298
ZNF644_t1	-1.05	0.198229941	0.01	130	0.96	7868.5	0	122	-0.22	8618	NA	NA	NA	NA
MRPS18B	1.11	0.010645887	0.01	130	0.96	7868.5	0.16	132	12.84	1882	0.03	120	2.34	7265
WNK1	1.01	0.776091725	0.01	140	0.96	7870	0.02	126	1.9	7218	0.01	155	0.6	8257
DNAH17	1.07	1.62E-06	0.02	96	0.95	7871	0	146	0.2	8330	0.13	110	7.84	4691
IGFL2	1.06	0.057456984	0.03	80	0.95	7872	-0.03	126	-2.64	10240	0.02	116	1.16	7940
SIX6	1.04	2.14E-05	0.01	114	0.95	7873	0.05	102	2.46	6848	0.07	116	4.8	6029
ASB6	1.06	0.003771221	0.01	120	0.94	7874	0.07	136	5.76	4918	0.05	129	4.02	6408
MIB2	1.07	0.025101866	0.03	84	0.94	7875	-0.07	79	-1.96	9790	0.03	72	0.6	8259
H2AFY2	1.05	0.295732779	0.02	96	0.94	7876	0.06	135	5.26	5185	-0.01	116	-0.45	8881
ZW10	1.04	0.230615227	0.02	110	0.94	7877	0.01	110	0.61	8062	0.09	115	5.55	5653
TFDP1	1.03	0.212080547	0.01	125	0.94	7878	-0.04	139	-3.95	11072	0.06	139	5.55	5660
ZNF773	1.06	0.162355341	0.01	131	0.94	7879	0.23	135	19.76	424	0.01	128	0.57	8279
EML2	1.03	0.463992228	0.02	102	0.94	7880	0.04	104	2.08	7096	-0.05	88	-2.04	9728
DENND2C_t1	1	0.771021203	0.01	124	0.94	7881	-0.04	114	-2.64	10247	NA	NA	NA	NA
CNN1	-1.17	0.221742463	0.02	98	0.94	7882	0.06	120	4.11	5838	-0.05	131	-4.33	10767
GAP43	1.07	0.000423226	0.01	144	0.94	7883	0.14	120	10.09	2829	0.1	142	8.78	4310
ZFP42	1.02	0.329572152	0.01	140	0.93	7884	-0.02	131	-1.85	9730	0.02	127	1.42	7800
TRIM43	1.01	0.400789747	0.01	115	0.93	7885	0.04	111	2.74	6648	-0.01	128	-0.5	8916
ZNF596	1.04	0.082161948	0.01	130	0.93	7886	0.07	119	4.59	5561	0.02	120	1.47	7769
TBL1Y	1.07	0.171473051	0.01	120	0.93	7887	0.04	108	2.2	7013	-0.01	109	-0.48	8897
PRLHR	1.01	0.491514403	0.01	120	0.93	7888	-0.04	118	-2.83	10355	-0.01	147	-1.02	9214
CCDC114	1.03	0.002520009	0.02	111	0.93	7889	-0.04	110	-2.24	9989	-0.05	100	-2.69	10035
CTSC_t1	-1.16	0.013729473	0.01	147	0.93	7891	0.12	142	10.7	2594	-0.19	131	-15.7	14518
FN3K	1.09	0.000908378	0.04	74	0.92	7892	0.02	107	1.18	7692	0.04	88	1.63	7658
CABS1	-1	0.758008649	0.01	120	0.92	7893	-0.04	155	-3.88	11023	-0.05	115	-3.45	10375
PTOV1_t1	-1.04	0.12151764	0.01	112	0.92	7894	NA	NA	NA	NA	NA	NA	NA	NA
SRSF3	-1.11	0.03151905	0.01	147	0.92	7895	0.06	135	5.52	5053	-0.13	137	-11.53	13411
PSTPIP2_t1	-1.03	0.195787279	0.01	120	0.92	7896	NA	NA	NA	NA	-0.05	163	-5.85	11387
HBE1	1.05	0.01018229	0.02	100	0.92	7897	-0.06	123	-4.56	11418	0.12	113	7.38	4880
MAPK12	1.05	0.036386984	0.02	100	0.92	7898	-0.17	117	-11.17	14289	0	107	-0.16	8704
CD70	1.12	0.020269702	0.01	112	0.92	7899	0.13	98	6.01	4779	-0.05	106	-2.95	10157
CCDC104	-1.02	0.78329757	0.02	94	0.91	7900	0.07	109	4.26	5748	-0.04	122	-3.05	10206
RNASE11	1.01	0.142098646	0.01	111	0.91	7901	0.01	123	0.95	7834	-0.05	129	-3.69	10477
HNRNPD_t1	-1.02	0.591280696	0.02	109	0.91	7902	0	110	-0.04	8517	0.08	130	6.66	5170
BCL7C	-1.01	0.730188951	0.03	79	0.91	7903	-0.01	77	-0.24	8632	0.03	108	1.5	7750
NOXA1	1.08	0.035594788	0.02	100	0.91	7904	-0.06	88	-2.44	10114	-0.01	80	-0.37	8832
FASTKD1	1.07	0.122882886	0.02	103	0.91	7905	0.03	113	2.12	7065	0.14	98	6.49	5244
GALE	1.01	0.813219433	0.02	103	0.91	7906	-0.05	119	-3.27	10653	-0.01	133	-0.63	9002
SLC6A4	1.04	0.142323839	0.01	150	0.9	7907	-0.12	135	-10.21	13957	0.06	150	5.9	5494
NDUFA11	1.05	0.089548381	0.03	76	0.9	7908	-0.02	80	-0.51	8825	0.01	80	0.4	8377
MYH1	1	0.86577061	0.02	108	0.9	7909	-0.02	125	-1.23	9315	-0.09	127	-7.14	11893
TRAPPC11_t2	-1.15	0.000808029	0.01	112	0.9	7910	-0.08	118	-5.39	11867	-0.06	122	-4.49	10836
MGST3	1	0.972466101	0.01	136	0.9	7911	0.11	142	10.55	2656	0.04	120	2.53	7166
USP30	1.04	0.084161823	0.01	127	0.9	7912	0.04	129	3.52	6171	0.07	137	6.36	5294
HOXB1	1.02	0.032008118	0.02	88	0.89	7913	-0.06	95	-2.55	10177	0.08	100	3.94	6443
SLC8A2	1.04	2.16E-05	0.02	108	0.89	7914	0.02	129	1.65	7384	0.05	119	3.59	6617
DISP1	1.16	0.017377062	0.08	61	0.89	7915	-0.01	94	-0.57	8860	0.15	84	5.03	5908
SUDS3	1.04	0.115824016	0.01	129	0.89	7916	0	135	0.26	8283	-0.01	128	-0.59	8978
MAGI2	-1.01	0.865602608	0.01	126	0.89	7917	-0.04	124	-2.8	10342	0.05	144	4.48	6194
NTSR1	1.03	0.003430564	0.01	124	0.89	7918	0.06	123	4.08	5856	0.03	125	2.11	7391
SNRNP70	1.01	0.675461845	0.01	132	0.89	7919	0.05	101	2.34	6930	-0.09	108	-5.38	11192
MED25	1.04	0.078743503	0.04	74	0.89	7920	-0.06	108	-3.44	10758	0.04	74	0.85	8108
MCCD1_t1	1.17	0.001876166	0.03	84	0.89	7921	NA	NA	NA	NA	0.11	100	5.26	5793
ZNF564	1.03	0.1759908	0.01	135	0.89	7922	-0.01	117	-0.98	9133	0.08	130	6.31	5318
ZNF536	1.01	0.420113867	0.01	120	0.88	7923	-0.02	125	-1.5	9496	0.09	120	6.08	5409
RASIP1	1.01	0.647740222	0.02	100	0.88	7924	-0.15	79	-4.33	11279	-0.05	108	-2.65	10018
SSTR5	1.05	1.96E-05	0.02	108	0.88	7925	-0.05	118	-3.2	10596	0.09	144	8.48	4433
TXNDC2	1.11	3.68E-12	0.02	89	0.88	7926	0.07	77	2	7144	0.07	88	2.64	7112
REM2	1.01	0.502110016	0.01	125	0.88	7927	0.06	124	4.26	5743	0.04	129	3.11	6866
ITGA11	1.12	0.006055083	0.01	121	0.88	7928	0.06	161	6.17	4684	-0.05	150	-5.3	11155
INO80B	1.03	0.055533987	0.02	100	0.88	7929	-0.12	90	-4.82	11550	0.02	109	1.47	7772
TACR3	1.07	1.26E-09	0.02	93	0.87	7930	0.06	106	3.54	6148	0.17	92	6.96	5054
GPR68_t1	1	0.831330474	0.01	115	0.87	7931	NA	NA	NA	NA	-0.01	120	-0.58	8968
PRKRIR	1.03	0.458092166	0.01	132	0.87	7932	0.05	137	4.78	5454	0.07	131	5.33	5763
TTLL11	1.01	0.74273386	0.01	108	0.87	7933	-0.03	88	-1.19	9287	-0.06	95	-2.6	10000
FCGR1B	-1.14	0.10586418	0.01	129	0.87	7934	0.09	115	5.61	5001	-0.15	120	-10.81	13184
TRPV3_t1	-1.01	0.21654337	0.01	144	0.87	7935	0.01	145	1.18	7691	NA	NA	NA	NA
IER5L	-1.32	0.001326568	0.01	112	0.86	7936	0.03	128	2.71	6674	-0.1	162	-11.69	13452
NR1H2	-1	0.945865472	0.02	105	0.86	7937	-0.07	118	-4.84	11557	-0.07	104	-3.77	10522
RTDR1	1.16	4.71E-07	0.05	68	0.86	7938	0.05	84	1.63	7405	0.13	88	5.11	5876
DUS3L	1.09	0.000660608	0.04	74	0.86	7939	0	84	-0.16	8585	0.06	87	2.08	7410
PRKCDBP_t1	1.12	0.124493778	0.01	116	0.86	7940	NA	NA	NA	NA	NA	NA	NA	NA
ZSCAN18_t1	-1.06	0.031569529	0.01	156	0.86	7941	NA	NA	NA	NA	-0.06	124	-4.58	10883
OR6W1P	-1.03	0.083382437	0.01	120	0.86	7942	-0.16	121	-11.55	14385	-0.08	120	-5.43	11214
HRH2	1.01	0.558980653	0.01	108	0.85	7943	-0.14	113	-8.73	13409	-0.07	100	-3.63	10453
DUX2	1.05	0.003702756	0.06	64	0.85	7944	0	64	-0.06	8522	0.18	61	1.93	7496
SCAMP2	1.07	0.00362031	0.02	97	0.85	7945	-0.03	98	-1.32	9373	-0.01	128	-1.03	9218
ANKMY2	1.02	0.363853209	0.01	130	0.85	7946	0.05	140	4.25	5752	0.07	116	4.58	6134
IRF9	-1.07	0.114123341	0.02	99	0.85	7947	-0.1	115	-6.75	12534	-0.12	103	-6.57	11677
CCDC60	1.01	0.450832955	0.01	130	0.84	7948	0.04	122	2.81	6605	-0.03	125	-2.17	9780
SAMD7	1.01	0.136594014	0.01	113	0.84	7949	-0.06	115	-3.73	10946	0.03	113	1.69	7629
KIAA1211L	1.06	0.111809021	0.02	104	0.84	7950	0.18	146	17.12	815	-0.08	125	-5.69	11315
GPR22	1.04	0.081160052	0.01	128	0.84	7951	-0.03	122	-1.82	9704	0.01	106	0.29	8442
CROCC	1.03	0.202012368	0.01	108	0.84	7952	-0.09	111	-5.51	11927	-0.02	116	-1.47	9433

GOLGA7	-1.01	0.884895822	0.01	120	0.84	7953	-0.03	130	-2.5	10154	-0.01	131	-0.66	9017
KIF1A	1.05	2.34E-05	0.01	125	0.83	7954	-0.02	156	-2.5	10155	0.08	120	5.75	5562
SLC9A1	1.01	0.611915946	0.01	120	0.83	7955	0.1	168	12	2127	-0.06	120	-4.15	10695
ENTPD7	1.03	0.394003462	0.01	135	0.83	7956	-0.04	138	-3.08	10525	0.05	136	4.63	6115
BRK1	1.07	0.003938297	0.01	134	0.83	7957	0.07	126	5.4	5121	0	155	0.17	8509
KDM4C	1.04	0.419960462	0.01	118	0.83	7958	0.06	125	4.23	5762	0.03	127	2.12	7385
SCD5_t1	-1	0.755091973	0.01	136	0.82	7959	0	118	-0.01	8491	0.01	130	0.49	8323
CDKN1B_t1	-1.09	0.094800124	0.01	129	0.82	7960	-0.11	128	-8.88	13462	NA	NA	NA	NA
GRK1	1.09	9.62E-16	0.02	100	0.82	7961	0.03	105	1.45	7519	0.1	102	5.09	5887
RNF133	1.03	0.000756254	0.02	97	0.82	7962	0.07	91	2.77	6628	0.07	96	3.08	6888
LCE3C	1.03	0.00835621	0.03	77	0.82	7963	-0.12	100	-6.16	12264	0.07	74	1.63	7665
CFL1	-1.01	0.59312395	0.01	105	0.82	7964	0.04	113	2.68	6693	-0.07	118	-4.69	10932
SNTG2	1.01	0.485671877	0.02	95	0.82	7965	-0.11	96	-4.88	11580	0.01	120	0.67	8214
XRN2	1.02	0.633465746	0.01	131	0.82	7966	-0.02	114	-1.44	9457	0.05	134	3.89	6477
TOMM7_t1	1.05	0.21660232	0.01	128	0.81	7967	NA	NA	NA	NA	0.1	130	7.74	4736
LDHC	1.17	3.53E-06	0.02	104	0.81	7968	-0.03	110	-1.96	9791	0.1	142	8.85	4279
TDRD5	1.02	0.038780811	0.01	126	0.81	7969	-0.01	110	-0.55	8850	0.08	114	5.16	5848
PLCXD2	-1.01	0.499792292	0.01	115	0.81	7970	-0.11	123	-8.22	13200	-0.06	114	-3.59	10429
CERS2	1.11	0.043473986	0.01	119	0.81	7971	-0.04	139	-3.2	10602	0.2	113	12.9	2921
ADAM18	-1.03	0.011546025	0.02	95	0.81	7972	-0.18	102	-9.37	13645	-0.12	126	-8.88	12553
TM2D2	-1.05	0.114870336	0.01	120	0.81	7973	-0.05	159	-4.95	11628	-0.04	150	-3.75	10509
FERD3L	1.05	3.31E-05	0.02	99	0.81	7974	-0.02	84	-0.64	8905	0.09	100	4.33	6274
EBF2	1.06	7.55E-10	0.01	133	0.81	7975	0.01	137	0.82	7924	0.07	131	5.64	5606
MRPL28	1.04	0.26585573	0.02	102	0.81	7976	-0.07	109	-4.2	11204	-0.02	142	-1.59	9491
LTBP2_t1	-1	0.987623276	0.01	130	0.81	7977	NA	NA	NA	NA	NA	NA	NA	NA
KIFC2	1.26	4.82E-06	0.02	88	0.81	7978	0.04	126	2.93	6534	0.11	124	7.77	4723
BAGE	1.28	1.80E-05	0.01	120	0.8	7979	0.08	124	5.58	5023	0.07	122	4.74	6056
IL5RA_t2	-1	0.757679163	0.02	95	0.8	7980	-0.06	92	-2.57	10195	-0.01	73	-0.31	8798
ZNF700	-1.07	0.002906936	0.01	114	0.8	7981	-0.02	115	-0.99	9141	-0.05	110	-2.82	10095
OSCP1	1	0.971969127	0.01	120	0.8	7982	0.03	109	1.6	7430	0	107	0.07	8573
SLC7A14	1.19	4.76E-05	0.01	150	0.8	7983	-0.02	153	-2.17	9944	0.08	152	7.8	4710
DEFB4A	1.08	7.47E-11	0.04	72	0.8	7984	0.04	84	1.24	7656	0.1	87	3.59	6616
TGIF2	-1.06	0.060147108	0.01	146	0.8	7985	0.05	133	4.08	5857	-0.15	131	-12.29	13642
LYNX1_t1	1.05	0.493959781	0.01	120	0.79	7986	-0.1	151	-9.6	13738	0.12	123	8.77	4314
BLOC1S1	-1.03	0.451086361	0.02	100	0.79	7987	-0.18	88	-6.97	12631	0	80	-0.14	8696
GDPD2	1.01	0.234505271	0.01	112	0.79	7988	-0.06	108	-3.57	10859	0.01	110	0.39	8384
SPAG4	1.22	1.31E-05	0.02	85	0.79	7989	0.05	76	1.41	7551	-0.01	78	-0.28	8778
PRAF2	1.06	0.026971219	0.01	104	0.79	7990	0.02	147	1.61	7418	0.06	116	3.72	6555
PGLYRP4_t1	1.01	0.416589062	0.01	150	0.78	7991	NA	NA	NA	NA	-0.04	115	-2.71	10041
HDAC9_t1	1.01	0.291282453	0.01	107	0.78	7992	0.08	94	3.47	6206	-0.01	93	-0.39	8846
IFITM2	-1.04	0.553588945	0.01	103	0.78	7993	-0.1	110	-5.71	12035	-0.08	123	-5.98	11437
PCED1B	-1.15	0.005216741	0.01	128	0.78	7994	-0.01	133	-0.44	8774	-0.09	129	-7	11848
TRIM42	1.03	0.005642574	0.01	112	0.78	7995	0.1	118	6.52	4501	0	92	-0.07	8661
TSC22D4_t1	-1.02	0.475481599	0.01	120	0.77	7996	0.03	114	1.97	7164	NA	NA	NA	NA
HIST1H4B	1.33	1.15E-05	0.02	83	0.77	7997	0.05	69	0.94	7846	0	92	-0.19	8727
ALPP	1.14	0.001136973	0.01	124	0.77	7998	-0.02	123	-1.15	9253	0.06	121	4.4	6247
ACSS1	1.04	0.126143866	0.01	156	0.77	7999	0.16	142	14.76	1340	-0.14	111	-8.3	12364
XAF1_t1	-1.53	1.14E-07	0.01	131	0.77	8000	NA	NA	NA	NA	-0.07	131	-5.77	11345
KRTAP13-1	1.04	0.000170707	0.01	104	0.76	8001	-0.02	99	-1.06	9189	0.15	105	8.41	4461
PAPD5_t1	-1.13	0.005111084	0.01	144	0.76	8002	-0.1	136	-8.21	13195	0.05	147	4.46	6212
NREP	1.02	0.812199615	0.01	126	0.76	8003	0.04	127	3.43	6227	0.13	127	9.65	4003
RBM23	-1.02	0.249710033	0.01	133	0.76	8004	-0.17	107	-9.81	13821	-0.04	116	-2.75	10060
RBMY1E	1.74	1.00E-07	0.01	134	0.76	8005	-0.04	110	-2.13	9917	0.1	112	5.99	5456
GPR132	1.04	0.000958373	0.01	114	0.76	8006	-0.02	125	-1.41	9441	0.15	120	10.32	3753
TNP2_t1	1.01	0.26377309	0.02	98	0.76	8007	NA	NA	NA	NA	NA	NA	NA	NA
CNN3	1.03	0.685353662	0.01	116	0.76	8008	-0.09	132	-7.62	12938	0.11	142	10.12	3830
RPS4Y1	-1.3	0.013535608	0.02	96	0.75	8009	0.03	81	0.99	7814	0.03	143	2.84	7018
PACSIN1	1.12	0.141067279	0.01	124	0.75	8010	0.05	133	3.99	5911	-0.03	130	-2.09	9753
TMEM42	1.19	0.000108229	0.01	148	0.75	8011	0.06	103	3.2	6387	0.15	113	9.57	4032
FBXW7	-1.09	5.02E-05	0.01	130	0.75	8012	0.03	130	2.09	7086	-0.01	116	-0.94	9157
VN1R2	-1.01	0.46189518	0.02	91	0.75	8013	0.04	125	3.06	6462	-0.06	120	-4.41	10806
GTF2H4	1.05	0.093148777	0.01	104	0.75	8014	-0.09	83	-2.84	10376	0.04	90	1.7	7627
SH3GL1	1	0.850602223	0.01	124	0.74	8015	-0.09	143	-8.46	13306	-0.03	117	-2.24	9818
AP1M2	1.15	0.019742256	0.01	113	0.74	8016	0.08	98	3.64	6093	0.04	108	2.28	7308
CNBD1	1.03	0.00127603	0.01	113	0.74	8017	0.05	108	2.73	6652	0.03	131	2.56	7153
IGLL1	1.08	0.021025687	0.03	78	0.74	8018	-0.04	99	-1.95	9780	0.08	93	3.23	6813
UCMA	1.03	0.030405221	0.01	100	0.74	8019	-0.12	109	-7.3	12791	-0.01	108	-0.37	8830
MAGEB4	-1	0.672636327	0.01	118	0.74	8020	-0.04	129	-3.52	10818	-0.06	124	-4.64	10909
TNNI3	1.1	0.004827902	0.02	96	0.74	8021	0	78	0.12	8389	0.01	90	0.35	8399
RPS6KA6	-1	0.806513859	0.01	158	0.74	8022	-0.06	135	-4.97	11640	-0.01	138	-0.84	9102
UPK2	1.01	0.424245067	0.02	96	0.74	8023	-0.04	152	-3.87	11015	0.04	97	2.03	7433
CCBP2	1.03	0.127064897	0.01	118	0.74	8024	-0.1	120	-7.23	12751	-0.02	122	-1.2	9298
PIBF1	1.08	0.101665085	0.01	134	0.73	8025	0.17	149	16.88	864	0.07	131	5.82	5536
LELP1	1.01	0.22816146	0.03	72	0.73	8026	0	105	0.01	8459	-0.02	100	-1.15	9272
ERAP2	1.03	0.030870984	0.01	146	0.73	8027	0.06	124	4.15	5809	0.04	135	3.23	6815
ATP10D	-1.05	0.134951497	0.01	134	0.73	8028	0.02	118	1.23	7662	-0.09	118	-6.21	11528
RTN4R	-1.18	0.02292568	0.01	133	0.73	8029	-0.21	98	-9.94	13859	-0.1	104	-5.34	11177
CELF1	1.04	0.191582922	0.01	132	0.73	8030	-0.1	123	-7.02	12661	0.04	143	3.64	6596
CELA3B	1.13	0.002248581	0.02	80	0.72	8031	0.03	92	1.25	7640	0.09	87	3.46	6688
KIAA1967	-1.01	0.747195063	0.01	101	0.72	8032	0.03	118	1.92	7199	-0.06	152	-6.57	11674
TNFRSF12A	-1.11	0.315722024	0.01	120	0.72	8033	0.02	117	1.32	7595	0.01	127	0.69	8199
SMARCB1	-1.01	0.805385396	0.02	97	0.72	8034	0.05	129	3.98	5915	-0.06	114	-3.63	10451
LRRC61	1.19	4.03E-05	0.01	112	0.71	8035	0.01	125	0.93	7848	0.05	120	3.36	6740
CDKL5	1.07	0.201971064	0.04	68	0.71	8036	0.16	98	7.68	3894	0.11	89	4.2	6341
RPAP2	1.06	0.000671508	0.01	120	0.71	8037	0.02	122	1.35	7579	0.05	113	2.94	6968
EMC1	1.01	0.760131926	0.01	138	0.71	8038	0.02	140	1.53	7478	0.01	151	0.58	8268
LIN7B	1.14	3.52E-05	0.01	132	0.7	8039	-0.07	114	-4.21	11211	0.09	120	6.13	5386
ACSS3	1.02	0.870183789	0.01	134	0.7	8040	0.08	120	5.82	4884	0.01	137	1.17	7935
ZCCHC6	-1.03	0.451913579	0.01	113	0.7	8041	-0.01	116	-0.83	9037	-0.03	117	-1.75	9583
ZNF69	1.03	0.143749153	0.01	133	0.7	8042	0.06	125	4.27	5732	0.01	120	0.46	8339
INPP5B_t1	-1.07	6.40E-05	0.01	156	0.7	8043	NA	NA	NA	NA	-0.07	135	-6.35	11595
CSF1_t2	1.04	0.009016079	0.07	60	0.7	8044	-0.01	68	-0.09	8552	0.07	57	0.46	8335
SLC24A3	1.02	0.112654403	0.01	148	0.69	8045	-0.04	131	-3.16	10580	0	153	0.49	8321
NADK	-1.08	0.034799094	0.01	128	0.69	8046	-0.06	130	-4.58	11427	-0.19	144	-18.07	14988
RNF11	-1.22	1.94E-05	0.01	129	0.69	8047	-0.15	117	-9.76	13802	0	136	0.24	8466
METTL25	1.01	0.426649023	0.01	118	0.69	8048	0.19	111	11.31	2356	0.02	110	1.23	7891
A4GALT	1.02	0.413717772	0.02	90	0.69	8049	0.11	113	6.64	4427	-0.02	100	-1.12	9261
RAB39A	-1	0.6860715	0.01	134	0.69	8050	0.01	130	1.02	7797	-0.16	129	-12.42	13674
RBM12	1.01	0.835434444	0.01	148	0.69	8051	-0.01	143	-0.75	8970	0.03	137	2.41	7224
NUDT11_t1	1.08	0.06838417	0.01	153	0.68	8052	0.06	157	5.96	4805	NA	NA	NA	NA
ZNF503	1.16	0.015101431	0.01	135	0.68	8053	0.04	154	3.99	5912	0.02	135	1.56	7711
CYP2F1	1.05	0.000267725	0.01	100	0.68	8054	0.05	105	3.01	6488	0.1	115	6.2	5359
CDKN2D	1.03	0.610388386	0.01	116	0.68	8055	0.1	125	7.64	3909	-0.01	110	-0.67	9018
CNEP1R1_t1	-1.05	0.167186642	0.01	142	0.67	8056	0.04	132	3.04	6472	NA	NA	NA	NA
TACR1	-1	0.965890538	0.02	82	0.67	8057	-0.11	108	-6.42	12381	-0.01	88	-0.22	8748
LUZP4	1.12	0.000204452	0.01	120	0.67	8058	0.1	134	8.35	3568	0.13	108	7.71	4750

DAZAP2	-1.02	0.617773368	0.01	132	0.67	8059	0.03	121	2.44	6861	-0.05	133	-4.53	10851
ZDHHC18	-1.09	7.82E-05	0.01	163	0.67	8060	-0.1	120	-7.24	12753	-0.12	123	-9.11	12626
RDH13	1.03	0.357978243	0.01	132	0.67	8061	-0.03	117	-2.04	9852	-0.01	120	-1.02	9213
CDC42BPA_t2	1.09	0.072728777	0.01	141	0.66	8062	-0.03	134	-2.83	10357	0.1	132	8.53	4410
MUM1	-1	0.928839418	0.01	123	0.66	8063	-0.09	118	-5.79	12085	-0.02	120	-1.27	9330
TRMT2B	1.06	0.00307408	0.01	134	0.66	8064	-0.03	127	-2.49	10143	0.05	128	4.01	6411
RHCE	1.04	0.398464477	0.01	121	0.66	8065	-0.04	104	-2.15	9929	0.03	109	1.8	7567
CCDC144NL	1.02	0.413817609	0.01	137	0.66	8066	-0.07	126	-5.64	12001	0.15	126	11.78	3274
PML_t3	1.03	0.428487053	0.01	135	0.66	8067	0.09	122	6.39	4568	-0.12	120	-8.7	12491
CENPB_t1	-1.1	0.00930189	0.01	100	0.66	8068	NA	NA	NA	NA	-0.07	100	-3.34	10331
SYN1_t1	1.02	0.047822705	0.01	125	0.66	8069	-0.03	129	-2.18	9949	0.02	122	1.46	7773
SP140L	1.04	0.018056795	0.01	106	0.66	8070	0.1	139	9.21	3182	-0.04	135	-2.98	10174
HNRNPH3	-1.1	0.002934692	0.01	130	0.66	8071	0.09	115	6.02	4769	-0.04	123	-3.11	10227
WDR74	1.13	5.30E-06	0.02	80	0.66	8072	0.03	97	1.3	7600	0.04	99	2.07	7413
CPEB2	1.02	0.196001111	0.01	127	0.66	8073	0.01	132	0.45	8176	0.1	147	9.44	4075
LYPD5	-1.11	0.000465381	0.01	154	0.65	8074	-0.06	132	-4.77	11518	-0.1	150	-9.86	12879
UAP1L1	-1.07	0.167038497	0.01	116	0.65	8075	0.04	126	2.87	6569	-0.18	120	-12.6	13727
PSMC5	1.19	8.87E-06	0.05	64	0.65	8076	0.01	90	0.57	8090	0.09	74	2.25	7321
GRB7	1.04	0.314238295	0.01	102	0.65	8077	-0.06	120	-4.08	11142	-0.06	111	-3.59	10431
NRBP1	1.02	0.525555352	0.01	135	0.65	8078	0.02	118	1.62	7412	-0.05	125	-3.9	10587
ETV3L	1.01	0.257714587	0.01	104	0.65	8079	0.04	136	3.71	6052	-0.04	116	-2.6	9998
GPR12	-1	0.913331715	0.01	145	0.65	8080	-0.13	135	-10.63	14113	-0.08	139	-6.81	11775
FAM3B	1.15	0.451358161	0.01	124	0.65	8081	0.07	106	3.78	6018	-0.07	114	-4.25	10736
LRRC8E	1.07	0.236624319	0.01	115	0.64	8082	0.06	147	5.72	4940	-0.02	102	-0.88	9130
RGPD6	-1.03	0.054215454	0.02	87	0.64	8083	0.08	83	2.51	6816	-0.01	72	-0.2	8730
KCNH2_t2	1.05	0.011156615	0.01	110	0.64	8084	-0.11	130	-8.59	13359	0	118	-0.15	8699
NPPB	1.03	0.365546586	0.01	140	0.64	8085	-0.05	92	-1.98	9806	-0.02	99	-1.03	9220
AHR	-1.13	0.089631145	0.01	144	0.64	8086	-0.01	131	-0.48	8805	0.01	127	0.46	8334
SLC7A9	1.1	0.106767478	0.01	114	0.64	8087	-0.06	105	-3.13	10562	0.05	84	1.66	7644
GAS5	1.35	4.09E-05	0.01	131	0.63	8088	0.19	115	12.18	2069	0.03	119	2.16	7362
CRY1	-1.06	0.181530794	0.01	126	0.63	8089	0.06	116	3.79	6013	0.03	159	3.78	6534
AHNAK_t1	1.03	0.022561385	0.01	106	0.63	8090	0.02	115	1.14	7714	0	124	0.34	8407
NPY	1.03	0.017895975	0.01	100	0.63	8091	-0.08	97	-3.86	11009	0.03	114	1.72	7621
RPS3A_t1	-1.08	0.175183944	0.01	108	0.63	8092	NA	NA	NA	NA	-0.03	94	-1.46	9429
MC5R	1.06	1.72E-05	0.01	112	0.63	8093	-0.08	92	-3.48	10786	0	96	-0.1	8678
CAPZA3	1.02	0.226112779	0.02	76	0.63	8094	-0.09	88	-3.41	10738	0.07	96	3.03	6913
HOPX_t1	1.03	0.465153163	0.01	119	0.63	8095	NA	NA	NA	NA	-0.08	127	-6.47	11627
PNKD_t1	1.06	0.089732935	0.02	90	0.62	8096	0	117	0.02	8455	0.06	84	1.93	7493
TUBB4A_t1	1.03	0.047860663	0.01	124	0.62	8097	NA	NA	NA	NA	-0.07	136	-6.08	11472
FASN	1.31	0.000390848	0.01	115	0.62	8098	0.08	120	5.81	4891	0.06	132	5.21	5825
PITRM1	1.06	0.072097301	0.01	123	0.62	8099	-0.03	105	-1.74	9644	0.04	148	4.08	6380
TEX13A	1.01	0.149690502	0.1	56	0.62	8100	-0.01	56	-0.08	8540	0.08	80	2.33	7270
GNAQ	1.01	0.309619497	0.01	116	0.62	8101	0.04	118	2.6	6760	0.04	127	3.34	6750
ANKFN1	1.3	9.75E-05	0.02	88	0.61	8102	0	86	0.08	8409	0.09	79	2.7	7086
VCP	1.02	0.57999196	0.01	125	0.61	8103	-0.06	122	-4.26	11247	0	137	-0.11	8683
ZNF645	1.04	0.000451951	0.01	110	0.61	8104	-0.03	123	-2.23	9987	0.02	107	0.98	8031
PKP1	1.02	0.094370208	0.01	131	0.61	8105	-0.1	150	-9.54	13711	0.01	128	0.79	8135
S100A11	-1.07	0.416111368	0.02	88	0.61	8106	0.17	111	10.67	2609	-0.17	88	-6.61	11697
HSFX1	1.02	0.281146422	0.02	88	0.61	8107	-0.14	72	-3.14	10565	-0.02	88	-0.74	9054
TBC1D4	-1.13	0.0137234	0.01	152	0.61	8108	0.04	130	3.58	6125	-0.13	135	-11.22	13311
TRAPPC6A	1.03	0.425339978	0.02	88	0.6	8109	-0.13	103	-6.75	12537	-0.05	104	-2.48	9940
ATP5S_t2	-1.04	0.332022796	0.01	120	0.6	8110	-0.08	127	-6.38	12363	-0.06	128	-4.6	10894
SRR	-1.03	0.218268876	0.01	130	0.6	8111	-0.19	126	-14.41	15082	-0.01	144	-0.53	8943
TRIM8	1.06	0.184320239	0.01	101	0.6	8112	-0.01	118	-0.52	8828	0.03	114	1.78	7582
PDZD3	1.01	0.721435135	0.01	111	0.59	8113	0.09	124	6.53	4496	-0.09	140	-7.68	12106
CTSF	-1.09	0.209692161	0.01	115	0.59	8114	-0.16	104	-8.65	13373	0.07	100	3.36	6736
KXD1	1.09	0.004570864	0.01	117	0.59	8115	0.25	104	13.48	1691	0.05	109	2.76	7047
PLEKHA7_t1	-1.02	0.185381287	0.01	126	0.59	8116	NA	NA	NA	NA	NA	NA	NA	NA
OR6A2	1.03	0.000410892	0.01	117	0.59	8117	0.07	94	2.86	6576	0.03	103	1.35	7831
MUTYH_t2	1.05	6.46E-06	0.01	91	0.59	8118	-0.05	104	-2.6	10219	0.07	102	3.39	6724
FAM170A	1.07	1.68E-09	0.01	114	0.59	8119	0	104	-0.15	8577	0.07	107	3.97	6425
PLCB4	1.05	0.246613371	0.01	127	0.59	8120	0.11	131	8.66	3433	0.05	143	4.98	5935
CCDC116	1.03	0.001493848	0.01	101	0.59	8121	0.09	120	6.41	4561	0.11	104	6.03	5436
SMNDC1	-1.1	0.028707584	0.01	120	0.58	8122	0.02	113	1.2	7677	-0.1	132	-8.41	12403
XAB2	1.04	0.026710235	0.02	80	0.58	8123	-0.02	88	-0.87	9053	0.02	106	1	8021
NRXN3_t1	1.1	0.111314168	0.01	154	0.58	8124	NA	NA	NA	NA	-0.05	137	-4.51	10841
CXXC11	1.05	7.04E-05	0.04	64	0.58	8125	-0.05	94	-2.08	9883	0.01	60	0.06	8578
DCAF12	1.03	0.092559547	0.01	140	0.58	8126	-0.03	124	-2	9822	-0.03	125	-1.95	9694
KLK6	1.02	0.027281988	0.01	106	0.58	8127	-0.05	113	-2.85	10383	0.03	120	2.1	7398
FAR1	-1.02	0.250342238	0.01	143	0.58	8128	0.14	139	12.45	1992	-0.02	138	-1.7	9549
PYDC1	1	0.986674526	0.01	97	0.58	8129	0.03	104	1.63	7406	-0.06	126	-4.73	10949
NPHS1	1.02	0.40466395	0.01	137	0.58	8130	-0.14	149	-13.92	14977	0.02	147	1.64	7653
SETD9	-1.08	0.066432121	0.01	120	0.57	8131	-0.02	116	-1.23	9313	-0.02	111	-1.46	9425
OPRK1	1.01	0.409103009	0.01	130	0.57	8132	-0.01	142	-1.16	9258	0.07	144	6.25	5338
SOX5	-1.01	0.716036365	0.01	121	0.57	8133	-0.04	128	-3.07	10515	0.05	158	5.61	5615
CHST8_t1	1.01	0.839939964	0.01	113	0.57	8134	NA	NA	NA	NA	NA	NA	NA	NA
KLHL10	1.05	8.78E-08	0.03	68	0.57	8135	-0.09	72	-2.03	9845	0.06	79	1.62	7674
LYG1	1.11	0.004705808	0.01	88	0.57	8136	0.13	107	7.67	3898	0.05	101	2.74	7061
GFM2_t2	1.03	0.481577993	0.01	98	0.57	8137	-0.08	97	-3.66	10910	0.18	115	11.59	3325
YIPF2	-1.02	0.441212732	0.01	110	0.57	8138	-0.05	108	-2.74	10305	-0.03	100	-1.26	9323
H3F3B_t1	-1.01	0.888397943	0.01	125	0.56	8139	NA	NA	NA	NA	-0.09	140	-7.68	12104
ZNF140	-1.03	0.283705423	0.01	126	0.56	8140	0.08	131	6.49	4522	-0.08	126	-6.07	11465
CD80_t1	-1.03	0.03217187	0.01	127	0.56	8141	-0.01	128	-0.49	8813	NA	NA	NA	NA
ZNF565	1.12	0.00034636	0.01	100	0.56	8142	0.1	106	5.34	5149	0.13	84	4.48	6193
IL12RB1_t1	1.01	0.221426572	0.02	80	0.56	8143	-0.05	83	-1.72	9633	-0.12	66	-1.89	9666
ZNF518B_t1	-1.04	0.116835664	0.01	134	0.56	8144	NA	NA	NA	NA	0.05	150	4.66	6097
CCDC80	1.04	0.267196798	0.01	121	0.55	8145	0.08	135	6.78	4362	-0.14	122	-10.29	13023
PDX1	1	0.822438333	0.01	123	0.55	8146	-0.03	131	-2.48	10140	0.04	144	3.97	6429
TOR1AIP1	-1.01	0.713200969	0.01	136	0.54	8147	-0.05	129	-4.15	11179	0.04	128	2.99	6933
PLXNB3	1	0.967096243	0.01	118	0.54	8148	-0.06	97	-2.73	10291	-0.01	131	-0.94	9160
ALKBH2	1.46	5.44E-12	0.04	64	0.54	8149	0.12	93	5.22	5205	0.2	74	4.69	6074
NAPRT1	-1.05	0.462736158	0.01	89	0.54	8150	-0.15	104	-8.32	13249	0.08	80	2.38	7240
HR	-1.01	0.543265555	0.01	113	0.54	8151	-0.15	140	-13.23	14810	-0.12	105	-6.5	11644
ZFAND2B	1.05	0.107154973	0.01	98	0.54	8152	-0.05	109	-2.74	10307	0.01	91	0.59	8264
FCRL1_t1	-1.02	0.050826954	0.01	127	0.54	8153	NA	NA	NA	NA	-0.02	130	-1.24	9314
CDK13_t2	1.06	4.55E-07	0.01	137	0.53	8154	0.03	143	2.73	6653	0.2	130	15.62	2174
ZNF324	1.09	0.000555872	0.01	126	0.53	8155	-0.05	130	-3.86	11011	0	129	0.34	8406
API5	1.05	0.026967988	0.01	134	0.53	8156	-0.05	131	-4.38	11304	0.12	146	11.84	3253
ZFAND2A	1.25	1.53E-05	0.01	97	0.53	8157	0.07	130	5.51	5056	0.17	111	10.58	3661
MOV10	-1.01	0.824294374	0.01	100	0.52	8158	-0.11	104	-6.11	12247	0.02	100	1.1	7970
ACTC1	1.05	0.211820504	0.01	134	0.52	8159	0.1	124	7.41	4029	-0.08	132	-6.35	11592
LYPLA2	-1.02	0.301113229	0.01	102	0.52	8160	-0.03	119	-1.98	9803	-0.07	103	-3.84	10551
DYNC1I2	1	0.982924882	0.01	132	0.52	8161	0.19	126	14.37	1433	0.02	136	1.75	7595
CHMP1B	-1.04	0.277703992	0.01	141	0.52	8162	-0.02	138	-2.03	9844	-0.14	138	-12.62	13740
ZBED3	1.25	0.000409119	0.01	104	0.52	8163	-0.03	121	-1.84	9718	0.1	103	5.23	5819
ZNF781	1.09	2.08E-06	0.01	133	0.52	8164	0.19	127	14.81	1326	0.16	123	11.58	3330

SPANXD	1.08	0.013884733	0.01	88	0.51	8165	-0.03	91	-1.21	9303	0.04	94	1.68	7635
ACAT2	-1.06	0.42372998	0.01	120	0.51	8166	-0.1	117	-6.81	12568	-0.04	134	-2.97	10170
BAI1	-1.01	0.666808305	0.01	108	0.51	8167	-0.13	127	-9.75	13796	-0.02	100	-1.25	9317
SMC5	-1.15	0.000908508	0.01	131	0.51	8168	0.01	119	0.97	7824	0.04	119	3.02	6916
TSTD2	1.01	0.275487329	0.01	135	0.51	8169	-0.01	138	-1.03	9165	0	129	-0.26	8771
CDH9	1.09	0.00067684	0.01	128	0.51	8170	-0.1	117	-6.41	12374	0.07	121	5.13	5862
CELF6	1.33	5.06E-06	0.01	112	0.5	8171	0.04	121	3.09	6444	0.11	136	9.03	4217
CXorf1	1.02	0.031882262	0.01	127	0.5	8172	0.01	144	0.71	7994	0.07	125	5.08	5889
CPA1	1.06	0.060530529	0.01	94	0.5	8173	0.09	118	6.4	4565	-0.06	103	-3.28	10300
KRTAP4-7	1.01	0.506413172	0.01	118	0.5	8174	-0.04	126	-2.82	10354	0.02	133	1.56	7709
RNASEL	-1.08	0.203128699	0.01	121	0.5	8175	0.08	120	5.92	4832	-0.03	150	-3.22	10276
CARD8	1	0.997087306	0.01	135	0.5	8176	0.01	150	0.57	8093	0.03	126	1.95	7479.5
DNMT3A_t2	-1.08	0.01026893	0.01	117	0.5	8177	0.05	117	3.18	6395	-0.19	116	-12.68	13756
CSNK2A2	1.04	0.287234558	0.01	102	0.5	8178	-0.06	118	-3.75	10953	0.01	103	0.61	8250
MAP3K4	-1	0.87629557	0.01	109	0.5	8179	0.04	111	2.32	6945	-0.05	126	-3.51	10396
EEF1A2	1.82	1.74E-08	0.01	88	0.49	8180	0	126	0.28	8276	0	100	0.07	8571
ASAH1	-1.24	0.000406617	0.01	120	0.49	8181	-0.01	147	-0.83	9035	-0.1	128	-7.56	12053
PHACTR4	1.03	0.364814978	0.01	142	0.49	8182	-0.02	135	-1.74	9638	0.05	156	5.57	5636
PDILT	-1	0.722185171	0.01	102	0.49	8183	0	92	-0.19	8608	0.01	97	0.67	8217
SCN2B	1.03	0.009237698	0.01	116	0.49	8184	0.09	139	8.06	3710	0.01	126	1.06	7984
ALG2	-1.02	0.550869343	0.01	127	0.49	8185	-0.01	128	-0.86	9051	-0.03	133	-2.77	10070
ADAMTS8	1.02	0.038114423	0.01	100	0.49	8186	-0.01	120	-1.05	9178	0	125	-0.14	8690
NTN5	1.07	0.004224087	0.01	104	0.49	8187	0	113	-0.31	8692	0.05	122	3.91	6461
EEF1G_t1	1.11	0.00553583	0.01	84	0.49	8188	NA	NA	NA	NA	0.02	100	1.18	7929
SIGLEC6	1.07	9.17E-08	0.01	112	0.49	8189	0.13	112	8.03	3732	0.08	111	4.97	5941
PDE4C	1.12	7.38E-08	0.01	147	0.49	8190	-0.02	137	-1.66	9596	0.07	153	7.58	4803
HIF1A	-1.09	0.173826546	0.01	121	0.48	8191	0.04	114	2.44	6866	-0.02	147	-2.42	9903
TRIT1	1.03	0.384417235	0.01	120	0.48	8192	0.1	112	6.33	4604	0.03	107	1.74	7605
WDR52	1.04	0.02841643	0.01	120	0.48	8193	0.01	114	0.55	8106	0.07	117	4.98	5939
WDR63	1.05	0.04603497	0.01	99	0.48	8194	-0.03	92	-1.1	9218	0.07	107	3.88	6484
SERPINA12	-1.06	0.390800551	0.01	104	0.48	8195	-0.11	84	-3.9	11040	0.09	80	2.78	7039
SLC35F1	1.02	0.058508397	0.01	129	0.48	8196	-0.03	133	-2.4	10086	0.01	150	1	8019
FUT1	1.08	0.26809779	0.01	116	0.48	8197	0.02	110	1.15	7707	-0.05	120	-3.41	10359
SPANXB2	1.04	0.096541503	0.01	88	0.47	8198	-0.04	107	-2.31	10031	0.01	69	0.24	8470
FLNC	1.08	0.073581898	0.01	116	0.47	8199	0.13	135	10.86	2527	-0.04	138	-3.96	10619
EEF1A1	1.06	0.267026718	0	145	0.47	8200	0.1	131	7.94	3778	-0.03	121	-1.97	9700
TYMP	-1.04	0.618330044	0.03	68	0.47	8201	-0.02	84	-0.56	8855	-0.05	82	-1.65	9521
DNHD1_t1	1.04	0.000801664	0.01	120	0.47	8202	0	138	-0.3	8683	-0.02	133	-1.56	9475
FZD9	1	0.964301692	0.01	107	0.46	8203	-0.01	122	-0.95	9109	-0.02	127	-1.61	9507
BBS2	-1	0.984589281	0.01	128	0.46	8204	0.03	106	1.41	7550	-0.03	134	-2.67	10027
FAM100B	-1.06	0.27975136	0.01	120	0.46	8205	0.07	120	4.77	5465	-0.15	121	-10.8	13182
ZNF337	1.12	3.51E-05	0.01	127	0.46	8206	0.03	140	2.58	6771	0.1	140	8.74	4322
HIST4H4	-1	0.901749428	0.03	65	0.46	8207	-0.09	121	-6.37	12361	0	70	0.03	8601
UTY_t1	-1.03	0.042951669	0.01	108	0.45	8208	-0.06	98	-2.81	10344	-0.03	95	-1.54	9467
FAM103A1	1.24	5.67E-07	0.01	136	0.45	8209	0.18	130	14.39	1426	0.2	139	18.09	1626
KLK15_t1	1.02	0.08801182	0.01	104	0.45	8210	-0.03	113	-1.98	9807	0.04	115	2.53	7168
GCM2	1.03	0.000180462	0.01	102	0.45	8211	0.02	110	1.38	7561	0.05	146	4.97	5940
ASPSCR1	1.27	1.90E-06	0.01	100	0.44	8212	-0.03	120	-2.3	10019	0.09	93	3.8	6527
IL18BP_t1	-1.02	0.692811268	0.01	108	0.44	8213	0.07	139	6.56	4474	NA	NA	NA	NA
ZP2	1.02	0.030363731	0.03	64	0.44	8214	-0.09	72	-1.89	9745	0.05	72	1	8022
RPS6	1.09	0.098004643	0.01	107	0.43	8215	0.02	103	0.91	7866	0.06	61	0.62	8243
DOCK2	-1.02	0.065617828	0.01	114	0.43	8216	-0.01	110	-0.69	8934	-0.09	137	-7.62	12083
PDE1C	1.02	0.126687426	0.01	121	0.43	8217	0.05	126	3.81	5998	0.06	145	5.84	5525
HEATR7B2	1.15	0.011059898	0.02	74	0.43	8218	0.06	77	1.64	7398	0.09	67	1.53	7731
SCGB1C1	1.01	0.449412094	0.02	72	0.43	8219	-0.01	99	-0.4	8748	0.05	80	1.4	7805
TFIP11	1.18	2.02E-06	0.01	100	0.43	8220	-0.03	90	-1.35	9396	0.07	107	3.87	6485
ZIM3	-1	0.797826036	0	136	0.43	8221	-0.12	143	-11.2	14299	0	156	-0.09	8671
PLA2G2D_t1	-1	0.90530775	0.01	121	0.42	8222	NA	NA	NA	NA	-0.04	129	-3.34	10327.5
CXorf22	1.04	0.000181812	0	144	0.42	8223	-0.01	119	-1.02	9160	0.01	110	0.78	8150
SRSF1_t1	1.02	0.595795542	0.01	125	0.42	8224	NA	NA	NA	NA	0.08	138	7	5035
C1QL1	1.07	0.041637807	0.01	109	0.42	8225	-0.12	104	-6.21	12292	0.03	114	2.22	7342
RNASE3	-1.01	0.711148684	0.01	106	0.42	8226	0.06	118	3.8	6004	-0.04	106	-2.04	9727
SLC35G3	-1.02	0.114444219	0.01	130	0.41	8227	-0.07	112	-4.59	11434	0.03	113	1.83	7544
DST_t1	1.02	0.496992942	0.01	129	0.41	8228	0	120	0.33	8254	0.07	151	7.36	4889
NTNG1	1.04	0.000301638	0.01	113	0.41	8229	0.01	122	0.39	8217	0	131	-0.36	8824
CD276	-1.01	0.704735448	0.01	111	0.41	8230	0.01	119	0.86	7895	0	120	-0.01	8628
SURF2	1.08	0.008573897	0.02	68	0.41	8231	0	97	-0.03	8510	0.02	80	0.5	8318
N6AMT2	-1.06	0.1487756	0.01	97	0.4	8232	-0.15	148	-14.7	15146	-0.08	111	-5.12	11091
KRT36	1.01	0.199304389	0.01	104	0.4	8233	0.01	140	0.83	7912	-0.01	99	-0.26	8772
SIRPD_t1	-1.01	0.367546659	0.01	104	0.4	8234	-0.02	113	-1.03	9164	NA	NA	NA	NA
SLC16A6_t1	-1.02	0.424525788	0	153	0.4	8235	NA	NA	NA	NA	-0.06	128	-4.54	10865
RP1	1	0.643987532	0.01	119	0.4	8236	-0.08	135	-7.06	12675	-0.09	123	-6.54	11663
IGSF11_t1	1.03	0.00025866	0	136	0.4	8237	NA	NA	NA	NA	0.07	139	6.49	5242
TNFRSF10A	1.01	0.899484291	0	133	0.4	8238	0.1	93	4.16	5802	-0.01	100	-0.58	8967
MTNR1A	1.01	0.08761281	0.04	60	0.4	8239	-0.05	113	-3.22	10615	0.04	80	1.27	7867
ASIC3	1.09	4.70E-05	0.04	60	0.39	8240	NA	NA	NA	NA	0.13	84	4.31	6284
LZIC	1.17	0.002551563	0.01	124	0.39	8241	-0.01	138	-1.25	9327	0.19	116	12.31	3106
TP53TG5	1.03	0.000742602	0.04	60	0.39	8242	-0.1	76	-2.58	10201	0	103	-0.24	8757
TAS2R43	1.03	0.011727548	0.07	56	0.39	8243	-0.01	64	-0.12	8564	0.06	68	1.01	8015
CCDC47	1.15	0.055100097	0	134	0.39	8244	0.04	106	2.39	6891	0.18	134	14.88	2365
FAM96B	-1.01	0.882853925	0.01	127	0.39	8245	-0.13	102	-7.02	12663	-0.05	104	-2.83	10100
CLDND2	1.05	0.154919617	0.01	77	0.39	8246	-0.09	59	-0.82	9031	0.01	80	0.38	8392
ESX1	1.03	0.004303381	0.01	110	0.39	8247	-0.02	95	-0.93	9098	0.03	116	2	7451
ISG15	1.02	0.828879066	0.04	60	0.39	8248	-0.09	80	-2.81	10346	-0.04	86	-1.43	9408
TMEM95	1.04	0.001256123	0.01	101	0.39	8249	-0.06	126	-4.79	11532	0.07	120	4.83	6014
LCMT2	1.07	0.035708852	0	133	0.39	8250	-0.06	120	-3.89	11033	0.04	127	3.08	6889
PADI2	1.01	0.17281491	0	130	0.38	8251	-0.03	123	-2.2	9966	0.06	124	4.54	6158
MFSD4	1.01	0.528068064	0.01	114	0.38	8252	-0.1	123	-7.24	12754	0.03	123	2.51	7178
RYK	-1.04	0.125031591	0	140	0.38	8253	0.06	121	4.2	5778	-0.08	143	-7.71	12119.5
PRRX1	1.01	0.521058293	0	135	0.37	8254	0.06	130	4.55	5586	0.19	137	16.62	1926
COMMD1_t1	1	0.924318459	0.01	98	0.37	8255	NA	NA	NA	NA	-0.05	103	-2.47	9932
CYP7A1	1.02	0.9181168	0	126	0.37	8256	-0.17	123	-12.42	14619	0.09	126	6.56	5208
RP2	-1.04	0.001990235	0	145	0.37	8257	0.02	151	2.13	7058	-0.05	138	-4.43	10817
SYTL4	-1.13	0.06468384	0	138	0.37	8258	-0.1	114	-6.36	12357	-0.02	121	-1.45	9423
MTMR10	-1	0.987118292	0	136	0.37	8259	-0.01	152	-0.68	8919	0.06	147	6.21	5351
MAP3K11	1.03	0.271152274	0.01	107	0.37	8260	0.03	120	2.23	6994	-0.02	103	-1	9205
STXBP1_t2	1.05	0.002654685	0	138	0.36	8261	-0.05	119	-3.62	10894	0.02	143	2.1	7397
ZNF649	-1.04	0.117660117	0	139	0.36	8262	0.13	115	8.62	3447	-0.03	124	-1.86	9641
SERP1	-1.05	0.37179888	0	149	0.36	8263	0.03	145	2.55	6797	-0.03	151	-2.75	10063
GPR110_t1	-1.02	0.078671065	0.01	120	0.36	8264	-0.04	139	-3.64	10904	-0.05	129	-3.96	10621
MAP1S	1.02	0.293793677	0.01	104	0.36	8265	0.01	125	0.69	8014	-0.05	88	-1.82	9623
ANKRD9	-1	0.927617153	0.01	88	0.36	8266	-0.16	105	-8.85	13453	0.02	120	1.18	7924
EPB42	1.01	0.606130195	0.01	80	0.36	8267	-0.07	128	-5.32	11832	-0.03	102	-1.77	9598
BMP15	1.04	0.000372232	0.02	72	0.36	8268	-0.1	76	-2.51	10160	0.14	64	1.95	7478
SGSH	1.01	0.805300413	0.01	115	0.35	8269	0.14	161	15.94	1063	-0.1	110	-6.21	11527
KIAA0040_t1	-1.04	0.080382233	0	134	0.35	8270	0.04	140	3.63	6095	NA	NA	NA	NA

BPIFA1	1.05	0.000406044	0.01	108	0.35	8271	-0.03	106	-1.7	9614	0.01	102	0.58	8271
CLCA2	1.06	0.018131628	0.01	120	0.35	8272	0.04	120	2.47	6846	0.05	113	3.19	6838
POFUT2	1.03	0.005836686	0.01	110	0.35	8273	0.1	120	6.68	4411	0.04	154	4.15	6350
FLJ43860	1.03	0.032538978	0.01	104	0.35	8274	-0.08	103	-4.46	11368	0.02	105	1.18	7926
GRIK1	-1.02	0.037960466	0.06	56	0.35	8275	-0.02	56	-0.1	8554	NA	NA	NA	NA
RABGAP1L_t1	1.07	0.016729273	0.01	104	0.35	8276	NA	NA	NA	NA	0.1	127	7.33	4901
NOS1AP	1.02	0.638258891	0	135	0.35	8277	-0.1	119	-7.09	12686	0.03	126	2.55	7157
SELV	1.03	0.006973783	0.01	95	0.34	8278	-0.04	148	-3.77	10961	0	101	-0.14	8695
CSDE1_t1	-1.06	0.235288649	0	125	0.34	8279	NA	NA	NA	NA	0.04	132	3.17	6850
ABCA12	1.02	0.158748266	0	123	0.34	8280	0.03	134	2.17	7031	0.17	115	11.08	3487
KCTD16	1.04	0.008920573	0	142	0.34	8281	0.07	134	5.75	4922	0.01	152	0.54	8297
KCNN1	-1.01	0.480601723	0.01	92	0.34	8282	-0.09	111	-5.58	11963	0.06	127	4.53	6161
AUP1	1.06	0.064580534	0.02	72	0.34	8283	-0.03	118	-2.32	10036	0.07	96	3.32	6759
PIGA_t1	-1.03	0.51797629	0	138	0.34	8284	NA	NA	NA	NA	0.03	165	3.75	6548
PRM2	1.03	0.005822582	0.01	108	0.34	8285	-0.03	122	-2.34	10049	0.01	88	0.31	8432
SAA2	-1.78	0.001073354	0.01	94	0.34	8286	-0.15	104	-7.93	13087	-0.13	103	-6.71	11741
LHX4	1.02	0.207902807	0.01	104	0.34	8287	-0.04	117	-2.9	10411	0.03	111	1.77	7587
DBX2	1.01	0.320371831	0	140	0.33	8288	-0.01	123	-0.83	9036	0.01	155	0.65	8230
AP2S1	-1.05	0.113463059	0.01	88	0.33	8289	-0.06	93	-2.71	10283	-0.08	100	-4.06	10661
HPS1_t2	1.04	0.211280766	0.01	113	0.33	8290	0.08	127	6.37	4576	-0.09	111	-5.53	11249
IL4I1	1.21	0.015037092	0.01	86	0.33	8291	0.1	60	0.98	7818	-0.06	68	-1.02	9215
KIAA1755	1.03	0.211242267	0	141	0.33	8292	0	136	0.41	8200	0.03	136	2.95	6963
FRS2	1.07	6.64E-08	0	147	0.33	8293	-0.04	127	-3.46	10777	0.06	138	5.38	5742
ELP5	-1.12	0.00337862	0.01	106	0.33	8294	-0.1	108	-5.77	12073	0.01	106	0.72	8182
ZNF214	1.05	0.102823143	0	120	0.33	8295	-0.07	114	-4.56	11415	0.04	124	3.13	6860
KIF21B	1.04	0.19595621	0	123	0.33	8296	0.06	144	5.5	5060	-0.07	128	-5.15	11096
BAZ2A	1.03	0.270193896	0	137	0.33	8297	0.05	134	4.42	5654	-0.06	141	-5.8	11363
ZNF471	1.03	0.118446984	0	146	0.32	8298	0.05	134	4.33	5696	0.05	126	4.14	6356
LEO1	1.08	0.208647508	0.01	105	0.32	8299	0	95	-0.21	8616	0.16	112	10.16	3809
ZP4	1.01	0.269838519	0	118	0.32	8300	-0.07	138	-6.48	12405	0.07	114	4.53	6164
TBC1D3F	-1.09	0.072715535	0	121	0.32	8301	0.06	114	3.8	6006	-0.11	129	-8.86	12548
SVOPL	1.04	0.015754909	0	116	0.32	8302	0.05	98	2.37	6909	0.01	104	0.72	8181
TBC1D17	1.08	0.001383858	0.01	88	0.32	8303	-0.09	134	-7.61	12933	-0.03	100	-1.69	9548
TMA7	1.02	0.640703362	0.01	103	0.32	8304	0.1	107	5.42	5110	0.08	122	5.77	5551
KCNC2_t1	1.02	0.035989471	0	142	0.32	8305	0.02	163	2.28	6972	0.06	168	7.36	4893
NUDT16	1	0.923641585	0	132	0.32	8306	-0.1	157	-10.36	14024	-0.03	166	-3.02	10191
ABHD14A	1.07	0.034996492	0.01	104	0.32	8307	-0.03	120	-2.27	10007	0.03	94	1.21	7905
SF3B5	1.12	3.85E-05	0	124	0.32	8308	0.11	101	5.66	4971	-0.04	104	-2.06	9742
SAA1	-1.89	0.000695798	0.01	113	0.32	8309	-0.14	104	-7.45	12850	-0.13	103	-7.07	11873
FRMPD2	1.06	0.016752444	0	130	0.31	8311	-0.02	126	-1.42	9445	0.03	139	3.04	6909
CDX2	-1.01	0.474842118	0	136	0.31	8312	-0.05	122	-3.34	10698	-0.01	114	-0.93	9155
EFR3A	-1.02	0.811733885	0	140	0.31	8313	-0.01	128	-0.6	8875	0.09	146	8.5	4425
INO80E	1.13	7.92E-06	0.01	99	0.31	8314	0.07	123	5.29	5171	0.05	100	2.31	7282
AMIGO2	-1.54	4.72E-08	0	134	0.3	8315	-0.03	126	-2.2	9964	-0.1	130	-8.29	12357
PRPF19	-1.01	0.652401685	0.01	100	0.3	8316	0.01	123	0.82	7919	-0.09	141	-8.07	12257
TRPC1	1.01	0.9209502	0	132	0.3	8317	0.13	118	9.14	3210	0.03	135	2.96	6955
IFI27L2	-1.02	0.812576888	0.02	68	0.3	8318	0.02	87	0.6	8070	-0.12	80	-3.69	10478
DOCK5	-1.05	0.119960479	0	134	0.3	8319	-0.13	145	-12.28	14579	-0.1	130	-8.11	12269
KLK1	1.03	0.012662908	0.02	68	0.3	8320	0	73	0.05	8431	0.05	84	1.69	7633
DTX1	-1.02	0.414076118	0	125	0.3	8321	-0.06	111	-3.91	11045	-0.13	115	-8.43	12410
OXSR1	1.04	0.089634341	0	117	0.29	8322	0.02	121	1.46	7516	0.08	133	6.37	5283
NOC4L	1.07	0.001230657	0.01	75	0.29	8323	-0.04	69	-0.75	8979	-0.04	60	-0.38	8842
TBP	1.04	0.083221447	0	116	0.29	8324	0.05	136	4.73	5491	0	126	-0.22	8750
RPL21	-1	0.999261464	0.01	79	0.29	8325	0.05	68	0.97	7821	-0.04	72	-0.83	9101
NCOA7_t1	-1.35	7.44E-05	0	137	0.29	8326	NA	NA	NA	NA	-0.07	132	-5.45	11220
CMA1	1.02	0.223474257	0.01	76	0.29	8327	0.05	60	0.51	8134	-0.03	52	-0.06	8656
ASAH2	1.06	0.005541953	0.01	88	0.29	8328	-0.11	108	-6.62	12476	0.17	84	5.87	5513
ARHGEF38	1.04	2.04E-05	0	116	0.29	8329	0.01	122	0.69	8013	0.13	127	9.67	3995
ELOVL4	1.01	0.604678012	0	128	0.29	8330	-0.06	144	-5.86	12118	-0.03	134	-2.41	9898
POLRMT_t1	1.11	0.001249767	0.01	80	0.28	8331	NA	NA	NA	NA	NA	NA	NA	NA
MTMR4_t1	1.09	0.00038988	0	142	0.28	8332	NA	NA	NA	NA	NA	NA	NA	NA
ASIP	1.03	0.0198719	0.01	88	0.28	8333	-0.11	120	-7.98	13106	-0.01	95	-0.25	8766
RAF1	1.08	0.013542685	0	129	0.28	8334	-0.12	135	-9.84	13826	0.1	138	8.97	4236
CBX5	1.01	0.755252849	0	136	0.28	8335	0.02	135	1.79	7281	-0.01	148	-1.35	9364
FOXN2	1.07	0.158044763	0	140	0.28	8336	0.04	140	3.84	5987	0.05	130	3.68	6576
ZNF175	-1.01	0.829916152	0	133	0.28	8337	0.02	129	1.29	7611	0.01	139	1.06	7985
IRS4	1.01	0.354440698	0.01	84	0.28	8338	-0.02	99	-0.78	9004	0.01	104	0.65	8227
CLRN1_t2	1.04	7.62E-05	0	109	0.27	8339	-0.01	117	-0.53	8840	0.09	120	6.12	5391
AVP	-1.18	0.000200838	0.03	60	0.27	8340	-0.08	111	-4.61	11446	-0.08	84	-2.67	10026
HEPH_t1	-1.11	3.24E-06	0	143	0.26	8341	NA	NA	NA	NA	-0.14	127	-10.58	13110
AKT2	-1.01	0.621586159	0	127	0.26	8342	-0.06	125	-4.58	11431	-0.07	128	-5.17	11106
ZNF397	1.03	0.297667566	0	110	0.26	8343	0.03	100	1.65	7388	0.05	100	2.42	7221
ZNF224	1.02	0.549714316	0.01	80	0.26	8344	0.09	102	4.61	5542	-0.03	91	-1.25	9316
SEC16B	-1.14	0.039460018	0	124	0.26	8345	-0.16	125	-11.82	14453	-0.04	114	-2.42	9904
KCNH1	1.05	2.13E-05	0	145	0.26	8346	-0.01	132	-0.53	8839	0.09	146	9.06	4203
LSM12_t1	1.09	0.006764056	0	150	0.26	8347	0.05	128	4.27	5736	NA	NA	NA	NA
DMRTC2	1.04	0.130236768	0	121	0.26	8348	-0.08	114	-5.05	11688	0.09	113	5.53	5668
CNPY4	1.08	0.027614091	0	131	0.25	8349	0.07	119	4.61	5551	0.04	136	3.64	6594
TEKT5	1.13	0.000184244	0.01	93	0.25	8350	0	104	0.11	8393	0.04	85	1.52	7739
PCGF5	-1.1	0.057204858	0	146	0.25	8351	-0.06	142	-5.59	11970	0.09	135	7.78	4718
ENKUR_t1	1.01	0.401940064	0	142	0.25	8352	NA	NA	NA	NA	NA	NA	NA	NA
EGLN2	1.11	0.001308263	0	118	0.25	8353	-0.02	123	-1.6	9556	0	116	-0.14	8693
FAM221B	1	0.721293062	0	126	0.25	8354	-0.01	130	-0.75	8977	0	128	0.09	8559
TMSB15B	1.01	0.886247116	0	112	0.25	8355	0.11	113	7.08	4207	-0.04	124	-2.96	10163
ALPK3	1.14	1.24E-05	0	131	0.25	8356	0.1	145	9.66	3000	0.01	150	0.73	8177
ZNF566	1.03	0.050782781	0	146	0.25	8357	0.07	134	6.3	4619	0.11	127	8.21	4533
DNASE1L1	1.06	0.063863282	0	134	0.25	8358	0.06	134	5.15	5253	-0.05	120	-3.84	10550
MOGAT3	1.09	0.002060557	0.01	96	0.25	8359	-0.08	112	-4.88	11584	0.05	100	2.51	7176
CD47_t1	1.09	0.092407168	0	143	0.24	8360	0.05	150	4.79	5453	-0.09	146	-8.17	12302
KCNS2	-1.01	0.392443837	0	125	0.24	8361	0.04	133	2.93	6528	-0.02	138	-1.34	9363
MEI1_t1	1.03	0.014154454	0	120	0.24	8362	NA	NA	NA	NA	0.01	88	0.48	8331
EDARADD	1.23	0.00011837	0	115	0.24	8363	0.09	119	6.43	4552	0.07	142	6.3	5320
EID3	-1.01	0.791599115	0	99	0.24	8364	0.11	96	4.98	5352	-0.08	100	-3.75	10515
C1QTNF3	1.79	3.52E-07	0	131	0.23	8365	0.04	133	3.47	6199	0.16	124	11.86	3246
PDLIM7_t1	1.02	0.58042761	0.01	87	0.23	8366	0.12	120	8.32	3584	-0.08	104	-4.49	10834
TCEB2_t1	-1.05	0.114629677	0.01	68	0.23	8367	-0.06	112	-3.8	10982	-0.04	70	-0.76	9059
CLASRP	1.07	0.012669554	0	98	0.23	8368	0.03	88	1.12	7732	0.01	68	0.1	8553
TAPBP_t3	1.05	0.01560752	0	124	0.23	8369	-0.04	134	-3.73	10949	-0.03	136	-2.28	9836
ZNF560	1.01	0.346089526	0.01	91	0.23	8370	0.14	111	8.47	3524	-0.02	91	-0.89	9132
T	1.02	0.333136188	0	116	0.23	8371	-0.11	129	-8.84	13447	-0.01	106	-0.45	8885
PPP1R3F	1.23	7.76E-08	0	146	0.23	8372	0.01	122	0.98	7817	0.18	110	10.66	3627
CDHR3	1.34	5.62E-09	0	131	0.23	8373	0.05	162	5.93	4825	0.23	139	20.12	1263
TDRD9	-1.04	0.42151809	0	110	0.23	8374	-0.04	126	-3.1	10545	-0.05	107	-3.06	10209
SCGN	1.46	0.00992845	0	104	0.23	8375	-0.01	120	-0.65	8908	0.11	118	7.19	4955
BAD	1.05	0.151332099	0	106	0.22	8376	0.01	112	0.56	8099	0.05	110	2.82	7027
CCNJ	-1.26	0.000550275	0	137	0.22	8377	0.05	125	3.98	5914	-0.01	143	-1.35	9365

MED18	1.19	3.49E-07	0	144	0.22	8378	-0.03	144	-3.09	10530	0.12	150	12.05	3178
COL1A2	1.03	0.484702086	0	152	0.22	8379	0.12	139	10.58	2641	-0.1	133	-7.98	12233
MTA2	1	0.857883547	0	113	0.22	8380	0.05	126	4.13	5824	0	120	0.05	8587
SGSM3	1.08	0.018883749	0	120	0.22	8381	0.1	131	8.2	3640	0.02	106	0.99	8027
CSN3	-1	0.891852652	0	110	0.21	8382	-0.14	146	-13.48	14865	0.06	135	5.39	5737
SPATA8	1.02	0.08762063	0	115	0.21	8383	-0.11	134	-8.97	13491	-0.02	104	-1.29	9338
MYF6	1.03	0.025920483	0	110	0.21	8384	-0.04	117	-2.55	10182	0.02	132	1.79	7574
MYF5	1.01	0.127913357	0	114	0.21	8385	0	109	0.09	8405	0.13	134	10.66	3629
TCF7L2	-1.03	0.17309976	0	143	0.21	8386	0.01	114	0.85	7898	0.02	141	2	7452
NRGN	-1.03	0.224299007	0	103	0.21	8387	-0.02	134	-2.09	9889	-0.11	135	-9.08	12616
TAF1L	1.02	0.168455064	0	136	0.21	8388	0.05	121	3.85	5980	0.01	112	0.92	8068
TRIM13	-1.19	0.001477115	0	148	0.2	8389	0.05	148	4.75	5472	-0.09	131	-6.93	11818
DISC1_t1	-1	0.95815472	0	120	0.2	8390	-0.01	98	-0.41	8755	-0.05	100	-2.4	9888
MAP2K2	1.16	4.85E-08	0.01	87	0.2	8391	0.03	113	1.58	7444.5	0.07	82	2.33	7272
USP4	-1.04	0.115838727	0	110	0.2	8392	0.11	149	11	2473	-0.11	121	-8.05	12251
KRT18	-1.03	0.721105819	0.01	68	0.2	8393	0.09	86	3.35	6286	-0.08	64	-1.1	9251
HHLA3_t2	1.03	0.17139338	0	100	0.2	8394	-0.07	134	-6.25	12306	0.02	113	1.18	7923
PELO	-1.16	0.002394684	0	145	0.19	8395	0.09	129	6.91	4293	-0.04	144	-3.71	10487
TBC1D8B	1.07	0.009153407	0	129	0.18	8396	0.04	147	3.59	6116	0.15	127	11.75	3283
PAPPA_t1	1.06	0.000912342	0	143	0.18	8397	NA	NA	NA	NA	0.08	136	6.81	5098
TUB	1.01	0.119886325	0	136	0.18	8398	0.1	146	9.58	3027	0.04	127	3.23	6814
ZNF362	1.06	0.079036319	0	120	0.18	8399	0.15	128	11.88	2161	-0.03	127	-2.16	9777
PARP9	-1.14	0.003050186	0	124	0.17	8400	-0.19	127	-14.43	15087	-0.13	119	-8.7	12490
KRTAP1-3	1.01	0.221330734	0	120	0.17	8401	0.06	125	4.36	5687	0.13	132	10.61	3649
PRY2	1.04	0.006285012	0	126	0.17	8402	-0.08	116	-5.17	11762	0.02	107	1.24	7886
ZMAT5	1.02	0.581295603	0	85	0.17	8403	-0.04	120	-2.54	10174	0.02	100	1.19	7919
SOX7_t1	1.02	0.243696585	0	131	0.17	8404	NA	NA	NA	NA	NA	NA	NA	NA
MRPL27	1.03	0.281571766	0	128	0.17	8405	-0.06	94	-2.78	10331	0.02	83	0.8	8130
KCTD4	1	0.962327866	0	131	0.16	8406	0.04	112	2.43	6873	-0.06	126	-4.63	10907
CACHD1	1.03	0.166327302	0	128	0.16	8407	0.01	135	0.87	7888	0	127	-0.21	8739
ATP13A1	1.12	0.000293443	0	92	0.16	8408	0.12	106	6.83	4336	-0.04	96	-1.66	9533
TNFAIP3	-1.01	0.854144166	0	131	0.16	8409	0.05	138	4.28	5727	-0.07	136	-6.08	11466
ATAD3A	1.08	0.018540448	0	92	0.16	8410	0.02	132	1.7	7347	-0.06	124	-4.18	10707
LLGL2_t2	1.13	0.00082335	0	100	0.16	8411	0	113	-0.18	8603	0	146	-0.4	8850
EIF2AK2	-1.1	0.022165165	0	151	0.16	8412	-0.05	127	-3.72	10940	-0.01	125	-1.07	9229
PPM1J	-1.04	0.036377584	0	106	0.15	8413	-0.1	104	-5.54	11938	-0.09	92	-3.84	10552
VPREB1	1.03	0.006860721	0	104	0.15	8414	-0.04	112	-2.3	10024	-0.04	119	-2.64	10009
SHANK2_t1	1.05	8.72E-08	0	150	0.15	8415	-0.04	144	-3.33	10692	0.12	162	13.17	2837
TP53	1.03	0.112231383	0	120	0.15	8416	0.02	132	1.24	7650	-0.04	128	-3.33	10320
P2RY6	-1.07	0.082359351	0	111	0.15	8417	0.05	92	2.17	7029	-0.15	104	-7.94	12211
PWWP2B	1.02	0.659504377	0	120	0.15	8418	0.05	132	3.9	5951	-0.14	125	-10.42	13062
TMEM126A	1.1	0.019628591	0.01	62	0.15	8419	0.05	83	1.8	7269	0.14	69	2.64	7116
FEZF2	1.01	0.354087365	0	113	0.15	8420	0.04	116	2.72	6662	0.08	111	5.15	5855
NAALADL1	1.02	0.476100727	0	121	0.14	8421	0.03	104	1.62	7413	-0.05	100	-2.68	10029
CADM1_t1	-1.06	0.566578956	0	146	0.14	8422	NA	NA	NA	NA	NA	NA	NA	NA
SDR39U1	-1.03	0.31631813	0	98	0.14	8423	-0.17	109	-10.23	13972	0.02	123	1.3	7851
ENSA_t1	1.07	0.058876234	0	121	0.14	8424	-0.04	124	-3.14	10564	0.04	115	2.7	7088
SULT2B1	1.08	1.04E-05	0.05	53	0.14	8425	0.03	92	1.27	7627	0.09	60	0.94	8051
GULP1_t1	1.05	0.578293581	0	126	0.14	8426	NA	NA	NA	NA	NA	NA	NA	NA
BANP_t1	1.01	0.716300717	0	113	0.14	8427	-0.08	111	-5.18	11764	0.02	108	0.98	8029
ALKBH3	1	0.985201977	0	101	0.14	8428	-0.02	104	-0.97	9123	0.03	120	1.85	7535
TMC2	1	0.666738944	0	132	0.14	8429	-0.15	111	-9.03	13520	0.04	120	2.71	7082
SMAD4	-1.04	0.152878043	0	151	0.14	8430	-0.06	138	-5.09	11711	0.03	155	2.93	6973
JAG1	-1.02	0.788880974	0	149	0.14	8431	0.19	130	15.16	1230	-0.1	129	-8.24	12332
CRYGB	1.03	0.002532163	0	89	0.14	8432	0.01	90	0.54	8109	0.03	118	2.38	7244
SPATA16	1.02	0.057375087	0	120	0.13	8433	-0.03	131	-2.38	10066	-0.05	100	-2.25	9824
SOX1	-1	0.871546993	0	136	0.13	8434	-0.01	137	-1.19	9286	0.01	149	1.44	7788
ZNF582	-1	0.79392636	0	120	0.13	8435	0.06	134	5.4	5119	-0.01	120	-0.44	8878
MAPK8IP3	1.07	2.95E-07	0	126	0.13	8436	0	125	0.25	8293	0.02	121	1.63	7666
MEGF8	1.06	0.013850627	0	127	0.13	8437	-0.02	128	-1.57	9530	-0.06	126	-4.9	11016
LRIT2	1.01	0.497883048	0	123	0.13	8438	0.02	134	1.68	7371	-0.03	120	-2.14	9771
PSG2	1.07	7.90E-09	0	124	0.13	8439	0.01	127	1.06	7769	0.04	120	2.78	7041
RHOXF1	1.05	0.010050351	0	91	0.13	8440	0.04	93	1.62	7411	0.05	127	3.9	6470
TSR3	1.07	0.067928107	0	93	0.12	8441	0	129	0.3	8262	0.06	101	3.07	6894
USPL1_t1	-1.18	1.06E-06	0	131	0.12	8442	NA	NA	NA	NA	-0.09	102	-4.69	10934
PFDN6	1.18	0.00049084	0	91	0.12	8443	-0.01	95	-0.31	8690	0.12	72	2.56	7155
TDRD10	-1.09	0.255301967	0	106	0.12	8444	-0.05	110	-2.97	10459	-0.02	127	-1.72	9564
NUB1	1.01	0.744440237	0	113	0.11	8445	-0.02	130	-1.33	9380	-0.02	108	-1.11	9254
NAT10	1.08	2.49E-05	0	120	0.11	8446	0.05	120	3.28	6335	-0.02	126	-1.57	9487
CATSPERD	1.02	0.074659494	0	80	0.11	8447	0.04	82	1.23	7661	0.09	78	2.61	7128
PPCDC	1.05	0.177321427	0	120	0.1	8448	0.07	127	5.15	5252	0.03	120	1.87	7523
RBPMS2	1.19	0.003994899	0	120	0.1	8449	-0.01	122	-0.47	8798	0.22	127	16.72	1898
ETV5_t1	-1.04	0.360692865	0	123	0.1	8450	NA	NA	NA	NA	-0.05	159	-5.9	11408
ARR3	1.06	1.58E-05	0.01	68	0.1	8451	-0.03	150	-2.96	10455	0.02	83	0.56	8283
ABHD13	1.02	0.416901219	0	137	0.09	8452	0.12	136	10.33	2749	0.03	158	3.04	6907
GDF1	-1.06	0.022029677	0.01	59	0.09	8453	-0.11	68	-1.97	9792	-0.07	60	-0.72	9042
IGFBPL1	1.06	0.00734406	0	102	0.09	8454	0.05	107	2.8	6615	-0.01	112	-0.89	9133
PRSS37	1.04	0.000163131	0	100	0.09	8455	0.01	110	0.88	7882	0.01	128	0.44	8345
VSTM4_t1	1.06	5.27E-07	0	152	0.09	8456	NA	NA	NA	NA	0.1	120	6.78	5113
DNAAF1	1	0.953321283	0	87	0.09	8457	0.07	109	3.88	5958	-0.07	94	-3.09	10220
SUPT6H	1.03	0.12516234	0	122	0.09	8458	-0.04	121	-3.06	10511	-0.02	118	-1.28	9337
BEX5	-1.04	0.549425386	0	100	0.08	8459	0.01	107	0.33	8250	-0.09	139	-8.26	12344
TNS3	1.04	0.445821014	0	134	0.08	8460	0.15	133	12.14	2087	-0.02	131	-1.44	9413
TMPRSS3	1.47	1.80E-05	0	99	0.08	8461	0.08	132	6.8	4352	-0.05	99	-2.43	9909
TRIM47	1.13	0.014121322	0	108	0.08	8462	0.18	141	16.82	874	-0.01	94	-0.64	9006
DLK1	1.07	0.504450714	0	105	0.08	8463	0.06	111	3.92	5941	-0.06	120	-4.33	10770
DDX21	-1.15	0.034728788	0	144	0.08	8464	0.09	121	6.21	4657	0	126	-0.36	8826
PRMT6	-1.08	0.195214124	0	134	0.08	8465	0.05	124	3.67	6074	-0.02	147	-2.38	9883
CD164_t1	-1.09	0.000627337	0	137	0.07	8466	-0.04	130	-3.59	10870	NA	NA	NA	NA
TBR1	1.01	0.524275712	0	129	0.07	8467	-0.02	148	-1.65	9587	0.04	131	3.49	6674
GUCA1C	1.01	0.482642144	0	100	0.07	8468	-0.02	128	-1.95	9773	-0.05	136	-4.39	10796
ELOVL3	1.09	0.001386234	0	119	0.07	8469	-0.06	101	-2.81	10345	0.17	119	11.87	3245
PRR4	1.08	6.69E-05	0	84	0.07	8470	0.03	104	1.64	7397	0.07	97	3.06	6900
CAPS2	-1.01	0.689798997	0	137	0.07	8471	-0.06	97	-3.03	10486	-0.02	105	-0.84	9103
SLC30A5_t2	1.13	0.000572389	0	146	0.07	8472	NA	NA	NA	NA	0.21	120	14.96	2348
MRGPRX4	1.05	5.07E-06	0	84	0.07	8473	-0.02	100	-1.23	9317	0.03	88	1.18	7931
SLC35A3	-1.03	0.516465762	0	126	0.07	8474	-0.03	135	-2.75	10311	0.09	139	7.64	4775
GPX8	1.16	0.020560259	0	153	0.07	8475	0.12	135	10.35	2740	-0.02	132	-1.87	9652
DFFA_t1	1.07	0.020298404	0	136	0.06	8476	0.06	136	5.13	5259	0.05	120	3.53	6655
YBEY	1.08	0.122190954	0	93	0.06	8477	-0.14	99	-6.79	12559	0.07	89	2.9	6991
FAM71B_t1	1.01	0.125753219	0	104	0.06	8478	NA	NA	NA	NA	0.09	93	3.9	6465
SMOX	1.18	0.000218333	0	111	0.06	8479	0.13	108	7.6	3933	0.05	141	4.12	6362
CHAT	1.03	0.113734834	0	72	0.06	8480	-0.08	92	-3.34	10701	0.07	91	3.07	6895
RPUSD3	1.2	1.87E-07	0	114	0.05	8481	0.03	107	1.98	7159	0.07	101	3.79	6532
TULP1	1.04	0.000748533	0	92	0.05	8482	0.02	104	1.19	7680	0.09	99	4.36	6263
KCNJ10	1.06	0.000299585	0	139	0.05	8483	-0.04	128	-2.88	10396	0.02	161	2.68	7094

MB21D1	-1	0.802355979	0	104	0.05	8484	0.14	96	6.54	4488	-0.11	113	-7.24	11935
SPRR3	1.09	2.98E-06	0	114	0.05	8485	-0.04	135	-3.04	10496	0.01	116	0.95	8046
METTL22_t1	1	0.877230404	0	97	0.05	8486	NA	NA	NA	NA	0.04	95	1.91	7504
MAN1B1_t1	-1.06	0.046874767	0	104	0.04	8487	NA	NA	NA	NA	-0.04	116	-2.59	9993
INSIG2	1.02	0.718553871	0	136	0.04	8488	-0.01	127	-1.07	9199	0.05	127	3.92	6459
KCNJ1	1	0.756788726	0	144	0.04	8489	0.15	108	8.99	3277	0.01	127	1.05	7993
UCN	1.1	0.022128507	0	98	0.04	8490	-0.08	100	-4.01	11105	0	100	0.15	8523
STXBP2_t1	-1.01	0.703610106	0	63	0.04	8491	0.02	93	0.88	7886	NA	NA	NA	NA
FNDC9	1.02	0.041132258	0	137	0.04	8492	0	123	0.13	8380	0.03	116	2.02	7442
MRPL36	1.1	0.001180598	0	111	0.03	8493	-0.04	112	-2.46	10129	0.17	124	12.41	3064
OGFOD2	1.08	0.007063205	0	115	0.03	8494	0.04	113	2.57	6780	0	120	0.05	8581
PROL1	1.05	0.014076298	0	126	0.03	8495	-0.09	108	-5.44	11895	0.02	93	0.83	8118
TSSC4	1.06	0.033229716	0	80	0.03	8496	-0.1	109	-6	12184	-0.05	100	-2.73	10049
PLA2G3	1.01	0.451967694	0	117	0.03	8497	0	124	-0.3	8675	0.01	140	1.27	7868
PSIP1_t2	1.07	0.047694472	0	133	0.03	8498	-0.01	126	-0.39	8742	0.06	148	6.31	5314
MARVELD3_t1	1.01	0.831330474	0	120	0.03	8499	-0.08	144	-7.25	12759	-0.04	152	-3.59	10428
RPL15	1.12	0.001809157	0	125	0.03	8500	0.07	120	5.16	5244	-0.02	139	-1.74	9573
PIK3C3	-1.11	0.01167562	0	116	0.03	8501	-0.02	108	-1.03	9166	-0.14	114	-8.69	12488
NPRL2	1.16	7.95E-09	0	92	0.03	8502	0.1	109	6.19	4671	0.1	95	4.54	6157
TSACC	1.31	7.82E-15	0	60	0.03	8503	0.07	69	1.24	7655	0.16	73	3.71	6560
PLBD2	1.09	2.46E-05	0	126	0.02	8504	-0.04	119	-2.86	10386	0.04	114	2.31	7287
TRIM25	-1.01	0.799948714	0	143	0.02	8505	-0.01	124	-1.04	9168	-0.03	119	-1.77	9599
MMP27	1.01	0.39655242	0	95	0.02	8506	-0.1	88	-3.65	10905	-0.01	99	-0.32	8804
GYPB	1.04	8.51E-06	0	113	0.02	8507	0.02	102	1.04	7783	0.09	100	4.75	6052
DIS3_t1	1.04	0.014159048	0	134	0.02	8508	NA	NA	NA	NA	0.03	137	2.37	7251
SP4	1.01	0.394011538	0	138	0.02	8509	-0.09	146	-8.37	13272	0.07	135	6.35	5297
TG	1.17	0.000164896	0	100	0.01	8510	-0.04	84	-1.29	9357	-0.02	94	-0.83	9100
OR10A4	1.02	0.195898931	0	78	0.01	8511	-0.06	68	-1.12	9232	-0.04	60	-0.37	8831
NFE2L1	1.09	0.050264388	0	119	0.01	8512	0.06	120	4.28	5724	0.02	139	1.74	7601
POLR3H	1.13	0.002419638	0	138	0.01	8513	0.07	141	5.99	4793	0.11	126	8.38	4475
CYLC2	-1.02	0.058191271	0	151	0.01	8514	-0.09	116	-5.83	12106	-0.13	111	-7.7	12117
TMX4	1.14	0.004968366	0	131	0.01	8515	0	129	-0.02	8502	0.11	119	7.29	4919
RGPD1	-1	0.909589798	0	120	0.01	8516	-0.02	129	-1.84	9715	-0.01	146	-1.1	9248
OTOA	-1.03	0.052942582	0	100	0	8517	0.01	108	0.63	8049	-0.04	109	-2.53	9965
RASGRP4	-1.01	0.634531818	0	108	0	8518	-0.03	122	-2.05	9859	-0.11	123	-8.33	12370
HOXD4	1.01	0.152054233	0	91	0	8519	0.01	88	0.45	8177	0	92	0.14	8534
SGK3	-1	0.960062671	0	139	-0.01	8520	-0.03	143	-2.8	10338	0.14	152	14.29	2525
TMCO4	1.03	0.38394606	0	110	-0.01	8521	0.01	149	1.36	7568	-0.05	132	-4.35	10777
RAPSN	-1.03	0.009254392	0	109	-0.01	8522	-0.19	120	-13.07	14768	-0.08	135	-6.58	11681
SETX	-1.2	0.000136349	0	142	-0.01	8523	-0.01	129	-0.68	8922	-0.1	119	-6.6	11693
PRTN3	1	0.823000132	0	87	-0.01	8524	-0.05	145	-5.08	11710	-0.02	100	-0.76	9061
TTTY20	1.03	0.010215438	0	115	-0.01	8525	0	104	0.07	8423	0.03	100	1.41	7802
CFL2	-1.13	0.102290591	0	133	-0.01	8526	-0.11	126	-8.27	13224	0.08	141	7.58	4800
PLEKHJ1	1.06	0.128532892	0	113	-0.01	8527	0.03	116	1.74	7319	-0.05	115	-3.33	10324
STK11IP	1.03	0.738933012	0	96	-0.01	8528	0.23	136	20	401	-0.11	120	-7.86	12183
IRF2_t1	-1.15	4.73E-06	0	144	-0.01	8529	NA	NA	NA	NA	NA	NA	NA	NA
ITGB5	-1.01	0.777529947	0	107	-0.02	8530	-0.04	120	-2.88	10399	-0.06	124	-4.15	10696
DEFB123	1.03	0.002891778	0	88	-0.02	8531	-0.04	138	-3.54	10834	0.15	81	4.5	6176
MOSPD3	1.05	0.069578014	0	100	-0.02	8532	-0.09	120	-6.11	12248	0.02	124	1.82	7553
PYY	1.02	0.124780271	0	84	-0.02	8533	-0.09	140	-8.45	13304	-0.02	114	-1.45	9421
ZNF780B	1.09	0.018807948	0	131	-0.02	8534	0	137	0.01	8457	0.09	143	8.46	4441
SKIV2L	1.04	0.195465262	0	88	-0.03	8535	-0.1	76	-2.61	10224	-0.05	80	-1.38	9381
SCN10A	1.03	0.000554774	0	60	-0.03	8536	NA	NA	NA	NA	NA	NA	NA	NA
ARHGEF19	1.09	0.019456688	0	109	-0.03	8537	0.11	145	10.48	2687	0.03	108	1.69	7631
ACSM1	1.56	0.001294977	0	93	-0.03	8538	-0.05	105	-2.97	10460	0.09	113	5.65	5601
NAT6	1.07	0.00677103	0	91	-0.03	8539	0.02	93	0.93	7851	0.06	80	1.73	7610
ITGA8	-1.04	0.112497282	0	88	-0.03	8540	0.11	106	5.9	4842	-0.02	80	-0.72	9041
DSPP	1.01	0.378332626	0	130	-0.03	8541	-0.09	126	-6.57	12451	0.04	103	1.97	7468
SFTA2	1.02	0.378410646	0	104	-0.03	8542	-0.1	113	-6.31	12330	0.01	80	0.34	8411
SPEF2_t2	1.05	0.003403133	0	76	-0.04	8543	0.14	76	3.75	6036	0.02	107	0.89	8086
LAMB1	-1.13	0.255700865	0	92	-0.04	8544	0.13	111	7.72	3872	-0.01	99	-0.51	8917
SPRR2G	1.03	0.061223142	0	115	-0.04	8545	0.04	104	2.03	7124	-0.01	92	-0.32	8805
DCDC2	1.14	0.120492702	0	140	-0.04	8546	0.07	135	5.67	4962	0.05	142	4.85	6004
CEACAM5	1.02	0.153575519	0	131	-0.04	8547	-0.14	139	-12.05	14519	0.14	131	11.13	3462
NTRK3_t1	1	0.827064915	0	116	-0.04	8548	-0.1	124	-7.71	12995	-0.11	90	-4.25	10735
FAM219A	-1.02	0.139341567	0	124	-0.04	8549	-0.04	152	-3.98	11094	-0.08	148	-7.91	12202
GLRA1	1.02	0.009428528	0	101	-0.04	8550	-0.02	111	-1.11	9226	0	94	-0.16	8703
TPGS1	-1.04	0.073165507	0	72	-0.04	8551	-0.14	92	-6.05	12215	0	104	0.17	8506
RLF	-1.13	0.005433816	0	145	-0.04	8552	0.02	132	1.51	7492	-0.03	122	-1.8	9611
NPAP1	1.05	8.33E-07	0	154	-0.04	8553	0.08	138	6.7	4402	0.05	130	3.81	6519
CPB1	1.04	0.117790417	0	124	-0.04	8554	-0.01	97	-0.66	8915	-0.01	104	-0.68	9021
TNFRSF18	1.05	0.002946259	0	100	-0.05	8555	0.04	106	2.28	6967	-0.04	90	-1.56	9477
EPM2A_t1	1.01	0.604332629	0	97	-0.05	8556	-0.01	103	-0.73	8958	0.02	122	1.59	7692
ATP6AP1L	1.14	0.001728174	-0.02	52	-0.05	8557	0.07	68	1.24	7646	0.13	80	3.88	6478
ITGB6	1.02	0.314991255	0	110	-0.05	8558	-0.06	72	-1.24	9321	0.01	80	0.23	8478
GLYCAM1	-1.01	0.577610202	0	124	-0.05	8559	-0.01	118	-0.35	8717	0.01	114	0.77	8156
AGFG2	1.21	5.68E-05	0	124	-0.05	8560	0.04	124	3.23	6359	0.11	138	9.95	3894
MAGEB2	1.38	4.59E-06	0	136	-0.06	8561	-0.02	99	-1	9144	0.09	144	8.71	4338
KLF1	1.03	0.009211599	0	117	-0.06	8562	-0.04	128	-3.06	10507	0.06	122	4.46	6207
RWDD2A	1.02	0.634303056	0	94	-0.06	8563	0.15	108	8.72	3404	-0.01	146	-1.05	9226
SP7	1.02	0.017306997	0	113	-0.06	8564	0.02	131	1.52	7484	0.09	120	5.98	5462
STARD7	1.08	0.007961575	0	125	-0.06	8565	0.06	131	4.5	5613	-0.05	135	-4.42	10808
NODAL	1.02	0.025021455	0	127	-0.06	8566	0.02	117	1.1	7744	0.08	120	5.44	5721
APMAP	1.09	0.255292339	0	118	-0.06	8567	-0.05	130	-4.18	11190	0.04	142	3.9	6468
FAM46B	1.07	2.94E-11	0	107	-0.06	8568	-0.03	131	-2.42	10096	0.02	155	2.56	7154
GABRB1	1.04	0.000438632	0	119	-0.06	8569	0.06	102	3.07	6458	0.06	120	4.12	6361
NEURL4	-1.01	0.558108645	0	126	-0.06	8570	-0.06	124	-4.74	11508	-0.13	120	-9	12589
FRRS1L	1	0.896357072	0	116	-0.06	8571	-0.12	82	-3.83	10999	-0.04	90	-1.56	9474
CLDN11_t1	-1.18	1.24E-10	0	128	-0.06	8572	-0.16	118	-10.74	14150	NA	NA	NA	NA
CNTRL_t1	-1.02	0.492198148	0	132	-0.07	8573	NA	NA	NA	NA	0	122	0.31	8433
KANK3	-1.01	0.423761461	0	86	-0.07	8574	-0.08	130	-6	12185	-0.1	96	-4.37	10784
LLGL1	1.05	4.14E-05	0	114	-0.07	8575	-0.04	113	-2.6	10222	0.05	121	3.2	6829
IL6R	1.02	0.805385396	0	144	-0.08	8576	-0.16	136	-14.05	15002	0.05	132	3.84	6508
PDE6B_t1	-1.03	0.125778217	0	122	-0.08	8577	NA	NA	NA	NA	-0.05	114	-3.19	10261
FBXL16	1.22	0.049526813	0	114	-0.08	8578	0.08	154	8.08	3701	-0.01	139	-1.14	9268
SYT1	1.14	0.001908862	0	144	-0.09	8579	-0.05	130	-4.14	11172	0.14	145	13.5	2737
SPINK4	1.09	0.005828817	0	88	-0.09	8580	0.02	84	0.79	7947	0.05	80	1.45	7785
CCDC53	1.28	7.30E-06	0	84	-0.09	8581	0.05	116	3.33	6297	0.14	98	6.74	5134
HIST1H4L	1.34	8.83E-06	0	109	-0.09	8582	0.04	88	1.63	7404	-0.01	98	-0.55	8953
IQSEC3	-1.03	0.009588829	-0.04	52	-0.09	8583	NA	NA	NA	NA	NA	NA	NA	NA
CPNE4	1.03	0.007357951	0	125	-0.09	8584	0.07	136	6.07	4744	0.06	127	4.46	6204
MYLK2	1.03	0.137725858	0	101	-0.09	8585	-0.09	122	-6.53	12425	-0.02	114	-1.45	9420
IL21	1.02	0.17918713	0	100	-0.09	8586	0.03	135	2.31	6949	0	121	-0.22	8742
OR51B5	1.06	1.69E-07	0	90	-0.09	8587	-0.06	120	-4.36	11292	0.03	80	0.93	8059
FAM122C	1.08	0.00288055	0	109	-0.09	8588	0.04	119	3.07	6453	0.11	134	9.56	4037
GNB1L	1.11	2.69E-05	0	101	-0.09	8589	0.07	120	5.01	5334	0.05	106	2.72	7068

TPD52L3	-1.02	0.07784036	0	123	-0.09	8590	-0.03	126	-2.4	10087	0.03	134	2.31	7290
CSGALNACT2	1	0.981405756	0	134	-0.09	8591	0.12	134	10.08	2833	0.04	135	3.37	6731
SRC	1.05	0.04190207	0	125	-0.09	8592	0.03	137	2.86	6581	-0.04	120	-3.14	10245
NUDT22	1.01	0.874696311	0	92	-0.1	8593	-0.06	72	-1.41	9440	0.02	83	0.76	8161
CCDC36_t1	1.02	0.095490631	0	113	-0.1	8594	0.23	113	14.53	1397	NA	NA	NA	NA
BRF1	1.01	0.642869106	0	135	-0.1	8595	-0.09	119	-6.08	12237	-0.07	116	-4.74	10960
COG7	1.12	3.29E-06	0	114	-0.1	8596	0.15	113	9.68	2986	0.06	100	3.03	6915
SPAG16_t2	-1.13	0.018804906	0	141	-0.1	8597	0.08	110	4.96	5369	0	127	0.24	8471
TMEM143	1.03	0.500185601	0	102	-0.1	8598	-0.03	120	-2.09	9892	-0.06	124	-4.1	10679
ANXA2R	1.31	1.37E-05	0	116	-0.1	8599	0.12	104	6.31	4613	0.03	134	2.18	7359
CDY2A	1.01	0.513788225	-0.01	60	-0.1	8600	NA	NA	NA	NA	-0.01	64	-0.15	8701
EFHD2_t1	-1.21	2.05E-05	0	110	-0.1	8601	NA	NA	NA	NA	NA	NA	NA	NA
MFSD12_t1	1.09	0.138870177	0	100	-0.11	8602	0.15	124	11.05	2454	-0.05	114	-3.07	10213
ST3GAL3_t1	1.02	0.270623357	0	122	-0.12	8603	-0.01	107	-0.81	9024	NA	NA	NA	NA
XIRP2_t1	-1.02	0.025404954	0	141	-0.12	8604	NA	NA	NA	NA	NA	NA	NA	NA
ASCL3	1.03	0.00892558	-0.01	70	-0.12	8605	0.18	79	5.11	5273	0.01	81	0.44	8348
NPB	1.02	0.130747992	0	84	-0.13	8606	-0.07	114	-4.72	11496	-0.03	92	-1.2	9297
LRRC43	-1	0.904289671	-0.02	56	-0.13	8607	0.03	72	0.64	8039	0	68	-0.03	8641
CHRNE	-1.04	0.076390305	0	136	-0.13	8608	-0.22	112	-13.73	14931	-0.1	137	-9.12	12632
EDN3	-1	0.858633242	0	130	-0.13	8609	0.03	142	2.9	6555	-0.02	150	-1.59	9496
KRTAP17-1	1.05	7.71E-06	0	115	-0.13	8610	0.05	124	3.33	6294	0.09	130	7.12	4988
LY96	-1.32	0.005520988	0	101	-0.13	8611	-0.04	68	-0.73	8955	-0.18	71	-3.69	10474
JMY	-1.12	0.002766633	0	139	-0.13	8612	-0.08	143	-7.11	12701	0.01	152	1.1	7967
HDX	1.06	0.010343498	0	125	-0.13	8613	0.12	145	10.94	2501	0.01	139	1.16	7938
SOX4	1.22	0.022659349	0	134	-0.13	8614	0.12	146	11.69	2237	0.03	145	3.09	6877
CECR1_t1	1.01	0.432446887	0	132	-0.13	8615	0.05	131	4.34	5694	-0.14	131	-11.56	13420
GSX2	1.01	0.424805006	-0.02	57	-0.14	8616	0.05	104	2.76	6633	-0.15	64	-2.07	9743
CHRNA3	1.09	0.006554706	0	122	-0.14	8617	0.1	143	8.96	3294	0.08	120	5.56	5646
CASP10_t1	1.01	0.520937881	0	104	-0.14	8618	0.11	126	8.55	3477	-0.12	136	-10.04	12940
SPIB	1.02	0.289618838	0	131	-0.14	8619	-0.09	136	-7.43	12843	-0.01	128	-0.77	9070
HELQ	-1.04	0.044652476	0	109	-0.14	8620	-0.05	109	-2.74	10299	0.03	113	2.09	7399
EIF3J	-1.04	0.548954936	0	128	-0.14	8621	0	119	-0.01	8493	0.05	147	4.57	6143
TTTY2	1.03	0.001532923	0	147	-0.14	8622	0.03	127	2.37	6905	0.01	127	0.96	8040
URM1	-1	0.852696817	0	143	-0.14	8623	-0.01	135	-0.45	8781	-0.05	127	-4.19	10709
KCTD9_t1	-1.13	0.000110612	0	120	-0.14	8624	NA	NA	NA	NA	NA	NA	NA	NA
TTC32	1.17	0.002917767	0	110	-0.14	8625	0.05	96	2.43	6874	0.08	122	5.63	5609
GSTA4	1.57	3.98E-10	0	103	-0.14	8626	0.13	122	9.1	3225	0.08	104	4.11	6367
PDK3	-1	0.912827374	0	126	-0.15	8627	0.02	116	1.24	7648	-0.09	122	-6.49	11633
CLK4_t1	-1.1	0.019742256	0	129	-0.15	8628	NA	NA	NA	NA	0.03	131	2.15	7370
TEDDM1	1.02	0.015944369	0	116	-0.15	8629	0.04	137	3.1	6434	0.08	124	5.78	5543
CCDC130	1.11	0.005216652	0	114	-0.15	8630	0.02	129	1.54	7475	0.02	91	0.93	8055
ZNF491	1.09	3.08E-13	0	123	-0.15	8631	0.1	124	7.04	4228	0.1	123	7.52	4823
LRRC36	1.03	0.287298855	0	96	-0.15	8632	0.04	103	2.37	6910	-0.01	104	-0.42	8862
LCE2B	1.02	0.090155785	0	120	-0.15	8633	-0.06	111	-3.89	11032	0.02	152	2.14	7377
IFRD2	1.13	0.004182773	0	114	-0.16	8634	0.06	127	4.86	5421	0.09	135	7.82	4704
FAM20A_t1	-1.05	0.378213544	0	120	-0.16	8635	NA	NA	NA	NA	0.04	122	2.8	7032
ARMC3	1.04	0.026753678	0	88	-0.16	8636	0.07	143	6.79	4356	0.02	88	0.86	8103
CDH12_t1	1.03	0.086759437	0	138	-0.16	8637	NA	NA	NA	NA	-0.07	121	-4.79	10984
RABGAP1	1.01	0.679128444	0	131	-0.16	8638	0.07	133	5.52	5054	0.01	131	0.88	8090
VPRBP	1.03	0.092318014	0	135	-0.16	8639	-0.01	120	-0.66	8911	0.05	112	3.35	6745
XIST	-1.15	0.044251286	0	147	-0.17	8640	0.01	158	0.92	7859	-0.01	147	-0.9	9137
SIL1	-1	0.989186614	0	104	-0.17	8641	-0.1	103	-5.55	11942	0.01	115	0.85	8113
ZYX	1.02	0.6822785	0	106	-0.17	8642	0.01	127	0.71	7996	-0.01	127	-0.57	8962
CAPN12	1.18	0.070919598	0	103	-0.18	8643	0.16	118	11.06	2450	0.06	94	2.68	7095
FAM125B_t1	-1	0.811025895	0	140	-0.18	8644	0.05	127	4.08	5855	0	134	-0.24	8761
KCTD5	-1.02	0.318412848	0	118	-0.18	8645	0.04	140	3.37	6264	-0.1	122	-7.48	12028
APBB2	-1.09	0.068185286	0	129	-0.18	8646	-0.07	148	-7.25	12758	0.07	159	7.73	4746
DPP9	1.06	0.015953779	0	120	-0.19	8647	-0.04	143	-3.48	10784	-0.03	152	-2.85	10115
SLC45A2	1.09	3.98E-05	-0.02	60	-0.19	8648	-0.04	74	-0.88	9066	0.04	56	0.27	8459
TTF1	1.05	0.006729301	0	136	-0.19	8649	0.06	107	3.17	6404	0.05	114	3.38	6727
HIVEP3	1.07	3.14E-05	0	138	-0.2	8650	0	140	0.35	8237	0.04	136	3.3	6775
ADCYAP1R1	1.05	0.002122887	0	140	-0.2	8651	-0.13	140	-11.35	14332	-0.03	147	-3.04	10195
PCGF2	1.06	0.023962788	0	115	-0.2	8652	-0.07	120	-4.58	11430	-0.07	128	-5.7	11319
UVRAG	-1.08	0.000376612	0	134	-0.2	8653	-0.07	137	-6.15	12262	-0.09	138	-8.3	12363
NRTN	1.06	0.340317629	-0.01	88	-0.2	8654	-0.05	120	-3.57	10851	0.07	104	3.77	6536
IFI35	-1.04	0.248823398	-0.01	88	-0.2	8655	-0.17	122	-12.12	14541	-0.13	115	-8.73	12498
FAM163A	1.01	0.494083226	0	117	-0.2	8656	-0.11	150	-10.98	14231	-0.04	112	-2.42	9902
CLCN1	-1.09	4.44E-06	-0.07	53	-0.2	8657	-0.2	88	-7.61	12931	-0.09	60	-0.88	9127
KCNJ4	1.02	0.030143256	0	102	-0.2	8658	0.02	135	2.06	7108	-0.08	110	-4.86	11002
GNPDA2	-1.06	0.26283031	0	138	-0.21	8659	0.06	140	5.06	5298	0	142	-0.01	8627
CBR1	-1.03	0.76900348	0	115	-0.21	8660	-0.16	116	-10.34	14019	0.03	127	2.64	7111
AGR3	1.01	0.733351039	0	97	-0.21	8661	0.1	112	6	4785	0.05	131	4.12	6364
CCDC84	-1.08	0.053193386	0	105	-0.21	8662	0.05	113	3.19	6388	-0.12	118	-8.3	12361
RPS3	1.09	0.095406012	0	126	-0.21	8663	0.03	139	2.98	6509	-0.04	131	-2.87	10123
ZEB1	1.02	0.31090431	0	133	-0.22	8664	0.09	146	8.93	3314	0.02	131	1.73	7614
TSHR_t1	-1.01	0.548867182	0	135	-0.22	8665	0.02	125	1.69	7358	0.02	148	2.31	7286
MLANA	1.07	0.103236336	0	152	-0.22	8666	-0.06	127	-4.25	11240	0.06	130	4.4	6246
PLCH2	1.03	0.001053735	0	97	-0.22	8667	-0.03	102	-1.58	9538	0.13	100	6.51	5228
MUC7	1.01	0.299791476	0	124	-0.22	8668	-0.16	124	-11.89	14474	0.01	144	0.55	8288
FAM176B	-1.13	0.000829006	0	99	-0.22	8669	0.03	126	2.09	7089	-0.17	104	-9.44	12739
GSTT1_t1	-1.16	0.003384626	0	100	-0.23	8670	NA	NA	NA	NA	0.08	132	6.92	5064
MSX2	1.04	0.00114945	0	138	-0.23	8671	-0.05	121	-3.57	10855	0.05	139	4.63	6110
FGF6	1.02	0.113033697	-0.01	80	-0.23	8672	-0.03	76	-0.9	9074	0.04	132	3.47	6685
GRIN3B	1.02	0.047556758	-0.01	78	-0.23	8673	-0.07	139	-5.87	12125	0.06	112	3.55	6646
TMEM187	1.02	0.726883992	0	105	-0.24	8674	-0.12	108	-6.8	12560	-0.01	120	-0.36	8825
CHRNA7	-1.02	0.303637579	0	124	-0.24	8675	-0.05	124	-3.87	11019	-0.08	120	-5.69	11314
SLC2A11	1.2	5.68E-07	0	130	-0.24	8676	0.07	127	5.67	4966	0.03	104	1.75	7596
ENSA_t2	1.08	0.026670214	0	121	-0.24	8677	-0.04	124	-3.08	10526	0.05	115	2.97	6943
TRAK1_t1	-1.01	0.565582339	0	141	-0.24	8678	-0.13	140	-11.61	14400	-0.07	134	-6.06	11462
SLURP1	-1.02	0.452759811	-0.01	84	-0.25	8679	-0.03	107	-1.67	9598	-0.04	103	-2.06	9740
KLHDC10	1.01	0.898035525	0	157	-0.25	8680	-0.01	138	-1.25	9328	0.09	147	8.25	4516
TBC1D26	1.02	0.031825049	0	123	-0.25	8681	-0.06	115	-3.83	10997	-0.02	108	-1.41	9398
ALDH1A2	1.01	0.384886575	0	120	-0.25	8682	-0.01	140	-1.17	9266	0.04	122	2.82	7028
RBFOX1_t1	1.03	0.056117242	0	130	-0.25	8683	0.08	137	6.86	4322	0.11	127	8.22	4527
FGFBP1	-1.01	0.613919737	0	117	-0.25	8684	-0.09	111	-5.69	12026	-0.02	119	-1.58	9489
SON_t2	-1.03	0.664838011	0	119	-0.25	8685	0.01	127	0.62	8054	-0.02	137	-1.4	9394
XKRY2	1	0.801609832	0	119	-0.25	8686	0.05	123	3.6	6114	0.01	114	0.72	8184
SLITRK2_t1	-1.01	0.358898051	0	131	-0.26	8687	NA	NA	NA	NA	0.03	127	1.96	7475
VIPAS39	1.13	5.18E-05	0	120	-0.26	8688	0.01	128	0.79	7951	0.06	124	4.35	6265
ATRN_t2	-1.08	0.260285432	-0.02	62	-0.26	8689	-0.26	118	-17.62	15684	0.03	76	0.71	8187
DNAJC16_t1	-1.01	0.69190199	0	126	-0.26	8690	NA	NA	NA	NA	0	150	-0.46	8887
MLPH_t2	-1.28	0.016358555	0	136	-0.26	8691	-0.08	128	-6.32	12335	-0.11	131	-8.97	12584
CCDC63	1.01	0.26818148	-0.01	79	-0.26	8692	-0.01	90	-0.42	8759	-0.05	104	-2.9	10135
ARIH1	1.05	0.018293745	0	124	-0.27	8693	-0.02	125	-1.58	9533	0.14	140	12.34	3091
COL2A1	1.2	0.001809377	0	107	-0.27	8694	0.07	113	4.6	5552	0.04	120	3.13	6858
PPP1R16B_t1	-1.01	0.196397461	0	132	-0.27	8695	NA	NA	NA	NA	-0.04	120	-2.65	10013

XRN1_t2	-1	0.834693616	0	148	-0.27	8696	0.03	135	2.3	6953	-0.03	148	-2.89	10130
FMR1NB	1.06	0.026266317	0	118	-0.27	8697	-0.03	94	-1.5	9492	0.08	93	3.57	6630
UTY_t2	-1.06	0.023984289	0	127	-0.27	8698	-0.02	114	-1.3	9369	-0.04	135	-3.29	10301
TASP1	1.21	8.36E-08	0	126	-0.27	8699	0.2	129	16.17	1004	0.17	131	13.76	2659
KCNJ13	-1.01	0.675756508	0	132	-0.27	8700	0.02	126	1.42	7547	-0.12	128	-9.3	12687
APOBEC1	1.02	0.025296203	-0.01	90	-0.27	8701	0.07	92	3.1	6432	0.09	104	5.06	5899
TAF1D	1.02	0.772445851	-0.01	100	-0.27	8702	0.14	108	8.14	3670	-0.04	84	-1.33	9358
HK1_t1	1.03	0.007196231	0	106	-0.27	8703	0.12	114	7.69	3887	0.12	138	10.4	3725
TRIM7_t2	1.06	3.30E-05	0	131	-0.27	8704	-0.01	135	-1.21	9297	0.03	115	2.11	7390
ADH5	-1.06	0.219967922	0	131	-0.28	8705	-0.04	131	-3.34	10703	0.14	137	12.21	3134
TNNT2_t1	-1	0.916182067	-0.01	97	-0.28	8706	NA	NA	NA	NA	NA	NA	NA	NA
LEPROTL1	-1.27	9.79E-05	0	158	-0.28	8707	0	126	-0.3	8681	-0.1	133	-8.64	12469
KIAA0408_t1	1.01	0.211875142	0	152	-0.28	8708	NA	NA	NA	NA	0.07	144	6.83	5094
AMFR_t1	-1.26	1.01E-06	0	130	-0.28	8709	-0.12	127	-9.19	13588	NA	NA	NA	NA
CSTA_t1	1.17	0.112354898	0	120	-0.28	8710	NA	NA	NA	NA	0.04	119	2.9	6994
HOXC5	1.01	0.428163101	0	107	-0.28	8711	-0.07	119	-4.7	11491	-0.02	114	-1.1	9246
PKD2L2	-1.01	0.484444586	-0.01	103	-0.28	8712	-0.02	115	-1.04	9169	0	132	0.39	8382
OR51D1	1.04	0.006429108	-0.01	92	-0.29	8713	-0.03	111	-2.08	9878	0.02	93	0.74	8169
CIDEC	1.12	0.012843515	-0.01	106	-0.29	8714	0.06	129	4.84	5433	0.06	108	3.27	6792
BCL2A1	1.16	0.005257988	0	120	-0.29	8715	0.08	140	7.19	4141	0.13	99	6.52	5223
TNFSF14	-1.51	0.000222169	0	115	-0.29	8716	-0.14	142	-12.6	14667	-0.07	120	-4.95	11033
TWIST1	1.01	0.628888564	-0.01	106	-0.29	8717	0.12	128	9.4	3108	-0.03	110	-1.9	9670
KLRC1_t1	-1.05	0.050624202	0	114	-0.29	8718	-0.02	127	-1.29	9358	-0.17	135	-14.42	14218
ZNF577	-1.05	0.405331687	0	129	-0.3	8719	0.14	123	10.49	2685	0	138	-0.21	8738
MED24	1.11	3.33E-06	0	118	-0.3	8720	0.04	108	2.33	6936	0.04	99	1.88	7518
IL29	1.04	0.004056142	0	114	-0.3	8721	-0.08	104	-4.23	11227	-0.05	111	-2.92	10143
CC2D1A	1.01	0.627766915	-0.01	105	-0.3	8722	-0.02	143	-1.97	9798	-0.08	120	-5.88	11397
OR7D4	1.04	0.003754827	-0.02	64	-0.3	8723	-0.03	116	-2.06	9867	0	80	0	8617
MS4A5	-1	0.681206894	-0.01	84	-0.3	8724	-0.04	86	-1.27	9344	-0.05	108	-3.18	10257
TNFRSF21_t1	-1.09	0.081579499	0	149	-0.3	8725	NA	NA	NA	NA	-0.09	132	-7.39	11990
NADSYN1	1.12	0.000784782	0	114	-0.3	8726	0.1	112	6.13	4711	-0.01	98	-0.38	8837
CDH20	1.02	0.176121803	0	133	-0.3	8727	0.04	116	2.71	6668	-0.14	111	-8.39	12394
NLRP10	-1	0.831503756	-0.01	82	-0.3	8728	0.03	69	0.63	8051	-0.02	91	-0.87	9120
CES2_t1	-1.23	0.058918703	0	152	-0.3	8729	-0.18	149	-17.64	15688	0.01	112	0.92	8066
STARD3NL	1.02	0.676874827	-0.01	108	-0.31	8730	0.14	136	12.05	2116	0.01	135	0.8	8134
DPF1	1.02	0.115688384	-0.01	110	-0.31	8731	-0.05	113	-3.33	10690	-0.02	118	-1.18	9283
KBTBD2	-1.03	0.470768703	0	126	-0.31	8732	0.13	115	8.41	3540	0.06	134	4.76	6045
CAMSAP1	-1.05	0.088012264	0	154	-0.31	8733	0.05	134	4.4	5661	-0.11	136	-9.59	12777
ZFP106	-1.08	0.065542688	0	163	-0.32	8734	0	139	-0.18	8604	-0.02	131	-1.22	9311
CTRB1	1.03	0.183763527	-0.02	64	-0.32	8735	-0.17	96	-7.79	13025	-0.01	71	-0.2	8733
CFC1	1.03	0.004623012	-0.01	110	-0.32	8736	-0.04	129	-2.93	10428	-0.03	127	-2.32	9851
TGM2_t1	-1.07	0.295504257	-0.01	102	-0.32	8737	-0.04	112	-2.7	10273	-0.06	109	-3.67	10467
ZNF426	-1.09	0.084472596	0	133	-0.33	8738	0.08	131	6.76	4372	0.01	121	0.9	8076
GPR1	1.05	1.53E-06	0	120	-0.33	8739	0.1	147	9.43	3096	0.07	152	7.49	4837
PLEC_t3	1	0.943674141	-0.01	93	-0.33	8740	-0.05	125	-3.45	10764	-0.11	131	-8.74	12508
FGF8	-1.08	0.025027069	-0.01	103	-0.33	8741	-0.09	94	-3.93	11059	-0.06	104	-3.13	10240
THAP7	1.03	0.193248959	-0.01	100	-0.33	8742	-0.09	80	-2.83	10362	0.02	88	0.65	8225
TTC22	1.04	0.005447544	0	125	-0.33	8743	-0.07	120	-4.61	11447	0.01	120	0.68	8204
TEC	-1.02	0.025918503	0	129	-0.34	8744	-0.03	111	-1.76	9659	0.02	116	1.24	7884
FANK1	1.05	0.116441306	-0.01	116	-0.34	8745	0.16	100	8.11	3692	-0.02	90	-0.97	9187
SPRR4	-1	0.817981642	-0.01	101	-0.34	8746	0.04	123	2.62	6743	0.02	107	1.13	7954
NEUROD2_t1	-1.01	0.552888374	-0.01	114	-0.34	8747	-0.06	130	-4.86	11569	NA	NA	NA	NA
ATPIF1	-1.04	0.545665639	0	121	-0.34	8748	-0.04	131	-3.6	10880	0.05	134	4.05	6392
SLC2A12	-1.01	0.861980476	0	132	-0.34	8749	-0.16	155	-16.47	15503	0.03	141	2.42	7222
SPATA7	-1.08	0.008337576	-0.01	79	-0.34	8750	-0.04	81	-1.19	9280	-0.03	72	-0.58	8974
CD99	1.02	0.703278326	-0.01	116	-0.34	8751	0.01	112	0.86	7896	-0.08	120	-5.81	11371
RGS19	-1.05	0.045130609	0	140	-0.34	8752	-0.05	111	-2.76	10321	-0.15	124	-11.34	13356
SYNM	1.02	0.804002844	0	130	-0.35	8753	0.02	125	1.86	7233	0.08	145	7.71	4751
OMA1_t1	-1.03	0.593487992	-0.01	117	-0.35	8754	NA	NA	NA	NA	NA	NA	NA	NA
RBM4B	1.06	0.005907684	-0.01	104	-0.35	8755	0.02	126	1.83	7260	0.04	132	3.59	6614
ITGA2B	1.01	0.498113627	-0.01	88	-0.35	8756	-0.05	101	-2.63	10236	0.02	101	0.85	8110
LPPR3	1.02	0.283035531	-0.02	70	-0.35	8757	-0.01	88	-0.3	8679	0.01	68	0.19	8498
SDK1_t2	1.02	0.105561283	0	132	-0.35	8758	NA	NA	NA	NA	0.1	120	6.74	5133
WBP2	-1.01	0.704652681	0	132	-0.35	8759	-0.03	147	-2.85	10382	-0.05	131	-4.16	10699
TCF23	1.02	0.209426362	-0.01	97	-0.36	8760	-0.15	137	-13.18	14791	0.01	91	0.52	8302
TAF1	1.05	0.009894852	0	152	-0.36	8761	0.07	135	5.96	4806	0.07	120	4.95	5953
DGKE	-1.01	0.428653336	0	133	-0.36	8762	0.04	147	4.24	5756	0.02	164	1.83	7551
ADH7	1.04	0.05792823	-0.01	119	-0.36	8763	0.01	116	0.59	8082	0.08	113	4.89	5981
GPR174	-1	0.811859431	-0.01	115	-0.36	8764	-0.01	104	-0.29	8668	0	106	-0.27	8774
CHRM4	-1.05	0.005297395	-0.01	76	-0.36	8765	-0.1	59	-0.88	9065	-0.08	88	-2.92	10141
DNAJA1_t1	-1.09	0.174883315	0	132	-0.36	8766	NA	NA	NA	NA	0.05	111	3.1	6871
RTN4RL1_t1	-1	0.772095167	-0.01	115	-0.36	8767	-0.08	141	-6.93	12618	NA	NA	NA	NA
CAV2_t1	-1.05	0.394536586	0	136	-0.37	8768	NA	NA	NA	NA	0.07	159	7.22	4939
CPA2	1	0.881149606	-0.01	88	-0.37	8769	-0.07	74	-1.77	9670	-0.06	81	-1.88	9658
PARL	1.07	0.01098309	-0.01	96	-0.37	8770	-0.04	133	-3.69	10927	-0.02	97	-1.07	9228
PENK	1	0.614207076	0	131	-0.37	8771	-0.07	98	-3.3	10669	-0.03	118	-1.78	9602
TMEM222	1.06	0.007575119	-0.01	102	-0.38	8772	0.04	140	3.88	5959	-0.05	111	-3.12	10231
TAS2R50	-1.07	0.004950724	-0.01	88	-0.38	8773	-0.06	102	-3.06	10509	-0.08	68	-1.36	9372
KRT4	1.08	2.91E-05	-0.01	110	-0.38	8774	0.01	107	0.54	8113	0.04	103	2.32	7274
ATP2B2	1.08	0.015112932	0	152	-0.38	8775	-0.05	142	-4.21	11214	0.05	146	5.02	5914
ATP2A1	1.03	0.034663735	-0.01	99	-0.38	8776	-0.02	114	-1.01	9154	-0.11	115	-6.83	11784
MGP	-1.32	0.004423421	-0.01	116	-0.38	8777	0.03	135	2.65	6720	-0.17	125	-12.66	13751
PCDHGA8_t1	1.02	0.022473128	-0.01	116	-0.39	8778	0.12	85	4.27	5733	0.01	128	0.94	8053
SLC16A3	-1.07	0.541852195	-0.01	100	-0.39	8779	0.16	109	9.21	3185	-0.1	99	-4.94	11029
GPR4_t1	-1	0.966212106	-0.01	109	-0.39	8780	NA	NA	NA	NA	0.01	135	0.58	8274
PRSS1	1.15	4.16E-05	-0.02	68	-0.39	8781	0.03	89	1.33	7589	-0.03	80	-1.01	9212
LIPH	1.08	0.077275514	-0.01	120	-0.39	8782	0.07	140	6.17	4689	-0.02	126	-1.43	9409
ARRDC2	-1.05	0.47386639	-0.01	112	-0.39	8783	-0.01	112	-0.78	9006	-0.07	128	-5.72	11323
VAX2	1.03	0.005687189	-0.01	99	-0.39	8784	0.09	129	7.23	4128	0.05	100	2.44	7210
FOXJ1	-1.01	0.524949916	-0.01	120	-0.39	8785	-0.11	142	-9.91	13849	-0.01	120	-0.8	9086
RBM39	1.03	0.591709939	0	137	-0.39	8786	0.04	139	3.42	6236	0.01	137	1.01	8016
CDSN_t1	-1	0.80978682	-0.01	106	-0.39	8787	-0.05	127	-3.9	11039	NA	NA	NA	NA
RHOV	1.08	0.125026755	-0.01	99	-0.39	8788	0.06	131	4.47	5626	-0.1	120	-7.23	11931
MSANTD2	1.01	0.623804504	-0.01	126	-0.39	8789	0.02	139	1.78	7297	0.01	124	0.92	8065
TM6SF1	-1.02	0.22310061	-0.01	124	-0.39	8790	0	120	0.24	8297	-0.04	126	-3.13	10242
TSSK6	1.03	0.005160816	-0.01	85	-0.4	8791	-0.04	113	-2.59	10207	0.08	120	5.83	5530
PGM2L1	-1.01	0.703188764	0	146	-0.4	8792	0.15	163	16.7	896	0.04	153	3.94	6444
SERPINH1	-1.03	0.519728227	-0.01	123	-0.4	8793	0.05	138	4.6	5556	-0.06	104	-3.48	10387
WBP1L	1.03	0.025521342	-0.01	117	-0.4	8794	-0.02	129	-1.95	9784	-0.02	120	-1.55	9472
BTN3A2	-1.05	0.374731663	-0.01	123	-0.4	8795	-0.01	135	-1.16	9263	-0.11	129	-8.74	12505
INSL5	1.01	0.260502956	-0.01	96	-0.4	8796	-0.06	102	-3.38	10721	0.02	128	1.31	7848
TPCN1_t1	1.04	0.19735774	-0.01	127	-0.4	8797	NA	NA	NA	NA	NA	NA	NA	NA
PTPN21	1.01	0.227186917	0	135	-0.4	8798	0.05	129	3.97	5921	0.03	135	2.48	7194
LUZP2	1.08	0.015018748	0	146	-0.4	8799	0.08	127	6.18	4672	-0.05	128	-3.72	10493
HM13_t2	1.05	0.205072951	-0.01	124	-0.41	8800	0.11	124	8.13	3682	-0.08	128	-5.89	11405
REG3G	1.69	1.04E-05	-0.01	120	-0.41	8801	0	111	0.19	8339	0.12	90	4.97	5945

TRAPPC5_t1	-1.06	0.068056448	-0.02	68	-0.41	8802	NA	NA	NA	NA	NA	NA	NA	NA
MKLN1	-1.02	0.554604083	0	142	-0.41	8803	-0.06	133	-4.66	11468	0.1	148	10.13	3825
HNRNPU_t2	1.1	4.02E-06	0	143	-0.41	8804	0.11	152	11.52	2300	0.02	146	2.17	7360
AKAP3	1.07	4.40E-05	-0.01	90	-0.41	8805	0.01	116	0.65	8036	0.07	117	4.56	6149
TAC1_t1	-1.05	0.057385705	-0.01	111	-0.41	8806	NA	NA	NA	NA	0.01	136	0.76	8162
CHMP4A	-1.13	0.013777024	-0.01	95	-0.41	8807	0.11	119	7.52	3980	-0.17	94	-7.67	12099
OR51B2	-1	0.83650338	-0.02	72	-0.42	8808	0.02	80	0.74	7975	-0.07	60	-0.72	9037
LPPR2_t1	-1.03	0.186103633	-0.01	113	-0.42	8809	NA	NA	NA	NA	NA	NA	NA	NA
IRAK2	-1	0.800128628	-0.01	123	-0.42	8810	-0.03	118	-1.89	9742	-0.1	130	-7.67	12098
IQCF1	1.05	0.000107855	-0.04	60	-0.42	8811	-0.03	64	-0.44	8775	0	68	-0.02	8633
MCHR1	1.12	0.005001859	0	137	-0.42	8812	-0.04	132	-3.49	10793	0.16	148	15.87	2108
CLEC7A_t1	-1.19	1.01E-10	-0.01	121	-0.42	8813	0.01	116	0.78	7957	-0.19	127	-14.45	14225
ADRBK1_t1	-1.12	6.91E-06	-0.01	120	-0.42	8814	-0.05	120	-3.37	10716	NA	NA	NA	NA
DOCK11	-1.2	0.006622766	0	135	-0.42	8815	0.07	94	3.21	6368	-0.08	136	-7.1	11882
SCGB2A1	1.32	0.000165538	-0.01	119	-0.42	8816	0	90	-0.01	8495	0.05	102	2.65	7107
BMP2K_t2	-1.11	0.000211159	-0.01	130	-0.42	8817	0.07	131	5.69	4957	-0.04	127	-3.15	10246
ABCA7	1	0.923469515	-0.02	72	-0.43	8818	-0.05	88	-1.75	9648	-0.05	90	-1.9	9669
NDUFS1	-1.07	0.233504651	-0.01	134	-0.43	8819	-0.01	114	-0.67	8918	0.01	125	0.83	8119
SDR16C5_t1	-1.09	0.000294	-0.01	126	-0.43	8820	NA	NA	NA	NA	NA	NA	NA	NA
RPL18A_t1	1.03	0.535470922	-0.02	76	-0.43	8821	0.06	95	2.59	6763	NA	NA	NA	NA
SCN4A	1.08	0.00039521	-0.01	126	-0.43	8822	-0.1	134	-8.25	13215	0.04	130	3.08	6887
FAM101A_t1	1.28	0.001538522	-0.01	115	-0.43	8823	NA	NA	NA	NA	NA	NA	NA	NA
CMTM4_t2	1.03	0.116180966	-0.01	134	-0.43	8824	-0.08	132	-6.25	12303	0.02	136	1.77	7585
SNUPN_t1	1.08	0.008610008	-0.01	109	-0.44	8825	NA	NA	NA	NA	0.08	107	4.47	6201
MMP20	1.02	0.128493662	-0.01	113	-0.44	8826	0.01	119	0.94	7843	0.12	123	8.6	4383
ARHGEF3	-1.03	0.63457832	-0.01	130	-0.44	8827	0.07	124	4.82	5438	-0.07	134	-5.62	11284
TEX101	1.08	0.009120948	-0.01	129	-0.44	8828	-0.01	112	-0.92	9094	0.02	100	0.96	8043
PHOX2B	1	0.648760524	-0.01	120	-0.44	8829	-0.08	120	-5.4	11871	0.03	127	1.96	7472
BTNL9	1.04	0.409028636	-0.01	115	-0.45	8830	-0.08	127	-6.18	12277	0.07	138	5.91	5490
HKR1	1.02	0.594416237	-0.01	122	-0.45	8831	0.12	124	8.73	3398	-0.06	118	-4.23	10728
PARP11	1.04	0.131060675	0	141	-0.45	8832	0.08	146	7.36	4056	0.03	130	2.36	7258
PASK	-1.11	0.008391701	-0.01	121	-0.45	8833	-0.08	127	-6.17	12270	-0.14	110	-8.41	12402
GRIK2_t1	1.02	0.044457058	-0.01	130	-0.45	8834	0.07	132	5.87	4863	0.04	120	2.71	7077
RNF141_t1	-1.06	0.067200332	0	145	-0.46	8835	NA	NA	NA	NA	NA	NA	NA	NA
NXPE1	-1.02	0.006817986	-0.01	95	-0.46	8836	-0.04	102	-2.22	9978	-0.11	100	-5.35	11181
MALSU1	1.07	0.086226564	-0.01	94	-0.46	8837	-0.07	104	-3.91	11047	0.11	120	7.73	4745
HSPB9	-1.08	0.077189621	-0.08	56	-0.46	8838	-0.24	64	-3.33	10695	-0.08	80	-2.48	9943
PCSK7_t1	1.03	0.32230463	-0.01	120	-0.46	8839	0.01	120	0.72	7989	NA	NA	NA	NA
MEMO1	-1	0.963600303	-0.01	101	-0.46	8840	0.1	137	8.36	3563	0.02	150	2.43	7218
RTTN	-1.01	0.327010459	-0.01	115	-0.46	8841	-0.08	134	-6.35	12350	-0.01	101	-0.37	8833
IQCK	-1.08	0.157514511	-0.01	123	-0.47	8842	0.08	120	5.92	4828	-0.09	149	-9.27	12675
SLC6A2	-1	0.974842119	-0.01	133	-0.47	8843	-0.23	128	-17.61	15680	-0.04	146	-3.69	10475
HIST1H2AC	1.04	0.023357234	-0.01	92	-0.47	8844	-0.03	86	-0.91	9089	0	72	-0.04	8644
ACR_t1	-1.03	0.215418959	-0.02	72	-0.47	8845	NA	NA	NA	NA	-0.05	95	-2.35	9871
SLC30A3	1.05	0.33584887	-0.01	110	-0.47	8846	-0.13	111	-7.83	13042	-0.03	100	-1.51	9452
VDR	1.12	0.206466448	-0.01	131	-0.47	8847	0.06	127	4.87	5412	-0.01	127	-0.67	9020
P4HB	1.14	0.009492867	-0.01	126	-0.47	8848	0	115	-0.08	8538	0.03	124	2.49	7184
FAM114A1	1.05	0.145713184	-0.01	125	-0.48	8849	0.03	136	2.48	6834	0.05	146	4.59	6129
KLHL17	1.05	0.003679457	-0.01	108	-0.48	8850	0.07	124	5.47	5078	0.05	128	3.71	6563
GLIPR1_t1	-1.26	0.000612173	-0.01	132	-0.48	8851	0.04	125	3.33	6296	NA	NA	NA	NA
OAS2	-1.15	0.000551035	0	147	-0.48	8852	-0.09	133	-7.29	12778	-0.21	125	-16.03	14593
CALB1	1.13	0.00151824	-0.01	143	-0.48	8853	0.02	128	1.82	7262	0.07	125	5.04	5906
METAP2	1.01	0.840214802	-0.01	147	-0.49	8854	0.05	116	3.47	6203	0.06	124	4.76	6044
NDUFV3	1.01	0.716017986	-0.01	104	-0.49	8855	0.02	133	1.7	7353	0.02	123	1.63	7662
FAM108A1_t1	-1.06	0.025377803	-0.01	110	-0.49	8856	NA	NA	NA	NA	-0.17	100	-8.66	12476
LGALS13	-1.01	0.450506581	-0.01	111	-0.49	8857	-0.05	154	-4.93	11615	-0.05	99	-2.23	9807
NMNAT3	1.1	0.065232878	-0.01	120	-0.49	8858	-0.01	118	-0.58	8863	0.05	117	3.33	6754
RHCG	1.2	1.77E-05	-0.01	92	-0.49	8859	0.02	121	1.08	7753	0.06	100	2.91	6983
FUCA2	1.02	0.638258891	-0.01	106	-0.49	8860	0.08	137	7.19	4143	-0.04	130	-3.32	10315
WDR88	1.02	0.041819966	-0.01	88	-0.5	8861	0.05	108	3.17	6400	0	104	-0.16	8705
TTC12	-1.09	0.014641441	-0.01	95	-0.5	8862	-0.06	110	-3.73	10951	-0.08	104	-4.18	10706
MIER2	1.05	0.138129232	-0.01	120	-0.5	8863	0.08	123	6.18	4677	-0.01	131	-0.42	8860
KCNIP3	1.1	0.176944907	-0.01	120	-0.5	8864	0.01	130	0.43	8185	-0.01	134	-0.85	9108
WFIKKN1	1.03	0.470571591	-0.03	67	-0.5	8865	-0.08	130	-6.01	12188	-0.08	100	-4.18	10703
MUSK	1.04	0.003896512	-0.02	80	-0.5	8866	0.03	52	0.06	8425	0.02	69	0.35	8400
PSMF1_t1	-1.05	0.214051174	-0.01	110	-0.5	8867	NA	NA	NA	NA	NA	NA	NA	NA
MUC1	1.11	0.104063407	-0.01	98	-0.5	8868	-0.01	140	-0.97	9126	-0.02	121	-1.29	9342
FAM75E1_t1	-1.01	0.211133509	-0.01	95	-0.5	8869	NA	NA	NA	NA	NA	NA	NA	NA
RAC3	-1	0.891682294	-0.01	102	-0.5	8870	-0.04	112	-2.47	10130	-0.01	101	-0.35	8819
LCA5L	1.04	0.090832955	-0.01	88	-0.5	8871	0.02	106	1.19	7683	0.04	104	2.42	7220
GPR110_t2	1	0.690029873	-0.01	126	-0.51	8872	-0.07	103	-3.5	10794	NA	NA	NA	NA
ACTL9	1	0.917024517	-0.03	69	-0.51	8873	-0.15	80	-4.6	11443	-0.02	68	-0.45	8883
MYH9	-1.01	0.911450326	-0.01	127	-0.51	8874	0.12	125	9.29	3150	0.02	122	1.75	7599
RPS17	1.16	0.004779377	-0.01	109	-0.51	8875	0.05	70	1.04	7780	0.16	93	6.67	5164
SERPINB7	1.07	8.07E-10	-0.01	124	-0.52	8876	0.02	114	1.51	7491	0.06	115	3.85	6501
COX17	1.02	0.578059866	-0.01	112	-0.52	8877	-0.11	84	-3.71	10937	0.01	103	0.3	8439
ZNF630	1.02	0.316964043	-0.01	119	-0.52	8878	0.12	111	7.5	3989	-0.06	114	-3.8	10538
FLT1	1.01	0.696499479	-0.01	130	-0.52	8879	0.01	144	1.25	7642	0.14	130	11.59	3326
ZNF287	1	0.772990731	-0.01	128	-0.52	8880	0.05	124	3.4	6244	-0.04	131	-3.32	10319
TMPRSS11E	1.02	0.004360825	-0.01	136	-0.52	8881	0.13	142	12.25	2047	0.06	123	4.57	6141
REEP5	-1.08	0.053337655	-0.01	131	-0.52	8882	0.01	124	0.47	8156	-0.05	120	-3.45	10376
CGB	1.02	0.070414631	-0.04	64	-0.52	8883	-0.05	88	-1.77	9673	0	60	-0.04	8645
GAPDH	1.14	0.007686546	-0.01	100	-0.53	8884	0.04	133	3.1	6435	0.03	102	1.81	7561
ZFAND1	-1.02	0.733314317	-0.01	137	-0.53	8885	0	125	-0.22	8619	0.01	132	0.64	8234
DLD	-1.1	0.174829647	-0.01	117	-0.53	8886	0.06	152	6.12	4718	0.09	127	6.71	5150
SHISA2	1.04	0.09092546	-0.01	126	-0.53	8887	0.12	140	11.2	2397	-0.08	125	-5.89	11403
ZAK_t2	-1.05	0.204617254	-0.01	114	-0.53	8888	-0.08	140	-7.56	12904	0.02	155	2.54	7163
CCL22	1	0.968449998	-0.01	137	-0.53	8889	0.18	127	13.59	1656	-0.15	123	-11.3	13349
HIST1H1A	1.3	1.07E-06	-0.02	74	-0.53	8890	0	72	0	8477	0.05	68	0.91	8074
KCNA2	1.03	0.008937783	-0.01	95	-0.54	8891	-0.11	114	-6.77	12546	0.03	112	1.89	7516
WDR69	1.01	0.655418593	-0.01	141	-0.54	8892	0.01	128	1.08	7754	0.04	115	2.45	7206
KIAA1033_t1	-1.09	0.028257205	-0.01	142	-0.54	8893	NA	NA	NA	NA	NA	NA	NA	NA
TMEM208	1.05	0.268196789	-0.01	116	-0.54	8894	-0.12	119	-8.59	13357	-0.04	106	-2.4	9891
BCAR3	-1.04	0.391558093	-0.01	144	-0.54	8895	-0.08	122	-5.49	11907	0	135	0.21	8485
WWOX_t3	1	0.999261464	-0.01	124	-0.55	8896	-0.13	124	-9.35	13639	0	151	0.5	8320
LARS2	1.05	0.186529718	-0.01	123	-0.55	8897	0.04	127	2.7	6678	0.02	116	1.46	7781
PCDHGC3	-1.01	0.216990602	-0.01	141	-0.55	8898	0.09	96	3.95	5929	-0.07	78	-1.82	9621
TTTY9A	1.01	0.239289427	-0.01	121	-0.55	8899	0.03	153	3	6497	0.08	122	5.86	5517
PAN3	-1.19	0.000439886	-0.01	151	-0.55	8900	-0.02	123	-1.55	9519	-0.17	148	-16.62	14717
FAM83B	1.04	0.001207017	-0.04	64	-0.55	8901	-0.03	60	-0.31	8686	0.05	91	1.86	7526
KCNK1	-1.14	0.367770217	-0.01	147	-0.55	8902	-0.07	108	-3.77	10964	0.01	137	0.66	8220
MBD3L1	1.03	0.000593898	-0.02	86	-0.56	8903	-0.03	101	-1.67	9599	0	125	-0.23	8753
ENO1_t1	1.14	0.02299947	-0.01	100	-0.56	8904	NA	NA	NA	NA	0.02	124	1.32	7840
NLRX1	-1.03	0.418299208	-0.02	80	-0.56	8905	-0.01	76	-0.29	8672	-0.07	88	-2.5	9954
DCST2	1.01	0.614332959	-0.09	56	-0.56	8906	-0.14	86	-5.07	11699	-0.04	68	-0.72	9038
ASH2L	-1.15	0.000623243	-0.01	115	-0.56	8907	-0.11	135	-9.06	13533	-0.04	125	-3.01	10182

MRPL51	1.14	0.000716989	-0.01	100	-0.56	8908	0.11	98	5.31	5168	0.14	112	8.56	4402
GPRASP2	-1.08	0.057947482	-0.01	127	-0.56	8909	-0.14	122	-10.05	13901	0.02	107	1.14	7948
SLC9A3	1	0.699117909	-0.04	63	-0.56	8910	-0.08	113	-4.84	11558	-0.12	84	-3.96	10617
CLPTM1	1.02	0.494121382	-0.01	102	-0.56	8911	-0.13	148	-12.53	14651	-0.01	100	-0.29	8783
AKAP6	-1	0.834740676	-0.01	144	-0.56	8912	-0.07	136	-5.91	12136	0.02	124	1.39	7809
TRPM5	1.06	9.40E-06	-0.01	124	-0.57	8913	-0.07	111	-4.13	11162	0.01	120	0.73	8178
DDX28	-1.01	0.849864347	-0.01	126	-0.57	8914	-0.11	109	-6.68	12503	-0.1	116	-6.35	11593
APBB1	1.01	0.806274705	-0.01	115	-0.57	8915	-0.1	110	-5.77	12080	-0.03	120	-1.94	9688
ING2	-1.17	5.75E-05	-0.01	120	-0.57	8916	-0.11	109	-6.48	12406	-0.15	127	-11.49	13397
MCAT_t2	-1	0.980519775	-0.01	120	-0.57	8917	-0.02	134	-1.49	9484	0.01	104	0.46	8338
SYT9	1.09	5.09E-05	-0.01	116	-0.57	8918	-0.06	124	-4.37	11297	0.02	137	1.78	7578
SLC22A15	1.33	0.000967855	-0.01	133	-0.57	8919	0.14	139	12.66	1941	0.04	130	3.42	6704
MED9	1.03	0.555829064	-0.01	116	-0.57	8920	-0.1	135	-8.25	13217	-0.01	154	-1.33	9357
ZBTB47	-1.03	0.594414998	-0.01	133	-0.58	8921	-0.02	143	-1.52	9506	-0.06	159	-7.06	11866
PATZ1_t1	1.13	0.000278184	-0.01	104	-0.58	8922	-0.04	137	-3.64	10901	0.04	118	2.49	7186
STMN4	1.05	5.56E-05	-0.01	105	-0.58	8923	-0.11	118	-7.33	12805	0.09	124	6.87	5078
TOP2B	-1	0.965155486	-0.01	118	-0.58	8924	0.19	114	12.09	2106	0.07	131	5.67	5593
SLC22A14	-1.02	0.204149698	-0.01	100	-0.58	8925	-0.11	102	-5.74	12048	-0.03	103	-1.68	9541
PSMB1	1.06	0.138804932	-0.01	105	-0.58	8926	0.14	97	6.67	4413	-0.03	143	-2.93	10151
MYH2	1.36	0.009566291	-0.02	85	-0.59	8927	0.06	99	3.12	6424	-0.02	107	-1.26	9324
ACP1_t1	1.01	0.373754195	-0.01	120	-0.59	8928	0.04	116	2.56	6790	0	128	0.38	8388
RNPS1	-1.01	0.451867481	-0.01	108	-0.59	8929	0.05	133	4.55	5588	-0.07	126	-5.67	11306
HLA-DQA1	-1	0.933673854	-0.01	109	-0.59	8930	0.02	121	1.25	7641	-0.09	121	-6.26	11556
PHKB	-1.06	0.300555207	-0.01	142	-0.59	8931	0.07	126	5.23	5200	0.11	122	7.79	4714
SH3BGRL3	-1.15	0.000197689	-0.01	103	-0.6	8932	0.06	164	6.77	4368	-0.19	118	-12.59	13724
NINJ2	1.09	0.083241748	-0.01	108	-0.6	8933	0.04	108	2.06	7105	0.01	100	0.42	8354
TRIM29_t1	1.02	0.502110016	-0.01	112	-0.6	8934	0.02	157	2.55	6800	NA	NA	NA	NA
GCC2_t1	-1.09	0.017306271	-0.01	139	-0.6	8935	0.08	125	6.36	4582	NA	NA	NA	NA
ALOX12	1	0.904074421	-0.01	107	-0.6	8936	0.07	113	4.33	5702	0.03	126	2.23	7332
DBNDD2	1.01	0.850846435	-0.01	125	-0.6	8937	0.08	123	5.63	4986	-0.11	123	-7.99	12235
KLK13	1	0.880119876	-0.01	122	-0.6	8938	-0.09	130	-7.05	12672	0.04	116	2.89	7000
SMEK1	-1.05	0.075022305	-0.01	140	-0.6	8939	-0.03	115	-1.93	9765	0.06	129	4.4	6248
ROS1	1.05	0.056614972	-0.01	100	-0.6	8940	-0.04	124	-2.9	10417	-0.07	91	-2.74	10058
TMEM116	1.07	0.129015617	-0.01	117	-0.6	8941	0.02	108	0.97	7820	0.01	113	0.71	8188
CCDC108_t1	1.01	0.535470922	-0.01	118	-0.6	8942	0	125	0.08	8414	-0.01	146	-0.97	9186
PKD1L2_t1	1.04	0.015759942	-0.01	112	-0.6	8943	0.02	159	1.66	7379	0.07	115	4.31	6279
PRR14L	1.01	0.182385855	-0.01	127	-0.6	8944	-0.01	126	-0.69	8925	-0.05	147	-5.11	11082
CD1A_t1	1	0.886929296	-0.01	110	-0.61	8945	NA	NA	NA	NA	-0.03	129	-1.99	9705
SGIP1_t1	1.04	0.067548869	-0.01	129	-0.61	8946	NA	NA	NA	NA	0.07	146	7.15	4970
CEACAM19	-1.02	0.401798456	-0.01	110	-0.61	8947	-0.1	115	-6.5	12412	-0.12	106	-6.9	11812
LTV1	1.07	0.166560112	-0.01	125	-0.61	8948	0.18	117	12	2128	0.05	119	3.18	6841
SNX17	1.01	0.751637341	-0.01	115	-0.61	8949	-0.11	110	-6.75	12532	-0.06	136	-5.5	11241
TTTY3	-1	0.854660298	-0.01	99	-0.61	8950	0.14	108	7.95	3775	-0.06	115	-4.22	10723
OLFM4_t1	-1.02	0.781688707	-0.01	118	-0.62	8951	0.07	114	4.79	5450	NA	NA	NA	NA
PDGFD	1.02	0.698852128	-0.01	137	-0.62	8952	0.17	121	12.13	2091	0.01	157	1.52	7732
GPX3	-1.27	0.012898558	-0.01	105	-0.62	8953	-0.1	145	-9.73	13789	-0.03	104	-1.75	9588
MOG	1.02	0.056422738	-0.01	142	-0.62	8954	0	139	-0.02	8507	0.02	150	1.65	7649
EVI5	1	0.820749967	-0.01	141	-0.62	8955	-0.01	162	-1.61	9561	0.04	150	3.84	6503
DENND2D	-1.31	3.53E-05	-0.01	125	-0.62	8956	0.04	131	3.22	6364	-0.16	157	-17.51	14888
MLLT3	1.05	0.161827103	-0.01	127	-0.63	8957	0.06	122	4.47	5629	-0.04	135	-2.98	10171
B3GALT6	-1	0.923993962	-0.01	144	-0.63	8958	-0.03	117	-2.31	10029	-0.08	111	-4.88	11008
FUT11	-1.02	0.469327119	-0.01	147	-0.63	8959	-0.1	123	-7.56	12906	-0.09	116	-5.65	11296
GHRH	1.03	0.109379583	-0.01	111	-0.64	8960	-0.07	100	-3.52	10817	0.09	104	5.01	5919
ANKAR	1.08	0.017258291	-0.03	72	-0.64	8961	0.02	92	0.76	7967	0.08	72	1.7	7626
FAT2	1.05	0.000478331	-0.01	116	-0.64	8962	-0.1	127	-7.72	13000	0.06	129	5.04	5904
ZCCHC5	1.01	0.181501672	-0.01	120	-0.64	8963	0.09	127	7.01	4240	0.07	126	5.46	5709
ZNF226_t2	1.2	0.001028975	-0.01	125	-0.64	8964	0.08	129	6.67	4414	-0.01	107	-0.59	8977
KALRN_t1	1.1	0.0539773	-0.01	156	-0.64	8965	0.07	131	6.02	4768	0.09	134	7.97	4633
LHPP	1.04	0.476896124	-0.01	127	-0.64	8966	-0.07	117	-4.94	11621	0.02	134	1.97	7466
SLC39A9	1.1	0.000522104	-0.01	120	-0.65	8967	0.02	147	2.05	7110	0.05	132	4.31	6281
TMEM252	1.06	2.35E-05	-0.01	127	-0.65	8968	-0.03	128	-2.73	10292	0.01	115	0.58	8269
FARS2	1.1	0.001515109	-0.01	100	-0.66	8969	-0.08	108	-4.68	11481	-0.03	92	-1.44	9415
EIF3A	-1.03	0.728087071	-0.01	149	-0.66	8970	0.1	127	7.92	3785	0.04	134	3.55	6639
CRIPAK	1.13	0.000296928	-0.01	104	-0.66	8971	-0.01	80	-0.26	8645	0.1	124	7.64	4773
CILP2	1.03	0.017891958	-0.01	107	-0.66	8972	0	117	-0.2	8613	-0.09	95	-3.89	10585
FST	-1.69	0.000144904	-0.01	126	-0.66	8973	-0.12	113	-7.77	13016	0	114	-0.2	8732
MLL5	-1.02	0.609300065	-0.01	113	-0.66	8974	0.09	130	6.86	4319	-0.02	129	-1.87	9650
PTPRT_t1	1.02	0.277160127	-0.01	121	-0.66	8975	NA	NA	NA	NA	0.07	135	5.94	5474
RFX1_t1	1.03	0.017308023	-0.01	114	-0.66	8976	NA	NA	NA	NA	0.04	130	3.28	6782
DNAAF3	1.01	0.35815411	-0.01	107	-0.67	8977	-0.11	115	-7.31	12798	0	119	0.23	8475
RAVER1	1.04	0.000522104	-0.01	115	-0.67	8978	0.09	127	6.97	4259	0.02	107	1.22	7901
PECAM1	-1.1	0.105060355	-0.01	131	-0.67	8979	0.07	146	6.39	4570	-0.11	141	-10.39	13049
DNAJC11	1.06	0.002024347	-0.01	126	-0.67	8980	-0.07	121	-5.16	11755	0.03	119	1.78	7576
ALPI	1.01	0.739874085	-0.01	114	-0.67	8981	-0.13	130	-10.8	14175	-0.04	103	-2.27	9829
PPCS_t1	-1.22	3.26E-06	-0.01	111	-0.68	8982	-0.16	116	-10.28	13995	NA	NA	NA	NA
TMEM145	1.2	9.99E-06	-0.02	88	-0.68	8983	-0.03	123	-2.13	9918	0	106	0.12	8544
SOLH_t1	-1	0.808261883	-0.01	111	-0.68	8984	NA	NA	NA	NA	-0.09	106	-4.92	11020
CXCL13	-1.01	0.907231554	-0.01	133	-0.68	8985	0.07	124	5.53	5049	-0.15	133	-12.14	13590
CTSC_t2	-1.13	0.038882573	-0.01	112	-0.68	8986	0.04	106	2.03	7127	-0.11	131	-8.83	12535
ST3GAL4	-1.2	0.000139178	-0.01	110	-0.68	8987	-0.06	113	-3.88	11022	-0.12	118	-8.19	12310
KBTBD5	-1.01	0.364118357	-0.01	111	-0.69	8988	-0.15	102	-7.95	13093	0.04	96	1.78	7577
AHSA2	1.11	0.036459147	-0.01	125	-0.69	8989	0.06	136	5.41	5112	0.04	154	4.24	6322
SLCO4C1	1.01	0.127000415	-0.01	140	-0.69	8990	-0.06	132	-4.82	11547	0.08	121	5.35	5758
CCL11	1	0.997399572	-0.01	99	-0.69	8991	0.06	105	3.49	6190	-0.17	131	-13.39	13952
KLRC1_t2	-1.3	2.33E-06	-0.01	114	-0.69	8992	-0.04	127	-2.75	10313	-0.19	135	-15.8	14538
TRIM17	1.04	0.000616723	-0.01	104	-0.69	8993	0.07	124	5.08	5282	0.02	100	0.99	8024
USH1G	1.06	1.84E-08	-0.01	108	-0.69	8994	0.14	133	11.65	2255	0.13	122	9.22	4152
PAK3	1.02	0.206658271	-0.01	116	-0.69	8995	0.04	148	3.6	6115	0.02	143	2.08	7408
ANXA9	1.16	0.021379022	-0.01	115	-0.69	8996	-0.12	120	-8.17	13180	0.09	96	4.03	6404
SCN3B_t1	1.01	0.640703362	-0.01	126	-0.69	8997	NA	NA	NA	NA	NA	NA	NA	NA
TNP1	1.02	0.096359428	-0.02	89	-0.7	8998	0.04	104	2.32	6941	0	104	0.17	8503
PRPF6	1.14	0.000230381	-0.02	87	-0.7	8999	0	86	-0.01	8497	-0.03	90	-1	9204
ATPAF2	-1.06	0.075022305	-0.01	115	-0.71	9000	-0.07	125	-5.26	11803	-0.01	152	-0.52	8930
GRIN3A	-1.03	0.15832093	-0.01	163	-0.71	9001	-0.06	147	-5.89	12131	-0.12	136	-10.13	12969
TIMP4	1.06	0.068653271	-0.01	100	-0.71	9002	0.06	108	3.73	6042	-0.14	103	-7.6	12070
KRTAP13-2	1.02	0.04592033	-0.01	101	-0.71	9003	-0.01	103	-0.28	8658	-0.04	102	-1.84	9634
CHRDL1	1.06	0.111233528	-0.01	130	-0.71	9004	-0.05	136	-4.69	11484	0.17	124	12.35	3086
PCID2	1.09	0.022788708	-0.01	113	-0.71	9005	0.09	113	5.98	4795	-0.04	115	-2.37	9878
CTXN1	1	0.962211525	-0.01	100	-0.71	9006	-0.09	123	-6.7	12512	-0.04	123	-3.1	10223
FBXO34	-1.01	0.515879266	-0.01	139	-0.71	9007	-0.08	115	-4.96	11630	-0.09	120	-6.24	11548
BCS1L	1.06	0.047145883	-0.07	60	-0.71	9008	0.02	125	1.14	7720	-0.01	80	-0.4	8852
LCE3D	1.05	0.000131441	-0.01	113	-0.72	9009	-0.13	108	-7.51	12879	0.09	109	5.54	5665
TGM5	1.01	0.303492638	-0.01	112	-0.72	9010	0.08	133	6.59	4456	-0.09	130	-6.81	11776
TACO1	1.08	0.138292169	-0.01	108	-0.72	9011	-0.05	114	-3.09	10532	0.04	104	2.33	7271
KISS1R	-1.32	7.47E-08	-0.01	105	-0.72	9012	-0.12	108	-6.99	12645	-0.08	105	-4.52	10846
SCN9A	-1.16	0.025972617	-0.01	153	-0.72	9013	-0.06	152	-5.99	12177	0.03	150	3.07	6896

ERP44	1.06	0.120644369	-0.01	151	-0.72	9014	0.13	120	8.98	3281	0.06	156	6.21	5356
PSMB10	-1.29	8.30E-07	-0.02	88	-0.72	9015	-0.13	76	-3.37	10718	-0.22	79	-6.28	11563
NES	1.14	0.000426253	-0.01	104	-0.72	9016	0.12	131	9.8	2938	0	100	0.04	8596
MRPS34	1.03	0.383152588	-0.02	85	-0.73	9017	0.06	107	3.42	6237	0.04	111	2.63	7119
KLHL30	1.02	0.069936493	-0.01	125	-0.73	9018	-0.07	120	-5.13	11736	0.04	127	2.77	7045
MBP_t2	-1.2	0.001167746	-0.01	139	-0.73	9019	-0.04	131	-3.28	10661	-0.04	151	-3.85	10563
KCNB1	-1.07	0.56855788	-0.01	126	-0.73	9020	-0.07	131	-5.29	11814	-0.01	131	-0.65	9009
VPS4B_t1	-1.25	1.81E-05	-0.01	132	-0.73	9021	NA	NA	NA	NA		-0.1	154	-10.29	13022
TBC1D29	1.05	0.000400391	-0.02	90	-0.73	9022	0	66	-0.07	8533	0.09	68	1.54	7726
HSD17B3	1.01	0.859065266	-0.02	88	-0.73	9023	0	108	0.17	8357	0.01	105	0.58	8275
LEFTY2	1.02	0.02652551	-0.01	130	-0.73	9024	0.13	124	9.47	3081	0.04	123	2.89	6999
NKD1	1.45	8.54E-07	-0.01	129	-0.74	9025	-0.05	134	-3.93	11055	0.13	143	11.98	3207
QRICH1	1.01	0.67207941	-0.01	128	-0.74	9026	0.06	122	4.57	5578	-0.03	113	-1.72	9563
TEAD3	1.03	0.073567073	-0.01	132	-0.74	9027	-0.1	146	-10.01	13885	0.02	128	1.23	7890
FBN1	-1.28	0.010566799	-0.01	131	-0.74	9028	0.04	131	2.85	6590	-0.13	139	-11.59	13423
VIMP	1.07	0.11507421	-0.01	124	-0.74	9029	0.07	122	4.82	5439	0.08	129	6.46	5259
NUDT2	1.16	9.52E-06	-0.01	124	-0.74	9030	0.04	100	2.21	7008	0.04	131	2.84	7021
SLC27A4	-1.03	0.357359809	-0.01	109	-0.74	9031	0	120	-0.3	8684	-0.04	103	-1.96	9696
ADAL	1.02	0.653577575	-0.01	125	-0.74	9032	-0.09	136	-7.97	13102	0.13	109	7.88	4670
IL1R2_t1	1.08	0.185837144	-0.02	85	-0.74	9033	NA	NA	NA	NA		0.07	60	0.67	8219
ERG_t1	1.11	0.010468391	-0.01	129	-0.75	9034	NA	NA	NA	NA		0.06	144	5.49	5687
POLDIP3	1.12	0.000541798	-0.01	138	-0.75	9035	0.04	125	3.21	6372	0.01	140	1.28	7863
PSMB9	1.08	0.224127301	-0.01	108	-0.75	9036	0	130	0.07	8424	-0.09	114	-5.97	11431
NLRP11	1.25	2.55E-05	-0.01	126	-0.75	9037	-0.11	115	-7.08	12684	0.06	113	4.09	6379
NME3	1.1	0.020304156	-0.01	105	-0.75	9038	0.12	152	12.28	2039	0.06	123	4.44	6223
NTMT1	-1.04	0.335817354	-0.02	86	-0.76	9039	0.03	113	1.58	7444.5	-0.08	88	-3.18	10255
TMEM200A	1.02	0.697324589	-0.01	149	-0.76	9040	0.1	135	8.67	3426	0	123	0.13	8539
ADRB3	1.01	0.676021069	-0.01	120	-0.76	9041	-0.15	144	-13.9	14971	-0.08	127	-6.42	11614
SRSF11	-1.13	0.08161502	-0.01	141	-0.76	9042	0.06	116	3.72	6050	-0.03	130	-2.33	9861
SERPINB6	-1.12	0.004377651	-0.02	84	-0.76	9043	-0.05	142	-4.9	11596	-0.07	90	-2.79	10081
ECHDC1_t1	-1.18	0.003144273	-0.01	138	-0.76	9044	-0.01	126	-0.4	8745	NA	NA	NA	NA
DPP6	1	0.997028928	-0.01	123	-0.76	9045	-0.01	124	-0.6	8873	0.06	138	5.45	5713
LIG4	1.01	0.534791981	-0.01	150	-0.76	9046	0.02	129	1.66	7382	0.08	131	6.52	5225
RARS2	-1.09	0.039021558	-0.05	64	-0.76	9047	-0.02	64	-0.32	8696	-0.04	92	-1.8	9612
MST4	1.11	0.221136774	-0.01	143	-0.77	9048	0.14	129	11.25	2381	0.12	136	9.93	3901
TNNI1	1.04	0.006013795	-0.01	132	-0.77	9049	-0.09	158	-10.04	13898	0	148	0.11	8547
REV1_t2	-1.12	8.41E-05	-0.01	135	-0.77	9050	-0.06	120	-4.51	11395	-0.03	122	-2.37	9880
XIRP1	1.03	0.016506385	-0.01	108	-0.77	9051	-0.08	109	-4.64	11457	0.01	127	0.9	8075
TRPC7	1.02	0.081019924	-0.02	98	-0.77	9052	-0.11	120	-8.01	13121	0.03	88	1.18	7927
GNB2L1	-1.02	0.710714148	-0.02	82	-0.77	9053	0.03	68	0.6	8074	-0.07	76	-1.75	9585
HIF3A	1	0.786378122	-0.01	131	-0.77	9054	0.02	148	1.68	7366	0.02	127	1.55	7718
GLI1	1.03	0.603483748	-0.01	104	-0.77	9055	0.09	113	5.6	5008	-0.04	103	-2.04	9724
EFNA5	1.04	2.48E-05	-0.01	133	-0.77	9056	0.06	151	6	4786	0.03	147	3.31	6768
SPACA5	-1.01	0.515661562	-0.02	88	-0.78	9057	0.06	92	2.69	6685	-0.02	104	-1.27	9328
ADD1_t2	1.04	0.208150025	-0.01	124	-0.78	9058	0.07	124	5.43	5102	0.02	135	1.72	7618
FAM57B	1.1	5.35E-05	-0.01	107	-0.78	9059	0	108	-0.27	8657	0.05	133	4.33	6272
RSF1_t1	-1.07	0.02032763	-0.02	102	-0.78	9060	NA	NA	NA	NA		0.01	122	0.4	8369
ALG12	1.06	0.003751214	-0.02	84	-0.78	9061	-0.03	124	-2.23	9988	-0.02	114	-1.43	9406
RGS8	1.02	0.024570667	-0.02	100	-0.79	9062	0.06	110	3.48	6196	0.06	88	2.29	7300
VASH1	-1.01	0.440694457	-0.01	137	-0.79	9063	0	144	0.11	8396	-0.15	132	-12.58	13722
CDK18	1.03	0.088600936	-0.01	131	-0.79	9064	-0.01	120	-0.48	8801	0.03	124	1.93	7495
GAS2L2_t1	1.01	0.332118095	-0.02	101	-0.79	9065	NA	NA	NA	NA	NA	NA	NA	NA
S100A5	-1.04	0.09701299	-0.02	87	-0.79	9066	-0.07	126	-5.51	11928	0.08	95	3.45	6694
SERGEF	1.01	0.814757627	-0.04	68	-0.79	9067	-0.1	70	-2.05	9860	-0.02	81	-0.61	8989
SST	1.05	0.188243635	-0.03	81	-0.79	9068	-0.02	113	-1.25	9330	0.11	128	8.3	4496
OR10C1	1.01	0.328889866	-0.03	80	-0.79	9069	-0.11	84	-3.81	10988	0	61	0.02	8609
UBR3	-1.08	0.097994256	-0.01	140	-0.79	9070	-0.01	117	-0.56	8853	0.07	146	6.68	5161
ERF	-1.11	0.004445784	-0.01	134	-0.8	9071	-0.05	126	-3.89	11030	-0.05	112	-3.12	10234
NR2E3	1.02	0.037543663	-0.01	115	-0.8	9072	0.04	108	2.36	6915	0.01	127	0.43	8352
TTC29	1.05	0.01633253	-0.01	136	-0.8	9073	0.08	101	3.84	5983	0.07	116	4.44	6219
AK2_t1	-1.03	0.529589779	-0.01	129	-0.8	9074	NA	NA	NA	NA	NA	NA	NA	NA
TLX3	-1.01	0.630087468	-0.01	114	-0.8	9075	-0.14	113	-8.73	13408	-0.06	146	-6.19	11518
RAB13	1.16	1.75E-05	-0.01	136	-0.8	9076	0.05	119	3.3	6317	0.15	123	11.3	3415
SSX2	1.09	0.000245384	-0.01	108	-0.8	9077	0.04	128	3.01	6491	0.02	127	1.52	7736
PAQR7	1.02	0.055696403	-0.01	107	-0.81	9078	-0.01	144	-0.69	8935	-0.09	119	-6.25	11549
CCDC134	1.02	0.184410873	-0.02	94	-0.81	9079	-0.08	95	-3.6	10881	0.02	94	0.95	8045
SERTAD2_t1	1.01	0.521565583	-0.01	147	-0.81	9080	NA	NA	NA	NA		0.08	153	8.44	4450
ZMYND10	1.09	1.90E-06	-0.01	105	-0.81	9081	-0.03	116	-1.83	9712	0.02	107	0.88	8094
ANKRD12_t1	-1.1	0.011677693	-0.01	135	-0.82	9082	NA	NA	NA	NA	NA	NA	NA	NA
BPIFB1	1.04	0.053147451	-0.02	104	-0.82	9083	0.01	107	0.75	7970	-0.04	93	-1.69	9545
ALX1	1.01	0.346963036	-0.02	102	-0.82	9084	-0.05	123	-3.7	10932	-0.01	131	-0.87	9123
ESRP1	1.05	0.237512856	-0.01	154	-0.82	9085	-0.1	136	-8.37	13273	0.1	147	9.84	3942
RPA4	-1.05	0.22624876	-0.01	130	-0.82	9086	-0.01	106	-0.43	8768	0.03	127	2.16	7363
PEF1	1.02	0.466096864	-0.01	141	-0.82	9087	-0.01	108	-0.32	8693	0.03	149	2.63	7121
MICAL1	-1.13	0.053819922	-0.02	99	-0.82	9088	0.13	94	5.79	4899	-0.17	88	-6.51	11654
PLIN3	1	0.97766437	-0.01	106	-0.83	9089	0.04	139	3.78	6022	-0.17	150	-16.58	14709
GP6	-1.02	0.237481611	-0.01	124	-0.83	9090	-0.13	131	-10.33	14015	-0.11	122	-7.57	12056
DDX42_t1	1.01	0.515440699	-0.01	112	-0.83	9091	NA	NA	NA	NA		0.05	127	3.53	6658
CD1B	1.01	0.689932227	-0.01	110	-0.83	9092	0.08	114	5.18	5228	-0.18	112	-10.88	13212
SARM1	1.07	0.045449108	-0.01	119	-0.83	9093	0.09	149	8.92	3322	-0.09	136	-8.05	12249
BANP_t2	1	0.960843398	-0.01	113	-0.83	9094	0.02	111	1.38	7560	0.07	108	3.82	6517
DCDC1	1	0.747940863	-0.01	110	-0.83	9095	-0.01	123	-0.85	9048	0.08	118	5.53	5670
KLC3	1.03	0.03177971	-0.01	109	-0.83	9096	-0.08	88	-3.16	10582	0.06	104	3.26	6800
EIF1AY	-1.15	0.058850233	-0.01	117	-0.83	9097	0.04	117	2.44	6865	-0.02	110	-1.04	9222
RAD23B_t1	-1.06	0.230757784	-0.01	138	-0.83	9098	NA	NA	NA	NA		0.05	144	4.53	6163
S100A13	-1.14	0.078891927	-0.01	108	-0.83	9099	0.05	113	2.92	6540	-0.18	100	-8.97	12583
CCDC50_t2	-1.09	0.016531338	-0.01	145	-0.83	9100	0	152	0.13	8378	-0.08	151	-8.4	12398
ADCY7_t1	-1.35	5.59E-06	-0.01	133	-0.83	9101	0.1	130	8.34	3580	NA	NA	NA	NA
C1QBP	-1.16	0.000574419	-0.02	104	-0.84	9102	-0.12	88	-4.46	11367	-0.09	99	-4.46	10826
SULT1B1	-1.02	0.813145199	-0.01	109	-0.84	9103	-0.06	104	-3.51	10801	0	80	-0.06	8655
HNRNPD_t2	-1.09	0.001137633	-0.01	109	-0.84	9104	-0.07	110	-3.98	11093	-0.16	130	-12.81	13795
DENND4A	-1.22	0.000473526	-0.01	150	-0.84	9105	-0.04	126	-2.74	10301	-0.03	120	-1.85	9636
TARS2	1.16	0.0001051	-0.02	106	-0.84	9106	-0.02	122	-1.58	9545	0.06	117	3.96	6433
POLR2B	-1.04	0.435504088	-0.01	109	-0.85	9107	0.1	90	4.01	5898	0.01	104	0.58	8272
THUMPD3	1.13	0.001267303	-0.01	153	-0.85	9108	0.04	143	3.5	6186	0.22	150	21.73	1013
EFHA1	-1.1	0.170063041	-0.01	142	-0.85	9109	0.03	129	2	7147	-0.06	128	-4.77	10971
TMCC2_t1	-1.04	0.003180644	-0.01	128	-0.85	9110	NA	NA	NA	NA		-0.02	112	-1.37	9377
TNNI2	1.08	0.125353716	-0.02	104	-0.85	9111	0.15	100	7.54	3968	-0.08	100	-3.88	10584
NMRAL1	-1.12	0.070724592	-0.03	80	-0.85	9112	0.01	92	0.33	8255	-0.08	102	-3.9	10590
ATP11A_t2	1.08	0.000667976	-0.01	135	-0.86	9113	NA	NA	NA	NA	NA	NA	NA	NA
RCOR1	1.04	0.001320036	-0.01	153	-0.86	9114	0.07	143	6.22	4652	0.05	147	4.95	5954
GUCA1A	1.03	0.001377702	-0.01	136	-0.86	9115	-0.07	138	-5.74	12053	-0.04	138	-3.91	10595
SENP8	1.01	0.672308	-0.01	156	-0.86	9116	-0.02	110	-1.07	9193	0.11	135	9.02	4221
NUAK2	-1.03	0.606031651	-0.01	125	-0.86	9117	0.01	145	0.59	8080	-0.12	132	-9.56	12774
NKPD1	1.04	0.006595567	-0.01	133	-0.86	9118	-0.03	128	-2.22	9980	0.01	130	0.52	8306
SRPK3_t1	-1.02	0.133569185	-0.02	97	-0.86	9119	-0.1	104	-5.49	11909	NA	NA	NA	NA

GTF3C1	1.04	0.039025964	-0.01	128	-0.86	9120	0.03	138	2.29	6965	-0.01	115	-0.79	9082
ENC1_t1	-1.38	6.51E-06	-0.01	146	-0.86	9121	NA	NA	NA	NA	-0.13	127	-10.23	13001
ADAM11_t1	1.03	0.016864577	-0.01	124	-0.86	9122	NA	NA	NA	NA	-0.04	146	-3.62	10448
SLITRK5	1.01	0.371281358	-0.01	132	-0.86	9123	0.1	126	7.63	3917	-0.02	138	-1.38	9380
DOHH	1.07	0.013720143	-0.02	101	-0.86	9124	-0.07	104	-3.76	10959	-0.01	117	-0.9	9139
MOK	-1.02	0.618364563	-0.01	132	-0.87	9125	0.14	121	9.81	2928	-0.13	148	-12.58	13723
EXT1	-1.03	0.596581299	-0.02	94	-0.87	9126	0.01	100	0.26	8291	-0.02	101	-0.81	9094
BFAR_t1	-1.12	8.06E-05	-0.01	150	-0.87	9127	NA	NA	NA	NA	NA	NA	NA	NA
DDX39B_t1	-1.11	1.59E-06	-0.03	84	-0.87	9128	NA	NA	NA	NA	-0.16	108	-9.32	12689
ZAN	1.03	0.073564247	-0.01	138	-0.87	9129	-0.05	86	-1.93	9764	-0.06	98	-3.02	10184
CNKSR1	1.02	0.124405787	-0.01	121	-0.87	9130	-0.06	115	-3.96	11077	-0.03	100	-1.54	9466
RNF10	-1.01	0.720545544	-0.01	137	-0.87	9131	-0.05	117	-3.04	10494	-0.04	129	-3.01	10183
UBIAD1	1.11	0.005496834	-0.01	142	-0.87	9132	-0.03	131	-2.39	10080	0.06	140	5.25	5800
TMEM41A	1.01	0.698727774	-0.01	136	-0.87	9133	-0.03	139	-2.39	10077	-0.01	131	-0.59	8983
OR10H1	1.05	4.51E-05	-0.02	95	-0.88	9134	-0.02	115	-1.48	9481	-0.06	103	-3.19	10267
TTC21A	-1.03	0.513958165	-0.03	80	-0.88	9135	0.01	122	0.84	7906	-0.07	90	-2.74	10053
CDR1	1.04	0.222263784	-0.01	134	-0.88	9136	0.03	80	1.03	7790	-0.06	95	-2.65	10016
TAOK2_t1	1.14	8.14E-06	-0.02	108	-0.88	9137	0.12	104	6.64	4429	0.07	100	3.67	6577
WNT1	1.05	6.60E-05	-0.02	108	-0.88	9138	-0.03	161	-3.02	10485	0.03	147	2.91	6985
SLC29A4	1.15	0.001091985	-0.02	104	-0.88	9139	0.05	124	3.44	6223	0.08	106	4.41	6242
EPB41_t2	1.01	0.761738131	-0.01	131	-0.88	9140	0.05	127	4.19	5787	0.03	145	2.47	7197
LDHA	-1.13	0.062219772	-0.01	125	-0.88	9141	-0.06	116	-4.16	11187	-0.01	125	-0.46	8889
FAM155B	1.06	0.076329988	-0.01	130	-0.88	9142	-0.02	145	-1.76	9662	0.02	140	1.67	7637
QPCT	-1.12	0.173146661	-0.01	110	-0.89	9143	0	112	0.11	8397	-0.13	112	-8.03	12243
ZNF3	1.13	2.89E-05	-0.01	123	-0.89	9144	0.04	126	2.8	6616	0.09	142	8.29	4497
KLHDC8B	1.03	0.508880655	-0.01	120	-0.89	9145	0.02	125	1.14	7711	-0.02	112	-1.08	9233
SLC25A1	-1.01	0.910217412	-0.02	108	-0.89	9146	-0.06	113	-3.8	10980	-0.04	104	-2.06	9738
SLC9A2	1.03	0.001750638	-0.01	132	-0.89	9147	0.08	125	6.05	4748	0.02	152	2.11	7395
RNASE6	-1.31	4.00E-05	-0.02	108	-0.89	9148	0.05	106	3	6492	-0.18	127	-13.9	14080
PCDH10_t2	-1.04	0.019542573	-0.01	111	-0.89	9149	-0.13	136	-10.9	14206	0.02	131	1.42	7794
AIFM3	1.13	0.000324492	-0.02	106	-0.89	9150	0.09	104	4.67	5509	0.01	117	0.72	8185
FLCN_t2	1.05	0.027935944	-0.01	114	-0.89	9151	0	126	0	8472	0.06	130	4.73	6064
OR56B1	1.02	0.04993619	-0.01	131	-0.89	9152	0.03	82	1	7809	-0.02	91	-1.01	9211
CD300LG	1.01	0.824067535	-0.01	136	-0.89	9153	-0.05	129	-3.62	10892	-0.01	132	-1.17	9276
MRPS5	-1.05	0.13586604	-0.02	101	-0.9	9154	-0.11	104	-6.05	12212	-0.07	107	-3.98	10630
COL4A6	1.14	0.011496478	-0.01	118	-0.9	9155	0.02	136	2.03	7123	0.04	123	3.23	6812
GAA	-1.01	0.76333447	-0.02	104	-0.9	9156	-0.12	104	-6.3	12321	0.03	119	1.85	7531
HIPK1_t2	-1.04	0.002640689	-0.06	64	-0.9	9157	-0.24	86	-8.54	13342	0	74	-0.02	8635
CHD2_t1	-1.03	0.048683217	-0.02	96	-0.9	9158	0.01	112	0.51	8132	0	103	0.1	8555
GOT1L1	1.01	0.521936305	-0.04	72	-0.9	9159	0.02	50	0	8471	0.04	102	1.82	7555
CYB561D2	1.18	1.49E-07	-0.01	120	-0.9	9160	0.1	104	5.16	5247	0.13	128	9.79	3953
GAPDHS	-1	0.98730841	-0.03	78	-0.9	9161	-0.05	92	-2.09	9887	-0.11	76	-2.86	10119
NXPH2	1	0.70268487	-0.01	128	-0.9	9162	0.03	128	2.27	6974	-0.19	150	-18.86	15148
SLC30A9	-1.02	0.526723571	-0.01	121	-0.9	9163	-0.02	138	-1.43	9450	0.09	134	7.25	4927
KLHL15	-1.06	2.69E-07	-0.01	144	-0.91	9164	-0.11	137	-9.38	13653	-0.09	148	-9.07	12615
RPL11	-1.06	0.178543606	-0.09	60	-0.91	9165	-0.02	84	-0.79	9012	-0.08	80	-2.47	9934
COASY	1.13	0.000281852	-0.01	115	-0.91	9166	0.06	104	3.05	6471	0.01	94	0.42	8356
PFKP	-1.03	0.727110791	-0.02	103	-0.91	9167	0.03	122	2.27	6978	-0.14	101	-7.39	11992
HPSE	-1.15	9.43E-05	-0.01	152	-0.91	9168	-0.02	127	-1.33	9381	-0.2	128	-15.22	14422
CCDC82	-1.11	0.032703402	-0.01	123	-0.91	9169	0.08	134	6.33	4601	-0.01	125	-0.77	9064
TNNC2	1.06	0.061630248	-0.03	86	-0.91	9170	-0.13	112	-8.32	13246	0.03	82	1.09	7977
LZTR1	1.04	0.029641933	-0.01	123	-0.92	9171	0.05	117	3.42	6235	-0.06	133	-5.18	11112
ITGA3	1.02	0.645052471	-0.01	120	-0.92	9172	0.08	123	5.89	4850	-0.08	123	-6.12	11487
PRKAA1	-1.13	0.008275061	-0.01	156	-0.92	9173	-0.05	124	-3.4	10728	0	168	-0.52	8928
ATP4B	1.01	0.326839835	-0.02	108	-0.92	9174	-0.07	135	-6.02	12192	-0.02	145	-1.92	9682
GIF	1.03	0.000891204	-0.02	88	-0.92	9175	0.02	113	1.3	7610	0.14	108	8.24	4518
OSBPL2_t1	1.03	0.299994438	-0.01	142	-0.92	9176	NA	NA	NA	NA	-0.06	131	-5.15	11095
DDX25	-1.02	0.060515968	-0.01	126	-0.92	9177	-0.07	92	-2.84	10364	-0.05	98	-2.2	9795
KLF3	-1.01	0.679032874	-0.01	136	-0.92	9178	-0.03	126	-2.16	9937	-0.02	168	-2.54	9974
TFF2	-1.01	0.927725141	-0.01	121	-0.92	9179	-0.13	126	-10.12	13928	-0.13	90	-5.12	11089
SOCS1_t1	-1.08	0.215171903	-0.01	146	-0.92	9180	0.05	124	3.86	5972	NA	NA	NA	NA
TMEM59L	1	0.894248706	-0.01	139	-0.93	9181	-0.11	116	-6.95	12625	0.01	115	0.79	8139
TRPC3	-1.01	0.677565577	-0.01	152	-0.93	9182	-0.01	113	-0.61	8882	-0.02	144	-1.53	9457
PNKD_t2	1.02	0.511272732	-0.01	138	-0.93	9183	-0.02	125	-1.64	9578	-0.03	122	-1.8	9614
GPAT2	1.05	2.73E-06	-0.02	107	-0.93	9184	-0.04	102	-2.1	9897	0.05	98	2.49	7183
SSTR4	-1.03	0.023788759	-0.09	60	-0.94	9185	-0.13	84	-4.56	11419	-0.07	78	-2.06	9741
PRR15L	-1.15	0.250279642	-0.01	120	-0.94	9186	0.02	126	1.8	7273	-0.13	130	-10.39	13045
GNB2	-1.08	0.007667641	-0.02	105	-0.94	9187	-0.03	124	-2.18	9946	-0.05	99	-2.57	9988
CRYGN	-1	0.669688777	-0.01	115	-0.94	9188	-0.06	81	-1.98	9802	-0.02	100	-1.19	9289
SP110	-1.16	0.000323194	-0.02	89	-0.94	9189	-0.06	104	-2.99	10466	-0.18	116	-11.81	13489
ANKRD42	-1.06	0.016828322	-0.01	120	-0.94	9190	-0.01	138	-0.97	9129	-0.03	147	-2.85	10112
STIM2	-1.04	0.010239256	-0.01	130	-0.94	9191	0.07	128	5.76	4920	-0.02	145	-1.44	9414
LRRC18	-1.02	0.136833284	-0.01	132	-0.94	9192	-0.11	113	-6.65	12492	-0.06	127	-4.59	10891
ADRA2C	1.2	0.063800248	-0.01	115	-0.95	9193	0	120	0.03	8443	0	97	0.01	8613
BRWD1_t2	-1.09	0.104872981	-0.01	134	-0.95	9194	-0.06	118	-4.23	11225	-0.04	143	-4.13	10689
ARMC4	1.08	0.05510503	-0.01	115	-0.95	9195	-0.09	120	-6.04	12208	-0.04	123	-2.77	10072
N4BP1	-1.05	0.007655782	-0.01	151	-0.95	9196	0.01	133	0.75	7973	-0.04	143	-3.66	10465
GPR37L1	1.04	0.007011735	-0.02	113	-0.95	9197	-0.08	135	-7.02	12662	-0.02	120	-1.15	9273
ZNF593	1.03	0.336435036	-0.02	92	-0.95	9198	0.03	77	0.86	7893	0.01	96	0.49	8322
MBIP	-1.09	0.082542354	-0.01	118	-0.95	9199	-0.08	116	-5.24	11795	-0.05	121	-3.83	10547
RBAK_t1	1.01	0.335065464	-0.01	133	-0.96	9200	0.05	137	4.58	5566	NA	NA	NA	NA
TMEM35	1.02	0.596581299	-0.01	125	-0.96	9201	0.07	125	4.97	5360	0.03	119	1.9	7511
DNAH1	-1	0.759083542	-0.07	64	-0.97	9202	-0.07	99	-3.35	10708	0.01	78	0.23	8473
GPR183	-1.81	9.57E-08	-0.02	109	-0.97	9203	0.03	111	2.1	7081	-0.17	113	-10.87	13206
TIGD4	-1.02	0.009205861	-0.03	88	-0.97	9204	0.01	100	0.33	8251	-0.1	112	-6.31	11576
MEX3D	1.11	0.006328001	-0.02	112	-0.97	9205	0.03	115	1.69	7361	-0.02	140	-1.83	9625
SLC25A16	1.02	0.674534413	-0.01	151	-0.98	9206	0	124	-0.15	8578	0.07	130	5.58	5632
CACNB1_t2	1.03	0.002395243	-0.02	90	-0.98	9207	0.13	88	4.93	5378	-0.03	94	-1.43	9407
CHCHD5	1.13	2.24E-05	-0.02	103	-0.98	9208	0.05	100	2.34	6931	0.01	80	0.41	8362
SLC38A10_t2	1.14	0.004160972	-0.02	112	-0.98	9209.5	-0.03	139	-2.99	10468	0	120	0.24	8467
RBCK1	1.12	0.00072664	-0.02	112	-0.98	9209.5	0.03	129	2.54	6804	-0.02	120	-1.07	9227
TRIAP1	-1.03	0.489457478	-0.01	138	-0.98	9211	-0.1	127	-7.95	13092	0.04	120	2.69	7091
ZFP82	1.01	0.712024509	-0.01	137	-0.98	9212	0.19	130	15.45	1156	0	99	-0.24	8760
LYZL6	-1.02	0.065780991	-0.01	123	-0.98	9213	-0.07	121	-5.31	11827	0.06	100	2.91	6982
ZNF558	1.08	0.092895751	-0.01	120	-0.98	9214	0.09	133	7.59	3937	0.05	122	3.59	6621
USHBP1_t1	-1.02	0.010276771	-0.02	109	-0.99	9215	NA	NA	NA	NA	-0.03	118	-1.88	9660
TEX11	1.5	9.89E-08	-0.02	100	-0.99	9216	0.06	90	2.3	6955	0.22	98	10.46	3704
CIB2	1.02	0.610560113	-0.01	118	-0.99	9217	0.03	116	2.27	6973	-0.04	117	-2.7	10039
IZUMO2	-1.04	0.019106724	-0.05	72	-1	9218	-0.1	95	-4.38	11306	-0.11	79	-3.27	10297
SLC9C2	1.01	0.173757922	-0.02	114	-1	9219	-0.09	124	-6.45	12393	0.07	142	6.05	5429
SPIRE2	1	0.954195126	-0.02	116	-1	9220	-0.03	135	-2.27	10006	0.02	128	1.24	7883
RPS14_t2	1.16	8.30E-05	-0.02	91	-1	9221	0.11	92	4.73	5490	0.02	114	1.47	7768
ARHGEF16	-1.23	0.031968551	-0.02	91	-1	9222	0	124	0.23	8299	-0.09	100	-4.29	10750
TTLL6_t1	1.03	0.004554789	-0.02	106	-1	9223	NA	NA	NA	NA	NA	NA	NA	NA
FAM19A2	1.02	0.208131464	-0.01	139	-1	9224	0.04	132	3.07	6455	-0.07	139	-6.63	11709
EIF3G	1.03	0.357249181	-0.02	100	-1.01	9225	-0.08	109	-4.73	11502	-0.1	104	-5.55	11258

REN	1.06	0.000145332	-0.02	96	-1.01	9226	0.14	130	11.18	2407	0.13	101	6.7	5157
TXK_t1	-1.04	0.001814596	-0.02	116	-1.01	9227	NA	NA	NA	NA	-0.12	127	-9.47	12752
PSMB7	-1.04	0.265856902	-0.02	94	-1.01	9228	-0.05	128	-3.53	10819	-0.07	84	-2.34	9863
NARFL	1.01	0.780708339	-0.01	127	-1.01	9229	0	108	-0.05	8521	-0.05	93	-2.05	9730
DNHD1_t2	-1.01	0.887236516	-0.02	93	-1.01	9230	-0.02	84	-0.62	8886	-0.07	105	-3.79	10533
WFDC12	1	0.993284873	-0.02	106	-1.01	9231	-0.06	106	-3.21	10611	-0.01	122	-0.56	8955
IFT20	1.06	0.082069227	-0.02	110	-1.01	9232	0.05	136	4.27	5729	-0.01	114	-0.92	9147
MYOF_t2	1	0.876516318	-0.01	123	-1.01	9233	0.04	116	2.76	6635	-0.08	111	-4.73	10955
MBOAT7	1.04	0.351271085	-0.02	110	-1.01	9234	0.07	127	5.24	5196	-0.08	134	-6.96	11829
FAM71D_t1	-1	0.915068357	-0.02	104	-1.01	9235	NA	NA	NA	NA	-0.04	100	-1.93	9686
YBX2	1.2	0.001902639	-0.02	116	-1.02	9236	-0.04	100	-1.92	9756	0.06	96	2.69	7092
FAM123B	1.13	0.015675722	-0.01	126	-1.02	9237	0	140	-0.34	8709	-0.01	144	-0.76	9060
SALL4	1.07	0.38927016	-0.02	100	-1.02	9238	-0.02	103	-0.86	9050	-0.06	106	-3.3	10309
KCND2	1.03	0.168357126	-0.01	148	-1.02	9239	0.04	111	2.71	6670	-0.02	150	-2.14	9773
ZNF415	-1.1	0.049872642	-0.01	118	-1.02	9240	0.07	124	5.28	5178	-0.04	121	-2.7	10040
KAT5	-1	0.96577961	-0.03	83	-1.02	9241	0	108	-0.03	8509	-0.09	102	-4.77	10973
AFF1	-1.05	0.038593735	-0.01	135	-1.02	9242	-0.08	146	-8.07	13137	-0.04	152	-3.66	10460
PLCZ1	1.02	0.110992307	-0.04	77	-1.02	9243	-0.03	86	-1.2	9289	0.02	90	0.79	8143
KCTD7	1.04	1.42E-06	-0.01	144	-1.02	9244	0.2	147	19.03	513	0	145	0.03	8603
LBX2	-1.01	0.770094293	-0.01	132	-1.02	9245	-0.17	113	-10.74	14153	-0.06	105	-3.43	10368
DOLPP1	1.09	0.041905045	-0.02	114	-1.03	9246	0.02	114	1.37	7565	0.02	116	1.26	7876
BNC2_t1	1.02	0.167326648	-0.01	134	-1.03	9247	0.08	143	7.49	3995	NA	NA	NA	NA
LY6G5B	1.05	3.43E-05	-0.02	100	-1.03	9248	0	118	-0.12	8566	-0.03	120	-2.44	9914
IDH1	-1.1	0.165279512	-0.02	111	-1.03	9249	-0.08	136	-6.56	12445	0	138	0.2	8491
EIF4B_t1	1.01	0.786619321	-0.01	127	-1.03	9250	NA	NA	NA	NA	-0.04	125	-2.83	10101
MBTPS2	1.01	0.518173584	-0.01	131	-1.03	9251	-0.04	123	-2.59	10210	0.03	140	3.12	6865
NPIP_t1	-1	0.933736872	-0.03	89	-1.03	9252	NA	NA	NA	NA	-0.07	94	-3.12	10229
LPAR2	-1.01	0.618043321	-0.02	109	-1.03	9253	0.03	142	2.42	6879	-0.08	118	-5.28	11150
VWA5A_t2	-1.08	0.176995516	-0.01	138	-1.04	9254	0.13	115	8.28	3607	-0.17	126	-12.57	13716
ZNF264	-1.09	0.031614722	-0.01	138	-1.04	9255	-0.04	159	-4.4	11330	-0.09	146	-8.4	12397
ATM	-1.02	0.219627828	-0.01	144	-1.04	9256	0.11	139	10.02	2858	-0.1	153	-9.86	12878
ANAPC15	1.03	0.33735463	-0.01	120	-1.04	9257	0	106	0.04	8434	0.01	120	0.53	8299
RBM15B	1	0.960701565	-0.01	151	-1.04	9258	0.01	147	0.97	7822	0.05	120	3.32	6762
FSHB	-1.03	0.034287883	-0.01	128	-1.04	9259	-0.12	146	-11.2	14298	-0.03	127	-2.45	9922
KCNH4	1.03	0.017659372	-0.02	107	-1.04	9260	-0.09	127	-6.75	12536	0	102	0.22	8482
ERC1	1.02	0.317852316	-0.01	151	-1.04	9261	0.06	147	5.44	5095	-0.07	157	-7.68	12107
KRAS	-1.06	0.124713915	-0.01	124	-1.04	9262	0.05	137	4.11	5841	0.03	138	2.64	7114
CA5A	1.04	0.681779386	-0.02	111	-1.04	9263	-0.04	103	-2.22	9973	0.09	109	5.05	5902
RAP1GDS1	-1.03	0.502100086	-0.01	142	-1.04	9264	0.01	132	1.19	7682	0.07	123	5.13	5864
PAPD7	-1.12	0.003485158	-0.02	113	-1.05	9265	-0.06	116	-3.68	10923	-0.04	122	-3.07	10215
SCO2	-1.05	0.155646387	-0.07	65	-1.05	9266	-0.04	88	-1.42	9442	-0.14	89	-5.56	11261
UMODL1	-1.08	0.014758858	-0.01	121	-1.05	9267	-0.1	129	-8	13112	-0.05	123	-3.79	10531
PAAF1	1.1	0.00938656	-0.02	110	-1.05	9268	0.06	111	3.88	5961	-0.05	115	-3.33	10321
LRRC48	-1	0.972764927	-0.02	92	-1.05	9269	0.08	133	6.27	4630	-0.09	111	-5.21	11123
LINGO2	-1.01	0.524949916	-0.01	123	-1.05	9270	-0.01	137	-0.73	8951	-0.09	117	-6.13	11488
BATF	-1.16	0.199729457	-0.02	98	-1.05	9271	0.14	91	5.8	4893	-0.15	105	-8.28	12355
ZCCHC13	1.01	0.530433603	-0.02	102	-1.05	9272	-0.08	100	-4.03	11117	-0.13	124	-9.98	12918
GFM1	-1.02	0.727186505	-0.01	130	-1.05	9273	-0.03	134	-2.61	10225	0.05	122	3.45	6691
SH3BP4	-1.01	0.770001039	-0.02	120	-1.06	9274	-0.08	143	-7.26	12767	-0.03	134	-2.52	9963
TMEM230_t1	1.02	0.550110284	-0.01	132	-1.06	9275	NA	NA	NA	NA	NA	NA	NA	NA
TRIM49	1.04	0.000296873	-0.02	104	-1.06	9276	-0.13	119	-9.03	13522	0.08	125	6.34	5303
FGF18_t1	1.03	0.637184137	-0.01	122	-1.06	9277	0.12	157	13.13	1792	NA	NA	NA	NA
SLC19A1	1.01	0.86365699	-0.02	111	-1.06	9278	0.06	133	5.03	5314	-0.06	126	-4.91	11017
TRIML2	1.05	5.80E-05	-0.03	84	-1.06	9279	-0.01	95	-0.58	8866	0.06	83	1.88	7519
CDK11B_t1	-1.11	4.61E-06	-0.18	56	-1.06	9280	NA	NA	NA	NA	NA	NA	NA	NA
DNAI1	1.03	0.050641887	-0.02	113	-1.06	9281	-0.13	124	-9.93	13857	-0.01	151	-1.38	9384
CHCHD8	1.22	1.77E-07	-0.02	112	-1.06	9282	0.12	115	8.04	3719	0.17	118	11.8	3263
KIF5C	1.01	0.876587798	-0.01	145	-1.07	9283	0.2	132	16.29	983	0.02	159	1.99	7456
OLIG2	1.01	0.171180392	-0.01	134	-1.07	9284	-0.09	140	-8.35	13263	0.05	112	3.25	6804
RYR1_t1	-1.01	0.657264184	-0.02	115	-1.07	9285	0.05	113	2.93	6535	-0.16	105	-8.57	12444
TM7SF2_t1	-1.03	0.707651739	-0.02	104	-1.07	9286	NA	NA	NA	NA	NA	NA	NA	NA
TNFSF18	-1	0.792392947	-0.02	120	-1.07	9287	0.02	104	0.81	7930	-0.06	100	-3.03	10192
TBCK	-1.05	0.20329382	-0.02	121	-1.07	9288	-0.03	127	-2.05	9863	0	134	0.4	8376
HSP90B1	1.09	0.267608036	-0.02	117	-1.07	9289	0.06	134	4.98	5354	0.08	135	6.98	5040
ACTBL2	1.02	0.711148684	-0.01	124	-1.07	9290	0.04	128	2.95	6520	-0.08	134	-6.79	11769
NDUFA13	-1.04	0.191054363	-0.04	77	-1.07	9291	-0.05	98	-2.39	10074	-0.11	88	-4.09	10678
MYH4	1.18	0.015864525	-0.04	80	-1.07	9292	0.04	108	2.05	7113	0.02	106	0.86	8106
PCNXL4	-1.01	0.750035386	-0.01	127	-1.07	9293	0.11	116	7.23	4129	-0.04	102	-2.21	9796
TTC16	-1.02	0.221447768	-0.02	120	-1.08	9294	-0.02	121	-1.26	9333	-0.15	117	-9.76	12834
ASIC2	1.09	1.69E-07	-0.01	124	-1.08	9295	0.02	142	2.01	7137	0.05	121	3.54	6652
CLDN12	1.13	2.81E-07	-0.01	131	-1.08	9296	0.04	134	3.45	6216	0.21	119	14.58	2439
TNK1	-1.01	0.640339859	-0.02	104	-1.08	9297	-0.12	144	-11.61	14399	-0.01	140	-1.19	9286
RLN3	-1.1	3.51E-07	-0.03	84	-1.08	9298	NA	NA	NA	NA	-0.07	60	-0.72	9039
ZNF441	1.04	0.00608594	-0.01	129	-1.08	9299	0.05	131	3.7	6065	0.12	143	10.78	3576
FAM161B	1.06	4.33E-06	-0.01	139	-1.08	9300	-0.01	126	-0.74	8961	0	118	0	8622
HMCN1	1.03	0.112654403	-0.01	125	-1.08	9301	-0.01	136	-1.2	9288	0.08	135	7.21	4942
CREB3L3_t1	-1.03	0.39966723	-0.02	115	-1.08	9302	NA	NA	NA	NA	-0.04	128	-2.76	10067
GSS	1.05	0.100452058	-0.01	126	-1.09	9303	-0.03	123	-2.25	10001	-0.06	115	-4.1	10680
CST6	-1.03	0.532271185	-0.02	97	-1.09	9304	0.03	112	1.59	7433	-0.18	100	-9.16	12646
REG1A	1.64	2.75E-05	-0.02	97	-1.09	9305	-0.09	129	-7.29	12779	0.09	123	6.51	5231
FRG2	1.06	1.41E-08	-0.01	142	-1.09	9306	0.12	146	11.46	2315	-0.03	141	-2.78	10077
ATP2A3_t1	1.06	0.008161737	-0.02	120	-1.09	9307	-0.05	128	-3.63	10897	-0.03	109	-1.6	9503
EPB41L4B_t1	1.02	0.192949636	-0.01	137	-1.09	9308	-0.01	132	-0.59	8869	0.02	137	2.09	7402
RNF212	-1.01	0.493983798	-0.01	144	-1.1	9309	-0.11	134	-9.33	13629	-0.02	150	-2.5	9953
WDR90_t1	1.03	0.006890264	-0.02	118	-1.1	9310	NA	NA	NA	NA	0.03	119	1.85	7537
FAM63B_t2	-1.1	0.027088985	-0.01	136	-1.1	9311	0.05	141	4.61	5546	-0.04	133	-3.42	10363
ALDOC	-1.01	0.932165288	-0.02	109	-1.1	9312	-0.12	120	-8.32	13248	0.01	107	0.67	8218
CEP57	-1.05	0.328889866	-0.01	133	-1.1	9313	-0.02	143	-2.13	9919	-0.01	132	-0.48	8899
YTHDC2	-1.04	0.205053025	-0.01	132	-1.1	9314	0.08	124	5.57	5025	0.11	127	8.44	4451
FKBP5_t1	-1.1	0.094370208	-0.01	155	-1.11	9315	NA	NA	NA	NA	-0.02	132	-1.73	9568
KCTD14	1.02	0.578293581	-0.02	111	-1.11	9316	0.02	126	1.51	7490	-0.14	126	-10.95	13238
GNLY	-1.17	0.003101195	-0.02	100	-1.11	9317	-0.02	115	-1.3	9365	-0.16	114	-10.24	13006
PMCH	-1.11	2.07E-08	-0.02	103	-1.11	9318	-0.11	97	-5.17	11758	-0.13	104	-6.9	11811
CPT1C	1.05	0.182680281	-0.04	77	-1.11	9319	0.11	89	4.4	5663	-0.01	97	-0.34	8817
ZNF787	1.09	0.003199288	-0.02	104	-1.11	9320	-0.01	104	-0.68	8923	-0.01	100	-0.64	9005
ST8SIA4_t1	-1.02	0.060872065	-0.01	138	-1.12	9321	-0.04	130	-3.32	10688	-0.06	119	-3.99	10639
ADAM32	1.04	0.171727004	-0.03	91	-1.12	9322	0.08	92	3.37	6269	0	104	0.11	8550
SLC25A41	-1.01	0.70909241	-0.03	90	-1.13	9323	-0.03	109	-2	9826	-0.07	88	-2.65	10015
GLRB	1.06	0.315882946	-0.01	137	-1.13	9324	0.03	119	2.03	7125	0.02	144	1.94	7486
CEBPA_t1	-1.04	0.623589907	-0.01	135	-1.13	9325	NA	NA	NA	NA	0.03	125	2.54	7161
BTG4	1.02	0.025368151	-0.03	91	-1.13	9326	0	103	-0.02	8498	0.11	105	6.06	5424
ADORA3_t1	-1.21	7.37E-05	-0.01	137	-1.14	9327	0.07	120	5.07	5291	-0.19	121	-13.76	14042
CARS2_t1	-1.04	0.326161228	-0.02	98	-1.14	9328	NA	NA	NA	NA	-0.07	84	-2.46	9929
DMPK	1.05	0.051803653	-0.02	109	-1.14	9329	0.04	124	2.89	6559	-0.01	141	-0.63	8997
PRSS3	1.1	0.225777766	-0.08	65	-1.14	9330	0.04	80	1.23	7659	-0.19	89	-7.41	11997
SNX5	-1.11	0.016680658	-0.01	142	-1.14	9331	-0.02	120	-1.19	9278	-0.02	143	-2.27	9831

ARMC10	1.06	0.119886325	-0.01	136	-1.14	9332	0.06	144	5.58	5019	0.06	150	5.53	5673
CHI3L2	1.01	0.913989231	-0.02	100	-1.14	9333	0.09	96	4.16	5805	-0.16	124	-11.53	13410
KLHDC7B	1.1	0.009438587	-0.02	120	-1.15	9334	0.01	141	1.31	7596	-0.08	108	-4.54	10863
MAP3K12	-1.03	0.345594503	-0.02	121	-1.15	9335	0.09	147	8.87	3346	-0.15	160	-16.58	14712
IL22RA2	1	0.693843214	-0.02	124	-1.15	9336	-0.13	129	-10.22	13968	-0.01	127	-0.39	8844
GUCA1B	1.03	0.561431704	-0.02	115	-1.15	9337	-0.05	131	-4.06	11127	0.14	120	9.9	3916
TMEM31	-1.02	0.111052501	-0.04	81	-1.15	9338	-0.07	70	-1.34	9394	-0.05	87	-1.74	9582
PLD5	1.01	0.73347573	-0.02	120	-1.15	9339	-0.05	136	-4.56	11420	0.03	123	1.94	7487
ZNF296	1.03	0.046581425	-0.03	90	-1.16	9340	-0.06	107	-3.59	10867	-0.01	91	-0.43	8870
PRADC1	-1.08	0.128513407	-0.01	130	-1.16	9341	-0.14	142	-13.21	14802	0	112	0.2	8496
ITGB4	1.12	0.000510772	-0.02	104	-1.16	9342	0	119	0.21	8326	-0.03	128	-2.56	9983
PPP3CB	-1	0.908245552	-0.01	146	-1.16	9343	0.13	114	8.56	3467	0	120	0.33	8422
NTSR2	1.07	4.86E-05	-0.02	111	-1.16	9344	0.12	104	6.55	4476	0.11	106	6.21	5355
HUNK	1.01	0.234572477	-0.01	136	-1.16	9345	0.19	139	16.54	935	-0.08	129	-6.52	11658
METTL10	-1.01	0.732728465	-0.01	147	-1.17	9346	-0.07	113	-4.66	11472	0.07	117	4.96	5948
TRHR	1.02	0.159826553	-0.03	92	-1.17	9347	-0.08	114	-5.01	11660	-0.02	99	-0.85	9106
NPAS1	-1.06	0.044998227	-0.06	68	-1.17	9348	-0.08	94	-3.51	10800	-0.13	85	-4.52	10848
CHRNA6	1.06	0.000294266	-0.01	148	-1.17	9349	0.01	130	1.01	7803	-0.02	126	-1.72	9566
ACTR3B_t2	-1.11	0.04442812	-0.02	122	-1.17	9350	-0.12	112	-7.36	12821	-0.06	126	-4.52	10847
AURKC	-1.02	0.360775332	-0.03	95	-1.17	9351	-0.15	92	-6.12	12255	-0.07	91	-2.91	10139
IL7	-1.01	0.880037991	-0.01	133	-1.17	9352	0.08	118	5.17	5238	0.05	127	4.07	6385
CASKIN2	-1.05	0.121411791	-0.02	103	-1.17	9353	-0.03	115	-1.82	9702	-0.09	107	-5.28	11151
KLF13	1.21	0.000453328	-0.02	125	-1.17	9354	0.09	138	7.84	3817	0.01	146	0.95	8047
YIPF5_t1	-1.07	0.008764975	-0.01	134	-1.18	9355	-0.1	139	-8.56	13345	-0.09	147	-8.8	12524
ELANE	-1.06	0.013864636	-0.06	69	-1.18	9356	0	132	-0.26	8642	-0.17	93	-7.28	11953
RESP18	1.03	0.000967988	-0.02	104	-1.18	9357	0.04	106	2.05	7112	0.01	128	0.5	8319
RPE65	1.01	0.156885472	-0.02	120	-1.18	9358	0.03	129	2.41	6884	0.05	126	3.69	6571
DOK7	1.21	0.003806315	-0.02	108	-1.19	9359	-0.04	122	-2.7	10275	0.03	144	2.43	7216
BEND6	1.04	0.000435294	-0.02	129	-1.19	9360	0.06	132	5.07	5295	0	132	0.06	8577
FAIM2_t1	1.01	0.735545896	-0.01	154	-1.19	9361	NA	NA	NA	NA	0.04	144	3.5	6668
ATP5A1	-1.08	0.109136686	-0.03	96	-1.19	9362	-0.11	144	-10.53	14074	-0.08	120	-5.42	11212
WDR78_t1	1.06	0.030976744	-0.02	98	-1.19	9363	0.14	82	4.47	5624	-0.02	83	-0.58	8966
MRAP_t1	1.19	0.026159618	-0.02	115	-1.19	9364	0.03	109	1.91	7212	0	127	0.29	8446
CCDC12	-1.03	0.202445821	-0.02	100	-1.2	9365	0.1	108	5.76	4917	-0.09	114	-5.61	11276
MMP8	1.02	0.070957108	-0.01	139	-1.2	9366	-0.08	134	-6.46	12396	0.02	142	1.39	7811
DISC1_t2	-1.13	0.044251033	-0.01	139	-1.2	9367	-0.02	132	-1.24	9318	-0.04	129	-2.88	10127
ATP5C1	-1.11	0.018873421	-0.01	150	-1.21	9368	-0.12	80	-3.58	10863	-0.04	99	-2.19	9791
WNT7B_t1	1.02	0.191038685	-0.01	131	-1.21	9369	NA	NA	NA	NA	0.04	123	2.95	6964
SAMD9L	-1.31	0.000316052	-0.02	120	-1.21	9370	0.04	154	4.63	5530	-0.12	135	-10.51	13088
KIAA2026	-1	0.814753821	-0.02	123	-1.21	9371	0.01	130	0.53	8119	0.04	127	3.39	6722
CA7	1.05	1.58E-05	-0.02	108	-1.21	9372	0	118	-0.17	8599	0.01	100	0.38	8389
CYP4F11	-1.1	0.292409268	-0.01	135	-1.21	9373	-0.07	140	-6.46	12400	-0.02	135	-1.4	9392
LOXL3	-1.07	0.019737663	-0.03	97	-1.21	9374	0.12	131	9.49	3066	-0.15	123	-11.24	13317
GLG1_t1	-1.04	0.264267261	-0.01	135	-1.21	9375	NA	NA	NA	NA	-0.12	127	-9.52	12767
POM121L12	1.05	8.00E-07	-0.01	134	-1.22	9376	-0.07	114	-4.56	11417	0.04	118	2.47	7196
ASMTL	-1.19	0.000329862	-0.05	76	-1.22	9377	-0.18	110	-10.66	14117	-0.12	80	-3.63	10452
TBK1	-1.04	0.374071337	-0.01	132	-1.22	9378	0.03	123	2.15	7044	-0.01	123	-0.87	9117
APOC1	-1.11	0.32236974	-0.03	88	-1.23	9379	-0.17	117	-11.66	14416	0	88	-0.06	8654
RNF19A_t1	-1.2	0.004492599	-0.01	140	-1.23	9380	NA	NA	NA	NA	0.06	126	4.44	6221
GPR50	-1.01	0.377055378	-0.08	66	-1.23	9381	-0.08	92	-3.37	10719	-0.05	88	-1.92	9677
BSCL2	1.04	0.224562825	-0.02	113	-1.23	9382	-0.1	136	-9.02	13512	-0.02	100	-0.78	9072
ALS2CL	1.13	0.005600876	-0.02	116	-1.23	9383	-0.04	135	-3.05	10503	0.07	133	5.58	5633
PHACTR1	-1.07	0.001261807	-0.02	104	-1.23	9384	0.05	126	3.85	5979	-0.12	109	-7.01	11851
HLA-DMB	-1.67	7.68E-09	-0.02	109	-1.24	9385	0.01	120	0.89	7875	-0.22	116	-14.6	14268
WDR34	-1.1	0.048824488	-0.04	81	-1.24	9386	-0.15	73	-3.47	10780	-0.07	100	-3.51	10399
B3GNT4	1.05	4.58E-05	-0.02	104	-1.24	9387	0.04	110	2.51	6818	0.06	90	2.37	7248
NPPA	1.08	0.040279344	-0.02	101	-1.24	9388	-0.15	137	-13	14756	-0.05	104	-2.58	9989
MMP3	1.21	0.000368138	-0.02	120	-1.25	9389	-0.07	137	-5.95	12157	0.04	132	2.97	6948
SLC5A7	1	0.833049415	-0.01	137	-1.25	9390	0.08	140	7.01	4243	-0.01	143	-1.37	9375
CACFD1	1.04	0.231487894	-0.02	120	-1.25	9391	-0.05	123	-3.66	10914	-0.06	122	-4.08	10675
AMD1	-1.08	0.209587449	-0.02	130	-1.25	9392	0.13	125	9.54	3042	-0.05	126	-3.84	10554
POU4F2	1.04	0.00034257	-0.02	124	-1.25	9393	-0.04	126	-3.11	10551	0.02	124	1.63	7657
HDDC3	1.11	0.003046752	-0.02	129	-1.25	9394	-0.11	120	-7.38	12827	0.03	127	2.28	7306
NLRP9	1.02	0.060463564	-0.02	119	-1.26	9395	-0.12	120	-8.69	13393	0.02	130	1.4	7806
SPPL2C	1.01	0.100810374	-0.03	88	-1.26	9396	-0.07	122	-4.7	11486	0.12	124	8.68	4351
PHF13	1	0.954826119	-0.02	126	-1.26	9397	0.01	124	0.51	8133	-0.1	129	-7.69	12112
TADA1	1.11	0.033401218	-0.01	137	-1.26	9398	-0.01	119	-1	9149	0.06	144	5.81	5538
FYTTD1	-1.21	6.77E-05	-0.01	144	-1.26	9399	-0.09	128	-7.23	12748	-0.11	125	-8.26	12347
MAZ_t2	1.07	0.002579654	-0.01	142	-1.26	9400	-0.01	113	-0.62	8887	0.05	112	3.37	6729
DOK3	-1.04	0.061517074	-0.04	84	-1.26	9401	0.05	119	3.34	6293	-0.19	100	-9.67	12799
HTR1A	1.01	0.58593015	-0.03	94	-1.26	9402	-0.18	123	-13.2	14798	0.04	107	2.36	7257
WNK3	1.1	0.201334238	-0.01	159	-1.27	9403	-0.05	135	-4.28	11255	0.15	153	15.85	2115
GAD2	-1.01	0.201325785	-0.02	115	-1.27	9404	-0.04	129	-3.11	10557	-0.09	127	-6.57	11679
STOML2	1.09	0.020830175	-0.01	137	-1.27	9405	-0.09	93	-3.98	11097	0.07	124	4.87	5989
CXorf40B	1.14	7.08E-05	-0.02	114	-1.27	9406	0.06	98	2.85	6584	0.06	116	3.96	6431
IL2_t1	-1.02	0.000592282	-0.02	106	-1.28	9407	NA	NA	NA	NA	-0.15	120	-10.65	13133
SPTLC3	1.02	0.56571174	-0.02	132	-1.28	9408	-0.01	138	-0.93	9099	0	154	-0.36	8827
PARD6A	-1.03	0.326383202	-0.03	88	-1.28	9409	-0.18	125	-13.3	14823	-0.05	86	-1.88	9655
ATP6V1G1	-1.02	0.691103706	-0.01	147	-1.28	9410	0.12	130	9.69	2982	0	123	-0.08	8666
FUT10	-1.1	0.001227982	-0.02	133	-1.29	9411	-0.01	126	-1.05	9180	-0.08	130	-6.58	11684
KAT2A	1.04	0.408785602	-0.02	109	-1.29	9412	0.05	152	5.23	5202	0.07	104	3.95	6440
HMBS	1.14	0.000446293	-0.03	100	-1.29	9413	0.09	107	4.99	5343	0	91	0.16	8516
HTR5A_t1	1.01	0.189385592	-0.02	133	-1.29	9414	NA	NA	NA	NA	0.08	127	6.21	5354
IL32	-1.1	0.226053513	-0.02	112	-1.29	9415	-0.11	126	-8.59	13360	-0.06	112	-3.93	10600
TMEM158	-1.09	0.060650252	-0.02	131	-1.29	9416	0.07	104	3.73	6043	-0.14	133	-11.46	13390
LINGO4	1.04	0.006290201	-0.02	133	-1.3	9417	-0.1	111	-5.81	12095	-0.02	120	-1.67	9537
SVIL	1	0.983873491	-0.02	110	-1.3	9418	-0.01	108	-0.34	8703	0.06	126	4.74	6060
SEMA4C	-1.18	0.001282522	-0.02	130	-1.3	9419	0.05	127	3.58	6127	-0.19	129	-14.91	14360
EDEM1	-1.17	3.34E-05	-0.02	134	-1.3	9420	-0.13	143	-11.91	14481	-0.1	149	-10.25	13009
ADAM12_t1	1.01	0.249489962	-0.02	112	-1.3	9421	0.03	115	1.76	7310	0	119	0.02	8612
UBTD1	-1.02	0.338277005	-0.03	100	-1.31	9422	-0.07	120	-5.09	11718	0	104	-0.25	8768
IGFLR1	-1.25	0.000693419	-0.03	97	-1.31	9423	-0.04	83	-1.34	9389	-0.19	100	-9.43	12731
TSPAN15	1.04	0.411887914	-0.02	122	-1.31	9424	0.02	131	1.27	7629	-0.06	120	-4.01	10646
NECAB2	-1.08	0.235596551	-0.03	90	-1.31	9425	-0.17	118	-11.63	14405	-0.06	90	-2.52	9960
LUZP1	1.05	0.001034518	-0.02	134	-1.31	9426	0.1	137	8.74	3394	0.05	133	3.86	6492
TAF10	1.18	5.77E-07	-0.02	119	-1.32	9427	-0.01	96	-0.38	8735	0.02	104	1.02	8008
LRRC56	1.05	0.253635855	-0.02	115	-1.32	9428	0.13	120	9.41	3102	0.03	100	1.73	7613
EPB49	-1.04	0.006834721	-0.02	110	-1.32	9429	-0.1	120	-7.26	12766	-0.17	136	-14.63	14277
DCTN6_t1	-1.18	7.22E-05	-0.02	113	-1.32	9430	NA	NA	NA	NA	-0.05	114	-3.46	10377
CEACAM21	-1.06	0.000116523	-0.03	99	-1.32	9431	-0.13	142	-11.56	14390	-0.06	100	-2.89	10128
HOXA7	1.19	0.000823267	-0.01	139	-1.32	9432	-0.02	131	-1.75	9655	0.05	123	3.87	6488
COL6A5	1.06	0.000879466	-0.02	104	-1.33	9433	-0.01	117	-0.57	8861	-0.04	119	-2.85	10111
MT4	-1.02	0.037048027	-0.13	60	-1.33	9434	-0.21	100	-10.6	14106	-0.01	68	-0.22	8749
SIRT6	1.07	0.000293733	-0.02	134	-1.33	9435	0.02	125	1.13	7725	-0.04	140	-3.38	10348
ITGB1_t2	1.02	0.681524631	-0.02	125	-1.33	9436	NA	NA	NA	NA	0.07	125	5.09	5885
SLC11A2_t1	1.11	4.16E-05	-0.02	132	-1.33	9437	0.11	122	8.06	3709	NA	NA	NA	NA

CSTF3_t1	-1.09	0.069036525	-0.02	123	-1.33	9438	-0.07	119	-4.5	11389	-0.04	118	-2.91	10137
LPAR4	1.01	0.32273686	-0.02	121	-1.33	9439	0.07	116	4.91	5388	-0.03	115	-1.74	9576
TRABD	1	0.96162815	-0.02	113	-1.34	9440	0.12	132	9.46	3085	-0.1	110	-6.22	11536
CSNK1G2	1.06	0.07895693	-0.03	100	-1.34	9441	0.07	120	5.12	5270	-0.09	115	-5.83	11380
FBLN1_t2	-1.02	0.750519002	-0.03	101	-1.34	9442	0.05	104	2.66	6704	-0.17	104	-9.27	12672
VKORC1L1	1.04	0.159206857	-0.02	114	-1.34	9443	0.11	115	7.07	4212	0.06	111	3.36	6735
ABP1	1.01	0.502357446	-0.06	72	-1.34	9444	-0.01	88	-0.54	8843	-0.03	80	-0.79	9078
HADHA	-1.14	1.05E-06	-0.03	102	-1.34	9445	-0.09	119	-5.95	12156	-0.05	148	-4.6	10895
TMPRSS11F	-1	0.990182402	-0.02	120	-1.35	9446	0.08	135	6.52	4499	-0.06	124	-4.39	10793
NFATC2	1.01	0.55510297	-0.02	130	-1.35	9447	0.01	134	1.02	7801	-0.1	141	-9.53	12768
CAPRIN2_t1	1.05	0.092069824	-0.02	105	-1.35	9448	NA	NA	NA	NA	NA	NA	NA	NA
MYO3B_t1	-1.01	0.225294815	-0.02	136	-1.35	9449	-0.1	135	-8.14	13172	NA	NA	NA	NA
TNNT3	1.01	0.080108506	-0.02	104	-1.35	9450	-0.03	113	-2.02	9836	0.09	120	6.57	5199
CHRNB4	1	0.862634075	-0.02	124	-1.35	9451	-0.07	129	-5.68	12017	-0.08	123	-5.58	11267
AHSP	-1	0.917568203	-0.03	99	-1.35	9452	-0.05	104	-2.52	10166	-0.04	88	-1.66	9532
TRPC4	1.05	0.097397927	-0.02	140	-1.35	9453	0.11	122	7.95	3770	-0.06	147	-5.76	11342
DHRS2_t1	1.2	0.385182026	-0.02	107	-1.35	9454	NA	NA	NA	NA	0.04	124	2.95	6965
GATAD2B	1.04	0.020095769	-0.02	128	-1.36	9455	-0.07	142	-6.08	12233	0.02	142	2.13	7382
ELAVL3_t2	1.02	0.051558229	-0.02	124	-1.36	9456	-0.17	148	-16.48	15504	0	137	0.12	8542
PVRL2_t1	1.02	0.666560481	-0.02	112	-1.36	9457	-0.1	127	-7.7	12987	-0.12	109	-7.21	11924
CLDN17	-1.01	0.227759382	-0.08	68	-1.36	9458	-0.07	84	-2.46	10128	-0.09	62	-1.1	9247
SEMG2	1	0.744150057	-0.02	120	-1.36	9459	-0.01	119	-0.62	8890	0	131	-0.06	8659
DNAJC15	-1.42	1.64E-09	-0.02	128	-1.36	9460	-0.03	121	-1.96	9786	-0.2	131	-16.48	14679
CCL7	1	0.931230915	-0.02	120	-1.36	9461	0.06	122	4.25	5751	-0.07	112	-4.28	10747
XYLB	1.03	0.38835968	-0.02	132	-1.36	9462	-0.08	125	-6.14	12261	0.06	121	3.95	6436
MXD1_t1	-1.13	0.00017125	-0.01	141	-1.36	9463	-0.02	132	-2.04	9853	NA	NA	NA	NA
FUS	-1.05	0.157350818	-0.02	141	-1.37	9464	0.04	118	2.87	6563	-0.13	136	-11.28	13343
CAMK1D_t1	-1.18	0.007360182	-0.02	131	-1.37	9465	-0.01	139	-0.52	8827	-0.12	120	-8.23	12323
CDK11B_t2	-1.1	0.0044088	-0.23	56	-1.37	9466	-0.04	69	-0.81	9025	-0.16	68	-2.8	10088
OPN4	1.03	0.029596471	-0.03	104	-1.37	9467	-0.09	140	-8.04	13127	-0.01	127	-0.55	8954
BPIFB2	1.08	0.007364315	-0.02	106	-1.38	9468	-0.02	104	-1.27	9342	-0.08	124	-5.76	11337
CHI3L1	-1.05	0.695753408	-0.02	111	-1.38	9469	0.03	116	1.84	7248	-0.11	105	-5.78	11353
ZNF358	-1.08	0.005442223	-0.05	79	-1.38	9470	-0.16	98	-7.61	12934	-0.11	95	-4.96	11036
PTGER1	1.07	0.003505306	-0.08	68	-1.38	9471	0.01	88	0.21	8325	-0.04	84	-1.32	9352
BARX2	-1	0.721441197	-0.02	120	-1.38	9472	-0.06	100	-2.94	10438	0	118	-0.17	8713
CCDC19	1.07	0.066597464	-0.02	141	-1.38	9473	0.13	108	7.34	4072	0.05	93	2	7448
PPP1R13B	-1.05	0.168114024	-0.02	106	-1.38	9474	-0.11	149	-10.99	14235	-0.17	118	-11.46	13391
RGS10	-1.3	0.005752483	-0.02	116	-1.39	9475	0.14	105	7.74	3861	-0.18	119	-12.18	13605
CLK2	1.16	3.43E-07	-0.02	116	-1.39	9476	0	121	0.29	8270	0.01	112	0.53	8300
SESN3	1.07	0.041634286	-0.02	140	-1.39	9477	-0.08	132	-6.55	12437	0.01	143	0.49	8325
MSRA	-1.14	0.000573681	-0.02	113	-1.39	9478	-0.11	116	-7.43	12842	-0.08	116	-5.25	11136
CLEC5A_t1	1.01	0.589143954	-0.02	131	-1.39	9479	NA	NA	NA	NA	0.05	128	3.52	6662
ZNF528	1.02	0.347173	-0.02	131	-1.39	9480	0.07	139	6.34	4595	0.03	124	1.96	7473
TEKT4	1.11	0.007645371	-0.04	84	-1.4	9481	-0.05	88	-2.01	9829	0.14	80	4.34	6271
PSMC3	-1.05	0.211991035	-0.09	66	-1.4	9482	-0.19	87	-6.93	12621	-0.08	84	-2.56	9985
OBFC1	-1.04	0.131348304	-0.01	155	-1.4	9483	-0.17	151	-16.82	15568	0.03	159	2.84	7017
ATAD3B	-1.02	0.327844702	-0.03	90	-1.4	9484	0	132	-0.06	8526	-0.15	108	-8.59	12454
PPP1R12C	-1.02	0.600317397	-0.03	93	-1.4	9485	-0.01	103	-0.77	8991	-0.02	113	-1.27	9325
SNCB	-1.04	0.335874888	-0.03	94	-1.4	9486	-0.02	124	-1.3	9361	-0.02	104	-1.09	9240
FKBP4_t1	1.04	0.157297806	-0.02	125	-1.4	9487	NA	NA	NA	NA	NA	NA	NA	NA
PVR	-1.04	0.518391202	-0.02	133	-1.4	9488	-0.12	158	-13	14754	-0.06	138	-5.45	11221
BDKRB2	-1.02	0.882107975	-0.02	120	-1.4	9489	0.04	140	3.91	5943	-0.07	136	-5.72	11326
MREG	-1.01	0.693208727	-0.02	132	-1.4	9490	-0.02	143	-1.95	9783	-0.05	156	-5.42	11208
ZPBP2	-1	0.828452239	-0.02	129	-1.4	9491	0.02	126	1.75	7318	0.02	124	1.35	7832
TRIB3	1.22	0.052811152	-0.02	122	-1.4	9492	0	125	0	8478	0.01	130	1.11	7961
HNF4A_t2	-1	0.900023745	-0.01	156	-1.41	9493	-0.09	128	-7.1	12693	0.01	134	0.56	8284
TOP3B	1.05	0.006228343	-0.04	88	-1.41	9494	-0.03	126	-1.98	9804	-0.07	118	-5.01	11051
ENPP5	-1.01	0.889133528	-0.02	137	-1.41	9495	0.15	120	10.24	2780	-0.15	160	-16.67	14731
GRIN1	-1.02	0.022841026	-0.03	104	-1.41	9496	-0.08	124	-6.06	12217	-0.09	112	-5.81	11367
RENBP	-1.09	0.000200838	-0.14	60	-1.41	9497	-0.02	104	-0.91	9088	-0.2	72	-4.41	10807
ALG5	-1.07	0.229128642	-0.03	104	-1.41	9498	0	139	0.37	8227	-0.06	111	-3.38	10345
SLC8A3	1.04	0.00439694	-0.02	122	-1.41	9499	-0.04	123	-2.82	10350	-0.01	128	-1	9203
KLRC2	-1.15	0.001979182	-0.02	116	-1.41	9500	0.01	127	1.15	7709	-0.16	135	-13.93	14089
NOC2L	-1.03	0.32479632	-0.03	98	-1.41	9501	-0.08	120	-5.75	12057	-0.06	121	-4.16	10700
ZNF239	1.25	0.018910214	-0.02	118	-1.41	9502	0.2	131	16.58	926	0.07	104	3.85	6500
FGFR1OP	-1.08	0.13639259	-0.02	124	-1.42	9503	-0.05	135	-3.95	11075	0.04	138	3.67	6578
AFP	1.46	0.009968061	-0.02	118	-1.42	9504	0.09	121	6.68	4408	0.09	97	4.22	6332
RIMKLB	1.01	0.600042486	-0.02	136	-1.42	9505	0.05	127	4.17	5797	0.02	136	1.62	7667
MRPL42P5	1.08	0.000112283	-0.02	116	-1.42	9506	0.17	131	13.95	1546	0.09	120	6.46	5258
LYAR	-1.01	0.910625555	-0.03	98	-1.43	9507	0.06	110	3.35	6282	0.06	117	4.33	6273
CAMKK1_t2	1	0.800086028	-0.02	127	-1.43	9508	NA	NA	NA	NA	0	124	0.33	8424
INHBB	-1.19	0.086384043	-0.01	152	-1.43	9509	-0.11	137	-9.95	13864	0.02	168	2.01	7447
PRSS53	-1.05	0.002790503	-0.03	96	-1.43	9510	-0.15	143	-13.62	14902	-0.06	99	-3.04	10200
SPRN	1.03	0.036488912	-0.02	120	-1.43	9511	-0.03	136	-2.59	10209	0.02	143	1.57	7708
ANKRD22	-1.11	0.109031539	-0.02	135	-1.43	9512	0.01	126	0.45	8170	-0.2	125	-14.86	14338
PKD2	1.05	0.096846054	-0.02	120	-1.43	9513	0.12	126	9.34	3125	0.03	142	3.04	6908
ASZ1	1.02	0.425131481	-0.02	111	-1.44	9514	0	126	0.15	8365	0.06	124	4.11	6366
SPAM1	1.02	0.049087516	-0.03	94	-1.44	9515	-0.05	72	-1.12	9234	0.07	104	3.88	6482
TAS2R9	1.01	0.332975483	-0.04	88	-1.44	9516	-0.12	76	-3.03	10491	-0.06	60	-0.56	8959
PAX5	1.01	0.267177586	-0.02	126	-1.44	9517	-0.12	135	-10.03	13890	0.05	126	3.76	6540
MRGPRE	-1.03	0.088524206	-0.08	68	-1.44	9518	-0.15	73	-3.41	10737	-0.13	80	-4.03	10650
MYOZ1	-1.02	0.183039143	-0.02	138	-1.44	9519	0.04	124	3.22	6365	-0.06	123	-4.61	10902
CNTN2_t1	1	0.82393441	-0.02	127	-1.44	9520	NA	NA	NA	NA	-0.05	135	-4.57	10873
TMEM190	-1.01	0.4606564	-0.06	76	-1.44	9521	-0.13	72	-2.84	10371	-0.06	68	-1.09	9238
ZNF276_t1	-1.05	0.074043837	-0.02	139	-1.44	9522	NA	NA	NA	NA	-0.1	128	-7.92	12204
CYP4B1	1.04	0.089663389	-0.03	102	-1.44	9523	0.11	130	9.16	3204	0.01	116	0.94	8052
SLC5A4	1.01	0.306354699	-0.03	92	-1.44	9524	-0.06	84	-2.14	9927	-0.04	72	-0.79	9084
MARCKSL1	-1.14	0.000822957	-0.03	106	-1.45	9525	-0.05	132	-4.5	11388	-0.15	124	-11	13258
TDP2	1.11	0.120118642	-0.02	130	-1.45	9526	0.02	129	1.93	7186	0.17	114	11.09	3478
SYNE2_t1	-1.05	0.442105142	-0.02	121	-1.45	9527	0.01	124	0.96	7827	-0.12	130	-9.85	12873
TIMM13	1.18	0.000283455	-0.02	116	-1.45	9528	0.12	120	8.13	3680	0.09	110	5.17	5845
USP18	-1.2	0.001161132	-0.03	102	-1.46	9529	-0.12	106	-6.91	12605	-0.13	135	-10.78	13173
BRS3	1.06	9.32E-06	-0.04	84	-1.46	9530	0.01	84	0.26	8288	0.12	80	3.55	6638
ZNF266	1.01	0.777076958	-0.02	114	-1.46	9531	-0.02	125	-1.71	9624	0	163	-0.51	8926
SRRM4	1.02	0.10974565	-0.02	138	-1.46	9532	-0.08	141	-7.48	12864	0.05	149	4.99	5927
POLR3B	-1.05	0.217770261	-0.02	128	-1.46	9533	-0.05	120	-3.53	10823	0.04	109	2.14	7376
CLDN19	1.11	0.000234569	-0.02	112	-1.46	9534	0.05	130	3.73	6040	0.08	120	5.56	5649
DHRS11	1.04	0.231477401	-0.02	138	-1.46	9535	-0.14	125	-10.74	14151	0.06	124	4.66	6092
CIB3	-1.1	0.000927724	-0.07	70	-1.46	9536	-0.2	91	-8	13115	-0.12	88	-4.73	10956
AMY1C	-1.42	5.38E-05	-0.07	72	-1.46	9537	-0.24	68	-4.24	11239	-0.04	80	-1.13	9263
SEMA4G	1.04	0.452138726	-0.03	107	-1.47	9538	-0.08	134	-6.67	12502	0.01	128	0.56	8287
POFUT1_t2	1.05	0.003309585	-0.02	133	-1.47	9539	0.05	125	3.45	6218	0.03	134	2.35	7262
IPO5_t1	-1.14	0.013430831	-0.02	133	-1.47	9540	0.05	137	4.26	5746	NA	NA	NA	NA
ERICH1	-1.2	0.000136224	-0.02	121	-1.47	9541	-0.02	108	-1.22	9311	-0.14	119	-9.65	12791
NEUROG1	-1.23	5.67E-05	-0.02	121	-1.47	9542	-0.02	116	-1.41	9435	-0.1	150	-9.64	12788
SDR9C7	1.01	0.504499043	-0.03	107	-1.47	9543	-0.06	111	-3.92	11052	-0.01	104	-0.58	8973

MRAP_t2	1.11	0.001645324	-0.03	104	-1.48	9544	0.08	129	6.5	4509	0.03	106	1.59	7691
DND1	1.03	0.339278516	-0.02	119	-1.48	9545	-0.02	104	-1.17	9268	-0.09	122	-6.45	11624
LCE4A	1.03	0.028142781	-0.07	72	-1.48	9546	-0.06	72	-1.21	9299	-0.01	110	-0.69	9029
TRPT1	-1.01	0.69277912	-0.04	88	-1.48	9547	-0.1	91	-4.15	11182	0.01	81	0.17	8508
TRIM51	-1	0.899562247	-0.03	93	-1.49	9548	0.12	119	7.96	3765	-0.08	132	-6.46	11625
FAM118A	-1.07	0.031294423	-0.02	120	-1.49	9549	0.01	153	0.89	7876	-0.11	121	-8.07	12259
CNOT7_t2	-1.03	0.398260245	-0.02	114	-1.49	9550	-0.01	116	-0.76	8985	-0.07	116	-4.53	10855
PTPLAD2_t1	-1.24	5.37E-09	-0.02	130	-1.49	9551	0.04	117	2.42	6881	NA	NA	NA	NA
TMEM242_t1	-1.05	0.003472484	-0.02	129	-1.49	9552	NA	NA	NA	NA	NA	NA	NA	NA
HSPA2	-1.09	0.157055897	-0.02	123	-1.5	9553	0.05	110	3.03	6477	-0.1	140	-9.43	12735
VPS51	-1.03	0.442525473	-0.03	100	-1.5	9554	-0.09	104	-4.89	11589	-0.04	109	-2.07	9744
GPA33_t1	-1.01	0.355870498	-0.02	132	-1.5	9555	NA	NA	NA	NA	-0.06	140	-5.05	11065
YIF1A	-1.03	0.444194855	-0.02	119	-1.5	9556	-0.05	91	-2	9825	0.03	115	2.06	7422
ECE2_t2	1.02	0.137165549	-0.02	111	-1.5	9557	-0.07	113	-4.54	11404	0.01	111	0.89	8084
FRZB_t1	-1.1	0.270842601	-0.01	158	-1.5	9558	NA	NA	NA	NA	-0.12	152	-12.24	13622
GABRQ	1.06	1.24E-06	-0.04	91	-1.51	9559	-0.03	114	-1.99	9815	0.05	100	2.66	7102
ALOX12B	1.01	0.510959879	-0.03	96	-1.51	9560	-0.12	97	-5.58	11958	-0.05	88	-1.77	9600
ESRRB	1.01	0.752114506	-0.02	122	-1.51	9561	-0.05	138	-4.15	11177	-0.06	113	-4.06	10665
F10	-1.27	0.001784556	-0.15	60	-1.51	9562	-0.19	100	-9.26	13607	-0.15	81	-4.53	10853
FUT4_t1	-1.01	0.737881286	-0.01	152	-1.51	9563	NA	NA	NA	NA	-0.16	148	-15.83	14542
RRAGA	1.13	0.000562084	-0.02	115	-1.51	9564	-0.03	112	-2.12	9912	0.09	130	7.18	4958
MMACHC	-1.15	0.002071142	-0.02	133	-1.51	9565	-0.2	124	-14.91	15187	-0.05	120	-3.66	10459
TAAR5	-1	0.895976514	-0.04	84	-1.52	9566	-0.16	93	-6.87	12593	-0.03	115	-1.86	9646
UQCRC1	-1.01	0.888924559	-0.05	80	-1.52	9567	-0.16	112	-9.69	13768	-0.01	100	-0.58	8969
CNTROB_t2	-1.02	0.172867215	-0.02	115	-1.52	9568	-0.12	104	-6.65	12490	-0.1	103	-5.46	11225
SH3BP1_t1	-1.06	6.27E-05	-0.03	107	-1.52	9569	-0.18	120	-12.41	14617	NA	NA	NA	NA
MEIS2_t1	1.02	0.75803746	-0.02	128	-1.52	9570	0.03	119	2.11	7076	-0.01	118	-0.34	8816
ROR1	1.14	0.096947067	-0.02	138	-1.53	9571	0.16	138	13.77	1607	0.01	139	0.53	8298
SLC15A4_t1	-1.17	0.000785579	-0.02	121	-1.53	9572	NA	NA	NA	NA	-0.05	120	-3.71	10491
H3F3C	1.04	0.467836572	-0.02	125	-1.53	9573	0.08	115	5.52	5055	-0.04	142	-3.22	10280
ACSBG1	1.03	0.032176281	-0.02	120	-1.53	9574	0.05	138	4.61	5540	0.05	116	3.55	6640
RUNX1	-1.04	0.425486438	-0.02	139	-1.53	9575	0.04	136	3.87	5964	-0.09	138	-8.15	12291
CUL1	-1.04	0.419757313	-0.03	108	-1.53	9576	-0.03	130	-2.53	10170	0.06	114	3.75	6545
ADRB1	-1.02	0.04110906	-0.02	126	-1.53	9577	-0.17	154	-17.37	15655	-0.01	128	-1.01	9209
ANKMY1_t2	1.01	0.441800485	-0.03	96	-1.53	9578	-0.06	102	-3.31	10684	-0.07	103	-3.51	10398
STAT3	-1.31	5.54E-10	-0.02	128	-1.54	9579	-0.18	139	-15.9	15395	-0.18	161	-20.37	15394
PEBP4	-1.01	0.739076047	-0.05	78	-1.54	9580	-0.07	122	-5.13	11735	-0.11	84	-3.7	10481
TAB1	1.04	0.157795604	-0.02	127	-1.54	9581	0	121	0.1	8399	-0.04	127	-2.7	10037
KIR2DS1	-1.04	0.001780512	-0.03	100	-1.54	9582	-0.18	108	-10.19	13950	-0.11	91	-4.69	10938
CRYGA	1.05	0.000213413	-0.03	98	-1.54	9583	-0.08	93	-3.25	10637	-0.02	98	-0.98	9191
NOTCH3	1.01	0.240378532	-0.02	112	-1.55	9584	-0.07	135	-5.59	11964	0.03	120	2.08	7406
NLGN3	-1	0.955538804	-0.03	104	-1.55	9585	-0.04	146	-3.6	10873	-0.08	115	-4.96	11035
PCBP3	-1.01	0.559890303	-0.03	104	-1.56	9586	-0.09	130	-6.92	12612	0.05	108	2.75	7056
ABCF3	-1.03	0.086696884	-0.03	104	-1.56	9587	-0.11	124	-8.26	13221	-0.13	100	-6.73	11747
GPATCH3	1.11	4.13E-05	-0.02	122	-1.56	9588	0	103	-0.23	8622	0.04	116	2.7	7090
TRIB2	1.09	0.238670027	-0.02	147	-1.56	9589	0.07	140	6.17	4681	-0.02	126	-1.21	9300
TUBGCP5	1.06	0.025239379	-0.01	160	-1.56	9590	-0.02	130	-1.56	9524	0.12	145	11.21	3442
RPL36AL	1.04	0.328889866	-0.03	101	-1.56	9591	0.02	103	1.1	7743	0.09	83	2.97	6947
JUP	-1.1	0.003553038	-0.03	111	-1.56	9592	-0.11	132	-8.99	13500	-0.06	108	-3.33	10322
SEL1L	-1.03	0.379209502	-0.02	131	-1.57	9593	-0.09	148	-9.14	13572	0.03	145	3.31	6766
PRSS57	1.01	0.506056195	-0.05	83	-1.57	9594	-0.05	108	-2.9	10414	-0.08	98	-3.88	10581
CSF2RA_t1	-1.18	9.25E-05	-0.02	136	-1.57	9595	0.03	117	2.11	7074	-0.19	132	-15.51	14484
KDM2B	-1.06	0.023117462	-0.02	120	-1.57	9596	0.02	131	1.66	7380	-0.13	149	-12.92	13824
TLR9	1	0.875497856	-0.04	88	-1.57	9597	0.03	132	2.51	6817	-0.09	100	-4.34	10773
CAMK2N1	1.01	0.819714188	-0.02	131	-1.57	9598	0.08	111	5.02	5323	0.07	131	5.27	5792
PRSS58	1	0.755162959	-0.05	84	-1.58	9599	0.01	100	0.55	8103	-0.01	83	-0.3	8794
BAP1	1.01	0.612494686	-0.02	118	-1.58	9600	-0.07	139	-6.49	12409	0.05	103	2.52	7171
TNIP3	-1.05	0.002420235	-0.02	135	-1.58	9601	-0.08	132	-6.88	12596	-0.11	125	-8.61	12459
EFCAB1_t1	-1	0.975450603	-0.02	118	-1.58	9602	-0.04	110	-2.25	9994	NA	NA	NA	NA
CCNY_t1	-1	0.987518039	-0.02	143	-1.58	9603	-0.07	142	-6.33	12339	0.04	141	3.41	6714
TAF1C_t1	-1.05	0.22127744	-0.02	120	-1.58	9604	0	114	0.29	8273	NA	NA	NA	NA
IL17RB	1.02	0.744669955	-0.02	119	-1.58	9605	0.09	107	5.39	5125	0.05	119	3.76	6541
MLN_t1	1.01	0.653190594	-0.04	88	-1.58	9606	-0.14	103	-7.47	12862	-0.02	89	-0.96	9180
CPA5	1.02	0.442976777	-0.04	88	-1.58	9607	-0.08	92	-3.44	10762	-0.06	92	-2.6	9996
TBKBP1	-1.05	0.048231898	-0.02	114	-1.58	9608	-0.06	162	-6.26	12312	-0.03	120	-2.24	9820
NXPH3	-1.01	0.655115214	-0.02	129	-1.58	9609	-0.11	155	-11.65	14411	-0.08	141	-7.34	11973
BOLL	1.05	0.000497335	-0.02	138	-1.59	9610	0.02	125	1.52	7483	0.07	132	5.77	5547
ANKRD44	-1.04	0.296981092	-0.02	120	-1.59	9611	0.06	104	3.01	6486	-0.17	114	-11.1	13283
PEBP1	-1.05	0.520708749	-0.02	123	-1.59	9612	-0.16	116	-10.75	14162	0.02	138	1.72	7622
SYNC	-1.04	0.258664548	-0.02	139	-1.59	9613	0.07	138	6.18	4680	-0.04	123	-2.72	10048
PDP1	-1.01	0.886831248	-0.02	147	-1.59	9614	0.07	130	5.77	4912	-0.07	126	-5.28	11147
CHODL	1.04	0.427011054	-0.02	137	-1.59	9615	-0.1	112	-6.17	12272	-0.06	128	-4.55	10866
PPP1R9A_t1	1.11	0.409189329	-0.02	148	-1.6	9616	0	165	0.28	8275	NA	NA	NA	NA
HOXB3_t1	-1.01	0.47781458	-0.02	133	-1.6	9617	NA	NA	NA	NA	0.03	151	2.86	7010
KCNK10	1	0.930293947	-0.02	154	-1.6	9618	-0.03	154	-3.46	10775	-0.07	154	-6.8	11770
UTF1	-1.09	0.013027805	-0.03	97	-1.6	9619	-0.14	92	-5.9	12132	-0.1	84	-3.5	10395
ZPLD1	1.01	0.647804368	-0.02	136	-1.6	9620	0.01	134	1.05	7773	-0.08	142	-7.16	11900
ADCY8	1.05	0.229476816	-0.03	96	-1.6	9621	-0.03	91	-1.21	9296	-0.12	122	-8.65	12473
WWC2	-1.12	0.001102874	-0.02	141	-1.6	9622	-0.09	130	-7.59	12927	-0.03	151	-3.21	10273
KANK2	1.09	0.014195175	-0.02	135	-1.61	9623	0.07	158	7.44	4016	0	152	0.2	8494
CACNG4	1.07	0.000522861	-0.02	126	-1.61	9624	-0.08	126	-5.93	12147	0.01	144	0.5	8315
AKIRIN2_t1	-1.03	0.373295468	-0.02	126	-1.61	9625	NA	NA	NA	NA	-0.09	136	-7.83	12174
CUL4A_t1	1.01	0.908039187	-0.02	119	-1.61	9626	NA	NA	NA	NA	0.04	137	3.4	6720
RGS1	-2.39	1.00E-07	-0.02	118	-1.62	9627	0.06	102	3.3	6320	-0.18	150	-17.54	14893
IMPDH1_t1	1	0.891814562	-0.02	128	-1.62	9628	NA	NA	NA	NA	-0.15	147	-14.39	14214
ANKRD34B	1.03	0.017148167	-0.02	145	-1.62	9629	-0.09	142	-8.11	13158	0.05	129	4.09	6377
BCAN_t2	1.02	0.072395216	-0.03	111	-1.63	9630	-0.09	120	-6.63	12480	-0.03	104	-1.71	9557
NUDT9	-1.16	0.005551694	-0.02	130	-1.63	9631	-0.09	120	-6.35	12348	0.03	135	2.25	7320
TTTY13	1.06	0.007035708	-0.02	121	-1.63	9632	0.09	149	8.68	3421	0	106	-0.21	8735
FAM70B	-1.12	2.49E-07	-0.13	63	-1.63	9633	NA	NA	NA	NA	-0.23	56	-1.37	9374
ABO	1.02	0.025007979	-0.03	98	-1.63	9634	-0.05	122	-3.24	10629	-0.03	100	-1.69	9547
RWDD1	-1	0.978802583	-0.03	104	-1.63	9635	-0.02	107	-1.35	9402	0.01	137	1.17	7932
DLG3	1.03	0.227111522	-0.02	132	-1.63	9636	0	136	0.2	8337	-0.04	136	-3.49	10393
TRAK2_t1	-1.05	0.279654252	-0.02	150	-1.63	9637	NA	NA	NA	NA	NA	NA	NA	NA
TMEM102_t1	-1.05	0.081368731	-0.06	76	-1.64	9638	NA	NA	NA	NA	-0.16	88	-5.98	11434
CRB2	1.01	0.36994947	-0.02	120	-1.64	9639	-0.05	123	-3.9	11042	-0.02	115	-1.3	9347
MMP16	1.03	0.013272317	-0.02	153	-1.64	9640	0.05	131	4.14	5817	0.04	126	2.74	7060
DCUN1D4	-1.06	0.303793279	-0.02	142	-1.64	9641	-0.02	131	-1.86	9732	0.12	142	10.94	3532
ZNF75D_t1	1.01	0.710555908	-0.02	131	-1.65	9642	NA	NA	NA	NA	0.06	141	5.13	5863
GPR62	1.03	0.157626432	-0.03	112	-1.65	9643	-0.09	132	-7.64	12951	-0.11	119	-7.84	12179
PPT2	1	0.969634829	-0.03	113	-1.65	9644	0.03	106	1.44	7533	0.02	105	0.86	8107
ITGA4	-1.08	0.004395504	-0.02	139	-1.65	9645	0.04	120	3.09	6440	-0.14	153	-14.47	14229
TM4SF1	-1.25	0.024055154	-0.02	120	-1.65	9646	0.12	142	10.87	2525	-0.13	130	-10.7	13150
IL17A	1.04	0.032084794	-0.02	144	-1.65	9647	-0.13	145	-12.56	14660	0.05	128	3.66	6586
DEFB104B	-1.02	0.143256703	-0.06	78	-1.66	9648	-0.15	72	-3.34	10702	-0.04	78	-1.03	9219
CRISP1	-1.01	0.160674393	-0.02	126	-1.66	9649	-0.07	114	-4.5	11384	-0.11	131	-9.27	12676

FAM115C_t1	-1.04	0.333815152	-0.04	96	-1.66	9650	NA	NA	NA	NA	-0.02	134	-1.63	9513
SNAP91	1.05	8.80E-06	-0.02	136	-1.66	9651	0.06	122	4.6	5557	0.02	122	1.49	7758
BEND5	-1.07	0.213058905	-0.06	80	-1.67	9652	-0.05	68	-0.81	9022	-0.12	99	-6.02	11442
LTA4H	1.03	0.430015439	-0.03	106	-1.67	9653	0.09	107	5.41	5115	0.07	111	4.56	6148
MAPK8IP2	1.08	2.03E-07	-0.03	113	-1.67	9654	-0.02	124	-1.51	9501	0.02	106	1.23	7894
CTBP1	1.06	0.088039479	-0.03	110	-1.67	9655	0.06	116	4.06	5871	-0.02	110	-1.42	9403
EDC4	-1.03	0.168594984	-0.02	123	-1.67	9656	-0.12	107	-6.72	12522	-0.16	117	-10.79	13179
PANX1_t1	1.08	0.111375146	-0.02	120	-1.67	9657	NA	NA	NA	NA	0.03	148	2.46	7201
ZC3H18	1.02	0.521117499	-0.02	118	-1.67	9658	-0.08	108	-4.42	11337	-0.01	135	-0.48	8896
IDO1	-1.03	0.771943669	-0.03	110	-1.67	9659	-0.03	124	-2.03	9841	-0.06	110	-3.37	10341
CCDC101	1.06	0.048075067	-0.06	79	-1.68	9660	-0.06	129	-4.41	11332	0	71	-0.05	8651
MAGEA11	1.1	0.000116173	-0.03	114	-1.68	9661	0.13	124	9.31	3139	0.1	113	6.19	5366
USMG5	1.05	0.200705466	-0.04	96	-1.68	9662	0	111	-0.08	8539	0.06	113	3.89	6475
GYG2	-1.11	0.039550649	-0.02	124	-1.68	9663	-0.11	132	-9.21	13593	0.01	117	0.61	8254
PRKACA	-1.02	0.191748943	-0.02	131	-1.68	9664	-0.06	122	-4.25	11241	-0.09	122	-6.8	11771
MON1A	1.09	0.007952351	-0.04	89	-1.68	9665	0.06	114	4.12	5834	0.09	104	4.71	6067
USP20	-1	0.979220196	-0.03	104	-1.68	9666	0	116	0.28	8274	-0.13	120	-9.1	12625
UPK1B	1.03	0.099589858	-0.02	139	-1.68	9667	0.02	136	1.33	7585	0.02	148	1.61	7678
BIN1_t1	1.1	0.040440456	-0.02	118	-1.68	9668	0.12	104	6.45	4542	NA	NA	NA	NA
CD300E	1.05	0.000121114	-0.02	126	-1.68	9669	0.01	140	1.06	7760	0.05	114	3.36	6738
CHGA	-1.01	0.621586159	-0.03	110	-1.69	9670	-0.17	114	-11.03	14251	-0.06	109	-3.73	10499
CARD10	1.08	0.1416718	-0.02	120	-1.69	9671	0.07	133	5.44	5096	0.06	125	4.63	6116
SECTM1_t1	-1.01	0.734844856	-0.02	122	-1.69	9672	-0.01	153	-0.83	9038	NA	NA	NA	NA
HSPB3	1.09	1.81E-05	-0.03	104	-1.69	9673	0.22	108	12.83	1886	0.06	111	3.96	6434
CSTF3_t2	1.27	6.76E-06	-0.03	99	-1.69	9674	0	105	-0.15	8580	0.06	132	4.56	6145
BLOC1S6	-1.01	0.588823553	-0.02	136	-1.7	9675	0.04	129	3.34	6292	0.04	133	3.31	6770
WDR38	1.02	0.114978163	-0.03	99	-1.7	9676	-0.07	119	-5.17	11760	0.03	88	1.26	7875
TMEM8B_t2	1.07	0.055732698	-0.02	130	-1.7	9677	0.01	124	1.04	7781	-0.02	113	-1.47	9434
PRAMEF16	1.01	0.861368083	-0.03	100	-1.7	9678	-0.11	94	-5.06	11693	0.04	100	1.76	7594
UQCC	1.1	0.001267303	-0.02	120	-1.7	9679	0.01	145	0.94	7841	0.07	110	4.38	6258
APOL5	1.09	0.04455354	-0.03	111	-1.7	9680	-0.02	95	-0.76	8980	0.07	87	2.5	7181
RABGEF1_t1	1	0.951391997	-0.02	131	-1.7	9681	NA	NA	NA	NA	0	148	0.07	8572
CCDC37	1	0.927979325	-0.04	88	-1.71	9682	-0.15	98	-6.98	12640	-0.01	88	-0.23	8755
ADAMTSL5_t1	1.02	0.180952161	-0.03	113	-1.71	9683	0.06	122	4.51	5609	NA	NA	NA	NA
PLD4	1.01	0.261031895	-0.06	81	-1.72	9684	-0.06	106	-3.39	10727	0	100	-0.12	8686
LHFPL5	1.02	0.210501216	-0.02	128	-1.72	9685	-0.08	138	-6.77	12542	0	114	0.27	8457
NRP2_t1	-1.02	0.053201216	-0.05	88	-1.72	9686	-0.04	103	-2.08	9879	0.02	82	0.7	8193
DHRS7	-1.03	0.644281938	-0.03	117	-1.72	9687	-0.03	116	-1.96	9785	-0.04	110	-2.16	9774
WRN	-1.08	0.025614644	-0.02	124	-1.72	9688	0.05	111	3.06	6466	-0.04	145	-4.27	10743
MED12L	1	0.856686978	-0.02	130	-1.72	9689	0.06	131	4.78	5457	0.02	160	2.72	7071
MGAT4C	-1.01	0.821127251	-0.1	68	-1.72	9690	-0.02	78	-0.55	8852	-0.12	56	-0.73	9045
SSTR3	1.05	0.001598143	-0.03	108	-1.73	9691	-0.09	96	-4.21	11215	0.02	100	0.79	8146
IL12RB2	1.01	0.82645827	-0.02	120	-1.73	9692	-0.04	108	-2.44	10112	-0.09	124	-6.5	11645
SYMPK	-1.09	0.009680596	-0.03	107	-1.73	9693	-0.04	80	-1.35	9401	-0.18	95	-8.11	12270
RUNX2_t1	1.03	0.090113338	-0.02	138	-1.73	9694	0.12	152	11.78	2199	0.03	154	2.9	6989
CACNB3	1.03	0.496030822	-0.03	103	-1.73	9695	0.02	136	2	7140	-0.07	119	-4.57	10875
KLK2_t1	1.03	0.000382923	-0.02	159	-1.74	9696	-0.12	146	-11.45	14359	0.05	122	3.67	6583
FAM124B	1.13	0.019814447	-0.03	117	-1.74	9697	0.01	115	0.85	7901	0.01	133	1.1	7964
CCR3	-1.01	0.259736952	-0.03	103	-1.74	9698	0.04	127	2.74	6650	0.05	107	2.67	7097
NTN3	-1.08	0.000516805	-0.12	64	-1.74	9699	-0.19	88	-7.18	12727	-0.21	68	-3.79	10527
GAL3ST3	-1.02	0.053955178	-0.02	121	-1.75	9700	-0.12	123	-8.41	13291	-0.05	117	-3.04	10197
EMX2	1.01	0.312289417	-0.02	122	-1.75	9701	-0.03	130	-2.68	10265	0.07	154	6.97	5050
TMEM119	1.02	0.151257431	-0.02	120	-1.75	9702	0.09	148	9.3	3146	0	123	-0.17	8717
XRCC3	1.02	0.22772497	-0.03	109	-1.75	9703	-0.13	118	-8.73	13413	0.02	104	1.16	7939
CIC	-1.01	0.874436779	-0.02	123	-1.75	9704	0	108	-0.17	8593	-0.1	135	-8.14	12285
BCAR1	-1.11	0.01386324	-0.03	104	-1.75	9705	-0.06	94	-2.46	10127	-0.13	100	-6.46	11626
TSPAN10_t1	-1.06	0.012478631	-0.05	87	-1.76	9706	NA	NA	NA	NA	-0.07	104	-4	10640
OTOS	-1.04	0.052199518	-0.08	72	-1.76	9707	-0.02	120	-1.58	9536	-0.14	120	-10.02	12932
PLA2G2A	-1.21	0.2471282	-0.03	114	-1.76	9708	-0.16	112	-9.7	13772	-0.12	111	-7.25	11938
CASC1	1.01	0.434589611	-0.02	143	-1.76	9709	0.08	110	4.99	5342	-0.1	122	-7.05	11862
NXF5	1.13	0.008013627	-0.02	121	-1.76	9710	0.01	105	0.75	7971	0	116	-0.11	8684
FOXC2	-1.18	0.000399856	-0.08	72	-1.76	9711	-0.18	104	-9.7	13773	-0.16	80	-4.9	11015
DAB2IP_t1	1.04	0.001125647	-0.04	92	-1.76	9712	0.02	88	0.69	8007	-0.07	102	-3.62	10443
SLC22A18	1.02	0.841683462	-0.06	80	-1.77	9714	-0.04	114	-2.84	10367	0.02	78	0.55	8293
RBMS1_t1	-1.03	0.02120226	-0.02	147	-1.77	9715	0.04	129	3.31	6312	0.02	147	1.98	7461
ATN1	1.01	0.633438731	-0.03	111	-1.77	9716	0.01	132	0.81	7933	-0.03	106	-1.47	9431
MIA	1.11	0.007808532	-0.04	92	-1.77	9717	0.05	89	2.04	7117	-0.07	80	-2.22	9803
LHX6	-1.04	0.190458362	-0.02	126	-1.77	9718	0.06	129	4.91	5387	-0.09	126	-6.48	11629
GFRA3	1.01	0.531995839	-0.03	102	-1.77	9719	-0.09	121	-6.05	12213	-0.08	142	-7.76	12147
Sep-12	1.03	0.078911814	-0.03	101	-1.78	9720	-0.07	115	-4.44	11350	-0.01	104	-0.5	8913
MRPL41	-1.1	0.038360428	-0.09	70	-1.78	9721	-0.12	94	-5.17	11757	-0.09	95	-3.85	10560
GYPA_t1	-1.01	0.295175817	-0.02	134	-1.78	9722	0.01	145	0.81	7929	NA	NA	NA	NA
PANX3	1.08	3.78E-10	-0.03	119	-1.78	9723	0.06	114	4.05	5882	0.06	106	3.27	6790
ARID3B	1.21	1.23E-06	-0.03	119	-1.79	9724	0.18	131	14.83	1320	0.04	130	3.28	6786
UQCRFS1	-1.04	0.266436117	-0.02	145	-1.79	9725	-0.05	113	-3.2	10597	-0.06	110	-3.8	10537
RAP1GAP2_t1	1.01	0.731654786	-0.02	129	-1.79	9726	NA	NA	NA	NA	0.01	127	0.68	8209
CNGB3	1.03	0.019234436	-0.02	130	-1.79	9727	0.01	121	1.03	7791	0.02	130	1.94	7490
CHRNG	1.02	0.050603581	-0.03	104	-1.79	9728	-0.11	131	-8.9	13467	0.02	123	1.47	7770
CA1	-1.14	0.000444582	-0.02	136	-1.79	9729	-0.18	106	-10.21	13960	-0.18	99	-8.93	12571
IAH1	1.04	0.269890458	-0.03	106	-1.79	9730	0	107	-0.19	8607	0	117	-0.03	8640
PCBP2	-1	0.989155409	-0.02	128	-1.79	9731	-0.1	121	-7.45	12849	-0.03	123	-1.94	9690
DCD	-1.06	0.263421377	-0.08	73	-1.79	9732	-0.05	88	-1.94	9766	0.03	83	1.07	7981
KCNH7_t1	1.03	0.008658132	-0.05	84	-1.79	9733	0.15	60	1.53	7476	0.03	76	0.68	8206
GBA2	1.04	0.281812929	-0.03	115	-1.8	9734	-0.11	111	-6.75	12535	0.05	96	2.24	7325
KLK15_t2	-1.01	0.652923683	-0.03	104	-1.8	9735	-0.04	113	-2.24	9990	-0.04	115	-2.79	10084
CHID1_t1	-1.06	0.210483661	-0.02	130	-1.8	9736	NA	NA	NA	NA	-0.1	120	-6.76	11759
KRTAP6-3	1.11	2.93E-07	-0.03	104	-1.8	9737	-0.05	118	-3.64	10902	0.08	134	6.97	5047
PCDH11X	1	0.751596907	-0.03	112	-1.81	9738	0.04	114	2.34	6926	0.01	131	0.71	8189
NTHL1	1.14	0.004732062	-0.04	100	-1.81	9739	-0.02	114	-1.43	9452	0.05	108	2.83	7022
GTF2E2	-1.19	4.05E-05	-0.02	132	-1.81	9740	-0.11	111	-6.52	12418	-0.12	112	-7.27	11951
PPARA	-1.02	0.466176503	-0.02	154	-1.81	9741	0.01	135	1.02	7793	-0.02	142	-2.03	9719
DEFB106B_t1	-1.02	0.201954107	-0.04	95	-1.81	9742	NA	NA	NA	NA	-0.01	107	-0.51	8918
GLTP	-1.06	0.083442363	-0.02	165	-1.82	9743	0.03	143	2.34	6929	-0.1	152	-9.71	12814
KIAA0355	1.01	0.908844621	-0.02	135	-1.82	9744	0.06	129	4.44	5640	-0.13	134	-10.54	13099
FKBP1B	-1.06	0.605404583	-0.02	129	-1.82	9745	0	124	-0.29	8671	-0.2	115	-12.86	13806
PPIG	-1.21	0.001302283	-0.03	109	-1.82	9746	0.04	95	1.77	7303	-0.08	131	-6.22	11535
RNF114	1.02	0.49759683	-0.03	117	-1.82	9747	0.07	140	6.48	4524	-0.16	130	-13.15	13884
SCEL	1.02	0.385704584	-0.02	133	-1.83	9748	0.08	147	7.45	4011	-0.04	123	-3.21	10272
YRDC	-1.08	0.025707137	-0.02	128	-1.83	9749	-0.04	144	-3.94	11066	-0.03	148	-2.69	10033
CETN1	1.02	0.060665257	-0.03	116	-1.83	9750	-0.01	118	-0.7	8943	0.07	111	4.02	6409
ARHGEF40	1.05	0.478811198	-0.03	120	-1.83	9751	0.09	129	7.11	4188	0	120	-0.02	8636
ISL1	-1	0.931616077	-0.02	126	-1.83	9752	-0.01	113	-0.9	9075	-0.07	135	-5.67	11305
RASSF8	1.02	0.165483539	-0.02	128	-1.83	9753	0.11	140	9.53	3044	0.06	120	4.16	6349
RAMP2	-1.05	0.024762544	-0.03	112	-1.83	9754	-0.02	123	-1.24	9322	-0.15	115	-9.88	12889
OR2A9P	-1.01	0.770770547	-0.03	114	-1.83	9755	0.01	123	1.08	7749	-0.1	143	-9.11	12631
PBXIP1	1.05	0.191435241	-0.02	136	-1.83	9756	-0.08	130	-6.8	12562	0.01	124	0.67	8216

ERH	1.05	0.153929538	-0.03	120	-1.83	9757	-0.03	136	-2.26	10003	0.01	111	0.33	8418
EPB41L1_t1	1.06	0.397817137	-0.02	126	-1.83	9758	-0.06	132	-4.72	11499	NA	NA	NA	NA
CACNA2D3	1.07	0.025307226	-0.02	124	-1.83	9759	-0.02	119	-1.3	9366	-0.08	110	-4.79	10982
AP4M1	1.01	0.784964538	-0.03	114	-1.83	9760	-0.07	95	-3.15	10575	-0.01	94	-0.29	8786
TCEA3	-1.09	0.295058744	-0.03	114	-1.84	9761	-0.13	99	-6.18	12278	0.05	111	3.28	6787
PGLS	1.04	0.182964384	-0.04	100	-1.84	9762	0.1	115	6.76	4371	-0.11	92	-4.51	10842
NHLH2	1	0.760367542	-0.03	123	-1.84	9763	-0.07	119	-5.02	11668	0	168	-0.4	8849
SMARCD3	-1.21	0.00922549	-0.05	84	-1.84	9764	0.02	120	1.4	7555	-0.2	91	-8.2	12313
TMEM163	1.1	0.005463044	-0.03	115	-1.84	9765	0.14	120	9.86	2913	-0.03	119	-1.89	9661
AZU1	-1.01	0.387087119	-0.12	66	-1.84	9766	-0.1	116	-6.36	12354	-0.09	84	-3.2	10271
METTL21B	1.09	3.53E-07	-0.02	139	-1.84	9767	0.11	141	9.68	2990	0.06	134	4.7	6072
ZNF490	1.12	8.84E-13	-0.02	126	-1.85	9768	0.01	137	0.62	8055	0.11	147	10.3	3762
INSL3	1.02	0.391972492	-0.04	101	-1.85	9769	0	129	-0.09	8547	-0.05	88	-2.08	9745
ZNF205	-1.25	1.66E-05	-0.03	120	-1.85	9770	-0.08	109	-4.56	11422	-0.1	115	-6.68	11728
NMUR2	1.03	0.005302791	-0.03	120	-1.85	9771	-0.16	120	-11.29	14321	-0.01	164	-0.87	9119
SCYL1_t1	-1.07	0.026112521	-0.04	92	-1.86	9772	0	138	0.23	8311	-0.11	110	-6.5	11640
TMEM176A	-1.29	0.000425015	-0.03	111	-1.86	9773	-0.19	127	-14.52	15108	-0.06	100	-2.87	10122
ACE	1.01	0.454699952	-0.03	105	-1.86	9774	-0.1	139	-9.07	13546	0.01	116	0.73	8173
ASB3	-1	0.914663953	-0.03	124	-1.86	9775	0.08	107	4.45	5637	-0.05	113	-3.04	10199
DEFB118	-1.01	0.527545562	-0.03	120	-1.86	9776	0.09	144	8.34	3579	-0.03	118	-2.01	9711
DNAJC5G	1.04	0.015473303	-0.03	109	-1.86	9777	-0.09	117	-5.77	12068	0.01	136	0.68	8208
TSC2	1.03	0.516081012	-0.04	92	-1.86	9778	-0.03	92	-1.33	9383	-0.01	120	-0.5	8911
UPK3A	1	0.958175563	-0.05	88	-1.86	9779	0.02	119	1.58	7442	-0.05	107	-2.94	10154
CNTN1	1.03	0.263621043	-0.02	133	-1.87	9780	-0.05	140	-4.61	11448	-0.01	130	-1.09	9239
CYYR1	-1.05	0.297188362	-0.03	124	-1.87	9781	-0.02	131	-1.98	9805	0.04	131	3.08	6884
ZC3H10	1.12	1.95E-05	-0.02	127	-1.87	9782	0.07	147	6.62	4439	0.07	100	3.37	6732
AP4S1	-1.09	0.008901754	-0.02	144	-1.87	9783	-0.02	133	-1.63	9576	-0.15	124	-10.98	13250
CMTM8	1.01	0.90791254	-0.03	122	-1.87	9784	-0.04	117	-2.54	10171	0.09	119	6.49	5245
HSD17B11_t1	-1.24	0.003497582	-0.02	130	-1.87	9785	NA	NA	NA	NA	0.03	154	2.74	7058
CALB2	1.06	0.00259581	-0.03	115	-1.87	9786	0.09	119	6.38	4575	0.03	112	1.67	7639
AWAT1	1.01	0.593849367	-0.03	121	-1.88	9787	-0.09	129	-7.39	12828	-0.03	102	-1.6	9501
HIST1H3I	-1.05	0.212158331	-0.13	64	-1.88	9788	-0.05	108	-3.11	10554	-0.12	70	-2.45	9925
FAM9C	-1.01	0.189272845	-0.02	128	-1.88	9789	-0.01	106	-0.4	8751	0.04	127	3.42	6703
SPATA25	1.02	0.78803298	-0.19	60	-1.88	9790	-0.1	66	-1.58	9534	-0.12	101	-5.88	11398
FITM1	-1.33	1.23E-14	-0.31	56	-1.89	9791	-0.27	56	-1.59	9550	-0.31	58	-2.52	9961
RAB39B_t1	-1.04	0.001248632	-0.02	145	-1.89	9792	0.03	130	2.43	6869	NA	NA	NA	NA
SPINK2	-1.04	0.129453784	-0.02	128	-1.89	9793	-0.03	101	-1.71	9630	-0.07	135	-6.18	11516
PPEF2	-1	0.720815071	-0.02	132	-1.9	9794	-0.05	121	-3.71	10938	0.01	134	1.24	7887
PAPL	1.06	0.00020191	-0.03	123	-1.9	9795	0.02	131	1.88	7226	-0.01	109	-0.52	8929
ADCY4	-1.12	0.015940641	-0.06	81	-1.9	9796	-0.01	110	-0.6	8877	-0.12	145	-11.47	13395
EEF1B2_t1	-1.07	0.09697052	-0.06	83	-1.9	9797	NA	NA	NA	NA	NA	NA	NA	NA
PACRG	-1.01	0.811552736	-0.03	125	-1.9	9799	-0.1	115	-6.76	12540	-0.1	124	-7.3	11958
TMEM214	-1.05	0.009513647	-0.03	110	-1.9	9800	-0.15	126	-11.68	14421	-0.19	125	-14.27	14176
GNL1	1.02	0.473235875	-0.02	159	-1.9	9801	-0.18	125	-13.46	14862	-0.1	132	-8.27	12349
EHD2_t1	1.05	0.479470381	-0.02	133	-1.91	9802	0.07	121	5.18	5230	NA	NA	NA	NA
RYR3	1.03	0.171813261	-0.02	128	-1.91	9803	0.01	129	0.67	8024	0.01	134	1.01	8013
KISS1	1.01	0.430431651	-0.05	88	-1.91	9804	-0.13	86	-4.52	11398	-0.02	100	-0.86	9113
CLCA4	1.01	0.168425081	-0.02	143	-1.91	9805	-0.03	120	-1.97	9799	0.04	123	3.21	6828
PRSS27	-1.02	0.092724887	-0.04	100	-1.91	9806	-0.1	111	-6.08	12231	-0.09	125	-6.86	11798
APOBEC3H	-1.02	0.53004223	-0.03	108	-1.91	9807	0.04	109	2.6	6759	-0.12	126	-9.12	12636
TRIM26_t1	-1	0.867592394	-0.03	120	-1.92	9808	NA	NA	NA	NA	NA	NA	NA	NA
PTPRD	-1.03	0.333180836	-0.02	168	-1.92	9809	-0.06	147	-5.95	12160	-0.05	152	-5.15	11097
FBXO6	1.08	0.021329249	-0.04	95	-1.92	9810	-0.07	135	-6.3	12324	0	120	0.12	8545
WDR3	1.01	0.842236603	-0.03	120	-1.92	9811	0.06	130	5.09	5279	0.07	119	4.77	6041
WBP11	-1.03	0.324938163	-0.02	138	-1.92	9812	0	112	-0.13	8568	-0.01	122	-0.95	9165
DUSP15	-1.17	3.68E-05	-0.04	100	-1.93	9813	-0.08	104	-4.25	11242	-0.12	104	-6.36	11596
TCP11	1.02	0.398260245	-0.04	100	-1.93	9814	-0.02	109	-0.92	9095	0.08	110	4.82	6020
GML	1.02	0.133165026	-0.06	84	-1.93	9815	-0.1	111	-6	12186	-0.01	120	-0.44	8879
C2CD4B	-1.24	1.40E-05	-0.03	107	-1.93	9816	-0.08	143	-7.25	12757	-0.11	128	-8.24	12328
PLBD1	-1.24	0.02304238	-0.04	96	-1.93	9817	0.18	97	8.31	3587	-0.16	118	-10.9	13218
ARMC12	1.02	0.223329346	-0.07	78	-1.93	9818	-0.04	52	-0.08	8544	-0.02	50	0	8620.5
TEX14_t1	1	0.735391672	-0.04	99	-1.93	9819	0.02	117	1.17	7694	0.01	98	0.45	8342
CSGALNACT1_t	-1.02	0.462170453	-0.03	111	-1.94	9820	NA	NA	NA	NA	0.01	146	0.8	8132
STRAP_t1	-1.01	0.785622112	-0.03	120	-1.94	9821	NA	NA	NA	NA	0.04	115	2.54	7165
DALRD3	1.14	4.93E-06	-0.03	113	-1.94	9822	0.03	142	3	6496	0.05	104	2.66	7100
CYP17A1	1.54	1.34E-07	-0.05	91	-1.94	9823	-0.06	117	-3.83	10993	0.16	94	7.1	4993
SPRR2E	1.01	0.816976366	-0.04	98	-1.94	9824	-0.04	134	-3.57	10858	-0.11	118	-7.57	12054
PSMD7	1.02	0.749647373	-0.03	121	-1.95	9825	-0.05	115	-3.53	10827	-0.07	131	-5.8	11366
COG8_t1	-1.09	0.02838196	-0.02	129	-1.95	9826	NA	NA	NA	NA	-0.04	129	-3.42	10365
PSG1	1.03	0.01631325	-0.02	130	-1.95	9827	0	124	-0.01	8492	0.01	122	0.85	8112
TNNC1	1.17	6.55E-06	-0.05	88	-1.95	9828	0	100	-0.14	8571	-0.02	88	-0.7	9034
LGALS1	-1.07	0.421406918	-0.1	69	-1.95	9829	0.16	88	5.98	4797	-0.11	68	-2.01	9712
GFI1B	-1.01	0.551571807	-0.05	92	-1.95	9830	-0.08	116	-5.23	11790	-0.05	112	-3.13	10237
CYFIP1	-1.1	0.004122246	-0.03	120	-1.95	9831	-0.03	127	-2.3	10020	0.01	120	0.48	8328
TFDP3	1.08	0.01941623	-0.03	117	-1.95	9832	0.07	104	3.65	6087	0.07	133	5.99	5459
PARP15	1.03	0.040929776	-0.03	116	-1.96	9833	0.06	120	4.54	5592	-0.06	113	-3.85	10568
MTA1	-1.02	0.444470889	-0.03	126	-1.96	9834	0.01	113	0.39	8211	-0.07	123	-5.1	11080
EPHA8_t2	-1.01	0.568922876	-0.04	94	-1.96	9835	0.01	113	0.6	8066	-0.14	93	-6.08	11473
ATOH7	1.01	0.644189113	-0.02	140	-1.96	9836	-0.09	115	-6.17	12269	0.04	117	2.35	7263
PGLYRP2	-4.7	2.14E-25	-0.33	56	-1.97	9837	-0.36	84	-12.4	14615	-0.32	64	-4.47	10832
RGS22	1.03	0.277846404	-0.04	103	-1.97	9838	-0.11	97	-5.13	11737	0	98	0.14	8527
SYT4	-1.04	0.001532738	-0.02	152	-1.97	9839	-0.08	124	-5.56	11948	-0.12	123	-8.8	12526
MAST4	-1.18	1.29E-05	-0.2	60	-1.97	9840	-0.14	94	-6.2	12288	-0.25	89	-9.73	12823
KAL1	-1.09	0.011045463	-0.02	134	-1.98	9841	0.03	133	2.34	6928	-0.11	144	-10.7	13151
REM1	1.01	0.32203324	-0.03	128	-1.98	9842	0.03	109	1.77	7304	0.04	115	2.72	7072
ALDH4A1_t1	-1.08	0.159777131	-0.03	121	-1.98	9843	-0.09	110	-5.24	11794	-0.05	101	-2.67	10024
NELL1	1.14	0.026784357	-0.03	117	-1.98	9844	-0.03	123	-2.54	10173	-0.02	113	-1.26	9322
SCAMP5	1.07	0.141238282	-0.03	129	-1.98	9845	0.07	150	7.38	4044	0.06	120	4.42	6240
UACA_t1	-1.02	0.569305856	-0.02	146	-1.98	9846	NA	NA	NA	NA	0.09	125	7.05	5007
TREX1	-1.16	0.001421859	-0.09	72	-1.98	9847	-0.17	76	-4.38	11302	-0.21	68	-3.75	10511
AP1B1	-1.09	0.001119954	-0.04	105	-1.98	9848	-0.09	140	-8.06	13133	-0.13	120	-9.13	12638
TM4SF18	-1.02	0.686274571	-0.02	148	-1.99	9849	0.03	143	2.67	6696	0.12	131	9.33	4110
OCLM	1	0.791483328	-0.03	120	-1.99	9850	-0.09	101	-4.49	11383	0.01	142	1.22	7903
GOLGA2P2Y	1.03	0.403762226	-0.04	98	-1.99	9851	0.05	131	4.15	5810	0.04	120	2.71	7078
ZBBX	-1	0.647740222	-0.03	119	-1.99	9852	-0.04	125	-3.31	10687	0.01	121	0.96	8038
RETN	-1.11	5.00E-05	-0.09	72	-1.99	9853	-0.12	84	-4.09	11147	-0.2	100	-10	12925
DEPTOR	1.09	0.096703891	-0.03	123	-1.99	9854	-0.02	111	-1.22	9309	0.13	150	13.48	2746
PPARGC1B	1.05	0.189028042	-0.02	143	-1.99	9855	-0.03	154	-3.31	10676	-0.02	144	-2.33	9856
CEP120	-1.04	0.123257138	-0.02	134	-1.99	9856	-0.07	119	-5.09	11716	0.08	145	7.26	4924
ATP6AP2	1.05	0.264488416	-0.02	131	-1.99	9857	0.1	113	6	4781	0.11	124	7.87	4678
TM9SF2_t1	-1.03	0.524790888	-0.03	123	-2	9858	-0.07	130	-5.61	11980	NA	NA	NA	NA
AVPR1B	1.01	0.6139983	-0.04	95	-2	9859	-0.11	111	-6.52	12422	0.02	100	1.15	7944
CORT_t1	1.11	0.008472867	-0.03	110	-2	9860	NA	NA	NA	NA	0.11	100	5.57	5640
SARNP	1.04	0.25303588	-0.03	114	-2	9861	0.1	100	4.87	5411	0.01	104	0.43	8349
NDUFS2	1.16	0.001258336	-0.04	104	-2	9862	0.03	113	1.93	7184	0.11	104	5.79	5541
GAST	-1.04	0.429432196	-0.07	78	-2	9863	-0.21	120	-14.55	15116	-0.19	80	-5.63	11289

MTRR	1.02	0.479356976	-0.02	137	-2.01	9864	0.13	123	9.49	3063	0.03	142	2.86	7014
NME4	-1.2	2.79E-05	-0.03	111	-2.01	9865	-0.1	119	-6.78	12550	-0.12	124	-8.6	12457
GTF2H1	1.01	0.835568216	-0.02	148	-2.01	9866	0.09	129	7.16	4158	-0.03	158	-3.06	10210
TAS2R13	1.05	6.78E-05	-0.02	132	-2.01	9867	-0.05	114	-3.3	10668	0.06	124	4.63	6113
CD7	1.02	0.414249871	-0.05	88	-2.01	9868	-0.01	128	-1.16	9260	-0.2	100	-9.86	12874
SF3B2	1.1	0.00051372	-0.04	103	-2.01	9869	0.04	111	2.37	6903	-0.02	101	-1.11	9255
FFAR1	-1.02	0.220145921	-0.13	66	-2.01	9870	-0.19	88	-7.3	12785	-0.07	80	-2	9706
CTPS2	-1	0.888873567	-0.02	133	-2.01	9871	-0.08	135	-7.13	12713	0.06	138	5.19	5831
MOCS2	-1	0.943424198	-0.03	109	-2.01	9872	-0.13	123	-9.55	13715	0.13	104	7.15	4971
CRYBB1	-1.01	0.43511567	-0.04	103	-2.01	9873	-0.03	104	-1.42	9443	0.02	101	0.84	8115
HCRT	-1.14	2.61E-12	-0.11	68	-2.02	9874	-0.16	83	-5.44	11889	-0.17	88	-6.49	11635
SLC35C2	-1.02	0.493959781	-0.03	111	-2.02	9875	-0.1	122	-7.22	12744	-0.07	113	-4.51	10839
AP3B2	1.02	0.09799551	-0.04	103	-2.02	9876	0.04	120	2.65	6715	-0.01	103	-0.29	8788
SPHK1	-1.07	0.493120636	-0.03	128	-2.02	9877	0.03	133	2.67	6699	-0.11	114	-6.81	11777
PSMB6	-1.12	0.010339423	-0.05	89	-2.02	9878	-0.16	87	-6.03	12203	-0.2	104	-10.73	13162
KRTAP4-2	1.02	0.029150888	-0.03	124	-2.02	9879	-0.06	123	-4.62	11455	0.09	119	6.22	5347
TGIF1	-1.26	2.77E-05	-0.03	113	-2.03	9880	-0.08	119	-5.53	11934	-0.1	142	-9.36	12701
SCN4B	1.1	0.04308607	-0.02	135	-2.03	9881	0	139	0.18	8343	0.07	141	6.69	5159
SEC11A	1.1	0.031492942	-0.03	124	-2.03	9882	0.13	148	12.3	2036	0.05	128	3.65	6591
GAN	-1.04	0.022334787	-0.03	126	-2.03	9883	-0.07	114	-4.41	11331	-0.08	131	-6.75	11757
TTC9C	-1.03	0.297924538	-0.03	117	-2.03	9884	-0.07	108	-4.26	11249	-0.03	118	-2.33	9855
NPAS3_t1	-1.03	0.055008075	-0.02	152	-2.03	9885	-0.19	150	-18.68	15823	-0.07	134	-5.8	11364
ANTXR1_t1	1.02	0.47523377	-0.05	95	-2.04	9886	0.07	131	5.38	5132	-0.1	107	-5.96	11428
RCHY1	-1.08	0.038269248	-0.02	140	-2.04	9887	0.02	134	2.09	7088	0.02	131	1.44	7786
KRT32	1	0.944529293	-0.03	120	-2.04	9888	-0.17	134	-14.37	15074	-0.03	113	-1.94	9691
COX18	-1.05	0.000871402	-0.02	152	-2.04	9889	-0.03	139	-2.4	10088	0.03	150	3.32	6763
SEPHS2	-1.08	0.241089474	-0.03	118	-2.04	9890	-0.05	123	-3.43	10748	0.03	131	2.7	7089
KCNA5	-1.01	0.736488952	-0.02	138	-2.05	9891	0.07	130	5.81	4888	-0.01	124	-0.7	9031
IFRD1	-1.08	0.152899377	-0.02	143	-2.05	9892	0	134	0.02	8454	0.03	122	2.4	7234
TCTN2	1.08	3.32E-10	-0.03	111	-2.05	9893	0	114	-0.09	8545	0.05	132	3.92	6458
NOP14	1	0.910217412	-0.05	91	-2.05	9894	-0.15	99	-7.2	12737	0.08	100	3.79	6531
VWC2	1.02	0.021520401	-0.03	124	-2.05	9895	0.08	121	5.58	5021	0.13	119	9.25	4133
ACTN3	1.01	0.844051523	-0.06	86	-2.06	9896	-0.01	84	-0.3	8677	-0.16	100	-7.99	12234
MAN2B1	-1.18	2.28E-05	-0.06	84	-2.06	9897	-0.17	118	-11.24	14312	-0.26	83	-8.6	12458
LIMS3	-1.07	0.303786094	-0.04	109	-2.07	9898	0.12	121	8.27	3613	-0.09	111	-5.57	11263
PRM1	-1.04	0.00076796	-0.04	100	-2.07	9899	-0.2	117	-13.17	14788	-0.09	120	-6.57	11672
TADA3_t1	-1.01	0.629674187	-0.03	116	-2.07	9900	NA	NA	NA	NA	-0.1	111	-6.04	11451
IFNGR1	-1.11	0.198129812	-0.03	118	-2.08	9901	0.09	118	5.9	4844	-0.07	138	-6.38	11605
ACTRT2	-1.01	0.388452623	-0.07	80	-2.08	9902	-0.11	88	-4.19	11199	-0.07	76	-1.8	9613
HYI_t1	-1.08	0.139395992	-0.03	115	-2.08	9903	NA	NA	NA	NA	-0.02	104	-0.99	9195
SNW1	1.01	0.787531922	-0.03	131	-2.08	9904	-0.04	115	-2.84	10365	-0.02	119	-1.65	9525
TSPAN9	-1.31	4.30E-09	-0.02	139	-2.08	9905	-0.11	125	-8.39	13283	-0.17	142	-15.34	14448
ATF7IP2_t1	-1.02	0.295867738	-0.03	120	-2.09	9906	NA	NA	NA	NA	0.03	151	2.88	7002
RLIM	-1.08	0.029777137	-0.02	153	-2.09	9907	0.04	140	3.23	6354	-0.06	158	-5.95	11422
H1F0	1.17	0.002824934	-0.03	129	-2.09	9908	0.04	140	3.45	6215	0.07	138	6.39	5280
DMWD	-1.09	6.10E-05	-0.04	102	-2.09	9909	-0.06	132	-4.76	11516	-0.12	110	-7.18	11911
CALY	-1.02	0.170229331	-0.1	72	-2.1	9910	-0.18	95	-8.24	13209	-0.06	102	-3.04	10202
SLTM	-1.03	0.4782949	-0.02	153	-2.1	9911	-0.04	115	-2.88	10397	0.12	135	10.14	3822
ALG11	-1.09	0.008197548	-0.03	133	-2.1	9912	-0.01	108	-0.69	8929	-0.09	117	-5.7	11322
DET1	1.16	0.002709013	-0.03	116	-2.1	9913	0.06	115	3.86	5971	0.11	138	9.41	4082
LY6D	1.04	0.020878917	-0.03	115	-2.1	9914	-0.05	157	-5.63	11991	-0.04	146	-4.17	10702
POU3F2	-1.02	0.295627285	-0.03	123	-2.1	9915	-0.16	134	-13.05	14764	-0.01	166	-0.73	9048
NPR2	1.05	0.405359745	-0.04	108	-2.1	9916	-0.01	132	-0.98	9135	-0.08	100	-4.06	10664
PITPNC1	1.06	0.006413372	-0.02	150	-2.1	9917	0.05	142	4.14	5813	0.05	134	4.18	6343
RPL10L	-1.08	0.071149417	-0.04	107	-2.11	9918	-0.05	82	-1.65	9589	-0.11	90	-4.6	10892
STX5	1.05	0.042094874	-0.02	146	-2.11	9919	-0.03	117	-1.73	9637	-0.07	120	-4.58	10880
SECISBP2	-1.05	0.183408029	-0.03	127	-2.11	9920	-0.01	113	-0.69	8928	0.04	112	2.39	7237
STON1-GTF2A1	-1.01	0.616765479	-0.04	105	-2.11	9921	0.14	106	7.59	3939	-0.02	117	-1.53	9463
LHFPL3	1.02	0.176353303	-0.03	132	-2.11	9922	0.09	129	6.88	4313	-0.01	117	-0.63	9001
TOX4	1.05	0.047555773	-0.03	133	-2.11	9923	-0.08	139	-7.28	12774	-0.01	128	-0.78	9075
EFCAB12	1.16	0.000149174	-0.04	106	-2.11	9924	-0.03	93	-1.36	9404	-0.02	104	-1.05	9224
FHL5	1.05	0.052993502	-0.03	112	-2.12	9925	0.08	127	6.17	4690	-0.01	128	-0.93	9151
PPARD	-1.02	0.723229924	-0.03	122	-2.12	9926	0.02	125	1.36	7569	-0.1	135	-8.18	12306
GAL3ST4	-1	0.774203602	-0.03	119	-2.12	9927	-0.01	116	-0.47	8795	-0.14	117	-9.29	12684
PIGZ	1.26	6.84E-06	-0.03	124	-2.12	9928	0.1	138	8.38	3550	0.11	129	9.05	4207
IKBKB_t1	-1.03	0.361724551	-0.03	134	-2.12	9929	NA	NA	NA	NA	-0.06	132	-4.84	10993
CLEC2D_t2	-1.08	0.051434758	-0.03	132	-2.12	9930	0.04	133	3.7	6063	-0.08	159	-9.16	12645
ASB9	-1.33	0.000200916	-0.03	129	-2.12	9931	-0.16	109	-9.44	13677	-0.04	123	-2.69	10032
CEACAM7	1.09	0.008098749	-0.02	136	-2.13	9932	0.15	138	12.82	1888	0.01	129	0.41	8363
KCNA10	1.02	0.034799094	-0.03	116	-2.13	9933	-0.05	88	-1.75	9652	0	60	0.02	8608
CRCP_t1	-1.1	0.00010984	-0.03	133	-2.13	9934	NA	NA	NA	NA	-0.1	156	-10.48	13082
CD96_t1	-1.11	0.029067477	-0.02	144	-2.13	9935	NA	NA	NA	NA	NA	NA	NA	NA
ZNF562	-1.03	0.214854672	-0.03	135	-2.13	9936	0.01	125	0.96	7828	0.02	134	1.79	7571
IRF3	-1.06	0.060587575	-0.04	101	-2.13	9937	-0.11	104	-6.12	12254	-0.18	71	-3.7	10486
WBSCR17	1.03	0.007118608	-0.03	120	-2.14	9938	-0.03	126	-2.65	10252	-0.01	127	-0.4	8853
ST6GALNAC2	1.23	7.73E-05	-0.03	120	-2.14	9939	0.03	129	1.99	7156	0.01	120	0.91	8070
AKAP17A	-1.1	0.043074868	-0.03	129	-2.14	9940	0.04	142	3.52	6173	-0.06	132	-4.72	10947
TRIML1	-1.02	0.034934904	-0.08	77	-2.14	9941	-0.12	109	-7.23	12750	-0.01	88	-0.41	8856
SMAD1_t1	-1.09	0.13562197	-0.02	156	-2.14	9942	NA	NA	NA	NA	-0.06	137	-5.42	11211
ASPHD1	1.22	0.009375421	-0.04	108	-2.14	9943	-0.01	120	-0.73	8956	0	108	0.16	8512
ADIPOQ	1.03	0.34697812	-0.03	125	-2.14	9944	-0.12	124	-8.83	13446	-0.04	124	-3.24	10286
PTH2	-1.01	0.756934372	-0.12	68	-2.15	9945	0.01	127	0.48	8151	-0.02	104	-1.1	9253
SYN3	1.05	0.000266672	-0.03	119	-2.15	9946	0.05	138	3.96	5926	0.04	121	2.65	7104
SLC39A11_t1	1.08	0.080957907	-0.04	110	-2.15	9947	NA	NA	NA	NA	0.05	136	4.68	6085
ELF5_t1	-1.01	0.432893173	-0.03	117	-2.15	9948	NA	NA	NA	NA	-0.05	135	-4.1	10681
NDUFB7_t1	-1.09	0.00859513	-0.12	68	-2.15	9949	NA	NA	NA	NA	NA	NA	NA	NA
COX5A	1.11	0.014458981	-0.02	141	-2.16	9950	-0.01	101	-0.75	8973	0.19	136	16.24	2021
FSIP2	-1	0.974301578	-0.04	108	-2.16	9951	0	91	0.11	8394	-0.13	104	-7.07	11870
SRBD1	-1.14	0.000107958	-0.03	124	-2.16	9952	-0.09	130	-7.1	12698	-0.08	130	-6.23	11538
PARK7	1.06	0.211582638	-0.03	121	-2.16	9953	0	113	0	8474	0.06	103	3.05	6905
LMOD3	-1.01	0.183240426	-0.03	136	-2.16	9954	0.04	136	3.32	6304	-0.02	131	-1.65	9522
FAM71E1	-1.01	0.824294374	-0.03	120	-2.17	9955	-0.03	88	-1.24	9324	-0.04	88	-1.54	9465
KRTAP11-1	-1.01	0.708933225	-0.03	121	-2.18	9956	-0.12	126	-9.27	13614	0.02	123	1.79	7573
ASCL1	1.09	0.172096298	-0.03	126	-2.18	9957	-0.06	152	-5.75	12058	0.05	149	4.49	6186
DDI1	1.03	0.009588568	-0.02	138	-2.18	9958	-0.01	112	-0.79	9013	0.05	126	3.95	6441
DDIT4	-1.03	0.784223839	-0.03	129	-2.18	9959	-0.05	114	-3.31	10681	-0.01	114	-0.96	9172
NHLRC2	-1.05	0.005958568	-0.03	128	-2.18	9960	-0.09	149	-8.87	13459	0.01	130	1.07	7982
AKAP8L	-1.05	0.086926354	-0.1	72	-2.18	9961	-0.1	88	-3.88	11021	-0.13	80	-3.79	10529
ASNA1	1	0.899568936	-0.04	104	-2.18	9962	-0.1	148	-9.69	13770	-0.04	122	-2.68	10028
ZFP161	-1.01	0.885985574	-0.03	117	-2.18	9963	-0.06	136	-5.14	11738	0.02	144	1.87	7524
ATL1	-1.06	0.053630786	-0.03	127	-2.19	9964	0.01	129	0.4	8204	-0.13	115	-8.51	12429
GTF2B	-1.17	2.14E-05	-0.02	145	-2.19	9965	-0.01	131	-0.9	9082	-0.19	117	-12.95	13831
PURG_t2	1.01	0.516781106	-0.03	120	-2.19	9966	-0.03	131	-2.12	9916	0	162	-0.08	8665
MYLPF	-1.01	0.43419704	-0.07	81	-2.19	9967	-0.19	117	-12.72	14698	-0.05	95	-2.46	9928
KRBA1	1.04	0.569116465	-0.04	104	-2.19	9968	0.17	110	10.19	2796	0.01	102	0.68	8207
ALPK2	-1.04	0.768405725	-0.03	120	-2.19	9969	0.08	110	4.91	5385	0.06	127	4.78	6037

FAM115A_t1	-1.06	0.013638797	-0.02	142	-2.19	9970	NA	NA	NA	NA	-0.03	122	-2.47	9936
MINK1	-1.05	0.006424198	-0.03	115	-2.19	9971	-0.06	124	-4.78	11527	-0.17	136	-14.92	14361
ELOF1	-1.02	0.530989751	-0.04	107	-2.2	9972	-0.14	125	-10.72	14140	-0.04	106	-2.03	9717
SYNPO2L	-1	0.966688715	-0.03	120	-2.2	9973	0.11	131	8.93	3315	-0.05	144	-5.06	11070
H1FNT	1	0.933736872	-0.06	89	-2.2	9974	-0.14	144	-12.71	14695	-0.04	98	-1.85	9640
SOBP	-1.09	0.38418012	-0.03	125	-2.2	9975	0.01	132	0.81	7926	-0.08	134	-7.1	11886
BHLHE23	-1.36	2.75E-07	-0.16	64	-2.2	9976	-0.18	93	-7.66	12960	-0.13	80	-3.98	10631
HMGB3P1	-1.09	0.281216725	-0.04	104	-2.2	9977	-0.12	127	-8.94	13479	0.08	127	6.42	5268
AIFM1	1	0.999317293	-0.05	96	-2.2	9978	-0.02	95	-1.07	9200	0.11	97	5.32	5768
MEIOB	1.09	0.015830075	-0.03	115	-2.21	9979	-0.03	100	-1.29	9352	0.03	136	2.8	7034
NAIP_t1	-1.16	0.006554706	-0.03	130	-2.21	9980	NA	NA	NA	NA	NA	NA	NA	NA
CDH23_t1	1.1	0.006806668	-0.04	105	-2.21	9981	NA	NA	NA	NA	0.02	124	1.61	7681
EPHB6	-1.1	0.051877001	-0.06	88	-2.21	9982	-0.06	122	-4.59	11437	-0.17	104	-8.96	12579
ANKRD46	-1.12	0.05829074	-0.03	122	-2.21	9983	-0.06	123	-4.49	11382	0.08	130	6.49	5239
STK11_t1	-1.09	0.015397637	-0.04	104	-2.21	9984	-0.05	107	-3.02	10482	NA	NA	NA	NA
ZBTB32	-1.05	0.230317785	-0.08	76	-2.21	9985	0.05	103	2.56	6789	-0.11	113	-7.16	11904
RPL36	-1.06	0.121918239	-0.04	104	-2.21	9986	0.01	88	0.26	8286	-0.16	88	-6.04	11452
PCSK1N_t1	-1.31	2.39E-06	-0.06	88	-2.21	9987	NA	NA	NA	NA	NA	NA	NA	NA
SLC22A11	1.16	0.000213399	-0.05	91	-2.22	9988	0.02	126	1.84	7250	-0.04	97	-1.66	9529
MYBPC3	-1	0.826489722	-0.04	102	-2.22	9989	-0.07	111	-4.02	11108	-0.07	131	-5.56	11259
TTC17	1	0.902912274	-0.03	127	-2.22	9990	0.01	134	0.78	7959	0.1	145	9.55	4041
OPRD1	-1.02	0.086441507	-0.04	104	-2.22	9991	-0.14	106	-8.01	13118	-0.06	87	-2.4	9892
IDH3B	-1.01	0.723033929	-0.06	88	-2.22	9992	-0.14	83	-4.77	11519	-0.07	71	-1.38	9378
RBMY2EP	-1.02	0.028138002	-0.03	128	-2.22	9993	-0.14	127	-11.14	14280	-0.03	128	-2.69	10034
SLC9A8	-1.01	0.432714219	-0.02	140	-2.22	9994	-0.16	140	-14.64	15133	-0.04	142	-3.79	10532
SPACA3	-1	0.910217412	-0.05	99	-2.22	9995	-0.04	77	-0.99	9142	-0.2	100	-9.81	12859
MYL5	-1.06	0.147340765	-0.05	99	-2.22	9996	-0.16	90	-6.34	12345	-0.07	73	-1.65	9524
ANP32B	-1.09	0.002237058	-0.04	107	-2.23	9997	-0.07	139	-5.99	12179	-0.19	116	-12.57	13719
KRT31	1.05	0.007176817	-0.04	102	-2.23	9998	-0.07	104	-3.59	10868	-0.01	111	-0.85	9107
NOTCH2	-1	0.946429186	-0.02	140	-2.23	9999	0.08	141	7.02	4232	-0.06	148	-6.3	11568
ASTN2_t1	1.06	0.104754849	-0.04	107	-2.23	10000	0.1	136	8.63	3446	-0.05	119	-3.12	10232
LRTM1	1.01	0.329433427	-0.05	98	-2.23	10001	-0.04	78	-1.26	9331	-0.01	115	-0.38	8839
PMS1	-1.02	0.733223018	-0.04	102	-2.23	10002	0.09	94	4.11	5840	0.05	122	3.34	6753
FAM123A	1.02	0.013930774	-0.03	125	-2.24	10003	0.04	112	2.44	6859	0.08	118	5.25	5805
ACPP_t1	1	0.757132157	-0.02	143	-2.24	10004	NA	NA	NA	NA	-0.03	142	-2.98	10173
FGF9	-1.01	0.802873667	-0.02	150	-2.24	10005	-0.03	136	-2.38	10071	-0.08	151	-7.69	12110
INHBA	-1.13	0.262802227	-0.04	100	-2.24	10006	0	142	-0.32	8697	0.01	105	0.8	8129
PRKCE	1.02	0.295741716	-0.02	144	-2.24	10007	-0.01	145	-1.06	9192	0.06	142	5.75	5559
TRO	-1.11	0.080296457	-0.04	105	-2.24	10008	0.07	108	4.1	5843	-0.04	114	-2.52	9964
ATP6V0E2	1.15	0.052971639	-0.04	104	-2.24	10009	-0.1	147	-9.94	13863	0.09	123	6.66	5169
FMO1	1.14	0.00889849	-0.04	113	-2.25	10010	0.04	118	2.48	6841	-0.04	113	-2.64	10011
SNX4	-1.03	0.529373362	-0.03	139	-2.25	10011	-0.06	134	-5.3	11819	0.04	134	3.4	6718
OVOL1	1	0.951419422	-0.03	130	-2.25	10012	-0.05	131	-3.92	11054	-0.06	131	-4.68	10929
IL5	1.02	0.076783618	-0.04	108	-2.25	10013	-0.05	119	-3.2	10601	0.1	108	5.75	5558
PAFAH1B1_t1	-1.25	0.00035379	-0.03	134	-2.26	10014	NA	NA	NA	NA	-0.08	143	-7.59	12066
COL12A1	-1.04	0.09053584	-0.03	134	-2.26	10015	0.03	114	2.14	7050	-0.07	131	-5.97	11429
SAFB2_t1	-1.03	0.130747992	-0.04	104	-2.26	10016	NA	NA	NA	NA	-0.07	106	-3.92	10598
TCEAL8	-1.11	0.311140391	-0.04	111	-2.26	10017	0.15	116	10.08	2834	-0.03	123	-1.92	9678
TULP2	1.02	0.112307043	-0.08	80	-2.26	10018	0.03	87	0.93	7847	0.01	89	0.41	8366
RIN2	-1.02	0.041267977	-0.03	134	-2.27	10019	0.08	121	5.45	5086	-0.02	136	-2.13	9770
HAGHL	-1.17	0.001442481	-0.05	98	-2.27	10020	-0.07	100	-3.53	10826	-0.06	88	-2.46	9930
AQP5	1.03	0.060116149	-0.05	97	-2.27	10021	-0.13	116	-8.38	13278	-0.02	121	-1.26	9320
MARVELD3_t2	-1.14	0.009640081	-0.02	144	-2.27	10022	-0.16	133	-13.59	14894	-0.09	132	-7.11	11889
PARP10	-1.01	0.565757693	-0.03	118	-2.27	10023	-0.08	113	-4.86	11568	-0.06	85	-2.05	9732
CGB2	-1.01	0.713200618	-0.16	64	-2.27	10024	-0.09	88	-3.39	10726	-0.04	60	-0.44	8877
PDAP1	1.08	0.030314331	-0.03	131	-2.27	10025	0.11	130	8.56	3469	0.06	149	5.88	5502
GFRA4	-1.27	1.70E-06	-0.03	131	-2.27	10026	-0.09	123	-6.43	12386	-0.05	120	-3.53	10410
SNPH	-1.03	0.213445276	-0.03	124	-2.28	10027	-0.05	136	-4.62	11452	-0.09	151	-8.93	12566
IL11	1.14	0.000152948	-0.03	124	-2.28	10028	0.02	112	1.29	7613	0.05	126	3.8	6528
PKD1	1.06	0.000525624	-0.03	116	-2.28	10029	0.07	122	5.22	5209	-0.04	111	-2.25	9821
TSPAN3	-1.07	0.31453001	-0.03	135	-2.28	10030	0.06	140	5.48	5069	-0.05	134	-4.01	10644
CDC26	-1	0.993199244	-0.04	115	-2.29	10031	-0.03	116	-2.09	9895	0	148	0.46	8337
ZNF621	1.05	1.39E-05	-0.03	138	-2.29	10032	-0.03	140	-2.57	10193	0.09	144	8.12	4575
KRTAP9-2	1.04	0.000493137	-0.02	143	-2.29	10033	-0.07	118	-4.67	11475	0.02	116	1.35	7833
CCL8	1.02	0.822390617	-0.04	104	-2.29	10034	0.06	127	4.89	5401	-0.02	128	-1.27	9331
CASC4_t2	-1.04	0.12915739	-0.03	130	-2.29	10035	-0.07	128	-5.44	11898	0	142	0.44	8344
PML_t4	-1.01	0.475063611	-0.04	103	-2.29	10036	0.03	136	2.49	6829	-0.1	155	-10.27	13015
NISCH	1.06	0.079459095	-0.04	104	-2.29	10037	0.04	113	2.64	6726	-0.03	104	-1.7	9550
BAZ1B	1.05	0.114462276	-0.03	126	-2.29	10038	0	145	0.22	8315	0.14	114	8.83	4287
RUFY2_t2	1.03	0.019339966	-0.02	145	-2.3	10039	-0.08	117	-5.62	11986	0.11	143	10.58	3660
GRID1_t1	-1.09	0.285822655	-0.03	130	-2.3	10040	NA	NA	NA	NA	-0.05	122	-3.39	10350
SFTPB	1.03	0.027831075	-0.02	152	-2.3	10041	-0.01	135	-0.92	9093	0	128	-0.16	8706
SCYL2	1.02	0.800085205	-0.03	140	-2.3	10042	0.11	113	6.78	4358	0.06	138	5.4	5734
TBC1D23	-1.08	0.026381885	-0.02	162	-2.3	10043	-0.02	117	-1.02	9156	-0.03	141	-2.39	9884
PITX3	-1.08	5.38E-05	-0.05	100	-2.31	10044	-0.17	106	-9.61	13743	-0.18	107	-10.22	12995
HAPLN3	-1.03	0.019168487	-0.03	118	-2.31	10045	-0.06	133	-5.16	11753	-0.1	115	-6.38	11603
RGCC_t1	-1.04	0.58112387	-0.04	114	-2.31	10046	0.05	110	3.19	6390	NA	NA	NA	NA
MTSS1L	-1.06	0.006771194	-0.03	118	-2.31	10047	-0.1	120	-7.3	12786	-0.08	124	-6.2	11524
ABCB7	-1.05	0.260743432	-0.03	117	-2.31	10048	-0.05	131	-4.17	11189	0.09	143	7.94	4646
PLA1A_t1	-1.33	0.001348396	-0.03	120	-2.31	10049	-0.08	111	-4.95	11625	NA	NA	NA	NA
XCL2	-1.09	0.174350632	-0.04	108	-2.31	10050	-0.02	113	-1	9150	-0.11	120	-7.4	11996
GPR56_t1	1.01	0.804044658	-0.04	106	-2.32	10051	NA	NA	NA	NA	-0.15	123	-10.65	13136
CSHL1_t2	1.02	0.33534665	-0.07	83	-2.32	10052	0	120	0.3	8263	-0.04	120	-2.79	10082
PHOSPHO1	1.03	0.00823393	-0.04	114	-2.33	10053	-0.02	135	-2.01	9830	0.05	108	2.76	7046
SELK	-1.03	0.364102044	-0.05	95	-2.33	10054	-0.02	106	-0.97	9124	-0.06	120	-4.29	10751
Mar-02	1.1	0.004261353	-0.04	102	-2.33	10055	-0.02	133	-1.84	9716	-0.01	153	-0.79	9083
SPESP1	1.13	0.012691367	-0.02	144	-2.33	10056	0.08	120	5.9	4845	0.09	122	6.63	5183
LARP4_t2	-1.03	0.496030822	-0.02	149	-2.34	10057	-0.02	158	-1.76	9663	0.05	147	4.97	5947
CD74	-1.52	2.99E-07	-0.04	106	-2.34	10058	-0.01	120	-0.37	8726	-0.28	107	-15.7	14519
ADAMTSL4	-1.05	0.261214436	-0.03	127	-2.34	10059	-0.12	126	-9.36	13641	0.03	104	1.54	7721
MRPL14	-1.01	0.892545574	-0.05	95	-2.34	10060	0.02	112	1.28	7623	-0.01	119	-0.54	8946
ITFG2	1	0.897554415	-0.03	120	-2.35	10061	0.01	121	0.39	8210	-0.01	115	-0.43	8868
MIOX	1.03	0.029490098	-0.05	100	-2.35	10062	-0.05	110	-2.73	10293	-0.01	116	-0.56	8956
OR7D2	1.02	0.123499931	-0.03	136	-2.35	10063	0.02	127	1.28	7624	0.03	122	2.01	7443
LRFN2	1.1	0.001405447	-0.04	111	-2.35	10064	-0.06	118	-3.95	11071	0.02	96	0.84	8114
SPHKAP	1.01	0.501671415	-0.02	152	-2.35	10065	-0.05	127	-3.47	10782	-0.04	125	-3.32	10318
HIST1H3G	-1.02	0.703699399	-0.06	88	-2.35	10066	-0.04	78	-1.16	9262	-0.13	68	-2.28	9837
ZFP2	1	0.905446282	-0.03	119	-2.35	10067	0.02	122	1.31	7597	0.05	116	3.23	6816
ADAM2	-1.02	0.060641694	-0.04	108	-2.36	10068	0.06	144	5.8	4892	-0.11	106	-6.32	11582
MSS51	1.02	0.051627109	-0.03	133	-2.36	10069	0.01	121	0.43	8187	0.04	115	2.31	7281
TXNDC17	-1.06	0.27220902	-0.03	136	-2.36	10070	-0.03	123	-2.07	9873	-0.01	118	-0.57	8963
GPR15	1.02	0.098093728	-0.07	84	-2.36	10071	-0.08	122	-5.48	11903	-0.08	91	-3.21	10274
ZIK1	1.07	0.022595947	-0.03	131	-2.36	10072	0.05	130	3.69	6066	-0.04	134	-2.94	10155
MUC16	1.02	0.113553716	-0.05	100	-2.37	10073	-0.05	73	-1.2	9290	0.01	86	0.37	8394
SLC25A23_t1	1.04	0.215520726	-0.03	118	-2.38	10074	NA	NA	NA	NA	-0.04	126	-2.69	10031
PRAP1	-1.41	0.006096257	-0.05	96	-2.38	10075	-0.06	114	-3.9	11036	-0.04	100	-1.93	9685

CLEC7A_t2	-1.15	2.93E-13	-0.1	73	-2.38	10076	-0.04	91	-1.61	9560	-0.29	92	-12.29	13639
SOX3	1	0.864178636	-0.03	128	-2.39	10077	-0.15	120	-10.74	14156	0.09	130	7.38	4882
SCUBE2	1.06	0.223799773	-0.03	120	-2.39	10078	0.09	129	7.15	4168	-0.09	123	-6.51	11653
UBTF_t1	-1.03	0.143849462	-0.03	136	-2.39	10079	NA	NA	NA	NA	NA	NA	NA	NA
LAMB3	1.26	0.028393932	-0.03	120	-2.39	10080	0.03	120	1.93	7192	0	123	0.25	8463
CLCN6_t1	1.02	0.02874883	-0.03	133	-2.39	10081	NA	NA	NA	NA	-0.03	158	-3.49	10388
KRT86	-1.03	0.579243452	-0.04	105	-2.39	10082	-0.15	126	-11.68	14422	-0.14	120	-9.8	12856
IFNE	1.01	0.433964332	-0.04	112	-2.39	10083	0.03	107	1.93	7187	-0.09	120	-6.02	11444
TMEM51	-1.48	0.000111459	-0.03	120	-2.39	10084	0.13	138	11.32	2349	-0.18	111	-11.02	13267
NKX2-5	-1.04	0.032398102	-0.05	95	-2.39	10085	-0.07	120	-4.87	11578	-0.15	140	-13.17	13892
MAPK11	1.02	0.759916786	-0.04	111	-2.39	10086	-0.11	153	-11.22	14306	-0.05	134	-4.22	10725
GABRG1	-1	0.919430088	-0.02	152	-2.4	10087	-0.03	127	-2.39	10072	-0.09	144	-8.37	12387
APRT	-1.08	0.05504851	-0.06	89	-2.4	10088	-0.09	130	-7.51	12878	-0.1	98	-4.58	10877
KIAA1191_t1	-1.07	0.091234229	-0.03	127	-2.4	10089	-0.02	155	-2.15	9931	NA	NA	NA	NA
PSME4	-1.04	0.335963292	-0.03	131	-2.4	10090	0.04	127	3.43	6228	0.03	121	2.08	7407
CYTH4	-1.05	0.139883039	-0.04	112	-2.4	10091	0.12	126	9.09	3233	-0.16	126	-12.16	13594
IGFBP2	-1.4	0.045470503	-0.04	115	-2.4	10092	-0.14	107	-7.76	13012	-0.08	109	-4.72	10945
AKAP1	1.14	0.004693478	-0.03	140	-2.4	10093	0	115	0.09	8406	0.09	151	9.59	4027
MKNK1	-1.06	0.050191139	-0.04	110	-2.4	10094	-0.05	130	-3.63	10896	-0.08	147	-8.04	12246
HPCAL1	-1.01	0.721435135	-0.04	112	-2.41	10095	0	137	-0.34	8708	-0.12	127	-9.44	12737
CYP2S1	1.02	0.315308937	-0.03	120	-2.41	10096	-0.02	139	-2.06	9866	-0.01	108	-0.49	8909
DNAJC1	-1.05	0.345831728	-0.06	93	-2.41	10097	-0.07	93	-3.1	10542	-0.02	128	-1.53	9461
SUV420H1_t2	-1.06	0.085515996	-0.03	126	-2.41	10098	-0.04	122	-2.58	10204	0.03	151	3.49	6670
LYL1	-1.13	3.60E-06	-0.08	80	-2.41	10099	-0.1	100	-4.83	11555	-0.21	97	-9.75	12830
G6PC3	-1.02	0.241380189	-0.03	123	-2.41	10100	-0.09	150	-9.04	13525	-0.17	100	-8.31	12366
PSMA4	1.04	0.456832485	-0.03	131	-2.41	10101	0.06	119	4.02	5893	0.06	112	3.46	6687
ZNF747	1.04	0.034227141	-0.04	114	-2.42	10102	-0.07	127	-5.69	12028	-0.02	123	-1.61	9506
MRPL23	-1.1	0.005263829	-0.08	80	-2.42	10103	-0.13	95	-5.86	12114	-0.08	84	-2.73	10050
COMTD1	-1.33	3.70E-06	-0.06	92	-2.42	10104	-0.19	92	-7.79	13024	-0.12	84	-3.94	10611
DPEP3	1.03	0.497545069	-0.08	79	-2.42	10105	-0.09	101	-4.51	11390	-0.12	97	-5.58	11265
NKX2-3	-1.02	0.011174779	-0.04	104	-2.42	10106	-0.08	125	-6.03	12201	-0.08	98	-4.04	10653
SPOCK2_t1	-1.07	0.001551309	-0.02	155	-2.43	10107	NA	NA	NA	NA	-0.1	123	-7.33	11969
AKT3_t2	-1.01	0.780010958	-0.06	89	-2.43	10108	0.02	129	1.78	7293	-0.13	113	-7.88	12189
USP13	-1.03	0.421387839	-0.03	131	-2.43	10109	-0.07	133	-5.74	12055	0.01	155	1.13	7950
IKBKG	1.16	0.000167493	-0.05	102	-2.43	10110	-0.02	115	-1.21	9300	-0.01	118	-0.91	9144
TMIE	1.15	3.11E-06	-0.03	129	-2.43	10111	0.08	115	5.02	5327	0.11	115	6.98	5044
DPCR1_t1	-1.06	0.046766038	-0.03	128	-2.43	10112	-0.01	123	-0.78	8996	NA	NA	NA	NA
LIPF	-1.01	0.153951414	-0.04	117	-2.43	10113	-0.01	92	-0.62	8885	0.03	98	1.47	7767
CPNE8_t1	-1.11	2.15E-05	-0.03	133	-2.43	10114	NA	NA	NA	NA	-0.02	132	-1.31	9349
MATN4	-1.04	0.15115485	-0.03	124	-2.44	10115	-0.24	101	-12.05	14518	-0.15	113	-9.28	12680
CLIC4_t1	-1.23	0.00017154	-0.03	132	-2.44	10116	NA	NA	NA	NA	0.06	150	6.32	5312
SYT15_t1	-1.06	0.002490171	-0.03	134	-2.44	10117	NA	NA	NA	NA	-0.13	94	-5.77	11346
KBTBD10	1.01	0.830157131	-0.04	113	-2.44	10118	0.07	128	5.54	5045	-0.05	125	-3.74	10508
S100A2	1.05	0.195315577	-0.04	106	-2.44	10119	0.07	112	4.12	5833	-0.07	134	-6.13	11491
Mar-10	1.05	2.62E-06	-0.04	111	-2.44	10120	0.01	120	0.76	7966	0.03	99	1.24	7880
CYP46A1	1	0.735307912	-0.05	100	-2.44	10121	-0.14	127	-10.55	14086	0	125	-0.31	8802
KLHL3	-1.01	0.243158053	-0.03	129	-2.44	10122	0	144	0.46	8160	-0.1	161	-11.61	13432
AIP_t1	-1.01	0.880179068	-0.04	107	-2.45	10123	NA	NA	NA	NA	-0.08	100	-3.87	10577
ZNF454	-1.01	0.688008517	-0.06	91	-2.45	10124	0.09	121	6.69	4406	-0.01	112	-0.7	9030
CNGA4	-1.03	0.050389165	-0.12	71	-2.45	10125	-0.05	87	-1.94	9767	-0.09	96	-3.94	10607
MED16	-1.02	0.631819896	-0.04	115	-2.45	10126	-0.12	84	-4.05	11123	-0.13	85	-4.53	10857
ARL6IP5	1.03	0.525019003	-0.04	120	-2.46	10127	0.1	133	8.33	3582	-0.01	148	-1.11	9257
MIER3	1.03	0.119210999	-0.02	155	-2.46	10128	-0.15	132	-12.39	14610	0.02	152	1.91	7507
NDFIP1	-1.05	0.270511946	-0.03	141	-2.46	10129	-0.12	143	-10.73	14146	0.01	136	1.08	7980
FCGBP	1.21	0.062698954	-0.06	91	-2.46	10130	0.08	98	3.86	5973	-0.09	131	-7.04	11859
PKHD1_t1	-1.05	0.001224577	-0.04	113	-2.46	10131	-0.02	116	-1.44	9455	-0.19	124	-14.38	14209
TTYH3	-1	0.99482476	-0.04	106	-2.46	10132	0.05	141	4.24	5757	-0.06	144	-5.82	11376
MRP63	-1.07	0.051982725	-0.02	150	-2.46	10133	-0.04	131	-3.25	10635	-0.12	128	-9.41	12723
SUMO1P1	1.03	0.21660232	-0.03	136	-2.47	10134	-0.02	139	-2.04	9854	0.05	148	5	5923
TRPC4AP	-1.06	0.000793174	-0.04	115	-2.47	10135	-0.13	126	-9.73	13786	-0.2	105	-11.12	13289
RABL2A_t1	1.05	0.103300185	-0.03	135	-2.47	10136	NA	NA	NA	NA	-0.06	124	-4.69	10939
U2AF1L4	-1.05	0.025564027	-0.08	81	-2.48	10137	-0.02	111	-1.41	9438	-0.22	111	-13.58	13991
DFNB31	1.04	0.038397882	-0.04	108	-2.48	10138	-0.06	127	-4.32	11274	0	121	-0.17	8712
B4GALNT4	-1	0.975528196	-0.06	93	-2.48	10139	-0.09	108	-5.34	11841	-0.19	98	-9.17	12648
MED13L	1.03	0.004491428	-0.03	142	-2.48	10140	0.17	135	14.23	1469	0.07	128	5.29	5782
MTM1	-1.03	0.47162276	-0.03	145	-2.49	10141	-0.09	124	-6.55	12439	0.12	134	9.72	3974
DNAJB12_t1	-1.04	0.270482811	-0.03	132	-2.49	10142	-0.1	116	-6.4	12370	-0.14	120	-10.1	12958
NOX5	-1.03	0.012237844	-0.04	108	-2.49	10143	-0.06	120	-4.13	11164	-0.07	137	-6.26	11557
MLF1	-1.11	0.054378023	-0.04	110	-2.49	10144	0.07	133	5.58	5018	-0.11	123	-7.92	12203
MAP6	1.01	0.402613103	-0.05	100	-2.49	10145	0.02	120	1.14	7713	-0.2	89	-7.8	12164
SMCP	1.02	0.086483203	-0.05	100	-2.5	10146	-0.16	109	-9.21	13595	-0.01	111	-0.69	9027
ACVR2B	1.08	0.039457829	-0.03	135	-2.5	10147	-0.06	137	-5.37	11858	0.12	132	9.82	3944
RANBP3_t2	-1	0.92787664	-0.03	132	-2.5	10148	0.12	118	7.9	3792	-0.1	122	-7.25	11943
PPP1R12B	-1.03	0.024704736	-0.02	153	-2.5	10149	-0.02	136	-1.64	9585	-0.02	131	-1.83	9632
SLC6A7	1.03	0.016169748	-0.04	120	-2.5	10150	-0.05	142	-4.4	11326	0	104	0.11	8549
GLIS3	1.03	0.004396107	-0.02	155	-2.51	10151	0	140	0.08	8411	0.06	142	5.88	5501
IFIH1	-1.2	0.001152942	-0.04	120	-2.51	10152	-0.08	112	-5.1	11722	-0.06	98	-2.93	10149
ADAM17_t1	-1.01	0.76502496	-0.03	123	-2.51	10153	NA	NA	NA	NA	-0.05	113	-3.02	10185
ZNF790	-1.02	0.671107313	-0.03	122	-2.51	10154	-0.03	87	-1.21	9295	-0.03	89	-1.28	9336
CUL5	-1.09	0.040348429	-0.03	138	-2.51	10155	0.04	144	3.98	5919	0.02	144	1.95	7482
LIMCH1	-1.09	0.022002225	-0.03	130	-2.51	10156	-0.07	163	-8.2	13188	-0.05	142	-4.65	10914
SFXN4	1.11	0.010068688	-0.03	133	-2.51	10157	0.01	117	0.64	8038	0.03	96	1.58	7700
APBB3	1.08	0.121777217	-0.03	130	-2.51	10158	-0.09	122	-6.53	12429	-0.01	107	-0.79	9079
TREML4	1.01	0.582995642	-0.03	127	-2.51	10159	0.16	118	10.66	2613	-0.1	120	-7.22	11926
SSTR1	1.05	0.0012494	-0.03	126	-2.51	10160	-0.1	131	-7.85	13052	0.06	122	4.04	6399
PLA2G6	-1.01	0.817396015	-0.06	91	-2.52	10161	-0.1	127	-7.56	12905	-0.07	120	-5.11	11086
AMELY	1.02	0.019294935	-0.06	95	-2.52	10162	0.01	112	0.37	8226	0.01	100	0.29	8445
CLMN	-1.1	0.107576976	-0.03	140	-2.52	10163	-0.13	139	-11.88	14470	-0.04	145	-3.58	10425
UNC79	-1.01	0.408785602	-0.04	111	-2.52	10164	0.03	146	2.91	6550	-0.07	107	-3.96	10616
ARC	-1.1	0.00203137	-0.05	99	-2.52	10165	-0.02	139	-2.15	9930	-0.12	140	-10.43	13065
PNMAL1_t1	-1.01	0.568121566	-0.03	141	-2.52	10166	NA	NA	NA	NA	0	124	0.15	8522
IL17F	-1.01	0.595285032	-0.03	133	-2.52	10167	-0.11	120	-7.97	13101	-0.14	105	-7.95	12216
KTN1	-1.16	0.051205309	-0.03	126	-2.53	10168	-0.04	131	-3.33	10691	-0.02	120	-1.53	9456
HSPA4L	-1.03	0.6402326	-0.04	119	-2.53	10169	0.03	118	2.17	7028	0.15	130	11.65	3311
B3GNT2	-1	0.958054381	-0.03	130	-2.53	10170	0.11	143	10.37	2732	0.05	122	3.89	6474
ERAS	1.05	0.000815387	-0.07	84	-2.53	10171	-0.03	81	-0.93	9101	0.05	68	0.87	8100
FRMD6	-1.21	0.0018891	-0.03	130	-2.53	10172	-0.01	147	-0.77	8989	-0.06	165	-7.43	12002
SCGB1D2	1.21	0.009360608	-0.05	103	-2.53	10173	-0.1	100	-4.97	11639	0.09	122	6.84	5092
FAM184A	1.06	0.396571028	-0.03	133	-2.53	10174	-0.03	112	-1.58	9541	0.11	127	8.23	4522
GALR3	-1.28	5.76E-06	-0.14	68	-2.54	10175	-0.12	100	-6.07	12225	-0.17	88	-6.57	11678
DDRGK1	1.04	0.315407418	-0.05	106	-2.54	10176	-0.17	93	-7.21	12741	-0.01	118	-0.41	8855
ZNF750	1.01	0.540708514	-0.03	136	-2.54	10177	-0.03	115	-2.03	9850	0.01	128	1.09	7976
TMEM38A	1.15	0.006354759	-0.05	100	-2.54	10178	0.04	135	3.34	6287	0.07	138	6.22	5348
PRDX5_t1	1.05	0.160097887	-0.03	124	-2.54	10179	NA	NA	NA	NA	-0.04	97	-1.94	9689
HIPK4	1.02	0.032293252	-0.05	101	-2.54	10180	-0.07	113	-4.23	11223	0.14	107	7.89	4665
ARFIP2	1.02	0.419494724	-0.04	120	-2.54	10181	0	131	-0.17	8598	-0.08	129	-6.52	11657

PNPLA6	-1.06	0.018234237	-0.03	125	-2.54	10182	-0.03	120	-2.42	10101	-0.12	148	-12.11	13579
TLN1	-1.01	0.711078647	-0.04	108	-2.54	10183	-0.04	130	-3.18	10588	-0.09	100	-4.39	10795
KRT77	1.01	0.57387915	-0.05	106	-2.54	10184	-0.03	126	-2.08	9882	0	142	0.28	8451
TNFRSF13B	-1.04	0.000395116	-0.07	84	-2.55	10185	0	111	-0.17	8601	-0.21	100	-10.7	13149
ELMOD1	1.06	0.151345589	-0.03	133	-2.55	10186	0.02	135	1.6	7432	0	125	-0.3	8796
ACHE	-1.05	0.023760101	-0.05	104	-2.55	10187	-0.06	111	-3.52	10812	-0.06	84	-1.91	9673
TNXB_t1	-1.33	2.25E-07	-0.07	87	-2.55	10188	NA	NA	NA	NA	NA	NA	NA	NA
CST2	1.04	0.21406308	-0.05	104	-2.55	10189	-0.13	126	-9.98	13875	0	102	0.11	8546
KRT25	1.01	0.467847166	-0.04	115	-2.55	10190	0.06	104	3.48	6191	0.08	113	5.03	5910
IL2RG	-1.07	0.032803462	-0.04	120	-2.55	10191	-0.01	124	-1.05	9179	-0.14	107	-7.89	12191
HIST1H2AH	1.06	0.212985006	-0.07	88	-2.55	10192	0.03	88	0.96	7830	0.05	79	1.47	7764
PIP5K1B	-1.02	0.603453175	-0.04	122	-2.55	10193	-0.03	102	-1.59	9551	-0.15	138	-13.61	14002
GPR35	1.07	0.00562028	-0.05	100	-2.55	10194	0.15	114	9.5	3059	-0.02	92	-0.87	9122
THBS3	1.25	2.73E-08	-0.06	92	-2.55	10195	0	102	-0.16	8589	0.07	100	3.46	6690
ADAD2	-1.03	0.301113229	-0.05	104	-2.56	10196	-0.16	83	-5.31	11826	-0.04	100	-2.08	9746
HOXA5	1.14	0.000823435	-0.02	158	-2.56	10197	-0.09	131	-7.29	12777	0	150	0.45	8340
PHC1_t1	-1.04	0.033072382	-0.03	139	-2.56	10198	NA	NA	NA	NA	-0.1	120	-7.32	11963
AGA	-1.21	0.000383595	-0.03	126	-2.56	10199	-0.12	117	-8.37	13271	-0.19	123	-13.84	14063
FOXC1	-1.3	0.002323858	-0.03	133	-2.56	10200	-0.01	123	-0.94	9105	-0.17	135	-14.22	14161
ZNF267	-1.11	0.016365574	-0.03	132	-2.56	10201	0.11	116	6.95	4273	-0.1	112	-5.92	11415
TIGD2	-1.36	0.000204581	-0.03	137	-2.57	10202	-0.21	124	-15.84	15375	0.04	103	1.88	7520
C4BPB	-1.29	0.015559206	-0.06	92	-2.57	10203	-0.16	88	-6.06	12216	0.04	80	1.17	7934
SYT12_t1	-1.03	0.025290263	-0.04	110	-2.57	10204	-0.11	108	-6.44	12392	NA	NA	NA	NA
UPK1A	1.08	1.52E-06	-0.04	111	-2.58	10205	-0.02	121	-1.11	9225	0.07	132	6.14	5380
GPR21	1.02	0.065635105	-0.13	70	-2.58	10206	0	68	-0.01	8487	-0.04	59	-0.36	8822
LCE1F	-1.09	1.84E-06	-0.06	97	-2.59	10207	-0.15	116	-9.61	13741	-0.15	100	-7.46	12017
PNMT	1.15	0.01094417	-0.05	107	-2.59	10208	-0.06	84	-1.99	9817	0	114	-0.31	8801
RFC3_t2	-1.02	0.227346145	-0.04	114	-2.59	10209	-0.05	124	-3.52	10813	-0.09	115	-5.98	11432
NBL1	-1.08	0.001589377	-0.03	127	-2.59	10210	-0.16	120	-11.32	14326	-0.05	120	-3.62	10449
LRWD1	-1.03	0.423923111	-0.05	100	-2.59	10211	0.01	120	0.57	8094	-0.02	91	-0.62	8992
SIRPG	-1.03	0.182125437	-0.04	109	-2.59	10212	0.03	132	2.07	7100	-0.21	127	-16.13	14613
DNAJC4	-1.1	0.000747101	-0.05	100	-2.59	10213	-0.19	104	-10.1	13917	-0.14	88	-5.25	11137
ZNF683	-1.21	0.000841421	-0.11	74	-2.59	10214	-0.11	76	-2.93	10432	-0.24	63	-3.1	10222
JPH2_t2	-1.04	0.119374754	-0.03	143	-2.59	10215	-0.02	126	-1.82	9699	-0.11	133	-8.99	12587
DLEC1	-1.06	0.003500198	-0.05	104	-2.59	10216	0.08	106	4.23	5760	-0.18	120	-12.25	13628
SSH3_t1	-1.03	0.482247158	-0.04	109	-2.6	10217	0.09	141	7.92	3786	NA	NA	NA	NA
B9D2_t1	-1.1	0.003770492	-0.07	87	-2.6	10218	NA	NA	NA	NA	-0.14	106	-7.76	12143
MMP17	-1.21	0.000142048	-0.06	91	-2.6	10219	-0.02	121	-1.46	9470	-0.1	100	-5.06	11072
GNB3	1.03	0.045405375	-0.04	120	-2.6	10220	-0.05	132	-4.46	11364	0.03	108	1.5	7748
SH2B2	-1.05	0.007862907	-0.1	76	-2.6	10221	-0.07	115	-4.68	11479	-0.11	94	-4.93	11026
FXYD5_t1	-1.22	4.83E-06	-0.05	103	-2.6	10222	NA	NA	NA	NA	-0.21	104	-11.28	13340
HNRNPM	1.01	0.669187321	-0.03	126	-2.6	10223	0.09	85	3.27	6339	-0.01	142	-1.3	9345
EIF2AK3	-1.19	9.12E-05	-0.04	124	-2.6	10224	-0.02	111	-1.4	9429	-0.12	128	-9.23	12664
SNX2	-1.09	0.135334042	-0.03	132	-2.6	10225	-0.06	138	-4.99	11652	0.05	155	5.05	5900
KIAA0319L	-1.06	0.012770886	-0.04	120	-2.6	10226	-0.13	136	-11.02	14248	-0.1	129	-8	12239
SLC9C1	-1.02	0.019319704	-0.04	116	-2.61	10227	-0.01	129	-0.95	9108	-0.05	131	-4.32	10763
SIK2	-1.33	1.67E-07	-0.03	127	-2.61	10228	-0.18	125	-13.18	14792	-0.09	131	-6.95	11823
ZDHHC19	1.02	0.096201793	-0.07	88	-2.61	10229	-0.08	126	-5.84	12107	0.01	131	0.51	8310
NTRK2_t1	1.06	0.000289821	-0.03	125	-2.61	10230	-0.11	141	-10.04	13896	0.05	124	3.42	6709
PIK3IP1_t1	-1.02	0.532315959	-0.04	116	-2.61	10231	NA	NA	NA	NA	-0.1	148	-9.38	12709
CAV3_t2	1	0.897446754	-0.04	109	-2.61	10232	-0.04	120	-2.95	10446	0.02	137	1.82	7558
SDC1	-1.1	0.155511918	-0.03	143	-2.61	10233	-0.15	130	-11.73	14433	-0.01	126	-0.76	9058
SPANXN3	-1.01	0.51382837	-0.1	75	-2.61	10234	-0.02	90	-0.97	9125	-0.03	95	-1.25	9318
TIAL1	-1.07	0.022772966	-0.02	157	-2.62	10235	-0.05	161	-5.34	11840	-0.01	124	-0.46	8886
ZDHHC15	1.01	0.710104769	-0.03	143	-2.62	10236	0.01	143	1	7810	0	153	-0.49	8905
N4BP2	-1.08	0.120596698	-0.03	141	-2.62	10237	-0.03	130	-2.33	10042	0.01	138	0.51	8311
GRK4	1.01	0.62014926	-0.08	84	-2.62	10238	-0.01	98	-0.48	8802	-0.02	107	-0.96	9174
NPHP3	-1.09	0.040134343	-0.03	128	-2.62	10239	0.07	143	6.31	4612	-0.08	123	-5.89	11399
RUNDC3A	1.02	0.148606226	-0.04	120	-2.62	10240	-0.05	116	-3.57	10850	-0.14	122	-9.93	12907
IL24	1.03	0.015524384	-0.04	120	-2.62	10241	0.02	134	1.7	7350	0.03	141	2.6	7130
YSK4	1	0.917523463	-0.05	107	-2.63	10242	0.04	119	2.66	6705	0.03	122	2.48	7188
JOSD1_t1	-1.06	0.003970299	-0.03	131	-2.63	10243	NA	NA	NA	NA	-0.05	128	-4.25	10734
RAD23A	1.05	0.091511041	-0.04	114	-2.63	10244	-0.06	112	-3.66	10917	0.04	91	1.56	7712
KREMEN1	-1	0.977388133	-0.04	122	-2.63	10245	-0.05	140	-4.65	11464	-0.14	153	-14.74	14307
OLR1	1.03	0.329339641	-0.03	136	-2.63	10246	0.02	155	2.3	6957	-0.11	139	-9.79	12848
ZNF599	1.03	0.010428672	-0.03	126	-2.64	10247	0.02	111	0.93	7852	0.01	122	0.88	8088
IFNA2	1.02	0.033887274	-0.04	120	-2.64	10248	0.02	140	1.97	7161	0.02	131	1.83	7546
INS	-1.02	0.195466046	-0.05	100	-2.64	10249	-0.06	93	-2.64	10242	-0.17	100	-8.61	12463
CRYBB3	-1.14	2.89E-06	-0.07	90	-2.64	10250	-0.17	105	-9.2	13590	-0.18	80	-5.52	11246
NUS1	-1.01	0.899568936	-0.03	149	-2.64	10251	0.09	152	9.36	3117	-0.03	149	-3.35	10335
TPSD1	-1.02	0.152899377	-0.03	132	-2.65	10252	0.11	95	4.76	5468	-0.06	114	-3.89	10586
NDUFA10	-1.13	0.000521877	-0.03	139	-2.65	10253	-0.11	148	-10.53	14075	-0.13	141	-12	13542
UCHL3	-1.06	0.221069261	-0.04	126	-2.66	10254	0.05	102	2.61	6750	-0.12	115	-7.92	12205
IRF7_t1	-1.12	7.77E-09	-0.12	72	-2.66	10255	-0.26	100	-13	14753	-0.25	68	-4.59	10888
PHF23_t1	-1.13	0.000327855	-0.04	125	-2.66	10256	-0.06	131	-5.01	11661	NA	NA	NA	NA
MECR	1.04	0.147002438	-0.03	128	-2.66	10257	-0.07	115	-4.37	11299	0.03	120	2.31	7283
SYT17	-1.32	0.000530388	-0.08	84	-2.66	10258	-0.06	134	-5.18	11767	-0.06	94	-2.48	9944
DIAPH2_t1	-1.25	0.001444918	-0.04	113	-2.67	10259	-0.1	144	-9.27	13615	0.03	129	2.11	7394
TMCO6	-1.05	0.337044011	-0.04	112	-2.67	10260	-0.14	107	-8.2	13187	-0.04	113	-2.47	9935
A4GNT	-1.04	0.080028045	-0.03	136	-2.67	10261	-0.16	126	-11.98	14494	-0.08	165	-9.37	12705
AANAT	-1.06	0.002148684	-0.07	89	-2.67	10262	-0.22	84	-7.61	12932	-0.08	88	-3.18	10254
MAP3K5	-1.4	0.000309336	-0.03	131	-2.67	10263	-0.13	132	-10.57	14099	-0.05	147	-4.88	11006
SIAH2	-1.2	0.006192025	-0.04	126	-2.67	10264	-0.22	116	-14.6	15121	-0.05	132	-3.94	10609
RSPO1	-1.04	0.019470717	-0.04	121	-2.68	10265	-0.17	146	-16.73	15552	-0.2	123	-14.26	14175
FUT7	-1.05	0.005332324	-0.07	88	-2.68	10266	-0.17	108	-9.98	13877	-0.13	100	-6.33	11586
SPRY1	-1.1	0.086635798	-0.04	120	-2.68	10267	0.1	107	5.53	5048	-0.13	111	-8.17	12298
PDE5A_t2	1.03	0.195778543	-0.03	134	-2.68	10268	0.11	124	8.05	3714	0	145	0.03	8605
FAM116B	1.01	0.557883669	-0.05	106	-2.68	10269	-0.1	133	-8.28	13232	-0.04	100	-1.89	9663
SLC16A10	-1.09	0.140593969	-0.04	117	-2.69	10270	-0.15	127	-11.93	14487	0.01	145	0.54	8296
TCEAL1	1.04	0.461639767	-0.04	125	-2.69	10271	-0.06	120	-4.18	11195	0.01	131	0.59	8261
SLC22A18AS	-1.02	0.64820564	-0.05	104	-2.69	10272	0	103	0.21	8329	-0.01	131	-0.89	9136
ZNRF4	1.01	0.385704584	-0.07	88	-2.69	10273	0	79	-0.07	8537	0.01	88	0.31	8431
IFI44	-1.64	7.48E-10	-0.03	130	-2.69	10274	-0.11	103	-5.57	11955	-0.2	113	-12.82	13796
LRRC8B	-1.13	0.038068621	-0.03	152	-2.69	10275	0.01	150	1.05	7771	-0.11	142	-10.56	13104
CCNC	-1.08	0.091731495	-0.04	120	-2.69	10276	-0.06	124	-4.19	11198	0	120	-0.31	8799
ADAM23	1.11	0.015268147	-0.04	118	-2.69	10277	0.05	116	2.98	6508	0.08	143	7.44	4857
AP2M1	-1.1	0.002722063	-0.06	96	-2.7	10278	-0.11	120	-8.03	13126	-0.12	128	-9.21	12658
BEND4	1.01	0.152249595	-0.03	151	-2.7	10279	-0.01	140	-0.5	8815	0.03	143	2.49	7182
CLEC3A	1.01	0.258400069	-0.04	127	-2.7	10280	-0.04	120	-2.97	10461	0.02	136	1.48	7760
BTNL3_t1	1.13	0.064287151	-0.04	127	-2.7	10281	NA	NA	NA	NA	0.02	112	1.26	7871
FRS3	1.03	0.094346121	-0.05	109	-2.71	10282	-0.11	152	-10.76	14165	-0.05	118	-3.71	10488
GRP	1.07	0.006658929	-0.03	130	-2.71	10283	0.03	96	1.24	7654	0.05	95	2.16	7367
CHP2	1.24	0.002077063	-0.04	114	-2.71	10284	-0.04	130	-3.09	10531	-0.02	120	-1.19	9284
UBP1	1.03	0.474609975	-0.03	135	-2.71	10285	0.04	122	3.08	6448	-0.04	120	-2.58	9992
C1QTNF4	-1.02	0.323776937	-0.1	76	-2.71	10286	-0.09	87	-3.21	10608	-0.02	88	-0.68	9022
FBXO24	1.04	0.159398729	-0.05	110	-2.72	10287	-0.11	110	-6.53	12426	0.03	102	1.32	7841

CHIA	-1.05	0.394011538	-0.06	96	-2.72	10288	-0.23	122	-16.73	15555	-0.14	100	-6.81	11774
FRMPD1	1.03	0.044221959	-0.05	103	-2.72	10289	-0.06	130	-4.89	11590	-0.01	114	-0.93	9149
TNC	-1.24	0.000622006	-0.03	129	-2.73	10290	-0.09	135	-7.52	12882	-0.19	129	-14.75	14311
GRIK2_t2	-1.04	0.005355151	-0.03	130	-2.73	10291	-0.14	132	-11.81	14448	-0.01	120	-0.42	8863
MMP13	-1	0.999466407	-0.03	144	-2.73	10292	-0.05	123	-3.78	10968	-0.02	160	-1.66	9528
IL31	-1	0.735864143	-0.04	124	-2.73	10293	-0.03	125	-2.42	10100	0	130	-0.35	8818
PGD	1.02	0.757132157	-0.04	111	-2.73	10294	-0.02	130	-1.77	9666	-0.06	118	-3.99	10637
OXCT1	1.16	0.056998084	-0.03	133	-2.73	10295	0.15	136	12.57	1965	-0.06	137	-5.04	11063
NOTCH4_t1	-1.03	0.084495488	-0.05	104	-2.74	10296	NA	NA	NA	NA	NA	NA	NA	NA
DNAJB3	1.13	0.010943509	-0.04	124	-2.74	10297	-0.02	125	-1.21	9298	-0.03	121	-1.99	9703
VPS37D	1.09	0.01121734	-0.04	120	-2.75	10298	0.05	120	3.84	5986	0.02	121	1.5	7751
PDIA4	-1.05	0.294007035	-0.04	127	-2.75	10299	-0.09	115	-5.71	12036	-0.04	127	-4.23	10729
HTRA1	-1.23	0.026630652	-0.04	113	-2.75	10300	-0.17	120	-11.74	14434	-0.05	122	-3.85	10562
FZD10	1.05	0.195713394	-0.03	129	-2.76	10301	0.13	126	9.69	2976	-0.07	118	-4.77	10974
ZBTB46	-1.04	0.000360429	-0.03	134	-2.76	10302	0.02	142	1.7	7352	-0.04	141	-4.07	10670
DUSP10	-1.49	4.41E-09	-0.05	108	-2.76	10303	-0.16	123	-11.8	14446	-0.12	111	-7.6	12074
KCNK15	-1.14	1.05E-06	-0.05	110	-2.76	10304	-0.09	122	-6.83	12576	-0.18	124	-13.41	13955
CHD3	-1.02	0.200124715	-0.06	100	-2.76	10305	0.03	132	2.32	6943	-0.13	114	-8.11	12271
KIF13B_t1	-1.03	0.094866871	-0.04	120	-2.76	10306	NA	NA	NA	NA	-0.08	126	-6.18	11512
HOXB4	-1.1	0.053846174	-0.03	147	-2.77	10307	0.01	136	1.12	7738	-0.18	121	-12.43	13676
PLN_t1	-1	0.888971205	-0.04	119	-2.77	10308	NA	NA	NA	NA	-0.03	140	-2.53	9968
MPP5	-1	0.938355585	-0.03	137	-2.77	10309	-0.04	132	-3.42	10740	0.08	143	7.75	4734
MYBPC2	1.05	0.180764447	-0.06	96	-2.78	10310	-0.14	95	-6.16	12268	-0.15	106	-8.23	12327
SCN1B	-1.04	0.001915459	-0.05	104	-2.79	10311	-0.16	123	-11.59	14397	-0.04	108	-2.6	9999
UMOD	1.04	0.019105282	-0.04	120	-2.79	10312	-0.1	93	-4.25	11243	-0.03	102	-1.53	9462
MYOD1	-1.19	3.47E-05	-0.04	119	-2.79	10313	-0.13	129	-10.57	14092	-0.08	121	-5.76	11338
MED15	-1.08	0.035055276	-0.04	124	-2.79	10314	0.08	137	6.89	4307	-0.21	137	-18.49	15078
FAM211A	-1.07	0.22195673	-0.05	107	-2.79	10315	0.06	134	5.34	5148	-0.1	111	-5.82	11375
HOXB2	-1.08	0.014968571	-0.04	113	-2.79	10316	0	131	-0.29	8665	-0.21	110	-12.47	13691
CELSR1	-1.02	0.650048228	-0.03	140	-2.8	10317	0	148	-0.17	8597	0.04	150	3.63	6600
BHLHE41	1.08	0.408833633	-0.04	123	-2.8	10318	0.17	141	15.53	1136	-0.12	160	-13.1	13871
C4BPA	-1.96	5.21E-05	-0.06	95	-2.8	10319	-0.13	99	-6.47	12401	-0.09	111	-5.72	11327
PIGO	1.19	4.23E-06	-0.03	133	-2.8	10320	-0.14	115	-9.38	13651	0.11	130	9.13	4178
DTWD2	-1.01	0.672265386	-0.03	144	-2.8	10321	-0.08	132	-6.4	12371	0.02	145	1.73	7612
SYNGR4	-1.28	5.30E-06	-0.15	69	-2.8	10322	-0.12	92	-5.15	11744	-0.14	52	-0.28	8780
HAND1	1.06	1.38E-06	-0.05	104	-2.81	10323	-0.03	120	-2.39	10081	0.04	139	3.57	6632
EXOC3L1	-1.14	7.08E-08	-0.17	67	-2.81	10324	-0.14	61	-1.54	9514	-0.26	60	-2.55	9978
CCL25	1.27	1.73E-05	-0.05	102	-2.81	10325	-0.04	105	-2.31	10032	0.13	103	7.04	5017
FAM177A1	-1.03	0.492371909	-0.03	155	-2.81	10326	-0.05	135	-4.36	11291	-0.02	134	-1.75	9586
KLK14	-1.03	0.076100428	-0.08	87	-2.82	10327	-0.11	92	-4.6	11442	-0.07	73	-1.57	9480
PLEKHB2	-1.16	0.000214868	-0.03	133	-2.82	10328	0.07	151	7.5	3983	-0.09	165	-10.92	13227
TMEM184C	1.03	0.312723595	-0.04	124	-2.82	10329	-0.03	128	-1.97	9793	0.08	127	6.12	5393
ACP1_t2	-1.06	0.127194785	-0.04	128	-2.82	10330	-0.03	133	-2.8	10340	-0.08	105	-4.24	10730
CD320	1.05	0.042613661	-0.06	100	-2.82	10331	-0.03	142	-2.41	10091	-0.03	100	-1.57	9479
FAM105A	-1.19	0.002313447	-0.03	135	-2.83	10332	0.04	155	4.18	5791	-0.15	131	-12.25	13624
CD3E_t1	-1.04	0.014443541	-0.04	119	-2.83	10333	NA	NA	NA	NA	NA	NA	NA	NA
YIPF5_t2	-1.03	0.470743674	-0.03	134	-2.83	10334	0.03	139	2.63	6734	0.07	147	6.36	5289
JSRP1	-1.11	0.00017796	-0.11	76	-2.83	10335	-0.04	99	-1.79	9687	-0.3	68	-5.41	11204
TOMM22	1.03	0.332808134	-0.04	119	-2.83	10336	0.02	112	0.99	7815	-0.03	119	-1.84	9633
AQP2	1.07	1.33E-06	-0.03	140	-2.83	10337	-0.05	136	-4.02	11112	0.02	134	1.82	7556
TCIRG1	-1.11	0.007323508	-0.1	78	-2.83	10338	0.02	92	0.87	7887	-0.27	80	-7.96	12221
KRT28	1.03	0.019102482	-0.05	110	-2.83	10339	0.14	116	9.06	3248	-0.07	140	-6.15	11496
PEX7	-1.01	0.835283739	-0.05	104	-2.83	10340	-0.08	111	-4.96	11636	-0.02	113	-1.42	9402
SUN5	-1.02	0.06838417	-0.11	76	-2.83	10341	-0.1	72	-2.12	9914	-0.11	66	-1.78	9603
CTSK	-1.1	0.365309263	-0.04	117	-2.83	10342	0.08	125	6.13	4713	-0.12	146	-11.68	13447
GLIPR1L1	-1.02	0.027086211	-0.05	105	-2.84	10343	-0.02	130	-1.62	9570.5	-0.17	104	-8.97	12582
EWSR1_t1	-1.08	0.007507779	-0.04	117	-2.84	10344	NA	NA	NA	NA	-0.15	104	-8.36	12383
MON2_t1	-1.05	0.06216623	-0.04	129	-2.84	10345	-0.03	144	-3.23	10616	NA	NA	NA	NA
BCAM_t1	-1.09	0.061081549	-0.06	99	-2.84	10346	NA	NA	NA	NA	-0.1	90	-4.02	10648
TMEM171	1.03	0.345831728	-0.05	104	-2.84	10347	-0.05	84	-1.59	9552	-0.05	101	-2.55	9979
EHBP1	-1.01	0.64516408	-0.03	133	-2.84	10348	-0.13	124	-9.39	13655	-0.01	143	-0.9	9140
HMP19	1.09	1.81E-09	-0.03	137	-2.85	10350	0.08	128	6.07	4745	0.07	138	6.03	5438
FAM5B	1.01	0.753996029	-0.04	114	-2.85	10351	-0.05	114	-3.51	10808	-0.13	110	-7.83	12176
CCDC108_t2	-1.41	3.65E-09	-0.16	68	-2.85	10352	-0.2	93	-8.48	13320	-0.13	88	-5.11	11085
ZFYVE19	1.02	0.638302968	-0.05	109	-2.86	10353	-0.08	94	-3.44	10761	0.06	113	3.93	6452
KCNJ2	-1.05	0.288331167	-0.03	145	-2.86	10354	0.11	149	10.8	2552	-0.07	137	-5.85	11385
NCOA1	-1.03	0.366627123	-0.03	135	-2.86	10355	0.03	132	2.55	6799	0	164	0.4	8373
NPSR1	-1.02	0.111141621	-0.07	94	-2.86	10356	-0.09	105	-5.19	11772	-0.05	92	-2.31	9842
IKZF3	-1.01	0.427175827	-0.03	143	-2.86	10357	0.01	149	0.68	8019	-0.16	153	-16.32	14651
TIMM44	-1.05	0.15583224	-0.07	93	-2.86	10358	-0.08	146	-7.25	12756	-0.08	116	-5.44	11216
NEK1	-1.04	0.029249078	-0.03	146	-2.86	10359	0.01	119	0.58	8085	0.02	126	1.31	7846
ARL11_t1	-1.04	0.04076576	-0.03	155	-2.86	10360	NA	NA	NA	NA	NA	NA	NA	NA
ANKRD11	-1.05	0.245495545	-0.04	120	-2.87	10361	0.04	127	3.42	6233	-0.03	120	-2.27	9828
SOAT2	1.12	0.263646195	-0.04	118	-2.87	10362	0.03	127	2.36	6911	0.02	129	1.81	7563
RRAS	-1.07	0.157254188	-0.04	116	-2.87	10363	0.02	109	1.13	7726	-0.1	98	-4.73	10954
NSMCE4A_t1	-1.04	0.277767671	-0.04	115	-2.87	10364	NA	NA	NA	NA	0.02	100	1.11	7959
PAPOLB	1.02	0.027418075	-0.03	143	-2.87	10365	-0.05	113	-3.44	10759	0.02	135	1.96	7476
HDAC5	1.1	0.006246948	-0.05	112	-2.87	10366	0.05	152	5.58	5020	0.01	126	1.12	7957
CASP8_t2	1.06	0.066076356	-0.03	144	-2.87	10367	0.2	119	14.1	1506	0	154	-0.43	8867
CRYBA1	1	0.610388386	-0.07	94	-2.88	10368	-0.01	117	-0.85	9046	0.05	108	2.98	6937
TSSK2	1.02	0.021937854	-0.07	93	-2.88	10369	-0.05	85	-1.84	9722	0.07	88	2.64	7115
RP1L1	-1.03	0.032831221	-0.03	150	-2.88	10370	-0.15	113	-9.71	13778	-0.08	116	-5.17	11110
RUNX2_t2	1.01	0.488317385	-0.03	138	-2.88	10371	NA	NA	NA	NA	0.01	154	0.76	8160
ZNF561_t1	-1.09	0.002313802	-0.04	131	-2.88	10372	NA	NA	NA	NA	NA	NA	NA	NA
KRT1	-1.08	0.082232159	-0.05	109	-2.88	10373	-0.03	120	-1.87	9737	-0.01	132	-1.08	9235
FSCN2	-1.02	0.197222737	-0.13	72	-2.89	10374	-0.15	88	-5.82	12098	-0.06	84	-2.18	9784
PXT1	1.02	0.106185023	-0.04	117	-2.89	10375	-0.07	124	-5.45	11900	0.03	123	2.26	7317
CBLC	-1.1	0.034117418	-0.12	74	-2.89	10376	-0.23	88	-8.57	13351	-0.06	88	-2.32	9849
ZCWPW1	-1.19	0.005420093	-0.1	78	-2.9	10377	-0.13	101	-6.7	12513	-0.09	88	-3.29	10302
PLEKHH2_t1	1.02	0.125433308	-0.03	139	-2.9	10378	NA	NA	NA	NA	0.03	139	2.99	6930
FUT9	1.01	0.409325973	-0.03	155	-2.9	10379	-0.01	133	-1.16	9265	-0.02	155	-1.73	9571
MORN3	1	0.615896618	-0.04	132	-2.9	10380	0.07	110	4.07	5864	-0.01	100	-0.53	8937
BPIFB4	1.02	0.205181344	-0.08	88	-2.91	10381	-0.04	142	-3.84	11002	-0.03	99	-1.62	9509
GAL3ST1	1.03	0.801674621	-0.05	111	-2.91	10382	0.04	104	2.39	6894	-0.15	115	-10.07	12952
KRTAP3-2	1.03	0.001422794	-0.05	114	-2.91	10383	-0.01	98	-0.51	8824	0.01	142	0.84	8117
MAST1	1.06	0.23340347	-0.06	99	-2.91	10384	-0.1	97	-4.71	11494	0.08	91	3.08	6882
WFDC10A	1	0.905387457	-0.08	88	-2.91	10385	-0.06	104	-3.07	10517	-0.01	84	-0.21	8736
ZNF526	1.03	0.18750065	-0.03	148	-2.91	10386	-0.06	139	-5.05	11683	-0.08	142	-7.73	12131
TRMT10A	-1.01	0.824067535	-0.03	151	-2.92	10387	-0.05	116	-3.44	10760	0.08	128	6.11	5397
CCDC124	1.11	8.66E-05	-0.05	104	-2.92	10388	0.05	117	3.02	6482	-0.03	100	-1.7	9554
GLRA3	-1	0.680624932	-0.04	130	-2.92	10389	-0.05	119	-3.76	10956	-0.12	128	-9.12	12633
SNURF	-1.21	0.005901389	-0.07	92	-2.92	10390	0.03	85	1.01	7804	-0.15	87	-5.69	11313
EPN3	1.06	0.150956602	-0.04	124	-2.92	10391	0.03	126	1.95	7176	-0.01	125	-1.08	9237
CEP57L1	1.05	0.185502423	-0.04	125	-2.92	10392	0.01	119	0.6	8075	0.06	144	5.3	5777
SP100	-1.13	0.025734939	-0.03	159	-2.93	10393	0.09	130	6.94	4279	-0.08	126	-5.75	11336
KRTAP4-11	1.01	0.71329868	-0.05	114	-2.93	10394	-0.1	126	-7.35	12812	-0.06	119	-3.86	10574

FBP2	1.02	0.260711063	-0.06	96	-2.93	10395	0.08	97	3.53	6160	-0.06	135	-4.88	11011
TMTC3	-1.01	0.632808172	-0.03	139	-2.93	10396	0.05	125	3.48	6198	0.05	146	5.07	5894
TRIM5_t1	-1.02	0.371998179	-0.03	136	-2.93	10397	-0.09	137	-8	13116	-0.01	149	-1.2	9299
THOC6	-1.02	0.314895017	-0.08	85	-2.93	10398	-0.04	111	-2.2	9967	-0.18	95	-8.1	12267
PTPRU	1.03	0.101640644	-0.05	109	-2.94	10399	-0.09	145	-8.42	13297	0.01	110	0.65	8223
CFDP1	-1.14	0.001036801	-0.05	112	-2.94	10400	-0.09	95	-3.97	11085	-0.09	116	-5.7	11320
DUSP26	-1.02	0.772095167	-0.04	130	-2.94	10401	0.15	123	11.17	2409	-0.15	128	-11.44	13382
HIPK3	-1.02	0.105447039	-0.03	135	-2.94	10402	0	103	0.17	8351	-0.05	134	-3.85	10557
STK33	1	0.937606127	-0.04	116	-2.94	10403	0.05	128	3.59	6119	-0.16	148	-15.23	14427
USP5	-1	0.88972116	-0.06	103	-2.95	10404	-0.09	100	-4.67	11476	-0.07	110	-4.33	10768
CCNY_t2	1.06	0.011421661	-0.03	143	-2.95	10405	0.06	142	5.38	5135	0.12	141	10.53	3679
KLRC3	-1.21	5.11E-07	-0.06	98	-2.95	10406	-0.04	63	-0.53	8833	-0.2	109	-11.78	13483
SIGLEC12	-1.04	0.070951429	-0.04	117	-2.95	10407	0.01	151	1.35	7577	-0.05	89	-2.1	9759
CAMKK2_t1	1.01	0.887922515	-0.04	123	-2.95	10408	-0.07	147	-7.24	12755	-0.05	113	-3.12	10233
TMCO5A	1.02	0.142756824	-0.04	131	-2.96	10409	-0.04	89	-1.7	9618	0	86	0.17	8507
STX8	-1.18	6.00E-07	-0.16	69	-2.96	10410	-0.21	99	-10.11	13924	-0.2	88	-7.67	12097
ETF1	-1.11	0.013102738	-0.03	137	-2.96	10411	-0.04	130	-3.09	10533	0.01	136	1.04	7996
NCKIPSD	-1.01	0.579363579	-0.07	95	-2.96	10412	-0.09	146	-8.23	13205	-0.07	155	-7.19	11915
PCDH11Y_t2	1.05	3.42E-06	-0.04	132	-2.96	10413	0.04	132	3.15	6408	0.08	139	7.09	4997
MYO1G_t1	-1.14	2.73E-06	-0.16	68	-2.96	10414	NA	NA	NA	NA	NA	NA	NA	NA
EIF4A1_t1	-1.29	5.58E-08	-0.05	108	-2.96	10415	NA	NA	NA	NA	NA	NA	NA	NA
WBP4	-1.02	0.635924115	-0.03	147	-2.96	10416	0.08	122	5.44	5093	-0.05	139	-4.42	10811
ARHGAP28	1.04	0.205548291	-0.04	122	-2.97	10417	-0.01	123	-0.7	8937	0.1	132	8.57	4396
GIPC3	-1.03	0.019339966	-0.05	111	-2.97	10418	-0.15	133	-12.49	14633	-0.09	132	-7.03	11858
CDC40	-1.11	0.00057761	-0.03	136	-2.98	10419	0.01	145	1.29	7615	-0.01	129	-0.88	9128
PALMD	-1.05	0.399302512	-0.03	157	-2.98	10420	-0.05	104	-2.47	10131	0.1	124	7.75	4733
RBM11	-1.03	0.146861901	-0.04	120	-2.98	10421	0	125	0.07	8417	-0.13	124	-9.68	12803
CAPZB	-1.07	0.102960863	-0.03	144	-2.99	10422	0.02	131	1.62	7414	-0.09	120	-6.31	11574
IKZF2_t1	1.01	0.616301622	-0.03	141	-2.99	10423	NA	NA	NA	NA	-0.02	134	-2.02	9716
SMYD4	-1.03	0.455511866	-0.04	131	-2.99	10424	0.01	132	0.92	7855	-0.07	120	-4.6	10893
HIRA	-1.26	0.000461546	-0.04	124	-2.99	10425	0.01	140	0.88	7883	-0.12	131	-9.83	12866
EMD	-1.01	0.858944181	-0.04	118	-2.99	10426	-0.11	118	-7.15	12719	-0.02	109	-0.94	9162
FAM154A	1.01	0.308811997	-0.04	124	-2.99	10427	0.01	104	0.63	8044	0.01	108	0.7	8192
DUX4	-1.37	1.35E-07	-0.21	64	-2.99	10428	-0.27	64	-3.73	10950	-0.21	61	-2.31	9846
KRTAP4-4	1	0.612173318	-0.04	123	-2.99	10429	-0.05	114	-3.1	10536	0.01	131	0.63	8241
FXYD4	-1.08	7.48E-05	-0.07	90	-2.99	10430	-0.22	102	-11.55	14388	-0.08	99	-3.86	10569
DNAJC27	-1.01	0.588641565	-0.03	136	-3	10431	0.02	130	1.23	7664	0.01	143	0.7	8194
CPNE6	1.02	0.248711176	-0.06	103	-3	10432	-0.04	140	-3.19	10595	0	88	0.08	8563
IFNA10	1	0.949947414	-0.06	104	-3	10433	-0.11	133	-9.07	13547	-0.02	106	-1.17	9279
ODF3L2	-1.06	0.00618261	-0.06	98	-3.01	10434	-0.11	123	-7.74	13004	-0.13	102	-6.63	11708
APEX1	1.13	1.03E-07	-0.04	129	-3.01	10435	0.02	129	1.35	7571	0	104	0.25	8464
TAS2R31	-1.1	3.87E-07	-0.12	76	-3.01	10436	-0.19	74	-4.51	11394	-0.09	84	-2.9	10134
QTRT1	1.02	0.529435451	-0.07	95	-3.02	10437	-0.09	86	-3.08	10519	-0.08	109	-4.46	10822
TMEM161A	1.07	0.04366759	-0.06	97	-3.02	10438	-0.04	108	-2.18	9954	0.02	110	0.92	8064
GSR	-1.02	0.688352571	-0.03	160	-3.02	10439	-0.06	127	-4.68	11478	-0.02	128	-1.45	9417
KCNK9	-1.03	0.094044512	-0.07	94	-3.02	10440	-0.12	116	-7.99	13111	-0.03	100	-1.71	9556
FAM198B	-1.15	0.06199302	-0.03	142	-3.02	10441	0.03	130	2.16	7037	-0.05	128	-3.79	10534
CMAHP	-1.25	0.006333182	-0.04	125	-3.03	10442	0.01	121	0.78	7956	-0.05	133	-3.75	10510
AVEN	-1.01	0.88189072	-0.04	120	-3.03	10443	-0.07	102	-3.76	10955	-0.04	126	-2.68	10030
MBD3	-1.03	0.301335895	-0.04	124	-3.03	10444	-0.08	127	-6.02	12193	-0.13	110	-7.56	12052
CXCL9	-1.09	0.579237367	-0.04	136	-3.03	10445	0	138	-0.23	8628	-0.08	121	-5.7	11317
TCEB2_t2	1.14	5.09E-05	-0.04	119	-3.03	10446	0.06	123	4.53	5597	0.11	130	8.88	4269
MYOC	-1.01	0.864125016	-0.05	107	-3.03	10447	-0.06	101	-2.87	10391	0.17	126	13.23	2820
SFN	-1.13	0.0003556	-0.06	105	-3.04	10448	-0.2	140	-18.33	15776	-0.06	106	-3.64	10454
CXXC4	1.07	0.384406222	-0.03	156	-3.04	10449	0.01	131	0.58	8087	0.12	147	11.29	3418
OLIG3	-1.08	0.001584746	-0.03	142	-3.04	10450	-0.12	125	-9.03	13519	-0.09	123	-6.84	11787
CELA2B	1.01	0.521028903	-0.06	104	-3.04	10451	-0.18	95	-8.1	13154	-0.04	83	-1.4	9391
TBX4	1.18	0.000399435	-0.04	122	-3.04	10452	-0.06	120	-4.05	11125	-0.04	120	-2.77	10071
ZYG11B_t1	-1.03	0.133801167	-0.03	144	-3.04	10453	NA	NA	NA		-0.05	131	-3.75	10514
NXPH1	1.02	0.583726434	-0.03	139	-3.04	10454	-0.08	138	-7.35	12818	-0.16	142	-14.93	14363
KCNN4	-1.05	0.396204868	-0.05	107	-3.04	10455	0	131	0.16	8363	-0.21	113	-13.16	13891
HNRPDL_t1	-1.16	0.001275725	-0.04	118	-3.04	10456	0.04	117	2.46	6852	NA	NA	NA	NA
CD101	-1.02	0.120470021	-0.05	110	-3.04	10457	-0.02	124	-1.3	9368	-0.16	122	-11.69	13453
LYPD2	-1.04	0.000857941	-0.05	108	-3.04	10458	-0.11	88	-4.33	11280	-0.04	100	-1.89	9664
POLH	1.04	0.000370567	-0.03	150	-3.05	10459	0.16	139	14.2	1480	0.02	142	1.95	7477
P2RX6P	1.01	0.279110118	-0.06	97	-3.05	10460	-0.15	102	-7.92	13082	-0.02	72	-0.34	8810
NDUFB6	1.03	0.420017129	-0.04	127	-3.05	10461	-0.18	123	-12.91	14733	0.08	130	6.17	5370
TREM1	-1.21	0.000351396	-0.05	113	-3.05	10462	-0.02	144	-1.58	9540	-0.16	119	-10.89	13215
DGCR8	1.02	0.431097585	-0.05	109	-3.05	10463	0.05	129	3.77	6023	-0.12	121	-8.27	12350
MORF4L1_t1	-1.13	0.023716495	-0.05	115	-3.05	10464	NA	NA	NA		-0.03	118	-2.11	9763
ITGAM	-1.04	0.457685527	-0.05	113	-3.05	10465	0.13	118	8.86	3348	-0.17	127	-13.01	13840
HIST2H3A	-1.1	0.006889547	-0.23	63	-3.05	10466	-0.09	77	-2.45	10119	-0.24	76	-6.35	11594
AQPEP	-1.04	0.118921789	-0.04	131	-3.06	10467	-0.06	135	-4.76	11517	0.05	159	5.37	5747
BZW1	-1.26	2.98E-08	-0.04	127	-3.06	10468	-0.09	128	-6.7	12511	-0.12	143	-10.71	13152
DGKB_t2	1.02	0.0166638	-0.03	142	-3.06	10469	-0.01	130	-0.74	8962	-0.08	132	-6.53	11661
LMOD1	-1.03	0.550965544	-0.04	128	-3.06	10470	-0.03	138	-2.99	10467	-0.1	123	-7.18	11913
NPC1L1_t1	-1.1	5.40E-06	-0.05	106	-3.06	10471	NA	NA	NA		-0.15	101	-7.87	12186
CA13_t1	-1.03	0.035761348	-0.04	134	-3.06	10472	-0.04	127	-2.89	10402	NA	NA	NA	NA
UBE2G1	-1.07	0.030776386	-0.03	151	-3.06	10473	-0.01	157	-1.17	9269	-0.03	133	-2.69	10036
PGLYRP1	-1.04	0.007863848	-0.17	68	-3.07	10474	-0.12	104	-6.55	12435	-0.18	80	-5.53	11251
CYLD_t1	-1.16	0.000122533	-0.04	136	-3.07	10475	-0.04	130	-3.25	10632	NA	NA	NA	NA
TTTY8	-1.01	0.44191378	-0.05	118	-3.07	10476	-0.12	144	-11.67	14419	0.06	122	4.56	6150
KLF5_t1	-1.07	0.555307559	-0.03	144	-3.07	10477	NA	NA	NA	NA	-0.13	136	-10.91	13221
PODXL2	1.04	0.08832406	-0.08	88	-3.08	10478	0.08	120	5.33	5155	-0.08	88	-2.97	10166
TMEM2_t1	-1.03	0.542215209	-0.03	159	-3.08	10479	NA	NA	NA	NA	-0.02	149	-2.19	9788
ARMCX3_t1	-1.69	3.60E-09	-0.04	130	-3.09	10480	NA	NA	NA	NA	NA	NA	NA	NA
PGAM2	1.09	0.013809992	-0.08	88	-3.09	10481	0.03	89	1.27	7631	-0.01	80	-0.2	8731
TNFAIP6	-1.04	0.360341259	-0.05	116	-3.09	10482	0.01	110	0.34	8248	-0.18	135	-15.26	14431
GPR116_t1	1	0.94678063	-0.04	126	-3.09	10483	0.1	118	7.08	4210	NA	NA	NA	NA
SLC25A44	1.18	0.000264891	-0.04	129	-3.09	10484	0.01	125	0.94	7844	0.07	151	7.41	4863
UBN1	-1.01	0.855638874	-0.04	123	-3.1	10485	0.05	126	3.62	6099	-0.08	144	-7.25	11939
MAK	1	0.639484921	-0.04	133	-3.1	10486	-0.02	127	-1.75	9657	-0.02	126	-1.16	9275
NFKBIB	-1.03	0.077071245	-0.1	81	-3.1	10487	-0.13	83	-4.4	11327	-0.12	94	-5.16	11101
RHBG	1.31	0.063036339	-0.05	112	-3.1	10488	-0.05	131	-4.15	11178	-0.06	106	-3.6	10433
HEATR4	-1.01	0.51137488	-0.04	123	-3.1	10489	-0.11	113	-6.74	12529	-0.04	117	-2.54	9970
PTTG1IP_t1	-1.06	0.123256672	-0.04	124	-3.1	10490	0.05	156	5.54	5047	NA	NA	NA	NA
GMEB1	1.03	0.264696962	-0.04	119	-3.1	10491	0.15	135	13.1	1805	-0.01	120	-0.77	9068
KRT2	-1.01	0.629687609	-0.06	102	-3.1	10492	-0.01	127	-0.5	8818	-0.05	126	-3.57	10422
SEPSECS	-1.05	0.179308829	-0.04	133	-3.11	10493	-0.1	131	-8.32	13252	0.05	155	4.93	5961
COX16	1.04	0.363060581	-0.05	113	-3.11	10494	-0.09	166	-10.32	14009	0.02	127	1.63	7664
MACROD2_t1	1.13	0.008884028	-0.03	143	-3.11	10495	NA	NA	NA	NA	NA	NA	NA	NA
SNX14	-1.11	0.062914071	-0.06	105	-3.11	10496	-0.02	108	-1.4	9433	0.04	127	3.18	6843
WNT7A	-1.01	0.400953301	-0.05	110	-3.11	10497	-0.05	122	-3.43	10752	-0.03	100	-1.32	9350
CPLX4	-1	0.617724041	-0.04	122	-3.11	10498	-0.04	148	-3.47	10779	0	124	-0.33	8808
PCDHGB7	-1.01	0.532271185	-0.05	117	-3.11	10499	0	115	-0.02	8504	-0.05	115	-3.29	10303
SIGLEC15	1.36	3.68E-05	-0.05	116	-3.11	10500	-0.08	112	-4.92	11608	0.04	111	2.26	7316

KRT13	1.01	0.295879827	-0.06	100	-3.12	10501	-0.14	119	-9.47	13687	0	100	0.08	8564
OR8J1	-1.02	0.35247902	-0.1	80	-3.12	10502	-0.16	77	-4.44	11351	-0.05	72	-1	9201
TNFSF8	1.01	0.203009422	-0.04	136	-3.12	10503	0	128	0.06	8426	-0.07	144	-6.27	11561
WNT8A	1	0.963383424	-0.04	120	-3.12	10504	0	118	0.26	8287	-0.01	120	-0.52	8933
MAP1A	1.01	0.508439811	-0.04	120	-3.12	10505	0.05	128	3.67	6075	-0.02	128	-1.19	9287
POU3F4	1.02	0.04485315	-0.05	114	-3.12	10506	-0.09	109	-5.48	11901	-0.03	110	-1.74	9577
SARS	1	0.982686506	-0.05	111	-3.12	10507	-0.06	124	-4.55	11409	-0.04	109	-2.21	9799
PLEKHH1	1.04	0.021915249	-0.03	159	-3.12	10508	0.02	135	1.56	7458	0.09	126	7.14	4977
APAF1	1.02	0.525284367	-0.03	145	-3.12	10509	0.09	126	6.66	4415	0.17	140	14.92	2358
EYA1	1.06	0.013222667	-0.04	136	-3.13	10510	-0.05	122	-3.59	10869	-0.07	136	-6.24	11545
SLC6A18	-1.04	0.060369715	-0.06	100	-3.13	10511	-0.13	92	-5.41	11875	-0.09	84	-2.96	10164
TMEM40	1.05	0.001884366	-0.04	136	-3.13	10512	-0.05	127	-3.5	10798	-0.01	132	-0.46	8888
LRRTM2	-1.03	0.033113611	-0.04	123	-3.13	10513	0.02	145	2.28	6969	-0.05	153	-4.86	11001
IRX6_t1	-1.02	0.054117332	-0.04	120	-3.13	10514	-0.09	118	-5.85	12113	NA	NA	NA	NA
FBXL15	-1.01	0.81523022	-0.09	84	-3.13	10515	-0.11	102	-5.51	11921	-0.03	88	-1.2	9293
ASCC2	1	0.997513144	-0.06	99	-3.13	10516	-0.08	112	-4.93	11618	-0.01	110	-0.72	9036
TMEM151A	1.01	0.573793354	-0.06	103	-3.14	10517	-0.09	128	-7.33	12803	-0.04	120	-2.47	9933
HSD11B1L_t1	1.06	0.056495574	-0.05	108	-3.14	10518	-0.02	127	-1.29	9355	-0.07	119	-4.5	10838
CPT1B	-1.05	0.431377994	-0.04	125	-3.14	10519	-0.05	92	-2.29	10015	-0.13	89	-5.26	11141
COL19A1	1	0.753020877	-0.03	152	-3.14	10520	-0.03	146	-2.62	10231	-0.04	157	-4.77	10972
DGKZ	-1.08	5.02E-05	-0.03	140	-3.14	10521	-0.12	151	-12.33	14592	-0.13	120	-9.42	12729
ZRSR2	-1	0.958096617	-0.09	84	-3.15	10522	0.01	61	0.07	8421	-0.08	101	-4.05	10658
EIF3L	-1.13	0.003782706	-0.06	104	-3.15	10523	-0.08	115	-5.39	11864	-0.07	132	-5.63	11287
ADAM6_t1	1.09	4.57E-05	-0.05	120	-3.16	10524	NA	NA	NA	NA	0.04	157	4.59	6127
SHISA4	1.04	0.596178567	-0.05	112	-3.16	10525	-0.1	133	-7.89	13069	-0.03	140	-3.05	10207
ITPRIPL1	-1.03	0.107430516	-0.05	110	-3.16	10526	0.03	84	0.92	7861	-0.18	107	-10.33	13031
BLVRB	-1.15	0.006628145	-0.07	94	-3.16	10527	-0.12	104	-6.41	12378	-0.12	98	-5.78	11352
EIF2C4	-1.11	0.000264137	-0.04	136	-3.17	10528	-0.08	135	-6.6	12464	-0.05	148	-5.06	11071
CCR9	1	0.806193009	-0.04	140	-3.17	10529	0	111	-0.3	8680	0.01	130	0.86	8104
CLK3_t1	-1.02	0.364057662	-0.05	115	-3.17	10530	NA	NA	NA	NA	0	107	0.13	8540
MB	1.02	0.497957595	-0.05	111	-3.17	10531	-0.11	104	-5.86	12117	0.07	118	5.1	5882
SCLT1	-1.09	0.004015184	-0.05	119	-3.17	10532	0.03	112	1.8	7271	-0.09	120	-6.41	11610
MTMR14_t2	-1.02	0.472581299	-0.05	115	-3.17	10533	-0.06	107	-3.68	10925	-0.09	120	-6.23	11539
PRAMEF3	1.08	0.03548716	-0.05	113	-3.17	10534	-0.08	111	-4.88	11582	0.07	100	3.4	6721
USP16	-1.04	0.501681754	-0.03	145	-3.17	10535	0.02	101	1.06	7763	-0.01	100	-0.53	8942
KRT9	1.05	0.036689865	-0.05	120	-3.17	10536	-0.11	123	-8.06	13132	-0.01	112	-0.88	9125
ALDH3A1	1.46	0.000112332	-0.06	107	-3.18	10537	-0.11	123	-8.21	13196	-0.03	108	-1.64	9516
FBXO22_t2	1.08	0.099884435	-0.04	123	-3.18	10538	-0.01	135	-0.87	9060	0.08	133	6.27	5329
SAR1B	-1.11	0.102082223	-0.04	132	-3.18	10539	-0.13	134	-10.82	14182	0.09	146	8.63	4376
NLRP4	1.02	0.252975044	-0.05	119	-3.18	10540	-0.1	130	-8.37	13276	-0.06	109	-3.41	10361
FOXL1	-1.04	0.021012126	-0.03	148	-3.18	10541	-0.18	140	-15.95	15406	-0.06	133	-5.11	11081
SCRN1	-1.14	0.16201346	-0.04	134	-3.19	10542	0.14	127	10.45	2703	-0.1	125	-7.71	12124
YIPF4	-1.15	0.000114059	-0.05	117	-3.19	10543	-0.11	121	-8.12	13165	-0.1	122	-6.97	11831
SLC1A5	-1.08	0.10118291	-0.05	115	-3.19	10544	0.08	149	8.19	3646	-0.17	104	-9.4	12717
TMEM43	-1.09	0.006839221	-0.05	120	-3.19	10545	-0.01	128	-0.41	8757	-0.14	129	-11.1	13284
NOVA1_t1	1.06	0.006268767	-0.05	110	-3.19	10546	0.11	108	6.41	4560	0.01	108	0.74	8168
ANXA5	-1.18	0.02068771	-0.05	120	-3.19	10547	0.13	116	8.65	3434	-0.16	123	-11.94	13523
APEH	1.01	0.805760505	-0.06	100	-3.19	10548	-0.08	143	-7.7	12989	0.03	96	1.42	7799
PRAMEF10	-1.12	0.123855792	-0.15	72	-3.19	10549	NA	NA	NA	NA	NA	NA	NA	NA
TSPAN2_t1	-1.06	0.180702774	-0.04	134	-3.19	10550	NA	NA	NA	NA	NA	NA	NA	NA
CBLB_t1	-1.02	0.089056015	-0.05	113	-3.2	10551	NA	NA	NA	NA	-0.07	119	-4.73	10953
CD72	-1.36	2.64E-06	-0.07	98	-3.2	10552	-0.08	133	-6.73	12528	-0.23	142	-21.38	15565
SRRM2	-1.02	0.263954872	-0.05	109	-3.2	10553	-0.03	115	-1.93	9762	0	108	-0.14	8697
HHLA2	-1.03	0.000337171	-0.05	110	-3.2	10554	-0.1	130	-7.74	13006	-0.18	137	-15.81	14540
EMCN	-1.2	0.00887806	-0.04	130	-3.21	10555	-0.01	144	-0.87	9062	0	148	0.21	8490
MEIS3	-1.02	0.049470825	-0.08	92	-3.21	10556	0.05	118	3.68	6068	-0.09	88	-3.43	10367
RNH1	-1.04	0.277782135	-0.06	104	-3.21	10557	0	117	-0.31	8691	-0.08	101	-4.08	10673
DNASE2	1.02	0.721435135	-0.04	128	-3.21	10558	-0.14	123	-10.32	14012	-0.03	111	-2.04	9725
CTSW_t1	-1.1	4.32E-06	-0.08	88	-3.22	10559	NA	NA	NA	NA	-0.22	88	-8.47	12416
SIGLECL1	1.01	0.170874667	-0.05	118	-3.22	10560	-0.01	133	-0.43	8767	0.04	104	2.08	7409
UBA3	1.03	0.230250771	-0.04	130	-3.22	10561	0.12	124	8.8	3375	0.06	147	6	5452
KCNJ11	1.02	0.022931006	-0.04	128	-3.22	10562	-0.01	138	-1.24	9326	0.02	107	0.88	8087
SPI1	-1.07	1.93E-06	-0.07	95	-3.22	10563	-0.04	128	-2.75	10314	-0.23	112	-14.01	14118
BOC	-1	0.957422556	-0.06	100	-3.23	10564	0.02	115	1.57	7452	-0.04	121	-2.92	10142
TGFB1	-1.23	1.23E-05	-0.06	103	-3.23	10565	-0.06	88	-2.45	10121	-0.2	96	-9.04	12603
PRR14	1.02	0.510039015	-0.07	97	-3.23	10566	0.09	100	4.46	5630	0.01	85	0.27	8461
TNFRSF8	-1.05	0.18207127	-0.04	142	-3.23	10567	0.06	125	4.46	5632	-0.12	124	-8.81	12528
PCNXL2	-1.03	0.596074655	-0.07	94	-3.23	10568	0.03	109	2.02	7129	-0.12	121	-8.58	12447
SLC6A11	1.02	0.140322563	-0.09	87	-3.23	10569	0.11	94	4.93	5377	0	68	0.03	8599
AKR1A1_t1	-1.21	9.91E-06	-0.08	92	-3.23	10570	-0.21	93	-8.87	13458	NA	NA	NA	NA
CAPRIN2_t2	-1.03	0.219749021	-0.06	105	-3.23	10571	0.09	120	6.44	4549	0.01	142	1.15	7942
HSPG2	-1.02	0.118101519	-0.06	108	-3.23	10572	0.06	128	4.89	5396	-0.11	110	-6.72	11746
RAB40AL	1.09	7.04E-05	-0.06	108	-3.24	10573	0.03	104	1.81	7268	0.04	104	2.18	7356
MON1B_t1	-1.09	0.006711855	-0.05	118	-3.25	10574	NA	NA	NA	NA	-0.14	130	-11.33	13355
CLCNKA	1.06	3.88E-05	-0.06	100	-3.25	10575	-0.07	111	-4.51	11391	-0.04	111	-2.49	9946
GLIS2	-1.1	0.002897837	-0.05	120	-3.25	10576	-0.13	141	-11.89	14477	-0.07	127	-5.44	11218
RGPD5_t1	-1.2	0.000547631	-0.04	128	-3.25	10577	0.06	131	4.62	5537	NA	NA	NA	NA
RPL32P3	1.02	0.222236742	-0.04	127	-3.25	10578	0	123	0.14	8368	-0.02	140	-1.35	9367
COX14	-1.11	0.030353433	-0.09	88	-3.25	10579	-0.14	107	-8.24	13211	-0.07	92	-3	10179
DIAPH2_t2	-1.16	0.000148086	-0.08	90	-3.26	10580	-0.06	82	-2	9821	-0.01	82	-0.36	8823
CCDC38	-1.46	7.66E-08	-0.14	74	-3.26	10581	-0.14	104	-7.78	13020	-0.11	92	-4.64	10912
KCNAB2	-1.06	0.104410405	-0.04	126	-3.26	10582	0.01	133	0.48	8152	-0.04	127	-2.81	10092
PVRL2_t2	-1.26	0.000126698	-0.06	104	-3.26	10583	-0.13	119	-8.79	13428	-0.09	115	-5.74	11331
PIK3CG	1	0.803058004	-0.04	135	-3.26	10584	0.12	151	12.06	2115	0	119	-0.15	8698
USP19	1	0.998297903	-0.05	118	-3.26	10585	-0.13	93	-5.63	11990	-0.01	104	-0.34	8813
ACVR2A	-1.07	0.001085455	-0.04	142	-3.27	10586	0.09	129	6.76	4374	-0.06	158	-6.71	11737
PTPRN2	1.05	0.032347311	-0.04	124	-3.27	10587	0.08	127	6.42	4555	0.05	127	3.9	6469
KRT16_t1	-1.1	0.024696819	-0.08	92	-3.27	10588	NA	NA	NA	NA	NA	NA	NA	NA
PRAMEF8	1.18	2.59E-06	-0.05	117	-3.27	10589	-0.02	104	-1.08	9208	0.03	102	1.5	7749
CAMKMT	1.04	0.000702224	-0.04	127	-3.27	10590	0.03	141	2.42	6882	0.09	127	7.13	4980
CRIP1	-1.28	0.00344433	-0.04	136	-3.27	10591	0.03	106	1.54	7474	-0.23	105	-12.64	13745
Mar-07	-1.14	0.000510082	-0.04	138	-3.27	10592	0.02	127	1.49	7500	-0.04	134	-3.77	10521
WNK2	1.01	0.672265386	-0.09	88	-3.28	10593	0.12	113	7.29	4100	-0.06	92	-2.43	9906
ARHGEF10	-1.04	0.018089125	-0.04	131	-3.28	10594	0.08	129	6.32	4605	-0.14	148	-13.44	13963
PHF11	-1.12	0.015117687	-0.05	118	-3.28	10595	0.07	152	6.98	4257	-0.14	130	-11.39	13368
SNRNP35	-1.06	0.015693442	-0.07	94	-3.29	10596	-0.06	80	-1.71	9625	-0.19	85	-6.79	11768
PSMD13	-1.07	0.032501407	-0.08	92	-3.29	10597	-0.15	114	-9.88	13837	-0.09	112	-5.88	11394
KCNG1	-1.02	0.361413562	-0.07	100	-3.29	10598	-0.03	124	-1.94	9769	-0.06	105	-3.42	10362
MYL7	1.03	0.041386172	-0.11	79	-3.29	10599	-0.08	102	-4.12	11161	-0.18	106	-10.06	12949
MTFMT	1.04	0.176033739	-0.03	148	-3.3	10600	-0.02	121	-1.64	9582	0.15	141	13.33	2788
PDPN_t2	1.03	0.677906962	-0.03	148	-3.3	10601	0.03	128	2.04	7118	-0.15	123	-11.12	13288
AMZ1	1.07	0.069085868	-0.04	144	-3.3	10602	0.11	146	10.76	2567	-0.01	120	-0.92	9146
TSSK3	-1.06	2.71E-05	-0.18	68	-3.3	10603	-0.17	100	-8.39	13286	-0.1	88	-3.85	10567
TXNDC15	1.02	0.656942597	-0.04	131	-3.3	10604	-0.07	131	-5.82	12101	-0.01	127	-0.81	9092
RLN1	1	0.731165222	-0.06	102	-3.3	10605	0.05	122	3.3	6318	-0.02	140	-1.86	9643
FLII	-1.03	0.489543204	-0.08	90	-3.31	10606	0.03	134	2.68	6691	-0.03	108	-1.55	9471

SIX3	-1.05	0.02166196	-0.05	116	-3.31	10607	-0.17	145	-16.48	15507	-0.07	122	-5.36	11186
FXR1	-1.01	0.844453123	-0.03	150	-3.31	10608	0.09	136	7.46	4008	0	143	0.08	8565
WNT10B	-1.03	0.127009036	-0.04	127	-3.31	10609	0.1	146	10.05	2841	-0.1	131	-7.94	12213
MLLT11	1.29	0.005726883	-0.05	112	-3.31	10610	0.05	120	3.58	6126	0.09	127	7.25	4928
ZNF366	-1.02	0.471228899	-0.06	104	-3.31	10611	-0.03	113	-2	9824	-0.03	124	-2.04	9729
KIR2DS4	-1.06	0.000599274	-0.04	130	-3.32	10612	0.02	145	2.11	7073	-0.18	119	-12.3	13644
SPPL2A	-1.01	0.885852255	-0.05	113	-3.32	10613	-0.05	100	-2.56	10187	0.13	108	7.51	4827
PIGB	1.01	0.777529947	-0.06	108	-3.32	10614	0.04	116	2.31	6948	0.1	110	5.83	5528
ST7	-1.06	0.067326715	-0.08	94	-3.32	10615	-0.2	104	-11.05	14258	-0.09	121	-6.09	11480
SGK2_t1	-1.06	0.394261572	-0.04	129	-3.32	10616	-0.08	112	-4.93	11614	NA	NA	NA	NA
TIMP1	-1.85	2.24E-06	-0.11	80	-3.32	10617	-0.02	134	-1.27	9340	-0.23	100	-11.4	13373
GPR143	1.01	0.825202756	-0.06	102	-3.33	10618	-0.09	120	-6.61	12469	-0.08	135	-6.72	11745
PLEK2	-1.33	0.003111251	-0.07	101	-3.33	10619	-0.04	106	-2.32	10041	-0.07	142	-6.63	11707
THBS2	-1.26	0.02326485	-0.05	122	-3.33	10620	0.01	127	0.72	7983	-0.15	146	-14.37	14207
ATPAF1_t1	-1.27	2.51E-08	-0.03	155	-3.33	10621	NA	NA	NA	NA	-0.1	134	-8.64	12470
HTR3A_t1	1.05	0.017483434	-0.07	100	-3.33	10622	-0.05	126	-3.61	10888	NA	NA	NA	NA
CPVL	-1.68	1.28E-09	-0.06	108	-3.34	10623	-0.12	93	-5.01	11662	-0.25	98	-11.83	13496
NDST1	-1.07	0.002795154	-0.04	126	-3.34	10624	-0.18	138	-16.06	15432	-0.05	139	-4.24	10731
ZNF341	1.05	0.000238664	-0.06	106	-3.35	10625	0.04	126	3.25	6345	0.08	134	6.93	5062
LRRC29	-1.02	0.028283379	-0.09	88	-3.35	10626	-0.12	127	-9.04	13527	-0.08	120	-5.7	11321
ZBTB5	-1.05	0.087556382	-0.04	134	-3.35	10627	-0.1	129	-8.12	13163	-0.09	130	-6.95	11824
SLC34A2_t1	1.02	0.24435533	-0.04	130	-3.35	10628	NA	NA	NA	NA	-0.13	140	-11.59	13424
TNKS2	-1.07	0.008594098	-0.03	146	-3.35	10629	-0.03	141	-2.91	10424	-0.08	129	-6.15	11500
NAGS_t1	-1.14	0.305547226	-0.05	122	-3.36	10630	NA	NA	NA	NA	NA	NA	NA	NA
GOLGA2	1.04	0.202690676	-0.05	118	-3.36	10631	0.04	143	3.67	6073	-0.07	150	-6.84	11790
NAGA	1.01	0.665564232	-0.04	128	-3.36	10632	-0.08	129	-6.64	12485	0.05	145	4.42	6236
GABBR1	-1.06	0.001408495	-0.04	130	-3.36	10633	-0.09	134	-7.57	12907	-0.12	141	-10.84	13193
NDUFC1	-1.16	0.000162704	-0.06	102	-3.36	10634	-0.17	139	-15.41	15293	-0.01	118	-0.68	9023
MKNK2_t1	-1.07	0.14235909	-0.07	100	-3.36	10635	0	104	-0.16	8590	NA	NA	NA	NA
ZCCHC8	-1.03	0.474109794	-0.04	129	-3.37	10636	0.02	140	1.52	7486	-0.05	150	-4.52	10849
TSPAN6	-1.01	0.831138543	-0.05	117	-3.37	10637	-0.07	121	-5.27	11810	0.06	130	4.97	5944
PMPCB	1.05	0.258187759	-0.04	143	-3.37	10638	0.04	143	3.8	6009	0.11	131	8.69	4347
KATNA1	1.01	0.803641604	-0.05	121	-3.38	10639	0.04	90	1.48	7501	0.06	136	4.81	6022
CCDC120	-1.02	0.481997574	-0.06	105	-3.38	10640	0	121	0.02	8450	-0.15	123	-10.72	13159
TAL1	-1.08	0.003379825	-0.04	130	-3.38	10641	-0.08	138	-6.87	12591	-0.08	146	-8.15	12287
FBXL12	1	0.985145161	-0.05	120	-3.38	10642	0	115	0	8468	-0.13	117	-8.51	12431
MGAT2	-1.02	0.739830335	-0.04	142	-3.39	10643	-0.1	131	-7.9	13070	0.04	145	3.6	6613
GPR176	-1.02	0.081895167	-0.05	121	-3.39	10644	-0.12	126	-8.75	13419	-0.09	120	-6.41	11609
S100A7A	1.02	0.058983727	-0.03	147	-3.39	10645	0.01	154	0.69	8011	0	150	0.16	8511
HOXD11	1.04	0.025239721	-0.06	109	-3.39	10646	-0.14	119	-9.46	13682	-0.01	114	-0.91	9142
HLA-DRA	-1.69	8.77E-09	-0.05	119	-3.39	10647	-0.01	123	-0.43	8769	-0.26	104	-14.05	14122
ITIH3	-1.27	0.050782781	-0.08	94	-3.39	10648	-0.26	108	-15.25	15265	-0.05	109	-3.23	10284
COX6A2	-1.28	4.06E-05	-0.19	68	-3.39	10649	-0.19	96	-8.64	13371	-0.17	60	-1.74	9581
PGBD3	-1.04	0.124352387	-0.05	123	-3.4	10650	-0.07	107	-3.94	11067	0.05	104	2.44	7211
ZDHHC1	-1.07	1.63E-06	-0.08	91	-3.4	10651	-0.13	129	-10.43	14044	-0.22	120	-15.31	14441
CDH22_t1	1.27	0.001616108	-0.06	107	-3.4	10652	NA	NA	NA	NA	-0.06	113	-3.8	10536
IFNW1	-1.02	0.066288371	-0.05	113	-3.4	10653	-0.17	116	-11.42	14349	0.03	145	2.41	7230
RTN4IP1	1.11	0.01578541	-0.04	127	-3.4	10654	0.06	132	4.65	5523	0.07	121	4.65	6102
PRSS23_t1	-1.38	9.06E-05	-0.04	136	-3.41	10655	NA	NA	NA	NA	-0.09	134	-7.26	11946
CD40LG	1.01	0.388228626	-0.05	116	-3.41	10656	0.01	152	1.26	7633	0.06	111	3.76	6537
OR12D2	-1.02	0.068624424	-0.1	84	-3.41	10657	-0.09	85	-3.19	10594	-0.07	72	-1.5	9445
LRIG1	-1.14	0.006104637	-0.04	136	-3.41	10658	-0.21	121	-14.7	15147	0.02	163	2.34	7266
PHKG2	-1.02	0.289809715	-0.05	115	-3.42	10659	-0.01	133	-0.73	8948	-0.06	124	-4.33	10766
IRF7_t2	-1.57	3.13E-13	-0.16	72	-3.42	10660	-0.22	100	-10.8	14177	-0.26	68	-4.67	10921
GK_t1	-1.26	1.75E-06	-0.04	145	-3.42	10661	-0.18	134	-14.86	15177	-0.09	141	-7.76	12142
PEX10_t1	1.08	0.002295621	-0.05	121	-3.42	10662	0.02	130	1.55	7463	0.05	123	3.33	6755
GLT8D1	1.05	0.376755445	-0.06	107	-3.42	10663	0.06	99	2.72	6667	0.09	112	5.75	5560
RSPO2	1.04	0.01345911	-0.06	110	-3.42	10664	0.07	122	4.98	5353	-0.04	151	-4.16	10697
ATXN7	-1.02	0.321495633	-0.05	123	-3.43	10665	-0.03	138	-2.35	10053	-0.06	136	-5.53	11253
NRG1_t1	1.02	0.082141033	-0.05	120	-3.43	10666	-0.08	122	-5.75	12060	0.02	120	1.28	7860
POU4F1	-1.01	0.583583707	-0.04	131	-3.43	10667	-0.05	136	-4.39	11308	-0.02	157	-2.1	9758
KRT23	1.19	0.401627544	-0.06	104	-3.43	10668	0.06	110	3.59	6122	-0.06	168	-6.61	11703
SLFN5	-1.13	0.000266245	-0.04	128	-3.44	10669	-0.04	138	-3.64	10900	-0.24	127	-18.61	15101
NECAP2_t1	-1.13	8.90E-07	-0.04	137	-3.44	10670	NA	NA	NA	NA	-0.18	113	-11.63	13438
CFHR2	-1.86	0.000418312	-0.06	107	-3.44	10671	-0.23	102	-12.14	14547	0	110	0.09	8558
WISP1_t1	-1.01	0.15202342	-0.04	148	-3.44	10672	-0.07	146	-7.01	12658	-0.06	137	-5.4	11201
GRIN2C	1.03	0.007538293	-0.05	119	-3.44	10673	-0.03	123	-1.85	9731	0.02	115	1.2	7918
TECR	-1.15	5.55E-05	-0.16	71	-3.44	10674	-0.24	103	-12.9	14730	-0.22	103	-11.7	13455
FAM176C_t1	-1.03	0.383510063	-0.07	100	-3.45	10675	NA	NA	NA	NA	NA	NA	NA	NA
PPY	1.04	0.092413104	-0.08	93	-3.45	10676	-0.09	116	-6.2	12287	-0.05	100	-2.4	9893
ZNF132	1	0.847968943	-0.06	104	-3.45	10677	-0.03	130	-2.43	10110	-0.06	120	-4.02	10647
EXTL1	1.03	0.001781029	-0.04	129	-3.45	10678	0.02	134	1.56	7455	-0.02	128	-1.74	9574
WDR13	-1.05	0.297700041	-0.1	86	-3.46	10679	-0.08	104	-4.56	11423	-0.12	89	-4.54	10864
LOR_t1	-1.04	0.017067369	-0.06	104	-3.46	10680	NA	NA	NA	NA	-0.08	109	-4.61	10896
CLIP2	1.07	0.097837014	-0.05	124	-3.47	10681	0.06	127	4.63	5533	-0.09	137	-7.76	12146
NAP1L2	1.34	0.000135615	-0.04	132	-3.47	10682	0.07	108	4.11	5837	0.04	135	2.98	6934
TRAPPC12	-1.01	0.814174751	-0.07	101	-3.47	10683	0.02	113	1.45	7522	-0.18	93	-7.84	12180
RBM4	1.1	3.16E-05	-0.04	128	-3.47	10684	-0.02	109	-1.32	9377	-0.02	103	-1.05	9225
HERC6_t1	-1.06	0.004065508	-0.05	126	-3.47	10685	-0.11	156	-11.81	14449	NA	NA	NA	NA
CITED4	-1.19	0.029191151	-0.07	102	-3.47	10686	-0.04	108	-2.49	10146	-0.13	114	-8.4	12399
LRRTM1	-1.01	0.694055266	-0.05	126	-3.47	10687	-0.04	116	-2.5	10153	-0.03	109	-1.53	9460
DYSF	-1.24	9.95E-07	-0.08	92	-3.49	10688	-0.13	145	-12.04	14514	-0.23	103	-11.94	13525
KIAA0182	-1.07	0.026399434	-0.04	143	-3.49	10689	0.03	132	2.17	7024	0	148	-0.28	8779
PDHX	-1.07	0.177009486	-0.03	162	-3.49	10690	-0.03	114	-2.02	9834	-0.01	119	-0.84	9105
NRXN2	1.04	0.400568875	-0.06	108	-3.49	10691	-0.01	126	-0.41	8756	-0.08	104	-4.47	10830
KRT76	-1.01	0.459185995	-0.06	108	-3.49	10692	-0.12	118	-7.88	13066	-0.09	153	-9.16	12644
MCHR2_t1	-1.01	0.192891463	-0.04	135	-3.5	10693	NA	NA	NA	NA	-0.01	130	-0.78	9074
DRD2	-1.01	0.476070357	-0.05	116	-3.5	10694	-0.11	144	-10.25	13978	-0.07	115	-4.63	10908
UNC45B	-1	0.93050172	-0.04	140	-3.5	10695	-0.03	138	-2.64	10238	-0.01	144	-0.58	8964
S100A6	-1.36	0.041386172	-0.07	102	-3.5	10696	0.08	99	3.7	6062	-0.2	109	-11.65	13442
PPP1R26	-1.04	0.494440559	-0.06	112	-3.5	10697	-0.02	112	-1.05	9176	-0.14	106	-7.98	12232
ZFAND4	1.04	0.052591803	-0.05	118	-3.51	10698	0.11	123	7.99	3747	0.09	131	7.22	4936
NUDT13_t1	-1.06	0.218215155	-0.04	140	-3.51	10699	-0.09	127	-7.03	12667	NA	NA	NA	NA
MPI	1	0.966230521	-0.06	107	-3.51	10700	-0.1	128	-7.88	13065	0.01	121	0.68	8203
FAM217B	1.02	0.184498069	-0.04	133	-3.51	10701	-0.01	135	-0.74	8965	0.08	153	8.49	4428
GDPD5	-1.04	0.054572101	-0.05	123	-3.51	10702	-0.09	115	-5.88	12127	-0.04	88	-1.7	9552
PAX8_t2	1.01	0.179421843	-0.05	122	-3.51	10703	0.05	161	5.78	4905	0.04	127	2.75	7051
LRRN4	1.02	0.258988088	-0.05	115	-3.52	10704	-0.1	117	-6.99	12648	-0.01	108	-0.44	8873
ZNF281	-1.09	0.168835138	-0.05	118	-3.52	10705	-0.11	120	-7.53	12890	0.01	129	0.99	8025
RASGRP3	-1.06	0.235368562	-0.03	162	-3.52	10706	0.04	119	2.7	6675	-0.12	146	-11.47	13394
DAXX	1.07	0.014356853	-0.08	96	-3.52	10707	-0.04	96	-1.78	9676	-0.04	99	-1.91	9672
SCGB2A2	-1.02	0.593484456	-0.05	115	-3.52	10708	-0.07	93	-3.14	10568	0	111	-0.09	8672
HDAC9_t2	-1.01	0.712035445	-0.03	157	-3.52	10709	-0.05	122	-3.4	10730	0.02	143	1.52	7737
SHISA5	1.07	0.059217523	-0.04	131	-3.52	10710	0.05	133	4.05	5877	0.07	115	4.69	6078
CTNNAL1	-1.28	0.001608938	-0.05	121	-3.52	10711	-0.02	98	-0.9	9081	-0.01	120	-0.69	9028
ZNF10	-1.13	0.0009366	-0.04	130	-3.52	10712	0.06	123	4.17	5799	-0.09	134	-7.19	11917

RPS6KA5_t1	-1.05	7.02E-05	-0.05	119	-3.53	10713	-0.07	108	-3.78	10972	-0.16	116	-10.68	13145
MAP4K1	-1.1	0.000143162	-0.09	90	-3.53	10714	0	126	0.01	8456	-0.29	124	-21.2	15539
LMO1	-1.03	0.057683663	-0.07	104	-3.53	10715	-0.21	104	-11.27	14320	-0.09	96	-4.03	10651
HLA-DOA	-1.08	1.38E-05	-0.04	138	-3.53	10716	0.01	144	0.78	7960	-0.17	151	-17.09	14814
RSPH1	1.11	0.219716285	-0.04	145	-3.53	10717	0.03	103	1.76	7313	-0.13	116	-8.64	12467
ALPK1	-1.17	5.19E-05	-0.05	127	-3.53	10718	-0.02	134	-1.57	9526	-0.13	129	-10.45	13074
CACNG8	1.04	0.008944223	-0.04	147	-3.53	10719	-0.06	141	-5.23	11787	0.02	156	2.57	7144
SVOP	1.1	0.003631458	-0.05	128	-3.53	10720	0.04	127	2.77	6627	0.09	127	6.71	5151
SNAPC5	1.02	0.541647228	-0.07	104	-3.53	10721	-0.01	104	-0.31	8689	0.1	120	6.69	5158
SIM2_t2	1.02	0.052643161	-0.04	131	-3.54	10722	0.03	123	1.89	7222	-0.14	110	-8.28	12353
GHITM	-1.17	6.69E-05	-0.05	121	-3.54	10723	-0.17	137	-14.78	15166	-0.08	132	-6.61	11701
SOST	-1.02	0.301554792	-0.06	109	-3.54	10724	-0.08	127	-6.39	12365	-0.02	130	-1.38	9382
SLAMF9	-1.11	6.37E-06	-0.06	108	-3.54	10725	-0.1	115	-6.8	12561	-0.11	85	-3.68	10472
KRTDAP	-1.03	0.357279292	-0.09	88	-3.54	10726.5	-0.07	112	-4.15	11181	-0.1	88	-3.66	10466
OXSM	-1.08	0.027679815	-0.09	88	-3.54	10726.5	-0.15	86	-5.42	11879	-0.04	103	-1.86	9642
ABCB6	1.18	0.009256996	-0.05	127	-3.54	10728	0.02	104	1.21	7672	0.01	96	0.52	8307
PLB1	-1.05	0.00883495	-0.06	106	-3.54	10729	0	124	-0.06	8528	-0.13	100	-6.51	11648
MDFI	-1.01	0.638285115	-0.04	130	-3.55	10730	-0.02	110	-1.38	9417	-0.13	100	-6.38	11601
ADAMTS3	1.01	0.208844157	-0.04	138	-3.55	10731	0.07	135	5.78	4906	0.02	131	1.47	7766
RGS4	-1.1	0.000752384	-0.04	150	-3.55	10732	-0.02	126	-1.38	9415	-0.17	127	-12.95	13832
ATP6V1G3	1.01	0.336826991	-0.06	112	-3.55	10733	0.06	117	4.27	5728	-0.1	106	-5.5	11239
DHX38	-1.04	0.159340193	-0.06	114	-3.55	10734.5	-0.15	104	-7.89	13068	-0.1	102	-5.38	11190
PDIA3	1.01	0.880265967	-0.06	114	-3.55	10734.5	-0.07	121	-5.09	11715	0.03	120	2.28	7305
TOMM20L	-1.01	0.713028751	-0.06	110	-3.55	10736	-0.13	122	-9.27	13616	-0.04	103	-2.05	9735
ERLIN2_t1	-1.11	0.013778568	-0.04	136	-3.55	10737	NA	NA	NA	NA	NA	NA	NA	NA
ARHGAP27_t1	-1.32	2.16E-08	-0.07	104	-3.55	10738	-0.17	111	-10.29	14000	-0.14	136	-12.36	13659
METTL17	1.05	0.037191653	-0.04	130	-3.55	10739	-0.09	73	-2.18	9953	-0.04	79	-1.19	9288
AQP4	-1.05	2.15E-05	-0.04	129	-3.55	10740	-0.06	146	-5.79	12082	-0.12	134	-9.97	12917
MAP3K6	-1.44	9.03E-10	-0.09	90	-3.56	10741	-0.02	122	-1.32	9376	-0.25	100	-12.73	13769
ZNF653	1.02	0.321857884	-0.05	115	-3.56	10742	-0.1	96	-4.4	11322	-0.08	104	-4.13	10691
FAM213A	-1	0.942701347	-0.05	120	-3.56	10743	-0.08	127	-5.96	12161	0.03	110	1.84	7539
THUMPD1_t1	-1.02	0.497032725	-0.04	142	-3.56	10744	0.08	147	8.05	3713	NA	NA	NA	NA
CCNG1	-1.06	0.298377463	-0.05	115	-3.57	10745	-0.02	150	-2.39	10084	0.02	118	1.28	7864
ITCH	-1.16	0.000378175	-0.04	130	-3.57	10746	-0.16	133	-13.17	14790	0.03	136	2.58	7141
PPAN-P2RY11	-1.04	0.009961694	-0.06	114	-3.57	10747	-0.09	104	-4.9	11599	-0.1	116	-6.6	11695
CCDC9	-1.06	0.003568659	-0.09	91	-3.57	10748	-0.08	120	-5.68	12020	-0.22	104	-11.76	13475
PRB2	-1.03	0.003275917	-0.08	94	-3.57	10749	-0.16	99	-7.64	12952	0	112	-0.08	8667
MZF1	-1	0.930592754	-0.05	118	-3.57	10750	-0.01	101	-0.74	8964	-0.09	100	-4.55	10869
ZCCHC14_t1	-1.23	2.14E-06	-0.04	134	-3.57	10751	NA	NA	NA	NA	-0.05	164	-5.91	11410
TTYH2	1.03	0.009535231	-0.06	112	-3.57	10752	0.06	141	5.15	5254	0.13	128	9.92	3908
MCTP2	-1	0.806554817	-0.04	140	-3.57	10753	0.11	150	11.19	2404	-0.1	139	-8.52	12434
FOXI1	-1.01	0.265914338	-0.06	114	-3.57	10754	-0.09	141	-7.98	13108	-0.07	120	-5.03	11062
FLT4_t1	-1	0.705871625	-0.06	108	-3.57	10755	-0.05	141	-4.29	11260	-0.05	124	-3.45	10374
GALK1	1.05	0.523238806	-0.07	100	-3.58	10756	-0.11	96	-4.94	11622	-0.07	106	-4.19	10710
IL28A	1.02	0.293404385	-0.07	100	-3.58	10757	-0.14	88	-5.16	11756	-0.05	101	-2.35	9872
CCK	-1.15	0.002604319	-0.09	92	-3.58	10758	-0.13	132	-11.01	14244	-0.16	119	-10.83	13190
CHORDC1	-1.26	7.88E-05	-0.04	152	-3.58	10759	0	125	0.14	8371	-0.06	136	-4.81	10988
FAM53B	-1.06	0.001429904	-0.04	132	-3.58	10760	0.06	139	5.48	5067	-0.14	145	-13.02	13844
NCR2	-1.02	0.126625642	-0.07	102	-3.58	10761	-0.1	71	-2.17	9941	-0.07	57	-0.51	8922
GPC6	-1.04	0.223329346	-0.04	138	-3.58	10762	-0.13	148	-12.48	14632	-0.08	147	-7.96	12222
MCOLN1	-1.04	0.210483661	-0.06	110	-3.58	10763	-0.04	84	-1.21	9293	-0.04	100	-2.02	9714
RALGDS_t1	-1.32	2.63E-05	-0.05	120	-3.59	10764	NA	NA	NA	NA	-0.17	116	-11.54	13412
SAFB	-1.03	0.067673471	-0.05	124	-3.59	10765	0.04	105	2.22	7000	-0.14	108	-7.93	12208
SOX17_t1	-1.36	3.92E-08	-0.05	123	-3.59	10766	NA	NA	NA	NA	-0.14	132	-11.09	13280
MGAT4B	1.04	0.366855346	-0.05	129	-3.59	10767	-0.11	115	-6.96	12628	0.05	111	3.28	6785
ZNF451_t1	1	0.937599616	-0.04	134	-3.59	10768	0.07	119	4.93	5380	0.07	134	5.55	5661
ANKRD35	1.01	0.945432146	-0.09	92	-3.6	10769	-0.1	95	-4.65	11465	-0.04	92	-1.53	9458
PRPS1L1	-1.11	0.005057289	-0.07	100	-3.6	10770	-0.13	88	-4.79	11536	-0.1	91	-4.12	10683
KIF2B	1.01	0.209215193	-0.08	93	-3.6	10771	-0.09	111	-5.19	11770	0.03	104	1.51	7743
RAB33B	-1.06	5.18E-05	-0.04	134	-3.6	10772	-0.09	140	-7.88	13064	-0.05	140	-4.63	10906
MYL4_t1	1.01	0.393508382	-0.08	95	-3.6	10773	NA	NA	NA	NA	-0.01	84	-0.29	8789
FAM167A	-1.05	0.042787517	-0.05	123	-3.61	10774	-0.04	134	-3.6	10874.5	-0.03	143	-2.6	9997
NR5A2	-1.02	0.409132605	-0.05	126	-3.61	10775	-0.03	124	-2.43	10107	0.01	124	0.92	8069
CCDC106	-1.03	0.092964837	-0.09	88	-3.61	10776	-0.17	104	-9.08	13549	-0.1	105	-5.52	11248
DARC	-1.01	0.602340372	-0.09	88	-3.61	10777	0.04	120	2.83	6597	-0.08	90	-3.35	10334
RAB11B	-1.01	0.722006585	-0.06	112	-3.61	10778	-0.09	122	-6.61	12472	-0.08	111	-4.85	10998
TP53I13_t1	-1.19	3.19E-06	-0.08	98	-3.61	10779	-0.2	125	-15.18	15244	NA	NA	NA	NA
OPRM1_t2	1.02	0.032841699	-0.04	136	-3.62	10780	-0.06	104	-3	10474	-0.01	119	-0.76	9062
SPATA9	1.02	0.051092739	-0.05	122	-3.62	10781	0.08	124	6	4784	-0.02	123	-1.5	9446
FAM221A	-1	0.942247203	-0.06	113	-3.62	10782	0.01	115	0.92	7860	0.02	112	1.52	7733
PITPNB	-1.05	0.176621779	-0.04	140	-3.62	10783	0.08	129	6.03	4765	-0.03	124	-2.03	9722
BRSK2	-1.01	0.609728524	-0.06	110	-3.62	10784	-0.11	122	-8.16	13176	0	114	-0.2	8734
ABCA2	-1.03	0.162230881	-0.07	103	-3.62	10785	-0.11	131	-8.69	13392	-0.13	116	-8.42	12408
ZFPM1	-1.42	1.19E-08	-0.09	89	-3.62	10786	-0.15	113	-9.49	13700	-0.17	123	-12.27	13633
ZNF444	-1.01	0.799536708	-0.06	111	-3.62	10787	0	110	0.19	8341	-0.08	122	-5.56	11262
EMID2	1.02	0.221625548	-0.05	120	-3.63	10788	-0.13	121	-9.1	13560	-0.01	116	-0.49	8903
TOE1	-1.04	0.119330022	-0.05	128	-3.63	10789	-0.09	125	-6.77	12543	-0.08	120	-5.51	11245
MYLIP	-1.01	0.8312186	-0.04	140	-3.63	10790	0.05	137	4.53	5601	-0.1	136	-8.93	12570
VTI1B	-1.09	0.009808503	-0.06	108	-3.63	10791	-0.17	104	-9.34	13634	-0.04	124	-3.05	10204
UROS	-1.16	9.87E-05	-0.07	104	-3.63	10792	-0.19	127	-14.31	15055	-0.06	100	-2.95	10158
GPR135	1.03	0.376622879	-0.06	107	-3.63	10793	-0.07	102	-3.7	10930	-0.02	112	-1.21	9306
PIN1	-1.09	0.000141958	-0.08	96	-3.64	10794	-0.15	131	-12.51	14644	-0.13	92	-5.31	11165
MRPL12	1.09	0.031569529	-0.06	108	-3.64	10795	0.05	101	2.3	6958	0.02	99	0.93	8054
C4B	-1.5	3.34E-07	-0.09	91	-3.64	10796	-0.23	95	-10.13	13929	-0.15	100	-7.37	11985
FAM35A	1.01	0.682318089	-0.06	113	-3.64	10797	-0.03	133	-2.25	9993	0.13	148	12.52	3039
RSL1D1	-1.05	0.237773187	-0.04	149	-3.65	10798	0.07	139	6.11	4723	-0.04	131	-3.55	10417
GLCCI1_t1	1.03	0.624501356	-0.04	138	-3.65	10799	0.06	139	5.7	4952	NA	NA	NA	NA
C3	-1.64	1.60E-06	-0.17	72	-3.65	10800	-0.32	136	-27.67	16431	-0.22	80	-6.75	11756
TLR10	-1.02	0.234117693	-0.05	127	-3.65	10801	-0.06	125	-4.43	11347	-0.04	120	-3.02	10187
DLK2	1.05	0.006047024	-0.08	94	-3.65	10802	0.01	103	0.63	8043	0.02	97	0.75	8163
ELP6	-1.01	0.791356081	-0.06	108	-3.66	10803	-0.09	127	-6.66	12495	-0.08	97	-3.58	10427
SLC27A6	1	0.918314639	-0.05	120	-3.66	10804	-0.01	98	-0.44	8777	0.04	123	2.73	7064
KIR2DL5A	-1.01	0.295614988	-0.03	159	-3.66	10805	-0.1	147	-9.85	13827	-0.07	155	-6.96	11828
CIR1	-1.04	0.398298425	-0.06	116	-3.66	10806	0.04	111	2.14	7053	-0.09	110	-5.39	11195
SLC25A2	-1.05	0.000538529	-0.06	113	-3.66	10807	-0.18	102	-9.28	13618	0.01	141	1.19	7921
TGM1	-1.02	0.308355856	-0.07	100	-3.66	10808	-0.01	118	-0.93	9097	-0.16	150	-15.66	14513
NEK11_t2	-1.02	0.413130753	-0.05	117	-3.66	10809	-0.02	104	-0.96	9121	-0.12	119	-8.12	12276
GARNL3	1.03	0.11095787	-0.06	115	-3.66	10810	0	136	-0.03	8514	-0.04	100	-2.23	9804
RAB30	-1.06	0.005727417	-0.06	115	-3.67	10811	0.06	126	4.58	5567	-0.13	152	-13.32	13925
CHST2	-1.16	0.012262427	-0.04	144	-3.67	10812	0.06	118	4.07	5869	-0.21	115	-13.55	13984
ZNF771	-1.08	0.04668425	-0.08	94	-3.67	10813	-0.02	135	-1.47	9473	-0.05	120	-3.6	10435
CABP2	-1.03	0.029310255	-0.05	117	-3.67	10814	-0.25	108	-14.46	15095	-0.1	98	-4.85	10997
PPP2R1B_t1	-1.4	3.26E-05	-0.12	80	-3.67	10815	-0.11	109	-6.3	12323	-0.11	151	-10.78	13176
WDR16	1.01	0.447779341	-0.05	121	-3.67	10816	-0.03	113	-1.84	9720	-0.11	138	-10.02	12933
DHX32	-1.19	0.00036197	-0.06	116	-3.67	10817	-0.03	110	-1.76	9665	-0.06	96	-2.84	10107
FIBCD1	1.02	0.117071403	-0.05	122	-3.67	10818	0	129	-0.15	8574	-0.06	123	-4.61	10898

ACO2	-1.07	0.056862814	-0.08	97	-3.67	10819	-0.01	111	-0.47	8800	-0.07	126	-5.48	11235
EPHB4	-1.1	0.000842696	-0.06	108	-3.67	10820	-0.07	107	-3.94	11064	-0.07	154	-7.69	12111
TCTE1	-1.01	0.490499071	-0.07	104	-3.68	10821	-0.08	64	-1.13	9239	-0.06	79	-1.81	9615
ADD3	-1.4	6.99E-06	-0.04	147	-3.68	10822	-0.01	121	-0.61	8883	-0.06	143	-6	11438
ACP5	-2.36	1.27E-14	-0.08	96	-3.68	10823	-0.01	120	-0.36	8721	-0.32	98	-15.3	14438
MAGEE2	-1.09	1.30E-06	-0.06	110	-3.69	10824	-0.26	120	-18.36	15783	-0.07	116	-4.76	10965
FOXN3	-1.02	0.672636327	-0.04	148	-3.69	10825	-0.05	138	-3.97	11086	0.02	135	1.92	7497
WNT10A	-1.11	0.000506847	-0.07	100	-3.69	10826	-0.04	129	-3.15	10574	-0.23	152	-23.26	15800
FGFR3	-1.28	0.006060673	-0.05	118	-3.69	10827	-0.05	131	-4.01	11104	-0.16	130	-12.9	13820
HAPLN2	1	0.842436719	-0.1	88	-3.69	10828	-0.02	100	-0.83	9034	-0.01	84	-0.5	8915
ADAP1	-1.06	0.000310017	-0.06	108	-3.69	10829	-0.07	101	-3.36	10711	-0.23	124	-17.25	14839
OXCT2	1	0.99102727	-0.07	104	-3.69	10830	-0.08	116	-4.95	11629	-0.02	107	-1.07	9232
EPHX1	-1.16	0.128565051	-0.06	107	-3.69	10831	-0.18	120	-12.75	14702	-0.05	88	-1.83	9631
PFKFB2_t2	1.03	0.139199475	-0.03	171	-3.7	10832	-0.16	151	-16.37	15486	0.06	151	6.53	5219
ZNF654	-1.08	0.000632125	-0.04	145	-3.7	10833	-0.06	128	-4.9	11594	0.02	143	2.24	7328
PTPRM	-1.02	0.787327247	-0.05	122	-3.7	10834	0.02	119	1.49	7498	-0.06	147	-5.67	11302
ACER1	1.01	0.686274571	-0.07	104	-3.7	10835	-0.16	131	-12.65	14676	-0.04	130	-3.04	10203
APLP1	-1.02	0.230802068	-0.07	103	-3.7	10836	-0.12	119	-8.47	13314	-0.1	102	-5.2	11115
PI3	-1.02	0.424882627	-0.1	88	-3.71	10837	-0.12	116	-7.77	13015	-0.11	100	-5.38	11189
TNS4	1.01	0.439203627	-0.05	124	-3.71	10838	0	129	-0.24	8637	-0.08	148	-7.72	12125
L3MBTL3	-1.16	1.69E-05	-0.05	130	-3.71	10839	0.05	118	3.23	6356	-0.08	138	-7.07	11872
GNA11	-1.04	0.230851963	-0.07	100	-3.72	10840	-0.02	130	-1.95	9777	-0.07	101	-3.33	10325
RAB7A	-1.03	0.210596064	-0.05	124	-3.72	10841	-0.03	133	-2.68	10263	-0.09	127	-6.97	11833
SRSF6	-1.1	7.43E-06	-0.04	156	-3.72	10842	-0.05	126	-4.11	11157	-0.12	127	-9.35	12696
OTOGL	-1	0.856880904	-0.04	135	-3.72	10843	-0.11	114	-6.95	12626	-0.04	130	-3.49	10389
MGEA5	-1.16	0.009645205	-0.05	120	-3.72	10844	-0.07	120	-5.08	11707	-0.08	131	-6.44	11619
CDX1	-1.01	0.530923091	-0.05	121	-3.73	10845	-0.07	121	-5.23	11788	-0.09	118	-6.43	11615
RTKN	-1.01	0.854396939	-0.08	99	-3.74	10846	-0.02	107	-0.86	9049	-0.03	133	-2.52	9962
KRT5	-1.02	0.364676699	-0.07	102	-3.74	10847	-0.1	116	-6.32	12337	-0.04	109	-2.55	9976
PHKG1	1.01	0.678323297	-0.05	120	-3.74	10848	-0.08	125	-6.26	12307	-0.1	127	-7.43	12006
ZMAT4	1.02	0.152624333	-0.06	113	-3.74	10849	0	112	-0.27	8650	-0.04	114	-2.35	9870
GABRA6	-1.01	0.500773813	-0.07	104	-3.74	10850	-0.07	130	-5.92	12142	0.01	125	0.89	8083
ITM2B	-1.4	8.39E-09	-0.04	136	-3.74	10851	-0.14	111	-8.26	13219	-0.19	115	-12.03	13553
SPRY3_t1	-1.02	0.438030688	-0.04	141	-3.74	10852	NA	NA	NA	NA	-0.02	143	-1.87	9651
LGALS7	-1.06	0.118101988	-0.11	84	-3.74	10853	-0.05	53	-0.14	8572	-0.07	76	-1.92	9679
KHDRBS2	-1.01	0.539242603	-0.04	141	-3.74	10854	-0.16	112	-9.91	13848	-0.02	125	-1.71	9559
BNIP3L_t1	-1.37	2.84E-08	-0.05	133	-3.75	10855	-0.05	121	-3.31	10685	NA	NA	NA	NA
GJC2	-1.05	0.17863805	-0.06	111	-3.75	10856	-0.07	104	-3.83	10992	-0.06	115	-3.83	10548
MSR1_t1	-1.29	1.18E-12	-0.04	136	-3.75	10857	0.01	128	1.02	7795	-0.18	138	-16.28	14642
OBSCN	-1.01	0.653577575	-0.06	117	-3.76	10858	-0.04	111	-2.37	10060	-0.05	108	-2.86	10118
ANKRD53_t1	1	0.708790662	-0.07	102	-3.76	10859	NA	NA	NA	NA	-0.04	104	-1.92	9680
IRX4	1	0.990229489	-0.07	104	-3.76	10860	0.04	143	4.09	5849	-0.07	147	-6.85	11791
LYZL2	-1	0.834706398	-0.07	104	-3.77	10861	-0.18	108	-10.25	13980	-0.02	113	-1.32	9351
EIF3F_t1	-1.04	0.230316119	-0.08	100	-3.77	10862	NA	NA	NA	NA	-0.18	92	-7.64	12089
MEOX1	1.01	0.532611881	-0.06	111	-3.77	10863	0.01	138	1.14	7717	-0.02	110	-1.4	9390
CLCNKB	-1.03	0.018851501	-0.08	100	-3.78	10864	-0.2	108	-11.71	14428	-0.03	111	-1.75	9589
SCUBE3	1.01	0.357359809	-0.06	115	-3.78	10865	-0.02	140	-1.89	9746	0.03	120	1.94	7489
DCTN1	-1.02	0.263439379	-0.08	100	-3.78	10866	-0.14	124	-10.42	14040	-0.07	103	-3.86	10575
ING3	1.02	0.572247701	-0.05	126	-3.78	10867	0.04	143	3.44	6225	0.02	131	1.48	7762
PLXNA4	1.04	0.043281256	-0.06	117	-3.78	10868	0.04	88	1.61	7425	0	98	-0.01	8625
GCNT1	-1.18	2.79E-05	-0.05	129	-3.79	10869	-0.04	126	-2.98	10462	-0.21	136	-17.67	14918
PDE4A_t1	-1.59	1.69E-07	-0.07	107	-3.79	10870	0.03	164	3.82	5995	NA	NA	NA	NA
LCE3E	-1.1	0.000274347	-0.06	113	-3.79	10871	-0.08	108	-4.8	11542	-0.11	113	-6.89	11809
ASB16	-1	0.779712244	-0.05	127	-3.8	10872	-0.1	121	-7.1	12696	-0.08	119	-5.32	11168
CLDN4	-1.07	0.322945857	-0.07	102	-3.8	10873	-0.07	127	-5.3	11823	-0.27	107	-15.13	14402
GAL3ST2	-1	0.867194772	-0.07	103	-3.8	10874	-0.12	100	-6.12	12256	0.01	104	0.45	8341
MS4A3	1	0.640416997	-0.05	131	-3.8	10875	0.01	111	0.79	7952	0.05	123	3.47	6683
SLC22A6	1.05	0.000436067	-0.1	88	-3.8	10876	-0.03	92	-1.45	9462	0.06	91	2.37	7252
FYCO1	-1	0.930293947	-0.05	133	-3.81	10877	0.07	128	5.43	5100	0.03	161	3.53	6659
PRSS16	1.05	0.091705949	-0.04	147	-3.81	10878	0.06	124	4.13	5823	-0.06	145	-5.81	11373
PPP3CC	-1.33	9.60E-10	-0.05	123	-3.81	10879	-0.11	104	-5.95	12159	-0.22	114	-14.27	14178
OPN1SW	-1.02	0.311531472	-0.06	112	-3.82	10880	-0.12	79	-3.36	10713	-0.03	108	-1.57	9483
FILIP1	-1.07	0.107300567	-0.06	114	-3.82	10881	-0.02	122	-1.58	9543	-0.02	121	-1.16	9274
ILK_t1	-1.06	0.11166859	-0.08	100	-3.82	10882	NA	NA	NA	NA	-0.16	113	-10.17	12979
HMGB1_t1	-1.15	0.000294627	-0.05	133	-3.82	10883	0.03	135	2.58	6773	NA	NA	NA	NA
RNFT1	-1.15	0.010941446	-0.04	154	-3.82	10884	-0.09	103	-4.91	11605	0.01	134	1.23	7896
KLF16	-1.05	0.014585292	-0.04	138	-3.83	10885	-0.04	120	-3.15	10569	-0.07	134	-5.74	11333
DCBLD1	-1.05	0.001006843	-0.09	93	-3.83	10886	-0.1	102	-5.04	11680	-0.13	103	-6.74	11752
UNC50	1.02	0.635466329	-0.06	112	-3.83	10887	0.01	147	0.71	7993	-0.03	108	-1.87	9653
IMPACT_t1	-1.04	0.419921271	-0.05	128	-3.83	10888	0.02	139	2.08	7094	NA	NA	NA	NA
PFKM	-1.04	0.558107228	-0.05	129	-3.83	10889	0.09	122	6.15	4703	-0.1	124	-7.28	11952
IFITM4P	-1.11	0.036837254	-0.06	115	-3.83	10890	-0.13	119	-9	13505	-0.15	102	-7.58	12061
COL9A1	1	0.865173051	-0.04	140	-3.84	10891	-0.13	131	-10.38	14032	-0.02	135	-1.42	9401
ACTL7B	1.01	0.464566788	-0.06	117	-3.84	10892	-0.12	99	-5.66	12007	-0.03	103	-1.6	9502
MMEL1	1.02	0.351783862	-0.05	120	-3.85	10893	-0.05	115	-3.29	10664	-0.02	104	-1.14	9271
RNF103	-1.04	0.47484435	-0.05	124	-3.85	10894	-0.02	114	-1.05	9181	0.04	104	2.38	7241
ADAM28_t1	-1.18	0.000973673	-0.06	119	-3.85	10895	0.04	115	2.42	6876	-0.14	142	-12.8	13790
ASB10	-1.08	0.013545204	-0.09	92	-3.85	10896	-0.17	147	-16.63	15535	-0.13	120	-9.09	12622
F5	-1.13	0.250932399	-0.04	147	-3.85	10897	-0.16	122	-11.24	14309	0.08	147	7.93	4648
MPG	-1.17	0.000270431	-0.09	93	-3.85	10898	-0.1	81	-3.18	10590	-0.18	91	-7.25	11937
RAB40C	-1.01	0.791773889	-0.04	154	-3.85	10899	-0.04	154	-3.82	10990	-0.04	139	-3.31	10311
COL3A1	-1.47	0.001208391	-0.06	115	-3.85	10900	0.02	130	1.85	7244	-0.15	139	-13.61	14004
SCARF2_t1	-1.28	3.75E-06	-0.06	115	-3.85	10901	NA	NA	NA	NA	-0.11	113	-7.11	11887
FRMPD4	-1.01	0.547335599	-0.04	143	-3.85	10902	-0.07	125	-5.55	11945	0	125	0.13	8536
TMEM106A	-1.09	5.92E-06	-0.05	134	-3.85	10903	-0.04	131	-3.27	10654	-0.15	120	-10.24	13007
IL12RB1_t2	-1.05	0.001922591	-0.07	109	-3.86	10904	-0.15	127	-11.6	14398	-0.12	119	-8.45	12414
TBC1D14	1.09	0.012802847	-0.05	129	-3.86	10905	0.1	146	9.97	2873	-0.01	122	-0.99	9196
PLEKHA4	-1.35	9.47E-09	-0.1	88	-3.86	10906	-0.07	108	-4.03	11114	-0.22	124	-15.99	14587
KRTAP5-9	1.02	0.259509886	-0.06	111	-3.86	10907	-0.03	120	-2.22	9976	-0.05	120	-3.75	10512
GDF5	1.01	0.339921303	-0.06	111	-3.86	10908	-0.11	138	-9.6	13734	0	120	-0.03	8637
SOX14	1.01	0.383208237	-0.06	117	-3.87	10909	-0.01	123	-0.98	9131	-0.09	123	-6.32	11577
SYT5	-1.04	0.064886908	-0.09	92	-3.87	10910	-0.16	151	-15.86	15380	-0.13	120	-9.25	12669
TOP1	-1.1	0.015695572	-0.05	125	-3.87	10911	-0.05	122	-3.9	11037	-0.08	121	-5.36	11185
KLC1	-1.15	5.15E-05	-0.08	97	-3.87	10912	-0.05	113	-2.98	10463	-0.24	113	-15.04	14385
VSTM4_t2	1.09	0.000256204	-0.04	144	-3.88	10913	0	149	0.04	8440	0.01	128	0.57	8281
CCDC151	-1.14	4.69E-07	-0.22	68	-3.88	10914	-0.17	108	-10.11	13922	-0.23	110	-13.93	14087
ARID5B_t1	-1.07	0.005391709	-0.05	132	-3.89	10915	NA	NA	NA	NA	-0.05	148	-4.58	10882
PGAP3	-1.02	0.14308484	-0.05	124	-3.89	10916	-0.19	130	-15.11	15225	-0.09	129	-6.85	11792
SLC35C1	-1.07	0.36752746	-0.06	114	-3.89	10917	-0.08	137	-6.91	12608	-0.05	111	-2.79	10083
PRSS21_t1	-1.01	0.607759782	-0.06	120	-3.89	10918	-0.16	130	-13.05	14765	-0.07	110	-4.16	10698
SLC38A11	1.13	0.025127317	-0.06	112	-3.89	10919	0.08	104	4.07	5866	0.08	127	5.93	5479
RTP3	-1.47	0.014884417	-0.09	93	-3.89	10920	-0.17	73	-3.97	11090	0.01	71	0.29	8447
NT5E	-1.14	0.006998985	-0.05	125	-3.9	10921	-0.14	120	-9.92	13852	-0.11	124	-7.95	12218
AKIRIN1	-1.17	3.94E-05	-0.05	131	-3.9	10922	0.02	131	1.73	7325	-0.14	155	-14.59	14266
AVIL	-1.12	0.009218573	-0.06	119	-3.9	10923	-0.08	140	-6.88	12598	-0.08	135	-6.73	11749
HIST1H3C	-1.02	0.678499568	-0.13	80	-3.9	10924	-0.02	100	-0.9	9083	-0.18	84	-6.16	11503

LY6G6C	-1.01	0.440789407	-0.04	143	-3.91	10925	-0.15	132	-12.27	14574	0.04	102	2.03	7429
PTCH2	-1.12	1.22E-07	-0.08	98	-3.91	10926	-0.18	90	-7.01	12659	-0.15	100	-7.54	12043
KLK7	1.02	0.052749613	-0.06	121	-3.91	10927	0.01	115	0.79	7950	0	122	0.23	8476
FAM189A2	-1.02	0.050197592	-0.06	116	-3.91	10928	-0.05	137	-4.18	11194	-0.03	129	-2.74	10057
POLD4_t1	-1.02	0.531511383	-0.07	108	-3.92	10929	NA	NA	NA	NA	NA	NA	NA	NA
KLHL24_t1	-1.06	0.100275546	-0.04	160	-3.92	10930	NA	NA	NA	NA	0	139	-0.16	8702
TBC1D1_t1	-1.05	0.314134512	-0.05	134	-3.92	10931	0.04	130	3.34	6290	NA	NA	NA	NA
SPTB_t1	-1.01	0.711148684	-0.06	118	-3.92	10932	-0.18	120	-12.42	14621	-0.07	96	-3.07	10214
TRAK1_t2	1.12	9.76E-07	-0.04	139	-3.92	10933	0.03	136	2.76	6631	0.1	124	7.32	4908
DAPL1	1.1	0.182216836	-0.05	124	-3.92	10934	0.01	97	0.56	8101	-0.11	120	-7.64	12090
CEBPD	-1.53	5.16E-08	-0.06	112	-3.93	10935	-0.11	104	-5.93	12149	-0.18	110	-10.67	13142
MRPS26	-1.01	0.751857318	-0.06	111	-3.93	10936	-0.09	111	-5.35	11849	-0.07	118	-4.79	10985
HAUS8	-1.04	0.141044608	-0.04	159	-3.94	10937	-0.01	128	-0.78	9007	-0.15	121	-10.64	13130
KRT24	1.02	0.1651796	-0.07	105	-3.94	10938	-0.02	106	-0.88	9063	0	115	0.23	8477
AMELX	1.01	0.442101725	-0.06	115	-3.94	10939	-0.1	108	-5.55	11941	-0.03	104	-1.47	9432
RAB11FIP3	1.04	0.413812964	-0.06	116	-3.94	10940	-0.01	131	-0.89	9071	-0.01	128	-0.51	8927
CDK5R2	-1.09	7.93E-06	-0.06	120	-3.94	10941	-0.18	128	-14.21	15032	-0.13	138	-11.73	13466
ENAM	1.04	0.005477403	-0.05	135	-3.95	10942	0.08	148	8.3	3594	0.05	128	4.27	6306
EFNB2	-1.02	0.168111318	-0.06	120	-3.95	10943	0.02	143	1.86	7238	0.06	148	6.08	5410
ABHD1	-1.14	0.102680472	-0.09	96	-3.95	10944	-0.1	104	-5.21	11779	-0.1	88	-3.63	10450
CTNND2_t1	1.23	0.056302928	-0.04	150	-3.95	10945	NA	NA	NA	NA	NA	NA	NA	NA
SMN1	1.12	0.011248713	-0.05	126	-3.95	10946	0.1	102	5.02	5321	0.09	138	7.9	4664
INPPL1	1.05	0.218083009	-0.04	143	-3.95	10947	0.13	110	7.97	3761	-0.08	113	-4.94	11030
APBB1IP_t1	-1.53	6.81E-07	-0.08	100	-3.95	10948	NA	NA	NA	NA	-0.13	123	-9.71	12810
MAGEH1	-1.22	0.008554187	-0.05	124	-3.96	10949	-0.01	115	-0.63	8895	-0.01	120	-0.87	9118
GLB1L2	-1.01	0.43333046	-0.07	104	-3.96	10950	-0.05	105	-2.71	10276	-0.05	145	-4.94	11032
SERINC5	1.03	0.488122099	-0.05	123	-3.96	10951	-0.05	104	-2.74	10302	0.05	114	3.5	6667
SCARB2	-1	0.937448643	-0.05	132	-3.96	10952	-0.06	134	-5.26	11807	0.08	151	8	4622
RIT2	1.02	0.028363174	-0.07	108	-3.97	10953	0.02	141	2.12	7066	0	124	0.34	8408
FKBP2	-1.17	0.00030934	-0.1	88	-3.97	10954	-0.11	107	-6.44	12391	-0.11	80	-3.37	10339
LPHN3	-1.09	0.00119366	-0.05	129	-3.97	10955	-0.06	123	-4.07	11131	-0.09	141	-7.84	12177
CHST12	1.09	0.031592948	-0.04	141	-3.97	10956	0.1	129	8.02	3735	-0.02	145	-1.67	9535
AGRP	1.01	0.685266637	-0.1	88	-3.97	10957	-0.06	88	-2.21	9968	-0.04	64	-0.59	8975
EPHA1	1.07	0.421509211	-0.08	97	-3.97	10958	-0.1	120	-7.34	12810	0.06	100	2.93	6972
CXorf26	-1.1	0.003119898	-0.05	125	-3.98	10959	0.01	112	0.79	7948	-0.1	121	-7.06	11868
TMEM55B	1.02	0.399745522	-0.05	128	-3.98	10960	-0.12	134	-9.69	13769	-0.09	153	-9.7	12807
TFAP2D	1	0.861946131	-0.1	91	-3.98	10961	0.07	117	4.45	5633	0.02	92	0.91	8071
TSC22D1	-1.11	0.169789039	-0.05	132	-3.99	10962	-0.05	139	-4.49	11379	-0.08	162	-9.15	12642
KRT3	-1.07	0.010371099	-0.08	100	-3.99	10963	-0.11	123	-7.7	12985	-0.2	120	-14.14	14146
MEF2C	-1.3	4.55E-05	-0.05	137	-3.99	10964	-0.01	127	-0.68	8920	-0.12	139	-10.75	13171
SHD	-1.19	0.098167689	-0.12	84	-4	10965	-0.27	95	-12.05	14520	-0.03	72	-0.65	9010
VAV3_t1	-1.03	0.031464879	-0.04	143	-4	10966	NA	NA	NA	NA	-0.01	132	-0.51	8920
NYX	-1.09	0.0040265	-0.07	104	-4	10967	-0.07	113	-4.13	11166	-0.21	100	-10.28	13017
IGSF6	-1.54	1.56E-10	-0.08	100	-4	10968	-0.01	100	-0.61	8880	-0.27	100	-13.7	14026
KLHL4_t1	-1.04	0.002605823	-0.06	114	-4.01	10969	NA	NA	NA	NA	-0.02	94	-0.71	9035
ASNSD1	-1.12	0.033485142	-0.08	103	-4.01	10970	-0.09	111	-5.5	11916	-0.03	116	-1.75	9592
RASEF	1.15	0.066016514	-0.04	141	-4.01	10971	0.03	128	2.68	6695	-0.03	130	-2.28	9833
KRTAP10-10	-1.12	5.06E-10	-0.12	84	-4.01	10972	-0.2	109	-12.09	14535	-0.07	111	-4.41	10804
TEX264	1	0.966688715	-0.05	129	-4.01	10973	-0.13	118	-8.85	13450	0.01	100	0.75	8164
RRNAD1	1.14	0.001098497	-0.08	103	-4.01	10974	-0.07	119	-4.73	11501	-0.03	99	-1.3	9343
RNF167	-1.08	0.000116152	-0.06	116	-4.02	10975	-0.2	124	-15.1	15220	-0.07	129	-5.23	11131
SMOC1	-1.45	1.61E-05	-0.05	125	-4.02	10976	-0.17	122	-12.04	14510	-0.12	140	-10.61	13119
IL27RA	-1.11	0.048401827	-0.06	113	-4.02	10977	0.02	135	1.61	7415	-0.18	130	-14.73	14305
RGS17	1.04	0.075564124	-0.06	120	-4.03	10978	-0.03	128	-1.99	9813	0	124	-0.11	8682
PET112	-1.06	0.136801083	-0.08	101	-4.03	10979	-0.11	120	-7.65	12958	-0.07	108	-3.98	10628
XKR6	-1.06	8.89E-05	-0.04	141	-4.03	10980	-0.03	120	-2.37	10061	-0.17	130	-13.59	13997
EIF4G1	-1.14	1.84E-05	-0.08	100	-4.03	10981	-0.15	112	-9.44	13673	-0.19	91	-7.71	12122
GLTSCR2	-1.11	0.070387407	-0.15	77	-4.03	10982	-0.06	96	-2.62	10233	-0.17	78	-4.66	10917
TH	1	0.90717386	-0.09	94	-4.03	10983	-0.01	110	-0.35	8719	0	127	-0.28	8782
KPNA6	-1.06	0.0062291	-0.03	174	-4.03	10984	-0.03	133	-2.19	9955	-0.08	137	-6.69	11731
MICALCL	-1.16	0.000721751	-0.06	120	-4.04	10985	-0.1	113	-6.5	12415	-0.13	120	-9.06	12612
CFH_t1	-1.33	0.021471347	-0.07	105	-4.04	10986	-0.19	119	-13.17	14787	-0.02	103	-1.1	9249
FRMD7	1	0.958089736	-0.05	124	-4.04	10987	-0.09	130	-6.99	12646	-0.02	122	-1.19	9291
SCAF4	-1.07	0.004360768	-0.07	108	-4.04	10988	-0.05	140	-4.59	11438	-0.1	115	-6.72	11744
ARHGEF6	-1.05	0.000665552	-0.05	127	-4.05	10989	0.03	140	3.13	6423	-0.22	138	-19.38	15232
HLA-DOB	-1.04	0.209283482	-0.07	105	-4.05	10990	-0.07	131	-5.63	11994	-0.17	123	-12.39	13666
SNRPN	-1.31	0.000636545	-0.08	100	-4.05	10991	-0.02	89	-0.66	8917	-0.17	104	-9.19	12651
TMEM59	-1.2	0.000265062	-0.05	129	-4.05	10992	-0.22	123	-16.32	15476	-0.08	129	-6.64	11714
GLIS1	-1.03	0.070241606	-0.08	104	-4.05	10993	-0.14	136	-12.33	14595	-0.11	102	-5.86	11388
KIRREL3	-1	0.915590007	-0.05	129	-4.06	10994	0.02	121	1.47	7506	-0.01	124	-0.65	9008
BNIP3	-1.08	0.327278966	-0.05	125	-4.06	10995	-0.09	119	-6.54	12432	0.02	142	1.92	7501
TMED6	-1.15	0.130154741	-0.08	99	-4.07	10996	-0.11	102	-5.83	12103	-0.16	104	-8.79	12523
MMP10	1.2	0.000327726	-0.07	111	-4.07	10997	0.08	119	5.62	4994	0.12	167	14.58	2435
SLC22A17	-1.04	0.078743503	-0.08	102	-4.07	10998	-0.1	105	-5.35	11844	-0.12	137	-10.39	13051
SPO11	-1.01	0.097449341	-0.06	121	-4.08	10999	-0.04	132	-3.26	10640	0.03	115	1.83	7552
SLC5A9	-1.06	0.113510535	-0.05	126	-4.08	11000	-0.05	130	-4.39	11309	0.01	123	0.87	8099
KLK9	-1.05	0.002904047	-0.09	98	-4.08	11001	-0.13	112	-8.34	13260	-0.12	111	-7.48	12029
GTPBP8	-1.11	0.002834044	-0.05	128	-4.08	11002	-0.14	93	-6.08	12229	-0.11	113	-6.76	11762
RPL3L	1.02	0.309343306	-0.08	100	-4.08	11003	-0.05	103	-2.42	10097	-0.03	113	-1.79	9605
OCM2	1.01	0.513135239	-0.07	108	-4.09	11004	0.01	100	0.3	8264	-0.05	101	-2.54	9972
RRH	-1	0.79145595	-0.08	104	-4.09	11005	-0.07	127	-5.13	11733	-0.05	124	-3.7	10485
RASGRP1_t1	-1.01	0.570223666	-0.06	124	-4.09	11006	NA	NA	NA	NA	NA	NA	NA	NA
ESAM	-1.14	0.002961198	-0.08	103	-4.09	11007	-0.05	155	-5.64	11996	-0.06	118	-4.18	10705
AASDH_t1	-1.08	0.024880483	-0.05	131	-4.09	11008	-0.06	109	-3.35	10710	NA	NA	NA	NA
MTO1	-1.15	0.00026282	-0.06	120	-4.09	11009	-0.13	104	-6.98	12638	0.02	133	1.39	7808
NSUN7	1	0.90530775	-0.07	111	-4.09	11010	0.09	125	7.03	4230	-0.06	119	-4.19	10712
POU5F1	1.09	0.001357308	-0.08	100	-4.09	11011	0.07	118	4.96	5368	0.06	95	2.67	7096
SDCCAG3	1.01	0.776230332	-0.06	117	-4.1	11012	-0.03	124	-2.57	10194	0	110	-0.28	8781
FRY	-1.1	0.143648278	-0.06	117	-4.1	11013	0.01	136	0.6	8076	-0.11	149	-10.47	13081
CCL27	1	0.836886741	-0.13	81	-4.11	11014	-0.1	87	-3.66	10918	0	88	0.05	8589
PRB3	-1.14	0.00029974	-0.14	80	-4.11	11015	-0.2	99	-9.91	13845	-0.13	89	-5.16	11099
VIT	-1.08	7.76E-05	-0.08	104	-4.11	11016	-0.17	131	-13.97	14989	-0.16	105	-8.87	12550
MAN2C1	-1.09	0.004872199	-0.09	98	-4.11	11017	-0.09	100	-4.7	11488	-0.08	89	-3.17	10250
SLC25A13	-1.12	0.047876418	-0.06	118	-4.11	11018	-0.1	124	-7.5	12874	-0.06	132	-5.06	11069
LYNX1_t2	-1.05	0.054639061	-0.1	91	-4.11	11019	-0.14	104	-7.37	12825	-0.14	93	-5.83	11381
CRX	-1.01	0.241089474	-0.06	120	-4.13	11020	-0.09	130	-7.12	12702	-0.05	120	-3.73	10501
PCBD2_t1	-1.02	0.449191612	-0.05	132	-4.13	11021	NA	NA	NA	NA	0.05	122	3.84	6505
ADD1_t3	-1.01	0.671542255	-0.06	124	-4.13	11022	-0.02	124	-1.58	9547	0.01	135	0.76	8157
DOK4_t1	-1.01	0.849228337	-0.05	137	-4.13	11023	NA	NA	NA	NA	-0.03	120	-2.42	9900
KLC2	-1.05	0.00074676	-0.07	108	-4.13	11024	-0.17	131	-13.41	14850	-0.11	120	-7.59	12062
WNT3	-1.04	0.494422277	-0.09	95	-4.13	11025	-0.14	118	-9.22	13599	-0.03	134	-2.37	9877
BCL2L14	-1.05	0.475576964	-0.05	126	-4.13	11026	-0.05	115	-2.95	10447	-0.15	119	-10.31	13029
FAM24A	-1.02	0.145889141	-0.07	109	-4.13	11027	-0.11	115	-7.36	12820	0.01	104	0.54	8294
SIAH3	1	0.951560873	-0.04	144	-4.14	11028	-0.07	131	-5.64	11995	0.06	125	4.69	6075
WDR49	1.01	0.093795733	-0.08	104	-4.14	11029	0.13	107	7.65	3904	0.03	126	2.03	7430
ERVFRD-1	-1.04	0.048878518	-0.07	113	-4.14	11030	-0.21	115	-13.84	14953	-0.05	135	-3.94	10610

GPC2	-1.02	0.522136609	-0.05	133	-4.14	11031	-0.08	135	-6.84	12582	-0.04	123	-2.72	10045
VPS13D_t1	-1.09	0.004649062	-0.06	125	-4.15	11032	-0.04	120	-2.57	10200	NA	NA	NA	NA
DMKN	-1.73	2.27E-05	-0.07	107	-4.15	11033	-0.04	116	-2.65	10251	-0.21	126	-16.27	14639
NCF4	-1.29	1.16E-08	-0.08	101	-4.15	11034	-0.01	106	-0.81	9021	-0.27	101	-13.9	14079
VASN	-1.13	0.031792997	-0.09	97	-4.15	11035	-0.02	120	-1.34	9390	-0.09	99	-4.65	10913
NAPA	-1.05	0.087529454	-0.07	109	-4.16	11036	-0.12	108	-6.77	12541	-0.16	107	-9.39	12714
NBPF9	-1.18	6.53E-05	-0.12	85	-4.16	11037	-0.16	64	-2.3	10021	-0.14	68	-2.5	9952
TCF25	-1.24	1.97E-09	-0.09	98	-4.16	11038	-0.12	123	-9.06	13537	-0.17	88	-6.3	11570
AAAS	-1.01	0.568770396	-0.14	80	-4.17	11039	-0.1	100	-5.05	11682	-0.16	129	-12.61	13733
LTC4S	-1.26	3.30E-10	-0.17	75	-4.17	11040	-0.1	88	-3.63	10898	-0.29	84	-9.8	12852
HIPK2	1.04	0.451143803	-0.05	129	-4.17	11041	-0.01	148	-0.87	9057	0.06	143	5.16	5850
CASP7	-1.22	2.19E-06	-0.05	130	-4.17	11042	-0.2	127	-15.17	15243	-0.07	140	-6.13	11490
ARHGEF2	-1.04	0.243016466	-0.06	117	-4.17	11043	-0.02	116	-1.19	9282	-0.1	110	-5.95	11421
OTUD6A	1.01	0.649657837	-0.05	139	-4.17	11044	-0.11	131	-8.68	13386	-0.03	145	-2.61	10001
SLC12A1_t1	1.01	0.124307148	-0.04	152	-4.18	11045	0.01	139	0.5	8138	NA	NA	NA	NA
TEKT1	-1.04	0.000154905	-0.16	76	-4.18	11046	-0.03	95	-1.18	9275	-0.22	102	-11.46	13393
ASB14	1.03	0.001817158	-0.05	130	-4.19	11047	0.09	156	9.12	3221	0.01	123	0.97	8037
CALML6	-1.01	0.736865852	-0.14	79	-4.19	11048	-0.12	120	-8.72	13401	-0.02	84	-0.73	9047
RPGRIP1	-1.18	1.54E-05	-0.09	98	-4.2	11049	0.05	88	1.98	7160	-0.25	92	-10.68	13146
TRUB2	-1	0.915841673	-0.05	136	-4.2	11050	-0.03	115	-1.77	9668	-0.08	122	-5.65	11298
CCDC70	-1.01	0.142169238	-0.09	99	-4.2	11051	-0.04	127	-3.46	10776	-0.23	98	-10.81	13185
MSH3	-1.12	0.007737859	-0.04	145	-4.2	11052	-0.18	131	-14.24	15042	0.08	120	5.29	5781
GAPT_t1	-1.09	1.51E-11	-0.04	145	-4.2	11053	NA	NA	NA	NA		-0.23	137	-19.84	15303
CAB39	-1.11	0.004673729	-0.06	120	-4.21	11054	-0.02	127	-1.56	9523	-0.02	141	-1.66	9531
COL9A2	1.04	0.114416246	-0.07	109	-4.21	11055	-0.02	114	-1	9145	-0.05	104	-2.5	9955
NPFF_t1	1.02	0.694030424	-0.08	100	-4.21	11056	NA	NA	NA	NA		-0.07	95	-3.34	10333
PSD	-1.05	0.034077595	-0.07	107	-4.21	11057	-0.1	110	-5.97	12164	-0.08	145	-7.59	12063
STAT5A_t1	-1.03	0.324914915	-0.05	131	-4.21	11058	0.04	135	3.71	6053	NA	NA	NA	NA
EMP3	-1.65	7.58E-17	-0.23	68	-4.21	11059	-0.09	90	-3.65	10906	-0.39	68	-6.99	11842
DLGAP2	-1.01	0.481577993	-0.05	133	-4.21	11060	-0.18	148	-17.95	15727	-0.01	148	-0.9	9141
CCL17	-1.04	0.255535179	-0.07	111	-4.21	11061	0.03	113	1.86	7240	-0.2	88	-7.43	12007
FAM197Y2P	-1.03	0.061386951	-0.07	111	-4.21	11062	-0.2	113	-12.43	14622	-0.04	119	-2.8	10089
SLCO2A1	1.07	0.299981568	-0.06	116	-4.22	11063	0.04	142	3.67	6077	-0.12	141	-11.08	13278
FMNL2	-1.12	0.008177242	-0.05	131	-4.22	11064	0.05	142	4.36	5680	-0.11	135	-9.35	12695
EVI5L	-1.01	0.41903442	-0.06	122	-4.22	11065	-0.05	142	-4.32	11275	-0.09	123	-6.8	11773
MEF2A	-1.09	0.001444922	-0.04	147	-4.22	11066	0.02	129	1.65	7385	0.05	156	5.82	5532
CST8	1.01	0.284215885	-0.06	126	-4.22	11067	0.05	93	2.26	6983	0.05	97	2.24	7324
COL25A1_t1	-1.01	0.22290687	-0.08	101	-4.23	11068	-0.19	104	-10.46	14051	-0.09	104	-4.68	10925
STX7	-1.12	0.002394684	-0.04	152	-4.23	11069	0.09	130	6.96	4266	-0.15	131	-11.89	13511
GRAMD4	-1.07	0.027587205	-0.06	123	-4.23	11070	-0.24	143	-22.02	16173	-0.04	132	-3.6	10436
TRIM63	1.03	0.444326301	-0.05	135	-4.23	11071	0.06	112	3.53	6162	-0.1	119	-7.03	11856
NXNL2	1	0.757112005	-0.05	128	-4.23	11072	0.05	145	4.35	5690	-0.02	133	-1.59	9493
CEP164_t1	1.02	0.277462689	-0.07	114	-4.24	11073	NA	NA	NA	NA		-0.12	129	-9.41	12721
GATAD2A	-1.04	0.143362298	-0.06	124	-4.24	11074	0.06	127	4.71	5496	-0.13	118	-8.95	12576
MYO3A	-1	0.951125403	-0.08	102	-4.24	11075	0.01	115	0.49	8147	-0.02	119	-1.09	9241
TMEM30A	-1.22	1.97E-06	-0.04	147	-4.24	11076	-0.09	130	-7.41	12835	-0.05	140	-4.74	10957
FRAT1	-1	0.921201625	-0.06	127	-4.25	11077	-0.16	158	-17.38	15657	-0.02	110	-1.01	9206
GPR17	1.05	6.53E-05	-0.06	116	-4.25	11078	0.01	129	0.86	7897	0	123	-0.15	8700
ZNF483	-1.01	0.49120424	-0.06	123	-4.25	11079	-0.01	160	-0.61	8884	-0.09	122	-6.49	11636
CAMK4	-1.05	3.98E-05	-0.06	124	-4.26	11080	-0.09	126	-6.64	12484	-0.18	124	-13.57	13990
SMR3A	-1	0.990727772	-0.08	104	-4.26	11081	-0.05	123	-3.69	10929	-0.14	101	-6.98	11836
KLHL22	-1	0.880179068	-0.08	103	-4.26	11082	0	104	0.07	8418	-0.1	100	-5.03	11059
UBAC2	1.02	0.505145927	-0.07	114	-4.26	11083	-0.02	134	-1.91	9754	-0.11	125	-8.34	12377
NCAN	-1.04	0.000440528	-0.06	120	-4.26	11084	-0.18	125	-13.4	14849	-0.07	129	-5.18	11113
SHPRH_t1	-1.08	0.045261217	-0.06	120	-4.26	11085	-0.02	152	-1.75	9653	0.03	134	2.39	7236
MAX_t1	-1.07	0.000312182	-0.06	127	-4.26	11086	-0.07	131	-5.43	11884	-0.2	106	-11.15	13295
CSDA	-1.5	5.61E-06	-0.08	104	-4.27	11087	0.08	120	5.49	5065	-0.19	107	-10.99	13255
TST	-1.21	0.015195675	-0.08	106	-4.27	11088	-0.2	112	-12.49	14636	-0.1	109	-5.76	11340
HORMAD2	-1.02	0.435170339	-0.05	128	-4.27	11089	-0.23	134	-19.19	15889	-0.02	109	-1.04	9223
SLC34A3	-1.33	4.87E-10	-0.11	88	-4.27	11090	-0.16	113	-9.92	13853	-0.19	83	-6.22	11534
COX4I1	-1.05	0.181426059	-0.06	117	-4.27	11091	-0.09	103	-4.99	11650	-0.04	115	-2.78	10076
EIF2C3	-1.12	0.006109805	-0.06	126	-4.28	11092	-0.03	119	-1.75	9651	-0.04	112	-2.35	9868
HSDL2	-1.12	0.001579054	-0.05	144	-4.28	11093	-0.04	114	-2.63	10235	0.01	121	0.51	8313
CLPB	-1.03	0.511178591	-0.09	99	-4.28	11094	-0.05	115	-3.48	10790	-0.06	104	-3.22	10279
UBASH3B	-1.01	0.393369045	-0.05	132	-4.28	11095	0.06	132	4.68	5505	-0.09	149	-8.93	12573
PLA2G12A	-1.21	2.25E-05	-0.05	134	-4.28	11096	-0.17	140	-15.7	15344	-0.04	153	-4.37	10781
ZFP91	-1	0.939110395	-0.05	132	-4.28	11097	0.02	141	1.37	7563	0.13	144	11.89	3239
TRH	1.01	0.646605878	-0.06	116	-4.28	11098	0.01	123	0.8	7943	-0.03	126	-2.23	9808
STX12	-1.19	4.53E-05	-0.05	130	-4.28	11099	-0.06	132	-5.29	11817	-0.06	158	-6.17	11507
ETFB	-1.21	0.000442046	-0.09	96	-4.28	11100	-0.13	97	-6.06	12221	-0.13	90	-5.13	11093
CPNE2	-1.16	0.000552415	-0.07	114	-4.28	11101	-0.02	116	-1.55	9518	-0.12	104	-6.34	11591
CEP95	1.01	0.919472334	-0.06	120	-4.29	11102	0.08	105	4.18	5793	-0.01	95	-0.45	8882
CAPN7	-1	0.951470056	-0.06	125	-4.29	11103	0.01	121	0.82	7923	0.12	152	11.99	3199
ADCY10	-1.08	0.137791343	-0.05	130	-4.31	11104	-0.07	122	-4.86	11573	-0.05	104	-2.94	10156
PMM2	-1.02	0.608085453	-0.05	129	-4.31	11105	0.14	125	10.32	2750	-0.11	131	-8.59	12455
POU6F2	-1.05	0.000806769	-0.13	84	-4.31	11106	-0.07	111	-4.13	11165	-0.14	78	-3.81	10539
CCDC94	-1.03	0.23030672	-0.06	117	-4.31	11107	-0.18	125	-13.84	14955	-0.17	100	-8.26	12348
UCP3_t1	1.02	0.496992942	-0.05	129	-4.31	11108	0.02	119	1.45	7524	-0.07	151	-6.57	11680
G3BP2	-1.07	0.016929331	-0.04	147	-4.31	11109	0.02	145	2.28	6968	-0.05	151	-4.79	10980
GDF9	1.03	0.064886908	-0.06	127	-4.32	11110	0.03	98	1.23	7657	0.19	108	10.77	3583
ADCYAP1	-1.05	0.005440788	-0.06	128	-4.32	11111	-0.02	131	-1.28	9350	0.01	130	0.96	8041
PYCARD	-1.3	0.000823435	-0.08	104	-4.33	11112	0.04	141	3.31	6311	-0.18	80	-5.3	11159
SELO	-1.24	6.20E-06	-0.11	91	-4.33	11113	-0.23	138	-20.34	16024	-0.16	100	-7.97	12226
MACROD1	-1.27	5.16E-09	-0.17	76	-4.34	11114	-0.18	104	-9.46	13685	-0.18	80	-5.47	11230
TMSB4X	-1.3	0.00084565	-0.06	118	-4.34	11115	0.08	145	7.73	3867	-0.21	120	-14.61	14271
STARD10	-1.2	0.008340196	-0.14	80	-4.34	11116	-0.14	104	-7.57	12909	-0.08	120	-5.3	11157
TUSC5_t1	-1	0.833686862	-0.06	128	-4.34	11117	NA	NA	NA	NA	NA	NA	NA	NA
GCNT2	-1.07	0.021616063	-0.05	132	-4.34	11118	0.06	127	4.62	5539	0.02	142	1.6	7684
STAP2	-1.04	0.466391863	-0.13	84	-4.35	11119	-0.16	109	-9.59	13730	-0.06	68	-1.14	9266
PRCD	-1.02	0.263483239	-0.07	112	-4.35	11120	-0.12	119	-8.58	13352	-0.04	117	-2.85	10110
ACTL7A	-1.26	8.45E-14	-0.17	76	-4.35	11121	-0.22	136	-18.85	15847	-0.3	126	-22.69	15742
MASP2_t1	-1.99	9.34E-11	-0.2	72	-4.35	11122	-0.29	100	-14.63	15131	-0.21	93	-8.94	12574
UBQLN3	-1.01	0.670939386	-0.07	109	-4.35	11123	-0.14	96	-6.5	12414	-0.05	108	-2.67	10025
CD151	-1.16	0.000163079	-0.09	100	-4.35	11124	-0.02	136	-1.36	9405	-0.16	120	-10.96	13242
IL9R	-1	0.769060887	-0.07	115	-4.35	11125	-0.04	108	-2.57	10191	-0.09	120	-5.98	11436
PLEKHB1	1.04	0.018542067	-0.06	127	-4.36	11126	0.06	144	5.96	4808	0.05	120	3.36	6742
CPT1A	-1.22	4.81E-05	-0.09	100	-4.37	11127	-0.18	106	-10.24	13977	-0.09	128	-7.32	11967
CYSLTR1	-1.05	0.004500507	-0.07	113	-4.37	11128	0.01	95	0.66	8029	-0.24	106	-13.33	13929
CRISP3	1.03	0.059380499	-0.06	121	-4.37	11129	0.05	124	3.39	6255	-0.09	129	-7.1	11884
EDF1	-1.1	0.001712051	-0.17	75	-4.37	11130	-0.01	104	-0.45	8783	-0.22	88	-8.43	12411
CYP24A1	1.01	0.657370547	-0.04	156	-4.38	11131	-0.15	141	-13.83	14951	-0.02	138	-1.52	9453
BARHL2	-1.02	0.017027791	-0.1	92	-4.38	11132	0.01	96	0.61	8063	-0.11	120	-7.91	12200
BEND2	1	0.669231404	-0.06	122	-4.38	11133	0.08	120	5.44	5089	0.03	128	2.3	7293
AGAP3_t2	-1.31	1.17E-06	-0.08	104	-4.39	11134	-0.09	120	-6.5	12411	-0.15	142	-14.08	14135
PPIE	-1.15	7.03E-07	-0.09	99	-4.39	11135	-0.12	98	-5.64	11998	-0.16	95	-7.28	11954
REC8	-1.24	1.05E-07	-0.09	98	-4.39	11136	-0.04	104	-2.13	9922	-0.26	111	-16.15	14619

MORN1	1.01	0.532333141	-0.07	109	-4.39	11137	-0.12	95	-5.5	11915	0.06	89	2.37	7253
POLD2	1	0.96343734	-0.08	103	-4.39	11138	-0.08	104	-4.34	11282	-0.04	104	-2.08	9748
SIGLEC8	-1.08	4.71E-05	-0.05	133	-4.39	11139	-0.12	131	-9.61	13740	-0.21	129	-16.52	14690
VEZT	-1.09	0.114638063	-0.05	140	-4.39	11140	0.11	131	8.85	3352	-0.06	138	-5.06	11068
FAM212A	-1.16	0.000971427	-0.13	83	-4.39	11141	-0.17	93	-7.32	12801	-0.25	88	-9.62	12784
FIS1_t1	-1.05	0.264282282	-0.1	96	-4.4	11142	-0.17	125	-12.54	14653	NA	NA	NA	NA
CMTM5	-1.02	0.088144751	-0.08	108	-4.4	11143	-0.07	104	-3.56	10847	-0.08	88	-3.02	10186
CMYA5_t1	-1.2	0.001100809	-0.06	120	-4.4	11144	NA	NA	NA	NA	-0.02	142	-1.83	9629
METRNL	-2.31	3.32E-17	-0.12	87	-4.4	11145	-0.04	140	-3.62	10893	-0.28	88	-10.71	13154
DPPA5	1.04	0.000790202	-0.08	107	-4.4	11146	-0.07	107	-3.79	10973	0.08	101	4.02	6410
WSCD2	-1	0.824294374	-0.07	112	-4.4	11147	-0.04	148	-3.87	11014	-0.12	134	-10.15	12974
PARD6G	-1.1	0.0696579	-0.05	138	-4.4	11148	0.18	143	16.47	944	-0.13	121	-9.03	12597
FGF7	1.04	0.005956616	-0.05	134	-4.4	11149	0.13	126	9.73	2965	0.03	132	2.59	7136
MRPS22	-1.06	0.188428543	-0.09	97	-4.4	11150	-0.12	89	-4.8	11539	-0.11	98	-5.23	11130
IGSF9	-1.83	6.95E-10	-0.12	88	-4.4	11151	-0.16	92	-6.93	12619	-0.22	100	-10.95	13236
Mar-09	-1.04	0.31930642	-0.06	128	-4.4	11152	-0.09	128	-6.91	12611	-0.09	131	-7.65	12091
PKHD1_t2	-1.08	0.000121084	-0.05	135	-4.41	11153	0.01	129	0.46	8162	-0.16	150	-15.74	14529
UBQLNL	-1.09	0.078297939	-0.06	127	-4.41	11154	-0.03	140	-2.89	10403	-0.1	134	-8.34	12378
HMG20B	1.01	0.680372589	-0.08	107	-4.41	11155	-0.11	123	-7.91	13073	-0.07	101	-3.54	10414
NDUFB1	-1.12	0.008457676	-0.12	88	-4.41	11156	-0.25	110	-14.74	15154	-0.04	76	-0.98	9193
DGKG	-1.11	0.006921822	-0.06	130	-4.41	11157	-0.08	141	-7.03	12668	-0.12	149	-11.44	13383
DACT2	-1.96	1.81E-06	-0.08	107	-4.41	11158	-0.09	120	-6.3	12326	-0.08	107	-4.77	10970
SNAP25	1.08	0.068264429	-0.07	115	-4.41	11159	-0.02	139	-1.37	9408	-0.01	152	-1.1	9244
TESC	1.01	0.933392726	-0.13	83	-4.41	11160	0	93	0.05	8432	-0.14	120	-10.09	12955
GPRC6A	-1.02	0.155250588	-0.12	88	-4.42	11161	0.05	136	4.67	5510	-0.09	106	-5.3	11156
PSMB8	-1.03	0.622009215	-0.06	128	-4.42	11162	-0.03	92	-1.45	9459	-0.13	91	-5.19	11114
LRIT1	-1.02	0.156912617	-0.08	108	-4.42	11163	-0.13	159	-14.63	15132	-0.04	114	-2.8	10086
TUBGCP2	-1.04	0.147339822	-0.08	106	-4.42	11164	-0.19	126	-14.5	15101	-0.07	142	-6.2	11523
CBX7	-1.04	0.519281095	-0.05	137	-4.42	11165	-0.06	141	-5.64	12000	-0.06	148	-6.06	11461
CSH1	-1.15	2.42E-06	-0.13	83	-4.42	11166	-0.22	120	-15.56	15314	-0.13	102	-6.86	11797
LSAMP	1.07	0.035298321	-0.05	145	-4.43	11167	-0.04	135	-3.46	10773	0.06	126	4.75	6055
PRAMEF2	-1.05	0.029597958	-0.07	112	-4.43	11168	-0.24	107	-13.45	14861	-0.1	109	-6.14	11495
TAGAP_t1	-1.13	9.01E-05	-0.09	101	-4.43	11169	-0.04	118	-2.45	10120	-0.22	131	-17.78	14939
GJB6	1.05	0.008155742	-0.05	136	-4.43	11170	-0.07	108	-4.28	11257	-0.08	130	-6.1	11481
JAK1	-1.24	8.92E-09	-0.05	132	-4.43	11171	-0.04	132	-2.9	10410	-0.08	120	-5.31	11162
SCLY	-1.05	0.320674177	-0.05	147	-4.44	11172	-0.02	122	-1.37	9413	-0.06	113	-3.54	10411
NLRP2	1.03	0.450580889	-0.08	104	-4.44	11173	0.07	118	4.86	5422	-0.05	109	-3.21	10275
ZNF684	-1.2	0.000816601	-0.07	110	-4.44	11174	-0.11	117	-7.2	12738	-0.07	147	-6.97	11835
IREB2	-1.01	0.782944774	-0.05	134	-4.45	11175	-0.06	142	-5.09	11713	0.01	139	1.32	7844
SLC26A9	1.06	0.174819748	-0.05	140	-4.45	11176	-0.02	124	-1.12	9237	0.06	123	4.24	6318
GALNTL4	-1.18	0.011338742	-0.07	113	-4.45	11177	-0.07	104	-3.94	11065	-0.06	111	-3.86	10571
RS1_t1	-1.01	0.567334824	-0.04	154	-4.46	11178	NA	NA	NA	NA	NA	NA	NA	NA
IL19	-1	0.915068357	-0.07	118	-4.46	11179	-0.15	108	-8.94	13480	0	145	0.03	8602
PKDCC	-1.15	0.065555391	-0.08	107	-4.46	11180	0	140	0.45	8174	-0.21	147	-20.01	15331
NDE1_t1	-1.01	0.57993778	-0.04	154	-4.46	11181	NA	NA	NA	NA	NA	NA	NA	NA
NFAM1	1.01	0.120783278	-0.05	137	-4.46	11182	0.1	120	6.7	4401	0	123	-0.01	8626
ST8SIA1	-1.07	0.052549784	-0.05	142	-4.46	11183	0.02	151	2.14	7052	-0.15	143	-13.92	14085
GPX4	-1.06	0.176583326	-0.09	100	-4.46	11184	-0.08	106	-4.51	11393	-0.02	120	-1.27	9329
NIPSNAP3B	-1.1	0.002598246	-0.05	132	-4.46	11185	-0.05	124	-3.48	10788	-0.06	125	-4.38	10788
LRCH3	1.01	0.573678786	-0.09	98	-4.46	11186	-0.12	123	-8.59	13358	-0.1	133	-8.25	12341
OR2L13	-1.01	0.571711651	-0.08	105	-4.46	11187	-0.03	120	-2.08	9875	-0.06	137	-5.05	11064
OPN1LW	-1.2	3.89E-11	-0.16	78	-4.46	11188	-0.25	92	-10.32	14011	-0.13	99	-6.33	11585
NUBP2	-1.01	0.855015583	-0.09	102	-4.47	11189	-0.07	95	-3.11	10549	-0.04	107	-2.43	9912
MMP14	-1.06	0.190696003	-0.06	120	-4.47	11190	0.11	125	8.04	3718	-0.16	120	-11.14	13292
ZKSCAN2	1.06	4.27E-06	-0.05	140	-4.47	11191	0.01	118	0.61	8057	0.1	132	7.95	4642
TDRD6	1.01	0.352155509	-0.05	137	-4.47	11192	-0.1	126	-7.56	12900	-0.04	133	-3.51	10400
OVGP1	-1.19	0.011097622	-0.12	87	-4.47	11193	-0.01	89	-0.48	8808	-0.12	94	-5.41	11203
ROBO3	-1.22	0.007241402	-0.09	100	-4.47	11194	0.02	90	0.88	7880	-0.09	100	-4.37	10782
PCYT2	-1	0.94540737	-0.07	115	-4.48	11195	-0.11	129	-8.32	13254	-0.06	120	-4.29	10749
CACNG7	-1	0.929524988	-0.07	112	-4.48	11196	-0.1	120	-6.71	12518	-0.02	127	-1.6	9497
CWC22	-1.11	0.005216741	-0.05	142	-4.48	11197	0.01	106	0.53	8117	-0.02	114	-1.46	9426
NR1D2	-1.06	0.169570236	-0.05	138	-4.48	11198	-0.08	125	-5.76	12066	0.12	132	9.57	4034
KCNMB2	-1.02	0.074137241	-0.06	124	-4.48	11199	-0.04	124	-3.21	10606	-0.08	135	-6.98	11840
GDPD4	1.04	0.034217701	-0.08	105	-4.48	11200	-0.09	135	-7.28	12775	0.02	132	1.91	7506
PMEL	-1.02	0.684086579	-0.09	99	-4.48	11201	-0.15	104	-8.24	13208	-0.06	84	-2.09	9750
LGALS7B	-1.05	0.185928613	-0.13	84	-4.48	11202	-0.12	53	-0.35	8718	-0.1	76	-2.5	9956
OSM	-1.65	2.39E-08	-0.06	128	-4.49	11203	-0.03	124	-2.28	10012	-0.19	126	-14.49	14238
PPRC1	-1.17	0.001888425	-0.07	112	-4.49	11204	0.01	117	0.91	7868	-0.15	140	-13.2	13895
CEP85L_t1	-1.06	0.002031413	-0.05	135	-4.49	11205	0.07	147	6.44	4545	-0.07	147	-7.18	11912
ROM1	-1.2	9.54E-05	-0.12	88	-4.49	11206	-0.11	115	-7.31	12792	-0.16	96	-7.17	11907
KDM5D	-1.24	3.54E-05	-0.06	123	-4.49	11207	-0.06	127	-4.4	11325	-0.15	123	-10.88	13210
GYS1	1.02	0.474109794	-0.08	108	-4.5	11208	0	145	-0.12	8565	-0.1	120	-7.25	11941
DSCR6	1.12	0.011073491	-0.05	142	-4.5	11209	-0.1	127	-7.99	13110	-0.01	136	-0.79	9077
STAC3	-1.05	0.004364745	-0.08	103	-4.5	11210	-0.16	114	-10.56	14090	-0.18	104	-9.77	12836
CDK17	-1.07	0.000718493	-0.05	149	-4.5	11211	0.04	140	3.99	5913	0	152	-0.34	8812
PITHD1	-1.15	2.00E-05	-0.08	107	-4.5	11212	-0.15	118	-10.01	13887	-0.19	108	-11.31	13351
ESRRA	1.02	0.707378842	-0.07	115	-4.5	11213	-0.03	124	-2.1	9900	0.02	142	1.6	7686
FPGS	-1.16	5.37E-05	-0.09	102	-4.51	11214	-0.12	112	-7.65	12959	-0.18	124	-13.42	13961
L3HYPDH	-1.05	0.299248266	-0.07	115	-4.51	11215	-0.08	100	-4.09	11148	-0.15	107	-8.51	12428
ZNF584	-1.06	0.020554827	-0.08	110	-4.51	11216	-0.2	116	-13.13	14778	-0.1	155	-10.12	12964
CRIP2	-1.13	0.010157033	-0.08	108	-4.51	11217	0.02	121	1.42	7542	-0.14	126	-10.88	13209
CA10	-1.03	0.013471904	-0.07	114	-4.52	11218	-0.12	133	-9.8	13816	-0.02	127	-1.73	9570
CAPS	1.09	0.043721561	-0.09	99	-4.52	11219	0.06	123	4.68	5507	-0.02	104	-1.3	9346
LMO7_t1	-1.34	7.45E-06	-0.05	134	-4.52	11220	NA	NA	NA	NA	NA	NA	NA	NA
TRAF1	1.06	0.192163628	-0.06	120	-4.52	11221	0.05	128	3.96	5927	-0.05	124	-3.94	10608
ELAVL2	1.02	0.008327643	-0.04	153	-4.52	11222	0.04	115	2.36	6914	0.05	145	4.38	6255
HSFY2_t2	1.01	0.486113817	-0.07	111	-4.53	11223	-0.11	99	-5.26	11805	0.02	121	1.56	7714
SMCR8	-1.05	0.053147451	-0.04	160	-4.53	11224	-0.22	150	-21.76	16151	-0.05	148	-5.14	11094
LCE2D	-1.12	0.000240148	-0.06	126	-4.53	11225	-0.1	106	-5.83	12105	-0.12	130	-9.75	12831
PRG3	-1.08	8.53E-06	-0.15	80	-4.53	11226	-0.18	100	-8.77	13424	-0.19	80	-5.62	11280
SLC39A4	-1.04	0.654854588	-0.1	96	-4.54	11227	0	135	0.32	8258	-0.09	92	-3.97	10625
BAALC_t1	-1.08	0.292555356	-0.06	128	-4.54	11228	-0.17	118	-11.65	14412	-0.07	131	-5.81	11369
SYTL1	-1.22	6.26E-05	-0.13	84	-4.54	11229	-0.07	80	-2.09	9886	-0.25	88	-9.61	12782
HLCS	-1.03	0.461995778	-0.06	127	-4.54	11230	-0.12	140	-10.36	14023	0.04	124	3.23	6817
TMEM141	-1.23	3.32E-06	-0.11	92	-4.54	11231	-0.09	130	-7.58	12917	-0.16	120	-11.25	13326
GLTSCR1	-1.15	3.67E-06	-0.05	144	-4.55	11232	-0.12	136	-9.93	13858	-0.13	124	-9.51	12761
FGF21	1.12	0.332955657	-0.04	154	-4.55	11233	0.01	112	0.71	7992	-0.01	95	-0.53	8935
NXNL1	-1.01	0.469631313	-0.07	112	-4.55	11234	-0.12	107	-6.99	12642	-0.08	100	-3.93	10602
CDH17	1.04	0.192891463	-0.06	132	-4.55	11235	0.03	108	1.99	7154	-0.01	139	-0.49	8907
MRPL39	-1.19	3.23E-05	-0.15	80	-4.55	11236	-0.15	78	-4.22	11218	-0.15	75	-3.81	10543
SCAMP4	-1.01	0.433279964	-0.08	108	-4.55	11237	-0.01	125	-0.45	8787	-0.1	121	-7.16	11902
MAG	-1.07	8.98E-05	-0.08	110	-4.55	11238	-0.09	111	-5.56	11950	-0.09	122	-6.25	11551
ZNF668	1.01	0.801816464	-0.11	90	-4.56	11239	-0.06	150	-5.98	12175	-0.1	111	-6.17	11508
BAHD1	-1.06	0.094156426	-0.06	128	-4.56	11240	-0.11	108	-6.39	12368	-0.1	168	-11.29	13345
LIPI	1.01	0.197271047	-0.07	114	-4.56	11241	-0.06	133	-5.08	11701	-0.01	136	-0.61	8988
NUP214	1.09	0.000448602	-0.05	135	-4.56	11242	0.01	108	0.3	8267	-0.11	99	-5.34	11176

APOC2	-1.4	0.000685693	-0.08	108	-4.56	11243	-0.16	109	-9.4	13661	-0.11	120	-8.05	12247
NDST2	-1.04	0.109179556	-0.08	107	-4.56	11244	0.03	109	1.65	7393	-0.17	106	-9.43	12733
NDUFB8	-1.18	1.48E-06	-0.11	92	-4.56	11245	-0.18	104	-9.54	13713	-0.16	96	-7.51	12038
CYP2U1	-1.07	0.039123218	-0.05	145	-4.57	11246	-0.13	134	-10.57	14098	-0.03	167	-3.16	10249
SH2D4A	-1.06	0.340620347	-0.05	149	-4.57	11247	-0.12	128	-9.49	13697	-0.04	129	-3.27	10298
PPP2R5B	1.01	0.588823553	-0.06	127	-4.57	11248	0.05	153	4.79	5449	-0.07	112	-4.27	10744
SLC2A10	-1.02	0.329267289	-0.06	122	-4.57	11249	-0.15	120	-10.67	14121	-0.05	141	-4.96	11037
WDR93	1	0.852342676	-0.14	83	-4.58	11250	-0.11	119	-7.31	12795	-0.07	120	-5.08	11076
BAX_t2	-1.28	3.88E-07	-0.08	107	-4.58	11251	-0.13	95	-5.74	12051	-0.14	113	-8.76	12513
HERC1	1.01	0.858780953	-0.07	116	-4.58	11252	-0.02	120	-1.22	9305	0.09	126	6.76	5127
PARP8	-1.32	4.12E-06	-0.05	141	-4.58	11253	0.07	135	6.33	4598	-0.11	137	-9.24	12667
SUSD1	-1.27	1.17E-07	-0.06	131	-4.58	11254	0.01	147	0.61	8058	-0.23	120	-15.84	14547
CTC1	-1.14	1.58E-06	-0.05	137	-4.58	11255	-0.08	129	-6.04	12205	-0.17	133	-14.47	14230
PFDN5_t1	-1.11	0.001062859	-0.15	81	-4.59	11256	NA	NA	NA	NA	-0.27	100	-13.7	14025
DLGAP1	1.01	0.735242866	-0.06	131	-4.59	11257	-0.04	135	-3.51	10804	-0.09	135	-7.79	12157
HAVCR2	-1.27	5.05E-08	-0.05	145	-4.59	11258	-0.02	121	-1.33	9384	-0.24	123	-17.61	14907
ZNF559	-1.06	0.111122622	-0.06	128	-4.59	11259	0.11	124	7.99	3751	-0.06	131	-4.76	10967
PDCD1LG2	-1.04	0.000112552	-0.05	138	-4.59	11260	-0.07	116	-4.92	11607	-0.18	149	-17.41	14866
BEX2	-1.32	0.146516576	-0.09	102	-4.59	11261	0.07	94	3.06	6461	-0.05	127	-4.11	10682
MRFAP1	1.01	0.850846435	-0.05	143	-4.6	11262	-0.12	115	-7.49	12870	0.04	107	2.02	7437
GMPR2_t1	1.01	0.825883989	-0.06	130	-4.6	11263	NA	NA	NA	NA	NA	NA	NA	NA
TCF7L1	-1.05	0.409189329	-0.05	138	-4.6	11264	-0.04	142	-3.27	10650	-0.03	150	-3.13	10236
LGALS2	-2.1	6.88E-10	-0.15	80	-4.6	11265	-0.01	92	-0.39	8740	-0.34	71	-7.17	11910
TSPY26P	1.01	0.195778543	-0.07	117	-4.61	11266	0.06	115	4.06	5875	0.08	113	5.29	5779
KRT85	1.02	0.304060988	-0.08	111	-4.61	11267	-0.1	131	-8.45	13305	-0.05	123	-3.6	10434
CACNG6	-1.06	2.30E-05	-0.05	134	-4.61	11268	-0.01	121	-0.68	8924	-0.15	126	-11.24	13316
ZNF24_t1	-1.16	3.17E-08	-0.05	147	-4.61	11269	NA	NA	NA	NA	-0.08	146	-8.05	12250
NOS1_t1	1.05	0.008640525	-0.05	152	-4.61	11270	-0.04	154	-3.7	10933	NA	NA	NA	NA
DNM1P46_t1	1	0.665903457	-0.05	136	-4.61	11271	NA	NA	NA	NA	NA	NA	NA	NA
SMOC2	-1.6	0.001279448	-0.06	129	-4.61	11272	0.03	130	2.03	7122	-0.12	148	-11.9	13512
SIAH1	-1.09	0.00356114	-0.06	123	-4.61	11273	0.04	113	2.22	7005	-0.13	119	-9.24	12666
TFDP2	1.05	0.03535726	-0.05	146	-4.61	11274	-0.03	154	-2.92	10427	0.07	147	6.8	5103
ELMOD3	1.07	0.008498985	-0.05	139	-4.61	11275	-0.02	109	-1.34	9393	-0.08	128	-6.43	11616
IMPG1	1	0.876516318	-0.06	125	-4.61	11276	-0.07	119	-4.56	11414	0.01	128	1.01	8014
ERGIC3_t2	-1.01	0.685245169	-0.09	102	-4.62	11277	-0.13	125	-9.52	13708	-0.1	93	-4.2	10718
SLC11A1_t1	-1.03	0.371120959	-0.05	138	-4.62	11278	-0.01	119	-0.95	9107	NA	NA	NA	NA
LRRC33	-1.07	0.000110015	-0.08	111	-4.62	11279	-0.12	117	-7.92	13079	-0.18	106	-10.17	12983
RAB3D	1.04	0.002202415	-0.06	123	-4.62	11280	0.18	127	13.73	1617	0	147	-0.34	8811
RBBP9	-1.07	0.151687995	-0.04	155	-4.62	11281	-0.18	147	-17.25	15632	0.07	128	5.82	5534
ATF4	-1.15	0.012913163	-0.08	108	-4.62	11282	0.08	86	2.91	6552	-0.18	104	-9.46	12744
WDR61	1.02	0.326896966	-0.07	116	-4.63	11283	-0.04	130	-3.31	10678	0.06	108	3.33	6756
MFN2	-1.11	0.029381597	-0.05	143	-4.63	11284	-0.14	124	-10.11	13925	-0.08	140	-7.32	11966
ALDH3B2	1	0.883960972	-0.07	115	-4.63	11285	-0.15	120	-10.85	14194	-0.06	120	-4.39	10792
NTN1	1.04	0.004846051	-0.06	122	-4.63	11286	0	135	0.34	8242	0.06	143	5.52	5676
ACIN1	-1.03	0.093771881	-0.07	120	-4.63	11287	-0.12	113	-7.54	12891	-0.15	122	-10.63	13127
MYH7B	1.08	0.031662645	-0.1	98	-4.63	11288	-0.11	113	-7.01	12652	-0.07	127	-5.48	11234
TCTN1	-1.03	0.494047904	-0.09	104	-4.63	11289	-0.05	112	-3.04	10498	-0.11	96	-4.96	11038
VAMP3	-1	0.999322496	-0.07	120	-4.64	11290	0.04	126	3	6498	-0.03	145	-2.45	9921
DOCK6	1.02	0.601964784	-0.06	124	-4.64	11291	0.09	105	4.96	5367	-0.04	120	-3.1	10224
TMEM179	1.04	5.16E-05	-0.08	107	-4.65	11292	-0.07	108	-4.02	11106	0.07	112	4.27	6307
GCAT	-1.13	0.090090291	-0.12	88	-4.65	11293	-0.11	81	-3.55	10842	-0.1	88	-3.81	10540
ABLIM2	-1.02	0.774336801	-0.08	109	-4.65	11294	-0.05	145	-4.78	11528	-0.01	122	-0.99	9200
SHROOM2	-1.21	0.000505486	-0.06	129	-4.66	11295	-0.18	140	-16.45	15502	-0.01	125	-0.49	8902
RAB38	-1	0.867883516	-0.08	111	-4.66	11296	0.13	103	6.91	4294	-0.17	127	-12.88	13811
PALD1	1	0.91225303	-0.05	139	-4.66	11297	0.05	120	3.74	6038	-0.07	125	-5.57	11264
KCNA6	-1.01	0.350603417	-0.05	148	-4.66	11298	-0.06	116	-4.08	11140	-0.03	125	-2.34	9862
OTOP2	-1.03	0.038403247	-0.11	94	-4.67	11299	-0.1	103	-5.16	11754	-0.05	104	-2.65	10020
ABCA10	-1.14	2.20E-05	-0.07	119	-4.68	11300	-0.04	123	-2.69	10270	-0.09	127	-6.64	11712
LIMA1	-1.36	1.74E-05	-0.06	131	-4.68	11301	0	138	0.36	8233	-0.09	138	-7.57	12059
PPM1A_t1	-1.08	1.84E-05	-0.05	146	-4.68	11302	-0.12	121	-8.26	13220	NA	NA	NA	NA
MKL2	1.05	0.481577993	-0.04	161	-4.68	11303	0.04	145	3.98	5917	0.07	140	6.43	5265
DYNLL2	1.04	0.37982881	-0.06	124	-4.69	11304	-0.07	119	-4.84	11560	-0.09	154	-8.9	12560
CHD1	-1.2	1.04E-05	-0.06	128	-4.69	11305	-0.04	129	-2.92	10425	-0.06	134	-4.77	10975
RNF40	1.02	0.244237529	-0.06	127	-4.69	11306	0	117	-0.25	8640	-0.1	135	-8.58	12450
ALPL	-1.18	2.46E-07	-0.11	92	-4.69	11307	-0.22	150	-22.22	16191	-0.23	96	-10.39	13046
LEPR_t1	-1.83	1.50E-05	-0.06	131	-4.7	11308	-0.22	133	-17.98	15732	-0.07	156	-6.95	11826
ITM2C	-1.53	2.58E-06	-0.06	132	-4.7	11309	-0.06	111	-3.46	10772	-0.23	144	-21.4	15568
DEDD2	-1.04	0.089123923	-0.08	112	-4.7	11310	-0.15	133	-12.61	14670	-0.13	145	-12.58	13721
B3GALT4_t1	-1.5	7.20E-09	-0.09	103	-4.7	11311	NA	NA	NA	NA	-0.27	94	-11.92	13515
CEACAM8	-1.02	0.324021277	-0.06	129	-4.7	11312	-0.09	124	-6.3	12322	-0.01	122	-0.64	9007
PHF2	-1.01	0.527269081	-0.08	110	-4.7	11313	0.02	134	2.1	7083	-0.14	128	-11.03	13271
HAS1	-1.08	0.00239752	-0.08	107	-4.71	11314	-0.12	89	-4.87	11577	-0.08	101	-3.85	10558
CCDC136	1	0.647745127	-0.05	137	-4.71	11315	-0.01	116	-0.87	9056	-0.06	123	-4.33	10765
UFSP2	-1.29	3.23E-08	-0.07	119	-4.71	11316	-0.1	139	-9.33	13632	-0.14	121	-9.87	12880
NR1D1	-1.07	0.239239796	-0.09	105	-4.71	11317	-0.04	118	-2.61	10226	0	120	0.33	8416
TRIM69	-1.04	0.131099659	-0.1	97	-4.71	11318	-0.05	94	-2.03	9846	-0.15	93	-6.44	11620
CLMP	1.01	0.678499568	-0.07	120	-4.71	11319	-0.02	139	-1.79	9680	-0.06	160	-6.68	11729
ASPHD2	-1.02	0.560638587	-0.06	131	-4.71	11320	-0.05	123	-3.88	11026	-0.12	125	-8.73	12502
COL20A1	-1.16	6.72E-08	-0.13	85	-4.72	11321	-0.22	116	-14.27	15049	-0.13	120	-9.08	12617
DDX47	-1.15	0.000447918	-0.07	114	-4.72	11322	0.06	107	3.7	6059	-0.11	125	-8.18	12307
TARS	1.02	0.754205251	-0.06	123	-4.72	11323	0.09	104	4.65	5517	0.05	142	4.52	6167
COL5A1_t1	-1.13	0.035645696	-0.05	146	-4.73	11324	NA	NA	NA	NA	NA	NA	NA	NA
PAK4	1	0.956773163	-0.09	101	-4.73	11325	-0.1	136	-8.93	13476	-0.06	114	-3.62	10447
SOX15	-1.02	0.011683971	-0.12	91	-4.73	11326	-0.05	102	-2.42	10095	-0.13	130	-10.07	12953
TSPAN1	-1.17	3.98E-07	-0.09	104	-4.73	11327	-0.24	120	-17	15589	-0.11	101	-5.61	11279
KIAA1217	-1.07	0.000246216	-0.07	119	-4.73	11328	-0.05	117	-3.67	10922	-0.03	149	-3.4	10356
JAKMIP1	-1.23	0.000565282	-0.12	90	-4.73	11329	-0.04	98	-1.79	9686	-0.25	143	-23.59	15841
PLCL2	-1.08	0.046501329	-0.07	115	-4.74	11330	-0.17	116	-11.27	14318	0.09	151	9.12	4180
GSG1	1.02	0.058321066	-0.08	108	-4.75	11331	0.03	126	2.21	7009	0.09	129	6.79	5111
TPP2	-1.04	0.246636944	-0.08	107	-4.75	11332	0.08	128	6.13	4709	-0.07	120	-4.88	11009
PTGFR_t1	1.17	0.000179371	-0.05	140	-4.76	11333	NA	NA	NA	NA	0	127	-0.18	8723
VGLL1	-1.01	0.296278673	-0.07	122	-4.76	11334	-0.07	113	-4.68	11480	-0.08	113	-5.01	11052
EGR4	-1.02	0.456832485	-0.06	124	-4.76	11335	0.05	122	3.6	6110	-0.05	126	-3.93	10606
UBXN1	-1.02	0.45462611	-0.1	97	-4.77	11336	-0.04	112	-2.31	10034	-0.1	104	-5.39	11197
ZNF157	-1.21	0.000121363	-0.1	98	-4.77	11337	-0.14	129	-11.01	14243	-0.11	101	-5.8	11360
CCDC54	1	0.761416697	-0.06	134	-4.78	11338	-0.12	93	-5.1	11721	-0.04	103	-2.05	9733
RSAD2	-1.08	0.00018134	-0.05	144	-4.78	11339	-0.07	136	-6.09	12240	-0.18	144	-17.14	14821
ZBTB16	-1.5	2.09E-08	-0.14	84	-4.78	11340	-0.14	67	-2.46	10124	-0.19	80	-5.66	11301
TGM7	1.02	0.232849829	-0.11	95	-4.78	11341	-0.12	104	-6.25	12302	-0.03	94	-1.36	9373
RAB9A	-1.11	0.013633994	-0.05	141	-4.79	11342	-0.04	115	-2.71	10284	-0.09	134	-7.9	12197
USP36	1.02	0.781446774	-0.07	121	-4.8	11343	0.08	147	7.47	4004	-0.11	112	-6.71	11738
VPS13C	-1.06	0.37323709	-0.06	130	-4.8	11344	0.11	134	8.83	3364	-0.07	127	-5.62	11281
GPSM3_t1	-1.38	5.45E-09	-0.07	120	-4.8	11345	NA	NA	NA	NA	-0.14	115	-9.22	12659
TEX26	-1.03	0.000994404	-0.2	74	-4.8	11346	-0.02	127	-1.67	9600	-0.26	84	-8.93	12567
KCNS3	-1.74	6.16E-08	-0.08	111	-4.81	11347	-0.05	120	-3.56	10843	-0.19	119	-13.11	13875
DPP7	-1.44	3.21E-10	-0.13	88	-4.81	11348	-0.18	100	-9.2	13591	-0.23	88	-8.72	12497

AQP11	-1.16	0.075877404	-0.07	115	-4.81	11349	-0.12	125	-9.11	13562	-0.05	120	-3.7	10483
ARAP1	-1.21	1.33E-07	-0.07	119	-4.82	11350	0.07	121	5.24	5199	-0.28	104	-15.16	14411
KRT27	-1.05	0.001162768	-0.11	93	-4.82	11351	-0.09	100	-4.71	11493	-0.07	109	-3.96	10623
CCDC42	-1.04	0.00032535	-0.1	98	-4.82	11352	-0.25	105	-13.77	14941	-0.05	104	-2.77	10075
STYK1	-1.02	0.476439548	-0.07	121	-4.82	11354	0.06	124	4.4	5660	-0.22	110	-13.13	13881
PGPEP1	1	0.886629533	-0.05	144	-4.82	11355	0.03	132	2.7	6681	-0.08	135	-6.79	11767
CDH4	-1.12	1.32E-09	-0.07	121	-4.82	11356	-0.23	131	-18.78	15837	-0.19	118	-12.81	13793
SLC5A10	1.07	0.038114423	-0.15	82	-4.83	11357	-0.03	128	-2.15	9932	0	100	0.03	8600
CELA1	1.03	0.139443028	-0.1	100	-4.83	11358	-0.07	113	-4.48	11375	-0.05	96	-2.19	9790
CCND3	1	0.875881782	-0.08	114	-4.83	11359	-0.01	113	-0.78	9009	-0.03	146	-2.74	10054
A2ML1	1	0.886055965	-0.06	128	-4.83	11360	-0.2	127	-15.64	15333	-0.11	128	-8.24	12330
KLRC4	-1.28	2.42E-07	-0.07	118	-4.83	11361	-0.05	119	-3.28	10656	-0.27	104	-14.78	14322
TLE4	-1.05	0.000978987	-0.06	126	-4.84	11362	-0.04	136	-3.48	10787	-0.15	139	-13.3	13922
DNAJA2	-1.17	0.000117241	-0.05	146	-4.84	11363	-0.05	113	-3.1	10540	-0.06	141	-5.83	11378
PAWR	-1.17	0.000145616	-0.05	147	-4.84	11364	-0.14	116	-9.22	13597	-0.02	124	-1.78	9601
SMCR7	1.03	0.366855346	-0.07	122	-4.84	11365	-0.06	132	-4.74	11509	0.02	112	0.94	8048
SLITRK3	-1.2	0.005854871	-0.07	124	-4.85	11366	-0.13	154	-13.57	14884	-0.21	127	-16.41	14667
RGR	1.01	0.39676885	-0.06	133	-4.85	11367	-0.12	158	-12.5	14640	-0.07	128	-5.49	11238
CDH2	-1.21	0.000356743	-0.06	127	-4.85	11368	-0.11	127	-8.74	13414	0	160	-0.18	8721
IL17RC	-1.58	1.92E-15	-0.27	68	-4.85	11369	-0.28	84	-9.44	13671	-0.3	73	-6.89	11810
RCVRN	-1.01	0.321635995	-0.09	102	-4.86	11370	-0.02	118	-1.49	9488	-0.02	100	-0.86	9109
TCEB3B	1	0.784804099	-0.07	118	-4.86	11371	-0.01	121	-0.75	8968	-0.02	124	-1.61	9504
ATG9A	1.05	0.116253612	-0.07	118	-4.86	11372	-0.02	123	-1.39	9427	-0.06	131	-4.53	10854
RARRES2	-1.51	1.88E-05	-0.13	88	-4.86	11373	-0.25	104	-13.72	14923	-0.16	100	-8.24	12329
RHBDD2	1.07	0.056136859	-0.06	126	-4.87	11374	-0.04	104	-2.02	9833	-0.01	111	-0.82	9098
PER3	1.06	0.023764169	-0.05	154	-4.87	11375	-0.15	146	-13.97	14988	0.14	126	10.69	3619
THRA_t2	-1.12	1.79E-05	-0.09	106	-4.87	11376	-0.14	113	-8.79	13430	-0.15	125	-11.24	13318
NMD3	-1.09	0.007973752	-0.06	127	-4.87	11377	-0.1	118	-6.54	12430	-0.01	121	-0.39	8845
TAF5L_t2	-1.13	0.002898668	-0.06	137	-4.87	11378	-0.11	120	-7.73	13003	-0.07	127	-5.12	11087
NCF2	-1.52	2.11E-06	-0.06	132	-4.88	11379	0.08	117	5.3	5170	-0.25	120	-17.28	14846
SFT2D1	-1.18	0.000185227	-0.07	124	-4.88	11380	0.02	144	2.31	6947	-0.22	124	-16.57	14705
PPP2CB	-1.24	0.000184212	-0.07	123	-4.88	11381	-0.18	106	-10.18	13945	-0.05	127	-4.08	10674
USP44	1.01	0.640433758	-0.07	120	-4.88	11382	-0.07	147	-6.76	12538	-0.08	126	-6.25	11554
TPT1	-1.16	0.002844547	-0.07	120	-4.88	11383	-0.03	100	-1.47	9478	-0.19	104	-10.36	13037
FGFBP2	-1.16	2.35E-05	-0.08	110	-4.89	11384	-0.04	130	-3.52	10816	-0.19	114	-12.18	13603
PGAP2	1.11	0.00295494	-0.06	129	-4.89	11385	0.07	124	5.26	5183	0.07	117	4.82	6016
ZNF689	1.02	0.532229644	-0.05	141	-4.9	11386	-0.07	153	-6.81	12569	-0.03	145	-3.32	10316
TCEAL4	1.01	0.83933296	-0.07	116	-4.9	11387	0.01	116	0.95	7833	0.08	112	5.01	5916
RNF217	-1.05	0.209953225	-0.06	138	-4.9	11388	0.03	139	2.75	6641	-0.06	120	-4.42	10810
CCBL2	-1.06	0.070164225	-0.07	119	-4.9	11389	-0.03	120	-2.32	10038	-0.04	111	-2.16	9775
ERLIN2_t2	-1.03	0.129476615	-0.06	139	-4.91	11390	0.07	154	7.79	3835	-0.14	120	-9.89	12890
RING1	1.04	0.196680523	-0.1	98	-4.91	11391	-0.16	108	-9.17	13578	-0.04	132	-2.92	10147
DBNL_t2	-1.01	0.519159401	-0.07	123	-4.91	11392	0	120	-0.08	8541	-0.14	114	-8.83	12534
RTBDN	-1.05	0.012303698	-0.12	92	-4.91	11393	-0.14	100	-7.12	12710	-0.12	90	-4.97	11039
TNFSF11	-1.24	2.26E-06	-0.05	140	-4.91	11394	-0.1	115	-6.44	12390	-0.12	130	-9.82	12863
HS3ST1	-1.21	0.011786434	-0.06	129	-4.92	11395	0.08	122	5.8	4894	-0.17	139	-15.15	14406
RFX7	1.03	0.408777719	-0.05	144	-4.92	11396	0.02	120	1.35	7573	0.06	154	6.24	5340
NRIP1	-1.22	0.001994243	-0.05	143	-4.92	11397	-0.04	134	-3.6	10883	-0.05	139	-4.51	10840
TRPV5	-1.1	9.80E-07	-0.1	100	-4.92	11398	-0.08	115	-5.04	11681	-0.12	87	-4.49	10837
SLC13A4	-1.06	8.82E-06	-0.09	102	-4.93	11399	-0.2	104	-10.58	14103	-0.17	89	-6.52	11656
UFL1	-1.2	0.006101592	-0.05	142	-4.93	11400	-0.01	124	-0.56	8856	-0.1	135	-8.49	12425
PCED1A	-1.11	0.000204665	-0.08	112	-4.93	11401	-0.32	88	-12.06	14523	-0.05	105	-2.49	9947
YPEL3	-1.07	0.072930077	-0.08	110	-4.93	11402	-0.07	104	-3.72	10942	-0.04	123	-3.03	10193
RSPH9	-1.03	0.01272872	-0.11	93	-4.94	11403	-0.13	103	-7.14	12715	-0.17	64	-2.32	9850
CAMK2N2	-1.16	3.78E-05	-0.07	124	-4.94	11404	-0.11	129	-9.02	13518	-0.13	120	-9.39	12713
SLC25A28_t1	-1.12	0.000395623	-0.1	98	-4.94	11405	NA	NA	NA	NA	-0.16	109	-9.47	12750
SLC14A2	1.03	0.122164393	-0.06	127	-4.94	11406	-0.1	135	-8.28	13228	-0.08	120	-5.42	11207
ATP2B1	-1.09	0.048043393	-0.06	131	-4.94	11407	0.02	137	1.87	7232	0.02	143	2.16	7366
SERPINC1	-1.77	0.001404551	-0.1	102	-4.94	11408	-0.33	103	-17.23	15627	-0.04	134	-3.72	10495
BCL2L2	1.01	0.751478331	-0.06	136	-4.94	11409	0.02	130	1.32	7591	-0.07	133	-5.67	11303
HEYL	-1.33	0.002573294	-0.06	138	-4.94	11410	0.06	132	4.85	5428	-0.16	123	-12	13538
SIPA1L1_t1	-1.12	0.0078865	-0.09	103	-4.95	11411	NA	NA	NA	NA	NA	NA	NA	NA
FAM49A	-1.46	2.03E-08	-0.05	141	-4.95	11412	-0.09	130	-7.2	12739	-0.24	133	-20.14	15356
WDR18	-1.03	0.427175827	-0.15	84	-4.95	11413	-0.07	108	-4.2	11200	-0.13	99	-6.5	11641
S100A9	-2.01	4.96E-11	-0.15	84	-4.95	11414	-0.1	106	-5.76	12067	-0.32	80	-9.72	12817
ZFYVE27	-1.05	0.245471366	-0.08	113	-4.95	11415	0.01	133	1.14	7724	-0.07	118	-4.68	10931
IL20	1.04	0.059616706	-0.05	151	-4.95	11416	-0.03	115	-2.02	9832	-0.05	131	-3.78	10524
TMEM120A	-1	0.95252194	-0.11	96	-4.96	11417	-0.12	98	-5.97	12166	0.02	88	0.65	8228
GRM1	-1	0.753268593	-0.05	147	-4.96	11418	0.05	135	4.64	5525	-0.17	163	-19.01	15175
MYEOV	-1.04	0.087090855	-0.1	99	-4.96	11419	-0.04	108	-2.3	10025	-0.14	123	-9.86	12875
SMTN	-1.13	0.000201338	-0.12	93	-4.96	11420	-0.03	119	-1.99	9818	-0.14	97	-6.44	11622
PRPH2	1.03	0.275185066	-0.07	118	-4.97	11421	0.12	133	9.75	2957	-0.03	124	-2.48	9939
GGTA1P	-1.49	4.36E-06	-0.07	120	-4.97	11422	-0.04	140	-3.21	10605	-0.16	113	-10.24	13004
GPR97	-1.12	4.93E-08	-0.08	112	-4.97	11423	-0.19	120	-13.34	14837	-0.09	120	-6.6	11694
GMEB2	1.05	0.024273356	-0.06	131	-4.97	11424	-0.05	135	-3.87	11012	-0.05	129	-3.83	10549
ADCY3	-1.42	4.15E-07	-0.07	120	-4.97	11425	-0.01	128	-0.76	8987	-0.24	114	-15.4	14457
FOSL1	-1.05	0.000131417	-0.09	104	-4.97	11426	-0.08	151	-8.29	13234	-0.1	155	-10.94	13232
IL3	-1.03	0.00268859	-0.09	105	-4.97	11427	-0.19	138	-16.92	15580	-0.06	119	-4.43	10818
EVX1	-1.28	2.13E-08	-0.16	81	-4.97	11428	-0.16	104	-8.8	13434	-0.16	68	-2.81	10094
SKIV2L2	1.01	0.885768133	-0.08	115	-4.98	11429	0	107	-0.1	8555	0.14	121	10.19	3799
RAB2B	-1.03	0.093268395	-0.07	124	-4.98	11430	-0.03	125	-2.47	10136	-0.16	150	-15.51	14485
TMLHE	-1.15	5.02E-06	-0.05	144	-4.98	11431	-0.1	144	-9.07	13544	-0.05	142	-4.57	10872
LCE2A	-1.07	1.04E-05	-0.08	112	-4.98	11432	-0.06	111	-3.89	11028	-0.11	110	-6.5	11638
TRPV2	-1.31	3.53E-05	-0.14	85	-4.98	11433	0.06	86	1.98	7157	-0.31	112	-19.01	15176
CCDC68	-1.47	4.96E-09	-0.06	140	-4.98	11434	-0.04	134	-3.06	10508	-0.1	129	-7.82	12170
ENTPD2	-1.04	0.031868661	-0.06	127	-4.98	11435	-0.11	114	-6.95	12627	-0.13	114	-8.32	12367
UNC93B1	-1.15	1.80E-06	-0.1	99	-4.99	11436	-0.06	109	-3.51	10807	-0.26	128	-20.25	15377
BTN3A3	-1.11	0.017165571	-0.06	128	-4.99	11437	-0.06	124	-4.74	11510	-0.22	103	-11.49	13398
SLC15A3	-1.44	1.37E-10	-0.09	103	-4.99	11438	-0.06	120	-3.92	11050	-0.33	90	-13.33	13928
KCTD15_t1	-1.01	0.838026512	-0.05	141	-4.99	11439	NA	NA	NA	NA	-0.03	135	-2.58	9990
FBXO18	-1.08	0.00431697	-0.1	100	-4.99	11440	-0.05	130	-4.35	11285	-0.1	120	-7.35	11976
HOXC6	1.01	0.235981094	-0.08	116	-4.99	11441	-0.04	138	-3.48	10785	0.02	122	1.1	7968
STAB1	-1.05	0.001606532	-0.07	119	-5	11442	-0.16	88	-6.11	12251	-0.05	100	-2.61	10003
ARRB2	-1.24	3.84E-08	-0.09	103	-5	11443	-0.13	137	-10.99	14232	-0.21	102	-11.15	13294
PSENEN	1.02	0.372780596	-0.09	103	-5	11444	-0.04	134	-3.36	10714	-0.1	100	-4.86	10999
NICN1	1.16	0.000586766	-0.06	131	-5	11445	0.1	140	8.87	3344	0.09	153	9.23	4147
TMEM55A	-1.13	0.038491566	-0.06	132	-5	11446	0.09	138	8.2	3639	-0.07	150	-6.96	11827
SLC12A5	1.01	0.301480336	-0.06	128	-5	11447	-0.03	125	-2.25	9997	-0.02	148	-1.5	9449
ECSCR	-1.14	0.014828698	-0.09	108	-5	11448	-0.04	103	-2.24	9991	-0.11	145	-10.58	13111
CHSY3	-1.15	0.000271969	-0.06	132	-5.01	11449	-0.07	110	-4.5	11386	-0.13	125	-9.73	12821
PF4V1	-1	0.916810983	-0.07	118	-5.01	11450	-0.02	108	-1.14	9246	-0.15	126	-11.03	13269
PSTPIP1	-1.25	3.36E-06	-0.14	86	-5.02	11451	-0.03	89	-1.16	9261	-0.28	100	-13.94	14095
DPYSL2_t1	-1.44	3.75E-07	-0.06	128	-5.02	11452	NA	NA	NA	NA	NA	NA	NA	NA
PRB4	-1.06	0.016851466	-0.13	88	-5.02	11453	-0.2	96	-9.01	13506	-0.15	104	-8.21	12319
ITSN1_t1	-1.27	2.32E-07	-0.06	130	-5.03	11454	NA	NA	NA	NA	-0.08	152	-8.23	12325
SLPI	-1.49	0.007796608	-0.09	104	-5.03	11455	-0.18	122	-12.87	14722	-0.04	144	-3.52	10404

SLC17A7	-1.05	0.010702863	-0.05	143	-5.03	11456	-0.1	122	-7.44	12847	-0.09	121	-6.19	11517
EMP1	-1.59	9.02E-05	-0.07	120	-5.03	11457	0.02	134	1.57	7449	-0.16	141	-14.73	14302
MYL9	-1.47	2.73E-07	-0.15	84	-5.03	11458	0.01	117	0.42	8190	-0.25	100	-12.41	13671
NLRP14	-1.02	0.058025016	-0.08	116	-5.03	11459	-0.13	126	-9.74	13794	-0.03	122	-2.44	9918
PPAP2C	1.09	0.275732931	-0.11	95	-5.04	11460	-0.03	92	-1.34	9388	-0.07	120	-4.75	10963
LIAS	1.06	0.071265545	-0.07	120	-5.04	11461	-0.03	113	-2.09	9890	0.05	118	3.52	6664
SLC13A2	1.04	0.205652047	-0.06	134	-5.04	11462	-0.08	132	-6.66	12494	0.01	143	0.83	8120
DDX58	-1.1	0.075859981	-0.05	153	-5.04	11463	-0.09	133	-7.67	12964	-0.06	142	-5.45	11219
PAX4_t1	1.05	0.002971372	-0.08	114	-5.04	11464	NA	NA	NA	NA	-0.02	120	-1.34	9362
RMND1	1.08	0.020063877	-0.09	108	-5.04	11465	0.05	139	4.39	5669	0	120	0.08	8562
LAG3	-1.89	1.28E-12	-0.28	68	-5.04	11466	-0.24	109	-13.88	14963	-0.33	62	-3.95	10612
COL16A1	-1.92	3.78E-07	-0.11	94	-5.04	11467	0.05	114	3.27	6342	-0.18	110	-10.89	13216
RAP1A	-1.12	0.032088455	-0.06	141	-5.05	11468	0.04	118	2.71	6673	-0.01	131	-1.03	9217
GPR20_t1	-1.08	0.093433102	-0.1	103	-5.05	11469	0.12	102	6.5	4512	NA	NA	NA	NA
CASQ1	1.07	5.21E-05	-0.06	139	-5.05	11470	0.02	129	1.34	7581	0.03	100	1.52	7735
CREBBP_t2	1.01	0.448115765	-0.06	133	-5.05	11471	-0.12	135	-10.19	13946	0.01	147	1.38	7822
GJB3	-1.05	0.078992141	-0.08	115	-5.06	11472	-0.11	114	-6.78	12552	-0.18	104	-9.9	12897
PLEKHG6	-1.03	0.077071245	-0.08	111	-5.06	11473	-0.18	127	-13.96	14986	-0.16	100	-7.79	12159
NRP2_t2	-1.03	0.013365291	-0.06	139	-5.06	11474	-0.11	124	-8.4	13289	0.02	146	1.99	7458
ESYT1	-1.02	0.53130467	-0.06	129	-5.06	11475	-0.12	118	-7.96	13096	-0.08	135	-6.61	11696
NUP54	-1.12	0.012631167	-0.05	146	-5.06	11476	-0.01	121	-0.85	9044	-0.01	136	-0.52	8931
RGMB	1.03	0.69728188	-0.06	131	-5.06	11477	-0.14	122	-10.31	14005	0.02	134	2.01	7446
CARD9	-1.3	8.25E-07	-0.08	111	-5.07	11478	0.04	125	3.08	6451	-0.27	119	-18.45	15071
PCDH1_t1	-1.11	0.001555198	-0.1	102	-5.07	11479	-0.17	104	-8.99	13497	-0.02	106	-1.14	9270
OLFM2	1.09	0.036090297	-0.09	104	-5.07	11480	-0.04	120	-2.95	10448	0.04	129	3.47	6681
CLDN8	1	0.82539287	-0.06	141	-5.07	11481	-0.06	119	-4.36	11293	-0.05	128	-4	10642
ING4	-1.05	0.0874802	-0.08	114	-5.07	11482	-0.11	120	-7.48	12865	-0.12	119	-8.47	12418
F2RL1	-1.13	0.010253118	-0.05	151	-5.07	11483	-0.03	135	-2.96	10456	-0.21	128	-16.34	14656
EIF4H_t2	-1.07	0.001721249	-0.08	116	-5.07	11484	-0.06	125	-4.27	11252	-0.1	150	-10.19	12988
EMILIN3	-1.01	0.51502603	-0.06	130	-5.07	11485	-0.14	134	-11.83	14459	-0.06	132	-4.59	10887
ABCC8	1.06	0.079141776	-0.09	107	-5.07	11486	-0.09	122	-6.65	12488	0	95	-0.19	8729
CAMK1D_t2	-1.36	0.000240217	-0.06	136	-5.08	11487	0	131	0.23	8303	-0.14	121	-10.07	12950
SPTB_t2	-1.1	0.000337387	-0.09	106	-5.08	11488	-0.04	132	-2.94	10436	-0.15	120	-10.39	13052
AK7	1.05	0.021215348	-0.07	124	-5.08	11489	0	146	0.3	8261	-0.04	127	-2.76	10068
KCNJ14	1.17	0.00110384	-0.07	127	-5.08	11490	0.03	118	1.89	7223	0.01	113	0.32	8427
METTL21D	-1.16	8.00E-06	-0.07	124	-5.08	11491	-0.07	109	-4.24	11237	-0.07	135	-5.87	11389
SLC13A3	-1.32	0.128570029	-0.07	127	-5.09	11492	-0.06	141	-5.57	11951	-0.1	120	-6.76	11761
FBXL13	-1.12	5.14E-06	-0.06	135	-5.09	11493	-0.16	116	-10.4	14036	-0.04	126	-3.39	10351
TTI2	-1.1	0.00271981	-0.07	123	-5.09	11494	-0.15	135	-13.15	14783	-0.19	126	-14.51	14247
PCYOX1	-1.06	0.061653231	-0.06	136	-5.09	11495	-0.09	154	-9.47	13688	-0.09	159	-10.34	13033
TMED3	-1.03	0.73735685	-0.06	135	-5.09	11496	0.23	104	12.39	2011	-0.09	134	-7.81	12167
SEMA6C	1.07	0.078457005	-0.05	146	-5.09	11497	-0.07	120	-5.25	11798	0.03	111	1.6	7689
PIP	-1.03	0.638258891	-0.09	104	-5.09	11498	-0.05	105	-2.9	10415	0	98	-0.18	8720
BAZ2B	-1.26	1.48E-06	-0.05	156	-5.09	11499	0	120	-0.15	8579	-0.09	124	-6.55	11667
CNTNAP5	1.03	0.073491929	-0.05	143	-5.09	11500	-0.12	143	-10.85	14195	-0.06	127	-4.8	10986
CDC37	1.04	0.198012513	-0.06	132	-5.09	11501	0.02	126	1.52	7487	-0.05	130	-4.07	10668
SH2B3	-1.03	0.126604844	-0.07	119	-5.09	11502	0.06	133	4.89	5400	-0.04	129	-3.47	10382
GFRA2_t1	-1.07	0.000322835	-0.08	116	-5.09	11503	NA	NA	NA	NA	-0.14	113	-8.89	12557
DIAPH1	-1.01	0.879497039	-0.08	114	-5.1	11504	-0.07	120	-5.02	11669	0.05	125	3.65	6589
RBM38	-1.03	0.054909046	-0.07	123	-5.1	11505	-0.05	152	-5.51	11926	-0.12	134	-10.03	12936
CELA2A	-1.11	0.019812859	-0.09	104	-5.1	11506	-0.07	95	-3.11	10552	-0.14	83	-4.66	10916
ELAC1	1	0.994911758	-0.06	131	-5.1	11507	-0.06	120	-4.21	11209	-0.07	128	-5.31	11166
FAM131B_t1	-1	0.799563852	-0.07	119	-5.1	11508	NA	NA	NA	NA	-0.04	135	-3.74	10505
ETHE1	-1.15	0.000878881	-0.13	88	-5.11	11509	-0.19	125	-13.93	14980	-0.15	121	-10.49	13085
KCNC2_t2	1.03	0.5317569	-0.06	129	-5.11	11510	-0.06	129	-4.6	11440	-0.03	154	-3.51	10397
HES4	-1.16	3.73E-06	-0.15	84	-5.11	11511	-0.07	93	-2.84	10370	-0.25	91	-10.06	12944
TMEM44_t1	-1.02	0.120598163	-0.08	115	-5.11	11512	-0.12	121	-8.39	13282	-0.07	120	-4.84	10995
AMBP	-1.27	0.006097657	-0.13	88	-5.11	11513	-0.34	130	-27.24	16422	-0.1	95	-4.52	10844
CTSS	-1.35	1.05E-13	-0.06	131	-5.11	11514	-0.11	135	-9.12	13567	-0.25	134	-21.2	15538
COLQ	1.08	0.006679435	-0.07	121	-5.12	11515	0.08	127	6.04	4757	0.03	120	2.45	7207
CCDC88B_t1	-1.2	4.24E-06	-0.09	104	-5.12	11516	NA	NA	NA	NA	-0.21	100	-10.31	13028
PEX6	1.16	2.71E-05	-0.06	132	-5.12	11517	0.07	95	3.05	6468	0.04	100	1.83	7543
XPO6	1.01	0.635897894	-0.1	100	-5.12	11518	0.02	165	2.07	7101	-0.09	116	-5.77	11347
TECRL	-1.01	0.337014549	-0.06	136	-5.12	11519	-0.02	134	-1.72	9632	0	131	-0.27	8777
SPATA20	-1.09	0.001199629	-0.13	90	-5.12	11520	-0.15	117	-10.35	14020	-0.18	80	-5.49	11237
RARRES3	-1.25	0.000477675	-0.08	114	-5.13	11521	-0.15	98	-7.15	12718	-0.17	90	-6.88	11807
ASTN2_t2	1.12	0.00240595	-0.06	134	-5.13	11522	-0.04	136	-3.43	10754	0.07	119	4.88	5985
GPR162_t1	-1.12	7.35E-09	-0.13	88	-5.13	11523	NA	NA	NA	NA	-0.17	92	-6.98	11838
PADI1	1.04	0.052391593	-0.09	105	-5.13	11524	-0.05	124	-3.67	10920	0.04	120	2.96	6953
GRAP	-1.06	2.44E-07	-0.09	110	-5.13	11525	-0.19	147	-18.02	15736	-0.18	115	-11.61	13433
KIAA0664	-1.33	4.09E-11	-0.1	99	-5.13	11526	-0.25	115	-16.08	15436	-0.19	125	-13.88	14072
TCN1	-1.06	0.362429564	-0.11	95	-5.13	11527	-0.01	92	-0.23	8626	-0.14	109	-8.49	12423
PCSK4	-1.01	0.907045049	-0.1	99	-5.13	11528	-0.06	108	-3.54	10833	-0.06	104	-3.08	10218
COL11A2	-1.06	0.04928037	-0.05	146	-5.14	11529	0.02	111	0.95	7838	-0.15	116	-9.87	12884
ROPN1	-1	0.951470056	-0.09	110	-5.14	11530	-0.07	116	-4.86	11570	-0.05	129	-3.6	10437
UBR7	1.06	0.194109258	-0.04	168	-5.14	11531	-0.14	136	-12.04	14512	0.14	166	16.33	2002
ADAMTS19	-1.03	0.005872236	-0.05	145	-5.14	11532	-0.12	122	-8.47	13315	-0.04	148	-3.54	10415
CCDC85B	-1.15	3.13E-05	-0.1	100	-5.14	11533	-0.05	92	-1.99	9812	-0.13	100	-6.53	11660
ARL16	1.12	0.000659431	-0.06	135	-5.15	11534	0.08	121	5.66	4968	0.02	153	2.27	7312
ANP32A	-1.07	0.019681572	-0.07	126	-5.15	11535	-0.07	127	-5.67	12011	0	120	0.08	8566
TMEM44_t2	-1.23	7.34E-05	-0.08	115	-5.15	11536	-0.01	121	-0.38	8731	-0.17	120	-12.13	13586
RGS7BP	-1.05	1.29E-05	-0.06	131	-5.15	11537	-0.21	130	-16.68	15544	-0.07	142	-6.7	11734
WFDC13	1.01	0.397052498	-0.06	135	-5.15	11538	-0.12	125	-8.66	13378	0.07	147	7.02	5028
GNPTG	-1.11	0.002339912	-0.14	88	-5.15	11539	-0.11	100	-5.4	11873	-0.18	126	-13.66	14011
FNDC1	-1.19	0.076504295	-0.06	140	-5.15	11540	-0.01	131	-1.04	9175	-0.14	133	-12.03	13555
GNG12	-1.21	1.23E-05	-0.07	129	-5.15	11541	-0.03	156	-2.91	10419	-0.08	144	-7.35	11979
KIF25	-1.15	0.048502959	-0.08	111	-5.15	11542	-0.17	80	-5.16	11749	-0.19	107	-10.97	13249
GPRC5B	-1.15	0.064800379	-0.07	127	-5.16	11543	0	119	0.16	8359	-0.19	128	-15.14	14403
VTN	-1.93	5.18E-12	-0.21	74	-5.16	11544	-0.39	96	-17.78	15709	-0.26	68	-4.72	10943
ZNHIT2	-1.09	0.000480978	-0.18	78	-5.16	11545	-0.12	79	-3.58	10862	-0.12	77	-3.36	10336
DYX1C1	1.12	0.013526035	-0.07	126	-5.16	11546	0.21	111	12.77	1907	0.01	122	0.43	8351
CAMK1	-1.09	0.00559638	-0.11	96	-5.16	11547	-0.11	120	-7.47	12857	-0.17	104	-9.05	12605
WNT6	-1.09	0.001078874	-0.09	109	-5.16	11548	-0.13	108	-7.35	12811	-0.14	91	-5.87	11392
SYCP2	1.01	0.756910441	-0.07	122	-5.16	11549	0.11	120	7.8	3830	0.04	110	2.32	7277
EIF4E3	-1.09	0.000516934	-0.06	135	-5.17	11550	-0.02	159	-2.65	10250	-0.28	151	-28.26	16206
SERINC3_t2	-1.04	0.089861442	-0.1	104	-5.17	11551	-0.2	103	-10.55	14087	-0.01	84	-0.44	8875
ZNF660	-1.02	0.173889629	-0.08	118	-5.17	11552	0.05	118	3.57	6128	-0.07	132	-6.12	11483
TRPM1	-1.01	0.434570384	-0.07	120	-5.18	11553	-0.07	122	-4.84	11563	0.07	126	5.21	5827
ADAM33_t1	-1.06	4.32E-05	-0.07	128	-5.18	11554	NA	NA	NA	NA	NA	NA	NA	NA
SLC12A4	-1.19	5.49E-06	-0.08	117	-5.18	11555	-0.14	120	-9.78	13809	-0.18	121	-12.91	13823
GALR2	-1.02	0.228394973	-0.17	80	-5.19	11556	-0.1	88	-3.83	10995	-0.11	100	-5.72	11325
VEGFC	-1.4	3.83E-06	-0.1	103	-5.19	11557	-0.04	114	-2.74	10300	-0.19	129	-14.65	14282
OSTC	-1.09	0.058980228	-0.07	122	-5.19	11558	0.03	114	2	7146	0	124	-0.35	8821
RSL24D1	-1.04	0.336992165	-0.06	144	-5.19	11559	0.03	125	2.44	6864	0.02	131	1.59	7694
IQCF2	-1.01	0.527723983	-0.13	89	-5.19	11560	-0.14	97	-6.65	12491	-0.07	100	-3.38	10346
KCNG4	-1.03	0.093098425	-0.08	112	-5.2	11561	-0.14	130	-11.29	14322	-0.02	113	-0.98	9190

TSHR_t2	-1.04	3.15E-05	-0.09	109	-5.2	11562	-0.11	111	-6.87	12594	-0.13	135	-11.01	13262
ERCC6L2	-1.1	2.38E-05	-0.05	146	-5.2	11563	-0.05	127	-4.12	11158	-0.1	131	-8.33	12376
MFSD10	-1.02	0.551623778	-0.08	116	-5.2	11564	-0.03	131	-2.66	10253	-0.17	120	-12.21	13612
CHURC1	-1.08	0.003321656	-0.07	129	-5.2	11565	0.02	134	1.65	7389	0.01	128	0.92	8067
KCTD12	-1.37	1.55E-07	-0.06	135	-5.2	11566	0.05	133	4.29	5718	-0.25	129	-19.55	15257
TXNDC11_t1	-1.25	1.51E-12	-0.12	92	-5.21	11567	NA	NA	NA	NA	-0.27	118	-18.09	14991
KIF1B_t2	-1.11	0.006482981	-0.05	155	-5.21	11568	-0.06	132	-4.84	11559	0.05	132	3.76	6539
NUP210L	1.03	0.013430831	-0.09	108	-5.21	11569	-0.07	103	-3.93	11057	-0.01	89	-0.22	8744
NAP1L4	-1.08	0.002755405	-0.08	115	-5.21	11570	-0.08	130	-6.64	12483	-0.15	126	-11.28	13342
CYP4Z1	-1.25	4.80E-07	-0.07	128	-5.22	11571	-0.15	121	-10.72	14143	-0.1	115	-6.71	11739
BCDIN3D	1.06	0.047352398	-0.07	127	-5.22	11572	-0.08	146	-7.38	12826	-0.04	140	-3.37	10340
DPPA4	-1.05	0.000794474	-0.06	132	-5.22	11573	-0.17	131	-14.14	15016	-0.06	136	-12	13543
TCAP	1.23	0.008603757	-0.1	105	-5.23	11574	-0.1	113	-6.02	12198	-0.01	104	-0.43	8871
SPG7_t1	1.01	0.620964699	-0.06	134	-5.23	11575	0.01	111	0.53	8123	-0.08	100	-4.19	10711
SELM	-1.02	0.874833316	-0.07	120	-5.23	11576	0.07	120	4.61	5545	-0.06	88	-2.1	9757
BMP1_t1	-1.05	0.030774623	-0.12	93	-5.23	11577	-0.15	123	-10.81	14178	-0.13	118	-8.68	12484
THAP4	-1.07	0.016672863	-0.11	97	-5.23	11578	-0.06	108	-3.31	10686	-0.24	104	-12.69	13758
KCNK12	-1.03	0.370978526	-0.09	108	-5.23	11579	-0.12	96	-5.43	11883	-0.09	94	-3.85	10559
MIOS	-1.06	0.112608833	-0.08	115	-5.24	11580	-0.11	129	-8.8	13433	0.04	120	2.79	7035
IL36B	-1.07	0.000879367	-0.07	123	-5.24	11581	-0.1	108	-5.99	12178	-0.09	107	-5.25	11139
UPP1	-1.1	0.063314611	-0.08	112	-5.24	11582	0	118	-0.15	8583.5	-0.11	127	-8.68	12485
MESDC2	-1	0.932765504	-0.07	128	-5.24	11583	-0.02	127	-1.7	9615	-0.04	145	-3.85	10555
SLIT1	-1.05	0.066945745	-0.06	134	-5.25	11584	-0.23	153	-23.5	16276	-0.13	148	-12.35	13657
ADAM12_t2	1.03	0.374169982	-0.07	129	-5.25	11585	0.04	131	2.99	6500	-0.03	144	-2.97	10169
LBP	-1.54	2.88E-06	-0.07	125	-5.25	11586	-0.16	113	-10.38	14033	-0.05	126	-3.53	10409
DICER1_t1	-1.24	9.01E-05	-0.06	140	-5.25	11587	-0.06	148	-6.13	12257	NA	NA	NA	NA
SSBP2	-1.11	0.010384929	-0.05	146	-5.25	11588	0.01	138	1.26	7637	-0.15	132	-12.22	13615
TBX10	-1.04	0.08109412	-0.07	123	-5.25	11589	-0.16	100	-7.84	13044	-0.06	103	-2.98	10172
SLAIN1	-1.42	2.97E-08	-0.07	121	-5.26	11590	-0.17	135	-14.62	15128	-0.3	152	-30.97	16331
INPP4B	-1.09	0.147260604	-0.07	124	-5.26	11591	0.07	120	4.72	5494	-0.09	119	-6.32	11583
BPY2B	1.02	0.476000916	-0.07	121	-5.26	11592	-0.05	167	-6.19	12282	-0.1	115	-6.24	11547
LOXL1	-1.8	4.25E-05	-0.08	115	-5.27	11593	0	138	0.1	8400	-0.21	111	-12.89	13812
SLC26A3	-1.04	0.327093229	-0.09	108	-5.27	11594	-0.07	111	-4.45	11358	-0.11	122	-7.67	12096
TMEM19_t1	1.13	0.015865405	-0.06	135	-5.28	11595	0.02	133	1.32	7592	NA	NA	NA	NA
KANSL2	1.02	0.461006594	-0.06	137	-5.28	11596	0.01	110	0.81	7934	-0.06	119	-4.14	10692
STMN3_t1	-1.08	0.00476685	-0.08	116	-5.28	11597	NA	NA	NA	NA	-0.18	131	-14.91	14357
MYH13	1.31	0.005858775	-0.1	105	-5.28	11598	0.12	116	7.67	3899	0.04	89	1.61	7679
IFNA14	-1	0.765191299	-0.1	103	-5.29	11599	0.03	137	2.91	6548	-0.05	97	-2.28	9834
GGA1_t2	1.04	0.209936156	-0.06	136	-5.29	11600	0.1	154	9.94	2880	0.04	114	2.82	7029
KRT15	-1.05	0.030487658	-0.08	113	-5.3	11601	-0.04	124	-3.21	10607	-0.22	117	-14.97	14373
GPR55	-1.02	0.171835084	-0.07	128	-5.3	11602	-0.18	146	-17.65	15690	-0.07	126	-5.39	11194
MDP1	-1.33	1.95E-10	-0.14	88	-5.3	11603	-0.29	80	-8.81	13440	-0.29	110	-17.66	14916
EMC6	-1.19	3.79E-06	-0.09	111	-5.3	11604	-0.22	124	-16.57	15522	-0.18	104	-9.88	12888
TMEM219	-1.1	0.001732126	-0.11	100	-5.31	11605	-0.08	84	-2.6	10216	-0.14	100	-7.22	11927
CDH16	1.06	0.462312307	-0.12	95	-5.31	11606	-0.05	108	-2.93	10431	-0.17	88	-6.44	11618
ISLR2	-1.04	0.024791202	-0.09	111	-5.32	11607	-0.19	125	-14.36	15072	-0.06	103	-3.02	10188
TRIP6	-1	0.951350651	-0.09	112	-5.32	11608	-0.02	129	-1.69	9612	-0.04	107	-2.23	9812
WDR73	1.07	0.059851817	-0.06	139	-5.32	11609	0.07	121	5.07	5287	0.01	120	0.93	8061
SMG6_t1	-1.15	2.63E-11	-0.09	111	-5.32	11610	-0.08	134	-6.98	12637	NA	NA	NA	NA
BTBD16	-1.55	4.80E-08	-0.22	74	-5.33	11611	-0.19	88	-7.26	12764	-0.18	84	-6.08	11471
LRRC3	-1.06	0.001007266	-0.07	128	-5.33	11612	-0.2	148	-19.22	15892	-0.06	144	-5.67	11307
STAR	1.08	0.056142456	-0.07	131	-5.33	11613	0.07	134	5.91	4834	-0.08	160	-9.17	12647
ATRNL1	-1.03	0.577816191	-0.06	141	-5.33	11614	-0.01	138	-0.89	9069	-0.1	127	-7.32	11962
EGFL8_t1	-1.04	0.010749088	-0.1	101	-5.33	11615	NA	NA	NA	NA	-0.08	93	-3.34	10330
PRDM5	-1.03	0.055222272	-0.09	109	-5.34	11616	-0.02	114	-0.98	9132	-0.16	114	-10.43	13067
RPL3_t1	-1.04	0.401207983	-0.13	90	-5.34	11617	-0.1	90	-3.91	11043	-0.11	113	-7.05	11861
SERPINA7	-2.95	2.72E-12	-0.08	118	-5.34	11618	-0.28	114	-17.92	15722	-0.14	133	-11.25	13325
SEC24A	-1.15	0.032971973	-0.05	152	-5.34	11619	-0.13	114	-8.08	13139	0.06	123	4.58	6132
YES1	-1.09	9.40E-05	-0.06	133	-5.34	11620	-0.01	135	-1.01	9153	0	139	0.33	8413
PQLC3	-1.33	7.11E-08	-0.07	123	-5.35	11621	0.02	109	1.04	7785	-0.22	166	-25.81	16016
ZNF540	-1.04	0.164965405	-0.07	126	-5.35	11622	0.01	127	0.55	8104	-0.02	126	-1.36	9369
USP38	-1.18	5.44E-09	-0.07	130	-5.35	11623	-0.12	125	-8.97	13488	-0.04	126	-3.33	10326
MYOZ3	1.04	0.002242753	-0.07	132	-5.35	11624	0.02	151	1.74	7322	0.04	146	3.87	6489
PIP5KL1	-1.02	0.195900116	-0.09	108	-5.35	11625	-0.15	113	-9.47	13689	-0.03	104	-1.41	9396
Sep-01	-1.12	4.50E-07	-0.16	84	-5.35	11626	-0.11	113	-6.99	12647	-0.32	100	-16.19	14626
MCF2	-1.01	0.686680792	-0.07	125	-5.35	11627	-0.11	134	-9.01	13507	-0.1	120	-6.74	11751
DAPK3	-1.04	0.080585391	-0.12	94	-5.36	11628	-0.07	115	-4.39	11318	-0.17	121	-12.39	13667
PCDH1_t2	-1.04	0.037759032	-0.1	103	-5.36	11629	-0.11	122	-7.78	13021	-0.08	108	-4.68	10930
FAM102B	-1.17	0.002490171	-0.06	141	-5.36	11630	0.02	140	1.43	7538	-0.12	139	-10.92	13228
GP2	1.12	0.021021974	-0.1	104	-5.37	11631	-0.05	135	-4.24	11233	-0.04	160	-4.59	10885
SLC25A32	-1.08	0.169590861	-0.06	139	-5.37	11632	0.01	125	1.05	7774	-0.02	145	-1.93	9684
KRT6C	-1.06	0.000267293	-0.09	110	-5.37	11633	-0.26	119	-17.95	15729	-0.1	122	-6.98	11837
FAM125B_t2	-1.06	0.047768719	-0.07	128	-5.37	11634	-0.08	120	-5.61	11981	-0.1	128	-7.81	12165
PHF7	1.05	0.07662963	-0.08	118	-5.37	11635	-0.03	111	-1.66	9592	0.02	104	1.26	7870
MXI1	-1.2	2.31E-05	-0.06	140	-5.37	11636	-0.16	121	-11.52	14378	-0.02	128	-1.83	9626
DCPS	-1.69	4.01E-09	-0.12	93	-5.37	11637	-0.2	88	-7.49	12868	-0.19	102	-9.7	12809
HFE2	-1.17	0.116078769	-0.07	128	-5.37	11638	-0.22	120	-15.43	15297	0.01	112	0.72	8183
SIAE_t1	-1.29	3.76E-06	-0.07	126	-5.38	11639	-0.14	108	-8.15	13174	NA	NA	NA	NA
LAMTOR1	1	0.99155756	-0.08	114	-5.38	11640	0.11	120	7.91	3788	-0.11	118	-7.75	12139
TSPAN16	-1	0.963807361	-0.1	106	-5.38	11641	-0.05	99	-2.39	10083	-0.18	87	-6.64	11713
KATNAL1_t1	-1.13	0.000457262	-0.06	136	-5.38	11642	NA	NA	NA	NA	NA	NA	NA	NA
EFNA2	-1.04	0.006994901	-0.08	120	-5.38	11643	-0.14	126	-10.78	14170	-0.1	120	-7.09	11877.5
SCRN2	-1.16	0.001857688	-0.11	97	-5.38	11644	-0.19	137	-16.65	15537	-0.15	103	-7.88	12187
TMPRSS13	-1.03	0.093739831	-0.08	115	-5.38	11645	-0.1	135	-8.54	13341	-0.1	119	-6.67	11723
C1QTNF6	1.02	0.581392326	-0.08	119	-5.38	11646	-0.07	119	-5.13	11734	0.06	141	5.82	5537
ASMT	-1.07	0.000131724	-0.12	95	-5.39	11647	-0.1	102	-5.16	11750	-0.1	103	-5.3	11154
NDUFV2	-1.22	1.81E-05	-0.13	93	-5.39	11648	-0.15	84	-5.26	11806	-0.12	126	-9.45	12741
CLDN16	1.02	0.120644369	-0.06	141	-5.39	11649	0.04	143	3.99	5908	0.02	152	2.08	7405
ROPN1B	1	0.989700094	-0.09	110	-5.39	11650	-0.09	116	-5.87	12124	-0.04	129	-3.08	10219
HRASLS	-1.03	0.359124024	-0.09	110	-5.4	11651	0.03	115	1.64	7394	-0.05	114	-3.11	10226
IRF2BPL	-1.08	0.024557769	-0.08	122	-5.4	11652	-0.15	118	-10.28	13993	-0.17	120	-11.85	13503
NFIX_t1	-1.08	0.178231518	-0.07	123	-5.41	11653	NA	NA	NA	NA	-0.09	142	-8.09	12262
TCN2	-1.22	1.92E-10	-0.09	109	-5.41	11654	-0.16	158	-17.02	15594	-0.3	120	-20.77	15457
MITF_t2	-1.11	0.0357647	-0.05	149	-5.42	11655	NA	NA	NA	NA	-0.03	134	-2.27	9832
KCNMA1_t2	1	0.931905147	-0.06	147	-5.42	11656	-0.15	152	-15.06	15211	-0.01	140	-0.77	9069
CD248	-1.15	0.024581659	-0.1	104	-5.42	11657	-0.02	98	-1.07	9197	-0.17	100	-8.69	12487
MYO7A_t1	-1.11	7.52E-05	-0.08	117	-5.42	11658	NA	NA	NA	NA	NA	NA	NA	NA
AS3MT	-1.22	0.042225939	-0.06	136	-5.42	11659	-0.22	121	-15.68	15338	0	121	-0.05	8648
LY75	-1.24	2.85E-06	-0.07	128	-5.43	11660	0.05	110	2.82	6602	-0.15	156	-15.38	14454
SH2D3A	-1.05	0.107208375	-0.1	104	-5.43	11661	-0.07	119	-4.79	11531	-0.19	100	-9.4	12716
FBXO15	-1.12	0.002783005	-0.11	101	-5.43	11662	-0.16	69	-3.12	10559	-0.09	101	-4.34	10774
PTPN7_t1	-1.11	0.010590572	-0.09	112	-5.43	11663	0.04	128	3.11	6431	NA	NA	NA	NA
WDR44	-1.3	8.26E-07	-0.09	110	-5.43	11664	-0.09	120	-6.32	12333	-0.12	132	-9.79	12845
UBE3A	-1.16	0.001384747	-0.07	130	-5.43	11665	-0.12	116	-7.72	12998	-0.03	139	-2.26	9827
ARHGEF25	-1.2	1.82E-07	-0.1	104	-5.43	11666	-0.02	107	-1.08	9207	-0.28	109	-16.29	14643
MAP3K2	-1.1	0.008961199	-0.05	164	-5.43	11667	-0.05	148	-5.25	11799	0.03	144	3.28	6788

CIB1_t1	-1.02	0.594414998	-0.12	94	-5.44	11668	NA	NA	NA	NA	0	139	-0.23	8752
CDH8	1.01	0.561031977	-0.06	145	-5.44	11669	-0.14	138	-12.08	14531	-0.03	123	-2.01	9707
DCAF5	-1	0.945498053	-0.06	148	-5.44	11670	-0.1	132	-8.12	13162	-0.06	142	-5.69	11312
FAHD2A	-1.8	6.35E-19	-0.3	68	-5.44	11671	-0.34	106	-19.09	15878	-0.37	100	-18.66	15113
ALOX5AP	-1.36	7.28E-06	-0.1	104	-5.44	11672	0.03	96	1.45	7521	-0.25	114	-16.3	14647
ZNF295_t1	-1.13	1.26E-08	-0.06	138	-5.45	11673	NA	NA	NA	NA	-0.07	133	-6.2	11522
ZPBP	1.04	0.001490805	-0.07	127	-5.45	11674	-0.05	93	-2.15	9935	0.01	85	0.21	8486
KLK2_t2	1.05	0.000393106	-0.05	159	-5.45	11675	-0.08	146	-7.41	12833	0	122	0.09	8557
TNFRSF9	1.06	0.061369235	-0.06	147	-5.45	11676	0.07	139	6.49	4520	-0.1	160	-11.46	13384
TRIM34_t1	-1.12	1.00E-05	-0.07	125	-5.45	11677	0	80	0.09	8407	-0.16	88	-5.95	11424
SZT2	-1.04	0.293235607	-0.07	126	-5.45	11678	-0.04	143	-4.13	11169	-0.08	126	-6.37	11598
FAM43A	-1.05	0.2161286	-0.08	118	-5.45	11679	0.07	120	4.7	5500	-0.11	145	-10.87	13205
CABP4	-1.03	0.122048886	-0.06	145	-5.45	11680	-0.12	135	-10.2	13953	-0.05	132	-4.4	10798
COBL_t1	1.02	0.101681126	-0.07	126	-5.46	11681	-0.11	127	-8.27	13223	NA	NA	NA	NA
ASTE1	-1.04	0.130666663	-0.09	113	-5.46	11682	-0.1	100	-5.23	11793	-0.05	115	-3.25	10291
NPHP1_t1	1.02	0.043252178	-0.07	132	-5.46	11683	NA	NA	NA	NA	0.02	103	0.88	8091
VPS4A	-1.13	1.87E-08	-0.09	114	-5.46	11684	-0.16	150	-15.84	15370	-0.18	106	-10.07	12951
SHANK1	-1.01	0.4035179	-0.11	100	-5.47	11685	-0.11	103	-5.9	12133	-0.03	99	-1.49	9442
EMP2	-1.16	0.011754578	-0.07	132	-5.47	11686	-0.05	134	-4	11100	-0.07	159	-7.27	11949
ROPN1L	-1.11	0.000962689	-0.18	80	-5.47	11687	-0.21	93	-8.89	13464	-0.29	111	-17.89	14961
FAM71A	1.01	0.320396894	-0.11	100	-5.48	11688	-0.02	92	-0.8	9015	0	80	0.04	8597
GPRC5C	1.03	0.63524736	-0.09	108	-5.48	11689	-0.1	156	-10.79	14174	-0.06	94	-2.65	10014
RMND5A	-1.06	0.08136914	-0.06	136	-5.48	11690	-0.01	130	-0.97	9127	-0.06	153	-6.09	11478
CDC5L	-1.03	0.610064349	-0.07	130	-5.49	11691	-0.05	147	-5	11659	0.08	151	8.11	4579
LBX1	-1.18	0.007479863	-0.12	97	-5.49	11692	-0.17	100	-8.34	13257	-0.14	104	-7.54	12045
CDC42EP1	-1.16	0.000540114	-0.11	100	-5.49	11693	-0.03	120	-2.44	10115	-0.14	108	-7.97	12229
FAM69C	1.03	0.471599569	-0.1	104	-5.49	11694	-0.09	110	-5.11	11728	-0.09	120	-6.48	11632
RSAD1	1.04	0.252352383	-0.09	114	-5.5	11695	-0.04	108	-2.36	10055	0.02	110	1.48	7763
FXR2	-1.16	8.17E-12	-0.1	104	-5.5	11696	-0.22	115	-14.2	15030	-0.2	124	-15.1	14396
FBXO27	1.15	0.000153717	-0.06	136	-5.5	11697	0.03	135	2.17	7027	0.09	126	6.52	5224
WFIKKN2_t1	-1.02	0.204332433	-0.08	120	-5.51	11698	NA	NA	NA	NA	-0.07	120	-4.82	10991
MRM1	-1.05	0.217262224	-0.11	100	-5.51	11699	-0.17	107	-9.79	13813	-0.13	111	-8.13	12278
RGS13	1.01	0.561826675	-0.07	130	-5.51	11700	0.19	112	11.97	2134	-0.17	119	-11.51	13404
DEFB125	-1.01	0.276916007	-0.1	105	-5.51	11701	-0.08	106	-4.49	11381	-0.11	124	-8.47	12417
CCR6	-1.07	0.193947817	-0.06	142	-5.51	11702	0.13	135	10.94	2499	-0.09	153	-8.95	12575
PPP2R1B_t2	-1.02	0.078875653	-0.06	136	-5.51	11703	-0.07	138	-5.79	12083	-0.03	146	-3.19	10265
FASLG_t1	-1.08	0.000149676	-0.1	106	-5.52	11704	NA	NA	NA	NA	-0.18	125	-13.75	14040
TRAFD1	1	0.817134277	-0.07	124	-5.52	11705	-0.02	137	-2.08	9884	-0.23	138	-20.56	15421
KCNA3	-1.05	2.46E-06	-0.07	132	-5.52	11706	-0.1	129	-7.83	13040	-0.27	127	-20.9	15484
OR2B2	-1.1	7.80E-08	-0.1	104	-5.52	11707	-0.24	99	-11.71	14427	-0.09	107	-5.23	11129
VAMP8	-1.29	3.16E-07	-0.08	118	-5.52	11708	-0.07	113	-4.16	11184	-0.23	107	-13.35	13935
RFNG	1.03	0.481577993	-0.09	115	-5.53	11709	-0.08	107	-4.52	11396	-0.04	111	-2.18	9786
GPR149	1.06	2.07E-06	-0.09	115	-5.53	11710	0	65	0.02	8451	-0.03	77	-0.75	9055
FAM174B	-1.08	0.211498704	-0.07	133	-5.53	11711	0.07	128	5.21	5216	-0.05	127	-4.22	10722
MATK	-1.1	2.17E-06	-0.16	84	-5.53	11712	-0.01	113	-0.47	8799	-0.28	100	-13.92	14084
GALNT3	1	0.956186268	-0.06	147	-5.54	11713	0.06	144	6.07	4741	-0.05	124	-3.92	10599
CHRM2_t2	1.03	0.15235545	-0.08	121	-5.54	11714	-0.08	130	-6.41	12372	-0.02	155	-2.56	9982
LGMN	-1.3	8.63E-10	-0.08	118	-5.54	11715	-0.17	105	-9.22	13598	-0.22	114	-14.4	14216
CDC34	-1.01	0.852097657	-0.06	140	-5.54	11716	-0.08	116	-5.11	11726	0.02	108	1.23	7889
PLLP	1.01	0.838553034	-0.1	108	-5.55	11717	-0.15	137	-12.63	14674	0.01	120	0.66	8221
THAP1	-1.02	0.689170069	-0.08	121	-5.55	11718	0.05	112	2.91	6549	0.03	153	3.25	6805
FMO4	-1.37	3.13E-05	-0.1	104	-5.55	11719	-0.18	101	-9.16	13574	-0.1	122	-7	11845
SYT14	1.04	0.037251322	-0.06	137	-5.55	11720	-0.1	116	-6.61	12467	0.04	143	3.82	6512
LGR6	-1.13	5.89E-06	-0.09	110	-5.55	11721	-0.09	98	-4.24	11235	-0.14	102	-7.45	12014
TIGIT	-1.15	0.006431505	-0.09	112	-5.56	11722	-0.03	131	-2.52	10162	-0.23	141	-21.28	15553
SPINT1	-1.14	0.048401827	-0.09	114	-5.56	11723	0.01	113	0.39	8213	-0.21	124	-15.65	14508
ABHD8	1.01	0.784922958	-0.07	126	-5.56	11724	-0.04	128	-2.98	10464	-0.09	117	-6.11	11482
SDHAF2	-1	0.993883411	-0.09	114	-5.56	11725	-0.03	124	-1.95	9774	-0.1	134	-8.5	12427
MAP2K6	1	0.98183922	-0.08	124	-5.56	11726	-0.05	110	-2.85	10381	-0.03	107	-1.8	9608
SPRR1B	1	0.958162637	-0.11	100	-5.56	11727	-0.14	129	-11.21	14302	-0.04	129	-3.18	10253
CYBA	-1.64	5.85E-15	-0.2	78	-5.56	11728	-0.05	95	-2.14	9928	-0.36	103	-19.04	15181
MGAT5B_t1	1.03	0.001109306	-0.08	120	-5.57	11729	NA	NA	NA	NA	0.07	127	5.56	5650
AUH	-1.05	0.384264208	-0.08	121	-5.58	11730	-0.1	127	-7.7	12990	0.05	142	4.55	6151
EFCAB3	1.01	0.330048847	-0.1	108	-5.58	11731	-0.09	113	-5.73	12046	-0.04	107	-2.44	9915
GYLTL1B	-1.21	5.66E-09	-0.09	113	-5.58	11732	-0.26	99	-12.65	14679	-0.09	110	-5.55	11257
SULT1C4	1.03	0.1772799	-0.08	121	-5.58	11733	0.11	122	8.01	3743	-0.03	110	-1.7	9551
RBPMS	-1.29	6.24E-06	-0.08	118	-5.58	11734	-0.04	99	-1.92	9757	-0.22	103	-11.9	13513
CXorf48	-1.02	0.501222016	-0.07	134	-5.58	11735	-0.09	116	-5.67	12014	0	131	-0.07	8662
CORO6	-1.07	0.003413266	-0.09	115	-5.58	11736	0.02	131	1.67	7377	-0.17	121	-12.35	13658
CTRB2	-1.13	1.81E-06	-0.17	82	-5.58	11737	-0.15	93	-6.63	12477	-0.17	84	-5.68	11311
CASP5	-1.34	2.58E-06	-0.09	110	-5.58	11738	-0.04	111	-2.45	10122	-0.25	117	-16.42	14670
WDR45	1.1	0.005051195	-0.08	119	-5.59	11739	-0.1	108	-5.8	12089	0.05	115	3.17	6846
ANXA13_t1	-1.64	5.22E-05	-0.07	133	-5.59	11740	NA	NA	NA	NA	-0.13	115	-8.25	12340
TCP10	-1.27	0.011420633	-0.15	88	-5.59	11741	-0.11	93	-4.91	11604	-0.17	108	-9.78	12843
DNTT	1.01	0.257283692	-0.1	108	-5.59	11742	-0.01	115	-0.79	9011	-0.08	121	-5.47	11229
MOCOS	-1.22	0.006825728	-0.16	84	-5.59	11743	-0.17	80	-5.22	11784	-0.05	89	-1.92	9676
DNAL4	-1.04	0.026478554	-0.08	120	-5.6	11744	-0.15	111	-9.13	13569	-0.08	128	-6.62	11706
ECH1	-1.42	3.32E-07	-0.14	89	-5.61	11745	-0.17	93	-7.19	12733	-0.24	78	-6.85	11793
LGALS9	-1.13	2.76E-05	-0.1	106	-5.61	11746	-0.1	131	-7.72	12996	-0.29	131	-23.4	15815
SCT	-1.04	0.001006186	-0.07	127	-5.61	11747	-0.13	80	-3.78	10967	-0.14	104	-7.72	12126
AFG3L2_t1	-1.1	0.018399267	-0.09	111	-5.61	11748	-0.21	117	-14.12	15013	NA	NA	NA	NA
IFT140	1.01	0.827226001	-0.1	108	-5.61	11749	0.02	141	2.01	7135	-0.08	126	-5.96	11427
NKX2-8	-1.36	7.52E-08	-0.07	126	-5.62	11750	-0.15	125	-11.11	14273	-0.13	109	-7.44	12009
AQP6	1.21	0.020332771	-0.09	113	-5.62	11751	-0.16	120	-11.01	14241	-0.12	119	-7.98	12231
MPO	-1.14	0.00171572	-0.08	122	-5.62	11752	-0.04	114	-2.37	10065	-0.06	110	-3.44	10371
ATP5D	-1.23	2.74E-06	-0.19	80	-5.62	11753	-0.22	73	-5.01	11665	-0.16	88	-6.19	11519
IL1RAPL2	1.02	0.050335244	-0.09	112	-5.62	11754	-0.08	135	-6.56	12443	0.02	122	1.76	7592
MFI2_t2	-1.02	0.174587947	-0.07	133	-5.63	11755	-0.01	122	-0.47	8796	-0.15	125	-11.02	13266
SLC36A3	-1.07	0.000129683	-0.09	111	-5.63	11756	-0.16	124	-11.88	14469	-0.19	121	-13.56	13988
DNAJA3	-1.08	0.026653915	-0.12	98	-5.63	11757	-0.06	124	-4.1	11152	-0.02	115	-1.5	9448
ADRA1D	-1.07	0.000148946	-0.07	128	-5.63	11758	-0.16	139	-14.18	15023	-0.12	114	-7.74	12135
KCTD8	1.01	0.481577993	-0.08	121	-5.63	11759	-0.09	154	-9.47	13690	-0.08	116	-5.5	11240
CPNE7	-1.01	0.674174923	-0.08	118	-5.63	11760	-0.17	128	-12.99	14749	-0.12	100	-6.22	11537
TRIP11_t1	1	0.943228618	-0.07	127	-5.63	11761	0	119	-0.03	8512	NA	NA	NA	NA
MRPL20_t1	-1.16	0.00039095	-0.08	121	-5.63	11762	NA	NA	NA	NA	-0.11	115	-7.21	11923
GZMM	-1.28	5.36E-16	-0.24	73	-5.63	11763	-0.16	107	-9.32	13626	-0.33	91	-13.46	13968
STX18_t1	1.02	0.555255459	-0.08	120	-5.63	11764	NA	NA	NA	NA	0	131	0.31	8430
CGN	-1.1	0.150730838	-0.07	128	-5.63	11765	-0.11	120	-7.58	12919	-0.06	144	-5.63	11291
HMGN5	-1	0.945722914	-0.09	115	-5.64	11766	-0.09	111	-5.37	11854	0.04	128	3.42	6707
EHMT1	1.03	0.379584963	-0.09	112	-5.64	11767	0.13	113	7.94	3777	-0.08	109	-4.91	11019
GEMIN8	1.09	0.000137346	-0.07	136	-5.64	11768	-0.02	131	-1.76	9660	-0.07	132	-6.05	11456
IP6K2_t2	1.03	0.523935866	-0.09	113	-5.64	11769	NA	NA	NA	NA	NA	NA	NA	NA
PTK7	-1.01	0.808853652	-0.1	109	-5.65	11770	-0.02	136	-2.07	9871	-0.14	125	-10.86	13201
SPRED2_t1	-1.08	0.082963747	-0.07	127	-5.65	11771	NA	NA	NA	NA	NA	NA	NA	NA
PHF12	-1.05	0.000168435	-0.07	132	-5.66	11772	-0.14	122	-9.99	13879	-0.08	125	-5.78	11354
CAPN11	1	0.93368767	-0.08	122	-5.66	11773	0.05	128	4.27	5735	-0.13	107	-7.35	11977

FILIP1L	-1.85	5.38E-17	-0.17	83	-5.66	11774	-0.07	119	-4.9	11598	-0.23	126	-17.71	14929
RER1	1.01	0.835072448	-0.07	128	-5.66	11775	-0.02	127	-1.83	9713	-0.05	136	-4.37	10780
GLRX5	-1.05	0.366092213	-0.09	112	-5.66	11776	-0.16	109	-9.17	13576	-0.02	140	-1.46	9424
CLEC14A	-1.2	0.003049567	-0.07	127	-5.66	11777	-0.03	154	-3.16	10577	-0.08	126	-5.79	11357
CHRNB1	-1.01	0.611078394	-0.05	160	-5.66	11778	-0.11	143	-10.35	14021	-0.1	150	-10.02	12934
BTBD7_t2	-1.11	8.93E-10	-0.08	117	-5.67	11779	NA	NA	NA	NA	-0.08	119	-5.64	11293
NEFM	1.04	0.066935631	-0.07	128	-5.67	11780	-0.02	95	-0.76	8983	0	125	-0.17	8719
PI16	-1.08	0.005463044	-0.09	114	-5.67	11781	-0.04	116	-2.64	10241	-0.19	112	-11.66	13443
OPA3	-1.13	0.000323899	-0.06	139	-5.67	11782	-0.12	147	-12.06	14522	-0.12	156	-12.29	13638
CALCOCO2	-1.06	0.113839553	-0.07	133	-5.67	11783	-0.05	128	-3.6	10874.5	-0.01	119	-0.52	8932
POLL	-1.07	0.000277796	-0.11	104	-5.68	11784	-0.15	123	-10.97	14227	-0.09	111	-5.42	11210
RNF170	-1.05	0.294833441	-0.08	124	-5.68	11785	-0.06	148	-5.94	12152	0.02	126	1.17	7933
KRT7	-1.53	9.57E-06	-0.16	86	-5.68	11786	-0.12	120	-8.38	13280	-0.26	94	-11.52	13405
DTX3_t1	-1.02	0.172584939	-0.11	104	-5.68	11787	NA	NA	NA	NA	-0.13	120	-8.76	12514
UNC5CL	-1.31	0.000695908	-0.08	125	-5.68	11788	-0.06	112	-3.5	10796	-0.06	120	-4.19	10713
SEPW1	-1.64	1.66E-11	-0.11	100	-5.68	11789	-0.03	125	-2.21	9971	-0.21	110	-12.83	13799
POLR2F	-1.03	0.398840457	-0.09	114	-5.68	11790	-0.08	150	-7.57	12913	0.03	118	1.98	7465
RNMTL1	-1.15	4.17E-05	-0.08	124	-5.69	11791	-0.17	118	-11.64	14409	-0.16	100	-8.14	12282
FAM216B	-1.03	0.00722667	-0.06	153	-5.69	11792	-0.04	139	-3.24	10626	-0.09	145	-8.13	12281
ATAD1_t1	-1.01	0.834891847	-0.07	132	-5.69	11793	NA	NA	NA	NA	0.06	131	4.98	5938
MYH14	-1.06	0.015755869	-0.09	110	-5.69	11794	-0.11	123	-8.29	13236	-0.09	124	-6.32	11579
PLCD4	1.1	0.002076774	-0.08	120	-5.69	11795	0.12	124	8.84	3360	-0.02	120	-1.72	9562
OXNAD1_t1	-1.07	0.070087451	-0.06	143	-5.69	11796	NA	NA	NA	NA	0.03	126	2.23	7337
IRS2_t1	-1.39	0.000253119	-0.07	126	-5.69	11797	NA	NA	NA	NA	-0.1	129	-8.18	12308
LYNX1_t3	-1.27	8.10E-07	-0.14	91	-5.69	11798	-0.15	104	-8.26	13218	-0.14	93	-6	11439
LONRF2	-1.24	0.029288311	-0.06	140	-5.7	11799	0	146	0.12	8388	-0.12	152	-12.03	13554
SPDEF	-1	0.94457891	-0.1	109	-5.7	11800	-0.08	102	-4.03	11113	-0.1	100	-4.76	10966
PLEKHG4B_t1	1.03	0.512822475	-0.07	129	-5.7	11801	NA	NA	NA	NA	-0.03	128	-2.13	9768
ZNF438	-1.23	4.72E-05	-0.1	110	-5.7	11802	-0.03	108	-1.49	9482	-0.17	106	-9.79	12846
NSMCE1	-1.02	0.736216877	-0.11	100	-5.7	11803	-0.07	95	-3.16	10578	-0.06	106	-3.44	10372
LDB1	-1.03	0.05128268	-0.07	134	-5.7	11804	-0.16	122	-11.19	14291	-0.11	91	-4.53	10859
ST6GALNAC5	-1.03	0.083806694	-0.07	127	-5.71	11805	-0.05	143	-4.96	11635	-0.04	129	-2.94	10153
NFAT5_t1	-1.25	4.08E-07	-0.09	117	-5.71	11806	-0.04	99	-2	9823	-0.13	140	-12.12	13583
STK17B	-1.47	5.56E-09	-0.06	143	-5.71	11807	0.03	144	2.87	6565	-0.22	128	-17.03	14804
TMEM110	1.02	0.677316458	-0.07	132	-5.71	11808	-0.15	129	-11.76	14437	0.08	153	8.7	4341
FGFR1OP2	-1.08	0.011568044	-0.05	155	-5.72	11809	0.04	126	3.31	6309	-0.15	135	-12.4	13669
NUDT8	-1.06	0.355326737	-0.13	93	-5.72	11810	-0.1	120	-6.81	12570	-0.09	140	-8.17	12303
SOX18	-1.26	5.60E-07	-0.1	110	-5.72	11811	-0.13	114	-8.33	13255	-0.17	133	-13.77	14045
CLK1	-1.32	1.28E-08	-0.11	102	-5.72	11812	-0.08	102	-4.38	11300	-0.17	114	-10.87	13204
WDR25_t1	-1.03	0.038436978	-0.13	95	-5.72	11813	NA	NA	NA	NA	NA	NA	NA	NA
TSHZ1	-1.08	0.001857688	-0.08	122	-5.72	11814	-0.08	119	-5.22	11783	-0.12	160	-13.5	13977
CACNA1I	1.06	0.039117236	-0.07	128	-5.73	11815	0.01	118	0.39	8218	-0.03	115	-2.12	9766
VIM	-1.4	0.000323939	-0.07	128	-5.73	11816	0.08	140	6.93	4284	-0.18	127	-13.7	14024
ROGDI	-1.08	0.001080753	-0.14	90	-5.73	11817	-0.14	133	-11.39	14340	-0.15	154	-16.03	14597
VASP	-1.26	5.31E-06	-0.05	155	-5.73	11818	0.04	140	4.02	5895	-0.25	103	-13.39	13951
NCEH1	-1.23	0.003970441	-0.05	156	-5.74	11819	-0.01	143	-0.7	8938	-0.18	138	-15.89	14558
WDR59	-1.15	0.000211509	-0.09	111	-5.74	11820	-0.09	110	-5.58	11957	-0.18	124	-13.38	13948
RASSF3	-1.01	0.450113909	-0.06	152	-5.74	11821	-0.15	125	-11.36	14335	-0.1	142	-9.37	12706
ATG16L1	-1.06	0.013388658	-0.08	122	-5.74	11822	-0.03	131	-2.6	10221	-0.16	171	-19.95	15317
SMARCAD1	-1.05	0.328988035	-0.07	133	-5.75	11823	0.05	131	4.07	5868	0.04	127	2.95	6958
CTXN3	1	0.915841673	-0.05	155	-5.75	11824	-0.05	125	-3.92	11053	-0.14	120	-9.82	12865
GBGT1	-1.05	4.73E-05	-0.11	104	-5.75	11825	-0.13	118	-9.02	13517	-0.17	130	-13.24	13907
SEMA3E	1.02	0.367748286	-0.07	137	-5.75	11826	0.01	127	0.79	7949	0.01	135	0.64	8232
BCL3	-1.73	2.13E-12	-0.13	94	-5.75	11827	-0.13	120	-9.39	13658	-0.17	120	-12.17	13599
TP53INP1	-1.3	0.007844866	-0.06	148	-5.75	11828	-0.02	125	-1.26	9335	-0.07	143	-6.16	11501
MAPK8	-1.02	0.244465586	-0.08	124	-5.75	11829	-0.14	103	-7.68	12971	-0.01	81	-0.29	8787
DIABLO	1.02	0.412260574	-0.08	125	-5.75	11830	0.04	126	2.81	6606	-0.13	134	-10.94	13233
HBM	-1.27	3.30E-08	-0.17	84	-5.76	11831	-0.19	70	-3.89	11034	-0.14	88	-5.26	11144
FCHO1	-1.1	0.008559901	-0.11	102	-5.77	11832	-0.03	129	-2.49	10147	-0.22	95	-9.8	12857
CASP4_t1	-1.42	4.86E-07	-0.11	103	-5.77	11833	NA	NA	NA	NA	-0.28	135	-23.44	15822
NDUFB10	-1.13	0.005352894	-0.15	88	-5.77	11834	-0.09	68	-1.55	9517	-0.07	102	-3.62	10442
MTHFR_t1	-1.18	0.000147493	-0.06	140	-5.77	11835	NA	NA	NA	NA	-0.08	125	-6.16	11502
GPR63	1.06	1.53E-05	-0.06	140	-5.77	11836	0	127	-0.31	8687	0.04	139	3.19	6834
WDR17	-1.02	0.120098397	-0.06	139	-5.78	11837	-0.05	135	-4.39	11315	0	145	0.41	8368
TOR1A	-1.12	0.000184748	-0.08	125	-5.78	11838	-0.15	137	-12.65	14678	-0.15	118	-9.95	12913
MAP3K8	-1.19	0.010200806	-0.07	129	-5.78	11839	0.03	110	1.79	7286	-0.09	124	-6.81	11780
TUT1	-1.15	4.81E-05	-0.19	80	-5.78	11840	-0.16	100	-8.23	13206	-0.18	72	-3.97	10626
TCERG1L	1.02	0.173452683	-0.07	133	-5.78	11841	-0.11	120	-7.69	12978	0.01	113	0.32	8426
GJD2	-1.02	0.064807563	-0.16	87	-5.78	11842	-0.13	67	-2.19	9959	-0.06	75	-1.54	9468
TRIM35_t1	-1.09	0.150448931	-0.07	130	-5.79	11843	NA	NA	NA	NA	-0.15	138	-12.76	13781
MUL1	-1.07	0.00751639	-0.09	111	-5.79	11844	-0.16	114	-10.47	14054	-0.11	144	-10.46	13080
KRTCAP3	-2.17	7.57E-17	-0.19	80	-5.79	11845	-0.17	100	-8.68	13390	-0.28	104	-14.86	14342
CRAT	-1.13	0.001429048	-0.12	99	-5.79	11846	-0.14	122	-10.07	13909	-0.15	112	-9.56	12775
BCL2L10	-1.21	0.069763581	-0.09	113	-5.79	11847	-0.22	102	-11.44	14355	-0.06	94	-2.5	9957
TMEM11	-1.12	0.000217645	-0.1	109	-5.8	11848	0.04	138	3.45	6213	-0.12	127	-9.06	12610
PHLDA3	-1.27	0.000404513	-0.12	100	-5.8	11849	-0.12	138	-10.21	13963	-0.14	140	-12.79	13789
TAF7L	1.02	0.44612209	-0.08	120	-5.8	11850	-0.02	119	-1.57	9528	-0.01	116	-0.4	8851
PELI1	-1.24	4.98E-08	-0.08	127	-5.8	11851	-0.03	166	-3.68	10924	-0.16	151	-15.92	14566
MTMR8	1.03	0.031345173	-0.08	120	-5.81	11852	-0.06	101	-3.07	10512	-0.02	109	-0.97	9182
MADCAM1_t1	-1.05	0.030508052	-0.1	107	-5.81	11853	NA	NA	NA	NA	-0.14	103	-7.46	12016
DCAF6	-1.1	0.038506291	-0.11	104	-5.81	11854	-0.13	118	-8.77	13425	0.06	113	4.08	6384
KCNJ3	-1.15	0.059343298	-0.07	128	-5.81	11855	-0.07	144	-6.39	12366	-0.04	132	-3.18	10256
GNGT2	-1.16	2.06E-10	-0.11	104	-5.81	11856	-0.07	132	-5.51	11924	-0.32	107	-18.21	15023
RNF208	-1.09	2.88E-05	-0.14	91	-5.82	11857	-0.21	103	-10.99	14234	-0.12	130	-9.68	12804
CNTN6	1.01	0.44141545	-0.1	108	-5.82	11858	-0.02	101	-1.23	9316	0.01	100	0.27	8458
GALNT6	-1.08	0.005864647	-0.07	133	-5.82	11859	0.02	132	1.72	7330	-0.23	132	-18.99	15172
ARHGAP4	-1.27	0.000716877	-0.14	92	-5.82	11860	0.08	118	5.39	5131	-0.15	120	-10.2	12991
VPS13A_t2	1.05	0.211413162	-0.07	137	-5.82	11861	0.05	112	3.4	6245	0.09	130	7.38	4884
SCAF11	-1.06	0.142668166	-0.06	140	-5.83	11862	-0.02	147	-1.5	9494	-0.02	153	-1.91	9671
SETD1A	-1.03	0.169800182	-0.13	96	-5.83	11863	-0.02	134	-1.81	9694	-0.05	114	-2.96	10162
KDELC2	-1.05	0.003787602	-0.07	129	-5.83	11864	-0.15	158	-15.9	15394	-0.04	132	-3.61	10438
SLC24A6	-1.14	0.000226496	-0.09	116	-5.83	11865	-0.08	130	-6.79	12557	-0.17	117	-11.4	13375
OCIAD2	-1.3	0.005709727	-0.09	112	-5.83	11866	-0.11	108	-6.52	12417	-0.05	120	-3.54	10412
MRPS6	-1.15	0.017837971	-0.08	120	-5.83	11867	-0.05	103	-2.87	10392	-0.13	108	-7.57	12055
ST8SIA2_t1	-1.11	0.001535461	-0.07	135	-5.84	11868	-0.12	142	-10.83	14186	NA	NA	NA	NA
DNAJA4	-1.06	0.193453907	-0.06	141	-5.84	11869	0.06	114	4.14	5819	-0.09	133	-7.06	11863
Mar-01	-1.28	9.30E-12	-0.08	120	-5.84	11870	-0.01	136	-0.89	9072	-0.22	134	-18.5	15081
MORC1	-1.15	9.59E-10	-0.09	113	-5.85	11871	-0.22	141	-19.66	15949	-0.14	115	-9.13	12640
SYDE1	-1.05	0.000374747	-0.09	112	-5.85	11872	-0.12	110	-7.18	12728	-0.08	141	-7.6	12072
ITIH5_t2	1.01	0.511867809	-0.07	133	-5.85	11873	0.06	135	4.87	5409	-0.09	133	-7.46	12015
RND1	-1.49	4.17E-14	-0.12	100	-5.85	11874	-0.28	115	-18.05	15740	-0.12	110	-7.16	11903
RPS29_t1	-1.13	0.002651145	-0.11	104	-5.86	11875	NA	NA	NA	NA	NA	NA	NA	NA
NOVA2	1.02	0.333628464	-0.09	114	-5.86	11876	-0.03	122	-1.98	9810	-0.02	142	-2.23	9814
SFTPD	-1.16	2.74E-07	-0.09	112	-5.86	11877	-0.25	88	-9.4	13662	-0.24	68	-4.34	10772
CA6	-1	0.839618468	-0.1	110	-5.86	11878	-0.16	102	-8.15	13175	-0.09	104	-4.64	10910
PCDHGA8_t2	-1.17	0.000193652	-0.07	132	-5.86	11879	-0.05	139	-4.07	11134	-0.09	126	-7.11	11888

GPR114	-1.02	0.520138379	-0.09	118	-5.87	11880	-0.02	150	-2.47	10139	-0.16	124	-11.85	13502
ALAS2_t1	-1.37	1.60E-09	-0.1	109	-5.87	11881	-0.16	118	-10.94	14219	NA	NA	NA	NA
KCNH8	1.07	0.15343683	-0.08	124	-5.88	11882	-0.07	127	-5.48	11905	-0.08	135	-6.95	11825
FPR3	-1.38	2.29E-12	-0.09	115	-5.88	11883	-0.07	114	-4.43	11345	-0.26	106	-14.65	14283
KNG1_t1	-1.82	7.13E-06	-0.11	104	-5.88	11884	NA	NA	NA	NA	NA	NA	NA	NA
SNAPC4_t1	-1.12	0.017459473	-0.11	105	-5.88	11885	-0.01	114	-0.78	9002	NA	NA	NA	NA
MYBL2	-1.26	2.93E-07	-0.12	98	-5.88	11886	-0.14	124	-10.64	14116	-0.13	116	-8.74	12509
FEZ2	-1.15	0.005252562	-0.07	137	-5.89	11887	0	116	-0.07	8531	-0.12	140	-10.8	13181
MTCH2	-1.07	0.226748507	-0.08	121	-5.89	11888	0	140	-0.31	8685	-0.02	133	-1.75	9584
DHRSX	-1.23	1.29E-05	-0.08	122	-5.89	11889	-0.2	125	-15.29	15271	-0.14	135	-12.25	13630
HES1	-1.27	4.41E-09	-0.07	133	-5.89	11890	-0.18	113	-11.17	14288	-0.14	121	-9.75	12833
AP1G2	-1.46	2.37E-18	-0.16	88	-5.89	11891	-0.23	106	-12.67	14682	-0.25	154	-26.2	16059
EHF	1.06	0.272154985	-0.05	159	-5.89	11892	-0.05	127	-3.77	10966	-0.12	138	-10.68	13147
LGALS9C_t1	-1.47	1.93E-08	-0.11	106	-5.9	11893	-0.05	131	-4.13	11168	NA	NA	NA	NA
OPTC	-1.09	1.57E-07	-0.11	106	-5.9	11894	-0.13	132	-10.72	14141	-0.16	110	-9.78	12841
CCNL2_t1	1.05	0.427175827	-0.08	124	-5.9	11895	-0.01	124	-0.7	8941	NA	NA	NA	NA
KRT8_t1	-1.37	3.75E-08	-0.13	96	-5.9	11896	NA	NA	NA	NA	-0.18	118	-12.41	13672
SNRPD3	-1.06	0.142581728	-0.09	116	-5.91	11897	0.12	115	8	3745	-0.11	121	-7.89	12193
DNM2	-1.05	0.061632093	-0.1	108	-5.91	11898	-0.04	120	-3.03	10487	-0.15	146	-14.58	14260
EBP	-1.23	0.011129846	-0.1	108	-5.91	11899	-0.22	141	-20.12	16005	-0.05	100	-2.43	9907
ORC2	-1.08	0.017193766	-0.06	143	-5.91	11900	0.06	154	6.61	4446	-0.07	122	-4.79	10981
RNF166_t1	-1.11	7.79E-07	-0.08	128	-5.91	11901	NA	NA	NA		-0.17	133	-14.42	14219
SF3A2	-1.19	1.63E-06	-0.16	87	-5.92	11902	-0.12	95	-5.44	11897	-0.17	124	-12.61	13731
VRK3	-1.09	0.002281003	-0.12	100	-5.92	11903	-0.23	117	-15.6	15321	-0.17	119	-11.51	13403
KRTAP4-12	1.01	0.398550128	-0.09	113	-5.92	11904	-0.06	128	-4.8	11540	-0.05	128	-3.95	10614
ZNF839_t1	-1.02	0.552426506	-0.08	120	-5.93	11905	NA	NA	NA		-0.06	140	-5.18	11111
SORCS3	1.03	0.126604844	-0.07	130	-5.93	11906	0.12	131	10.09	2827	-0.1	135	-8.61	12460
CLEC4A	-1.39	7.65E-10	-0.09	113	-5.93	11907	-0.01	113	-0.66	8914	-0.25	115	-16.39	14664
CDH26	-1.02	0.331717974	-0.08	121	-5.93	11908	-0.01	131	-0.87	9052	-0.08	125	-6.06	11458
IFT74	-1.05	0.273779555	-0.1	107	-5.93	11909	-0.01	83	-0.27	8656	0.01	111	0.53	8301
TTC18	-1.12	0.114655869	-0.08	127	-5.93	11910	-0.15	96	-6.74	12530	-0.03	103	-1.83	9630
R3HDML	1	0.716724506	-0.08	120	-5.93	11911	-0.11	110	-6.58	12456	-0.06	117	-4.29	10752
AASDHPPT_t1	-1.07	0.213100004	-0.07	136	-5.94	11912	NA	NA	NA	NA	NA	NA	NA	NA
ARID2	1	0.999957322	-0.06	149	-5.94	11913	-0.03	142	-2.74	10304	0.09	127	7.04	5012
PHF1	-1.07	0.006373262	-0.1	110	-5.95	11914	-0.12	134	-10.26	13983	-0.17	100	-8.61	12462
DDX43	-1.21	0.006353922	-0.1	110	-5.95	11915	-0.02	109	-1.42	9444	-0.13	104	-6.86	11799
NSUN2	1.02	0.694708899	-0.09	120	-5.96	11916	-0.02	114	-1.3	9360	0.04	144	3.85	6502
ATXN10	1.04	0.247865224	-0.07	133	-5.96	11917	-0.01	139	-0.66	8912	0.01	146	0.88	8093
TBL1X	-1.02	0.659524142	-0.07	130	-5.96	11918	0.01	122	0.95	7836	-0.09	135	-7.32	11965
NQO2	-1.19	0.008043937	-0.14	92	-5.96	11919	-0.11	107	-6.33	12341	-0.03	90	-1.29	9340
LIPA	-1.47	2.04E-09	-0.09	120	-5.96	11920	-0.1	130	-8.39	13284	-0.2	147	-19.87	15306
CABP1	-1	0.956093741	-0.11	106	-5.96	11921	-0.11	110	-6.31	12328	-0.14	104	-7.29	11955
PGF	-1.04	0.184349337	-0.1	109	-5.96	11922	-0.04	146	-4.15	11180	-0.13	135	-10.88	13208
POLR1D_t2	-1.02	0.541254875	-0.08	127	-5.96	11923	0.04	132	2.91	6551	-0.08	123	-5.85	11386
CYP4F22	1.06	0.087672827	-0.09	119	-5.96	11924	-0.1	124	-7.41	12834	-0.07	108	-4.04	10656
TMEM205	-1.22	0.000304304	-0.17	86	-5.97	11925	-0.3	97	-14.02	14996	-0.16	84	-5.29	11153
NEU3	1.06	0.057354019	-0.08	123	-5.97	11926	-0.03	111	-1.63	9575	0.02	115	1.22	7900
SIPA1	-1.14	0.000248086	-0.08	121	-5.97	11927	-0.08	136	-7.3	12790	-0.16	113	-9.77	12838
GPR119	-1.16	1.24E-09	-0.12	100	-5.97	11928	-0.16	104	-8.91	13470	-0.18	100	-8.96	12580
OAS1	-1.31	3.30E-07	-0.11	105	-5.97	11929	-0.19	115	-12.06	14525	-0.22	136	-19.04	15182
ZNF114	1.01	0.422697907	-0.08	127	-5.97	11930	-0.11	125	-8.42	13295	-0.02	120	-1.27	9333
AP1M1	1	0.900356846	-0.11	105	-5.97	11931	-0.04	130	-3.21	10609	-0.03	119	-2.32	9853
FAM181A	-1.11	4.82E-08	-0.11	106	-5.97	11932	-0.11	112	-6.93	12620	-0.14	131	-11.36	13362
FAM107A	-1.03	0.134320545	-0.08	122	-5.98	11933	-0.05	125	-3.55	10836	-0.11	120	-7.5	12036
SFXN1	-1.1	7.38E-05	-0.06	145	-5.98	11934	-0.18	139	-16.08	15437	-0.01	140	-1.17	9277
SORCS1	-1.03	0.063036339	-0.08	122	-5.98	11935	-0.03	140	-2.85	10384	-0.06	136	-5.17	11105
MYO1B_t1	-1.24	3.04E-06	-0.07	140	-5.98	11936	-0.14	120	-9.67	13761	NA	NA	NA	NA
LMCD1	-1.26	0.002060606	-0.1	111	-5.99	11937	0.04	97	1.7	7355	-0.16	95	-7.35	11975
AMBN	1.01	0.778867277	-0.09	120	-5.99	11938	-0.11	117	-7.43	12845	-0.08	128	-5.89	11404
FEM1C_t1	-1.09	0.002193428	-0.07	134	-6	11939	NA	NA	NA		0.05	134	4.39	6253
CAPN5	-1.02	0.486267414	-0.1	113	-6	11940	-0.18	124	-13.32	14831	-0.11	131	-8.66	12480
FZD7	-1.13	0.047455991	-0.08	124	-6	11941	0.09	135	8.05	3717	-0.12	120	-8.59	12456
PURA	-1.01	0.720872609	-0.07	141	-6.01	11942	-0.08	127	-5.91	12135	-0.01	149	-0.86	9111
ERP27	1.05	0.556197487	-0.1	113	-6.01	11943	0	135	0.41	8194	-0.14	115	-9.2	12653
CSDC2_t1	-1.06	0.043945528	-0.09	117	-6.01	11944	NA	NA	NA	NA	NA	NA	NA	NA
WDPCP	1.09	1.20E-05	-0.07	136	-6.01	11945	0.06	115	3.85	5981	0.08	120	5.72	5574
CHMP7	-1.1	0.00076464	-0.07	131	-6.01	11946	-0.09	138	-8.1	13152	-0.17	120	-12.17	13600
ARL6IP4_t1	1.06	0.058162321	-0.09	116	-6.01	11947	-0.06	108	-3.7	10934	0.01	100	0.49	8326
LAT	-1.02	0.619585888	-0.12	99	-6.01	11948	0.01	121	0.81	7937	-0.13	113	-8.49	12422
MIS12	-1.12	0.003575808	-0.07	135	-6.02	11949	-0.11	138	-9.81	13822	-0.12	122	-8.73	12499
ITM2A	-1.39	0.001198555	-0.08	124	-6.02	11950	0.01	112	0.69	8002	-0.21	125	-16.01	14588
HK1_t2	-1.08	0.133077677	-0.11	106	-6.02	11951	0.07	114	4.22	5764	-0.18	138	-16.25	14635
PRDM2_t1	-1.12	0.001187041	-0.1	113	-6.03	11952	-0.01	132	-0.88	9068	-0.11	135	-9.07	12613
IL28RA_t1	-1.16	0.086987232	-0.08	123	-6.03	11953	-0.11	129	-8.46	13310	-0.12	152	-12.72	13766
RFTN1	-1.44	1.29E-05	-0.07	135	-6.03	11954	-0.14	120	-9.46	13684	-0.18	136	-15.63	14503
JAM3_t1	-1.08	0.005526794	-0.07	131	-6.03	11955	NA	NA	NA	NA	NA	NA	NA	NA
KIAA1324L_t1	1.03	0.162352662	-0.07	136	-6.03	11956	NA	NA	NA		-0.01	134	-1.03	9221
PCP4	1.12	0.063086372	-0.09	115	-6.03	11958	-0.04	113	-2.78	10329	-0.06	110	-3.45	10373
ADORA2B	1.07	0.23957902	-0.08	123	-6.04	11959	0.01	116	0.43	8186	0	104	-0.25	8763
MAX_t2	-1.16	8.36E-08	-0.13	98	-6.04	11960	-0.08	105	-4.38	11305	-0.24	102	-12.47	13688
ATXN1	-1.04	0.095595931	-0.07	131	-6.04	11961	0	133	0.21	8323	0.04	155	3.71	6562
DUOX2	1.08	0.043850532	-0.08	129	-6.04	11962	-0.08	120	-5.94	12150	-0.06	126	-4.52	10845
THG1L	-1.12	0.002705928	-0.09	116	-6.04	11963	-0.04	106	-2.12	9915	-0.18	124	-13.26	13911
CTDNEP1	-1.17	1.12E-05	-0.11	106	-6.04	11964	-0.11	168	-12.87	14726	-0.22	126	-16.66	14729
DDX19A	-1.11	0.000363541	-0.09	120	-6.04	11965	-0.16	132	-12.78	14709	-0.19	128	-14.76	14313
SRCIN1	-1.29	0.000652572	-0.07	133	-6.04	11966	-0.13	130	-10.04	13899	-0.07	122	-5.03	11060
GALNT2	-1.15	0.002734983	-0.08	124	-6.04	11967	-0.03	131	-2.71	10286	-0.13	128	-9.78	12842
THOP1	-1.21	0.007421673	-0.13	97	-6.05	11968	-0.09	113	-5.45	11899	-0.15	148	-14.53	14250
SNX21_t2	-1.02	0.260535383	-0.06	149	-6.05	11969	-0.14	120	-9.8	13819	-0.06	120	-4.53	10852
BCL11A_t1	1.01	0.595938505	-0.06	146	-6.05	11970	NA	NA	NA		-0.12	144	-11.17	13300
ESRP2	-1.3	3.14E-06	-0.09	118	-6.05	11971	-0.17	125	-12.83	14719	-0.17	131	-13.99	14109
UNC80	1.01	0.342459547	-0.07	135	-6.06	11972	-0.02	134	-2.02	9840	-0.06	124	-4.39	10794
BIRC6	-1.05	0.109801904	-0.07	137	-6.06	11973	0.03	138	2.41	6887	-0.03	144	-3.24	10289
CECR5	-1.11	0.002401534	-0.1	110	-6.06	11974	-0.08	116	-5.36	11853	-0.08	117	-5.6	11275
NOS3	-1.23	4.48E-09	-0.12	100	-6.06	11975	-0.14	112	-8.4	13290	-0.19	91	-7.94	12210
CCDC83	1.02	0.024291981	-0.07	132	-6.07	11976	0.07	126	5.03	5317	0	138	0.08	8561
TSKS	-1.05	0.004422152	-0.17	85	-6.07	11977	-0.02	80	-0.53	8837	-0.18	84	-6.25	11550
TUBGCP3_t1	-1	0.872870049	-0.1	109	-6.07	11978	NA	NA	NA		-0.07	146	-6.86	11796
NEUROG2	-1.01	0.122177186	-0.08	122	-6.07	11979	-0.05	127	-4.21	11212	-0.12	126	-9.22	12663
PDE4DIP_t2	-1.24	1.90E-06	-0.09	115	-6.07	11980	-0.23	119	-16.07	15434	-0.12	123	-9.11	12628
SUMF1	-1.05	0.15202342	-0.09	116	-6.07	11981	-0.05	103	-2.65	10249	-0.14	133	-11.38	13366
COPZ2	-1.29	8.11E-06	-0.17	86	-6.07	11982	-0.15	116	-10.07	13908	-0.24	88	-9.05	12606
KRCC1	-1.21	0.000415542	-0.09	118	-6.08	11983	-0.14	124	-10.7	14132	-0.14	126	-10.28	13021
MCCC2	-1.13	0.005714766	-0.08	126	-6.08	11984	-0.08	121	-5.5	11914	0.02	136	1.51	7740
HBG1_t1	-1.61	4.20E-12	-0.19	82	-6.08	11985	-0.13	88	-5.01	11666	NA	NA	NA	NA
ZNF331_t1	-1.67	6.19E-07	-0.08	129	-6.08	11986	-0.14	147	-13.7	14920	NA	NA	NA	NA

CCL15	1.14	0.001107937	-0.07	132	-6.09	11987	-0.07	109	-4.33	11278	0.02	114	1.24	7885
FAM65A	-1.02	0.110795875	-0.12	99	-6.09	11988	-0.08	101	-4.05	11124	-0.08	120	-5.89	11401
TOB1	-1.24	0.000865419	-0.05	162	-6.09	11989	-0.23	115	-14.77	15162	0	137	-0.25	8767
MAB21L3	1	0.965502076	-0.08	124	-6.09	11990	-0.08	133	-6.24	12301	0.02	132	1.46	7775
UCP2	-1.38	1.23E-09	-0.11	108	-6.09	11991	-0.03	118	-1.72	9635	-0.34	103	-18.07	14990
ARNTL	-1.15	0.029369378	-0.09	118	-6.1	11992	-0.14	127	-10.61	14110	-0.1	132	-7.79	12160
NHLRC4	-1.18	6.39E-15	-0.11	107	-6.1	11993	-0.14	113	-9.03	13521	-0.31	104	-16.53	14693
DECR2	-1.16	0.006748648	-0.12	100	-6.1	11994	-0.1	124	-7.04	12671	-0.11	119	-7.27	11948
STK32C	-1.13	0.001148134	-0.12	100	-6.1	11995	0.01	124	0.54	8114	-0.22	108	-12.93	13825
KIF5A	-1	0.894949153	-0.08	126	-6.1	11996	-0.05	118	-3.23	10621	-0.03	134	-2.43	9913
KIRREL	1	0.732584557	-0.1	111	-6.1	11997	0.06	126	4.8	5444	-0.12	133	-9.55	12772
PLEKHH3	-1.08	0.144864288	-0.09	118	-6.1	11998	-0.01	103	-0.7	8939	-0.07	107	-4.18	10704
P4HA3	-1.15	1.51E-08	-0.11	104	-6.11	11999	-0.05	110	-2.9	10412	-0.18	115	-11.7	13456
FSCN1	-1.1	1.14E-05	-0.1	110	-6.12	12000	-0.07	114	-4.2	11206	-0.18	120	-12.79	13786
LRRC8C	-1.28	2.62E-11	-0.07	143	-6.12	12001	-0.04	133	-3.43	10749	-0.24	140	-21.92	15647
METRN	-1.51	7.44E-06	-0.16	88	-6.13	12002	-0.12	88	-4.61	11445	-0.1	84	-3.53	10407
ICAM1	-1.46	8.08E-06	-0.1	112	-6.13	12003	-0.03	143	-2.62	10229	-0.19	145	-18.12	15000
USP9X	1	0.981017218	-0.06	150	-6.13	12004	-0.04	140	-3.55	10840	0.05	147	4.64	6104
DYRK4	-1.1	0.012693446	-0.08	123	-6.13	12005	0.01	125	1.12	7731	-0.11	120	-7.73	12132
NAA60	-1.06	0.024711902	-0.07	141	-6.13	12006	-0.05	132	-4.15	11176	-0.06	111	-3.38	10347
SAMD14	-1.04	0.000301964	-0.09	122	-6.13	12007	-0.03	125	-2.55	10176	-0.1	120	-7.15	11896
ABCA11P	-1.11	0.002428214	-0.09	119	-6.14	12008	0	118	-0.23	8629	-0.07	120	-4.61	10899
GLT25D2	1.04	0.149597005	-0.07	134	-6.14	12009	0.05	134	4.21	5774	-0.07	128	-5.68	11308
FCRLB	1.01	0.928869636	-0.09	120	-6.14	12010	-0.08	104	-4.44	11353	-0.1	104	-5.34	11178
IGF2R	-1.04	0.320566815	-0.08	130	-6.14	12011	-0.03	120	-1.79	9681	0	164	-0.13	8687
HBP1_t1	-1.1	0.004197564	-0.08	126	-6.14	12012	-0.06	138	-5.33	11834	NA	NA	NA	NA
ZFP3	1.01	0.398550128	-0.07	143	-6.14	12013	0.01	139	1.12	7734	-0.03	146	-2.45	9926
FLT3LG	-1.03	0.04726117	-0.12	100	-6.14	12014	-0.08	104	-4.43	11343	-0.15	108	-8.49	12424
GZMB	-1.97	2.05E-13	-0.2	81	-6.15	12015	-0.14	116	-9.24	13603	-0.37	111	-22.69	15743
TEX29	1	0.839567133	-0.16	88	-6.15	12016	-0.14	96	-6.66	12493	-0.14	120	-10.06	12948
KIAA1551	-1.04	0.326161228	-0.06	145	-6.15	12017	-0.01	123	-0.73	8959	-0.07	125	-5.28	11152
CYP11A1	-1.04	0.297544634	-0.21	80	-6.15	12018	-0.11	120	-7.55	12896	-0.06	87	-2.21	9800
LRCH2	-1.02	0.601096143	-0.06	155	-6.15	12019	-0.04	132	-3.43	10747	-0.02	147	-2.38	9882
CHAD	-1.03	0.416439813	-0.13	99	-6.16	12020	-0.23	128	-17.62	15683	0	100	0.25	8465
FOXN4	1.71	3.34E-05	-0.09	117	-6.16	12021	-0.04	151	-4.42	11335	-0.01	129	-0.42	8861
KLHL20	-1.07	0.110211644	-0.09	120	-6.16	12022	-0.04	147	-3.51	10810	0	157	-0.51	8923
VSNL1	-1.12	0.22872182	-0.09	122	-6.16	12023	-0.05	129	-4.3	11263	0.12	132	9.99	3884
TSPYL4	-1.05	0.271942389	-0.06	159	-6.16	12024	-0.04	137	-3.15	10571	-0.11	137	-9.28	12681
RNF182	1.03	0.239260812	-0.08	130	-6.16	12025	-0.02	134	-1.27	9343	0.02	123	1.73	7615
RIN3	-1.26	8.99E-08	-0.13	98	-6.16	12026	-0.15	132	-11.91	14482	-0.29	108	-16.98	14798
SYT8	1.02	0.326642693	-0.12	100	-6.16	12027	0.08	97	3.72	6049	-0.13	100	-6.27	11562
MUSTN1	1.02	0.667921095	-0.16	89	-6.16	12028	-0.14	120	-9.76	13803	-0.04	100	-2.13	9769
FABP7	-1	0.664771975	-0.1	111	-6.17	12029	-0.05	104	-2.91	10420	-0.02	108	-1.27	9326
CD86_t1	-1.33	3.79E-06	-0.09	119	-6.17	12030	NA	NA	NA	NA	-0.25	125	-18.75	15129
AMN	-1.31	6.14E-08	-0.16	88	-6.18	12031	-0.1	107	-5.53	11933	-0.21	83	-6.87	11802
ARNT2	-1	0.994911758	-0.08	128	-6.18	12032	-0.01	137	-1.03	9162	-0.13	134	-11.03	13270
NINJ1	-1.16	0.000183723	-0.13	96	-6.18	12033	-0.1	127	-7.72	12997	-0.2	128	-15.56	14489
ATP12A	-1.01	0.32230463	-0.12	100	-6.18	12034	-0.08	121	-5.39	11866	-0.12	147	-11.31	13352
OASL	-1.37	0.000185509	-0.11	107	-6.18	12035	-0.23	122	-16.69	15545	-0.19	106	-10.92	13224
SDHC	-1	0.953759401	-0.07	136	-6.18	12036	-0.05	135	-4.56	11416	-0.09	126	-6.87	11805
STUB1	-1.05	0.143448678	-0.12	103	-6.19	12037	-0.1	108	-5.82	12100	-0.06	120	-4.17	10701
CCDC74B_t1	-1.1	0.08348995	-0.07	142	-6.2	12038	NA	NA	NA	NA	-0.2	110	-12	13544
CD3D	-1.45	3.34E-05	-0.13	98	-6.2	12039	0.01	109	0.78	7961	-0.27	108	-15.4	14456
FAM109A	-1.06	0.00919023	-0.09	122	-6.2	12040	-0.06	142	-5.82	12102	-0.13	119	-8.85	12543
RHOJ_t1	-1.05	0.046421858	-0.05	166	-6.21	12041	NA	NA	NA	NA	NA	NA	NA	NA
SFXN5	-1.01	0.517821729	-0.1	113	-6.21	12042	-0.13	125	-9.56	13718	-0.12	127	-9.04	12602
DRP2	1.01	0.257239546	-0.07	134	-6.22	12043	-0.1	134	-8.82	13442	0.01	133	1.02	8007
TIMM8B	1.01	0.838810146	-0.09	118	-6.22	12044	0.02	102	1.15	7705	-0.08	95	-3.56	10418
ANKRD18A	-1.03	0.032000979	-0.08	124	-6.22	12045	-0.15	115	-9.91	13846	-0.09	126	-6.6	11691
MAOB	-1.22	0.011786133	-0.09	119	-6.22	12046	-0.22	140	-20.23	16015	0	133	0.08	8560
ABCG8	-1.16	0.000264129	-0.12	102	-6.22	12047	-0.22	145	-20.44	16036	-0.12	121	-8.82	12532
ZDHHC4	-1.24	0.001477437	-0.13	97	-6.23	12048	-0.08	91	-3.29	10662	-0.13	104	-7	11847
SLC28A2	1.04	0.37070967	-0.08	127	-6.23	12049	-0.04	131	-3.05	10502	-0.09	120	-6.05	11454.5
SYNDIG1	1	0.850733518	-0.09	118	-6.23	12050	-0.04	151	-4.3	11265	-0.06	120	-3.95	10615
SREK1	-1.07	0.272099997	-0.06	147	-6.23	12051	0.04	131	3.01	6489	0.02	132	1.82	7554
MLL4	-1.02	0.637456347	-0.11	106	-6.23	12052	-0.04	115	-2.47	10138	-0.18	130	-14.24	14171
NOVA1_t2	1.01	0.702598069	-0.07	139	-6.24	12053	-0.08	131	-6.27	12314	-0.06	149	-5.9	11407
DBF4B_t2	-1.06	0.009962263	-0.09	118	-6.24	12054	-0.17	104	-9.05	13531	-0.11	123	-7.74	12136
RHOD	-1.16	0.000630015	-0.12	102	-6.24	12055	-0.11	111	-6.99	12644	-0.06	100	-2.9	10131
ECSIT	-1.05	0.214970297	-0.1	112	-6.24	12056	-0.18	91	-7.46	12853	-0.09	101	-4.41	10803
ITPR3	-1.18	0.042862024	-0.09	120	-6.24	12057	0.08	120	5.41	5114	-0.22	108	-12.85	13805
WISP2	-2.01	1.15E-08	-0.14	94	-6.24	12058	-0.06	123	-4.35	11288	-0.28	103	-14.63	14275
NOTCH2NL	1.01	0.901328397	-0.08	129	-6.25	12059	0.05	158	5.07	5283	-0.01	155	-0.57	8961
OXA1L	1.09	0.001528884	-0.09	120	-6.25	12060	-0.06	134	-5.06	11694	-0.01	130	-0.44	8872
GCFC2	-1.24	1.97E-05	-0.09	121	-6.25	12061	-0.06	142	-5.23	11792	-0.08	135	-6.48	11631
PAPD5_t2	-1.09	0.006453602	-0.07	144	-6.25	12062	-0.1	136	-8.68	13389	0.03	147	2.47	7198
ATP1B4	-1	0.913905316	-0.07	134	-6.26	12063	-0.08	124	-6.02	12197	-0.03	131	-2.72	10044
LRP1	-1.17	5.46E-05	-0.11	107	-6.26	12064	-0.18	127	-13.68	14915	-0.07	101	-3.57	10420
ENDOG	-1.34	3.90E-08	-0.21	80	-6.27	12065	-0.25	79	-7.23	12752	-0.15	80	-4.44	10820
CDK10_t1	-1.21	5.26E-09	-0.14	95	-6.27	12066	NA	NA	NA	NA	-0.19	100	-9.61	12780
RABL6	-1.05	0.108683624	-0.1	113	-6.27	12067	0.02	143	2.16	7034	-0.2	110	-12.11	13578
IGFBP7	-1.67	9.09E-07	-0.12	102	-6.27	12068	-0.01	124	-0.93	9100	-0.2	110	-12.18	13604
BCL2	-1.25	0.000865904	-0.07	146	-6.27	12069	-0.06	138	-5.67	12013	-0.14	142	-12.75	13776
STAP1	-1.13	3.44E-07	-0.11	109	-6.27	12070	-0.05	130	-3.94	11068	-0.26	131	-21.11	15522
SEPT6_t1	-1.26	0.000401405	-0.1	114	-6.28	12071	-0.03	129	-2.53	10168	-0.06	125	-4.57	10876
PLEKHA6_t1	-1.01	0.8962735	-0.08	124	-6.28	12072	NA	NA	NA	NA	NA	NA	NA	NA
COQ10B	-1.17	1.11E-05	-0.07	139	-6.28	12073	-0.01	139	-1.07	9194	-0.08	138	-7.23	11930
NUDC	-1.05	0.174700092	-0.08	124	-6.28	12074	-0.15	120	-10.16	13935	-0.07	148	-6.78	11765
EEFSEC	-1.01	0.451246518	-0.09	120	-6.28	12075	-0.14	151	-13.87	14962	-0.08	111	-4.94	11028
PELP1	-1.07	0.107135346	-0.24	76	-6.28	12076	-0.1	107	-5.8	12091	-0.24	100	-11.77	13481
SLCO1A2	-1	0.985761167	-0.08	128	-6.28	12077	-0.07	149	-6.57	12450	-0.01	144	-0.53	8936
ANGPTL4	-2.64	3.39E-13	-0.11	107	-6.28	12078	-0.27	120	-18.81	15845	-0.15	95	-6.57	11675
CDK19	1.03	0.012284564	-0.08	127	-6.29	12079	-0.03	139	-2.75	10310	-0.02	164	-2.09	9752
CSF2RA_t2	-1.33	1.43E-06	-0.07	136	-6.29	12080	-0.01	117	-0.37	8728	-0.25	132	-20.22	15371
ICAM4	-1.03	0.366827839	-0.12	104	-6.29	12081	-0.11	127	-8.57	13349	-0.15	122	-11.01	13264
AGMAT_t1	-1.51	4.53E-09	-0.07	136	-6.29	12082	-0.15	147	-14.86	15178	NA	NA	NA	NA
TRAM2	-1.46	3.14E-11	-0.08	128	-6.29	12083	-0.12	134	-10	13883	-0.17	143	-15.89	14561
TRIM15	-1.18	0.004682206	-0.09	120	-6.3	12084	-0.11	128	-8.63	13368	-0.11	92	-4.67	10923
CDC42EP3	-1.2	0.005019201	-0.08	131	-6.3	12085	0.01	140	0.97	7825	-0.16	140	-14.65	14285
ANTXR2	-1.12	1.72E-11	-0.07	144	-6.3	12086	-0.09	122	-6.26	12309	-0.11	136	-9.69	12806
EPC1	-1.09	0.0009332	-0.06	151	-6.3	12087	-0.09	118	-6.03	12200	-0.07	150	-6.97	11832
LRRC27	1.01	0.2045832	-0.07	141	-6.3	12088	0.01	143	0.6	8069	0.03	164	3.62	6605
IFT43_t1	-1.19	5.10E-06	-0.1	114	-6.3	12089	NA	NA	NA	NA	-0.16	116	-10.74	13167
PRB1	-1.01	0.137560109	-0.17	88	-6.3	12090	-0.01	107	-0.7	8936	-0.04	90	-1.63	9514
HSD3B1_t1	-1.04	0.330184745	-0.09	120	-6.31	12091	NA	NA	NA	NA	-0.05	127	-3.52	10402
ACRBP	-1.01	0.678336414	-0.11	108	-6.31	12092	-0.03	124	-2.08	9877	-0.16	103	-8.44	12413

GOLGA5	-1.21	0.001288736	-0.11	108	-6.31	12093	-0.2	104	-10.68	14125	-0.04	133	-3.7	10480
P2RX1	-1.02	0.271129561	-0.08	127	-6.31	12094	-0.01	132	-0.95	9115	-0.12	120	-8.24	12335
MAT2B	1.02	0.710700987	-0.07	145	-6.31	12095	0.03	122	2.26	6979	0	141	0.34	8404
SLC22A12	-1.05	0.00325751	-0.08	128	-6.31	12096	-0.13	126	-9.86	13832	-0.14	100	-7.16	11899
FMNL1	-1.17	1.19E-07	-0.11	107	-6.31	12097	-0.03	107	-1.83	9708	-0.28	111	-16.94	14792
FAAH2	-1.58	7.70E-09	-0.09	123	-6.31	12098	-0.33	88	-12.54	14655	-0.11	98	-5.09	11077
NEGR1	-1.01	0.168561416	-0.08	128	-6.32	12099	0	154	-0.49	8812	0.02	136	1.73	7611
SIX5	-1.02	0.095720849	-0.12	104	-6.32	12100	-0.12	120	-8.25	13216	-0.1	112	-6.17	11506
TREH	-1.57	4.69E-21	-0.29	72	-6.32	12101	-0.34	92	-14.2	15029	-0.25	115	-16.54	14697
GOLT1A	-1.13	0.036756716	-0.1	113	-6.32	12102	-0.09	127	-7.3	12788	-0.06	120	-4.21	10720
CHCHD10	-1.33	2.50E-05	-0.13	99	-6.33	12103	-0.15	102	-8.06	13134	-0.07	111	-4.54	10860
RLN2	-1.02	0.28100661	-0.08	132	-6.33	12104	0.08	118	5.32	5161	-0.23	135	-19.73	15288
FBXO7	-1.01	0.785431147	-0.11	110	-6.33	12105	-0.12	135	-10.22	13965	-0.08	151	-8.27	12351
DOK1	-1.12	1.43E-07	-0.11	106	-6.33	12106	0	108	0.22	8318	-0.35	116	-22.88	15763
ENDOU	-1.02	0.119578697	-0.09	120	-6.34	12107	0.03	115	1.92	7204	-0.01	110	-0.69	9025
CHST11	-1.18	0.003858713	-0.07	136	-6.34	12108	0.02	140	2.05	7111	-0.19	154	-19.79	15293
DLG2_t1	-1.04	0.018917523	-0.08	129	-6.34	12109	NA	NA	NA	NA	-0.04	153	-3.99	10633
PDGFC_t1	-1.07	2.23E-06	-0.07	138	-6.35	12110	NA	NA	NA	NA	-0.12	137	-10.52	13093
MRVI1	-1.14	0.009706947	-0.08	126	-6.35	12111	0.04	158	4.76	5466	-0.13	129	-10.01	12927
IFNA4	-1.08	4.43E-07	-0.11	106	-6.35	12112	-0.14	133	-12	14499	-0.07	103	-3.57	10423
ACTR1B	-1.12	3.49E-05	-0.11	106	-6.35	12113	-0.17	146	-15.84	15373	-0.2	120	-14.01	14116
PBX3	-1.05	0.366433606	-0.08	134	-6.36	12114	-0.11	133	-9.04	13524	-0.02	146	-2.28	9838
LTA	-1.09	1.38E-06	-0.11	110	-6.36	12115	-0.18	110	-11.05	14257	-0.12	100	-5.87	11390
FAAH	-1.25	1.14E-08	-0.17	88	-6.36	12116	-0.27	136	-22.82	16237	-0.25	88	-9.34	12693
COL9A3	-1.11	0.284215885	-0.12	104	-6.36	12117	-0.12	118	-8.13	13171	-0.16	122	-11.53	13409
CPXM1	-1.1	5.70E-08	-0.17	88	-6.36	12118	-0.16	72	-3.46	10771	-0.11	80	-3.19	10266
CYP4X1	-1.44	5.42E-09	-0.12	105	-6.36	12119	-0.16	132	-13.03	14760	-0.11	131	-8.64	12468
ANO9	-1.05	0.121324088	-0.09	120	-6.37	12120	-0.04	95	-1.58	9539	-0.19	118	-12.9	13818
ANTXR1_t2	-1.57	2.16E-05	-0.08	133	-6.37	12121	-0.02	135	-1.97	9796	-0.17	126	-12.94	13829
RPS6KA2	-1.07	0.156843607	-0.07	142	-6.37	12122	-0.01	132	-0.64	8901	-0.17	153	-17.57	14897
NOTCH1	-1.15	1.09E-05	-0.1	114	-6.37	12123	0.03	131	2.22	7004	-0.18	123	-13.08	13860
FARP1	-1.1	0.0546342	-0.1	112	-6.37	12124	0.07	127	5.65	4974	-0.18	120	-12.3	13646
TAOK2_t2	-1.24	3.08E-11	-0.14	96	-6.38	12125	-0.21	111	-12.91	14736	-0.22	104	-12.02	13549
IRS1	-1.2	0.014510776	-0.08	130	-6.38	12126	-0.14	123	-10.1	13916	-0.05	128	-3.87	10576
TMC4	-1.23	0.000573681	-0.14	97	-6.38	12127	-0.07	110	-4.31	11270	-0.18	94	-8.14	12283
CBLN4	1.01	0.775474215	-0.07	136	-6.38	12128	-0.08	120	-5.36	11850	-0.03	119	-2.25	9822
MMP23B	-1.61	1.76E-12	-0.21	80	-6.38	12129	-0.12	93	-5.02	11671	-0.31	104	-16.88	14778
DAG1_t1	-1.01	0.7449189	-0.08	127	-6.38	12130	NA	NA	NA	NA	0	133	-0.27	8776
SLC1A6	-1.03	0.106177037	-0.09	124	-6.38	12131	-0.19	105	-10.45	14046	-0.04	95	-1.64	9517
NLRP1_t1	-1.08	0.010988774	-0.1	113	-6.39	12132	-0.03	108	-1.6	9555	-0.13	111	-7.73	12134
CSPG4P1Y	-1.11	1.18E-08	-0.1	112	-6.39	12133	-0.19	124	-13.87	14960	-0.13	122	-9.42	12725
CYP1B1	-1.01	0.952191812	-0.08	126	-6.39	12134	-0.04	130	-3.12	10558	-0.06	142	-5.32	11170
NDUFS7_t1	-1.06	0.086631248	-0.09	119	-6.39	12135	-0.17	80	-4.99	11651	NA	NA	NA	NA
SLC23A1	-1.14	0.066915549	-0.11	109	-6.39	12136	-0.11	118	-7.58	12920	-0.01	123	-0.95	9169
SLC35A2_t2	-1.09	1.58E-05	-0.14	95	-6.39	12137	-0.27	110	-16.26	15469	-0.16	114	-10.01	12926
PLAUR	-1.8	1.05E-12	-0.11	107	-6.39	12138	-0.06	102	-2.95	10443	-0.26	110	-15.57	14490
RIMBP2	1.07	0.000266877	-0.08	134	-6.4	12139	0.03	140	3.07	6454	-0.09	140	-8.03	12242
LRRC55	1.01	0.363368355	-0.06	162	-6.4	12140	0.05	134	4.23	5763	0.09	136	7.65	4769
OR10H2	-1.38	1.81E-15	-0.21	80	-6.4	12141	-0.21	93	-9.07	13539	-0.27	100	-13.47	13970
ARMCX1	-1.4	8.72E-06	-0.09	119	-6.41	12142	-0.02	118	-1.28	9347	-0.16	119	-10.92	13226
SMARCA1	-1.24	0.013879894	-0.08	126	-6.41	12143	-0.09	115	-5.87	12123	0.03	135	2.92	6980
GRIN2D	-1.33	1.53E-06	-0.11	109	-6.41	12144	-0.14	130	-11.43	14350	-0.14	133	-11.54	13416
TMEM99	1.06	0.356054696	-0.06	151	-6.41	12145	-0.1	133	-8.12	13169	0	134	0	8618
SERPINF1	-1.61	2.74E-08	-0.27	74	-6.42	12146	-0.32	127	-24.33	16319	-0.23	111	-14.27	14179
LAMA5	-1.01	0.770617963	-0.11	111	-6.42	12147	0.06	92	2.35	6916	-0.09	110	-5.51	11243
SLITRK4	1.11	0.003310674	-0.08	132	-6.42	12148	0.18	131	14.32	1450	0.02	141	1.83	7548
KCND3	-1.02	0.079087582	-0.12	103	-6.42	12149	-0.1	120	-7.32	12800	-0.07	122	-4.89	11013
ASB12	-1.12	9.55E-09	-0.12	104	-6.42	12150	-0.21	106	-11.96	14491	-0.02	108	-1.21	9303
P2RY8	-1.22	0.002313867	-0.09	118	-6.42	12151	0.01	149	1.44	7531	-0.21	123	-15.3	14440
ALKBH5	-1.07	0.001227411	-0.08	134	-6.42	12152	-0.17	134	-14.44	15089	-0.03	122	-1.85	9638
CARD14_t1	-1.01	0.621586159	-0.12	103	-6.42	12153	-0.11	104	-5.96	12163	-0.13	106	-7.37	11986
SCGB1A1	-1.16	7.14E-07	-0.13	99	-6.42	12154	-0.25	98	-12.23	14567	-0.2	118	-13.29	13921
USP9Y	-1.04	0.05754718	-0.08	131	-6.42	12155	0.03	138	2.33	6938	-0.14	142	-13.08	13862
UHRF1BP1L	-1.02	0.685315621	-0.11	110	-6.42	12156	-0.01	104	-0.58	8865	0.13	108	7.76	4726
LRRK1	-1.15	4.44E-10	-0.09	123	-6.43	12157	-0.11	134	-9.27	13612	-0.24	142	-22.03	15663
OR52K2	-1.01	0.740542356	-0.13	98	-6.43	12158	-0.08	72	-1.66	9595	-0.1	104	-5.35	11180
NLRP7	-1.02	0.196641606	-0.12	104	-6.43	12159	0	120	-0.35	8712	-0.2	108	-11.4	13371
ERBB2	1.02	0.247371973	-0.08	132	-6.43	12160	-0.07	104	-3.94	11069	-0.03	116	-1.71	9558
KCNE1	-1.03	0.05900828	-0.09	124	-6.43	12161	-0.01	136	-0.84	9042	-0.06	128	-4.94	11027
FZD4	-1.05	0.022834215	-0.07	140	-6.44	12162	-0.11	146	-10.16	13936	-0.13	164	-14.42	14220
XCL1	-1.21	0.003723426	-0.08	128	-6.44	12163	-0.04	124	-2.69	10269	-0.13	125	-9.8	12854
SEMA3B_t1	1.05	0.604991943	-0.11	110	-6.44	12164	-0.07	92	-2.79	10334	NA	NA	NA	NA
PATZ1_t2	1.15	0.000301135	-0.08	127	-6.44	12165	-0.02	120	-1.11	9227	0.02	115	1.05	7990
ZNF280B	1.01	0.489594924	-0.08	134	-6.45	12166	-0.09	142	-8.02	13123	0	135	0.07	8576
HSCB	-1.11	0.005086045	-0.08	131	-6.45	12167	0.04	127	2.87	6566	-0.07	120	-4.69	10936
CLIP3_t1	-1.01	0.171242404	-0.1	112	-6.45	12168	NA	NA	NA	NA	-0.13	110	-7.59	12067
SUGP2_t2	1.03	0.388228626	-0.07	142	-6.45	12169	0.02	121	1.56	7461	-0.03	130	-2.48	9937
NANOG	1.02	0.707103499	-0.09	122	-6.45	12170	-0.04	111	-2.5	10151	0	127	0.22	8479
LCA5	-1.2	1.58E-07	-0.1	114	-6.45	12171	0.01	133	0.9	7870	-0.17	136	-14.89	14350
CLEC4E	-1.79	3.30E-13	-0.09	119	-6.45	12172	-0.05	131	-4.41	11334	-0.26	127	-20.12	15353
NDUFS3	-1.05	0.177107881	-0.12	104	-6.46	12173	-0.13	99	-6.38	12364	-0.08	87	-2.91	10140
CTSE_t1	1.03	0.438067858	-0.07	147	-6.46	12174	NA	NA	NA	NA	NA	NA	NA	NA
HSD3B7	-1.16	1.79E-05	-0.12	104	-6.46	12175	-0.23	111	-14.31	15059	-0.08	116	-5.44	11217
KRT6B	1.01	0.735341111	-0.08	135	-6.46	12176	-0.03	142	-2.47	10132	-0.08	120	-5.77	11349
PYGM	-1.04	0.004860779	-0.09	121	-6.46	12177	-0.07	144	-6.91	12610	-0.16	100	-7.9	12198
IFITM5	-1.28	3.05E-05	-0.17	88	-6.46	12178	-0.15	158	-16.5	15510	-0.16	100	-8.14	12284
KBTBD7	-1.1	0.011737498	-0.1	117	-6.46	12179	-0.09	119	-6.42	12382	-0.11	131	-9.21	12656
PREP	-1.15	4.41E-06	-0.1	116	-6.47	12180	-0.07	136	-6.33	12340	-0.17	120	-11.97	13531
GALNT7	-1.13	0.029303383	-0.07	143	-6.47	12181	0.04	129	3.38	6257	-0.17	135	-14.8	14326
MMP26	-1	0.843340994	-0.1	115	-6.47	12182	-0.02	120	-1.48	9479	-0.03	100	-1.38	9379
DUSP7	1.02	0.093906783	-0.1	118	-6.48	12183	-0.01	130	-0.75	8974	-0.02	131	-2.01	9710
STS	-1.07	0.000281698	-0.08	130	-6.48	12184	-0.13	140	-11.3	14324	-0.06	134	-5.17	11107
NLRP8	-1.04	0.001834424	-0.09	123	-6.48	12185	-0.17	143	-15.37	15283	-0.02	150	-2.25	9823
FAM151A	-3.52	2.70E-16	-0.25	76	-6.48	12186	-0.2	88	-7.51	12876	-0.23	80	-6.87	11800
CRNN	-1.02	0.059279435	-0.1	114	-6.48	12187	-0.11	100	-5.28	11811	-0.07	126	-5.12	11092
GPAM	1.05	0.528915855	-0.07	138	-6.48	12188	0.02	132	1.83	7254	-0.01	138	-1.17	9278
UBE2L6	-1.23	1.32E-05	-0.1	118	-6.48	12189	-0.15	144	-14.23	15039	-0.24	127	-18.26	15037
SLC24A1	-1.01	0.529079364	-0.09	120	-6.49	12190	-0.05	122	-3.3	10671	-0.03	123	-2.1	9754
CD8B	-1.1	0.007771746	-0.11	111	-6.49	12191	-0.08	113	-4.92	11609	-0.29	104	-15.9	14562
SERPINB9	-1.07	0.00113994	-0.08	136	-6.49	12192	-0.04	147	-3.73	10948	-0.14	142	-12.68	13754
ACY3	-1.26	7.92E-06	-0.13	99	-6.5	12193	-0.24	114	-15.34	15277	-0.09	100	-4.32	10761
TNFSF13	-1.3	3.47E-08	-0.13	100	-6.5	12194	-0.02	127	-1.82	9703	-0.25	102	-12.84	13800
ELP4	1.03	0.304504989	-0.09	121	-6.5	12195	-0.01	131	-0.61	8881	0	106	-0.23	8756
GPC5	1.07	0.033814107	-0.11	111	-6.5	12196	0.02	127	1.8	7277	0.04	123	3.05	6903
TXLNB	1.01	0.86577061	-0.08	134	-6.5	12197	0.1	129	7.57	3953	-0.06	152	-5.66	11300
ARRDC1_t1	-1.06	0.000220745	-0.11	109	-6.5	12198	NA	NA	NA	NA	-0.19	90	-7.47	12022

USP2	-1	0.852478019	-0.08	127	-6.5	12199	-0.1	136	-8.21	13198	-0.02	152	-2.45	9924
MOB3A	-1.14	7.49E-05	-0.07	137	-6.51	12200	0.04	133	3.56	6140	-0.27	130	-21.28	15552
SERPINE2	-1.03	0.720092453	-0.07	138	-6.51	12201	0.03	128	2.09	7090	-0.03	138	-3	10180
PLA2G15	-1.06	0.000840414	-0.09	125	-6.51	12202	-0.11	136	-9.87	13835	-0.14	115	-9.41	12724
FAM19A1	-1.03	0.015637603	-0.09	125	-6.51	12203	-0.13	120	-8.76	13422	-0.16	151	-16.37	14660
FGFRL1_t1	-1.3	8.10E-09	-0.11	109	-6.51	12204	-0.07	129	-5.5	11918	NA	NA	NA	NA
ERI2	1.01	0.704391566	-0.09	121	-6.52	12205	-0.01	113	-0.53	8834	0.03	128	2.59	7134
RET	-1.05	0.029619678	-0.09	123	-6.52	12206	-0.17	116	-10.96	14226	-0.17	147	-16.86	14773
HLA-DRB4	-1.89	2.14E-11	-0.12	104	-6.52	12207	-0.13	115	-8.29	13235	-0.33	146	-31.24	16336
ARSG	1.03	0.238399481	-0.08	127	-6.53	12208	-0.02	124	-1.37	9414	-0.07	122	-4.74	10958
HCAR2	1	0.966688715	-0.08	131	-6.53	12209	-0.1	118	-6.67	12499	-0.01	110	-0.55	8950
TMEM37	-1.4	1.02E-05	-0.1	116	-6.53	12210	-0.14	136	-11.63	14406	-0.13	109	-7.78	12152
ITSN2_t2	-1.07	0.001953623	-0.08	136	-6.53	12211	0.03	109	1.53	7480	-0.03	120	-2.36	9875
STAT6	-1.17	0.002553652	-0.09	120	-6.54	12212	0.04	120	2.96	6518	-0.08	114	-5.4	11202
PRSS50	-1.05	0.045941106	-0.13	102	-6.54	12213	-0.06	100	-2.94	10440	0.06	88	2.29	7301
GRIN2B	-1.02	0.123310954	-0.08	129	-6.54	12214	0	131	0.08	8415	-0.1	126	-7.96	12223
TFEB	-1.08	0.01923355	-0.08	131	-6.55	12215	-0.11	109	-6.53	12423	-0.06	120	-3.93	10603
TTN_t2	1.03	0.541782971	-0.09	121	-6.55	12216	-0.09	129	-6.99	12649	-0.05	137	-4.75	10962
PDLIM7_t2	1.03	0.00248248	-0.13	100	-6.55	12217	0.08	114	4.96	5362	-0.04	118	-2.92	10144
BATF3	-1.43	1.08E-10	-0.14	96	-6.55	12218	-0.02	140	-1.7	9617	-0.29	110	-17.4	14863
CNPPD1	-1.03	0.070949821	-0.1	116	-6.56	12219	-0.07	120	-5.23	11786	-0.15	116	-9.98	12922
DEXI	-1.13	0.009378791	-0.09	127	-6.56	12220	-0.09	124	-6.55	12440	-0.03	120	-1.79	9607
CHRNA4	-1	0.935176977	-0.09	126	-6.57	12221	-0.18	142	-16.38	15488	0	146	0.14	8526
AMICA1	-1.31	3.21E-08	-0.1	113	-6.57	12222	-0.05	129	-4.33	11277	-0.18	96	-8.35	12382
RAP1B	-1.12	0.06198486	-0.08	135	-6.57	12223	0.07	130	5.32	5163	-0.07	131	-5.81	11370
SCYL1_t2	-1.24	7.36E-12	-0.16	92	-6.57	12224	-0.13	138	-11.49	14370	-0.22	110	-13.37	13941
ACYP2	-1.24	0.000636839	-0.1	118	-6.58	12225	-0.12	119	-8.44	13301	-0.08	106	-4.7	10942
PFN3	-1.09	0.029994234	-0.19	84	-6.58	12226	-0.1	80	-2.86	10385	-0.08	80	-2.42	9899
UBE2G2	-1.04	0.106520555	-0.09	125	-6.58	12227	-0.1	135	-8.1	13148	-0.06	126	-4.59	10886
RPL29_t1	-1.1	0.01990081	-0.09	124	-6.58	12228	NA	NA	NA	NA	NA	NA	NA	NA
VGLL3_t1	1.01	0.703480747	-0.06	157	-6.59	12229	NA	NA	NA	NA	-0.07	153	-7.23	11929
RBFOX2	-1.04	0.209836536	-0.08	137	-6.59	12230	-0.02	120	-1.68	9606	-0.03	141	-2.32	9848
DSG1	-1.02	0.448354257	-0.07	148	-6.59	12231	-0.18	134	-15.48	15300	-0.06	157	-6.54	11664
IZUMO4_t1	-1.23	2.64E-06	-0.21	82	-6.59	12232	-0.27	104	-14.41	15081	-0.15	84	-5.16	11100
CTAGE1	1.03	0.003514206	-0.07	139	-6.59	12233	0.03	126	2.18	7021	0.03	138	2.66	7101
SLC39A2	-1.02	0.238622582	-0.1	118	-6.59	12234	-0.1	133	-8.5	13324	-0.04	103	-2.33	9857
KCNH3	1.02	0.120644369	-0.14	98	-6.6	12235	0	100	-0.08	8542	-0.06	95	-2.61	10004
NDUFA9	-1.03	0.470559346	-0.09	120	-6.6	12236	-0.13	130	-10.51	14067	-0.04	126	-2.72	10047
TAS1R1	1	0.953088806	-0.14	97	-6.6	12237	-0.12	99	-5.91	12141	-0.07	92	-3.13	10235
HDGF	1.03	0.469756208	-0.08	128	-6.6	12238	0	135	-0.27	8652	-0.02	124	-1.46	9428
GPR75	1.01	0.443124798	-0.09	120	-6.6	12239	-0.07	86	-2.55	10181	-0.01	108	-0.77	9066
PFN1_t1	-1.21	1.14E-10	-0.16	92	-6.61	12240	NA	NA	NA	NA	-0.25	128	-19.5	15249
ANGPT4	1.01	0.737511256	-0.1	119	-6.61	12241	0	128	-0.1	8557	-0.12	116	-7.93	12207
TSPAN31	-1.17	0.000217413	-0.1	119	-6.61	12242	-0.27	111	-16.38	15490	-0.1	128	-8.11	12272
PANK2	-1.04	0.216534733	-0.09	122	-6.62	12243	0	103	0.08	8410	-0.07	125	-5.05	11066
FZD5	-1	0.968278617	-0.08	136	-6.62	12244	-0.06	139	-5.51	11920	0.02	136	1.29	7856
FAM195A	-1.21	8.63E-05	-0.17	88	-6.62	12245	-0.16	91	-6.62	12475	-0.11	133	-8.72	12493
HTR2B	-1.35	0.000521284	-0.13	101	-6.63	12246	0.01	116	0.9	7874	-0.16	100	-7.82	12172
CHST15	-1.44	3.17E-07	-0.09	126	-6.64	12247	-0.04	138	-3.78	10969	-0.18	149	-18.11	14997
DCP1B	-1.17	0.000299019	-0.17	89	-6.64	12248	-0.15	118	-10.14	13931	-0.13	102	-6.6	11690
ARHGAP27_t2	-1.14	1.52E-07	-0.09	120	-6.64	12249	-0.06	135	-5.52	11930	NA	NA	NA	NA
LACTB	-1.12	0.006490502	-0.13	100	-6.64	12250	-0.11	111	-6.59	12460	0.01	97	0.27	8456
S1PR2	-1.09	3.88E-08	-0.09	120	-6.64	12251	-0.16	131	-13.22	14804	-0.07	150	-6.62	11704
TTTY14_t1	-1.09	0.030544946	-0.07	152	-6.65	12252	NA	NA	NA	NA	NA	NA	NA	NA
CLCA1	1.01	0.490816965	-0.11	109	-6.65	12253	-0.03	109	-1.77	9671	-0.01	98	-0.44	8880
RAB27A	-1.24	0.000189135	-0.08	132	-6.66	12254	-0.02	137	-1.51	9504	-0.08	132	-6.72	11743
KIF3C	-1.04	0.013690182	-0.07	139	-6.66	12255	0.02	130	1.46	7515	-0.18	126	-13.31	13924
RBM5	1.02	0.552817792	-0.1	114	-6.66	12256	0.04	142	3.98	5916	-0.01	118	-0.61	8987
GPHB5	-1.13	8.00E-08	-0.12	105	-6.66	12257	-0.23	127	-17.82	15714	-0.09	94	-3.76	10519
PPP4C	-1.12	0.000424371	-0.1	120	-6.67	12258	-0.03	111	-1.97	9800	-0.05	110	-2.81	10093
JAKMIP2	-1.02	0.736685961	-0.07	149	-6.68	12259	-0.09	132	-7.11	12699	0.01	136	1.1	7971
ZG16B	-1.15	0.112555406	-0.11	111	-6.68	12260	-0.03	92	-1.3	9362	-0.21	97	-9.83	12867
TMEM217_t1	-1.12	7.07E-06	-0.09	125	-6.68	12261	NA	NA	NA	NA	-0.08	141	-6.94	11821
CRABP1	-1.05	0.006077723	-0.12	106	-6.68	12262	-0.18	98	-8.82	13444	-0.09	138	-8.24	12337
PKIG	-1.17	2.62E-08	-0.11	113	-6.68	12263	-0.16	138	-13.74	14933	-0.08	112	-4.88	11007
OSBPL1A	-1.22	0.000647424	-0.07	144	-6.69	12264	0.04	120	3.03	6479	-0.04	147	-3.42	10364
TRMT11	-1.25	3.70E-08	-0.1	117	-6.69	12265	-0.04	108	-2.18	9952	-0.18	130	-14.33	14200
SGK1	-2.37	3.66E-17	-0.09	121	-6.69	12266	-0.18	140	-16.25	15466	-0.25	137	-21.9	15642
IFIT3	-1.39	4.10E-08	-0.09	127	-6.69	12267	-0.18	125	-13.49	14868	-0.15	129	-11.64	13439
SEC23A_t1	-1.11	0.115938921	-0.07	147	-6.69	12268	NA	NA	NA	NA	-0.01	151	-0.74	9050
SLC16A12	1.04	0.005721701	-0.07	151	-6.69	12269	0.08	139	7.36	4054	-0.01	131	-0.51	8924
PRKCSH	-1.05	0.167263293	-0.09	125	-6.7	12270	-0.18	132	-14.71	15148	-0.11	111	-6.84	11788
ING1	-1.04	0.150498366	-0.09	123	-6.7	12271	-0.14	138	-12.55	14657	-0.06	149	-5.59	11270
IL1RL2	-1	0.881660669	-0.1	114	-6.7	12272	-0.15	109	-9.08	13554	-0.08	97	-3.95	10613
PTPN6	-1.19	3.20E-08	-0.16	92	-6.7	12273	-0.07	110	-3.96	11083	-0.34	103	-18.01	14981
KIAA0141_t1	-1.1	0.006931138	-0.08	135	-6.7	12274	-0.04	131	-3.56	10846	NA	NA	NA	NA
RSPH3	-1.01	0.656407186	-0.11	111	-6.71	12275	-0.07	89	-2.62	10232	-0.13	117	-8.78	12520
DHTKD1	-1.29	0.00042617	-0.08	136	-6.71	12276	-0.1	131	-8.16	13177	-0.06	121	-4.06	10660
KLHL6_t1	-1.36	1.02E-06	-0.08	136	-6.72	12277	0.02	141	1.79	7289	NA	NA	NA	NA
TEKT2	-1.08	0.001119084	-0.2	84	-6.72	12278	-0.03	71	-0.69	8933	-0.28	90	-11.11	13286
SHPRH_t2	-1.14	0.005199316	-0.11	113	-6.72	12279	-0.04	135	-3.29	10665	-0.04	134	-3.74	10507
AHDC1_t1	-1.12	0.000128823	-0.12	105	-6.72	12280	NA	NA	NA	NA	NA	NA	NA	NA
RPUSD2	1.06	0.091346602	-0.09	128	-6.73	12281	-0.05	106	-2.83	10360	0.05	106	2.53	7167
SDR42E1_t1	-1.04	0.008508061	-0.1	120	-6.73	12282	NA	NA	NA	NA	-0.14	154	-14.54	14252
ARHGAP15	-1.28	1.83E-09	-0.11	110	-6.73	12283	0.02	130	1.47	7509	-0.27	106	-15.29	14434
ARF6	-1.1	0.001504136	-0.07	146	-6.73	12284	-0.13	129	-10.09	13913	-0.16	139	-14.44	14224
VPS18	-1.04	0.284643864	-0.1	120	-6.73	12285	-0.13	150	-12.61	14669	-0.09	120	-6.58	11683
RASL10B	1.07	0.153897988	-0.09	127	-6.73	12286	-0.1	121	-6.81	12567	-0.13	119	-9.05	12604
FAM96A	-1.12	0.063289903	-0.08	134	-6.73	12287	-0.19	121	-13.77	14940	0.03	107	1.66	7646
CBWD2	-1.11	0.022997509	-0.1	120	-6.74	12288	-0.07	121	-5.32	11831	-0.01	125	-0.89	9134
GP1BA	-1.06	0.018688039	-0.11	111	-6.74	12289	-0.18	109	-10.71	14136	-0.12	131	-9.67	12802
NARG2_t1	-1.02	0.105684294	-0.07	141	-6.74	12290	NA	NA	NA	NA	-0.01	127	-1.09	9243
NLRC3_t1	-1.03	0.05543417	-0.08	137	-6.74	12291	NA	NA	NA	NA	-0.15	130	-12.29	13636
RPL34_t1	-1.08	0.058162321	-0.09	121	-6.74	12292	NA	NA	NA	NA	NA	NA	NA	NA
IFI16_t1	-1.92	1.62E-11	-0.11	109	-6.75	12293	NA	NA	NA	NA	-0.26	112	-16.19	14624
SLC16A8	-1.03	0.01593158	-0.12	104	-6.75	12294	-0.13	120	-9.4	13663	-0.12	88	-4.56	10870
TSPAN32	-1.15	2.62E-05	-0.16	93	-6.75	12295	-0.07	111	-4.31	11269	-0.28	122	-19.98	15327
SEMA3C	-1.09	0.077994618	-0.09	122	-6.76	12296	-0.02	136	-2.03	9847	-0.19	151	-18.78	15135
TSHZ2	1	0.940174201	-0.07	145	-6.76	12297	-0.08	132	-6.53	12427	0.01	146	0.87	8097
IFNA21	-1.01	0.602072795	-0.09	125	-6.76	12298	-0.13	149	-12.76	14704	0.04	126	3.24	6809
FLRT3	-1.91	4.12E-08	-0.09	127	-6.77	12299	-0.15	147	-14.07	15007	-0.1	145	-9.69	12805
ST7L_t2	1.05	0.000198387	-0.08	130	-6.77	12300	0.02	127	1.8	7274	-0.04	129	-3.54	10413
CPNE5	-1.14	0.06370597	-0.07	141	-6.77	12301	-0.01	127	-1.11	9223	-0.2	112	-12.23	13617
FH	-1.18	0.016783499	-0.08	132	-6.77	12302	-0.21	113	-13.35	14840	-0.09	120	-6.05	11454.5
NNMT	-3.91	2.98E-17	-0.2	84	-6.77	12303	-0.26	88	-9.91	13850	-0.31	78	-8.57	12446
MRPL40	-1.15	0.000737115	-0.14	100	-6.77	12304	-0.14	94	-6.2	12286	-0.09	101	-4.62	10903

KCNMB1	-1.07	0.082477818	-0.09	122	-6.77	12305	0.08	105	4.57	5574	-0.22	110	-13.48	13971
MEP1B	-1.1	0.000487151	-0.15	95	-6.77	12306	-0.27	92	-11.2	14300	-0.09	92	-3.88	10582
LCN15	-1.21	6.48E-12	-0.21	83	-6.77	12307	-0.29	97	-13.44	14858	-0.26	90	-10.28	13019
DAAM1	-1.28	1.12E-05	-0.08	138	-6.78	12308	-0.1	140	-8.73	13406	-0.09	149	-8.98	12586
TRIM60	-1	0.886770324	-0.1	118	-6.78	12309	0.01	115	0.41	8202	0.02	104	1.06	7986
SIRT2	-1.15	3.10E-07	-0.11	112	-6.78	12310	-0.21	123	-15.39	15287	-0.17	96	-7.93	12206
GPC4	-1.2	0.001279448	-0.07	150	-6.78	12311	0.04	136	3.47	6207	-0.17	135	-14.04	14121
SP140_t1	-1.04	0.029054354	-0.12	106	-6.79	12312	NA	NA	NA	NA	-0.15	104	-8.12	12275
KLF14	-1.5	1.12E-12	-0.12	105	-6.79	12313	-0.2	115	-12.71	14693	-0.2	100	-10.1	12956
MATN1	1	0.888312654	-0.1	121	-6.79	12314	-0.12	124	-9.06	13536	-0.04	124	-2.93	10152
LMAN2L	-1.06	0.059690005	-0.07	143	-6.8	12315	-0.08	126	-6.07	12226	-0.14	135	-12.01	13545
TRIM22	-1.86	3.26E-14	-0.1	118	-6.8	12316	-0.13	123	-9.55	13716	-0.24	132	-19.94	15316
LARP6	-1.43	0.003184832	-0.13	103	-6.8	12317	0.04	101	1.95	7173	-0.23	95	-10.19	12987
TCEAL5	-1.35	6.46E-05	-0.11	112	-6.8	12318	-0.06	104	-3.03	10493	-0.25	104	-13.73	14032
NRN1L	-1.09	0.012877798	-0.13	104	-6.8	12319	-0.11	93	-4.73	11504	-0.22	96	-10.11	12959
IL15RA	-1.33	1.39E-08	-0.1	121	-6.81	12320	-0.11	121	-8.08	13142	-0.16	140	-14.65	14284
KRT35	-1.03	0.035598113	-0.14	100	-6.81	12321	-0.07	100	-3.31	10680	-0.05	131	-3.96	10620
BEST4	-1.17	1.13E-07	-0.09	124	-6.81	12322	-0.14	120	-9.87	13834	-0.17	140	-14.87	14345
NEUROG3	-1.39	1.11E-07	-0.1	119	-6.81	12323	-0.2	111	-12.18	14558	-0.11	114	-7	11844
YPEL5	-1	0.946937158	-0.08	134	-6.81	12324	0.1	114	6.1	4729	-0.03	139	-2.34	9864
FAM209A	-1.19	1.30E-06	-0.17	90	-6.81	12325	-0.03	89	-1.1	9217	-0.2	80	-6.08	11470
HEXDC	-1.06	0.054047341	-0.16	92	-6.81	12326	-0.18	118	-12.5	14642	-0.19	84	-6.61	11699
NUP88	-1.2	3.86E-05	-0.09	123	-6.81	12327	-0.17	94	-7.57	12910	-0.12	98	-5.88	11395
CACNA1F	-1.3	5.46E-15	-0.26	76	-6.81	12328	-0.29	96	-13.3	14828	-0.22	88	-8.26	12345
KAT8	-1.13	2.75E-05	-0.17	91	-6.82	12329	-0.11	84	-3.89	11029	-0.2	96	-9.3	12686
PFKFB3	-1.1	0.003077458	-0.06	155	-6.82	12330	-0.02	127	-1.19	9281	-0.1	144	-9.67	12801
RPL7L1	-1.09	0.013835404	-0.11	111	-6.83	12331	-0.01	138	-1.01	9155	-0.1	132	-8.33	12372
TRIM9_t2	1.1	0.077475775	-0.07	143	-6.84	12332	-0.05	149	-5.4	11872	-0.04	128	-2.91	10138
FAM47C	-1.07	0.023058184	-0.12	106	-6.84	12333	-0.21	102	-10.84	14191	-0.17	108	-9.8	12855
BACE2_t1	-1.4	0.000130457	-0.09	127	-6.84	12334	NA	NA	NA	NA	-0.19	118	-13.15	13887
EXD3	-1.21	1.48E-13	-0.2	85	-6.85	12335	-0.14	69	-2.66	10259	-0.26	68	-4.7	10940
GSTO1	-1.36	1.50E-06	-0.14	98	-6.85	12336	-0.21	95	-9.49	13696	-0.17	88	-6.31	11573
GSTO2	1.04	0.611892958	-0.13	104	-6.85	12337	-0.05	90	-2.1	9902	-0.08	92	-3.24	10288
ZSCAN10	-1.37	1.59E-07	-0.12	106	-6.86	12338	-0.13	113	-8.21	13194	-0.17	115	-11.12	13287
SEC24C	-1.02	0.410274587	-0.09	124	-6.86	12339	-0.06	120	-3.96	11080	-0.09	119	-6.42	11613
ATP5B	-1.01	0.836886741	-0.13	101	-6.86	12340	-0.14	120	-9.65	13754	-0.06	108	-3.72	10497
RAP2C	1	0.957255122	-0.07	153	-6.86	12341	-0.04	125	-2.8	10336	0.12	159	12.89	2924
TMEM86B_t1	-1.33	6.58E-08	-0.13	104	-6.86	12342	-0.17	124	-12.94	14742	NA	NA	NA	NA
IL20RB	-1.33	0.012136475	-0.1	119	-6.87	12343	-0.05	133	-3.93	11061	-0.1	112	-6.29	11567
IFT88	-1.21	9.43E-05	-0.11	115	-6.87	12344	-0.09	112	-5.8	12088	-0.12	115	-7.65	12092
NR2C1_t2	-1.05	0.340493619	-0.08	139	-6.87	12345	0	130	-0.09	8546	-0.06	133	-4.59	10890
GKN1	1.02	0.086405044	-0.09	123	-6.87	12346	-0.03	94	-1.54	9512	0.05	126	4.03	6403
KAT2B_t1	-1.26	0.000476193	-0.09	124	-6.87	12347	NA	NA	NA	NA	-0.02	138	-1.35	9366
SPPL2B_t1	-1.02	0.552563719	-0.13	104	-6.87	12348	-0.08	150	-8.01	13120	NA	NA	NA	NA
HACL1	1.01	0.934105419	-0.14	100	-6.88	12349	-0.14	103	-7.6	12928	0.02	117	1.3	7855
ACAP1	-1.14	4.05E-07	-0.19	87	-6.88	12350	-0.11	88	-4.1	11149	-0.36	72	-7.99	12237
MMP28_t1	1.01	0.582741448	-0.09	126	-6.88	12351	-0.05	104	-2.77	10325	-0.01	103	-0.38	8841
HIVEP2	-1.24	4.49E-05	-0.09	124	-6.88	12352	-0.04	162	-4.44	11354	-0.14	151	-14.64	14279
CXXC5	-1.17	0.000283813	-0.17	90	-6.88	12353	-0.12	122	-8.87	13454	-0.12	106	-6.82	11781
ZNF513	-1.01	0.793276924	-0.12	108	-6.89	12354	-0.1	114	-6.69	12508	-0.08	103	-4.06	10662
HHATL	1.01	0.847968943	-0.14	100	-6.89	12355	-0.04	78	-1.24	9319	-0.02	84	-0.77	9067
CPA3	-1.42	3.57E-07	-0.11	111	-6.89	12356	0	97	0.23	8300	-0.2	120	-13.94	14094
LBH_t1	-1.3	1.76E-07	-0.11	113	-6.9	12357	NA	NA	NA	NA	-0.15	131	-11.84	13498
TLR1	-1.54	2.83E-12	-0.18	89	-6.9	12358	-0.12	113	-7.6	12929	-0.22	123	-16.4	14666
LHB	-1.1	3.67E-06	-0.16	94	-6.9	12359	-0.11	110	-6.85	12584	-0.15	76	-3.84	10553
GRIPAP1_t1	-1.03	0.363805599	-0.11	112	-6.9	12360	NA	NA	NA	NA	-0.16	104	-8.77	12517
TNK2_t2	-1.26	3.66E-07	-0.1	120	-6.91	12361	0	121	0	8479	-0.2	101	-10.01	12928
ADAM21	-1.04	0.020138864	-0.13	104	-6.91	12362	-0.07	125	-5.14	11739	-0.07	108	-4.08	10671
FAM120B	1.02	0.559343452	-0.1	116	-6.92	12363	-0.04	122	-2.77	10323	-0.08	123	-5.87	11391
DARS	-1.1	0.079182667	-0.09	131	-6.92	12364	0.04	118	2.67	6698	-0.06	131	-4.92	11025
PTER	-1.05	0.201434717	-0.06	167	-6.92	12365	-0.12	127	-9.46	13680	0.03	129	2.42	7223
FAM76B_t1	-1.16	0.00487736	-0.08	140	-6.92	12366	NA	NA	NA	NA	-0.07	144	-6.55	11668
RYR1_t2	-1.02	0.102536124	-0.11	115	-6.93	12367	-0.04	113	-2.45	10118	-0.22	105	-12.1	13575
PNPLA2	-1.16	0.000460053	-0.08	133	-6.93	12368	-0.17	148	-16.6	15528	-0.11	124	-8.22	12320
PLCD1	-1.22	2.12E-07	-0.16	93	-6.93	12369	-0.08	118	-5.34	11839	-0.21	99	-10.06	12945
P2RX3	-1.03	0.119940276	-0.17	92	-6.93	12370	-0.12	125	-8.94	13481	-0.12	80	-3.66	10463
DERL3	1.03	0.213863466	-0.08	135	-6.93	12371	0.06	132	4.91	5389	-0.09	118	-6.04	11450
ASXL1_t1	-1.1	0.001584746	-0.09	124	-6.94	12372	NA	NA	NA	NA	NA	NA	NA	NA
CLEC4F	-1	0.834706398	-0.11	113	-6.94	12373	0	127	0.09	8408	-0.05	101	-2.55	9977
HEMGN	1	0.7662536	-0.09	131	-6.94	12374	-0.06	111	-3.61	10889	-0.06	139	-4.98	11044
SGCG	1.01	0.672490335	-0.12	110	-6.94	12375	-0.03	129	-2.44	10116	-0.07	111	-4.52	10850
GALC	-1.08	0.001190446	-0.09	126	-6.94	12376	-0.01	149	-0.69	8926	-0.08	128	-6.07	11464
EVL	-1.4	1.40E-05	-0.11	116	-6.94	12377	-0.06	117	-3.76	10957	-0.18	104	-9.82	12864
SLC19A2	-1.42	4.26E-05	-0.07	147	-6.95	12378	-0.07	130	-5.25	11801	-0.07	130	-5.78	11351
ANGEL1	1.04	0.221285158	-0.09	128	-6.95	12379	0.02	127	1.83	7257	-0.09	135	-7.24	11932
SIGLEC9	-1.23	4.17E-15	-0.17	92	-6.95	12380	-0.14	113	-8.54	13339	-0.36	102	-18.94	15163
HLA-DPA1	-2.06	2.10E-12	-0.09	129	-6.95	12381	-0.05	123	-3.56	10844	-0.28	113	-17.73	14932
ACOT11_t2	-1.28	5.50E-06	-0.11	113	-6.95	12382	-0.06	131	-4.72	11500	-0.23	115	-14.82	14331
TRIOBP_t2	1.02	0.583534682	-0.09	131	-6.96	12383	-0.02	135	-1.51	9503	-0.09	134	-7.67	12101
ABCC11	-1.04	0.465097587	-0.07	152	-6.96	12384	-0.05	99	-2.47	10135	-0.04	109	-2.26	9826
FHOD1	-1.19	0.000416504	-0.11	115	-6.96	12385	0.01	89	0.26	8290	-0.21	91	-8.42	12406
COL6A3_t2	-1.19	0.006003685	-0.1	120	-6.96	12386	0	132	0	8483	-0.22	142	-20.4	15397
ZNF800	-1.06	0.052937213	-0.08	134	-6.96	12387	-0.01	127	-1.03	9163	-0.03	144	-2.71	10042
SLC17A4	1.06	0.510810022	-0.07	149	-6.96	12388	-0.06	139	-4.93	11612	0.09	145	8.89	4263
NFIL3	-2.08	1.83E-18	-0.14	101	-6.96	12389	-0.25	105	-13.61	14898	-0.23	127	-17.89	14958
FECH	-1.12	0.011728418	-0.07	153	-6.97	12390	-0.11	135	-9.44	13672	-0.14	142	-12.89	13813
SLC25A17	1.02	0.368170924	-0.09	128	-6.97	12391	-0.07	139	-6.58	12454	0.06	127	4.83	6013
WNT5B	-1.11	0.11974999	-0.11	115	-6.97	12392	-0.23	120	-15.94	15403	-0.03	125	-1.93	9687
CRELD1	-1.05	0.158937569	-0.12	109	-6.97	12393	-0.08	123	-5.97	12165	-0.11	119	-7.58	12060
ZER1_t1	-1.15	2.68E-07	-0.11	111	-6.97	12394	NA	NA	NA	NA	-0.2	120	-13.76	14044
EPHA10	-1.02	0.182447966	-0.1	123	-6.97	12395	-0.12	127	-9.41	13666	-0.03	148	-2.81	10091
KCNE4	-1.32	0.001376424	-0.08	139	-6.98	12396	-0.04	150	-4.46	11362	-0.1	133	-8.66	12478
SKI_t1	-1.02	0.273275687	-0.09	132	-6.98	12397	NA	NA	NA	NA	-0.09	138	-8.09	12261
PANK4	-1.05	0.011401603	-0.12	107	-6.99	12398	-0.15	128	-11.32	14325	-0.16	115	-10.51	13091
FOXA2_t1	-1.04	0.710933866	-0.08	136	-6.99	12399	0	133	-0.39	8736	NA	NA	NA	NA
MMP25	-1.32	1.24E-07	-0.12	107	-6.99	12400	-0.11	129	-8.53	13336	-0.25	127	-19.16	15200
CAMP	-1.66	1.92E-12	-0.21	84	-6.99	12401	-0.12	97	-5.73	12041	-0.32	88	-12.11	13580
FOXO4	1.03	0.009545225	-0.1	118	-6.99	12402	0.03	131	2.75	6639	0.02	111	1.23	7893
RHBDL1	-1.19	0.000295541	-0.11	111	-6.99	12403	-0.08	120	-5.77	12079	-0.13	100	-6.64	11710
TMEM8A	-1.08	0.028808777	-0.07	152	-7	12404	-0.17	104	-8.96	13487	-0.12	120	-8.56	12440
SLC17A6	1.04	3.08E-05	-0.06	162	-7	12405	0.02	127	1.42	7544	-0.01	142	-0.87	9124
CFHR1	-2.07	2.29E-06	-0.13	105	-7	12406	-0.27	121	-19.28	15899	-0.09	106	-5.17	11104
RASD1	-2.27	9.76E-07	-0.1	120	-7.01	12407	-0.16	108	-9.47	13691	-0.15	146	-14.24	14168
EBI3_t1	-1.53	3.36E-11	-0.1	120	-7.01	12408	-0.07	99	-3.56	10845	NA	NA	NA	NA
NTAN1	-1.12	0.019742791	-0.17	92	-7.01	12409	0.02	88	0.83	7914	-0.01	90	-0.3	8793
LAMP2_t2	-1.34	2.24E-05	-0.08	142	-7.01	12410	-0.11	143	-10.22	13966	-0.01	140	-0.88	9126

MAP3K3	-1.14	3.55E-05	-0.08	141	-7.02	12411	-0.06	130	-4.48	11376	-0.22	132	-18.4	15063
REXO1	-1.02	0.06379537	-0.08	135	-7.02	12412	-0.06	137	-5.23	11789	-0.11	136	-9.66	12798
HEXIM2	-1.05	0.278036654	-0.17	92	-7.02	12413	-0.17	138	-14.62	15127	-0.1	100	-5	11049
PCDH7_t1	-1.02	0.037423518	-0.09	125	-7.02	12414	-0.2	126	-14.88	15181	-0.03	146	-2.42	9901
DDX3Y	-1.63	1.65E-07	-0.09	131	-7.03	12415	-0.02	144	-1.79	9683	-0.12	131	-9.9	12893
VENTX	1	0.978555299	-0.09	132	-7.03	12416	0.12	130	9.42	3101	-0.05	116	-3.48	10386
PCDH12	-1.13	0.000931497	-0.14	101	-7.03	12417	-0.14	98	-6.92	12613	-0.12	100	-5.79	11358
RSPH4A_t1	-1.03	0.00156863	-0.1	118	-7.03	12418	NA	NA	NA	NA	0.03	121	2.09	7401
TMOD1	-1.2	0.014076379	-0.09	125	-7.03	12419	-0.14	139	-12.6	14666	-0.07	150	-6.8	11772
CORO7	-1.19	4.51E-10	-0.13	106	-7.03	12420	-0.1	131	-7.7	12988	-0.27	120	-18.99	15170
GRIK3	1.01	0.392699694	-0.08	138	-7.04	12421	-0.1	131	-7.7	12981	0.01	140	1.07	7983
PLA2R1	-1.13	2.70E-09	-0.1	122	-7.04	12422	-0.1	147	-9.8	13818	-0.19	150	-18.58	15094
SLC25A26_t1	-1.06	0.069090938	-0.12	111	-7.04	12423	NA	NA	NA	NA	0	121	0.05	8583
LMAN1L	-1.18	7.58E-12	-0.15	97	-7.05	12424	-0.22	121	-15.52	15308	-0.16	107	-9.38	12708
CTPS1	-1.15	0.0172584	-0.09	130	-7.05	12425	-0.17	140	-15.74	15352	-0.1	123	-7.39	11991
PRMT10	-1.17	8.67E-07	-0.15	98	-7.06	12426	-0.11	92	-4.54	11403	-0.08	122	-5.76	11341
CENPC1	-1.24	6.00E-06	-0.08	143	-7.06	12427	-0.1	111	-6.27	12316	-0.1	121	-7.38	11988
CD44	-1.81	1.79E-08	-0.1	121	-7.06	12428	0	122	0.01	8467	-0.27	121	-19.27	15217
HYAL2	-1.25	3.55E-09	-0.12	110	-7.06	12429	-0.04	113	-2.82	10352	-0.26	117	-17.4	14864
RADIL	-1.23	0.000247612	-0.16	95	-7.06	12430	-0.09	120	-6.36	12358	-0.18	100	-8.96	12581
ARVCF	-1.55	5.21E-12	-0.13	104	-7.07	12431	-0.08	120	-5.8	12090	-0.18	114	-11.36	13363
HIVEP1	-1.26	4.29E-08	-0.11	116	-7.07	12432	-0.12	140	-10.75	14158	-0.07	125	-5.33	11173
TPO	1	0.845164613	-0.12	107	-7.07	12433	0.02	115	1.3	7607	0.01	111	0.64	8235
SH3D19_t1	-1.32	0.000260756	-0.08	144	-7.07	12434	NA	NA	NA	NA	0.02	143	2.29	7298
CD300LB	1	0.841071238	-0.1	123	-7.07	12435	-0.1	122	-6.92	12615	-0.1	126	-7.76	12148
ZNF768	-1.05	0.190847846	-0.11	115	-7.08	12436	-0.09	99	-4.39	11314	-0.01	88	-0.43	8869
TEX13B	-1.03	0.003382015	-0.16	94	-7.08	12437	-0.14	108	-8.23	13203	-0.1	92	-4.13	10687
FAM104B	-1.01	0.418445728	-0.11	116	-7.08	12438	-0.14	127	-10.92	14212	0	136	-0.19	8726
CAB39L_t1	-1.09	0.027133539	-0.07	156	-7.08	12439	-0.04	137	-3.71	10935	NA	NA	NA	NA
HBD	-1.19	0.005669685	-0.12	108	-7.09	12440	-0.14	100	-6.89	12599	-0.16	89	-6.09	11476
HSPA6	-1.28	0.001733382	-0.08	138	-7.09	12441	-0.07	116	-4.64	11459	-0.12	130	-9.99	12924
NAGLU	-1.46	5.45E-13	-0.19	88	-7.09	12442	-0.31	110	-18.74	15828	-0.26	124	-19.23	15208
NDN	-1.59	8.31E-06	-0.11	113	-7.09	12443	-0.03	133	-2.66	10258	-0.17	124	-12.67	13752
LPIN3	-1.04	0.036882249	-0.11	117	-7.09	12444	-0.11	128	-8.19	13185	-0.05	118	-3.32	10317
RAB14_t1	-1.21	5.35E-06	-0.09	132	-7.1	12445	NA	NA	NA	NA	-0.08	151	-7.9	12196
KANK1	-1.35	0.000334968	-0.1	120	-7.1	12446	-0.13	129	-10.54	14082	-0.07	134	-5.94	11419
LHX1	-1.23	4.57E-09	-0.1	120	-7.1	12447	-0.2	134	-16.52	15514	-0.19	116	-12.59	13726
CDCP2	-1.18	0.006209176	-0.11	115	-7.1	12448	-0.3	108	-17.43	15663	-0.11	118	-7.79	12156
IL17C	-1.01	0.573738274	-0.13	104	-7.11	12449	-0.22	114	-13.79	14946	-0.05	97	-2.45	9920
NGEF	-1.12	0.08196094	-0.12	107	-7.11	12450	-0.09	152	-9.52	13709	-0.11	108	-6.22	11530
NPVF	1.02	0.122699865	-0.11	112	-7.11	12451	-0.07	126	-5	11654	-0.02	118	-1.17	9280
TRIM14_t2	-1.18	0.000100304	-0.09	132	-7.11	12452	-0.1	135	-8.61	13365	-0.14	133	-11.7	13457
RAB32_t1	-1.12	0.0165706	-0.08	142	-7.12	12453	-0.02	123	-1.13	9242	NA	NA	NA	NA
TK2_t1	-1.31	4.35E-12	-0.1	124	-7.12	12454	NA	NA	NA	NA	-0.2	137	-17.72	14931
OGFR	-1.15	7.47E-05	-0.12	107	-7.12	12455	-0.04	99	-2.17	9938	-0.18	102	-9.62	12783
NT5C2	-1.14	6.67E-05	-0.08	141	-7.12	12456	-0.04	135	-3.1	10539	-0.1	160	-10.59	13112
RPL4	1.02	0.768433228	-0.13	104	-7.12	12457	0.01	106	0.63	8047	-0.07	93	-2.85	10113
FOXR1	-1.01	0.603173945	-0.15	96	-7.12	12458	0.05	98	2.55	6801	-0.06	112	-3.73	10498
ACOT4	-1.26	0.000420292	-0.16	95	-7.12	12459	-0.24	88	-8.97	13490	-0.11	104	-5.79	11356
ZNF451_t2	-1.06	0.157365137	-0.08	134	-7.13	12460	-0.01	119	-0.46	8789	0.02	134	1.81	7559
TSPAN13	-1.14	0.196397461	-0.09	131	-7.14	12461	0.11	108	6.18	4678	-0.06	121	-4.33	10769
PDLIM1	-1.21	8.50E-06	-0.08	138	-7.14	12462	-0.12	115	-7.82	13037	-0.16	106	-8.93	12565
CA9	1.02	0.817601642	-0.17	93	-7.14	12463	-0.09	112	-5.7	12034	-0.21	95	-9.36	12700
FBXO25_t1	-1.19	0.001689875	-0.11	118	-7.14	12464	-0.15	113	-9.66	13756	-0.17	130	-13.48	13972
COL18A1	-1.15	0.043207794	-0.12	110	-7.15	12465	-0.16	129	-12.43	14623	-0.05	121	-3.66	10462
KCTD20	-1.12	0.001327731	-0.07	147	-7.15	12466	-0.03	138	-2.34	10045	-0.03	121	-2.48	9942
TREML2_t1	-1.23	7.21E-09	-0.12	111	-7.15	12467	-0.14	115	-8.99	13501	NA	NA	NA	NA
PPP1R17	1.01	0.543143359	-0.09	129	-7.16	12468	0.03	131	2.48	6842	-0.04	121	-2.75	10062
PCNX	-1.04	0.307144411	-0.06	161	-7.16	12469	-0.07	138	-5.86	12119	-0.08	133	-6.29	11566
B3GNT9_t1	-1.12	8.82E-10	-0.11	114	-7.16	12470	NA	NA	NA	NA	-0.15	104	-8.15	12290
PPP1R14A	-1.53	4.24E-08	-0.11	115	-7.16	12471	-0.02	124	-1.44	9458	-0.25	140	-22.8	15756
HOXA1	-1.01	0.156998289	-0.1	123	-7.17	12472	-0.19	127	-14.65	15136	-0.05	133	-4.26	10737
FBXW4_t1	-1.05	0.040802672	-0.12	109	-7.17	12473	NA	NA	NA	NA	NA	NA	NA	NA
CSTF1	-1.11	0.001994917	-0.07	146	-7.17	12474	-0.28	139	-25.05	16348	-0.08	119	-5.39	11198
HERC3	-1.06	3.47E-05	-0.06	168	-7.17	12475	-0.07	129	-5.76	12063	-0.01	155	-1.57	9484.5
ATP1A2	-1.09	0.293513647	-0.07	149	-7.17	12476	0.02	151	2.36	6912	-0.11	156	-12.05	13560
STRC	1.08	0.019650695	-0.11	115	-7.18	12477	-0.09	76	-2.22	9983	0.01	84	0.42	8357
LTBP3_t1	-1.01	0.476804628	-0.14	100	-7.18	12478	NA	NA	NA	NA	-0.07	110	-4.32	10760
ZDHHC11	-1.42	0.000301021	-0.14	103	-7.18	12479	-0.15	113	-9.68	13766	-0.15	108	-8.63	12465
TRIM2	-1.06	0.215609159	-0.08	142	-7.19	12480	0.02	137	2.11	7072	0.02	152	2.16	7365
MEGF10	-1.02	0.029842143	-0.06	163	-7.19	12481	-0.04	136	-3.64	10903	-0.07	135	-5.61	11277
OCA2	-1	0.986889209	-0.1	119	-7.19	12482	-0.07	128	-5.31	11828	-0.12	114	-7.55	12049
LRP5L	-1.12	0.020081407	-0.12	109	-7.19	12483	0.03	118	1.83	7255	-0.09	100	-4.4	10800
NBR1	-1.12	0.033412494	-0.08	138	-7.19	12484	-0.19	122	-13.72	14928	-0.05	122	-3.76	10520
MSR1_t2	-1.13	2.11E-07	-0.1	124	-7.19	12485	0.02	120	1.49	7497	-0.19	142	-17.19	14832
RDH12	-1.23	0.005139332	-0.11	114	-7.19	12486	-0.16	141	-14.45	15092	-0.01	133	-0.64	9004
ANAPC13	-1.01	0.753794449	-0.08	138	-7.19	12487	0.05	127	4.17	5798	-0.05	110	-3.25	10292
HVCN1	-1.27	1.15E-11	-0.11	116	-7.2	12488	-0.02	120	-1.06	9186	-0.31	112	-19.28	15218
RARG_t1	-1.06	0.000650596	-0.1	120	-7.2	12489	-0.05	117	-3.53	10825	NA	NA	NA	NA
LAMP5	-1.18	0.053530444	-0.09	129	-7.2	12490	-0.02	126	-1.21	9304	-0.25	128	-19.22	15207
HDAC7_t1	-1.3	3.93E-05	-0.11	113	-7.2	12491	0.05	113	3.38	6262	NA	NA	NA	NA
LAD1	-1.17	0.128000524	-0.1	119	-7.2	12492	0	116	0.11	8395	-0.07	107	-4.08	10676
PLEKHO1	-1.43	8.76E-08	-0.1	119	-7.2	12493	0.01	101	0.4	8206	-0.26	101	-13.41	13959
ISCU	-1.11	0.010250706	-0.08	142	-7.21	12494	0.01	104	0.3	8268	-0.04	115	-2.45	9923
SIGIRR	-1.73	1.08E-13	-0.24	80	-7.21	12495	-0.17	104	-9.38	13650	-0.23	92	-9.66	12796
PAF1_t1	-1.02	0.45727133	-0.13	106	-7.21	12496	NA	NA	NA	NA	NA	NA	NA	NA
OLIG1	-1.07	0.002517352	-0.1	123	-7.21	12497	-0.05	110	-2.95	10445	-0.06	129	-4.92	11022
ADAMTS2	-1.18	1.53E-12	-0.17	92	-7.22	12498	-0.13	104	-6.8	12563	-0.15	104	-7.96	12220
CDR2	-1.11	0.044334676	-0.09	130	-7.22	12499	-0.01	143	-0.76	8984	-0.01	132	-0.81	9090
SERPINA6	-1.92	5.32E-09	-0.19	89	-7.22	12500	-0.39	100	-19.6	15944	-0.19	118	-13.04	13851
NTNG2	-1.09	0.012704102	-0.13	104	-7.23	12501	-0.11	124	-8	13114	-0.27	123	-20.03	15336
DKK3_t1	-1.55	0.000144904	-0.1	119	-7.23	12502	NA	NA	NA	NA	NA	NA	NA	NA
RPS6KA5_t2	-1.09	0.000150324	-0.09	130	-7.23	12503	-0.07	140	-6.06	12219	-0.09	169	-11.19	13304
MX2	-1.48	4.80E-09	-0.11	116	-7.24	12504	-0.02	120	-1.09	9215	-0.21	120	-14.46	14227
DPPA2	-1	0.970657698	-0.12	111	-7.24	12505	-0.06	106	-3.57	10856	-0.08	114	-5.25	11138
SEZ6L	-1.02	0.072863395	-0.09	135	-7.24	12506	-0.05	131	-3.78	10970	-0.13	128	-10.52	13094
CTDSP2_t1	-1.1	0.047154451	-0.09	133	-7.24	12507	0	134	-0.01	8496	NA	NA	NA	NA
ADAMTS7	-1.42	1.45E-12	-0.17	93	-7.24	12508	-0.2	111	-12.07	14529	-0.23	100	-11.71	13460
CD19	-1.2	0.006199	-0.16	95	-7.25	12509	-0.06	140	-5.43	11885	-0.16	111	-10.04	12939
CDH24	-1.42	1.98E-08	-0.13	106	-7.25	12510	-0.21	142	-19.02	15872	-0.17	100	-8.3	12362
RAPGEF4_t1	-1.19	2.59E-06	-0.08	136	-7.26	12511	-0.21	129	-16.22	15461	NA	NA	NA	NA
SPRYD3	1.01	0.662744107	-0.11	119	-7.26	12512	-0.01	135	-0.53	8838	-0.08	124	-5.68	11309
FAM5C	-1.45	3.80E-09	-0.15	100	-7.26	12513	-0.27	119	-18.78	15838	-0.11	109	-6.61	11702
RNF39	-1.01	0.65329248	-0.07	154	-7.26	12514	-0.12	131	-9.46	13683	-0.07	119	-4.91	11018
ZNHIT1	-1.1	0.028597612	-0.22	83	-7.26	12515	-0.15	129	-11.94	14488	-0.1	120	-7.02	11853
ITGB2	-1.46	7.33E-06	-0.1	126	-7.26	12516	0.08	111	4.78	5458	-0.25	106	-14.23	14164

BMPR1B	1	0.828345826	-0.07	153	-7.26	12517	0	140	-0.01	8486	0.02	149	2.03	7432
GCSAML	-1.01	0.144615943	-0.09	129	-7.26	12518	-0.06	131	-4.77	11520	-0.04	127	-2.7	10038
KRTAP12-1	-1.03	0.017058455	-0.13	106	-7.26	12519	-0.17	158	-18.73	15826	-0.09	100	-4.61	10901
MAFA	-1.32	6.34E-08	-0.1	120	-7.26	12520	-0.19	115	-12.3	14584	-0.15	113	-9.4	12720
CHD4	1.04	0.10236437	-0.11	118	-7.27	12521	0.01	117	0.89	7878	-0.08	120	-5.59	11271
DNAJC19_t1	-1.12	0.009250178	-0.08	143	-7.27	12522	NA	NA	NA	NA	-0.09	132	-7.26	11947
PDIA5	-1.49	1.17E-10	-0.17	93	-7.27	12523	-0.3	111	-18.53	15801	-0.23	131	-18.85	15146
MBNL1_t1	-1.14	7.69E-08	-0.09	130	-7.27	12524	0.04	134	2.99	6505	-0.2	152	-19.94	15315
SH3BGRL	-1.62	2.49E-07	-0.1	121	-7.27	12525	0	144	0.33	8253	-0.21	138	-18.15	15008
GJB2	-1.66	7.23E-09	-0.09	129	-7.27	12526	-0.22	125	-16.81	15567	-0.25	119	-17.49	14882
AEBP1	-2.52	4.80E-16	-0.17	92	-7.27	12527	-0.06	120	-3.97	11089	-0.29	100	-14.37	14208
EBPL_t1	-1.4	1.58E-06	-0.11	114	-7.28	12528	NA	NA	NA	NA	-0.2	124	-14.59	14264
FAM32A	-1.02	0.412260574	-0.09	132	-7.28	12529	-0.12	122	-8.71	13397	-0.09	126	-7.17	11909
SPHK2	-1.08	0.259018534	-0.13	108	-7.29	12530	-0.06	125	-4.46	11365	-0.12	144	-10.9	13219
BTBD1	-1.09	0.137318439	-0.11	117	-7.29	12531	-0.03	131	-2.71	10285	-0.02	129	-1.88	9654
MUC20	-1.03	0.574778303	-0.09	128	-7.3	12532	-0.05	154	-5.67	12012	-0.11	138	-10.06	12946
GDNF_t1	1.01	0.543850818	-0.09	128	-7.3	12533	NA	NA	NA	NA	-0.02	132	-1.29	9339
PNN	-1.14	0.079664253	-0.08	140	-7.3	12534	-0.06	119	-3.88	11024	-0.07	126	-5.22	11127
NFE2L2	-1.38	2.21E-06	-0.11	114	-7.3	12535	-0.15	118	-10.29	13999	-0.13	129	-10.4	13054
USP47	-1.07	0.254370124	-0.08	137	-7.3	12536	-0.11	118	-7.68	12974	0.03	148	2.81	7030
IL13RA1_t1	-1.27	1.18E-06	-0.09	127	-7.31	12537	NA	NA	NA	NA	-0.05	124	-3.44	10370
PCMT1_t1	-1.03	0.410706428	-0.09	132	-7.31	12538	NA	NA	NA	NA	NA	NA	NA	NA
LIF_t1	-1.03	0.027928679	-0.08	142	-7.31	12539	NA	NA	NA	NA	-0.11	134	-9.33	12691
TSPYL2	-1.09	8.47E-06	-0.14	104	-7.31	12540	-0.11	118	-7.52	12884	-0.17	155	-18.28	15040
APTX	1.01	0.718549394	-0.07	156	-7.31	12541	-0.1	132	-7.85	13054	0	119	-0.21	8740
NAP1L5	-1.34	1.07E-06	-0.07	149	-7.31	12542	-0.2	148	-19.92	15978	-0.16	120	-10.91	13223
KLHDC4	-1.11	6.86E-05	-0.1	120	-7.31	12543	-0.2	87	-7.31	12797	-0.11	119	-7.52	12042
TC2N	-1.1	0.120794541	-0.08	146	-7.32	12544	0.01	122	0.39	8212	-0.19	123	-13.99	14111
TBX2	-1.28	0.000373294	-0.11	118	-7.32	12545	-0.02	128	-1.18	9277	-0.16	116	-10.41	13057
CDIPT	-1.12	0.002169258	-0.09	130	-7.32	12546	-0.14	119	-9.93	13855	-0.07	103	-3.72	10492
CXCR3	-1.29	2.18E-06	-0.12	109	-7.32	12547	-0.09	115	-5.98	12171	-0.25	118	-17.14	14822
VAV2	1.01	0.523435967	-0.09	128	-7.33	12548	-0.04	120	-2.71	10280	-0.04	148	-4.26	10740
POLR2C	-1.11	0.002548935	-0.13	107	-7.33	12549	-0.25	107	-14.17	15020	-0.12	126	-8.91	12562
GUCA2B	-1.05	0.47781458	-0.19	89	-7.33	12550	-0.08	105	-4.59	11433	-0.1	87	-3.71	10489
PCP2	-1.18	1.02E-06	-0.19	88	-7.33	12551	-0.19	111	-11.86	14468	-0.17	80	-4.98	11042
PKD1L2_t2	1.07	0.055433675	-0.13	106	-7.33	12552	-0.05	123	-3.96	11082	-0.06	99	-3.18	10258
OMD	-1.13	4.90E-05	-0.12	111	-7.33	12553	-0.08	114	-5.2	11773	-0.22	119	-15.22	14423
KLHL9	-1.08	0.171929108	-0.09	135	-7.34	12554	0.01	124	0.53	8121	-0.04	132	-3.11	10228
FAM120C	-1.16	7.45E-06	-0.1	127	-7.34	12555	-0.16	135	-13.74	14932	-0.18	136	-15.38	14455
FJX1	-1.28	3.33E-05	-0.15	100	-7.34	12556	-0.07	122	-4.9	11597	-0.15	120	-10.3	13027
CXCL16	-1.28	6.85E-09	-0.12	111	-7.35	12557	-0.21	120	-14.78	15165	-0.28	123	-20.23	15374
KRT19	-3.17	1.00E-14	-0.25	80	-7.36	12558	-0.1	90	-3.8	10985	-0.39	81	-11.97	13530
PRRG2	-1.16	2.90E-07	-0.13	108	-7.36	12559	-0.19	133	-16.01	15418	-0.15	133	-12.47	13687
KIF19	-1.01	0.117534888	-0.08	138	-7.36	12560	-0.08	109	-4.5	11387	-0.16	100	-8.13	12277
ELP2	-1.2	5.94E-05	-0.13	105	-7.37	12561	-0.22	96	-10.34	14018	-0.11	108	-6.36	11597
BCMO1	-1.05	0.01967874	-0.12	114	-7.37	12562	-0.14	111	-8.75	13418	-0.1	162	-11.51	13402
IL4	-1.03	0.002501986	-0.13	105	-7.37	12563	-0.08	85	-2.71	10278	-0.12	76	-3.2	10268
STATH	-1.01	0.647430098	-0.09	134	-7.37	12564	-0.22	137	-19.14	15885	0.06	132	4.63	6112
VPS36	-1.14	0.000332741	-0.09	129	-7.37	12565	-0.01	140	-1.14	9249	-0.16	132	-13.12	13879
GNA15	-1.05	0.004815549	-0.08	140	-7.38	12566	-0.05	120	-3.28	10659	-0.14	121	-9.71	12811
SLC29A1	1.1	0.018700648	-0.08	139	-7.38	12567	0.01	134	0.81	7935	0.02	100	0.85	8111
CORO2B	-1.11	1.29E-06	-0.09	130	-7.38	12568	-0.13	132	-10.84	14188	-0.15	127	-11.7	13458
WDR27	-1.04	0.065002255	-0.11	116	-7.38	12569	-0.05	117	-3.64	10899	-0.15	100	-7.72	12127
ARSJ	-1.25	9.95E-05	-0.09	134	-7.39	12570	-0.08	124	-6.1	12242	-0.12	133	-10.03	12937
LIM2	-1.07	0.000388199	-0.17	93	-7.39	12571	-0.17	100	-8.72	13402	-0.15	96	-7.13	11891
CNTN4_t1	-1.02	0.182300362	-0.1	126	-7.39	12572	0.13	128	10.39	2722	-0.1	138	-8.99	12588
LRRC49	-1.01	0.759499035	-0.13	109	-7.39	12573	0.09	105	4.95	5371	-0.18	121	-12.89	13815
SLC38A3	-1.23	0.001422812	-0.14	102	-7.39	12574	-0.25	140	-22.14	16181	-0.09	127	-7.19	11914
CASP1	-1.88	4.59E-14	-0.16	97	-7.4	12575	-0.1	78	-2.9	10413	-0.31	80	-9.45	12740
VSIG2	-1.45	3.79E-14	-0.19	88	-7.4	12576	-0.21	83	-7.04	12670	-0.3	64	-4.22	10726
SEC63_t1	-1.11	0.047667918	-0.07	150	-7.4	12577	NA	NA	NA	NA	NA	NA	NA	NA
DPEP2	-1.45	2.22E-18	-0.21	86	-7.4	12578	-0.13	112	-8.07	13136	-0.37	90	-14.64	14278
FAM171A1	-1.38	0.000203646	-0.11	117	-7.4	12579	-0.01	117	-0.34	8705	-0.16	124	-11.65	13441
FCRL3_t1	-1.04	0.246944539	-0.13	109	-7.4	12580	NA	NA	NA	NA	NA	NA	NA	NA
SASH3_t1	-1.04	0.054041278	-0.1	121	-7.41	12581	NA	NA	NA	NA	-0.15	122	-10.57	13106
SLC18A2	-1.04	4.68E-05	-0.09	133	-7.41	12582	-0.09	134	-7.2	12735	-0.08	131	-6.22	11532
TOX2	-1.18	0.043721561	-0.11	117	-7.41	12583	-0.16	117	-10.54	14083	-0.13	115	-8.35	12380
WDR78_t2	-1.02	0.556197487	-0.09	135	-7.41	12584	0.09	147	8.94	3310	-0.11	121	-7.57	12057
UNC5C_t1	-1.03	0.000282688	-0.08	140	-7.41	12585	NA	NA	NA	NA	-0.15	148	-14.26	14174
TRAPPC10	-1.03	0.377821667	-0.09	134	-7.41	12586	-0.07	131	-5.98	12174	-0.02	131	-1.61	9508
ESPNL	1.01	0.910653491	-0.1	125	-7.41	12587	-0.01	128	-0.75	8978	-0.07	115	-4.86	11000
DUSP14	-1.4	8.19E-12	-0.12	111	-7.42	12588	-0.1	118	-6.71	12520	-0.19	110	-11.19	13305
GPR25	-1.27	1.76E-08	-0.22	83	-7.42	12589	-0.3	64	-4.19	11197	-0.2	68	-3.59	10430
GALNTL2	-1.14	0.011598461	-0.08	138	-7.42	12590	-0.03	139	-2.99	10470	-0.08	158	-8.54	12438
KCNK13	-1.02	0.456838169	-0.1	121	-7.43	12591	0.01	113	0.91	7863	-0.18	126	-13.91	14082
RANBP6	-1.03	0.28641196	-0.08	145	-7.43	12592	-0.05	116	-3.46	10769	-0.03	118	-1.86	9649
MAMLD1	-1.14	0.054561942	-0.09	132	-7.43	12593	-0.08	139	-7.22	12745	-0.12	112	-7.24	11934
GALNT12	1.05	0.227574773	-0.1	126	-7.43	12594	0.07	157	8.01	3742	-0.18	134	-14.71	14298
ARX	1.04	0.023524305	-0.09	133	-7.43	12595	-0.08	151	-8.06	13135	-0.01	155	-0.56	8958
PIK3AP1	-1.11	0.000544514	-0.09	135	-7.44	12596	-0.08	123	-5.97	12168	-0.17	130	-13.98	14106
FBXW5	-1.14	0.000682595	-0.11	120	-7.44	12597	-0.23	109	-13.29	14822	-0.09	120	-6.24	11543
SNX29	-1.14	3.59E-06	-0.1	127	-7.44	12598	-0.03	149	-3.02	10483	-0.22	135	-19.1	15189
KCNMB4	-1.53	4.88E-10	-0.08	145	-7.44	12599	0	147	-0.44	8779	-0.13	118	-8.93	12572
MYBPH	-1.33	0.002236052	-0.14	104	-7.44	12600	-0.05	128	-3.6	10879	-0.28	154	-28.75	16244
PGRMC1	-1.39	9.64E-06	-0.11	119	-7.44	12601	-0.29	139	-26.12	16383	0	135	-0.14	8694
SLC35E2	1.06	0.000248396	-0.08	141	-7.45	12602	0.01	137	0.67	8025	-0.01	153	-0.81	9093
PCDH7_t2	-1.1	4.55E-05	-0.12	111	-7.45	12603	-0.04	127	-3.4	10729	-0.23	115	-15.19	14416
RNASET2	-1.36	8.88E-11	-0.21	86	-7.45	12604	-0.08	95	-3.41	10736	-0.38	84	-13.04	13850
CNPY3	-1.12	0.000669886	-0.16	97	-7.45	12605	-0.18	119	-12.53	14647	-0.21	158	-22.66	15734
OPRL1	-1.05	3.02E-05	-0.08	148	-7.45	12606	-0.09	110	-5.5	11913	-0.05	123	-3.91	10596
GREB1_t2	-1.04	0.040221317	-0.1	128	-7.45	12607	-0.15	135	-12.87	14723	-0.18	120	-12.52	13706
GAK	1.02	0.627920349	-0.1	121	-7.45	12608	-0.02	129	-1.35	9397	-0.13	129	-10.65	13132
DUOXA2	-1.02	0.326253223	-0.15	100	-7.45	12609	-0.19	104	-10.29	13998	-0.13	100	-6.49	11634
FXYD7	-1.01	0.351606931	-0.15	100	-7.46	12610	-0.1	112	-6.3	12325	-0.03	106	-1.79	9606
INA	-1.12	5.79E-12	-0.11	120	-7.46	12611	-0.13	128	-9.86	13833	-0.25	114	-16.01	14591
TNFRSF13C	-1.04	0.000758986	-0.11	117	-7.46	12612	-0.12	120	-8.69	13391	-0.15	100	-7.45	12012
NAGK	-1.41	5.51E-16	-0.14	104	-7.46	12613	-0.1	124	-7.33	12806	-0.39	122	-27.93	16188
SEMG1	-1	0.959142457	-0.09	132	-7.47	12614	0.04	114	2.56	6791	-0.08	104	-4.31	10757
SMC6	1.01	0.835200347	-0.09	131	-7.47	12615	0.01	119	0.87	7891	0.09	133	7.37	4886
CREM_t2	1	0.728204113	-0.1	126	-7.47	12616	0.13	135	10.68	2606	-0.02	148	-1.96	9697
RCC1	-1.09	0.000119735	-0.1	128	-7.48	12617	-0.19	132	-15.71	15345	-0.15	110	-9.14	12641
AAMP	-1.12	0.00042068	-0.13	108	-7.48	12618	-0.12	109	-6.91	12606	-0.26	124	-19.34	15227
EVI2A	-1.43	3.73E-09	-0.1	123	-7.48	12619	0.02	128	1.28	7622	-0.25	128	-19.72	15285
KLHDC2	-1.25	8.96E-06	-0.17	95	-7.48	12620	-0.22	102	-11.26	14316	-0.09	131	-7.08	11875
CERS4	-1.13	0.004610777	-0.14	104	-7.48	12621	-0.06	106	-3.42	10742	-0.19	92	-8.13	12279
USP51	-1.26	6.92E-07	-0.09	138	-7.49	12622	-0.07	107	-4.02	11111	-0.22	127	-16.93	14791

SPNS3	-1.08	0.000107406	-0.17	95	-7.49	12623	-0.13	124	-9.64	13749	-0.22	100	-11.01	13263
TMEM130	-1.03	0.577821773	-0.11	117	-7.5	12624	-0.02	138	-1.95	9776	-0.14	122	-9.88	12885
CTNNBL1	-1.19	3.32E-07	-0.22	84	-7.5	12625	-0.31	108	-18.27	15766	-0.13	97	-6.16	11504
CREBRF	-1.24	0.000361953	-0.07	153	-7.5	12626	-0.06	128	-4.74	11507	-0.15	148	-15.17	14412
GNB4	-1.5	3.93E-13	-0.07	152	-7.5	12627	0	138	-0.13	8567	-0.24	141	-21.94	15650
NRP1_t2	-1.36	1.24E-05	-0.09	130	-7.5	12628	0.05	135	4.37	5674	NA	NA	NA	NA
SLC39A5	-2.5	1.38E-17	-0.33	73	-7.51	12629	-0.3	87	-11.07	14261	-0.26	109	-15.08	14395
FRMD1	1.04	0.00981265	-0.1	124	-7.51	12630	-0.04	141	-3.24	10624	-0.05	131	-4.33	10764
HLA-DRB5_t1	-1.91	6.24E-11	-0.13	108	-7.51	12631	NA	NA	NA	NA	-0.33	120	-22.78	15752
TRIM34_t2	-1.07	5.80E-05	-0.08	140	-7.51	12632	-0.13	117	-8.46	13309	-0.18	130	-14.09	14136
FOLR3	-1.1	0.020743793	-0.2	87	-7.51	12633	-0.06	78	-1.81	9696	-0.23	75	-5.74	11332
SYF2	-1.07	0.22195673	-0.1	125	-7.52	12634	0.03	123	1.92	7195	-0.07	115	-4.24	10733
EMR2	-1.37	5.67E-09	-0.09	137	-7.52	12635	0	120	0.17	8350	-0.28	140	-25.37	15984
CCL16	-1.42	0.000682069	-0.08	141	-7.52	12636	-0.12	115	-7.79	13026	0.01	122	0.57	8280
USP8	-1.17	0.004243713	-0.09	131	-7.53	12637	-0.08	146	-7.79	13022	0	151	-0.1	8676
PTX3	1.05	0.562355068	-0.11	120	-7.53	12638	0.04	127	2.75	6644	-0.14	130	-11.05	13273
BTBD2	-1.03	0.113895708	-0.12	112	-7.53	12639	-0.12	124	-9.07	13542	-0.22	126	-16.51	14685
NFAT5_t2	-1.33	1.21E-05	-0.08	139	-7.53	12640	-0.04	150	-3.91	11046	-0.13	142	-11.96	13528
MS4A4A	-1.85	9.16E-13	-0.1	122	-7.54	12641	-0.09	115	-5.73	12043	-0.26	120	-18.31	15043
FAR2	-1.04	0.16614252	-0.09	135	-7.54	12642	0	115	-0.03	8513	-0.15	130	-12.26	13632
ZIM2	1.13	0.008772915	-0.11	120	-7.55	12643	0.03	120	1.83	7253	0.05	118	3.22	6822
CYP2D6	-2.5	2.79E-12	-0.24	81	-7.55	12644	-0.3	88	-11.47	14366	-0.19	84	-6.39	11606
PTGR2_t1	1.01	0.852696817	-0.09	134	-7.56	12645	NA	NA	NA	NA	0.14	161	15.07	2311
NXPE4	-1.01	0.331183579	-0.12	113	-7.56	12646	0.08	109	4.65	5520	-0.08	111	-5.11	11084
HCRTR1	-1.06	0.001214531	-0.13	108	-7.56	12647	-0.07	125	-4.9	11595	-0.14	138	-12.17	13602
CACNA2D4	-1.14	1.27E-05	-0.08	143	-7.56	12648	0.02	134	1.89	7220	-0.21	109	-12.45	13685
TMEM140	-1.26	0.000160272	-0.11	120	-7.56	12649	-0.12	131	-9.58	13727	-0.18	111	-11.02	13265
SERP2	-1.34	0.000633279	-0.09	130	-7.56	12650	0.08	124	5.84	4879	-0.2	112	-12.29	13643
OR2F1	-1.09	5.30E-08	-0.21	86	-7.57	12651	-0.24	93	-10.17	13943	-0.16	123	-11.94	13522
ZNF549	-1.03	0.314719712	-0.08	146	-7.57	12652	0.14	140	12.6	1955	-0.03	150	-2.87	10120
CPOX	-1.2	2.85E-06	-0.1	126	-7.57	12653	-0.22	129	-17.63	15686	-0.15	120	-10.43	13066
ELL_t1	-1.14	1.90E-06	-0.11	120	-7.57	12654	NA	NA	NA	NA	-0.21	163	-24.07	15878
L2HGDH	-1.13	0.000621344	-0.08	141	-7.58	12655	-0.23	135	-19.8	15962	0	135	-0.14	8691
PDE6C	1	0.963905635	-0.15	99	-7.58	12656	-0.2	110	-11.75	14435	-0.06	95	-2.83	10099
VGF	-1.09	0.000128418	-0.15	99	-7.58	12657	-0.23	109	-13.47	14863	-0.17	110	-10.44	13069
GNG7_t1	-1.13	0.000251417	-0.11	120	-7.59	12658	-0.11	131	-9.3	13623	NA	NA	NA	NA
FAM83C	-1	0.797524801	-0.09	131	-7.59	12659	-0.07	136	-5.99	12180	-0.13	120	-8.91	12561
HEATR8	-1.12	0.092094477	-0.14	106	-7.59	12660	-0.21	121	-14.9	15184	-0.11	78	-3.17	10252
TMEM134	-1.11	0.002014571	-0.16	98	-7.6	12661	-0.15	92	-6.16	12266	-0.16	119	-10.83	13188
LRRC16B	-1.16	4.26E-08	-0.2	88	-7.6	12662	-0.28	117	-18.77	15834	-0.24	90	-9.42	12728
ZNF136	-1.05	0.00615255	-0.1	128	-7.61	12663	0.01	118	0.53	8122	-0.08	129	-6.64	11711
PICK1	-1.07	7.53E-05	-0.14	104	-7.61	12664	-0.17	118	-11.34	14327	-0.17	108	-10.12	12965
ADAMTS4	-1.81	1.51E-09	-0.12	116	-7.61	12665	-0.04	132	-3.15	10573	-0.21	123	-15.04	14390
SLC27A3	-1.14	0.016811376	-0.14	104	-7.61	12666	-0.17	88	-6.61	12466	-0.15	106	-8.39	12392
SPRED1	-1.08	0.000840589	-0.08	146	-7.62	12667	0.08	154	8.52	3491	-0.09	154	-9.09	12621
ITPR1	-1.27	4.85E-05	-0.11	120	-7.62	12668	-0.08	127	-6.4	12369	-0.13	143	-12.07	13568
CD209	-1.19	1.61E-05	-0.11	122	-7.62	12669	-0.12	120	-8.22	13199	-0.19	129	-14.93	14362
GPR98	-1.34	0.005363722	-0.12	114	-7.63	12670	-0.1	132	-7.84	13047	-0.09	135	-7.78	12154
ANKRD37	-2.24	1.82E-13	-0.16	98	-7.63	12671	-0.18	115	-11.89	14473	-0.23	93	-10.04	12941
IFT57_t1	-1.38	8.18E-05	-0.11	119	-7.63	12672	0.04	129	3.42	6232	NA	NA	NA	NA
CMTM7	-1.3	1.20E-05	-0.13	109	-7.63	12673	0.01	132	0.52	8125	-0.22	111	-13.37	13944
CCDC73	1.02	0.070475195	-0.1	128	-7.63	12674	0	107	-0.01	8490	0.06	117	4.08	6383
CRLS1	-1.07	0.32456125	-0.09	137	-7.63	12675	-0.08	123	-6.02	12194	-0.04	157	-4.27	10742
PDE7A	-1.02	0.623159423	-0.08	140	-7.63	12676	0.14	131	11.71	2229	-0.09	139	-8.02	12241
PCDHB4	1.03	0.10763463	-0.09	132	-7.63	12677	0.04	126	2.89	6558	-0.01	152	-0.55	8952
TYROBP	-1.84	5.62E-16	-0.18	93	-7.64	12678	-0.1	114	-6.12	12252	-0.36	100	-17.78	14937
STARD8	-1.19	8.39E-06	-0.11	120	-7.64	12679	-0.15	127	-11.78	14443	-0.08	126	-5.95	11425
PRR5-ARHGAP8	-1.08	0.018351251	-0.13	108	-7.65	12680	-0.09	126	-6.77	12548	-0.18	122	-13.23	13906
RPL22_t1	-1.26	7.92E-06	-0.11	122	-7.65	12681	NA	NA	NA	NA	NA	NA	NA	NA
TPSAB1	-1.27	9.05E-09	-0.13	111	-7.66	12682	0.01	97	0.28	8277	-0.27	100	-13.41	13958
TMEM9B_t1	-1.1	0.002842273	-0.1	129	-7.66	12683	NA	NA	NA	NA	-0.11	162	-12.63	13743
LRP5	-1.25	5.87E-07	-0.18	93	-7.66	12684	-0.25	104	-13.38	14847	-0.2	116	-13.33	13931
LSR	-1.49	5.83E-08	-0.14	104	-7.66	12685	-0.1	106	-5.69	12027	-0.16	112	-9.89	12891
SYNGR3	-1.27	2.17E-14	-0.13	111	-7.66	12686	-0.24	140	-21.99	16166	-0.19	118	-13.16	13889
NAB1	-1.22	5.65E-06	-0.08	145	-7.66	12687	-0.03	142	-2.55	10179	-0.09	127	-6.67	11725
APCDD1L	1.02	0.188842002	-0.11	120	-7.66	12688	-0.1	122	-7.07	12681	-0.05	118	-3.19	10263
TMEM175	-1.14	1.04E-05	-0.13	109	-7.66	12689	-0.07	120	-4.97	11641	-0.19	105	-10.38	13044
ATP2C2	-1.07	0.129468613	-0.18	93	-7.67	12690	-0.12	148	-11.65	14413	-0.13	123	-9.28	12682
A1BG	-1.27	0.00038682	-0.19	91	-7.67	12691	-0.17	104	-9.05	13532	-0.1	122	-7	11843
FAM134C_t1	1	0.999094749	-0.08	152	-7.67	12692	NA	NA	NA	NA	-0.08	126	-5.81	11368
CXCR4	-2.26	6.18E-10	-0.11	118	-7.67	12693	0	126	-0.15	8576	-0.26	122	-18.64	15107
GPR78	-1.3	2.89E-06	-0.1	126	-7.68	12694	-0.17	125	-12.99	14750	-0.11	123	-7.86	12184
FGF3	-1.14	1.57E-07	-0.13	108	-7.68	12695	-0.06	114	-3.87	11016	-0.21	103	-11.26	13331
SLC30A4	-1.01	0.785541361	-0.1	126	-7.68	12696	-0.06	131	-4.91	11603	0.09	152	8.73	4328
CMTM3_t1	-1.18	2.22E-05	-0.09	133	-7.68	12697	NA	NA	NA	NA	-0.22	120	-15.62	14501
CHRNA2	-1	0.932963002	-0.11	122	-7.69	12698	-0.19	127	-14.35	15065	-0.06	118	-3.83	10546
PITPNM1	-1.09	0.003663932	-0.11	119	-7.69	12699	-0.12	117	-8.31	13244	-0.2	100	-9.9	12896
CTSD	-1.22	6.38E-06	-0.13	110	-7.69	12700	-0.11	134	-9.41	13665	-0.21	104	-11.24	13322
NRP2_t3	-1.12	3.25E-07	-0.09	140	-7.69	12701	-0.04	139	-3.66	10919	-0.18	155	-18.39	15062
SCD5_t2	-1.16	8.30E-07	-0.1	131	-7.7	12702	-0.05	113	-2.87	10389	-0.26	113	-16.17	14622
FAM214B_t1	-1.12	4.71E-11	-0.09	133	-7.7	12703	-0.19	133	-15.69	15342	NA	NA	NA	NA
CD207	-1.02	0.071149417	-0.12	112	-7.71	12704	-0.05	120	-3.62	10891	-0.14	133	-11.21	13309
KIAA1683	-1.45	1.41E-06	-0.24	82	-7.72	12705	-0.19	111	-11.63	14404	-0.22	100	-10.78	13177
ERGIC1_t1	-1.03	0.194499598	-0.1	131	-7.72	12706	NA	NA	NA	NA	-0.04	133	-3.13	10239
LXN	-1.58	3.90E-05	-0.15	100	-7.72	12707	0.01	104	0.47	8155	-0.24	114	-15.6	14493
TBX1_t2	-1.34	8.49E-07	-0.11	120	-7.73	12708	-0.09	128	-6.69	12509	-0.24	119	-16.36	14657
CBY1	-1.09	0.005757769	-0.12	113	-7.73	12709	0	107	-0.16	8587	-0.12	147	-11.25	13324
WNT3A	1.07	0.156256571	-0.13	108	-7.73	12710	-0.1	125	-7.5	12875	-0.06	120	-4.46	10823
PFKFB1	-1.57	1.70E-06	-0.17	95	-7.74	12711	-0.21	108	-12.28	14577	-0.08	117	-5.47	11228
SLC25A45_t1	-1.05	0.130660856	-0.08	144	-7.74	12712	NA	NA	NA	NA	NA	NA	NA	NA
PTPRK	-1.14	0.060977719	-0.11	121	-7.75	12713	0.01	130	0.54	8115	-0.11	143	-10.55	13103
ZMYND15	-1.26	1.73E-07	-0.1	130	-7.75	12714	-0.21	124	-15.9	15389	-0.27	80	-8.23	12322
LZTFL1	-1.14	0.008236853	-0.09	132	-7.75	12715	-0.02	136	-1.38	9422	-0.09	128	-6.76	11758
ZNF546_t1	-1	0.96343734	-0.08	143	-7.75	12716	NA	NA	NA	NA	NA	NA	NA	NA
NKG7	-1.46	1.60E-10	-0.18	94	-7.75	12717	-0.1	103	-5.3	11824	-0.34	133	-28.56	16232
SCAPER	1.02	0.525917533	-0.08	145	-7.76	12718	-0.08	128	-6.41	12373	0.05	134	4.35	6268
CALCA_t1	1.17	0.005046672	-0.1	131	-7.76	12719	0	106	-0.26	8646	-0.04	105	-2.23	9809
VPREB3	-1.1	2.52E-06	-0.17	97	-7.76	12720	-0.21	92	-8.88	13461	-0.17	115	-10.94	13234
HCAR3	-1.74	8.13E-09	-0.1	131	-7.76	12721	-0.15	118	-10.43	14043	-0.22	107	-12.39	13668
FGL1	-2.09	3.53E-05	-0.1	127	-7.77	12722	-0.2	110	-11.76	14438	-0.13	109	-7.5	12035
YTHDC1_t1	-1.18	8.95E-07	-0.09	136	-7.77	12723	NA	NA	NA	NA	-0.13	139	-11.8	13487
HDAC9_t3	-1.08	5.19E-05	-0.09	132	-7.77	12724	-0.02	151	-1.74	9642	-0.15	131	-12.48	13695
TMPRSS5	-1.1	0.030504612	-0.14	104	-7.77	12725	0	126	0.01	8458	-0.16	102	-8.25	12342
SHROOM3	-1.47	6.34E-08	-0.09	137	-7.78	12726	-0.18	158	-19.91	15975	-0.21	142	-18.87	15151
CFB	-1.84	1.49E-09	-0.19	92	-7.78	12727	-0.36	78	-10.16	13939	-0.23	101	-11.71	13463
TM7SF3_t1	-1.25	5.36E-06	-0.07	156	-7.78	12728	NA	NA	NA	NA	-0.09	137	-7.81	12166

PYHIN1	-1.26	3.40E-09	-0.14	107	-7.79	12729	-0.09	114	-5.55	11943	-0.26	130	-20.59	15428
SLC22A2	1.01	0.308834064	-0.14	106	-7.79	12730	-0.05	130	-3.86	11008	-0.06	124	-4.2	10716
SLC39A12	-1.02	0.062830946	-0.11	118	-7.79	12731	-0.05	116	-3.39	10725	-0.03	157	-3.73	10500
PIM2	-1.31	0.000256136	-0.09	137	-7.79	12732	0.01	112	0.46	8163	-0.27	111	-16.65	14722
LOXL4	-1.09	0.491234924	-0.09	139	-7.8	12733	0.08	157	8.6	3458	-0.06	138	-5.32	11171
KCNK3	-1.01	0.3924014	-0.08	144	-7.8	12734	-0.07	127	-5.08	11706	-0.03	125	-2.31	9845
KIAA1456	-1.14	4.19E-06	-0.08	145	-7.8	12735	-0.02	143	-1.84	9719	-0.17	160	-19.24	15210
ADCY1	-1.02	0.194507969	-0.09	136	-7.8	12736	-0.09	150	-9.33	13628	-0.13	156	-14.17	14152
MPP2	1.02	0.03156563	-0.07	158	-7.8	12737	-0.02	133	-1.87	9739	0.03	125	1.98	7460
NRG4	-1.06	0.051200626	-0.1	125	-7.8	12738	-0.13	127	-10.27	13989	-0.1	162	-10.72	13160
ALOXE3	-1	0.93986706	-0.12	116	-7.81	12739	-0.14	125	-10.3	14001	-0.06	111	-3.96	10622
CAMK1G_t1	-1.06	0.000233556	-0.12	116	-7.81	12740	NA	NA	NA	NA	NA	NA	NA	NA
GDF15	-1.63	0.000247472	-0.16	100	-7.81	12741	-0.08	102	-4.35	11286	-0.11	117	-7.39	11994
RABEP2	-1.08	0.000410421	-0.13	112	-7.82	12742	0.04	130	3.21	6373	-0.23	130	-18.52	15084
PAQR6	-1.02	0.799889295	-0.11	119	-7.82	12743	-0.12	151	-12.34	14597	-0.12	104	-6.73	11750
SRSF9	1.01	0.843642639	-0.12	113	-7.82	12744	0.02	127	1.21	7671	-0.13	126	-10.13	12968
ZC3H12A	-1.64	2.13E-09	-0.13	108	-7.82	12745	-0.09	117	-5.79	12084	-0.24	112	-15.07	14393
HECTD2_t2	1.02	0.481496678	-0.08	144	-7.82	12746	0.09	128	7.23	4126	0.02	141	1.97	7469
TACR2	-1.11	2.57E-06	-0.15	103	-7.82	12747	-0.12	124	-8.53	13337	-0.22	125	-16.72	14737
ABCA5_t1	-1.11	0.183328675	-0.09	134	-7.83	12748	NA	NA	NA	NA	0.01	151	0.62	8246
SLC25A42	-1.03	0.266126712	-0.1	125	-7.83	12749	-0.04	124	-3.32	10689	-0.09	113	-5.39	11196
ELK1	-1.07	0.000305078	-0.12	117	-7.84	12750	-0.15	135	-12.53	14649	-0.15	107	-8.72	12496
NPHP4	-1.1	0.128600927	-0.11	121	-7.84	12751	-0.07	111	-4.18	11192	-0.09	123	-6.9	11813
MARK2_t1	-1.07	0.004607297	-0.11	120	-7.84	12752	0.03	147	3.39	6253	-0.17	121	-12.27	13635
SLC7A3	-1.04	0.000113914	-0.13	110	-7.84	12753	-0.19	114	-12.04	14513	-0.13	100	-6.33	11587
TRMT10B	1.02	0.540207707	-0.1	127	-7.85	12754	-0.02	137	-1.97	9797	-0.08	132	-6.73	11748
PROK1	1.1	0.183558435	-0.15	104	-7.85	12755	-0.24	124	-17.83	15715	-0.11	111	-6.67	11720
ITIH6	-1.04	0.017979528	-0.1	132	-7.85	12756	-0.18	129	-14.38	15077	-0.1	119	-7.09	11879
TSNAXIP1	-1.13	0.000327641	-0.16	100	-7.85	12757	-0.08	116	-5.39	11863	-0.2	84	-6.88	11808
CCDC69	-1.12	3.40E-05	-0.12	118	-7.86	12758	-0.21	131	-17.17	15612	-0.18	120	-12.65	13748
PMP22	-1.59	5.01E-06	-0.1	130	-7.86	12759	0.01	126	0.81	7931	-0.21	144	-20.05	15341
ZNF688	-1.14	0.000118168	-0.16	100	-7.86	12760	-0.14	100	-7.09	12687	-0.19	97	-9.03	12595
NR2F1	-1.97	1.40E-11	-0.23	84	-7.86	12761	-0.03	114	-2.15	9934	-0.26	84	-8.84	12541
ISG20	-1.45	2.66E-07	-0.2	90	-7.86	12762	-0.02	93	-0.69	8930	-0.26	99	-12.81	13792
PLAT	-1.77	3.53E-07	-0.1	131	-7.86	12763	-0.03	124	-2.29	10017	-0.19	129	-14.68	14294
CTRC	-1.65	3.15E-16	-0.24	83	-7.86	12764	-0.31	104	-16.98	15587	-0.28	82	-9.02	12593
INPP5K	-1.13	0.002206702	-0.12	115	-7.87	12765	-0.05	140	-4.93	11611	-0.19	115	-12.44	13681
SNX33	-1.1	3.13E-05	-0.1	132	-7.88	12766	-0.2	130	-15.99	15416	0.01	126	1.04	8000
SCG5	-1.39	5.36E-05	-0.14	108	-7.89	12767	-0.18	123	-13.42	14853	-0.14	135	-11.77	13480
LCE1A	-1.07	0.016419464	-0.11	120	-7.89	12768	-0.04	124	-2.61	10228	-0.14	100	-7.23	11928
DNAJC17	-1.1	0.000262955	-0.25	81	-7.89	12769	-0.2	88	-7.71	12991	-0.15	79	-4.39	10791
TUSC3	-1.94	9.04E-09	-0.1	133	-7.9	12770	-0.06	144	-5.44	11893	-0.21	132	-16.89	14781
GFPT2	-1.66	7.83E-09	-0.12	116	-7.9	12771	-0.02	108	-1.35	9398	-0.24	109	-14.35	14204
NCK2	-1.03	0.102835366	-0.1	131	-7.9	12772	-0.08	139	-6.74	12531	-0.16	132	-13.06	13858
LRSAM1	1.03	0.215068616	-0.16	100	-7.9	12773	-0.12	104	-6.36	12353	-0.13	110	-7.55	12047
IGFBP5_t1	-1.05	0.204149698	-0.1	129	-7.91	12774	NA	NA	NA	NA	-0.16	157	-17.47	14876
FCER1G	-1.32	1.23E-15	-0.16	99	-7.92	12775	-0.09	119	-6.27	12313	-0.33	90	-13.33	13930
BIN3_t1	-1.13	1.65E-05	-0.12	115	-7.92	12776	-0.08	131	-6.48	12404	NA	NA	NA	NA
S100A12	-2.21	6.65E-20	-0.26	80	-7.92	12777	-0.23	126	-17.52	15674	-0.34	96	-15.59	14492
PIGY_t1	-1.15	0.001215295	-0.11	125	-7.92	12778	NA	NA	NA	NA	NA	NA	NA	NA
EFCAB6	-1.1	1.09E-06	-0.17	98	-7.93	12779	-0.15	108	-8.5	13325	-0.17	84	-5.8	11362
ZDHHC14	-1.11	0.061600179	-0.09	134	-7.93	12780	-0.08	119	-5.57	11953	-0.05	148	-4.63	10904
THAP6_t1	-1.11	0.009435843	-0.08	144	-7.93	12781	NA	NA	NA	NA	NA	NA	NA	NA
VILL	-1.15	1.56E-05	-0.2	90	-7.93	12782	-0.03	124	-2.25	10000	-0.34	109	-20.06	15342
HMHB1	-1.16	1.61E-12	-0.18	95	-7.93	12783	-0.2	88	-7.48	12867	-0.16	102	-8.4	12396
LCE1C	-1.13	0.000939659	-0.13	110	-7.93	12784	-0.07	129	-5.8	12092	-0.13	104	-6.86	11795
COQ4	-1.19	9.42E-07	-0.16	101	-7.94	12785	-0.18	128	-14.05	15003	-0.31	108	-18.1	14995
KCNK6	1.01	0.883229646	-0.1	131	-7.94	12786	0.04	144	3.76	6029	-0.16	131	-13.21	13904
CREG2	-1.03	0.320879775	-0.1	128	-7.94	12787	-0.25	141	-23.04	16253	0.02	150	1.6	7683
NBPF3_t1	-1.19	0.000358191	-0.09	140	-7.94	12788	NA	NA	NA	NA	NA	NA	NA	NA
NALCN	-1.02	0.202293278	-0.11	123	-7.95	12789	0.05	140	4.85	5427	-0.05	118	-3.39	10354
CNTLN	-1.1	0.023058184	-0.11	122	-7.95	12790	-0.16	125	-12.23	14566	0.02	121	1.74	7600
IFT122	-1.17	4.89E-08	-0.2	90	-7.95	12791	-0.13	100	-6.59	12459	-0.26	100	-12.76	13780
FBXO2	-1.01	0.960701565	-0.12	114	-7.95	12792	-0.09	116	-5.86	12121	-0.01	113	-0.45	8884
FABP3	-1.33	2.54E-08	-0.16	100	-7.96	12793	0.01	124	0.46	8158	-0.28	129	-22.42	15711
REEP3_t1	-1.2	0.001259276	-0.07	158	-7.96	12794	NA	NA	NA	NA	-0.06	137	-4.88	11005
MOB3C	-1.11	1.51E-08	-0.11	121	-7.96	12795	-0.11	146	-11.02	14247	-0.24	123	-17.52	14890
KAZALD1	-1.13	0.030440062	-0.1	128	-7.96	12796	0.01	106	0.61	8061	-0.17	125	-12.5	13696
CDHR5_t1	-1.32	1.81E-06	-0.13	111	-7.96	12797	-0.26	131	-20.69	16064	-0.17	115	-11.27	13338
ARL4D	-1.36	6.36E-05	-0.13	110	-7.96	12798	-0.33	112	-20.16	16010	-0.14	109	-8.41	12401
HRASLS2	-1.16	0.191019785	-0.14	109	-7.97	12799	-0.06	91	-2.57	10196	-0.07	106	-4.07	10666
SH2D1B_t1	-1.06	2.63E-05	-0.11	122	-7.97	12800	NA	NA	NA	NA	-0.14	121	-10.16	12976
LTB4R	-1.02	0.359520353	-0.11	120	-7.98	12801	-0.17	141	-15.48	15304	-0.09	131	-7.34	11971
SLC27A1_t1	-1.31	1.52E-05	-0.11	121	-7.98	12802	0	130	0.39	8215	NA	NA	NA	NA
LCE2C_t1	-1.05	4.73E-05	-0.1	129	-7.98	12803	NA	NA	NA	NA	NA	NA	NA	NA
SSTR2	-1.62	1.07E-08	-0.1	128	-7.99	12804	-0.13	122	-9.37	13646	-0.2	148	-19.22	15204
TARSL2	-1.1	0.002442636	-0.1	127	-7.99	12805	-0.1	126	-7.89	13067	-0.03	142	-2.59	9995
RAB33A	-1.38	1.08E-10	-0.14	107	-8	12806	-0.08	95	-3.77	10963	-0.29	96	-13.52	13983
ARHGEF10L	1.05	0.547922324	-0.09	138	-8.01	12807	-0.11	115	-7.27	12771	0.02	100	0.77	8155
SPATA6	-1.01	0.74203935	-0.09	141	-8.01	12808	0.05	125	3.38	6259	0	132	-0.26	8770
KIR3DL1	-1.05	0.00618844	-0.07	159	-8.02	12809	-0.12	145	-11.54	14384	-0.14	147	-13.73	14030
PPAPDC2	-1.19	0.000996334	-0.11	123	-8.02	12810	-0.1	124	-7.27	12768	-0.12	127	-9.52	12764
SACM1L	-1.31	3.25E-06	-0.1	128	-8.02	12811	-0.12	120	-8.33	13256	-0.02	163	-1.89	9665
SLC5A2	-1.21	7.31E-14	-0.21	89	-8.02	12812	-0.26	105	-14.31	15057	-0.2	113	-12.65	13746
ATP5O	-1.11	0.046789314	-0.15	104	-8.02	12813	-0.13	92	-5.38	11859	-0.15	85	-5.1	11079
GSTP1	-2.54	3.10E-17	-0.24	84	-8.03	12814	-0.1	85	-3.45	10763	-0.4	92	-16.83	14762
NEXN	-1.74	4.79E-09	-0.13	110	-8.03	12815	-0.01	120	-0.4	8752	-0.22	114	-14.31	14193
MOXD1	-1.4	0.001719777	-0.1	130	-8.03	12816	-0.01	115	-0.76	8988	-0.23	127	-17.68	14922
ZNF598	1.02	0.587230149	-0.08	146	-8.03	12817	0.07	147	6.56	4470	-0.02	132	-1.73	9572
PITPNA	-1.18	2.27E-06	-0.1	131	-8.04	12818	-0.11	130	-8.9	13466	-0.23	130	-18.33	15053
GCA	-1.39	8.30E-07	-0.09	139	-8.04	12819	0.02	136	1.77	7306	-0.17	146	-16.41	14668
SLC16A11	-1.55	1.58E-08	-0.11	120	-8.04	12820	-0.23	88	-8.58	13353	-0.2	79	-5.77	11348
ITPR2	-1.27	0.000601629	-0.08	146	-8.05	12821	-0.14	135	-12.19	14560	-0.04	138	-3.74	10506
CDC42BPG	-1.14	6.14E-09	-0.14	106	-8.05	12822	-0.23	138	-20.3	16019	-0.12	95	-5.34	11175
LGI3	-1.16	2.99E-10	-0.12	116	-8.05	12823	-0.19	144	-17.97	15731	-0.11	124	-8.17	12304
ODF3L1	-1.32	3.46E-09	-0.15	104	-8.05	12824	-0.11	118	-7.43	12844	-0.17	100	-8.41	12400
FBXO25_t2	-1.22	0.002276446	-0.12	118	-8.06	12825	-0.13	113	-8.35	13261	-0.16	130	-12.53	13708
CTNND1	-1.17	0.000115429	-0.1	130	-8.07	12826	-0.07	132	-5.43	11887	-0.09	120	-6.32	11581
CHRM5	-1.06	0.005644624	-0.11	126	-8.07	12827	-0.23	110	-13.53	14878	-0.12	127	-9.38	12707
PIGL_t1	-1.05	0.100119342	-0.11	121	-8.07	12828	0.03	144	2.52	6811	NA	NA	NA	NA
MSL2	-1.09	0.000115845	-0.09	137	-8.07	12829	-0.12	119	-8.57	13350	-0.18	143	-17.06	14809
SHANK2_t2	1.07	0.282861138	-0.08	150	-8.08	12830	-0.09	144	-8.66	13381	0.06	162	6.68	5163
HCST	-1.52	1.88E-10	-0.18	95	-8.08	12831	-0.04	108	-2.6	10217	-0.33	115	-21.67	15614
CSF3R	-1.24	3.19E-12	-0.21	88	-8.08	12832	-0.19	125	-14.35	15066	-0.36	123	-26.17	16054
TRIM58	-1.07	2.00E-06	-0.09	136	-8.08	12833	-0.18	131	-14.35	15064	-0.06	139	-5.38	11193
CD27	-1.27	5.69E-05	-0.14	107	-8.08	12834	-0.05	130	-3.76	10960	-0.26	120	-17.96	14971

ZNF548	-1.02	0.487329571	-0.09	141	-8.09	12835	0.06	152	5.65	4984	-0.09	131	-7.6	12073
DAB2IP_t2	-1.01	0.830220389	-0.11	124	-8.09	12836	-0.01	133	-0.92	9091	-0.18	121	-12.52	13704
SLFN12	-1.02	0.635956905	-0.1	133	-8.09	12837	0.11	122	7.95	3772	-0.08	118	-5.32	11172
MARK3	1.01	0.653569515	-0.13	114	-8.1	12838	-0.16	110	-9.9	13842	0.01	134	0.58	8276
OTP	-1.03	0.044224928	-0.1	134	-8.1	12839	-0.11	135	-9.74	13791	-0.05	143	-4.58	10878
IL10RB	-1.12	0.000487886	-0.1	128	-8.1	12840	-0.09	130	-7.25	12762	-0.22	147	-21.79	15630
ABCA6	-2.04	3.01E-07	-0.19	93	-8.1	12841	-0.18	97	-8.25	13214	-0.11	100	-5.53	11250
ITGAX	-1.32	8.90E-07	-0.12	120	-8.11	12842	0.01	125	0.55	8102	-0.25	135	-20.97	15498
GANAB_t1	-1.08	0.010459986	-0.11	124	-8.11	12843	-0.08	127	-5.89	12130	NA	NA	NA	NA
JPH3	1.04	0.015565103	-0.13	113	-8.11	12844	-0.04	130	-3.38	10722	-0.03	134	-2.89	10129
SFTPC	-1.11	9.75E-09	-0.19	92	-8.11	12845	-0.18	100	-8.78	13426	-0.19	114	-12.44	13682
CARD17	-1.42	3.83E-10	-0.13	113	-8.11	12846	-0.03	99	-1.24	9320	-0.27	105	-14.85	14337
TMPRSS11B	-1.01	0.408497246	-0.09	144	-8.12	12847	-0.03	135	-2.93	10430	-0.09	126	-6.87	11801
GALM	-1.14	0.001200366	-0.11	127	-8.12	12848	-0.15	128	-12	14498	-0.15	141	-13.89	14074
PRDM8	1	0.9004117	-0.09	137	-8.12	12849	-0.09	142	-8.72	13400	-0.02	123	-1.74	9579
TLR6_t1	-1.09	4.09E-09	-0.1	133	-8.12	12850	NA	NA	NA	NA	NA	NA	NA	NA
C1QC	-1.91	1.24E-18	-0.14	110	-8.13	12851	-0.15	108	-8.87	13456	-0.35	104	-18.78	15133
TRIM48	-1.03	0.010872221	-0.14	108	-8.13	12852	-0.16	116	-10.7	14129	-0.06	138	-5.21	11120
APOM_t1	-1.73	8.73E-07	-0.18	94	-8.13	12853	-0.17	106	-9.74	13792	NA	NA	NA	NA
UNC5A	-1.03	0.025063153	-0.16	100	-8.15	12854	-0.13	133	-10.81	14181	-0.13	112	-7.77	12150
RYR2	1.08	0.032088455	-0.12	120	-8.15	12855	-0.04	131	-3.3	10672	-0.04	143	-4	10643
UBXN4	-1.01	0.714202798	-0.1	133	-8.15	12856	0.04	115	2.58	6777	-0.04	136	-3.81	10541
TANC1_t1	-1.42	2.19E-07	-0.09	141	-8.16	12857	NA	NA	NA	NA	-0.19	150	-18.9	15156
KCNC3	-1.21	3.78E-14	-0.16	100	-8.16	12858	-0.26	126	-19.99	15983	-0.2	112	-12.56	13712
FAM181B	-1.03	0.036126572	-0.13	115	-8.16	12859	-0.19	115	-12.61	14668	-0.17	104	-9.4	12718
TCEA2	-1.19	0.024055154	-0.21	89	-8.17	12860	-0.23	118	-15.94	15404	-0.12	130	-9.82	12861
ICAM3	-1.13	0.029825042	-0.22	88	-8.17	12861	-0.08	120	-5.42	11878	-0.26	90	-10.28	13018
DAB1	-1.05	0.013776501	-0.18	95	-8.18	12862	-0.16	98	-7.78	13019	-0.1	111	-6.15	11498
TCTN3	-1.08	0.021931199	-0.14	107	-8.18	12863	-0.04	124	-3.1	10538	-0.11	126	-8.04	12245
TPSG1	-1.16	0.000507667	-0.16	100	-8.18	12864	-0.15	113	-9.43	13670	-0.16	108	-9.07	12614
FBXL18	-1.04	0.066641817	-0.09	144	-8.19	12865	-0.18	147	-17.48	15670	-0.1	152	-10.54	13101
PTGIR	-1.04	0.007730278	-0.14	109	-8.19	12866	-0.09	129	-7.46	12855	-0.14	106	-7.7	12116
CD200R1_t1	-1.17	3.83E-11	-0.13	115	-8.19	12867	-0.12	121	-8.8	13432	-0.21	122	-15.21	14419
HDGFRP3	-1.21	0.016851466	-0.11	127	-8.19	12868	0.01	141	0.52	8128	-0.21	118	-14.03	14119
SLFN13	-1.05	0.17696266	-0.09	138	-8.19	12869	0.02	126	1.45	7523	-0.1	139	-8.78	12521
LCN12	-1.29	2.08E-12	-0.22	88	-8.19	12870	-0.28	93	-12.12	14542	-0.25	100	-12.43	13677
SCGB3A2	-1.03	0.188977818	-0.13	114	-8.2	12871	-0.13	110	-7.68	12972	-0.08	88	-3.03	10194
SNX18	-1.08	1.69E-05	-0.12	119	-8.2	12872	-0.23	118	-15.89	15388	-0.05	143	-4.72	10944
PNPO	-1.08	0.026998639	-0.11	122	-8.2	12873	-0.16	127	-12.06	14521	-0.09	150	-8.79	12522
PRSS22	-1.24	1.56E-08	-0.14	109	-8.2	12874	-0.08	105	-4.48	11377	-0.3	93	-12.71	13764
SLC2A4RG	1.02	0.788083989	-0.1	130	-8.21	12875	-0.13	124	-9.6	13736	0.06	140	5.77	5549
RERG	-1.17	0.003781124	-0.1	134	-8.21	12876	-0.01	133	-0.61	8878	-0.12	140	-10.98	13252
MIER1_t2	-1.28	2.37E-06	-0.1	132	-8.22	12877	-0.06	136	-5.15	11748	-0.18	157	-19.22	15206
THNSL2	-1.26	4.04E-06	-0.12	116	-8.23	12878	-0.2	131	-15.84	15371	-0.11	100	-5.75	11334
CCDC92	-1.07	0.001240363	-0.16	100	-8.23	12879	0.03	128	2.31	6950	-0.25	120	-17.2	14833
MAPK15	-1.06	0.000115105	-0.17	98	-8.24	12880	-0.11	105	-6.23	12299	-0.07	100	-3.61	10440
FAM133B_t1	-1.42	7.19E-12	-0.09	140	-8.25	12881	-0.04	120	-2.59	10213	NA	NA	NA	NA
CD79A	-1.35	9.66E-12	-0.14	108	-8.26	12882	-0.14	132	-11.82	14454	-0.32	110	-18.98	15169
POMT2	1.07	0.018559121	-0.12	120	-8.26	12883	0.05	147	4.45	5635	-0.07	139	-6.5	11646
SBK1	-1.01	0.623194003	-0.1	132	-8.27	12884	-0.1	148	-9.58	13726	-0.05	130	-3.78	10525
LTBR	-1.18	3.93E-07	-0.16	101	-8.27	12885	-0.15	130	-12.37	14607	-0.13	112	-8.24	12338
ZXDC_t1	-1.08	0.004782965	-0.12	117	-8.27	12886	-0.13	119	-9.09	13556	-0.13	129	-10.43	13064
SPN	1.02	0.085096606	-0.09	144	-8.27	12887	-0.03	143	-2.8	10343	-0.03	153	-3.23	10283
TMEM30B	-1.01	0.335685879	-0.09	144	-8.27	12888	-0.12	128	-9.02	13516	-0.05	126	-4.03	10652
CST9	-1.07	1.53E-06	-0.13	115	-8.28	12889	-0.16	115	-10.53	14080	-0.1	130	-7.89	12192
SLC35G5	-1.04	0.004671411	-0.15	104	-8.28	12890	-0.05	68	-0.94	9104	-0.04	81	-1.38	9383
IFNA5	-1.01	0.15805033	-0.13	115	-8.28	12891	-0.06	165	-7.35	12817	-0.04	100	-2.19	9789
CCDC115_t1	-1.14	3.00E-06	-0.12	120	-8.29	12892	NA	NA	NA	NA	NA	NA	NA	NA
PDXP	-1.08	0.030380178	-0.1	132	-8.29	12893	-0.19	120	-13.59	14893	-0.02	103	-0.96	9173
EGFL7	-1.37	7.24E-09	-0.2	92	-8.3	12894	-0.09	98	-4.18	11191	-0.24	98	-11.54	13414
ATAD3C	-1.28	2.57E-08	-0.12	120	-8.3	12895	-0.14	143	-13.31	14829	-0.19	152	-19.89	15307
SLC20A1_t1	-1.3	1.59E-07	-0.11	128	-8.3	12896	NA	NA	NA	NA	-0.18	113	-11.26	13330
LRRN2	-1.18	7.74E-06	-0.11	124	-8.3	12897	-0.1	120	-7.17	12723	-0.2	120	-13.94	14096
ARHGAP24_t1	-1.24	4.04E-07	-0.09	140	-8.3	12898	NA	NA	NA	NA	-0.03	145	-3.01	10181
NDRG4	-1.07	0.075052296	-0.12	122	-8.31	12899	-0.13	129	-10.27	13990	-0.19	108	-11.11	13285
NUCB1	-1.13	0.000163432	-0.14	110	-8.31	12900	-0.28	120	-19.91	15974	-0.18	108	-10.22	12994
ZC3HAV1L	-1.05	0.05494024	-0.1	131	-8.31	12901	-0.18	115	-11.92	14485	-0.09	147	-8.33	12374
TGOLN2	-1.11	0.022885408	-0.09	140	-8.31	12902	0.02	130	1.64	7400	-0.07	171	-8.73	12500
CDH15	-1.23	0.008745073	-0.22	88	-8.31	12903	0.02	120	1.58	7447	-0.12	94	-5.24	11133
SLC26A10	-1.25	3.49E-11	-0.17	100	-8.31	12904	-0.07	124	-5.02	11667	-0.21	123	-15.5	14480
SMPDL3A	-1.26	1.67E-05	-0.15	104	-8.31	12905	-0.12	93	-4.96	11632	-0.08	125	-6.09	11475
NTF4	-1.05	0.012988743	-0.1	134	-8.32	12906	0.05	108	2.67	6700	-0.06	103	-3.2	10269
MNDA	-2.26	4.74E-16	-0.17	98	-8.32	12907	-0.08	128	-6.11	12244	-0.29	122	-20.98	15500
PRR5_t1	-1.31	6.67E-06	-0.17	100	-8.32	12908	NA	NA	NA	NA	-0.18	132	-14.64	14280
PGR	1.02	0.353525106	-0.08	160	-8.32	12909	-0.02	150	-2.1	9899	-0.01	150	-1.1	9245
FAM3D	-1.07	0.02412781	-0.12	120	-8.33	12910	-0.03	108	-1.84	9723	-0.2	122	-14.63	14276
NEO1	-1.1	0.052035692	-0.1	132	-8.33	12911	-0.03	139	-2.43	10105	-0.09	145	-8.1	12268
GATA4	-1.28	1.13E-06	-0.12	120	-8.33	12912	-0.22	134	-18.7	15824	-0.19	126	-14.67	14291
PGM3	-1.27	0.000737294	-0.11	126	-8.34	12913	0.01	135	1.08	7751	-0.11	131	-9.16	12643
BTLA	-1.06	0.040886027	-0.08	149	-8.34	12914	0.02	132	1.56	7460	-0.2	135	-16.59	14713
POLDIP2	1.05	0.224642384	-0.1	135	-8.34	12915	-0.04	122	-3.12	10561	0.02	123	1.26	7873
EPS8L1	-1.44	8.69E-08	-0.19	94	-8.34	12916	-0.03	119	-1.74	9641	-0.32	117	-21.73	15622
PIAS4	-1.09	0.000260722	-0.22	88	-8.36	12917	-0.15	126	-11.16	14285	-0.23	107	-13.35	13936
CD47_t2	-1.09	0.008433812	-0.09	143	-8.36	12918	0.06	150	6.19	4669	-0.08	146	-7.3	11959
ABCB1	-1.14	0.106459113	-0.13	114	-8.36	12919	-0.11	120	-7.79	13027	0.07	123	4.83	6010
BRWD1_t3	-1.15	0.001279093	-0.09	148	-8.37	12920	-0.07	120	-5.06	11692	-0.11	137	-9.36	12699
TFAP2B	-1.04	0.35907322	-0.1	138	-8.37	12921	-0.13	152	-13.3	14826	-0.12	141	-10.83	13192
COQ5	-1.03	0.515698564	-0.12	122	-8.38	12922	-0.12	96	-5.57	11952	-0.1	116	-6.86	11794
PAX1	1.01	0.587150267	-0.08	151	-8.38	12923	-0.06	131	-5.2	11776	-0.08	167	-9.43	12730
NOD2	-1.18	0.00079545	-0.12	122	-8.38	12924	-0.01	156	-1.18	9271	-0.17	135	-14.81	14328
LFNG_t1	-1.27	8.23E-11	-0.19	95	-8.39	12925	-0.17	118	-11.63	14403	-0.19	109	-11.18	13303
RGS3_t2	-1.28	8.75E-05	-0.12	120	-8.39	12926	-0.02	110	-1.33	9385	-0.22	122	-15.87	14553
PADI3	1.03	0.01205218	-0.11	123	-8.39	12927	0	120	-0.17	8595	0	106	-0.16	8708
MS4A2	-1.1	0.001248603	-0.11	127	-8.4	12928	0.08	138	7.44	4013	-0.19	127	-14.33	14198
IAPP	-1.05	0.072827198	-0.12	118	-8.4	12929	-0.1	135	-8.53	13334	-0.09	132	-7.48	12025
SYVN1_t1	-1.05	0.087839474	-0.12	123	-8.4	12930	NA	NA	NA	NA	NA	NA	NA	NA
GDA_t1	-1.31	3.55E-05	-0.11	126	-8.4	12931	-0.17	142	-15.69	15340	NA	NA	NA	NA
TBC1D22A	-1.02	0.445058276	-0.11	129	-8.4	12932	0	124	0.24	8296	-0.15	136	-12.74	13773
RFTN2	-1.04	0.077827006	-0.11	126	-8.4	12933	0.05	148	4.43	5649	-0.07	121	-5.02	11055
DENND1C	-1.27	2.22E-08	-0.17	100	-8.41	12934	-0.01	117	-0.75	8967	-0.28	122	-20.16	15359
ADPGK	-1.09	0.001331587	-0.11	126	-8.41	12935	-0.02	135	-1.48	9480	-0.11	124	-8.18	12305
HOOK2	-1.22	3.78E-05	-0.11	127	-8.41	12936	-0.12	98	-5.76	12064	-0.15	100	-7.61	12080
SLC52A1	-1.16	3.23E-11	-0.1	133	-8.41	12937	-0.3	159	-33.07	16481	-0.17	106	-9.47	12751
FANCL	-1.14	0.016202206	-0.11	127	-8.42	12938	-0.08	116	-5.44	11888	-0.13	119	-9.09	12620
FSHR	-1.08	1.37E-06	-0.12	118	-8.42	12939	-0.16	122	-11.67	14420	-0.14	132	-11.09	13281
RBP4	-1.33	0.000355334	-0.14	112	-8.42	12940	-0.33	104	-17.72	15700	-0.12	125	-8.74	12507

SMG1_t1	-1.18	3.15E-06	-0.1	135	-8.42	12941	NA	NA	NA	NA	-0.1	155	-10.04	12938
HPS6	-1.16	3.93E-06	-0.11	126	-8.43	12942	-0.19	123	-14.08	15008	-0.19	90	-7.5	12034
SNAI3	-1.19	1.88E-09	-0.16	104	-8.43	12943	-0.1	140	-8.75	13417	-0.28	118	-18.97	15167
ASGR1	-1.46	0.000349389	-0.16	104	-8.43	12944	-0.21	104	-11.55	14389	-0.16	119	-10.72	13161
CEBPE	-1.5	4.96E-14	-0.21	91	-8.44	12945	-0.21	97	-9.88	13839	-0.29	153	-30.09	16290
TP53INP2	-1.17	0.002064916	-0.12	122	-8.44	12946	-0.27	133	-22.22	16189	-0.1	139	-9.27	12679
AIM1	-1.43	1.43E-11	-0.13	116	-8.44	12947	-0.12	119	-8.13	13170	-0.21	115	-13.38	13949
AQP8	-1.08	0.01690248	-0.11	129	-8.44	12948	-0.27	112	-16.83	15571	-0.08	124	-5.82	11374
AJAP1	-1.2	8.88E-10	-0.14	112	-8.44	12949	-0.14	122	-9.8	13820	-0.28	131	-22.64	15730
GC	-1.47	2.85E-06	-0.14	111	-8.44	12950	-0.31	104	-16.75	15557	-0.07	99	-3.34	10329
GNG13	-1.04	0.026466087	-0.21	91	-8.45	12951	-0.12	129	-9.1	13559	-0.13	97	-6.06	11460
TNMD	-1.02	0.461032675	-0.08	151	-8.45	12952	-0.19	95	-8.37	13275	-0.09	92	-3.96	10624
SLC4A3	-1.01	0.831257392	-0.17	100	-8.46	12953	-0.04	158	-4.24	11236	-0.19	99	-9.22	12661
MYH3	-1.02	0.846139063	-0.11	125	-8.46	12954	-0.05	110	-2.73	10295	-0.13	106	-7.54	12044
MLC1	-1.11	0.00012573	-0.12	119	-8.46	12955	0	116	-0.3	8674	-0.26	155	-26.9	16120
STIM1	1.02	0.49071695	-0.12	118	-8.47	12956	-0.11	126	-8.58	13356	0	127	0.3	8438
TSPAN4_t1	-1.39	1.21E-11	-0.18	97	-8.47	12957	-0.16	128	-12.32	14589	NA	NA	NA	NA
KLHL34	1.3	0.000164097	-0.13	115	-8.48	12958	0.08	110	4.56	5582	0.05	105	2.78	7038
SGCD_t1	-1	0.973278349	-0.12	118	-8.48	12959	0.05	119	3.22	6366	-0.18	91	-7.19	11916
MOB2	-1.07	0.005996995	-0.16	103	-8.48	12960	-0.09	124	-6.71	12517	-0.22	120	-15.24	14428
DHH	-1.01	0.587159292	-0.11	124	-8.48	12961	-0.16	138	-13.84	14954	-0.12	114	-7.6	12068
TRERF1	-1.12	0.008892716	-0.09	147	-8.48	12962	0.01	134	1.08	7747	-0.19	154	-20.16	15361
Sep-15	-1.02	0.640704037	-0.09	148	-8.49	12963	0.1	118	6.65	4418	-0.01	123	-0.38	8840
DRD1	1.01	0.811389926	-0.12	118	-8.49	12964	-0.14	135	-12.03	14509	-0.01	138	-1.01	9210
ABCA1	-1.38	4.53E-06	-0.11	128	-8.5	12965	-0.06	127	-4.79	11533	-0.02	147	-2.21	9797
CTSB_t1	-1.44	2.50E-11	-0.1	131	-8.5	12966	NA	NA	NA	NA	-0.25	150	-24.52	15916
PRODH	-1.04	0.675611726	-0.16	104	-8.5	12967	-0.16	119	-10.7	14131	-0.19	108	-10.79	13178
PCOLCE	-2.15	1.09E-15	-0.24	86	-8.5	12968	-0.24	76	-6.31	12329	-0.22	78	-6.24	11546
LTBP4_t1	-1.22	4.79E-15	-0.2	93	-8.5	12969	NA	NA	NA	NA	-0.21	120	-14.71	14299
HCN2	-1.22	0.000758732	-0.1	134	-8.51	12970	-0.21	114	-13.5	14871	-0.14	137	-12.05	13561
PRAC	-1.23	2.99E-12	-0.22	89	-8.51	12971	-0.08	86	-2.96	10454	-0.19	84	-6.49	11637
DDT	-1.5	1.74E-08	-0.2	93	-8.51	12972	-0.23	130	-18.78	15835	-0.23	100	-11.72	13464
UPK3B	-1.17	1.66E-14	-0.17	100	-8.51	12973	-0.17	123	-12.08	14532	-0.16	154	-16.81	14758
CXCL3	-1.32	2.93E-05	-0.12	122	-8.51	12974	-0.04	144	-3.98	11095	-0.23	124	-16.78	14749
SLC43A1	-1.29	3.48E-05	-0.13	114	-8.51	12975	-0.15	133	-12.33	14594	-0.12	103	-6.22	11531
SLC25A10	-1.04	0.567857027	-0.13	114	-8.51	12976	-0.12	116	-8.21	13197	-0.05	121	-3.79	10528
NFE2	-1.77	1.11E-14	-0.17	100	-8.51	12977	-0.17	108	-9.71	13779	-0.27	106	-15.21	14421
SPECC1_t2	-1.2	0.013533492	-0.1	132	-8.52	12978	0.07	145	6.77	4367	-0.21	128	-16.64	14720
TXNRD2	-1.29	5.46E-10	-0.16	103	-8.52	12979	-0.18	97	-8.48	13316	-0.21	100	-10.62	13123
ZADH2	-1.09	3.68E-12	-0.08	156	-8.52	12980	-0.03	134	-2.64	10239	-0.14	147	-13.73	14031
EEPD1	1.02	0.79009187	-0.1	132	-8.52	12981	-0.04	128	-3.03	10490	-0.05	141	-4.55	10868
PPP5C	-1.06	0.003657792	-0.15	105	-8.52	12982	-0.11	100	-5.68	12016	-0.18	127	-14.03	14120
LCE1D	-1.26	2.71E-06	-0.16	103	-8.52	12983	-0.15	84	-4.97	11638	-0.17	88	-6.34	11590
ENPP3	-1.07	0.380951677	-0.09	144	-8.53	12984	-0.07	111	-4.03	11116	0	110	0.05	8584
AGER	-1.09	7.51E-05	-0.17	100	-8.53	12985	-0.09	106	-5.12	11732	-0.21	131	-17.3	14848
CAAP1	-1.05	0.322837597	-0.11	131	-8.53	12986	-0.08	133	-6.66	12496	0.01	120	0.38	8391
MTG1	-1.07	0.188691643	-0.12	122	-8.53	12987	-0.04	111	-2.2	9965	-0.08	109	-4.68	10927
CD14	-2.74	1.73E-25	-0.25	84	-8.53	12988	-0.34	90	-13.68	14916	-0.33	98	-15.91	14565
GIPC1	-1.06	0.02608665	-0.15	106	-8.53	12989	-0.12	108	-6.78	12553	-0.12	96	-5.7	11318
PHLPP1	-1.2	6.49E-05	-0.13	116	-8.54	12990	-0.19	126	-14.3	15054	-0.2	116	-13.09	13865
CASP10_t2	-1.2	2.48E-06	-0.09	145	-8.54	12991	-0.02	138	-1.74	9647	NA	NA	NA	NA
TEX2	-1.13	0.01016459	-0.11	128	-8.54	12992	-0.09	133	-7.36	12819	-0.08	148	-8.14	12286
HECTD3	-1.11	6.09E-07	-0.08	160	-8.54	12993	-0.19	152	-19	15869	-0.21	127	-16.31	14649
MATN2	-1.04	0.347635507	-0.11	131	-8.54	12994	-0.07	121	-5.24	11796	-0.02	132	-1.83	9627
WIPF1_t1	-1.18	7.76E-07	-0.11	130	-8.55	12995	NA	NA	NA	NA	-0.25	126	-19.33	15223
GSTM2	-1.2	1.06E-07	-0.11	127	-8.55	12996	-0.08	127	-5.99	12182	-0.19	120	-12.96	13835
CD37	-1.26	9.28E-09	-0.13	117	-8.55	12997	-0.03	138	-2.36	10058	-0.29	106	-16.22	14629
TOX_t1	1.03	0.479379189	-0.1	135	-8.55	12998	NA	NA	NA	NA	-0.1	137	-8.72	12495
PLOD1	-1.04	0.36303395	-0.12	124	-8.55	12999	-0.1	133	-8.17	13179	-0.02	124	-1.48	9436
STK31	1.13	0.043239233	-0.1	133	-8.55	13000	0	90	0	8476	0.02	113	1.15	7943
CARD16	-1.66	3.35E-13	-0.14	113	-8.56	13001	-0.07	89	-2.84	10372	-0.31	107	-17.55	14894
TIMP2	-1.5	3.59E-06	-0.1	132	-8.56	13002	0.02	136	1.9	7216	-0.28	152	-28.85	16250
PTPRCAP	-1.14	1.48E-05	-0.16	104	-8.56	13003	-0.13	120	-8.98	13494	-0.21	88	-7.95	12215
RHOH	-1.41	2.63E-07	-0.13	115	-8.56	13004	-0.08	127	-6.07	12224	-0.29	115	-18.96	15165
TBC1D2	-1.04	0.461425451	-0.13	114	-8.57	13005	-0.16	114	-10.39	14035	-0.09	110	-5.12	11090
HRASLS5	-1.04	0.015156055	-0.1	136	-8.57	13006	-0.17	157	-18.09	15747	-0.14	126	-10.3	13026
MRFAP1L1	-1.03	0.387087119	-0.11	126	-8.57	13007	-0.15	119	-10.32	14008	-0.01	107	-0.78	9076
OXER1	-1.15	3.00E-08	-0.23	88	-8.57	13008	-0.28	116	-18.3	15772	-0.15	108	-8.58	12448
IPPK	-1.2	2.50E-06	-0.1	136	-8.57	13009	-0.07	152	-6.72	12525	-0.2	125	-14.73	14304
MAP9_t1	-1.15	0.006152043	-0.08	152	-8.57	13010	NA	NA	NA	NA	-0.15	134	-12.93	13826
STK32A	-1.03	0.003757847	-0.15	107	-8.57	13011	-0.08	101	-3.87	11013	-0.08	110	-4.88	11010
ABCB11	-1.58	4.54E-06	-0.13	114	-8.58	13012	-0.13	115	-8.35	13262	-0.08	120	-5.66	11299
MST1R	-1.17	3.87E-05	-0.16	103	-8.58	13013	-0.15	122	-11.08	14266	-0.14	100	-6.94	11822
OSBPL9	-1.4	1.36E-05	-0.1	139	-8.59	13014	-0.08	116	-5.32	11830	-0.12	134	-9.74	12827
NAB2_t1	-1.16	9.14E-09	-0.16	105	-8.59	13015	NA	NA	NA	NA	-0.24	139	-21.68	15616
LRRC28	-1.32	3.52E-07	-0.16	104	-8.59	13016	-0.2	129	-15.85	15377	-0.15	114	-9.76	12835
PRKG1	1.01	0.847374965	-0.1	137	-8.59	13017	0	135	0.22	8314	-0.02	155	-1.76	9597
CST7	-1.37	1.08E-11	-0.2	92	-8.6	13018	-0.1	135	-8.52	13331	-0.36	89	-13.99	14108
SYTL3_t1	-1.82	1.24E-14	-0.17	100	-8.61	13019	NA	NA	NA	NA	-0.35	107	-20.05	15340
MPV17L2	1.04	0.387131762	-0.14	112	-8.61	13020	-0.06	118	-4.01	11101	0	108	-0.18	8722
ARSA	-1.06	0.0990279	-0.11	131	-8.62	13021	-0.13	152	-13.57	14883	-0.15	152	-15.14	14404
HYAL3	1.17	0.003812875	-0.13	115	-8.62	13022	0.02	134	1.35	7574	-0.01	120	-0.65	9013
GZMH	-1.76	4.79E-13	-0.28	81	-8.62	13023	-0.07	100	-3.61	10886	-0.39	102	-20.04	15338
PLTP	-1.74	4.24E-12	-0.16	104	-8.62	13024	-0.08	118	-5.48	11904	-0.29	99	-14.27	14180
UBAP1	-1.2	1.58E-07	-0.13	116	-8.62	13025	-0.21	114	-13.27	14818	-0.15	137	-13.15	13888
GRAP2	1	0.95578417	-0.11	126	-8.63	13026	-0.03	125	-2.6	10215	-0.1	125	-7.81	12168
PLXNA3	-1.09	8.35E-06	-0.12	122	-8.63	13027	-0.22	143	-20.7	16066	-0.09	121	-6.71	11736
DSG3	-1	0.910513455	-0.08	154	-8.63	13028	-0.02	128	-1.79	9685	-0.02	125	-1.85	9639
ZNF511	-1.25	8.38E-07	-0.18	98	-8.63	13029	-0.24	124	-17.88	15718	-0.13	111	-8.09	12263
RNF151	-1.11	1.85E-12	-0.26	83	-8.63	13030	-0.16	81	-4.9	11600	-0.25	68	-4.41	10805
P2RY10	-1.02	0.166058032	-0.13	116	-8.63	13031	-0.06	122	-3.97	11088	-0.15	119	-10.24	13005
XCR1	-1.45	4.87E-18	-0.23	88	-8.63	13032	-0.26	100	-13.01	14757	-0.21	116	-13.89	14076
ST3GAL1	-1.21	0.021478213	-0.11	129	-8.64	13033	-0.14	139	-12.06	14528	-0.03	131	-2.36	9874
IL8	-1.57	0.000139638	-0.1	135	-8.64	13034	-0.04	144	-3.88	11025	-0.17	135	-14.3	14190
PIH1D3	-1	0.904242215	-0.09	150	-8.64	13035	-0.01	110	-0.39	8737	-0.03	125	-2.2	9792
SEC14L3_t1	-1.36	0.000486186	-0.11	128	-8.64	13036	NA	NA	NA	NA	-0.13	137	-10.96	13244
RHOBTB1	-1.18	0.113282343	-0.11	130	-8.64	13037	0.02	119	1.54	7473	-0.18	112	-11.26	13329
ZNF423	-1.02	0.220948243	-0.12	124	-8.64	13038	0.03	117	2.18	7016	-0.13	131	-10.85	13198
NRBF2	-1.4	1.99E-15	-0.11	126	-8.65	13039	-0.21	131	-16.79	15562	-0.14	147	-13.58	13992
ACTL6B	-1.24	4.82E-07	-0.24	86	-8.65	13040	-0.1	126	-7.79	13030	-0.18	111	-11.1	13282
NAT8B	-1.76	4.64E-08	-0.23	88	-8.65	13041	-0.17	92	-7.34	12808	-0.19	88	-7.06	11867
BARHL1	-1.17	3.20E-07	-0.19	95	-8.65	13042	-0.21	139	-18.68	15822	-0.17	88	-6.42	11611
PIP4K2A	-1.35	1.94E-10	-0.1	135	-8.65	13043	-0.05	148	-4.47	11369	-0.3	127	-22.9	15765
GLTPD1_t1	-1.26	1.49E-08	-0.12	124	-8.65	13044	NA	NA	NA	NA	NA	NA	NA	NA
USP7	-1.03	0.371527653	-0.13	115	-8.65	13045	0	120	0.35	8239	-0.03	131	-2.54	9973
ALKBH8	-1.01	0.600722629	-0.08	158	-8.66	13046	-0.06	120	-4.29	11259	-0.08	123	-5.81	11372

STK10	-1.32	4.59E-10	-0.11	126	-8.66	13047	0	139	-0.23	8631	-0.26	131	-21.01	15503
LMX1A	-1.07	0.000835278	-0.1	133	-8.66	13048	-0.19	132	-15.18	15245	-0.15	126	-11.77	13478
LRRC6	-1.03	0.512705378	-0.13	116	-8.66	13049	0.05	110	2.91	6547	0	119	-0.31	8797
RHOT2	-1.14	5.60E-06	-0.15	106	-8.67	13050	-0.1	120	-7.19	12731	-0.21	123	-15.14	14405
HSPB2	-1.92	8.10E-12	-0.19	95	-8.67	13051	-0.08	120	-5.72	12040	-0.3	87	-10.95	13239
RBM25	-1.16	0.001574105	-0.1	135	-8.67	13052	0	128	0.25	8292	-0.12	155	-12.1	13576
PSMG1	-1.03	0.438243441	-0.1	134	-8.67	13053	-0.05	112	-3.08	10522	-0.03	120	-1.83	9628
INSIG1	-1.74	6.14E-06	-0.12	120	-8.67	13054	-0.26	139	-22.75	16234	-0.12	144	-11.61	13431
STAC2	1.06	0.021961113	-0.08	157	-8.68	13055	-0.05	130	-3.77	10965	-0.03	136	-2.35	9869
CRP	-1.89	0.000687957	-0.08	153	-8.68	13056	-0.14	124	-10.1	13918	-0.15	127	-11.28	13339
USF2	-1.23	4.75E-14	-0.16	104	-8.68	13057	-0.18	109	-10.8	14176	-0.22	108	-12.96	13833
TPRG1	-1.43	1.04E-05	-0.11	128	-8.68	13058	-0.1	152	-10.1	13914	-0.17	139	-15.24	14429
ZFP36L2	-1.3	8.25E-05	-0.09	150	-8.68	13059	0.01	123	1.06	7768	-0.18	153	-19.02	15179
PVRL3	-1.33	5.30E-06	-0.08	154	-8.68	13060	-0.16	123	-11.59	14396	-0.11	156	-11.15	13296
RDH11	-1.06	0.366354361	-0.11	128	-8.68	13061	-0.15	119	-10.1	13920	-0.04	154	-4.43	10814
MEIS2_t2	-1.15	0.00913904	-0.11	128	-8.69	13062	-0.05	160	-5.64	11999	-0.08	118	-5.5	11242
TMEM129	-1.12	0.004638636	-0.11	128	-8.69	13063	-0.23	123	-16.7	15548	-0.06	121	-4.61	10900
CD38	-1.74	2.05E-09	-0.11	126	-8.69	13064	-0.08	116	-5.49	11910	-0.32	111	-19.76	15290
C3AR1	-1.34	7.30E-11	-0.13	115	-8.69	13065	-0.07	112	-4.36	11294	-0.26	108	-14.87	14346
DGKQ	-1.02	0.257916791	-0.13	118	-8.7	13066	-0.08	120	-5.85	12112	-0.17	120	-11.84	13501
MSMO1	-1.46	5.35E-06	-0.08	153	-8.7	13067	-0.17	135	-14.57	15118	-0.07	144	-6.55	11669
NIPSNAP3A	-1.19	0.001480717	-0.09	146	-8.7	13068	-0.08	116	-5.33	11833	-0.1	129	-7.88	12190
SYK	-1.42	3.62E-09	-0.1	133	-8.7	13069	0.04	134	2.94	6524	-0.27	152	-27.58	16163
CORO1A	-1.58	8.80E-13	-0.2	93	-8.7	13070	-0.06	106	-3.6	10878	-0.37	88	-14.11	14141
LUC7L	-1.29	3.08E-12	-0.13	117	-8.71	13071	-0.07	109	-3.93	11056	-0.23	101	-11.96	13526
FAM178B	-1.24	1.99E-13	-0.22	90	-8.71	13072	-0.26	113	-16.4	15493	-0.2	100	-9.97	12916
PREX1	-1.52	1.30E-09	-0.11	130	-8.71	13073	0.02	137	1.75	7317	-0.27	120	-18.68	15120
PRODH2	-2.15	1.04E-11	-0.32	77	-8.71	13074	-0.27	88	-10.19	13947	-0.26	68	-4.67	10924
DCP1A	-1.07	0.094455654	-0.1	135	-8.72	13075	0.05	131	4.36	5683	-0.18	131	-14.22	14162
DYRK1B	-1.03	0.045882165	-0.14	113	-8.72	13076	-0.05	111	-3.25	10638	-0.17	120	-11.92	13516
CDR2L	-1.14	7.05E-05	-0.12	121	-8.73	13077	-0.04	135	-3.28	10660	-0.23	122	-16.51	14686
NELL2	1.01	0.828122724	-0.12	121	-8.73	13078	-0.06	130	-4.42	11341	-0.06	131	-4.46	10824
EIF4A2_t1	-1.12	0.064129955	-0.09	147	-8.73	13079	-0.02	108	-0.9	9079	NA	NA	NA	NA
EPO	-1.34	1.12E-05	-0.12	124	-8.73	13080	-0.1	149	-9.96	13866	-0.24	102	-12.32	13653
ST18	1	0.998004542	-0.09	152	-8.74	13081	-0.02	142	-1.66	9594	-0.03	165	-3.53	10408
NDOR1	-1.04	0.045023929	-0.12	120	-8.74	13082	0.01	141	0.66	8033	-0.19	120	-13.22	13905
FKBP7	-1.03	0.546764352	-0.11	130	-8.75	13083	0.02	114	1.53	7481	-0.02	134	-2.01	9709
P4HA1	-1.65	1.17E-07	-0.1	140	-8.75	13084	-0.25	157	-27.23	16420	-0.08	140	-7.55	12048
COL6A2_t1	-1.92	1.09E-17	-0.22	90	-8.75	13085	-0.13	136	-11.23	14307	-0.31	113	-19.72	15284
TIAM1	-1.07	0.000235265	-0.11	127	-8.76	13086	-0.14	132	-11.54	14383	-0.13	130	-10.22	12997
CLPP	-1.29	3.06E-11	-0.18	100	-8.76	13087	-0.19	117	-12.76	14706	-0.17	127	-12.94	13830
LONP2	-1.34	9.41E-10	-0.09	147	-8.76	13088	-0.14	139	-12.55	14659	-0.13	150	-12.6	13728
H1FX	-1.09	0.08578364	-0.14	112	-8.76	13089	-0.03	113	-2.1	9901	-0.19	110	-11.67	13445
VCAN	-1.03	0.775894557	-0.1	138	-8.77	13090	0.08	127	6.35	4591	-0.14	141	-12.52	13703
SLC29A2_t1	-1.21	1.15E-12	-0.14	114	-8.77	13091	NA	NA	NA	NA	-0.14	120	-9.92	12902
FBLN2	-1.48	8.81E-06	-0.11	132	-8.77	13092	-0.04	137	-3.83	10994	-0.23	135	-19.71	15283
PRDM1	-1.11	0.007909603	-0.1	134	-8.77	13093	0.02	140	1.54	7467	-0.17	136	-14.91	14358
TMEM179B	-1.04	0.174587947	-0.14	114	-8.78	13094	-0.07	108	-4.34	11284	-0.12	105	-6.6	11689
LONRF3	-1.14	1.30E-05	-0.12	126	-8.78	13095	-0.19	104	-10.03	13893	-0.08	114	-5.07	11075
RNF5	-1.09	0.011265437	-0.1	139	-8.78	13096	-0.2	126	-15.06	15213	-0.02	131	-1.97	9699
ZC2HC1C_t1	-1.1	1.45E-08	-0.14	114	-8.78	13097	-0.19	129	-15.32	15276	-0.17	130	-13.77	14047
GIPR	-1.08	0.003336302	-0.14	111	-8.78	13098	-0.21	111	-12.7	14691	-0.18	107	-9.98	12921
NHS	-1.1	0.00327766	-0.08	162	-8.78	13099	0.01	133	0.63	8050	-0.11	150	-10.86	13200
ECI1	-1.49	8.13E-12	-0.22	90	-8.78	13100	-0.22	91	-9.08	13551	-0.24	108	-14.2	14156
CBFB_t2	-1.29	5.23E-10	-0.09	143	-8.78	13101	-0.06	137	-4.95	11627	-0.27	145	-25.38	15985
DYNLRB2	-1.05	0.250558126	-0.16	106	-8.78	13102	-0.12	114	-7.69	12976	-0.13	100	-6.7	11735
PDE9A	-1.21	0.004069654	-0.17	101	-8.79	13103	-0.01	96	-0.54	8841	-0.21	108	-12.2	13608
NPNT	-1.12	0.191054363	-0.12	124	-8.79	13104	0.04	128	2.87	6570	-0.15	160	-15.95	14577
HTRA3_t1	-1.4	0.004728197	-0.13	119	-8.79	13105	NA	NA	NA	NA	NA	NA	NA	NA
PSG7	-1	0.935867131	-0.12	122	-8.8	13106	-0.08	126	-5.93	12148	-0.07	124	-5.42	11206
LFNG_t2	-1.25	0.00121897	-0.13	120	-8.8	13107	0.08	120	5.58	5022	-0.18	127	-14.07	14133
HLA-DRB1	-1.76	4.09E-10	-0.15	108	-8.8	13108	-0.05	119	-3.27	10649	-0.34	118	-22.82	15759
NR4A2	-2.34	6.45E-17	-0.13	117	-8.81	13109	-0.19	142	-17.21	15619	-0.24	120	-16.68	14732
SLC38A5	-1.16	4.62E-09	-0.18	98	-8.81	13110	-0.23	106	-12.69	14687	-0.21	101	-10.83	13187
CYB5R2	-1.53	1.32E-09	-0.14	113	-8.81	13111	-0.03	106	-1.9	9749	-0.22	112	-13.56	13987
FAHD1	-1.12	0.024108951	-0.14	114	-8.81	13112	-0.08	140	-7.32	12799	-0.04	120	-2.5	9951
PHEX	-1.05	0.034209052	-0.12	123	-8.81	13113	-0.16	98	-7.68	12973	-0.1	130	-7.87	12185
IZUMO4_t2	-1.13	6.57E-05	-0.28	82	-8.81	13114	-0.19	104	-10.16	13937	-0.21	84	-7.02	11852
TF	-1.48	0.002112217	-0.17	102	-8.81	13115	-0.24	131	-19.41	15916	-0.11	118	-7.49	12031
CNIH2	-1.08	0.010278611	-0.15	108	-8.81	13116	-0.18	108	-10.71	14138	-0.16	113	-10.13	12967
MAPRE3	-1.07	1.99E-05	-0.11	128	-8.82	13117	-0.17	124	-12.62	14672	-0.16	146	-15.44	14465
NDUFV1	-1.06	0.128782336	-0.12	123	-8.82	13118	-0.1	114	-6.26	12311	-0.1	72	-2.22	9802
SLC25A11	-1.32	2.34E-11	-0.14	114	-8.83	13119	-0.33	100	-16.44	15499	-0.19	116	-12.73	13768
CRHR2	-1.01	0.355510072	-0.13	118	-8.84	13120	-0.08	127	-5.91	12137	-0.06	123	-4.38	10785
NR4A3	-1.13	4.63E-05	-0.16	107	-8.84	13121	-0.08	119	-5.56	11946	-0.21	123	-15.12	14400
PLEKHG3	-1.16	0.002068773	-0.14	114	-8.85	13122	-0.14	121	-10.06	13903	-0.11	123	-8.17	12300
POLR3GL	1.05	0.240089078	-0.12	123	-8.85	13123	-0.04	142	-3.85	11006	-0.01	108	-0.46	8890
RNF168	-1.02	0.245675508	-0.1	136	-8.86	13124	-0.08	135	-7.09	12689	-0.08	139	-6.84	11789
KIFC3	-1.16	2.65E-06	-0.13	116	-8.86	13125	-0.21	119	-14.18	15022	-0.22	115	-14.56	14256
FBXL3	-1.37	5.35E-08	-0.12	122	-8.86	13126	-0.09	134	-7.65	12954	-0.2	133	-16.55	14701
TMEM186	1.05	0.155878007	-0.1	136	-8.86	13127	0	127	0	8481	0	103	0.21	8489
PBOV1	1.01	0.412993195	-0.1	136	-8.86	13128	-0.07	146	-7.19	12734	-0.05	153	-4.66	10915
SGCA	-2.31	3.68E-21	-0.22	91	-8.86	13129	-0.16	120	-11.51	14374	-0.35	107	-20.16	15360
THEM5	-1.35	2.10E-12	-0.22	90	-8.87	13130	-0.13	126	-9.58	13729	-0.27	80	-8.07	12256
SWSAP1	1	0.913905316	-0.1	139	-8.87	13131	-0.07	143	-6.65	12489	-0.11	148	-11.22	13312
MLIP	-1.69	0.00010022	-0.14	115	-8.87	13132	-0.2	92	-8.58	13355	-0.05	120	-3.26	10295
CD33_t1	-1.44	1.18E-14	-0.15	110	-8.88	13133	NA	NA	NA	NA	-0.31	118	-20.79	15465
CTBP2_t1	-1.09	0.000669848	-0.1	142	-8.88	13134	0	135	-0.1	8553	-0.2	141	-18.24	15029
PPM1M	-1.12	0.000294689	-0.16	107	-8.88	13135	-0.03	116	-1.92	9755	-0.25	105	-13.96	14101
SOX10_t1	1.02	0.140230085	-0.11	134	-8.88	13136	-0.04	124	-3.17	10587	NA	NA	NA	NA
SLC16A14_t1	-1.06	0.419468755	-0.11	134	-8.89	13137	NA	NA	NA	NA	NA	NA	NA	NA
MGAM	-1.03	0.339838635	-0.13	120	-8.89	13138	-0.11	117	-7.33	12802	-0.14	123	-10.02	12930
SIVA1	-1.07	0.224562825	-0.15	108	-8.89	13139	-0.17	122	-12.5	14639	-0.09	141	-7.78	12153
HLA-DRB3	-1.86	6.22E-12	-0.17	101	-8.89	13140	-0.09	107	-4.99	11653	-0.35	118	-23.66	15847
EMID1_t1	-1.04	0.019788484	-0.11	130	-8.89	13141	NA	NA	NA	NA	-0.14	100	-6.76	11760
PERP	-1.27	0.001170176	-0.11	128	-8.89	13142	0.01	124	0.91	7867	-0.05	129	-3.82	10545
KCNV2	-1.02	0.2184184	-0.13	120	-8.89	13143	-0.08	108	-4.4	11321	-0.13	119	-9.12	12634
AEN	-1.15	0.00157817	-0.13	120	-8.9	13144	-0.05	131	-4.09	11143	-0.13	123	-9.25	12670
IL18	-1.81	2.16E-15	-0.18	100	-8.9	13145	0.01	133	0.7	7998	-0.35	143	-32.4	16376
WDR65	-1.02	0.071551008	-0.11	134	-8.9	13146	-0.1	121	-7.03	12664	-0.06	120	-3.92	10597
MMP2	-1.16	0.000344857	-0.13	119	-8.91	13147	-0.03	136	-2.92	10426	-0.26	143	-24.06	15876
APLP2_t1	-1.21	1.31E-06	-0.1	138	-8.91	13148	-0.11	128	-8.91	13471	NA	NA	NA	NA
C1QTNF2	-1.13	6.00E-09	-0.13	120	-8.91	13149	-0.1	137	-8.46	13308	-0.21	135	-18.24	15032
EFHA2	-1.35	9.83E-06	-0.11	134	-8.92	13150	-0.13	142	-11.67	14418	-0.1	136	-8.24	12336
PTRF_t1	-1.14	2.07E-05	-0.1	136	-8.92	13151	NA	NA	NA	NA	-0.22	124	-16.37	14661
UBASH3A	-1.18	4.57E-05	-0.13	118	-8.92	13152	-0.02	109	-0.91	9087	-0.22	105	-11.99	13536

APOBEC3C	-1.35	1.82E-10	-0.17	103	-8.92	13153	-0.06	104	-3.05	10504	-0.34	107	-19.11	15193
CTNNBIP1	-1.01	0.798696524	-0.12	125	-8.92	13154	-0.08	150	-7.84	13046	-0.07	121	-5.25	11140
MPND	-1.07	0.144806447	-0.18	100	-8.92	13155	-0.19	84	-6.42	12379	-0.08	77	-2.29	9839
BRD2	-1.1	0.002475515	-0.1	143	-8.92	13156	-0.06	138	-5.52	11931	-0.15	117	-10.14	12970
IER3	-1.46	0.000669185	-0.11	128	-8.93	13157	0.08	129	6.6	4453	-0.18	114	-11.27	13335
MAK16	-1.27	3.30E-08	-0.09	145	-8.93	13158	-0.13	138	-11.4	14345	-0.12	154	-12.4	13670
PRH2	-1	0.987831308	-0.13	120	-8.94	13159	-0.13	141	-12.03	14508	0.01	141	0.96	8044
CEPT1	-1.25	5.13E-12	-0.13	118	-8.94	13160	-0.16	129	-12.92	14739	-0.16	113	-9.77	12837
SPEF1	1.01	0.512705378	-0.14	115	-8.94	13161	-0.07	120	-5.17	11759	0	108	0.14	8530
P2RX7_t1	-1.05	0.094527198	-0.12	122	-8.94	13162	NA	NA	NA	NA	NA	NA	NA	NA
FAM18B2	-1.32	2.23E-07	-0.15	111	-8.94	13163	-0.13	110	-7.57	12912	-0.16	112	-9.95	12914
WDR64	-1.05	2.99E-05	-0.17	104	-8.94	13164	-0.05	118	-3.31	10675	-0.23	131	-18.54	15089
BST2	-1.59	1.45E-09	-0.13	120	-8.94	13165	-0.2	127	-15.24	15261	-0.23	107	-12.9	13821
NFKB1	-1.29	1.57E-11	-0.12	122	-8.94	13166	-0.07	122	-5.08	11704	-0.23	129	-18.47	15075
HDHD3	-1.05	0.230195541	-0.16	106	-8.95	13167	-0.12	104	-6.56	12444	-0.09	95	-4.23	10727
TRAP1	-1.2	1.09E-06	-0.15	108	-8.95	13168	-0.19	99	-9.13	13568	-0.11	94	-4.72	10946
SPSB4	-1.1	0.005973617	-0.11	129	-8.95	13169	-0.05	131	-3.96	11078	-0.08	136	-7.09	11876
TSC22D2	-1.11	9.07E-10	-0.13	117	-8.96	13170	-0.07	118	-4.69	11482	-0.15	132	-12.7	13762
PLEKHA1	-1.08	0.230116453	-0.11	135	-8.96	13171	-0.05	130	-4.03	11118	0.07	126	5.53	5674
ERN1	-1.05	0.00359188	-0.14	115	-8.96	13172	-0.16	123	-12.03	14506	-0.1	110	-6.17	11505
SVEP1	-1.04	0.000106504	-0.1	136	-8.97	13173	-0.1	127	-7.51	12880	-0.03	117	-1.76	9593
GABRG2_t2	1.02	0.187929248	-0.11	135	-8.97	13174	-0.06	134	-5.12	11730	-0.06	119	-3.93	10604
SDC4	-1.2	4.59E-06	-0.11	130	-8.97	13175	-0.16	127	-12.35	14599	-0.12	149	-12.07	13566
PBX4	-1.41	6.83E-07	-0.14	112	-8.98	13176	0.04	125	2.84	6592	-0.24	101	-12.43	13679
SH3TC1	-1.51	5.88E-16	-0.3	80	-8.98	13177	-0.2	88	-7.55	12898	-0.39	120	-27.51	16160
TMEM123	-1.27	0.003280221	-0.1	140	-8.98	13178	-0.13	125	-10.06	13902	0	137	-0.03	8639
AMPD2	-1.14	0.000193545	-0.15	109	-8.98	13179	-0.12	109	-7.35	12813	-0.21	120	-14.95	14367
FAM212B	-1.06	0.008081379	-0.1	136	-8.98	13180	-0.14	145	-13.5	14870	-0.1	136	-8.85	12544
IFIT2_t1	-1.46	1.35E-05	-0.11	129	-8.98	13181	NA	NA	NA	NA	NA	NA	NA	NA
FAM169B	-1.01	0.733359344	-0.11	134	-8.99	13182	-0.07	112	-4.26	11248	-0.09	136	-8.06	12252
KIAA0226L	-1.49	1.64E-12	-0.1	136	-8.99	13183	-0.06	130	-4.43	11344	-0.25	127	-19.4	15233
KCNA4	1	0.761400618	-0.12	123	-9	13184	0.06	118	4.01	5897	-0.1	159	-11.31	13350
FAM214A	-1.34	3.39E-05	-0.15	112	-9	13185	-0.14	116	-9.44	13674	-0.08	143	-7.65	12093
IL13RA2	-4.83	4.60E-25	-0.18	101	-9	13186	-0.25	92	-10.54	14081	-0.29	92	-12.08	13569
TMC6	-1.58	3.61E-10	-0.17	102	-9	13187	-0.01	114	-0.9	9078	-0.35	100	-17.46	14872
RRAD	-1.47	4.07E-09	-0.16	107	-9.01	13188	-0.1	107	-5.66	12008	-0.29	94	-12.79	13788
CD22	-1.06	0.000127613	-0.16	108	-9.01	13189	0.01	134	0.5	8137	-0.26	132	-21.48	15586
MEST	-1.31	1.49E-05	-0.12	125	-9.02	13190	-0.02	132	-1.3	9363	-0.1	138	-8.65	12472
ATG2B_t1	-1.22	0.000140305	-0.11	134	-9.02	13191	NA	NA	NA	NA	NA	NA	NA	NA
SH2D2A	-1.36	4.80E-12	-0.17	103	-9.02	13192	-0.15	102	-7.88	13063	-0.26	110	-15.47	14471
PPBP	-1.35	1.41E-08	-0.1	136	-9.02	13193	-0.1	125	-7.28	12772	-0.2	131	-16.52	14689
DNAJB4_t1	-1.4	1.21E-08	-0.1	139	-9.03	13194	-0.06	120	-4.48	11373	NA	NA	NA	NA
HBA1	-2.58	2.98E-27	-0.18	99	-9.03	13195	-0.19	107	-10.69	14126	-0.22	100	-11.22	13313
FNBP1	-1.25	4.41E-08	-0.12	126	-9.04	13196	-0.05	127	-3.85	11005	-0.23	151	-22.81	15758
TBC1D9	-1.53	1.99E-07	-0.14	116	-9.04	13197	-0.07	131	-5.62	11988	-0.13	132	-10.58	13108
CALCA_t2	-1.02	0.591273378	-0.09	152	-9.05	13198	-0.05	107	-3	10475	-0.05	118	-3.36	10337
PPP2R5E	-1.08	0.067504796	-0.09	148	-9.05	13199	0	124	0.13	8385	-0.06	138	-5.03	11058
MINPP1	-1.07	0.15689525	-0.09	156	-9.05	13200	-0.03	120	-2.44	10113	0	122	0.11	8552
CXorf65	-1.29	2.89E-08	-0.13	118	-9.05	13201	0.01	109	0.37	8225	-0.25	126	-18.89	15154
CHPF	-1.11	0.000335541	-0.18	100	-9.05	13202	-0.11	127	-8.27	13227	-0.23	100	-11.71	13459
MAGEE1	-1.09	7.99E-07	-0.12	124	-9.06	13203	-0.26	111	-15.74	15349	-0.17	130	-13.38	13946
PRDM14	-1.01	0.499251091	-0.18	99	-9.06	13204	-0.03	104	-1.6	9559	-0.14	100	-7.02	11854
FFAR2	-1	0.799781591	-0.11	133	-9.06	13205	-0.13	135	-10.85	14193	-0.07	120	-5.06	11073
MDN1	-1.15	3.08E-07	-0.18	100	-9.06	13206	-0.16	115	-10.36	14026	-0.13	120	-8.87	12549
ACBD7	-1.05	0.020478627	-0.12	127	-9.07	13207	-0.11	156	-12.16	14551	-0.11	138	-9.24	12668
CRY2	-1.01	0.589332839	-0.13	120	-9.07	13208	-0.18	152	-18.59	15809	-0.06	128	-4.59	10889
KLK8	-1.29	5.63E-09	-0.24	88	-9.07	13209	-0.28	104	-15.02	15201	-0.2	100	-9.79	12849
FAM83E	-1.02	0.029947841	-0.15	109	-9.07	13210	-0.09	139	-7.97	13104	-0.09	145	-8.17	12297
DGKA_t1	-1.17	2.04E-06	-0.15	109	-9.07	13211	NA	NA	NA	NA	NA	NA	NA	NA
APOBR	-1.4	6.79E-12	-0.15	112	-9.08	13212	-0.04	121	-2.84	10373	-0.27	92	-11.23	13314
WNT9B	-1.05	0.025920866	-0.15	111	-9.08	13213	-0.14	119	-9.54	13714	-0.08	112	-5.15	11098
IQGAP1	-1.54	5.27E-10	-0.12	123	-9.08	13214	0.01	158	1.19	7679	-0.22	148	-21.68	15615
CCDC172	-1.07	3.14E-06	-0.13	119	-9.08	13215	-0.17	128	-13.22	14805	-0.1	118	-6.81	11779
OS9_t1	-1.19	2.00E-08	-0.12	124	-9.08	13216	-0.29	120	-20.07	15995	-0.18	121	-12.93	13827
LRRN3	-1.04	0.000133738	-0.13	120	-9.09	13217	-0.16	108	-9.29	13620	-0.1	117	-6.67	11724
SAV1_t1	-1.25	1.63E-07	-0.11	136	-9.09	13218	-0.19	129	-14.97	15193	NA	NA	NA	NA
LRP3	-1.28	1.11E-06	-0.13	121	-9.09	13219	-0.1	120	-7.03	12665	-0.13	120	-8.88	12555
EPS8L2	-1.25	5.26E-06	-0.13	121	-9.1	13220	-0.18	142	-16.38	15489	-0.18	102	-9.36	12698
CADM3	-1.21	3.84E-06	-0.17	104	-9.1	13221	-0.18	132	-14.88	15179	-0.17	128	-13.02	13846
FBXL2	-1.05	0.0874802	-0.13	122	-9.1	13222	0.02	134	1.49	7495	-0.07	121	-4.97	11041
CHST13	-1.06	0.211498704	-0.13	120	-9.1	13223	-0.1	119	-6.95	12624	-0.08	120	-5.65	11297
CCDC149	1	0.954854085	-0.11	135	-9.1	13224	-0.1	135	-8.1	13150	-0.01	120	-0.63	9000
ADAM28_t2	-1.2	0.000202468	-0.1	145	-9.1	13225	-0.01	98	-0.43	8770	-0.16	122	-11.44	13378
FAM167B	-1.32	1.90E-15	-0.25	86	-9.11	13226	-0.24	123	-17.18	15615	-0.29	90	-11.74	13471
NCRNA00185	-1.39	0.000204665	-0.12	123	-9.11	13227	-0.04	124	-2.6	10223	-0.14	120	-9.46	12748
SLC6A1_t1	1.01	0.804305744	-0.13	120	-9.11	13228	-0.12	130	-9.75	13800	NA	NA	NA	NA
FOXE3	-1.13	2.75E-12	-0.11	136	-9.12	13229	-0.17	149	-16.61	15532	-0.19	104	-10.51	13090
LDOC1L_t1	-1.5	7.85E-08	-0.13	122	-9.13	13230	NA	NA	NA	NA	-0.25	132	-20.43	15404
ZC3HAV1_t2	-1	0.927986632	-0.1	144	-9.14	13231	0.06	139	5.61	5002	-0.01	130	-0.77	9065
HOOK1_t1	-1.35	3.01E-10	-0.11	136	-9.14	13232	-0.13	138	-11.48	14367	NA	NA	NA	NA
NFIB_t1	-1.31	2.94E-05	-0.11	132	-9.15	13233	NA	NA	NA	NA	-0.1	163	-11.55	13417
CMAS	-1.17	0.002485515	-0.13	120	-9.15	13234	-0.13	109	-7.66	12961	-0.02	124	-1.47	9430
PCDH20	1.03	0.669128789	-0.12	128	-9.15	13235	-0.1	120	-6.9	12602	-0.09	135	-7.97	12230
MACF1	-1.22	3.80E-11	-0.11	133	-9.15	13236	-0.08	118	-5.29	11812	-0.19	136	-16.19	14625
FN1_t1	-1.15	0.005482761	-0.16	109	-9.15	13237	-0.14	103	-7.67	12965	-0.11	109	-6.51	11647
FAM190B	-1.22	3.25E-06	-0.1	138	-9.15	13238	-0.1	126	-7.96	13100	-0.06	144	-5.8	11359
LMF1	-1.05	0.111806991	-0.13	120	-9.16	13239	-0.13	122	-9.57	13723	-0.11	115	-7.36	11981
ORAI2_t1	-1.04	0.010794614	-0.1	144	-9.17	13240	NA	NA	NA	NA	-0.2	150	-20.39	15395
SLCO3A1	-1.09	0.011236835	-0.12	128	-9.17	13241	-0.09	146	-8.65	13376	-0.14	143	-12.57	13717
LY86	-1.63	1.40E-16	-0.16	107	-9.17	13242	-0.08	119	-5.58	11960	-0.39	86	-14.07	14129
PTCRA	-1.08	0.000133868	-0.18	100	-9.18	13243	-0.16	118	-11.02	14246	-0.23	84	-7.71	12123
COBLL1	-1.6	3.88E-11	-0.14	117	-9.18	13244	-0.18	123	-13.19	14794	-0.1	130	-7.61	12081
RAX	1.05	0.020269702	-0.12	126	-9.18	13245	-0.06	136	-5.09	11714	-0.01	125	-0.41	8857
PROC	-1.63	1.91E-08	-0.19	98	-9.18	13246	-0.27	114	-17.25	15630	-0.14	104	-7.52	12039
GSTT2	-1.19	0.044118193	-0.11	135	-9.18	13247	-0.07	124	-4.98	11644.5	-0.13	111	-7.65	12094
GMPR	-1.14	0.027468209	-0.18	100	-9.19	13248	-0.16	115	-10.57	14095	-0.15	131	-12.25	13625
ALDOA_t2	-1.28	3.75E-06	-0.18	100	-9.19	13249	-0.17	140	-15.07	15214	-0.17	91	-6.87	11804
GDAP1L1	-1.02	0.22665775	-0.12	127	-9.19	13250	-0.13	135	-10.75	14163	-0.05	111	-3	10178
SBSPON_t1	1.22	0.017015914	-0.11	133	-9.19	13251	0.08	167	9.78	2941	NA	NA	NA	NA
LRRC17	-1.14	0.000608503	-0.11	132	-9.19	13252	0.01	117	0.46	8161	-0.07	122	-5.31	11164
RIPK3	-1.28	1.37E-13	-0.18	100	-9.2	13253	-0.03	97	-1.53	9510	-0.35	99	-16.96	14795
TGFB1I1	-1.12	1.26E-05	-0.15	112	-9.2	13254	-0.09	114	-5.93	12146	-0.2	127	-15.08	14394
GADD45G	-2.52	1.22E-13	-0.17	104	-9.2	13255	-0.29	112	-17.78	15710	-0.21	112	-13.04	13854
PNRC2	-1.08	0.151265939	-0.1	146	-9.21	13256	0.04	121	3.04	6474	-0.04	140	-3.7	10484
GNAS_t3	-1.28	8.81E-06	-0.19	98	-9.21	13257	NA	NA	NA	NA	-0.32	110	-19.46	15243
DST_t2	-1.11	0.002508445	-0.11	133	-9.21	13258	NA	NA	NA	NA	-0.02	136	-1.43	9410

EFHD1	-1.15	0.017424902	-0.1	138	-9.21	13259	-0.14	137	-11.83	14457	-0.04	131	-3.04	10198
SPATA22	-1	0.891939105	-0.12	127	-9.22	13260	-0.13	98	-6.31	12331	-0.04	125	-3.29	10304
TMEM132D	-1.02	0.105111844	-0.13	123	-9.22	13261	-0.05	134	-4.49	11378	0.01	132	0.97	8036
MYCBP2_t1	-1.21	2.50E-05	-0.13	122	-9.22	13262	NA	NA	NA	NA	NA	NA	NA	NA
DIP2A_t2	1.01	0.44575211	-0.11	134	-9.23	13263	-0.07	138	-6	12183	-0.02	144	-1.63	9515
APOA1	-2.64	2.46E-14	-0.32	79	-9.23	13264	-0.38	92	-15.9	15392	-0.33	77	-9.02	12594
C8G	-1.41	0.000141234	-0.18	100	-9.23	13265	-0.21	109	-12.15	14550	-0.06	80	-1.79	9604
CTAGE5_t1	-1.16	0.014609459	-0.14	118	-9.23	13266	-0.11	126	-8.09	13145	-0.05	131	-3.87	10580
EBLN2	-1.17	0.007375323	-0.14	114	-9.23	13267	0.04	153	3.96	5925	-0.15	120	-10.52	13095
GPX6	1.01	0.241380189	-0.11	132	-9.24	13268	0	132	0.03	8442	-0.01	123	-0.98	9189
KIAA0922	-1.06	2.11E-07	-0.14	115	-9.25	13269	-0.19	107	-10.74	14152	-0.08	113	-4.9	11014
CHTF8	-1.15	5.96E-07	-0.12	128	-9.25	13270	-0.24	115	-15.54	15311	-0.22	131	-17.7	14925
VIPR2	-1.03	0.10420338	-0.15	112	-9.25	13271	-0.09	124	-6.71	12516	-0.11	115	-7.25	11936
SIRT1	-1.32	4.45E-08	-0.12	128	-9.25	13272	-0.13	123	-9.37	13647	-0.15	148	-14.77	14318
EAPP	-1.2	7.29E-06	-0.15	110	-9.26	13273	-0.32	112	-19.58	15941	-0.23	98	-10.88	13211
CASS4	-1.11	6.08E-08	-0.16	109	-9.26	13274	-0.19	111	-11.63	14407	-0.1	123	-7.32	11964
TSNARE1	1.01	0.485412742	-0.17	104	-9.26	13275	-0.02	100	-0.81	9023	-0.08	120	-5.58	11266
POLR2J2	-1.09	2.31E-05	-0.17	104	-9.27	13276	-0.11	130	-9.07	13540	-0.15	115	-9.7	12808
MFAP2	-1.02	0.632448636	-0.13	122	-9.27	13277	-0.03	110	-1.51	9499	-0.15	109	-8.69	12489
CD55_t1	-1.23	0.00010172	-0.1	140	-9.27	13278	-0.07	120	-5.15	11743	NA	NA	NA	NA
RRN3_t1	-1.06	0.000430457	-0.11	137	-9.27	13279	NA	NA	NA	NA	-0.02	132	-1.49	9440
MCOLN2	-1.18	0.000613062	-0.12	127	-9.28	13280	0	143	-0.02	8503	-0.27	142	-24.72	15938
CPAMD8	-1.41	3.03E-11	-0.25	87	-9.28	13281	-0.26	123	-19.29	15900	-0.3	90	-12.01	13547
CDHR5_t2	-1.5	0.000118485	-0.15	111	-9.28	13282	-0.24	131	-19.41	15914	-0.17	115	-10.98	13251
PKHD1L1	-1.04	0.054906356	-0.09	149	-9.28	13283	-0.06	132	-5.04	11677	-0.16	121	-11.01	13259
SLC22A3	-1.16	0.104538973	-0.11	136	-9.28	13284	-0.11	119	-7.3	12787	-0.1	132	-8.29	12358
NAALAD2	-1.03	0.101446822	-0.11	132	-9.28	13285	-0.1	112	-6.11	12245	-0.15	130	-12.29	13641
SPACA1	-1.01	0.549837326	-0.13	119	-9.28	13286	-0.12	123	-9.04	13526	0.02	127	1.54	7727
AP3S2_t1	-1.12	0.002285973	-0.09	152	-9.29	13287	NA	NA	NA	NA	NA	NA	NA	NA
HNMT_t1	-1.42	1.26E-06	-0.14	117	-9.29	13288	-0.2	130	-16.21	15460	-0.11	128	-8.45	12415
RAI2	-1.7	6.21E-10	-0.15	114	-9.29	13289	-0.14	112	-8.76	13421	-0.23	142	-20.82	15471
TLR3	-1.48	8.46E-10	-0.17	106	-9.29	13290	-0.19	102	-9.64	13751	-0.18	97	-8.53	12436
NGRN	1.04	0.3862158	-0.11	138	-9.29	13291	-0.04	111	-2.36	10057	0.04	118	2.76	7048
CARD11	-1.08	0.000532236	-0.19	100	-9.29	13292	-0.02	132	-1.57	9532	-0.27	129	-21.19	15534
LCE5A	-1.25	0.00015021	-0.16	108	-9.29	13293	-0.23	120	-16.33	15480	-0.14	106	-7.73	12133
CD97	-2.23	4.63E-19	-0.2	96	-9.29	13294	-0.07	119	-4.78	11524	-0.34	126	-25.76	16008
GLYATL2	1.03	0.328757913	-0.11	132	-9.3	13295	-0.1	112	-6.08	12234	-0.03	113	-1.6	9498
CYGB	-1.13	1.05E-09	-0.15	113	-9.3	13296	-0.17	139	-15.55	15312	-0.16	117	-10.67	13140
CREB3	-1.02	0.557410143	-0.14	116	-9.3	13297	-0.1	114	-6.31	12327	-0.15	121	-10.39	13048
GPHA2	-1.02	0.088966931	-0.1	143	-9.31	13298	-0.11	97	-4.94	11623	-0.09	122	-6.32	11584
CRABP2	1.02	0.272269604	-0.13	120	-9.32	13299	-0.07	122	-4.98	11646	-0.05	123	-3.52	10403
TNRC6C	1.02	0.120229525	-0.1	147	-9.32	13300	0.04	133	3.52	6167	0.02	169	2.97	6941
KIF17	-1.22	1.51E-11	-0.17	104	-9.32	13301	-0.13	131	-10.74	14155	-0.25	120	-17.65	14913
AP2A2_t1	-1.15	1.51E-07	-0.12	125	-9.32	13302	NA	NA	NA	NA	-0.22	128	-17.25	14840
TMED1	-1.23	2.77E-08	-0.17	104	-9.33	13303	-0.22	103	-11.66	14414	-0.23	107	-13.2	13897
BLNK	-1.45	1.03E-08	-0.14	117	-9.33	13304	-0.22	138	-19.17	15887	-0.2	108	-11.34	13358
CCR1	-1.03	0.036776424	-0.14	119	-9.33	13305	-0.06	142	-5.51	11919	-0.09	132	-7.12	11890
CD247	-1.7	5.55E-13	-0.16	107	-9.33	13306	-0.09	130	-6.9	12601	-0.32	125	-23.89	15864
CNR2	-1.04	0.002375841	-0.13	120	-9.34	13307	-0.14	120	-9.45	13679	-0.17	113	-10.41	13060
ANKRD10_t1	-1.17	0.000360218	-0.13	124	-9.34	13308	NA	NA	NA	NA	-0.13	139	-11.46	13388
KLC4_t1	-1.03	0.043049903	-0.12	129	-9.34	13309	-0.1	101	-5.19	11768	-0.12	101	-6.02	11443
DNAJC3	-1.11	0.05020928	-0.1	146	-9.34	13310	-0.03	126	-2.38	10067	-0.06	148	-5.8	11365
NKD2	-1.3	3.64E-06	-0.13	121	-9.34	13311	-0.08	109	-4.57	11425	-0.11	100	-5.36	11187
CMTM2	-1.33	3.65E-09	-0.15	113	-9.34	13312	-0.1	116	-6.79	12558	-0.23	109	-13.62	14008
GMIP	-1.23	4.88E-06	-0.14	115	-9.34	13313	0.08	120	5.57	5029	-0.29	105	-16.06	14603
SURF1	-1.19	9.60E-05	-0.16	107	-9.34	13314	-0.19	92	-7.92	13078	-0.14	94	-6.18	11514
NPDC1	-1.85	1.60E-21	-0.21	95	-9.35	13315	-0.12	111	-7.56	12903	-0.34	100	-17.1	14816
GALNT8	-1.01	0.779859311	-0.18	102	-9.35	13316	-0.12	143	-10.7	14130	-0.21	104	-11.12	13290
HOXA2	-1.04	0.091532106	-0.13	120	-9.35	13317	-0.16	120	-11.09	14268	-0.11	135	-9.02	12592
MEFV	-1.18	1.10E-12	-0.15	111	-9.35	13318	-0.2	132	-16.44	15500	-0.16	146	-15.56	14488
GCHFR	-1.46	8.50E-08	-0.23	90	-9.35	13319	-0.3	124	-22.13	16180	-0.2	105	-10.75	13170
SH2D3C	-1.31	3.19E-14	-0.21	95	-9.35	13320	-0.14	113	-8.65	13372	-0.27	122	-19.45	15239
HPGDS	-1.12	0.04662654	-0.13	122	-9.36	13321	0.01	124	0.39	8219	-0.07	128	-5.21	11122
MRPS2	-1.21	1.69E-08	-0.15	114	-9.36	13322	-0.16	119	-10.78	14171	-0.23	114	-14.87	14344
SPATA2L	-1.65	4.25E-18	-0.16	107	-9.36	13323	-0.23	124	-17.16	15609	-0.26	100	-12.75	13777
RASSF1	-1.08	0.066502029	-0.15	114	-9.36	13324	0.08	127	6.08	4736	-0.17	104	-8.92	12563
ECM2	-1.81	3.08E-09	-0.13	120	-9.36	13325	-0.2	128	-15.8	15363	-0.14	129	-10.72	13156
FAM43B_t1	-1.43	2.29E-08	-0.16	108	-9.37	13326	NA	NA	NA	NA	-0.15	121	-10.59	13114
CNDP2	-1.21	1.54E-09	-0.1	140	-9.37	13327	-0.13	148	-12.3	14585	-0.26	135	-22.37	15708
ACBD4	-1.2	5.45E-05	-0.15	111	-9.38	13328	-0.09	125	-6.69	12507	-0.12	120	-8.65	12474
CTDSP1_t1	-1.1	4.45E-08	-0.14	118	-9.38	13329	-0.2	133	-16.76	15558	-0.2	120	-14.3	14191
DNASE1L2	-1.37	2.55E-19	-0.22	92	-9.38	13330	-0.22	99	-10.74	14157	-0.34	100	-17.23	14836
ALDH1L1	-2.11	8.46E-07	-0.19	100	-9.38	13331	-0.19	103	-10.02	13888	-0.21	101	-10.84	13195
TTYH1	-1.04	0.086926354	-0.17	106	-9.38	13332	-0.05	130	-4.07	11137	-0.16	116	-10.4	13055
PRRT2	-1.14	0.020735511	-0.14	115	-9.38	13333	0.01	118	0.91	7865	-0.15	122	-10.73	13164
TGFB3	-1.08	1.48E-05	-0.15	112	-9.38	13334	-0.06	124	-4.14	11173	-0.2	118	-13.51	13979
MFAP3L_t1	-1.07	0.049711245	-0.1	146	-9.39	13335	-0.09	138	-8.11	13159	-0.11	141	-10.2	12989
TXNIP	-1.72	7.46E-09	-0.13	122	-9.39	13336	-0.04	120	-2.68	10264	-0.26	158	-27.74	16172
AGPAT2	-2.04	1.71E-23	-0.25	87	-9.39	13337	-0.16	132	-13.31	14830	-0.37	110	-22.44	15714
SIT1	-1.14	8.44E-05	-0.17	106	-9.4	13338	-0.06	114	-4.08	11141	-0.25	120	-17.36	14854
RGS2	-2.93	2.20E-12	-0.13	121	-9.4	13339	-0.04	118	-2.89	10404	-0.28	124	-20.61	15430
DEFB1	-3.34	3.80E-09	-0.21	94	-9.41	13340	-0.04	97	-2.07	9870	-0.26	104	-14.1	14137
SRF	1.03	0.091731495	-0.11	132	-9.41	13341	0.02	135	1.92	7206	-0.05	140	-4.35	10775
HLA-DQB1	-1.81	6.46E-10	-0.13	121	-9.41	13342	-0.03	117	-1.89	9744	-0.31	120	-21.85	15635
TOPORS	-1.13	0.004045278	-0.11	137	-9.41	13343	-0.18	124	-13.63	14906	-0.1	122	-7.2	11920
DIRAS1	1.07	0.016910933	-0.12	129	-9.41	13344	0.03	138	2.53	6807	-0.02	128	-1.71	9560
IGHMBP2	-1.13	8.82E-06	-0.13	120	-9.41	13345	-0.26	122	-19.01	15870	-0.2	136	-17.15	14825
USH1C	1.24	0.000216615	-0.15	113	-9.42	13346	0.03	126	2.32	6940	-0.02	113	-1.09	9242
NID2	-1.29	0.000113049	-0.16	110	-9.42	13347	-0.05	119	-3.33	10693	-0.11	133	-9.38	12710
TCF4	-1.42	7.09E-07	-0.11	139	-9.42	13348	0.01	141	0.6	8068	-0.23	180	-30.53	16314
MAP7	-1.08	0.043418141	-0.09	151	-9.42	13349	-0.03	124	-1.9	9748	-0.06	150	-6.28	11564
SYNGR1_t2	-1.02	0.163535113	-0.11	135	-9.42	13350	-0.1	147	-9.24	13601	-0.12	126	-8.77	12515
SEMA4B	-1.14	9.94E-11	-0.17	104	-9.43	13351	-0.24	128	-18.85	15848	-0.14	133	-11.73	13467
ORM1	-1.77	1.38E-06	-0.17	104	-9.43	13352	-0.26	93	-11.03	14249	-0.18	117	-12.16	13592
GPC1	-1.52	3.63E-10	-0.17	107	-9.43	13353	-0.11	137	-9.22	13596	-0.25	126	-19.05	15184
CST9L	-1.03	0.001279448	-0.1	143	-9.43	13354	-0.13	135	-10.91	14210	-0.08	116	-5.01	11053
CACNA1B	1	0.920607509	-0.12	132	-9.44	13355	-0.02	132	-1.33	9379	-0.04	118	-2.41	9897
HMOX2_t1	-1.1	0.003204708	-0.15	115	-9.44	13356	NA	NA	NA	NA	-0.13	115	-8.52	12433
TRPC5	1	0.946479243	-0.11	137	-9.44	13357	-0.05	139	-4.2	11205	0.01	126	0.38	8386
TBRG1	-1.16	3.31E-08	-0.13	125	-9.44	13358	-0.05	133	-4.45	11357	-0.23	139	-20.44	15405
TNFRSF17	-1.84	5.34E-09	-0.17	107	-9.45	13359	-0.04	108	-2.46	10126	-0.34	98	-16.3	14646
BMPR2	-1.24	2.66E-06	-0.09	151	-9.45	13360	0	162	0.18	8347	-0.02	164	-2.39	9885
PDE3A	1.01	0.289274575	-0.11	138	-9.45	13361	0.05	119	3.37	6270	0.01	141	0.74	8170
GTF2H5	-1.15	0.023192735	-0.09	151	-9.45	13362	-0.14	136	-11.8	14445	-0.03	163	-2.85	10116
COPS7A	-1.03	0.207678657	-0.15	115	-9.45	13363	-0.03	130	-2.47	10137	-0.08	118	-5.45	11222
AKR7A2	-1.18	5.13E-06	-0.12	130	-9.46	13364	-0.23	139	-20.15	16008	-0.15	139	-13.6	14000

TNFAIP8L2	-1.6	3.08E-19	-0.18	104	-9.46	13365	-0.1	131	-8.23	13202	-0.36	131	-29.27	16267
DOCK10	-1.5	1.47E-09	-0.12	129	-9.46	13366	-0.04	118	-2.66	10256	-0.22	136	-18.73	15127
HMGB4	-1.04	0.000261486	-0.25	88	-9.46	13367	-0.14	84	-4.71	11495	-0.06	72	-1.4	9389
HERC5	-1.42	3.41E-09	-0.16	111	-9.47	13368	-0.16	123	-11.9	14479	-0.19	97	-8.88	12554
CREBL2	-1.13	0.00043458	-0.08	164	-9.47	13369	-0.03	128	-2.69	10267	-0.07	132	-5.61	11278
PTGDS	-3.16	1.05E-15	-0.25	88	-9.47	13370	-0.08	113	-4.81	11544	-0.35	100	-17.5	14883
CCIN	-1.36	1.04E-22	-0.31	81	-9.47	13371	-0.32	92	-13.29	14819	-0.24	104	-13.15	13886
GBP4	-1.55	1.43E-08	-0.12	131	-9.47	13372	-0.11	157	-11.83	14458	-0.29	133	-24.1	15879
MIA2_t1	-1.28	4.35E-09	-0.19	99	-9.48	13373	-0.19	110	-11.19	14293	NA	NA	NA	NA
NUMBL	-1.14	1.49E-05	-0.14	120	-9.48	13374	-0.17	171	-20.77	16071	-0.24	152	-24.88	15948
BANK1	-1.23	0.000361368	-0.1	142	-9.48	13375	-0.01	121	-0.38	8732	-0.17	143	-16.01	14589
ADAMTS13_t1	-1.05	0.009661227	-0.12	129	-9.49	13376	-0.1	103	-5.04	11679	-0.23	98	-10.88	13207
HS3ST3B1	-1.03	0.079459095	-0.14	120	-9.49	13377	-0.13	140	-11.49	14371	-0.08	106	-4.69	10935
TAF15	-1.14	0.00051428	-0.14	118	-9.49	13378	-0.11	110	-6.55	12438	-0.15	107	-8.61	12461
ZNF488	-1.06	0.000472624	-0.15	112	-9.5	13379	-0.11	141	-9.61	13739	-0.13	143	-11.77	13479
VMO1	-2.82	2.48E-35	-0.25	88	-9.5	13380	-0.26	94	-11.27	14319	-0.41	88	-15.46	14470
MFAP3L_t2	1.01	0.617576831	-0.1	146	-9.5	13381	-0.07	138	-6.39	12367	-0.02	141	-1.82	9619
SFXN2	-1.28	1.04E-07	-0.15	113	-9.51	13382	-0.27	123	-19.36	15911	-0.19	119	-12.84	13801
CCR7	-2.3	3.35E-13	-0.19	100	-9.51	13383	-0.09	141	-8.04	13128	-0.32	120	-22.35	15704
NEURL	-1.02	0.623497086	-0.14	120	-9.52	13384	-0.01	116	-0.87	9059	-0.18	126	-13.62	14007
KIR2DL2	-1.04	0.024547131	-0.09	159	-9.52	13385	-0.1	145	-9.45	13678	-0.17	119	-11.76	13474
ARHGEF1	-1.12	1.84E-05	-0.14	116	-9.53	13386	-0.09	129	-7.27	12769	-0.29	108	-16.72	14738
DNAJB9	-1.25	0.005444951	-0.13	124	-9.53	13387	-0.13	130	-10.27	13991	0	127	-0.3	8791
SEL1L3	-1.63	8.67E-06	-0.13	125	-9.53	13388	0	132	-0.1	8556	-0.25	142	-23.08	15785
PVRL1_t2	-1.02	0.549236864	-0.13	125	-9.53	13389	0.09	142	8.18	3655	-0.14	135	-12.09	13573
TESK2	-1.24	4.20E-09	-0.14	118	-9.54	13390	-0.2	127	-15.13	15231	-0.23	141	-21.25	15547
SH2B1	1.01	0.570271915	-0.14	119	-9.54	13391	-0.03	120	-2.03	9843	-0.09	110	-5.21	11124
CHN2	-1	0.926439054	-0.1	146	-9.55	13392	-0.07	131	-5.59	11966	0.03	120	1.89	7514
PCIF1	-1.05	0.000863507	-0.16	111	-9.55	13393	-0.16	118	-10.78	14172	-0.16	102	-8.09	12264
ITGBL1	-1.65	1.49E-06	-0.14	118	-9.55	13394	-0.03	131	-2.11	9910	-0.24	100	-11.83	13497
ZNF167_t1	-1.12	0.026982183	-0.13	123	-9.56	13395	0.1	131	8.12	3685	-0.07	116	-4.61	10897
BTD	-1.36	2.57E-07	-0.2	97	-9.56	13396	-0.33	117	-22.16	16184	-0.16	154	-16.84	14769
KEAP1	-1.03	0.407533602	-0.14	120	-9.56	13397	-0.13	116	-8.48	13319	-0.17	113	-10.89	13214
PIGV	-1.37	1.29E-09	-0.17	106	-9.56	13398	-0.23	124	-17.17	15611	-0.16	106	-9.12	12637
RPS15A_t1	-1.21	1.16E-05	-0.22	94	-9.56	13399	NA	NA	NA	NA	-0.22	88	-8.39	12390
SH3RF1_t1	-1.1	2.34E-05	-0.12	132	-9.57	13400	NA	NA	NA	NA	NA	NA	NA	NA
HABP2	-1.56	1.93E-11	-0.15	113	-9.58	13401	-0.23	146	-21.88	16158	-0.13	127	-9.66	12794
RND2	-1.1	0.020258007	-0.13	123	-9.58	13402	-0.18	122	-13.21	14800	-0.17	134	-14.66	14290
ART1	-1.57	1.42E-10	-0.23	92	-9.58	13403	-0.3	99	-14.73	15153	-0.22	88	-8.33	12369
HAPLN4	-1.02	0.301554792	-0.15	115	-9.58	13404	-0.09	141	-8.47	13311	-0.11	120	-7.75	12141
MRPL34	-1.12	0.00813063	-0.17	107	-9.58	13405	-0.12	99	-5.78	12081	-0.17	123	-12.5	13697
NR2F6	-1	0.936090672	-0.13	122	-9.58	13406	-0.05	124	-3.35	10707	0.03	88	1.04	7999
TTC39B	-1.35	1.68E-15	-0.11	137	-9.58	13407	-0.18	143	-17.01	15590	-0.2	141	-17.83	14948
INSR	-1.07	0.130104097	-0.12	131	-9.59	13408	-0.11	120	-7.56	12902	-0.04	135	-3.07	10212
VEGFA_t2	-1.06	0.052993502	-0.1	148	-9.59	13409	NA	NA	NA	NA	NA	NA	NA	NA
TET2	-1.1	3.65E-08	-0.12	129	-9.59	13410	-0.03	139	-2.94	10442	-0.17	139	-15.03	14380
LITAF	-1.24	1.01E-05	-0.11	135	-9.59	13411	0.04	124	2.62	6738	-0.18	133	-15.03	14384
MMRN2_t1	-1.13	9.43E-05	-0.12	131	-9.6	13412	NA	NA	NA	NA	-0.14	120	-9.67	12800
TTTY23	-1.23	3.47E-13	-0.17	108	-9.6	13413	-0.27	137	-23.6	16284	-0.21	120	-14.7	14295
SHBG	-3.57	1.52E-24	-0.28	84	-9.61	13414	-0.3	84	-10.37	14027	-0.31	75	-7.84	12181
IGSF22	-1.09	0.001313096	-0.16	109	-9.61	13415	-0.01	113	-0.37	8727	-0.29	108	-16.67	14730
PPAPDC3	-1.03	0.582026017	-0.11	137	-9.62	13416	0	131	-0.05	8520	-0.17	128	-13.1	13867
SLC30A1	-1.24	0.00015023	-0.12	129	-9.62	13417	-0.2	130	-16.04	15431	-0.09	110	-5.43	11213
UBE2D3	-1.24	4.22E-06	-0.11	134	-9.62	13418	-0.1	126	-7.65	12957	-0.15	142	-13.36	13939
DAGLB	-1.01	0.799536708	-0.17	106	-9.62	13419	-0.05	115	-3.08	10518	-0.11	115	-7.25	11940
CLSTN2	-1.16	8.75E-05	-0.16	112	-9.62	13420	-0.12	131	-9.61	13742	-0.26	123	-19.1	15191
RASL11A	-1.22	6.52E-08	-0.19	101	-9.63	13421	-0.11	112	-7	12650	-0.25	110	-14.96	14369
FRMD4A_t1	-1.27	2.56E-05	-0.14	120	-9.64	13422	NA	NA	NA	NA	-0.14	129	-11.24	13323
GRB14	-1.72	2.50E-11	-0.18	104	-9.64	13423	-0.08	114	-4.85	11566	-0.23	120	-16.16	14620
SHF	-1.35	5.85E-05	-0.18	104	-9.64	13424	-0.2	110	-11.97	14493	-0.12	100	-6.22	11533
SMAP2_t1	-1.19	1.44E-12	-0.12	128	-9.64	13425	NA	NA	NA	NA	-0.22	126	-16.93	14790
SGMS2	-1.29	1.29E-05	-0.1	151	-9.64	13426	-0.14	128	-11.26	14314	-0.07	156	-7.26	11945
CXCR2	-1.05	0.001327711	-0.12	129	-9.65	13427	-0.12	146	-11.78	14442	-0.08	123	-6.18	11515
PHLDB1	-1.25	1.11E-06	-0.15	114	-9.65	13428	-0.01	114	-0.43	8773	-0.25	121	-18.09	14992
MBD4	-1.23	1.79E-06	-0.11	134	-9.65	13429	-0.09	131	-7.22	12743	-0.1	153	-9.83	12868
SYT2	-1.07	0.002189967	-0.13	127	-9.65	13430	-0.16	136	-13.43	14855	-0.11	142	-10.05	12943
TSHZ3	-1.45	8.29E-08	-0.13	124	-9.65	13431	-0.01	146	-0.82	9030	-0.24	147	-23.48	15826
CHDH	-1.22	0.000243838	-0.13	127	-9.66	13432	-0.13	136	-10.86	14196	-0.05	122	-3.53	10406
GLT1D1	-1.89	1.29E-09	-0.16	111	-9.66	13433	-0.25	134	-20.99	16084	-0.13	123	-9.61	12781
SLC46A3	-1.4	1.06E-06	-0.11	138	-9.66	13434	-0.19	144	-17.57	15678	-0.15	131	-11.81	13488
LYN	-1.2	0.000391862	-0.11	142	-9.67	13435	0.07	133	6.18	4676	-0.18	143	-16.91	14788
CDKL2	-1.01	0.567549137	-0.12	131	-9.67	13436	0.22	143	20.53	333	-0.16	141	-14.65	14288
EGFR	-1.55	3.38E-10	-0.11	137	-9.67	13437	-0.29	127	-22.35	16203	-0.09	138	-7.72	12129
DUSP16	-1.08	0.00017125	-0.13	123	-9.67	13438	-0.24	128	-18.85	15849	-0.15	143	-13.73	14033
UCN2	-1.25	7.06E-06	-0.15	114	-9.67	13439	-0.07	126	-5.37	11855	-0.18	108	-10.59	13113
SIRT5_t1	-1.01	0.782535481	-0.13	123	-9.67	13440	-0.07	105	-4.09	11146	-0.03	125	-2.03	9720
TMEM139	-1.15	0.084828163	-0.15	116	-9.68	13441	-0.03	135	-2.83	10358	-0.16	110	-9.64	12789
PRPF8	-1.13	0.000299053	-0.16	111	-9.68	13442	-0.22	112	-13.67	14911	-0.25	98	-11.88	13508
CASR	-1.03	0.300931352	-0.16	112	-9.69	13443	-0.04	130	-3.53	10822	-0.24	136	-20.42	15402
RALYL	-1.02	0.5154617	-0.1	145	-9.69	13444	-0.08	117	-5.66	12006	-0.15	125	-11.2	13307
ENO2	-1.16	0.004381336	-0.12	133	-9.69	13445	-0.01	128	-0.56	8854	-0.24	123	-17.64	14910
ITLN1	-6.36	3.35E-34	-0.32	80	-9.69	13446	-0.3	88	-11.24	14313	-0.45	77	-12.23	13619
RCN1_t1	-1.26	6.02E-05	-0.1	145	-9.7	13447	NA	NA	NA	NA	NA	NA	NA	NA
ST8SIA4_t2	-1.15	1.16E-08	-0.1	144	-9.7	13448	0	130	-0.32	8695	-0.28	136	-24	15870
TPBG	-1.2	0.000378725	-0.14	118	-9.7	13449	-0.05	119	-3.65	10907	-0.26	129	-20.28	15379
LRRC71	-1.02	0.199555946	-0.17	106	-9.71	13450	-0.04	116	-2.73	10294	-0.1	82	-3.2	10270
UGT3A1_t1	-1.2	1.05E-06	-0.12	132	-9.71	13451	NA	NA	NA	NA	NA	NA	NA	NA
GPR18	-1.25	3.91E-08	-0.18	105	-9.71	13452	-0.05	106	-2.75	10312	-0.29	96	-13.13	13880
GCK	1.14	0.001112246	-0.15	114	-9.71	13453	0	131	0.07	8422	-0.03	104	-1.39	9386
CLEC4C	-1.07	2.71E-10	-0.18	105	-9.71	13454	-0.18	130	-14.1	15011	-0.21	120	-14.85	14336
PTP4A1	-1.39	4.05E-06	-0.09	158	-9.72	13455	-0.17	131	-13.37	14844	0.01	158	1.59	7695
UGT2B4	-1.47	0.003995826	-0.13	127	-9.72	13456	-0.15	118	-10.26	13984	-0.11	121	-7.48	12023
ISOC2	-1.35	2.98E-08	-0.19	100	-9.73	13457	-0.26	121	-18.64	15814	-0.17	104	-8.93	12569
AGBL4	-1.02	0.046136579	-0.14	122	-9.73	13458	-0.06	114	-4.1	11151	-0.07	114	-4.63	10905
BCKDHA	-1.31	6.91E-11	-0.18	105	-9.73	13459	-0.24	111	-14.47	15098	-0.23	122	-16.85	14771
DHRS7B	-1.22	2.34E-06	-0.12	130	-9.74	13460	-0.11	121	-7.77	13014	-0.13	131	-10.35	13035
JAK3_t1	-1.17	7.46E-07	-0.13	123	-9.74	13461	-0.11	122	-7.7	12986	NA	NA	NA	NA
ELMO3	-1.38	4.98E-10	-0.16	112	-9.74	13462	-0.22	88	-8.25	13213	-0.32	91	-13.2	13900
DDC	-1.39	0.010138384	-0.14	120	-9.74	13463	-0.06	100	-3.24	10627	-0.02	115	-1.61	9505
PTGER4_t1	-1.78	1.03E-09	-0.12	131	-9.74	13464	NA	NA	NA	NA	-0.27	129	-21.02	15505
CX3CL1_t1	-1.35	0.000682804	-0.12	129	-9.75	13465	-0.08	141	-7.36	12822	NA	NA	NA	NA
WIBG	1.04	0.124337919	-0.11	136	-9.75	13466	0	104	0.01	8463	-0.1	116	-6.71	11740
SUCNR1	-1.14	6.99E-07	-0.16	112	-9.76	13467	-0.12	119	-8.54	13340	-0.24	126	-17.87	14956
NBPF11_t1	-1.18	0.00043281	-0.1	148	-9.76	13468	NA	NA	NA	NA	-0.12	122	-8.72	12494
PGM2	-1.07	0.421125091	-0.1	149	-9.76	13469	-0.07	142	-6.82	12572	0	135	-0.25	8764
NRXN1_t1	-1.03	0.021743798	-0.09	158	-9.76	13470	-0.04	138	-3.42	10739	NA	NA	NA	NA

PRUNE2_t1	-1.71	3.96E-14	-0.11	137	-9.76	13471	-0.13	134	-10.7	14134	NA	NA	NA	NA
TTC25	-1.11	0.008515613	-0.18	105	-9.77	13472	-0.02	104	-0.9	9077	-0.21	118	-13.99	14107
ARHGAP31	-1.18	1.12E-10	-0.1	149	-9.77	13473	-0.03	144	-3.26	10643	-0.27	121	-19.45	15241
ZNF622	1.04	0.225288818	-0.15	117	-9.77	13474	-0.07	96	-3.31	10682	-0.01	119	-0.53	8939
DCLK2	-1.04	0.093395315	-0.16	110	-9.77	13475	-0.02	140	-1.85	9725	-0.14	142	-13.04	13852
PATL1	-1.07	0.004469454	-0.09	159	-9.77	13476	0.04	119	2.58	6776	-0.12	135	-10.41	13061
FGFR2_t1	-1.19	0.00017125	-0.13	124	-9.78	13477	-0.08	112	-5.16	11751	-0.16	135	-13.87	14069
KLHL14	-1.06	0.000162296	-0.13	127	-9.78	13478	-0.08	131	-6.69	12510	-0.23	118	-15.68	14515
BAIAP2_t1	-1.11	0.034868879	-0.1	151	-9.78	13479	-0.04	139	-3.34	10697	-0.16	110	-9.88	12887
MAGI1	-1.15	1.29E-09	-0.1	148	-9.78	13480	-0.08	134	-7.12	12707	-0.12	150	-11.59	13425
FSTL3	-1.41	2.37E-07	-0.14	122	-9.78	13481	-0.06	148	-5.65	12004	-0.24	133	-19.6	15263
DMD_t1	-1.36	0.000656174	-0.1	145	-9.79	13482	NA	NA	NA	NA	NA	NA	NA	NA
ODZ1	1.02	0.415981842	-0.12	132	-9.8	13483	-0.05	130	-3.66	10909	-0.01	155	-0.73	9046
MCF2L	-1.03	0.028241219	-0.12	129	-9.8	13484	-0.14	132	-11.43	14352	-0.07	128	-5.63	11290
GJB4	-1.03	0.04049006	-0.16	110	-9.8	13485	-0.15	136	-13.25	14813	-0.09	111	-5.2	11118
HTR1F	-1.01	0.471256556	-0.11	137	-9.81	13486	0	146	-0.07	8534	-0.12	148	-12.19	13606
PEX10_t2	-1.11	0.000128992	-0.14	121	-9.81	13487	-0.12	130	-9.49	13698	-0.19	123	-13.52	13980
CTDSP1_t2	-1.05	0.175284349	-0.14	118	-9.82	13488	-0.07	133	-6.01	12190	-0.16	120	-11.54	13415
AREG	-2.47	1.87E-11	-0.2	100	-9.82	13489	-0.06	101	-2.88	10394	-0.28	118	-18.81	15140
NME8	-1.06	0.00405458	-0.17	107	-9.82	13490	-0.02	117	-1.38	9418	-0.18	93	-7.74	12137
GCLM	-1.07	0.274245615	-0.12	135	-9.82	13491	-0.03	143	-2.53	10167	0	150	0.4	8370
CWH43	-1.1	2.95E-05	-0.18	106	-9.83	13492	-0.09	108	-5.39	11862	-0.28	96	-12.78	13784
PKLR	-1.1	0.289809715	-0.14	121	-9.83	13493	-0.12	157	-12.68	14683	-0.05	140	-4.07	10669
CDC16	-1.15	0.000194587	-0.26	88	-9.83	13494	-0.01	103	-0.36	8723	-0.19	104	-10.37	13042
QPRT_t1	-1.24	0.001707157	-0.13	123	-9.83	13495	-0.09	128	-6.78	12551	NA	NA	NA	NA
APOBEC3G	-1.42	2.03E-08	-0.13	123	-9.83	13496	-0.08	127	-5.88	12128	-0.33	125	-24.59	15923
UGT3A2	1.11	0.001075785	-0.11	140	-9.84	13497	0.02	120	1.4	7552	-0.03	111	-2.02	9715
ADAMTSL3_t1	-1.91	6.68E-13	-0.17	109	-9.84	13498	NA	NA	NA	NA	NA	NA	NA	NA
PRKCZ	-1.35	0.000240703	-0.18	106	-9.84	13499	-0.18	108	-10.48	14057	-0.18	120	-12.47	13690
CEACAM6	-1.01	0.902296841	-0.1	149	-9.84	13500	-0.04	154	-4.38	11307	-0.06	139	-5.36	11188
TNFRSF14	-1.34	1.25E-08	-0.15	115	-9.85	13501	-0.13	114	-8.48	13317	-0.32	114	-20.54	15420
COL8A2	-1.66	6.71E-07	-0.14	120	-9.85	13502	-0.03	125	-2.46	10123	-0.27	147	-25.73	16003
PRDX6	-1.19	0.003198109	-0.12	130	-9.85	13503	-0.11	123	-8.3	13241	-0.1	136	-8.75	12512
NBPF14	-1.19	0.000435592	-0.18	106	-9.85	13504	-0.12	115	-7.8	13033	-0.11	122	-7.61	12076
PPP3CA_t1	-1.25	2.24E-09	-0.12	134	-9.85	13505	NA	NA	NA	NA	-0.14	138	-12.74	13771
RHOG	-1.18	2.31E-08	-0.16	112	-9.86	13506	-0.08	111	-4.65	11461	-0.32	127	-24.37	15904
TCAIM_t2	-1.2	0.006660837	-0.13	128	-9.87	13507	-0.13	119	-8.85	13452	0	127	0.05	8586
CBLN3	-1.15	5.02E-09	-0.14	122	-9.87	13508	-0.24	130	-19.42	15919	-0.09	134	-7.61	12079
RFPL3	-1.05	0.00445556	-0.12	134	-9.87	13509	-0.05	115	-3.46	10770	-0.03	107	-1.53	9459
NCF1_t1	-1.63	3.34E-16	-0.15	114	-9.87	13510	NA	NA	NA	NA	NA	NA	NA	NA
OSCAR_t2	-1.17	1.34E-05	-0.19	103	-9.88	13511	-0.04	115	-2.46	10125	-0.3	122	-21.4	15569
RASAL1	-1.19	2.93E-07	-0.1	145	-9.88	13512	-0.13	127	-10.36	14025	-0.1	148	-10.11	12960
SNCA	-1.25	0.0001036	-0.13	125	-9.88	13513.5	-0.02	136	-2	9828	-0.23	127	-17.91	14967
ADIPOR2	-1.19	0.001314561	-0.13	125	-9.88	13513.5	-0.14	154	-15.02	15203	-0.12	143	-10.82	13186
IQSEC2_t1	-1.21	2.44E-10	-0.13	127	-9.88	13515	NA	NA	NA	NA	NA	NA	NA	NA
DDX5	-1.21	0.003826444	-0.11	137	-9.88	13516	-0.04	132	-3.22	10613	-0.15	134	-12.32	13652
GIT2_t2	-1.11	0.000318587	-0.15	117	-9.88	13517	0	108	0	8484	-0.18	123	-13.32	13926
RSPH10B	-1.14	8.67E-14	-0.18	106	-9.89	13518	-0.18	115	-11.89	14475	-0.14	110	-8.57	12442
FAM162A	-1.25	0.000999598	-0.14	120	-9.89	13519	-0.12	107	-7.09	12688	-0.03	93	-1.48	9439
UNC5B	-1.25	0.014029443	-0.11	137	-9.89	13520	0.03	135	2.82	6601	-0.14	125	-10.6	13115
MRPL54	-1.41	9.65E-14	-0.27	87	-9.89	13521	-0.3	91	-12.5	14638	-0.34	88	-12.9	13819
OSBPL11	-1.22	0.000309054	-0.1	146	-9.9	13522	-0.02	119	-1.35	9403	-0.12	150	-11.84	13500
SOCS6	-1.29	1.89E-10	-0.11	142	-9.9	13523	-0.11	124	-7.85	13053	-0.14	144	-13.02	13848
NOXO1	-1.25	1.52E-08	-0.19	101	-9.9	13524	-0.2	80	-6.11	12250	-0.17	100	-8.39	12391
OTOF	-1.05	0.002221133	-0.13	127	-9.91	13525	-0.11	124	-7.85	13055	-0.19	126	-14.33	14199
MAMDC2	-1.09	0.001532738	-0.12	131	-9.91	13526	-0.12	118	-8.36	13267	-0.12	120	-8.16	12294
MFSD6L	-1.1	0.001694165	-0.15	116	-9.91	13527	-0.01	89	-0.5	8816	-0.25	114	-16.05	14602
TRAF3IP3	-1.83	4.68E-13	-0.22	96	-9.91	13528	-0.05	105	-2.66	10255	-0.34	97	-15.93	14571
DPEP1	-1	0.91225303	-0.15	114	-9.91	13529	-0.04	109	-2.18	9948	-0.19	82	-5.93	11417
FIBIN	-1.07	0.005102779	-0.13	129	-9.91	13530	-0.14	120	-9.48	13692	-0.1	141	-9.5	12758
HBEGF_t1	-1.28	3.52E-11	-0.14	121	-9.92	13531	-0.11	134	-9.07	13545	NA	NA	NA	NA
HSD17B4	-1.24	0.012303072	-0.12	131	-9.93	13532	-0.15	119	-10.46	14048	-0.03	112	-2.05	9734
LCP1	-1.67	5.35E-09	-0.11	139	-9.93	13533	-0.03	142	-2.75	10308	-0.27	123	-19.43	15237
TEPP	-1.12	6.10E-05	-0.16	111	-9.94	13534	-0.18	116	-11.82	14455	-0.16	130	-12.51	13700
IFNAR2	-1.11	9.10E-06	-0.17	107	-9.94	13535	-0.01	146	-0.7	8940	-0.2	134	-16.74	14742
IFNAR1	-1.31	1.43E-09	-0.11	143	-9.94	13536	-0.05	135	-4.15	11175	-0.19	129	-15.2	14418
CLDND1	-1.16	2.42E-05	-0.1	152	-9.94	13537	-0.05	134	-3.8	10978	-0.16	138	-14.47	14228
CPZ	-1.54	8.57E-09	-0.2	100	-9.95	13538	-0.07	98	-3.51	10802	-0.32	151	-32.73	16383
ARHGAP29	-1.37	4.27E-08	-0.1	148	-9.95	13539	-0.11	133	-9.17	13579	-0.06	138	-5.11	11083
SNX9	-1.16	6.10E-08	-0.13	126	-9.95	13540	-0.11	143	-10.17	13941	-0.14	155	-14.86	14339
KRT34	1	0.9932113	-0.15	115	-9.95	13541	-0.13	122	-9.02	13508	0	115	-0.1	8677
APOL3	-1.2	0.049567775	-0.13	124	-9.95	13542	-0.12	104	-6.56	12442	-0.12	104	-6.32	11580
ANXA1	-1.93	1.67E-09	-0.16	114	-9.96	13543	-0.01	124	-1.09	9214	-0.25	111	-15.47	14474
IL13	-1.07	0.174777271	-0.14	119	-9.96	13544	0.01	140	1.06	7762	-0.24	121	-16.73	14739
MAML2	-1.06	2.11E-05	-0.13	125	-9.96	13545	-0.08	136	-6.77	12545	-0.1	120	-6.65	11715
TRIM21	-1.07	0.001624671	-0.16	112	-9.96	13546	-0.09	135	-7.39	12829	-0.23	110	-14.07	14128
PROSC	-1.3	1.57E-09	-0.13	127	-9.96	13547	-0.19	132	-15.38	15285	-0.15	129	-12.08	13570
INHBC	-1.14	4.41E-09	-0.14	120	-9.97	13548	-0.18	130	-14.44	15090	-0.16	132	-12.76	13778
CD79B	-1.2	1.65E-13	-0.17	110	-9.97	13549	-0.18	120	-12.73	14700	-0.26	88	-9.93	12909
SLC20A2	-1.26	0.002240096	-0.13	124	-9.97	13550	-0.21	127	-16.26	15468	-0.07	122	-5.07	11074
LIMS2	-1.38	9.65E-11	-0.16	114	-9.97	13551	-0.29	119	-19.75	15956	-0.22	127	-16.99	14800
LDOC1	-1	0.908269062	-0.14	120	-9.97	13552	0.07	143	6.65	4422	-0.15	131	-11.79	13484
OGN	-1.19	0.013459083	-0.11	139	-9.99	13553	-0.04	130	-3.05	10501	-0.17	123	-12.31	13648
NAPSA	-1.67	8.38E-21	-0.26	89	-9.99	13554	-0.19	86	-6.77	12549	-0.41	94	-18.09	14993
PLA2G12B	-1.33	0.001718996	-0.15	118	-9.99	13555	-0.17	112	-10.66	14118	-0.06	114	-3.79	10530
LAT2_t1	-1.45	7.65E-08	-0.16	113	-10	13556	-0.02	131	-1.37	9412	NA	NA	NA	NA
HDLBP	-1.12	0.001830183	-0.09	162	-10	13557	-0.07	128	-5.74	12054	-0.19	124	-13.75	14039
SCAP	-1.08	0.083596356	-0.19	104	-10	13558	-0.11	91	-4.6	11441	-0.09	120	-6.14	11493
STK38L	-1.07	0.032715385	-0.12	136	-10	13559	0	142	0.1	8401	-0.03	144	-2.43	9908
SLC35A5	-1.13	6.72E-06	-0.1	147	-10.01	13560	-0.2	128	-15.48	15302	-0.08	127	-6.5	11642
SRP54	-1.13	0.00943081	-0.16	114	-10.02	13561	-0.1	130	-8.12	13168	-0.15	140	-13.75	14036
SSPN	-1.47	1.38E-06	-0.1	154	-10.04	13562	0	136	0.16	8360	-0.26	126	-19.45	15240
SERPINB8_t1	-1.7	1.76E-09	-0.14	120	-10.04	13563	-0.17	149	-17.28	15637	-0.18	136	-15.19	14417
GBP7	-2.77	1.80E-11	-0.23	94	-10.04	13564	-0.28	97	-13.19	14796	-0.21	120	-14.52	14248
OTOR	-1.01	0.43297484	-0.12	133	-10.04	13565	-0.2	103	-10.73	14149	0.08	123	5.7	5579
SLC1A2	-2.17	3.73E-06	-0.11	141	-10.05	13566	-0.23	158	-25.03	16347	-0.08	155	-8.16	12293
RILP_t1	-1.72	3.30E-15	-0.22	96	-10.05	13567	NA	NA	NA	NA	NA	NA	NA	NA
ACACB	-1.36	5.37E-07	-0.11	142	-10.05	13568	-0.2	120	-14.02	14997	-0.17	124	-12.61	13732
PDE6G	-1.06	3.08E-06	-0.19	103	-10.05	13569	0	120	0.07	8416	-0.25	113	-15.76	14532
HLF	-2.18	1.61E-08	-0.14	124	-10.07	13570	-0.17	146	-15.9	15391	-0.12	143	-11.57	13421
APBA2_t1	-1.08	0.0013317	-0.14	120	-10.07	13571	0	123	-0.01	8494	NA	NA	NA	NA
MS4A12	-1.23	3.06E-11	-0.14	122	-10.07	13572	-0.21	106	-11.7	14424	-0.07	102	-3.69	10476
SLC16A13	-1.1	3.77E-05	-0.16	114	-10.07	13573	-0.19	115	-12.2	14561	-0.11	88	-4.08	10672
NFX1_t1	-1.14	3.66E-06	-0.13	127	-10.07	13574	-0.24	126	-18.38	15787	-0.17	132	-13.98	14104
JHDM1D	-1.57	6.13E-14	-0.12	136	-10.07	13575	-0.1	145	-9.18	13581	-0.18	142	-16.56	14703
LMF2	-1.05	0.002311046	-0.16	112	-10.07	13576	-0.18	111	-11.04	14254	-0.12	110	-7.1	11881

SHMT2	-1.23	8.49E-05	-0.17	108	-10.09	13577	-0.25	126	-18.72	15825	-0.19	121	-13.81	14054
S100A4	-1.67	4.58E-09	-0.14	124	-10.1	13578	0	108	0	8475	-0.3	112	-18.77	15132
PIK3CD_t1	-1.09	2.08E-07	-0.13	126	-10.1	13579	NA	NA	NA	NA	NA	NA	NA	NA
HNRNPF	-1.11	0.000212129	-0.14	123	-10.1	13580	-0.05	127	-3.52	10811	-0.18	129	-14.49	14237
CUX1_t2	-1.05	0.113772044	-0.16	115	-10.1	13581	-0.13	114	-8.17	13181	-0.11	148	-11.27	13334
LPAR5	-1.18	3.32E-09	-0.13	129	-10.1	13582	-0.03	135	-2.72	10288	-0.31	137	-27	16130
NAT1	-1.59	1.11E-13	-0.14	120	-10.1	13583	-0.2	129	-15.77	15357	-0.21	126	-16.21	14627
SGCD_t2	-1.06	0.163803782	-0.13	130	-10.1	13584	-0.01	145	-1.27	9339	-0.24	134	-19.82	15300
FAM210B	-1.14	0.015441905	-0.11	140	-10.11	13585	-0.12	139	-10.73	14144	-0.08	127	-6.12	11486
BMP10	-1.04	0.000122049	-0.14	122	-10.11	13586	-0.16	86	-5.6	11975	-0.1	88	-3.62	10446
WDFY2	-1.29	2.80E-12	-0.13	128	-10.11	13587	-0.09	136	-7.8	13031	-0.3	127	-22.73	15748
MBOAT2_t1	-1.13	0.016358382	-0.12	136	-10.11	13588	-0.03	133	-2.89	10408	NA	NA	NA	NA
WDR81	-1.25	4.21E-08	-0.17	111	-10.11	13589	-0.19	119	-13.25	14812	-0.21	129	-16.38	14662
XPNPEP2	-1.1	0.01567827	-0.19	104	-10.11	13590	-0.14	123	-10.52	14068	-0.09	133	-7.31	11961
TMEM47	-1.71	3.00E-13	-0.13	130	-10.11	13591	-0.2	140	-18.16	15756	-0.1	130	-7.65	12095
TGFBI	-1.19	0.017505719	-0.16	115	-10.12	13592	-0.06	135	-5.12	11729	0.03	135	2.72	7075
CLCF1	-1.14	0.001977336	-0.15	117	-10.12	13593	0	113	-0.09	8551	-0.16	121	-11.61	13430
LPO	-1.18	1.86E-09	-0.17	108	-10.12	13594	-0.25	131	-20.32	16022	-0.11	123	-7.68	12105
LAMB2	-1.35	2.04E-08	-0.3	84	-10.12	13595	-0.21	105	-11.55	14386	-0.21	120	-14.74	14306
MARCH5_t1	-1.08	0.019462722	-0.12	136	-10.13	13596	NA	NA	NA	NA	0.03	140	2.45	7204
PTAFR	-1.6	6.84E-13	-0.14	120	-10.14	13597	-0.05	127	-3.69	10928	-0.36	156	-38.23	16469
ENPP7	-1.06	0.107392009	-0.17	108	-10.14	13598	-0.16	104	-8.68	13387	-0.04	88	-1.45	9418
BRSK1	-1.02	0.016360753	-0.19	104	-10.14	13599	-0.04	101	-2.17	9943	-0.11	118	-7.3	11956
BLCAP	-1.07	0.130467404	-0.13	130	-10.14	13600	-0.1	150	-10.26	13988	-0.09	147	-9.11	12627
SDS_t1	-2.49	6.67E-16	-0.18	107	-10.14	13601	NA	NA	NA	NA	NA	NA	NA	NA
ISLR	-1.91	1.87E-13	-0.18	106	-10.15	13602	-0.09	119	-6.2	12285	-0.31	103	-16.56	14704
DEFA3	-5.25	8.61E-22	-0.24	93	-10.15	13603	-0.2	106	-11.15	14283	-0.27	100	-13.58	13993
ATP6V1G2	1.03	0.229459262	-0.14	121	-10.15	13604	-0.11	120	-7.63	12941	-0.09	119	-6.37	11599
C1QTNF5	-1.27	3.61E-06	-0.16	115	-10.16	13605	-0.05	130	-3.66	10916	-0.22	109	-12.98	13838
ZNF579	-1.28	7.51E-06	-0.18	107	-10.17	13606	-0.2	120	-13.74	14934	-0.2	115	-12.94	13828
DENND2A	-1.44	2.91E-14	-0.24	93	-10.17	13607	-0.12	104	-6.37	12360	-0.33	95	-15.03	14382
ABCA13	1	0.998077181	-0.14	122	-10.17	13608	-0.09	133	-7.6	12930	-0.14	124	-10.37	13043
MSRB1	-1.32	3.64E-05	-0.15	118	-10.17	13609	-0.11	151	-11.07	14262	-0.19	107	-11.06	13276
ACSF3	-1.23	2.42E-09	-0.14	121	-10.17	13610	-0.17	122	-12.24	14570	-0.16	135	-14	14112
AADAC	-1.88	7.17E-07	-0.14	125	-10.17	13611	-0.18	118	-12.11	14538	-0.17	133	-14.06	14126
IPCEF1	-1.08	1.60E-08	-0.11	142	-10.17	13612	-0.02	134	-2.05	9858	-0.19	160	-20.75	15453
RORB	-1.01	0.312302636	-0.13	127	-10.17	13613	0.01	122	0.37	8230	-0.13	127	-9.78	12839
PPP1R15A	-1.61	1.34E-20	-0.25	91	-10.18	13614	-0.18	71	-3.8	10977	-0.32	112	-19.54	15256
LDHAL6B	-1.02	0.126695366	-0.13	128	-10.18	13615	-0.13	106	-7.08	12683	0.01	104	0.34	8403
R3HDM2_t1	-1.13	1.70E-05	-0.13	127	-10.18	13616	NA	NA	NA	NA	NA	NA	NA	NA
RXFP1	-1.12	1.37E-12	-0.15	120	-10.18	13617	-0.15	127	-11.19	14294	-0.2	151	-20.01	15330
PDE1A_t1	-1.31	1.07E-07	-0.15	116	-10.18	13618	NA	NA	NA	NA	-0.22	115	-14.01	14115
RNF128_t1	-1.37	2.90E-05	-0.12	132	-10.18	13619	NA	NA	NA	NA	NA	NA	NA	NA
CYP27A1	-1.9	4.38E-12	-0.19	103	-10.19	13620	-0.24	132	-19.99	15984	-0.29	122	-20.67	15440
ENGASE	-1.02	0.580534968	-0.18	107	-10.2	13621	-0.04	124	-3.16	10579	-0.15	104	-8.23	12324
SCNN1A	-1.11	0.000514916	-0.12	136	-10.2	13622	-0.22	130	-17.27	15634	-0.21	132	-17.55	14895
SDF4	-1.16	7.63E-06	-0.14	123	-10.21	13623	-0.16	111	-9.92	13851	-0.21	108	-12.02	13551
CD53	-1.82	4.50E-13	-0.16	115	-10.21	13624	-0.03	121	-1.99	9814	-0.34	116	-22.5	15720
RPUSD4	-1.11	0.00143465	-0.18	107	-10.22	13625	-0.01	122	-0.82	9029	-0.18	120	-12.5	13698
FAM8A1	-1.08	0.299838369	-0.1	153	-10.22	13626	-0.04	136	-3.27	10651	0.01	148	1.12	7955
USH2A_t2	1.01	0.307670423	-0.11	144	-10.22	13627	0.01	135	0.61	8060	-0.03	132	-2.82	10096
CALHM2	-1.36	7.01E-07	-0.12	132	-10.23	13628	0.01	112	0.35	8235	-0.27	141	-24.38	15905
TMEM176B	-1.5	1.41E-06	-0.2	100	-10.23	13629	-0.33	120	-22.95	16247	-0.22	133	-17.89	14959
HSD17B12	-1.25	1.13E-05	-0.13	126	-10.23	13630	0	119	0.17	8352	-0.13	131	-10.26	13011
RBFOX1_t2	-1.01	0.463933153	-0.13	130	-10.24	13631	-0.09	137	-7.66	12963	-0.08	128	-6.3	11569
IL20RA	-1.07	0.099564644	-0.13	127	-10.24	13632	-0.1	137	-8.89	13465	-0.19	134	-16.03	14594
RIC3	-1.08	0.000813659	-0.13	131	-10.24	13633	-0.12	145	-11.51	14375	-0.14	149	-13.49	13973
PCSK6_t1	-1.97	7.72E-12	-0.3	84	-10.24	13634	-0.27	80	-8.23	13204	-0.2	82	-6.31	11575
TMEM63B	-1.09	0.000143162	-0.19	104	-10.25	13635	-0.1	120	-7.07	12680	-0.08	140	-7.19	11918
LTF	-1.2	0.000439363	-0.23	95	-10.25	13636	-0.16	111	-9.7	13771	-0.28	94	-12.43	13675
ULK2	-1.36	4.89E-07	-0.09	159	-10.26	13637	0	130	-0.02	8499	-0.16	163	-18.55	15090
BATF2	-1.28	1.67E-05	-0.13	132	-10.26	13638	-0.06	149	-5.67	12015	-0.32	120	-22.06	15667
PGM5_t1	-1.11	7.73E-09	-0.16	115	-10.26	13639	NA	NA	NA	NA	-0.2	134	-16.65	14724
HSD17B14	1.01	0.898761994	-0.14	126	-10.26	13640	-0.15	113	-9.31	13625	-0.02	106	-1.2	9295
LPCAT3	-1.36	1.79E-09	-0.16	114	-10.27	13641	-0.2	133	-16.29	15472	-0.17	123	-12.07	13567
LGALS12	-1.06	0.012066523	-0.15	119	-10.27	13642	-0.16	125	-12.27	14575	-0.17	120	-12	13537
PDLIM4	-1.18	1.12E-05	-0.12	139	-10.27	13643	-0.11	124	-8.4	13288	-0.18	135	-15.1	14397
BMP1_t2	-1.15	5.05E-08	-0.19	104	-10.27	13644	-0.18	108	-10.56	14089	-0.18	116	-12.02	13552
RPP25	-1.41	1.51E-07	-0.14	126	-10.27	13645	-0.03	127	-2.28	10010	-0.24	120	-16.59	14714
NFIC	-1.03	0.36281525	-0.1	154	-10.27	13646	-0.13	140	-12.06	14526	-0.06	140	-5.84	11383
PLAGL1_t1	-1.48	7.87E-10	-0.15	119	-10.28	13647	0.03	114	1.72	7336	NA	NA	NA	NA
WARS	-1.23	3.10E-06	-0.16	114	-10.28	13648	-0.13	121	-9.08	13548	-0.32	129	-25.32	15977
HEMK1_t1	-1.12	0.000911406	-0.14	122	-10.29	13649	NA	NA	NA	NA	NA	NA	NA	NA
TGFA	-1.17	1.43E-06	-0.12	137	-10.29	13650	0.01	136	1.16	7703	-0.18	154	-19.1	15192
EVC2	-1.09	0.002381949	-0.16	114	-10.29	13651	0.02	141	1.7	7348	-0.26	134	-22.22	15693
L1TD1	-1.04	0.039695979	-0.13	129	-10.3	13652	0.01	148	0.54	8107	-0.18	120	-12.8	13791
TEX30	-1.24	0.000358451	-0.17	111	-10.3	13653	-0.16	109	-9.7	13775	-0.1	112	-5.92	11413
SYN1_t2	-1.36	4.12E-07	-0.14	125	-10.3	13654	-0.19	129	-15.29	15272	-0.16	122	-11.5	13401
SELENBP1	-1.24	0.011816581	-0.15	119	-10.32	13655	-0.16	147	-16	15417	-0.05	98	-2.33	9858
MAFK_t1	-1.04	0.004228363	-0.11	143	-10.32	13656	NA	NA	NA	NA	-0.07	136	-6.3	11572
RCN3	-1.3	4.33E-14	-0.3	84	-10.32	13657	-0.11	130	-8.73	13407	-0.33	88	-12.43	13680
TMEM52	-1.04	0.105065622	-0.17	110	-10.32	13658	-0.17	106	-9.76	13801	-0.08	114	-5.21	11126
TMED10	-1.14	0.003736342	-0.11	140	-10.32	13659	-0.18	124	-13.14	14780	-0.1	146	-9.53	12770
CCDC65	-1.02	0.110873633	-0.18	109	-10.33	13660	0.07	100	3.67	6071	-0.09	108	-5.31	11163
NLRP1_t2	-1.23	8.82E-10	-0.16	113	-10.33	13661	-0.07	104	-3.57	10857	-0.24	121	-16.89	14780
PRIMA1	-1.05	0.010623812	-0.12	135	-10.33	13662	-0.16	156	-17.45	15665	-0.11	132	-9.35	12694
PODN	-1.25	1.12E-11	-0.22	97	-10.33	13663	-0.1	134	-8.08	13141	-0.29	100	-14.48	14231
BTAF1	-1.06	0.074213086	-0.12	134	-10.34	13664	-0.07	139	-6.46	12394	0.03	150	2.54	7164
PNPLA4	-1.2	4.19E-06	-0.12	138	-10.34	13665	-0.21	140	-19.07	15877	-0.08	142	-6.93	11817
RAC2	-1.09	0.004806224	-0.15	120	-10.35	13666	-0.05	123	-3.45	10766	-0.21	129	-16.47	14678
STX1B	-1.02	0.059547483	-0.12	136	-10.35	13667	-0.09	132	-7.06	12676	-0.12	123	-8.42	12409
CR1_t1	-1.09	1.62E-09	-0.11	142	-10.36	13668	NA	NA	NA	NA	NA	NA	NA	NA
GALT_t1	-1.21	0.000117647	-0.2	101	-10.36	13669	NA	NA	NA	NA	-0.12	104	-6.38	11604
FOXD3	-1.14	2.22E-05	-0.16	115	-10.37	13670	-0.21	118	-13.95	14983	-0.18	109	-10.4	13053
PJA2	-1.25	0.000146961	-0.12	140	-10.37	13671	-0.16	128	-12.72	14699	-0.06	146	-5.98	11435
PTGDR	-1.08	0.00037782	-0.11	143	-10.37	13672	-0.04	116	-2.63	10234	-0.18	115	-11.52	13408
SUMO1	-1.08	0.025179222	-0.14	122	-10.38	13673	-0.02	136	-1.96	9788	-0.07	137	-6.05	11457
TPST1	-1.54	5.88E-08	-0.15	120	-10.38	13674	-0.14	115	-9.26	13609	-0.23	130	-18.72	15123
SEPT6_t2	-1.02	0.363590521	-0.11	142	-10.38	13675	-0.06	135	-5.25	11800	-0.02	139	-1.39	9385
RCOR2_t1	-1.26	3.79E-06	-0.14	124	-10.39	13676	NA	NA	NA	NA	NA	NA	NA	NA
NEU4	-1.82	1.21E-07	-0.19	105	-10.4	13677	-0.21	109	-12.35	14601	-0.16	100	-8.24	12334
PARP6	-1.32	4.70E-05	-0.17	113	-10.4	13678	-0.05	118	-3.23	10617	-0.18	88	-7	11850
VWCE	-1.61	0.000161937	-0.21	100	-10.4	13679	-0.11	110	-6.64	12486	-0.14	124	-10.45	13076
GJC3	1	0.878285473	-0.16	116	-10.4	13680	-0.14	108	-8.34	13259	0	120	0.3	8436
DDX26B	-1.26	0.000287722	-0.12	140	-10.4	13681	0.04	115	2.37	6907	-0.13	128	-9.98	12920
LILRA5_t1	-1.2	9.31E-15	-0.16	115	-10.4	13682	-0.18	100	-9.18	13583	-0.36	112	-22.17	15683

KIAA0284	-1.23	7.81E-07	-0.17	113	-10.41	13683	-0.09	131	-7.49	12872	-0.26	115	-16.88	14776
TTC7B	-1.09	2.30E-05	-0.13	129	-10.41	13684	-0.03	128	-2.41	10090	-0.11	120	-7.4	11995
LRFN4	-1.14	0.002176409	-0.12	137	-10.41	13685	-0.07	107	-3.97	11087	-0.2	104	-11.06	13275
PAN2	-1.05	0.292017716	-0.15	120	-10.41	13686	-0.17	115	-10.92	14211	-0.06	125	-4.22	10724
ADHFE1	-1.44	3.22E-05	-0.19	104	-10.41	13687	-0.18	111	-11.12	14276	-0.15	132	-12	13539
CD3G	-1.67	2.13E-09	-0.19	104	-10.41	13688	-0.04	113	-2.42	10102	-0.33	114	-21.41	15572
EIF5A	-1.24	8.99E-12	-0.13	130	-10.42	13689	-0.24	118	-16.24	15465	-0.26	103	-13.94	14092
PIGQ	-1.15	4.63E-05	-0.16	116	-10.42	13690	-0.14	134	-11.57	14393	-0.15	114	-9.92	12901
TNFAIP1	-1.18	4.08E-05	-0.14	122	-10.42	13691	-0.18	130	-14.44	15088	-0.21	141	-18.87	15150
GGT1_t1	-1.59	5.32E-16	-0.33	82	-10.43	13692	NA	NA	NA	NA	-0.36	80	-10.89	13213
FAM26F	-2.78	1.54E-17	-0.19	106	-10.43	13693	-0.14	84	-4.92	11610	-0.34	112	-21.11	15523
LRRC34	-1.06	0.002039408	-0.19	106	-10.43	13694	0.03	123	2.11	7075	-0.17	107	-9.94	12910
COTL1	-1.44	1.88E-17	-0.18	107	-10.44	13695	-0.06	120	-4.37	11298	-0.39	120	-26.98	16129
GET4_t1	-1.04	0.398036939	-0.14	126	-10.44	13696	NA	NA	NA	NA	-0.07	119	-4.75	10961
RPP14	-1.09	0.006739556	-0.13	131	-10.44	13697	-0.14	123	-10.57	14094	-0.12	133	-9.59	12778
ARTN	-1.17	2.83E-05	-0.14	124	-10.44	13698	-0.17	112	-10.77	14168	-0.12	119	-8.07	12260
PRKCH	-1.46	1.92E-10	-0.15	118	-10.44	13699	-0.08	134	-7.09	12691	-0.28	127	-21.49	15589
LIPE	-1.06	0.270882355	-0.16	117	-10.44	13700	-0.03	113	-1.78	9675	-0.08	103	-4.26	10741
CD9	-1.33	0.000154114	-0.14	125	-10.45	13701	-0.1	108	-5.7	12030	-0.14	116	-9.35	12697
WFDC9	-1.03	0.010288325	-0.14	124	-10.45	13702	-0.11	98	-5.35	11846	-0.04	88	-1.62	9510
HAAO	-1.76	1.31E-19	-0.25	92	-10.46	13703	-0.33	130	-26.21	16388	-0.25	104	-13.4	13953
ISYNA1	-1.37	3.06E-10	-0.16	115	-10.47	13704	-0.07	104	-3.97	11084	-0.28	115	-18.48	15076
NDUFAB1	-1.08	0.073852814	-0.18	109	-10.47	13705	-0.02	118	-1.68	9603	-0.05	115	-3.14	10243
UCK1	-1.23	7.96E-07	-0.16	115	-10.47	13706	-0.14	127	-11.06	14260	-0.16	110	-9.62	12785
ZAP70	-1.63	1.69E-11	-0.18	108	-10.48	13707	-0.08	103	-4.18	11196	-0.32	128	-24.71	15937
LRMP	-1.54	1.15E-11	-0.17	110	-10.48	13708	-0.11	118	-7.17	12722	-0.26	99	-12.61	13735
FGD2	-1.1	1.12E-06	-0.13	130	-10.48	13709	-0.15	127	-11.45	14360	-0.26	104	-13.79	14048
AOC2	-1.01	0.515100862	-0.16	114	-10.48	13710	-0.08	114	-5.34	11838	-0.02	119	-1.39	9387
POMC	-1.28	5.32E-18	-0.31	84	-10.48	13711	-0.12	124	-8.76	13423	-0.38	116	-24.9	15952
CASP6	-1.14	0.000620467	-0.13	133	-10.5	13712	-0.08	140	-6.9	12604	-0.11	152	-11.44	13381
SPINT2	-2.49	7.67E-12	-0.17	111	-10.5	13713	-0.07	100	-3.5	10797	-0.29	110	-17.67	14917
KIF12	-1.34	2.09E-12	-0.16	117	-10.51	13714	-0.1	130	-7.84	13045	-0.25	92	-10.5	13087
ERCC5	-1.44	1.57E-08	-0.28	88	-10.51	13715	-0.18	97	-8.34	13258	-0.26	101	-13.37	13942
GUSB	-1.22	8.11E-06	-0.28	88	-10.51	13716	-0.28	100	-13.78	14943	-0.19	90	-7.47	12021
ONECUT1	-1.08	0.377973968	-0.12	139	-10.53	13717	-0.1	129	-8.02	13124	0.01	114	0.94	8050
GNAO1_t1	-1.09	0.000665688	-0.12	141	-10.54	13718	-0.2	136	-17.05	15598	-0.14	147	-13.21	13902
LIME1	-1.41	2.09E-10	-0.28	87	-10.54	13719	-0.3	120	-21	16088	-0.2	88	-7.68	12103
GNRHR_t2	-1.02	0.301403527	-0.13	129	-10.54	13720	-0.12	133	-9.68	13765	-0.07	124	-5.21	11119
MRPL46_t1	-1.34	2.50E-08	-0.25	92	-10.55	13721	NA	NA	NA	NA	NA	NA	NA	NA
MZB1	-2.18	7.09E-14	-0.21	101	-10.55	13722	-0.1	120	-7.26	12765	-0.4	110	-24.29	15902
CST3	-1.35	2.23E-10	-0.21	100	-10.56	13723	-0.18	122	-12.98	14747	-0.28	100	-14.07	14130
ADORA2A_t1	-1.17	2.54E-10	-0.15	122	-10.56	13724	NA	NA	NA	NA	-0.23	128	-17.79	14941
GSPT1	-1.12	0.000324267	-0.11	146	-10.57	13725	-0.02	131	-1.56	9525	-0.1	143	-9.65	12793
LAPTM5_t1	-1.56	2.32E-11	-0.14	124	-10.57	13726	-0.05	120	-3.3	10674	NA	NA	NA	NA
MICU1	-1.27	2.83E-07	-0.16	118	-10.57	13727	-0.25	133	-20.4	16029	-0.21	122	-14.96	14370
UBA7	-1.22	3.16E-06	-0.19	106	-10.57	13728	-0.23	115	-15.14	15233	-0.26	104	-13.98	14105
AOAH	-1.64	1.13E-18	-0.2	104	-10.57	13729	-0.11	105	-6.07	12228	-0.38	118	-25.79	16012
MXRA7_t1	1.01	0.883251543	-0.12	137	-10.57	13730	0.1	137	8.99	3276	-0.09	126	-7.1	11883
LYPD6	1.06	0.113963874	-0.13	133	-10.57	13731	-0.05	155	-5.6	11976	-0.18	134	-15.29	14435
LCAT	-3.11	6.97E-33	-0.38	78	-10.58	13732	-0.37	92	-15.69	15339	-0.39	88	-14.75	14312
TCEAL6	-1.44	3.00E-06	-0.15	119	-10.58	13733	-0.07	128	-5.77	12071	-0.28	116	-18.2	15018
IQGAP3	-1.11	5.68E-05	-0.18	110	-10.59	13734	-0.22	144	-20.99	16085	-0.2	127	-15.17	14413
TAS2R20	-1.02	0.112555406	-0.13	132	-10.59	13735	-0.06	111	-3.82	10991	-0.13	114	-8.22	12321
RXRA_t1	-1.27	1.67E-07	-0.12	138	-10.59	13736	NA	NA	NA	NA	NA	NA	NA	NA
CES5A	-1.08	0.003553519	-0.18	108	-10.59	13737	-0.14	82	-4.55	11411	-0.16	104	-8.53	12435
CCL19	-4.67	2.30E-22	-0.27	89	-10.6	13738	-0.17	92	-7.26	12763	-0.4	115	-26.32	16073
MBNL2	-1.59	8.92E-07	-0.12	136	-10.6	13739	-0.06	134	-5.09	11717	-0.18	152	-18.35	15056
FCRL5	-1.04	0.218677362	-0.14	126	-10.61	13740	-0.06	162	-6.22	12295	-0.18	120	-12.73	13770
SEC24B	-1.64	5.62E-13	-0.15	122	-10.62	13741	-0.14	121	-10.28	13996	-0.16	125	-11.86	13504
CLPS	-1.06	0.172916641	-0.18	110	-10.62	13742	-0.3	120	-20.68	16062	-0.15	143	-13.55	13985
LRRC32	-1.71	1.79E-10	-0.16	118	-10.62	13743	-0.02	149	-2.2	9963	-0.26	134	-22.17	15682
SESN1	-1.26	4.62E-09	-0.11	149	-10.62	13744	-0.09	126	-6.68	12504	-0.14	138	-12.71	13763
MLN_t2	-1.5	2.74E-11	-0.28	88	-10.62	13745	-0.32	103	-17.07	15601	-0.25	89	-9.59	12779
ARHGEF4_t1	-1.02	0.012401526	-0.16	115	-10.62	13746	-0.06	130	-4.73	11503	-0.08	142	-7.43	12004
SLC51A_t1	-2.14	1.37E-07	-0.2	104	-10.63	13747	-0.3	108	-17.3	15643	NA	NA	NA	NA
MLXIPL_t1	-1.31	0.001471806	-0.16	115	-10.63	13748	NA	NA	NA	NA	NA	NA	NA	NA
HHEX	-1.23	0.000231033	-0.15	120	-10.63	13749	-0.14	119	-9.67	13758	-0.17	141	-15.84	14545
SERHL2	-1.22	0.000346965	-0.2	104	-10.63	13750	-0.21	107	-12.22	14563	-0.18	108	-10.24	13003
PCCB	-1.52	9.70E-12	-0.2	102	-10.64	13751	-0.27	109	-15.69	15341	-0.26	113	-16.51	14684
GJD3	-1.12	9.95E-05	-0.15	123	-10.64	13752	-0.22	129	-17.37	15653	-0.13	125	-9.42	12726
FABP1	-2.33	4.08E-07	-0.19	105	-10.64	13753	-0.26	99	-12.63	14675	-0.2	104	-10.78	13174
BAAT	-1.72	5.20E-06	-0.15	120	-10.65	13754	-0.18	150	-18.41	15793	-0.15	139	-13.11	13877
HSPB7	-1.05	0.086989144	-0.18	110	-10.65	13755	0	132	0.37	8228	-0.21	109	-12.37	13660
CMPK1	-1.31	9.47E-06	-0.13	135	-10.66	13756	-0.12	148	-12.09	14534	-0.13	155	-13.66	14013
GOLIM4	-1.28	4.69E-07	-0.17	112	-10.66	13757	-0.08	109	-4.81	11543	-0.12	130	-9.45	12742
SCO1	-1.28	1.00E-08	-0.13	133	-10.66	13758	-0.17	127	-13.38	14845	-0.11	129	-8.85	12542
HECW2	-1.05	0.001968231	-0.14	124	-10.66	13759	-0.09	170	-11.01	14242	-0.04	145	-4.21	10719
ABTB2	-1.16	0.009968454	-0.15	120	-10.66	13760	-0.18	128	-14.38	15075	-0.07	123	-5.27	11146
PACSIN3	-1.46	3.32E-09	-0.15	123	-10.67	13761	-0.07	129	-5.6	11974	-0.33	113	-20.77	15458
CDC42EP5	-1.32	1.29E-05	-0.24	94	-10.68	13762	-0.26	104	-13.87	14961	-0.22	80	-6.51	11652
PHRF1	-1.06	0.10917054	-0.14	128	-10.68	13763	-0.07	119	-4.77	11521	-0.13	104	-7.06	11865
CHST9	-1.52	4.42E-08	-0.14	128	-10.68	13764	-0.05	108	-2.94	10435	-0.13	127	-10.22	12993
SLC31A1	-1.24	2.10E-09	-0.14	129	-10.69	13765	-0.18	140	-15.98	15412	-0.18	150	-17.59	14903
CD36	-1.58	0.000155048	-0.13	132	-10.69	13766	-0.12	105	-6.83	12577	-0.09	118	-6.24	11544
PEMT	-2.13	1.70E-18	-0.31	84	-10.69	13767	-0.3	131	-24.45	16322	-0.26	108	-14.93	14364
NLGN4X	-1.03	0.351155897	-0.11	144	-10.7	13768	-0.06	133	-5.35	11848	-0.06	138	-5.09	11078
NCKAP5	-1.01	0.335574314	-0.11	148	-10.7	13769	0.04	132	3.22	6361	0.03	158	2.95	6959
TLR2	-1.96	1.44E-15	-0.14	129	-10.7	13770	-0.04	136	-3.21	10610	-0.33	138	-28.89	16255
KCNQ5	-1.14	2.30E-08	-0.12	141	-10.7	13771	-0.26	136	-22.37	16205	-0.09	131	-7.42	11998
MTERFD3	-1.06	0.210601426	-0.18	110	-10.7	13772	-0.04	105	-2.43	10109	0.01	119	0.83	8122
FOLR2	-1.74	8.80E-15	-0.2	104	-10.7	13773	-0.18	108	-10.22	13964	-0.31	99	-15.15	14408
TMEM18_t1	-1.19	8.85E-06	-0.13	130	-10.7	13774	NA	NA	NA	NA	NA	NA	NA	NA
CLDN5_t1	-1.47	3.65E-10	-0.16	116	-10.7	13775	NA	NA	NA	NA	-0.23	115	-15.03	14381
HENMT1	-1.33	0.000742565	-0.14	124	-10.7	13776	0.04	103	1.91	7211	-0.21	137	-18.25	15034
GZMA	-2.1	1.54E-11	-0.21	100	-10.7	13777	-0.08	79	-2.28	10011	-0.34	111	-20.79	15467
PDE3B	-1.28	1.34E-05	-0.11	145	-10.71	13778	-0.13	138	-11.1	14271	-0.04	151	-4.38	10786
RGL2	-1.2	6.99E-07	-0.18	110	-10.71	13779	-0.02	113	-1.04	9172	-0.19	90	-7.48	12026
REEP1	-1.17	0.000355004	-0.13	134	-10.71	13780	-0.17	156	-18.4	15792	-0.18	149	-18.06	14987
ANKH_t1	-1.06	0.184300073	-0.12	136	-10.72	13781	NA	NA	NA	NA	NA	NA	NA	NA
C5AR1_t1	-1.46	3.11E-14	-0.13	131	-10.73	13782	-0.09	126	-6.67	12500	NA	NA	NA	NA
VAV1	-1.82	5.88E-18	-0.21	102	-10.73	13783	-0.11	117	-7.07	12678	-0.37	93	-16.09	14606
SLC25A4	-1.28	4.83E-06	-0.14	129	-10.73	13784	-0.28	130	-22.35	16201	-0.2	123	-14.41	14217
FRAS1	-1.32	0.020747847	-0.13	133	-10.73	13785	0.04	131	3.46	6209	-0.26	132	-21.1	15521
GPR77	-1.02	0.249971136	-0.2	105	-10.73	13786	-0.09	101	-4.8	11541	-0.08	120	-5.63	11285
REV3L	-1.19	0.000534509	-0.13	135	-10.73	13787	-0.06	121	-4.29	11262	-0.11	119	-7.45	12013
DMRTC1	-1.09	0.000179272	-0.16	116	-10.74	13788	-0.09	118	-6.14	12260	-0.2	120	-13.68	14019

TSR2	-1.21	1.76E-09	-0.17	115	-10.74	13789	-0.24	115	-15.63	15332	-0.23	123	-17.03	14805
PEX16	-1.09	0.006218047	-0.21	100	-10.75	13790	-0.16	123	-11.43	14351	-0.13	113	-8.33	12375
PTPN22_t1	-1.13	4.79E-07	-0.13	134	-10.75	13791	-0.02	120	-1.27	9345	-0.22	142	-20.45	15406
RLBP1	-1.02	0.050009245	-0.13	132	-10.75	13792	-0.12	114	-7.96	13099	-0.1	123	-7.27	11950
TRAPPC8	-1.2	0.000579665	-0.12	136	-10.75	13793	-0.1	127	-7.57	12914	-0.05	137	-4.67	10920
WNT16	-1.01	0.183627177	-0.12	136	-10.75	13794	0	120	0.33	8256	-0.07	119	-4.73	10951
TMBIM4	-1.01	0.88714586	-0.16	119	-10.75	13795	-0.11	120	-7.5	12873	-0.11	150	-10.65	13131
HMBOX1	-1.35	8.93E-08	-0.14	127	-10.75	13796	-0.05	150	-5.29	11815	-0.15	132	-12.45	13686
MAN1A1_t1	-1.29	1.21E-06	-0.12	139	-10.76	13797	NA	NA	NA	NA	-0.09	130	-7.47	12019
ORAI3	-1.21	2.37E-05	-0.19	108	-10.76	13798	-0.15	129	-12.02	14503	-0.19	120	-13.06	13857
FCGR3B	-2.06	5.10E-23	-0.19	107	-10.77	13799	-0.21	122	-15.22	15253	-0.35	145	-33.64	16408
REPS2	-1.23	0.000124435	-0.11	146	-10.77	13800	-0.2	130	-16.12	15445	-0.08	162	-8.92	12564
OSBP	-1.14	5.79E-07	-0.13	133	-10.77	13801	-0.08	131	-6.61	12470	-0.11	127	-8.73	12501
RAI14	-1.03	0.529559132	-0.15	124	-10.77	13802	-0.12	122	-8.36	13268	0.14	130	10.89	3552
LRRC47_t1	-1.11	1.14E-05	-0.13	132	-10.79	13803	NA	NA	NA	NA	NA	NA	NA	NA
CYP7B1	-1.25	0.000925023	-0.15	124	-10.79	13804	-0.19	119	-13.19	14793	-0.11	127	-8.75	12510
ARHGEF4_t2	-1.18	0.002440807	-0.16	117	-10.79	13805	0.01	130	0.83	7913	-0.15	142	-13.49	13974
SORBS3	-1.53	6.98E-15	-0.19	106	-10.79	13806	-0.27	120	-18.67	15820	-0.2	111	-12.2	13607
PLA2G4A	-1.32	1.25E-05	-0.17	113	-10.79	13807	-0.06	118	-3.98	11096	-0.3	118	-20.66	15436
AKR1C4	-1.88	4.94E-08	-0.17	113	-10.8	13808	-0.22	94	-9.79	13812	-0.18	124	-13.41	13956
NOTUM	1.24	0.019199422	-0.15	124	-10.8	13809	-0.06	96	-2.91	10421	-0.13	100	-6.3	11571
CALML4	-1.17	0.02827284	-0.14	128	-10.8	13810	-0.2	139	-18.13	15752	0.04	120	2.74	7062
LRG1	-2.07	4.85E-12	-0.2	104	-10.8	13811	-0.26	117	-17.51	15673	-0.25	121	-17.66	14915
SREBF1	-1.23	0.014927224	-0.15	120	-10.8	13812	0.02	111	1.13	7730	-0.1	113	-6.48	11630
AVPR1A	-1.96	1.45E-09	-0.13	134	-10.8	13813	-0.26	122	-18.43	15794	-0.21	133	-17.81	14946
UAP1	-1.41	2.49E-06	-0.14	127	-10.81	13814	-0.24	118	-16.19	15458	-0.09	129	-7.16	11906
RFX2	-1.24	8.13E-07	-0.15	120	-10.81	13815	-0.13	122	-9.5	13704	-0.2	124	-15.15	14409
FOXA3_t1	-1.34	4.71E-07	-0.11	148	-10.81	13816	NA	NA	NA	NA	NA	NA	NA	NA
VCL_t1	-1.18	4.52E-07	-0.16	119	-10.81	13817	NA	NA	NA	NA	NA	NA	NA	NA
TBATA	-1.04	0.015668455	-0.15	121	-10.81	13818	-0.15	106	-8.18	13184	-0.1	139	-8.61	12464
HSD11B1L_t2	-1.12	7.72E-06	-0.22	100	-10.82	13819	-0.21	106	-11.86	14467	-0.21	119	-14.28	14184
ZNF521	-1.27	1.23E-05	-0.13	135	-10.82	13820	-0.08	115	-5.14	11740	-0.16	135	-13.63	14010
ARHGDIB	-2.07	2.55E-17	-0.22	99	-10.83	13821	-0.05	134	-4.26	11250	-0.38	110	-22.7	15745
COL6A2_t2	-1.82	1.07E-12	-0.19	108	-10.83	13822	-0.08	118	-5.53	11932	-0.29	100	-14.55	14255
PLSCR1	-1.62	8.86E-10	-0.13	131	-10.83	13823	-0.08	142	-7.72	12999	-0.23	127	-17.7	14923
MYH11_t1	-1.61	1.10E-06	-0.14	129	-10.83	13824	-0.01	144	-0.58	8867	-0.26	156	-27.04	16132
COL4A3BP	-1.24	4.54E-06	-0.12	144	-10.83	13825	-0.04	142	-3.55	10835	-0.08	139	-7.3	11957
YY1	-1.06	0.037485713	-0.17	115	-10.84	13826	-0.18	130	-14.55	15115	-0.12	139	-10.67	13143
SLC25A22	-1.1	0.047870932	-0.15	122	-10.84	13827	-0.16	139	-13.88	14964	-0.09	110	-5.2	11117
ZBTB20	-1.39	4.27E-08	-0.14	129	-10.84	13828	-0.17	119	-11.81	14447	-0.19	143	-17.41	14865
SPTBN2	-1.2	1.55E-08	-0.22	100	-10.84	13829	-0.27	122	-19.62	15945	-0.16	100	-8.17	12299
HPN	-1.79	2.37E-11	-0.26	92	-10.84	13830	-0.28	127	-21.3	16124	-0.27	119	-18.97	15168
CEACAM4	-1.37	1.11E-20	-0.26	91	-10.85	13831	-0.25	92	-10.55	14085	-0.39	117	-26.25	16062
KIAA0895L	-1.12	0.001597588	-0.14	128	-10.85	13832	-0.02	140	-1.71	9628	-0.21	135	-17.68	14921
CLRN3	-6.09	9.12E-20	-0.25	93	-10.85	13833	-0.32	126	-24.53	16324	-0.27	135	-22.84	15762
OAT	-1.91	2.37E-05	-0.12	141	-10.86	13834	-0.09	139	-8.29	13233	-0.12	123	-8.67	12482
PI4K2B	-1.26	0.002536299	-0.11	150	-10.86	13835	-0.16	129	-12.83	14718	0.01	144	1.2	7917
NUDT6	-1.62	1.85E-09	-0.22	99	-10.87	13836	-0.26	101	-13.12	14776	-0.18	105	-9.96	12915
BIN2	-1.49	2.74E-18	-0.18	111	-10.87	13837	-0.04	126	-3.38	10723	-0.36	103	-19.32	15222
WFDC5	1.01	0.371281358	-0.15	122	-10.87	13838	-0.07	130	-5.65	12003	-0.05	129	-4.14	10693
GRM8	-1.73	6.85E-11	-0.19	108	-10.87	13839	-0.26	120	-17.87	15717	-0.37	120	-25.55	15992
SHOC2	-1.19	8.91E-05	-0.12	137	-10.87	13840	-0.08	127	-6.46	12399	-0.02	144	-2.1	9755
TCEAL3	-1.5	7.91E-07	-0.16	119	-10.87	13841	-0.08	104	-4.1	11150	-0.28	113	-17.58	14901
CLEC2B	-2.04	1.99E-17	-0.19	108	-10.88	13842	-0.08	150	-7.92	13083	-0.34	124	-25.06	15958
TNFRSF1B	-1.35	8.26E-17	-0.16	120	-10.88	13843	-0.15	147	-15.03	15205	-0.29	144	-27.2	16143
TBC1D10B	-1.18	5.82E-08	-0.17	115	-10.88	13844	-0.09	158	-10	13884	-0.19	120	-13.11	13874
MB21D2_t1	-1.16	0.000479936	-0.12	140	-10.89	13845	NA	NA	NA	NA	NA	NA	NA	NA
TIE1	-1.38	4.69E-13	-0.21	102	-10.89	13846	-0.14	122	-10.25	13979	-0.27	100	-13.67	14017
PDZRN4	-1.04	0.004361466	-0.13	132	-10.89	13847	-0.13	121	-9.09	13555	-0.13	126	-10.14	12971
SLC7A2	-1.26	3.71E-10	-0.12	138	-10.9	13848	-0.28	133	-22.85	16243	-0.13	132	-10.37	13040
CFHR4	-4.22	4.91E-19	-0.19	108	-10.9	13849	-0.3	113	-18.65	15815	-0.2	130	-15.95	14576
NFATC1_t1	-1.31	6.83E-10	-0.17	113	-10.9	13850	-0.01	132	-0.93	9096	-0.29	134	-24.18	15888
ELL2	-1.72	4.85E-10	-0.13	136	-10.9	13851	-0.18	137	-15.63	15331	-0.15	140	-13.94	14093
ACOX1	-1.13	1.67E-06	-0.12	139	-10.91	13852	-0.18	141	-16.33	15481	-0.13	141	-11.89	13510
SLA	-2.13	3.92E-14	-0.16	120	-10.91	13853	-0.04	151	-4.47	11371	-0.34	147	-33.17	16396
DDX3X_t1	-1.36	2.68E-09	-0.14	130	-10.92	13854	-0.02	144	-1.68	9608	NA	NA	NA	NA
SLC26A1_t2	-1.38	3.30E-08	-0.13	134	-10.92	13855	-0.2	126	-15.29	15273	-0.16	120	-11.17	13299
DDAH1	-1.2	0.021771539	-0.12	141	-10.92	13856	-0.15	154	-15.27	15268	0.05	156	5.71	5576
ASB2	-1.33	3.93E-06	-0.16	120	-10.92	13857	-0.03	103	-1.38	9421	-0.28	102	-14.53	14249
OR12D3	-1.47	5.40E-16	-0.15	124	-10.92	13858	-0.32	112	-19.68	15951	-0.2	123	-14.65	14287
EFEMP2	-1.55	3.66E-13	-0.2	104	-10.92	13859	-0.05	123	-3.84	11001	-0.32	112	-19.8	15295
P2RX5	-1.17	0.000108951	-0.13	132	-10.92	13860	-0.08	138	-6.83	12580	-0.28	110	-16.78	14748
COX19	1.11	0.00303374	-0.14	128	-10.92	13861	0.04	146	3.85	5976	0	136	0.05	8590
TEX14_t2	1.01	0.460241627	-0.22	99	-10.93	13862	0.01	117	0.49	8148	-0.14	98	-6.59	11686
CXCR1	-1.18	9.18E-07	-0.18	112	-10.93	13863	-0.23	108	-13.32	14833	-0.18	122	-12.87	13809
IL1RL1	-1.86	8.70E-15	-0.29	88	-10.93	13864	-0.17	85	-5.81	12094	-0.36	125	-26.64	16097
MPDU1	-1.44	3.38E-18	-0.19	108	-10.93	13865	-0.3	118	-20.36	16025	-0.3	120	-21.19	15536
SLC25A27	-1.17	2.31E-06	-0.14	129	-10.93	13866	-0.17	132	-14.06	15004	-0.15	151	-15.52	14486
CRTC3	-1.04	0.237259513	-0.14	128	-10.94	13867	0.05	126	3.91	5945	-0.07	136	-6.2	11521
TKTL1	1.08	0.466201022	-0.14	130	-10.94	13868	-0.07	124	-4.98	11644.5	-0.05	109	-3.24	10285
G6PC	-1.97	1.76E-16	-0.11	147	-10.94	13869	-0.31	121	-21.98	16164	-0.19	152	-19.04	15180
CFHR3	-2.24	3.43E-10	-0.17	116	-10.95	13870	-0.25	115	-16.49	15508	-0.17	130	-13.95	14097
USO1	-1.28	3.15E-05	-0.17	116	-10.95	13871	-0.12	125	-8.67	13383	-0.14	119	-9.62	12786
SBNO2	-1.23	4.32E-11	-0.21	103	-10.95	13872	-0.2	101	-10.42	14038	-0.32	107	-18.24	15031
GGTLC2	-1.83	1.21E-16	-0.34	82	-10.95	13873	-0.05	115	-2.99	10465	-0.36	88	-13.73	14029
CHAMP1	-1.07	0.00386091	-0.13	135	-10.95	13874	-0.13	117	-8.47	13312	-0.14	127	-10.83	13191
CARHSP1	-1.35	1.13E-08	-0.15	124	-10.95	13875	-0.15	139	-13.59	14889	-0.22	140	-19.59	15261
NEUROD4	-1.04	0.001046483	-0.12	142	-10.96	13876	-0.13	122	-9.67	13763	-0.09	158	-10.03	12935
IL36G	1.01	0.737411233	-0.16	118	-10.96	13877	-0.11	120	-7.74	13005	-0.02	106	-1.12	9258
SLC23A3	-1.07	0.20329382	-0.16	120	-10.96	13878	-0.08	107	-4.35	11289	-0.13	112	-8.01	12240
CALCOCO1	-1.04	0.104081827	-0.13	136	-10.96	13879	-0.06	162	-6.46	12398	-0.18	112	-11.17	13301
ELF1	-1.4	6.53E-15	-0.15	124	-10.96	13880	-0.11	128	-8.72	13403	-0.25	135	-21.17	15530
AP1S2_t1	-1.36	2.17E-07	-0.12	139	-10.96	13881	NA	NA	NA	NA	NA	NA	NA	NA
PHF17_t1	-1.24	2.13E-08	-0.16	120	-10.96	13882	-0.2	133	-16.67	15542	-0.19	124	-14.28	14185
CSF2RB	1	0.910774375	-0.15	124	-10.97	13883	-0.04	121	-3.16	10583	-0.1	141	-9.49	12756
IFI44L	-2.15	5.43E-19	-0.14	128	-10.97	13884	-0.11	140	-10.09	13912	-0.28	135	-23.57	15839
GIGYF2	-1.08	0.001472808	-0.13	132	-10.97	13885	-0.01	147	-1.11	9229	-0.16	138	-13.71	14027
NXN	-1.2	2.84E-09	-0.13	132	-10.97	13886	-0.15	123	-11.02	14245	-0.21	123	-15.3	14437
PDCD1	-1.28	9.95E-06	-0.16	119	-10.97	13887	-0.11	123	-8.19	13186	-0.31	110	-18.34	15055
GGT5_t1	-2.04	1.03E-28	-0.27	90	-10.98	13888	NA	NA	NA	NA	-0.4	92	-16.8	14756
SAMD3	-1.35	1.63E-09	-0.19	109	-10.98	13889	-0.09	120	-6.34	12344	-0.29	140	-26	16030
ARSE	-1.49	6.49E-06	-0.21	103	-10.98	13890	-0.18	120	-12.62	14671	-0.23	114	-14.82	14329
CYP26B1_t1	1.01	0.671924476	-0.13	136	-10.99	13891	NA	NA	NA	NA	-0.05	130	-4.2	10715
CCDC25_t1	-1.36	8.47E-08	-0.15	123	-10.99	13892	-0.17	137	-15.19	15248	NA	NA	NA	NA
TOLLIP_t1	-1.12	0.001151581	-0.14	128	-11	13893	NA	NA	NA	NA	-0.12	120	-8.07	12255
PMAIP1	-1.1	0.005882831	-0.12	144	-11	13894	-0.08	137	-6.96	12630	-0.24	130	-19.05	15183

SHOX	-1.05	0.000369734	-0.16	117	-11	13895	-0.12	123	-8.84	13448	-0.11	116	-7.44	12008
NKAPL	-1.1	0.000204581	-0.14	131	-11.01	13896	-0.11	112	-6.76	12539	-0.19	125	-14.21	14158
PRDM2_t2	-1.03	0.11971954	-0.12	145	-11.02	13897	-0.1	136	-8.36	13264	-0.03	120	-1.82	9620
CSK	-1.28	4.09E-14	-0.18	112	-11.02	13898	-0.17	111	-10.52	14070	-0.35	151	-35.51	16439
NFKBID	-1.13	1.05E-08	-0.18	111	-11.02	13899	0	104	-0.03	8508	-0.24	117	-15.75	14530
CLPTM1L	1.07	0.182967439	-0.13	132	-11.02	13900	0.03	120	2.14	7051	-0.03	126	-2.09	9749
DOCK1	-1.09	0.02479965	-0.15	122	-11.02	13901	-0.01	121	-0.64	8902	-0.11	132	-8.87	12551
FDX1	-1.43	1.33E-09	-0.14	131	-11.02	13902	-0.22	120	-15.19	15246	-0.16	151	-15.76	14533
KRT80_t1	-1.1	1.67E-09	-0.16	121	-11.02	13903	NA	NA	NA	NA	-0.17	125	-12.68	13755
MED11	-1.23	1.02E-09	-0.19	108	-11.03	13904	-0.22	131	-18.1	15749	-0.25	120	-17.83	14950
HSD17B10	-1.27	4.18E-06	-0.18	111	-11.04	13905	-0.25	129	-19.66	15948	-0.11	99	-5.48	11231
HEXIM1	-1.19	0.000182066	-0.15	125	-11.04	13906	-0.16	143	-15.03	15208	-0.14	126	-10.54	13098
C1QB	-2.49	1.35E-21	-0.2	105	-11.05	13907	-0.18	107	-10.41	14037	-0.38	105	-21.06	15512
NHLRC3_t2	-1.32	1.17E-07	-0.12	140	-11.05	13908	-0.09	125	-6.99	12643	-0.23	135	-19.33	15224
ZNF713	-1.18	4.55E-08	-0.16	120	-11.05	13909	-0.13	146	-12.49	14634	-0.15	119	-10.66	13138
ABCC3	-1.36	0.000504128	-0.16	120	-11.05	13910	-0.1	132	-7.82	13038	-0.15	139	-12.98	13837
FYB	-1.9	3.73E-14	-0.13	135	-11.05	13911	-0.05	139	-4.03	11115	-0.28	138	-24.94	15954
SEMA4A	-1.49	6.04E-20	-0.19	109	-11.05	13912	-0.25	146	-23.67	16287	-0.24	102	-12.63	13744
ITIH2	-1.66	7.61E-08	-0.26	93	-11.06	13913	-0.3	116	-20.08	15999	-0.17	119	-11.69	13454
D2HGDH	-1.28	1.65E-07	-0.18	111	-11.06	13914	-0.11	120	-7.95	13091	-0.29	103	-15.45	14468
PPP1R14D	-1.33	4.20E-15	-0.18	110	-11.07	13915	-0.34	144	-31.66	16474	-0.09	111	-5.76	11339
FAM92B	-1.03	0.105413748	-0.14	129	-11.08	13916	-0.06	113	-3.9	11038	-0.22	127	-16.97	14796
PEX5_t1	-1.07	0.003256278	-0.13	136	-11.08	13917	NA	NA	NA	NA	-0.12	113	-7.44	12010
IKZF1_t1	-1.19	1.29E-09	-0.14	128	-11.08	13918	-0.01	131	-0.77	8993	NA	NA	NA	NA
ARHGEF12_t1	-1.09	9.68E-07	-0.1	164	-11.09	13919	-0.11	123	-8.31	13243	NA	NA	NA	NA
TMEM39B	-1.08	5.09E-05	-0.15	124	-11.09	13920	-0.09	152	-9.67	13759	-0.13	116	-8.63	12466
FAM211B	-1.31	1.83E-10	-0.22	100	-11.1	13921	-0.03	109	-2.04	9851	-0.24	114	-15.04	14386
AQP1_t1	-1.35	0.000147365	-0.14	128	-11.1	13922	NA	NA	NA	NA	-0.26	134	-21.48	15583
ANKRD33	-1.05	0.003284839	-0.17	114	-11.11	13923	-0.19	115	-12.37	14608	-0.12	110	-7.06	11864
RDH8	-1.03	0.09478673	-0.15	126	-11.12	13924	-0.3	148	-29.31	16455	-0.03	109	-1.82	9618
FDXR	-1.13	0.054795935	-0.18	111	-11.12	13925	-0.15	88	-5.55	11944	-0.1	128	-8.16	12295
JUN	-2.19	1.03E-16	-0.15	122	-11.12	13926	-0.22	135	-18.4	15791	-0.24	124	-17.49	14881
SLC34A1	-1.04	0.075626509	-0.2	105	-11.13	13927	-0.11	119	-7.84	13048	-0.19	132	-15.58	14491
UQCRC2	-1.26	2.40E-05	-0.29	88	-11.13	13928	-0.09	120	-6.04	12207	-0.17	100	-8.31	12365
RBP1	-2.69	1.18E-16	-0.26	92	-11.13	13929	-0.08	103	-4.06	11126	-0.4	126	-30.19	16297
TNFRSF1A_t1	-1.44	3.98E-15	-0.15	124	-11.13	13930	NA	NA	NA	NA	-0.24	120	-16.47	14677
SERPINA4	-3.23	5.28E-21	-0.33	84	-11.13	13931	-0.37	124	-27.72	16432	-0.36	118	-24.53	15917
DCXR	-1.93	9.27E-08	-0.29	88	-11.14	13932	-0.25	113	-15.52	15309	-0.24	86	-8.68	12486
KCTD11	-1.17	8.73E-05	-0.17	116	-11.14	13933	-0.05	124	-3.7	10931	-0.26	132	-20.95	15494
HP	-2.45	1.15E-10	-0.23	98	-11.14	13934	-0.25	84	-8.66	13380	-0.27	104	-14.57	14258
HGD	-1.53	3.57E-06	-0.18	112	-11.14	13935	-0.29	98	-14.14	15015	-0.16	104	-8.82	12530
IFITM1	-1.97	6.16E-16	-0.2	105	-11.15	13936	-0.2	132	-16.4	15494	-0.39	150	-38.75	16475
DEFA4	-1.42	3.83E-06	-0.16	119	-11.15	13937	-0.16	101	-8.29	13237	-0.22	106	-12.17	13601
DESI1	-1.24	2.91E-06	-0.15	123	-11.15	13938	-0.05	134	-4.04	11121	-0.17	130	-13.59	13998
KIRREL2_t2	-1.13	3.66E-07	-0.16	118	-11.16	13939	-0.19	129	-14.96	15191	-0.14	104	-7.43	12003
FAM129B	-1.18	0.000288531	-0.17	115	-11.16	13940	-0.17	120	-12.14	14548	-0.15	130	-11.73	13465
ITGAL_t1	-1.26	3.04E-06	-0.13	136	-11.16	13941	-0.04	157	-3.9	11041	NA	NA	NA	NA
LRRC4C	-1.6	2.99E-23	-0.27	91	-11.16	13942	-0.16	88	-6.03	12199	-0.35	95	-15.66	14512
CCNI	-1.43	2.79E-16	-0.17	115	-11.17	13943	-0.12	96	-5.58	11962	-0.25	81	-7.79	12158
KCNJ8	-2.12	2.16E-10	-0.17	115	-11.17	13944	-0.3	124	-21.99	16167	-0.15	126	-11.07	13277
ANXA3	-2.08	5.46E-12	-0.2	105	-11.17	13945	-0.08	116	-5.21	11781	-0.35	109	-20.46	15408
SPG21	-1.01	0.89345587	-0.19	110	-11.18	13946	-0.07	126	-5.7	12033	-0.02	135	-1.3	9344
ARHGAP44	-1.16	0.088360954	-0.16	119	-11.18	13947	-0.12	130	-9.66	13755	-0.14	132	-11.64	13440
ANXA7	-1.1	0.018156786	-0.14	128	-11.18	13948	-0.12	108	-6.73	12527	-0.06	129	-4.94	11031
TTC31	-1.1	0.000206661	-0.2	106	-11.19	13949	-0.14	158	-15.12	15229	-0.19	124	-13.95	14099
CLEC11A	-1.41	5.48E-14	-0.16	120	-11.19	13950	-0.1	130	-8.04	13129	-0.26	100	-13.25	13909
SASH1_t1	-1.27	2.13E-07	-0.16	121	-11.19	13951	NA	NA	NA	NA	-0.16	161	-17.77	14935
COQ2	-1.2	1.48E-07	-0.15	125	-11.19	13952	-0.17	122	-11.96	14490	-0.25	129	-19.62	15270
C1QA	-2.12	3.21E-17	-0.22	102	-11.19	13953	-0.17	114	-10.75	14160	-0.39	108	-22.34	15703
RUNDC3B	-1.38	0.000196889	-0.15	127	-11.2	13954	-0.15	131	-11.84	14460	0.01	132	1.02	8005
HEBP2	-1.26	1.81E-05	-0.14	131	-11.2	13955	-0.07	131	-5.98	12172	-0.14	115	-8.86	12547
WNT4	-1.2	0.015293119	-0.15	124	-11.2	13956	-0.16	122	-11.34	14329	-0.18	118	-12.37	13661
SORBS1	-1.1	6.42E-05	-0.12	140	-11.21	13957	-0.14	122	-9.73	13787	-0.07	130	-5.64	11294
ADAMTS5	-1.47	1.11E-06	-0.13	138	-11.21	13958	0	133	-0.24	8635	-0.13	145	-12.65	13747
MEGF11	-1.05	0.002581555	-0.14	131	-11.21	13959	-0.06	127	-4.39	11317	-0.07	118	-4.49	10835
ABCC2	-1.28	0.012916208	-0.12	144	-11.22	13960	-0.1	107	-5.57	11956	-0.01	97	-0.7	9033
MMADHC	-1.24	2.44E-06	-0.15	124	-11.22	13961	-0.11	125	-8.57	13348	-0.19	132	-15.86	14552
SNX10_t1	-1.19	0.053424205	-0.12	147	-11.23	13962	NA	NA	NA	NA	-0.02	132	-1.58	9488
NAP1L3	-1.12	2.54E-05	-0.13	134	-11.23	13963	-0.02	105	-1.11	9224	-0.24	143	-21.89	15640
PCSK2	1	0.941002651	-0.14	130	-11.23	13964	-0.06	144	-5.44	11890	-0.05	118	-3.16	10248
B4GALT1	-1.33	3.00E-09	-0.13	134	-11.24	13965	-0.15	151	-15.35	15280	-0.16	124	-12.15	13591
SOX6	-1.02	0.06668288	-0.14	132	-11.24	13966	-0.07	159	-8.11	13157	-0.17	139	-14.91	14355
ACOX2	-1.98	7.70E-09	-0.26	94	-11.24	13967	-0.28	93	-12.1	14537	-0.21	105	-11.73	13470
PPM1A_t2	-1.17	7.36E-05	-0.13	139	-11.24	13968	-0.26	135	-22.11	16179	-0.08	143	-7.43	12001
ZFAND5_t1	-1.4	2.23E-08	-0.13	136	-11.24	13969	NA	NA	NA	NA	NA	NA	NA	NA
GPR124_t1	-1.75	3.84E-13	-0.17	118	-11.25	13970	-0.02	147	-1.51	9502	NA	NA	NA	NA
BMF	-1.13	0.057312148	-0.15	123	-11.25	13971	0.03	131	2.52	6815	-0.13	130	-10.01	12929
DIO1	-1.72	6.26E-07	-0.16	120	-11.25	13972	-0.21	143	-19.09	15879	-0.17	147	-16.01	14592
RPL3_t2	-1.33	8.26E-09	-0.28	90	-11.25	13973	-0.2	90	-7.93	13089	-0.26	113	-16.09	14607
WDTC1_t1	-1.02	0.212785985	-0.13	139	-11.25	13974	NA	NA	NA	NA	-0.12	120	-8.41	12404
GRIA1	-1.07	2.59E-05	-0.14	129	-11.25	13975	-0.18	132	-14.59	15120	-0.11	130	-8.47	12419
FSCN3	-1.48	1.05E-22	-0.3	88	-11.26	13976	-0.28	108	-16.35	15482	-0.33	88	-12.38	13663
CD6_t1	-1.72	3.06E-15	-0.17	115	-11.26	13977	NA	NA	NA	NA	-0.24	136	-20.77	15459
PDCD4	-1.14	0.004399683	-0.13	136	-11.26	13978	-0.09	137	-8.09	13144	-0.02	130	-1.89	9662
IGSF10	-1.21	6.96E-09	-0.18	113	-11.26	13979	-0.11	132	-8.96	13484	-0.18	124	-13.26	13916
TSC22D3	-1.59	1.14E-14	-0.19	109	-11.27	13980	-0.14	124	-10.42	14039	-0.23	123	-16.8	14755
NIT1	-1.14	0.002007388	-0.16	121	-11.27	13981	-0.17	136	-14.75	15157	-0.11	122	-8.2	12315
GABRA5	-1.03	0.048564126	-0.15	125	-11.27	13982	-0.04	147	-4.22	11219	-0.08	113	-5	11048
BEST1	-1.08	0.039424901	-0.14	129	-11.29	13983	-0.04	122	-2.67	10260	-0.13	98	-6.22	11529
PLCG2	-1.36	3.87E-07	-0.21	103	-11.29	13984	-0.16	143	-14.65	15138	-0.18	112	-11.03	13268
RSG1	-1.16	3.95E-12	-0.16	121	-11.29	13985	-0.24	120	-16.58	15523	-0.18	112	-11.3	13348
TAGLN	-1.57	6.39E-12	-0.14	128	-11.29	13986	-0.04	94	-1.95	9782	-0.27	101	-13.92	14086
TM2D3	-1.05	0.203412441	-0.14	128	-11.3	13987	-0.07	104	-3.6	10882	-0.11	123	-8.12	12274
ACTN1_t1	-1.22	6.46E-06	-0.16	122	-11.3	13988	NA	NA	NA	NA	NA	NA	NA	NA
ODF2L	-1.05	0.054925832	-0.14	132	-11.3	13989	0.08	134	6.58	4465	-0.2	128	-15.62	14499
TNFSF10	-1.42	7.51E-05	-0.12	143	-11.31	13990	-0.22	147	-21.02	16091	-0.1	112	-6.5	11639
RIN1	-1.13	4.83E-09	-0.14	131	-11.31	13991	-0.06	119	-4.13	11167	-0.16	80	-4.73	10952
CCL3L3_t1	-1.87	1.69E-19	-0.19	110	-11.31	13992	NA	NA	NA	NA	NA	NA	NA	NA
SLC47A1	-1.37	0.000438813	-0.15	127	-11.31	13993	-0.25	121	-17.71	15697	0	122	-0.22	8741
NPY5R_t1	-1.12	7.49E-11	-0.22	102	-11.32	13994	NA	NA	NA	NA	-0.25	126	-19.35	15229
HECTD1	-1.38	4.58E-05	-0.15	128	-11.33	13995	-0.19	148	-18.66	15819	-0.11	130	-8.88	12552
FOXS1	-1.14	1.00E-06	-0.21	104	-11.34	13996	-0.16	118	-10.99	14233	-0.21	95	-9.27	12678
EPN2	-1.19	8.15E-05	-0.12	147	-11.34	13997	-0.03	124	-2.08	9876	-0.05	140	-4.19	10708
FAIM3	-1.2	9.68E-09	-0.14	130	-11.34	13998	-0.18	140	-16.12	15448	-0.2	127	-15.44	14466
H6PD_t1	-1.23	5.72E-11	-0.13	135	-11.34	13999	NA	NA	NA	NA	-0.21	145	-20.12	15352
KCNJ9	-1.07	4.89E-08	-0.14	134	-11.35	14000	-0.23	120	-16.01	15421	-0.11	113	-6.67	11722

KCTD21	-1.01	0.74203935	-0.13	136	-11.35	14001	-0.1	125	-7.67	12966	-0.03	116	-2.26	9825
MS4A15	-1.29	3.23E-13	-0.2	108	-11.35	14002	-0.27	137	-23.72	16289	-0.2	107	-11.59	13426
LYSMD2	-1.18	0.000224965	-0.15	125	-11.35	14003	-0.06	108	-3.45	10767	-0.06	150	-5.84	11382
LGALS3BP	-1.27	2.51E-06	-0.17	118	-11.36	14004	-0.1	108	-5.61	11982	-0.28	121	-20.14	15354
GAMT_t1	-1.74	2.31E-10	-0.2	107	-11.36	14005	-0.26	99	-12.79	14711	-0.23	92	-9.46	12747
TMEM159	-1.09	0.027841343	-0.14	129	-11.37	14006	0.05	131	4.18	5788	-0.19	164	-21.63	15610
TCEAL2	-1.03	0.24299651	-0.16	121	-11.37	14007	-0.09	120	-6.27	12318	-0.05	117	-3.19	10262
APOC3	-1.86	9.71E-07	-0.18	114	-11.38	14008	-0.32	111	-19.33	15906	-0.18	102	-9.46	12746
ASPDH	-1.23	1.53E-13	-0.27	92	-11.38	14009	-0.27	104	-14.34	15063	-0.24	88	-9.23	12665
PDZRN3	-1.47	1.13E-09	-0.19	110	-11.38	14010	-0.09	110	-5.53	11936	-0.26	138	-22.99	15776
SLAMF1	-1.12	0.006226168	-0.12	148	-11.38	14011	0.02	124	1.79	7285	-0.22	124	-15.92	14567
BNC1	-1.03	0.00956377	-0.13	139	-11.38	14012	-0.04	119	-2.83	10359	-0.1	152	-10.6	13118
PPYR1	-1.18	1.91E-09	-0.17	117	-11.38	14013	-0.27	128	-20.84	16075	-0.22	115	-14.42	14221
SLC39A14	-1.21	1.96E-09	-0.15	128	-11.38	14014	-0.19	126	-14.27	15048	-0.25	126	-19.1	15190
EN2	-1.28	4.79E-07	-0.15	126	-11.38	14015	-0.12	127	-9.39	13657	-0.14	134	-11.46	13385
CMKLR1	-1.17	9.80E-11	-0.14	130	-11.38	14016	-0.07	134	-5.73	12045	-0.33	135	-27.92	16183
CHST14_t1	-1.03	0.044212253	-0.16	121	-11.39	14017	-0.14	110	-8.69	13394	NA	NA	NA	NA
FCGR3A	-2.87	1.26E-22	-0.2	107	-11.39	14018	-0.19	122	-13.89	14966	-0.38	145	-35.79	16447
GPER	-1.55	0.000258432	-0.2	108	-11.39	14019	-0.17	135	-14.68	15142	-0.13	153	-13.62	14006
PPP1R32	-1.19	5.13E-07	-0.19	109	-11.39	14020	-0.18	120	-12.33	14593	-0.19	84	-6.59	11688
ZC2HC1C_t2	-1.32	8.27E-11	-0.18	114	-11.39	14021	-0.26	129	-20.39	16027	-0.19	130	-14.81	14327
CLEC4D	-1.24	1.92E-08	-0.14	131	-11.39	14022	-0.13	127	-9.71	13777	-0.32	133	-26.82	16115
GSDMA	-1.23	2.73E-16	-0.18	113	-11.4	14023	-0.29	118	-19.8	15961	-0.23	118	-15.97	14581
GBP5	-1.72	2.56E-06	-0.16	120	-11.4	14024	-0.05	129	-3.84	11000	-0.26	127	-20.24	15376
CALM1	-1.35	4.33E-11	-0.11	155	-11.41	14025	-0.19	126	-14.17	15021	-0.1	146	-9.73	12819
CABP5	-1.11	1.66E-08	-0.16	122	-11.41	14026	-0.13	140	-11.67	14417	-0.15	126	-11.36	13364
LTB	-1.78	9.10E-07	-0.23	100	-11.41	14027	-0.03	83	-1.06	9191	-0.31	102	-16.27	14638
PLRG1	-1.12	0.009089285	-0.14	131	-11.42	14028	-0.02	119	-1.17	9267	-0.07	136	-5.72	11324
UPP2	-1.3	0.033472828	-0.14	132	-11.42	14029	-0.18	134	-15.03	15207	-0.07	144	-6.18	11513
TNFSF12	-1.8	2.01E-16	-0.22	102	-11.42	14030	-0.1	121	-7.16	12720	-0.38	108	-22.3	15700
EMX2OS	-1.02	0.307957381	-0.14	134	-11.42	14031	-0.04	143	-3.59	10866	-0.15	153	-15.86	14550
PLSCR3	-1.33	1.88E-15	-0.16	122	-11.42	14032	-0.12	130	-9.57	13721	-0.26	127	-20.4	15399
PIWIL4	-1.44	3.60E-10	-0.18	112	-11.43	14033	-0.02	117	-1.56	9522	-0.3	116	-20.01	15332
PDE4DIP_t3	-1.85	1.49E-13	-0.18	114	-11.43	14034	-0.24	111	-14.52	15109	-0.28	115	-18.27	15038
LILRA4	-1.39	1.34E-19	-0.23	99	-11.44	14035	-0.16	93	-6.82	12573	-0.41	98	-19.6	15262
TRPM7	-1.15	0.001288918	-0.13	136	-11.44	14036	-0.09	128	-7.18	12730	-0.07	137	-6.25	11552
AFMID	-1.05	0.267906641	-0.14	133	-11.44	14037	-0.03	138	-2.8	10339	-0.12	121	-8.67	12483
DUSP2	-2.15	3.69E-18	-0.16	121	-11.45	14038	-0.13	153	-12.92	14738	-0.33	120	-23.01	15779
HBQ1	-1.27	3.94E-11	-0.18	115	-11.45	14039	-0.17	98	-8.29	13239	-0.23	67	-3.86	10570
GHRL	-1.17	3.46E-13	-0.23	100	-11.45	14040	-0.12	101	-6.21	12289	-0.32	116	-21.27	15550
PDCD7_t1	-1.12	0.000774551	-0.15	124	-11.45	14041	-0.06	134	-5.08	11705	NA	NA	NA	NA
SIN3A	-1.01	0.645005256	-0.13	135	-11.45	14042	-0.05	132	-3.97	11091	-0.06	131	-5.26	11142
CNN2_t1	-1.3	2.07E-07	-0.21	104	-11.46	14043	NA	NA	NA	NA	NA	NA	NA	NA
LONRF1_t1	-1.37	1.27E-17	-0.14	134	-11.46	14044	-0.16	130	-12.67	14681	NA	NA	NA	NA
SEC31B	-1.43	6.59E-09	-0.17	119	-11.46	14045	-0.08	134	-6.71	12515	-0.28	135	-23.57	15837
METTL7B	-1.63	2.43E-10	-0.21	104	-11.46	14046	-0.21	116	-13.68	14914	-0.24	126	-18.58	15095
BMX	-1.25	2.45E-10	-0.19	110	-11.46	14047	-0.11	116	-7.35	12814	-0.24	114	-15.61	14497
MFSD12_t2	-1.24	1.49E-08	-0.23	100	-11.47	14048	-0.16	113	-9.77	13805	-0.29	103	-15.33	14447
PLCB2	-1.2	5.17E-13	-0.16	122	-11.47	14049	-0.07	122	-5.36	11851	-0.22	112	-13.87	14068
FN1_t2	-1.2	0.001267179	-0.15	127	-11.48	14050	-0.13	136	-10.79	14173	-0.09	126	-7.15	11898
COL25A1_t2	-1.03	0.023760101	-0.13	137	-11.48	14051	-0.12	147	-11.22	14305	-0.08	141	-7.71	12121
CD48	-1.88	3.77E-13	-0.17	116	-11.48	14052	-0.04	121	-3	10473	-0.35	92	-14.56	14257
TMEM25	-1.15	1.45E-07	-0.16	120	-11.48	14053	-0.19	120	-13.55	14882	-0.22	130	-17.25	14842
SH3BP5	-1.57	7.71E-12	-0.14	131	-11.48	14054	-0.04	141	-3.34	10700	-0.22	138	-19.58	15260
ZHX3	-1.04	0.427175827	-0.12	148	-11.48	14055	-0.13	137	-10.91	14209	-0.06	131	-4.66	10919
SLC39A8	-1.58	2.40E-08	-0.17	119	-11.49	14056	-0.22	167	-25.29	16356	-0.08	128	-6.44	11623
CLEC1B	-13.59	2.51E-51	-0.36	82	-11.49	14057	-0.3	90	-12.12	14540	-0.4	110	-24.26	15899
TNFRSF10B	-1.35	4.67E-11	-0.13	141	-11.5	14058	-0.15	124	-11.11	14272	-0.21	158	-22.67	15737
CYP3A5	-2.02	1.19E-07	-0.17	117	-11.5	14059	-0.27	90	-10.83	14184	-0.06	105	-3.04	10201
BAIAP3	-1.13	0.000144219	-0.19	110	-11.51	14060	-0.13	104	-6.83	12578	-0.11	105	-6.06	11459
TUSC1_t1	-1.29	1.58E-05	-0.13	137	-11.51	14061	NA	NA	NA		-0.12	137	-10.5	13086
HGFAC	-4.89	1.09E-28	-0.33	85	-11.51	14062	-0.32	111	-19.32	15905	-0.37	83	-12.35	13656
DENND5B	-1.12	0.02580985	-0.12	150	-11.51	14063	-0.08	139	-6.78	12555	-0.04	130	-3.43	10369
CFH_t2	-2.05	8.92E-09	-0.14	135	-11.51	14064	-0.23	110	-13.87	14959	-0.15	96	-6.83	11783
TMC8_t1	-1.18	3.64E-14	-0.15	126	-11.52	14065	NA	NA	NA		-0.25	120	-17.32	14849
FA2H	-1.21	0.022102396	-0.12	147	-11.52	14066	-0.03	117	-1.71	9629	-0.27	146	-26.13	16046
FAM82A2	-1.11	0.004679591	-0.13	140	-11.53	14067	-0.13	120	-9.09	13558	-0.06	123	-4.43	10819
GABARAP	-1.3	1.44E-15	-0.15	128	-11.53	14068	-0.23	134	-19.49	15928	-0.33	114	-20.8	15469
FERMT3	-1.41	3.93E-13	-0.16	121	-11.53	14069	-0.04	120	-2.49	10142	-0.34	103	-18	14978
FAM78A	-1.4	1.51E-08	-0.13	136	-11.53	14070	-0.01	138	-1.14	9248	-0.28	143	-26.02	16033
CREB3L1	-1.08	0.000917681	-0.16	120	-11.53	14071	-0.09	123	-6.75	12533	-0.19	131	-15.3	14439
XPA	-1.09	0.007922619	-0.18	115	-11.54	14072	-0.16	115	-10.21	13961	-0.09	112	-5.41	11205
STX2	-1.06	0.14475134	-0.15	126	-11.54	14073	-0.02	127	-1.39	9425	-0.07	127	-5.6	11274
PHYHIP	-1.02	0.298175864	-0.16	120	-11.54	14074	0	148	0.02	8449	-0.04	123	-2.84	10104
MAGEB1	-1.02	0.604991943	-0.17	117	-11.54	14075	-0.16	108	-9.35	13638	-0.08	108	-4.43	10815
SHROOM1	-1.19	0.007752541	-0.14	130	-11.55	14076	-0.06	131	-5.18	11765	-0.12	120	-8.37	12384
SLC2A9	-1.4	7.39E-14	-0.21	106	-11.55	14077	-0.21	99	-10.32	14013	-0.25	104	-13.29	13920
RBMS3_t1	-1.16	9.41E-14	-0.23	100	-11.55	14078	-0.06	112	-3.57	10848	-0.26	102	-13.46	13969
HSPB8	-1.09	0.168561416	-0.13	140	-11.55	14079	0.01	132	1.15	7706	-0.12	132	-9.73	12825
MMAB	-1.04	0.404246708	-0.17	120	-11.56	14080	-0.11	145	-10.52	14069	-0.06	148	-5.62	11283
FOLR1	-1.01	0.879561993	-0.14	132	-11.56	14081	-0.1	109	-5.74	12052	-0.14	88	-5.3	11160
NBPF15	-1.1	0.085091412	-0.12	148	-11.56	14082	-0.06	120	-4.06	11129	-0.07	122	-5.24	11135
OVCA2_t1	-1.27	6.04E-11	-0.18	114	-11.56	14083	NA	NA	NA	NA	NA	NA	NA	NA
GSN	-1.82	2.51E-13	-0.24	99	-11.57	14084	-0.1	100	-5.15	11745	-0.28	102	-14.65	14286
AFAP1L2_t1	-1.31	7.78E-14	-0.15	126	-11.57	14085	-0.11	131	-8.81	13438	-0.28	120	-19.68	15278
CA14	-1.58	0.00011601	-0.17	117	-11.58	14086	-0.17	106	-9.35	13636	-0.07	117	-4.99	11046
TMEM87A	-1.1	0.101290762	-0.14	131	-11.58	14087	0.05	159	5.56	5030	-0.15	117	-10.28	13020
SLC16A1	-1.26	0.000111561	-0.15	129	-11.58	14088	-0.15	131	-12.35	14602	-0.09	122	-6.25	11555
IGF1R	-1.01	0.158037193	-0.15	126	-11.58	14089	0.06	159	6.37	4577	-0.13	143	-11.92	13517
SQRDL	-1.22	0.000108697	-0.15	129	-11.58	14090	-0.1	95	-4.66	11470	-0.17	109	-10.29	13024
IDH2	-1.59	1.65E-09	-0.24	98	-11.59	14091	-0.21	100	-10.31	14004	-0.25	126	-18.72	15125
ACVR1C	-1.32	3.00E-05	-0.12	148	-11.59	14092	-0.11	139	-9.36	13642	-0.02	147	-2.01	9708
POLG	-1.14	3.46E-05	-0.19	110	-11.59	14093	-0.1	107	-5.69	12029	-0.16	104	-8.48	12421
GNG2_t1	-1.04	0.012958034	-0.14	135	-11.59	14094	NA	NA	NA		-0.16	143	-15.21	14420
GPR82	-1.03	0.00396042	-0.14	132	-11.6	14095	-0.11	139	-9.72	13784	-0.13	153	-13.66	14015
RASSF9	-1.18	0.000451914	-0.22	102	-11.6	14096	-0.1	126	-7.8	13032	-0.25	107	-14.23	14163
CTIF	-1.18	2.76E-05	-0.14	133	-11.61	14097	-0.11	130	-8.79	13431	-0.14	145	-13.56	13989
BCORP1	-1.02	0.059961365	-0.14	133	-11.62	14098	0.01	132	1.07	7758	-0.16	130	-13.14	13883
GLTPD2	-2.1	5.11E-16	-0.19	112	-11.63	14099	-0.31	98	-14.8	15171	-0.27	105	-14.97	14371
CHKB	-1.17	1.94E-05	-0.24	99	-11.63	14100	0.02	126	1.55	7466	-0.18	98	-8.66	12477
PDK4	-1.46	5.08E-05	-0.16	125	-11.63	14101	-0.15	159	-16.32	15477	-0.07	127	-5.69	11316
DFFA_t2	1.01	0.672600076	-0.14	136	-11.63	14102	-0.03	136	-2.57	10192	-0.05	150	-4.54	10862
DIO3	-1.19	5.24E-05	-0.19	110	-11.63	14103	-0.08	134	-6.36	12355	-0.24	114	-15.04	14388
DHRS4	-1.51	3.77E-11	-0.23	100	-11.64	14104	-0.32	108	-18.66	15817	-0.21	117	-14.07	14131
CLEC3B	-3.56	8.54E-31	-0.3	89	-11.64	14105	-0.3	96	-13.8	14947	-0.39	108	-22.64	15731
VWA8_t1	-1.33	3.79E-08	-0.17	120	-11.64	14106	-0.18	104	-9.98	13874	-0.17	119	-11.46	13392

ZDHHC5	-1.09	0.000199643	-0.17	120	-11.64	14107	-0.1	118	-6.59	12463	-0.14	110	-8.26	12346
AIM2	-1.5	2.80E-10	-0.16	123	-11.65	14108	-0.05	107	-3.12	10560	-0.33	103	-17.59	14902
RGS9	-1.06	0.281812929	-0.21	106	-11.65	14109	-0.09	114	-5.97	12169	-0.19	104	-10.26	13012
NLRP12_t1	-1.09	5.22E-09	-0.19	110	-11.66	14110	NA	NA	NA	NA	-0.2	120	-13.79	14050
TNFAIP8	-1.26	0.000649767	-0.12	148	-11.66	14111	-0.02	114	-0.97	9128	-0.16	115	-10.11	12961
IFIT1	-1.67	2.82E-08	-0.14	136	-11.66	14112	-0.23	143	-21.73	16148	-0.14	128	-10.55	13102
ARGLU1	-1.14	0.001209157	-0.16	122	-11.66	14113	0	132	0.21	8321	-0.21	133	-17.84	14951
GALNS	-1.08	0.000135347	-0.15	129	-11.67	14114	-0.11	108	-6.47	12403	-0.12	124	-8.58	12449
AOX1	-2.84	3.64E-09	-0.16	125	-11.67	14115	-0.21	127	-16.29	15473	-0.19	115	-12.56	13714
BOK	-1.53	2.20E-08	-0.18	115	-11.67	14116	-0.26	114	-16.53	15517	-0.21	127	-15.8	14539
MYO1E	-1.14	6.29E-05	-0.17	119	-11.68	14117	-0.15	146	-14.25	15043	-0.1	125	-7.57	12058
PC	-1.68	2.29E-09	-0.25	96	-11.68	14118	-0.22	104	-11.82	14451	-0.25	90	-9.82	12862
RELN	-1.95	1.21E-08	-0.14	133	-11.68	14119	-0.05	140	-4.38	11303	-0.15	148	-14.45	14226
LAX1_t1	-1.06	0.000583837	-0.15	126	-11.68	14120	NA	NA	NA	NA	-0.23	118	-15.73	14528
ACOX3	-1.09	0.013760444	-0.16	124	-11.69	14121	-0.1	127	-7.68	12969	-0.12	145	-11.69	13451
MYH11_t2	-1.66	2.92E-07	-0.15	129	-11.69	14122	-0.02	144	-1.77	9669	-0.26	156	-27.54	16162
PRSS21_t2	-1.15	6.93E-07	-0.17	120	-11.69	14123	-0.24	130	-19.17	15888	-0.18	110	-10.96	13246
IL1RN	-1.8	1.01E-11	-0.15	129	-11.7	14124	-0.21	128	-16.15	15452	-0.22	114	-13.99	14110
ARFGAP3	-1.15	0.005957245	-0.15	127	-11.72	14125	-0.01	135	-0.82	9032	-0.12	116	-8.15	12288
NR5A1	-1.02	0.556293847	-0.18	116	-11.73	14126	-0.09	140	-8.39	13285	-0.19	123	-13.91	14081
FBXL14	-1.13	5.64E-05	-0.14	131	-11.73	14127	-0.24	138	-20.96	16081	-0.22	118	-14.7	14297
GYPC	-1.25	9.01E-07	-0.17	120	-11.74	14128	-0.08	120	-5.79	12086	-0.31	100	-15.29	14436
NLRC4	-1.67	1.08E-23	-0.2	108	-11.74	14129	-0.13	89	-5.25	11802	-0.37	104	-20.12	15350
HDHD2	-1.42	4.18E-13	-0.17	120	-11.74	14130	-0.2	110	-12.16	14552	-0.16	120	-11.13	13291
ECHDC3	-1.68	5.53E-06	-0.17	121	-11.75	14131	-0.16	115	-10.2	13952	-0.12	105	-6.55	11666
FBLN1_t3	-2.06	1.46E-08	-0.16	122	-11.75	14132	0.02	128	1.18	7689	-0.29	122	-20.95	15493
PON3	-2.41	4.96E-11	-0.27	94	-11.76	14133	-0.34	94	-14.79	15167	-0.24	115	-15.35	14449
RPS6KA1	-1.25	4.12E-10	-0.21	106	-11.77	14134	-0.06	126	-4.46	11366	-0.26	106	-14.54	14251
TFF3	-1.51	0.000228056	-0.15	131	-11.78	14135	-0.08	120	-5.61	11979	-0.21	141	-18.83	15141
CRBN_t1	-1.22	5.44E-06	-0.14	134	-11.78	14136	NA	NA	NA	NA	NA	NA	NA	NA
CD2	-1.86	5.60E-08	-0.19	111	-11.78	14137	-0.03	114	-1.61	9562	-0.3	110	-18.26	15035
TSPYL1	-1.07	3.88E-05	-0.14	135	-11.79	14138	-0.13	133	-11.19	14295	-0.15	137	-13.02	13843
SYNPO	-1.37	3.39E-09	-0.15	128	-11.79	14139	-0.12	142	-11.39	14341	-0.17	128	-13.05	13856
HLA-DPB1_t1	-1.81	4.09E-10	-0.11	154	-11.79	14140	-0.04	129	-3.23	10619	NA	NA	NA	NA
CHSY1	-1.68	8.86E-10	-0.15	128	-11.79	14141	-0.04	141	-3.83	10998	-0.19	144	-18.16	15014
CROT	-1.08	0.109176286	-0.13	144	-11.79	14142	-0.05	134	-4.21	11208	0.02	126	1.49	7757
NPR3	-1.25	0.000532016	-0.11	154	-11.8	14143	-0.09	151	-9.49	13699	-0.05	128	-4.04	10655
ANK2	-1.08	0.013575284	-0.15	130	-11.81	14144	-0.07	145	-6.56	12441	-0.09	130	-7.1	11885
F12	-1.79	7.32E-06	-0.22	104	-11.81	14145	-0.27	118	-18.17	15758	-0.1	89	-3.93	10605
CCNH	-1.35	4.61E-11	-0.22	104	-11.81	14146	-0.18	90	-7.18	12726	-0.21	104	-11.56	13418
TWIST2	-1.06	0.130869832	-0.16	124	-11.81	14147	-0.02	124	-1.71	9627	-0.18	104	-9.9	12898
POLM	-1.07	0.002450151	-0.14	134	-11.82	14148	-0.12	137	-10.71	14135	-0.13	149	-13.35	13937
MAL	-1.24	4.38E-06	-0.17	120	-11.82	14149	-0.08	124	-6.11	12246	-0.26	102	-13.59	13996
VIP	1.03	0.463678451	-0.15	128	-11.82	14150	-0.08	128	-5.91	12138	-0.1	123	-7.51	12037
METTL7A_t1	-1.54	1.92E-07	-0.13	142	-11.83	14151	NA	NA	NA	NA	-0.12	138	-10.63	13126
SULT1A2	-2.29	8.25E-19	-0.28	92	-11.83	14152	-0.31	92	-12.87	14725	-0.28	97	-13.2	13899
RASL11B	-1.31	9.42E-08	-0.19	112	-11.83	14153	-0.08	118	-5.59	11967	-0.28	120	-19.83	15302
ILVBL	-1.38	5.56E-11	-0.18	116	-11.83	14154	-0.18	115	-11.62	14402	-0.28	111	-16.84	14766
DPPA3	1	0.653496153	-0.12	145	-11.83	14155	-0.01	107	-0.32	8698	-0.04	132	-2.97	10168
RAB31_t1	-1.68	2.38E-10	-0.12	147	-11.83	14156	NA	NA	NA	NA	NA	NA	NA	NA
UGCG	-1.52	7.89E-11	-0.22	103	-11.84	14157	-0.1	97	-4.65	11463	-0.22	136	-18.54	15087
UBE2R2	-1.12	1.77E-05	-0.12	147	-11.84	14158	-0.08	130	-6.19	12280	-0.07	128	-5.77	11344
FXYD1	-2.92	2.58E-17	-0.28	92	-11.84	14159	-0.3	137	-26.17	16386	-0.26	90	-10.27	13013
EML3	-1.14	8.66E-07	-0.19	111	-11.84	14160	-0.14	128	-10.84	14190	-0.19	100	-9.52	12765
LARGE	-1.01	0.918553962	-0.17	120	-11.85	14161	-0.18	120	-12.46	14630	-0.05	128	-4.24	10732
PRPF4	-1.07	0.000666821	-0.14	135	-11.85	14162	-0.09	123	-6.27	12315	-0.2	152	-19.92	15311
MBNL1_t2	-1.49	2.12E-09	-0.15	130	-11.85	14163	-0.04	134	-3.66	10912	-0.24	152	-24.58	15920
STAG3	-1.77	3.45E-09	-0.19	112	-11.86	14164	-0.17	120	-11.99	14496	-0.2	120	-14.18	14153
TPK1	-1.48	6.33E-14	-0.16	124	-11.86	14165	-0.16	128	-12.13	14544	-0.21	125	-15.98	14585
ZNF213	-1.01	0.48259267	-0.14	135	-11.86	14166	-0.05	144	-5.05	11685	-0.1	126	-7.49	12032
LILRB1	-1.69	1.85E-20	-0.18	115	-11.86	14167	-0.09	148	-8.79	13429	-0.35	108	-20.18	15366
FXYD2	-2.07	4.48E-14	-0.3	90	-11.86	14168	-0.04	100	-2.19	9961	-0.36	120	-25.41	15986
HTR7	-1.2	2.85E-06	-0.14	135	-11.87	14169	-0.01	146	-0.74	8963	-0.22	136	-18.67	15114
TCP10L	-1.47	0.000659431	-0.16	125	-11.87	14170	-0.15	147	-14.42	15083	-0.18	121	-13.1	13869
SLC25A36	-1.5	8.42E-07	-0.12	149	-11.87	14171	0.05	144	4.27	5737	-0.25	134	-20.8	15468
OCEL1	-1.18	0.004130876	-0.18	115	-11.87	14172	-0.17	100	-8.55	13343	-0.14	126	-10.94	13231
CC2D2B	-1.04	0.156574446	-0.16	124	-11.88	14173	-0.01	114	-0.32	8699	-0.13	136	-10.86	13202
DLST	-1.22	5.71E-06	-0.18	117	-11.88	14174	-0.2	124	-14.56	15117	-0.17	116	-10.96	13245
ELF2	-1.32	7.97E-11	-0.18	117	-11.88	14175	-0.13	122	-9.24	13602	-0.17	133	-14.27	14181
CCS	-1.41	3.80E-13	-0.3	89	-11.88	14176	-0.31	104	-16.97	15585	-0.29	115	-18.53	15085
AP2A1	-1.2	1.16E-08	-0.22	105	-11.88	14177	-0.26	121	-18.61	15812	-0.21	110	-12.31	13647
SLIT2	-1.58	5.76E-07	-0.14	138	-11.89	14178	-0.05	145	-4.3	11268	-0.26	105	-14.21	14160
CACNA2D2	-1.09	4.72E-05	-0.17	119	-11.89	14179	-0.12	155	-12.68	14686	-0.21	128	-16.48	14680
SLC15A1	-1.03	0.373590449	-0.14	135	-11.89	14180	-0.06	140	-5.02	11670	-0.09	124	-6.92	11815
IL37	-1.07	0.00318412	-0.17	122	-11.89	14181	-0.15	102	-7.82	13039	-0.14	117	-9.39	12715
P2RX2	-1.22	1.02E-15	-0.24	100	-11.9	14182	-0.2	120	-14.23	15040	-0.23	102	-12.01	13548
ARL14	-1.45	0.000312837	-0.17	118	-11.9	14183	-0.08	128	-6.49	12408	-0.22	100	-11.2	13308
SRGAP3	-1.08	0.001327446	-0.17	120	-11.9	14184	-0.07	134	-5.85	12109	-0.13	124	-9.53	12769
PHYHD1	-1.81	9.83E-18	-0.24	100	-11.9	14185	-0.23	132	-18.75	15829	-0.34	103	-18.16	15012
TMEM154	-2.04	1.96E-15	-0.14	135	-11.91	14186	-0.08	121	-5.86	12120	-0.28	143	-26.05	16037
PIGR_t1	-2.05	1.50E-07	-0.15	130	-11.92	14187	NA	NA	NA	NA	-0.14	116	-9.27	12673
NDST4	-1.09	2.77E-05	-0.21	108	-11.92	14188	-0.14	92	-5.73	12044	-0.11	80	-3.19	10264
AKAP13	-1.08	2.20E-05	-0.13	143	-11.93	14189	-0.12	125	-8.71	13398	-0.12	147	-11.93	13519
P2RY14	-1.38	3.25E-06	-0.16	125	-11.93	14190	-0.06	119	-4.04	11120	-0.19	127	-14.32	14195
CACNA1H	-1.28	6.00E-05	-0.2	109	-11.94	14191	-0.21	127	-15.86	15381	-0.22	127	-16.59	14715
CRYAB	-1.23	0.012154409	-0.19	113	-11.95	14192	0.02	85	0.67	8027	-0.18	155	-19.24	15211
TNXB_t2	-4.42	2.25E-36	-0.32	87	-11.95	14193	-0.24	118	-16.12	15449	-0.43	100	-21.44	15576
ADRA2A	-1.24	0.00127095	-0.15	131	-11.95	14194	-0.05	131	-3.93	11060	-0.19	123	-13.81	14057
PMP2	-1.03	0.03499809	-0.12	149	-11.96	14195	-0.03	119	-1.76	9661	-0.1	136	-8.44	12412
HMG20A	-1.13	1.97E-05	-0.16	125	-11.97	14196	-0.17	128	-13.08	14770	-0.09	124	-6.98	11839
HAGH	-1.54	2.24E-09	-0.24	100	-11.97	14197	-0.27	114	-17.29	15641	-0.23	107	-12.9	13817
TFB1M	-1.15	0.002063688	-0.17	121	-11.97	14198	-0.02	144	-2.01	9831	-0.23	142	-20.79	15466
RUSC2	-1.11	4.72E-05	-0.23	102	-11.97	14199	-0.13	109	-7.53	12888	-0.26	100	-12.77	13782
SLC8A1_t1	-1.59	1.43E-18	-0.16	127	-11.97	14200	-0.1	131	-7.96	13097	NA	NA	NA	NA
ZNF385B	-1.51	8.99E-07	-0.13	140	-11.98	14201	-0.19	125	-13.9	14970	-0.12	136	-10.51	13089
METTL20	-1.12	4.59E-05	-0.18	116	-11.98	14202	-0.19	131	-15.52	15307	-0.18	119	-12.22	13614
CLECL1	-1.23	2.22E-09	-0.15	128	-11.98	14203	0.02	115	1.6	7427	-0.29	108	-16.6	14716
HELZ	-1.08	0.09701299	-0.11	158	-11.99	14204	-0.06	127	-5	11656	-0.08	147	-7.54	12046
MEGF6_t1	-1.38	3.77E-07	-0.15	128	-12	14205	NA	NA	NA	NA	NA	NA	NA	NA
GPR45	-1.01	0.547559823	-0.19	113	-12	14206	-0.08	112	-4.77	11522	-0.05	120	-3.65	10457
ZNF394	-1.1	0.001588943	-0.17	120	-12	14207	-0.1	131	-8.27	13225	-0.25	121	-17.85	14954
FAM82A1	-1.71	1.33E-14	-0.29	91	-12	14208	-0.22	112	-13.95	14982	-0.27	106	-14.91	14359
MMP15_t1	-1.07	0.000949722	-0.12	150	-12.01	14209	NA	NA	NA	NA	-0.13	122	-9.48	12754
CPSF7	-1.12	1.98E-06	-0.16	123	-12.01	14210	-0.14	131	-11.17	14287	-0.29	130	-22.91	15766
NLN	-1.32	4.83E-07	-0.13	144	-12.01	14211	-0.05	143	-4.28	11258	-0.07	153	-6.7	11733
PMM1	-1.21	8.48E-05	-0.22	104	-12.01	14212	-0.13	114	-8.21	13193	-0.23	110	-13.68	14022

ADAMTSL1	-1.02	0.157033831	-0.14	134	-12.01	14213	-0.15	100	-7.28	12773	-0.05	90	-1.92	9675
MSRB2	-1.1	0.04955492	-0.16	123	-12.01	14214	-0.1	126	-7.29	12784	-0.09	121	-6.27	11560
PILRA	-1.14	0.001075503	-0.2	111	-12.02	14215	-0.05	119	-3.21	10604	-0.25	111	-15.43	14463
PF4	-1.34	4.29E-08	-0.15	130	-12.02	14216	-0.16	121	-11.57	14392	-0.26	124	-19.5	15248
FKBP10	-1.46	1.41E-05	-0.17	120	-12.02	14217	0.02	129	1.88	7224	-0.26	135	-21.94	15651
PEPD	-1.3	0.000431268	-0.21	108	-12.02	14218	-0.11	135	-9.05	13529	-0.11	116	-7.49	12033
SERPINB8_t2	-1.46	6.15E-11	-0.17	120	-12.02	14219	-0.23	122	-16.35	15483	-0.2	124	-14.77	14316
HIBCH	-1.53	7.29E-09	-0.2	109	-12.02	14220	-0.18	110	-11	14239	-0.16	113	-9.9	12895
GAS2	-1.63	2.63E-07	-0.16	124	-12.02	14221	-0.13	133	-10.68	14124	-0.15	127	-11.44	13379
ART4	-2.08	1.19E-17	-0.24	101	-12.03	14222	-0.2	84	-6.92	12614	-0.28	85	-9.78	12840
FTCD	-3.37	1.41E-20	-0.35	84	-12.03	14223	-0.32	88	-12.03	14505	-0.35	91	-14.51	14246
NYAP1	-1.03	0.014033518	-0.14	136	-12.03	14224	-0.12	125	-9.21	13594	-0.09	144	-8.59	12452
ZNF395_t1	-1.17	1.61E-05	-0.14	134	-12.03	14225	NA	NA	NA	NA	-0.22	124	-16.44	14671
AVPI1	-1.77	3.70E-16	-0.16	126	-12.04	14226	-0.37	123	-26.77	16408	-0.21	115	-13.81	14053
HPD	-3.68	3.00E-13	-0.3	90	-12.04	14227	-0.29	108	-16.66	15538	-0.25	92	-10.41	13058
KCNK7	-2.14	1.02E-19	-0.32	88	-12.04	14228	-0.31	86	-11.26	14317	-0.33	80	-9.88	12886
PDK2	-1.18	2.76E-05	-0.2	110	-12.05	14229	-0.19	118	-13	14755	-0.22	104	-12.12	13582
EPHB1	-1.1	2.31E-06	-0.18	117	-12.05	14230	-0.21	133	-17.68	15695	-0.16	136	-13.61	14003
CNIH	-1.04	0.542960189	-0.12	154	-12.05	14231	-0.11	127	-8.53	13333	0.03	158	2.98	6936
ST6GAL1	-1.68	2.53E-09	-0.13	143	-12.05	14232	-0.24	118	-16.09	15439	-0.21	127	-16.52	14687
EXOC3L2	-1.18	3.21E-06	-0.13	144	-12.05	14233	-0.14	127	-10.46	14050	-0.17	123	-12.16	13596
SLC7A4	-1.09	4.34E-06	-0.18	118	-12.05	14234	-0.1	109	-6.18	12275	-0.17	101	-8.59	12453
TAPBPL	-1.19	2.87E-06	-0.17	123	-12.07	14235	-0.19	105	-10.33	14014	-0.17	110	-10.18	12985
CD84_t1	-1.32	8.06E-14	-0.12	147	-12.07	14236	NA	NA	NA	NA	-0.28	126	-21.41	15571
PHACTR2	-1.21	4.69E-06	-0.12	149	-12.08	14237	0	144	-0.41	8754	-0.09	157	-9.29	12683
RGS6	1.01	0.718636212	-0.15	132	-12.08	14238	-0.09	148	-8.41	13293	0	130	-0.35	8820
FAM59A	-1.63	2.71E-10	-0.14	134	-12.08	14239	-0.17	146	-16.38	15487	-0.24	143	-21.94	15652
SPRR2C	-1.04	0.016045805	-0.11	160	-12.09	14240	-0.09	134	-7.95	13094	-0.08	118	-5.42	11209
VAMP2	-1.09	0.000372864	-0.17	120	-12.09	14241	-0.16	132	-13.27	14815	-0.2	136	-17.53	14891
NIM1	-1.02	0.449539642	-0.16	128	-12.09	14242	-0.04	99	-2.2	9962	-0.11	145	-10.12	12963
CARTPT	-1.24	1.17E-10	-0.18	117	-12.1	14243	-0.27	104	-14.4	15080	-0.15	134	-12.61	13737
AUTS2	-1.55	1.10E-05	-0.16	124	-12.1	14244	-0.17	143	-15.4	15290	-0.05	126	-3.49	10392
BLID	-1.04	0.021892051	-0.17	120	-12.11	14245	-0.19	111	-11.89	14472	-0.12	110	-7.21	11922
KRT71	-1.04	0.019904713	-0.22	104	-12.11	14246	-0.11	116	-6.99	12641	-0.1	123	-7.47	12020
POU6F1	-1.19	7.47E-12	-0.14	136	-12.11	14247	-0.15	134	-12.99	14751	-0.21	152	-21.37	15564
TTC19	-1.22	2.78E-11	-0.14	136	-12.11	14248	-0.2	128	-15.22	15256	-0.11	140	-10.22	12998
VSTM2L	-1	0.928693186	-0.16	126	-12.12	14249	-0.16	123	-11.73	14431	-0.13	124	-9.9	12899
SLC37A2	-1.43	2.45E-13	-0.14	136	-12.12	14250	-0.06	143	-5.77	12070	-0.27	121	-19.37	15231
PEX3	-1.24	3.59E-05	-0.16	125	-12.12	14251	-0.11	122	-7.7	12982	-0.07	126	-5.06	11067
CDNF	-1.25	5.22E-10	-0.18	117	-12.12	14252	-0.19	125	-14.36	15069	-0.17	127	-13.37	13943
PRPSAP2	-1.09	0.008529882	-0.14	138	-12.12	14253	-0.1	115	-6.35	12352	0.01	115	0.56	8285
CLDN1_t1	-1.53	6.55E-12	-0.17	121	-12.12	14254	-0.14	120	-9.63	13747	NA	NA	NA	NA
DAPP1	-1.46	1.16E-11	-0.14	136	-12.12	14255	-0.01	128	-1.09	9213	-0.3	120	-20.91	15486
SLCO4A1	-1.59	2.79E-13	-0.16	124	-12.13	14256	-0.07	115	-4.4	11329	-0.32	110	-19.15	15199
MMP28_t2	-1.03	0.072687753	-0.18	119	-12.13	14257	-0.08	114	-5.26	11804	-0.1	121	-7.16	11905
LCK	-1.42	3.50E-06	-0.18	116	-12.13	14258	-0.04	118	-2.77	10322	-0.3	104	-16.1	14610
CREG1	-1.39	1.87E-08	-0.14	137	-12.13	14259	-0.23	126	-17.28	15636	-0.13	114	-8.24	12333
NGF	-1.88	3.39E-16	-0.19	114	-12.14	14260	-0.35	134	-29.81	16463	-0.27	88	-10.25	13008
G0S2	-2.24	1.47E-07	-0.18	119	-12.14	14261	-0.17	134	-14.38	15076	-0.21	148	-20.59	15427
CLSTN3	-1.15	2.08E-07	-0.22	105	-12.14	14262	-0.16	140	-14.35	15068	-0.25	142	-23.02	15780
ZNF167_t2	1	0.821144922	-0.15	132	-12.14	14263	-0.09	119	-6.41	12375	-0.05	117	-3.1	10225
LAMC2_t1	-1.05	0.276287351	-0.16	125	-12.14	14264	0.03	122	1.96	7166	-0.16	146	-15.26	14430
HEPACAM	-1	0.991045065	-0.18	116	-12.15	14265	-0.14	130	-11.15	14282	-0.1	120	-6.68	11727
SLIT3	-1.22	0.001433509	-0.16	126	-12.15	14266	-0.03	136	-2.56	10189	-0.19	120	-13.18	13893
EDN1	-2.08	3.32E-13	-0.19	114	-12.15	14267	-0.06	134	-5.44	11892	-0.28	151	-28.09	16195
FAM13A	-1.08	2.92E-06	-0.14	134	-12.15	14268	-0.01	133	-1.16	9259	-0.11	136	-9.74	12828
AMACR	-1.31	0.017859001	-0.12	153	-12.15	14269	-0.15	139	-13.48	14864	-0.06	138	-5.17	11109
CLIC2	-1.62	1.14E-14	-0.15	132	-12.15	14270	-0.09	122	-6.21	12291	-0.28	127	-21.5	15591
MAP2K4	-1.36	2.14E-07	-0.13	141	-12.16	14271	-0.08	140	-7.29	12781	-0.14	136	-11.91	13514
PHOX2A	-1.37	5.15E-08	-0.21	109	-12.16	14272	-0.25	124	-18.66	15816	-0.21	110	-12.5	13699
FES	-2.08	4.39E-19	-0.24	100	-12.17	14273	-0.25	126	-19.27	15897	-0.33	91	-13.67	14016
SNAI1	-2.28	6.12E-18	-0.19	114	-12.18	14274	-0.08	124	-5.66	12009	-0.3	107	-17.11	14818
HIGD1A	-1.39	6.44E-06	-0.16	128	-12.18	14275	-0.18	147	-17.93	15724	-0.1	133	-8.2	12314
GLIPR2	-1.55	1.82E-09	-0.14	135	-12.19	14276	0	112	0.03	8447	-0.33	143	-30.39	16308
NINL	-1.09	0.342071391	-0.16	126	-12.19	14277	-0.1	131	-7.99	13109	-0.01	136	-0.94	9158
AQP9	-2.57	9.91E-07	-0.14	136	-12.19	14278	-0.18	124	-13.15	14782	-0.06	120	-4.43	10816
CSF2	-1.05	6.46E-07	-0.17	123	-12.2	14279	-0.2	118	-13.7	14921	-0.16	95	-7.36	11980
GCGR	-3.35	1.33E-15	-0.31	89	-12.2	14280	-0.33	105	-18.27	15768	-0.29	100	-14.28	14186
OSBPL6	-1.51	6.50E-07	-0.16	124	-12.2	14281	-0.17	133	-13.82	14948	-0.11	145	-10.67	13141
HSPA12A	-1.22	0.001736053	-0.13	144	-12.2	14282	-0.01	146	-0.53	8835	-0.13	144	-11.77	13477
TRPV1	-1.28	4.46E-06	-0.18	119	-12.21	14283	-0.17	131	-13.53	14877	-0.17	123	-12.52	13705
ZFAND6	-1.13	0.037762701	-0.17	120	-12.21	14284	-0.07	115	-4.4	11328	-0.01	126	-0.5	8912
C8B	-3.68	2.08E-25	-0.31	90	-12.21	14285	-0.39	86	-13.95	14981	-0.32	97	-14.84	14335
ZFYVE28_t1	-1.28	8.63E-07	-0.21	108	-12.21	14286	NA	NA	NA	NA	-0.19	130	-14.82	14330
ADSSL1	-1.36	0.000490565	-0.21	108	-12.22	14287	-0.17	135	-14.32	15061	-0.1	104	-5.47	11227
ACO1	-1.46	5.03E-09	-0.23	104	-12.22	14288	-0.35	146	-33.46	16482	-0.16	132	-13.26	13912
HES7	-1.32	4.83E-06	-0.18	117	-12.23	14289	-0.22	120	-15.25	15266	-0.18	141	-15.94	14572
ADAM19	-1.18	8.80E-05	-0.12	150	-12.23	14290	-0.02	130	-1.43	9447	-0.18	137	-15.72	14524
PLK2	-1.57	2.71E-08	-0.16	128	-12.24	14291	-0.16	117	-10.73	14147	-0.21	111	-12.77	13783
DHRS3	-1.25	3.53E-05	-0.17	124	-12.25	14292	-0.22	134	-18.4	15790	-0.1	128	-7.95	12217
KALRN_t2	-1.33	6.66E-05	-0.13	144	-12.26	14293	-0.15	130	-12.36	14604	-0.06	131	-4.92	11023
COL6A1	-1.1	7.70E-06	-0.18	119	-12.26	14294	-0.1	120	-7.01	12656	-0.19	120	-12.98	13839
CES3_t1	-1.25	0.000147683	-0.16	127	-12.26	14295	NA	NA	NA	NA	-0.15	123	-10.63	13129
DCHS1	-1.31	6.19E-18	-0.24	102	-12.27	14296	-0.13	126	-9.94	13860	-0.31	130	-25.01	15957
KLHL2	-1.45	8.13E-12	-0.14	138	-12.27	14297	-0.21	122	-15.19	15247	-0.06	120	-3.99	10634
NRG1_t2	-1.74	6.82E-10	-0.17	121	-12.27	14298	-0.2	122	-14.73	15152	-0.26	148	-25.34	15981
LARP7	-1.28	7.07E-07	-0.13	142	-12.27	14299	-0.1	120	-7.17	12725	-0.16	126	-11.81	13491
CCT6B	-1.28	2.50E-06	-0.24	102	-12.28	14300	-0.14	113	-8.52	13330	-0.21	97	-9.74	12826
SH2D1A	-1.26	6.78E-06	-0.14	136	-12.28	14301	-0.04	114	-2.74	10298	-0.24	131	-19.22	15205
NRN1_t1	-1.19	3.77E-07	-0.18	120	-12.29	14302	-0.12	126	-9.08	13553	NA	NA	NA	NA
PTPRO	-1.2	2.08E-07	-0.14	140	-12.29	14303	0.06	125	4.87	5410	-0.21	134	-17.42	14869
HBA2	-2.77	7.91E-28	-0.19	115	-12.3	14304	-0.19	94	-8.37	13270	-0.23	123	-16.8	14754
WNT11_t1	-1.26	1.43E-13	-0.18	120	-12.3	14305	NA	NA	NA	NA	NA	NA	NA	NA
EGFLAM	-1.23	3.81E-05	-0.17	121	-12.31	14306	-0.13	144	-12.18	14559	-0.16	128	-12.12	13581
BTBD6	-1.44	8.84E-12	-0.18	120	-12.32	14307	-0.28	103	-14.62	15130	-0.21	103	-11.32	13354
CEP85L_t2	-1.11	1.73E-07	-0.21	109	-12.33	14308	0.03	98	1.48	7505	-0.15	110	-8.73	12503
ZNF439	-1.12	0.017698945	-0.16	127	-12.33	14309	0.15	118	10.2	2793	-0.1	130	-8.04	12244
MAMDC4	-1.41	2.42E-05	-0.23	104	-12.33	14310	-0.11	92	-4.53	11402	-0.28	122	-20.14	15355
ADARB1_t2	-1.38	3.12E-09	-0.17	122	-12.34	14311	-0.04	141	-3.43	10750	-0.19	130	-15.23	14426
TNFAIP2	-1.39	5.12E-06	-0.17	121	-12.34	14312	-0.1	134	-8.81	13441	-0.22	123	-15.83	14544
TNFRSF11B	-1.9	2.44E-11	-0.18	120	-12.34	14313	-0.05	121	-3.72	10944	-0.26	129	-20.23	15373
ZBTB4	-1.54	4.92E-13	-0.16	126	-12.35	14314	-0.13	135	-10.88	14200	-0.33	151	-32.96	16391
QKI_t2	-1.45	1.49E-11	-0.14	141	-12.35	14315	-0.11	138	-9.63	13748	-0.2	159	-21.36	15562
SEMA6B	-1.18	5.41E-08	-0.23	104	-12.36	14316	-0.15	128	-11.81	14450	-0.24	143	-22.67	15736
SRRM1	-1.16	1.03E-08	-0.19	114	-12.36	14317	0	122	-0.29	8670	-0.2	122	-14.34	14201
PTMS	-1.19	3.83E-07	-0.23	103	-12.36	14318	-0.22	116	-14.75	15159	-0.2	119	-13.77	14046

KLKB1	-4.26	1.68E-24	-0.33	88	-12.36	14319	-0.38	118	-25.75	16369	-0.34	123	-24.73	15939
TRIM44	-1.1	0.004467247	-0.15	135	-12.37	14320	-0.11	145	-10.44	14045	-0.03	140	-2.84	10105
NKAIN3	-1.01	0.359703355	-0.19	114	-12.37	14321	-0.05	107	-3.02	10481	-0.04	120	-2.77	10074
SCN11A	-1.02	0.228555271	-0.13	143	-12.37	14322	-0.11	129	-8.38	13277	0.05	130	3.99	6416
KERA	-1.09	2.03E-06	-0.14	137	-12.38	14323	-0.21	133	-17.28	15638	-0.09	123	-6.78	11764
RDX	-1.2	0.019339966	-0.13	144	-12.39	14324	-0.02	134	-1.74	9639	0.05	134	3.88	6483
SIGLEC1_t1	-1.4	1.60E-23	-0.24	101	-12.39	14325	NA	NA	NA	NA	-0.38	129	-30.23	16300
KRTAP1-1_t1	-1.2	1.62E-13	-0.2	113	-12.39	14326	NA	NA	NA	NA	-0.13	115	-8.16	12292
ABI3	-1.46	3.32E-19	-0.25	100	-12.39	14327	-0.23	110	-13.72	14926	-0.39	120	-27.65	16165
ENTPD8	-1.17	5.95E-11	-0.2	111	-12.39	14328	-0.22	101	-11.24	14310	-0.18	119	-12.61	13734
RNF130	-1.49	3.78E-16	-0.25	100	-12.4	14329	-0.29	102	-14.99	15198	-0.19	116	-12.72	13765
ZKSCAN1	-1.03	0.693133864	-0.14	140	-12.4	14330	-0.04	147	-4.33	11281	-0.01	134	-0.61	8990
RAB8A	-1.25	2.60E-13	-0.21	110	-12.4	14331	-0.16	122	-11.52	14379	-0.23	120	-16.01	14590
APOC4	-2.45	6.04E-09	-0.22	107	-12.41	14332	-0.25	122	-17.79	15711	-0.27	75	-6.87	11803
S1PR4	-1.77	4.56E-14	-0.23	104	-12.41	14333	-0.13	100	-6.67	12501	-0.38	112	-23.35	15807
RAB34	-1.73	1.61E-07	-0.21	110	-12.41	14334	-0.04	110	-2.43	10108	-0.25	155	-25.85	16021
HADH_t1	-1.5	2.02E-11	-0.22	106	-12.41	14335	NA	NA	NA	NA	NA	NA	NA	NA
FGD5	-1.08	6.21E-05	-0.13	149	-12.41	14336	-0.11	125	-8.08	13138	-0.16	151	-15.88	14555
PDLIM2_t2	-1.36	1.49E-08	-0.2	113	-12.41	14337	-0.25	108	-14.46	15094	-0.16	157	-16.98	14797
HAL	-3.19	1.37E-14	-0.19	116	-12.42	14338	-0.3	132	-24.89	16338	-0.14	142	-13.09	13864
ZCCHC2	-1.58	2.58E-12	-0.14	142	-12.44	14339	-0.14	133	-11.91	14480	-0.18	133	-15.15	14407
PCDH7_t3	-1.08	0.000442402	-0.13	145	-12.44	14340	-0.03	132	-2.51	10161	-0.19	138	-17.1	14815
LDHB	-1.81	6.09E-08	-0.16	126	-12.45	14341	0.03	94	1.26	7638	-0.29	126	-21.77	15625
SH3GL2	-1.05	0.039297256	-0.17	124	-12.45	14342	-0.08	123	-5.75	12059	-0.15	127	-11.37	13365
TMEFF2	-1.06	2.22E-05	-0.13	145	-12.46	14343	-0.08	104	-4.22	11217	-0.1	135	-8.23	12326
WFDC3	-1.18	2.07E-14	-0.26	98	-12.46	14344	-0.23	125	-17.22	15622	-0.33	100	-16.65	14725
EI24	-1.41	1.96E-09	-0.19	115	-12.46	14345	-0.28	124	-20.46	16039	-0.24	123	-17.38	14861
GLRX	-1.45	3.79E-07	-0.16	130	-12.47	14346	-0.18	128	-13.73	14929	-0.1	119	-6.61	11698
HCN4	-1.31	4.52E-06	-0.18	120	-12.47	14347	-0.2	131	-16.51	15513	-0.18	120	-12.37	13662
TMEM63A	-1.31	2.22E-06	-0.21	110	-12.47	14348	-0.11	111	-6.54	12431	-0.15	122	-10.91	13222
MAMSTR	-1.07	0.000992244	-0.2	112	-12.47	14349	-0.19	94	-8.36	13265	-0.14	102	-7.19	11919
TPPP3	-1.31	3.89E-11	-0.18	120	-12.48	14350	0	104	0.05	8428	-0.24	104	-12.69	13760
MADD	-1.11	1.12E-09	-0.16	129	-12.48	14351	-0.17	120	-12.08	14530	-0.24	123	-17.39	14862
ATP5J	-1.2	0.000408215	-0.18	120	-12.48	14352	-0.21	93	-8.91	13472	-0.14	107	-7.77	12149
JAK2	-1.3	5.20E-08	-0.15	133	-12.49	14353	-0.04	114	-2.39	10076	-0.24	132	-19.7	15281
EIF4E2	-1.08	0.0171776	-0.27	97	-12.49	14354	0	100	-0.24	8633	-0.24	107	-13.81	14058
SIK3_t1	-1.23	4.09E-08	-0.14	141	-12.49	14355	NA	NA	NA	NA	NA	NA	NA	NA
CRYAA_t1	-1.36	0.001394463	-0.22	108	-12.5	14356	-0.27	150	-26.88	16410	NA	NA	NA	NA
RERGL	-1.33	3.81E-05	-0.24	103	-12.5	14357	-0.05	135	-4.08	11139	-0.18	135	-15.48	14475
RXRB	-1.06	0.006698653	-0.15	135	-12.5	14358	-0.14	140	-12.4	14616	-0.18	112	-11.28	13341
S100A8	-4.84	2.79E-26	-0.21	110	-12.51	14359	-0.17	87	-6.35	12351	-0.36	84	-12.39	13664
CYP4F12	-2.07	3.42E-14	-0.34	87	-12.52	14360	-0.33	83	-11.05	14259	-0.26	108	-15.32	14445
CD180	-1.15	2.10E-11	-0.16	130	-12.52	14361	0.03	123	2.52	6814	-0.28	127	-21.7	15618
CREM_t3	-1.61	4.65E-13	-0.16	126	-12.52	14362	-0.17	135	-14.43	15084	-0.16	148	-15.62	14498
DOK2	-1.35	4.01E-16	-0.16	127	-12.53	14363	-0.14	114	-8.98	13493	-0.34	115	-22.06	15665
GSTK1	-1.21	0.000416264	-0.23	105	-12.53	14364	-0.24	118	-16.19	15456	-0.16	100	-7.76	12145
RASSF5_t1	-1.05	0.000574563	-0.13	147	-12.54	14365	-0.08	148	-7.53	12886	-0.24	150	-24.21	15891
ECI2	-1.34	0.000278523	-0.18	120	-12.54	14366	-0.19	108	-11.04	14256	-0.09	125	-6.97	11830
DNAH2	-1.36	1.39E-14	-0.23	104	-12.54	14367	-0.14	100	-7.23	12749	-0.29	128	-22.64	15732
TAPT1	-1.21	1.04E-07	-0.14	140	-12.54	14368	-0.24	122	-17.19	15616	-0.09	143	-8.77	12516
LSP1	-1.49	7.77E-07	-0.14	142	-12.54	14369	0	122	-0.22	8621	-0.25	128	-19.62	15267
AMOT	-1.06	0.369059143	-0.13	149	-12.55	14370	-0.07	140	-6.55	12436	-0.1	142	-9.27	12677
JMJD1C_t1	-1.32	5.54E-11	-0.15	133	-12.55	14371	-0.12	138	-10.56	14088	NA	NA	NA	NA
CADM2	-1.01	0.395427256	-0.14	140	-12.56	14372	-0.08	134	-6.51	12416	-0.03	152	-2.65	10012
SIGLEC5_t1	-1.12	1.25E-08	-0.17	124	-12.56	14373	NA	NA	NA	NA	-0.3	126	-22.74	15749
SNAP23	-1.11	1.55E-05	-0.15	134	-12.56	14374	-0.09	133	-7.2	12736	-0.09	147	-9.02	12591
PISD	-1.18	5.40E-07	-0.16	127	-12.56	14375	-0.03	126	-2.62	10230	-0.26	116	-16.85	14770
DHX58	-1.46	7.11E-11	-0.21	109	-12.57	14376	-0.24	126	-18.29	15771	-0.33	126	-25.08	15961
TNFSF13B	-1.66	2.14E-11	-0.17	126	-12.57	14377	-0.04	125	-3.1	10535	-0.29	135	-24.4	15908
SOD3	-2.05	1.90E-12	-0.2	112	-12.58	14378	-0.06	119	-3.93	11063	-0.24	111	-14.86	14343
FKRP	-1.13	2.46E-05	-0.14	137	-12.59	14379	-0.1	150	-10.04	13895	-0.29	131	-23.69	15849
SOX8_t1	-1.51	9.27E-13	-0.17	122	-12.59	14380	NA	NA	NA	NA	-0.21	126	-15.65	14507
TNS1	-1.03	0.15654116	-0.14	138	-12.61	14381	-0.02	140	-1.82	9700	-0.07	140	-6.17	11510
TPST2	-1.18	0.002119819	-0.17	123	-12.61	14382	-0.08	146	-7.87	13060	-0.08	117	-5.21	11121
SLC35A1_t1	-1.28	5.27E-08	-0.19	116	-12.61	14383	NA	NA	NA	NA	-0.23	129	-17.79	14943
APOL2_t1	-1.13	6.19E-05	-0.17	124	-12.61	14384	-0.06	135	-4.96	11633	NA	NA	NA	NA
PTK6	-1.33	8.65E-07	-0.21	110	-12.61	14385	-0.15	131	-12.54	14654	-0.23	122	-16.46	14675
RBM7	-1.37	8.96E-10	-0.17	125	-12.61	14386	-0.14	137	-11.76	14439	-0.16	131	-12.81	13794
DEFA5	-1.53	4.48E-16	-0.26	98	-12.61	14387	-0.3	94	-13.17	14786	-0.21	112	-13.02	13845
ARMCX2	-1.53	2.66E-09	-0.16	128	-12.61	14388	-0.02	111	-1.14	9244	-0.3	124	-21.86	15637
PSD3	-1.36	0.000428809	-0.12	154	-12.62	14389	-0.25	160	-27.96	16436	0.01	144	1.15	7947
ART5	-1.04	0.178973385	-0.18	120	-12.62	14390	-0.09	84	-2.95	10449	-0.14	121	-9.93	12906
SCTR	-1.36	1.00E-11	-0.33	88	-12.62	14391	-0.19	99	-9.17	13580	-0.34	112	-20.94	15489
GRIA3	-1.03	0.140274156	-0.14	142	-12.62	14392	-0.18	139	-15.92	15398	-0.08	123	-6.12	11484
EDNRA	-1.18	6.21E-05	-0.12	154	-12.63	14393	0.03	141	2.74	6649	-0.09	123	-6.51	11650
DGCR6	-1.36	1.93E-08	-0.21	109	-12.64	14394	-0.23	112	-14.27	15047	-0.24	120	-16.86	14772
NUMA1	-1.1	0.000453782	-0.15	132	-12.64	14395	-0.14	116	-9.36	13643	-0.19	142	-17.71	14927
PLEK	-1.71	7.71E-12	-0.17	124	-12.64	14396	-0.02	148	-1.83	9711	-0.31	137	-26.66	16101
TTPA	-1.3	0.002212351	-0.13	147	-12.64	14397	-0.1	127	-7.53	12887	-0.03	128	-2.24	9815
MXRA8	-1.16	4.75E-06	-0.18	121	-12.64	14398	-0.02	143	-1.4	9434	-0.24	122	-16.92	14789
CACNG3	-1.17	6.88E-10	-0.16	127	-12.65	14399	-0.13	118	-9.02	13515	-0.13	115	-8.21	12318
SERPINA3	-1.95	4.91E-08	-0.21	109	-12.65	14400	-0.29	101	-14.74	15155	-0.23	100	-11.46	13386
NUDT16L1	-1.1	0.005397276	-0.18	121	-12.65	14401	-0.11	125	-8.6	13363	-0.17	106	-9.73	12820
SHB_t1	-1.19	1.09E-05	-0.16	127	-12.65	14402	-0.23	148	-22.29	16197	NA	NA	NA	NA
NPTX2	-1.15	0.062428848	-0.17	125	-12.65	14403	-0.17	113	-10.43	14042	-0.11	125	-8.05	12248
ARSF	-1.23	1.62E-06	-0.25	101	-12.66	14404	-0.15	104	-8.1	13153	-0.25	124	-18.38	15060
SYNE1_t1	-1.41	5.66E-14	-0.23	104	-12.66	14405	-0.09	114	-5.51	11923	-0.26	128	-20.09	15345
SLC1A7	-1.36	0.00796491	-0.17	124	-12.66	14406	0.01	123	0.94	7840	-0.26	115	-16.99	14799
FADS6	1.01	0.716425042	-0.16	127	-12.66	14407	-0.1	127	-7.46	12854	-0.08	140	-7.56	12051
CASP14	-1.05	0.000440758	-0.16	128	-12.67	14408	-0.23	128	-18.03	15739	-0.12	129	-9.19	12652
PROX1	-1.45	3.02E-06	-0.12	152	-12.67	14409	-0.21	136	-17.77	15705	-0.1	152	-9.79	12847
RRBP1	-1.33	6.08E-07	-0.23	104	-12.68	14410	-0.22	140	-19.59	15942	-0.19	100	-9.42	12727
OPN1MW	-1.05	0.001076309	-0.18	120	-12.68	14411	-0.11	106	-6.01	12189	-0.09	108	-5.24	11132
TBX21	-1.52	2.27E-10	-0.22	107	-12.69	14412	-0.08	114	-5.21	11782	-0.34	134	-28.59	16234
PIK3R5	-1.15	9.45E-14	-0.18	120	-12.69	14413	-0.12	124	-8.65	13375	-0.28	116	-18.67	15116
PPP2R4	-1.1	0.00122008	-0.21	111	-12.69	14414	-0.09	120	-5.97	12167	-0.24	139	-21.53	15598
ATP2A3_t2	-1.43	3.72E-11	-0.18	120	-12.69	14415	-0.07	137	-6.1	12241	-0.34	120	-24.04	15874
Sep-04	-1.52	1.20E-05	-0.2	114	-12.7	14416	-0.17	104	-9.08	13550	-0.21	103	-10.9	13220
CTTNBP2	-1.55	4.71E-08	-0.18	121	-12.7	14417	-0.08	112	-5	11657	-0.23	145	-21.4	15570
FUZ_t1	-1.24	7.64E-06	-0.18	119	-12.7	14418	NA	NA	NA	NA	-0.33	92	-13.66	14014
PTPRS_t2	-1.04	0.08297997	-0.16	131	-12.72	14419	0.01	149	0.78	7953	-0.13	120	-8.88	12556
ZNF80	-1.19	1.67E-12	-0.15	135	-12.73	14420	-0.27	120	-19.05	15876	-0.15	146	-14.23	14165
SLC6A16	-1.4	8.40E-13	-0.19	117	-12.73	14421	-0.22	136	-18.91	15855	-0.22	117	-15.04	14391
EPN1	-1.2	2.22E-11	-0.21	110	-12.74	14422	-0.3	118	-20.54	16046	-0.28	119	-19.62	15268
CRTAC1	-1.14	0.000877427	-0.18	122	-12.74	14423	-0.13	130	-10.78	14169	-0.29	115	-18.93	15159
RCAN2	-1.98	5.50E-10	-0.13	146	-12.75	14424	-0.03	135	-2.84	10369	-0.23	139	-20.17	15364

APOBEC3A	-1.62	1.30E-13	-0.25	102	-12.75	14425	-0.15	128	-11.46	14362	-0.26	132	-20.99	15501
LPPR5	-1.01	0.192479821	-0.14	142	-12.75	14426	-0.1	132	-8.32	13253	-0.12	143	-10.74	13168
S100B	-1.2	8.23E-06	-0.11	164	-12.75	14427	-0.01	122	-0.81	9018	-0.25	133	-21.15	15527
MAP3K14	-1.21	0.001161132	-0.21	112	-12.76	14428	-0.16	140	-14.61	15124	-0.21	147	-20.54	15418
SEMA6A	-1.26	7.68E-11	-0.16	131	-12.76	14429	-0.04	132	-3.03	10488	-0.18	135	-15.64	14504
CEBPB_t1	-1.29	1.21E-10	-0.21	111	-12.76	14430	-0.19	112	-11.52	14377	NA	NA	NA	NA
SNHG5	-1.13	0.01695118	-0.13	152	-12.76	14431	-0.03	114	-1.8	9693	-0.09	112	-5.62	11282
ART3	-1.05	0.008544574	-0.12	160	-12.76	14432	-0.02	116	-1.55	9520	-0.18	120	-12.48	13692
GMFG	-2.35	3.28E-23	-0.24	104	-12.76	14433	-0.14	88	-5.38	11860	-0.42	94	-18.62	15102
CSF1R	-2.5	1.62E-21	-0.24	104	-12.77	14434	-0.18	126	-13.58	14887	-0.4	130	-31.74	16354
CFI	-2.74	3.06E-16	-0.24	103	-12.77	14435	-0.26	98	-12.45	14625	-0.25	106	-13.96	14102
SLC5A5	-1.02	0.140555187	-0.15	136	-12.79	14436	-0.13	127	-10.03	13894	-0.12	131	-9.74	12829
EIF5	-1.24	3.63E-05	-0.12	156	-12.79	14437	-0.23	144	-21.93	16159	-0.01	148	-1.01	9208
TTC38	-1.59	6.90E-11	-0.28	95	-12.79	14438	-0.3	124	-22.2	16188	-0.29	125	-21.5	15590
WT1	1.02	0.412260574	-0.14	139	-12.8	14439	-0.08	138	-7.45	12852	-0.1	140	-8.66	12479
HOXB5	-1.19	0.000150992	-0.2	113	-12.8	14440	-0.08	120	-5.77	12075	-0.26	111	-16.11	14612
ALLC	-1.67	2.59E-24	-0.34	88	-12.8	14441	-0.26	111	-15.6	15322	-0.36	101	-18.39	15061
SRPX2	-1.53	1.67E-07	-0.18	120	-12.81	14442	-0.01	106	-0.77	8990	-0.2	95	-9.13	12639
SIX2	-1.24	7.63E-11	-0.16	128	-12.81	14443	-0.24	123	-17.73	15702	-0.2	133	-16.89	14782
NEBL	1.17	0.002797438	-0.12	156	-12.82	14444	0	141	0.44	8181	-0.12	126	-9.04	12599
CLDN2	-1.08	0.223632109	-0.18	120	-12.83	14445	-0.11	122	-7.9	13072	-0.16	135	-13.2	13898
SLC2A3_t1	-1.32	1.03E-06	-0.13	149	-12.83	14446	NA	NA	NA	NA	-0.14	152	-14.15	14149
MFSD7	-1.67	1.56E-17	-0.28	96	-12.84	14447	-0.16	92	-6.9	12603	-0.32	101	-16.37	14659
LIPC	-2.86	1.17E-16	-0.37	85	-12.84	14448	-0.26	107	-14.67	15140	-0.32	67	-5.48	11232
LGR4_t1	-1.21	2.89E-05	-0.14	145	-12.85	14449	-0.14	120	-10.06	13904	NA	NA	NA	NA
RRP8	-1.16	1.92E-06	-0.17	126	-12.85	14450	-0.21	124	-15.86	15378	-0.14	110	-8.37	12385
NUP98_t2	-1.28	2.80E-08	-0.15	134	-12.86	14451	-0.14	122	-10.38	14029	-0.15	121	-10.57	13105
SLFN11	-1.7	9.02E-12	-0.16	128	-12.86	14452	-0.04	113	-2.3	10026	-0.31	131	-24.97	15955
CPEB4_t1	-1.22	4.52E-08	-0.15	136	-12.86	14453	NA	NA	NA	NA	NA	NA	NA	NA
CSH2	-1.23	3.29E-12	-0.26	100	-12.86	14454	-0.27	136	-23.43	16272	-0.17	122	-12.45	13684
NELF_t1	-1.07	0.009316483	-0.16	132	-12.87	14455	NA	NA	NA	NA	-0.16	121	-11.25	13327
PMEPA1	-1.36	0.012625375	-0.14	142	-12.87	14456	-0.02	149	-1.63	9574	-0.23	148	-22.68	15740
SUCLG1	-1.33	1.08E-07	-0.17	127	-12.87	14457	-0.19	108	-11.03	14250	-0.18	128	-13.66	14012
LCP2	-1.93	2.35E-22	-0.18	120	-12.87	14458	-0.1	125	-7.64	12946	-0.37	127	-28.35	16212
CFP	-6.24	3.37E-51	-0.38	84	-12.88	14459	-0.28	104	-15.36	15281	-0.47	88	-17.89	14960
TGM2_t2	-1.58	1.03E-08	-0.17	126	-12.88	14460	-0.13	138	-11.77	14441	-0.2	137	-17.58	14900
CYTH1	-1.05	0.2478189	-0.19	118	-12.88	14461	0.01	120	0.74	7976	-0.16	126	-12.21	13613
ORM2	-2.08	7.69E-10	-0.24	104	-12.89	14462	-0.3	93	-12.81	14712	-0.25	117	-16.66	14727
CISH_t1	-1.52	3.82E-07	-0.2	114	-12.89	14463	-0.11	121	-8.05	13131	NA	NA	NA	NA
FAM54B	-1.2	4.71E-08	-0.2	114	-12.89	14464	-0.21	134	-17.48	15667	-0.18	126	-13.52	13982
PTGIS_t1	-1.09	5.99E-11	-0.13	146	-12.9	14465	NA	NA	NA	NA	-0.08	123	-5.54	11254
GPLD1	-1.46	0.000376154	-0.15	135	-12.9	14466	-0.19	143	-17.89	15720	-0.06	140	-5.28	11148
ITPKB	-1.14	1.82E-06	-0.14	142	-12.91	14467	-0.08	134	-6.8	12564	-0.21	152	-21.53	15597
RGS16_t1	-1.18	0.04529834	-0.18	120	-12.91	14468	NA	NA	NA	NA	-0.27	124	-20.01	15333
KLRD1	-1.35	7.09E-11	-0.15	138	-12.91	14469	-0.09	135	-7.75	13007	-0.36	159	-38.79	16476
SPIRE1	-1.39	1.32E-07	-0.15	134	-12.91	14470	0	143	0.33	8252	-0.17	149	-16.36	14658
SLC9A5	-1.13	4.06E-11	-0.18	121	-12.91	14471	-0.12	135	-10.28	13997	-0.15	121	-10.98	13254
PHF17_t2	-1.34	5.69E-07	-0.15	139	-12.92	14472	-0.15	140	-13.36	14842	NA	NA	NA	NA
ADPRM	-1.06	0.156527813	-0.17	124	-12.92	14473	-0.08	104	-4.27	11253	-0.12	121	-8.78	12519
ACADVL	-1.86	3.98E-25	-0.27	98	-12.93	14474	-0.29	124	-21.27	16120	-0.34	100	-16.84	14768
TPCN2	-1.09	0.018333199	-0.16	132	-12.93	14475	-0.09	159	-9.39	13654	-0.09	135	-7.96	12224
DZIP1	-1.17	0.006157788	-0.15	135	-12.93	14476	0.11	140	10.23	2783	-0.22	138	-19.62	15269
MRGPRF_t1	-1.44	3.67E-17	-0.16	132	-12.93	14477	-0.17	107	-9.9	13844	NA	NA	NA	NA
SYT7	1	0.93424274	-0.17	124	-12.94	14478	-0.17	133	-13.85	14957	-0.1	127	-7.63	12088
RBMS1_t2	-1.44	8.85E-08	-0.13	147	-12.94	14479	0.05	129	3.95	5930	-0.19	147	-18.48	15077
FGR_t1	-1.08	5.01E-09	-0.18	120	-12.94	14480	NA	NA	NA	NA	-0.29	114	-18.6	15098
CETP	-4.82	3.58E-42	-0.38	84	-12.95	14481	-0.32	100	-15.83	15368	-0.42	93	-18.23	15027
LHFP	-1.69	1.37E-06	-0.13	146	-12.95	14482	0.01	124	0.7	7997	-0.22	138	-19.02	15177
ICOS	-1.26	7.37E-07	-0.14	140	-12.95	14483	-0.12	127	-9.04	13528	-0.23	131	-18.68	15119
SUSD2	-1.28	0.000553855	-0.12	161	-12.95	14484	-0.11	142	-9.72	13783	-0.17	101	-8.9	12559
TENC1	-1.56	1.09E-16	-0.28	96	-12.96	14485	-0.17	115	-11.2	14301	-0.36	117	-24.2	15890
TINAGL1_t1	-1.21	1.39E-18	-0.24	103	-12.96	14486	NA	NA	NA	NA	-0.3	119	-20.74	15449
ABCB4	-1.9	1.14E-07	-0.27	98	-12.96	14487	-0.24	89	-9.27	13613	-0.12	84	-4.2	10717
SLC40A1	-1.5	4.69E-07	-0.14	140	-12.96	14488	-0.21	130	-16.51	15511	-0.08	129	-6.23	11540
BTK	-1.37	2.02E-13	-0.19	120	-12.96	14489	-0.07	108	-4.07	11132	-0.35	124	-26.17	16053
ADORA3_t2	-1.42	1.05E-18	-0.2	115	-12.96	14490	-0.1	111	-6.29	12320	-0.33	111	-19.96	15323
CACNA1E	-1.31	2.83E-06	-0.15	135	-12.99	14491	-0.11	151	-10.89	14204	-0.12	140	-11.18	13302
MESDC1	-1.23	7.99E-06	-0.16	131	-12.99	14492	-0.01	125	-0.96	9120	-0.21	138	-18.76	15131
TTTY21	-1.03	0.000378466	-0.17	125	-12.99	14493	-0.08	122	-5.63	11989	-0.13	99	-6.21	11525
CDK3	-1.14	1.25E-06	-0.23	106	-13	14494	-0.22	144	-20.32	16021	-0.23	105	-12.76	13779
CAMKK2_t2	-1.04	0.32503086	-0.17	126	-13	14495	-0.03	125	-2.11	9909	-0.16	123	-11.63	13437
EDAR	-1.04	0.035173483	-0.16	133	-13	14496	-0.06	132	-5.15	11741	-0.06	153	-6.15	11497
TBC1D10A	-1.32	1.84E-15	-0.26	100	-13.01	14497	-0.2	126	-15.24	15260	-0.33	126	-24.7	15936
OTOP3	-1.26	1.35E-13	-0.22	108	-13.01	14498	-0.28	115	-18.5	15799	-0.18	100	-9.2	12655
PHLDA1	-1.67	9.15E-14	-0.17	126	-13.01	14499	-0.16	151	-15.78	15359	-0.22	146	-21.43	15574
HPR	-3.33	1.92E-15	-0.27	98	-13.01	14500	-0.34	84	-11.52	14381	-0.33	104	-17.97	14973
CCDC89	-1.07	1.31E-07	-0.19	120	-13.02	14501	-0.09	114	-5.62	11985	-0.12	151	-12.17	13597
CCND1	-1.33	5.08E-08	-0.16	131	-13.02	14502	-0.16	139	-14.46	15097	-0.21	151	-21.13	15526
KANK4	-1.69	9.71E-08	-0.17	127	-13.02	14503	-0.17	135	-14.11	15012	-0.16	153	-16.14	14616
FETUB	-2.86	3.12E-11	-0.24	105	-13.03	14504	-0.41	120	-28.45	16445	-0.17	120	-12.2	13609
JUNB	-3.26	2.87E-23	-0.23	107	-13.04	14505	-0.18	122	-12.76	14705	-0.36	110	-21.47	15582
ATG2A	-1.61	2.15E-19	-0.26	101	-13.04	14506	-0.24	119	-16.87	15573	-0.33	120	-22.98	15774
ZC3H7B	-1.16	0.000147816	-0.17	126	-13.04	14507	-0.08	130	-6.57	12446	-0.14	120	-9.8	12853
SPAG9	-1.17	0.001257372	-0.13	152	-13.04	14508	-0.04	140	-3.54	10832	-0.01	129	-0.99	9198
HAMP	-8.61	5.80E-40	-0.27	98	-13.04	14509	-0.28	88	-10.7	14133	-0.39	90	-15.49	14478
CES2_t2	-1.77	2.32E-09	-0.13	152	-13.04	14510	-0.33	149	-32.65	16480	-0.2	112	-12.53	13707
DNAJB12_t2	-1.12	0.000124884	-0.16	132	-13.05	14511	-0.13	137	-10.94	14218	NA	NA	NA	NA
NTRK3_t2	-1.11	9.66E-09	-0.2	116	-13.05	14512	-0.06	124	-4.39	11310	-0.25	90	-9.82	12860
ZDHHC8	-1.11	4.31E-06	-0.23	108	-13.05	14513	-0.21	139	-19.05	15875	-0.21	120	-15.01	14378
AMOTL1_t1	-1.58	2.05E-11	-0.15	138	-13.06	14514	NA	NA	NA	NA	NA	NA	NA	NA
S100Z	-1.1	1.24E-09	-0.18	124	-13.06	14515	-0.06	105	-3.25	10633	-0.24	120	-16.46	14674
SPSB3_t1	-1.41	1.48E-18	-0.26	100	-13.06	14516	NA	NA	NA	NA	NA	NA	NA	NA
FPR1	-2.78	4.04E-26	-0.2	114	-13.06	14517	-0.14	108	-8.24	13210	-0.35	91	-14.5	14242
EVI2B	-1.7	7.19E-19	-0.2	115	-13.06	14518	-0.08	112	-5.18	11763	-0.33	113	-20.58	15424
RNF146	-1.2	2.04E-05	-0.17	126	-13.07	14519	-0.03	139	-2.69	10271	-0.2	128	-15.37	14453
CCNL1	-1.75	1.03E-13	-0.17	126	-13.07	14520	-0.12	120	-8.72	13399	-0.25	134	-21.18	15533
CYBB	-2.37	5.52E-18	-0.16	130	-13.09	14521	-0.1	140	-8.99	13496	-0.3	147	-29.3	16268
IL28RA_t2	-1.19	3.02E-05	-0.18	123	-13.09	14522	-0.23	129	-18.01	15735	-0.21	152	-21.08	15517
SCHIP1	-1.27	0.001537773	-0.17	125	-13.1	14523	-0.07	116	-4.84	11561	-0.1	120	-6.74	11754
CRYZ	-1.3	0.002887997	-0.16	132	-13.1	14524	-0.07	126	-5.58	11959	-0.09	135	-7.61	12077
MPRIP_t1	-1.17	7.83E-05	-0.14	144	-13.11	14525	NA	NA	NA	NA	NA	NA	NA	NA
RGL4	-1.96	3.28E-18	-0.27	98	-13.11	14526	-0.12	98	-5.8	12093	-0.4	115	-26.2	16060
KCNAB3	-1.08	4.22E-13	-0.18	123	-13.11	14527	-0.21	129	-16.95	15583	-0.22	125	-16.53	14691
CALD1_t1	-1.27	0.008698758	-0.18	122	-13.11	14528	-0.09	135	-7.83	13043	NA	NA	NA	NA
ALKBH7	-1.44	1.68E-11	-0.23	107	-13.11	14529	-0.25	124	-18.68	15821	-0.27	155	-27.91	16181
LRRFIP1	-1.22	0.000419989	-0.14	145	-13.12	14530	-0.08	128	-6.03	12202	-0.1	134	-8.35	12381

ZNF274	-1.16	5.26E-05	-0.19	120	-13.12	14531	-0.08	126	-6.25	12305	-0.24	121	-16.8	14753
NET1	-1.21	0.007120804	-0.16	132	-13.13	14532	-0.12	138	-10.6	14107	-0.03	131	-2.32	9854
CRYM	-1.24	0.03951325	-0.15	135	-13.13	14533	-0.2	129	-15.97	15409	0.05	111	2.88	7001
ACP2	-1.15	4.00E-07	-0.18	125	-13.13	14534	-0.21	129	-16.63	15533	-0.22	148	-21.73	15623
PSAT1	-2.1	1.53E-11	-0.19	120	-13.14	14535	-0.15	127	-11.4	14346	-0.15	122	-11.01	13261
SAMSN1	-2.3	4.66E-19	-0.19	120	-13.14	14536	-0.09	130	-7.33	12804	-0.3	136	-26.16	16051
KLRF1	-1.44	8.29E-19	-0.2	115	-13.15	14537	-0.11	138	-9.72	13785	-0.25	123	-18.15	15007
ARHGAP30_t1	-1.63	2.28E-14	-0.16	132	-13.16	14538	-0.06	124	-4.18	11193	-0.37	111	-22.29	15699
AASS	-1.83	8.08E-09	-0.17	129	-13.17	14539	-0.21	138	-18.23	15763	-0.13	141	-11.44	13380
GSDMB	-1.4	1.85E-07	-0.22	110	-13.17	14540	-0.19	104	-10.53	14078	-0.21	92	-9	12590
PGA3_t1	-1.46	7.23E-17	-0.25	103	-13.17	14541	NA	NA	NA	NA	-0.3	81	-9.21	12657
ALDH7A1	-1.38	1.66E-05	-0.14	141	-13.17	14542	-0.31	131	-25.15	16350	-0.09	119	-6.02	11445
BNIP2	-1.48	1.88E-10	-0.15	136	-13.17	14543	-0.17	132	-13.66	14908	-0.14	131	-11.59	13427
ZFP36	-3.62	2.29E-31	-0.24	104	-13.17	14544	-0.2	130	-15.66	15337	-0.37	140	-33.19	16397
CGNL1	-1.38	0.000722655	-0.18	122	-13.17	14545	-0.2	150	-20.31	16020	-0.03	153	-3.08	10216
ATP1B2	-1.3	8.61E-13	-0.18	122	-13.17	14546	-0.14	146	-13.01	14758	-0.23	139	-20.35	15386
ETNK2_t1	-1.28	1.44E-05	-0.18	124	-13.19	14547	NA	NA	NA	NA	-0.1	120	-6.77	11763
RNF13	-1.17	5.38E-05	-0.16	132	-13.19	14548	-0.15	119	-10.09	13911	-0.14	117	-9.66	12795
CUTC	-1.27	2.95E-08	-0.18	125	-13.19	14549	-0.19	104	-10.26	13987	-0.16	120	-10.96	13243
SPATA18	-1.11	6.95E-09	-0.15	136	-13.2	14550	-0.17	151	-17.03	15595	-0.14	141	-12.62	13738
LILRA1	-1.15	4.97E-11	-0.17	128	-13.2	14551	-0.16	155	-17.07	15603	-0.18	114	-11.56	13419
PALM2	-1.12	5.94E-09	-0.12	160	-13.21	14552	-0.15	149	-14.55	15114	-0.04	158	-3.78	10526
GK_t2	-1.46	1.17E-10	-0.14	145	-13.21	14553	-0.22	134	-18.73	15827	-0.23	141	-21.22	15542
LRRN1	-1.2	5.93E-06	-0.21	114	-13.22	14554	-0.09	116	-5.84	12108	-0.27	130	-21.29	15555
F2RL2	1.01	0.394625424	-0.17	127	-13.23	14555	0.09	140	8.41	3539	-0.04	122	-2.95	10161
FAM18B1	-1.45	6.63E-09	-0.17	128	-13.24	14556	-0.13	130	-10.24	13975	-0.17	131	-13.56	13986
NCALD	-1.32	3.44E-07	-0.18	123	-13.24	14557	-0.12	132	-10.21	13959	-0.2	131	-16.03	14595
APOL6	-1.2	2.14E-09	-0.14	145	-13.24	14558	-0.14	142	-13.3	14827	-0.12	137	-10.02	12931
PPARGC1A_t1	-2.74	4.66E-13	-0.15	141	-13.25	14559	-0.28	134	-23.36	16265	NA	NA	NA	NA
FLNA	-1.39	6.29E-08	-0.22	111	-13.25	14560	-0.04	101	-1.86	9734	-0.32	122	-22.7	15744
FMOD	-2.99	1.66E-13	-0.19	120	-13.26	14561	-0.07	131	-5.81	12096	-0.28	132	-22.68	15738
ALDH1A1	-1.42	0.000924559	-0.17	130	-13.27	14562	-0.09	106	-5.16	11752	-0.08	135	-7.15	11897
LIPG	-1.67	1.15E-07	-0.17	126	-13.29	14563	-0.18	126	-13.58	14885	-0.15	150	-14.89	14352
DEF6	-1.3	4.82E-07	-0.18	122	-13.29	14564	0	120	0.18	8349	-0.29	110	-17.17	14827
APP	-1.7	4.42E-14	-0.19	120	-13.29	14565	-0.19	116	-12.57	14661	-0.25	113	-15.5	14479
ZNF502	-1.29	6.08E-07	-0.14	144	-13.3	14566	0.03	123	2.07	7099	-0.11	120	-7.59	12065
PRPS1_t1	-1.13	9.71E-05	-0.14	144	-13.31	14567	NA	NA	NA	NA	NA	NA	NA	NA
LST1_t1	-2.13	6.31E-23	-0.25	104	-13.31	14568	-0.14	121	-9.67	13760	NA	NA	NA	NA
CDK9_t1	-1.11	2.73E-08	-0.19	121	-13.31	14569	-0.19	135	-15.83	15367	NA	NA	NA	NA
CSF3	-1.02	0.134073146	-0.19	120	-13.31	14570	-0.02	148	-1.49	9489	-0.09	130	-7.59	12064
LARP1B_t1	-1.51	1.90E-10	-0.17	129	-13.31	14571	-0.24	136	-20.75	16069	-0.12	121	-8.28	12354
STON1	-1.47	1.21E-06	-0.17	130	-13.31	14572	0.04	126	3.18	6394	-0.23	120	-16.23	14630
SNX22	-1.12	0.001475065	-0.18	124	-13.31	14573	-0.16	142	-14.81	15173	-0.17	132	-13.86	14065
PRELP	-1.74	3.43E-17	-0.15	136	-13.32	14574	-0.07	135	-6.05	12210	-0.23	128	-18.33	15051
DLC1_t1	-1.06	0.002145369	-0.19	122	-13.32	14575	-0.12	108	-7.07	12679	-0.04	123	-2.8	10087
PPP2R2D	-1.31	2.22E-18	-0.25	104	-13.32	14576	-0.31	126	-23.71	16288	-0.28	110	-16.81	14760
GAMT_t2	-1.59	1.07E-11	-0.16	133	-13.32	14577	-0.27	129	-21.51	16138	-0.21	136	-17.88	14957
TSPAN18	-1.13	4.65E-08	-0.2	115	-13.32	14578	-0.1	134	-8.12	13167	-0.14	151	-14.58	14261
HRC	-1.08	0.002290691	-0.28	98	-13.33	14579	-0.13	98	-6.21	12293	-0.17	97	-8.15	12289
SCML4	-1.03	0.022860309	-0.13	153	-13.33	14580	-0.04	126	-3.31	10677	-0.26	149	-26.11	16041
FAM129C	-1.11	3.84E-09	-0.19	120	-13.34	14581	-0.1	123	-6.95	12623	-0.14	148	-13.35	13938
CYTIP	-2.49	4.73E-17	-0.16	133	-13.35	14582	-0.07	115	-4.45	11356	-0.33	109	-19.29	15220
DMD_t2	-1.19	9.74E-09	-0.16	133	-13.35	14583	-0.1	130	-7.81	13036	-0.17	113	-10.72	13157
GFOD2	-1.52	5.16E-13	-0.21	115	-13.35	14584	-0.33	101	-16.76	15559	-0.3	114	-18.94	15162
TGFBR2_t1	-1.35	7.46E-07	-0.17	127	-13.36	14585	NA	NA	NA	NA	-0.19	158	-20.66	15435
ITGB7	-1.21	0.000247263	-0.23	108	-13.36	14586	-0.09	104	-4.88	11583	-0.22	117	-14.89	14351
KIAA0247	-1.25	8.61E-07	-0.15	138	-13.36	14587	-0.07	141	-6.64	12481	-0.28	159	-30.57	16319
N4BP2L2	-1.33	5.70E-05	-0.19	120	-13.36	14588	-0.09	128	-7.35	12816	-0.26	159	-28.41	16218
PDSS2	-1.27	2.51E-07	-0.15	142	-13.37	14589	-0.17	123	-12.55	14656	-0.11	156	-11.6	13428
AKAP7	-1.34	8.08E-06	-0.15	142	-13.37	14590	-0.01	114	-0.54	8846	-0.23	151	-23.52	15834
CCNT1	-1.01	0.521936305	-0.14	145	-13.37	14591	-0.17	132	-13.69	14917	-0.09	142	-8.19	12309
LILRB3	-2.2	1.05E-18	-0.17	128	-13.38	14592	-0.04	135	-3.44	10757	-0.34	123	-24.51	15915
UGT1A8	-1.58	2.84E-05	-0.16	133	-13.38	14593	-0.14	106	-7.86	13057	-0.1	120	-7	11846
RNF186	-1.01	0.879506396	-0.15	142	-13.38	14594	-0.09	103	-5	11658	-0.09	147	-8.38	12389
FHOD3	-1.21	0.057743488	-0.19	120	-13.38	14595	0	109	-0.25	8639	-0.21	110	-12.33	13655
FNDC4	-2.09	2.52E-18	-0.27	100	-13.39	14596	-0.23	120	-16.03	15428	-0.3	120	-20.77	15461
NEFH	-1.49	6.06E-10	-0.18	124	-13.39	14597	-0.07	126	-5.23	11791	-0.32	115	-20.84	15476
LEFTY1	-1.27	0.001172244	-0.15	142	-13.39	14598	-0.04	104	-2.34	10046	-0.24	130	-19.18	15203
TIMP3	-1.39	4.31E-06	-0.17	131	-13.39	14599	-0.05	142	-4.37	11296	-0.2	134	-16.58	14707
MPP1	-1.54	8.69E-14	-0.2	116	-13.39	14600	-0.18	112	-11.34	14328	-0.31	109	-18.29	15042
ALOX5	-1.96	5.73E-15	-0.19	119	-13.4	14601	-0.05	113	-2.87	10390	-0.32	129	-25.17	15968
TRPV6	-1.25	4.36E-06	-0.22	112	-13.4	14602	-0.19	108	-11.21	14304	-0.33	117	-21.82	15631
AGBL2	-1.11	0.278065294	-0.16	133	-13.4	14603	-0.13	138	-11.04	14252	-0.03	138	-2.23	9806
CELF2_t1	-1.51	2.07E-07	-0.15	142	-13.41	14604	0.01	158	1.19	7681	-0.27	134	-22.37	15707
COL4A3_t1	-1.2	3.17E-05	-0.15	141	-13.41	14605	NA	NA	NA	NA	-0.2	151	-19.96	15320
UGT2B17_t1	-2.83	1.77E-11	-0.18	124	-13.41	14606	NA	NA	NA	NA	-0.12	131	-9.93	12908
EXOC3L4	-1.81	2.76E-17	-0.29	97	-13.42	14607	-0.24	124	-17.79	15712	-0.35	99	-17.19	14830
DGCR6L	-1.36	4.35E-09	-0.23	109	-13.42	14608	-0.23	112	-14.02	14999	-0.23	120	-16.27	14640
FCER1A	-2.22	2.30E-18	-0.21	114	-13.43	14609	-0.11	106	-6.07	12227	-0.26	113	-16.08	14605
CES1	-1.97	2.78E-07	-0.24	105	-13.44	14610	-0.25	74	-5.92	12143	-0.29	112	-18.14	15005
CHST4	-2.13	1.19E-10	-0.17	127	-13.44	14611	-0.17	115	-10.83	14187	-0.31	116	-20.17	15363
PMPCA	-1.4	3.65E-13	-0.25	103	-13.45	14612	-0.14	123	-10.42	14041	-0.28	124	-20.37	15393
SLC35G1	-1.09	0.007207561	-0.19	120	-13.45	14613	-0.17	134	-13.91	14973	-0.08	139	-7.39	11993
CCL3	-2.61	1.07E-21	-0.22	110	-13.45	14614	-0.1	109	-5.98	12173	-0.36	115	-23.15	15790
GCSH	-1.52	4.97E-08	-0.2	118	-13.45	14615	-0.17	135	-14.04	15001	-0.13	123	-9.46	12745
OSBPL5	-1.63	7.14E-12	-0.22	111	-13.45	14616	-0.07	133	-5.42	11877	-0.33	118	-22.49	15719
NR1I3	-2.25	9.22E-09	-0.25	104	-13.45	14617	-0.25	132	-20.81	16072	-0.14	109	-8.55	12439
PXMP2	-2.01	2.46E-13	-0.31	94	-13.45	14618	-0.38	105	-20.7	16067	-0.24	120	-16.79	14752
RNF152	-2.02	2.56E-17	-0.17	130	-13.46	14619	-0.23	115	-14.88	15180	-0.22	120	-15.42	14460
CYB5A	-1.53	4.42E-06	-0.19	120	-13.46	14620	-0.24	115	-15.41	15291	-0.17	104	-9.4	12719
MASP1_t1	-1.36	7.95E-14	-0.19	121	-13.46	14621	-0.31	127	-23.75	16291	-0.27	126	-20.17	15362
CPB2	-2.42	3.01E-09	-0.22	112	-13.46	14622	-0.25	105	-13.67	14912	-0.22	108	-12.88	13810
GTF2F1	-1.06	0.066205636	-0.17	129	-13.47	14623	-0.07	135	-5.56	11949	-0.21	120	-14.84	14333
PRICKLE1	-1.44	8.95E-11	-0.21	114	-13.47	14624	-0.07	118	-4.62	11451	-0.28	136	-24.5	15913
SLC25A37	-1.35	5.96E-13	-0.19	120	-13.49	14625.5	-0.23	120	-16.39	15491	-0.21	136	-17.65	14914
FBXO39	-1.25	7.44E-10	-0.19	120	-13.49	14625.5	-0.24	108	-13.76	14937	-0.1	115	-6.51	11651
AES	-1.33	1.21E-16	-0.21	114	-13.5	14627	-0.11	121	-7.47	12860	-0.29	120	-20.46	15409
EXOSC6_t1	-1.27	1.29E-15	-0.16	137	-13.5	14628	NA	NA	NA	NA	-0.16	128	-12.48	13693
HMX2	-1.11	6.62E-07	-0.23	108	-13.5	14629	-0.12	157	-12.65	14677	-0.21	104	-11.29	13344
ESR1	-1.08	5.52E-05	-0.14	145	-13.5	14630	-0.23	141	-21.13	16105	-0.15	158	-15.72	14525
TXN2	-1.17	4.52E-06	-0.25	104	-13.51	14631	-0.15	109	-8.98	13492	-0.17	104	-9.27	12674
GCLC	-1.4	6.11E-06	-0.17	129	-13.51	14632	-0.18	127	-13.55	14881	-0.13	131	-10.17	12980
IGFBP6	-1.64	7.57E-11	-0.23	108	-13.51	14633	-0.1	107	-5.49	11906	-0.32	89	-12.39	13665
TMEM177	-1.08	0.177131934	-0.17	128	-13.52	14634	-0.09	130	-7.34	12809	-0.06	136	-5.32	11169
MEF2BNB-MEF	-1.66	2.10E-17	-0.2	119	-13.52	14635	-0.36	88	-13.49	14869	-0.27	88	-10.45	13075
CCL4	-3.19	1.36E-20	-0.25	105	-13.52	14636	-0.07	121	-4.86	11572	-0.39	120	-27.29	16152

CYP4F8	-2.01	4.05E-12	-0.35	89	-13.52	14637	-0.32	105	-17.35	15649	-0.25	129	-19.92	15310
CTSG	-1.98	9.74E-13	-0.23	109	-13.53	14638	-0.07	94	-3.18	10591	-0.28	91	-11.6	13429
UGT1A6	-1.64	0.000308348	-0.16	133	-13.53	14639	-0.09	106	-5.2	11775	-0.15	120	-10.61	13121
TRIM10_t2	-1.26	1.24E-05	-0.18	125	-13.54	14640	-0.21	133	-17.55	15676	-0.1	135	-8.12	12273
COX7A1	-1.68	2.54E-09	-0.19	123	-13.55	14641	-0.08	90	-3.26	10642	-0.3	96	-13.76	14043
DBC1	-1.19	6.98E-08	-0.24	107	-13.55	14642	-0.23	118	-15.62	15325	-0.32	119	-21.87	15639
IL23A	-1.3	3.44E-08	-0.22	111	-13.56	14643	-0.18	112	-11.16	14284	-0.3	117	-20.27	15378
TMED2_t1	-1.11	0.009373998	-0.17	131	-13.56	14644	-0.12	132	-9.74	13793	NA	NA	NA	NA
ZMYND12	-1.3	2.04E-07	-0.14	146	-13.57	14645	-0.2	119	-13.68	14913	-0.2	132	-16.04	14600
ZNF784	-1.28	9.22E-08	-0.19	121	-13.57	14646	-0.19	120	-13.59	14892	-0.21	154	-22.25	15697
GNMT	-4.16	1.23E-19	-0.3	95	-13.58	14647	-0.41	94	-18.24	15764	-0.29	100	-14.33	14196
COPS2	-1.09	0.129417824	-0.16	136	-13.58	14648	0.06	142	5.07	5296	-0.03	163	-3.7	10482
XAGE3	1.17	0.008594323	-0.15	142	-13.58	14649	-0.01	86	-0.48	8804	0.06	109	3.39	6723
PHKA2	-1.15	0.003256529	-0.19	120	-13.59	14650	-0.08	145	-7.49	12869	-0.12	116	-8.13	12280
EVPL	-1.29	2.11E-10	-0.2	118	-13.59	14652	-0.22	100	-11.08	14265	-0.23	123	-16.84	14767
ATF3	-3.18	2.55E-24	-0.19	122	-13.6	14653	-0.22	121	-15.84	15372	-0.26	136	-22.51	15724
IRAK3	-1.35	4.72E-13	-0.13	153	-13.61	14654	-0.08	154	-8.21	13189	-0.27	135	-23.06	15783
CD300C	-1.49	3.17E-22	-0.25	104	-13.61	14655	-0.16	156	-17.31	15644	-0.4	116	-26.25	16066
HIC1	-1.07	8.72E-08	-0.2	118	-13.61	14656	-0.16	142	-14.55	15113	-0.23	125	-17.17	14828
SIDT1	-1.12	0.000343581	-0.17	128	-13.61	14657	-0.09	146	-8.61	13366	-0.25	137	-22.09	15672
MGST1	-1.59	8.93E-08	-0.21	116	-13.62	14658	-0.15	121	-10.36	14022	-0.14	129	-11.04	13272
IFNG	-1.07	0.176627802	-0.14	150	-13.63	14659	-0.04	114	-2.33	10043	-0.26	116	-16.87	14774
PTPRN	-1.36	8.31E-16	-0.25	104	-13.65	14660	-0.31	127	-23.81	16296	-0.17	109	-10.17	12978
RASSF2	-2.11	3.39E-17	-0.19	123	-13.65	14661	-0.06	136	-5.49	11908	-0.29	161	-32.34	16375
SBDS	-1.19	0.000111047	-0.14	146	-13.66	14662	-0.07	125	-5.62	11987	-0.01	110	-0.72	9044
HSD11B1	-2.84	1.10E-09	-0.2	120	-13.66	14663	-0.21	103	-11.04	14255	-0.23	127	-17.51	14887
TUFM	-1.16	1.43E-05	-0.25	105	-13.66	14664	-0.16	132	-13.04	14763	-0.21	116	-14.06	14127
HNF1B_t1	-1.06	0.001986805	-0.18	125	-13.66	14665	NA	NA	NA	NA	-0.12	121	-8.77	12518
TRIM5_t2	-1.1	5.66E-05	-0.17	130	-13.66	14666	-0.17	137	-15.01	15200	-0.14	122	-9.91	12900
AFAP1L2_t2	-1.01	0.392054689	-0.18	126	-13.67	14667	-0.07	131	-5.99	12176	-0.15	120	-10.28	13016
IL21R	-1.2	0.000114797	-0.16	133	-13.67	14668	-0.1	143	-9.07	13541	-0.36	152	-36.25	16451
ABI3BP	-1.8	9.51E-14	-0.2	118	-13.68	14669	-0.16	126	-12.36	14605	-0.27	127	-21.09	15518
ADAP2_t1	-1.82	2.25E-16	-0.18	128	-13.69	14670	NA	NA	NA	NA	-0.31	136	-26.88	16119
ZFP1	-1.35	3.68E-15	-0.19	122	-13.69	14671	-0.19	138	-16.69	15546	-0.18	126	-13.91	14083
NPHS2	-1.06	0.156267584	-0.17	130	-13.69	14672	-0.01	149	-0.73	8954	-0.09	119	-6.54	11665
VEPH1	-1.05	0.027409346	-0.16	134	-13.69	14673	-0.09	130	-7.58	12923	-0.19	133	-15.9	14563
KCNQ1	-1.55	4.06E-10	-0.24	108	-13.69	14674	-0.12	104	-6.41	12376	-0.33	120	-22.96	15771
PXDN	-1.77	2.45E-16	-0.22	113	-13.7	14675	-0.12	136	-10.21	13958	-0.3	145	-28.39	16216
CXCL1	-2.62	2.04E-08	-0.2	117	-13.72	14676	-0.11	120	-7.96	13095	-0.29	100	-14.67	14293
TMEM170A	-1.18	9.22E-10	-0.15	139	-13.72	14677	-0.17	114	-10.87	14198	-0.23	142	-20.89	15482
MAOA	-1.62	1.52E-09	-0.19	124	-13.72	14678	-0.22	137	-18.75	15830	-0.13	149	-12.47	13689
SLC7A8	-1.81	1.69E-28	-0.23	109	-13.72	14679	-0.19	153	-19.52	15932	-0.41	120	-28.65	16236
AGXT	-2.65	5.21E-15	-0.25	104	-13.73	14680	-0.39	127	-29.92	16466	-0.31	110	-18.64	15105
CCM2	-1.21	2.00E-06	-0.25	105	-13.73	14681	-0.17	116	-11.13	14277	-0.23	133	-19	15173
SEC14L4	-1.98	5.45E-07	-0.19	121	-13.74	14682	-0.21	133	-17.05	15599	-0.2	127	-15.11	14398
AIF1	-2.25	3.11E-18	-0.21	115	-13.74	14683	-0.13	111	-7.87	13059	-0.4	117	-26.55	16093
SIRT3	-1	0.986844572	-0.16	137	-13.74	14684	-0.07	128	-5.53	11935	-0.05	127	-3.96	10618
PLXNB1	-1.3	3.69E-07	-0.25	106	-13.75	14685	-0.12	118	-8.08	13143	-0.26	111	-15.71	14522
TCEAL7	-1.06	3.03E-06	-0.17	130	-13.76	14686	-0.07	127	-5.01	11664	-0.15	130	-11.67	13446
SYTL2_t1	-1.64	2.30E-11	-0.18	125	-13.76	14687	-0.05	113	-3.01	10478	-0.24	141	-21.4	15567
LAMA2	-2.46	1.70E-16	-0.22	113	-13.77	14688	-0.08	126	-6.42	12385	-0.3	102	-15.63	14502
WAS	-1.27	5.34E-22	-0.16	138	-13.77	14689	-0.22	131	-17.94	15726	-0.19	88	-7.37	11984
NCOR1	-1.16	0.000100796	-0.16	136	-13.78	14690	-0.03	126	-2.26	10004	-0.13	136	-11.52	13406
FAM46A	-1.93	1.83E-14	-0.15	139	-13.79	14691	-0.23	136	-20	15986	-0.23	143	-21.06	15510
ADM	-1.71	1.22E-07	-0.14	150	-13.79	14692	-0.11	104	-5.76	12065	-0.13	128	-10.4	13056
TBC1D10C	-1.86	7.19E-10	-0.21	116	-13.79	14693	-0.07	105	-3.88	11020	-0.31	100	-15.5	14482
RAB17	-2.01	6.38E-17	-0.28	100	-13.79	14694	-0.34	120	-23.8	16294	-0.35	133	-29.23	16264
FCRL2	-1.11	0.008735749	-0.18	127	-13.8	14695	-0.04	103	-2.18	9951	-0.23	121	-16.42	14669
SLC22A13	-1.05	0.043238284	-0.21	117	-13.8	14696	-0.17	128	-13.17	14785	-0.14	109	-8.17	12301
BACH2	-1.12	0.002218997	-0.15	142	-13.8	14697	-0.12	158	-13.44	14859	-0.17	154	-17.5	14885
LRRC7	-1.04	6.38E-05	-0.17	131	-13.81	14698	-0.06	117	-3.92	11051	-0.19	124	-13.74	14034
APOB	-1.86	3.37E-06	-0.23	111	-13.81	14699	-0.25	89	-9.59	13731	-0.15	131	-12.24	13621
MOGAT1	-1.6	1.09E-08	-0.28	100	-13.82	14700	-0.21	60	-2.1	9898	-0.18	60	-1.82	9624
GIMAP2	-1.44	8.50E-09	-0.17	130	-13.82	14701	-0.07	120	-4.78	11526	-0.25	129	-19.74	15289
CST5	-1.49	1.87E-15	-0.28	100	-13.82	14702	-0.2	137	-17.68	15693	-0.34	110	-20.51	15415
F2R	-1.82	5.38E-16	-0.17	133	-13.83	14703	-0.06	134	-4.74	11506	-0.22	118	-14.99	14374
ABCC9	-1.69	4.99E-11	-0.18	127	-13.83	14704	-0.19	129	-14.84	15176	-0.16	139	-13.89	14075
RBP5	-2.14	1.97E-11	-0.23	110	-13.83	14705	-0.33	168	-38.79	16491	-0.17	115	-11.32	13353
PKP3	-1.24	0.001926515	-0.2	118	-13.84	14706	-0.13	103	-7.1	12694	-0.29	100	-14.74	14308
LDHD	-1.96	3.20E-12	-0.25	106	-13.84	14707	-0.31	140	-27.93	16435	-0.32	100	-16.24	14633
PRKAR2B	-1.69	1.14E-11	-0.2	120	-13.84	14708	-0.09	122	-6.19	12281	-0.27	137	-23.9	15865
HPS5	-1.65	1.50E-12	-0.18	128	-13.84	14709	-0.16	126	-12.02	14504	-0.23	144	-21.21	15540
NBEA	-1.44	2.39E-09	-0.16	136	-13.84	14710	0	144	-0.19	8606	-0.22	145	-20.54	15419
PHGDH	-3.29	4.00E-25	-0.32	93	-13.85	14711	-0.29	122	-20.58	16056	-0.41	131	-33.5	16405
MICALL2_t1	-1.35	7.37E-09	-0.25	106	-13.85	14712	NA	NA	NA	NA	-0.25	126	-18.66	15112
UBXN10_t1	-1.47	0.000682548	-0.14	146	-13.86	14713	NA	NA	NA	NA	NA	NA	NA	NA
CRHR1	-1.35	7.59E-20	-0.28	100	-13.86	14714	-0.28	158	-29.86	16464	-0.29	118	-19.82	15297
LY9	-1.16	6.33E-09	-0.26	104	-13.86	14715	-0.04	133	-3.42	10745	-0.31	110	-18.67	15115
CNNM3	-1.14	0.000384124	-0.15	140	-13.86	14716	-0.23	116	-15.41	15292	-0.18	122	-13.21	13901
GJD4	-1.05	0.001052378	-0.18	127	-13.87	14717	-0.19	113	-12.26	14571	-0.2	104	-11	13257
DLL1	-1.65	2.80E-11	-0.17	133	-13.87	14718	-0.16	116	-10.32	14007	-0.21	146	-19.97	15325
NMRK1	-1.85	1.88E-20	-0.21	115	-13.88	14719	-0.28	122	-20.42	16031	-0.25	133	-20.92	15487
MAPK10	-1.17	7.73E-05	-0.18	126	-13.9	14720	-0.03	127	-2.04	9856	-0.25	140	-22.81	15757
GPR125	-1.49	7.26E-12	-0.2	120	-13.9	14721	-0.26	143	-24.17	16311	-0.22	133	-18.13	15002
IL26	-1.1	2.08E-13	-0.2	120	-13.9	14722	-0.14	113	-8.85	13449	-0.23	127	-17.47	14874
SLC5A3	-1.06	0.087337347	-0.13	153	-13.9	14723	-0.09	142	-8.56	13344	-0.19	139	-16.88	14779
RORC_t1	-1.38	3.50E-05	-0.21	115	-13.9	14724	NA	NA	NA	NA	-0.24	139	-21.46	15578
MCCC1	-1.37	7.50E-10	-0.24	107	-13.91	14725	-0.21	105	-11.4	14343	-0.17	109	-10.14	12973
CTBP2_t2	-1.54	4.32E-07	-0.15	142	-13.92	14726	0.06	135	4.97	5361	-0.31	141	-28.08	16194
IRF1	-1.43	6.88E-11	-0.2	120	-13.92	14727	-0.18	139	-16.11	15442	-0.28	153	-28.54	16231
ETFDH	-2.76	8.40E-22	-0.3	97	-13.92	14728	-0.27	104	-14.35	15067	-0.26	100	-13.07	13859
CELF2_t2	-1.3	4.58E-09	-0.17	132	-13.93	14729	-0.02	158	-2.26	10002	-0.31	134	-26.25	16064
UBL3	-1.44	1.31E-07	-0.17	132	-13.93	14730	-0.18	128	-13.93	14979	-0.17	131	-14.05	14123
PAFAH2	-1.19	2.62E-09	-0.17	133	-13.94	14731	-0.18	131	-14.2	15028	-0.15	121	-10.46	13079
CDC42EP2	-1.49	6.85E-17	-0.24	108	-13.94	14732	-0.11	140	-10.07	13906	-0.35	126	-26.24	16061
COL10A1	-1.18	0.043489839	-0.15	146	-13.95	14733	-0.01	108	-0.82	9027	-0.26	129	-20.66	15437
DACT1_t1	-2.12	5.15E-14	-0.18	126	-13.95	14734	-0.08	143	-7.25	12761	NA	NA	NA	NA
RNF126	-1.14	0.00010957	-0.24	109	-13.95	14735	-0.09	115	-5.89	12129	-0.21	100	-10.63	13124
FSTL1	-1.83	1.12E-08	-0.18	126	-13.95	14736	-0.1	140	-9.27	13611	-0.23	143	-21.76	15624
CTNNA3	-1.14	1.52E-07	-0.17	130	-13.96	14737	-0.18	123	-13.16	14784	-0.21	120	-14.6	14269
SERTAD3	-1.24	3.67E-10	-0.19	124	-13.96	14738	-0.06	124	-4.39	11320	-0.36	121	-25.26	15973
ESD	-1.52	1.82E-13	-0.2	119	-13.96	14739	-0.13	108	-7.52	12883	-0.3	112	-18.53	15086
GATA3	-1.09	0.001248125	-0.19	123	-13.96	14740	-0.03	115	-1.91	9750	-0.28	153	-28.88	16254
EOMES	-1.45	3.19E-10	-0.18	126	-13.97	14741	-0.07	131	-5.91	12139	-0.33	116	-21.62	15608
HNRNPK_t2	-1.16	1.94E-06	-0.16	138	-13.97	14742	-0.1	124	-7.41	12838	-0.17	123	-12.65	13749
ITGA5	-1.14	0.000116959	-0.15	146	-13.97	14743	-0.13	132	-10.46	14047	-0.14	135	-11.62	13435

F13B	-1.86	1.95E-05	-0.16	138	-13.97	14744	-0.25	112	-15.27	15269	-0.01	110	-0.74	9052
MYLK	-1.47	6.98E-08	-0.18	129	-13.98	14745	-0.17	147	-16.35	15484	-0.15	130	-12.3	13645
EPB41L2	-1.35	1.44E-10	-0.16	140	-13.98	14746	-0.05	123	-3.61	10887	-0.2	124	-14.86	14341
CD200R1_t2	-1.07	4.64E-08	-0.19	123	-13.98	14747	-0.11	98	-5.08	11703	-0.19	103	-10.18	12986
FAM150A	-1.12	4.18E-07	-0.21	117	-13.98	14748	-0.13	118	-8.56	13346	-0.33	138	-29.17	16263
PPIL4	-1.22	3.04E-09	-0.16	139	-13.99	14749	-0.07	121	-5.19	11769	-0.13	119	-8.75	12511
MAX_t3	-1.29	1.12E-10	-0.2	119	-13.99	14750	-0.08	142	-7.37	12824	-0.4	120	-27.73	16171
MRPL16	-1.22	4.05E-09	-0.23	110	-14	14751	-0.18	104	-9.79	13814	-0.21	104	-11.39	13369
CLEC12A	-1.4	2.45E-21	-0.21	118	-14	14752	-0.15	118	-10.14	13932	-0.34	110	-20.66	15438
SAA4	-4.69	6.34E-24	-0.35	90	-14	14753	-0.33	129	-26.17	16387	-0.38	88	-14.42	14223
CRK	-1.39	2.52E-17	-0.17	132	-14	14754	-0.25	130	-20.02	15989	-0.23	117	-15.7	14517
LEPREL2	-1.43	5.03E-11	-0.2	120	-14	14755	-0.04	101	-2.25	9996	-0.32	104	-17.14	14824
CCL21	-1.56	1.49E-16	-0.26	104	-14.01	14756	-0.21	143	-19.9	15972	-0.33	104	-17.96	14970
SRPR	-1.16	0.000213623	-0.19	124	-14.01	14757	-0.09	120	-6.44	12388	-0.22	136	-18.85	15147
MSC	-1.09	0.005123393	-0.19	125	-14.02	14758	0.01	136	1.27	7628	-0.26	120	-18.37	15057
PROCR	-1.54	6.72E-06	-0.18	126	-14.02	14759	0	125	0.14	8370	-0.26	91	-10.74	13166
NRG1_t3	-1.97	1.06E-12	-0.22	114	-14.03	14760	-0.22	150	-21.99	16168	-0.29	120	-20.07	15343
IL17RE	-1.17	5.38E-05	-0.21	116	-14.03	14761	-0.16	115	-10.21	13962	-0.22	105	-11.86	13505
MGAT1	-1.32	5.23E-17	-0.25	107	-14.03	14762	-0.32	113	-19.85	15967	-0.35	128	-27.24	16148
KIR3DL3	-1.04	0.007305797	-0.13	159	-14.03	14763	-0.07	112	-4.25	11246	-0.11	123	-8.34	12379
HCK	-1.8	4.77E-19	-0.23	111	-14.04	14764	-0.09	123	-6.57	12448	-0.43	134	-35.72	16446
MLXIPL_t2	-1.15	9.02E-07	-0.22	115	-14.05	14765	-0.1	134	-8.16	13178	-0.21	102	-11.05	13274
OSTF1	-1.46	1.72E-14	-0.22	113	-14.05	14766	-0.16	119	-11.38	14339	-0.29	148	-28.15	16196
SPG7_t2	-1.23	7.15E-10	-0.21	116	-14.05	14767	-0.2	131	-15.9	15393	-0.25	144	-23.69	15850
SCN7A	-1.03	0.00645659	-0.16	136	-14.05	14768	-0.13	119	-9.29	13622	-0.19	122	-13.89	14077
CTGF	-2.14	7.58E-09	-0.17	134	-14.07	14769	-0.07	120	-4.79	11538	-0.2	124	-15.03	14379
FOXRED1	-1.23	1.22E-06	-0.25	106	-14.07	14770	-0.18	119	-12.28	14578	-0.27	130	-21.52	15594
SETBP1	-1.36	2.25E-06	-0.15	147	-14.07	14771	-0.07	150	-7.05	12674	-0.2	151	-20.19	15367
NUBP1	-1.1	0.001296452	-0.19	125	-14.07	14772	-0.13	121	-9.15	13573	-0.15	117	-10.17	12982
LIMD2_t1	-1.62	1.25E-12	-0.18	129	-14.08	14773	NA	NA	NA	NA	NA	NA	NA	NA
GFRA1	-1.42	5.89E-06	-0.14	152	-14.09	14774	-0.19	140	-17.11	15605	-0.19	138	-16.53	14695
SCGB3A1	-1.43	4.26E-12	-0.37	88	-14.09	14775	-0.21	93	-8.98	13495	-0.34	68	-6.05	11453
RCBTB2	-1.54	6.28E-19	-0.17	133	-14.1	14776	-0.09	126	-6.58	12457	-0.3	133	-24.7	15935
F2_t1	-1.97	2.48E-08	-0.23	112	-14.11	14777	NA	NA	NA	NA	-0.23	102	-12.21	13610
EPB41L3	-1.56	4.49E-14	-0.13	156	-14.11	14778	-0.1	128	-7.72	13001	-0.28	134	-23.59	15840
SETD3	-1.25	3.78E-13	-0.2	122	-14.12	14779	-0.17	118	-11.79	14444	-0.18	124	-13.38	13950
MYH7	-1.15	4.16E-13	-0.22	113	-14.13	14780	-0.23	121	-16.23	15463	-0.2	86	-7.38	11989
KLF2_t1	-1.13	8.92E-11	-0.22	114	-14.13	14781	NA	NA	NA	NA	-0.18	136	-15.42	14461
FLI1	-1.68	1.22E-11	-0.17	135	-14.13	14782	-0.06	131	-4.91	11601	-0.31	154	-32.06	16364
FAM123C	-1.12	4.43E-06	-0.17	135	-14.13	14783	-0.2	127	-15.22	15257	-0.11	140	-10.23	13002
KLHL36	-1.49	7.04E-23	-0.24	109	-14.13	14784	-0.27	108	-15.63	15327	-0.37	93	-15.88	14557
RAB3IL1	-1.34	3.35E-15	-0.2	121	-14.13	14785	-0.09	126	-6.82	12575	-0.29	114	-18.63	15104
GJA4	-1.58	2.42E-12	-0.2	120	-14.14	14786	-0.11	127	-8.1	13151	-0.27	119	-18.45	15072
LAP3	-1.53	6.86E-09	-0.15	145	-14.14	14787	-0.17	116	-11.45	14361	-0.22	122	-15.93	14570
NNT	-1.2	0.011032037	-0.2	122	-14.14	14788	-0.14	110	-8.11	13156	-0.02	152	-1.67	9534
CYP2A6	-6.07	3.35E-19	-0.28	100	-14.15	14789	-0.37	92	-15.6	15323	-0.31	99	-15.28	14432
RNF135_t1	-1.12	1.80E-11	-0.16	140	-14.15	14790	-0.15	130	-11.64	14408	-0.31	119	-21.07	15513
CCDC57	-1.23	4.72E-05	-0.29	98	-14.15	14791	-0.17	102	-9.02	13509	-0.27	89	-10.66	13139
PAOX	-1.25	0.000127053	-0.16	136	-14.15	14792	-0.2	98	-9.57	13720	-0.14	104	-7.63	12087
CYP2J2	-2.2	6.29E-14	-0.26	104	-14.15	14793	-0.3	101	-15.21	15250	-0.25	104	-13.33	13932
GOLPH3	-1.14	0.005398519	-0.14	150	-14.16	14794	-0.07	128	-5.07	11700	-0.09	137	-7.88	12188
PBLD_t1	-2.32	8.87E-25	-0.35	90	-14.16	14795	-0.33	102	-17.18	15614	-0.32	84	-10.96	13241
TRIM46	-1.08	9.07E-07	-0.19	123	-14.16	14796	-0.17	120	-12.01	14501	-0.07	116	-4.66	10918
GLOD4	-1.46	5.05E-17	-0.19	125	-14.17	14797	-0.2	119	-13.72	14925	-0.34	111	-20.71	15446
SLC32A1	-1.07	1.87E-05	-0.2	120	-14.17	14798	-0.2	110	-12.29	14581	-0.12	104	-6.44	11621
RWDD2B	-1.16	0.000409597	-0.17	135	-14.18	14799	-0.19	115	-12.45	14628	-0.11	122	-7.62	12084
NDNF	-1.08	1.13E-10	-0.19	126	-14.18	14800	-0.23	131	-18.35	15779	-0.22	131	-17.71	14930
GPR173	-1.16	5.33E-08	-0.18	128	-14.19	14801	-0.15	137	-13.44	14857	-0.15	136	-12.69	13757
YPEL2_t1	-1.26	9.31E-11	-0.18	129	-14.19	14802	-0.15	138	-13.48	14867	NA	NA	NA	NA
MANBA	-1.18	5.53E-06	-0.2	121	-14.2	14803	-0.08	113	-5.2	11774	-0.24	129	-18.8	15137
GLI3	-1.43	8.02E-09	-0.17	133	-14.2	14804	-0.07	156	-7.61	12935	-0.27	129	-21.48	15585
BCL2L13	-1.02	0.300527425	-0.16	137	-14.2	14805	0	140	0.01	8460	-0.12	137	-10.18	12984
FHL2	-2.6	6.43E-12	-0.2	120	-14.2	14806	-0.02	119	-1.7	9613	-0.24	118	-16.29	14645
MAN2B2	-1.23	4.75E-07	-0.2	120	-14.21	14807	-0.23	140	-20.91	16079	-0.21	124	-15.43	14462
ABTB1	-1.15	5.08E-08	-0.21	119	-14.22	14808	-0.21	101	-10.95	14223	-0.27	125	-20.46	15407
DDR2	-1.2	6.03E-07	-0.28	100	-14.22	14809	-0.04	120	-3.03	10492	-0.23	128	-17.71	14928
GLDC	-2.62	7.24E-14	-0.21	117	-14.23	14810	-0.23	126	-17.79	15713	-0.19	117	-13.05	13855
CBX6	-1.13	1.32E-05	-0.2	120	-14.23	14811	-0.11	128	-8.89	13463	-0.18	114	-11.35	13360
LPXN	-1.33	6.86E-12	-0.17	133	-14.23	14812	-0.05	111	-3.22	10612	-0.33	126	-24.79	15943
P2RY12	-1.3	3.49E-21	-0.3	97	-14.24	14813	-0.19	101	-9.75	13795	-0.39	99	-19.07	15186
ADAMTS17	-1.24	1.29E-12	-0.2	123	-14.25	14814	-0.27	123	-19.86	15968	-0.13	144	-12.54	13710
SLC7A5	-1.33	4.34E-05	-0.16	137	-14.26	14815	-0.12	149	-11.56	14391	-0.19	142	-17.9	14962
SLC28A3	-1.02	0.11671131	-0.16	138	-14.26	14816	0.08	128	6.21	4656	-0.12	132	-9.79	12850
SARDH	-1.14	3.02E-06	-0.16	142	-14.26	14817	-0.22	105	-11.94	14489	-0.17	101	-8.86	12546
ACSL5	-1.86	5.62E-11	-0.18	128	-14.26	14818	-0.24	124	-17.5	15672	-0.2	120	-14.24	14169
RARA	-1.14	8.92E-11	-0.19	124	-14.27	14819	-0.18	113	-11.52	14380	-0.23	122	-16.4	14665
SYTL2_t2	-1.14	8.88E-10	-0.19	125	-14.27	14820	-0.03	113	-1.83	9710	-0.19	141	-17.07	14811
CHRNB2	-1.03	0.128565051	-0.16	142	-14.28	14821	-0.09	151	-9.17	13577	-0.09	146	-8.73	12504
PNOC	-1.33	1.84E-07	-0.25	106	-14.28	14822	-0.1	108	-6.08	12230	-0.32	126	-24.06	15877
F8	-1.24	1.95E-11	-0.17	136	-14.28	14823	-0.22	143	-20.55	16050	-0.21	136	-18.15	15009
TRIM74	-1.37	1.14E-08	-0.22	116	-14.28	14824	-0.11	126	-8.7	13395	-0.26	92	-10.92	13225
ARAP2	-1.44	1.39E-09	-0.15	143	-14.29	14825	-0.09	116	-5.85	12110	-0.21	147	-20.49	15413
MXRA7_t2	-1.04	0.276052053	-0.13	158	-14.3	14826	0.04	123	2.61	6753	-0.11	116	-7.34	11974
XBP1_t1	-1.32	2.62E-07	-0.18	131	-14.3	14827	-0.07	118	-4.49	11380	-0.26	161	-29.06	16261
ZBTB45	-1.11	0.001480417	-0.12	167	-14.3	14828	-0.12	136	-9.98	13876	-0.1	104	-5.36	11184
TM6SF2	-1.37	6.38E-17	-0.29	100	-14.3	14829	-0.25	122	-17.95	15728	-0.28	97	-13.03	13849
CYP2A13	-3.93	4.12E-17	-0.29	100	-14.3	14830	-0.33	90	-13.39	14848	-0.31	88	-11.71	13462
STK40	-1.46	8.56E-13	-0.2	123	-14.3	14831	-0.13	133	-10.96	14225	-0.28	129	-22.19	15688
AHSG_t1	-1.59	0.000342945	-0.22	116	-14.31	14832	NA	NA	NA	NA	-0.16	101	-7.97	12228
SCP2_t1	-1.56	2.15E-07	-0.21	118	-14.31	14833	-0.19	126	-14.52	15106	-0.13	134	-10.78	13175
CD244	-1.15	2.41E-12	-0.19	125	-14.31	14834	-0.24	133	-20.03	15990	-0.26	124	-19.09	15188
MVK	-1.19	3.04E-05	-0.21	117	-14.31	14835	-0.13	114	-8.08	13140	-0.21	120	-14.39	14213
RAPGEF5	-1.24	1.33E-05	-0.17	133	-14.31	14836	-0.16	137	-13.89	14965	-0.07	132	-5.59	11269
ARPP21_t1	-1.17	0.007362913	-0.19	126	-14.32	14837	-0.24	123	-17.38	15656	-0.08	138	-7	11849
UGT2A1	-1.32	2.58E-10	-0.18	131	-14.32	14838	-0.26	102	-13.33	14834	-0.19	99	-9.41	12722
AIG1	-1.78	3.00E-13	-0.27	104	-14.32	14839	-0.28	132	-23.24	16261	-0.27	146	-25.51	15990
GSTA5	-2.39	4.69E-09	-0.28	101	-14.33	14840	-0.39	99	-19.09	15881	-0.19	107	-10.74	13165
RNF185	-1.17	3.81E-07	-0.19	126	-14.33	14841	-0.21	145	-19.93	15981	-0.17	117	-11.22	13310
MARVELD1	-2.14	5.98E-12	-0.19	126	-14.33	14842	0	136	0.41	8196	-0.31	131	-25.01	15956
HADHB	-1.62	2.43E-17	-0.2	120	-14.35	14843	-0.19	128	-14.77	15163	-0.23	154	-24.2	15889
CNRIP1	-1.72	5.57E-17	-0.2	120	-14.35	14844	-0.12	124	-9.09	13557	-0.31	134	-26.25	16065
EMR1	-2.13	1.52E-24	-0.28	101	-14.36	14845	-0.19	105	-10.49	14062	-0.43	120	-30.27	16302
LYRM5	-1.59	1.38E-10	-0.24	110	-14.36	14846	-0.24	115	-15.56	15315	-0.11	118	-7.6	12069
SDHB	-1.47	3.40E-12	-0.24	109	-14.37	14847	-0.22	84	-7.59	12925	-0.26	101	-13.04	13853
MAP1LC3A	-1.45	4.07E-16	-0.33	93	-14.37	14848	-0.32	94	-14.06	15005	-0.34	102	-17.62	14908
PRDM16_t2	-1.24	0.00166118	-0.2	122	-14.37	14849	-0.02	130	-1.46	9469	-0.29	143	-27.39	16155

SAMD10	-1.82	6.07E-19	-0.18	132	-14.38	14850	-0.27	131	-21.66	16146	-0.16	127	-12.32	13650
KYNU_t1	-2.26	6.76E-15	-0.22	114	-14.39	14851	-0.14	97	-6.72	12524	-0.21	102	-10.84	13197
GIPC2	-1.46	3.97E-08	-0.16	140	-14.41	14852	-0.14	147	-13.3	14825	-0.13	127	-9.65	12792
HPGD_t1	-2.69	9.80E-11	-0.15	143	-14.41	14853	-0.18	129	-14.36	15071	NA	NA	NA	NA
NRG1_t4	-1.62	1.53E-10	-0.21	120	-14.41	14854	-0.22	122	-15.74	15351	-0.27	120	-18.64	15108
CLINT1	-1.23	2.82E-06	-0.17	136	-14.42	14855	-0.08	116	-5.5	11912	-0.09	126	-6.65	11716
NAMPT_t1	-2.12	2.88E-14	-0.15	146	-14.42	14856	NA	NA	NA	NA	-0.16	129	-12.41	13673
MRPS25	-1.15	0.007333745	-0.14	150	-14.42	14857	-0.11	127	-8.39	13287	-0.09	133	-7.52	12040
LRRC25	-1.52	1.25E-12	-0.22	115	-14.42	14858	-0.13	133	-11.12	14275	-0.36	115	-23.51	15833
KIR2DL4	-1.05	0.025123219	-0.13	159	-14.42	14859	-0.17	145	-15.95	15405	-0.11	119	-7.75	12138
C2CD4A	-1.28	1.09E-06	-0.17	134	-14.42	14860	-0.18	148	-17.26	15633	-0.15	126	-11.69	13449
PRDX4	-1.31	2.90E-09	-0.19	126	-14.43	14861	-0.17	108	-9.57	13722	-0.19	101	-9.72	12818
SC5DL	-1.75	8.05E-08	-0.15	146	-14.43	14862	-0.17	136	-14.77	15161	-0.08	149	-8.07	12254
COL23A1	-1.11	4.43E-07	-0.18	130	-14.43	14863	-0.19	115	-12.59	14665	-0.29	114	-18.46	15073
PUS10	-1.33	1.95E-11	-0.17	134	-14.43	14864	-0.19	119	-13.1	14774	-0.2	119	-13.59	13999
CD68	-1.67	1.01E-15	-0.18	132	-14.44	14865	-0.13	131	-10.77	14167	-0.33	120	-23.28	15802
ARAF	-1.26	1.25E-10	-0.21	118	-14.45	14866	-0.34	114	-21.66	16145	-0.23	120	-16.07	14604
SLC2A8_t1	-1.14	0.001227907	-0.18	131	-14.45	14867	-0.13	129	-10.25	13981	NA	NA	NA	NA
GLT8D2	-1.68	5.28E-16	-0.25	109	-14.46	14868	-0.07	122	-4.78	11525	-0.32	119	-21.78	15627
PUS3	-1.32	1.91E-07	-0.19	125	-14.46	14869	-0.19	107	-10.58	14101	-0.19	115	-12.25	13626
SLC27A5	-2.84	2.16E-12	-0.29	100	-14.47	14870	-0.38	127	-29.3	16454	-0.23	93	-9.75	12832
KLF7	-1.2	2.64E-07	-0.13	160	-14.48	14871	0.02	138	1.77	7300	-0.19	155	-20.43	15403
ZBTB7A	-1.18	3.25E-10	-0.26	106	-14.48	14872	-0.2	118	-13.77	14939	-0.38	124	-28.4	16217
NFIA	-1.54	1.02E-10	-0.15	144	-14.49	14873	-0.2	130	-16.4	15492	-0.13	143	-11.82	13494
ZBP1_t1	-1.24	2.55E-07	-0.2	122	-14.52	14874	-0.07	131	-5.73	12042	NA	NA	NA	NA
RNF180	-1.3	3.69E-19	-0.2	123	-14.52	14875	-0.09	137	-7.55	12899	-0.27	130	-21.72	15621
TMEM88	-1.34	6.59E-09	-0.21	120	-14.53	14876	-0.13	112	-7.75	13011	-0.22	103	-11.52	13407
CNKSR3	-1.73	1.59E-13	-0.21	118	-14.53	14877	-0.22	145	-20.48	16042	-0.23	132	-18.72	15124
PRIC285	-1.08	4.48E-05	-0.18	131	-14.53	14878	-0.13	138	-11.36	14333	-0.19	126	-14.29	14188
CECR1_t2	-1.2	8.21E-10	-0.18	132	-14.53	14879	-0.18	131	-14.36	15070	-0.3	131	-24.39	15906
VAT1L	1.03	0.762423167	-0.19	128	-14.54	14880	-0.14	133	-12.03	14507	-0.06	127	-4.72	10948
CITED2	-1.56	1.94E-12	-0.2	122	-14.54	14881	-0.2	124	-14.62	15125	-0.25	136	-21.51	15592
NR4A1	-3.87	8.51E-26	-0.24	111	-14.54	14882	-0.23	118	-15.48	15303	-0.33	104	-17.77	14936
ZC3H6	-1.13	0.004320682	-0.18	132	-14.55	14883	-0.06	138	-5.43	11882	-0.07	153	-6.78	11766
SDCBP2	-2.13	3.36E-13	-0.21	119	-14.55	14884	-0.18	118	-12.29	14580	-0.31	126	-23.42	15818
GNAI2	-1.36	4.51E-18	-0.25	108	-14.55	14885	-0.18	140	-16.28	15471	-0.34	112	-20.97	15496
ARID4A_t1	-1.22	3.54E-09	-0.17	136	-14.55	14886	NA	NA	NA	NA	NA	NA	NA	NA
ORMDL3_t1	-1.14	9.58E-05	-0.18	132	-14.56	14887	NA	NA	NA	NA	-0.19	120	-13.45	13967
AGPAT9	-1.94	4.84E-11	-0.19	126	-14.56	14888	-0.23	118	-15.4	15289	-0.09	122	-6.26	11558
SPTBN1_t1	-1.05	0.112809294	-0.15	149	-14.56	14889	-0.04	123	-3.08	10524	-0.04	132	-3.64	10456
PDE11A_t1	-1.13	0.000710269	-0.17	138	-14.57	14890	NA	NA	NA	NA	NA	NA	NA	NA
SERPIND1	-1.31	8.92E-05	-0.23	114	-14.58	14891	-0.3	120	-21.12	16104	-0.06	124	-4.58	10881
MRPS18C	-1.17	5.79E-06	-0.21	118	-14.58	14892	-0.21	129	-16.41	15496	-0.1	129	-8.25	12339
SEC62_t1	-1.09	5.69E-08	-0.16	141	-14.59	14893	NA	NA	NA	NA	-0.14	130	-11.23	13315
NUDT9P1	-1.2	2.02E-08	-0.19	127	-14.59	14894	-0.11	131	-8.87	13455	-0.19	136	-16.15	14618
SAT1	-1.73	9.86E-16	-0.17	134	-14.6	14895	-0.26	118	-17.37	15654	-0.22	108	-12.87	13808
GPHN	-1.31	1.76E-09	-0.18	129	-14.6	14896	-0.25	115	-16.45	15501	-0.25	133	-20.9	15483
PEX11G	-1.41	4.39E-16	-0.29	100	-14.61	14897	-0.25	93	-10.73	14148	-0.39	103	-20.74	15450
GTF2IRD2	-1.31	1.49E-10	-0.19	125	-14.61	14898	-0.2	108	-11.53	14382	-0.22	119	-15.41	14459
S100A14	-1.64	1.03E-05	-0.18	131	-14.62	14899	0.03	117	2.27	6977	-0.29	120	-20.48	15412
GIMAP1	-1.74	3.42E-14	-0.21	120	-14.64	14900	-0.13	137	-11.1	14270	-0.35	125	-26.12	16043
DCTD_t1	-1.43	6.75E-22	-0.22	116	-14.64	14901	-0.24	122	-17.37	15652	NA	NA	NA	NA
OR11A1	-1.01	0.420651019	-0.2	124	-14.65	14902	-0.09	119	-5.96	12162	-0.12	122	-8.8	12525
EML1	-1.47	1.17E-09	-0.16	140	-14.65	14903	-0.09	132	-7.61	12936	-0.17	141	-15.9	14564
PROS1_t1	-2.1	1.37E-13	-0.23	115	-14.67	14904	NA	NA	NA	NA	-0.23	116	-15.11	14399
TSKU	-1.65	1.57E-08	-0.21	119	-14.68	14905	-0.14	135	-12.32	14588	-0.24	132	-19.63	15272
KDSR	-1.43	1.85E-16	-0.16	141	-14.68	14906	-0.2	167	-23.14	16256	-0.22	136	-19.15	15197
KCNK4	-1.16	1.11E-09	-0.17	136	-14.68	14907	-0.18	131	-14.8	15170	-0.14	100	-6.94	11820
DES	-1.35	4.65E-07	-0.24	110	-14.69	14908	-0.1	108	-5.57	11954	-0.37	108	-21.32	15558
SLC22A9_t1	-1.23	0.000484567	-0.14	152	-14.71	14909	-0.19	111	-11.85	14464	NA	NA	NA	NA
NPAS3_t2	-1.35	1.59E-06	-0.14	152	-14.73	14910	-0.14	150	-14.49	15100	-0.19	134	-16.18	14623
HBB	-5.99	2.28E-26	-0.28	102	-14.74	14911	-0.23	88	-8.6	13364	-0.33	98	-15.65	14506
CD302	-2.1	9.74E-15	-0.17	137	-14.74	14912	-0.27	130	-21.32	16128	-0.18	159	-19.26	15216
PGRMC2	-1.21	6.60E-06	-0.2	122	-14.75	14913	-0.21	132	-17.25	15631	-0.09	156	-9.84	12870
CXCL6	-1.77	1.57E-07	-0.19	129	-14.75	14914	-0.1	130	-7.9	13071	-0.28	167	-32.2	16370
Mar-01	-1.5	2.30E-07	-0.18	130	-14.75	14915	-0.27	123	-19.91	15973	-0.2	136	-17.08	14812
FLNB	-1.15	0.000174619	-0.23	115	-14.75	14916	-0.09	143	-8.09	13146	-0.12	123	-8.5	12426
S1PR1	-1.59	3.38E-15	-0.19	129	-14.75	14917	-0.14	130	-11.15	14281	-0.26	159	-28.45	16219
ZRANB1_t1	-1.31	1.65E-07	-0.17	136	-14.76	14918	-0.24	119	-16.32	15478	NA	NA	NA	NA
NDUFAF1	-1.42	4.38E-09	-0.27	104	-14.76	14919	-0.24	94	-10.6	14109	-0.14	96	-6.52	11655
SCARA5	-1.3	2.49E-19	-0.17	138	-14.76	14920	-0.24	142	-21.77	16152	-0.22	132	-18.32	15046
TMEM173	-1.61	6.08E-13	-0.21	120	-14.76	14921	-0.05	131	-3.79	10974	-0.35	111	-21.07	15515
CPXM2	-2.62	5.45E-17	-0.19	126	-14.76	14922	-0.08	129	-6.06	12222	-0.29	134	-24.21	15892
ARHGEF7_t2	-1.13	7.32E-05	-0.19	129	-14.77	14923	-0.09	135	-7.94	13090	-0.18	125	-13.58	13994
ADARB2	-1.06	0.001905066	-0.18	131	-14.78	14924	-0.19	138	-16.36	15485	-0.16	156	-16.53	14694
SLC9A3R2	-1.77	5.11E-16	-0.24	111	-14.78	14925	-0.25	126	-19.37	15912	-0.33	175	-41.63	16487
RXFP2	-1.32	1.79E-11	-0.23	113	-14.78	14926	-0.25	127	-19.09	15880	-0.22	146	-21.12	15525
KLHDC1	-1.2	3.58E-07	-0.21	122	-14.78	14927	-0.19	150	-19.29	15901	-0.11	137	-9.22	12662
ZBTB22	-1.17	1.43E-09	-0.18	132	-14.79	14928	-0.16	110	-9.65	13752	-0.23	127	-17.5	14884
EPS8	-1.3	4.48E-05	-0.18	130	-14.79	14929	0.02	118	1.04	7782	-0.19	126	-14.48	14232
BCKDHB_t1	-2.07	4.04E-20	-0.34	94	-14.81	14930	-0.28	112	-17.32	15645	-0.37	130	-29.92	16285
MRPS31	-1.41	1.21E-08	-0.24	113	-14.81	14931	-0.16	78	-4.42	11340	-0.24	99	-11.98	13533
SIK1_t1	-2.62	3.50E-22	-0.15	149	-14.81	14932	NA	NA	NA	NA	NA	NA	NA	NA
SESN2	-1.06	0.060392242	-0.17	135	-14.81	14933	-0.09	142	-8.6	13361	-0.12	150	-11.63	13436
TDO2	-3.61	3.35E-20	-0.26	106	-14.82	14934	-0.29	105	-16.13	15450	-0.25	129	-19.49	15245
CHRD	-2.05	5.19E-16	-0.26	107	-14.82	14935	-0.26	147	-25.29	16355	-0.27	124	-20.33	15385
HLA-DRB6	-1.24	4.34E-06	-0.19	127	-14.82	14936	0.02	124	1.58	7448	-0.23	139	-20.37	15392
ABLIM3	-1.67	4.74E-15	-0.21	120	-14.83	14937	-0.25	127	-19.22	15893	-0.24	118	-15.99	14586
XBP1_t2	-1.4	4.90E-11	-0.18	131	-14.83	14938	-0.19	118	-12.76	14703	-0.23	161	-25.06	15959
NPTXR	-1.51	4.80E-17	-0.2	125	-14.84	14939	-0.3	143	-28.12	16439	-0.21	150	-20.88	15481
GBP1	-1.7	1.07E-08	-0.16	143	-14.85	14940	-0.14	132	-11.19	14296	-0.24	127	-18.2	15019
DPF3	-1.13	1.84E-08	-0.24	113	-14.85	14941	-0.31	123	-22.97	16249	-0.17	143	-15.76	14531
LRBA	-1.24	1.25E-06	-0.18	133	-14.85	14942	-0.1	115	-6.63	12478	-0.13	141	-12.11	13577
CD1E_t1	-1.12	6.75E-08	-0.16	144	-14.87	14943	-0.02	119	-1.29	9356	-0.17	128	-13.01	13841
ABR	-1.72	1.74E-09	-0.21	120	-14.88	14944	0	147	0.01	8464	-0.37	150	-36.98	16459
MASP1_t2	-1.55	6.07E-10	-0.21	120	-14.88	14945	-0.31	122	-22.33	16200	-0.25	128	-19.25	15213
LILRA5_t2	-1.24	3.10E-18	-0.29	101	-14.89	14946	-0.26	103	-13.59	14891	-0.43	100	-21.27	15549
MCEE	-1.47	2.50E-12	-0.21	122	-14.89	14947	-0.19	95	-8.38	13279	-0.24	111	-14.62	14274
POLE	-1.11	1.28E-07	-0.21	121	-14.9	14948	-0.15	130	-12.04	14515	-0.25	122	-17.68	14920
KCNJ15	-1.61	1.49E-10	-0.24	112	-14.9	14949	-0.17	127	-13.12	14775	-0.26	118	-17.5	14886
PSAP	-1.45	1.74E-13	-0.21	121	-14.9	14950	-0.2	113	-12.82	14714	-0.34	156	-35.55	16440
CDC42BPB	-1.21	4.49E-08	-0.23	116	-14.9	14951	-0.18	145	-17.05	15597	-0.23	146	-22	15661
HLX	-1.26	1.08E-06	-0.23	116	-14.91	14952	-0.27	125	-20.15	16007	-0.17	110	-10.41	13059
GUCY1B3	-1.43	3.54E-10	-0.18	135	-14.91	14953	-0.04	134	-3.42	10744	-0.18	143	-16.55	14698
PAK7	1	0.928839418	-0.19	129	-14.91	14954	-0.05	124	-3.45	10765	-0.21	123	-15.46	14469
NFKBIA	-1.42	2.64E-08	-0.21	120	-14.91	14955	-0.23	128	-18.15	15755	-0.27	124	-19.93	15312

LPAR6	-1.58	4.95E-14	-0.22	118	-14.92	14956	-0.09	120	-6.2	12284	-0.29	112	-18.12	15001
RARB	-1.29	8.47E-15	-0.17	137	-14.92	14957	-0.05	138	-4.65	11466	-0.24	129	-18.64	15106
HGF	-1.58	1.37E-21	-0.33	95	-14.92	14958	-0.17	101	-8.45	13302	-0.39	110	-23.48	15829
S100A16	-1.07	0.224983643	-0.16	144	-14.92	14959	-0.02	123	-1.62	9572	-0.14	103	-7.2	11921
P2RY1	-1.12	2.31E-06	-0.2	125	-14.93	14960	0	115	0.13	8376	-0.16	123	-11.46	13389
CA3	-1.14	0.003797631	-0.16	145	-14.93	14961	-0.06	118	-4.09	11144	-0.22	131	-17.48	14877
HK3_t1	-1.71	4.34E-30	-0.31	98	-14.93	14962	NA	NA	NA	NA	-0.45	118	-30.91	16328
SORCS2	-1.14	0.04603497	-0.21	121	-14.94	14963	-0.2	130	-16.18	15455	-0.07	111	-4.47	10828
SRSF8_t1	-1.15	4.77E-13	-0.17	137	-14.94	14964	NA	NA	NA	NA	-0.2	150	-19.96	15321
SLC4A10	-1.04	0.000621017	-0.18	135	-14.94	14965	-0.03	144	-2.36	10056	-0.2	138	-18.02	14983
RRAS2	-1	0.964247646	-0.15	150	-14.95	14966	-0.02	135	-1.63	9577	0.03	133	2.71	7080
CARD6	-1.49	8.86E-07	-0.18	133	-14.96	14967	-0.03	107	-1.75	9658	-0.27	133	-22.08	15669
Mar-02	-1.71	1.20E-11	-0.25	110	-14.96	14968	-0.31	125	-23.6	16283	-0.2	119	-13.75	14037
CRTAM	-1.35	3.53E-08	-0.18	135	-14.96	14969	-0.04	134	-3.18	10589	-0.31	127	-23.71	15851
GLYAT_t1	-2.03	9.95E-14	-0.25	111	-14.97	14970	-0.28	114	-17.93	15723	-0.17	119	-12.02	13550
LMO4	-1.1	0.064340358	-0.16	146	-14.97	14971	0.02	159	2.62	6742	-0.22	149	-21.54	15600
TTC8	-1.2	0.000159928	-0.18	131	-14.97	14972	-0.03	107	-1.8	9691	-0.2	110	-12.17	13598
CD4	-1.41	4.36E-17	-0.17	140	-14.98	14973	-0.1	126	-7.91	13076	-0.26	142	-24.26	15897
CYP2A7	-7.3	4.23E-19	-0.3	100	-14.99	14974	-0.37	90	-14.75	15156	-0.31	104	-16.83	14763
AK2_t2	-1.11	0.013747741	-0.17	136	-15	14975	-0.15	130	-11.85	14463	-0.04	158	-4.35	10778
KRTAP1-5	-1.06	1.10E-05	-0.2	125	-15	14976	-0.12	136	-10.1	13919	-0.11	134	-8.83	12537
FBLN5	-1.79	9.15E-21	-0.19	127	-15.01	14977	-0.13	123	-9.25	13605	-0.33	119	-22.79	15755
NDFIP2	-1.27	5.88E-06	-0.14	155	-15.01	14978	-0.08	140	-7.31	12796	-0.16	152	-16.04	14599
PRRT1	-1.15	2.54E-10	-0.23	114	-15.01	14979	-0.28	145	-26.43	16401	-0.22	108	-12.67	13753
MFSD8	-1.21	4.76E-08	-0.16	144	-15.01	14980	-0.23	144	-21.19	16110	-0.13	131	-10.6	13116
PAH	-2.19	1.12E-07	-0.25	109	-15.02	14981	-0.23	120	-15.8	15364	-0.17	124	-12.29	13640
URGCP	-1.16	6.08E-05	-0.22	118	-15.03	14982	-0.2	138	-17.22	15623	-0.2	100	-9.92	12903
PDE4B_t2	-1.37	1.20E-13	-0.14	155	-15.03	14983	-0.16	136	-13.72	14927	-0.25	151	-24.75	15941
PEX14	-1.39	5.41E-11	-0.25	111	-15.03	14984	-0.23	139	-20.62	16060	-0.28	120	-19.58	15259
PROZ	-2.84	2.21E-22	-0.3	100	-15.03	14985	-0.25	118	-16.88	15575	-0.28	116	-18.38	15058
FAS	-1.47	7.39E-09	-0.2	125	-15.04	14986	-0.14	124	-10.59	14104	-0.13	125	-9.44	12738
METTL14	-1.39	7.75E-11	-0.21	120	-15.04	14987	-0.2	118	-13.59	14888	-0.2	153	-21.05	15508
SLC44A2	-1.57	1.46E-15	-0.21	120	-15.04	14988	-0.11	135	-9.6	13737	-0.32	110	-19.42	15234
GPR153	-1.3	2.79E-05	-0.18	134	-15.05	14989	-0.21	145	-19.79	15959	-0.17	141	-15.44	14467
MARCO	-9.98	5.37E-59	-0.4	88	-15.05	14990	-0.31	104	-16.92	15579	-0.49	115	-31.6	16345
SMPD1	-1.38	2.39E-10	-0.23	116	-15.06	14991	-0.25	115	-16.52	15515	-0.15	111	-9.17	12649
HEY2_t1	-1.16	4.91E-09	-0.18	132	-15.06	14992	NA	NA	NA	NA	NA	NA	NA	NA
FEZ1	-1.47	1.26E-21	-0.25	110	-15.06	14993	-0.2	118	-13.27	14817	-0.37	110	-21.9	15641
FAM46C	-1.16	2.73E-08	-0.16	146	-15.07	14994	-0.09	154	-9.73	13788	-0.33	139	-29.27	16266
TNF_t1	-1.06	1.72E-05	-0.19	129	-15.07	14995	NA	NA	NA	NA	-0.22	158	-23.75	15853
FUT5	-1.32	1.76E-06	-0.2	126	-15.07	14996	-0.06	126	-4.7	11489	-0.18	117	-12.14	13588
ATP11C_t1	-1.53	2.10E-06	-0.14	154	-15.07	14997	-0.16	129	-12.55	14658	-0.09	132	-7.6	12071
PRAM1	-1.49	3.44E-23	-0.28	103	-15.07	14998	-0.19	84	-6.58	12455	-0.44	106	-24.46	15911
KRT222	-1.49	5.00E-05	-0.2	126	-15.08	14999	-0.1	126	-7.81	13035	-0.12	113	-7.73	12130
UCP3_t2	-1.18	2.51E-09	-0.22	120	-15.08	15000	-0.18	140	-16.58	15524	-0.21	145	-20.36	15387
MAD1L1	-1.26	3.00E-05	-0.26	108	-15.09	15001	-0.26	107	-14.75	15160	-0.2	100	-9.79	12844
SGCB	-1.61	5.11E-08	-0.12	171	-15.09	15002	-0.01	146	-1.36	9407	-0.25	154	-26.48	16084
VKORC1	-1.42	5.67E-12	-0.23	116	-15.09	15003	-0.21	111	-12.54	14652	-0.21	113	-13.26	13913
SLC43A3	-1.28	0.000307154	-0.19	130	-15.09	15004	-0.23	107	-13.23	14809	-0.06	143	-5.72	11328
ULK1_t1	-1.01	0.912433981	-0.14	155	-15.09	15005	NA	NA	NA	NA	NA	NA	NA	NA
GZMK	-2.43	1.19E-17	-0.25	110	-15.1	15006	-0.11	116	-6.97	12636	-0.37	147	-35.66	16444
WEE1	-1.3	7.80E-06	-0.15	150	-15.11	15007	-0.12	138	-10.6	14108	-0.13	143	-11.69	13448
BAIAP2_t2	-1.59	1.62E-10	-0.27	106	-15.12	15008	-0.17	123	-12.24	14568	-0.26	140	-23.36	15808
PCK2	-1.93	1.67E-08	-0.28	104	-15.12	15009	-0.35	104	-18.94	15858	-0.19	107	-10.58	13109
PLAA	-1.05	0.167780476	-0.16	146	-15.13	15010	-0.1	140	-9.33	13630	0.05	140	4.74	6059
SDSL	-1.62	3.62E-06	-0.23	116	-15.14	15011	-0.27	117	-17.78	15707	-0.11	98	-5.27	11145
SLC9A9	-1.15	4.08E-13	-0.17	137	-15.14	15012	-0.15	144	-14.3	15051	-0.29	112	-18.22	15026
KYNU_t2	-2.13	4.73E-18	-0.25	110	-15.14	15013	-0.16	134	-13.22	14807	-0.25	112	-15.71	14521
DYRK1A_t2	-1.14	1.42E-09	-0.19	128	-15.15	15014	-0.29	142	-26.82	16409	-0.12	128	-9.04	12598
IGFBP1	-3.91	2.68E-12	-0.2	124	-15.15	15015	-0.26	144	-24.26	16317	-0.26	131	-21.25	15544
F3	-1.36	3.71E-09	-0.24	114	-15.15	15016	-0.1	122	-6.85	12583	-0.29	151	-28.86	16252
BMP5	-1.5	7.12E-24	-0.3	100	-15.16	15017	-0.25	97	-11.76	14436	-0.36	91	-14.64	14281
PCBD1	-1.44	3.14E-08	-0.24	112	-15.16	15018	-0.28	120	-19.57	15937	-0.19	120	-13.61	14001
GPR83	-1.07	7.98E-08	-0.2	124	-15.16	15019	-0.07	148	-7.16	12721	-0.24	135	-20.37	15390
PSMA2	-1.24	3.83E-05	-0.2	126	-15.16	15020	-0.19	107	-10.88	14202	-0.06	146	-5.88	11396
LAMC2_t2	-1.28	0.002997064	-0.21	123	-15.18	15021	-0.03	139	-2.72	10289	-0.28	140	-24.93	15953
PLD1	-1.31	2.34E-09	-0.17	140	-15.19	15022	-0.18	126	-13.34	14838	-0.17	143	-16.25	14637
MS4A6A_t1	-3.06	7.29E-29	-0.24	114	-15.19	15023	-0.16	114	-10.47	14055	-0.37	122	-26.79	16111
RBL2_t1	-1.38	2.53E-10	-0.22	118	-15.2	15024	-0.15	132	-12.17	14553	NA	NA	NA	NA
ACSM2B	-2.1	9.41E-07	-0.22	118	-15.2	15025	-0.25	108	-14.71	15149	-0.21	122	-14.77	14317
GAS6	-1.78	4.60E-16	-0.26	109	-15.2	15026	-0.16	111	-9.75	13797	-0.35	100	-17.68	14919
MPC1	-1.99	1.00E-16	-0.21	121	-15.21	15027	-0.17	116	-11.36	14334	-0.27	123	-19.72	15286
SELL	-1.21	8.21E-11	-0.15	150	-15.22	15028	-0.13	134	-10.55	14084	-0.29	140	-26.3	16071
UBXN8	-1.71	8.50E-13	-0.23	117	-15.24	15029	-0.23	110	-14.09	15009	-0.25	123	-17.9	14963
ATF5	-2.57	6.58E-13	-0.24	113	-15.25	15030	-0.22	123	-15.88	15384	-0.28	108	-16.53	14692
CRISPLD2_t1	-1.76	4.52E-19	-0.2	126	-15.26	15031	NA	NA	NA	NA	-0.29	128	-22.39	15710
MME_t1	-1.69	0.000129807	-0.14	162	-15.26	15032	NA	NA	NA	NA	NA	NA	NA	NA
BCHE	-4.48	2.02E-17	-0.3	101	-15.26	15033	-0.24	128	-18.5	15798	-0.31	111	-18.73	15126
SYT13	-1.43	0.000158113	-0.2	127	-15.27	15034	-0.12	132	-9.5	13703	-0.24	138	-21.37	15563
FZD8	-1.48	7.81E-11	-0.19	130	-15.27	15035	-0.12	123	-8.93	13477	-0.26	138	-22.84	15761
TMEM100	-1.61	1.07E-09	-0.2	126	-15.27	15036	-0.09	122	-6.26	12310	-0.24	128	-18.83	15142
BLK	-1.18	7.69E-06	-0.29	103	-15.27	15037	-0.13	117	-8.72	13405	-0.23	106	-13.1	13870
XRCC5	-1.22	3.73E-06	-0.2	127	-15.27	15038	-0.21	143	-19.78	15958	-0.19	115	-12.16	13593
TPPP2	-2.53	2.45E-21	-0.32	97	-15.27	15039	-0.32	90	-12.84	14721	-0.33	90	-13.24	13908
CNGA1	-1.77	1.85E-12	-0.21	124	-15.28	15040	-0.25	109	-14.51	15104	-0.19	105	-10.48	13083
APOH	-2.13	2.05E-06	-0.24	115	-15.28	15041	-0.34	134	-28.88	16452	-0.19	112	-11.84	13499
VWA8_t2	-1.17	2.67E-06	-0.18	133	-15.28	15042	-0.15	134	-12.39	14612	-0.15	130	-11.98	13534
SRSF4_t1	-1.22	4.57E-17	-0.19	130	-15.28	15043	-0.11	112	-6.88	12597	NA	NA	NA	NA
DECR1	-1.44	6.06E-07	-0.25	111	-15.29	15044	-0.21	88	-7.85	13051	-0.09	101	-4.84	10994
UGT2B15	-3.17	5.47E-11	-0.23	117	-15.29	15045	-0.29	102	-15.12	15230	-0.15	108	-8.66	12481
NME5	-1.34	3.68E-09	-0.21	122	-15.31	15046	-0.04	128	-2.88	10401	-0.31	104	-16.5	14681
TBC1D15	-1.22	9.23E-06	-0.15	154	-15.32	15047	-0.07	130	-5.74	12047	-0.16	141	-14.93	14365
DNMT3L	-1.97	6.98E-09	-0.28	104	-15.32	15048	-0.23	100	-11.7	14423	-0.23	80	-6.93	11816
TMEM26_t1	-1.08	1.28E-10	-0.17	141	-15.32	15049	NA	NA	NA	NA	-0.26	138	-22.71	15746
RAB25	-1.4	1.25E-07	-0.32	98	-15.33	15050	-0.14	115	-9.06	13534	-0.38	120	-26.93	16125
PPP2R2A	-1.61	9.97E-19	-0.2	127	-15.33	15051	-0.23	134	-19.63	15946	-0.29	129	-23.16	15791
BGN	-1.76	5.58E-25	-0.29	103	-15.34	15052	-0.19	120	-13.02	14759	-0.35	111	-21.21	15541
PRCP	-1.95	5.30E-22	-0.24	115	-15.37	15053	-0.26	139	-22.72	16230	-0.37	127	-28.75	16243
SGPP1	-1.28	4.65E-13	-0.18	136	-15.37	15054	-0.12	128	-9.11	13561	-0.23	154	-23.86	15863
PHYH	-2.21	3.00E-14	-0.24	113	-15.37	15055	-0.33	119	-22.86	16244	-0.24	114	-15.44	14464
RAD54L2	-1.34	1.65E-09	-0.28	104	-15.38	15056	-0.19	104	-10.32	14010	-0.3	96	-13.8	14052
RNF123	-1.3	1.28E-09	-0.27	106	-15.38	15057	-0.28	96	-13.08	14769	-0.22	113	-13.68	14018
KLK10	-1.04	0.00431242	-0.18	134	-15.39	15058	-0.09	147	-8.95	13482	-0.1	156	-10.84	13194
GDF6_t1	-1.14	1.12E-11	-0.21	125	-15.39	15059	NA	NA	NA	NA	-0.22	120	-15.36	14451
C1QTNF7_t1	-1.11	2.15E-05	-0.21	124	-15.39	15060	NA	NA	NA	NA	-0.24	127	-18.55	15091
ANO3	-1.35	2.73E-13	-0.17	141	-15.4	15061	-0.16	153	-16.74	15556	-0.19	139	-17.23	14835

SLA2	-1.15	4.25E-08	-0.21	123	-15.4	15062	-0.11	119	-7.25	12760	-0.32	123	-23.62	15843
CTAGE5_t2	-1.3	1.05E-08	-0.23	118	-15.4	15063	-0.19	126	-14.15	15017	-0.11	131	-8.95	12577
MFRP	-1.19	9.00E-10	-0.22	121	-15.41	15064	-0.25	130	-19.85	15966	-0.2	114	-12.74	13774
LARP1B_t2	-1.38	5.74E-15	-0.2	126	-15.41	15065	-0.23	101	-11.92	14484	-0.16	123	-12.04	13557
LDLR_t1	-2.03	5.56E-18	-0.17	143	-15.42	15066	NA	NA	NA	NA	-0.31	168	-36.59	16455
TARP	-1.51	2.21E-11	-0.23	118	-15.42	15067	-0.07	128	-5.3	11818	-0.32	110	-19	15174
SERTAD1	-2.44	1.10E-24	-0.25	111	-15.42	15068	-0.23	92	-9.75	13799	-0.39	99	-19.24	15209
MACC1	-1.1	1.92E-05	-0.17	140	-15.42	15069	-0.08	132	-6.27	12317	-0.28	151	-27.86	16178
SHC1_t2	-1.21	4.07E-06	-0.23	118	-15.44	15070	-0.22	127	-16.59	15526	-0.21	118	-14.24	14170
LY6E	-2.87	2.95E-30	-0.29	104	-15.44	15071	-0.2	119	-14.01	14993	-0.47	120	-32.82	16386
LTB4R2_t1	-1.17	2.62E-15	-0.18	135	-15.45	15072	NA	NA	NA	NA	-0.23	107	-13.2	13896
IL10	-1.15	5.07E-06	-0.18	136	-15.46	15073	-0.11	119	-7.29	12782	-0.2	114	-12.89	13814
HSD17B8	-1.49	1.24E-08	-0.24	114	-15.46	15074	-0.31	104	-16.92	15581	-0.23	93	-9.72	12816
MYO16_t1	-1.08	0.003881299	-0.17	141	-15.46	15075	NA	NA	NA	NA	-0.13	136	-11.49	13396
ROBO4_t1	-1.63	3.47E-15	-0.22	120	-15.46	15076	-0.16	120	-11.4	14344	NA	NA	NA	NA
RAB20	-1.64	1.56E-17	-0.19	131	-15.48	15077	-0.21	122	-15.24	15259	-0.31	124	-23.21	15795
HMGCLL1	-1.17	5.52E-16	-0.23	116	-15.49	15078	-0.14	121	-9.67	13764	-0.22	121	-15.65	14509
PPFIBP1_t1	-1.4	7.34E-16	-0.19	130	-15.49	15079	NA	NA	NA	NA	-0.22	139	-19.52	15254
SALL1	-1.41	6.44E-05	-0.16	149	-15.49	15080	-0.19	128	-14.66	15139	-0.17	129	-13.26	13914
GPR156	-1.25	1.33E-07	-0.18	136	-15.49	15081	-0.22	130	-17.67	15692	-0.19	136	-15.96	14578
GIMAP7	-1.99	1.49E-16	-0.26	109	-15.49	15082	-0.12	98	-5.75	12056	-0.38	115	-24.64	15928
EPM2A_t2	-1.13	2.81E-09	-0.18	137	-15.5	15083	-0.13	123	-9.19	13589	-0.1	154	-10.13	12966
ICAM2	-1.96	2.16E-18	-0.29	104	-15.5	15084	-0.16	101	-7.93	13088	-0.38	117	-25.34	15982
CCDC146	-2.2	2.19E-13	-0.2	128	-15.5	15085	-0.13	134	-10.52	14071	-0.26	127	-19.66	15275
ETFA_t1	-1.36	2.11E-06	-0.22	120	-15.5	15086	NA	NA	NA	NA	NA	NA	NA	NA
CWC15	-1.11	0.001634932	-0.16	145	-15.51	15087	-0.06	134	-4.69	11485	-0.18	133	-15	14376
ITFG1	-1.29	2.05E-07	-0.18	134	-15.51	15088	-0.17	117	-11.37	14338	-0.12	113	-7.85	12182
MORC3	-1.37	2.40E-08	-0.19	132	-15.51	15089	-0.13	164	-14.37	15073	-0.18	128	-14.38	14210
MLYCD	-1.63	2.81E-13	-0.2	126	-15.51	15090	-0.25	136	-21.26	16116	-0.26	137	-22.43	15712
TCF21	-1.73	5.84E-25	-0.3	102	-15.52	15091	-0.15	118	-10.24	13976	-0.42	129	-32.83	16388
NR1H4	-1.59	1.27E-09	-0.24	116	-15.52	15092	-0.16	111	-9.78	13811	-0.11	126	-8.51	12430
DFNB59	-1.2	3.56E-06	-0.22	119	-15.52	15093	-0.11	95	-5.04	11674	-0.05	99	-2.48	9938
LGI1	-1.07	0.008132298	-0.22	122	-15.53	15094	-0.04	123	-2.91	10423	-0.15	122	-10.44	13072
LATS2	-1.64	4.76E-19	-0.19	133	-15.54	15095	-0.21	128	-16.56	15519	-0.32	131	-25.83	16018
PCDH9_t1	-1.17	3.17E-06	-0.16	146	-15.55	15096	-0.04	143	-3.61	10890	-0.22	155	-23.48	15828
RUNX3	-1.64	5.36E-14	-0.21	123	-15.55	15097	-0.08	143	-7.63	12944	-0.37	144	-34.69	16428
SYNE2_t2	-1.18	1.78E-11	-0.22	121	-15.56	15098	-0.21	124	-15.35	15279	NA	NA	NA	NA
DPYD	-1.47	2.61E-10	-0.17	142	-15.57	15099	-0.23	117	-15.34	15278	-0.12	124	-9.04	12600
MRPL19	-1.21	0.0006822	-0.18	135	-15.57	15100	-0.11	148	-11.16	14286	-0.11	137	-9.2	12654
MMP7	-2.82	5.83E-08	-0.19	133	-15.57	15101	0.01	136	0.74	7974	-0.29	137	-25.19	15969
LYRM1	-1.36	5.53E-10	-0.22	120	-15.57	15102	-0.14	129	-10.95	14222	-0.12	121	-8.2	12317
TMEM133	-1.29	1.84E-09	-0.19	130	-15.57	15103	-0.14	123	-10.53	14076	-0.1	131	-7.9	12194
SLC25A18	-2.59	2.64E-17	-0.24	114	-15.57	15104	-0.27	132	-21.82	16154	-0.28	136	-23.75	15854
ETV3	-1.02	0.259879304	-0.13	168	-15.57	15105	-0.12	134	-10.38	14031	-0.04	113	-2.64	10008
IL16_t1	-1.43	1.87E-17	-0.2	128	-15.58	15106	-0.1	146	-9.4	13660	-0.32	128	-25.33	15979
PTPN9	-1.13	1.62E-08	-0.18	138	-15.58	15107	-0.12	136	-10.48	14060	-0.22	126	-16.62	14718
MAB21L1	-1.01	0.114665839	-0.16	146	-15.59	15108	-0.03	155	-3.66	10913	-0.08	132	-6.56	11671
ACCS	-1.32	4.56E-09	-0.26	111	-15.59	15109	-0.13	123	-9.44	13675	-0.28	102	-14.33	14197
TEK	-3	7.46E-28	-0.25	113	-15.59	15110	-0.23	135	-19.27	15896	-0.31	147	-30.45	16310
KIR2DS2	-1.11	9.32E-09	-0.19	130	-15.59	15111	-0.15	145	-14.18	15025	-0.25	119	-17.56	14896
CYP3A7	-2.19	1.70E-07	-0.21	126	-15.6	15112	-0.3	125	-22.44	16210	-0.15	119	-10.1	12957
LMO2	-1.65	9.75E-16	-0.26	111	-15.6	15113	-0.13	127	-9.84	13824	-0.31	108	-18.05	14985
SLCO1B3	-8.29	1.89E-18	-0.23	117	-15.61	15114	-0.23	121	-16.29	15474	-0.29	134	-24.22	15894
CCRN4L_t1	-1.15	3.23E-12	-0.22	120	-15.61	15115	NA	NA	NA	NA	-0.13	140	-11.81	13493
INHBE	-1.34	0.004038252	-0.22	120	-15.62	15116	-0.12	113	-7.83	13041	-0.12	132	-9.55	12771
INPP1	-1.56	3.02E-14	-0.2	129	-15.62	15117	-0.24	123	-17.38	15658	-0.26	126	-19.89	15308
MMP19	-2.43	1.67E-14	-0.19	131	-15.62	15118	-0.02	128	-1.6	9558	-0.28	144	-25.86	16022
TNRC6A	-1.01	0.586150747	-0.14	159	-15.63	15119	-0.03	156	-2.83	10361	-0.05	161	-5.63	11288
NKX3-1	-1.07	0.039642502	-0.17	140	-15.63	15120	-0.13	116	-8.51	13328	-0.13	133	-10.92	13229
PEX11A	-1.31	1.07E-06	-0.17	144	-15.64	15121	-0.14	132	-11.12	14274	-0.15	138	-13.49	13976
CCDC90B	-1.24	4.82E-05	-0.18	138	-15.64	15122	-0.09	108	-5.3	11820	-0.18	130	-14	14113
TGDS	-1.95	4.98E-14	-0.22	122	-15.64	15123	-0.23	123	-16.56	15520	-0.21	122	-15.32	14444
PNPLA5	-1.15	3.75E-14	-0.17	143	-15.65	15124	-0.25	112	-15.72	15346	-0.16	104	-8.39	12393
KCNAB1_t1	-1.11	8.56E-07	-0.2	130	-15.66	15125	NA	NA	NA	NA	-0.18	142	-16.69	14735
SULT1E1	-2.11	1.74E-11	-0.19	132	-15.67	15126	-0.21	129	-16.9	15577	-0.21	131	-17.19	14831
IBTK_t1	-1.16	0.00042367	-0.18	135	-15.67	15127	NA	NA	NA	NA	NA	NA	NA	NA
RGAG4	-1.42	3.10E-09	-0.2	127	-15.68	15128	-0.14	137	-12.17	14554	-0.35	140	-31.77	16356
ATP8B3	-1.39	2.31E-12	-0.29	104	-15.68	15129	-0.21	123	-15.1	15223	-0.29	126	-22.26	15698
CABP7	-1.48	3.70E-13	-0.19	133	-15.69	15130	-0.26	112	-16.11	15444	-0.2	125	-15.35	14450
HOXB6	-1.51	1.76E-09	-0.22	120	-15.69	15131	-0.13	113	-8.02	13122	-0.29	105	-15.98	14584
IBA57	-1.09	7.01E-07	-0.17	144	-15.69	15132	-0.22	140	-19.56	15936	-0.15	160	-16.63	14719
CD69	-2.81	1.88E-15	-0.2	127	-15.69	15133	-0.08	121	-5.51	11922	-0.31	163	-34.97	16434
PRF1	-1.28	1.22E-10	-0.25	113	-15.7	15134	-0.14	112	-8.62	13367	-0.36	110	-21.85	15636
PEX12	-1.03	0.521902037	-0.15	155	-15.71	15135	-0.03	134	-2.14	9925	-0.02	136	-1.33	9354
NCOA4	-1.26	1.20E-05	-0.16	146	-15.71	15136	-0.22	138	-19.54	15933	-0.14	124	-10.2	12990
CD83	-1.94	8.80E-16	-0.18	138	-15.71	15137	-0.03	147	-2.56	10190	-0.33	126	-24.9	15951
F7_t1	-1.24	4.07E-07	-0.24	116	-15.71	15138	NA	NA	NA	NA	-0.27	120	-18.96	15166
DENND3	-1.47	3.07E-14	-0.21	124	-15.71	15139	-0.06	124	-4.7	11490	-0.37	122	-26.95	16127
FUOM	-1.93	2.63E-12	-0.25	112	-15.71	15140	-0.29	108	-16.88	15574	-0.24	108	-13.72	14028
EDN2	-1.43	6.69E-10	-0.26	111	-15.71	15141	-0.22	119	-15.17	15240	-0.33	120	-23.18	15792
MGRN1	-1.21	4.02E-05	-0.23	119	-15.72	15142	-0.1	138	-9.07	13538	-0.18	121	-12.78	13785
ADRB2	-1.6	6.51E-10	-0.2	129	-15.72	15143	-0.21	125	-15.79	15361	-0.25	118	-16.88	14777
CDC14B_t1	-1.32	7.91E-08	-0.21	124	-15.72	15144	NA	NA	NA	NA	-0.15	134	-12.63	13742
TRAT1	-1.41	1.70E-11	-0.23	118	-15.72	15145	-0.06	125	-4.83	11553	-0.31	129	-24.75	15942
PRDX3	-1.46	2.00E-08	-0.14	159	-15.73	15146	-0.2	134	-16.56	15521	-0.07	142	-6.04	11449
RPS9	-1.32	5.24E-14	-0.22	120	-15.73	15147	-0.2	88	-7.62	12939	-0.3	84	-10.05	12942
ATHL1	-1.21	0.003188397	-0.26	111	-15.73	15148	-0.1	122	-7.12	12709	-0.2	102	-10.37	13039
KRTAP9-4	-1.18	2.13E-10	-0.14	159	-15.74	15149	-0.3	116	-19.71	15954	-0.19	129	-15.32	14443
CCDC125	-1.09	0.052914279	-0.18	136	-15.76	15150	-0.09	136	-7.91	13075	-0.08	155	-8.28	12356
ITGAD_t1	-2.7	3.09E-23	-0.21	125	-15.76	15151	-0.18	118	-12.23	14565	NA	NA	NA	NA
ARHGAP25	-1.98	3.29E-20	-0.28	107	-15.77	15152	-0.11	112	-6.71	12519	-0.42	104	-22.77	15750
PTGS1	-1.57	1.51E-14	-0.18	136	-15.77	15153	-0.13	150	-12.78	14710	-0.3	136	-26.14	16047
KLF11	-1.31	6.98E-11	-0.15	154	-15.77	15154	-0.19	143	-17.96	15730	-0.21	152	-21.19	15535
BASP1	-3.07	3.31E-22	-0.26	111	-15.77	15155	-0.13	127	-9.71	13781	-0.39	120	-27.24	16147
SGSM2	-1.24	3.37E-09	-0.24	115	-15.77	15156	-0.16	111	-10.03	13891	-0.32	115	-20.59	15426
ACY1	-1.48	9.55E-08	-0.28	106	-15.77	15157	-0.29	100	-14.39	15078	-0.15	99	-7.45	12011
EEF2	-1.24	2.14E-08	-0.23	120	-15.78	15158	-0.18	130	-14.61	15123	-0.24	104	-13.08	13861
ZBTB48	-1.24	1.75E-08	-0.29	104	-15.79	15159	-0.25	93	-10.92	14213	-0.31	126	-23.22	15796
TMEM53	-1.3	1.78E-07	-0.24	116	-15.79	15160	-0.27	121	-18.88	15850	-0.23	123	-16.87	14775
ABCG4	-1.07	0.000396788	-0.22	123	-15.79	15161	-0.05	127	-4.01	11102	-0.21	120	-14.48	14233
BRD1	-1.22	5.23E-08	-0.21	127	-15.8	15162	-0.04	145	-3.41	10734	-0.28	135	-23.57	15838
GPT	-1.78	1.15E-11	-0.23	120	-15.8	15163	-0.31	100	-15.63	15329	-0.19	122	-13.74	14035
WDR66_t1	-1.65	3.39E-22	-0.34	97	-15.8	15164	NA	NA	NA	NA	-0.41	109	-24.14	15882
BAIAP2_t3	-1.18	2.55E-05	-0.21	127	-15.82	15165	-0.2	123	-14.82	15174	-0.25	140	-22.09	15674
GSPT2	-1.76	9.67E-13	-0.16	148	-15.82	15166	-0.12	127	-8.99	13498	-0.29	115	-18.89	15155
RAB36	-1.19	8.51E-10	-0.26	112	-15.83	15167	-0.17	133	-14.23	15036	-0.27	146	-26.14	16048

LETMD1	-1.04	0.212605961	-0.17	144	-15.83	15168	-0.07	124	-4.93	11619	-0.16	111	-9.66	12797
MS4A6A_t2	-3.26	1.66E-30	-0.27	109	-15.83	15169	-0.18	104	-9.86	13831	-0.43	127	-33.41	16401
AFM	-6.59	7.58E-21	-0.38	92	-15.84	15170	-0.34	102	-17.88	15719	-0.29	115	-18.92	15158
EMILIN1	-2.46	1.91E-26	-0.29	104	-15.84	15171	-0.16	119	-10.74	14154	-0.41	101	-21.07	15514
LURAP1L	-1.79	5.36E-13	-0.19	133	-15.84	15172	-0.12	129	-9.75	13798	-0.25	140	-22.06	15666
TERF2IP	-1.29	2.98E-14	-0.2	130	-15.84	15173	-0.14	103	-7.47	12863	-0.3	128	-23.39	15814
DSEL_t1	-1.16	9.80E-09	-0.2	129	-15.85	15174	NA	NA	NA	NA	-0.19	144	-17.8	14945
MYADML	-1.11	7.92E-08	-0.19	135	-15.85	15175	-0.18	126	-13.78	14942	-0.15	124	-10.94	13230
LPHN2	-1.44	1.12E-14	-0.21	127	-15.86	15176	-0.1	127	-7.68	12970	-0.27	127	-21	15502
BIRC2	-1.25	1.92E-05	-0.2	130	-15.86	15177	-0.13	126	-9.85	13829	-0.08	124	-5.91	11411
CRYL1	-1.92	3.70E-11	-0.23	118	-15.87	15178	-0.28	120	-19.41	15915	-0.28	142	-25.93	16026
FSD1	-1.18	3.46E-08	-0.22	121	-15.88	15179	-0.27	118	-18.17	15757	-0.16	102	-8.1	12266
EXPH5	-1.9	2.14E-10	-0.19	132	-15.88	15180	-0.16	137	-14.1	15010	-0.2	125	-15.31	14442
NEK9	-1.07	0.000943505	-0.17	145	-15.88	15181	-0.17	127	-13.36	14841	-0.08	155	-8.54	12437
INVS	-1.21	7.31E-08	-0.25	113	-15.88	15182	-0.14	108	-7.92	13077	-0.2	120	-14.14	14147
IL6	-1.76	3.73E-09	-0.23	120	-15.88	15183	-0.13	119	-8.67	13382	-0.31	128	-24.28	15901
ARHGAP9	-1.94	1.75E-15	-0.22	123	-15.89	15184	-0.07	104	-3.59	10871	-0.36	95	-16.25	14634
SLC6A12	-2.02	2.33E-10	-0.31	101	-15.89	15185	-0.27	123	-19.96	15982	-0.17	126	-13.09	13863
KHK	-1.95	2.31E-09	-0.27	108	-15.89	15186	-0.31	120	-22.01	16170	-0.24	111	-14.75	14309
MAFF	-1.84	3.60E-17	-0.21	127	-15.89	15187	-0.12	135	-10.05	13900	-0.27	155	-28.17	16199
NOSTRIN	-2.7	5.32E-26	-0.24	115	-15.89	15188	-0.21	143	-19.71	15952	-0.29	131	-23.63	15845
FAM228A_t1	-1.22	7.87E-14	-0.24	116	-15.9	15189	NA	NA	NA	NA	-0.3	125	-22.65	15733
APOBEC3F	-1.28	7.50E-10	-0.17	144	-15.9	15190	-0.03	137	-2.47	10134	-0.36	123	-26.12	16044
ERN2	-1.07	0.001350454	-0.2	128	-15.9	15191	-0.15	131	-12.39	14611	-0.14	121	-10.14	12972
MS4A1	-1.18	1.18E-06	-0.19	133	-15.9	15192	-0.09	137	-7.69	12977	-0.22	150	-22.39	15709
FAM9B	-1.73	1.36E-10	-0.23	120	-15.91	15193	-0.19	126	-14.62	15126	-0.18	126	-13.31	13923
FAM160A2	-1.23	4.02E-08	-0.27	110	-15.92	15194	-0.18	97	-8.64	13370	-0.28	111	-16.9	14785
FAM131C	-1.28	1.40E-11	-0.21	127	-15.92	15195	-0.2	120	-13.9	14969	-0.24	103	-12.82	13797
ELOVL6	-1.46	8.27E-06	-0.2	130	-15.92	15196	-0.08	133	-6.54	12433	-0.17	131	-13.96	14100
MARK2_t2	-1.18	7.07E-11	-0.23	120	-15.92	15197	-0.24	147	-23.39	16268	-0.28	121	-20.12	15351
COQ9	-1.25	3.42E-07	-0.26	112	-15.93	15198	-0.24	119	-16.31	15475	-0.15	120	-10.46	13078
GLYCTK	-1.38	8.87E-05	-0.2	130	-15.93	15199	-0.24	120	-16.8	15564	-0.1	126	-7.52	12041
CYP3A43_t1	-1.81	1.85E-10	-0.23	120	-15.93	15200	-0.22	134	-18.78	15836	-0.21	123	-15.18	14415
MST1	-1.64	1.36E-07	-0.32	100	-15.95	15201	-0.24	96	-10.94	14220	-0.27	84	-9.03	12596
NTS	-1.25	0.114164075	-0.23	120	-15.95	15202	-0.07	112	-4.32	11273	-0.27	129	-21.28	15554
TAT_t1	-2.59	1.13E-16	-0.21	126	-15.95	15203	NA	NA	NA	NA	-0.26	123	-18.89	15152
ANGPTL7	-1.01	0.755898739	-0.22	124	-15.96	15204	-0.08	108	-4.86	11567	-0.19	103	-10.27	13014
NUDT10	-1.11	2.83E-11	-0.18	137	-15.97	15205	-0.12	155	-12.88	14728	-0.26	142	-24.14	15884
PTPN13	-1.91	2.24E-13	-0.15	154	-15.98	15206	-0.04	123	-2.64	10244	-0.25	106	-14.23	14166
PRSS8	-1.15	1.38E-08	-0.23	120	-15.98	15207	-0.13	131	-10.3	14002	-0.22	102	-11.46	13387
CTAGE15P	-1.27	1.67E-07	-0.22	122	-15.98	15208	-0.25	106	-13.85	14956	-0.14	154	-14.87	14347
TBXAS1	-1.91	3.07E-23	-0.25	113	-15.98	15209	-0.18	115	-11.42	14348	-0.42	143	-38.72	16474
SMARCA2	-1.42	4.49E-13	-0.25	113	-15.98	15210	-0.16	114	-9.95	13865	-0.22	139	-19.52	15252
SLAMF7	-2.04	1.05E-10	-0.21	127	-15.99	15211	-0.03	136	-2.89	10409	-0.35	125	-26.42	16078
SLC35F3	-1.02	0.601096143	-0.21	126	-15.99	15212	-0.03	129	-2.03	9849	-0.19	154	-19.61	15266
SEC24D	-1.47	7.75E-12	-0.23	119	-15.99	15213	-0.14	127	-10.72	14139	-0.22	142	-20.01	15328
WFDC1	-1.86	9.68E-21	-0.27	110	-15.99	15214	-0.13	124	-9.7	13776	-0.38	103	-20.14	15357
TREML1	-2.73	2.31E-20	-0.23	119	-16	15215	-0.32	124	-23.51	16278	-0.3	124	-22.17	15684
INPP5D	-1.8	1.75E-12	-0.18	137	-16	15216	-0.04	129	-3.53	10829	-0.33	112	-20.15	15358
TFR2	-2	7.13E-18	-0.29	106	-16	15217	-0.37	127	-28.39	16444	-0.27	96	-12.56	13713
GATA5	-1.27	5.37E-06	-0.23	120	-16	15218	-0.15	112	-9.41	13667	-0.27	125	-19.96	15322
ADRA1A	-1.37	2.52E-17	-0.25	115	-16	15219	-0.31	109	-18.47	15797	-0.31	116	-20.72	15447
NLRP3	-1.71	1.58E-14	-0.22	122	-16.01	15220	-0.08	104	-4.08	11138	-0.35	112	-21.46	15579
CABLES1	-1.43	1.41E-08	-0.19	136	-16.02	15221	-0.06	149	-5.69	12025	-0.19	154	-20.09	15346
MYO1F	-1.19	5.77E-10	-0.25	113	-16.02	15222	-0.01	109	-0.65	8906	-0.33	127	-25.78	16009
GPT2	-2.64	2.84E-14	-0.22	123	-16.03	15223	-0.21	125	-15.82	15366	-0.3	152	-30.1	16292
CKMT2	-1.14	0.030654256	-0.3	104	-16.03	15224	-0.1	112	-6.15	12263	-0.38	103	-19.9	15309
SLC6A13	-2.05	1.64E-13	-0.32	100	-16.04	15225	-0.27	121	-19.43	15920	-0.23	107	-13.28	13918
GORASP1	-1.12	4.62E-05	-0.21	127	-16.04	15226	-0.22	125	-16.26	15470	-0.14	127	-10.48	13084
RANBP3L	-1.21	6.64E-12	-0.16	148	-16.05	15227	-0.17	128	-13.15	14781	-0.19	136	-15.98	14583
FNDC5	-1.87	2.61E-06	-0.21	126	-16.06	15228	-0.25	114	-16.03	15427	-0.12	130	-9.89	12892
FGF23	-1.42	9.10E-08	-0.23	121	-16.07	15229	-0.3	121	-21.09	16099	-0.25	120	-17.27	14845
HOXA4	-1.49	2.98E-09	-0.18	140	-16.08	15230	-0.14	111	-8.82	13443	-0.21	142	-19.65	15273
OGDHL	-3.18	1.05E-12	-0.24	118	-16.09	15231	-0.38	112	-23.66	16286	-0.24	124	-17.52	14889
PTPN3	-1.25	1.27E-09	-0.2	129	-16.09	15232	-0.17	134	-13.99	14991	-0.18	134	-14.88	14349
SIGLEC7_t1	-1.39	3.11E-20	-0.22	123	-16.09	15233	NA	NA	NA	NA	NA	NA	NA	NA
FAM188A	-1.31	2.63E-11	-0.2	129	-16.1	15234	-0.18	119	-12.59	14664	-0.19	126	-14.12	14143
BPHL	-1.42	5.89E-08	-0.21	128	-16.1	15235	-0.21	148	-20.11	16004	-0.23	124	-17.08	14813
POR	-1.41	3.77E-05	-0.23	121	-16.11	15236	-0.2	119	-13.72	14922	-0.2	100	-9.87	12881
GIMAP4	-2.27	2.81E-20	-0.25	115	-16.12	15237	-0.15	139	-12.91	14734	-0.38	139	-34.22	16419
ALDH4A1_t2	-1.53	7.19E-08	-0.23	121	-16.12	15238	-0.21	120	-15.03	15206	-0.27	119	-18.89	15153
FCGR2B	-4.47	2.25E-22	-0.21	127	-16.13	15239	-0.28	119	-19.66	15950	-0.31	133	-25.94	16027
RBKS	-1.47	3.84E-07	-0.22	125	-16.13	15240	-0.23	102	-12.14	14549	-0.21	128	-16.58	14710
SPRY4	-1.22	4.23E-08	-0.17	144	-16.13	15241	0	122	-0.15	8582	-0.18	147	-17.05	14808
ADRA1B	-1.16	3.40E-10	-0.23	121	-16.14	15242	-0.17	108	-9.96	13869	-0.27	127	-21.06	15509
GPR84	-1.12	9.58E-07	-0.22	124	-16.14	15243	-0.11	102	-5.68	12018	-0.22	97	-10.57	13107
CDKN1C	-2.35	4.05E-14	-0.23	120	-16.14	15244	-0.15	131	-11.85	14462	-0.36	134	-30.42	16309
ZXDA	-1.17	1.76E-08	-0.19	135	-16.15	15245	-0.22	123	-16.41	15497	-0.15	146	-14.58	14262
ARG1	-3.14	4.08E-10	-0.26	112	-16.15	15246	-0.31	110	-18.61	15811	-0.22	110	-13.18	13894
SH3YL1	-2.06	6.33E-16	-0.22	124	-16.16	15247	-0.06	139	-4.94	11624	-0.3	145	-28.82	16249
ITGA1	-1.47	2.99E-08	-0.19	135	-16.16	15248	-0.17	129	-13.26	14814	0	124	0.33	8417
MNT	-1.33	8.26E-13	-0.22	122	-16.17	15249	-0.06	140	-5.15	11747	-0.34	140	-30.51	16313
HERPUD1	-1.52	1.58E-13	-0.21	126	-16.17	15250	-0.17	118	-11.49	14369	-0.34	129	-26.69	16104
EDA	-1.24	3.01E-05	-0.18	139	-16.18	15251	-0.12	136	-9.92	13854	-0.26	151	-26.57	16094
ABHD6	-1.75	4.28E-11	-0.32	101	-16.18	15252	-0.21	136	-18.03	15738	-0.24	120	-16.65	14723
CDH19	-1.16	1.42E-07	-0.2	132	-16.19	15253	-0.15	132	-11.91	14483	-0.25	124	-18.29	15041
AMHR2	-1.28	4.08E-20	-0.32	101	-16.19	15254	-0.26	116	-17.17	15613	-0.44	98	-21.32	15560
SPAG7	-1.57	1.02E-21	-0.21	126	-16.19	15255	-0.28	110	-17.04	15596	-0.32	100	-16.16	14621
CSRP1_t1	-1.78	1.55E-15	-0.21	129	-16.21	15256	NA	NA	NA	NA	NA	NA	NA	NA
ENTPD5	-1.43	2.36E-10	-0.23	121	-16.23	15257	-0.24	109	-13.91	14972	-0.21	126	-16.27	14641
RYBP_t1	-1.22	2.69E-05	-0.17	146	-16.23	15258	NA	NA	NA	NA	-0.1	153	-10.46	13077
ANXA10	-5.49	1.49E-25	-0.28	107	-16.23	15259	-0.38	135	-32.37	16478	-0.31	136	-26.26	16067
DNAJB5	-1.08	0.101524887	-0.16	153	-16.24	15260	-0.14	123	-10.23	13970	-0.08	119	-5.47	11226
MXRA5	-1.72	1.81E-18	-0.25	114	-16.24	15261	-0.17	120	-12.09	14536	-0.31	107	-17.86	14955
B3GAT1	-2.93	3.07E-21	-0.32	100	-16.24	15262	-0.31	131	-25.43	16362	-0.31	109	-18.41	15064
RECK	-1.7	4.56E-09	-0.22	124	-16.24	15263	-0.02	126	-1.47	9475	-0.21	134	-17.98	14975
SF1_t1	-1.37	7.19E-18	-0.2	130	-16.25	15264	-0.11	123	-7.71	12992	-0.27	119	-18.86	15149
CELF4	-1.1	2.33E-06	-0.21	126	-16.25	15265	-0.21	147	-20.18	16012	-0.21	126	-15.61	14496
ALDH1A3	-1.94	3.83E-12	-0.17	145	-16.26	15266	-0.14	120	-9.63	13746	-0.24	126	-17.91	14965
CTSO	-1.44	1.37E-12	-0.23	121	-16.26	15267	-0.28	132	-22.83	16238	-0.25	129	-19.48	15244
PTPN22_t2	-1.24	4.86E-08	-0.19	134	-16.26	15268	-0.03	120	-1.84	9717	-0.28	142	-25.31	15975
PRKX	-1.37	1.80E-08	-0.16	149	-16.26	15269	0.03	135	2.57	6779	-0.28	146	-26.48	16085
NFATC1_t2	-2.08	6.15E-17	-0.22	124	-16.26	15270	-0.07	142	-6.12	12253	-0.33	129	-26.18	16055
LRRK2	-1.72	8.08E-18	-0.22	125	-16.27	15271	-0.2	126	-15.16	15238	-0.32	145	-30.71	16321
RGL1	-1.76	5.15E-17	-0.23	122	-16.27	15272	-0.17	152	-17.63	15687	-0.32	136	-27.48	16157
ARL6IP4_t2	-1.14	0.000162584	-0.25	116	-16.27	15273	-0.25	108	-14.33	15062	-0.19	100	-9.37	12704

CPEB1	-1.08	0.036529779	-0.18	142	-16.28	15274	-0.02	156	-1.7	9622	-0.19	157	-19.93	15313
AKNA	-1.17	9.99E-12	-0.2	133	-16.28	15275	-0.22	137	-19.25	15895	-0.32	149	-31.47	16344
IP6K3	-1.1	0.281864913	-0.19	137	-16.29	15276	-0.02	133	-1.75	9654	-0.23	114	-14.97	14372
FAM126B	-1.23	4.47E-10	-0.17	145	-16.29	15277	-0.12	144	-11.41	14347	-0.18	137	-15.77	14535
FAM109B	-1.13	7.49E-10	-0.19	136	-16.29	15278	-0.04	145	-3.8	10984	-0.18	130	-14.17	14151
NAT8	-2.16	3.86E-09	-0.22	123	-16.3	15279	-0.17	94	-7.69	12975	-0.18	124	-13.1	13872
FERMT2	-1.63	3.63E-14	-0.2	131	-16.3	15280	-0.23	138	-20.39	16028	-0.16	136	-13.42	13962
RNF149	-1.43	3.40E-16	-0.19	138	-16.32	15281	-0.1	129	-7.57	12908	-0.35	138	-30.56	16318
FGG	-2.51	5.55E-11	-0.29	106	-16.32	15282	-0.26	125	-19.42	15917	-0.31	109	-18.11	14996
THYN1	-1.34	2.06E-14	-0.23	120	-16.33	15283	-0.1	100	-4.82	11548	-0.39	104	-21.26	15548
DHODH	-2.35	3.08E-17	-0.19	136	-16.34	15284	-0.3	138	-26.04	16380	-0.29	132	-23.8	15858
SIRT5_t2	-1.46	2.52E-09	-0.22	125	-16.34	15285	-0.24	136	-20.59	16059	-0.18	155	-18.59	15096
CLDN14	-1.77	3.87E-06	-0.24	118	-16.35	15286	-0.24	88	-9.29	13621	-0.18	119	-12.25	13629
PXDC1	-1.66	4.22E-19	-0.22	123	-16.36	15287	-0.27	120	-18.6	15810	-0.27	122	-19.51	15251
VCAM1	-2.32	8.23E-10	-0.22	126	-16.36	15288	-0.05	130	-3.76	10958	-0.21	123	-15.54	14487
SDHD_t1	-1.66	6.20E-16	-0.22	124	-16.37	15289	-0.24	110	-14.43	15085	NA	NA	NA	NA
RASGEF1B	-1.5	4.26E-23	-0.23	120	-16.37	15290	-0.27	123	-19.35	15909	-0.3	106	-16.78	14747
ERP29	-1.32	8.75E-10	-0.26	114	-16.37	15291	-0.23	115	-14.67	15141	-0.34	123	-25.12	15965
CD82	-1.48	6.42E-17	-0.21	127	-16.38	15292	-0.34	131	-27.34	16424	-0.27	120	-18.64	15110
JMJD1C_t2	-1.44	2.68E-13	-0.2	133	-16.38	15293	-0.14	138	-12.67	14680	-0.18	124	-13.45	13966
ACAA1	-2.29	2.43E-20	-0.37	94	-16.38	15294	-0.32	112	-20.14	16006	-0.32	102	-16.81	14757
TFAP2E	-1.05	5.64E-05	-0.23	120	-16.38	15295	-0.08	108	-4.81	11545	-0.16	126	-12.03	13556
FARP2	-1.19	1.62E-12	-0.3	104	-16.38	15296	-0.28	117	-19.04	15874	-0.3	100	-15.23	14425
CEP85	-1.33	1.66E-13	-0.19	136	-16.39	15297	-0.21	119	-14.16	15018	-0.18	121	-12.91	13822
TMEM156	-1.19	1.54E-05	-0.23	120	-16.39	15298	-0.08	116	-5.3	11822	-0.27	126	-20.83	15475
KLF8	-1.53	3.73E-15	-0.21	128	-16.4	15299	-0.15	147	-14.64	15134	-0.36	137	-31.01	16333
ANGPTL6	-4.25	1.64E-38	-0.39	92	-16.4	15300	-0.32	98	-15.27	15270	-0.46	95	-20.54	15417
DERA	-1.47	4.81E-10	-0.21	127	-16.42	15301	-0.19	116	-12.83	14717	-0.15	120	-10.61	13120
SPP2	-6.74	8.87E-25	-0.33	100	-16.42	15302	-0.42	150	-42.24	16492	-0.31	107	-17.8	14944
ATP8B4	-1.52	9.26E-16	-0.23	121	-16.43	15303	-0.09	131	-7.64	12945	-0.29	123	-20.93	15488
SLC17A2	-2.23	6.90E-14	-0.22	126	-16.44	15304	-0.25	134	-20.9	16078	-0.27	118	-18.1	14994
LRAT	-1.09	2.99E-06	-0.19	138	-16.44	15305	-0.15	133	-12.53	14648	-0.14	143	-12.82	13798
CXCR6	-1.6	5.36E-09	-0.17	148	-16.44	15306	-0.05	129	-3.91	11049	-0.31	146	-29.54	16272
CDH5	-1.34	1.31E-19	-0.24	119	-16.44	15307	-0.2	135	-16.93	15582	-0.27	127	-20.78	15464
CCDC71L_t1	-1.17	4.47E-09	-0.16	151	-16.44	15308	NA	NA	NA	NA	NA	NA	NA	NA
MGST2	-1.38	9.12E-08	-0.18	140	-16.45	15309	-0.24	98	-11.52	14376	-0.1	130	-7.8	12161
TNR	-1.51	1.05E-23	-0.24	119	-16.45	15310	-0.31	120	-21.96	16163	-0.26	111	-15.95	14575
RAB37	-1.33	0.000148776	-0.17	145	-16.45	15311	-0.06	127	-4.52	11397	-0.28	128	-21.84	15633
RIPK1	-1.25	5.42E-10	-0.17	147	-16.45	15312	-0.16	122	-11.64	14410	-0.19	124	-14.29	14187
ACAD8	-1.18	1.36E-06	-0.21	128	-16.46	15313	-0.14	109	-8.27	13222	-0.22	120	-15.47	14473
ADAMTSL2	-2.63	2.98E-27	-0.32	101	-16.47	15314	-0.22	120	-15.66	15336	-0.38	128	-30.01	16289
GLS2	-4.95	3.44E-20	-0.34	98	-16.47	15315	-0.36	119	-25.01	16344	-0.26	100	-13.1	13866
NDNL2	-1.11	0.001157269	-0.22	126	-16.47	15316	-0.16	122	-11.45	14357	-0.18	132	-14.72	14301
MTUS2	-1.03	0.088600936	-0.14	168	-16.47	15317	-0.08	138	-6.67	12498	-0.1	141	-9.09	12623
CCDC158	-1.17	5.83E-07	-0.27	112	-16.48	15318	-0.18	104	-9.62	13744	-0.16	104	-8.57	12443
TMBIM6_t1	-1.3	1.18E-05	-0.2	132	-16.48	15319	NA	NA	NA	NA	-0.13	135	-10.72	13158
RBBP6_t2	-1.35	5.15E-06	-0.17	148	-16.48	15320	-0.04	111	-2.32	10040	-0.15	120	-10.54	13100
OLFML1	-1.13	4.80E-06	-0.24	119	-16.48	15321	-0.14	127	-11	14237	-0.15	120	-10.69	13148
PPFIBP2_t1	-1.43	4.87E-16	-0.21	128	-16.48	15322	NA	NA	NA	NA	-0.19	106	-10.85	13199
PLK3	-2.67	9.07E-22	-0.24	119	-16.49	15323	-0.1	123	-7.61	12937	-0.36	108	-20.94	15490
TNRC6B_t1	-1.12	0.007729736	-0.15	160	-16.5	15324	NA	NA	NA	NA	NA	NA	NA	NA
R3HDM4	-1.17	6.41E-09	-0.24	118	-16.5	15325	-0.18	123	-13.13	14777	-0.2	149	-19.54	15255
MFSD2A	-2.88	2.66E-18	-0.32	101	-16.51	15326	-0.29	104	-15.51	15306	-0.27	99	-13.4	13954
FCN2_t1	-9.62	1.94E-49	-0.39	92	-16.51	15327	NA	NA	NA	NA	NA	NA	NA	NA
OVOL2	-1.06	0.319843225	-0.18	140	-16.52	15328	-0.04	131	-2.85	10379	-0.26	99	-12.85	13803
KIAA1737	-1.24	2.94E-06	-0.15	160	-16.52	15329	-0.15	133	-12.17	14556	-0.14	133	-11.36	13361
CUX2	-1.03	0.338518879	-0.21	129	-16.53	15330	-0.12	120	-8.38	13281	-0.08	126	-6.14	11492
TRPM8	-2.56	1.61E-12	-0.18	141	-16.53	15331	-0.19	135	-16.32	15479	-0.24	139	-21.25	15546
BCL2L1_t2	-1.05	0.0276766	-0.19	139	-16.53	15332	-0.1	141	-8.99	13503	-0.17	115	-11.09	13279
PIK3C2G	-1.92	1.11E-14	-0.2	134	-16.54	15333	-0.22	106	-12.45	14626	-0.2	120	-13.79	14049
PICALM	-1.3	2.41E-10	-0.24	120	-16.54	15334	-0.05	124	-3.89	11031	-0.22	132	-17.97	14974
IMP3	-1.17	2.86E-05	-0.24	119	-16.54	15335	-0.17	99	-8.24	13207	-0.18	130	-14.24	14167
DOCK8	-1.1	5.07E-07	-0.2	134	-16.55	15336	-0.02	153	-1.85	9727	-0.27	130	-21.58	15605
CCR5	-1.46	3.01E-07	-0.17	148	-16.55	15337	0.01	122	0.67	8026	-0.31	123	-22.44	15713
NEIL1	-1.65	1.65E-12	-0.28	110	-16.56	15338	-0.24	119	-16.76	15560	-0.21	154	-22.07	15668
SOCS3_t1	-1.23	8.82E-13	-0.19	139	-16.56	15339	NA	NA	NA	NA	-0.23	132	-18.92	15157
PYGL	-1.65	1.23E-09	-0.23	122	-16.56	15340	-0.27	124	-20.17	16011	-0.16	100	-7.82	12171
PTCHD1	-1	0.914663953	-0.21	129	-16.57	15341	-0.11	136	-9.82	13823	-0.15	126	-11.61	13434
SLX1A	-1.15	0.002200104	-0.17	145	-16.57	15342	-0.12	90	-4.74	11511	-0.15	92	-6.47	11628
AKT1	-1.2	9.75E-14	-0.23	123	-16.58	15343	-0.25	114	-15.74	15350	-0.31	119	-21.27	15551
LARP1B_t3	-1.49	1.02E-10	-0.21	128	-16.58	15344	-0.2	127	-15.17	15242	-0.15	155	-16.25	14636
GRM2	-2.07	7.20E-19	-0.22	124	-16.58	15345	-0.31	129	-24.16	16310	-0.28	122	-20.3	15380
RAB26	-1.7	2.27E-13	-0.31	104	-16.59	15346	-0.26	129	-20.41	16030	-0.21	152	-21.7	15619
STAT4	-1.28	6.00E-14	-0.19	138	-16.6	15347	-0.07	156	-7.64	12947	-0.2	119	-13.5	13978
BZRAP1	-1.54	2.30E-12	-0.24	120	-16.6	15348	-0.15	141	-13.84	14952	-0.38	144	-35.48	16438
LPAR1	-1.08	0.000376869	-0.2	132	-16.6	15349	-0.09	126	-6.77	12547	-0.25	131	-20.42	15401
THBS1	-1.91	8.38E-15	-0.23	123	-16.6	15350	-0.08	132	-6.36	12359	-0.25	133	-20.65	15434
BEX1	-2.99	2.17E-18	-0.23	123	-16.62	15351	-0.13	88	-5.03	11672	-0.3	127	-23.11	15788
AGAP8	-1.06	0.024802814	-0.18	144	-16.62	15352	-0.1	107	-5.94	12151	-0.04	111	-2.53	9966
HCLS1	-2.67	5.54E-23	-0.27	112	-16.62	15353	-0.13	110	-7.92	13080	-0.42	118	-28.31	16209
CDH23_t2	-1.03	0.347860548	-0.21	128	-16.62	15354	-0.17	120	-11.9	14478	-0.16	119	-11.29	13347
ATP6V0A4	1	0.893932954	-0.21	129	-16.64	15355	-0.03	91	-1.26	9338	-0.16	137	-13.97	14103
ACOT12	-2.67	1.32E-14	-0.31	104	-16.64	15356	-0.31	98	-15.01	15199	-0.25	103	-13.37	13940
LEPR_t2	-1.81	1.61E-11	-0.26	114	-16.64	15357	-0.31	129	-24.25	16316	-0.21	122	-15.12	14401
RCSD1	-1.75	3.68E-18	-0.23	122	-16.64	15358	-0.09	150	-9.37	13648	-0.39	129	-30.73	16322
BIVM	-1.3	1.42E-06	-0.19	138	-16.65	15359	-0.17	131	-13.62	14903	-0.1	120	-6.81	11778
LILRB2	-2.26	7.06E-25	-0.23	123	-16.66	15360	-0.13	127	-10.2	13956	-0.37	108	-21.55	15601
EFS	-1.81	3.63E-14	-0.25	117	-16.66	15361	-0.13	117	-8.42	13296	-0.31	126	-23.34	15804
WISP1_t2	-1.21	9.17E-05	-0.17	148	-16.66	15362	-0.1	146	-9.89	13841	-0.23	137	-19.97	15324
SUOX	-1.31	2.64E-07	-0.28	109	-16.67	15363	-0.29	128	-22.84	16241	-0.22	115	-14.28	14183
EZR	-1.28	5.56E-10	-0.21	131	-16.67	15364	-0.06	125	-4.23	11229	-0.29	143	-26.94	16126
PTPN5	-1.31	1.42E-10	-0.23	123	-16.67	15365	-0.28	118	-18.94	15859	-0.23	130	-18.44	15070
ZZEF1	-1.35	4.95E-11	-0.21	128	-16.68	15366	-0.13	127	-10.08	13910	-0.29	127	-22.57	15728
FBXO17	-1.23	0.00068353	-0.23	124	-16.68	15367	-0.09	129	-7.17	12724	-0.17	108	-9.9	12894
MOAP1	-1.25	1.31E-10	-0.25	117	-16.68	15368	-0.34	118	-23.05	16254	-0.23	139	-20.31	15382
PXN	-1.14	0.000773076	-0.19	139	-16.69	15369	0.05	122	3.7	6064	-0.17	134	-14.5	14241
ARFGAP2	-1.2	2.74E-11	-0.23	124	-16.69	15370	-0.25	131	-20.05	15991	-0.28	114	-18.22	15025
ANKFY1	-1.31	4.60E-20	-0.22	127	-16.7	15371	-0.25	139	-22.23	16193	-0.29	153	-30.18	16296
HMGN3	-1.17	0.00034206	-0.2	135	-16.7	15372	0.01	117	0.57	8095	-0.21	120	-15.03	14383
HS3ST3A1	-1.25	5.89E-20	-0.29	108	-16.7	15373	-0.23	129	-18.18	15759	-0.28	131	-22.71	15747
DDX19B	-1.34	2.21E-19	-0.24	120	-16.7	15374	-0.31	104	-16.58	15525	-0.38	114	-24.54	15918
LECT2	-4.18	7.05E-13	-0.31	104	-16.7	15375	-0.21	115	-13.61	14899	-0.22	131	-18.19	15016
PPP1R3C	-1.49	0.000500245	-0.19	137	-16.73	15376	-0.1	133	-8.27	13226	-0.08	133	-6.52	11659
CYP3A43_t2	-1.26	8.68E-12	-0.24	120	-16.73	15377	-0.26	134	-22.04	16175	-0.24	132	-19.83	15301
GAB3	-1.34	2.73E-13	-0.17	150	-16.74	15378	-0.02	140	-1.47	9474	-0.3	132	-24.88	15949
HAO2	-6.26	2.51E-25	-0.36	96	-16.75	15379	-0.34	122	-24.21	16312	-0.3	128	-23.2	15793

RUNX1T1	-1.52	2.40E-08	-0.19	136	-16.75	15380	-0.05	128	-3.71	10936	-0.2	143	-18.6	15097
PPFIBP1_t2	-1.29	4.00E-13	-0.21	130	-16.78	15381	-0.1	133	-8.3	13240	-0.22	139	-19.26	15215
PLIN2	-2.72	2.44E-19	-0.22	127	-16.79	15382	-0.2	98	-9.63	13745	-0.26	126	-19.67	15277
MRPL35_t2	-1.12	0.010071067	-0.16	153	-16.79	15383	-0.06	137	-5.6	11972	-0.07	141	-6.33	11589
SYBU	-1.81	2.41E-09	-0.17	148	-16.79	15384	-0.2	129	-16.11	15443	-0.21	153	-21.17	15531
TNKS1BP1	-1.22	7.30E-12	-0.24	120	-16.8	15385	-0.2	130	-15.75	15354	-0.35	120	-24.69	15933
PIM3	-1.66	1.97E-13	-0.14	167	-16.8	15386	-0.13	130	-10.16	13938	-0.24	125	-18.06	14986
LMBR1L	-1.17	2.76E-09	-0.21	131	-16.8	15387	-0.13	110	-7.58	12922	-0.28	123	-20.73	15448
FGA_t1	-2.5	1.83E-13	-0.28	110	-16.81	15388	-0.34	126	-25.81	16371	-0.27	131	-21.64	15611
GRAMD3	-1.25	9.60E-06	-0.23	123	-16.81	15389	-0.12	140	-10.47	14053	-0.09	120	-6.58	11685
ZFP36L1	-1.18	7.11E-10	-0.24	120	-16.81	15390	-0.21	126	-15.62	15326	-0.28	135	-23.43	15821
UGT2B11	-2.59	1.83E-14	-0.31	104	-16.81	15391	-0.31	95	-14	14992	-0.27	86	-9.84	12872
CPS1	-2.82	4.30E-13	-0.23	123	-16.84	15392	-0.32	139	-28.53	16448	-0.18	125	-13.81	14056
IL6ST	-1.61	6.15E-14	-0.2	133	-16.85	15393	-0.17	124	-12.68	14684	-0.17	151	-16.75	14744
PDE8B	-1.84	1.18E-10	-0.2	135	-16.85	15394	-0.09	136	-7.4	12831	-0.27	119	-18.43	15069
SPG20	-1.53	4.96E-17	-0.19	139	-16.85	15395	-0.11	123	-8.09	13147	-0.25	147	-24.4	15909
HCN1_t1	-1	0.755654094	-0.19	140	-16.85	15396	NA	NA	NA	NA	-0.12	146	-11.93	13518
HSD17B2	-3.97	2.04E-23	-0.34	100	-16.86	15397	-0.3	114	-18.98	15866	-0.38	85	-13.29	13919
MAFB	-1.55	3.03E-10	-0.2	136	-16.87	15398	-0.24	148	-23.94	16299	-0.29	157	-31.29	16339
FAM101B	-1.16	1.95E-08	-0.2	135	-16.87	15399	-0.14	133	-11.88	14471	-0.23	128	-18.14	15006
PPL	-1.19	1.30E-06	-0.25	117	-16.88	15400	-0.19	146	-17.78	15706	-0.3	125	-22.79	15754
SF1_t2	-1.18	3.20E-14	-0.21	130	-16.88	15401	-0.09	123	-6.42	12380	-0.32	119	-21.95	15653
ADI1_t1	-1.74	2.90E-09	-0.17	148	-16.88	15402	-0.17	123	-12.46	14631	NA	NA	NA	NA
ARL4C	-1.91	2.25E-14	-0.17	149	-16.88	15403	-0.06	137	-5.43	11880	-0.33	130	-26.64	16098
COL14A1_t1	-2.07	3.14E-14	-0.21	130	-16.89	15404	NA	NA	NA	NA	NA	NA	NA	NA
ATG16L2	-1.7	4.90E-19	-0.3	107	-16.9	15405	-0.18	92	-7.71	12993	-0.33	124	-24.26	15898
SLC17A9	-1.51	1.42E-17	-0.31	104	-16.9	15406	-0.27	120	-19	15868	-0.29	108	-16.74	14743
AXL	-2.79	4.50E-35	-0.29	108	-16.9	15407	-0.24	139	-21.1	16102	-0.43	120	-30.27	16301
KLC4_t2	-2.39	2.86E-21	-0.31	104	-16.9	15408	-0.39	118	-26.26	16392	-0.27	116	-17.91	14966
PER2	-1.52	1.06E-11	-0.24	120	-16.91	15409	-0.12	129	-9.74	13790	-0.3	133	-25.31	15976
O3FAR1	-1.1	3.20E-06	-0.19	139	-16.91	15410	-0.15	135	-12.91	14737	-0.15	153	-15.62	14500
KLF12	-1.3	0.000414861	-0.15	160	-16.92	15411	-0.08	135	-7.14	12716	-0.1	143	-9.22	12660
FMO5_t1	-1.78	7.01E-09	-0.16	154	-16.93	15412	NA	NA	NA	NA	NA	NA	NA	NA
SELE	-2.12	3.81E-12	-0.2	135	-16.93	15413	-0.16	134	-13.2	14797	-0.2	142	-18.85	15145
DUSP1	-3.08	7.04E-22	-0.21	130	-16.94	15414	-0.28	126	-21.06	16096	-0.31	118	-20.76	15454
LRP6	-1.16	6.46E-06	-0.19	140	-16.94	15415	-0.25	135	-21.1	16101	-0.11	154	-11.42	13376
LMBRD1	-1.5	7.39E-11	-0.31	104	-16.94	15416	-0.26	111	-15.93	15400	-0.23	113	-14.21	14159
NFX1_t2	-1.37	3.27E-14	-0.21	129	-16.94	15417	-0.31	115	-20.05	15992	-0.25	106	-14.11	14140
BMPER	-1.16	1.10E-13	-0.2	134	-16.95	15418	-0.24	120	-17.02	15593	-0.22	127	-16.7	14736
FLVCR2	-1.21	0.00017402	-0.19	139	-16.96	15419	-0.12	136	-10.27	13992	-0.2	151	-20.36	15389
LEAP2	-2.92	3.02E-14	-0.31	104	-16.97	15420	-0.19	120	-13.59	14895	-0.26	116	-17.45	14870
SLC9B2	-1.31	2.92E-12	-0.24	121	-16.97	15421	-0.25	113	-15.83	15369	-0.24	135	-20.08	15344
DIRAS3	-1.91	8.42E-21	-0.27	113	-16.98	15422	-0.37	102	-19.22	15891	-0.3	126	-22.97	15773
NOX1_t2	-1.12	0.006105355	-0.17	151	-16.98	15423	-0.19	104	-10.17	13940	-0.17	147	-16.33	14654
TOM1	-1.09	0.001411935	-0.21	132	-16.99	15424	-0.15	134	-12.52	14646	-0.2	129	-16.11	14611
ATP7B	-1.23	4.87E-05	-0.17	148	-16.99	15425	-0.05	141	-4.69	11483	-0.24	120	-16.55	14700
FUCA1	-1.56	1.39E-18	-0.21	130	-17.02	15426	-0.27	114	-17.5	15671	-0.32	117	-21.54	15599
IGJ	-6.1	2.11E-25	-0.27	114	-17.03	15427	-0.12	128	-9.57	13719	-0.45	126	-34.26	16420
DMRTA1	-1.41	6.87E-07	-0.25	119	-17.03	15428	-0.22	134	-18.09	15748	-0.2	115	-13.11	13873
SLC22A23	-1.13	0.00015138	-0.16	159	-17.04	15429	-0.08	141	-6.85	12586	-0.17	132	-13.89	14073
TRIB1	-2.56	5.54E-23	-0.24	120	-17.04	15430	-0.21	141	-18.77	15833	-0.27	123	-19.86	15305
EFNB1	-1.34	4.11E-08	-0.21	132	-17.04	15431	0.02	132	1.86	7239	-0.27	120	-19.14	15195
PLEKHO2	-1.45	3.40E-11	-0.18	146	-17.04	15432	-0.03	138	-2.57	10198	-0.34	138	-29.59	16273
ETS1_t1	-1.14	3.39E-12	-0.21	131	-17.05	15433	NA	NA	NA	NA	NA	NA	NA	NA
ACVR1B_t2	-1.5	2.07E-18	-0.2	134	-17.06	15434	-0.31	127	-23.53	16279	-0.2	154	-20.78	15462
ALDH2_t1	-2.68	4.82E-17	-0.3	107	-17.06	15435	NA	NA	NA	NA	-0.33	111	-20.11	15349
STON2	-1.03	0.041694567	-0.16	156	-17.06	15436	-0.03	147	-3.25	10631	-0.09	146	-8.2	12316
PPP1R3D	-1.06	0.066730027	-0.15	163	-17.07	15437	0.02	136	1.3	7602	-0.17	131	-13.8	14051
CLEC10A_t1	-1.53	1.34E-26	-0.21	130	-17.07	15438	-0.24	127	-18.28	15769	-0.31	126	-23.82	15860
CES4A	-1.26	9.54E-06	-0.27	113	-17.07	15439	-0.26	120	-18.08	15746	-0.23	120	-15.92	14568
C5	-2.33	2.97E-10	-0.21	132	-17.08	15440	-0.31	124	-22.88	16245	-0.2	142	-18.19	15017
CPN1	-2.03	6.57E-13	-0.27	114	-17.08	15441	-0.35	102	-18.07	15744	-0.24	96	-10.95	13235
SNRK_t1	-1.21	6.48E-12	-0.22	129	-17.09	15442	-0.13	131	-10.53	14079	NA	NA	NA	NA
GRM7	-1.01	0.265744634	-0.23	125	-17.09	15443	-0.15	110	-8.74	13416	-0.13	144	-11.97	13532
CARKD	-1.36	1.55E-08	-0.21	131	-17.1	15444	-0.25	148	-24.09	16307	-0.26	120	-18.32	15044
EPAS1	-1.26	2.94E-11	-0.19	138	-17.1	15445	-0.16	130	-12.93	14740	-0.21	138	-18.84	15143
KIAA1161	-1.09	0.029940818	-0.23	123	-17.1	15446	-0.1	131	-8.12	13166	-0.12	137	-10.39	13047
IL1RAP_t1	-5.43	2.01E-26	-0.31	106	-17.1	15447	-0.25	123	-18.06	15741	-0.3	116	-20.1	15348
UBXN6	-1.14	0.000131725	-0.19	139	-17.1	15448	-0.18	105	-9.85	13828	-0.17	115	-11.24	13319
GABRB2	-1.05	0.000329243	-0.17	150	-17.1	15449	-0.09	135	-7.41	12840	-0.06	144	-6.09	11479
DSCAM	-1.22	4.66E-20	-0.32	104	-17.11	15450	-0.28	134	-23.75	16290	-0.33	130	-26.19	16057
GALP	-1.06	0.244966841	-0.21	131	-17.13	15451	-0.17	111	-10.33	14016	-0.03	111	-1.93	9683
MYO18A	-1.19	6.54E-06	-0.24	120	-17.14	15452	-0.27	136	-23.4	16269	-0.21	128	-16.24	14632
SULT1A1	-2.63	3.14E-22	-0.28	112	-17.14	15453	-0.28	104	-15.05	15209	-0.3	87	-10.97	13248
DEPDC1B	-1.67	1.45E-09	-0.17	152	-17.14	15454	-0.19	110	-11.34	14330	-0.15	116	-9.83	12869
CP	-2.73	1.31E-13	-0.21	130	-17.14	15455	-0.26	129	-20.59	16058	-0.19	120	-13.34	13934
ACOT2_t1	-1.58	2.15E-10	-0.22	129	-17.15	15456	NA	NA	NA	NA	NA	NA	NA	NA
IRF8	-2	2.82E-27	-0.25	119	-17.16	15457	-0.29	120	-20.16	16009	-0.38	150	-38.29	16471
SEMA5A	-1.24	2.33E-11	-0.19	139	-17.16	15458	-0.21	139	-18.38	15788	-0.16	135	-13.94	14090
BCKDK	-1.28	6.21E-08	-0.25	119	-17.17	15459	-0.15	119	-10.56	14091	-0.24	120	-16.83	14764
HNMT_t2	-1.25	5.38E-08	-0.16	160	-17.17	15460	-0.19	120	-12.99	14752	-0.12	126	-9.46	12743
SRGN	-3.01	2.28E-24	-0.21	132	-17.17	15461	-0.1	115	-6.82	12574	-0.35	117	-23.77	15856
GDI2	-1.16	0.000968727	-0.22	127	-17.18	15462	-0.11	129	-8.5	13326	-0.16	125	-12.25	13627
ENPEP	-1.55	5.69E-09	-0.18	147	-17.19	15463	-0.24	113	-15.09	15219	-0.09	139	-8.07	12253
STX11	-1.52	7.82E-20	-0.17	149	-17.19	15464	-0.18	129	-14.02	14995	-0.4	154	-41.47	16485
NXF3	-1.89	1.97E-32	-0.31	105	-17.19	15465	-0.25	98	-12.05	14517	-0.4	120	-28.06	16193
SEC14L2	-1.99	9.76E-10	-0.25	119	-17.2	15466	-0.29	126	-22.23	16192	-0.17	129	-13.14	13882
LRRFIP2_t2	-1.21	1.18E-06	-0.21	131	-17.21	15467	-0.12	127	-9.54	13712	-0.08	115	-5.02	11056
DNAJB14	-1.6	4.49E-15	-0.21	133	-17.22	15468	-0.19	142	-17.23	15626	-0.27	124	-20.21	15369
REL	-1.55	1.93E-16	-0.19	139	-17.22	15469	-0.15	139	-13.48	14866	-0.24	126	-18.25	15033
APCS	-2.68	9.63E-10	-0.23	124	-17.22	15470	-0.13	98	-6.34	12342	-0.25	120	-17.49	14880
NXF1	-1.23	2.81E-10	-0.26	115	-17.22	15471	-0.2	102	-10.26	13986	-0.34	121	-23.82	15859
PRG2	-1.27	2.85E-10	-0.26	116	-17.22	15472	-0.28	89	-10.91	14208	-0.24	93	-10.44	13073
TMPRSS6	-1.75	1.19E-09	-0.29	110	-17.23	15473	-0.24	108	-14.21	15031	-0.16	95	-7.17	11908
ABCC6_t2	-1.46	4.69E-08	-0.23	126	-17.23	15474	-0.25	118	-16.73	15554	-0.25	113	-15.47	14472
ITGB3_t1	-1.16	1.45E-05	-0.23	126	-17.25	15475	NA	NA	NA	NA	NA	NA	NA	NA
MFAP4	-8.22	1.07E-34	-0.32	104	-17.26	15476	-0.19	143	-17.41	15662	-0.42	120	-29.44	16270
ASPRV1	-1.34	9.60E-10	-0.27	113	-17.26	15477	-0.16	112	-9.84	13825	-0.28	121	-19.8	15294
SNX15_t2	-1.24	4.92E-14	-0.28	112	-17.27	15478	-0.23	136	-19.36	15910	-0.29	127	-21.97	15657
MRC2	-1.19	1.19E-19	-0.25	119	-17.28	15479	-0.18	120	-12.94	14741	-0.27	108	-15.64	14505
CYB5D2	-1.51	3.84E-15	-0.25	120	-17.29	15480	-0.34	116	-22.73	16231	-0.28	113	-17.75	14934
IL33	-3.26	7.71E-32	-0.28	112	-17.3	15481	-0.26	131	-21.15	16107	-0.31	133	-25.75	16006
ZFYVE21	-1.41	5.01E-16	-0.23	125	-17.31	15482	-0.18	119	-12.69	14689	-0.27	137	-23.86	15862
DAAM2	-1.25	3.15E-05	-0.21	134	-17.31	15483	-0.23	128	-17.6	15679	-0.24	149	-23.47	15825
CELF3	-1.07	9.02E-07	-0.23	125	-17.32	15484	-0.17	145	-15.87	15382	-0.19	142	-17.37	14856
SEPP1	-1.88	6.67E-07	-0.22	129	-17.32	15485	-0.25	131	-19.93	15979	-0.13	124	-9.86	12876

NLGN2	-1.2	1.53E-11	-0.25	120	-17.33	15486	-0.14	126	-10.69	14128	-0.2	112	-12.29	13637
RGS18	-2.22	9.59E-24	-0.23	124	-17.33	15487	-0.11	118	-7.65	12956	-0.36	127	-27.77	16174
TAGAP_t2	-1.85	7.17E-17	-0.2	135	-17.34	15488	-0.09	131	-7.09	12690	-0.36	130	-28.67	16238
FCN1	-1.65	3.08E-17	-0.28	112	-17.36	15489	-0.15	150	-15.11	15227	-0.43	100	-21.42	15573
SIDT2	-1.3	7.07E-08	-0.22	130	-17.36	15490	-0.11	133	-9.48	13695	-0.31	142	-28.49	16223
USP15_t1	-1.36	4.65E-12	-0.17	154	-17.36	15491	-0.09	142	-8.37	13274	NA	NA	NA	NA
ZMAT3	-1.29	1.11E-12	-0.18	146	-17.36	15492	-0.15	156	-15.8	15362	-0.17	160	-18.75	15130
STX17	-1.16	3.05E-06	-0.21	131	-17.37	15493	-0.14	152	-14.51	15102	-0.17	122	-12.33	13654
CTSL1	-1.82	2.06E-19	-0.25	120	-17.38	15494	-0.25	125	-18.53	15800	-0.28	142	-26.18	16056
SLC3A1	-3.49	1.10E-13	-0.25	119	-17.4	15495	-0.12	102	-6.07	12223	-0.25	106	-14.05	14124
PPIF	-1.5	1.15E-07	-0.22	130	-17.4	15496	-0.21	126	-16.2	15459	-0.23	119	-15.73	14526
LRRC4	-1.35	1.83E-26	-0.29	111	-17.41	15497	-0.23	107	-13.3	14824	-0.33	147	-32.2	16371
CD52	-2.07	1.45E-12	-0.21	132	-17.42	15498	-0.04	100	-1.79	9690	-0.37	138	-32.83	16387
IRF4	-1.05	0.058577368	-0.17	150	-17.44	15499	-0.05	138	-4.7	11487	-0.19	138	-17.12	14820
SLC24A4	-1.17	1.49E-17	-0.19	140	-17.44	15500	-0.16	146	-15.15	15236	-0.22	152	-22.19	15687
ZC3H14	-1.15	1.62E-05	-0.22	129	-17.45	15501	-0.19	134	-15.65	15334	-0.18	157	-19.3	15221
COL4A4	-1.7	2.01E-11	-0.2	136	-17.45	15502	-0.17	156	-17.93	15725	-0.3	140	-26.68	16103
TMEM204	-2.14	1.28E-16	-0.25	121	-17.45	15503	-0.1	129	-8.05	13130	-0.35	111	-21.17	15529
CCL2	-4.65	9.19E-24	-0.28	113	-17.45	15504	-0.12	135	-10.52	14072	-0.36	119	-24.51	15914
SMAD7	-1.49	5.62E-19	-0.21	135	-17.46	15505	-0.12	131	-10.11	13923	-0.3	123	-22.24	15696
PPP1R1A	-1.67	8.90E-12	-0.2	139	-17.46	15506	-0.2	118	-13.63	14905	-0.15	120	-10.53	13097
SLC4A4_t1	-2.18	3.02E-13	-0.17	155	-17.46	15507	NA	NA	NA	NA	-0.2	142	-18.54	15088
STMN2	-1.74	1.13E-06	-0.21	133	-17.47	15508	-0.04	151	-4.04	11122	-0.27	136	-23.34	15805
MC2R	-1.39	6.56E-16	-0.23	127	-17.48	15509	-0.28	143	-26.24	16391	-0.23	130	-18.64	15111
KLRG1	-1.06	0.000160161	-0.23	127	-17.49	15510	-0.03	143	-2.85	10380	-0.19	112	-11.8	13486
FLT4_t2	-1.14	8.61E-12	-0.22	129	-17.5	15511	-0.2	104	-10.93	14216	-0.22	124	-16.55	14699
SLCO2B1	-1.78	7.19E-10	-0.25	120	-17.5	15512	-0.37	127	-28.56	16449	-0.17	120	-12.09	13571
UGT2B28	-4.73	2.55E-24	-0.32	104	-17.5	15513	-0.34	100	-17.19	15617	-0.31	86	-11.27	13333
FURIN	-1.53	4.57E-14	-0.24	122	-17.5	15514	-0.22	118	-15.25	15264	-0.18	129	-14.16	14150
HDC	-1.58	6.51E-13	-0.25	119	-17.51	15515	-0.12	112	-7.43	12846	-0.3	104	-16.31	14648
EGR2	-3.95	6.94E-21	-0.22	130	-17.51	15516	-0.06	140	-5.29	11813	-0.38	120	-26.46	16083
ITK_t1	-1.5	8.87E-12	-0.19	141	-17.51	15517	-0.05	144	-4.84	11565	NA	NA	NA	NA
DEPDC7	-3.45	4.31E-20	-0.29	110	-17.51	15518	-0.29	104	-15.76	15355	-0.28	119	-19.44	15238
CARD14_t2	-1.55	7.04E-14	-0.22	128	-17.51	15519	-0.27	118	-18.33	15775	-0.2	130	-15.89	14560
PLIN1	-1.81	9.84E-17	-0.22	131	-17.52	15520	-0.17	139	-15.25	15263	-0.26	120	-18.34	15054
NIPSNAP1_t1	-1.31	6.29E-09	-0.25	121	-17.53	15521	NA	NA	NA	NA	-0.2	137	-17.7	14926
LIN28A	-1.08	8.50E-05	-0.21	134	-17.53	15522	-0.22	135	-18.81	15843	-0.37	122	-26.74	16107
GOT1	-2.09	3.29E-13	-0.22	128	-17.54	15523	-0.41	113	-26.01	16379	-0.21	111	-12.6	13730
UTP3	-1.26	1.31E-06	-0.23	127	-17.55	15524	-0.08	108	-4.64	11460	-0.18	120	-12.45	13683
INF2_t2	-1.41	9.20E-18	-0.25	119	-17.55	15525	-0.18	108	-10.51	14066	-0.35	103	-18.64	15109
FAM110C	-1.47	3.63E-12	-0.23	128	-17.56	15526	-0.25	138	-21.93	16160	-0.22	152	-21.94	15649
SLC17A1	-2.22	2.55E-14	-0.28	112	-17.59	15527	-0.2	129	-15.63	15330	-0.24	107	-13.61	14005
TRAF4	-1.51	3.39E-20	-0.27	116	-17.59	15528	-0.31	141	-28.07	16437	-0.27	113	-17	14801
GREM2_t1	-1.91	4.46E-14	-0.22	131	-17.61	15529	-0.25	139	-22.2	16187	NA	NA	NA	NA
GLI2_t1	-1.18	1.20E-06	-0.19	141	-17.61	15530	-0.05	123	-3.49	10791	NA	NA	NA	NA
MCC	-1.63	5.20E-18	-0.17	151	-17.62	15531	-0.23	131	-18.9	15852	-0.19	146	-18.68	15118
GALNTL1	-1.9	1.25E-20	-0.26	119	-17.62	15532	-0.2	136	-17.62	15682	-0.34	149	-33.89	16414
ENO3	-3.66	7.03E-22	-0.4	94	-17.62	15533	-0.29	100	-14.68	15143	-0.29	88	-10.83	13189
HINT2	-1.35	1.10E-08	-0.23	127	-17.63	15534	-0.25	97	-11.61	14401	-0.21	81	-6.62	11705
EPHX3	-1.23	3.34E-16	-0.27	115	-17.63	15535	-0.23	118	-15.88	15387	-0.25	104	-13.45	13965
TCTEX1D1	-1.63	4.77E-11	-0.25	120	-17.64	15536	-0.24	120	-16.97	15586	-0.22	144	-20.32	15383
IL1RAP_t2	-2.41	7.05E-17	-0.21	136	-17.66	15537	NA	NA	NA	NA	NA	NA	NA	NA
TTC39C	-1.44	9.51E-05	-0.18	150	-17.67	15538	-0.31	133	-25.99	16377	-0.08	119	-5.54	11255
DNALI1	-2.03	6.86E-13	-0.23	128	-17.67	15539	-0.19	132	-15.54	15310	-0.29	130	-23.29	15803
ENDOV	-1.09	0.007395762	-0.24	123	-17.68	15540	-0.2	127	-15.15	15235	-0.27	115	-17.63	14909
TCL1A	-1.65	2.64E-13	-0.29	111	-17.69	15541	-0.22	120	-15.73	15347	-0.31	139	-27.24	16149
ISOC1	-1.45	9.75E-11	-0.22	131	-17.69	15542	-0.3	124	-22.04	16174	-0.14	139	-12.32	13651
FMO3	-2.6	1.35E-10	-0.29	111	-17.69	15543	-0.22	129	-17.07	15602	-0.23	125	-16.89	14783
PIM1	-1.51	2.86E-08	-0.17	154	-17.69	15544	-0.05	125	-3.49	10792	-0.31	117	-20.96	15495
FAM65B	-1.54	8.74E-18	-0.23	126	-17.69	15545	-0.18	120	-12.3	14583	-0.36	143	-33.15	16394
IL17RA	-1.17	9.88E-07	-0.18	149	-17.7	15546	-0.08	141	-7.07	12682	-0.31	160	-34.43	16425
TJP2_t1	-1.33	3.84E-11	-0.18	149	-17.71	15547	-0.22	120	-15.39	15288	-0.15	123	-11.24	13321
H19	-3.61	1.26E-18	-0.25	122	-17.72	15548	-0.26	124	-19.12	15884	-0.33	157	-35.21	16436
NFKBIZ	-2.11	1.13E-18	-0.21	134	-17.72	15549	-0.13	141	-12.04	14511	-0.32	131	-25.52	15991
TMEM121	-2.09	1.25E-20	-0.32	106	-17.72	15550	-0.27	108	-15.9	15396	-0.32	104	-17.36	14853
PARVG	-1.42	1.89E-18	-0.22	131	-17.73	15551	-0.09	136	-7.4	12830	-0.37	107	-21.25	15545
CD226	-1.15	2.66E-17	-0.21	133	-17.73	15552	-0.13	127	-9.97	13873	-0.29	112	-18.13	15003
SLC25A38	-1.09	0.103374736	-0.21	135	-17.74	15553	-0.2	156	-21.15	16108	-0.07	128	-5.44	11215
ARL6IP1_t1	-1.51	4.91E-09	-0.21	136	-17.75	15554	NA	NA	NA	NA	-0.18	155	-18.61	15099
EMR3	-1.09	0.004165123	-0.3	110	-17.75	15555	-0.07	100	-3.51	10803	-0.25	135	-21.47	15581
LILRB5	-1.58	1.38E-27	-0.26	118	-17.75	15556	-0.23	124	-16.67	15539	-0.35	137	-30.36	16306
TPD52L1	-1.72	1.55E-08	-0.22	129	-17.75	15557	-0.15	123	-11.19	14297	-0.22	114	-13.81	14055
GBP6	-1.18	2.54E-08	-0.18	147	-17.76	15558	-0.16	135	-13.21	14799	-0.2	160	-21.66	15613
KLF6_t1	-2.29	3.62E-22	-0.22	130	-17.77	15559	NA	NA	NA	NA	NA	NA	NA	NA
BHLHE40	-1.69	1.17E-21	-0.18	150	-17.77	15560	-0.17	132	-14.31	15058	-0.23	137	-20.4	15398
ZG16	-4.96	3.43E-21	-0.31	107	-17.77	15561	-0.36	130	-28.49	16446	-0.29	114	-18.41	15065
CD5	-1.1	9.39E-05	-0.2	140	-17.78	15562	-0.05	137	-4.62	11450	-0.17	110	-10.35	13034
SLC37A4	-1.71	7.24E-13	-0.3	109	-17.79	15563	-0.33	124	-24.33	16320	-0.33	129	-26.08	16040
SCRT2	-1.37	4.18E-08	-0.21	134	-17.79	15564	-0.22	122	-15.88	15383	-0.14	126	-10.65	13135
ALB	-3.86	1.57E-13	-0.34	103	-17.8	15565	-0.34	120	-23.58	16282	-0.3	119	-20.83	15473
FOS	-7.38	1.33E-34	-0.25	120	-17.8	15566	-0.22	133	-18.66	15818	-0.37	112	-23	15777
PTPRC	-2.21	5.30E-17	-0.21	136	-17.82	15567	-0.05	128	-3.87	11018	-0.32	130	-25.72	16002
OS9_t2	-1.3	2.01E-12	-0.24	124	-17.83	15568	-0.33	120	-23.04	16252	-0.25	121	-17.9	14964
CLPX	-1.42	1.54E-09	-0.24	123	-17.84	15569	-0.19	115	-12.3	14586	-0.14	106	-7.69	12109
C1QTNF1_t1	-1.5	2.00E-24	-0.27	115	-17.84	15570	NA	NA	NA	NA	-0.37	127	-28.77	16245
SCN5A_t2	1.01	0.334520934	-0.18	151	-17.85	15571	-0.1	118	-7.01	12651	-0.06	112	-3.42	10366
DHRS12	-1.72	2.20E-16	-0.23	129	-17.85	15572	-0.21	120	-14.65	15137	-0.35	131	-28.51	16227
GRTP1	-1.31	2.69E-06	-0.19	146	-17.87	15573	-0.24	120	-16.52	15516	-0.12	127	-9.37	12703
SOS1	-1.26	9.00E-12	-0.2	141	-17.87	15574	-0.23	136	-19.88	15969	-0.17	150	-16.95	14793
ARHGAP30_t2	-3.03	3.80E-19	-0.22	132	-17.88	15575	-0.35	124	-26.24	16390	-0.25	111	-15.48	14477
COA5	-1.14	3.12E-05	-0.24	124	-17.89	15576	-0.08	143	-7.78	13017	-0.15	129	-11.54	13413
FAM107B	-1.29	2.72E-05	-0.21	137	-17.89	15577	-0.15	144	-14.29	15050	-0.11	152	-10.73	13163
FGL2	-2.34	1.17E-19	-0.16	159	-17.9	15578	-0.1	125	-7.52	12881	-0.32	121	-23	15778
ZNF18	-1.28	2.80E-09	-0.28	114	-17.9	15579	-0.19	108	-10.94	14221	-0.19	128	-15.01	14377
CYP3A4	-4	7.87E-14	-0.24	126	-17.91	15580	-0.29	135	-24.7	16330	-0.24	133	-19.84	15304
MAP2K3_t1	-1.75	1.51E-19	-0.31	108	-17.92	15581	NA	NA	NA	NA	-0.29	130	-22.95	15770
PLA2G5	-1.91	1.27E-25	-0.28	115	-17.93	15582	-0.17	127	-12.91	14735	-0.37	121	-26.17	16052
JMJD7-PLA2G4	-1.14	0.002618823	-0.24	126	-17.96	15583	-0.18	108	-10.68	14123	-0.15	113	-9.52	12766
LGI4	-1.19	2.33E-05	-0.2	139	-17.98	15584	-0.12	126	-9.14	13571	-0.21	120	-14.57	14259
GEM	-3.33	6.39E-16	-0.2	142	-18	15585	-0.05	119	-3.58	10860	-0.29	112	-18.15	15011
SDHA_t1	-1.51	2.06E-16	-0.27	116	-18	15586	NA	NA	NA	NA	-0.28	120	-19.82	15298
PVRIG	-1.36	3.65E-12	-0.29	113	-18	15587	-0.18	96	-8.41	13292	-0.36	89	-13.88	14071
SEMA4D	-1.19	4.77E-08	-0.17	153	-18	15588	-0.02	159	-2.43	10104	-0.31	121	-21.72	15620
HPCAL4	-1.01	0.382328927	-0.19	146	-18.01	15589	0.07	140	6.26	4636	-0.15	136	-12.59	13725
ABCG2	-2.05	5.75E-12	-0.25	121	-18.03	15590	-0.18	126	-13.78	14945	-0.17	133	-14.01	14114
ALDH5A1	-1.16	0.000564873	-0.19	145	-18.03	15591	-0.13	136	-10.98	14228	-0.1	130	-7.7	12114

FAM100A	-1.49	4.86E-22	-0.26	119	-18.04	15592	-0.24	123	-17.33	15646	-0.3	114	-19.25	15214
PPAP2B	-2.64	1.49E-27	-0.24	124	-18.04	15593	-0.3	123	-21.75	16150	-0.31	133	-25.81	16015
DBT	-1.31	1.37E-10	-0.18	148	-18.06	15594	-0.2	139	-17.53	15675	-0.15	149	-14.59	14267
TOR4A_t1	-1.49	7.71E-12	-0.25	123	-18.06	15595	NA	NA	NA	NA	-0.32	119	-22.21	15691
CXorf21	-1.34	1.84E-13	-0.22	133	-18.06	15596	0	135	0.33	8249	-0.36	150	-35.69	16445
POU2AF1	-2.97	2.10E-11	-0.19	143	-18.06	15597	-0.04	134	-2.96	10453	-0.35	121	-24.81	15944
NR1I2	-2.28	4.14E-13	-0.26	120	-18.06	15598	-0.24	120	-17.15	15608	-0.22	124	-16.52	14688
ERLIN1	-1.42	8.74E-15	-0.19	146	-18.07	15599	-0.24	131	-19.3	15903	-0.19	134	-16.29	14644
RHOB	-1.85	4.26E-18	-0.22	133	-18.09	15600	-0.18	123	-12.83	14715	-0.35	143	-32.21	16372
GPR182	-1.3	1.69E-23	-0.26	120	-18.09	15601	-0.28	115	-18.06	15743	-0.29	141	-26.29	16069
GPR65	-1.84	7.20E-20	-0.22	133	-18.09	15602	-0.09	147	-8.53	13335	-0.38	141	-34.34	16421
FNDC3A	-1.64	6.78E-09	-0.21	137	-18.09	15603	-0.14	135	-11.86	14466	-0.23	146	-22.19	15686
PID1	-1.47	1.21E-07	-0.19	143	-18.09	15604	-0.22	134	-18.2	15760	-0.17	158	-18.32	15045
SDPR	-2.09	3.48E-23	-0.22	132	-18.1	15605	-0.2	132	-16.01	15423	-0.28	149	-27.51	16159
FUT6	-1.3	4.66E-06	-0.22	131	-18.1	15606	-0.1	131	-8.02	13125	-0.21	120	-14.38	14212
TMIGD2	-1.37	1.12E-21	-0.24	125	-18.11	15607	-0.26	131	-21.03	16092	-0.26	120	-18.32	15048
PKNOX2	-1.19	2.76E-09	-0.26	120	-18.11	15608	-0.1	124	-7.45	12851	-0.24	120	-16.68	14733
FCAR	-1.11	4.07E-07	-0.2	140	-18.11	15609	-0.22	111	-13.43	14854	-0.16	122	-11.81	13490
CYP4V2	-1.89	4.67E-14	-0.24	127	-18.13	15610	-0.32	133	-26.68	16406	-0.25	139	-22	15660
GRK5	-1.65	7.86E-15	-0.26	119	-18.13	15611	-0.15	102	-7.55	12897	-0.33	139	-29.71	16277
IL10RA_t1	-1.14	1.79E-14	-0.23	129	-18.15	15612	NA	NA	NA	NA	-0.36	136	-30.93	16330
DAO	-2.55	1.42E-09	-0.28	115	-18.18	15613	-0.27	108	-15.58	15320	-0.19	138	-17.02	14803
SERPING1	-1.97	1.20E-18	-0.34	104	-18.19	15614	-0.36	107	-20.58	16057	-0.39	106	-21.79	15629
TMPRSS4	-1.13	0.000586766	-0.18	149	-18.19	15615	-0.18	124	-13.35	14839	-0.21	121	-14.62	14273
GSTA2_t1	-2.55	5.66E-10	-0.23	129	-18.2	15616	-0.35	113	-22.09	16177	NA	NA	NA	NA
GPR180_t1	-1.73	1.60E-11	-0.2	140	-18.22	15617	NA	NA	NA	NA	-0.17	150	-16.73	14741
LUM	-5.6	1.53E-15	-0.21	136	-18.23	15618	-0.09	122	-6.34	12343	-0.3	129	-23.51	15831
FOXO3	-1.15	2.53E-08	-0.2	142	-18.24	15619	-0.07	160	-7.86	13058	-0.26	134	-22.1	15675
HECA	-1.65	5.09E-12	-0.2	142	-18.25	15620	-0.11	125	-8.49	13323	-0.22	155	-23.43	15820
LDB2	-1.45	2.62E-24	-0.28	115	-18.25	15621	-0.18	105	-9.65	13753	-0.34	132	-28.16	16197
RND3	-5.7	7.53E-31	-0.26	120	-18.25	15622	-0.19	134	-16.01	15420	-0.33	133	-27.18	16140
TPMT_t1	-1.32	1.15E-13	-0.23	131	-18.25	15623	-0.2	131	-15.92	15399	NA	NA	NA	NA
CYB561D1	-1.09	0.000686864	-0.17	157	-18.26	15624	-0.13	142	-12.31	14587	-0.17	132	-14.1	14139
GLCE	-1.2	0.002310695	-0.22	133	-18.27	15625	-0.03	132	-2.39	10078	-0.01	148	-0.67	9019
GATA6	-1.59	2.23E-10	-0.18	154	-18.27	15626	-0.12	129	-9.67	13762	-0.3	131	-24.61	15924
AGL	-1.92	1.93E-11	-0.19	147	-18.28	15627	-0.22	132	-18.06	15742	-0.13	147	-12.74	13775
LPA_t1	-2.99	3.37E-13	-0.34	104	-18.28	15628	NA	NA	NA	NA	NA	NA	NA	NA
APCDD1	-1.8	1.25E-07	-0.24	126	-18.29	15629	-0.05	117	-3.34	10699	-0.29	145	-27.52	16161
SPIC	-1.7	2.62E-22	-0.29	114	-18.29	15630	-0.22	109	-12.69	14688	-0.38	111	-23.08	15786
STARD5_t1	-1.29	5.06E-16	-0.31	110	-18.32	15631	NA	NA	NA	NA	-0.31	120	-21.55	15603
MYOT	-1.14	7.94E-05	-0.24	126	-18.32	15632	-0.13	127	-10.39	14034	-0.17	143	-16.14	14615
SLC16A9	-1.37	1.28E-05	-0.24	126	-18.32	15633	-0.19	125	-14.45	15091	-0.23	147	-21.92	15645
SERPINF2	-3	4.10E-25	-0.37	100	-18.33	15634	-0.39	122	-27.89	16434	-0.37	113	-23.47	15824
SCP2_t2	-2.43	1.03E-16	-0.27	119	-18.36	15635	-0.28	130	-22.66	16223	-0.21	113	-13.34	13933
CCDC8	-1.25	1.29E-13	-0.26	121	-18.37	15636	-0.14	121	-10.28	13994	-0.29	132	-23.68	15848
CH25H	-3.09	2.74E-15	-0.26	120	-18.37	15637	-0.08	119	-5.8	12087	-0.34	104	-18.15	15010
EPCAM	-3.68	8.36E-09	-0.18	152	-18.39	15638	0.01	119	0.81	7928	-0.26	115	-16.81	14761
RNF112	-1.82	1.93E-16	-0.28	115	-18.4	15639	-0.12	124	-9	13504	-0.35	137	-30.22	16299
GNPNAT1	-1.56	1.44E-12	-0.18	150	-18.4	15640	-0.27	139	-24.15	16309	-0.11	137	-9.79	12851
ANO1	-2.52	1.83E-10	-0.24	126	-18.4	15641	-0.3	143	-28.13	16440	-0.17	132	-13.68	14020
AGXT2L1	-3.01	2.04E-08	-0.27	119	-18.42	15642	-0.29	118	-19.48	15925	-0.15	127	-11.49	13400
ACSM2A	-2.08	6.48E-09	-0.26	121	-18.42	15643	-0.29	116	-18.97	15864	-0.22	106	-12.05	13559
IL1R1_t1	-1.45	1.96E-08	-0.17	160	-18.43	15644	NA	NA	NA	NA	-0.26	143	-23.85	15861
ACMSD	-2	6.17E-12	-0.24	128	-18.43	15645	-0.34	91	-14.06	15006	-0.18	117	-11.93	13520
ACE2	-1.61	0.000601498	-0.23	131	-18.43	15646	-0.08	139	-6.97	12633	-0.19	122	-13.93	14088
PCTP	-1.49	5.89E-10	-0.21	139	-18.44	15647	-0.16	113	-10.1	13915	-0.25	141	-22.92	15769
KDR	-1.16	6.12E-12	-0.24	128	-18.45	15648	-0.2	126	-15.48	15301	-0.15	150	-15.48	14476
RDH5	-2.99	1.99E-30	-0.35	103	-18.46	15649	-0.32	113	-19.93	15980	-0.4	100	-19.77	15291
MFNG_t1	-1.64	9.82E-14	-0.26	120	-18.47	15650	NA	NA	NA	NA	-0.41	117	-27.16	16139
CYP8B1	-3.18	8.40E-12	-0.26	120	-18.49	15651	-0.3	136	-25.81	16372	-0.22	118	-14.77	14319
FAM180A	-1.22	5.51E-15	-0.23	132	-18.5	15652	-0.29	128	-22.61	16220	-0.24	131	-19.33	15225
LAMC3	-2.14	7.77E-16	-0.25	125	-18.5	15653	-0.11	146	-10.85	14192	-0.32	120	-22.16	15680
SOWAHC	-1.47	4.11E-10	-0.18	151	-18.5	15654	-0.12	133	-9.91	13847	-0.15	150	-14.5	14244
FGFR1	-2.28	1.36E-17	-0.26	120	-18.51	15655	-0.09	139	-8.1	13155	-0.33	152	-33.75	16411
GPRASP1	-1.52	7.23E-13	-0.24	128	-18.51	15656	-0.09	116	-5.75	12061	-0.22	125	-16.54	14696
BEGAIN	-1.15	0.002591503	-0.34	104	-18.51	15657	-0.17	111	-10.13	13930	-0.28	138	-24.58	15921
REEP6	-2.05	1.31E-10	-0.3	112	-18.51	15658	-0.19	128	-15.03	15204	-0.28	100	-13.82	14059
MGLL	-1.84	2.33E-12	-0.28	116	-18.51	15659	-0.2	127	-15.26	15267	-0.29	142	-26.51	16088
CYB5D1	-1.04	0.014624858	-0.19	145	-18.51	15660	-0.1	128	-7.57	12915	-0.15	127	-11.43	13377
UBR4	-1.19	1.27E-07	-0.2	143	-18.52	15661	-0.13	105	-7.03	12669	-0.24	104	-12.87	13807
PPAPDC1A	-1.69	1.00E-17	-0.28	117	-18.52	15662	-0.1	106	-5.59	11965	-0.32	115	-20.77	15460
SLC27A2	-3.15	1.11E-18	-0.26	120	-18.53	15663	-0.34	118	-23.39	16266	-0.25	112	-15.23	14424
PRKRA	-1.44	2.75E-18	-0.23	132	-18.55	15664	-0.11	145	-10.57	14096	-0.29	127	-22.1	15676
PLA2G16	-2.26	2.15E-14	-0.29	113	-18.56	15665	-0.28	93	-12.13	14545	-0.32	133	-26.76	16108
ANGPTL2	-1.1	0.066334447	-0.17	162	-18.56	15666	-0.02	140	-1.66	9597	-0.15	131	-11.86	13506
CCDC48	-1.69	1.23E-18	-0.27	120	-18.56	15667	-0.16	124	-11.51	14373	-0.31	110	-18.49	15079
EPB41L4A	-1.47	1.93E-12	-0.21	139	-18.57	15668	-0.12	147	-11.48	14368	-0.33	134	-27.41	16156
FBXO21	-1.5	3.13E-11	-0.26	122	-18.58	15669	-0.14	139	-12.4	14614	-0.27	139	-24	15868
GLUD2	-2.32	8.06E-19	-0.31	110	-18.58	15670	-0.2	103	-10.82	14183	-0.32	120	-22.56	15726
SERINC1	-1.53	1.58E-09	-0.17	157	-18.59	15671	-0.21	115	-13.91	14974	-0.21	128	-16.65	14726
ST6GALNAC6	-1.6	9.64E-17	-0.27	120	-18.59	15672	-0.2	128	-15.39	15286	-0.38	132	-31.21	16335
SLC25A25	-2.63	1.56E-15	-0.18	151	-18.6	15673	-0.3	137	-26.42	16399	-0.22	144	-20.84	15477
DNAJC12	-2.57	1.94E-15	-0.21	138	-18.62	15674	-0.19	115	-12.22	14564	-0.26	120	-18.41	15067
ANPEP	-2.02	7.53E-16	-0.36	102	-18.62	15675	-0.28	104	-15.17	15239	-0.32	107	-18.03	14984
MT1A	-3.86	4.37E-18	-0.34	105	-18.62	15676	-0.25	80	-7.47	12861	-0.32	100	-15.94	14573
POLR2E	-1.25	3.97E-09	-0.23	131	-18.63	15677	-0.18	129	-14.01	14994	-0.24	127	-18.63	15103
NR2F2	-1.38	5.58E-08	-0.22	134	-18.65	15678	-0.05	132	-3.72	10941	-0.18	135	-15.68	14516
TRIM50	-1.12	0.005739417	-0.24	128	-18.65	15679	-0.15	119	-10.46	14049	-0.24	97	-11.27	13337
FCGRT	-1.81	4.98E-21	-0.35	104	-18.65	15680	-0.35	107	-19.92	15977	-0.38	93	-16.39	14663
PLEKHG1	-1.19	2.38E-06	-0.2	145	-18.65	15681	-0.01	144	-0.91	9084	-0.16	129	-12.25	13631
CDO1	-2.5	3.22E-09	-0.24	129	-18.67	15682	-0.24	110	-14.22	15034	-0.15	128	-11.73	13468
BMP6	-1.76	1.08E-24	-0.28	117	-18.68	15683	-0.25	123	-18.02	15737	-0.37	140	-33.27	16399
FAM50B	-1.33	9.68E-05	-0.22	134	-18.68	15684	-0.19	120	-13.09	14772	-0.19	122	-14.01	14117
STEAP3_t1	-1.63	5.26E-16	-0.27	120	-18.69	15685	NA	NA	NA	NA	-0.26	128	-20.67	15439
RNF135_t2	-1.87	6.86E-17	-0.24	129	-18.69	15686	-0.11	130	-8.65	13374	-0.38	119	-26.06	16039
GPR52	-1.2	1.37E-10	-0.2	145	-18.7	15687	-0.23	107	-13.24	14811	-0.2	100	-9.87	12882
NID1	-1.37	3.59E-10	-0.21	140	-18.7	15688	-0.24	142	-21.74	16149	-0.14	144	-13.26	13910
ETS2_t1	-2.23	5.61E-26	-0.25	125	-18.71	15689	NA	NA	NA	NA	NA	NA	NA	NA
RBM6	-1.17	5.12E-05	-0.32	109	-18.72	15690	-0.06	103	-3.22	10614	-0.18	105	-10.12	12962
ID4	-2.44	5.46E-15	-0.22	134	-18.72	15691	-0.06	133	-4.64	11458	-0.3	131	-24.44	15910
CBS	-1.6	3.63E-09	-0.27	120	-18.72	15692	-0.31	128	-24.04	16306	-0.23	111	-13.75	14038
NAV2_t1	-1.13	4.38E-08	-0.23	131	-18.72	15693	NA	NA	NA	NA	-0.15	146	-14.25	14173
PNPLA3	-1.59	1.76E-05	-0.26	121	-18.74	15694	-0.21	124	-15.3	15275	-0.21	133	-17.14	14823
CDKN1A_t1	-1.33	1.01E-10	-0.23	131	-18.74	15695	-0.22	126	-16.79	15561	-0.26	156	-27.21	16145
ZSCAN22	-1.74	2.40E-20	-0.23	131	-18.75	15696	-0.29	144	-27.6	16429	-0.21	143	-19.46	15242
VNN1	-4.65	7.29E-19	-0.22	136	-18.75	15697	-0.17	141	-15.88	15386	-0.23	126	-17.84	14952

PTGR1	-1.81	4.96E-09	-0.28	117	-18.75	15698	-0.1	110	-5.74	12049	-0.27	110	-16.1	14609
GCKR	-2.3	2.82E-16	-0.36	102	-18.76	15699	-0.3	104	-15.98	15411	-0.33	108	-19.17	15201
VSIG4	-3.94	9.17E-36	-0.3	113	-18.77	15700	-0.26	117	-17.68	15694	-0.42	118	-28.31	16210
QSOX1	-3	1.71E-17	-0.2	143	-18.77	15701	-0.12	129	-9.48	13694	-0.3	122	-21.44	15575
SLC10A1	-3.35	4.15E-17	-0.34	106	-18.77	15702	-0.4	116	-26.43	16400	-0.25	115	-16.33	14653
DIP2C	-1.56	1.52E-11	-0.23	133	-18.78	15703	-0.24	132	-19.44	15922	-0.23	143	-21.68	15617
SIGLEC10	-1.28	3.17E-12	-0.19	149	-18.78	15704	-0.07	158	-7.7	12983	-0.39	132	-31.66	16347
CRISPLD1	-1.22	3.59E-15	-0.23	132	-18.78	15705	-0.24	128	-18.36	15782	-0.23	164	-26.43	16081
ADM2	-1.26	4.08E-10	-0.24	128	-18.79	15706	-0.25	140	-22.26	16195	-0.16	152	-15.96	14579
ZEB2	-1.99	1.70E-17	-0.22	137	-18.8	15707	-0.1	127	-7.46	12856	-0.33	155	-34.69	16429
SAT2	-1.77	2.93E-13	-0.24	128	-18.8	15708	-0.3	121	-21	16087	-0.24	100	-12.24	13620
MUM1L1	-1.6	3.33E-09	-0.23	133	-18.81	15709	-0.06	139	-5.06	11690	-0.27	164	-31	16332
CYP1A1	-1.55	0.001340735	-0.22	135	-18.82	15710	-0.23	141	-21.14	16106	-0.09	128	-7.34	11972
DCAF11	-1.47	1.50E-08	-0.19	151	-18.82	15711	-0.3	139	-26.61	16402	-0.21	126	-15.93	14569
ADK	-1.84	1.73E-13	-0.27	121	-18.82	15712	-0.29	128	-22.62	16221	-0.21	129	-16.81	14759
CTH	-3.55	9.35E-13	-0.2	144	-18.82	15713	-0.29	123	-21.09	16100	-0.23	131	-18.71	15122
TMPRSS2	-2.25	1.75E-19	-0.21	140	-18.84	15714	-0.29	125	-21.96	16162	-0.26	124	-19.52	15253
EIF2AK4	-1.44	5.83E-12	-0.23	131	-18.84	15715	-0.25	131	-20.07	15996	-0.13	120	-8.82	12531
GADD45A	-2.22	3.42E-18	-0.25	124	-18.84	15716	-0.27	106	-14.93	15189	-0.27	147	-25.82	16017
MMP21	-1.15	4.26E-12	-0.19	148	-18.84	15717	-0.27	98	-12.95	14743	-0.2	124	-14.73	14303
JAM2	-1.58	1.60E-11	-0.18	153	-18.84	15718	-0.1	142	-9.6	13735	-0.29	140	-25.7	16001
NEK6	-1.32	5.98E-08	-0.18	155	-18.85	15719	0.01	121	0.8	7944	-0.33	120	-23.21	15794
SORBS2_t1	-1.62	1.36E-12	-0.24	129	-18.85	15720	NA	NA	NA	NA	-0.24	131	-19.07	15187
EHD1	-1.19	7.98E-06	-0.18	156	-18.85	15721	0.03	137	2.63	6730	-0.26	135	-22.48	15718
ARRDC4	-2.03	1.01E-09	-0.25	124	-18.87	15722	-0.22	128	-17.41	15661	-0.17	136	-14.9	14354
BCKDHB_t2	-1.48	8.02E-13	-0.23	133	-18.88	15723	-0.21	151	-21.42	16134	-0.19	130	-15.29	14433
DAPK1	-1.11	4.08E-06	-0.19	149	-18.88	15724	-0.13	125	-9.89	13840	-0.27	136	-23.34	15806
B3GNT8	-1.24	1.95E-10	-0.27	119	-18.91	15725	-0.03	127	-2.43	10106	-0.36	123	-26.04	16036
DMD_t3	-1.09	3.91E-07	-0.2	145	-18.92	15726	-0.25	134	-21.02	16089	-0.16	140	-14.59	14265
GSTA3	-2.76	7.85E-12	-0.28	117	-18.93	15727	-0.36	108	-20.99	16086	-0.21	107	-12.04	13558
ZSWIM7	-1.26	8.34E-08	-0.23	134	-18.94	15728	-0.16	114	-9.97	13872	-0.14	127	-10.9	13217
NUMB	-1.25	1.93E-11	-0.27	120	-18.95	15729	-0.22	123	-15.85	15376	-0.22	134	-18.18	15015
GCDH	-2.12	7.65E-17	-0.34	106	-18.96	15730	-0.33	113	-20.94	16080	-0.31	100	-15.5	14481
MCL1_t1	-1.83	8.00E-17	-0.2	147	-18.96	15731	NA	NA	NA	NA	-0.25	143	-22.84	15760
MRPS36	-1.25	5.55E-07	-0.22	136	-18.97	15732	-0.15	106	-8.43	13300	-0.07	122	-5.4	11200
SLC14A1	-1.08	0.022836725	-0.21	139	-18.97	15733	-0.02	137	-2.03	9842	-0.14	137	-12.24	13623
KCNK5	-1.53	1.99E-06	-0.23	133	-18.99	15734	-0.16	127	-12.68	14685	-0.25	120	-17.82	14947
CDA	-1.79	1.23E-16	-0.26	124	-19	15735	-0.27	146	-25.91	16374	-0.31	124	-23.04	15781
GYS2	-2	3.90E-20	-0.34	106	-19	15736	-0.3	111	-18.56	15805	-0.27	120	-18.99	15171
POU2F2	-1.12	2.46E-12	-0.24	130	-19.02	15737	-0.14	134	-11.77	14440	-0.27	145	-25.79	16013
SLC31A2	-2.21	1.18E-25	-0.25	127	-19.02	15738	-0.22	122	-16.04	15430	-0.36	133	-30.14	16293
TANK_t2	-1.43	8.03E-10	-0.2	144	-19.03	15739	-0.12	124	-8.8	13435	-0.28	114	-17.65	14912
DENND4C	-1.35	3.58E-11	-0.23	133	-19.03	15740	-0.16	120	-11	14236	-0.14	149	-14.12	14142
DNASE1L3	-9.31	7.69E-49	-0.33	108	-19.05	15741	-0.34	88	-12.74	14701	-0.4	89	-15.79	14536
NT5DC1	-1.37	2.24E-07	-0.19	152	-19.06	15742	-0.18	125	-13.29	14820	-0.08	125	-6.12	11485
RALGAPA2	-1.31	6.79E-07	-0.24	129	-19.06	15743	-0.25	134	-21.27	16121	-0.14	156	-14.6	14270
RCL1	-3.59	8.85E-25	-0.31	111	-19.07	15744	-0.33	123	-23.76	16292	-0.35	118	-23.48	15827
IL2RB	-2.12	1.80E-20	-0.22	137	-19.07	15745	-0.17	141	-15.36	15282	-0.35	132	-28.71	16240
HEPN1	-1.37	5.08E-05	-0.26	123	-19.08	15746	-0.24	109	-14.43	15086	-0.24	110	-14.61	14272
CDH1	-2.62	7.13E-18	-0.23	134	-19.08	15747	-0.21	144	-20.08	15997	-0.25	129	-19.66	15276
SAMD11	-2.15	2.28E-24	-0.35	105	-19.09	15748	-0.17	100	-8.52	13332	-0.41	93	-17.42	14868
CAMK2D_t2	-2.06	6.38E-22	-0.24	130	-19.09	15749	-0.28	148	-27.07	16414	-0.28	152	-29.04	16260
TAB2	-1.45	1.51E-07	-0.25	126	-19.1	15750	-0.09	120	-6.65	12487	-0.18	140	-16.64	14721
SLC25A47	-5.26	7.20E-34	-0.37	102	-19.11	15751	-0.33	117	-22.14	16182	-0.44	114	-28.34	16211
SLC2A2	-2.53	5.96E-09	-0.23	132	-19.13	15752	-0.23	137	-20.44	16038	-0.21	154	-21.48	15587
ANXA8L2	-2.8	9.47E-24	-0.34	106	-19.15	15753	-0.21	124	-15.3	15274	-0.41	132	-33.46	16404
HRG	-3.6	8.55E-12	-0.26	123	-19.16	15754	-0.42	110	-25.01	16345	-0.26	110	-15.67	14514
APOA5	-3.11	2.99E-16	-0.26	123	-19.17	15755	-0.2	111	-12.42	14620	-0.31	104	-16.5	14683
ANGPTL3	-2.96	2.19E-16	-0.27	120	-19.17	15756	-0.31	133	-26.09	16382	-0.23	114	-14.77	14315
NCR3	-1.28	1.53E-13	-0.25	128	-19.17	15757	-0.2	100	-10.04	13897	-0.3	97	-14.31	14194
BAALC_t2	-1.3	2.61E-06	-0.25	128	-19.17	15758	-0.22	118	-14.99	15197	-0.18	131	-14.49	14239
SHPK	-1.62	5.08E-19	-0.26	124	-19.18	15759	-0.28	139	-24.99	16343	-0.28	150	-28.17	16200
ASL	-1.94	1.48E-13	-0.24	130	-19.18	15760	-0.33	119	-22.7	16228	-0.22	104	-11.71	13461
PDE1B	-1.24	7.06E-17	-0.26	124	-19.19	15761	-0.26	106	-14.54	15112	-0.25	132	-20.21	15370
SV2B	-1.13	0.153158365	-0.25	128	-19.2	15762	0.01	156	1.21	7673	-0.08	131	-6.19	11520
CRHBP	-15.16	5.25E-56	-0.34	106	-19.21	15763	-0.31	115	-19.92	15976	-0.39	135	-33.55	16407
AMDHD1	-2.79	3.74E-15	-0.27	122	-19.22	15764	-0.34	116	-22.53	16212	-0.21	122	-14.78	14323
ZFPM2	-1.19	1.00E-10	-0.22	139	-19.22	15765	-0.05	120	-3.53	10820	-0.28	150	-28.24	16205
ZBTB43	-1.25	6.08E-11	-0.21	142	-19.22	15766	-0.17	160	-18.35	15780	-0.23	141	-20.83	15474
CD1E_t2	-1.31	9.83E-11	-0.2	144	-19.23	15767	-0.02	119	-1.28	9348	-0.33	128	-25.73	16004
NAAA_t1	-1.88	9.99E-26	-0.29	116	-19.24	15768	-0.22	131	-18.11	15751	-0.42	127	-32.42	16377
DNAJC22	-1.44	2.82E-08	-0.25	127	-19.24	15769	-0.16	135	-13.82	14950	-0.19	120	-13.38	13947
PRICKLE2	-1.63	6.33E-17	-0.21	142	-19.24	15770	-0.07	159	-7.84	13049	-0.28	146	-26.5	16087
MPDZ	-1.98	2.67E-14	-0.29	116	-19.24	15771	-0.2	120	-14.31	15056	-0.2	143	-18.33	15050
CX3CR1	-1.54	5.80E-09	-0.24	130	-19.25	15772	0.04	148	4.21	5771	-0.31	127	-24.18	15887
ITIH4	-2.1	8.00E-10	-0.3	114	-19.25	15773	-0.37	118	-25.19	16353	-0.32	112	-19.63	15271
OLFML3_t1	-2.28	9.32E-30	-0.34	107	-19.26	15774	NA	NA	NA	NA	-0.43	135	-36.4	16453
FGFR2_t2	-1.78	1.62E-11	-0.26	124	-19.26	15775	-0.11	112	-7.01	12654	-0.29	135	-24.3	15903
RDH16	-4.05	6.93E-25	-0.34	107	-19.27	15776	-0.38	124	-28.09	16438	-0.31	105	-17.02	14802
RASSF5_t2	-1.1	4.63E-11	-0.2	147	-19.28	15777	-0.11	148	-10.91	14207	-0.31	150	-31.17	16334
EPHB3	-1.29	4.37E-07	-0.29	116	-19.33	15778	-0.06	116	-4.14	11174	-0.37	126	-28.5	16225
PTN_t1	-2.48	1.35E-20	-0.26	124	-19.33	15779	NA	NA	NA	NA	-0.33	147	-31.99	16363
EPB41L4B_t2	-1.33	1.42E-09	-0.2	146	-19.33	15780	-0.19	120	-13.42	14852	-0.18	138	-16.22	14628
SULT2A1	-3.03	1.31E-11	-0.24	130	-19.34	15781	-0.28	131	-22.43	16209	-0.24	131	-19.66	15274
UGT2B10	-1.96	3.26E-09	-0.24	129	-19.34	15782	-0.27	131	-22	16169	-0.22	143	-20.42	15400
ITIH1	-1.96	1.96E-09	-0.3	115	-19.36	15783	-0.41	98	-19.49	15927	-0.22	101	-11.4	13370
ABCG5	-2	8.97E-08	-0.27	122	-19.36	15784	-0.29	122	-21.05	16095	-0.23	126	-17.42	14867
PTH1R	-3.64	1.11E-40	-0.4	99	-19.39	15785	-0.28	107	-16.12	15446	-0.46	85	-16.05	14601
FAM176A	-1.97	1.22E-10	-0.23	133	-19.4	15786	-0.25	115	-16.56	15518	-0.26	121	-18.11	14998
FLT3	-1.56	3.26E-20	-0.29	116	-19.41	15787	-0.13	134	-10.75	14159	-0.36	109	-21.04	15507
SERPINA1	-1.62	1.17E-10	-0.26	126	-19.41	15788	-0.27	134	-22.74	16232	-0.26	119	-17.85	14953
SH3BP2_t1	-1.3	4.02E-12	-0.18	160	-19.42	15789	-0.15	130	-11.97	14492	NA	NA	NA	NA
EPHA3_t2	-1.19	7.04E-13	-0.25	129	-19.42	15790	NA	NA	NA	NA	-0.3	123	-21.62	15609
C6	-4.91	4.93E-23	-0.29	116	-19.42	15791	-0.38	119	-26.06	16381	-0.25	139	-22.11	15677
SLC35D1	-1.31	3.58E-07	-0.18	159	-19.43	15792	-0.19	144	-17.77	15704	-0.16	152	-16.46	14673
IL4R	-1.92	7.04E-19	-0.28	120	-19.43	15793	-0.25	108	-14.52	15107	-0.32	116	-20.95	15491
TNFRSF10C	-1.52	8.85E-21	-0.26	124	-19.48	15794	-0.13	125	-10.02	13889	-0.36	100	-17.93	14969
SLC16A5	-2.19	6.81E-23	-0.32	111	-19.5	15795	-0.09	100	-4.37	11295	-0.37	90	-14.84	14334
FLRT2_t1	-1.66	1.09E-12	-0.24	130	-19.52	15796	NA	NA	NA	NA	-0.3	140	-26.67	16102
AK4	-1.43	0.000387914	-0.21	144	-19.52	15797	-0.05	139	-4.88	11585	-0.08	146	-7.96	12225
THEG	-1.06	0.220381771	-0.31	114	-19.53	15798	-0.05	104	-2.5	10157	-0.16	119	-11.34	13359
PFN1P2	-1.28	7.73E-13	-0.24	132	-19.53	15799	-0.11	124	-8.36	13266	-0.26	134	-21.79	15628
OXT	-1.88	2.42E-13	-0.28	120	-19.53	15800	-0.17	104	-9.3	13624	-0.29	80	-8.8	12527
IDNK	-2.33	1.28E-21	-0.31	114	-19.53	15801	-0.28	118	-18.79	15840	-0.31	121	-22.03	15664
ABCA8	-2.27	2.77E-14	-0.26	126	-19.56	15802	-0.3	119	-20.82	16073	-0.24	138	-20.85	15479
PINK1	-1.52	1.41E-14	-0.27	122	-19.56	15803	-0.24	132	-20.07	15993	-0.27	116	-17.6	14905

RFPL2	-1.26	1.80E-06	-0.23	134	-19.57	15804	-0.09	120	-6.55	12434	-0.15	136	-12.57	13718
MT1E	-6.43	4.34E-20	-0.35	106	-19.57	15805	-0.27	112	-16.63	15534	-0.33	104	-17.58	14899
SLC41A2	-2	7.61E-14	-0.26	124	-19.57	15806	-0.27	129	-21.24	16115	-0.19	116	-12.52	13702
RGS11	-1.34	2.35E-12	-0.27	122	-19.57	15807	-0.09	120	-6.19	12283	-0.29	107	-16.76	14746
EFNB3	-1.52	9.03E-29	-0.33	109	-19.57	15808	-0.29	144	-27.18	16418	-0.37	116	-24.63	15927
ACADM	-2.05	3.66E-14	-0.21	145	-19.61	15809	-0.24	122	-17.48	15668	-0.19	138	-16.91	14787
BST1	-1.78	1.53E-18	-0.3	116	-19.62	15810	-0.08	122	-5.68	12019	-0.32	111	-19.5	15247
DPYS	-2.27	1.73E-09	-0.28	120	-19.62	15811	-0.33	115	-21.19	16111	-0.2	138	-17.49	14879
CAPN3	-1.43	1.18E-13	-0.3	116	-19.62	15812	-0.21	134	-17.29	15639	-0.29	127	-21.99	15659
SLC6A5	-1.57	1.02E-16	-0.28	120	-19.63	15813	-0.33	98	-16.01	15422	-0.32	95	-14.34	14202
GPR171	-1.93	5.14E-14	-0.22	139	-19.64	15814	-0.1	104	-5.32	11829	-0.34	111	-20.6	15429
HAS2	-1.26	3.50E-11	-0.28	120	-19.64	15815	-0.17	128	-13.41	14851	-0.31	139	-27.76	16173
SYAP1	-1.45	7.27E-10	-0.23	136	-19.65	15816	-0.15	138	-13.61	14901	-0.15	128	-11.73	13469
EHHADH	-2	3.73E-11	-0.25	128	-19.65	15817	-0.21	133	-17.61	15681	-0.16	116	-10.71	13153
TNN	-1.64	3.93E-14	-0.28	120	-19.65	15818	-0.19	125	-14.52	15110	-0.23	128	-18.2	15021
PTGER2	-1.62	4.05E-19	-0.25	130	-19.66	15819	-0.15	116	-10.2	13951	-0.32	145	-30.33	16305
SHMT1	-2.27	2.35E-13	-0.25	129	-19.66	15820	-0.37	134	-31.29	16473	-0.14	118	-9.72	12815
NAAA_t2	-2.64	1.57E-29	-0.29	117	-19.66	15821	-0.26	130	-20.89	16077	-0.38	120	-26.28	16068
SLC17A8	-1.01	0.317380143	-0.23	136	-19.68	15822	-0.12	126	-9.23	13600	-0.22	121	-15.89	14559
SUSD3	-1.59	2.91E-05	-0.18	161	-19.7	15823	0.04	119	2.81	6610	-0.17	105	-9.48	12753
RPH3AL	-1.55	2.76E-24	-0.29	119	-19.71	15824	-0.31	127	-23.98	16303	-0.38	133	-31.71	16352
ADAMTS1_t1	-2.36	7.11E-19	-0.26	127	-19.71	15825	NA	NA	NA	NA	NA	NA	NA	NA
DCAF8	-1.04	0.28654453	-0.22	140	-19.71	15826	-0.18	139	-15.81	15365	-0.06	143	-5.89	11402
DRAXIN	-1.04	0.001455385	-0.21	145	-19.72	15827	-0.09	120	-6.47	12402	-0.11	122	-7.69	12108
CAMK2B	-1.8	8.16E-15	-0.28	120	-19.73	15828	-0.24	136	-20.54	16048	-0.25	120	-17.16	14826
SPATA6L	-1.67	4.51E-17	-0.21	143	-19.73	15829	-0.21	134	-17.48	15669	-0.3	127	-22.99	15775
ALAS1	-1.68	3.94E-12	-0.3	116	-19.73	15830	-0.21	99	-10.26	13982	-0.26	118	-17.37	14858
ACOT1	-1.54	3.03E-08	-0.25	129	-19.74	15831	-0.28	113	-17.66	15691	-0.15	88	-5.79	11355
PIK3R1	-1.37	9.89E-11	-0.21	142	-19.75	15832	-0.13	132	-10.52	14073	-0.16	132	-13.15	13885
CYR61_t1	-4.68	3.93E-25	-0.27	123	-19.75	15833	-0.16	140	-14.82	15175	NA	NA	NA	NA
MT1X	-4.38	1.39E-18	-0.35	107	-19.77	15834	-0.25	92	-10.67	14122	-0.31	119	-21.6	15606
NMUR1	-1.49	2.75E-19	-0.3	115	-19.77	15835	-0.16	131	-13.21	14803	-0.34	124	-25.07	15960
GSTA1	-2.58	5.21E-10	-0.25	129	-19.78	15836	-0.37	119	-25.55	16365	-0.18	128	-14.08	14134
ARHGEF26	-1.94	3.31E-16	-0.24	132	-19.78	15837	-0.33	125	-24.77	16333	-0.19	145	-18.33	15049
HNRNPA0	-1.44	2.90E-13	-0.23	136	-19.79	15838	-0.18	117	-11.73	14432	-0.21	124	-15.36	14452
RPS27L	-1.32	2.70E-07	-0.25	130	-19.8	15839	-0.16	129	-12.34	14598	-0.14	123	-10.21	12992
GPBAR1	-1.32	2.03E-17	-0.28	120	-19.8	15840	-0.13	104	-7.12	12703	-0.4	106	-22.17	15685
PHF10	-1.39	5.49E-12	-0.26	125	-19.8	15841	-0.21	103	-10.94	14217	-0.2	105	-11.24	13320
DGAT2	-1.59	8.90E-06	-0.28	121	-19.8	15842	-0.24	118	-16.41	15498	-0.17	133	-14.21	14157
NTRK2_t2	-1.51	2.92E-08	-0.23	137	-19.82	15843	-0.11	128	-8.66	13379	-0.33	137	-28.48	16222
KRT81	-1.79	3.08E-21	-0.22	140	-19.82	15844	-0.26	121	-18.33	15777	-0.33	120	-23.25	15799
PQLC1	-1.66	4.80E-21	-0.29	119	-19.83	15845	-0.28	124	-20.55	16049	-0.37	150	-37.36	16466
MMAA	-1.6	2.68E-13	-0.23	137	-19.83	15846	-0.16	126	-12.24	14569	-0.24	143	-22.36	15706
STRADB	-1.45	9.08E-15	-0.19	156	-19.84	15847	-0.17	121	-12.36	14606	-0.17	122	-12.48	13694
HMOX1	-3.07	7.62E-22	-0.31	115	-19.84	15848	-0.18	110	-10.57	14097	-0.39	104	-20.82	15472
TMEM57	-1.33	6.04E-09	-0.28	120	-19.85	15849	-0.19	136	-16.49	15509	-0.15	140	-13.95	14098
CSRNP3	-1.06	1.36E-06	-0.23	137	-19.86	15850	-0.1	140	-8.72	13404	-0.18	139	-15.61	14494
PRRG4	-1.61	1.64E-16	-0.22	141	-19.86	15851	-0.27	135	-22.7	16227	-0.19	168	-21.99	15658
AKAP12	-1.42	1.52E-07	-0.25	128	-19.86	15852	-0.18	136	-15.22	15255	-0.11	125	-8.19	12311
CLC	-1.51	5.98E-13	-0.29	119	-19.86	15853	-0.14	115	-9.26	13610	-0.32	94	-14.25	14172
CYP4F2_t1	-1.87	7.35E-14	-0.31	114	-19.86	15854	-0.3	127	-23.12	16255	NA	NA	NA	NA
CSRNP1	-3.51	1.50E-35	-0.3	117	-19.87	15855	-0.3	161	-33.62	16483	-0.39	144	-36.34	16452
ID1	-6.19	7.50E-31	-0.35	107	-19.88	15856	-0.21	108	-12.04	14516	-0.39	117	-26.05	16038
LTK	-2.28	8.30E-37	-0.38	103	-19.89	15857	-0.25	128	-19.51	15931	-0.45	128	-35.04	16435
BAG1	-1.31	1.27E-07	-0.23	136	-19.89	15858	-0.24	127	-18.31	15773	-0.2	129	-15.84	14548
PYROXD2	-1.93	7.44E-17	-0.36	105	-19.9	15859	-0.16	108	-9.32	13627	-0.4	93	-17.34	14850
AGMO	-1.82	4.34E-09	-0.26	127	-19.9	15860	-0.27	135	-22.69	16226	-0.16	130	-12.72	13767
GPM6B	-1.19	3.30E-07	-0.26	127	-19.9	15861	-0.05	161	-5.77	12077	-0.25	113	-15.94	14574
STBD1	-1.67	5.91E-12	-0.24	132	-19.91	15862	-0.27	123	-19.42	15918	-0.18	126	-13.83	14062
ARHGAP10	-2.1	2.68E-23	-0.28	120	-19.91	15863	-0.17	125	-12.82	14713	-0.35	120	-24.58	15922
NR3C2	-1.68	2.21E-19	-0.24	133	-19.92	15864	-0.26	139	-22.84	16240	-0.27	149	-26.3	16072
CIRBP	-1.33	7.46E-10	-0.25	131	-19.93	15865	-0.14	134	-11.82	14452	-0.35	123	-25.83	16019
ALDH3A2	-1.4	6.59E-06	-0.21	144	-19.93	15866	-0.23	136	-19.39	15913	-0.09	161	-10.16	12977
VPS37B	-1.6	1.79E-18	-0.28	121	-19.94	15867	-0.12	164	-13.22	14806	-0.4	144	-37.2	16464
GINM1	-1.31	9.89E-10	-0.29	120	-19.98	15868	-0.19	106	-10.37	14028	-0.21	112	-12.96	13834
CDC37L1	-2.19	1.04E-23	-0.29	120	-19.99	15869	-0.27	120	-18.94	15857	-0.3	126	-22.68	15739
MTHFS	-1.61	2.26E-09	-0.26	128	-20	15870	-0.32	155	-33.63	16485	-0.15	128	-11.74	13472
SULF2	-3.06	1.40E-18	-0.35	108	-20.03	15871	-0.15	125	-11.08	14264	-0.32	123	-23.41	15816
ADAM8	-1.58	5.26E-08	-0.23	138	-20.03	15872	-0.06	117	-4.02	11109	-0.28	113	-17.46	14871
GJB1	-1.23	0.007494795	-0.23	136	-20.04	15873	-0.21	114	-13.54	14880	-0.12	114	-7.68	12102
ANXA6	-1.53	3.76E-12	-0.28	122	-20.05	15874	-0.21	126	-15.99	15415	-0.24	120	-16.91	14786
PROK2	-2.16	1.69E-13	-0.23	136	-20.05	15875	-0.17	143	-16.04	15429	-0.28	126	-21.16	15528
COQ6	-1.22	2.22E-06	-0.28	122	-20.05	15876	-0.25	108	-14.23	15038	-0.2	101	-10.39	13050
PPP1R3B	-1.84	1.67E-18	-0.25	129	-20.06	15877	-0.25	132	-20.54	16047	-0.28	128	-21.48	15584
SORD_t1	-2.42	1.69E-12	-0.26	127	-20.06	15878	-0.32	129	-25.15	16351	NA	NA	NA	NA
PRKAG2	-1.67	2.06E-16	-0.25	129	-20.08	15879	-0.28	124	-20.51	16044	-0.26	131	-21.35	15561
MTHFD1	-2.32	6.15E-17	-0.28	121	-20.08	15880	-0.35	126	-26.4	16396	-0.32	112	-19.94	15314
CBFA2T3	-1.98	8.70E-26	-0.29	120	-20.09	15881	-0.19	122	-13.89	14967	-0.39	130	-30.91	16329
BBOX1	-4.74	6.40E-18	-0.36	106	-20.09	15882	-0.3	104	-16.02	15425	-0.18	117	-12.13	13585
WSB1	-1.74	1.31E-13	-0.23	136	-20.1	15883	-0.05	139	-4.63	11456	-0.27	127	-20.74	15451
APOL4	-1.51	8.97E-13	-0.25	130	-20.1	15884	-0.23	163	-26.29	16394	-0.24	127	-18.78	15134
AOC3	-1.77	1.25E-14	-0.29	119	-20.13	15885	-0.11	143	-10.57	14093	-0.31	121	-22.09	15671
RAPH1_t1	-1.81	9.70E-17	-0.23	137	-20.13	15886	-0.24	154	-25.36	16358	NA	NA	NA	NA
LRRC19	-1.47	2.73E-06	-0.2	152	-20.14	15887	-0.15	151	-15.1	15221	-0.18	127	-13.86	14066
GRPEL1	-1.48	2.07E-16	-0.26	127	-20.14	15888	-0.31	123	-22.71	16229	-0.24	113	-15.06	14392
CCDC28A	-1.41	1.02E-08	-0.2	153	-20.14	15889	-0.15	98	-6.97	12632	-0.2	124	-14.48	14234
EVC	-1.12	1.95E-05	-0.33	111	-20.15	15890	-0.09	140	-7.75	13008	-0.3	140	-27.07	16133
CACNB2	-1.03	0.43841052	-0.21	146	-20.17	15891	-0.1	137	-8.85	13451	-0.1	130	-8.07	12258
FBXO3_t2	-1.47	1.18E-12	-0.26	127	-20.18	15892	-0.26	122	-18.79	15841	-0.17	133	-14.2	14154
NPR1	-1.45	2.33E-16	-0.28	121	-20.19	15893	-0.17	125	-12.51	14643	-0.36	103	-19.18	15202
GBE1	-1.3	0.003036462	-0.19	154	-20.19	15894	-0.19	115	-12.12	14543	-0.14	119	-9.39	12712
PDLIM5_t1	-1.8	6.50E-22	-0.22	142	-20.2	15895	NA	NA	NA	NA	NA	NA	NA	NA
CHST7	-2.14	5.84E-19	-0.25	131	-20.2	15896	-0.24	148	-23.95	16300	-0.31	108	-18.12	14999
AKR7A3	-2.27	1.66E-17	-0.31	116	-20.2	15897	-0.39	110	-23.26	16262	-0.26	105	-14.42	14222
KCNN2	-4.03	2.78E-30	-0.33	111	-20.21	15898	-0.29	120	-20.58	16055	-0.35	134	-29.59	16274
IQGAP2	-1.68	1.41E-09	-0.26	129	-20.22	15899	-0.19	124	-14.18	15026	-0.14	122	-9.95	12912
ZNF592	-1.18	1.69E-07	-0.21	145	-20.23	15900	-0.14	136	-12.35	14600	-0.18	150	-17.73	14933
TMEM105	-1.19	1.08E-05	-0.23	140	-20.27	15901	-0.18	141	-16.02	15424	-0.17	126	-12.85	13804
AR	-2.99	1.02E-12	-0.24	136	-20.28	15902	-0.26	131	-21.07	16098	-0.21	146	-19.82	15299
SYT10	-1.08	1.13E-06	-0.21	145	-20.29	15903	-0.19	128	-14.98	15196	-0.14	151	-14.35	14205
FOXF1	-1.64	6.42E-17	-0.27	124	-20.3	15904	-0.14	121	-10.18	13944	-0.34	133	-28.3	16208
MYO15A_t1	-1.06	0.013705807	-0.27	124	-20.3	15905	NA	NA	NA	NA	-0.16	120	-10.98	13253
PRG4	-4.19	3.00E-13	-0.29	120	-20.31	15906	-0.24	143	-22.02	16172	-0.2	121	-13.94	14091
CHST3	-1.08	0.006559041	-0.2	150	-20.32	15907	-0.02	172	-3.04	10499	-0.25	130	-19.61	15264
ITGA9	-1.25	2.62E-21	-0.27	124	-20.35	15908	-0.11	122	-8.15	13173	-0.35	112	-21.55	15602
LYPD6B	-1.09	0.076504295	-0.26	128	-20.35	15909	-0.05	116	-3.2	10599	-0.36	150	-35.98	16449

LILRA2	-1.43	1.33E-22	-0.27	126	-20.37	15910	-0.17	109	-9.8	13817	-0.41	91	-16.75	14745
ASS1	-3.9	2.73E-25	-0.41	100	-20.37	15911	-0.36	102	-18.91	15854	-0.39	114	-24.82	15945
IL16_t2	-1.67	2.32E-18	-0.26	128	-20.37	15912	-0.13	146	-12.7	14692	-0.42	128	-32.52	16380
MS4A7	-3.06	2.75E-27	-0.27	126	-20.37	15913	-0.18	151	-18.32	15774	-0.42	167	-49.37	16496
CNTN4_t2	-1.23	1.86E-13	-0.27	126	-20.37	15914	-0.1	128	-7.54	12894	-0.3	138	-26.58	16095
MT1G_t1	-5.3	3.55E-22	-0.35	109	-20.38	15915	NA	NA	NA	NA	NA	NA	NA	NA
ATP11C_t2	-1.19	4.09E-07	-0.2	154	-20.38	15916	-0.19	129	-14.9	15185	-0.15	132	-12.51	13701
DPT	-2.43	7.38E-22	-0.33	112	-20.39	15917	-0.21	149	-20.44	16037	-0.37	129	-29.08	16262
ABCA9	-2.26	4.19E-23	-0.26	128	-20.4	15918	-0.32	134	-26.95	16412	-0.33	121	-23.36	15809
PIK3R4	-1.31	9.34E-14	-0.21	146	-20.41	15919	-0.28	107	-16.19	15457	-0.18	120	-12.65	13750
LPIN2_t1	-2	1.82E-15	-0.2	152	-20.42	15920	NA	NA	NA	NA	NA	NA	NA	NA
GABARAPL1	-2.9	1.53E-25	-0.33	112	-20.43	15921	-0.32	140	-28.79	16451	-0.35	148	-34.1	16417
GPR126_t1	-1.8	2.71E-16	-0.26	130	-20.48	15922	-0.2	122	-14.22	15035	-0.26	137	-22.2	15690
TTR	-3.38	4.09E-13	-0.29	120	-20.5	15923	-0.39	128	-30.11	16470	-0.3	137	-25.76	16007
TMEM125	-1.76	1.70E-10	-0.3	118	-20.51	15924	-0.09	122	-6.61	12471	-0.37	127	-28.46	16221
PROP1	-1.53	1.04E-14	-0.27	127	-20.52	15925	-0.26	104	-14.19	15027	-0.28	107	-15.83	14543
ANXA4	-1.31	0.000241115	-0.22	143	-20.56	15926	0.01	139	0.96	7826	-0.23	146	-22.12	15679
SLCO5A1	-2.43	1.00E-19	-0.29	120	-20.57	15927	-0.25	158	-27.44	16427	-0.22	113	-13.86	14067
KLK11	-1.31	7.04E-12	-0.33	112	-20.57	15928	-0.13	121	-9.44	13676	-0.36	118	-24.27	15900
DHRS1_t1	-2.28	6.62E-20	-0.32	115	-20.58	15929	NA	NA	NA	NA	NA	NA	NA	NA
C1S	-2.07	9.01E-11	-0.22	145	-20.58	15930	-0.3	147	-29.5	16456	-0.29	146	-27.92	16184
CD160	-1.43	2.08E-17	-0.27	126	-20.59	15931	-0.17	118	-11.83	14456	-0.32	107	-17.99	14976
DLG4	-1.24	1.33E-13	-0.23	141	-20.6	15932	-0.14	140	-12.5	14637	-0.34	122	-24.69	15934
APBA1	-1.13	2.93E-05	-0.23	140	-20.62	15933	-0.22	128	-16.8	15565	-0.2	139	-18.2	15022
SKAP1	-3.52	9.43E-25	-0.34	111	-20.64	15934	-0.26	122	-18.8	15842	-0.33	114	-20.85	15478
MYO10_t1	-1.47	1.43E-17	-0.24	137	-20.64	15935	NA	NA	NA	NA	-0.3	135	-25.23	15972
NSUN6	-1.72	1.81E-15	-0.26	129	-20.64	15936	-0.25	96	-11.66	14415	-0.26	106	-14.71	14300
GSTM2P1	-1.36	4.11E-11	-0.19	156	-20.64	15937	-0.28	142	-25.94	16376	-0.25	120	-17.25	14838
UPB1	-1.99	1.56E-10	-0.28	123	-20.68	15938	-0.31	121	-22.33	16199	-0.22	110	-13.44	13964
TSPAN12	-1.62	2.52E-10	-0.25	133	-20.69	15939	-0.11	119	-7.56	12901	-0.19	127	-14.55	14253
CBLN2	-1.06	0.036000313	-0.27	128	-20.7	15940	-0.15	136	-13.07	14767	-0.25	128	-19.7	15282
CFD	-3.61	3.02E-27	-0.27	128	-20.72	15941	-0.18	120	-12.34	14596	-0.42	130	-33.86	16412
ASGR2	-2.04	1.53E-12	-0.27	126	-20.73	15942	-0.3	100	-15.17	15241	-0.34	113	-21.49	15588
SAMD4A	-1.38	1.56E-07	-0.23	139	-20.74	15943	-0.23	128	-18.15	15754	-0.23	139	-20.76	15455
TSLP	-2.6	8.94E-21	-0.27	127	-20.76	15944	-0.26	133	-21.82	16155	-0.3	132	-24.62	15926
HSH2D	-1.59	7.07E-12	-0.24	136	-20.77	15945	-0.12	152	-12.3	14582	-0.38	120	-26.3	16070
DSCAML1	-1.2	4.28E-12	-0.32	115	-20.77	15946	-0.08	114	-5.1	11724	-0.37	110	-21.96	15654
PALM	-1.35	6.37E-11	-0.27	127	-20.78	15947	-0.15	145	-14.51	15103	-0.29	120	-20.01	15329
LTBP1	-1.79	2.02E-13	-0.22	144	-20.79	15948	-0.09	116	-6.06	12220	-0.29	145	-27.96	16190
GRAMD1C	-1.35	1.97E-17	-0.22	143	-20.79	15949	-0.25	120	-17.72	15699	-0.25	136	-21.32	15556
GADD45B	-3.76	2.98E-25	-0.22	145	-20.8	15950	-0.29	114	-18.81	15846	-0.32	158	-34.17	16418
RILPL2	-1.61	4.42E-16	-0.23	141	-20.81	15951	-0.13	120	-9.33	13631	-0.33	122	-24.11	15881
PEX13	-1.25	5.28E-07	-0.21	147	-20.81	15952	-0.17	131	-13.5	14873	-0.12	172	-14.35	14203
SYNE1_t2	-2.3	3.15E-30	-0.39	104	-20.81	15953	-0.22	114	-14.3	15053	-0.37	128	-28.57	16233
ANG	-2.45	4.15E-13	-0.32	116	-20.82	15954	-0.36	123	-26.29	16393	-0.3	112	-18.41	15066
PNPLA7	-1.59	3.49E-28	-0.34	111	-20.83	15955	-0.3	101	-15.21	15249	-0.45	117	-29.9	16283
CCL5	-2.12	5.41E-16	-0.25	133	-20.83	15956	-0.11	131	-8.96	13486	-0.39	128	-30.58	16320
MASP2_t2	-3.17	2.27E-16	-0.38	105	-20.85	15957	-0.31	115	-20.47	16040	-0.33	117	-22.31	15701
GCH1_t1	-2.16	5.00E-22	-0.27	128	-20.85	15958	NA	NA	NA	NA	-0.29	126	-21.77	15626
RSPO3_t1	-3.62	1.35E-29	-0.31	118	-20.85	15959	NA	NA	NA	NA	-0.37	119	-25.27	15974
KLRK1	-1.4	6.73E-14	-0.23	141	-20.89	15960	-0.1	130	-7.79	13023	-0.37	137	-31.84	16357
CXCL2_t1	-3.72	2.56E-29	-0.25	133	-20.89	15961	NA	NA	NA	NA	-0.29	121	-20.78	15463
NCKAP1L	-1.53	8.46E-17	-0.23	139	-20.9	15962	-0.12	108	-6.81	12565	-0.42	105	-22.97	15772
PVALB	-1.7	2.35E-28	-0.35	110	-20.92	15963	-0.31	122	-22.56	16217	-0.39	114	-25.11	15963
CACNA1A	-1.67	1.02E-14	-0.26	130	-20.93	15964	-0.38	141	-34.15	16486	-0.25	120	-17.47	14873
NR0B2	-2.88	2.70E-19	-0.32	115	-20.94	15965	-0.21	118	-13.95	14985	-0.34	123	-24.61	15925
RNF144B	-1.58	1.01E-13	-0.26	131	-20.95	15966	-0.16	125	-12.13	14546	-0.29	120	-20.22	15372
DIO3OS	-1.5	2.67E-09	-0.27	127	-20.96	15967	-0.21	145	-20.07	15994	-0.25	148	-24.11	15880
ACSM5	-3.1	6.01E-22	-0.33	114	-20.97	15968	-0.34	109	-20.23	16016	-0.34	120	-23.5	15830
FAM149A	-1.6	5.91E-19	-0.28	126	-20.97	15969	-0.33	119	-22.8	16235	-0.32	155	-34.07	16416
IL1B	-3.32	5.40E-22	-0.29	122	-20.99	15970	-0.11	117	-7.18	12729	-0.42	117	-27.83	16176
UNC13D	-1.42	5.58E-17	-0.3	120	-20.99	15971	-0.17	109	-9.79	13815	-0.46	106	-25.9	16025
XPC	-1.16	1.11E-06	-0.29	122	-21	15972	-0.3	114	-19.23	15894	-0.22	118	-14.78	14321
SLC16A4	-1.18	5.30E-07	-0.28	126	-21.04	15973	-0.21	125	-15.75	15353	-0.2	119	-13.58	13995
KCNH7_t2	-1.14	4.29E-14	-0.22	147	-21.06	15974	-0.18	115	-11.93	14486	-0.17	129	-13.52	13981
GIMAP5	-1.84	2.53E-19	-0.25	134	-21.07	15975	-0.18	120	-12.72	14697	-0.39	122	-27.84	16177
RFPL1	-1.35	1.54E-09	-0.25	134	-21.08	15976	-0.21	113	-12.97	14745	-0.21	104	-11.2	13306
ARRB1	-1.58	9.69E-22	-0.28	126	-21.08	15977	-0.2	150	-20.01	15987	-0.29	123	-21.03	15506
GABRP	-1.38	1.19E-15	-0.28	126	-21.1	15978	-0.23	138	-20.51	16045	-0.35	127	-27.28	16151
EPHX2	-3.12	3.26E-19	-0.37	107	-21.11	15979	-0.4	117	-26.64	16405	-0.35	118	-23.71	15852
ID3	-2.18	1.27E-32	-0.3	120	-21.12	15980	-0.25	118	-17.05	15600	-0.38	121	-27.21	16144
ABAT	-1.76	8.12E-12	-0.21	151	-21.13	15981	-0.23	129	-18.37	15785	-0.28	143	-25.87	16023
THBD	-2.81	2.80E-23	-0.24	138	-21.16	15982	-0.12	118	-8.28	13229	-0.34	150	-33.68	16410
ABCD4	-1.26	5.38E-08	-0.2	155	-21.18	15983	-0.21	129	-16.4	15495	-0.18	116	-11.99	13535
SLC28A1	-1.61	1.85E-12	-0.35	111	-21.19	15984	-0.25	134	-20.84	16074	-0.27	111	-16.34	14655
FHL1	-1.84	9.46E-21	-0.28	125	-21.2	15985	-0.19	128	-14.51	15105	-0.28	129	-22.5	15723
PZP	-3.44	9.97E-27	-0.29	123	-21.21	15986	-0.24	104	-12.9	14731	-0.34	104	-18.55	15092
ANKS4B	-1.27	1.09E-10	-0.29	124	-21.22	15987	-0.22	136	-18.98	15867	-0.23	116	-15.17	14414
PDE2A	-4.05	1.43E-38	-0.35	110	-21.22	15988	-0.29	127	-21.99	16165	-0.42	120	-29.38	16269
CYP2C9	-5.11	1.48E-24	-0.33	114	-21.24	15989	-0.34	129	-26.95	16411	-0.33	145	-31.75	16355
SLC19A3	-1.91	2.86E-16	-0.25	135	-21.24	15990	-0.17	143	-15.58	15318	-0.21	156	-21.96	15656
DNAJC25	-1.51	1.19E-11	-0.24	140	-21.24	15991	-0.23	131	-18.76	15831	-0.14	133	-11.39	13367
ACOT13	-1.46	1.63E-08	-0.22	145	-21.25	15992	-0.18	129	-13.99	14990	-0.18	130	-14.67	14292
INTS10	-1.84	1.81E-22	-0.29	123	-21.26	15993	-0.23	120	-16.17	15453	-0.35	106	-19.81	15296
ECHS1	-1.95	6.10E-15	-0.37	108	-21.26	15994	-0.26	106	-14.68	15144	-0.31	95	-14.14	14145
CLN8_t1	-1.43	5.29E-21	-0.22	148	-21.26	15995	NA	NA	NA	NA	-0.33	150	-33.13	16392
CNTN3_t1	-1.69	7.45E-19	-0.26	133	-21.26	15996	NA	NA	NA	NA	-0.25	133	-20.64	15432
PAPSS2	-1.67	2.15E-12	-0.26	133	-21.27	15997	-0.3	127	-23.22	16258	-0.13	124	-9.52	12763
DOK6_t1	-1.17	4.27E-05	-0.2	158	-21.27	15998	NA	NA	NA	NA	NA	NA	NA	NA
ID2_t1	-2.99	6.39E-30	-0.25	135	-21.28	15999	NA	NA	NA	NA	-0.33	142	-30.01	16288
ENG	-2.18	2.92E-32	-0.3	120	-21.28	16000	-0.25	114	-16.03	15426	-0.39	102	-20.1	15347
PRNP	-2.1	1.29E-13	-0.24	138	-21.3	16001	-0.11	135	-9.11	13564	-0.28	153	-28.52	16229
WNT2	-1.2	1.98E-14	-0.25	134	-21.32	16002	-0.17	116	-11.24	14311	-0.34	129	-26.65	16100
FAM102A	-2.34	9.25E-20	-0.25	137	-21.34	16003	-0.23	157	-24.33	16321	-0.3	149	-29.75	16280
BTG2	-2.15	1.00E-16	-0.23	143	-21.34	16004	-0.16	131	-13.06	14766	-0.34	116	-22.57	15727
ARPP21_t2	-1.13	0.007600646	-0.17	173	-21.35	16005	-0.22	129	-17.44	15664	-0.03	141	-2.99	10175
TGFBR3	-1.65	1.20E-17	-0.28	127	-21.36	16006	-0.21	134	-17.64	15689	-0.26	140	-23.12	15789
PON1	-1.31	1.51E-12	-0.18	172	-21.37	16007	-0.28	132	-22.63	16222	-0.12	120	-8.32	12368
PRKCQ	-1.19	6.70E-11	-0.22	148	-21.39	16008	-0.12	136	-10.01	13886	-0.3	120	-20.9	15485
TRAF6	-1.24	7.15E-15	-0.19	160	-21.4	16009	-0.16	137	-14.26	15044	-0.25	147	-24.04	15872
INMT	-1.51	1.69E-21	-0.25	136	-21.41	16010	-0.22	130	-17.84	15716	-0.39	123	-28.21	16202
CHIC2	-1.5	1.24E-25	-0.27	128	-21.42	16011	-0.16	105	-8.76	13420	-0.36	121	-25.5	15989
CD8A	-2.07	2.94E-13	-0.24	138	-21.43	16012	-0.09	121	-6.46	12397	-0.37	141	-33.52	16406
EDNRB	-2.04	7.31E-23	-0.31	120	-21.44	16013	-0.18	124	-13.54	14879	-0.32	141	-28.94	16257
SLC25A20	-1.75	2.07E-15	-0.31	120	-21.45	16014	-0.3	127	-23.23	16260	-0.27	119	-18.93	15160
MYH10_t1	-1.35	9.56E-11	-0.19	162	-21.47	16015	NA	NA	NA	NA	-0.16	128	-12.16	13595

NIT2	-1.72	8.69E-16	-0.31	119	-21.47	16016	-0.23	110	-13.76	14938	-0.29	106	-16.03	14598
CXCR2P1	-1.31	2.15E-13	-0.29	123	-21.48	16017	-0.26	146	-24.73	16332	-0.33	131	-26.43	16080
CMTM6	-1.62	5.23E-12	-0.29	124	-21.5	16018	-0.23	120	-15.86	15379	-0.25	127	-19.15	15198
SAMD5	-2.67	5.44E-19	-0.26	134	-21.52	16019	-0.12	142	-11.45	14358	-0.42	153	-42.79	16491
SLC17A3	-2.44	3.87E-17	-0.4	104	-21.52	16020	-0.21	93	-8.87	13457	-0.32	111	-19.37	15230
TBC1D2B	-1.4	5.32E-17	-0.32	117	-21.53	16021	-0.21	154	-21.48	16136	-0.34	138	-29.9	16284
F9	-5.07	1.61E-16	-0.32	118	-21.54	16022	-0.32	144	-30.07	16469	-0.25	135	-21.32	15559
UGT2B7	-3.64	5.98E-20	-0.32	117	-21.55	16023	-0.34	108	-19.58	15938	-0.3	90	-12.06	13562
AMT	-1.47	1.20E-07	-0.27	131	-21.55	16024	-0.28	140	-25.44	16363	-0.14	133	-11.74	13473
KDM2A	-1.18	5.78E-12	-0.23	142	-21.56	16025	-0.17	168	-19.89	15971	-0.29	157	-30.53	16316
CAPN6	-1.26	0.000116201	-0.28	128	-21.59	16026	-0.12	133	-9.77	13804	-0.29	142	-26.9	16121
HEBP1	-1.89	1.07E-23	-0.31	119	-21.6	16027	-0.23	97	-10.73	14145	-0.32	117	-21.66	15612
PSD4	-1.54	1.03E-17	-0.31	120	-21.6	16028	-0.35	136	-30.39	16471	-0.38	125	-28.51	16228
FGA_t2	-3.79	4.85E-13	-0.24	139	-21.61	16029	-0.25	119	-17.08	15604	-0.24	134	-20.4	15396
ABHD14B	-1.18	1.49E-13	-0.25	135	-21.61	16030	-0.32	124	-24.03	16305	-0.24	127	-18.14	15004
CBR4	-1.99	1.54E-21	-0.3	123	-21.61	16031	-0.27	136	-23.48	16275	-0.31	129	-24.57	15919
NR1H3	-1.33	5.63E-10	-0.35	111	-21.62	16032	-0.28	124	-20.57	16052	-0.32	127	-24.69	15932
CNTFR	-1.37	2.22E-13	-0.25	138	-21.63	16033	-0.26	111	-15.93	15401	-0.28	134	-23.38	15811
NKIRAS1	-1.34	7.43E-11	-0.23	144	-21.65	16034	-0.24	120	-16.97	15584	-0.11	141	-10.37	13041
HERPUD2	-1.08	0.000733181	-0.2	161	-21.65	16035	-0.11	125	-8.58	13354	-0.08	120	-5.26	11143
ZNF462	-2.06	4.22E-14	-0.22	147	-21.65	16036	-0.14	102	-7.51	12877	-0.29	102	-15.33	14446
TMEM178A	-1.09	0.009375421	-0.24	139	-21.66	16037	-0.12	139	-10.75	14161	-0.18	137	-16.03	14596
RIPK4	-1.81	6.45E-17	-0.23	144	-21.68	16038	-0.2	119	-13.52	14874	-0.27	120	-18.61	15100
FUT3	-1.13	7.32E-05	-0.23	146	-21.7	16039	0	136	0	8470	-0.24	100	-11.94	13524
CD300A_t1	-1.66	4.04E-26	-0.27	130	-21.71	16040	NA	NA	NA	NA	NA	NA	NA	NA
CLDN10	-3.08	1.79E-19	-0.33	115	-21.73	16041	-0.17	131	-13.76	14936	-0.39	123	-28.46	16220
C1R	-3.29	3.82E-22	-0.31	119	-21.73	16042	-0.37	123	-27.23	16421	-0.39	120	-27.08	16135
TIPARP	-1.67	3.24E-11	-0.24	141	-21.76	16043	-0.15	136	-12.88	14727	-0.27	126	-20.7	15444
LGSN	-2.01	1.98E-08	-0.21	155	-21.78	16044	-0.14	139	-12.88	14729	-0.11	132	-8.84	12539
PLSCR4	-2.83	1.88E-26	-0.29	126	-21.81	16045	-0.23	130	-18.14	15753	-0.36	146	-34.49	16426
PLAC8	-7.84	2.15E-40	-0.33	117	-21.82	16046	-0.22	116	-14.23	15037	-0.43	124	-32.12	16365
ZNF410	-1.16	1.07E-05	-0.22	150	-21.82	16047	-0.17	140	-15.09	15217	-0.21	110	-12.69	13759
ZBED1_t1	-1.52	8.08E-15	-0.29	125	-21.82	16048	NA	NA	NA	NA	-0.33	123	-24.04	15875
ARMC5	-1.33	1.50E-06	-0.28	127	-21.85	16049	-0.23	129	-18.21	15761	-0.18	135	-14.94	14366
OTC	-2.4	3.76E-10	-0.25	136	-21.86	16050	-0.26	113	-16.07	15433	-0.14	120	-9.71	12813
SLCO1B1	-3.63	3.50E-13	-0.3	122	-21.87	16051	-0.28	124	-20.43	16034	-0.29	140	-25.65	15997
OCIAD1	-1.19	0.000293152	-0.28	127	-21.89	16052	-0.15	111	-8.96	13485	-0.15	114	-9.63	12787
GUCY1A3	-2.2	4.65E-19	-0.23	145	-21.92	16053	-0.1	141	-8.9	13468	-0.32	158	-34.82	16430
ACADL	-2.47	4.74E-17	-0.32	118	-21.93	16054	-0.27	114	-17.15	15607	-0.27	125	-20.31	15381
SLC45A3	-1.96	9.35E-16	-0.33	116	-21.93	16055	-0.16	144	-14.89	15182	-0.32	120	-22.22	15692
NTF3	-3.16	4.01E-32	-0.32	118	-21.93	16056	-0.26	113	-16.67	15540	-0.37	127	-28.49	16224
APLNR	-1.15	2.92E-15	-0.26	133	-21.93	16057	-0.17	130	-13.38	14846	-0.31	134	-25.95	16028
PPP1R10	-1.36	6.40E-12	-0.25	137	-21.93	16058	-0.18	124	-13.08	14771	-0.3	120	-21.2	15537
GLUD1	-1.92	3.78E-12	-0.28	128	-21.94	16059	-0.12	128	-9.34	13635	-0.27	146	-26.2	16058
PNRC1	-1.76	2.91E-17	-0.22	148	-21.98	16060	-0.21	127	-16.09	15438	-0.31	133	-26.01	16032
C9	-8.72	2.21E-25	-0.29	126	-22	16061	-0.34	118	-22.85	16242	-0.27	113	-17.04	14806
PCCA	-1.78	1.51E-14	-0.31	122	-22	16062	-0.24	119	-16.73	15553	-0.26	134	-21.96	15655
IGFALS	-12.55	4.43E-41	-0.37	110	-22.02	16063	-0.35	91	-14.18	15024	-0.39	120	-27.22	16146
GATM	-2.1	7.08E-09	-0.29	126	-22.05	16064	-0.27	127	-20.43	16035	-0.2	103	-10.67	13144
CYP2E1	-3.99	3.07E-12	-0.27	131	-22.07	16065	-0.24	92	-9.94	13862	-0.28	115	-18.42	15068
THEMIS2	-2.1	7.56E-22	-0.22	151	-22.08	16066	-0.13	127	-10.12	13927	-0.4	107	-22.92	15768
SRD5A1	-2.19	9.79E-19	-0.28	129	-22.08	16067	-0.32	139	-28.24	16442	-0.31	127	-23.65	15846
AK3	-1.55	2.56E-11	-0.23	148	-22.1	16068	-0.21	127	-16.08	15435	-0.21	137	-17.96	14972
ASB13	-1.38	1.40E-07	-0.24	141	-22.1	16069	-0.25	124	-18.55	15803	-0.15	120	-10.26	13010
UGP2	-2.38	3.84E-15	-0.29	125	-22.11	16070	-0.26	121	-18.81	15844	-0.25	101	-12.55	13711
CHD2_t2	-1.18	1.01E-06	-0.21	154	-22.11	16071	-0.05	136	-4.57	11426	-0.17	153	-17.6	14906
PDHB	-1.17	1.43E-05	-0.25	138	-22.11	16072	-0.16	117	-10.95	14224	-0.25	125	-19.02	15178
PDLIM5_t2	-2.03	2.31E-18	-0.26	134	-22.12	16073	-0.25	129	-19.6	15943	-0.28	151	-28.72	16241
PGM1	-1.7	2.95E-15	-0.3	125	-22.13	16074	-0.34	117	-22.53	16213	-0.27	122	-19.69	15279
CHP1_t1	-1.26	2.79E-11	-0.26	136	-22.15	16075	NA	NA	NA	NA	NA	NA	NA	NA
KAZN_t1	-1.35	4.49E-19	-0.31	122	-22.15	16076	NA	NA	NA	NA	-0.31	151	-31.72	16353
DBH	-5.23	1.64E-36	-0.41	104	-22.16	16077	-0.34	151	-34.56	16487	-0.45	120	-31.66	16348
FCN3	-13.69	9.25E-58	-0.41	104	-22.18	16078	-0.32	108	-18.36	15784	-0.51	100	-25.34	15980
DLC1_t2	-2.03	1.95E-24	-0.25	138	-22.19	16079	-0.21	129	-16.86	15572	-0.29	140	-25.78	16011
FOSB	-4.95	2.94E-34	-0.26	135	-22.2	16080	-0.2	125	-15.02	15202	-0.36	126	-27.37	16154
C8A	-4.68	8.15E-23	-0.32	120	-22.22	16081	-0.35	124	-25.68	16368	-0.33	120	-23.28	15801
FAM65C	-2.45	6.15E-29	-0.32	120	-22.23	16082	-0.31	138	-27.29	16423	-0.41	129	-32.51	16379
JMJD8	-1.27	1.26E-14	-0.26	135	-22.23	16083	-0.17	132	-13.6	14896	-0.31	117	-20.75	15452
SDHAP1	-1.47	4.99E-18	-0.24	143	-22.25	16084	-0.31	126	-23.78	16293	-0.29	144	-27.69	16167
SCRG1	-1.63	4.99E-15	-0.29	128	-22.26	16085	-0.17	106	-9.64	13750	-0.31	120	-21.92	15646
NAT2	-5.62	2.58E-29	-0.33	117	-22.26	16086	-0.27	110	-16.26	15467	-0.35	111	-21.62	15607
PPP2R5C	-1.33	5.90E-08	-0.22	150	-22.29	16087	-0.12	146	-11.47	14365	-0.2	151	-19.95	15318
NTN4	-2.48	2.68E-23	-0.24	144	-22.29	16088	-0.16	147	-15.57	15317	-0.29	138	-25.81	16014
NRIP3	-1.54	9.47E-21	-0.22	152	-22.31	16089	-0.28	130	-22.41	16208	-0.2	128	-15.82	14541
KLF9	-2.19	9.95E-14	-0.24	142	-22.31	16090	-0.23	131	-18.98	15865	-0.23	139	-20.77	15456
CDKN1A_t2	-1.57	2.23E-12	-0.28	131	-22.32	16091	-0.22	126	-16.65	15536	-0.3	156	-31.36	16342
STOM	-1.86	5.79E-25	-0.24	145	-22.33	16092	-0.24	129	-18.92	15856	-0.3	145	-28.37	16213
EID1_t1	-1.26	8.52E-05	-0.24	142	-22.33	16093	-0.07	114	-4.77	11523	NA	NA	NA	NA
TBX15	-3.89	3.30E-16	-0.28	130	-22.34	16094	-0.25	127	-18.96	15863	-0.23	139	-20.32	15384
MYD88	-1.25	6.35E-14	-0.32	120	-22.37	16095	-0.29	123	-21.27	16119	-0.26	118	-18	14979
FAH	-1.67	8.49E-17	-0.37	110	-22.38	16096	-0.34	118	-23.44	16274	-0.26	120	-18.24	15030
PPM1K	-1.41	9.47E-12	-0.26	136	-22.38	16097	-0.18	139	-15.78	15358	-0.28	153	-28.38	16215
TSPAN7	-2.14	8.42E-24	-0.29	128	-22.4	16098	-0.25	145	-23.5	16277	-0.39	134	-32.81	16385
COLEC11	-6.96	5.09E-29	-0.34	115	-22.41	16099	-0.18	138	-15.96	15408	-0.46	116	-30.17	16295
AQP7	-2.16	5.72E-13	-0.32	119	-22.41	16100	-0.21	104	-11.44	14353	-0.2	76	-5.23	11128
RORA	-1.93	3.56E-14	-0.23	146	-22.43	16101	-0.22	151	-21.79	16153	-0.23	144	-21.57	15604
ACAT1	-2.52	6.72E-18	-0.31	122	-22.45	16102	-0.28	118	-18.79	15839	-0.3	139	-26.51	16090
NDST3	-1.48	1.16E-24	-0.3	126	-22.46	16103	-0.28	138	-24.83	16337	-0.32	144	-30.53	16315
DIS3L	-1.19	4.43E-05	-0.29	127	-22.48	16104	-0.15	120	-10.48	14058	-0.15	142	-13.83	14060
N4BP2L1	-2.92	2.23E-26	-0.29	127	-22.49	16105	-0.29	126	-22.1	16178	-0.36	138	-31.31	16340
ARAP3	-1.44	4.69E-12	-0.27	134	-22.49	16106	-0.05	119	-3.16	10576	-0.29	133	-24.24	15896
EPHA7	-1.23	1.52E-10	-0.23	147	-22.51	16107	-0.24	140	-21.26	16117	-0.19	138	-16.32	14650
EGR3	-1.9	2.36E-17	-0.26	135	-22.52	16108	-0.07	120	-5.07	11697	-0.33	155	-34.37	16424
GGT3P	-1.85	6.99E-18	-0.35	114	-22.53	16109	-0.06	121	-3.96	11079	-0.36	124	-26.36	16076
MAF	-1.75	5.71E-16	-0.29	127	-22.53	16110	-0.25	132	-20.42	16032	-0.32	147	-31.43	16343
B3GNT7	-1.36	1.51E-08	-0.22	151	-22.54	16111	-0.29	133	-24.47	16323	-0.21	109	-12.62	13741
GALNT14	-2.92	1.48E-29	-0.3	126	-22.55	16112	-0.24	104	-13.04	14761	-0.39	115	-25.61	15995
GP5	-1.18	1.46E-11	-0.24	143	-22.55	16113	-0.23	132	-19.02	15871	-0.18	134	-15.41	14458
MMP24	-1.04	0.000529697	-0.27	135	-22.58	16114	-0.13	128	-10.2	13955	-0.18	123	-13.41	13960
JDP2	-2.23	6.72E-27	-0.31	123	-22.61	16115	-0.22	121	-15.73	15348	-0.44	140	-39.43	16477
SUN2	-1.68	1.40E-21	-0.32	120	-22.61	16116	-0.32	120	-22.54	16214	-0.33	130	-26.12	16045
XKR4	-1.08	1.44E-05	-0.25	139	-22.62	16117	-0.16	141	-14.73	15151	-0.27	145	-25.68	16000
SLC23A2_t1	-1.74	1.27E-14	-0.22	154	-22.63	16118	NA	NA	NA	NA	-0.27	139	-24	15871
GSTZ1	-2.63	1.42E-18	-0.36	113	-22.66	16119	-0.35	117	-23.16	16257	-0.34	93	-14.51	14245
QDPR	-1.86	1.23E-11	-0.29	129	-22.67	16120	-0.34	108	-19.82	15964	-0.23	122	-16.24	14631
ECM1	-5.1	6.21E-49	-0.37	111	-22.69	16121	-0.28	130	-22.57	16218	-0.45	118	-30.28	16303

ADH1A_t1	-4.59	2.31E-22	-0.34	116	-22.7	16122	-0.33	100	-16.7	15549	NA	NA	NA	NA
ECHDC2	-1.85	1.27E-11	-0.23	147	-22.7	16123	-0.27	132	-22.01	16171	-0.21	109	-12.14	13589
DIXDC1	-1.39	1.88E-15	-0.23	149	-22.7	16124	-0.22	162	-24.21	16313	-0.24	134	-20.18	15365
EPOR_t1	-1.48	2.25E-14	-0.29	128	-22.71	16125	NA	NA	NA	NA	NA	NA	NA	NA
TFPI2	-4.65	3.53E-30	-0.28	132	-22.76	16126	-0.25	113	-15.9	15390	-0.36	129	-28.81	16248
PHLDB2_t1	-1.72	2.32E-10	-0.24	145	-22.77	16127	NA	NA	NA	NA	-0.19	128	-14.9	14353
ECE1	-1.37	1.24E-17	-0.31	124	-22.77	16128	-0.2	131	-15.91	15397	-0.34	136	-29.24	16265
OLFM1_t1	-1.81	4.99E-17	-0.33	119	-22.78	16129	-0.18	120	-12.33	14590	-0.4	146	-38.01	16468
GIMAP8_t1	-1.81	8.26E-20	-0.22	154	-22.79	16130	NA	NA	NA	NA	-0.35	120	-24.24	15895
TLR4_t1	-1.58	7.55E-21	-0.25	142	-22.81	16131	-0.15	156	-16.12	15447	NA	NA	NA	NA
ZCCHC24	-1.45	1.03E-17	-0.21	159	-22.83	16132	-0.24	138	-21.32	16127	-0.28	137	-24.04	15873
SLC1A1_t1	-2.59	5.81E-11	-0.22	152	-22.84	16133	NA	NA	NA	NA	-0.22	151	-22.09	15673
WDR72	-2.66	7.32E-18	-0.24	145	-22.85	16134	-0.14	146	-13.17	14789	-0.26	136	-22.45	15715
AMOTL2	-1.97	6.67E-16	-0.28	133	-22.87	16135	-0.2	133	-16.51	15512	-0.39	157	-41.48	16486
ANGPTL1_t1	-1.11	1.66E-12	-0.27	134	-22.89	16136	-0.17	120	-11.71	14426	NA	NA	NA	NA
MT1F	-6.4	9.56E-22	-0.36	114	-22.89	16137	-0.26	145	-24.83	16336	-0.32	93	-13.62	14009
ANK3_t1	-1.19	1.41E-11	-0.22	153	-22.9	16138	-0.12	133	-10.11	13921	-0.22	129	-17.36	14852
PITPNM3	-1.03	5.34E-05	-0.25	142	-22.91	16139	-0.21	137	-18.35	15781	-0.21	154	-21.84	15634
GDF2	-3.49	2.15E-40	-0.42	104	-22.91	16140	-0.33	118	-22.15	16183	-0.46	100	-23.23	15797
CTBS	-1.58	1.94E-16	-0.23	151	-22.92	16141	-0.19	152	-18.91	15853	-0.2	128	-15.88	14556
PBX1	-1.18	5.28E-05	-0.27	134	-22.92	16142	-0.16	134	-13.52	14875	-0.3	154	-30.86	16324
RTN4	-1.64	1.04E-08	-0.28	131	-22.92	16143	-0.19	111	-11.3	14323	-0.14	139	-12.7	13761
BACE1	-1.35	5.16E-08	-0.27	134	-22.96	16144	-0.16	139	-13.86	14958	-0.21	146	-20.02	15335
PROM1	-1.36	3.94E-11	-0.27	135	-22.96	16145	-0.12	122	-8.43	13298	-0.33	135	-28.26	16207
TMEM66	-1.58	4.36E-18	-0.24	147	-22.97	16146	-0.25	112	-15.76	15356	-0.33	128	-25.68	15999
CLIC6	-2.34	2.44E-15	-0.31	125	-22.97	16147	-0.12	117	-7.8	13034	-0.39	139	-34.36	16423
NAPSB	-2.42	1.28E-30	-0.31	125	-22.98	16148	-0.23	130	-18.44	15795	-0.47	123	-34.58	16427
FMO2	-1.64	1.06E-18	-0.27	134	-22.98	16149	-0.16	134	-13.66	14909	-0.32	147	-30.9	16327
OLFM1_t2	-1.82	1.00E-19	-0.3	126	-22.99	16150	-0.2	113	-12.9	14732	-0.39	136	-33.17	16395
TMEM71	-1.93	3.59E-22	-0.27	134	-23	16151	-0.17	122	-11.98	14495	-0.33	136	-28.81	16247
GAS1	-2.42	3.31E-24	-0.3	127	-23	16152	-0.2	128	-15.22	15254	-0.34	133	-28.18	16201
SRSF5	-1.71	4.37E-25	-0.33	120	-23.01	16153	-0.24	122	-17.23	15624	-0.4	131	-32.3	16374
ENPP1	-1.8	4.64E-11	-0.26	140	-23.02	16154	-0.22	140	-19.58	15940	-0.16	143	-14.88	14348
SLC38A4	-3.63	4.85E-15	-0.26	139	-23.02	16155	-0.34	135	-29.25	16453	-0.2	135	-16.89	14784
GABRB3	-1.13	0.001962136	-0.23	149	-23.03	16156	-0.14	130	-11.21	14303	-0.14	127	-10.6	13117
HIBADH	-1.48	5.91E-08	-0.29	129	-23.06	16157	-0.18	129	-13.95	14984	-0.16	120	-11.16	13297
APOL1_t1	-1.96	3.77E-22	-0.31	124	-23.07	16158	NA	NA	NA	NA	NA	NA	NA	NA
CCPG1	-1.34	5.74E-09	-0.19	171	-23.08	16159	-0.12	143	-11.26	14315	-0.05	130	-4.29	10748
KLF4	-2.25	3.40E-20	-0.28	133	-23.08	16160	-0.12	118	-8.18	13182	-0.35	120	-24.68	15930
MAN1C1	-1.73	5.94E-25	-0.29	131	-23.1	16161	-0.26	130	-20.69	16065	-0.36	130	-29	16258
ADH1C	-3.95	3.10E-18	-0.35	116	-23.1	16162	-0.3	98	-14.54	15111	-0.35	99	-17.04	14807
PECR	-1.71	1.59E-14	-0.29	131	-23.11	16163	-0.32	120	-22.27	16196	-0.27	112	-16.83	14765
SIGLEC11_t1	-1.86	4.38E-28	-0.34	119	-23.14	16164	NA	NA	NA	NA	-0.43	120	-30	16287
RNF122	-1.89	4.09E-21	-0.3	127	-23.14	16165	-0.1	140	-9.38	13649	-0.4	108	-23.38	15810
NXPE3	-1.38	1.70E-12	-0.18	180	-23.18	16166	-0.01	147	-0.78	8998	-0.27	153	-27.87	16179
ADRA2B	-1.78	2.86E-21	-0.33	121	-23.2	16167	-0.28	129	-22.18	16186	-0.38	118	-26.04	16034
NPY1R	-3.97	1.71E-33	-0.29	130	-23.2	16168	-0.2	122	-14.17	15019	-0.32	140	-28.91	16256
PER1	-2.83	2.75E-30	-0.33	120	-23.2	16169	-0.3	116	-19.65	15947	-0.4	120	-27.78	16175
FPR2	-1.62	1.76E-16	-0.28	132	-23.21	16170	-0.2	109	-12	14500	-0.38	134	-32.2	16368
TFAP2C	-1.32	4.16E-16	-0.33	120	-23.23	16171	-0.27	143	-25.02	16346	-0.35	127	-26.93	16124
UST	-1.21	0.00440971	-0.23	150	-23.23	16172	-0.2	156	-20.69	16063	-0.24	131	-19.07	15185
TRPV4	-1.72	3.48E-23	-0.32	123	-23.26	16173	-0.23	103	-12.27	14572	-0.4	125	-29.7	16276
CYP1A2_t1	-2.66	2.35E-28	-0.38	112	-23.27	16174	-0.32	135	-27.2	16419	NA	NA	NA	NA
ENOSF1	-1.32	1.21E-06	-0.23	152	-23.28	16175	-0.2	161	-22.31	16198	-0.17	151	-16.79	14750
PHACTR3	-1.54	4.37E-29	-0.37	113	-23.33	16176	-0.24	136	-20.64	16061	-0.39	123	-28.8	16246
RANBP10	-1.22	3.40E-05	-0.25	143	-23.34	16177	-0.25	127	-18.95	15861	-0.24	155	-25.37	15983
SLC18A1	-3.22	2.58E-22	-0.32	123	-23.36	16178	-0.29	123	-21.31	16126	-0.26	114	-16.68	14734
C2CD2L	-1.28	5.61E-13	-0.25	145	-23.37	16179	-0.04	109	-2.41	10092	-0.29	133	-24.15	15886
SPON1	-3.23	3.56E-16	-0.26	139	-23.37	16180	-0.12	122	-8.67	13385	-0.33	131	-26.41	16077
PCDH9_t2	-2.66	6.46E-26	-0.24	146	-23.37	16181	-0.19	143	-17.33	15647	-0.36	155	-38.31	16472
ACADS	-2.67	2.27E-28	-0.32	124	-23.37	16182	-0.3	120	-20.97	16082	-0.35	96	-16.33	14652
HMGCS2	-2.3	2.74E-18	-0.26	140	-23.39	16183	-0.36	124	-26.62	16404	-0.3	124	-21.86	15638
KDM8	-2.33	8.91E-26	-0.32	123	-23.4	16184	-0.35	145	-33.63	16484	-0.33	116	-21.53	15596
ALDOB	-2.85	9.15E-13	-0.28	135	-23.42	16185	-0.43	124	-32.14	16477	-0.26	119	-17.78	14938
AGXT2	-3.23	5.02E-16	-0.28	133	-23.44	16186	-0.3	123	-21.85	16156	-0.27	132	-22.33	15702
LILRA3	-1.62	3.74E-22	-0.33	121	-23.45	16187	-0.23	104	-12.43	14624	-0.47	95	-21.24	15543
CYP2C8	-7.88	5.10E-34	-0.37	113	-23.46	16188	-0.36	127	-27.6	16430	-0.37	129	-29.01	16259
APOF	-11.13	3.97E-29	-0.36	115	-23.54	16189	-0.36	112	-22.54	16215	-0.38	115	-24.85	15946
AQP3	-2.36	9.35E-25	-0.37	114	-23.55	16190	-0.27	125	-20.37	16026	-0.39	131	-31.25	16337
PEG3	-1.02	0.672502531	-0.22	156	-23.55	16191	0.02	130	1.24	7653	-0.12	140	-10.79	13180
CYP39A1	-5.25	1.31E-29	-0.33	122	-23.55	16192	-0.32	130	-25.85	16373	-0.33	130	-26.5	16086
GFOD1	-1.58	5.07E-10	-0.28	135	-23.56	16193	-0.2	140	-17.7	15696	-0.21	131	-17.11	14819
CPT2	-1.91	1.25E-19	-0.27	138	-23.56	16194	-0.26	139	-22.92	16246	-0.34	120	-24.14	15883
SERPINA5_t1	-2.09	1.25E-11	-0.3	128	-23.6	16195	NA	NA	NA	NA	NA	NA	NA	NA
LHX2	-3.48	2.36E-28	-0.32	124	-23.61	16196	-0.2	107	-11.46	14364	-0.42	100	-21.07	15516
PTS	-1.53	4.15E-14	-0.25	145	-23.68	16197	-0.19	104	-10.47	14052	-0.23	146	-22.11	15678
C7	-5.99	3.47E-36	-0.31	126	-23.69	16198	-0.21	148	-21.04	16093	-0.39	152	-40.25	16479
AZGP1	-2.26	1.80E-14	-0.32	123	-23.7	16199	-0.35	104	-18.95	15862	-0.26	95	-11.49	13399
AKAP2	-2.55	6.13E-23	-0.28	136	-23.71	16200	-0.27	123	-19.49	15929	-0.28	147	-26.8	16112
FOXO1	-2.04	1.52E-24	-0.25	143	-23.71	16201	-0.2	134	-16.83	15570	-0.31	134	-26.35	16075
CPED1_t1	-2.34	2.28E-23	-0.25	146	-23.72	16202	NA	NA	NA	NA	NA	NA	NA	NA
THRSP_t1	-7.93	4.53E-17	-0.3	130	-23.74	16203	NA	NA	NA	NA	-0.29	121	-20.51	15416
MRPL32	-1.36	9.11E-13	-0.34	120	-23.75	16204	-0.18	123	-13.32	14832	-0.25	127	-19.34	15226
ASPA	-2.28	1.24E-23	-0.27	137	-23.77	16205	-0.26	118	-18	15734	-0.28	113	-17.93	14968
PARVA_t1	-1.18	1.57E-05	-0.24	148	-23.81	16206	NA	NA	NA	NA	NA	NA	NA	NA
SPATA13	-1.15	4.84E-05	-0.27	139	-23.82	16207	-0.11	139	-10.16	13934	-0.26	124	-19.29	15219
IL7R	-3.08	1.54E-18	-0.28	134	-23.82	16208	-0.13	145	-12.06	14524	-0.37	122	-26.76	16109
MT1M	-7.63	3.56E-22	-0.34	120	-23.83	16209	-0.28	112	-17.36	15651	-0.35	104	-18.94	15161
PCDH8	-1.18	2.45E-13	-0.29	132	-23.83	16210	-0.27	120	-18.62	15813	-0.17	118	-11.83	13495
PLIN5	-1.29	1.27E-09	-0.26	140	-23.84	16211	-0.28	125	-20.74	16068	-0.29	147	-27.92	16182
OIT3	-32.36	2.44E-52	-0.38	112	-23.85	16212	-0.31	129	-24.61	16327	-0.44	104	-23.55	15836
MUT	-2.19	1.06E-13	-0.31	127	-23.85	16213	-0.24	130	-19.16	15886	-0.22	128	-17.29	14847
GNG11	-1.73	4.36E-14	-0.24	150	-23.88	16214	-0.17	138	-15.37	15284	-0.24	102	-12.31	13649
GNAO1_t2	-1.34	4.34E-15	-0.28	135	-23.89	16216	-0.28	122	-19.82	15963	-0.23	129	-17.79	14940
SFRP5	-1.58	3.00E-20	-0.35	118	-23.89	16217	-0.17	124	-12.45	14627	-0.42	118	-28.51	16226
IFT46	-1.5	1.37E-17	-0.26	141	-23.9	16218	-0.29	124	-21.2	16113	-0.38	98	-18.38	15059
ST3GAL6	-2.15	1.79E-19	-0.28	136	-23.9	16219	-0.2	120	-13.91	14975	-0.3	144	-27.95	16189
SLC16A2	-1.84	4.43E-10	-0.29	133	-23.91	16220	-0.31	126	-23.27	16263	-0.16	127	-12.62	13739
MT2A_t1	-4.08	2.05E-17	-0.34	120	-23.92	16221	-0.25	128	-19.58	15939	NA	NA	NA	NA
ADH6	-3.13	2.59E-19	-0.33	122	-23.93	16222	-0.3	101	-15.42	15295	-0.32	104	-17.36	14855
RAMP3	-2.09	3.30E-29	-0.3	130	-23.93	16223	-0.28	131	-23	16251	-0.36	109	-21.1	15519
PCSK6_t2	-1.46	5.80E-08	-0.25	144	-23.94	16224	-0.32	109	-18.9	15851	-0.16	109	-9.49	12757
IGFBP4	-2.53	2.99E-25	-0.34	120	-23.96	16225	-0.34	146	-32.56	16479	-0.35	124	-25.95	16029
PRKCB_t1	-2.47	7.47E-21	-0.24	150	-23.97	16226	NA	NA	NA	NA	-0.36	132	-29.81	16281
SNX1	-1.26	4.36E-09	-0.28	136	-23.98	16227	-0.25	132	-20.48	16043	-0.21	143	-19.7	15280
TMEM56	-1.79	5.75E-13	-0.24	150	-24.01	16228	-0.2	134	-17.14	15606	-0.2	146	-19.25	15212

VTCN1	-1.72	1.96E-10	-0.3	131	-24.02	16229	-0.09	148	-8.92	13474	-0.37	127	-28.73	16242
KCNK17	-2.21	1.05E-28	-0.38	114	-24.02	16230	-0.24	130	-19.34	15908	-0.42	143	-38.7	16473
SMAD6	-1.55	3.48E-23	-0.34	121	-24.03	16231	-0.2	112	-12.57	14662	-0.38	104	-20.68	15441
CYP2C18	-2.38	3.85E-16	-0.32	124	-24.04	16232	-0.33	129	-26.01	16378	-0.23	121	-16.5	14682
VSX1	-1.82	8.35E-19	-0.31	128	-24.05	16233	-0.3	131	-24.13	16308	-0.34	124	-24.88	15947
MYOM2	-3.65	8.00E-31	-0.33	123	-24.1	16234	-0.27	105	-15.07	15215	-0.42	147	-40.79	16484
ACSM3	-2.8	1.67E-23	-0.35	119	-24.11	16235	-0.23	96	-10.53	14077	-0.33	102	-17.2	14834
BAI3	-1.48	1.85E-23	-0.36	117	-24.15	16236	-0.21	129	-16.6	15529	-0.41	146	-39.52	16478
C1RL	-2.46	1.28E-24	-0.34	122	-24.19	16237	-0.32	135	-27.57	16428	-0.34	144	-31.98	16362
GNA14	-1.68	1.37E-27	-0.28	136	-24.27	16238	-0.23	128	-17.55	15677	-0.34	116	-22.47	15717
FREM1	-1.39	7.13E-10	-0.31	128	-24.31	16239	-0.11	132	-9.13	13570	-0.31	139	-27.93	16187
MT1H	-3.88	6.72E-18	-0.35	120	-24.31	16240	-0.24	144	-22.96	16248	-0.31	94	-13.69	14023
TMSB4Y	-2.97	1.99E-22	-0.3	130	-24.32	16241	-0.3	129	-23.58	16281	-0.26	126	-19.61	15265
DSE	-2.27	3.81E-22	-0.24	152	-24.34	16242	-0.08	124	-5.77	12074	-0.36	112	-22.16	15681
LEPREL1	-3.6	1.08E-35	-0.35	119	-24.34	16243	-0.17	124	-12.83	14716	-0.41	135	-34.92	16431
REPS1	-1.77	1.88E-21	-0.32	125	-24.35	16244	-0.2	121	-14.45	15093	-0.36	123	-26	16031
RPP25L	-1.26	1.25E-07	-0.32	126	-24.36	16245	-0.27	112	-16.79	15563	-0.18	117	-12	13540
KIAA2013	-1.25	3.21E-10	-0.24	153	-24.37	16246	-0.19	120	-13.59	14890	-0.24	133	-19.72	15287
RGN	-2.5	1.12E-14	-0.34	122	-24.38	16247	-0.31	109	-18.07	15745	-0.34	118	-23.23	15798
ERRFI1_t1	-2.42	1.37E-25	-0.29	135	-24.39	16248	NA	NA	NA	NA	-0.34	132	-28.06	16192
HOGA1	-2.22	2.07E-20	-0.35	120	-24.39	16249	-0.2	129	-15.98	15410	-0.33	148	-32.75	16384
UNC93A_t1	-1.74	1.03E-05	-0.32	127	-24.41	16250	NA	NA	NA	NA	NA	NA	NA	NA
FOLH1_t1	-2.15	3.48E-22	-0.29	134	-24.42	16251	-0.32	100	-16.17	15454	-0.25	99	-12.23	13618
CHRNA10	-1.17	9.53E-08	-0.29	133	-24.43	16252	-0.11	146	-11.01	14240	-0.29	108	-16.58	14708
FAM134B_t1	-3.57	4.44E-20	-0.33	125	-24.45	16253	NA	NA	NA	NA	-0.27	127	-20.57	15423
ACSL1	-3.1	3.70E-20	-0.26	145	-24.46	16254	-0.32	123	-23.29	16264	-0.3	140	-27.02	16131
MYO9A	-1.58	5.25E-15	-0.28	136	-24.46	16255	-0.11	140	-9.56	13717	-0.3	120	-21.1	15520
TNFAIP8L1	-1.29	5.12E-07	-0.25	147	-24.53	16256	-0.26	131	-21.18	16109	-0.24	137	-20.7	15445
FAM19A5	-1.52	4.36E-06	-0.3	131	-24.53	16257	-0.02	135	-2.1	9903	-0.24	112	-14.84	14332
CYP4A11_t1	-4.06	1.26E-31	-0.36	119	-24.57	16258	NA	NA	NA	NA	NA	NA	NA	NA
MT1B	-5.38	1.83E-19	-0.35	120	-24.58	16259	-0.27	124	-20.11	16003	-0.32	89	-12.53	13709
BCL11B	-1.49	1.10E-18	-0.26	143	-24.59	16260	-0.15	137	-12.87	14724	-0.39	136	-33.29	16400
AHNAK_t2	-2.2	7.82E-17	-0.33	125	-24.61	16261	-0.25	111	-15.13	15232	-0.26	123	-18.74	15128
FXYD6	-2.14	3.69E-31	-0.32	128	-24.65	16262	-0.26	120	-18.1	15750	-0.39	110	-23.63	15844
DCN	-15.12	3.20E-35	-0.36	118	-24.71	16263	-0.2	131	-16.6	15530	-0.44	120	-30.89	16325
PTK2B	-1.22	2.46E-15	-0.31	131	-24.72	16264	-0.23	124	-16.9	15576	-0.36	143	-33.67	16409
SFRP1_t1	-4.33	8.11E-30	-0.33	126	-24.76	16265	NA	NA	NA	NA	-0.35	124	-25.78	16010
EGR1	-6.98	5.33E-31	-0.3	133	-24.76	16266	-0.2	123	-14.26	15045	-0.42	151	-42.36	16490
TJP2_t2	-1.72	7.48E-18	-0.25	149	-24.79	16267	-0.22	120	-15.1	15222	-0.27	123	-19.95	15319
MGMT	-1.8	1.04E-18	-0.35	120	-24.81	16268	-0.32	131	-25.77	16370	-0.37	113	-23.42	15819
IER2	-2.4	1.83E-24	-0.3	134	-24.82	16269	-0.21	128	-16.48	15506	-0.4	134	-33.88	16413
ZXDC_t2	-1.16	4.29E-07	-0.28	139	-24.82	16270	-0.21	137	-18.53	15802	-0.24	160	-26.52	16092
PPTC7	-1.33	6.99E-08	-0.25	151	-24.83	16271	-0.18	126	-13.73	14930	-0.16	138	-14.49	14240
CD1D_t1	-1.62	2.33E-23	-0.31	131	-24.89	16272	NA	NA	NA	NA	-0.43	134	-36.15	16450
CXCL12_t1	-10.44	2.22E-39	-0.33	126	-24.9	16273	-0.19	128	-15.06	15212	-0.45	144	-42.25	16489
CIDEB	-2.18	4.66E-24	-0.36	120	-24.94	16274	-0.37	118	-24.95	16341	-0.37	109	-21.91	15643
PLG	-2.19	2.55E-09	-0.27	144	-24.94	16275	-0.33	126	-24.89	16339	-0.3	106	-16.57	14706
ANKRD55	-1.9	2.31E-26	-0.34	123	-24.98	16276	-0.27	124	-20.2	16014	-0.36	134	-30.48	16312
GBA3	-7.15	1.86E-22	-0.35	121	-25	16277	-0.37	105	-20.56	16051	-0.27	124	-19.98	15326
CAND2	-2.22	1.08E-18	-0.27	141	-25.01	16278	-0.15	115	-10	13881	-0.34	115	-22.22	15694
GPD1	-2.35	9.30E-15	-0.37	118	-25.03	16279	-0.28	134	-23.43	16273	-0.28	131	-22.5	15721
TACSTD2	-4.01	6.32E-18	-0.29	135	-25.06	16280	-0.14	127	-10.5	14064	-0.39	122	-27.92	16185
DDB2	-1.77	1.99E-17	-0.29	136	-25.15	16281	-0.28	114	-17.77	15703	-0.29	121	-20.81	15470
TSPYL5	-2.18	1.01E-21	-0.26	146	-25.17	16282	-0.14	127	-10.58	14100	-0.38	143	-35.41	16437
CFLAR	-1.41	8.73E-24	-0.23	159	-25.18	16283	-0.24	139	-21.59	16140	-0.4	143	-37.2	16465
CLEC10A_t2	-2.27	1.10E-21	-0.31	130	-25.19	16284	-0.13	127	-9.72	13782	-0.42	126	-31.87	16358
PDE7B	-1.26	1.17E-16	-0.27	144	-25.23	16285	-0.2	135	-16.91	15578	-0.23	126	-17.79	14942
PKP2	-2.15	2.24E-21	-0.32	130	-25.24	16286	-0.31	122	-22.6	16219	-0.26	131	-21.32	15557
IL18RAP	-2.38	4.29E-25	-0.33	127	-25.24	16287	-0.21	108	-12.27	14573	-0.38	122	-27.61	16164
FYN	-1.91	1.34E-22	-0.34	124	-25.25	16288	-0.18	130	-14.77	15164	-0.43	157	-46.32	16495
SERPINE1	-2.58	5.00E-21	-0.27	144	-25.26	16289	-0.13	135	-10.98	14229	-0.31	171	-36.94	16457
GPR126_t2	-1.7	2.92E-18	-0.32	130	-25.31	16290	-0.22	122	-15.65	15335	-0.32	137	-27.69	16168
SELPLG	-1.42	7.71E-15	-0.27	145	-25.32	16291	-0.13	121	-9.49	13701	-0.37	120	-26.04	16035
RETSAT	-1.78	1.98E-20	-0.33	127	-25.32	16292	-0.28	117	-18.56	15806	-0.34	125	-25.49	15988
SLC22A10	-3.04	1.25E-25	-0.33	126	-25.32	16293	-0.31	108	-17.98	15733	-0.3	136	-25.6	15994
A2M	-3.09	2.13E-17	-0.32	130	-25.34	16294	-0.28	108	-15.96	15407	-0.31	89	-12.06	13564
PTPLA	-1.1	0.025318123	-0.3	134	-25.34	16295	0.01	99	0.37	8223	-0.17	95	-7.83	12175
BTG1	-1.53	4.41E-14	-0.25	150	-25.35	16296	-0.1	130	-7.68	12967	-0.32	142	-29.72	16279
HMGCL	-2.16	1.00E-23	-0.35	123	-25.44	16297	-0.31	112	-19.43	15921	-0.4	116	-26.25	16063
MYCT1	-1.55	1.56E-27	-0.31	132	-25.47	16298	-0.27	132	-22.36	16204	-0.38	131	-30.79	16323
DUSP5	-2.47	9.34E-22	-0.26	148	-25.49	16299	-0.14	120	-9.88	13836	-0.39	127	-30.2	16298
ATOH8	-2.56	6.40E-28	-0.31	133	-25.49	16300	-0.25	136	-21.27	16122	-0.33	131	-26.81	16114
LYVE1	-5.98	4.73E-33	-0.33	127	-25.49	16301	-0.24	132	-19.72	15955	-0.36	139	-32.12	16366
AGTR1	-2.84	7.18E-21	-0.33	127	-25.5	16302	-0.21	124	-15.44	15299	-0.35	127	-26.96	16128
OAZ2	-1.43	2.41E-18	-0.39	116	-25.5	16303	-0.26	133	-21.62	16143	-0.31	124	-22.67	15735
KLF10	-1.98	7.34E-17	-0.24	155	-25.52	16304	-0.17	135	-14.14	15014	-0.23	135	-19.43	15236
ZNF609	-1.08	9.40E-05	-0.28	142	-25.52	16305	-0.07	129	-5.77	12072	-0.14	150	-13.83	14061
ANK3_t2	-4.68	3.83E-21	-0.25	153	-25.53	16306	-0.09	133	-7.22	12746	-0.31	129	-24.74	15940
HOMER2	-1.55	2.21E-08	-0.3	135	-25.55	16307	-0.3	124	-22.55	16216	-0.12	122	-8.39	12395
KIAA0430	-1.35	2.17E-10	-0.26	148	-25.57	16308	-0.11	130	-8.7	13396	-0.31	158	-33.42	16402
CRADD	-1.38	1.98E-08	-0.26	150	-25.6	16309	-0.29	110	-17.39	15659	-0.15	143	-14.06	14125
KMO_t1	-3.47	8.32E-24	-0.28	142	-25.61	16310	-0.31	135	-26.69	16407	NA	NA	NA	NA
TIMD4	-8.55	5.07E-36	-0.36	121	-25.64	16311	-0.27	142	-24.72	16331	-0.43	92	-18.07	14989
AKR1D1_t1	-4.72	4.49E-22	-0.31	134	-25.65	16312	-0.31	130	-24.83	16335	NA	NA	NA	NA
PCK1_t1	-6.9	1.97E-25	-0.28	142	-25.72	16313	-0.35	119	-24.24	16315	NA	NA	NA	NA
PLCXD3	-2.85	5.32E-21	-0.27	147	-25.72	16314	-0.16	149	-15.78	15360	-0.35	134	-29.67	16275
FBXO8	-1.75	1.85E-18	-0.29	140	-25.74	16315	-0.21	119	-14.59	15119	-0.28	128	-21.92	15644
ARMC6	-1.4	5.77E-13	-0.35	125	-25.97	16316	-0.23	115	-14.9	15183	-0.28	92	-11.69	13450
CNDP1	-12.47	9.71E-32	-0.37	120	-25.97	16317	-0.31	120	-21.86	16157	-0.43	120	-30.09	16291
OAF_t1	-1.67	9.27E-13	-0.27	145	-25.98	16318	NA	NA	NA	NA	NA	NA	NA	NA
MBL2	-4.86	3.67E-20	-0.32	132	-25.98	16319	-0.19	131	-15.08	15216	-0.27	154	-27.71	16169
BDH1	-1.84	6.04E-14	-0.27	145	-25.99	16320	-0.32	128	-25.24	16354	-0.3	167	-34.94	16433
CD1C	-1.66	7.73E-19	-0.24	159	-26.01	16321	-0.11	129	-8.45	13303	-0.35	143	-32.96	16390
GRASP	-3.14	4.34E-28	-0.28	143	-26.02	16322	-0.18	116	-11.71	14430	-0.4	103	-21.46	15580
FGB	-3.18	3.86E-11	-0.27	146	-26.06	16323	-0.29	140	-26.15	16385	-0.28	151	-27.9	16180
BHMT2	-1.69	6.22E-06	-0.24	157	-26.07	16324	-0.19	125	-14.46	15096	-0.1	150	-10.23	13000
ANKS1A	-1.61	1.45E-22	-0.25	154	-26.11	16325	-0.18	142	-16.22	15462	-0.35	149	-34.92	16432
PBLD_t2	-3.19	1.21E-20	-0.29	139	-26.13	16326	-0.29	139	-25.57	16366	-0.27	147	-25.88	16024
HSD17B6	-3.03	1.76E-12	-0.27	147	-26.13	16327	-0.39	112	-23.95	16301	-0.28	107	-15.8	14537
CD81	-2.41	4.66E-28	-0.41	114	-26.14	16328	-0.28	124	-20.76	16070	-0.46	108	-26.52	16091
DMGDH	-3.21	2.22E-16	-0.37	121	-26.15	16329	-0.28	103	-14.75	15158	-0.23	114	-14.66	14289
ANAPC16	-1.2	3.06E-08	-0.32	132	-26.23	16330	-0.2	131	-15.99	15414	-0.21	152	-21.83	15632
ALDH9A1	-1.59	1.20E-14	-0.27	146	-26.27	16331	-0.25	128	-19.31	15904	-0.27	118	-18.57	15093
UROC1	-2.9	5.54E-22	-0.39	118	-26.29	16332	-0.34	138	-29.77	16462	-0.34	120	-24	15869
CDHR2	-3.79	2.09E-38	-0.43	111	-26.3	16333	-0.29	104	-15.43	15298	-0.46	100	-23.07	15784
AGXT2L2	-1.54	1.86E-21	-0.37	122	-26.31	16334	-0.24	112	-15.11	15226	-0.39	106	-22.08	15670

NADKD1	-2.03	9.23E-12	-0.3	139	-26.32	16335	-0.16	141	-14.21	15033	-0.23	128	-17.99	14977
ITPRIP	-1.84	1.02E-25	-0.26	152	-26.32	16336	-0.18	135	-15.05	15210	-0.36	139	-31.9	16359
GHR_t1	-6.13	6.05E-27	-0.37	122	-26.35	16337	NA	NA	NA	NA	-0.32	138	-27.72	16170
IYD	-2.89	3.71E-20	-0.3	139	-26.36	16338	-0.29	138	-25.92	16375	-0.24	141	-21.39	15566
CD163	-4.58	6.10E-31	-0.3	138	-26.37	16339	-0.21	97	-9.88	13838	-0.39	141	-35.56	16441
PCSK6_t3	-1.89	9.42E-14	-0.33	130	-26.44	16340	-0.41	125	-30.81	16472	-0.26	117	-17.25	14841
PLGLB1_t1	-4.43	1.27E-24	-0.35	125	-26.45	16341	NA	NA	NA	NA	-0.31	131	-25.21	15971
MASP1_t3	-3.24	9.69E-42	-0.4	116	-26.47	16342	-0.33	147	-31.83	16475	-0.45	141	-40.79	16483
MAP2K1	-2.21	1.40E-27	-0.37	122	-26.47	16343	-0.29	106	-16.1	15440	-0.32	163	-35.84	16448
AADAT	-3.4	9.95E-27	-0.34	128	-26.5	16344	-0.34	125	-25.35	16357	-0.3	120	-21.01	15504
VIPR1	-3.68	2.29E-37	-0.35	126	-26.56	16345	-0.29	143	-27.15	16415	-0.42	126	-31.68	16350
ST6GAL2	-1.12	3.55E-14	-0.27	147	-26.57	16346	-0.13	146	-12.38	14609	-0.33	137	-28.38	16214
TPRG1L	-1.37	2.94E-10	-0.29	143	-26.57	16347	-0.21	155	-22.35	16202	-0.24	146	-22.78	15751
BDH2	-1.81	3.97E-19	-0.24	160	-26.64	16348	-0.25	131	-20.57	16053	-0.22	124	-15.97	14582
PARP16	-1.21	1.45E-05	-0.33	131	-26.65	16349	-0.2	133	-16.83	15569	-0.13	121	-9.11	12630
MYZAP	-1.13	3.87E-07	-0.31	136	-26.65	16350	-0.11	131	-8.73	13410	-0.22	108	-12.84	13802
CLYBL	-2.05	5.95E-20	-0.33	132	-26.66	16351	-0.24	129	-18.76	15832	-0.38	127	-29.49	16271
F11	-2.93	7.43E-16	-0.31	136	-26.66	16352	-0.37	129	-29.5	16457	-0.31	129	-24.22	15893
LINC00261	-1.87	1.28E-09	-0.34	128	-26.67	16353	-0.19	144	-17.4	15660	-0.22	145	-20.86	15480
IGF2_t1	-5.45	2.53E-17	-0.33	130	-26.7	16354	NA	NA	NA	NA	NA	NA	NA	NA
BSDC1	-1.39	8.46E-17	-0.31	137	-26.7	16355	-0.19	122	-14.03	15000	-0.26	119	-18.16	15013
FANCC	-1.95	2.15E-14	-0.34	128	-26.74	16356	-0.21	133	-17.29	15640	-0.33	129	-25.74	16005
SATB1	-2.08	3.38E-20	-0.28	146	-26.75	16357	-0.21	140	-18.55	15804	-0.36	135	-30.9	16326
SRD5A2	-8.09	3.89E-27	-0.37	122	-26.75	16358	-0.36	125	-27.15	16416	-0.34	133	-28.23	16204
TMEM45A	-3.41	1.01E-18	-0.32	133	-26.75	16359	-0.15	108	-8.47	13313	-0.31	135	-26.51	16089
HPX	-3.88	2.42E-24	-0.38	120	-26.76	16360	-0.43	89	-16.67	15541	-0.39	94	-17.24	14837
PTPRB	-2.31	5.20E-28	-0.26	151	-26.76	16361	-0.21	150	-20.58	16054	-0.34	143	-31.64	16346
FBP1	-4.04	1.85E-21	-0.36	124	-26.8	16362	-0.37	130	-29.63	16459	-0.32	111	-19.49	15246
MRO	-1.94	4.68E-22	-0.27	150	-26.82	16363	-0.24	134	-19.84	15965	-0.39	124	-28.88	16253
BHMT	-3.54	1.15E-14	-0.34	128	-26.83	16364	-0.28	121	-20.09	16001	-0.19	128	-14.48	14235
CFTR	-4.5	4.43E-24	-0.34	128	-26.83	16365	-0.18	126	-13.67	14910	-0.39	126	-29.94	16286
GNE	-2.41	6.41E-23	-0.31	136	-26.84	16366	-0.32	136	-27.15	16417	-0.32	135	-27.34	16153
TUBE1	-2.24	1.93E-26	-0.37	123	-26.84	16367	-0.27	121	-19.45	15923	-0.31	123	-22.69	15741
ACADSB	-1.89	2.73E-12	-0.28	147	-26.93	16368	-0.19	132	-15.21	15251	-0.28	146	-26.87	16117
CYFIP2	-1.55	6.34E-10	-0.34	129	-26.93	16369	-0.2	147	-18.95	15860	-0.31	128	-23.95	15867
MMRN1	-1.59	2.61E-16	-0.3	139	-27	16370	-0.24	127	-18.26	15765	-0.31	138	-27.24	16150
CSAD	-2.2	4.94E-24	-0.31	138	-27.05	16371	-0.29	127	-22.67	16225	-0.31	128	-24.49	15912
SLC38A2_t1	-2.47	3.29E-23	-0.25	157	-27.07	16372	NA	NA	NA	NA	-0.35	146	-33.46	16403
CACNG2	-1.6	7.82E-19	-0.27	150	-27.22	16373	-0.31	134	-26.34	16395	-0.24	148	-23.06	15782
MIP	-1.16	4.34E-18	-0.32	135	-27.29	16374	-0.29	142	-26.41	16398	-0.29	144	-27.12	16138
TMEM220	-1.75	1.03E-13	-0.27	151	-27.31	16375	-0.28	128	-21.6	16141	-0.3	141	-27.19	16141
ATP6V1B2	-2	1.45E-26	-0.28	146	-27.33	16376	-0.28	127	-21.63	16144	-0.27	151	-27.11	16137
SOCS2	-2.68	2.99E-25	-0.33	134	-27.37	16377	-0.28	123	-20.08	15998	-0.31	152	-31.94	16361
CLEC4G	-14.17	1.82E-59	-0.35	129	-27.39	16378	-0.32	105	-17.35	15650	-0.42	110	-25.33	15978
LIFR	-1.51	1.94E-27	-0.28	148	-27.51	16379	-0.17	140	-14.91	15186	-0.3	153	-30.46	16311
PCDH18	-1.28	4.42E-10	-0.27	151	-27.54	16380	-0.06	126	-4.2	11207	-0.29	132	-23.51	15832
KCNN3	-1.84	5.82E-18	-0.29	144	-27.55	16381	-0.29	148	-28.59	16450	-0.25	156	-26.61	16096
GRHPR_t1	-2.05	6.13E-15	-0.36	127	-27.59	16382	NA	NA	NA	NA	-0.3	112	-18.79	15136
FGD4	-1.75	1.89E-12	-0.28	148	-27.64	16383	-0.16	135	-13.72	14924	-0.28	131	-22.63	15729
FOLH1_t2	-4.22	9.06E-27	-0.33	134	-27.64	16384	-0.34	100	-17.23	15628	-0.29	99	-14.15	14148
FAM150B	-2.23	5.87E-21	-0.31	140	-27.69	16385	-0.2	128	-15.55	15313	-0.41	120	-28.53	16230
DACH1	-2.28	7.39E-26	-0.28	150	-27.8	16386	-0.22	129	-17.28	15635	-0.34	130	-26.9	16123
KBTBD11	-5.33	7.63E-29	-0.31	140	-27.82	16387	-0.27	156	-28.25	16443	-0.33	135	-27.98	16191
RCAN1	-3.89	8.75E-30	-0.32	138	-27.85	16388	-0.3	128	-23.23	16259	-0.34	125	-25.63	15996
MLF2	-1.4	1.90E-19	-0.39	122	-27.93	16389	-0.26	128	-20.3	16018	-0.35	108	-20.57	15422
FOLH1B	-4.15	1.55E-26	-0.33	134	-27.94	16390	-0.34	100	-17.21	15620	-0.28	99	-13.49	13975
ZNF330	-1.6	8.90E-22	-0.32	138	-27.95	16391	-0.3	125	-22.74	16233	-0.27	123	-20.01	15334
CXCL14	-17.71	3.08E-53	-0.37	125	-27.98	16392	-0.29	126	-21.95	16161	-0.43	135	-36.51	16454
GPM6A	-4.49	4.50E-35	-0.34	133	-27.99	16393	-0.26	126	-19.55	15935	-0.38	163	-42.99	16492
CCBE1	-1.55	1.18E-26	-0.33	136	-28.07	16394	-0.24	151	-24.02	16304	-0.37	131	-29.72	16278
PNP	-2.24	1.22E-22	-0.29	146	-28.08	16395	-0.23	143	-21.28	16123	-0.35	140	-31.28	16338
CAT	-2.45	3.28E-20	-0.36	129	-28.14	16396	-0.36	109	-21.27	16118	-0.31	110	-18.46	15074
MAT1A	-3.29	2.22E-18	-0.35	130	-28.14	16397	-0.34	133	-28.52	16447	-0.38	114	-24.4	15907
SEMA6D_t2	-1.48	8.57E-12	-0.3	144	-28.24	16398	-0.11	134	-9.02	13514	-0.31	142	-28.21	16203
A1CF	-1.7	1.92E-11	-0.27	154	-28.26	16399	-0.25	152	-25.5	16364	-0.2	142	-18.81	15139
STAB2	-5.01	2.69E-55	-0.36	128	-28.27	16400	-0.32	116	-21.21	16114	-0.42	148	-40.75	16482
CXCL12_t2	-7.98	6.34E-38	-0.32	138	-28.3	16401	-0.19	142	-17.24	15629	-0.43	132	-35.62	16443
SUCLG2	-1.95	1.17E-27	-0.36	129	-28.34	16402	-0.38	129	-29.64	16461	-0.34	125	-25.2	15970
PTGS2	-1.91	5.92E-13	-0.3	146	-28.34	16403	-0.13	128	-10.2	13954	-0.38	148	-37.01	16461
ALDH1B1_t1	-2.3	4.95E-19	-0.38	125	-28.35	16404	NA	NA	NA	NA	-0.29	127	-22.23	15695
SPRY2	-2.03	1.33E-20	-0.35	130	-28.39	16405	-0.17	149	-16.6	15531	-0.42	132	-34.36	16422
ST6GALNAC1	-1.21	9.68E-09	-0.32	139	-28.4	16406	-0.18	124	-13.29	14821	-0.39	103	-20.51	15414
PAMR1	-3.29	1.01E-33	-0.38	124	-28.43	16407	-0.26	140	-23.41	16271	-0.46	120	-32.44	16378
PANK1	-2.18	1.94E-21	-0.29	148	-28.65	16408	-0.28	149	-28.2	16441	-0.28	120	-19.51	15250
HAND2	-1.86	6.14E-23	-0.32	140	-28.68	16409	-0.16	148	-15.58	15319	-0.39	131	-31.67	16349
GPR146	-1.68	2.33E-16	-0.31	143	-28.68	16410	-0.26	132	-21.06	16097	-0.31	116	-20.48	15411
KCNJ16	-2.6	8.87E-16	-0.34	134	-28.72	16411	-0.15	148	-14.3	15052	-0.4	130	-32.28	16373
NDEL1	-1.44	2.72E-26	-0.3	146	-28.77	16412	-0.35	110	-21.11	16103	-0.37	151	-36.94	16458
SLC5A1	-1.47	9.26E-17	-0.31	142	-28.79	16413	-0.14	126	-10.72	14142	-0.37	130	-29.85	16282
SORL1	-3.08	2.80E-22	-0.32	140	-28.79	16414	-0.27	145	-25.39	16360	-0.29	131	-23.39	15813
SPTBN1_t2	-1.22	1.01E-07	-0.24	171	-28.81	16415	-0.12	142	-11	14238	-0.19	163	-20.98	15499
IGFBP3_t1	-5.12	3.20E-35	-0.36	130	-28.82	16416	-0.26	123	-19.28	15898	NA	NA	NA	NA
GLYAT_t2	-6.2	1.21E-23	-0.38	126	-28.91	16417	-0.33	140	-29.64	16460	-0.3	123	-21.93	15648
CPEB3_t1	-2.11	5.74E-25	-0.32	140	-29.03	16418	NA	NA	NA	NA	-0.32	143	-30.16	16294
GRHL2	-1.22	1.24E-05	-0.35	134	-29.05	16419	-0.11	140	-10.34	14017	-0.38	135	-32.13	16367
SLC13A5	-2.61	3.93E-13	-0.29	149	-29.06	16420	-0.32	159	-35.31	16490	-0.27	127	-20.97	15497
SELP	-2.28	4.00E-29	-0.28	156	-29.22	16421	-0.2	107	-11.5	14372	-0.37	101	-18.68	15117
ASPN	-4.26	5.25E-20	-0.25	167	-29.36	16422	-0.07	120	-5.21	11780	-0.31	136	-27.07	16134
DUSP6	-2.76	2.57E-23	-0.32	141	-29.42	16423	-0.15	136	-12.49	14635	-0.41	120	-28.62	16235
SLITRK6	-1.97	1.17E-23	-0.35	133	-29.43	16424	-0.24	116	-15.98	15413	-0.37	120	-26.11	16042
CCDC3	-4.87	2.57E-31	-0.33	140	-29.45	16425	-0.16	124	-12.08	14533	-0.41	124	-30.55	16317
RNF150	-1.68	2.34E-18	-0.3	148	-29.53	16426	-0.16	144	-14.62	15129	-0.34	128	-26.88	16118
COMT	-2	1.14E-22	-0.4	124	-29.73	16427	-0.27	120	-18.57	15807	-0.44	107	-25.11	15964
CNBP	-1.5	5.91E-25	-0.29	152	-29.81	16428	-0.28	139	-24.53	16325	-0.37	141	-33.23	16398
MTTP	-2.4	1.25E-10	-0.28	156	-29.86	16429	-0.23	123	-16.98	15588	-0.19	121	-13.27	13917
HRSP12	-2.33	8.46E-13	-0.28	156	-29.86	16430	-0.34	109	-19.79	15960	-0.17	121	-12.06	13565
DAK	-2.34	1.48E-16	-0.39	127	-29.89	16431	-0.3	133	-24.65	16329	-0.31	131	-24.88	15950
FXN	-1.56	4.07E-21	-0.29	152	-29.89	16432	-0.21	142	-19.48	15926	-0.36	153	-37.01	16462
FAM83F	-1.63	3.10E-19	-0.36	132	-29.9	16433	-0.26	136	-22.39	16206	-0.38	120	-26.77	16110
SLC22A1	-6.2	1.56E-25	-0.38	129	-29.92	16434	-0.37	104	-19.88	15970	-0.36	90	-14.29	14189
CILP	-2.72	5.04E-22	-0.31	148	-30.07	16435	-0.17	128	-13.09	14773	-0.4	140	-35.61	16442
TTPAL_t1	-1.28	1.20E-06	-0.3	151	-30.07	16436	NA	NA	NA	NA	-0.22	150	-22.5	15722
EPHA2	-2.19	5.00E-24	-0.3	151	-30.12	16437	-0.22	127	-17.22	15621	-0.36	130	-28.66	16237
HDAC6	-1.62	3.06E-15	-0.34	139	-30.18	16438	-0.26	109	-15.11	15224	-0.34	112	-20.95	15492
ACAA2	-2.84	1.17E-22	-0.32	143	-30.2	16439	-0.29	115	-18.57	15808	-0.31	106	-17.1	14817
PDXDC1	-1.4	6.35E-14	-0.31	149	-30.26	16440	-0.16	128	-12.46	14629	-0.21	136	-18.23	15028

PDGFRA	-5.53	1.56E-27	-0.31	147	-30.29	16441	-0.17	123	-12.62	14673	-0.39	160	-43.4	16493
CYP2B6	-1.52	4.85E-21	-0.35	137	-30.35	16442	-0.3	126	-22.45	16211	-0.34	120	-23.9	15866
NDRG2_t1	-2.31	9.92E-23	-0.37	132	-30.37	16443	NA	NA	NA	NA	-0.38	121	-26.69	16105
HHIP	-2.69	1.14E-32	-0.34	139	-30.38	16444	-0.23	149	-22.83	16239	-0.39	111	-23.61	15842
FAM20C	-1.26	2.27E-05	-0.32	146	-30.51	16445	-0.26	133	-21.4	16130	-0.22	126	-16.44	14672
PDE8A	-1.27	2.63E-11	-0.31	149	-30.52	16446	-0.16	116	-10.89	14203	-0.16	123	-11.79	13485
IGF1	-3.36	2.73E-16	-0.33	144	-30.58	16447	-0.3	133	-24.79	16334	-0.2	143	-18.85	15144
CMBL	-2.45	8.93E-15	-0.37	133	-30.77	16448	-0.32	127	-24.91	16340	-0.25	134	-20.62	15431
ACAD11	-2.02	2.28E-18	-0.33	144	-30.78	16449	-0.26	114	-16.71	15550	-0.28	139	-24.68	15931
PELI2	-1.59	4.48E-18	-0.29	158	-30.95	16450	-0.13	148	-12.7	14690	-0.38	130	-30.38	16307
NGFR	-2.32	4.42E-37	-0.4	128	-31	16451	-0.23	137	-19.71	15953	-0.44	111	-26.8	16113
ADH4	-10.62	6.40E-23	-0.36	136	-31.07	16452	-0.35	111	-21.59	16139	-0.34	119	-23.42	15817
RBMS3_t2	-4.03	3.52E-28	-0.33	145	-31.09	16453	-0.17	147	-16.7	15547	-0.38	135	-32.72	16382
GPR128	-3.57	7.40E-28	-0.34	142	-31.13	16454	-0.29	130	-23.55	16280	-0.35	137	-30.31	16304
TBXA2R_t2	-2.51	2.29E-37	-0.39	131	-31.21	16455	-0.3	118	-20.42	16033	-0.45	124	-32.93	16389
ALDH8A1	-4.01	2.14E-22	-0.37	135	-31.33	16456	-0.33	131	-26.4	16397	-0.37	122	-26.9	16122
MKNK2_t2	-1.3	1.84E-13	-0.28	162	-31.55	16457	-0.25	135	-21.02	16090	-0.33	122	-23.54	15835
GLYATL1	-4.66	6.82E-21	-0.36	137	-31.56	16458	-0.33	124	-24.64	16328	-0.33	154	-33.95	16415
POLR1E	-1.56	2.08E-19	-0.3	155	-31.77	16459	-0.26	152	-26.22	16389	-0.27	118	-18.22	15024
STEAP4	-1.58	1.72E-19	-0.32	149	-31.79	16460	-0.18	135	-15.56	15316	-0.38	164	-43.62	16494
JAGN1	-1.26	8.87E-11	-0.37	137	-31.79	16461	-0.2	109	-11.71	14429	-0.34	130	-27.2	16142
NOL4	-1.31	3.15E-05	-0.3	155	-31.81	16462	-0.18	126	-13.61	14900	-0.16	147	-15.97	14580
XDH	-1.71	1.74E-15	-0.33	147	-31.82	16463	-0.37	131	-29.91	16465	-0.29	131	-23.1	15787
IL18R1_t1	-1.36	2.98E-22	-0.31	152	-31.88	16464	NA	NA	NA	NA	-0.28	140	-25.17	15967
ST6GALNAC3	-1.38	1.34E-16	-0.26	171	-31.91	16465	-0.2	135	-17.16	15610	-0.22	123	-15.87	14554
COLEC10	-2.55	7.67E-34	-0.34	145	-32.2	16466	-0.26	131	-21.4	16131	-0.4	142	-36.91	16456
GIMAP6	-1.8	1.21E-21	-0.32	152	-32.23	16467	-0.17	153	-17.9	15721	-0.43	147	-42.12	16488
SERPINA10	-2.26	7.12E-13	-0.31	154	-32.31	16468	-0.33	116	-21.61	16142	-0.28	102	-14.55	14254
ALDH6A1	-3.17	6.75E-20	-0.36	139	-32.38	16469	-0.35	119	-23.81	16295	-0.36	119	-24.64	15929
SRPX	-6.03	5.96E-33	-0.35	142	-32.42	16470	-0.19	104	-10	13880	-0.4	145	-38.27	16470
GOT2_t1	-2.01	2.12E-19	-0.36	141	-32.43	16471	NA	NA	NA	NA	-0.39	135	-33.13	16393
ADAMTS13_t2	-6.26	3.78E-48	-0.42	129	-32.89	16472	-0.28	103	-14.79	15168	-0.48	98	-22.9	15764
CCRL1	-1.46	1.31E-19	-0.34	146	-32.97	16473	-0.22	135	-19.03	15873	-0.37	119	-25.58	15993
BCO2_t1	-5.07	6.07E-33	-0.34	146	-33.06	16474	NA	NA	NA	NA	NA	NA	NA	NA
SLC22A7_t1	-2.75	4.17E-17	-0.34	148	-33.18	16475	NA	NA	NA	NA	NA	NA	NA	NA
CYP26A1	-7.12	9.19E-39	-0.37	141	-33.38	16476	-0.3	107	-17.3	15642	-0.29	114	-18.7	15121
RNF125	-2.53	9.50E-28	-0.35	146	-33.81	16477	-0.24	154	-25.18	16352	-0.35	159	-37.98	16467
HAO1	-2.7	8.30E-12	-0.31	161	-33.89	16478	-0.35	121	-24.54	16326	-0.25	126	-19.35	15228
EHD3	-2.53	3.15E-35	-0.38	140	-34.32	16479	-0.25	124	-18.29	15770	-0.43	136	-37.06	16463
TMEM27	-7.08	9.29E-32	-0.37	144	-34.65	16480	-0.24	149	-23.39	16267	-0.4	103	-21.06	15511
PIPOX	-1.9	7.53E-16	-0.36	148	-35.46	16481	-0.39	139	-34.77	16488	-0.23	152	-23.39	15812
SMAD9	-1.3	1.33E-18	-0.37	148	-36.1	16482	-0.23	144	-21.41	16133	-0.34	127	-26.42	16079
CA2	-2.59	1.04E-22	-0.35	154	-36.22	16483	-0.26	110	-15.63	15328	-0.23	128	-18.26	15036
IVD	-1.76	4.71E-25	-0.36	152	-36.36	16484	-0.36	139	-31.98	16476	-0.35	165	-40.29	16480
DCUN1D3	-1.67	6.54E-23	-0.4	142	-36.49	16485	-0.25	120	-17.73	15701	-0.37	149	-36.99	16460
MPPED1	-4.35	1.49E-26	-0.34	161	-37.98	16486	-0.29	114	-18.45	15796	-0.36	161	-40.4	16481
CD5L	-7.45	4.78E-37	-0.35	161	-38.63	16487	-0.29	125	-21.47	16135	-0.41	129	-32.62	16381
HSD17B13	-6.7	2.13E-29	-0.37	167	-42.73	16488	-0.32	127	-24.97	16342	-0.37	123	-27.09	16136
MPZL1_t2	1.45	2.03E-09	NA	NA	NA	NA	0.32	146	30.39	7	NA	NA	NA	NA
ZNF107_t2	1.33	1.48E-06	NA	NA	NA	NA	0.28	155	29.79	9	0.18	150	18.48	1554
TPM3_t5	1.44	2.12E-15	NA	NA	NA	NA	0.31	141	28.33	22	NA	NA	NA	NA
NOX4_t2	1.53	2.75E-15	NA	NA	NA	NA	0.33	135	28.32	23	NA	NA	NA	NA
HSF2_t2	1.27	1.69E-08	NA	NA	NA	NA	0.32	138	27.79	29	NA	NA	NA	NA
GK5_t2	1.3	6.49E-09	NA	NA	NA	NA	0.27	154	27.56	33	0.19	150	19.02	1448
NUP43_t2	1.52	2.40E-18	NA	NA	NA	NA	0.3	142	27.42	37	0.29	123	21.21	1082
MECOM_t3	1.26	1.84E-08	NA	NA	NA	NA	0.27	150	27.38	38	0.3	131	24.07	714
TUBA1C_t2	1.74	5.56E-15	NA	NA	NA	NA	0.26	151	26.73	43	0.25	115	16.47	1963
PHPT1_t2	1.39	2.13E-13	NA	NA	NA	NA	0.28	145	26.34	48	NA	NA	NA	NA
MTPN_t2	1.31	3.31E-07	NA	NA	NA	NA	0.27	147	26.2	52	0.27	130	21.22	1081
PPME1_t2	1.23	2.51E-17	NA	NA	NA	NA	0.28	142	26.19	53	NA	NA	NA	NA
DYNLL1_t2	1.51	1.17E-18	NA	NA	NA	NA	0.36	123	25.92	65	NA	NA	NA	NA
ATP6V1C1_t2	1.61	7.07E-11	NA	NA	NA	NA	0.25	151	25.65	66	NA	NA	NA	NA
PTBP3_t2	1.14	5.36E-08	NA	NA	NA	NA	0.26	147	25.6	67	NA	NA	NA	NA
NAA38_t2	1.2	3.28E-06	NA	NA	NA	NA	0.28	141	25.44	69	NA	NA	NA	NA
HNRNPA3_t2	1.55	1.16E-13	NA	NA	NA	NA	0.28	140	25.24	72	0.27	141	24.72	647
CCDC93_t2	1.44	2.05E-17	NA	NA	NA	NA	0.3	133	24.92	82	NA	NA	NA	NA
NPEPPS_t2	1.18	8.20E-07	NA	NA	NA	NA	0.24	152	24.9	83	0.17	126	12.99	2883
CXorf36_t2	1.2	7.79E-19	NA	NA	NA	NA	0.3	132	24.83	86	0.35	135	29.6	261
RGS5_t2	1.68	3.89E-05	NA	NA	NA	NA	0.26	144	24.56	96	NA	NA	NA	NA
PTMA_t2	1.31	9.27E-08	NA	NA	NA	NA	0.33	123	24.26	105	NA	NA	NA	NA
SUB1_t2	1.51	2.27E-13	NA	NA	NA	NA	0.27	138	23.93	118	0.26	155	27.14	414
ZBTB41_t2	1.45	1.29E-11	NA	NA	NA	NA	0.22	157	23.41	133	0.32	142	29.64	259
RABIF_t2	1.4	5.60E-21	NA	NA	NA	NA	0.24	148	23.34	136	NA	NA	NA	NA
YWHAZ_t2	1.23	8.09E-05	NA	NA	NA	NA	0.26	140	23.31	139	0.23	125	16.95	1841
EP400_t2	1.13	8.75E-18	NA	NA	NA	NA	0.24	148	23.27	141	0.2	135	17.42	1748
RBM8A_t2	1.43	2.13E-16	NA	NA	NA	NA	0.26	139	23.09	145	0.36	159	39.77	17
ZNF493_t2	1.3	3.70E-09	NA	NA	NA	NA	0.27	135	23.02	149	NA	NA	NA	NA
CCNYL1_t2	1.22	9.13E-07	NA	NA	NA	NA	0.24	145	22.96	154	NA	NA	NA	NA
VPS35_t2	1.21	0.000528938	NA	NA	NA	NA	0.27	134	22.85	160	NA	NA	NA	NA
SNX27_t2	1.58	1.52E-24	NA	NA	NA	NA	0.28	132	22.81	162	NA	NA	NA	NA
PLXDC1_t2	1.2	3.37E-11	NA	NA	NA	NA	0.23	148	22.77	166	NA	NA	NA	NA
GRPEL2_t2	1.33	1.58E-12	NA	NA	NA	NA	0.27	134	22.76	167	0.34	146	32.77	129
HN1_t2	1.49	7.30E-16	NA	NA	NA	NA	0.29	128	22.53	183	NA	NA	NA	NA
UBFD1_t2	1.23	7.89E-09	NA	NA	NA	NA	0.3	126	22.46	186	0.18	158	19.34	1377
HNRNPU_t3	1.21	2.02E-07	NA	NA	NA	NA	0.22	152	22.24	200	NA	NA	NA	NA
CACYBP_t2	1.61	4.38E-13	NA	NA	NA	NA	0.22	151	22.05	212	NA	NA	NA	NA
HIATL1_t2	1.1	0.0039349	NA	NA	NA	NA	0.22	151	22.03	216	NA	NA	NA	NA
PRIM2_t2	1.26	6.59E-15	NA	NA	NA	NA	0.29	126	22	220	NA	NA	NA	NA
METTL9_t2	-1.06	0.231729466	NA	NA	NA	NA	0.2	157	21.49	248	NA	NA	NA	NA
ZNF100_t2	1.04	0.002957686	NA	NA	NA	NA	0.25	135	21.35	255	NA	NA	NA	NA
GNPTAB_t2	1.1	0.113218724	NA	NA	NA	NA	0.23	143	21.27	260	0.12	158	13.37	2773
PLXNA1_t2	1.11	0.111595753	NA	NA	NA	NA	0.27	127	21.06	288	NA	NA	NA	NA
VPS45_t2	1.28	5.81E-15	NA	NA	NA	NA	0.22	145	20.96	298	NA	NA	NA	NA
SERF1B_t2	1.29	6.59E-10	NA	NA	NA	NA	0.25	133	20.87	305	NA	NA	NA	NA
TPR_t2	1.44	5.60E-10	NA	NA	NA	NA	0.29	123	20.83	312	0.23	120	16.31	2007
CBX1_t2	1.46	9.79E-17	NA	NA	NA	NA	0.32	115	20.82	313	NA	NA	NA	NA
NAA40_t2	1.16	1.43E-08	NA	NA	NA	NA	0.22	143	20.77	317	NA	NA	NA	NA
ABCC4_t2	1.15	2.05E-07	NA	NA	NA	NA	0.19	158	20.49	341	NA	NA	NA	NA
LYRM2_t2	1.17	0.000128048	NA	NA	NA	NA	0.23	140	20.49	343	NA	NA	NA	NA
RAB11A_t2	1.23	2.75E-07	NA	NA	NA	NA	0.23	139	20.46	345	0.29	132	23.52	788
KITLG_t2	1.44	3.06E-09	NA	NA	NA	NA	0.25	132	20.4	352	0.21	129	16.43	1975
PPP1R2_t2	1.18	0.002587321	NA	NA	NA	NA	0.25	132	20.37	356	NA	NA	NA	NA
RBBP5_t2	1.2	4.10E-17	NA	NA	NA	NA	0.22	144	20.37	357	NA	NA	NA	NA
LRP12_t2	1.3	9.76E-05	NA	NA	NA	NA	0.23	139	20.34	362	0.2	149	20.23	1242
MMS22L_t2	1.44	1.92E-15	NA	NA	NA	NA	0.26	128	20.28	369	NA	NA	NA	NA
ZNF714_t2	1.04	0.011777654	NA	NA	NA	NA	0.23	139	20.23	373	NA	NA	NA	NA

MTHFD1L_t2	1.4	3.15E-14	NA	NA	NA	NA	0.27	124	20.22	375	NA	NA	NA	NA
KLHL12_t2	1.34	8.79E-18	NA	NA	NA	NA	0.22	143	20.17	382	NA	NA	NA	NA
HDAC4_t2	1.32	7.91E-07	NA	NA	NA	NA	0.25	132	20.11	393	NA	NA	NA	NA
RALGPS1_t2	1.11	1.12E-07	NA	NA	NA	NA	0.19	155	20.09	394	NA	NA	NA	NA
ASPM_t2	4.3	3.88E-29	NA	NA	NA	NA	0.26	126	20.05	397	0.43	110	25.91	516
CDC42SE1_t2	1.18	0.000593741	NA	NA	NA	NA	0.2	152	19.96	405	0.1	127	8.07	4596
ORAOV1_t2	1.36	3.60E-09	NA	NA	NA	NA	0.26	126	19.84	417	NA	NA	NA	
NETO2_t2	1.17	2.73E-08	NA	NA	NA	NA	0.24	132	19.7	432	NA	NA	NA	
DCLK1_t2	1.11	0.02295149	NA	NA	NA	NA	0.22	138	19.41	462	NA	NA	NA	
POU2F1_t2	1.03	0.003678805	NA	NA	NA	NA	0.2	145	19.35	471	NA	NA	NA	
NUDCD1_t2	1.61	1.93E-16	NA	NA	NA	NA	0.26	124	19.18	490	0.43	138	37.85	39
CA12_t2	1.96	1.15E-09	NA	NA	NA	NA	0.21	140	19.12	494	NA	NA	NA	
DIAPH3_t3	1.41	1.20E-18	NA	NA	NA	NA	0.25	127	19.08	505	NA	NA	NA	
FMNL3_t2	1.01	0.336079607	NA	NA	NA	NA	0.18	155	19.05	511	0.03	132	2.37	7250
SLC7A11_t2	1.14	1.78E-07	NA	NA	NA	NA	0.2	147	19.01	516	NA	NA	NA	
TPM4_t2	-1.1	0.259317474	NA	NA	NA	NA	0.21	141	19	519	NA	NA	NA	
TRIM32_t2	1.44	2.07E-20	NA	NA	NA	NA	0.24	130	18.92	529	NA	NA	NA	
SNRPC_t2	1.37	7.98E-18	NA	NA	NA	NA	0.22	136	18.86	542	0.33	122	24.1	708
UCHL5_t2	1.34	4.93E-08	NA	NA	NA	NA	0.22	136	18.77	550	NA	NA	NA	
GMFB_t2	1.31	8.71E-12	NA	NA	NA	NA	0.19	151	18.73	553	NA	NA	NA	
TICRR_t2	1.47	5.23E-20	NA	NA	NA	NA	0.22	135	18.66	564	0.4	113	25.35	578
PARPBP_t2	1.2	2.17E-13	NA	NA	NA	NA	0.26	122	18.64	567	NA	NA	NA	
DBF4_t2	1.5	2.65E-16	NA	NA	NA	NA	0.24	126	18.62	569	0.37	122	26.68	441
TAF9_t3	1.59	1.17E-16	NA	NA	NA	NA	0.25	124	18.55	577	NA	NA	NA	
AP4E1_t2	1.22	0.000133287	NA	NA	NA	NA	0.2	141	18.55	578	NA	NA	NA	
ZNF257_t2	1.22	6.25E-10	NA	NA	NA	NA	0.24	128	18.43	594	NA	NA	NA	
TP53RK_t2	1.1	3.07E-06	NA	NA	NA	NA	0.19	146	18.41	598	NA	NA	NA	
CKS1B_t2	2.04	5.37E-26	NA	NA	NA	NA	0.31	109	18.39	603	NA	NA	NA	
RP9_t2	1.46	2.68E-23	NA	NA	NA	NA	0.27	118	18.2	621	NA	NA	NA	
POP1_t2	1.2	1.89E-12	NA	NA	NA	NA	0.21	138	18.14	636	NA	NA	NA	
GNRHR2_t2	1.14	0.000256004	NA	NA	NA	NA	0.18	153	18.12	637	NA	NA	NA	
EIF5A2_t2	1.36	5.36E-06	NA	NA	NA	NA	0.22	132	18.02	659	NA	NA	NA	
H2AFZ_t2	1.97	2.04E-20	NA	NA	NA	NA	0.29	113	18.02	660	NA	NA	NA	
ATP11A_t3	1.1	0.022974202	NA	NA	NA	NA	0.17	153	18.01	662	0.03	150	3.46	6689
UBE2W_t2	1.13	7.86E-05	NA	NA	NA	NA	0.19	143	17.97	668	NA	NA	NA	
DDX10_t2	1.1	2.67E-09	NA	NA	NA	NA	0.24	125	17.91	684	0.26	117	17.2	1784
CDCA7_t2	1.73	5.38E-10	NA	NA	NA	NA	0.23	126	17.83	695	NA	NA	NA	
RACGAP1_t2	2.88	5.86E-32	NA	NA	NA	NA	0.29	112	17.82	697	0.43	164	48.79	2
MYCBP_t2	1.2	2.58E-05	NA	NA	NA	NA	0.19	143	17.72	709	NA	NA	NA	
DESI2_t2	1.36	4.67E-10	NA	NA	NA	NA	0.23	127	17.7	713	0.3	138	26.28	475
PRMT2_t2	-1.02	0.582133927	NA	NA	NA	NA	0.18	149	17.7	715	NA	NA	NA	
RAB3B_t2	1.23	4.51E-09	NA	NA	NA	NA	0.17	153	17.69	720	0.22	127	16.85	1861
GPR161_t2	1.07	0.008896659	NA	NA	NA	NA	0.17	153	17.69	721	0.04	150	4.21	6337
KIAA1244_t2	1.45	1.68E-07	NA	NA	NA	NA	0.18	150	17.58	738	NA	NA	NA	
KCTD10_t2	1.01	0.733369171	NA	NA	NA	NA	0.18	147	17.49	749	NA	NA	NA	
ZNF431_t2	1.08	0.015656211	NA	NA	NA	NA	0.17	155	17.46	752	NA	NA	NA	
NUP160_t2	1.1	0.029714143	NA	NA	NA	NA	0.25	120	17.41	762	NA	NA	NA	
CDCA3_t2	1.68	1.26E-25	NA	NA	NA	NA	0.24	121	17.34	768	0.37	136	32.23	153
HNRNPL_t2	1.12	3.30E-07	NA	NA	NA	NA	0.2	135	17.34	771	0.01	125	1.04	7994
NSF_t2	1.12	5.27E-10	NA	NA	NA	NA	0.2	138	17.3	776	NA	NA	NA	
STK39_t2	1.47	4.00E-07	NA	NA	NA	NA	0.18	145	17.3	780	NA	NA	NA	
SKA1_t2	1.93	8.26E-23	NA	NA	NA	NA	0.2	136	17.21	799	0.41	121	29.12	289
PIP4K2B_t2	1.24	2.43E-10	NA	NA	NA	NA	0.18	143	17.06	827	NA	NA	NA	
KIAA1468_t2	1.03	0.396027132	NA	NA	NA	NA	0.21	131	17.03	832	NA	NA	NA	
YWHAQ_t2	1.31	5.70E-08	NA	NA	NA	NA	0.2	135	16.99	845	0.22	135	18.97	1460
EIF1AD_t2	1.15	4.58E-09	NA	NA	NA	NA	0.19	138	16.96	854	NA	NA	NA	
ARL1_t2	1.24	2.79E-06	NA	NA	NA	NA	0.22	126	16.93	855	NA	NA	NA	
UBE2Q2_t2	1.15	4.17E-05	NA	NA	NA	NA	0.23	122	16.9	861	NA	NA	NA	
ELAVL1_t2	1.2	5.93E-12	NA	NA	NA	NA	0.21	131	16.9	862	0.23	145	21.43	1059
ANGPT2_t2	1.16	1.13E-08	NA	NA	NA	NA	0.17	151	16.82	875	NA	NA	NA	
ROBO1_t2	2.55	7.19E-16	NA	NA	NA	NA	0.23	124	16.75	887	0.3	159	32.65	135
ANGEL2_t2	1.29	3.81E-08	NA	NA	NA	NA	0.22	125	16.61	916	NA	NA	NA	
CCT6A_t2	1.57	2.34E-12	NA	NA	NA	NA	0.26	115	16.61	917	NA	NA	NA	
SMPD4_t2	1.13	4.92E-05	NA	NA	NA	NA	0.24	120	16.6	921	NA	NA	NA	
FKBP9_t2	1.22	8.96E-06	NA	NA	NA	NA	0.19	137	16.59	922	NA	NA	NA	
SLC7A6_t2	1.1	1.46E-08	NA	NA	NA	NA	0.2	134	16.57	927	NA	NA	NA	
DDA1_t2	1.14	1.19E-08	NA	NA	NA	NA	0.18	140	16.56	931	0.12	137	10.54	3676
WDR26_t2	1.19	8.66E-05	NA	NA	NA	NA	0.18	140	16.56	932	NA	NA	NA	
USP3_t2	1.17	5.97E-05	NA	NA	NA	NA	0.19	139	16.52	938	0.24	147	22.84	860
ZNF764_t2	1.31	3.20E-10	NA	NA	NA	NA	0.21	130	16.44	953	NA	NA	NA	
ZCCHC17_t2	1.19	7.12E-08	NA	NA	NA	NA	0.23	121	16.44	954	NA	NA	NA	
ITGAV_t2	1.15	0.124242077	NA	NA	NA	NA	0.2	133	16.41	957	NA	NA	NA	
SRSF10_t3	1.11	0.008671938	NA	NA	NA	NA	0.19	135	16.4	959	NA	NA	NA	
PTPN14_t2	1.14	6.77E-07	NA	NA	NA	NA	0.19	136	16.39	961	NA	NA	NA	
TWISTNB_t2	1.19	2.95E-05	NA	NA	NA	NA	0.2	131	16.38	963	NA	NA	NA	
ZNF468_t2	1.15	0.002337908	NA	NA	NA	NA	0.23	120	16.38	964	NA	NA	NA	
RNF34_t2	1.24	4.58E-09	NA	NA	NA	NA	0.21	128	16.37	967	NA	NA	NA	
ZNF2_t2	1.12	6.75E-14	NA	NA	NA	NA	0.22	124	16.36	970	0.18	131	14.85	2371
FAM40B_t2	1.43	2.39E-14	NA	NA	NA	NA	0.19	134	16.3	980	0.33	138	29.35	276
PPP1CC_t2	1.21	0.00026911	NA	NA	NA	NA	0.2	131	16.26	989	NA	NA	NA	
PDDC1_t2	1.3	3.63E-08	NA	NA	NA	NA	0.18	139	16.23	996	NA	NA	NA	
DAD1_t2	1.35	7.18E-17	NA	NA	NA	NA	0.22	123	16.19	1000	NA	NA	NA	
ZNF248_t2	1.14	4.71E-05	NA	NA	NA	NA	0.2	132	16.18	1003	NA	NA	NA	
METTL1_t2	1.28	9.45E-13	NA	NA	NA	NA	0.21	126	16.15	1009	0.16	116	10.24	3783
DUSP18_t2	1.42	3.42E-20	NA	NA	NA	NA	0.2	131	16.14	1010	0.17	142	15.42	2213
CDKN2A_t2	1.56	2.78E-12	NA	NA	NA	NA	0.22	123	16.08	1025	NA	NA	NA	
CEP89_t2	1.21	6.94E-13	NA	NA	NA	NA	0.29	106	16.04	1032	0.29	100	14.41	2485
TRIM6_t2	1.36	2.38E-06	NA	NA	NA	NA	0.17	142	16.03	1039	NA	NA	NA	
FAM171B_t2	1.06	0.001200662	NA	NA	NA	NA	0.19	134	16.01	1046	0.11	126	8.67	4360
PDPK1_t2	1.24	3.74E-07	NA	NA	NA	NA	0.19	135	16	1048	0.15	156	15.72	2149
PKNOX1_t2	1.17	2.08E-07	NA	NA	NA	NA	0.18	139	15.95	1060	NA	NA	NA	
INTS4_t2	1.09	2.19E-07	NA	NA	NA	NA	0.26	112	15.89	1070	0.21	100	10.36	3737
UBE2E3_t2	1.23	6.61E-06	NA	NA	NA	NA	0.18	136	15.87	1075	0.16	119	11.2	3445
SMAD2_t2	1.09	1.84E-08	NA	NA	NA	NA	0.2	130	15.81	1087	NA	NA	NA	
SMARCC1_t2	1.16	1.59E-07	NA	NA	NA	NA	0.18	136	15.79	1091	0.15	124	11.41	3376
PPP2R5A_t2	1.57	8.98E-13	NA	NA	NA	NA	0.19	132	15.75	1102	0.35	134	29.81	254
PARD3B_t3	1.18	0.000118401	NA	NA	NA	NA	0.17	145	15.7	1106	0.16	128	12.38	3075
GPSM2_t2	1.96	6.60E-25	NA	NA	NA	NA	0.23	118	15.62	1116	0.44	114	27.97	372
HDDC2_t2	1.02	0.642464244	NA	NA	NA	NA	0.17	142	15.55	1132	NA	NA	NA	
FTH1_t2	1.61	3.75E-12	NA	NA	NA	NA	0.26	110	15.45	1154	NA	NA	NA	
POLR3A_t2	1.16	3.02E-11	NA	NA	NA	NA	0.16	145	15.4	1169	NA	NA	NA	
ZNF585A_t2	1.05	0.000186747	NA	NA	NA	NA	0.16	144	15.37	1178	0.06	130	4.63	6118
MAP4K5_t2	1.04	0.123310954	NA	NA	NA	NA	0.2	128	15.31	1189	0.12	120	8.4	4467
FAM175A_t2	1.09	7.55E-07	NA	NA	NA	NA	0.18	135	15.29	1198	NA	NA	NA	
NMNAT2_t2	1.05	0.112586137	NA	NA	NA	NA	0.16	143	15.24	1213	NA	NA	NA	
PREPL_t2	1.19	0.003055166	NA	NA	NA	NA	0.2	127	15.19	1227	NA	NA	NA	

ZNF557_t2	1.14	6.08E-07	NA	NA	NA	NA	0.16	145	15.14	1238	NA	NA	NA	NA
DYNC2LI1_t2	1.16	2.58E-05	NA	NA	NA	NA	0.21	122	15.14	1239	NA	NA	NA	NA
GPD1L_t2	1.12	0.079584668	NA	NA	NA	NA	0.17	140	15.1	1249	0.11	128	8.54	4408
ATXN2L_t3	1.1	0.0328989	NA	NA	NA	NA	0.22	120	15.09	1251	NA	NA	NA	NA
CRNKL1_t2	1.17	1.83E-06	NA	NA	NA	NA	0.18	133	15.09	1252	NA	NA	NA	NA
ITGA2_t2	1.65	7.34E-12	NA	NA	NA	NA	0.19	131	15.09	1253	NA	NA	NA	NA
CEP112_t2	1.13	9.40E-05	NA	NA	NA	NA	0.17	138	15.08	1257	NA	NA	NA	NA
ZNF160_t2	1.02	0.529578719	NA	NA	NA	NA	0.18	133	15.04	1266	0.12	129	9.56	4035
MSANTD3_t2	1.18	0.00024936	NA	NA	NA	NA	0.22	118	15.01	1275	0.13	103	7.05	5008
ZNF761_t2	1.15	0.016762712	NA	NA	NA	NA	0.21	120	14.99	1284	NA	NA	NA	NA
DUSP19_t2	1.14	4.77E-07	NA	NA	NA	NA	0.18	132	14.97	1287	NA	NA	NA	NA
ANAPC7_t2	1.24	1.36E-13	NA	NA	NA	NA	0.24	112	14.92	1301	NA	NA	NA	NA
MAPRE1_t2	1.05	0.287769468	NA	NA	NA	NA	0.2	126	14.89	1310	NA	NA	NA	NA
TMEM194A_t2	1.1	6.66E-09	NA	NA	NA	NA	0.16	140	14.81	1327	NA	NA	NA	NA
RABGGTB_t2	1.18	0.003178445	NA	NA	NA	NA	0.17	134	14.69	1353	NA	NA	NA	NA
ZNF776_t2	1.05	6.18E-06	NA	NA	NA	NA	0.16	143	14.68	1358	0.08	138	7.22	4934
ATP6V1C2_t2	1.05	0.003293619	NA	NA	NA	NA	0.17	134	14.65	1364	NA	NA	NA	NA
MCAM_t2	1.26	1.34E-06	NA	NA	NA	NA	0.19	129	14.65	1366	NA	NA	NA	NA
SIPA1L3_t2	1.29	3.73E-11	NA	NA	NA	NA	0.2	124	14.64	1368	0.23	119	15.83	2123
ADAT2_t2	1.09	0.0039349	NA	NA	NA	NA	0.18	130	14.61	1373	NA	NA	NA	NA
GOLM1_t2	1.66	1.15E-11	NA	NA	NA	NA	0.19	127	14.58	1383	NA	NA	NA	NA
UBQLN1_t3	1.19	1.00E-06	NA	NA	NA	NA	0.17	135	14.57	1386	0.19	143	17.52	1730
RFWD2_t2	1.4	4.25E-13	NA	NA	NA	NA	0.22	117	14.54	1396	NA	NA	NA	NA
ZDHHC13_t2	1.06	0.012832182	NA	NA	NA	NA	0.22	116	14.52	1398	NA	NA	NA	NA
ZNF498_t2	1.12	1.19E-08	NA	NA	NA	NA	0.19	128	14.5	1406	NA	NA	NA	NA
OTUB2_t2	1.26	2.21E-16	NA	NA	NA	NA	0.16	140	14.44	1415	0.34	120	23.88	737
PURB_t2	1.14	0.00378186	NA	NA	NA	NA	0.16	139	14.43	1420	NA	NA	NA	NA
RAB6B_t2	1.03	0.064516497	NA	NA	NA	NA	0.16	143	14.43	1421	NA	NA	NA	NA
HINFP_t2	1.12	4.04E-05	NA	NA	NA	NA	0.2	122	14.38	1429	0.03	113	1.86	7528
GGNBP2_t2	1.21	8.98E-06	NA	NA	NA	NA	0.17	132	14.34	1440	NA	NA	NA	NA
ATP1B1_t2	1.56	8.38E-08	NA	NA	NA	NA	0.15	148	14.33	1441	0.22	139	19.21	1407
RBM34_t2	1.38	1.17E-09	NA	NA	NA	NA	0.2	120	14.3	1455	NA	NA	NA	NA
AMPD3_t2	1.07	0.278036654	NA	NA	NA	NA	0.19	126	14.29	1457	NA	NA	NA	NA
LEPROT_t2	-1.1	0.038620666	NA	NA	NA	NA	0.15	146	14.27	1460	NA	NA	NA	NA
HOOK3_t2	1.09	0.046808747	NA	NA	NA	NA	0.16	139	14.24	1467	NA	NA	NA	NA
ICK_t2	1.75	2.30E-10	NA	NA	NA	NA	0.16	137	14.19	1484	0.25	135	21.19	1087
GMCL1_t2	1.3	3.61E-15	NA	NA	NA	NA	0.23	111	14.15	1491	0.32	139	28.39	345
UBE2N_t2	1.16	2.27E-05	NA	NA	NA	NA	0.19	124	14.14	1496	NA	NA	NA	NA
GATS_t2	1.2	4.48E-13	NA	NA	NA	NA	0.19	125	14.12	1500	NA	NA	NA	NA
POLR1B_t2	1.08	0.000399689	NA	NA	NA	NA	0.16	136	14.08	1513	NA	NA	NA	NA
SYPL1_t2	1.37	1.50E-08	NA	NA	NA	NA	0.22	114	14	1529	0.35	132	28.3	353
CD59_t2	1.14	0.02827206	NA	NA	NA	NA	0.12	163	13.95	1545	0.04	134	3.64	6597
ICA1L_t3	1.01	0.314846678	NA	NA	NA	NA	0.14	151	13.88	1569	0.01	143	1.1	7962
SEPT9_t2	1.24	1.98E-06	NA	NA	NA	NA	0.17	132	13.81	1597	NA	NA	NA	NA
CABIN1_t2	1.12	0.000124998	NA	NA	NA	NA	0.2	120	13.81	1598	NA	NA	NA	NA
FAM86C1_t2	1.09	7.26E-10	NA	NA	NA	NA	0.15	142	13.74	1614	NA	NA	NA	NA
ZSCAN29_t2	1.29	2.47E-12	NA	NA	NA	NA	0.13	152	13.73	1616	0.31	134	26.09	493
ZNF614_t2	1.01	0.901936531	NA	NA	NA	NA	0.13	152	13.71	1620	NA	NA	NA	NA
GAS8_t2	1.26	3.09E-10	NA	NA	NA	NA	0.13	159	13.67	1633	0.21	119	14.37	2497
DR1_t2	1.09	0.017440044	NA	NA	NA	NA	0.17	132	13.66	1635	NA	NA	NA	NA
MLEC_t2	1.26	4.31E-07	NA	NA	NA	NA	0.16	135	13.65	1639	NA	NA	NA	NA
ETV1_t2	1.19	1.28E-05	NA	NA	NA	NA	0.17	132	13.62	1648	0.18	147	17.22	1783
INPP5F_t2	1.09	0.020486965	NA	NA	NA	NA	0.18	127	13.6	1652	NA	NA	NA	NA
ZNF343_t2	1.22	9.28E-12	NA	NA	NA	NA	0.15	139	13.58	1664	NA	NA	NA	NA
CAMK2G_t2	1.19	5.61E-07	NA	NA	NA	NA	0.2	117	13.57	1666	NA	NA	NA	NA
G2E3_t2	1.19	3.08E-05	NA	NA	NA	NA	0.14	146	13.55	1671	NA	NA	NA	NA
RBBP4_t2	1.15	0.000106332	NA	NA	NA	NA	0.16	135	13.54	1674	0.13	132	10.93	3533
KIF21A_t2	1.14	0.041803167	NA	NA	NA	NA	0.17	128	13.53	1677	NA	NA	NA	NA
ZNF682_t2	1.17	0.007213321	NA	NA	NA	NA	0.18	123	13.48	1690	NA	NA	NA	NA
KCNE1L_t2	1.5	1.39E-10	NA	NA	NA	NA	0.16	132	13.47	1692	NA	NA	NA	NA
SKA2_t2	1.23	3.15E-08	NA	NA	NA	NA	0.17	131	13.41	1707	NA	NA	NA	NA
CHCHD3_t2	1.33	1.45E-11	NA	NA	NA	NA	0.17	128	13.37	1724	NA	NA	NA	NA
GTSE1_t2	1.73	7.15E-23	NA	NA	NA	NA	0.17	127	13.31	1745	0.31	128	24.47	673
NAA15_t2	1.04	0.227380028	NA	NA	NA	NA	0.16	132	13.3	1750	NA	NA	NA	NA
FAM126A_t2	1.08	6.56E-08	NA	NA	NA	NA	0.16	131	13.13	1791	NA	NA	NA	NA
SLC6A6_t2	-1.25	0.004674967	NA	NA	NA	NA	0.12	157	13.13	1793	NA	NA	NA	NA
SCML2_t2	1.05	9.05E-05	NA	NA	NA	NA	0.17	127	13.11	1799	NA	NA	NA	NA
DIMT1_t2	1.16	7.85E-06	NA	NA	NA	NA	0.14	146	13.11	1802	0.23	120	15.77	2139
TNPO2_t2	1.02	0.235754566	NA	NA	NA	NA	0.13	149	13.06	1810	NA	NA	NA	NA
RHOQ_t2	1.09	0.058903559	NA	NA	NA	NA	0.16	133	13.06	1811	NA	NA	NA	NA
SKP1_t3	1.19	0.000371075	NA	NA	NA	NA	0.16	129	13.02	1824	NA	NA	NA	NA
HAUS6_t2	1.27	5.14E-05	NA	NA	NA	NA	0.15	138	12.99	1835	0.25	128	19.77	1309
GAPVD1_t2	1.04	0.043845091	NA	NA	NA	NA	0.17	128	12.97	1843	0.13	145	12.53	3036
ARIH2_t2	1.18	3.51E-05	NA	NA	NA	NA	0.15	135	12.9	1868	NA	NA	NA	NA
KIAA0528_t2	1.04	0.581657362	NA	NA	NA	NA	0.14	139	12.89	1870	NA	NA	NA	NA
PGAM5_t2	1.18	3.06E-11	NA	NA	NA	NA	0.2	116	12.88	1872	0.24	119	16.71	1903
ANKRD26_t2	1.14	7.17E-05	NA	NA	NA	NA	0.16	130	12.84	1883	NA	NA	NA	NA
CHMP4B_t2	1.11	0.000649572	NA	NA	NA	NA	0.16	128	12.84	1885	NA	NA	NA	NA
RHBDL2_t2	1.12	0.008839035	NA	NA	NA	NA	0.16	131	12.82	1892	NA	NA	NA	NA
ALYREF_t2	1.44	2.32E-15	NA	NA	NA	NA	0.24	104	12.81	1895	NA	NA	NA	NA
RCOR3_t2	1.31	6.63E-10	NA	NA	NA	NA	0.17	125	12.8	1897	0.3	130	23.84	743
ZFYVE26_t2	1.32	7.19E-11	NA	NA	NA	NA	0.17	124	12.8	1899	0.14	149	13.79	2649
DYM_t2	1.23	2.49E-06	NA	NA	NA	NA	0.2	113	12.79	1902	0.21	130	16.43	1977
RAB1A_t2	1.09	0.139240519	NA	NA	NA	NA	0.15	134	12.74	1916	NA	NA	NA	NA
SETD8_t2	1.05	0.017745272	NA	NA	NA	NA	0.18	119	12.67	1938	0.05	131	4.45	6215
SF3B3_t2	1.07	2.15E-07	NA	NA	NA	NA	0.13	147	12.6	1954	NA	NA	NA	NA
ALG1_t2	1.31	4.27E-18	NA	NA	NA	NA	0.14	139	12.57	1963	NA	NA	NA	NA
RAB3IP_t2	1.07	0.289346991	NA	NA	NA	NA	0.13	143	12.47	1988	NA	NA	NA	NA
PCDHB12_t2	1.09	0.006474865	NA	NA	NA	NA	0.12	151	12.46	1989	0.06	118	3.88	6480
CDH6_t2	1.01	0.872624975	NA	NA	NA	NA	0.13	145	12.44	1994	NA	NA	NA	NA
ZNF600_t2	1.2	0.000393671	NA	NA	NA	NA	0.23	103	12.42	2002	NA	NA	NA	NA
TBCD_t2	1.3	4.93E-13	NA	NA	NA	NA	0.16	127	12.38	2014	0.13	132	10.6	3657
AIFM2_t2	1.63	1.14E-11	NA	NA	NA	NA	0.18	119	12.37	2017	0.15	128	11.37	3393
PUM1_t2	1.1	0.004394047	NA	NA	NA	NA	0.15	135	12.35	2022	NA	NA	NA	NA
IGF2BP1_t2	1.39	1.18E-09	NA	NA	NA	NA	0.14	140	12.31	2033	NA	NA	NA	NA
RPL23A_t2	1.18	0.001157741	NA	NA	NA	NA	0.16	125	12.3	2035	NA	NA	NA	NA
TMEM169_t2	1.16	2.48E-09	NA	NA	NA	NA	0.15	133	12.28	2041	NA	NA	NA	NA
PPP6C_t2	1.06	0.011376234	NA	NA	NA	NA	0.12	152	12.23	2055	0.08	149	8.15	4561
PDCD6IP_t2	1.14	0.038190213	NA	NA	NA	NA	0.13	142	12.23	2056	0.19	147	18.82	1499
PGAP1_t2	1.05	6.27E-05	NA	NA	NA	NA	0.16	124	12.19	2064	NA	NA	NA	NA
HSPA13_t2	1.15	0.018594652	NA	NA	NA	NA	0.18	119	12.19	2065	NA	NA	NA	NA
HMGN1_t2	1.18	0.001854191	NA	NA	NA	NA	0.15	132	12.17	2078	0.17	120	11.99	3202
SLC16A6_t2	-1.01	0.757674703	NA	NA	NA	NA	0.13	146	12.13	2089	NA	NA	NA	NA
ADAM17_t2	-1.07	0.107743521	NA	NA	NA	NA	0.12	147	12.11	2096	NA	NA	NA	NA
ZNF704_t2	1.1	0.046610475	NA	NA	NA	NA	0.12	150	12.11	2097	NA	NA	NA	NA
UACA_t2	1.09	0.046256928	NA	NA	NA	NA	0.15	130	12.01	2126	NA	NA	NA	NA

RPS7_t2	1.5	6.44E-13	NA	NA	NA	NA	0.28	93	11.98	2131	0.31	68	5.49	5689
SLC25A12_t2	1.07	0.001661952	NA	NA	NA	NA	0.17	119	11.88	2160	0.12	156	13.06	2867
BOLA3_t2	1.67	3.54E-26	NA	NA	NA	NA	0.2	109	11.86	2166	0.41	100	20.46	1204
HSPA4_t2	1.34	3.15E-07	NA	NA	NA	NA	0.17	120	11.86	2168	NA	NA	NA	NA
UBOX5_t2	1.15	0.000366112	NA	NA	NA	NA	0.12	149	11.82	2178	0.16	119	11.01	3512
DOCK4_t2	1.12	0.028275279	NA	NA	NA	NA	0.14	137	11.82	2180	0.24	139	21.56	1039
EPG5_t2	1.1	3.25E-07	NA	NA	NA	NA	0.14	135	11.82	2181	NA	NA	NA	NA
ZNF25_t2	-1.01	0.803567638	NA	NA	NA	NA	0.16	124	11.79	2192	NA	NA	NA	NA
TMEM38B_t2	1.68	3.66E-22	NA	NA	NA	NA	0.13	138	11.75	2214	NA	NA	NA	NA
ZFP30_t2	1.04	0.004745784	NA	NA	NA	NA	0.13	143	11.71	2227	NA	NA	NA	NA
MFSD5_t2	1.36	1.66E-18	NA	NA	NA	NA	0.17	120	11.71	2228	NA	NA	NA	NA
PRR7_t2	1.17	1.75E-05	NA	NA	NA	NA	0.17	118	11.69	2233	NA	NA	NA	NA
POC1A_t2	1.8	8.64E-22	NA	NA	NA	NA	0.2	108	11.69	2234	0.33	150	33.19	115
FRYL_t2	1.03	0.000240922	NA	NA	NA	NA	0.16	124	11.68	2240	NA	NA	NA	NA
CAPRIN1_t3	1.1	0.036790354	NA	NA	NA	NA	0.13	137	11.67	2244	NA	NA	NA	NA
IGF2BP2_t2	1.38	0.000290559	NA	NA	NA	NA	0.17	120	11.65	2259	NA	NA	NA	NA
VDAC1_t2	1.22	7.46E-08	NA	NA	NA	NA	0.15	130	11.62	2269	0.26	138	22.57	888
PRUNE_t2	1.07	1.88E-09	NA	NA	NA	NA	0.15	126	11.57	2281	NA	NA	NA	NA
STRAP_t2	1.02	0.689476601	NA	NA	NA	NA	0.14	130	11.56	2285	NA	NA	NA	NA
MRPL33_t2	1.11	0.005669685	NA	NA	NA	NA	0.16	123	11.54	2291	NA	NA	NA	NA
ZNF589_t2	1.08	0.01106307	NA	NA	NA	NA	0.1	163	11.54	2292	NA	NA	NA	NA
LSG1_t2	1.14	2.82E-05	NA	NA	NA	NA	0.14	134	11.52	2299	0.16	120	11.21	3440
PPP1CB_t2	1.04	0.217277672	NA	NA	NA	NA	0.15	128	11.49	2309	NA	NA	NA	NA
RPL35_t2	1.31	3.89E-11	NA	NA	NA	NA	0.28	91	11.44	2324	0.21	84	7.25	4929
BMS1_t2	1.32	3.30E-11	NA	NA	NA	NA	0.1	161	11.42	2328	0.31	129	24.45	674
KIAA1919_t2	1.08	5.73E-07	NA	NA	NA	NA	0.17	117	11.35	2342	0.09	154	9.09	4192
[truncated: 2,307,002 more chars]
